# Identification and characterization of cold-responsive microRNAs in tea plant (*Camellia sinensis*) and their targets using high-throughput sequencing and degradome analysis

**DOI:** 10.1186/s12870-014-0271-x

**Published:** 2014-10-21

**Authors:** Yue Zhang, Xujun Zhu, Xuan Chen, Changnian Song, Zhongwei Zou, Yuhua Wang, Mingle Wang, Wanping Fang, Xinghui Li

**Affiliations:** Tea Research Institute, Nanjing Agricultural University, Weigang No.1, Nanjing, 210095 Jiangsu Province P. R. China; College of Horticulture, Nanjing Agricultural University, Nanjing, 210095 P. R. China; Molecular population genetics group, Temasek lifesciences laboratory, 1 Research link, National University of Singapore, Singapore, 117604 Singapore

**Keywords:** *Camellia sinensis*, MicroRNA, Cold-response, Microarray, Target identification

## Abstract

**Background:**

MicroRNAs (miRNAs) are approximately 19 ~ 21 nucleotide noncoding RNAs produced by Dicer-catalyzed excision from stem-loop precursors. Many plant miRNAs have critical functions in development, nutrient homeostasis, abiotic stress responses, and pathogen responses via interaction with specific target mRNAs. *Camellia sinensis* is one of the most important commercial beverage crops in the world. However, miRNAs associated with cold stress tolerance in *C. sinensis* remains unexplored. The use of high-throughput sequencing can provide a much deeper understanding of miRNAs. To obtain more insight into the function of miRNAs in cold stress tolerance, Illumina sequencing of *C. sinensis* sRNA was conducted.

**Result:**

Solexa sequencing technology was used for high-throughput sequencing of the small RNA library from the cold treatment of tea leaves. To align the sequencing data with known plant miRNAs, we characterized 106 conserved *C. sinensis* miRNAs. In addition, 215 potential candidate miRNAs were found, among, which 98 candidates with star sequences were chosen as novel miRNAs. Both congruously and differentially regulated miRNAs were obtained, and cultivar-specific miRNAs were identified by microarray-based hybridization in response to cold stress. The results were also confirmed by quantitative real-time polymerase chain reaction. To confirm the targets of miRNAs, two degradome libraries from two treatments were constructed. According to degradome sequencing, 455 and 591 genes were identified as cleavage targets of miRNAs from cold treatments and control libraries, respectively, and 283 targets were present in both libraries. Functional analysis of these miRNA targets indicated their involvement in important activities, such as development, regulation of transcription, and stress response.

**Conclusions:**

We discovered 31 up-regulated miRNAs and 43 down-regulated miRNAs in ‘Yingshuang’, and 46 up-regulated miRNA and 45 down-regulated miRNAs in ‘Baiye 1’ in response to cold stress, respectively. A total of 763 related target genes were detected by degradome sequencing. The RLM-5′RACE procedure was successfully used to map the cleavage sites in six target genes of *C. sinensis*. These findings reveal important information about the regulatory mechanism of miRNAs in *C. sinensis*, and promote the understanding of miRNA functions during the cold response. The miRNA genotype-specific expression model might explain the distinct cold sensitivities between tea lines.

**Electronic supplementary material:**

The online version of this article (doi:10.1186/s12870-014-0271-x) contains supplementary material, which is available to authorized users.

## Background

MicroRNAs (miRNAs) are a class of non-coding RNAs, approximately 19 ~ 21 nucleotides (nt) long, that function as post-transcriptional regulators in eukaryotes [1]. The miRNA gene is processed by Dicer-like proteins into a stem-loop miRNA::miRNA* duplex, after transcription by Pol II or Pol III enzyme into primary miRNA [2]. The miRNA::miRNA* duplex is then cleaved and transported from the nucleus into the cytoplasm. Single stranded miRNA then joins with Argonaute (AGO) to form an RNA-induced silencing complex (RISC) [3]. Finally, the RISC down-regulates targets by either cleaving the target mRNAs or repressing translation [4]. Plants miRNAs have important functions in response to biotic and abiotic stresses [5]. In recent years, numerous miRNAs have been identified in plant genomes [6], suggesting that the identification of their target RNAs is essential for the functional analysis of miRNA.

Cold stress negatively affects plant growth and development by causing tissue injury and delayed growth, which significantly restrict the spatial distribution of plants and productivity of economic crops [7]. Besides transcriptional regulation, miRNAs are also involved in cold-responsive gene regulatory networks. Sunkar and Zhu [8] showed that the expression levels of miR393 and miR319c are up-regulated by cold treatment. Microarray analysis revealed that approximately 17% of *Arabidopsis* miRNAs are up-regulated in response to low temperature at early stages of cold treatment [9]. The abundance of miR169 and miR172 in *Arabidopsis* challenged with cold stress was determined via a computational, transcriptome-based approach and microarray analysis [10,11]. Solexa sequencing analysis showed that the expression levels of three conserved miRNAs (miR169e, miR172b, and miR397) and 25 predicted miRNAs exhibit significant changes in response to cold stress in *Brachypodium* [12]. Cold resistance of the plant depends on different regulatory gene expression types related to physiology, metabolism, and growth [13]. In rice, 18 cold responsive rice miRNAs were identified using microarrays, and the members of the miR171 family showed diverse expression patterns [14]. Deep sequencing led to the identification of 30 cold responsive miRNAs in *Populous tomentosa* [15]. Although miRNAs have been extensively studied in *Arabidopsis* and other plant species, no systematic examination of miRNA has been performed on *C. sinensis*. Prabu [16] and Das [17] identified numerous conserved miRNAs and their targets in *C. sinensis* through in silico analysis. Six novel small RNA candidates were isolated and cloned; the small RNAs were validated through expression analysis in young and old leaves, during non-dormant and dormant growth phases of *C. sinensis* [18]. However, further study is needed to elucidate the functions of miRNAs at a genome-wide level in response to cold stress in *C. sinensis*.

Tea plant (*C. sinensis*) is one of the most important commercial beverage crops in the world. Cold stress may negatively affect the growth, development, and spatial distribution of tea plant, decreasing its yield and quality. Generally, cultivar specific expression exhibits strong relevance to the physiological functions of the corresponding cultivars [19]. Understanding cultivar-specific expression patterns of miRNA is necessary to gain insight into the functions of miRNA. Thus, ‘Yingshuang’ (YS, a cold-tolerant tea plant cultivar) and ‘Baiye 1’ (BY, a cold-sensitive tea plant cultivar) were chosen as two cultivars. In our study, high-throughput Solexa sequencing (Illumina Genome Analyzer) was employed to identify the *C. sinensis* miRNAs, which were responsive to cold stress, and 106 conserved miRNAs were obtained in the small RNA library. A selected number of cold-responsive and new miRNAs were then validated by Quantitative real-time polymerase chain reaction (qRT-PCR) combined with computational analysis. The identified miRNAs and their potential miRNA targets were predicted and confirmed by degradome sequencing. Abundantly conserved sequenced signatures were identified as the targets cleaved by conserved miRNAs, and novel miRNAs targeted different genes with various biological functions.

## Results

### High-throughput sequencing of small RNAs in tea plant

**Figure 1:**
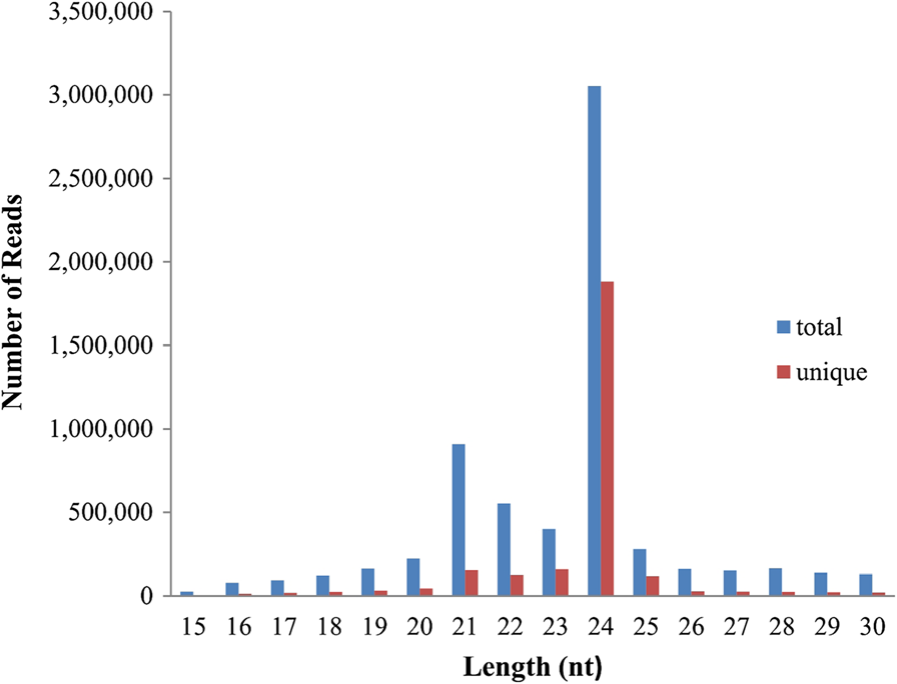
**Length distribution of small RNA sequences obtained in the tea plant libraries.**

Tea plants were stored at 4°C and 28°C for 1, 4, 8, 12, 24 and 48 h, respectively. A small RNA library of tea leaves, which was generated from a mixture of total RNAs from each cold-treatment stage, was subjected to high-throughput sequencing by the Illumina platform. Raw sequences were first subjected to an Illumina Pipeline filter provided by the supplier (Solexa 0.3). A total of 9,700,042 raw reads, representing 3,145,122 distinct sequences, were obtained. Reads without small RNA sequences, ranging from 15 nt to 30 nt in length, were filtered (Figure 1). The majority of the RNA sequences ranged from 19 nt to 25 nt in size. The most abundant small RNAs in the library were 24 nt long. The distribution of 24 nt small RNAs was approximately 45.96% and 69.67% in the total and unique sequences, respectively, whereas the distribution of 21 nt small RNAs in the total and unique sequences was approximately 13.68% and 5.67%, respectively. A total of 1,319,524 clean reads were obtained from the tea plant, including Rfam, rRNA, tRNA, snoRNA, snRNA, miRNA, other ncRNA, and repeats (Table 1). These clean reads were obtained by removing adaptor/acceptor sequences, filtering low quality tags, and cleaning the contaminants formed by adaptor-adaptor ligations and shorts RNAs less than 15 nt.

**Table 1:** **Distribution of small RNAs among different categories**

**RNA category**	**Counts**	**Percent (%)**	**Unique**	**Percent (%)**
Rfam	635584	48.17	70542	45.93
rRNA	393836	29.84	40951	26.66
tRNA	155847	11.81	13397	8.72
snoRNA	15579	1.18	3799	2.47
snRNA	10881	0.82	3615	2.35
other ncRNA	73742	5.59	12972	8.45
repeats	34055	2.58	8317	5.41
total	1319524	100.00	153593	100.00

Rfam(V 10.0) ftp://ftp.sanger.ac.uk/pub/databases/Rfam/9.1/ repeat-repbase (V13.12) http://www.girinst.org/repbase/update/index.html.

### Conserved miRNAs in tea plant

To identify the conserved miRNAs in tea plant, we compared our dataset with known plant miRNAs, such as miRNA precursors and mature miRNAs, in miRBase 19.0 by miRAlign. Following the BLASTN searches and further sequence analysis, 106 unique sequences belonging to 25 families in the small RNA library were found to be orthologs of known miRNAs from other plant species, which were previously deposited in the miRBase database (Additional file 1: Table S1). Moreover, 57 miRNA*s were identified, which are considered to be strong evidence of bona fide miRNAs [20]. Previous studies have predicted some miRNAs in tea plant. Six conserved miRNAs (csn-miR156a, csn-miR164, csn-miR169, csn-miR171a, csn-miR399, and csn-miR408) were identified and verified to those reported previously [16,17,21,22].

**Figure 2:**
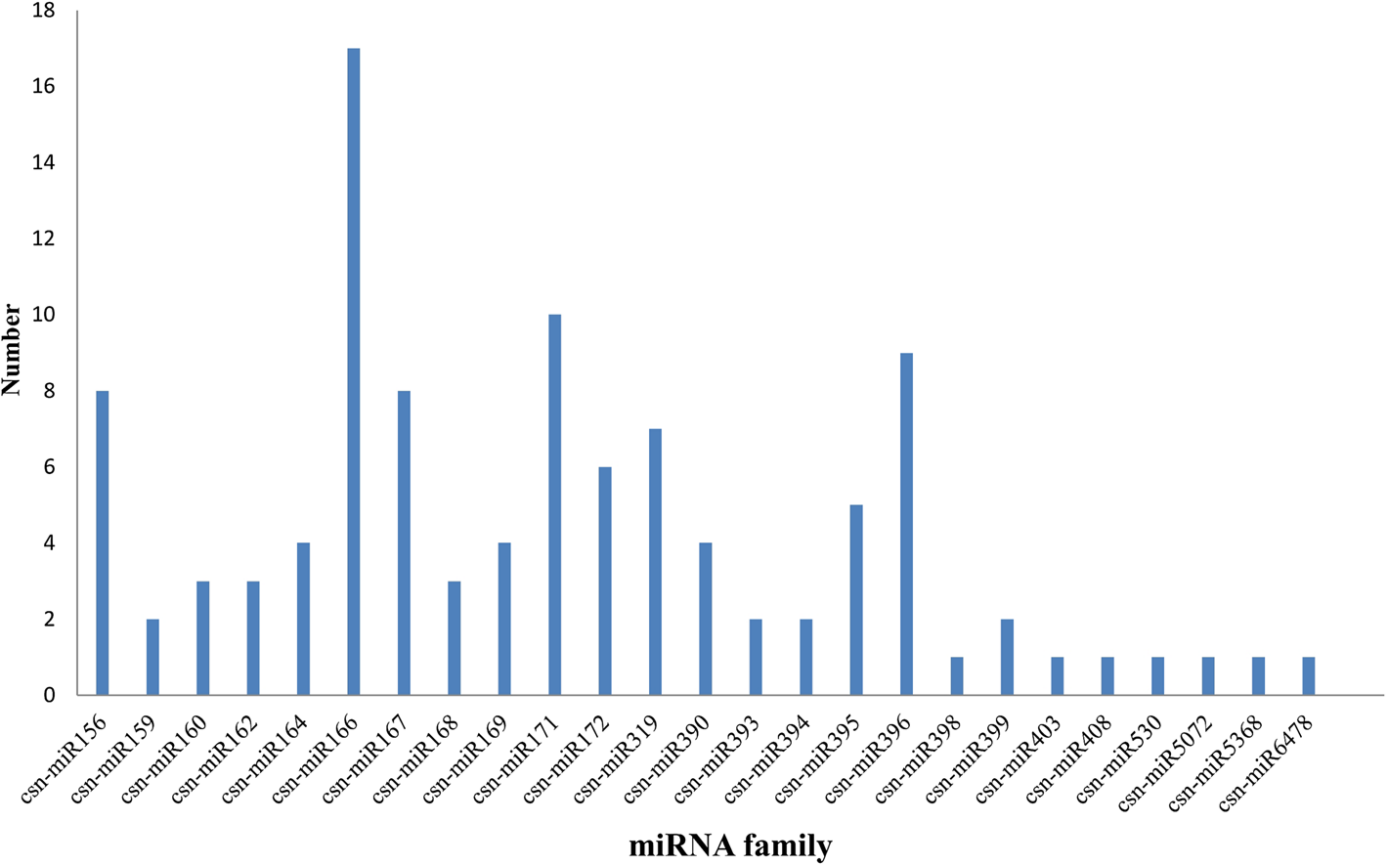
**Number of distinct members present in conserved miRNA fimilies in** ***C. sinensis*** **under cold stress.**

The number of different conserved miRNA family members was analyzed. The majority of the 25 miRNA families contained several members, and two families (miR166 and miR171) possessed multiple members, with 17 and 10 members, respectively, whereas seven miRNA families had only one member (Figure 2). The frequency of diverse members sequenced from the same or different miRNA families also varied drastically, ranging from one to 159,305 times. Among the 25 identified conserved csn-miRNAs, six miRNAs (csn-miR166a-1, csn-miR166a-2, csn-miR166a-3, csn-miR166a-4, csn-miR166a-5, and csn-miR166a-6) had the most reads, reaching up to 159,305. Moreover, four miRNA families were represented in tens of thousands, whereas some miRNAs (e.g., csn-miR156d, csn-miR395b, and csn-miR396f) had only one read sequenced. This large discrepancy in the expression levels of csn-miRNAs, deduced from the number of reads sequenced, could reflect the divergence of potential functions during the different stages of cold stress.

### Novel miRNAs in tea plant

In our study, the stem-loop structure of miRNA precursors was used to predict novel miRNAs, and the secondary structures of novel miRNA precursors were obtained by Mfold [23]. The secondary hairpin structures of the representative miRNAs are listed in Additional file 2: Figure S1. A total of 215 sequences were predicted to be potentially non-conserved miRNAs from the remaining unannotated sRNAs. Using updated plant miRNA annotation criteria [20], 98 sequences were recognized as novel miRNAs with high confidence and designated as novel *C. sinensis* miRNAs (Additional file 3: Table S2). The length of the mature miRNAs varied from 19 nt to 25 nt, with the majority being 24 nt long. Furthermore, the length of the novel miRNA precursors ranged from 72 nt to 264 nt, with an average length at 150 nt. The minimum free energy (MFE) of these novel miRNA precursors varied from −114.3 kcal mol^−1^ to −17.6 kcal mol^−1^, with an average of −61.79 kcal mol^−1^. The MFE index (MFEI) is a unique criterion to designate miRNAs. The MFEI is calculated by the equation: MFEI = (100 × MFE/L) / (G + C)% (L: the length of pre-miRNA). The sequence is most likely to be miRNA when the MFEI is more than 0.85 [24]. These novel miRNAs showed lower abundance levels, compared with the conserved miRNAs, which was consistent with previous studies [25-27]. The novel miRNAs showed different expression levels, and their normalized reads were from one to 1644. Thus, csn-smR30 (1644), csn-smR65 (794), and csn-smR80 (668) were the most abundant miRNAs. Most novel miRNAs were observed in less than 100 times, whereas 43 (43.89%) csn-smRNAs were sequenced in less than 10 times. The nucleotide bias at each position of the 98 novel identified miRNA (Additional file 3: Table S2) shoved that the first nucleotide of the new miRNA genes tended to be (U) in general. As expected, miRNAs are loaded to the RISC assisted by AGO1. Research has shown that AGO1 proteins have more affinity with uracil in the 5′ terminus of miRNA, thus resulting in cloned miRNA sequences with uracil nucleotide bias in the first position [28].

### miRNA microarray chip content and hybridization of arrays

Microarray-based hybridization was performed to analyze the expression of the newly identified miRNAs in tea plant. The sequences from miRBase (http://microrna.sanger.ac.uk/sequences/), and newly identified sequences in this study were used as probes for chip hybridization. The miRNAs showed different expression profiles under cold stress (4, 12, and 24 h) and non-treated conditions. Among the 3511 miRNA probes by microarray, a total of 303 and 349 conserved miRNAs were observed in ‘Yingshuang’ (YS, a cold-tolerant tea plant cultivar) and ‘Baiye 1’ (BY, a cold-sensitive tea plant cultivar) respectively (Additional file 4: Figure S2 and Additional file 5: Figure S3). The detected miRNAs were defined as a value of hybridization signal greater than 500, expression of miRNAs was significant difference when the signal ratio greater than 2 (|log|>1) and *p* was less than 0.01. Based on this principle, 158 tea plant miRNAs were differentially expressed compared with expression patterns under different cold stress stages in YS, and 159 miRNAs were differentially expressed in BY (Additional file 6: Table S3 and Additional file 7: Table S4), including 87 conserved miRNAs (p <0.01 and Signal >500) in both cultivars.

Both congruously and differentially regulated miRNAs were observed in our study, as well as cultivar-specific miRNAs. The majority of the differentially expressed miRNAs showed different expression patterns either among three cold stress stages, or between two tea cultivars. In YS, all of 31 miRNAs showed up-regulated trends, for example miR164, miR167, miR168, miR171, and so on, whereas 43 miRNAs (miR156, miR319, miR474, miR529, and the rest) showed down-regulated trends. By contrast, 46 miRNAs presented up-regulated trends, for instance miR168, miR474, miR1160, and so forth, while 45 miRNAs (miR159, miR166, miR171, miR529, etc.) presented down-regulated trends in BY. Three miRNA families (miR168, miR152, and miR2936) were uniformly regulated at four cold stress stages in the two plant cultivars. However, the expression level of miR171 and miR474 gradually increased in YS, but gradually declined in BY. These results strongly indicate that the regulatory patterns may be in accordance with delayed expression patterns in the cold-sensitive tea cultivar, which partly explains the distinct cold sensitivities between the two cultivars.

**Figure 3:**
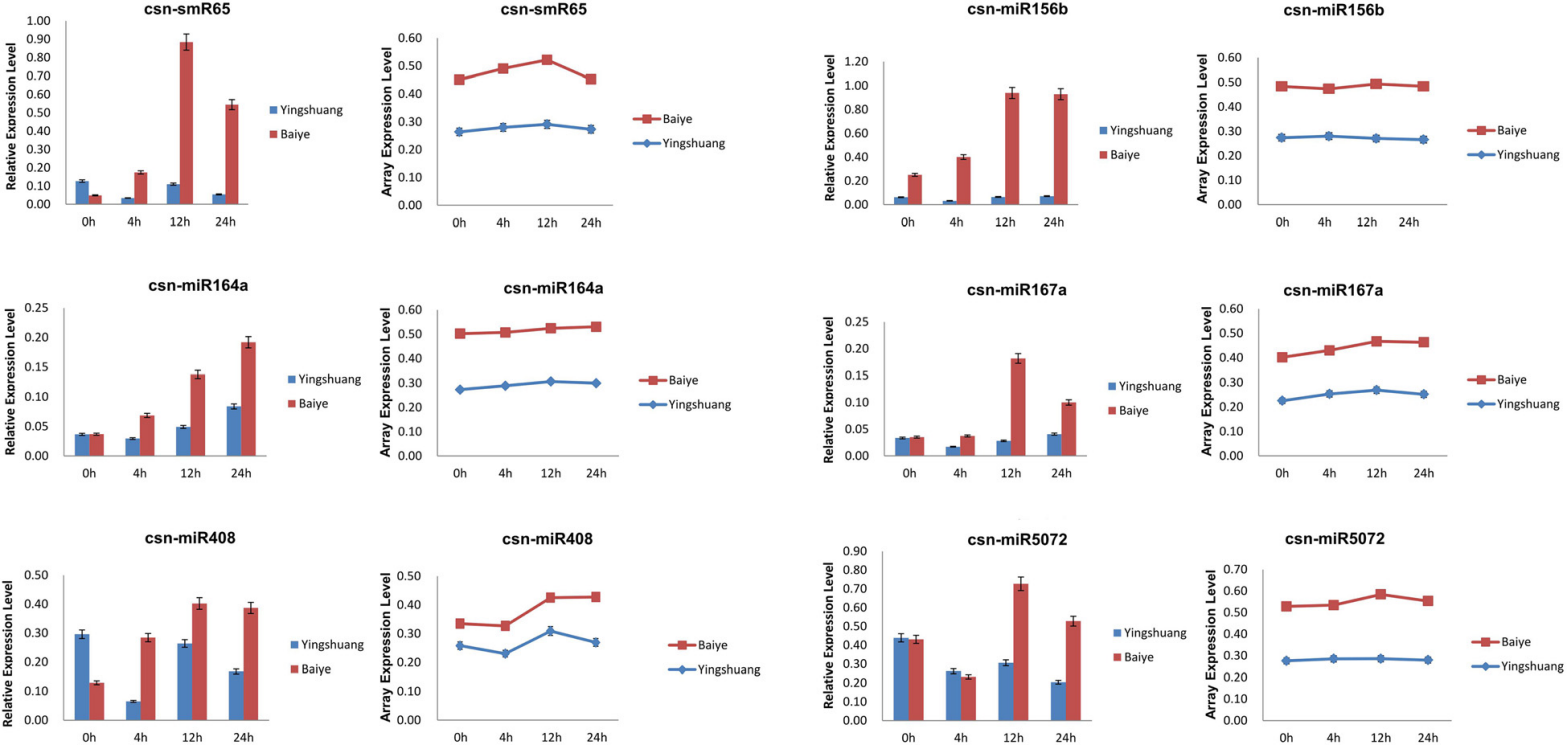
**Expression of miRNA in two tea lines with or without cold stress treatments.** Real-time PCR validation through the column chart displays, and line graph shows the differential expression of the same miRNA in tea leaf. Error bars represent standard deviation (n = 3).

To confirm the microarray results, the abundance of several miRNAs was further analyzed by qRT-PCR. The result of qRT-PCR and abundance profiles of the microarray shared similar trends (Figure 3). Discrepancies were also found in the magnitude of response at different cold stress stages, which could be due to cross hybridization between the probe and other highly homologous miRNA family members. The discrepancies could also be due to data normalization between the two methods. The qRT-PCR data were normalized to the abundance of 5.8S rRNA, whereas the microarray data were normalized to the global abundance of all miRNAs detected by microarray. The use of 5.8S rRNA was technically unfeasible as the normalization standard for microarray data.

### Targets identification for tea plant miRNA

We performed genome-wide analysis of miRNA-cleaved mRNAs to identify miRNA targets using high-throughput degradome sequencing technology [29,30]. We sequenced 9,224,714 and 6,736820 signatures for each library (−C and + C). After removing duplications, 7,439,589 and 5,376,267 distinct reads were obtained for -C and + C libraries, respectively. Alignment of the distinct sequences to tea plant expressed sequence tag (EST) sequences yielded 37,088 and 37,011 unique signatures for -C and + C libraries, respectively. We identified sliced targets for known miRNA and novel miRNA candidates based on the method of CleaveLand pipeline [31]. The abundance of sequences was plotted on each transcript (Additional file 8: Figure S4 and Additional file 9: Figure S5), and the sliced target transcripts were grouped into five classes according to the relative abundance of tags at the target sites [29]. Based on this approach, Category 0 and Category 1 have more than one raw read at the position. The abundance at the position is equal to the maximum on the transcript, and only one maximum is on the transcript. Category 2 has more than one raw read at the position. The abundance at the position is less than the maximum but higher than the median for the transcript. Category 3 has more than one raw read at the position. The abundance at the position is equal or less than the median for the transcript. Category 4 has only one raw read at the position.

**Figure 4:**
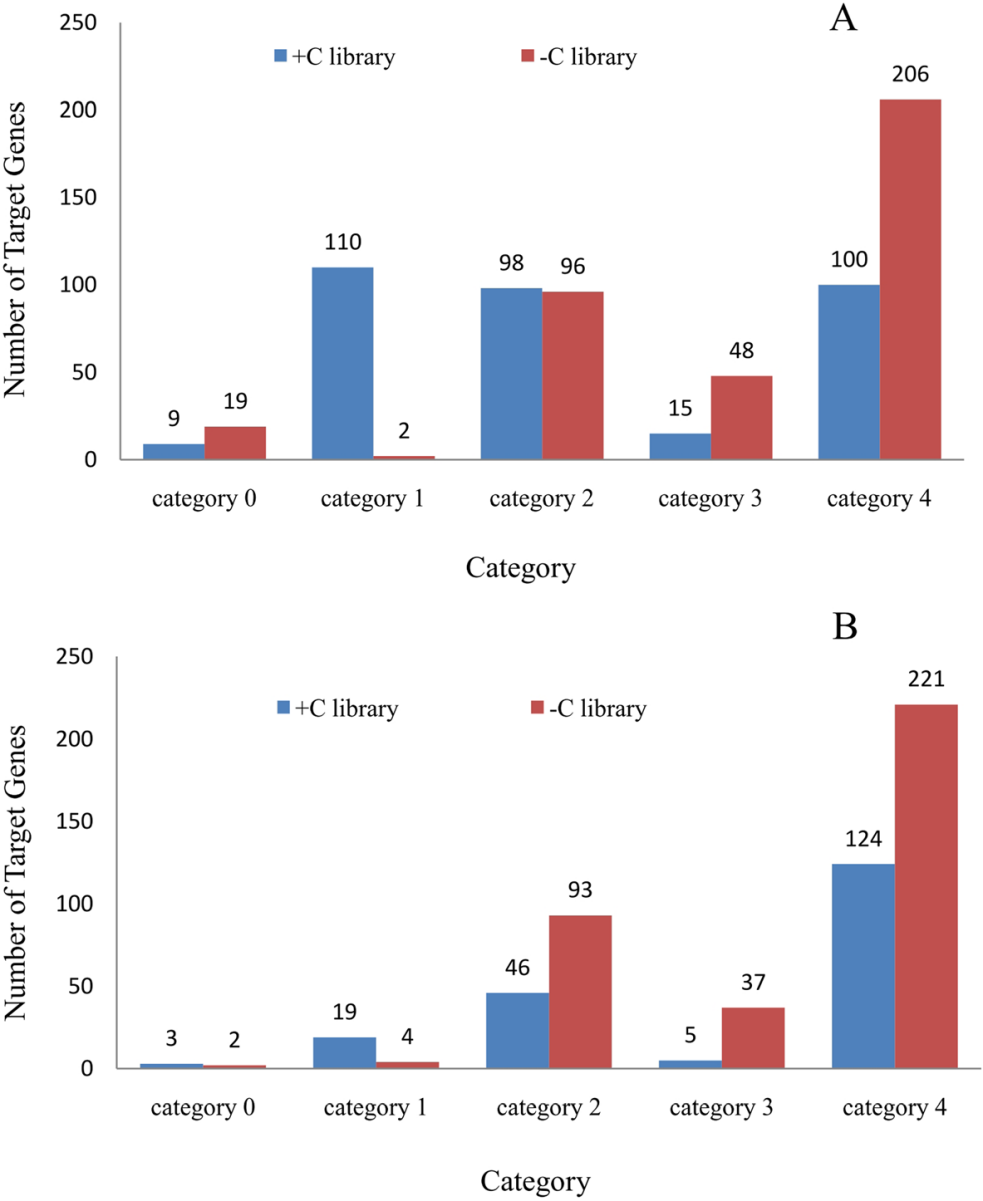
**Distribution of confirmed miRNA targets, separated by category in conserved miRNAs (A) and novel miRNAs (B).**

**Figure 5:**
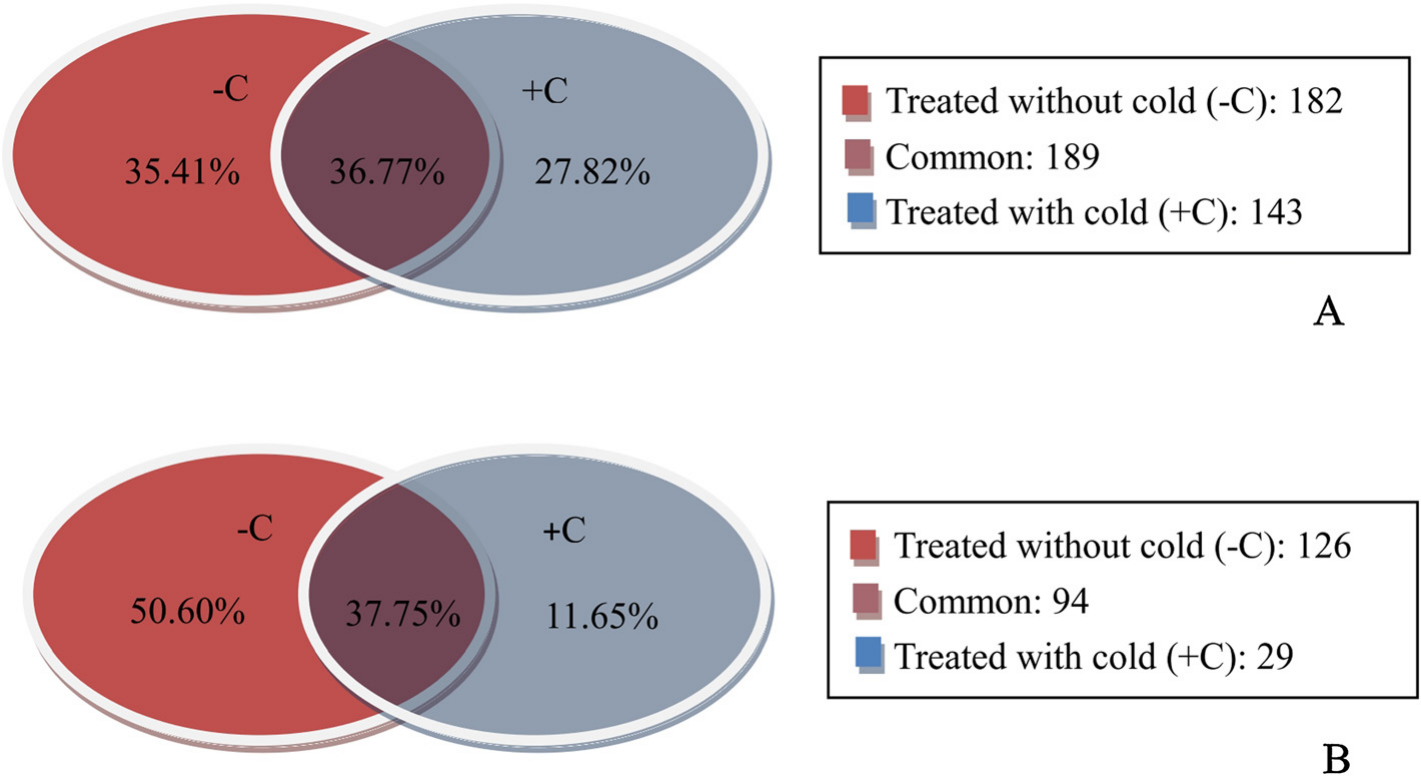
**Summary of common and specific targets between -C and + C libraries, targets of known miRNAs (A) and targets of new miRNA candidates (B).**

A total of 514 target transcripts were identified for 13 known miRNA families (Additional file 10: Table S5) based on our dataset, which shows most of the targets cleaved by the conserved miRNAs. A total of 332 targets in the + C library were identified for known conserved miRNA families, from which 9 (2.71%), 110 (33.13%), 98 (29.52%), 15 (4.52%) and 100 (30.12%) were grouped into categories 0, 1, 2, 3, and 4, respectively. For the -C library, 371 targets were identified, from which 19 (5.12%), 2 (0.54%), 96 (25.88%), 48 (12.94%), and 206 (55.52%) were grouped into category 0, 1, 2, 3, and 4, respectively (Figure 4A). Of these targets, 35.41% (182) were identified in the -C library, 27.82% (143) were identified in the + C library, and 36.77% (189) were present in both conditions (Figure 5A). Among the 13 conserved miRNA families, four (miR167, miR390, miR393, and miR398) were identified to have less than 10 targets, whereas the others target multiple transcripts. miR319 and miR160 had the highest number of targets, with 126 and 85 transcripts, respectively (Additional file 10: Table S5).

Forty novel miRNAs from 249 new candidates targets were identified (Additional file 11: Table S6). Profiling of the targets from the + C library showed that 3 (2.44%), 19 (15.45%), 46 (37.40%), 5 (4.06%), and 50 (40.65%) targets could be classified into categories 0, 1, 2, 3, and 4, respectively, and 2 (0.91%), 4 (1.82%), 93 (42.27%), 37 (16.82%), and 84 (38.18%) targets in the -C library fell into category 0, 1, 2, 3, and 4, respectively (Figure 4B). Of these targets, 50.60% (126) were identified in the -C library, 11.65% (29) were identified in the + C library, and 37.75% (94) were present in both conditions (Figure 5B). The distribution patterns of the two libraries differed, which suggests that the cleavage of targets by miRNAs was affected by cold stress.

Based on BLASTX analysis, 39.58% of the identified miRNA targets were generally homologous to conserved target genes that have already been found in *Arabidopsis thaliana*. Most of these conserved target genes were protein-coding genes, including zinc finger family protein (C2H2 and C3HC4 type), late embryogenesis abundant family protein (LEA), dormancy/auxin-associated family protein, and drought-responsive family protein, which are involved in plant growth, differentiation, development, and abiotic stress, respectively [32-35]. Among the identified miRNA targets, VQ motif-containing protein was a miR160 and miR408 target. The VQ motif represents the core of a protein-protein interaction domain, which is consistent with the interaction between another VQ motif protein with an RNA polymerase σ-factor [36,37]. Thus, the identified tea plant miRNAs could regulate a wide range of genes in development and other physiological processes.

### Identification of miRNA-guided cleavage of target mRNA using RLM-RACE

**Figure 6:**
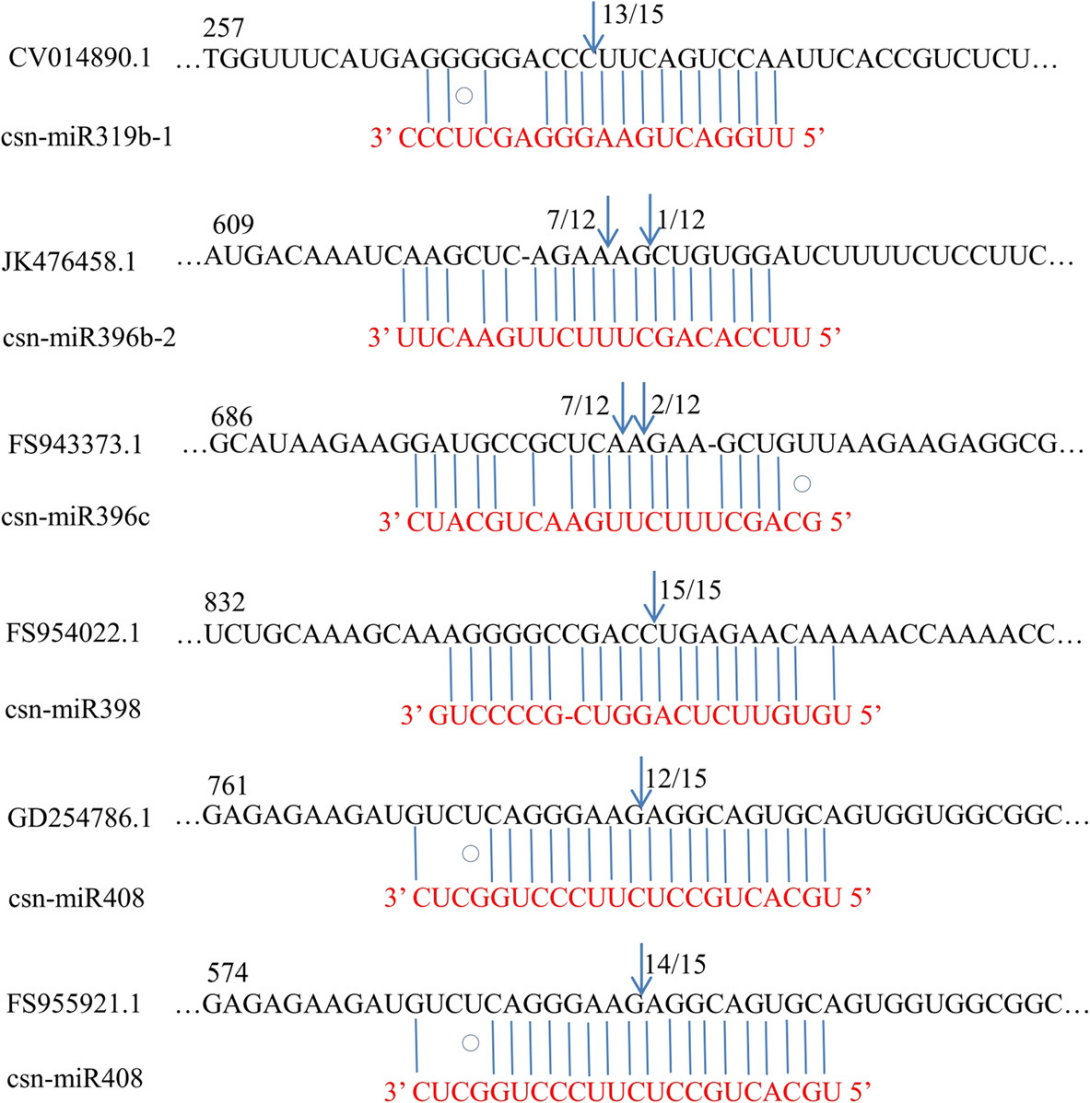
**Mapping of the mRNA cleavage sites by RNA ligase-mediated 5′RACE.** Each top strand (black) depicts a miRNA complementary site, and each bottom strand depicts the miRNA (red). Watson-Crick pairing (vertical dashes) and G:U wobble paring (circles) are indicated. RNA ligase--mediated 5′RACE was used to map the cleavage sites. The partial mRNA sequences from the target genes were aligned with the miRNAs. The numbers indicate the fraction of cloned PCR products terminating at different positions.

miRNAs, like small interfering RNA (siRNA), can direct the cleavage of their mRNA targets when these messages have extensive complementarity to the miRNAs [38-41]. This miRNA-directed cleavage can be detected by using a modified form of 5′ RNA ligase-mediated RACE (RLM-5′ RACE) because the 3′ product of the cleavage has two diagnostic properties: (1) a 5′ terminal phosphate, making it a suitable substrate for ligation to an RNA adaptor using T4 RNA ligase, and (2) a 5′ terminus that maps precisely to the nucleotide that pairs with the tenth nucleotide of the miRNA [39,42]. To verify the nature the csn-miRNA target genes and study how the csn-miRNA regulate their target gene, RLM-5′ RACE experiment was employed, which was carried out in this study for further characterization of csn-miRNAs functions. All six of the csn-miRNAs 5′ end of the mRNA fragment mapped to the nucleotide that pairs to the tenth nucleotide of one of the miRNAs validated by PCR (Figure 6). CV014890.1, JK476458.1, FS943373.1, FS954022.1, GD254786.1, and FS955921.1 were confirmed as the real targets of csn-miR319b-1, csn-miR396b-2, csn-miR396c, csn-miR398, and csn-miR408 respectively, since all the 5′ ends of the mRNA fragments were mapped to the nucleotide the pairs to the eleventh nucleotide of miRNA with higher frequencies than depicted for each pairing oligo. From the precise sequences of the csn-miRNAs results, we know that the miRNA-guided cleavage in *C. sinensis* obeyed the principle that base-paring to the 5′ ‘seed’ region of the miRNA was the dominant factor for the miRNA target recognition, and that the cleavage site was mostly located at the eleventh nucleotide, just 3′ of the ‘seed’ sequence [43]. All the six targets were found to have specific cleavage sites corresponding to the miRNA complementary sequences and might be regulated by the miRNAs in the style of siRNAs [44] directing the cleavage of mRNA targets with extensive complementarity to the miRNAs [42]. FS943373.1 is similar to *Arabidopsis* proteins coded by plant calmodulin-binding protein-related, FS954022.1 coded for a protein highly homologous to rubredoxin-like superfamily protein, GD254786.1 coded for a transposable element gene, while FS955921.1 code for a protein highly homologous to VQ motif-containing protein (Table 2).

**Table 2:** **Primers used for modified 5′ RLM-RACE mapping of the miRNA cleavage sites and putative target protein**

**miRNAs**	**Targets gene**	**Putative target protein**	**Conserved gene in** ***A. thaliana*** **(E-scroe)**	**Gene-specific primer**	**Nested gene-specific primer**
csn-miR319b-1	CV014890.1	Unknown protein		CCACGCTGGGCACTGTATGATGAT	
csn-miR396b-2	JK476458.1	Unknown protein		ATTCCCGCCAACAGCATCAATGTC	CACGCATCACCAAACACAGCGATAAG
csn-miR396c	FS943373.1	Plant calmodulin-binding protein-related	AT5G07820.1(5E-06)	AACGCTTTCCACCACCACCTCCAAG	CGCAGTCACCTCGGCTTTCTTAGC
csn-miR398	FS954022.1	Rubredoxin-like superfamily protein	AT1G80230.1(5E-34)	CTTCACCTCCAGGGCATCCAACAAT	GTCCCGTGGCAATAGGCATCACATCT
csn-miR408	GD254786.1	Transposable element gene	AT5G29056.1(4E-07)	GCCAGGGAGAGAGCAAATGAAGAAGTTC	CCAGCCTTGTTCACACTGACCACATTGT
csn-miR408	FS955921.1	VQ motif-containing protein	AT1G28280.1(1E-12)	GCCAGGGAGAGAGCAAATGAAGAAGTTC	CCAGCCTTGTTCACACTGACCACATTGT

### Gene ontology (GO) function analysis of targets

**Figure 7:**
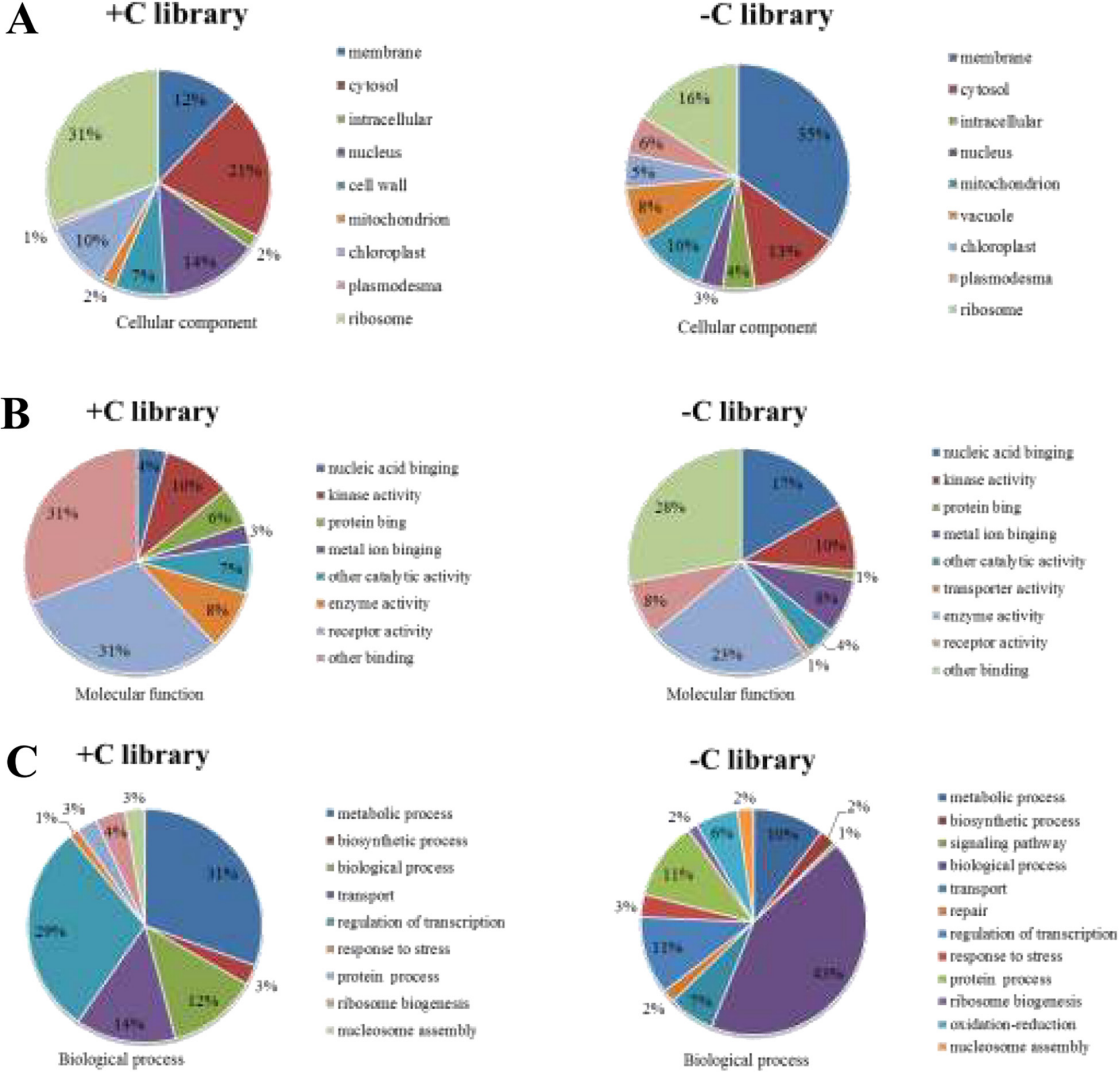
**Gene ontology of the predicted targets for 57 differentially expressed miRNAs.** Categorization of miRNA-target genes was performed according to the cellular component **(A)**, molecular function **(B)** and biological process **(C)**.

GO categories were assigned to all targets, including 514 known targets and 249 new candidates, according to three ontologies in GO: cellular component, molecular function, and biological process (Figure 7). Comparing the target gene functions of two libraries, more than 50% of the genes were classified into cellular component, of which 11 genes function belong to cell wall in + C library, however, there was no such target genes in -C library (Figure 7A). Based on the molecular function, genes were finally classified into eight classes, the three mainly represented GO terms were receptor activity (31%), other binding (31%), and kinase activity (10%) in + C library, while other binding accounted for 28%, followed by enzyme activity for 23%, receptor activity only 8% in -C library (Figure 7B). In the biological process, the target gene functions focused on the metabolic process (31%) and regulation of transcription (29%) in + C library, while the two class processes were only 10% and 11% in –C library, respectively (Figure 7C). This difference in the function of the target genes showed tea plant cell structure was severe damaged under cold stress. Moreover, stress-response genes were also identified as miRNA targets, including salt stress response, heat shock protein binding, and water deprivation response. The results imply the possible function of miRNAs in the regulation of biological processes involved in cold-stress.

## Discussion

### Identification of miRNAs in tea plant

Many highly conserved miRNAs that exhibit particular expression patterns with specific timing and tissue specificity, have critical functions in growth, development, differentiation, apoptosis, metabolism and biotic and abiotic stress responses, regulating specific target mRNAs. Some tea plant miRNAs and their target genes have been identified using bioinformatical approaches in previous reports. Fourteen new *C. sinensis* miRNAs were recently identified from 47,452 available *C. sinensis* ESTs, and these miRNAs potentially target 51 mRNAs, which can act as transcription factors, and participate in transcription and signal transduction [21]. Recent advances in high-throughput sequencing methods have revolutionized the identification of low-abundance, novel miRNAs in various species [27,45-47]. However, no comprehensive study on a novel miRNA discovery has been reported for tea plant. This study aimed to identify the evolutionary known and potentially novel tea plant-specific miRNAs recovered from cold stress tea plant libraries. The differential expression of miRNAs associated with cold stress response was also analyzed. Thus, approximately nine million sRNA raw reads were obtained from the sRNA library, in which 25 conserved miRNA families and 98 potentially novel miRNAs were successfully identified. The read number varied from one (miR156, miR395, and miR396) to 159,305 (miR166) (Additional file 1: Table S1), suggesting dramatically varied expression patterns among each miRNA family. However, only a small proportion of the conserved and novel tea miRNAs was detected, because of the unavailability of full genome sequences of tea plant. The number of miRNAs identified from tea plant appears to be far from saturation, and numerous unknown miRNAs remain to be discovered.

For a broader perspective of high-throughput sequencing of small RNAs from tea plant, we observed that small RNAs of 24 nt dominated the library of unique species, which was reported for other plant species, such as *A. thaliana* [27], *Citrus trifoliata* [48], *Medicago truncatula* [49], and *Citrus sinensis* [50]. Length distribution analysis is effective in assessing the composition of small RNA samples. The overall distribution pattern of small RNAs (21 nt sRNAs =5.67%, 24 nt sRNAs =69.67%) in tea plant was significantly different from that in *Populous trichocarpa*, a model forest species, in which 21 nt RNAs are more abundant (37.16%) and 24 nt RNAs are less frequent (<5%) [51]. A difference was also present between tea plant small RNAs and monocot species of maize [52]. These results indicate that the small RNA transcriptome was complex across plant species, and could be significantly different between phylogenetically distant plant families [53].

Based on deep sequencing and hairpin structure prediction, we successfully identified 98 novel miRNAs, and 53 novel miRNAs with complementary miRNA* strands. Precursors of these miRNAs formed secondary hairpin structures with free energies ranging from −114.3 kcal mol^−1^ to −17.6 kcal mol^−1^ (average 61.79 kcal mol^−1^) (Additional file 3: Table S2). The secondary structures are not a unique feature of miRNAs because random inverted repeats may also form hairpin structures [54]. Identification of anti-sense miRNAs (miRNAs*) of the candidate miRNAs provides credible evidence that these miRNAs are authentic miRNAs [20]. One difference between conserved and novel miRNAs is that novel miRNAs are expressed at substantially lower levels or in a tissue-specific or environmentally inducible pattern [27,55].

### Different expression profiles of cold-responsive miRNAs between two tea plant cultivars

**Figure 8:**
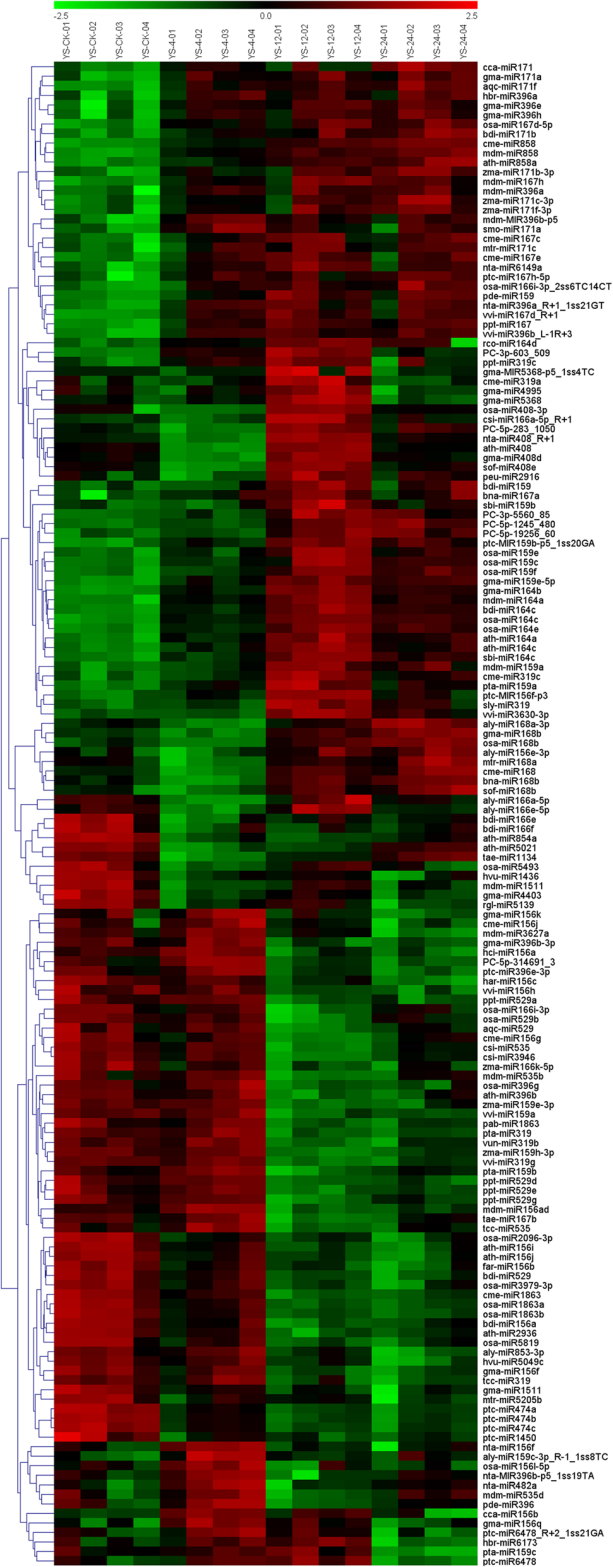
**Differential expression of miRNAs at four metamorphic stages (CK, 4 h, 12 h, and 24 h) by hierarchical clustering in ‘Yingshuang’.** Red indicates that a gene is highly expressed at that stage, whereas green indicates the opposite. The absolute signal intensity ranges from −2.5 to +2.5, with corresponding color changes from blue to green, yellow and red. The signal of expression was detected by microarray with four probe repeats. YS-CK: control; YS-4: 4°C for 4 h; YS-12: 4°C for 12 h; YS-24: 4°C for 24 h.

**Figure 9:**
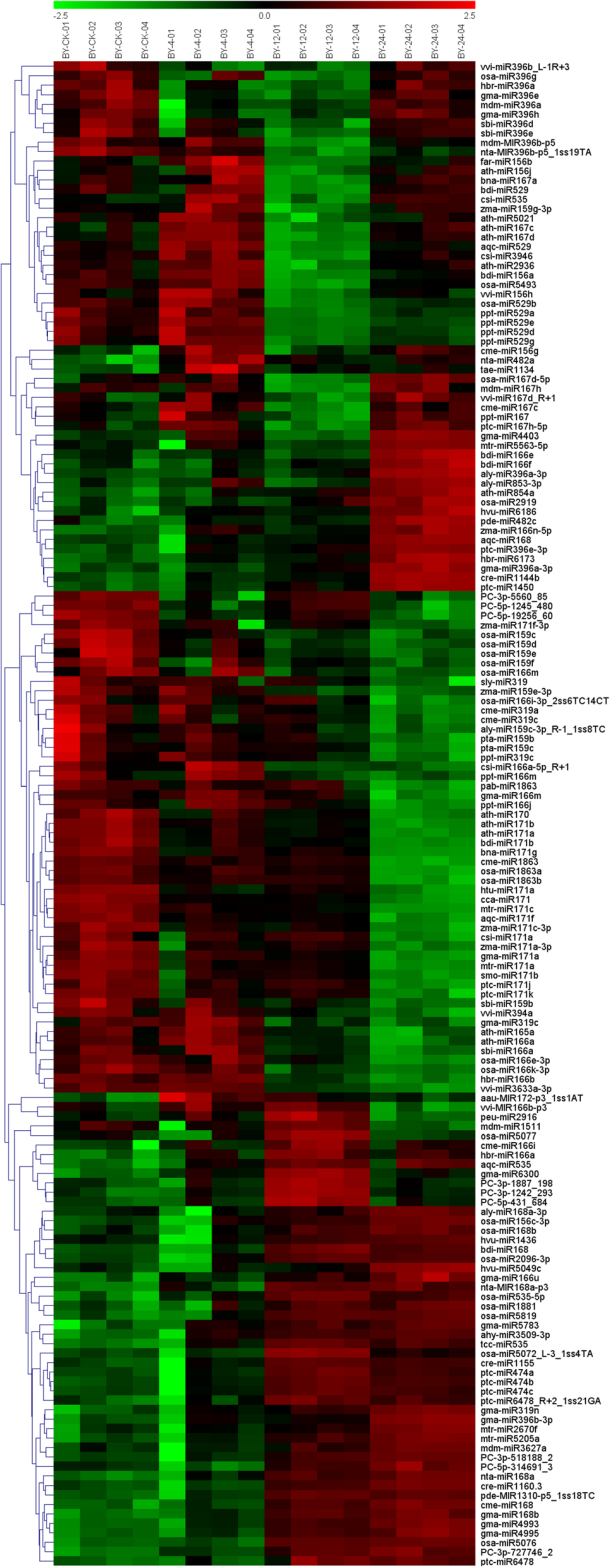
**Differential expression of miRNAs at four metamorphic stages (CK, 4 h, 12 h, and 24 h) by hierarchical clustering in ‘Baiye 1’.** Red indicates that a gene is highly expressed at that stage, whereas green indicates the opposite. The absolute signal intensity ranges from −2.5 to +2.5, with corresponding color changes from blue to green, yellow and red. The signal of expression was detected by microarray with four probe repeats. BY-CK: control; BY-4: 4°C for 4 h; BY-12: 4°C for 12 h; BY-24: 4°C for 24 h.

The cultivar-specific and cold-responsive miRNAs were further determined using a customized microarray. A total of 18 and 14 conserved cold-responsive miRNA families were identified from YS and BY, respectively. Moreover, six and nine new cold-responsive miRNA families were identified from YS and BY, respectively. A quarter of miRNAs were down-regulated (27.2% in YS and 28.3% in BY), indicating that the expression of target genes controlled by these miRNAs was turned on to adapt to cold stress. We found that many members of families (miR156, miR159, and miR396) showed no consistent regulatory pattern for cold stress response, thereby suggesting different functions of miRNAs from the same family (Figures 8 and 9). Moreover, there was a certain degree of overlap of cold-stress pathway of miR168, miR529, and miR2936 by comparing miRNAs expression patterns between different cultivars. By contrast, the expression of miR164, miR408, miR1511, miR5368, miR172, miR482, miR529, and miR1160 was significantly different in the two cultivars (Figures 8 and 9).

miRNAs are known to silence genes post-transcriptionally by guiding target mRNAs for degradation or repressing translation [5]. NADP-ME is a key enzyme that catalyzes the oxidative decarboxylation of L-malate to yield pyruvate, CO_2_, and NADPH in the presence of a divalent cation [56]. In plants, malate can be decarboxylated in the mitochondrial matrix through the action of NAD-ME to produce pyruvate, which is oxidized by the tricarboxylic acid cycle. The genes for these two enzymes are predicted to be the target of miR474. The miR474 family was down-regulated in YS and up-regulated in BY. The result indicates that a cold induced, non-specific responding pathway was possibly involved in the maintenance of energy supply.

The miR171 family is a largely conserved miRNA family, and microarray assay revealed that miR171a in *Arabidopsis*, is induced at 6 h in response to cold stress [11]. In our study, the miR171 family was significantly up-regulated in YS and down-regulated in BY, which suggests that miR171 members may perform different functions. AGO1 is regulated by miR168, AGO1-catalyzed mRNA cleavage, and is the only member of the *Arabidopsis* AGO family involved in the miRNA-directed mRNA cleavage processes. The co-adjustment of miR168 and AGO1 indicates negative feedback regulation in the miRNA pathway [57]. In our study, miR168 was found to be a cold-responsive miRNA and the members of the csn-miR168 family were induced in the two tea cultivars after 12 h of cold treatment.

### Degradome analysis of cold responsive targets for tea plant miRNAs

Accurate identification of target genes is essential to reveal the regulatory function of miRNA. Previous work on gene identification in tea plants was limited to bioinformatics prediction [21]. The recently developed high-throughput experimental approach allows the identification of target genes for known and new miRNAs [29,58]. A total of 514 potential targets for 13 known miRNA families and 249 new tea-specific targets for 40 novel miRNA families in tea plant were identified by degradome sequencing (Additional file 10: Table S5 and Additional file 11: Table S6). Numerous highly conserved miRNAs were identified, and did not have detectable sliced targets, including miR159, miR166, miR168, and miR394 (Additional file 1: Table S1). The novel miRNAs targeted different genes covering various predicted functions (Additional file 11: Table S6). For example, csn-smR5749 targets nine genes of the Ras-related, small GTP-binding protein family, while csn-smR2592 affects zinc finger (C3HC4-type RING finger) protein family. These two protein families have been reported to be involved in various physiological processes in plants [33,59]. By contrast, csn-smR2845 targets the dormancy or auxin-associated protein family, which has an important function in the inhibition of growth and differentiation [35]. Recently, an increasing number of genetic and biochemical results showed that plant miRNA-guided silencing has a widespread translational inhibitory component [60,61].

Neighboring nucleotides (the ninth or 11^th^ positions) are cleavage sites for some plant miRNAs in certain cases [29,58]. This observation accounts for the occasional positional heterogeneity of mature miRNAs [55] and their cleavage products [62,63]. Upon comparing the two degradome libraries, we classified many cold-responsive miRNA targets as categories 0, 1, and 2 (Additional file 10: Table S5), thereby confirming the accuracy of our degradome analysis, because categories 0, 1, and 2 are characteristic for miRNA targets in *Arabidopsis* [29]. The most obvious difference between the targets of cold-treated and cold-free miRNAs was that 254 of 371 cold-free miRNA targets belonged to categories 3 and 4 (Additional file 10: Table S5), in which cleavage abundance was lower than the median on the target transcripts. The target distribution in each library was generally similar to those recently reported in other plant species [46,64]. Moreover, the distribution pattern and transcript abundance of the targets between the two libraries differed, suggesting that the cleavage of targets by miRNAs was affected by cold stress. We also found target transcripts that were regulated by pairs of miRNAs. The miR160/miR319 pair targeted five different targets, the miR160/miR408 pair controlled two motif elements, the miR319/miR396 pair targeted protein prenyltransferase, and the miR171/miR396 pair targeted the Ras-related, small GTP-binding family protein (Additional file 10: Table S5). This phenomenon suggests a combinatorial gene regulation pathway using a pair of miRNAs in tea plants [46].

Degradome sequencing confirmed that csn-miR164 targeted a LEA protein. LEA proteins represent one of the functional elements, which are important factors in maintaining the viability of an organism and its biological structures in the ametabolic low water state, such as cold, heat, and drought [65]. Part of the mechanism could be related to protein stabilization but other mechanisms have also been suggested for LEA proteins, because LEA proteins and other hydrophilins can preserve enzyme activity *in vitro* after desiccation or freezing [66-70]. In addition, csn-miR319 was detected to target a zinc ion binding and trans-membrane amino acid transporter protein family. Metal ion (copper, zinc, or iron) binding is important in normal respiratory function and the process of protein damage during oxidative stress in *A. thaliana*. Strong correlations exist between the sets of immobilized metal affinity chromatography-interacting proteins, which are proteins predicted to contain metal-binding motifs, and protein sets that are oxidized or degraded during abiotic stress [71]. Zinc ion binding increases the structural complexity of abscisic acid stress ripening proteins, which is associated with DNA binding [72,73]. The WW-domain-binding protein (WBP), which was previously found only in animals, has been predicted in tea plants and targeted by csn-miR408. WW-domains are small protein modules found in various proteins that participate in cell signaling or regulation [74]. The WBP targets proline-rich sequence-recognizing domains, which are important for selectivity in signaling [75]. The conserved miRNA families may have functions concerning the regulation of cytothesis, abscisic acid stress, signaling, and maintaining viability of biological structures in response to cold stress in tea plants.

The degradome sequencing data shows six novel miRNAs (csn-smR35, csn-smR3146, csn-smR5749, csn-smR7277, csn-smR8111, and csn-smR9722), which differentially target some cold-responsive genes in the cold-treated library. csn-smR5749 was predicted to target a KH domain protein. The KH domain proteins are important in RNA metabolism, vegetative, and reproductive development [76-78]. These proteins also have functions in organogenesis in stem cells by interacting with other proteins [79]. The drought-responsive protein gene family was predicted to be the target of csn-smR9722, suggesting the existence of crosstalk between drought and cold stress signaling pathways.

miRNA generally function as negative regulators of gene expression by mediating the cleavage of target mRNAs [39] or by repressing their translation [80], the cleavage of target mRNAs appear to be predominant mode of gene regulation by plant miRNAs [81]. To verify the nature the csn-miRNA target genes and study how the csn-miRNA regulate their target gene, we performed the RACE on unigene to detect and clone the mRNA fragment corresponding precisely to the product of miRNA processing. In total, we performed RLM-5′RACE assays on six target genes i.e. the representative targets of five conserved miRNAs (Figure 6). This result showed that the degradome analysis was an efficient and powerful approach which can be successfully used to validate the sequences of miRNAs.

## Conclusions

This study identified 106 known miRNAs and 98 potentially novel miRNAs, based on the constructed miRNAomes between two tea plant cultivars, YS and BY. A total of 18 and 14 conserved cold responsive miRNA families were identified from YS and BY, respectively, using a customized microarray. A total of 455 and 591 genes were identified as cleavage targets of miRNAs from cold treatments and control libraries, respectively, and 283 targets were present in both libraries. The RLM-5′RACE procedure was successfully used to map the cleavage sites in six target genes of *C. sinensis*. GO annotation revealed that highly ranked miRNA target genes were those implicated in the developmental process, regulation of transcription and stress response. These findings provide valuable information for further functional characterization of miRNAs associated with cold stress in tea plants*.*

## Methods

### Plant materials and cold stress treatment

Two tea plant cultivars, ‘Yingshuang’ (YS, a cold-tolerant tea plant cultivar) and ‘Baiye 1’ (BY, a cold-sensitive tea plant cultivar), were culture-grown under a 12-h light (28°C)/12-h dark (22°C) photoperiod (photo intensity 1800 Lx photos m^−2^.s^−2^) for 20 days. To analyze the miRNAs of tea leave under cold stress, tea plants were stored at 4°C for 1, 4, 8, 12, 24, and 48 h. For the controls, tea plants were stored at 28°C for 1, 4, 8, 12, 24, and 48 h. The treated and untreated young leaves were then harvested into liquid nitrogen and stored at −80°C until total RNA extraction.

### Small RNA library construction and sequencing

Total RNA was isolated with TRIzol reagent (Invitrogen, Carlsbad, CA, USA) according to the manufacturer’s instructions. RQ1 RNase-Free DNase (Promega, Madison, WI, USA) was used to remove genomic DNA contamination. The quality and integrity of RNAs were examined using an Agilent 2100 Bioanalyzer. After taking equal amounts of controls and six-stage total RNAs (4°C for 1, 4, 8, 12, 24, and 48 h), these samples were pooled together for the construction of a cold-stressed small-RNA library (CL).

Total RNA was purified by electrophoretic separation on a 15% Tris-Borate-EDTA (TBE)-urea denaturing polyacrylamide gel, and small RNA regions (15 to 30 nucleotide bands) were excised and recovered. The small purified RNAs were then ligated with 5′ adaptors (Illumina, San Diego, CA, USA), and the ligation products (40 to 60 bases in length) were purified on a Novex 15% TBE-Urea gel to remove unligated adaptors. Subsequently, a 3′ adaptor (Illumina) was ligated to the 5′ ligation products. After gel purification on a Novex 10% TBE-Urea gel (Invitrogen), RNA fragments with adaptors at both ends (70 to 90 bases in length) were reverse transcribed, and the resulting cDNAs were subjected to 15 PCR cycles using the adaptor primers. The resulting small RNA library was then sequenced by Genome Analyzer GA-I (Illumina, San Diego, CA, USA).

### Identification of conserved and novel miRNAs in tea plant

Clean full-length reads were obtained from raw sequences after removing all low-quality reads, contaminants, and reads smaller than 15 nt. The extracted clean reads were used to calculate length distribution, and mapped to the *P. trichocarpa* genome sequence, and publicly available tea EST sequences using SOAP [82]. Sequence matching non-coding RNAs, including rRNA, tRNA, snRNA, and snoRNA, in the NCBI GenBank (http://www.ncbi.nlm.nih.gov/genbank/), repeats (http://www.girinst.org/repbase/update/index.html) and Rfam (http://www.sanger.ac.uk/software/Rfam) databases were eliminated. Unique miRNA sequences were aligned with known miRNA sequences from miRBase 19.0 (http://www.mirbase.org/) with a maximum of two mismatches. The obtained sequences were used to predict secondary structures by RNAfold software (http://www.tbi.univie.ac.at/RNA/) to identify whether these precursors form a typical miRNA stem-loop structure. The remaining unknown sRNAs were analyzed, and novel miRNAs were predicted using Mireap (http://sourceforge.net/projects/mireap/files/latest/download). The novel miRNAs were screened according to the recently published criteria of novel miRNAs [20]. The novel miRNAs and their corresponding miRNA*s formed a stem-loop duplex with less than four mismatched bases, derived from opposite stem-arms. The novel miRNAs were typically named using a ‘miR’ prefix to denote miRNAs, and a three-letter prefix to denote the species (e.g. ‘csn’ representing *Camellia. sinensis*).

### miRNA microarray chip content and hybridization of arrays

The miRNA chip included 3228 miRNA probes corresponding to the miRNA transcripts listed in Sanger miRBase release 19.0, and 283 novel miRNAs probes were found in tea plant. Microarray assay was performed using a service provider (LC Sciences, Houston, USA). Total RNAs was isolated separately from leaf samples of 4 h, 12 h, and 24 h of cold-treated and non-treated materials. Total RNA samples (4 μg to 8 μg) were then 3′-extended with a poly (A) tail using poly (A) polymerase. An oligonucleotide tag was ligated to the poly (A) tail for fluorescent dye staining. Hybridization was performed overnight on a μParaflo microfluidic chip using a micro-circulation pump (Atactic Technologies) [30,83]. On the microfluidic chip, each detection probe consisted of a chemically modified nucleotide-coding segment complementary to the target miRNA from miRBase or other RNAs, and a spacer segment of polyethylene glycol to extend the coding segment away from the substrate. The detection probes were made by in situ synthesis using photogenerated reagent chemistry. The hybridization melting temperatures were balanced by chemical modifications of the detection probes. Hybridization was performed using 100 μL of 6xSSPE buffer (0.90 M NaCl, 60 mM Na_2_HPO_4_, 6 mM EDTA, pH 6.8) containing 25% formamide at 34°C. After RNA hybridization, tag-conjugating Cy3 dye was circulated through the microfluidic chip for staining. Fluorescence images were collected using a laser scanner (GenePix 4000B, Molecular Device), and digitized using Array-Pro image analysis software (Media Cybernetics).

Data were analyzed by first substracting the background and then normalizing the signals using a locally-weighted regression filter [84]. An miRNA signal was accepted as detectable if it met two conditions: signal intensity higher than three times the background standard deviation, and spot CV <0.5 (CV = signal standard deviation/signal intensity). Signals from four technical replicates of each RNA derived from stressed and control plants were compared using paired and two-tailed Student’s *t* test. Only signals with p <0.01 were considered as significant. Clustering analysis was performed using Cluster 3.0, and the heat map was visualized using Heatmap builder and TreeView [85].

### qRT-PCR analysis of miRNA expression

The sRNAs (<200 nt from samples treated with cold conditions (4°C) for 4, 12, and 24 h were isolated using an mirVana miRNA isolation kit (Ambion, Austin, TX, USA). Stem-loop reverse transcription PCR (RT-PCR), which can be applied in small RNA cloning and multiple assays for better specificity and efficiency, was used for reverse transcription [86]. A stem-looped RT-PCR Primer and qRT-PCR primers (Additional file 12: Table S7) were designed. One Step Prime-Script miRNA cDNA Synthesis Kit (TAKARA) and Platinum SYBR Green qPCR SuperMix-UDG (Invitrogen) were used. The 5.8S rRNA was used as the internal control [87]. Thereafter, qRT-PCR was performed using a total reaction volume of 20 μL, which contained 0.5 μL of diluted cDNA, 8 μM primer mix, 10.0 μL of 2× SYBR Green Mix, and 8.7 μL ddH_2_O. The reactions were carried out in an iCycler iQ qRT-PCR detection system (BIO-RAD) with the following amplification conditions: activation at 50°C for 2 min; 95°C for 2 min; followed by 39 cycles at 95°C for 15 s, and 60°C for 30 s; and final holding at 4°C. The cycle threshold (CT) was defined as the fractional cycle number at which the fluorescence passes the fixed threshold. All reactions were performed in triplicate. The qRT-PCR results were normalized as follow: sample CT values were determined and standardized based on the 5.8S gene control prime reaction, and the 2^-ΔΔCT^ method was applied to calculate the relative changes in gene expression from qRT-PCR experiments [88].

### Degradome library construction and target identification

To obtain the potential target mRNAs, two degradome libraries were constructed from tea leaves treated with cold (+C) and cold-free (−C) as previously described [58,64]. In brief, poly(A)-enriched RNA molecules were isolated and ligated to an RNA oligonucleotide adaptor containing a 3′ MmeI recognition site, and the ligated products were used to generate first-strand cDNA by RT. A short PCR was then used to amplify the cDNA. To obtain sufficient quantities of DNA, the product was ligated to a double-stranded DNA adaptor, and subjected to gel purification again for PCR amplification. The final cDNA library was purified and sequenced on Illumina GAIIx following the manufacturer’s instruction.

Raw sequence reads were obtained using Illumina Pipeline v1.5 software to remove adaptor sequences and low quality sequencing reads. The extracted sequencing reads with lengths of 20 nt and 21 nt were then used to identify potentially cleaved targets by CleaveLand pipeline, as previously described [29,31]. The degradome reads were mapped to *A. thaliana* and tea plant sequences of mRNA and ESTs were downloaded from NCBI (http://www.ncbi.nlm.nih.gov/). Only the perfectly matched alignments for the given read were kept and extended to 35 nt to 36 nt by adding 15 nt of upstream sequences. All resulting reads (t-signature) were reverse-complemented and aligned to the miRNA identified in our study. No more than five mismatches of the alignments were allowed. Alignments in which the 5′ degradome sequence position coincided with the 10^th^ nucleotide of miRNA were retained and scored using a previously described method [89]. The target was selected and categorized based on cellular component, molecular function, and biological process. All identified targets were subjected to BlastX analysis to search for similarity. GO analysis was used to uncover the miRNA-gene regulatory network on the biological process and molecular function, as previously described by Xie et al. [90].

### Modified 5′ RNA ligase-mediated RACE for the mapping of mRNA cleavage sites

Total RNA was extracted from young leaves of different treated and CK using Trizol reagent. Poly(A)^+^ mRNA was purified from all kinds of pooled sample RNA using the PolyA kit (Promega, Madison, WI), according to manufacturer’s instructions. A modified procedure for RLM-5′ RACE was followed with the GeneRacer Kit (Invitrogen, CA), as described previously [91]. The PCR amplifications were performed using the GenRacer 5′ primer and the gene-specific primer (Table 2). Nested PCR amplifications were performed using the GeneRacer 5′ nested primer and the nested gene-specific nested primer (Table 2). PCR reactions were separated by agarose gel electrophoresis, and distinct bands of the appropriate size for miRNA-mediated cleavage were purified (excised gel slices corresponded to a size range of ~200 base pairs), cloned, and sequenced.

### Date access

The sRNA sequence data from this study have been submitted to Gene Expression Omnibus (GEO) under accession NO. GSE61719 at website: http://www.ncbi.nlm.nih.gov/geo/query/acc.cgi?token=uvmvkkcixrepvyv&acc=GSE61719.

## Additional files

Additional file 1: Table S1. Mature sequences of conserved miRNA in *C. sinensis* with cold stress. Additional file 2: Figure S1. Mature and precursor sequences and the predicted stem-loop structures of newly identified miRNAs from *C. sinensis.*
Additional file 3: Table S2. Novel miRNAs and potentially novel miRNAs identified from predicted RNA hairpins in *C. sinensis* with cold stress*.*
Additional file 4: Figure S2. t-plot for targets of the known miRNAs found in + C and -C libraries of *C. sinensis*. Signature abundance throughout the length of the transcript is show. Arrows indicate signature consistent with miRNA-directed cleavage. Additional file 5: Figure S3. t-plot for targets of the new miRNA candidates found in + C and -C libraries of *C. sinensis*. Signature abundance throughout the length of the transcript is show. Arrows indicate signature consistent with miRNA-directed cleavage. Additional file 6: Table S3. Relative expression analysis of cold-responsive miRNAs in ‘Yingshuang’. The threshold of *p* value was set 0.01, ***indicated *p* value less than 0.0001, **indicated *p* value from 0.0001 to 0.001, *indicated *p* value from 0.001 to 0.01, respectively. Additional file 7: Table S4. Relative expression analysis of cold-responsive miRNAs in ‘Baiye 1’. The threshold of *p* value was set 0.01, ***indicated *p* value less than 0.0001, ** indicated *p* value from 0.0001 to 0.001, *indicated *p* value from 0.001 to 0.01, respectively. Additional file 8: Figure S4. Microarray analysis of the known and new miRNAs from tea plant cultivar ‘Yingshaung’ treated with cold and cold-free. Additional file 9: Figure S5. Microarray analysis of the known and new miRNAs from tea plant cultivar ‘Baiye 1’ treated with cold and cold-free. Additional file 10: Table S5. Targets for conserved miRNAs from *C. sinensis* treated with cold (+C) and without cold (−C). Additional file 11: Table S6. Targets for new miRNA candidates from *C. sinensis* treated with cold (+C) and without cold (−C). Additional file 12: Table S7. Sequences of primers used for the reverse transcription and Quantitative real-time PCR experiments.

## Abbreviations

MiRNA: MicroRNA
*C. sinensis*: *Camellia sinensis*
AGO: Argonaute
RISC: RNA-induced silencing complex
qRT-PCR: Quantitative real-time PCR
rRNA: Ribosomal RNA
tRNA: Transfer RNA
snoRNA: Small nucleolar RNA
snRNA: Small nuclear RNA
ncRNA: Non-coding RNA
MFE: Minimum free energy
MFEI: Minimum free energy index
*A. thaliana*: *Arabidopsis thaliana*
LEA: Late embryogenesis abundant
RLM-5′ RACE: 5′ RNA ligase-mediated RACE
GO: Gene ontology
sRNA: Small RNA
EST: Expressed sequence tag
NADPH: Nicotinamide adenine dinucleotide 2′-phosphate reduced tetrasodium salt
ARF: Auxin-responsive factor
AUX/IAA: Auxin/ indole-3-acetic acid
WBP: WW-domain-binding protein
YS: Yingshuang
BY: Baiye 1
TBE: Tris-Borate-EDTA
RT-PCR: Reverse transcription PCR
CT: Cycle threshold
