# Supplementary material for: Identification and characterization of cold-responsive microRNAs in tea plant (Camellia sinensis) and their targets using high-throughput sequencing and degradome analysis
Source: BMC Plant Biol. 2014 Oct 21;14:271. doi: 10.1186/s12870-014-0271-x (PMC4209041; doi:10.1186/s12870-014-0271-x)
Supplement: Additional file 1: Table S1. — Mature sequences of conserved miRNA in C. sinensis with cold stress. [file 12870_2014_271_MOESM1_ESM.pdf]

Table S1 Mature sequence of conserved miRNA in *C. sinensis* with cold stress

miRNA Family	miRNA name	miR_seq	Type	Len	Reads	GenomeID	Strand	miRNA Reference	Hairpin Length	CG%	dG	MFEI	miRNA*
csn-miR156	csn-miR156a-1	UUGACAGAAGAUAGAGAGCAC	5'	21	346	scaffold_5	-	ptc-MIR156g	119	46.3	-58.5	1	Y
	csn-miR156a-2	UUGACAGAAGAUAGAGAGCAC	5'	21	346	scaffold_4	-	ptc-MIR156h	99	44.7	-46.3	1	Y
	csn-miR156b	CUGACAGAAGAGAGUGAGCAC	5'	21	607	scaffold_18	-	ptc-MIR156f	92	43.6	-54.1	1.2	Y
	csn-miR156c	UGACAGAAGAGAGUGAGCACA	5'	21	54	scaffold_18	+	ptc-MIR156e	91	37	-42.4	1.1	Y
	csn-miR156d	GCUCACUUCUCUUUCUGUCAUU	3'	22	1	scaffold_18	+	cme-MIR156j	91	37	-42.4	1.1	Y
	csn-miR156e-1	UGUUGACAGAAGAAAGAGAGC	5'	21	2	scaffold_4	-	aly-MIR156h	99	44.8	-46.3	1	N
	csn-miR156e-2	UGUUGACAGAAGAAAGAGAGC	5'	21	2	scaffold_5	-	smo-MIR156c	125	46.6	-66.8	1.1	N
	csn-miR156f	CUGACAGAAGAGAGUGAGCACU	5'	22	2	scaffold_6	-	cca-MIR156b	127	45.9	-64.4	1.1	N
csn-miR159	csn-miR159-1	UUUGGAUUGAAGGGAGCUCUA	3'	21	17325	scaffold_15	+	ptc-MIR159b	176	45.5	-78.4	0.9	Y
	csn-miR159-2	UUUGGAUUGAAGGGAGCUCUA	3'	21	17325	scaffold_12	-	ptc-MIR159a	174	44.3	-75.4	0.9	Y
csn-miR160	csn-miR160-1	UGCCUGGCUCCCUGUAUGCCA	5'	21	59	scaffold_8	-	ptc-MIR160b	86	53.1	-51.5	1	Y
	csn-miR160-2	UGCCUGGCUCCCUGUAUGCCA	5'	21	59	scaffold_1	+	ptc-MIR160a	95	51	-56	1.1	N
	csn-miR160-3	UGCCUGGCUCCCUGUAUGCCA	5'	21	59	scaffold_10	+	ptc-MIR160c	86	52	-52.1	1	Y
csn-miR162	csn-miR162a-1	UCGAUAAACCUCUGCAUCCAG	3'	21	561	scaffold_15	+	ptc-MIR162b	101	44.9	-42.3	0.9	Y
	csn-miR162a-2	UCGAUAAACCUCUGCAUCCAG	3'	21	561	scaffold_12	+	ptc-MIR162a	105	47.7	-43.5	0.9	Y
	csn-miR162b	UCGAUAAACCUCUGCAUCCAGUG	3'	23	3	scaffold_15	+	bdi-MIR162	121	44.1	-48.2	0.9	N
csn-miR164	csn-miR164a	UGGAGAAGCAGGGCACGUGCA	5'	21	869	scaffold_13	-	ptc-MIR164c	80	57.3	-41.1	0.9	Y
	csn-miR164b-1	UGGAGAAGCAGGGCACGUGCG	5'	21	4	scaffold_13	-	vvi-MIR164a	98	54.8	-50.3	0.9	N
	csn-miR164b-2	UGGAGAAGCAGGGCACGUGCG	5'	21	4	scaffold_14	+	aly-MIR164c	172	53.4	-74	0.8	N
	csn-miR164c	CAUGUGCCCGUCUUCCCCAUC	3'	21	256	scaffold_16	+	rco-MIR164c	141	45.8	-55.2	0.8	N
csn-miR166	csn-miR166a-1	UCGGACCAGGCUUCAUUCCCC	3'	21	159305	scaffold_5	-	ptc-MIR166d	101	47.6	-50.3	1	Y

	csn-miR166a-2	UCGGACCAGGCUUCAUUCCCC	3'	21	159305	scaffold_8	-	ptc-MIR166g	96	51.5	-54.9	1	Y
	csn-miR166a-3	UCGGACCAGGCUUCAUUCCCC	3'	21	159305	scaffold_7	-	ptc-MIR166f	103	46.7	-51.3	1	Y
	csn-miR166a-4	UCGGACCAGGCUUCAUUCCCC	3'	21	159305	scaffold_2	+	ptc-MIR166c	123	41.8	-57.8	1	Y
	csn-miR166a-5	UCGGACCAGGCUUCAUUCCCC	3'	21	159305	scaffold_14	+	ptc-MIR166j	132	39.9	-45.1	0.8	Y
	csn-miR166a-6	UCGGACCAGGCUUCAUUCCCC	3'	21	159305	scaffold_1	+	ptc-MIR166a	153	34.1	-52.8	0.9	Y
	csn-miR166b-1	UCUCGGACCAGGCUUCAUUCC	3'	21	327	scaffold_944	-	ptc-MIR166n	326	38.1	-85.5	0.7	Y
	csn-miR166b-2	UCUCGGACCAGGCUUCAUUCC	3'	21	327	scaffold_13	+	ptc-MIR166o	313	37.2	-79.6	0.7	Y
	csn-miR166c	GGAAUGUUGUCUGGCUCGAGG	5'	21	759	scaffold_7	-	ptc-MIR166q	144	44.4	-67.1	1	Y
	csn-miR166d-1	GGACCAGGCUUCAUUCCUC	3'	19	225	scaffold_12	-	vvi-MIR166b	92	45.6	-39	0.8	Y
	csn-miR166d-2	GGACCAGGCUUCAUUCCUC	3'	19	225	scaffold_15	-	osa-MIR166i	147	45.1	-55.9	0.8	N
	csn-miR166e	UCGGACCAGGCUUCAUUCCCU	3'	21	753	scaffold_5	-	vvi-MIR166f	101	47.1	-50.3	1	Y
	csn-miR166f	GAAUGUUGGCUGGCUCGAGGC	5'	21	4	scaffold_7	-	zma-MIR166m	113	46.2	-56.7	1	N
	csn-miR166g	UCGGACCAGGCUUCAUUCCCU	3'	21	753	scaffold_7	-	nta-MIR166h	113	46.2	-56.7	1	Y
	csn-miR166h-1	UCGGACCAGGCUUCAUUCCUC	3'	21	59653	scaffold_15	-	vvi-MIR166b	136	50.7	-57.3	0.8	N
	csn-miR166h-2	UCGGACCAGGCUUCAUUCCUC	3'	21	59653	scaffold_8	-	vvi-MIR166b	96	52	-54.9	1	N
	csn-miR166i	UCGGACCAGGCUUCAUCCCCC	3'	21	79	scaffold_5	-	mdm-MIR166f	164	45.3	-64	0.8	N
csn-miR167	csn-miR167a-5p	UGAAGCUGCCAGCAUGAUCUGU	5'	22	64	76159_gi|319832051	-	tcc-MIR167c	102	42.6	-49.3	1.1	Y
	csn-miR167a-3p	AGAUCAUAUGGCAGCUUCACC	3'	21	68	76159_gi|319832051	-	lja-MIR167	102	42.6	-49.3	1.1	Y
	csn-miR167b-1	UGAAGCUGCCAGCAUGAUCUA	5'	21	90	scaffold_5	+	ptc-MIR167d	103	47.6	-38.9	0.8	Y
	csn-miR167b-2	UGAAGCUGCCAGCAUGAUCUA	5'	21	90	scaffold_5	+	ptc-MIR167c	106	44.4	-51.7	1.1	Y
	csn-miR167c-1	UGAAGCUGCCAGCAUGAUCUC	5'	21	11	scaffold_2	-	vvi-MIR167c	108	46.5	-49.5	0.9	N
	csn-miR167c-2	UGAAGCUGCCAGCAUGAUCUC	5'	21	11	scaffold_2	-	vvi-MIR167d	82	45.5	-38.4	1	N
	csn-miR167d	UGAAGCUGCCAGCAUGAUCUAG	5'	22	4319	76159_gi|319869237	+	vvi-MIR167d	134	50.7	-61.5	0.9	N
	csn-miR167e	AGAUCAUGUGGUAGCUUCACC	3'	21	5	scaffold_5	+	nta-MIR167d	108	43.9	-53.5	1.1	N

csn-miR168	csn-miR168a-1	UCGCUUGGUGCAGGUCGGGAA	5'	21	1884	scaffold_3	-	ptc-MIR168a	156	46.9	-58	0.8	Y
	csn-miR168a-2	UCGCUUGGUGCAGGUCGGGAA	5'	21	1884	scaffold_4	-	ptc-MIR168b	134	46.3	-53.3	0.8	Y
	csn-miR168b	CCCGCCUUGCAUCAACUGAAUU	3'	22	61	scaffold_4	-	nta-MIR168a	153	49	-71.6	0.9	Y
csn-miR169	csn-miR169a-1	UAGCCAAGGAUGACUUGCCUCC	5'	22	6	scaffold_1	-	ptc-MIR169j	134	41.6	-50.5	0.9	Y
	csn-miR169a-2	UAGCCAAGGAUGACUUGCCUCC	5'	22	6	scaffold_17	-	ptc-MIR169l	140	38.5	-49.8	0.9	Y
	csn-miR169b	AGGCAAUCUCCUUGGCUAAC	3'	20	49	scaffold_1	-	ptc-MIR169i	117	44.7	-52.2	0.9	N
	csn-miR169c	CGGCAAGUUGUCAUUGGCUACA	3'	22	29	76159_gi|319831552	+	aly-MIR169a	140	47.9	-79.6	1.1	N
csn-miR171	csn-miR171a-1	UGAUUGAGCCGUGCCAAUAUC	3'	21	768	scaffold_21	+	ptc-MIR171b	99	36.9	-53.7	1.4	Y
	csn-miR171a-2	UGAUUGAGCCGUGCCAAUAUC	3'	21	768	scaffold_15	+	ptc-MIR171h	93	43	-44.4	1	N
	csn-miR171b	CGAUGUUGGUGAGGUUCAAUC	5'	21	5	scaffold_18	-	ptc-MIR171d	93	42	-37.4	0.9	Y
	csn-miR171c-1	UGAGCCGAAUCAAUAUCACU	3'	20	2	scaffold_12	-	ptc-MIR171m	21	46.5	-43.9	0.9	N
	csn-miR171c-2	UGAGCCGAAUCAAUAUCACU	3'	20	2	scaffold_12	-	ptc-MIR171n	101	45.5	-42.6	0.9	N
	csn-miR171d	UAUUGGCCUGGUUCACUCAGA	5'	21	90	scaffold_5	+	gma-MIR171j	167	34.3	-49.612	0.8	Y
	csn-miR171e-5p	CGAUGUUGGUGAGGUUCAAUC	5'	21	5	scaffold_6	-	gma-MIR171k	99	40.4	-43.2	1	Y
	csn-miR171e-3p	UUGAGCCGCGCCAAUAUCACU	3'	21	3	scaffold_6	-	gma-MIR171k	99	40.4	-43.2	1	Y
	csn-miR171f	UUGAGCCGCGCCAAUAUCACU	3'	21	3	scaffold_18	-	vvi-MIR171f	93	42	-37.4	0.9	Y
	csn-miR171g	UAUUGGUGCGGUUCAAUAAGA	5'	21	2	scaffold_21	+	vvi-MIR171c	84	42.9	-48.1	1.3	N
csn-miR172	csn-miR172a-1	AGAAUCUUGAUGAUGCUGCAU	3'	21	168	scaffold_8	+	ptc-MIR172a	118	38.8	-54.3	1.1	Y
	csn-miR172a-2	AGAAUCUUGAUGAUGCUGCAU	3'	21	168	scaffold_10	-	ptc-MIR172c	117	40.6	-53.7	1	Y
	csn-miR172b	UGCGGCAUCAUCAAGAUUCAC	5'	21	79	scaffold_1	+	ptc-MIR172d	171	48.9	-99.6	1.1	Y
	csn-miR172c	UGGGAAUCCUGAUGAUGCUGC	3'	21	5	scaffold_9	+	ptc-MIR172e	132	45.9	-57.5	0.9	N
	csn-miR172d	AGAAUCUUGAUGAUGCUGCAU	3'	21	168	scaffold_9	-	ptc-MIR172b	133	41.4	-54.6	0.9	N
	csn-miR172e	AGAAUCUUGAUGAUGCUGCAUU	3'	22	11	scaffold_10	-	nta-MIR172h	139	39.3	-62.4	1.1	Y
csn-miR319	csn-miR319a-5p	AGCUGCCGACUCAUUCAUUCU	5'	21	2	scaffold_19	+	ptc-MIR319g	191	46.1	-78.6	0.8	N

	csn-miR319a-3p	UUGGACUGAAGGGAGCUCC	3'	19	2	scaffold_19	+	ptc-MIR319g	191	46.1	-78.6	0.8	N
	csn-miR319b-1	UUGGACUGAAGGGAGCUCCC	3'	20	283	scaffold_19	+	ptc-MIR319d	192	46.6	-81.9	0.9	N
	csn-miR319b-2	UUGGACUGAAGGGAGCUCCC	3'	20	283	scaffold_13	+	ptc-MIR319c	195	43.9	-81.1	0.9	N
	csn-miR319b-3	UUGGACUGAAGGGAGCUCCC	3'	20	283	scaffold_3	+	ptc-MIR319b	184	34.6	-65.3	1	Y
	csn-miR319c	UUGGACUGAAGGGAGCUCC	3'	19	2	scaffold_13	+	ptc-MIR319f	200	44.6	-87.6	1	N
	csn-miR319d	AGCUGCCGACUCAUUCAUUCU	5'	21	2	scaffold_13	+	ptc-MIR319f	200	44.6	-87.6	1	N
csn-miR390	csn-miR390a-1	AAGCUCAGGAGGGAUAGCGCC	5'	21	351	scaffold_1	+	ptc-MIR390a	117	42	-48.8	1	N
	csn-miR390a-2	AAGCUCAGGAGGGAUAGCGCC	5'	21	351	scaffold_9	+	ptc-MIR390c	121	40.7	-58.9	1.2	N
	csn-miR390b	CGCUAUCUAUCCUGAGUUUCA	3'	21	12	scaffold_6	+	gma-MIR390c	123	42.6	-53.6	1	Y
	csn-miR390c	CUGGCGCUAUCUAUCCUGAGU	3'	21	3	scaffold_1	+	tcc-MIR390b	136	42.3	-64.1	1.1	N
csn-miR393	csn-miR393-1	UCCAAAGGGAUCGCAUUGAUC	5'	21	58	scaffold_8	+	ptc-MIR393a	83	48.2	-44.6	1.1	Y
	csn-miR393-2	UCCAAAGGGAUCGCAUUGAUC	5'	21	58	scaffold_10	-	ptc-MIR393b	82	50	-44.2	1.1	Y
csn-miR394	csn-miR394-1	UUGGCAUUCUGUCCACCUCC	5'	20	7104	scaffold_2	-	ptc-MIR394a	147	44.2	-79.6	1.2	Y
	csn-miR394-2	UUGGCAUUCUGUCCACCUCC	5'	20	7104	scaffold_5	+	ptc-MIR394b	147	41.5	-62.2	1	N
csn-miR395	csn-miR395a-1	CUGAAGUGUUUGGGGGAACUC	3'	21	1121	scaffold_761	+	ptc-MIR395j	91	49	-43.5	0.9	Y
	csn-miR395a-2	CUGAAGUGUUUGGGGGAACUC	3'	21	1121	scaffold_6	+	ptc-MIR395c	90	43.6	-42.2	1	Y
	csn-miR395a-3	CUGAAGUGUUUGGGGGAACUC	3'	21	1121	scaffold_16	+	ptc-MIR395g	91	48	-42.3	0.9	Y
	csn-miR395b	CUGAAGGGUUUGAAGGAACUC	3'	21	1	scaffold_2	+	ptc-MIR395a	83	41.2	-75.1	0.8	N
	csn-miR395c	CUGAAGUGUUUGGGGGAACUCUU	3'	23	2	scaffold_761	-	mtr-MIR395p	124	51.5	-61	0.9	N
csn-miR396	csn-miR396a-1	UUCCACAGCUUUCUUGAACUG	5'	21	37418	scaffold_6	+	ptc-MIR396a	117	37.4	-46.7	1	Y
	csn-miR396a-2	UUCCACAGCUUUCUUGAACUG	5'	21	37418	scaffold_18	+	ptc-MIR396b	130	37.1	-49.8	1	Y
	csn-miR396b-1	UUCCACAGCUUUCUUGAACUU	5'	21	11614	scaffold_6	-	ptc-MIR396d	166	40.4	-70.5	1.1	N
	csn-miR396b-2	UUCCACAGCUUUCUUGAACUU	5'	21	11614	scaffold_18	-	ptc-MIR396e	141	37.9	-58.1	1.1	Y
	csn-miR396c	ACAGCUUUCUUGAACUGCAUC	5'	21	160	scaffold_18	+	mdm-MIR396b	170	39.2	-61.9	0.9	Y

	csn-miR396d-1	UUCCACAGCUUUCUUGAACUUU	5'	22	168	scaffold_18	-	nta-MIR396a	145	37.7	-62.5	1.1	Y
	csn-miR396d-2	UUCCACAGCUUUCUUGAACUUU	5'	22	168	scaffold_6	-	nta-MIR396a	156	41.5	-65.6	1	N
	csn-miR396e	ACAGCUUUCUUGAACUGCAUC	5'	21	160	scaffold_6	+	mdm-MIR396b	117	37.3	-46.7	1	Y
	csn-miR396f	UCCACAGCUUUCUUGAACUUUC	5'	22	1	scaffold_3	+	vvi-MIR396b	98	42.3	-44.4	1	N
csn-miR398	csn-miR398	UGUGUUCUCAGGUCGCCCCUG	3'	21	108	scaffold_9	-	ptc-MIR398c	94	55.7	-44.4	0.8	N
csn-miR399	csn-miR399a	UGCCAAAGGAGAGUUGCCCUG	3'	21	2	scaffold_4	-	vvi-MIR399b	106	46.4	-45.8	0.9	N
	csn-miR399b	UGCCAAAGGAGAGUUGCCCUU	3'	21	7	scaffold_12	-	cme-MIR399g	120	42.1	-52	1	N
csn-miR403	csn-miR403	UGUUAGAUUCACGCACAAACU	3'	21	3	scaffold_10	-	vvi-MIR403d	111	45.3	-48.6	0.9	N
csn-miR408	csn-miR408	UGCACUGCCUCUUCCCUGGCUC	3'	22	210	scaffold_2	+	nta-MIR408	188	47.9	-83.8	0.9	N
csn-miR530	csn-miR530	UGCAUUUGCACCUGCACCUGA	5'	21	100	scaffold_9	-	ptc-MIR530a	144	41	-73.5	1.2	N
csn-miR5072	csn-miR5072	AUCCCCAGCGGAGUCGCCA	3'	19	5	scaffold_1	-	osa-MIR5072	61	49.3	-23.6	0.7	N
csn-miR5368	csn-miR5368	UCUGGAAGAGCUAGAAUUCUAACC	3'	24	55	47452_gi|393741702	+	gma-MIR5368	170	57.1	-76.2	0.8	N
csn-miR6478	csn-miR6478	CCGACCUUAGCUCAGUUGGUAGA	5'	23	215	scaffold_9	-	ptc-MIR6478	138	47.8	-44.3	0.7	N

Note: ‘Y’ and ‘N’ with and without express signal
