# Supplementary material for: Identification and characterization of cold-responsive microRNAs in tea plant (Camellia sinensis) and their targets using high-throughput sequencing and degradome analysis
Source: BMC Plant Biol. 2014 Oct 21;14:271. doi: 10.1186/s12870-014-0271-x (PMC4209041; doi:10.1186/s12870-014-0271-x)
Supplement: Additional file 2: Figure S1. — Mature and precursor sequences and the predicted stem-loop structures of newly identified miRNAs from C. sinensis. [file 12870_2014_271_MOESM2_ESM.pdf]

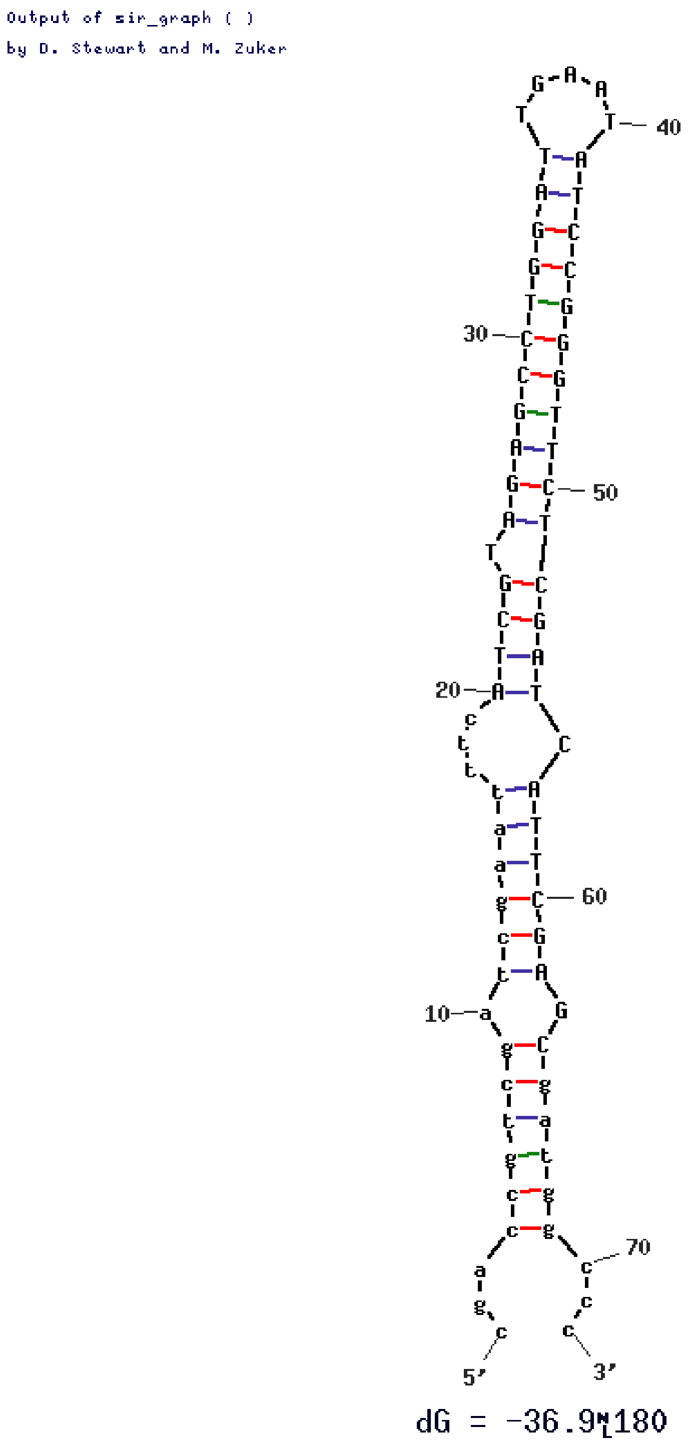

csn-smR1

AUCCGGGUUCUCGAUCAUUCGAGC

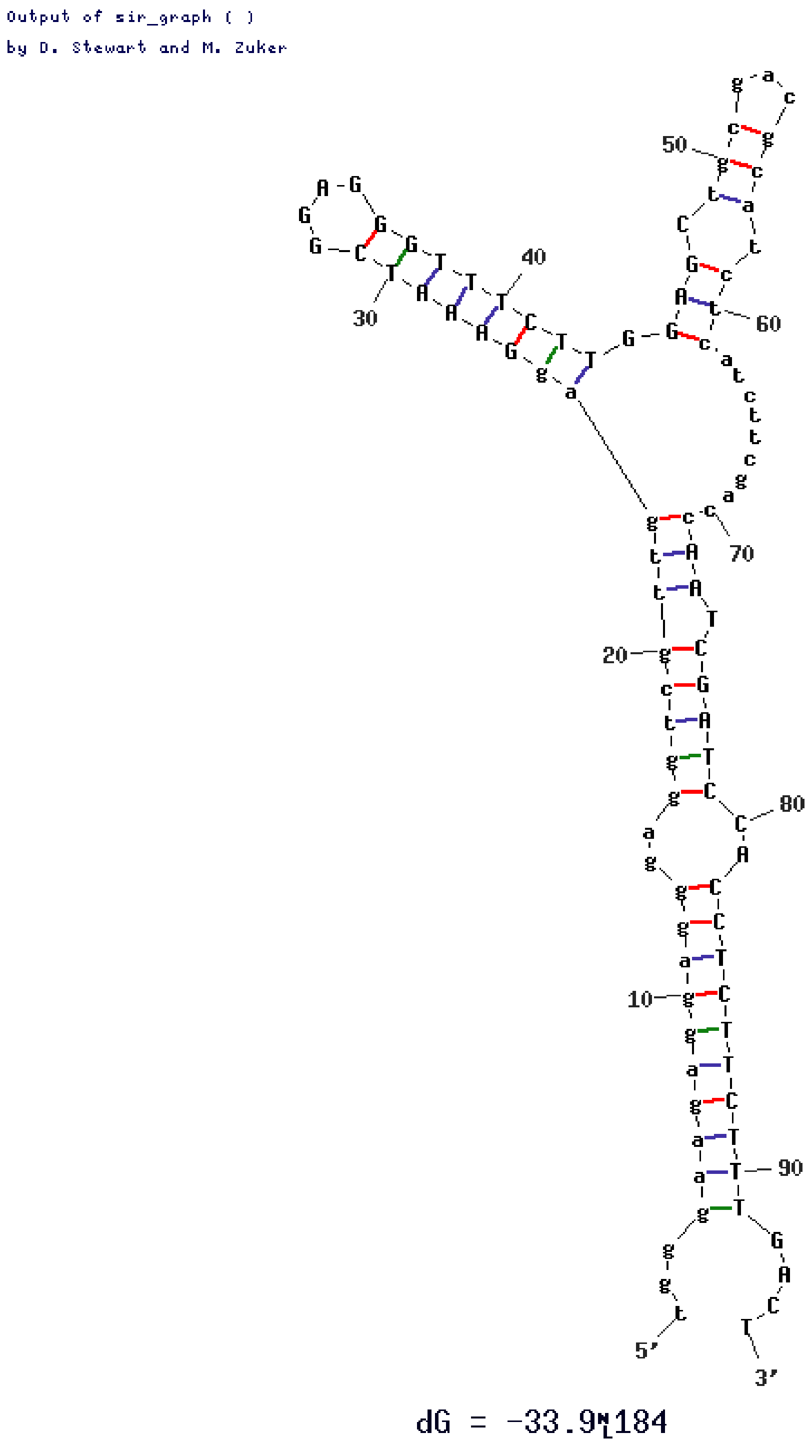

csn-smR2
AAUCGAUCCACCUCUUCUUUGACU

Output of sir_graph ( )
by D. Stewart and M. Zuker

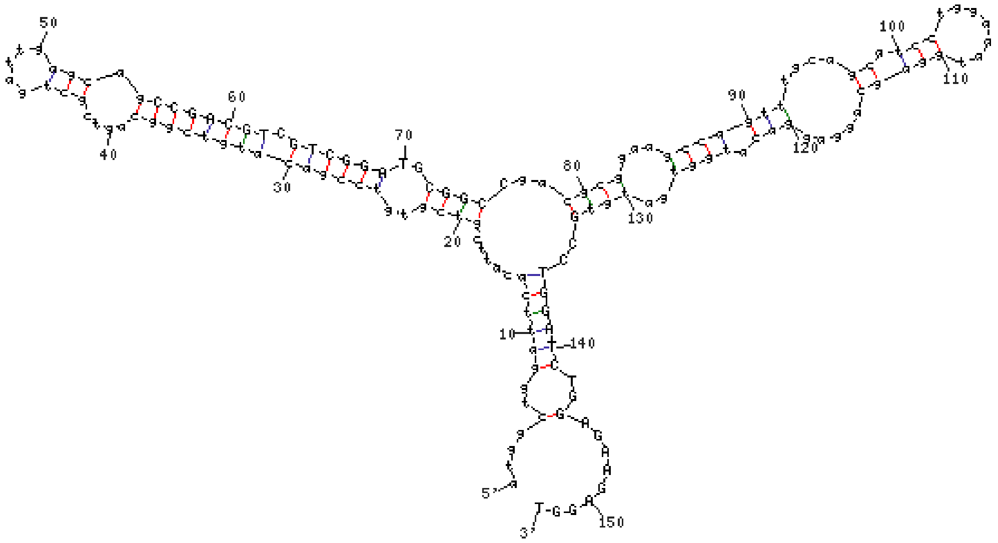

dG = −59.8[illegible]187

csn-smR3
CCGACGUCGUCGGAUGCGGCC

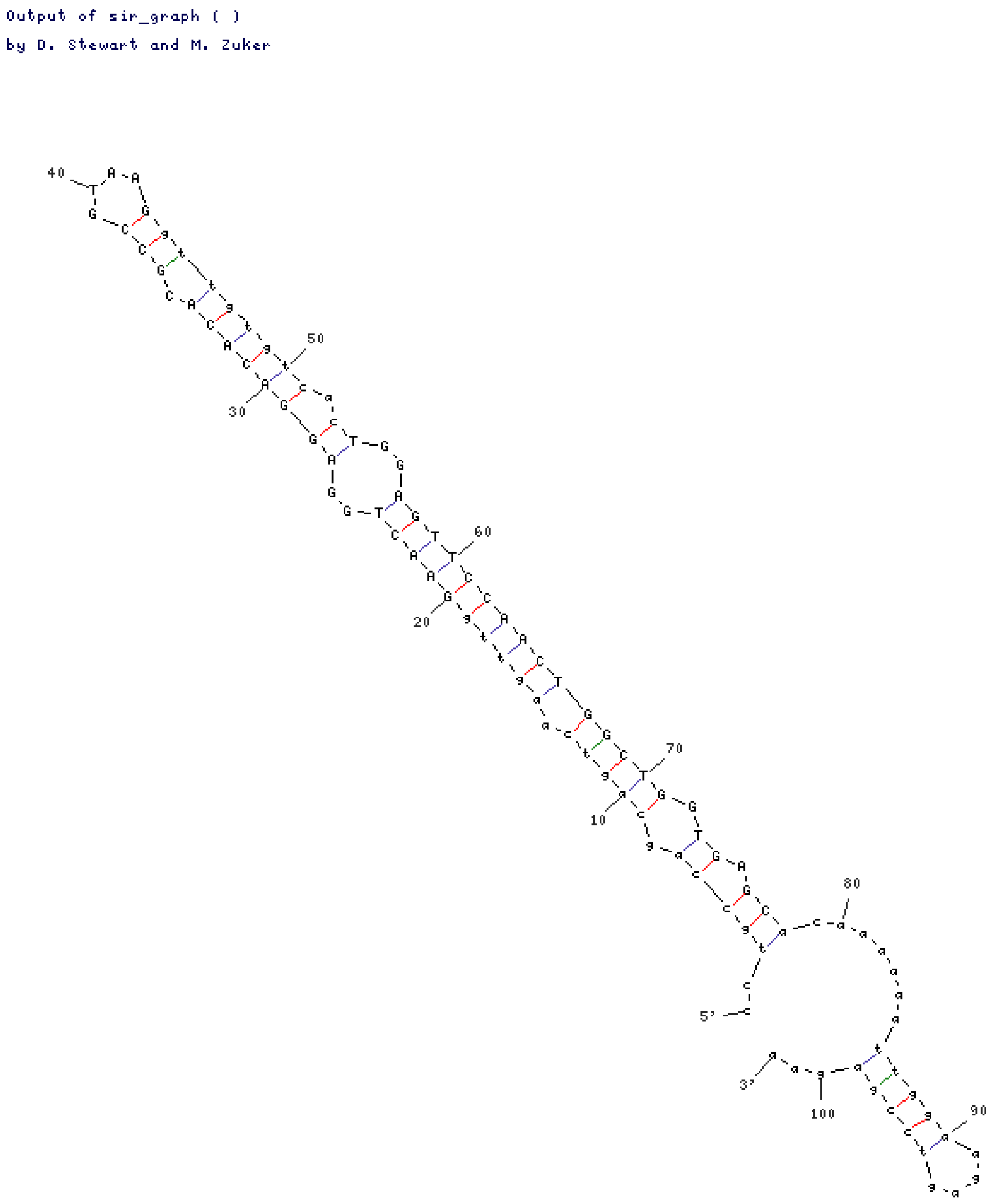

dG = -48.7[illegible]191

csn-smR4-5p-1
GAACUGGAGGACACACGCCGUAAG

Output of sir_graph ( )
by D. Stewart and M. Zuker

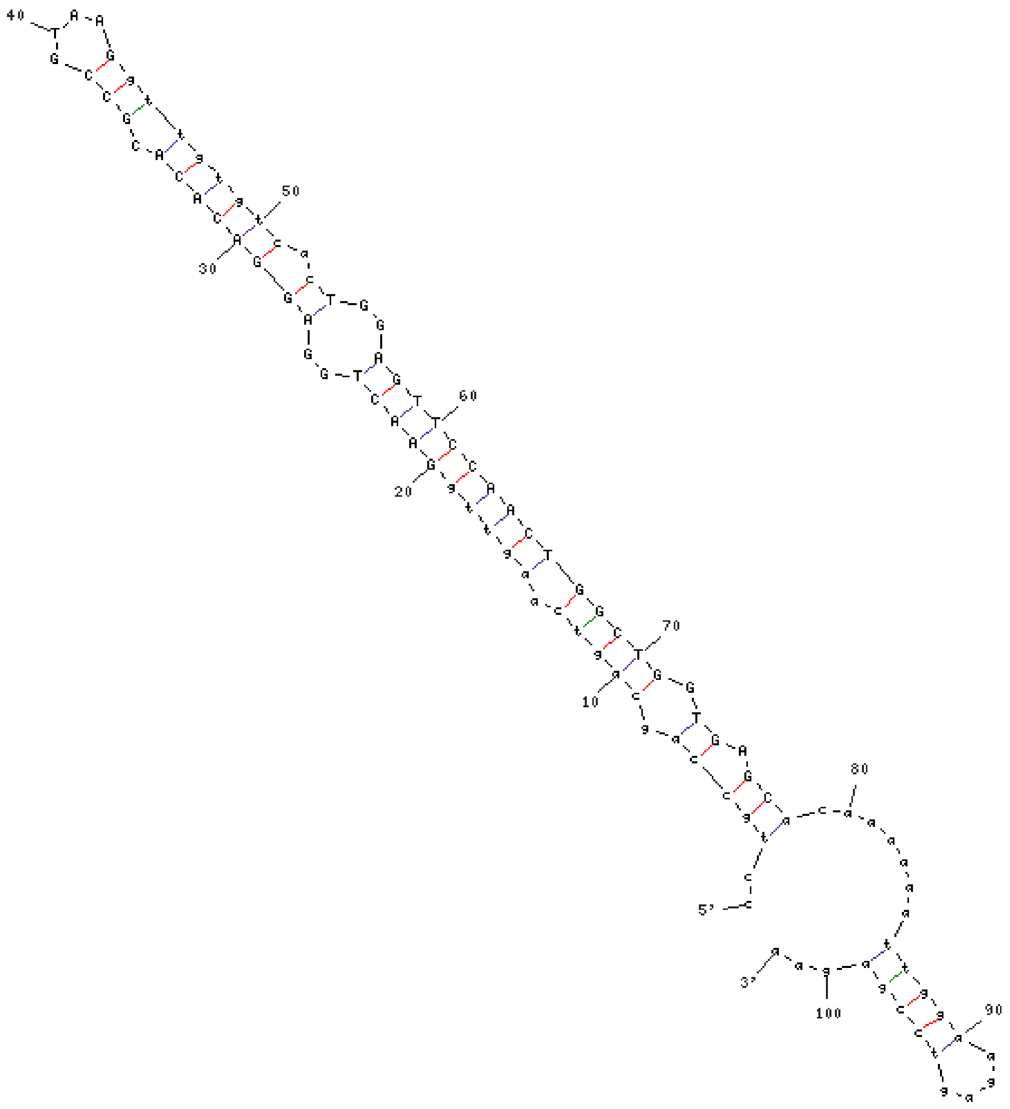

dG = -48.7[illegible]192

csn-smR4-3p-1
UGGAGUUCCAACUGGCUGGGGAGC

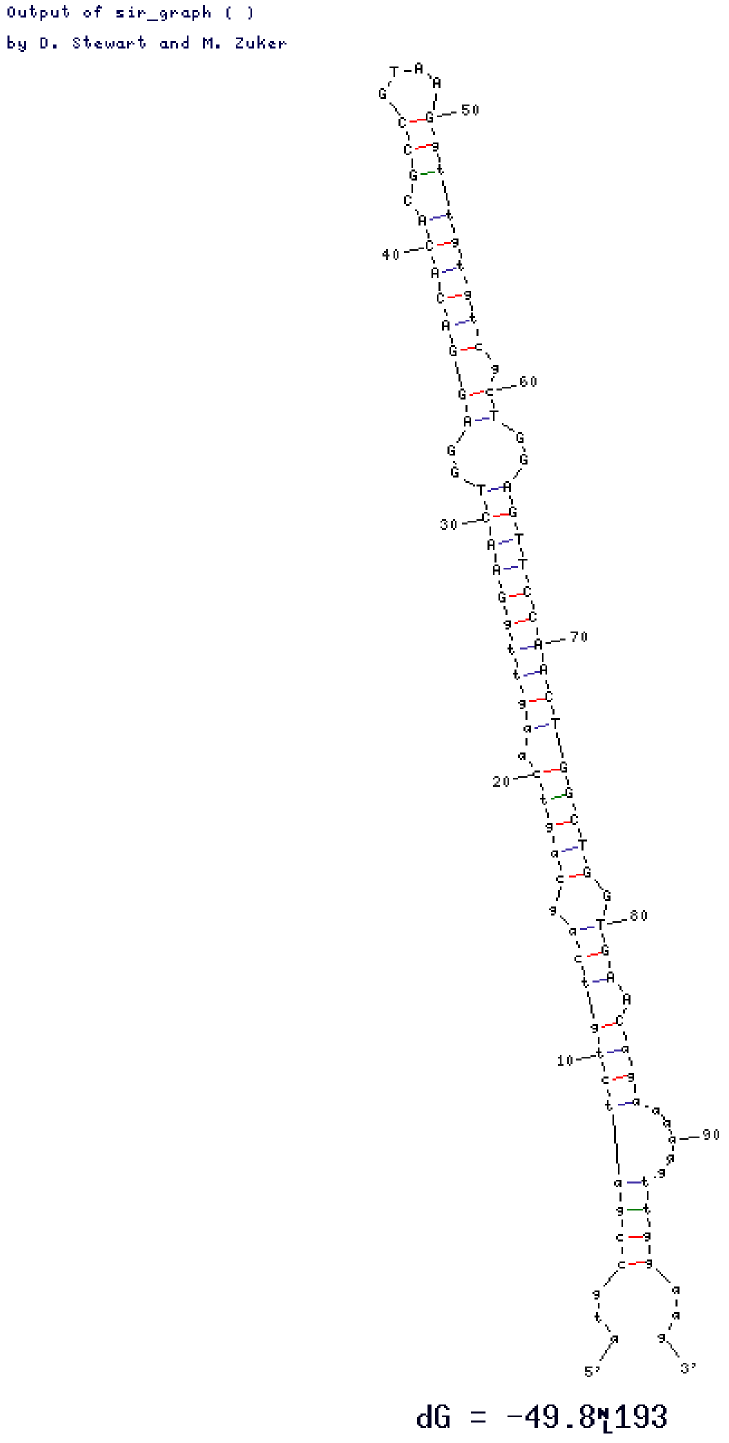

csn-smR4-5p-2
GAACUGGAGGACACACGCCGUAAG

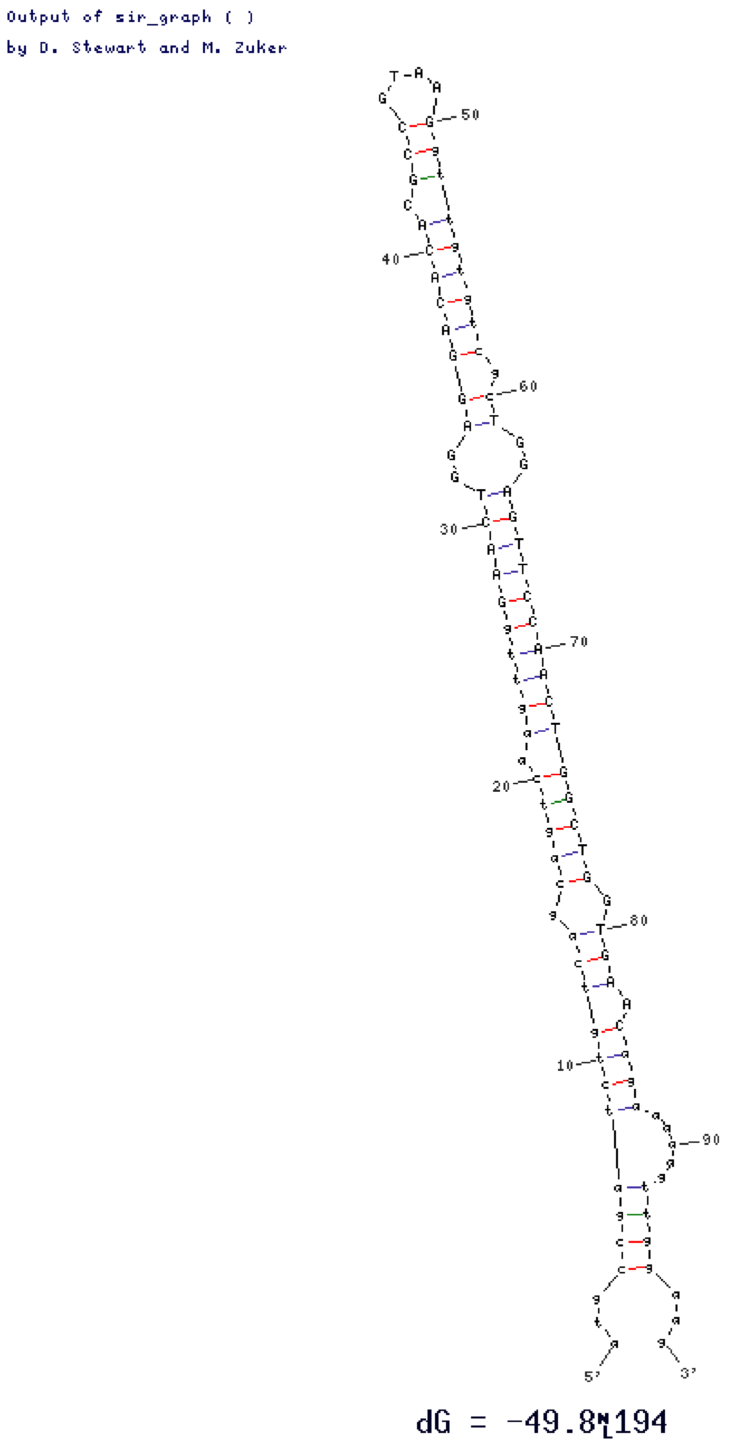

csn-smR4-3p-2
UGGAGUUCCAACUGGCUGGGGAGC

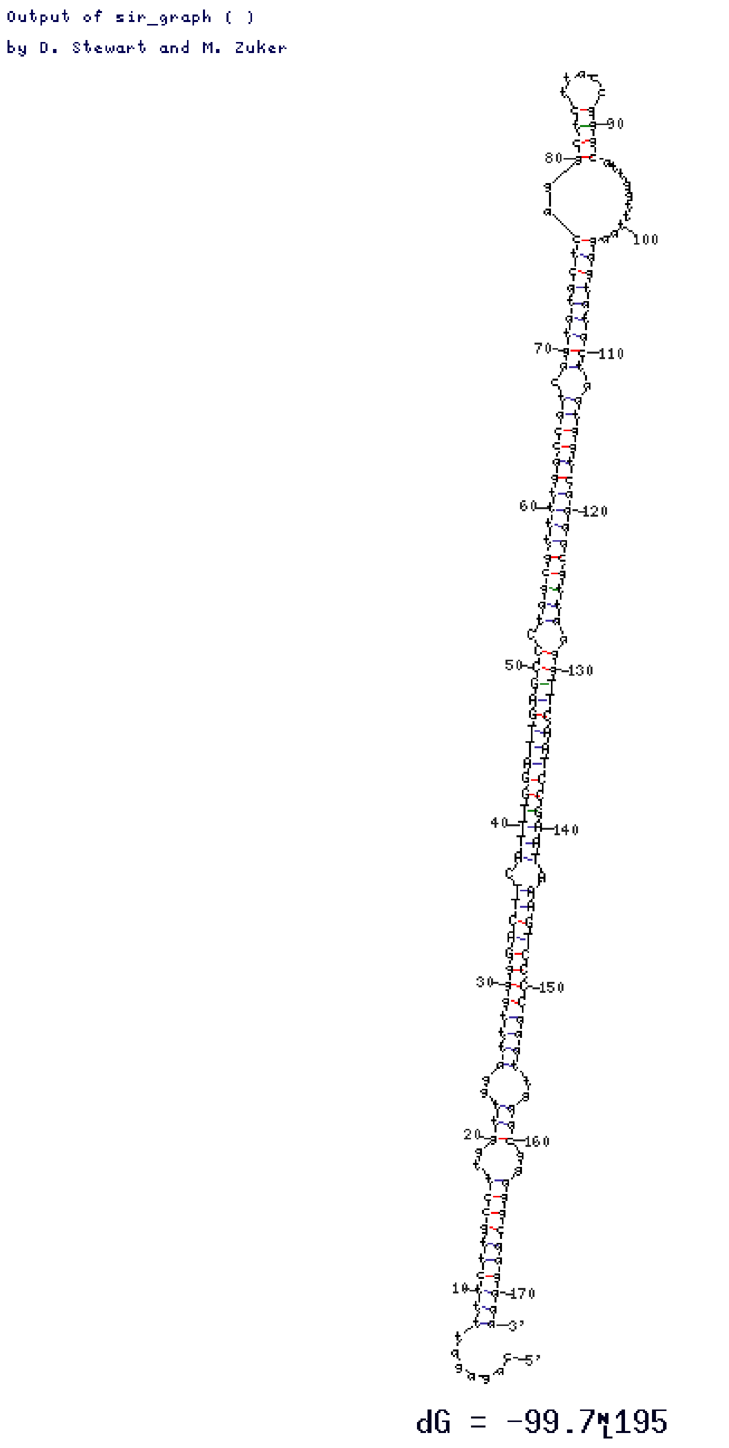

csn-smR5-5p-1
GACUUCAUUUGGAUUGAGCCC

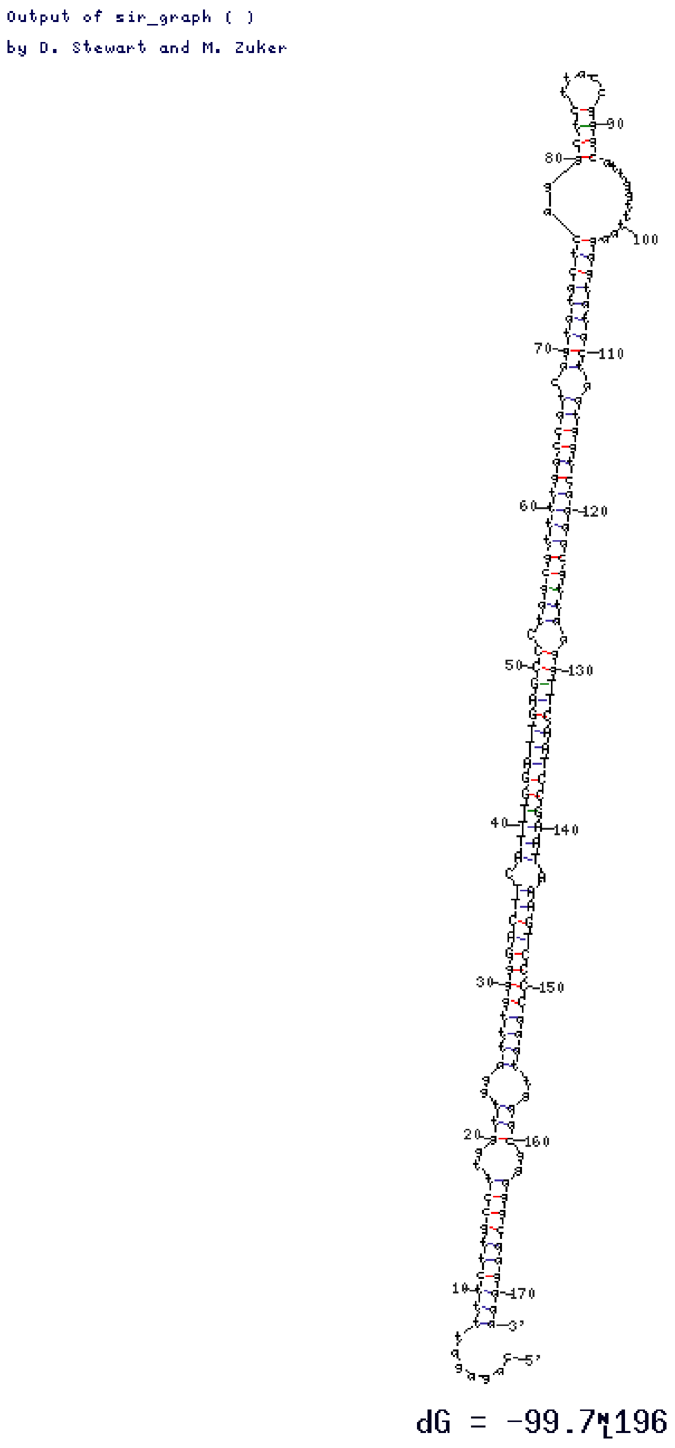

csn-smR5-3p-1
UUCAAUCCGAAUAAAGUCCCC

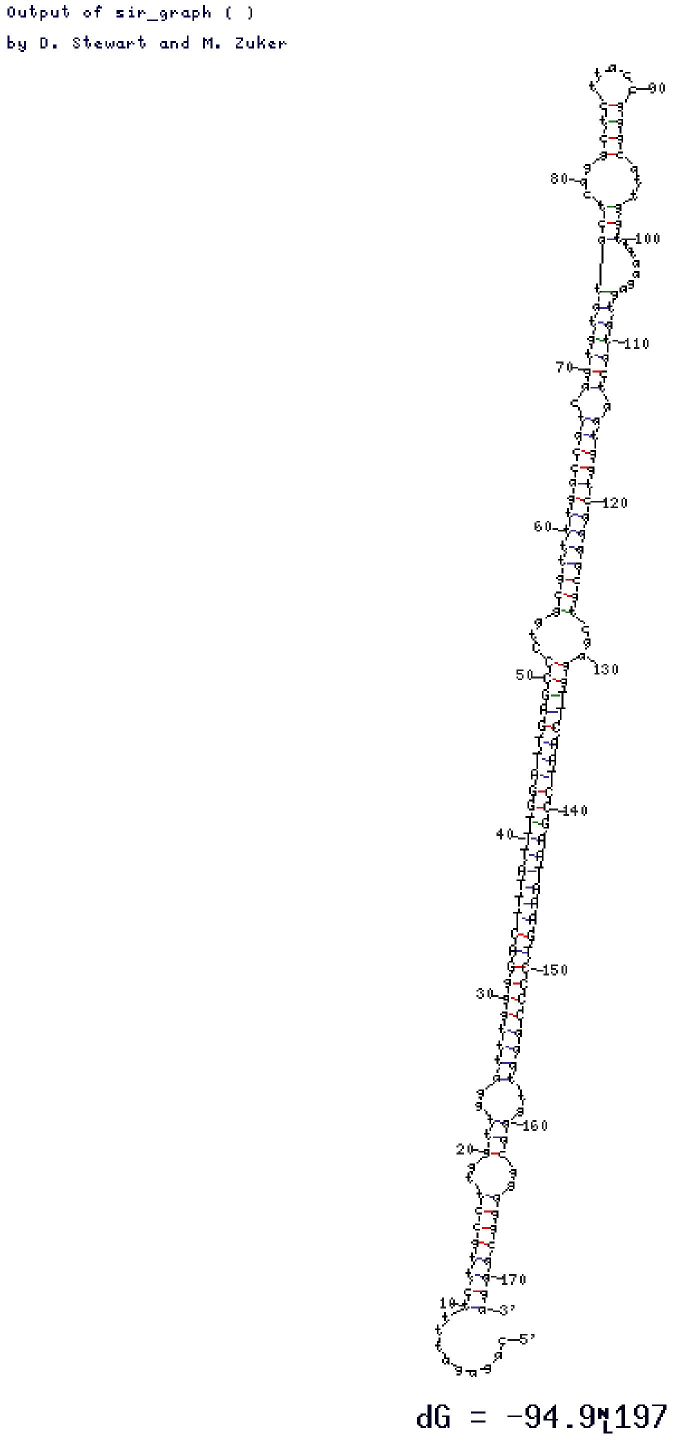

csn-smR5-5p-2
GACUUCAUUUGGAUUGAGCCC

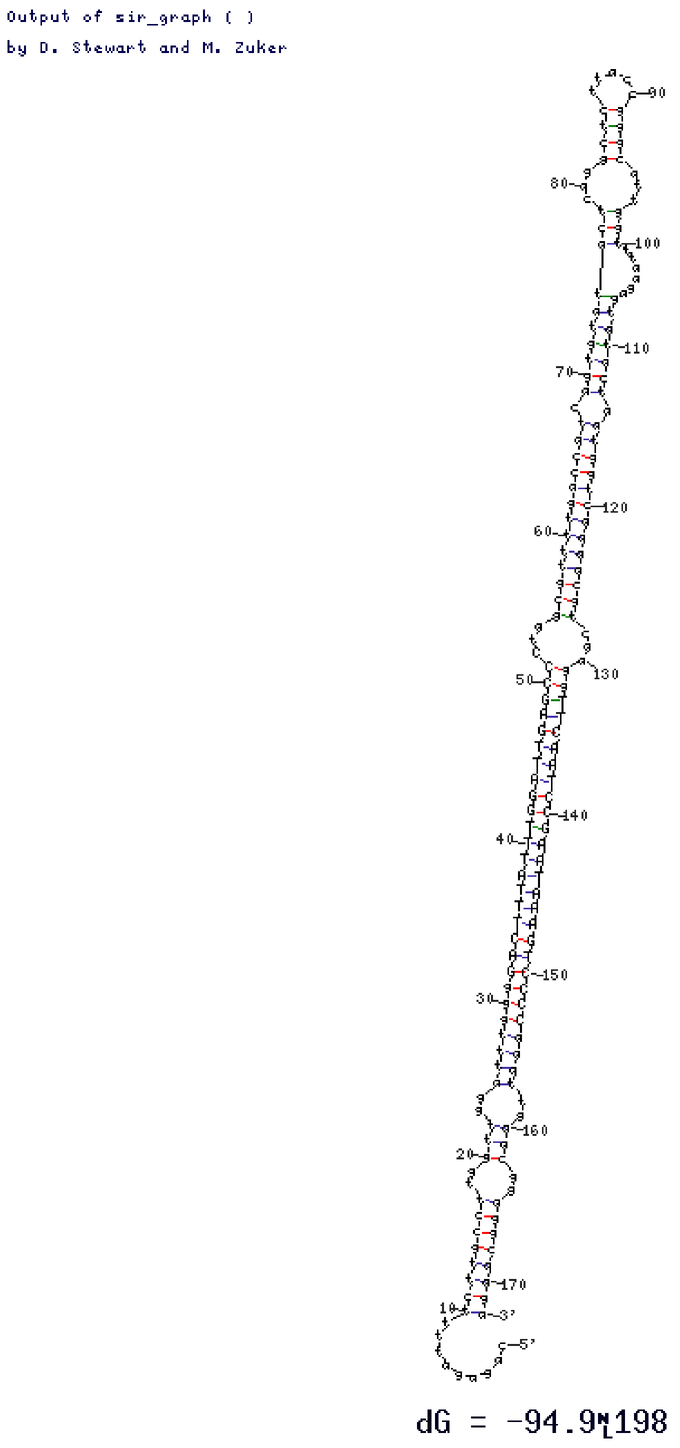

csn-smR5-3p-2
UUCAAUCCGAAUAAAGUCCCC

Output of sir_graph ( )

by D. Stewart and M. Zuker

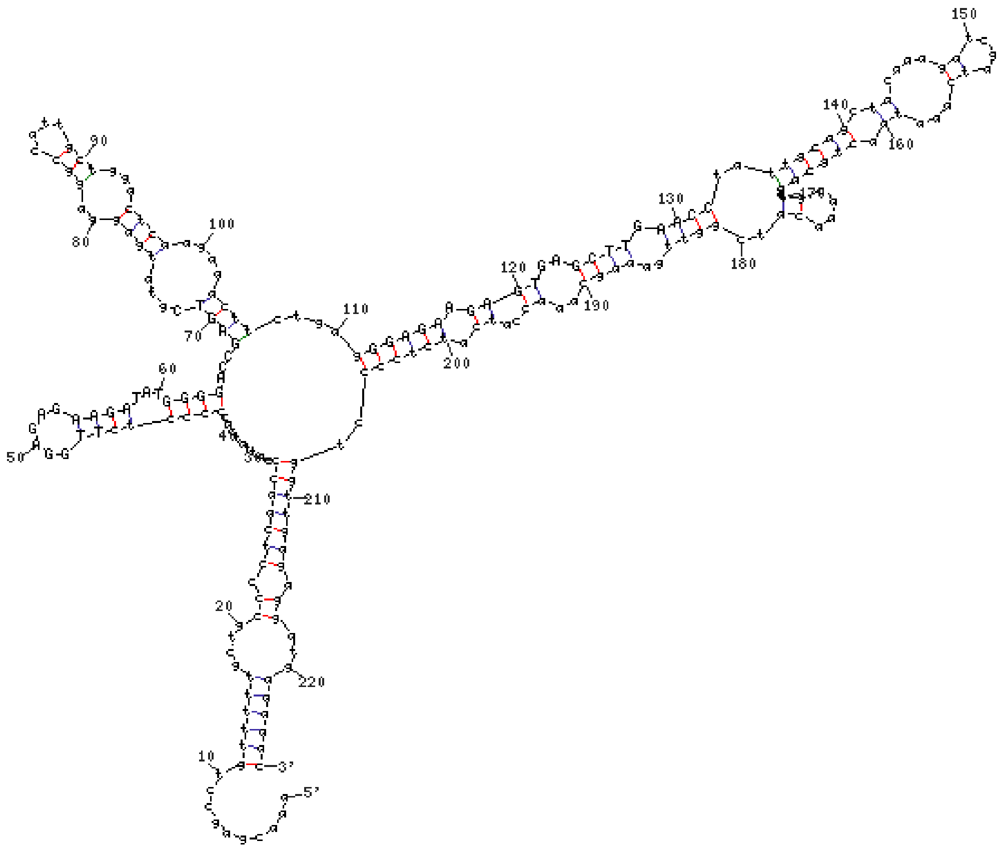

dG = -55.6[illegible]202

csn-smR6

GGAGAAGAGUGAGCUUGAACC

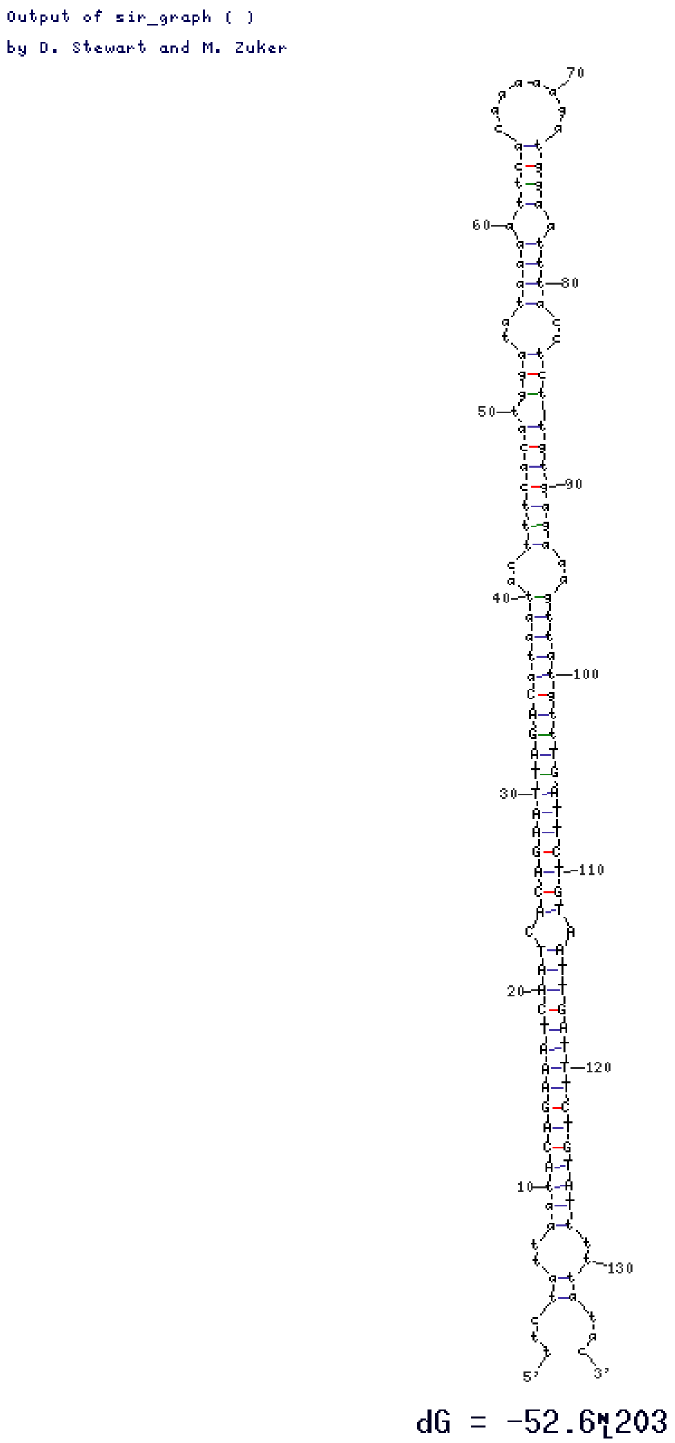

csn-smR7-5p
ACAGAAAUCAAUCACAGAAUUAGAC

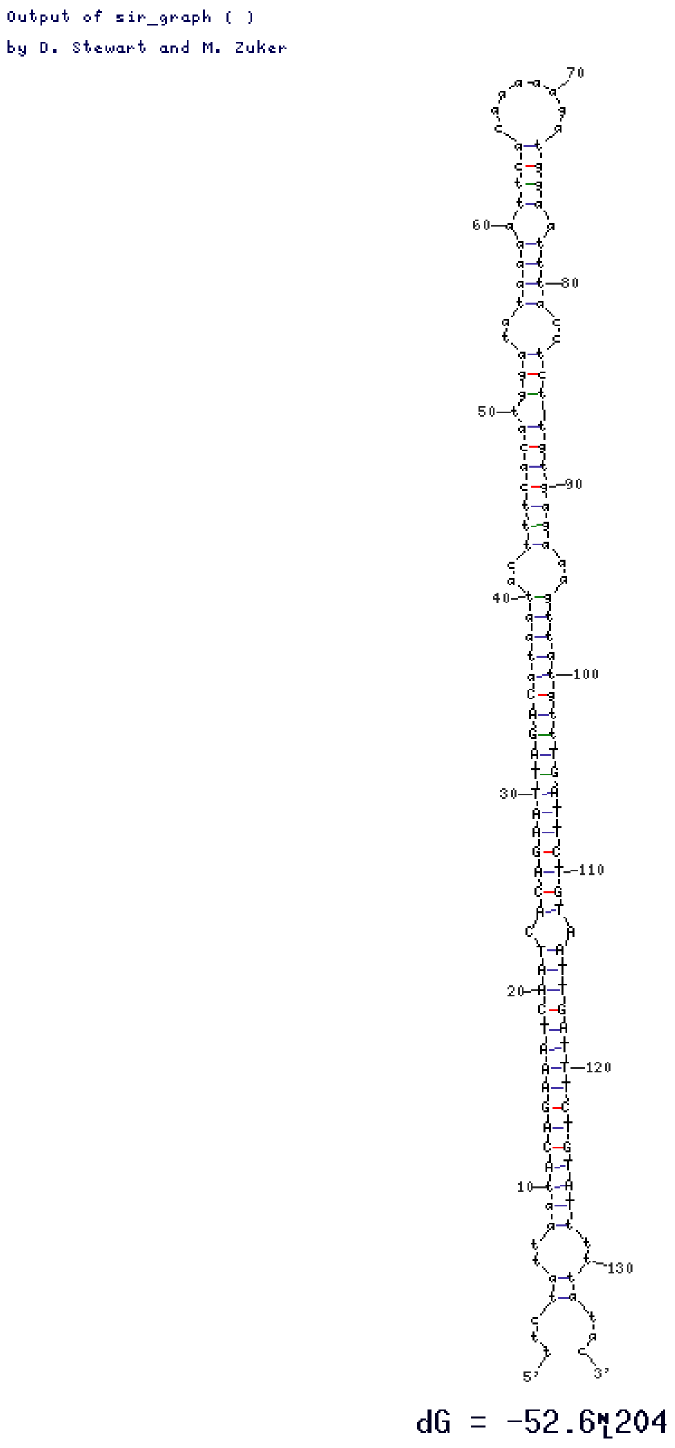

csn-smR7-3p
UGAUUCUGUAAUUGAUUUCUGUAU

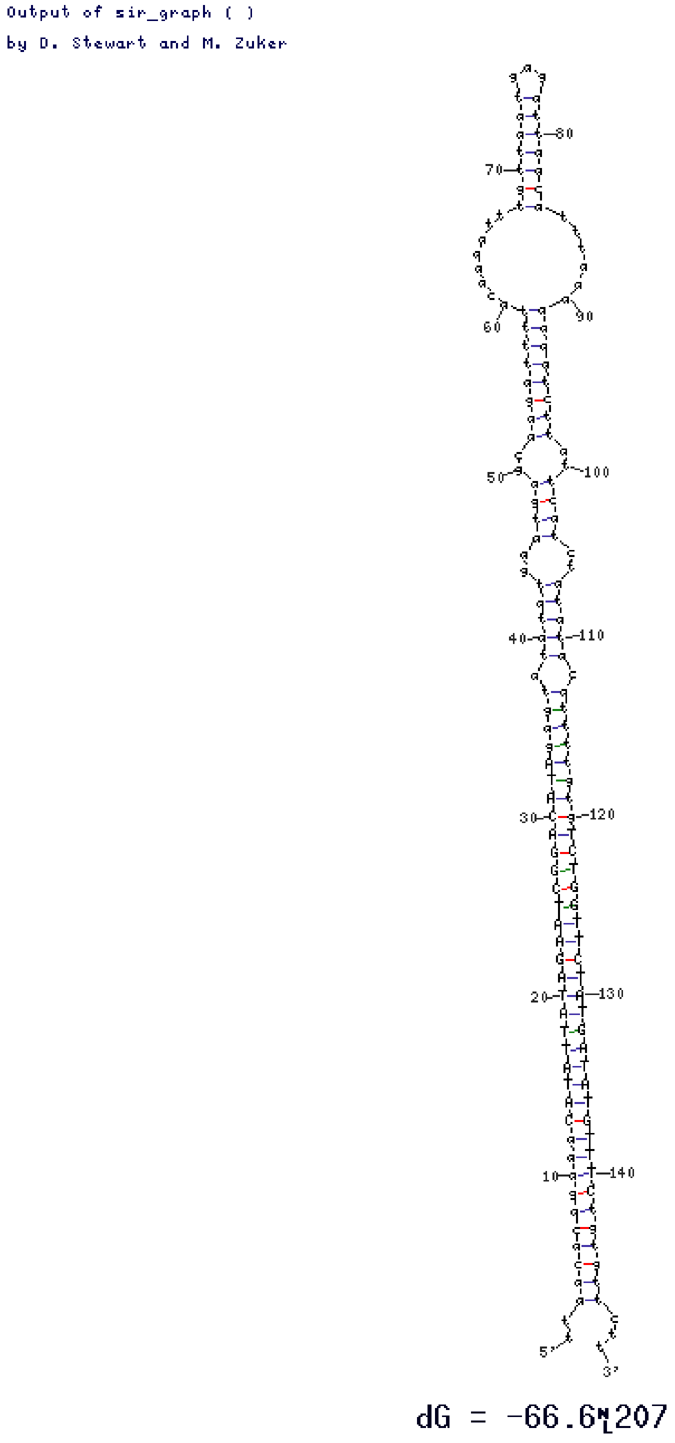

csn-smR8
CAUAUUAUAGAAUCGGACAUA

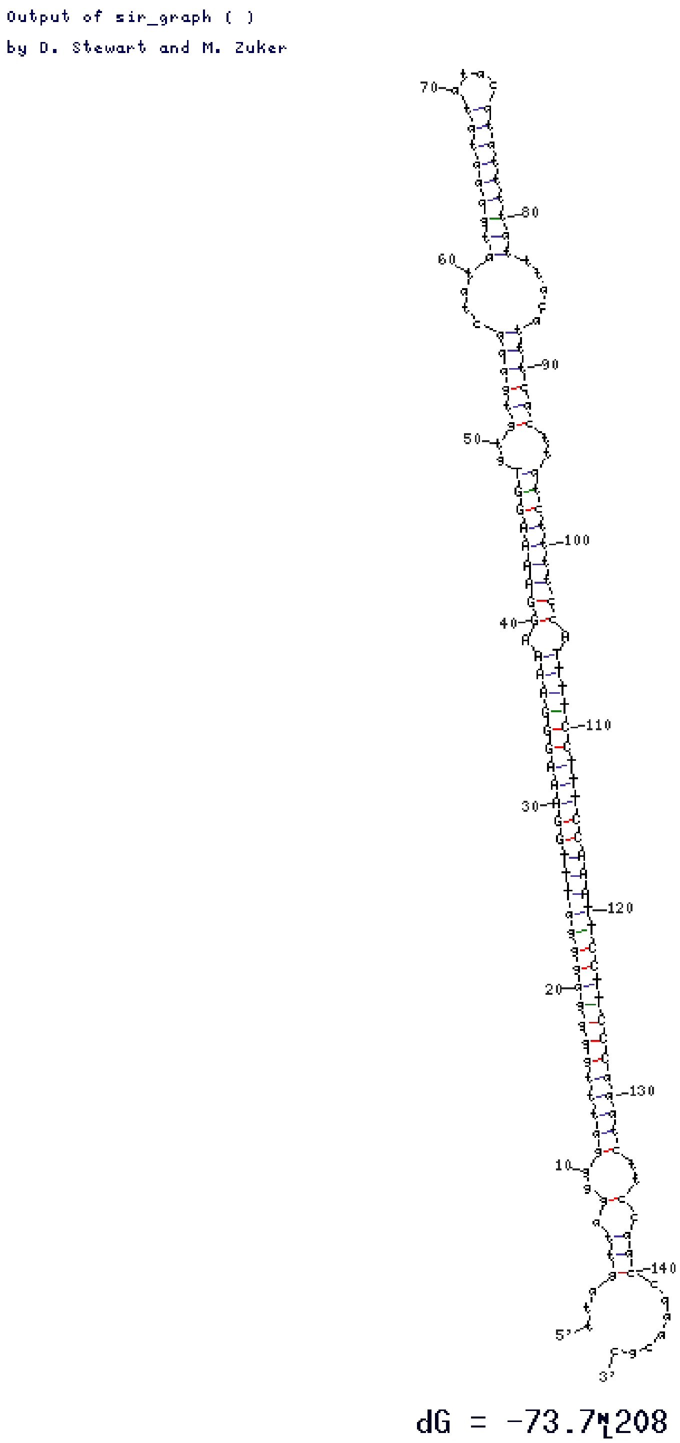

csn-smR9-5p-1

UUUGGAAAGGGAAAUGGAAAAGGU

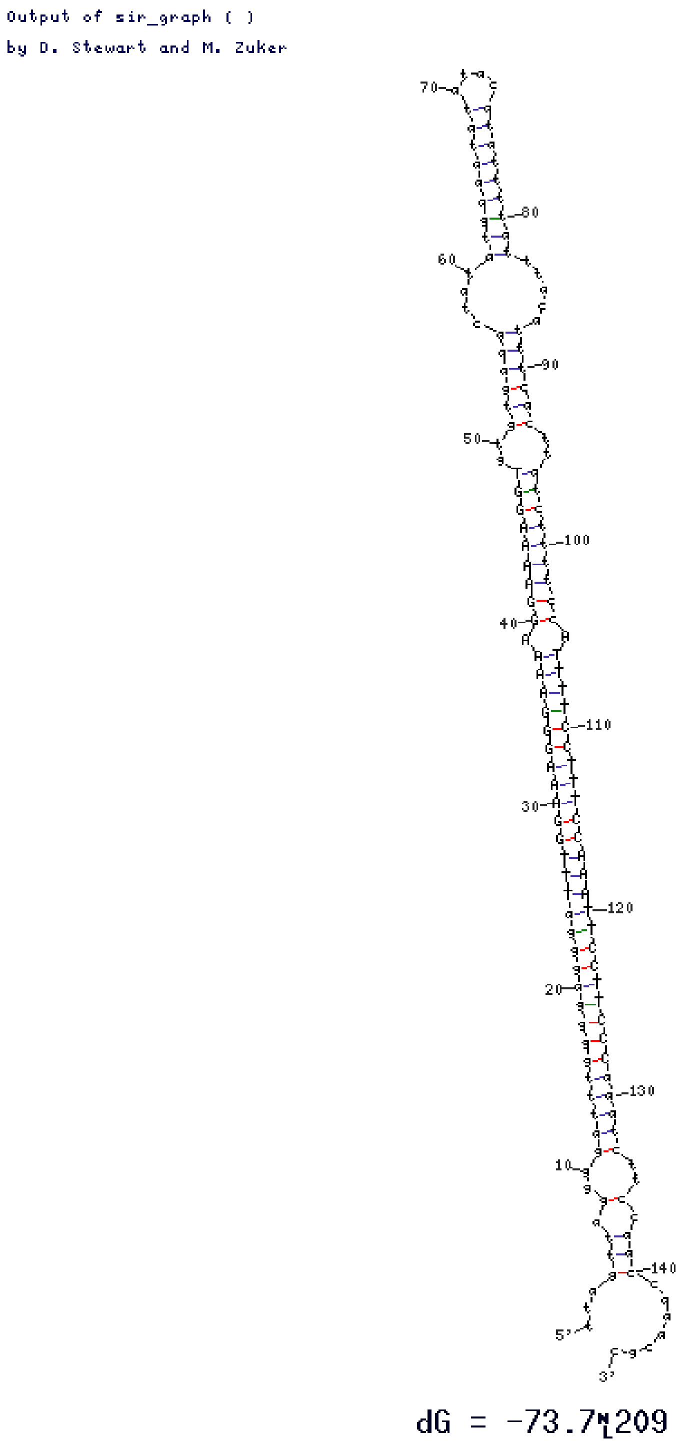

csn-smR9-3p-1
AUUUCCCUUUCCAAAUUCCUUCCC

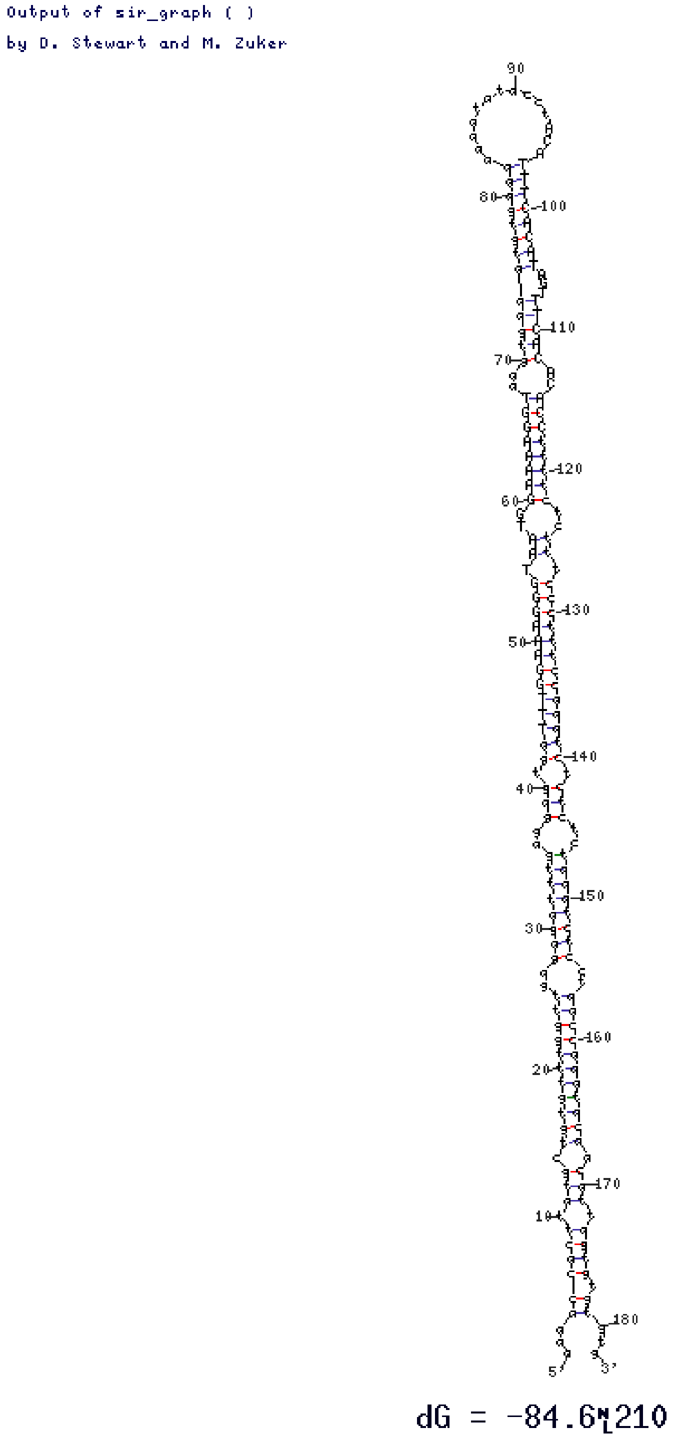

csn-smR9-5p-2
UUUGGAAAGGGAAAUGGAAAAGGU

Output of sir_graph ( )
by D. Stewart and M. Zuker

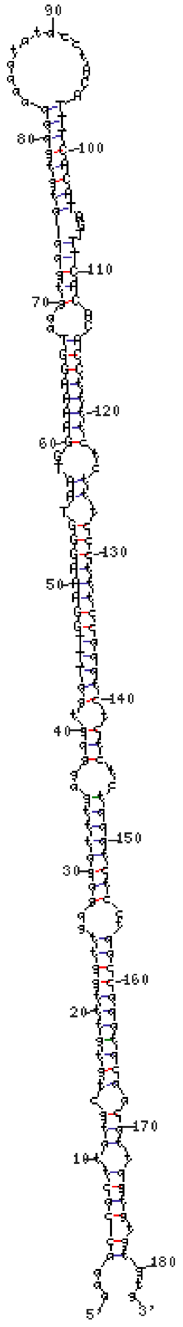

dG = −84.6[illegible]211

csn-smR9-3p-2
AAAUUUCACAUAGUUUCACACACC

Output of sir_graph ( )
by D. Stewart and M. Zuker

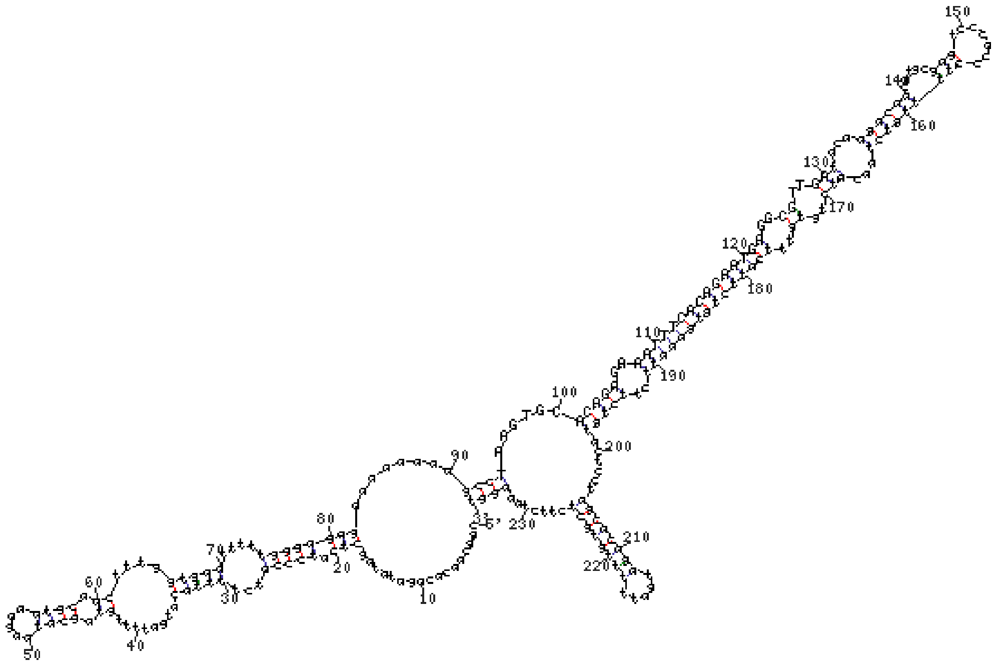

dG = -68.9[illegible]213

csn-smR10-1
UUUCACAGAAUGAGGCGUUGA

Output of sir_graph ( )
by D. Stewart and M. Zuker

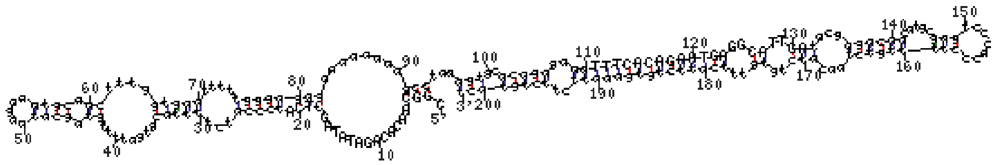

dG = -58.8[illegible]215

csn-smR10-2
UUUCACAGAAUGAGGCGUUGA

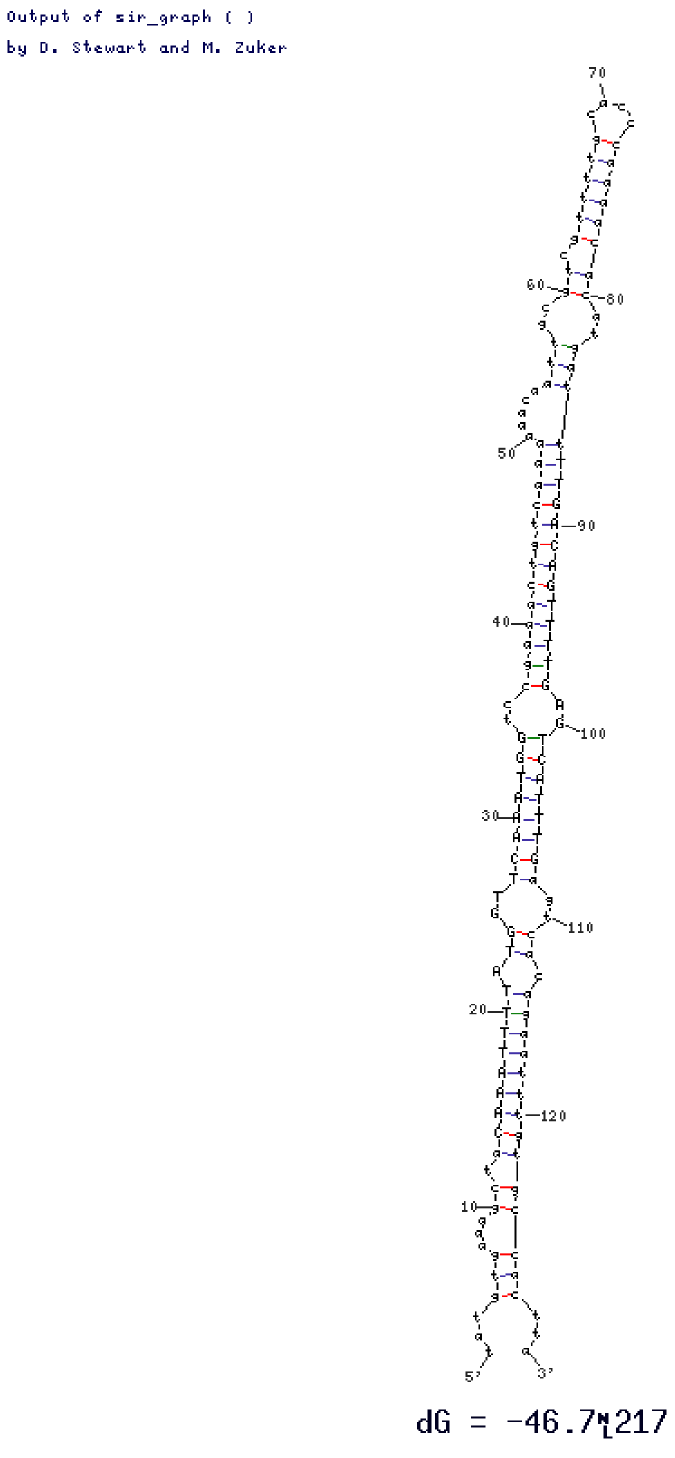

csn-smR11-1
UUGACAGUUUUGAGCCAUUUG

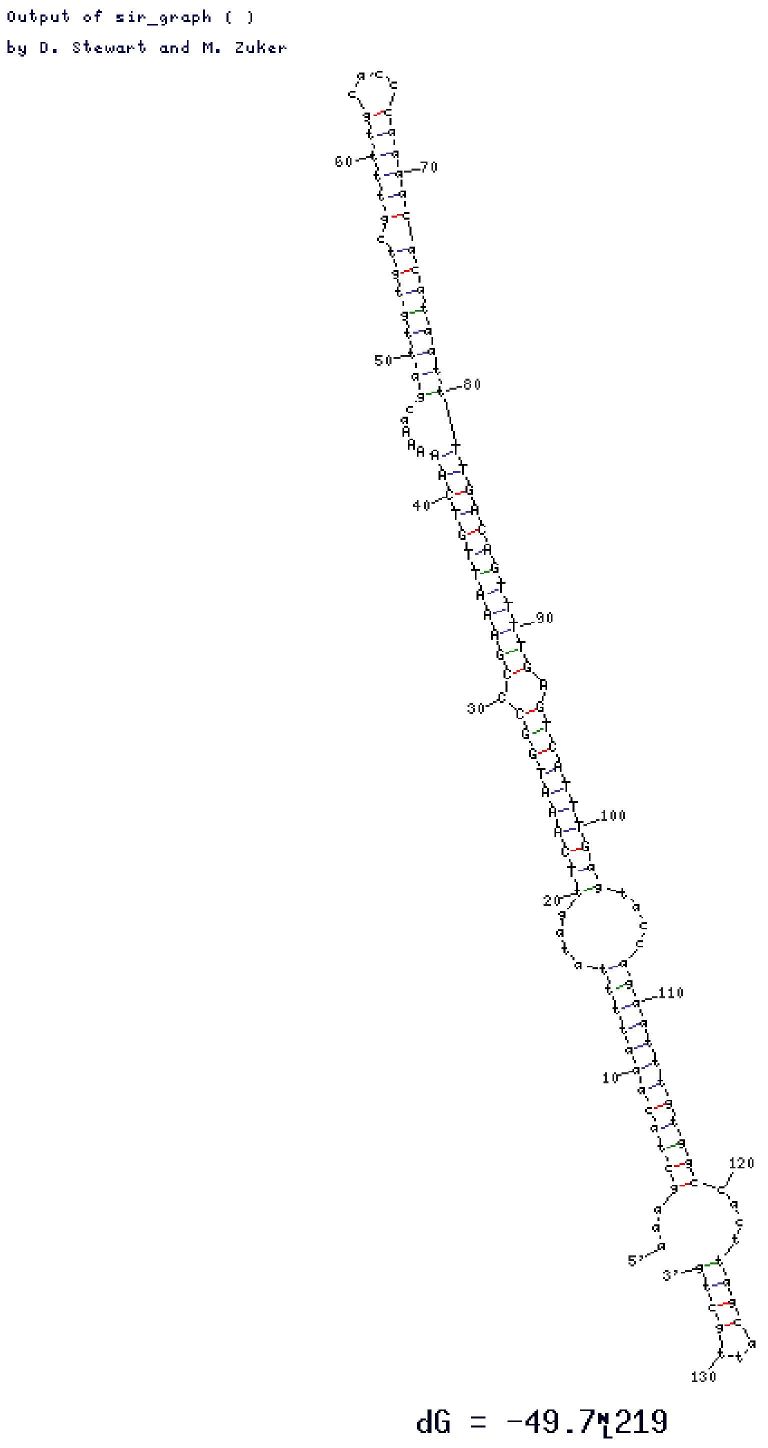

csn-smR11-2
UUGACAGUUUUGAGCCAUUUG

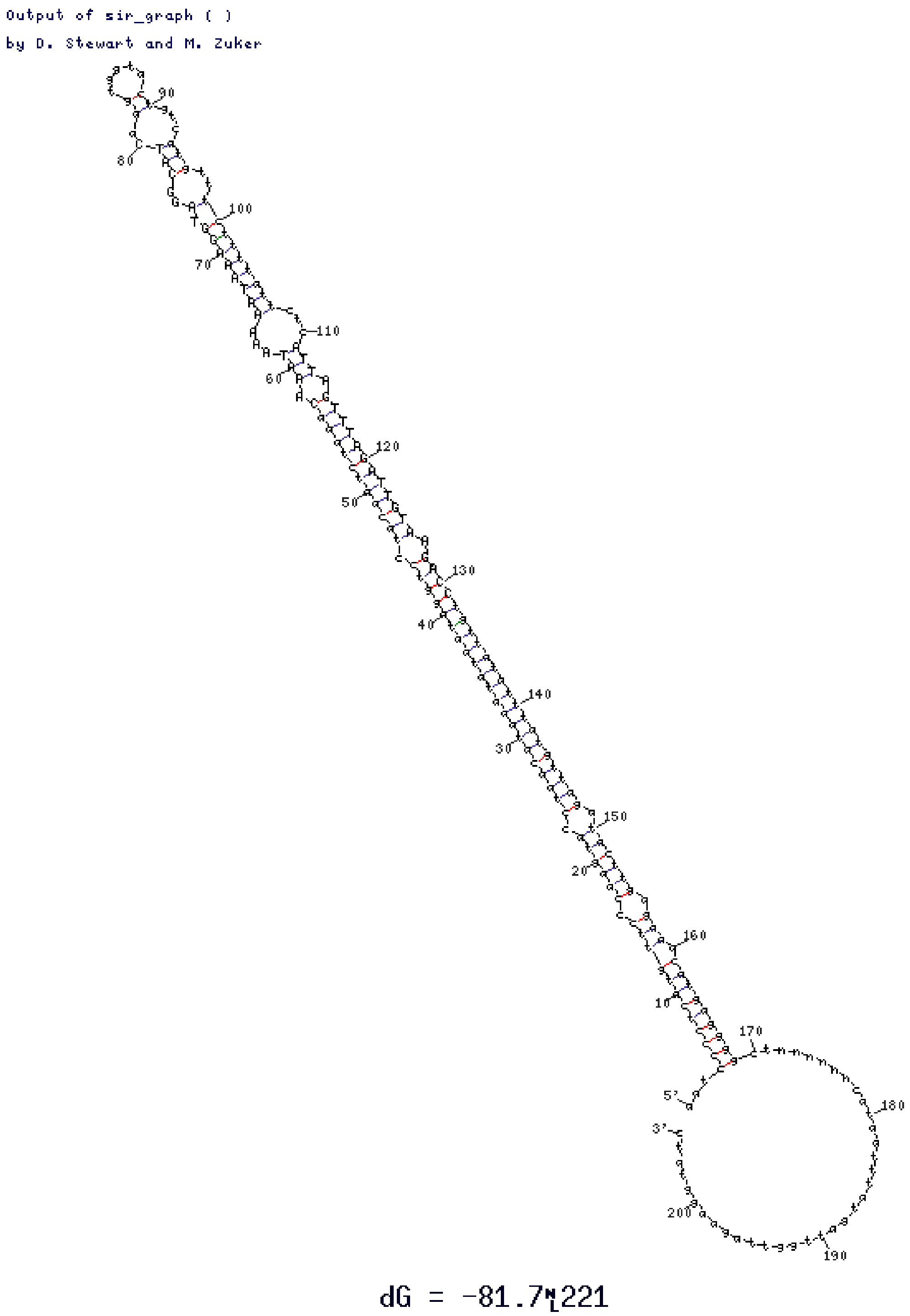

csn-smR12-1

AUUAGUUUAGAUUGUAGGACC

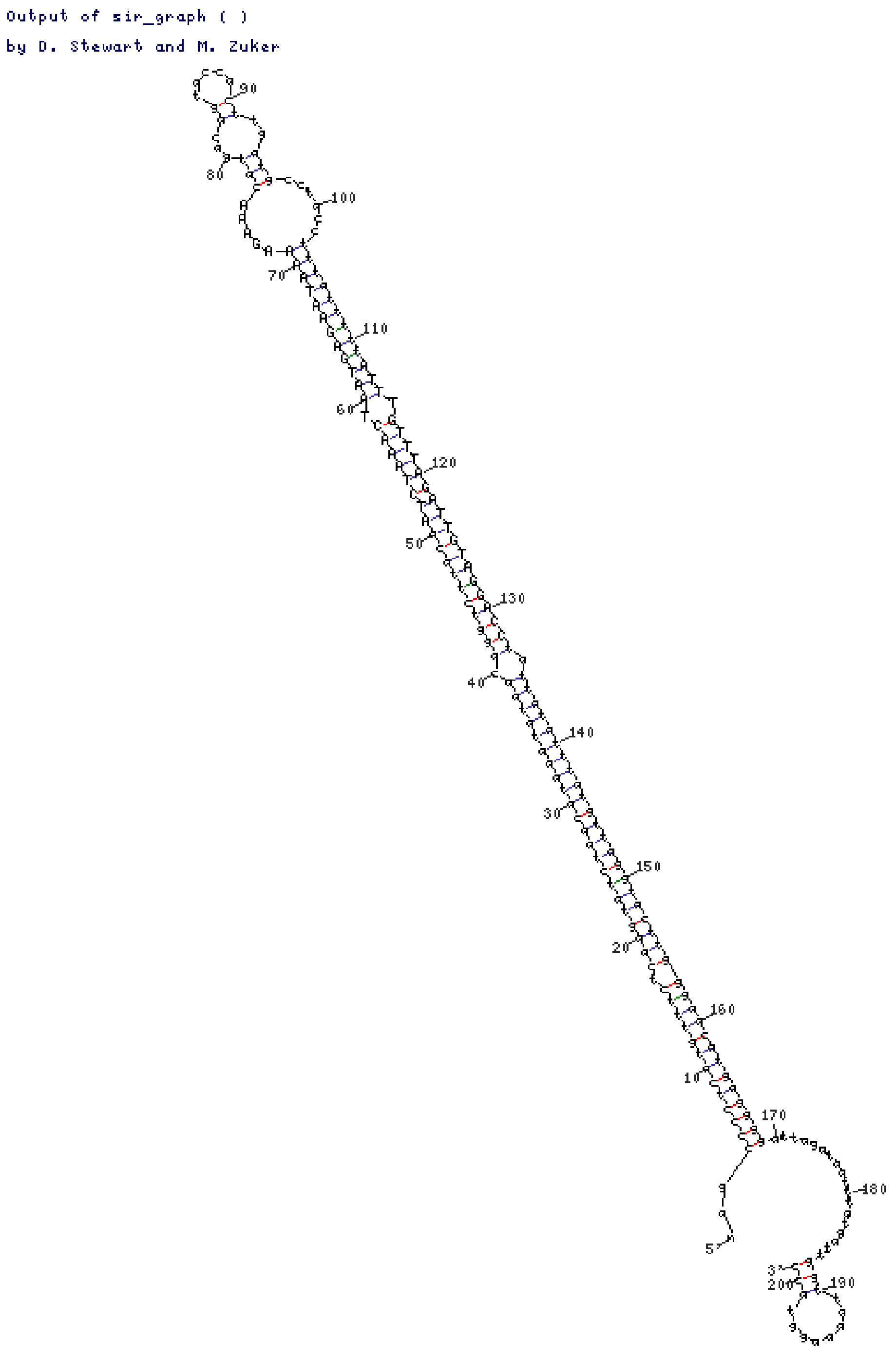

dG = -89.8 [illegible]223

csn-smR12-2
AUUAGUUUAGAUUGUAGGACC

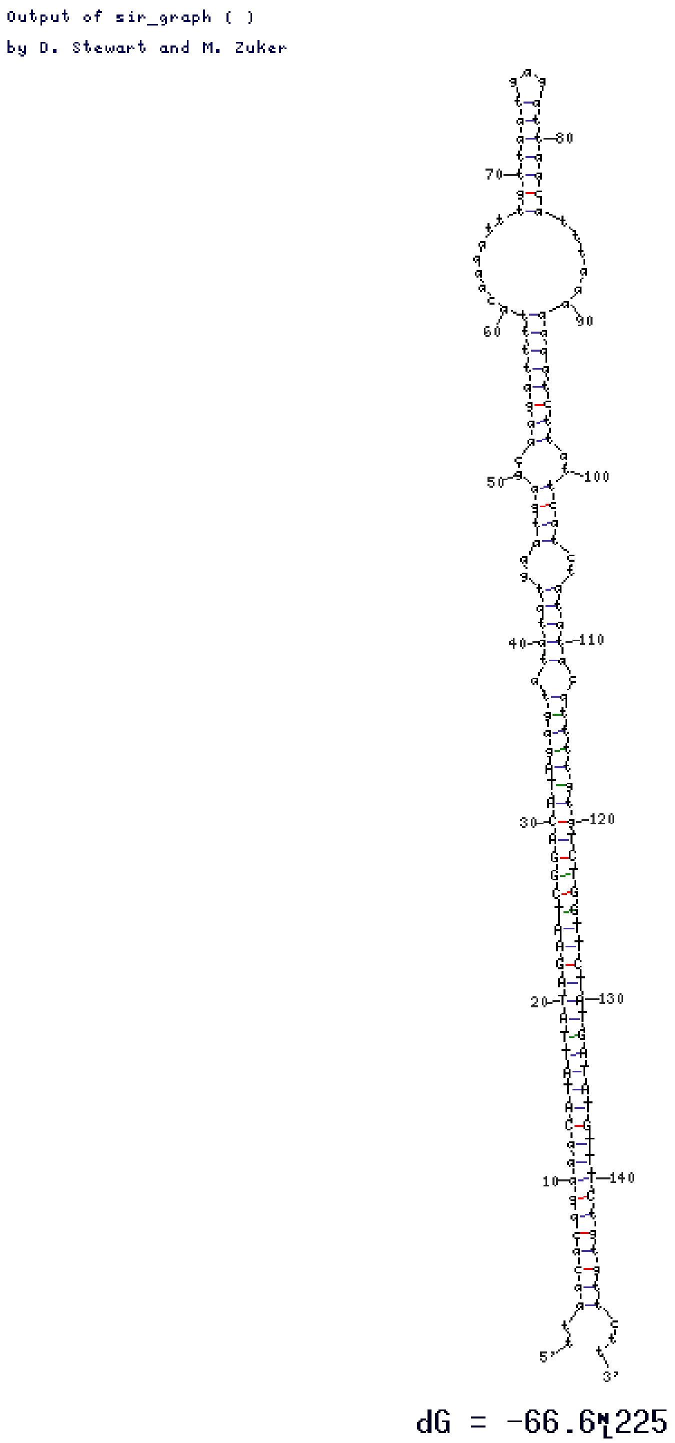

csn-smR13-1
UCUGGUUCUAUGAUAUGUUUC

Output of sir_graph ( )
by D. Stewart and M. Zuker

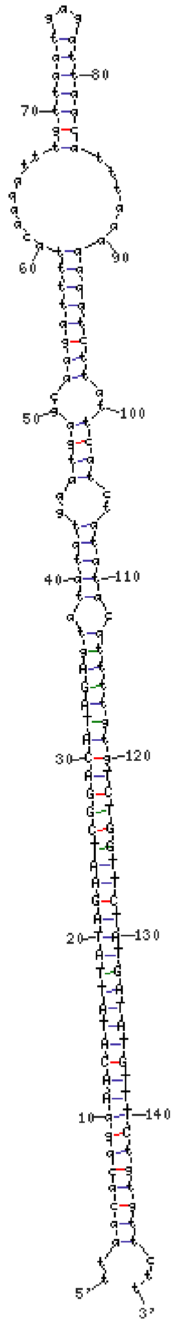

dG = -66.6 227

csn-smR13-2
UCUGGUUCUAUGAUAUGUUUC

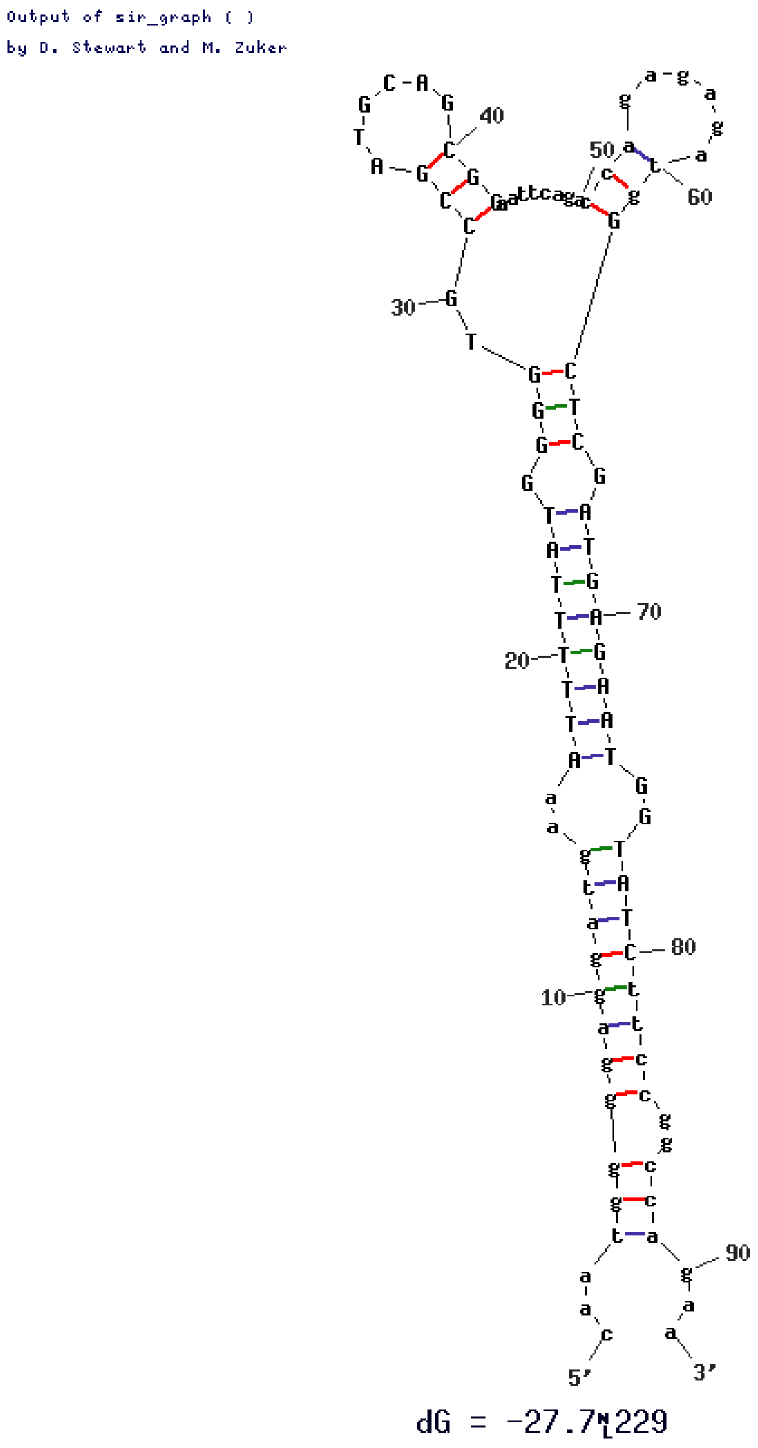

csn-smR14
UCUCGAUGAGAAUGGUAUC

Output of sir_graph ( )
by D. Stewart and M. Zuker

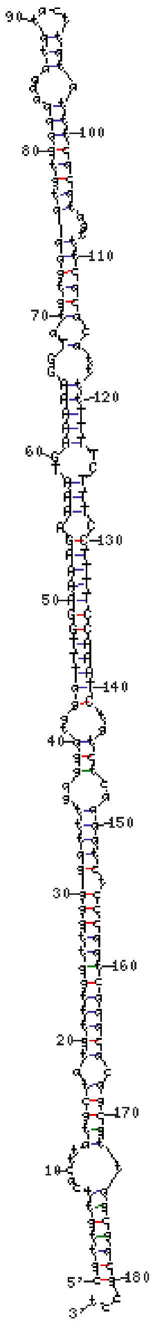

dG = -75.7 [illegible] 230

csn-smR15-5p
UUUGGAAAAGAAAAUGAAAAAGGU

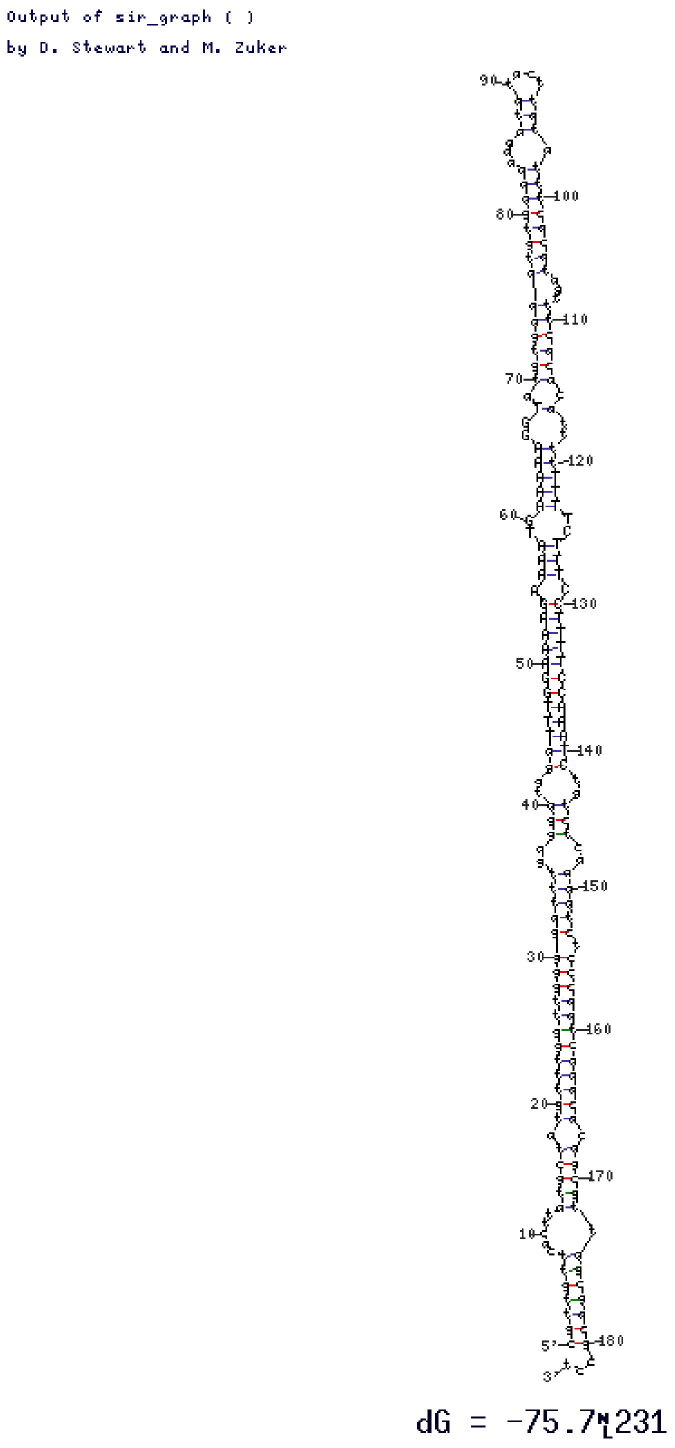

csn-smR15-3p
UUUUCUUUCCUUUUCCAAAUC

Output of sir_graph ( )
by D. Stewart and M. Zuker

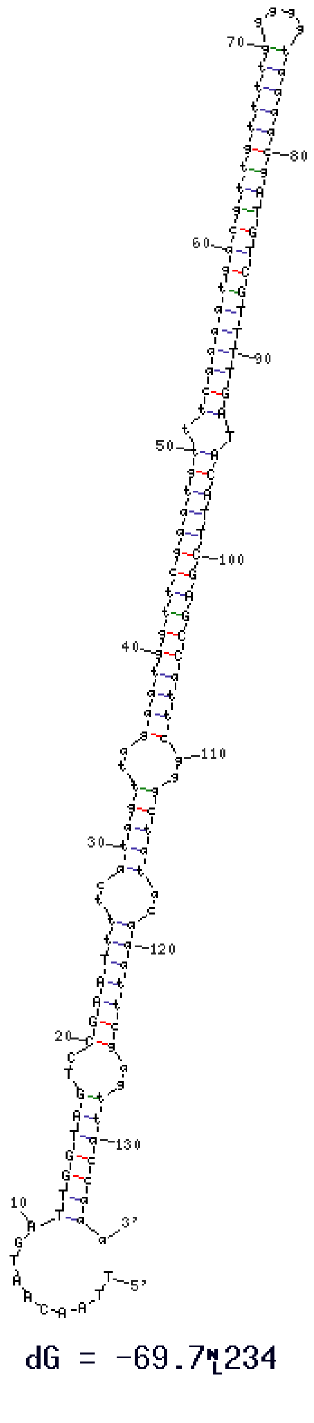

dG = -69.7 ␤234

csn-smR17-5p
UUAACAAUGAUUGGUAGUCCGAAU

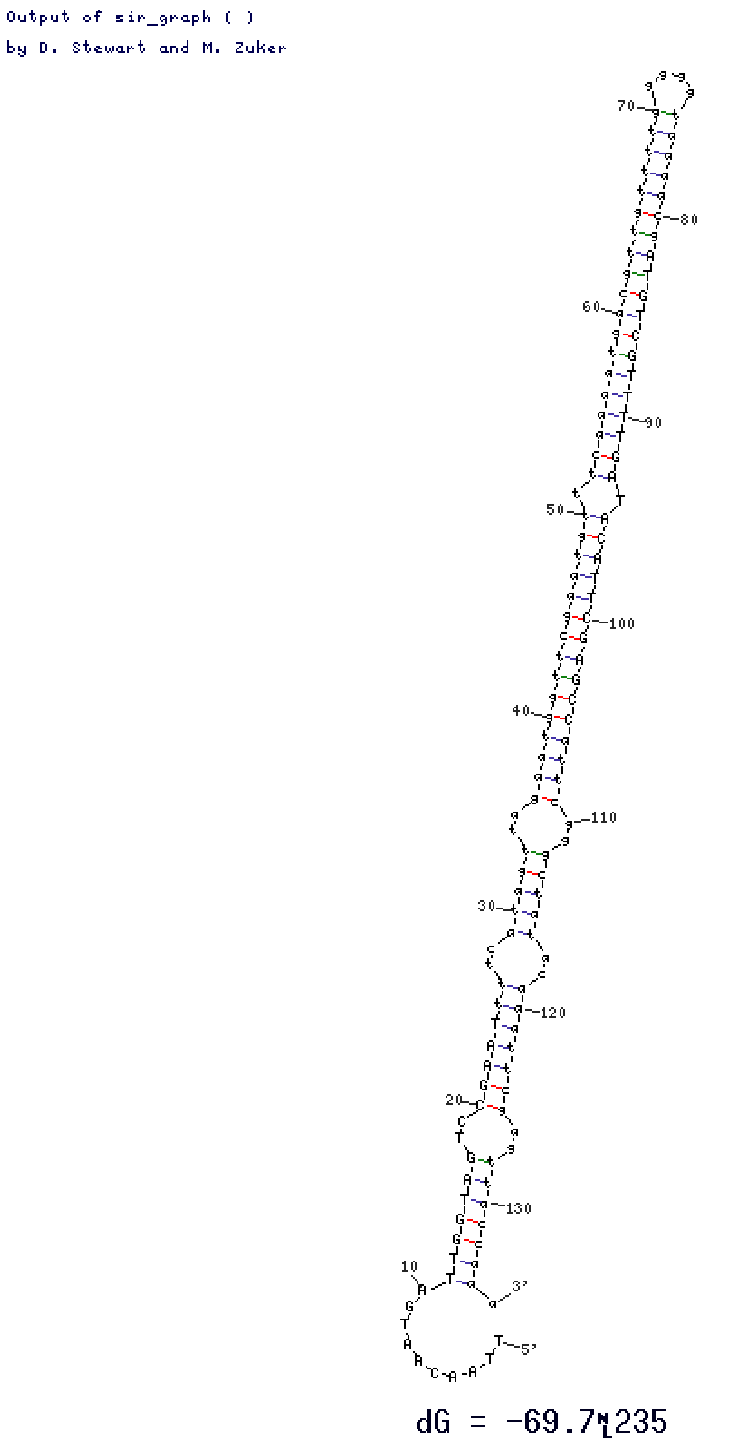

csn-smR17-3p
AUGUCGUUUUGAUACAUUGGAGCC

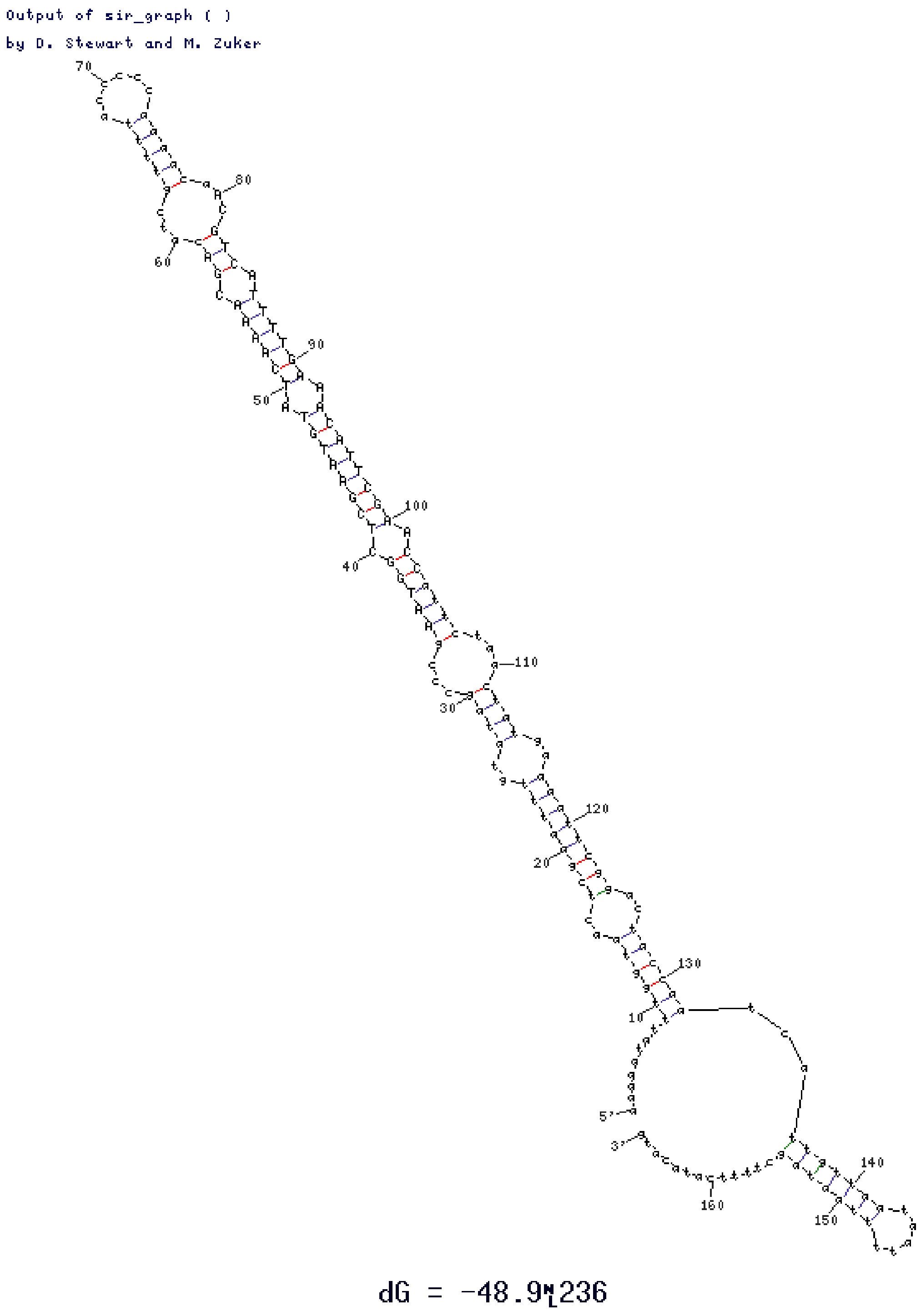

csn-smR18-5p
AAUGGCUCAAAUGUAUCAAAACGA

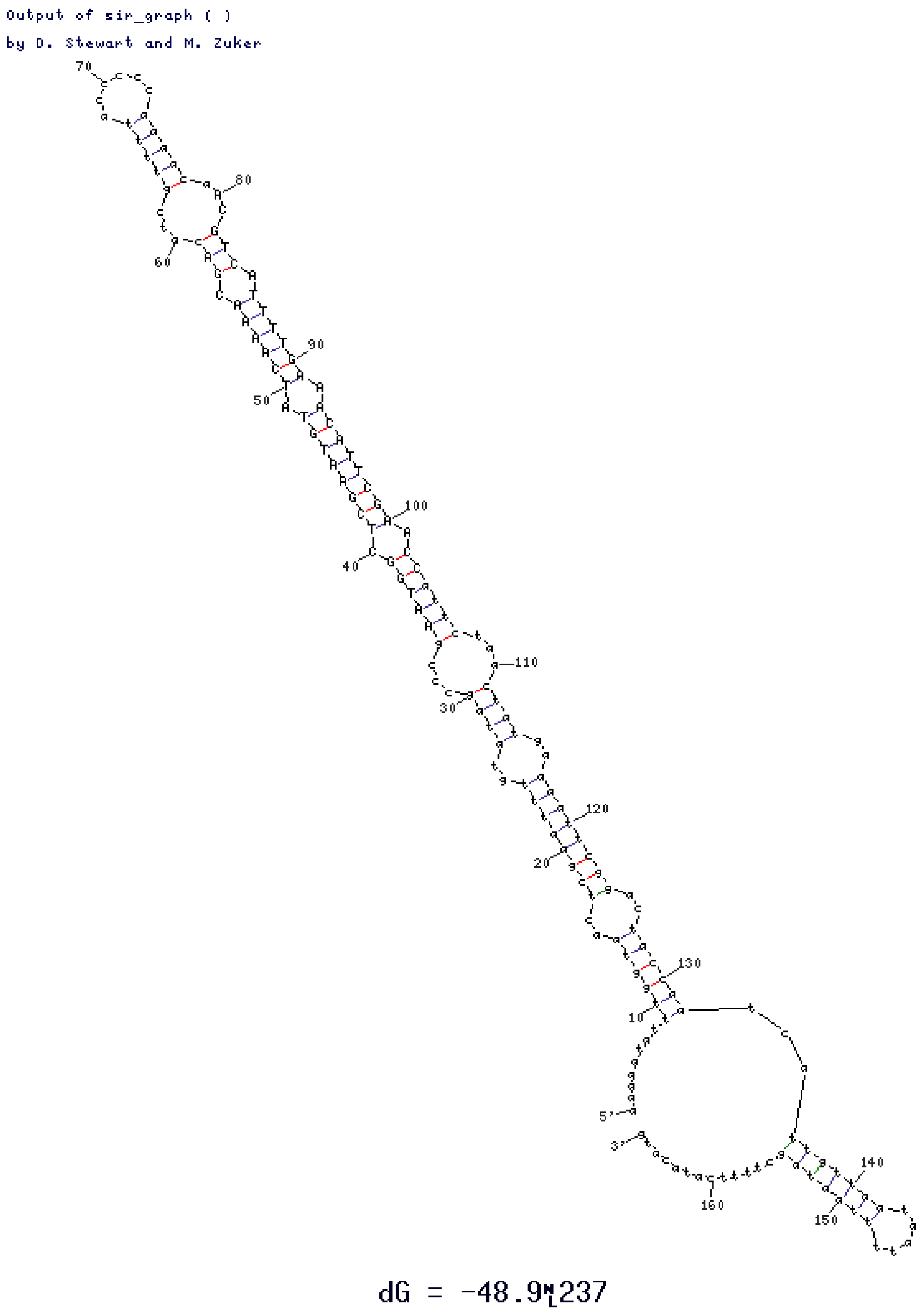

csn-smR18-3p
ACGUCAUUUUGAGACAUUCGAACC

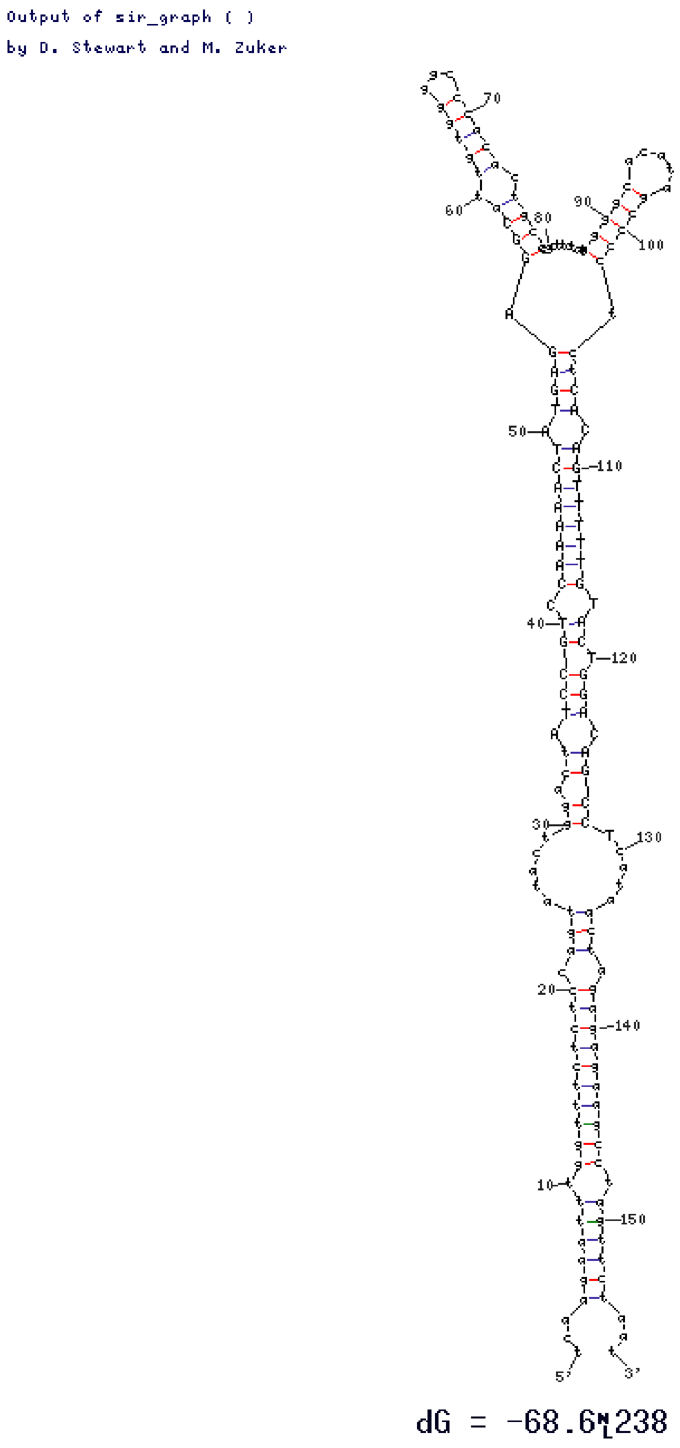

csn-smR19-5p
AUCCGUCCAAAAGCUAUGAGAGGC

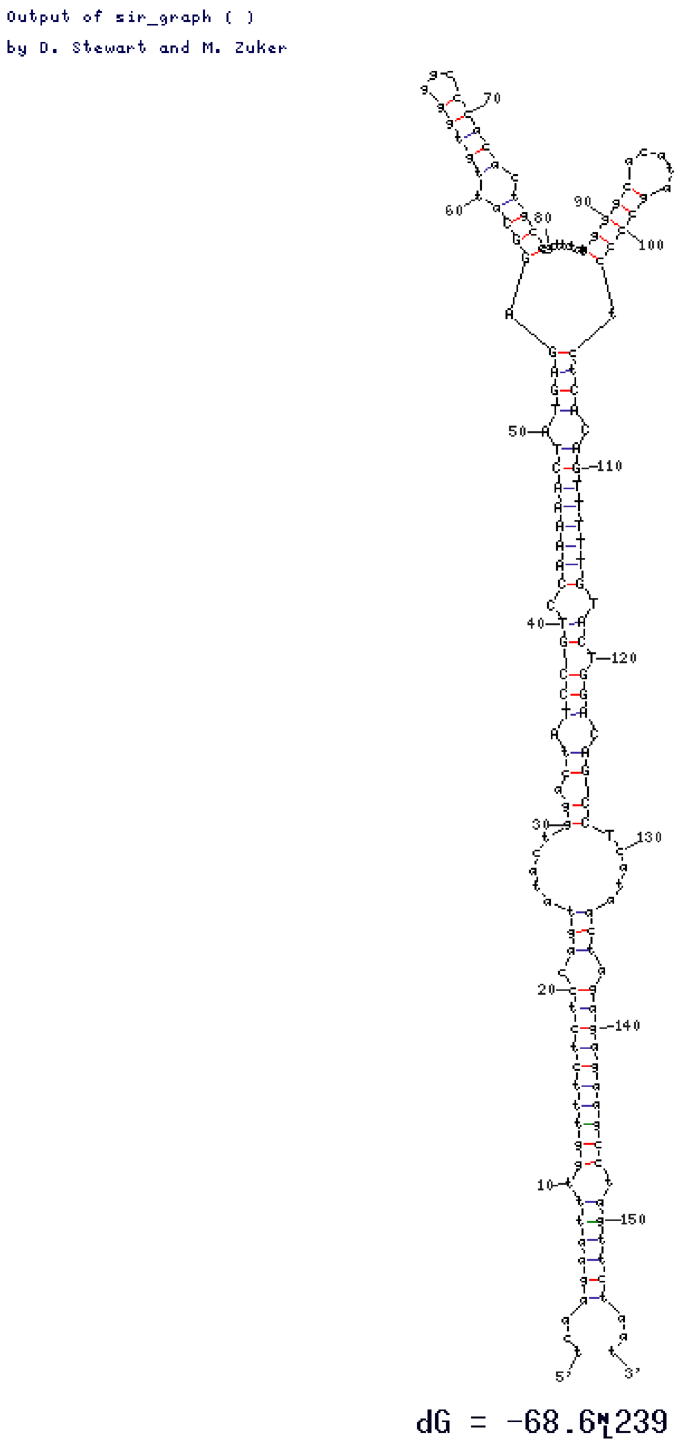

csn-smR19-3p
CACAGUUUUUGUACUGGACAGCCU

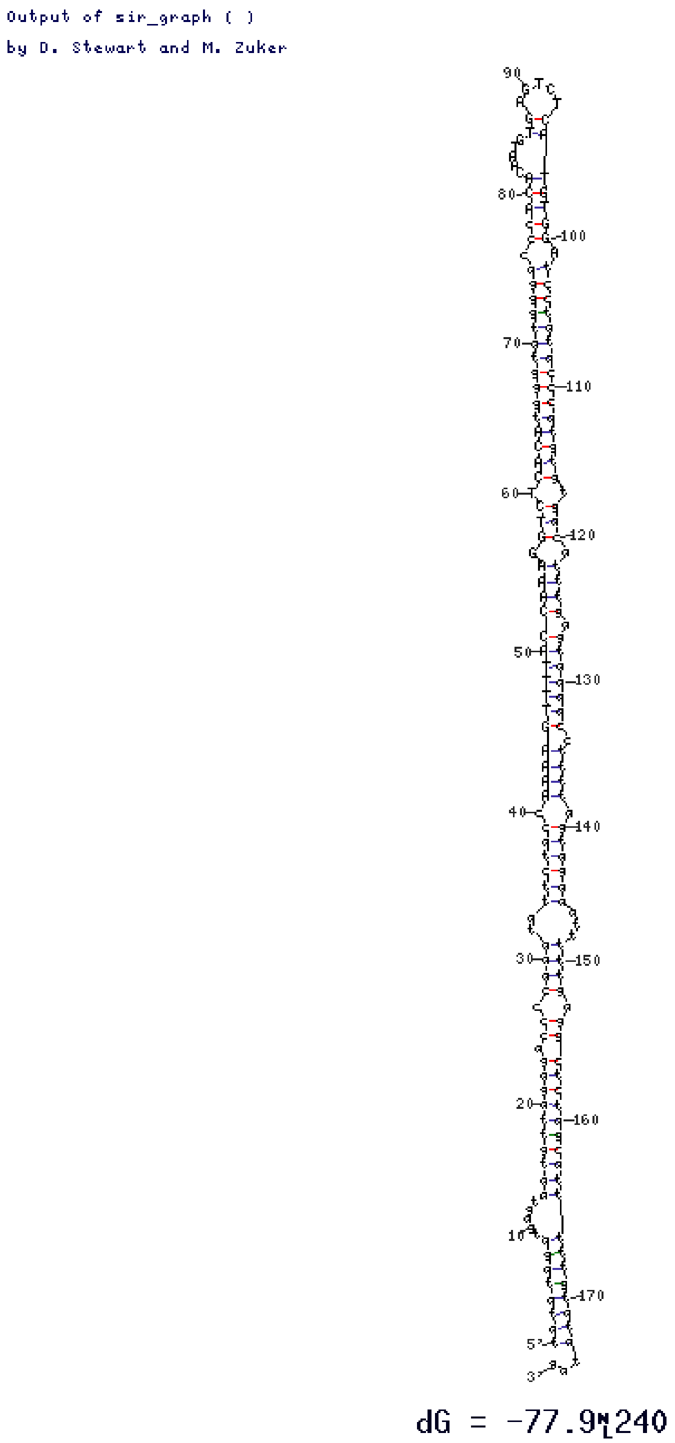

csn-smR20-5p
AAAAGUUUUACCAAAUGUCUCACA

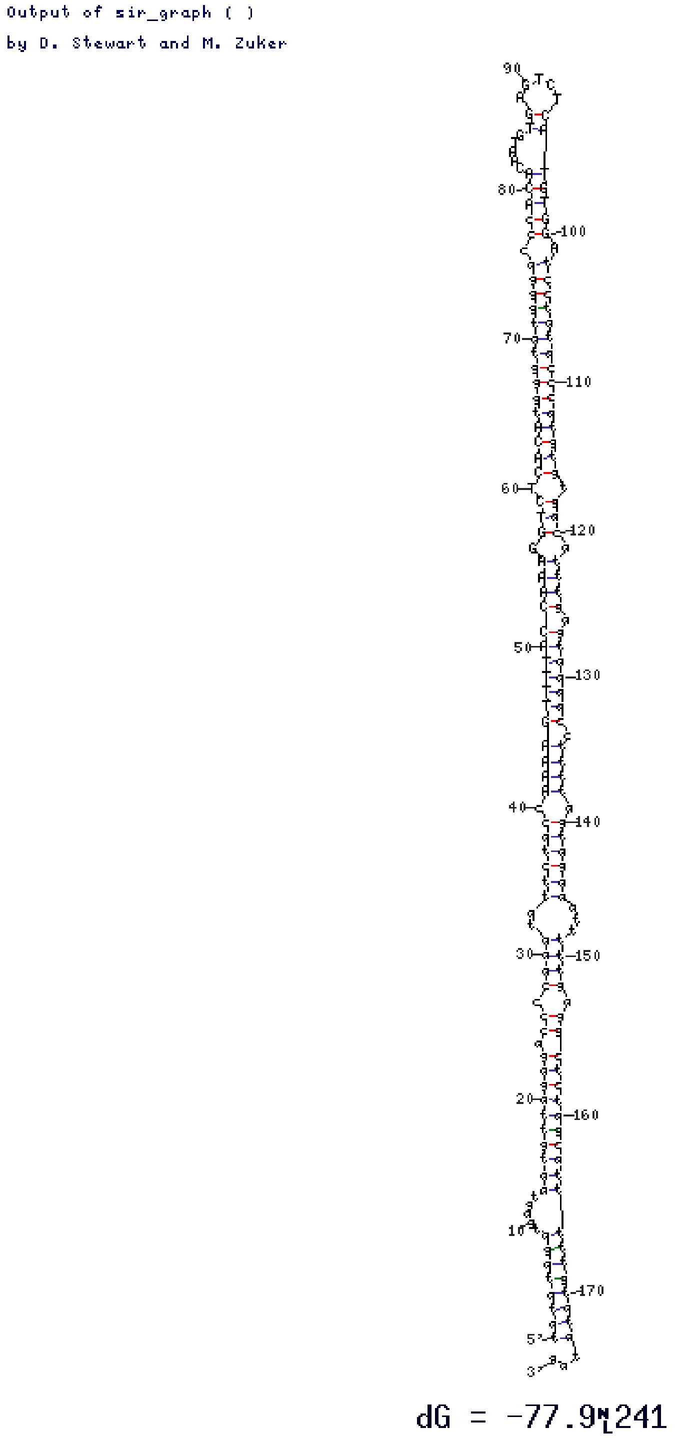

csn-smR20-3p
ACACAAUGUGGGUCUCAUGUGGA

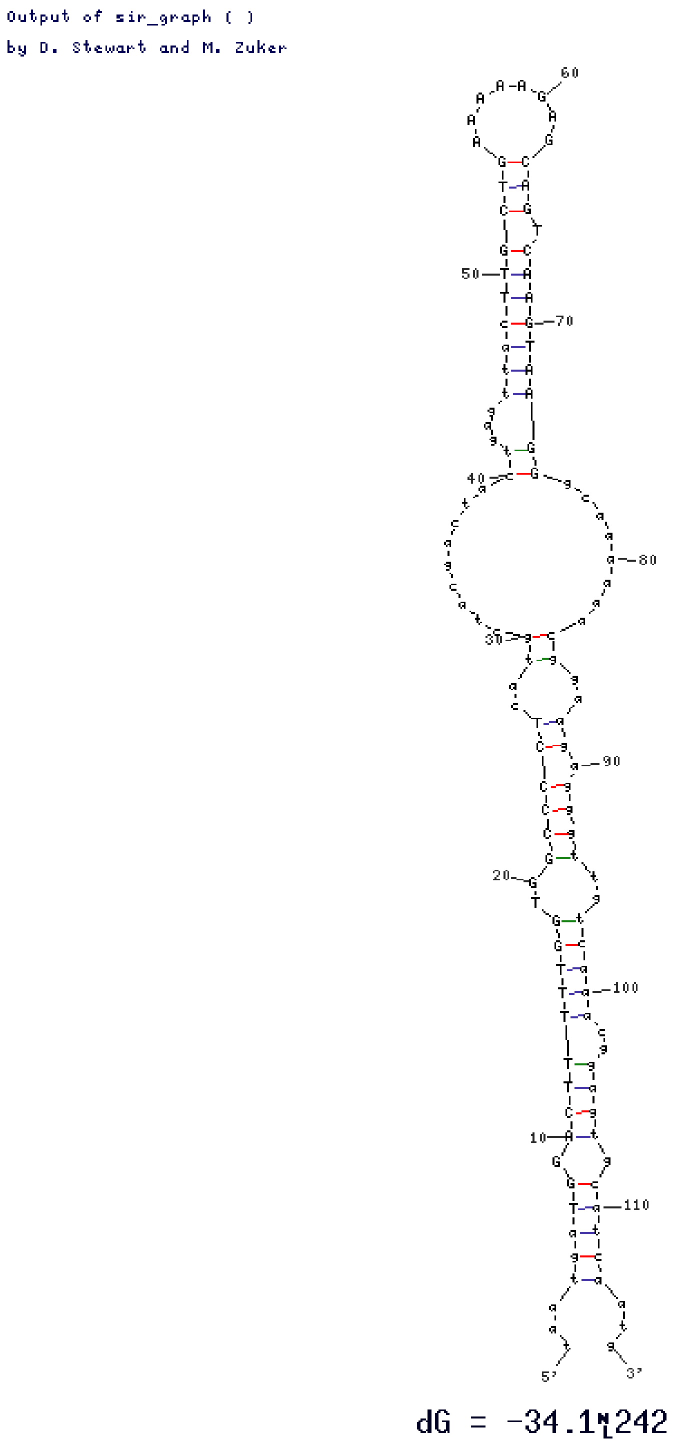

csn-smR21-5p
UGGACUUUUUGGUGGCCCCU

Output of sir_graph ( )
by D. Stewart and M. Zuker

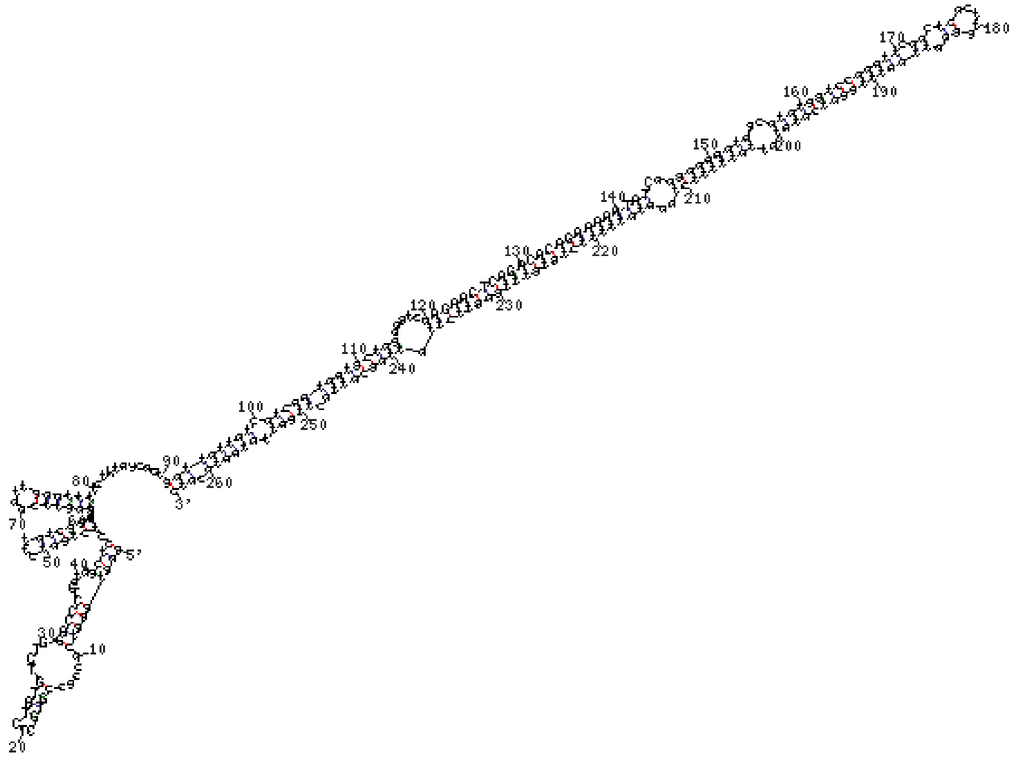

dG = -91.2 [illegible]245

csn-smR22
AGAACUCAGACACAGAAAAAUAUC

Output of sir_graph ( )
by D. Stewart and M. Zuker

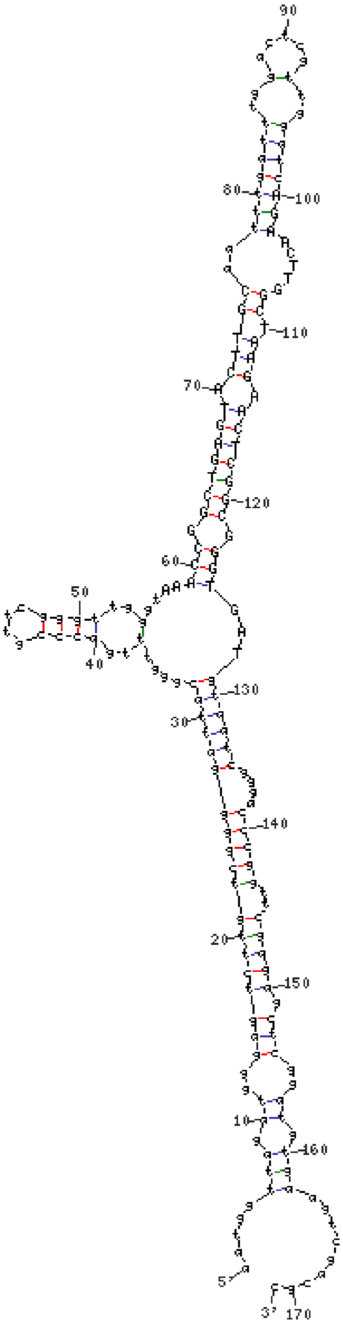

dG = -57.9[illegible]246

csn-smR23-5p
AAACCGGCCGAGUACUUGC

Output of sir_graph ( )
by D. Stewart and M. Zuker

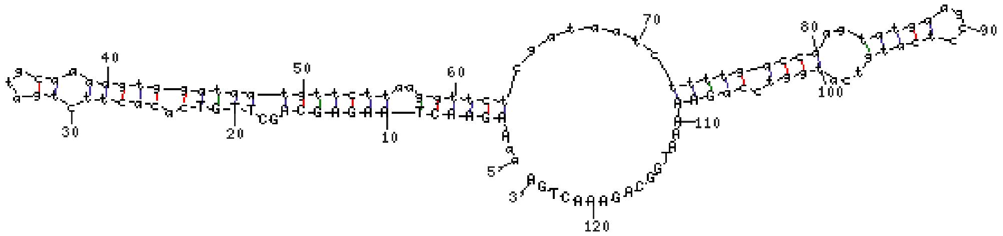

dG = -29.5

csn-smR25
AAGAACUAAGAGCAGCUUGU

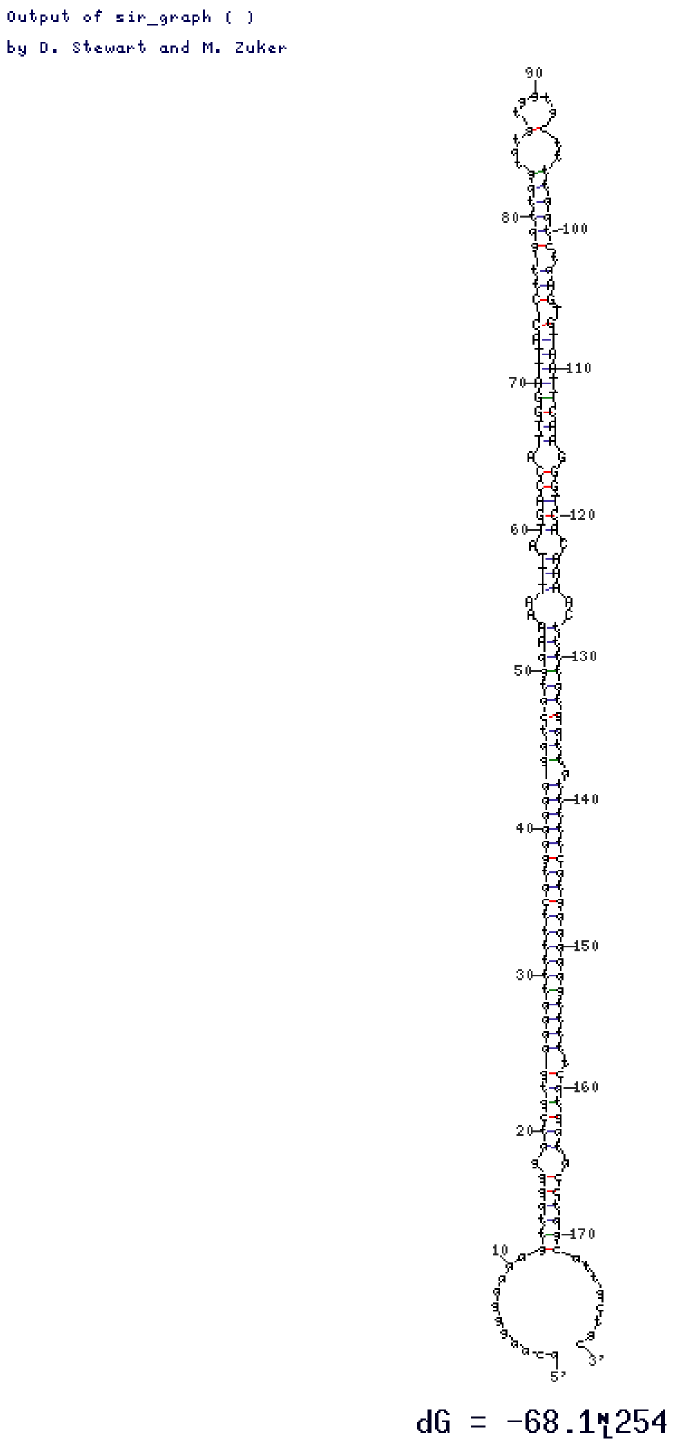

csn-smR27
AAAAUUUAUGACCCUUGGAUUACC

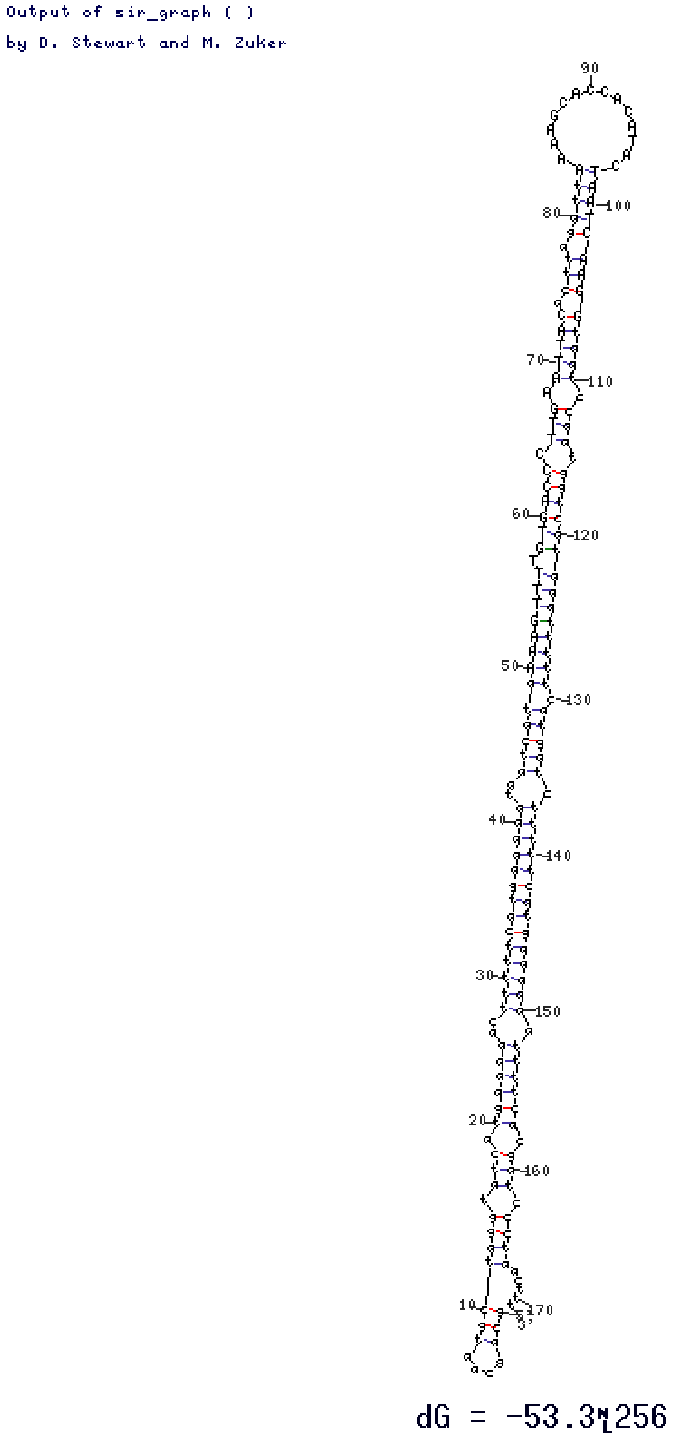

csn-smR28-5p
AAAGUUUUGUGACCCUUGGAUUAC

Output of sir_graph ( )
by D. Stewart and M. Zuker

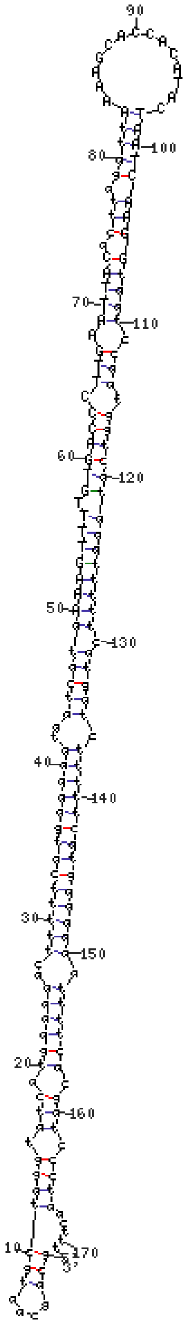

dG = -53.3 ?257

csn-smR28-3p
AAAAGCACCACACACUAAUCAAGG

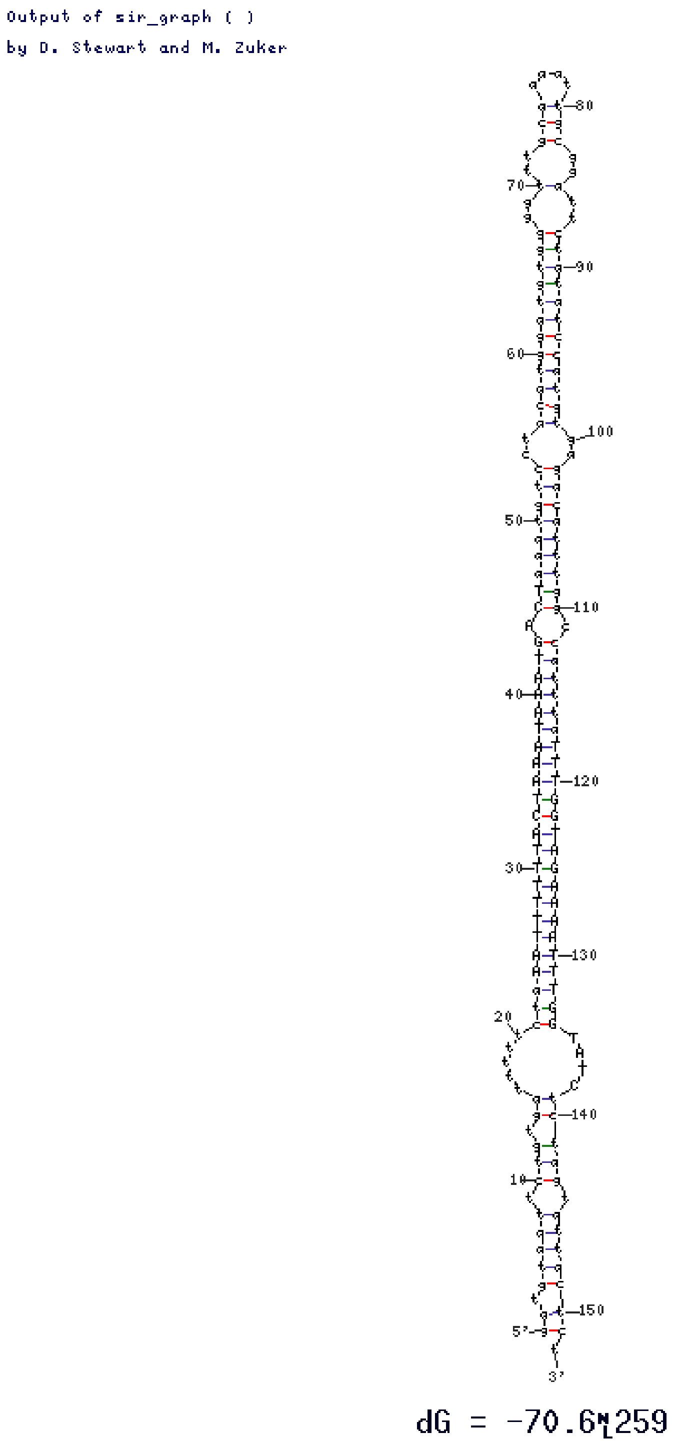

csn-smR29
UUUGGUAGAAAAUUUGGUAUC

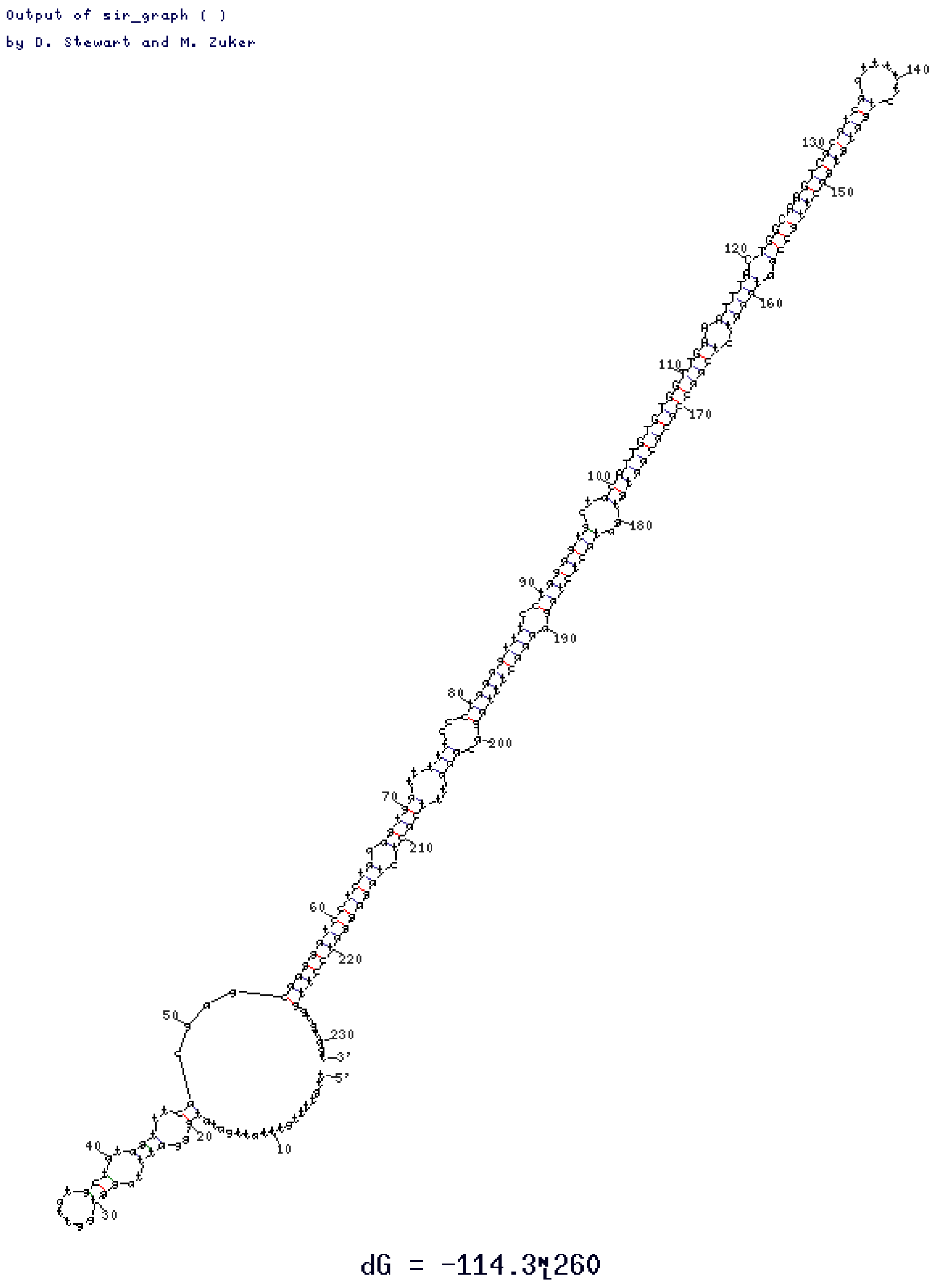

csn-smR30
AUUGUGUGGUUGAGAUUUACUGGC

Output of sir_graph ( )
by D. Stewart and M. Zuker

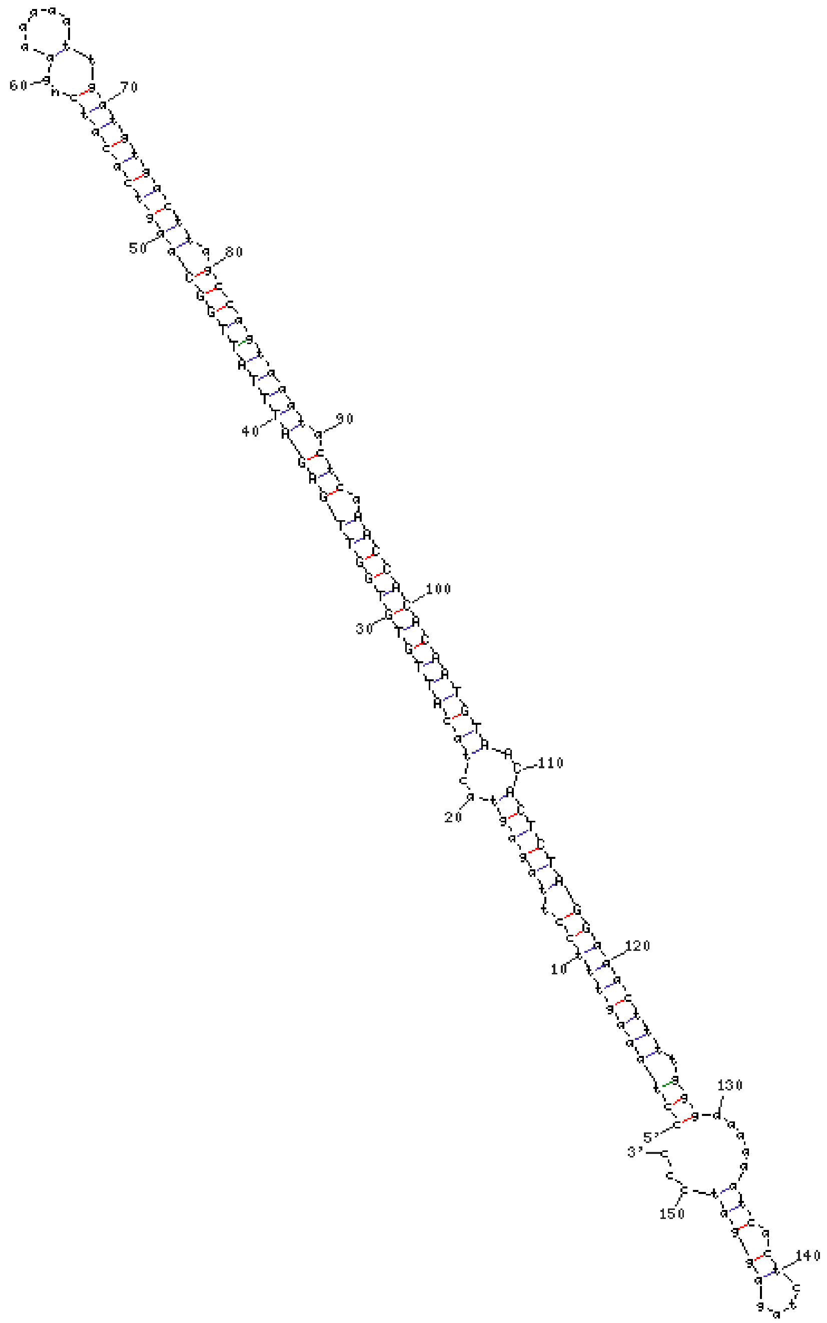

dG = -75.6 L262

csn-smR31-5p
AUUGUGUGGUUGAGAUUUAUUGGC

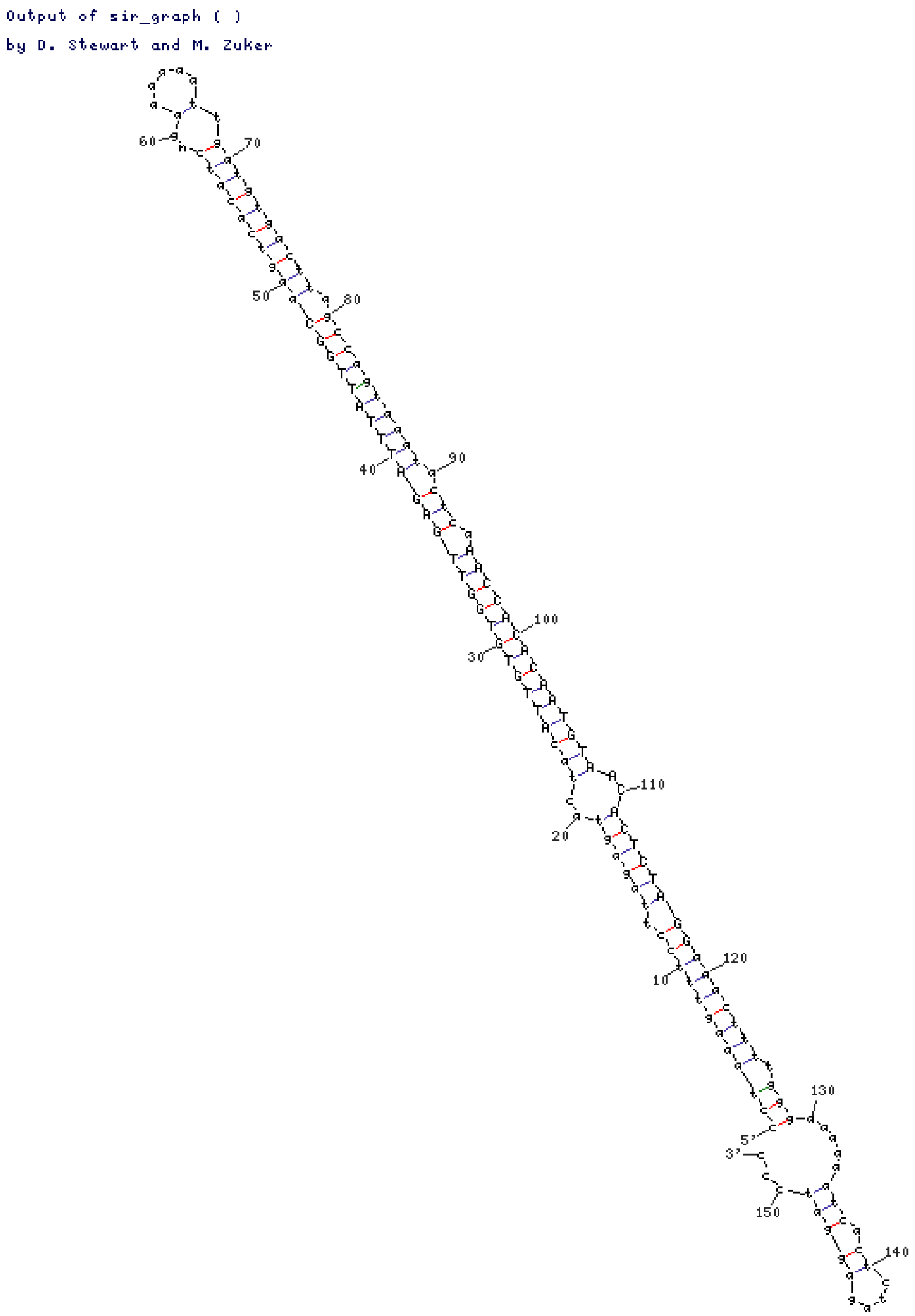

dG = −75.6[illegible]263

csn-smR31-3p
AACCACACAAUGUAACACUCUAGG

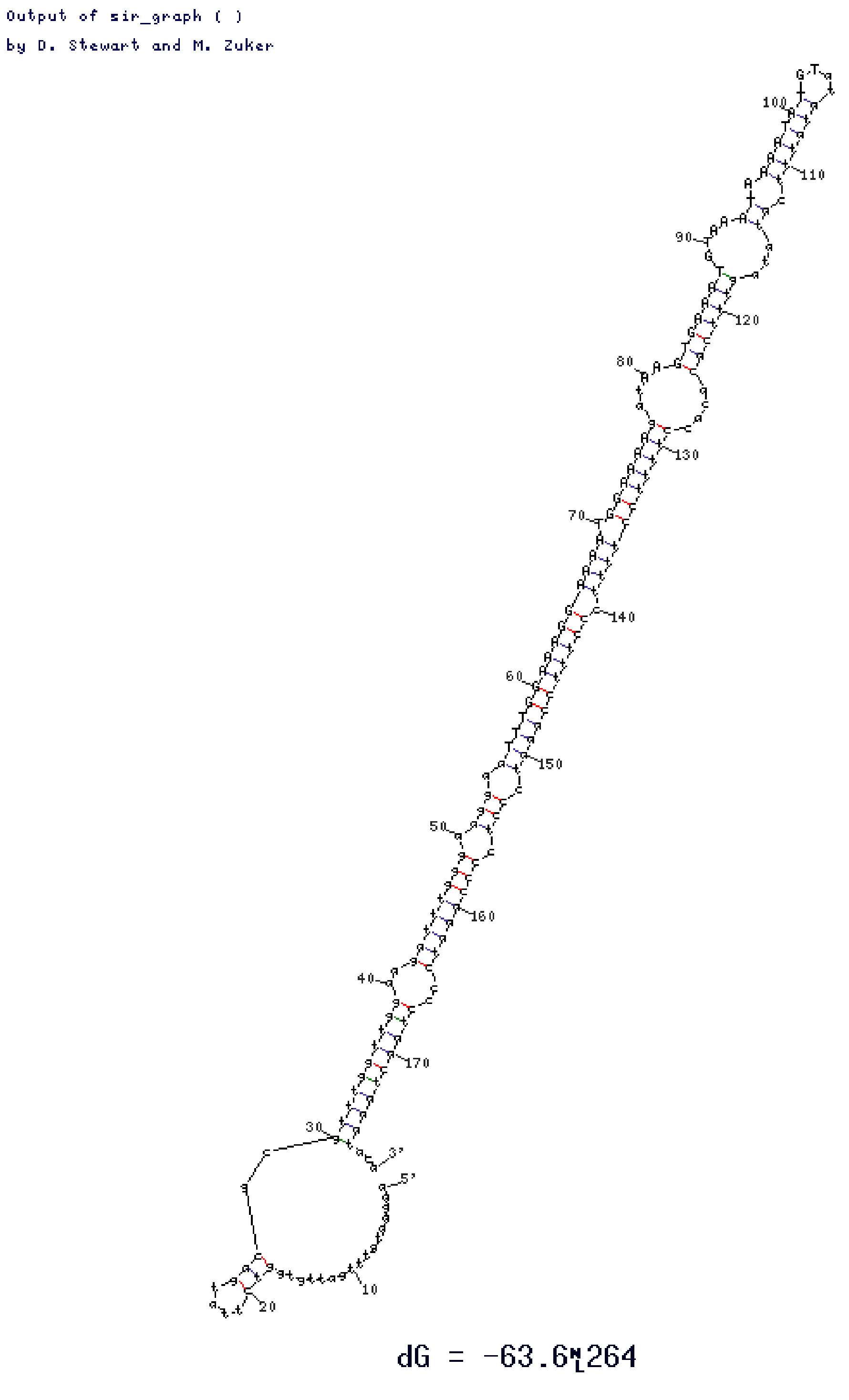

csn-smR32
UUUGGAAAGGGAAAUGGAAAA

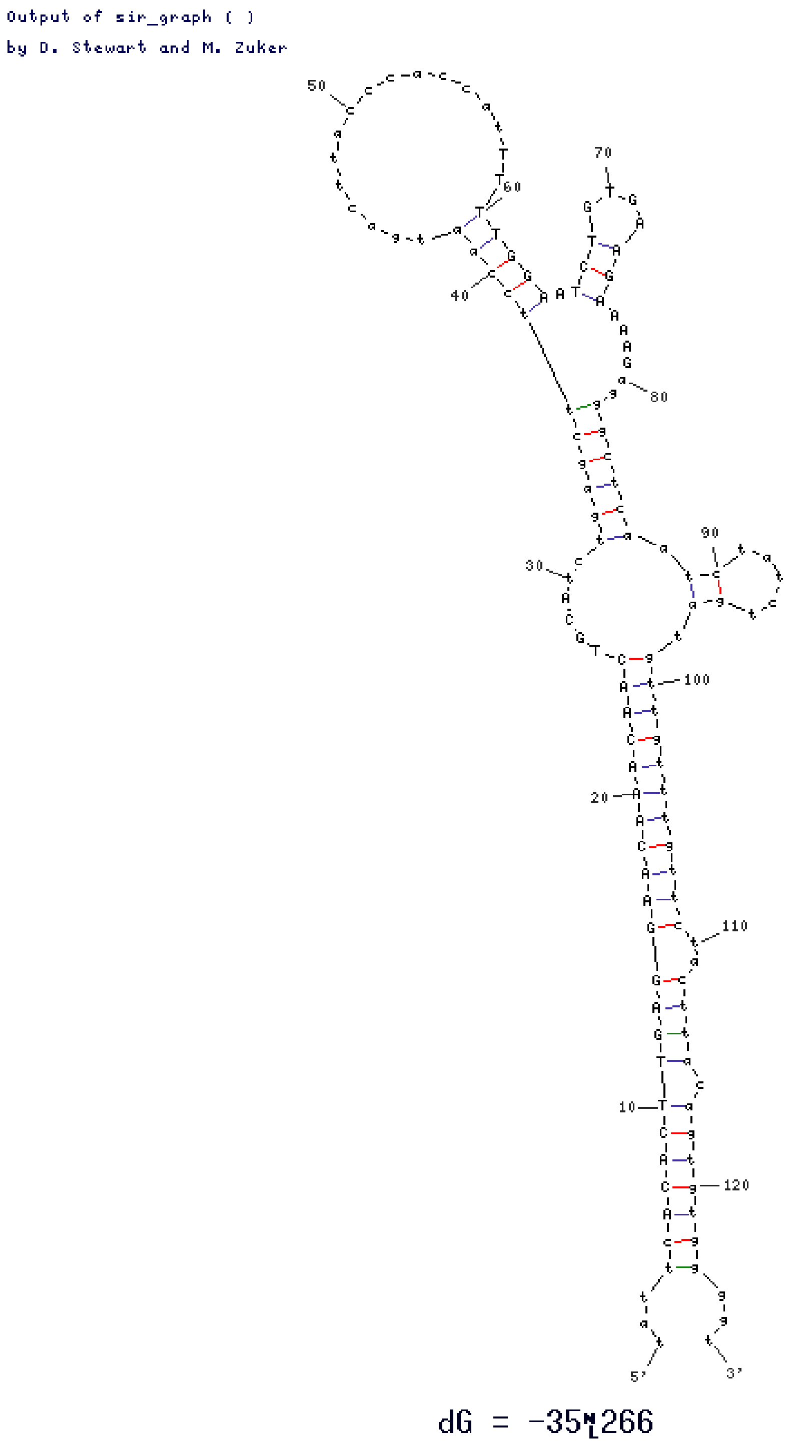

csn-smR33-5p
ACACUUGAGGAACAAACAACUGCA

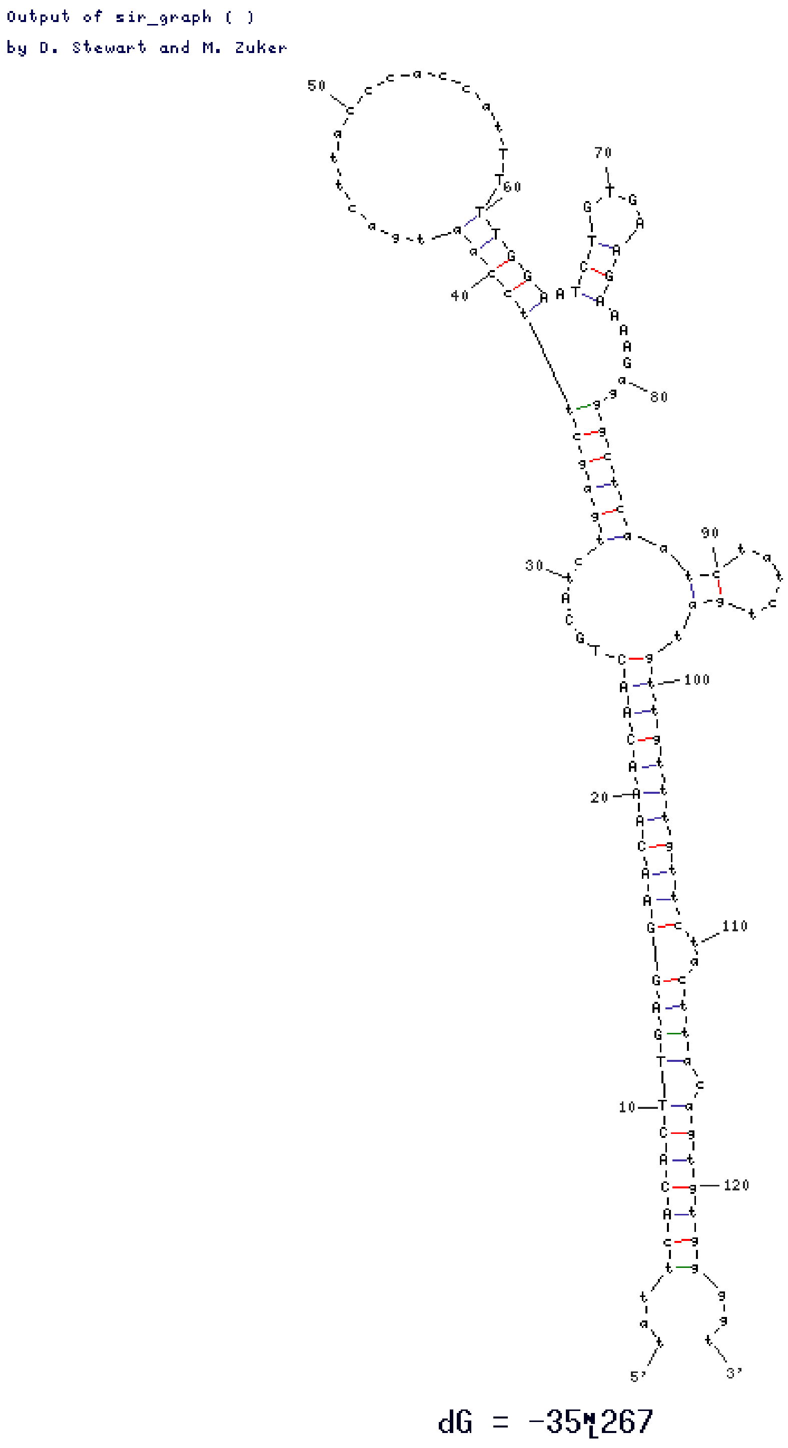

csn-smR33-3p
UUUUGGAGUCUGUGAAGAAAAG

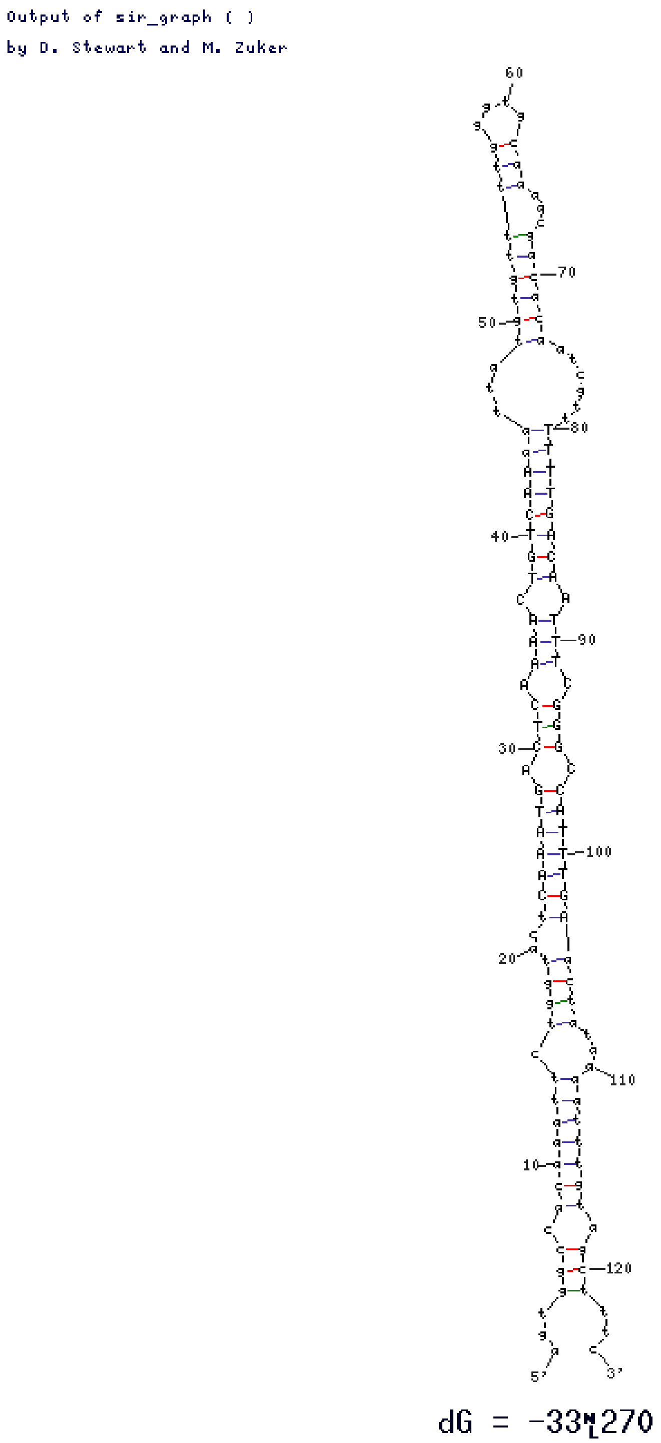

csn-smR34-5p
CAAAUGGCUCAAAACUGUCAA

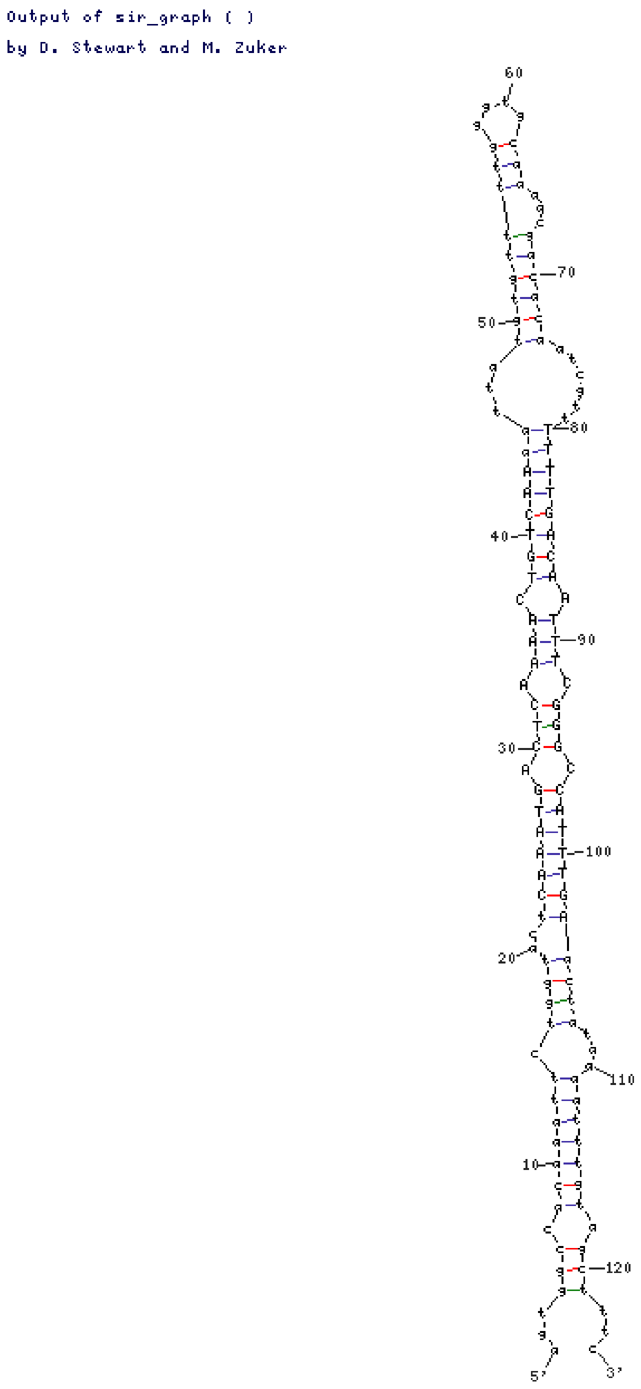

dG = −33[illegible]271

csn-smR34-3p
UUUUGACAUUUUCGGGCCAUUUGA

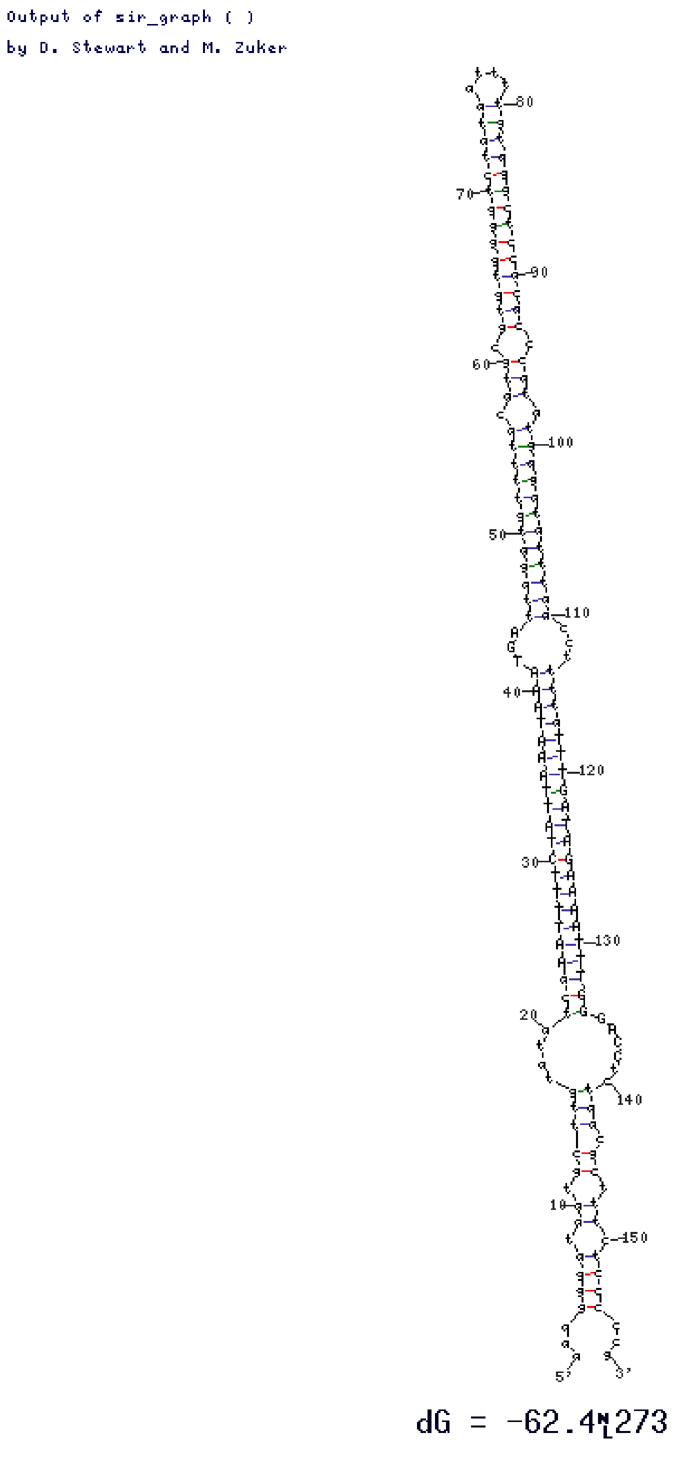

csn-smR35
UUUGGUAGAAAAUUUGGGACC

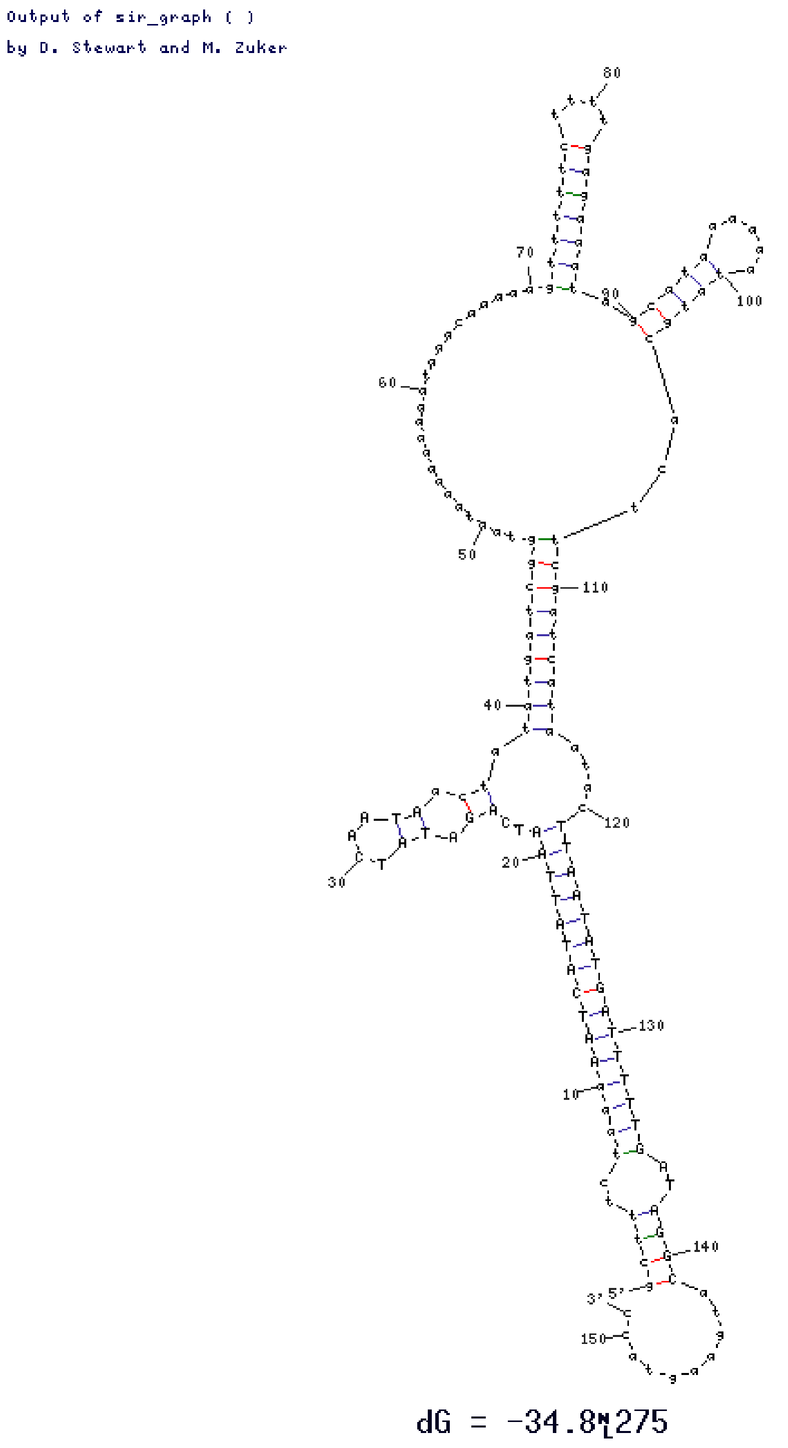

csn-smR36
UUAAUAUGAUUUUUGGUAGGC

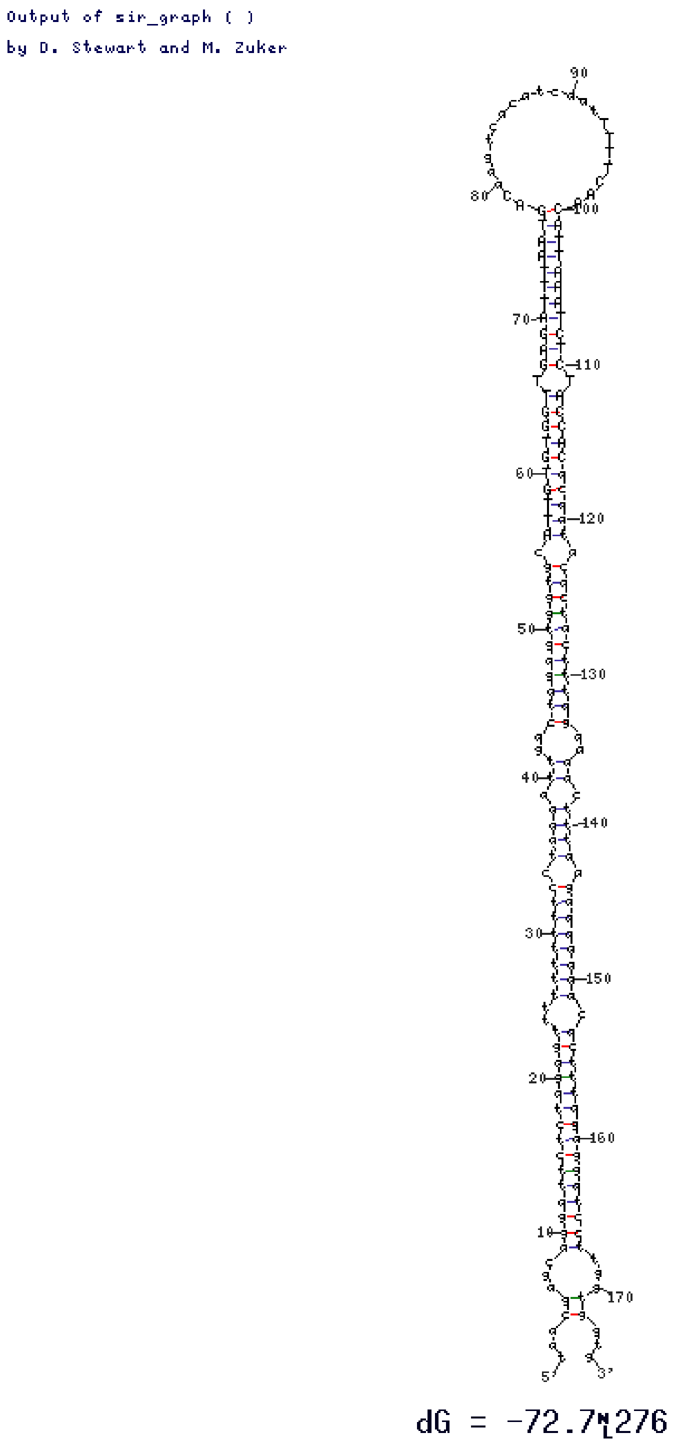

csn-smR37
AUUGUGUGGUUGAGAUUUAAUGAC

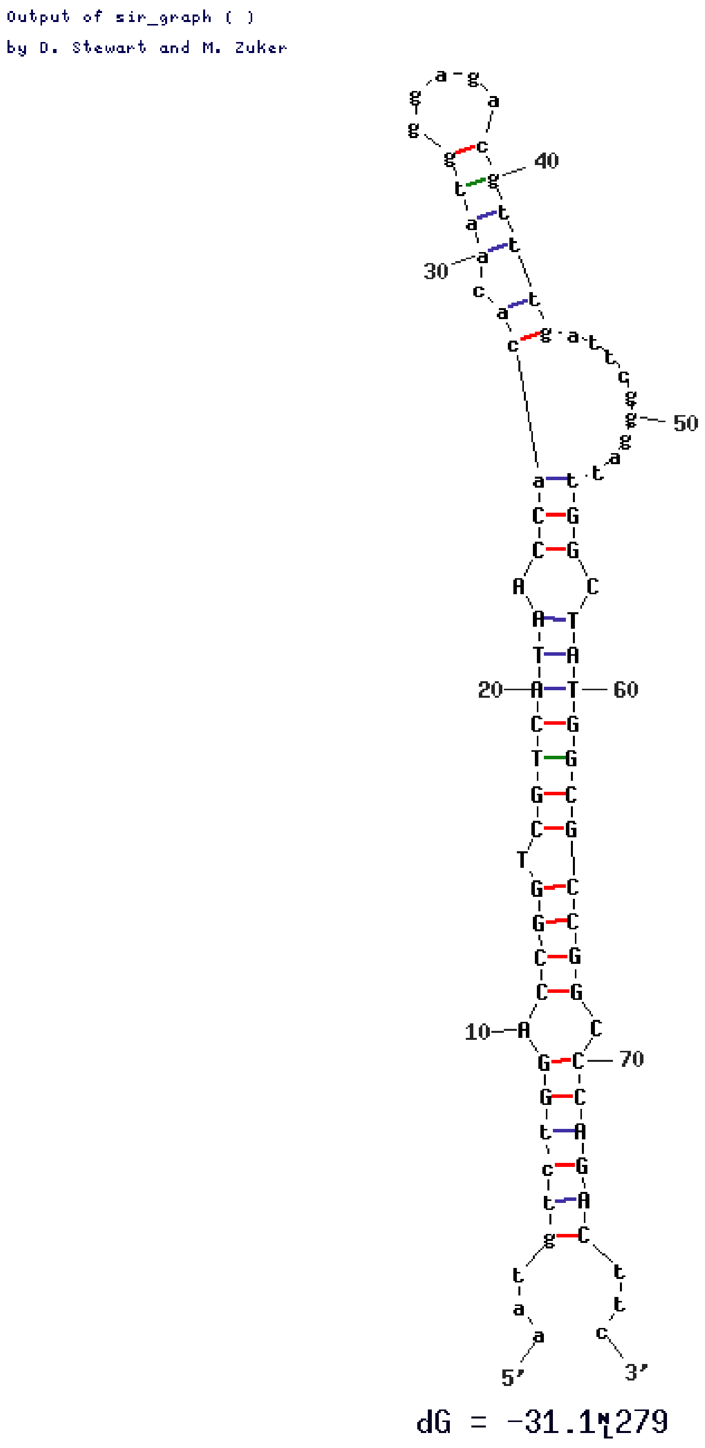

csn-smR38-3p
GGCUAUGGCGCCGGCCCAGAC

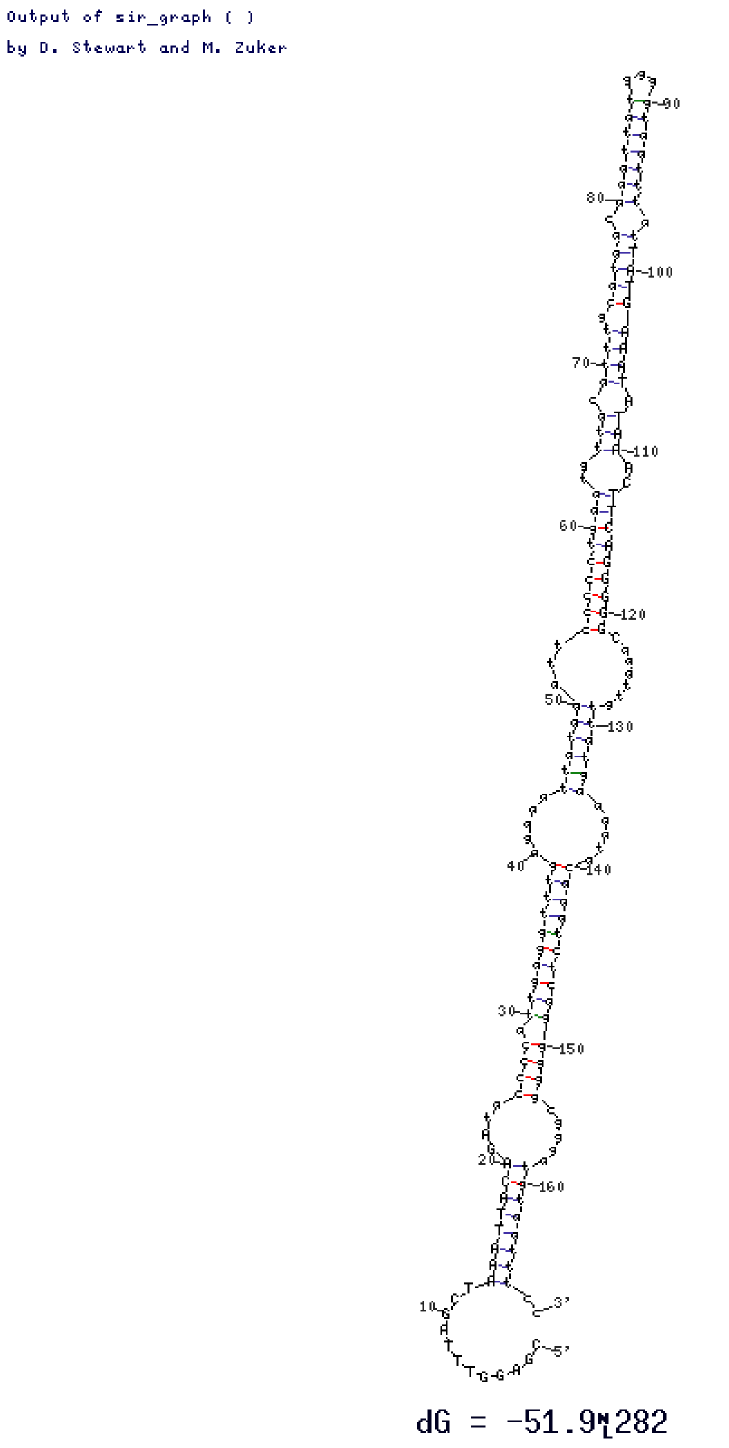

csn-smR39-5p
CGAGGUUUAGCUAAAUUACAGA

Output of sir_graph ( )
by D. Stewart and M. Zuker

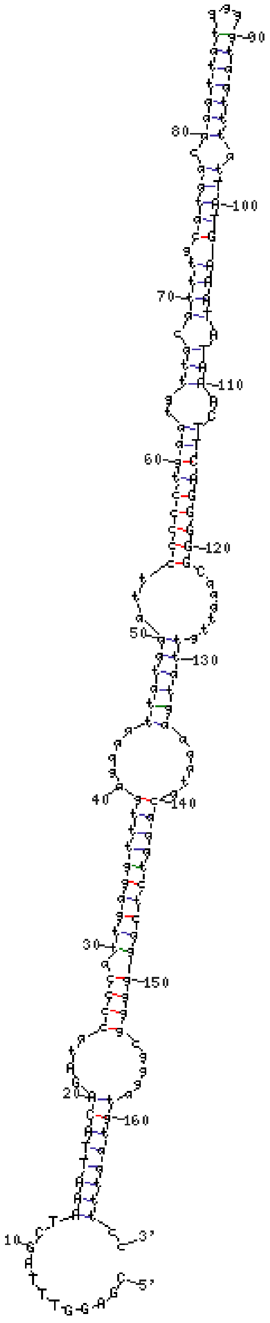

dG = -51.9 283

csn-smR39-3p
UAUGAAAUAUAAACUUCAGGGGGC

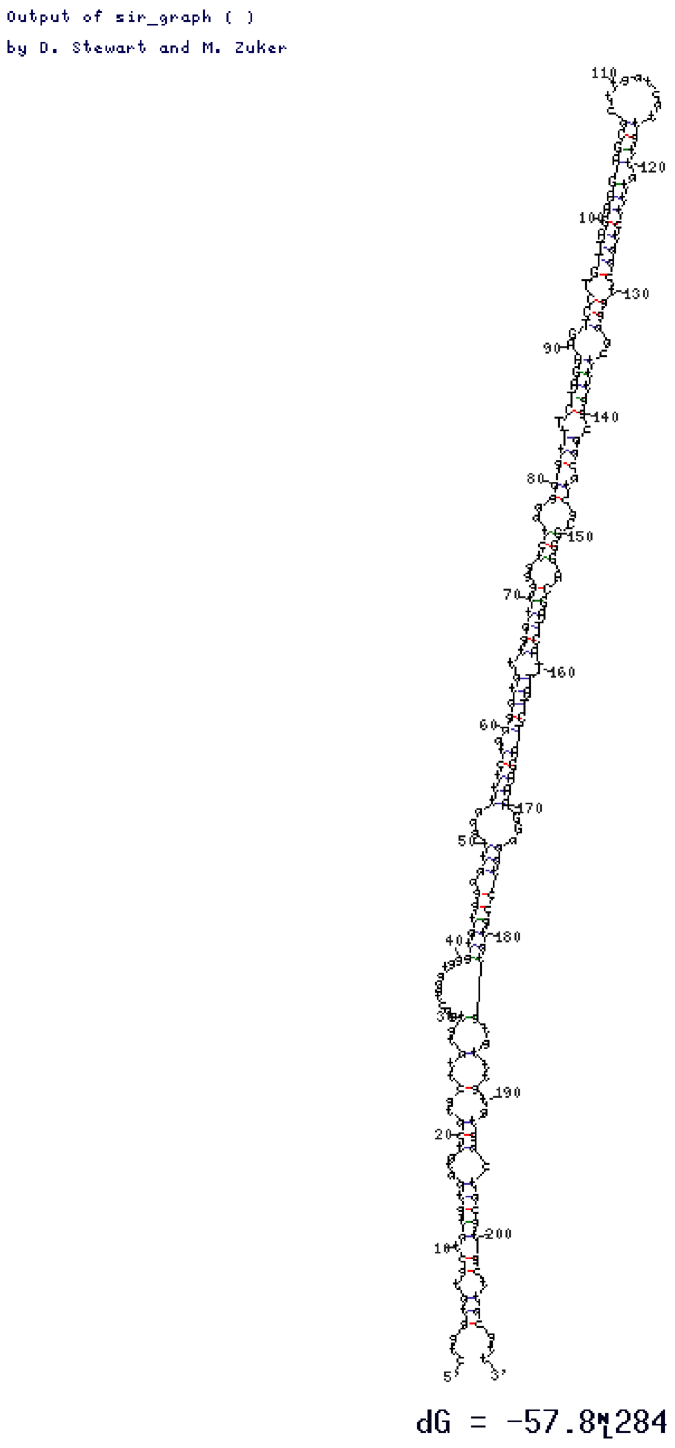

csn-smR40-5p
UUCUAGAAGUCCUAUUAGAAGAGC

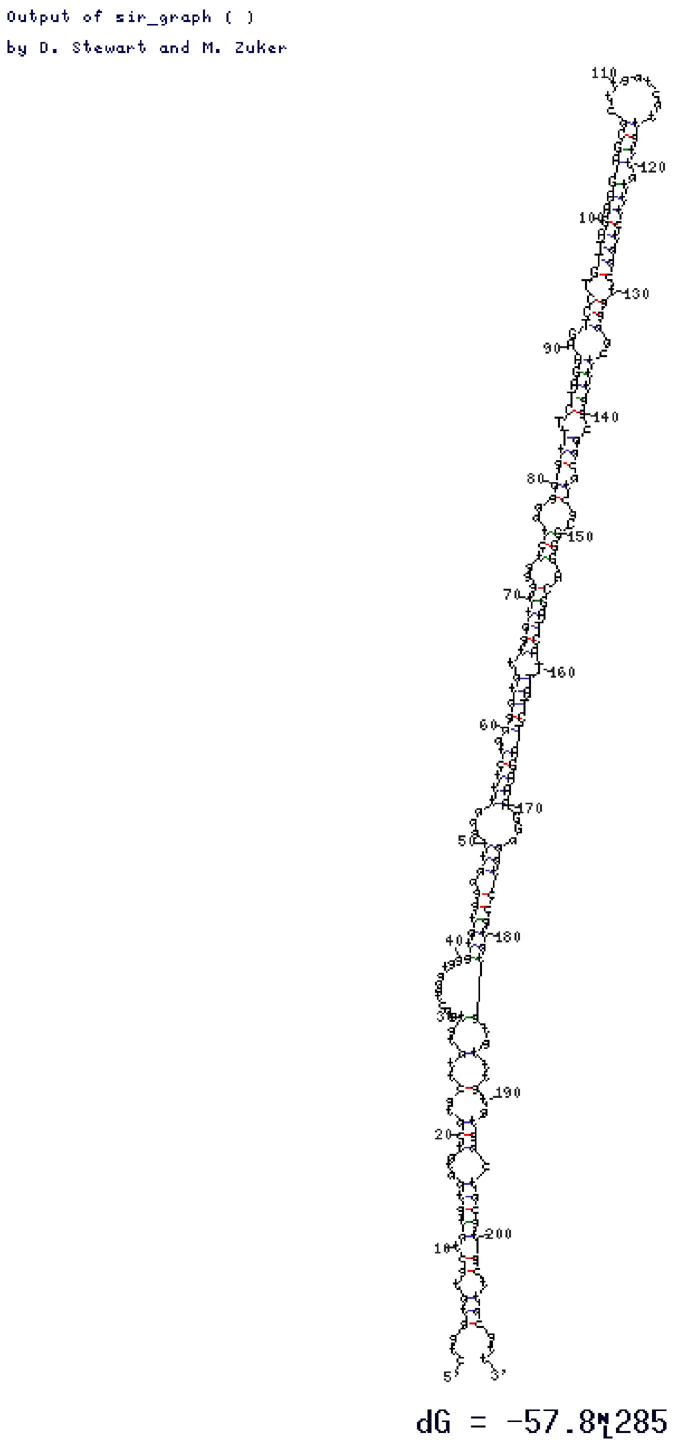

csn-smR40-3p
CGGGACGAUCAUUAUUUAGAAAGG

Output of sir_graph ( )
by D. Stewart and M. Zuker

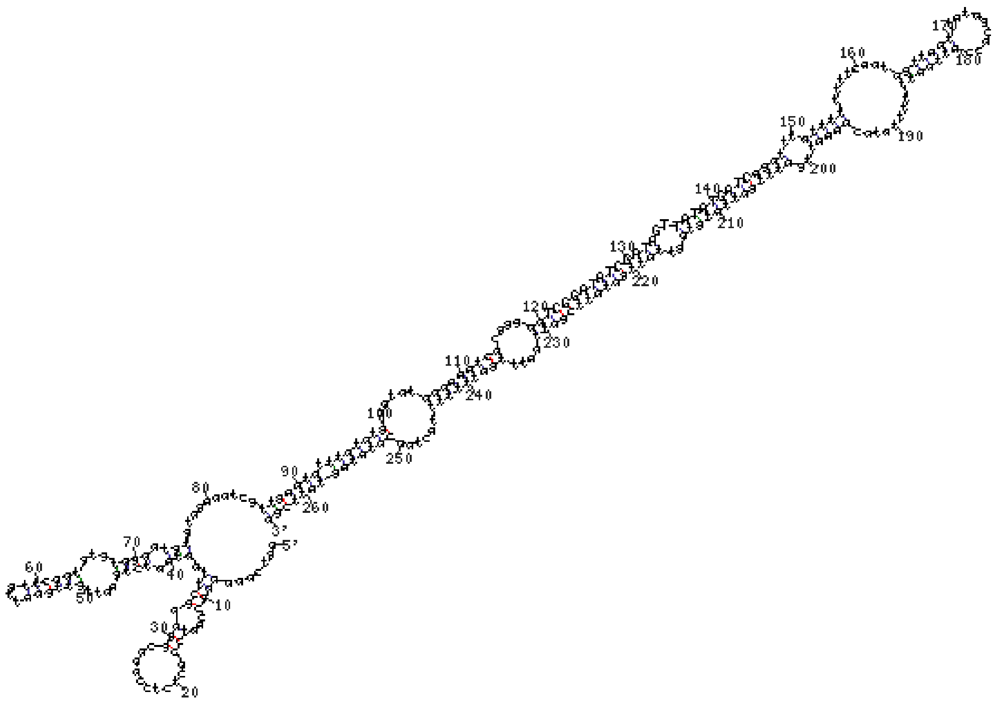

dG = -62.1␤286

csn-smR41-5p
CGGAUAUCGAUAGUUAUGUGA

Output of sir_graph ( )
by D. Stewart and M. Zuker

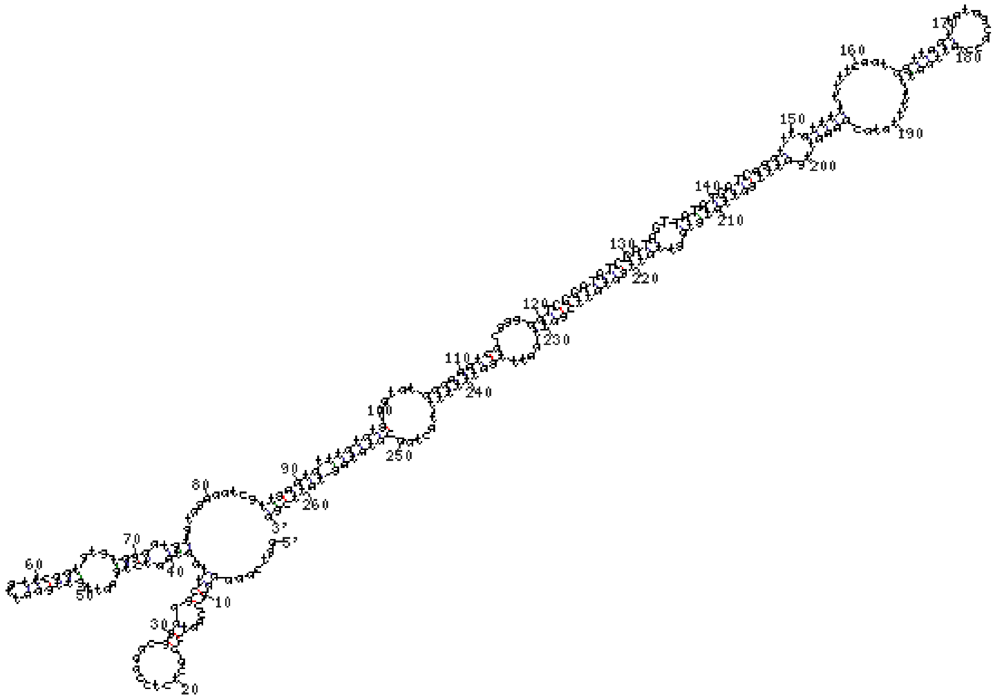

dG = -62.1 287

csn-smR41-3p
UCGGAUAUCGAUAGCUAUAUGAUC

Output of sir_graph ( )
by D. Stewart and M. Zuker

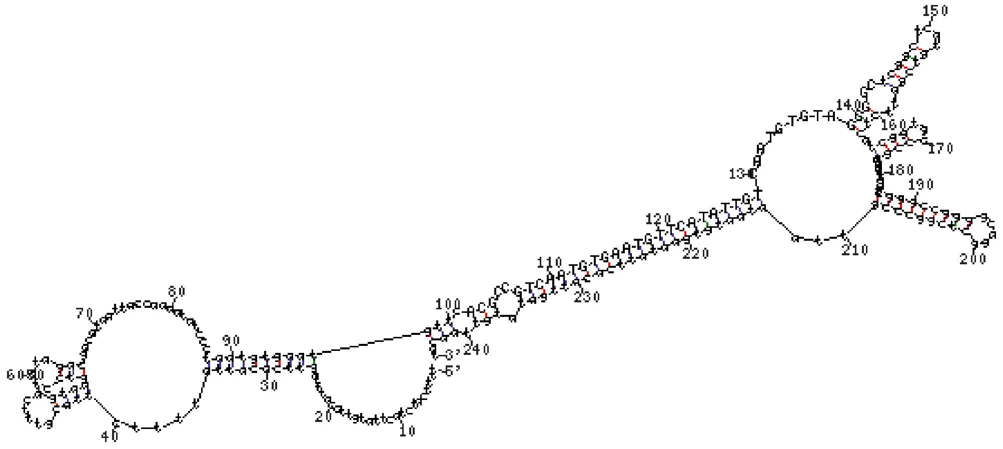

dG = -75.7 [illegible] 288

csn-smR42
ACACCGUCAAUGUGAAUGUUCAUA

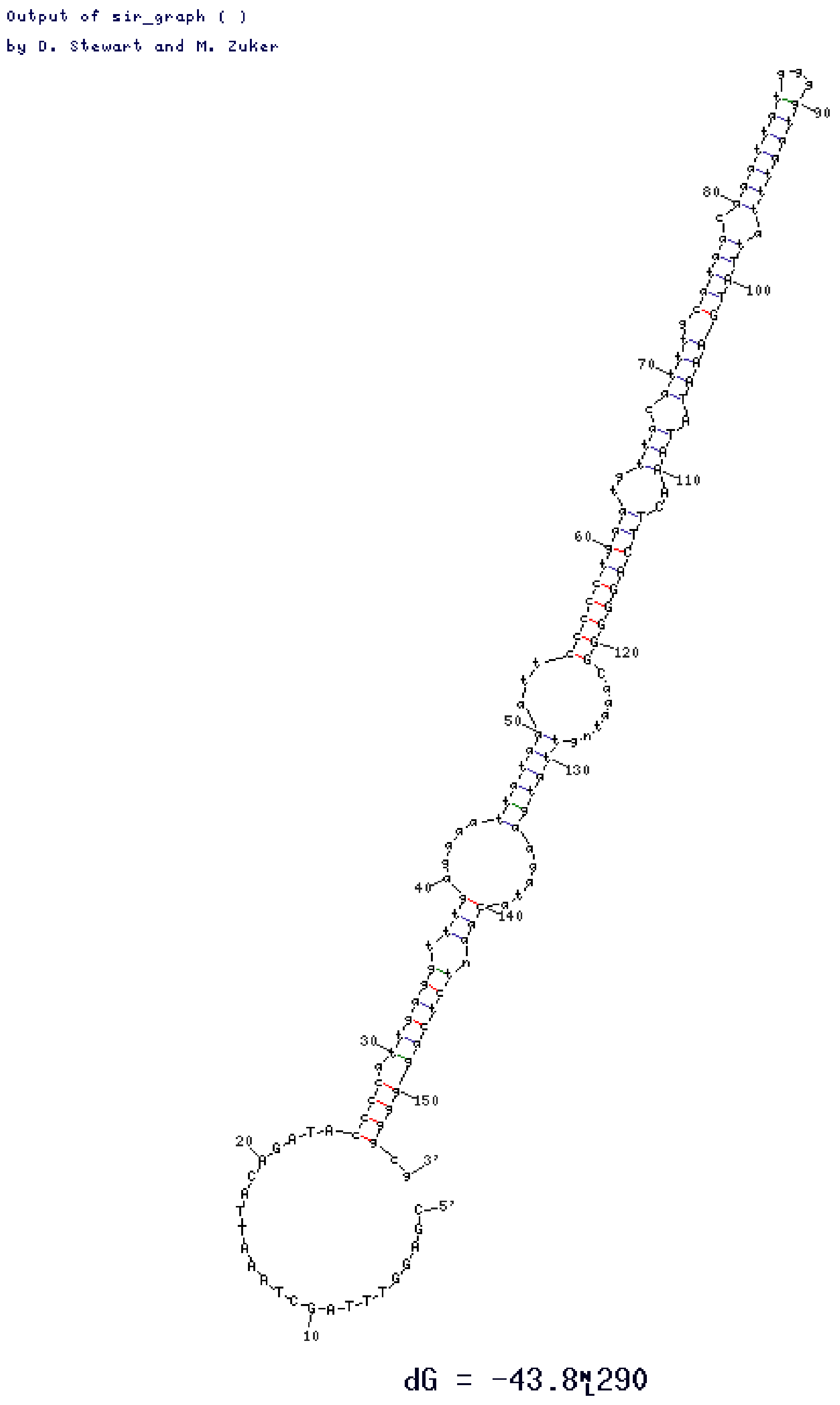

csn-smR43-5p
CGAGGUUUGGCUAAAUUACAGAUA

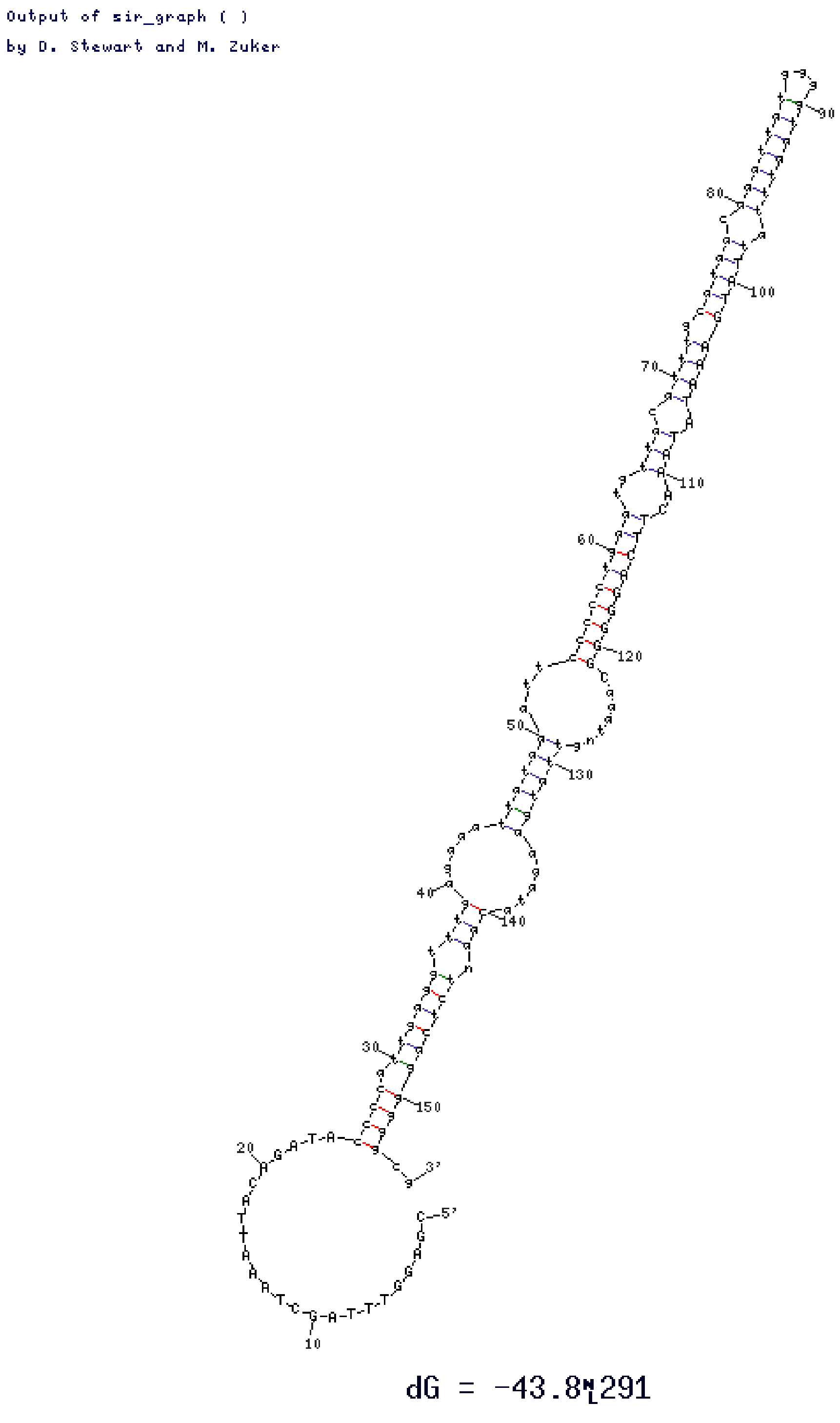

csn-smR43-3p
UAUGAAAUAUAAACCUCAGGGGGC

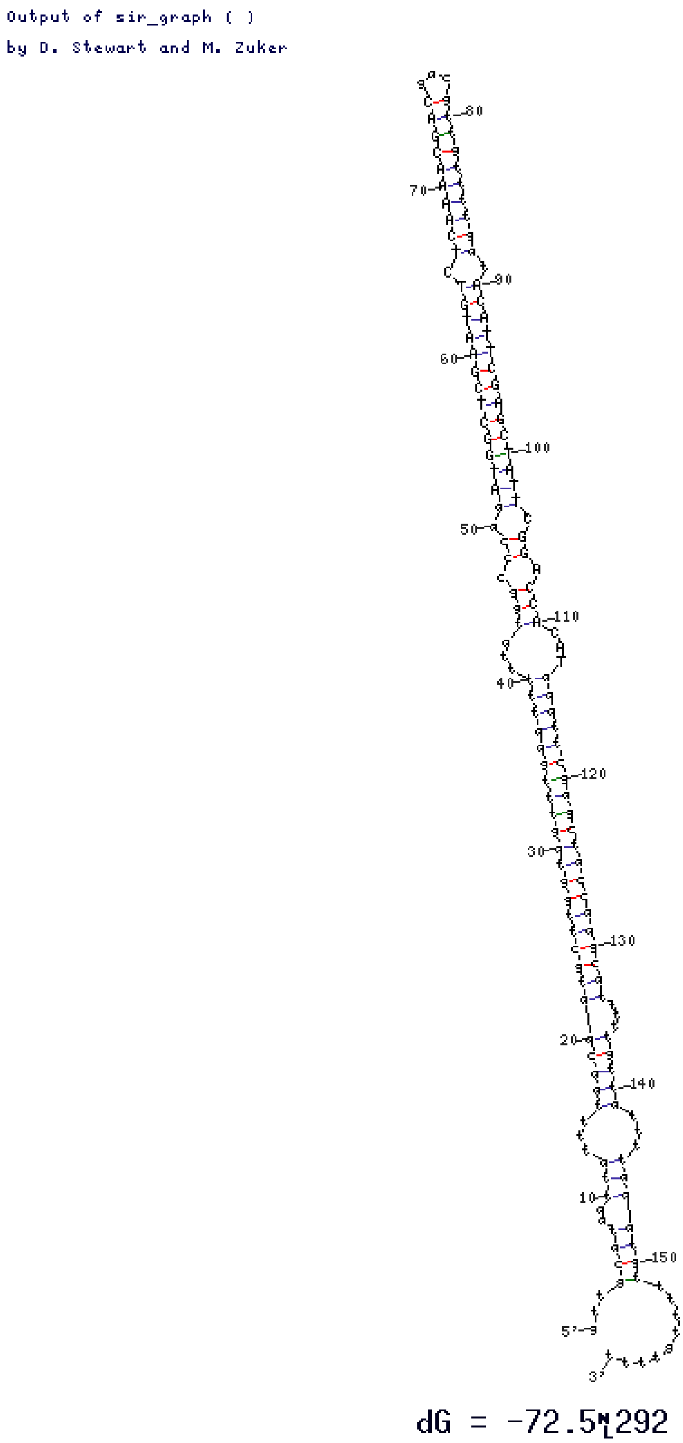

csn-smR44-5p
AUGACUCGAAUGUCUCAAAACGAC

Output of sir_graph ( )
by D. Stewart and M. Zuker

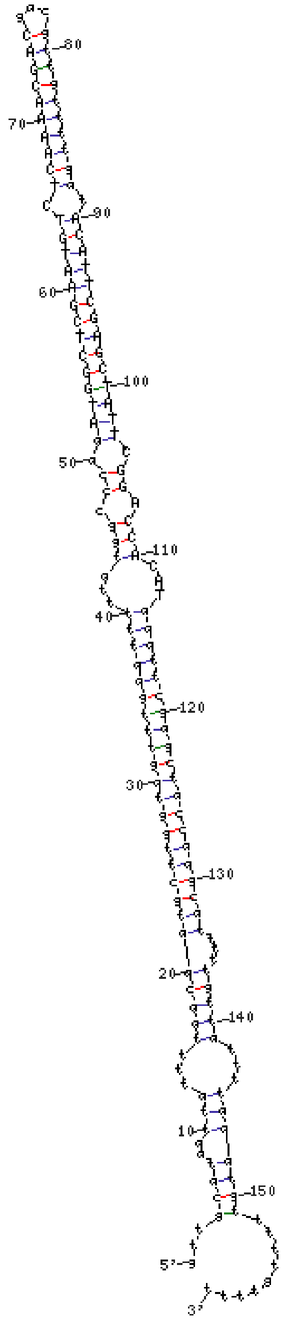

csn-smR44-3p
ACAUUCGAGCUAUUCGGACCACAC

Output of sir_graph ( )
by D. Stewart and M. Zuker

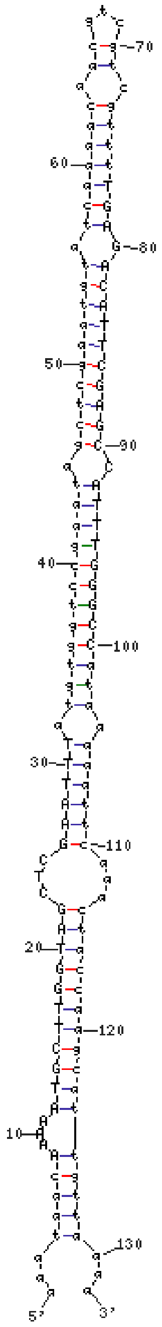

dG = -66.5 [illegible] 294

csn-smR45-5p
AAAAAUGAUUGGUAGCUCGAAUUU

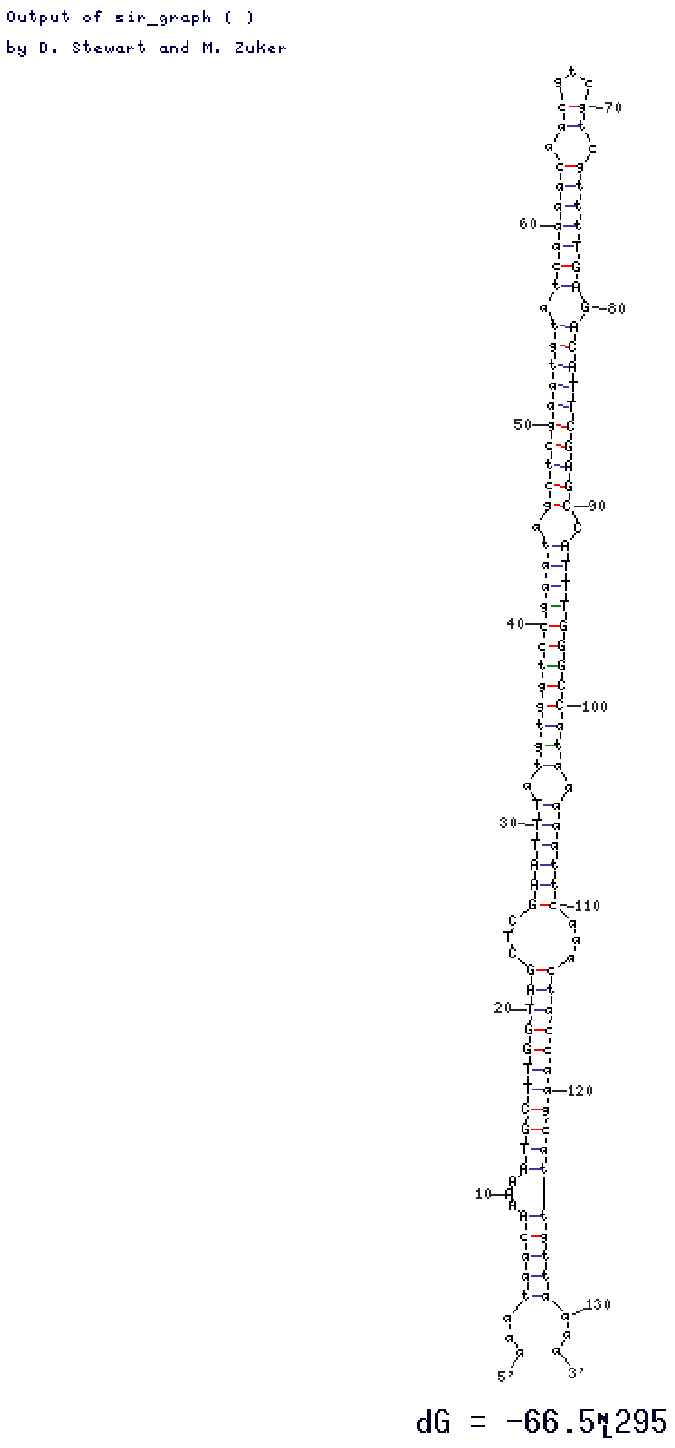

csn-smR45-3p
UGAGACAUUCGAGCCAUUUGAGCC

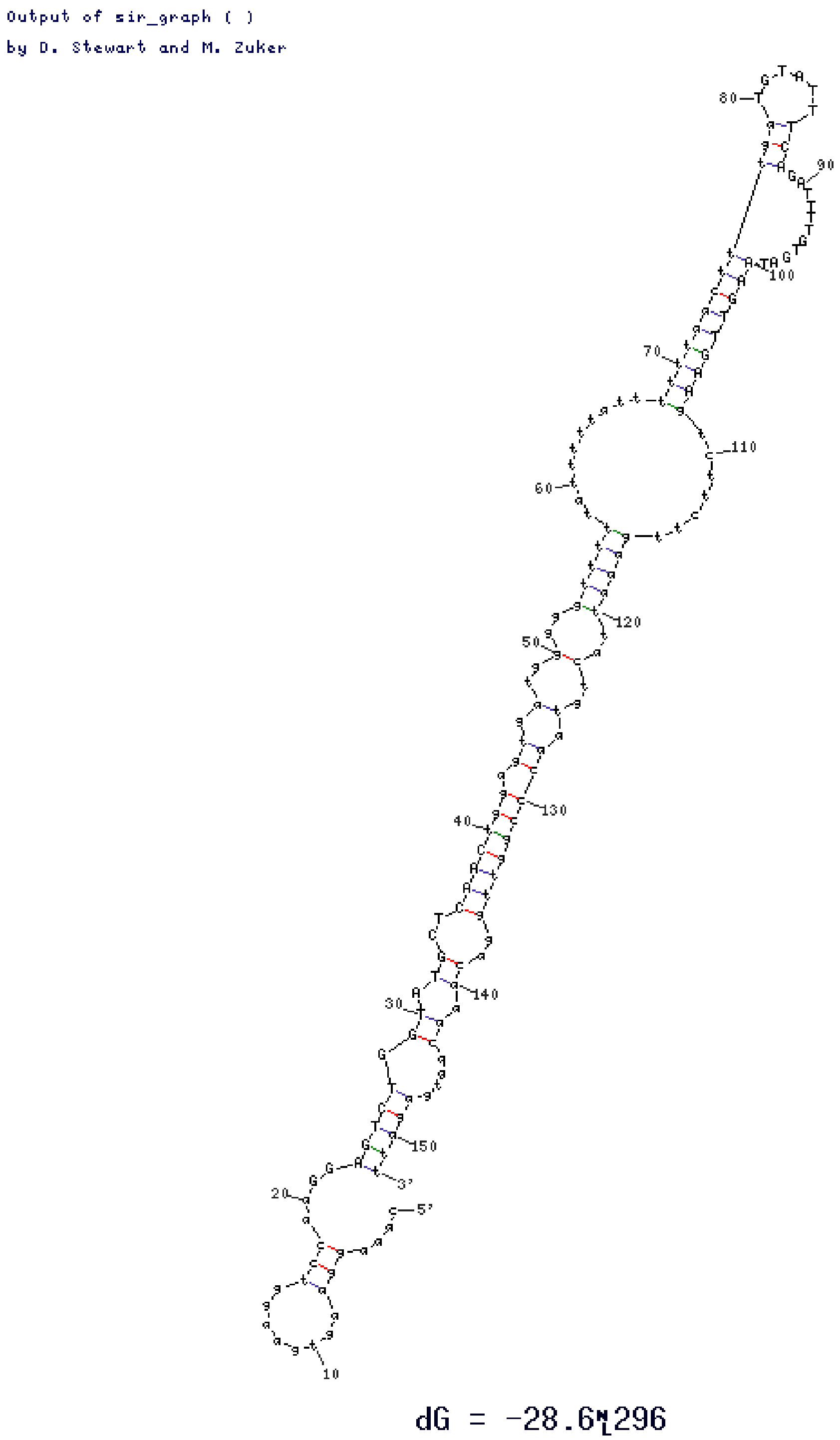

csn-smR46
GGAGUCUGGUAUGCUCAAC

Output of sir_graph ( )
by D. Stewart and M. Zuker

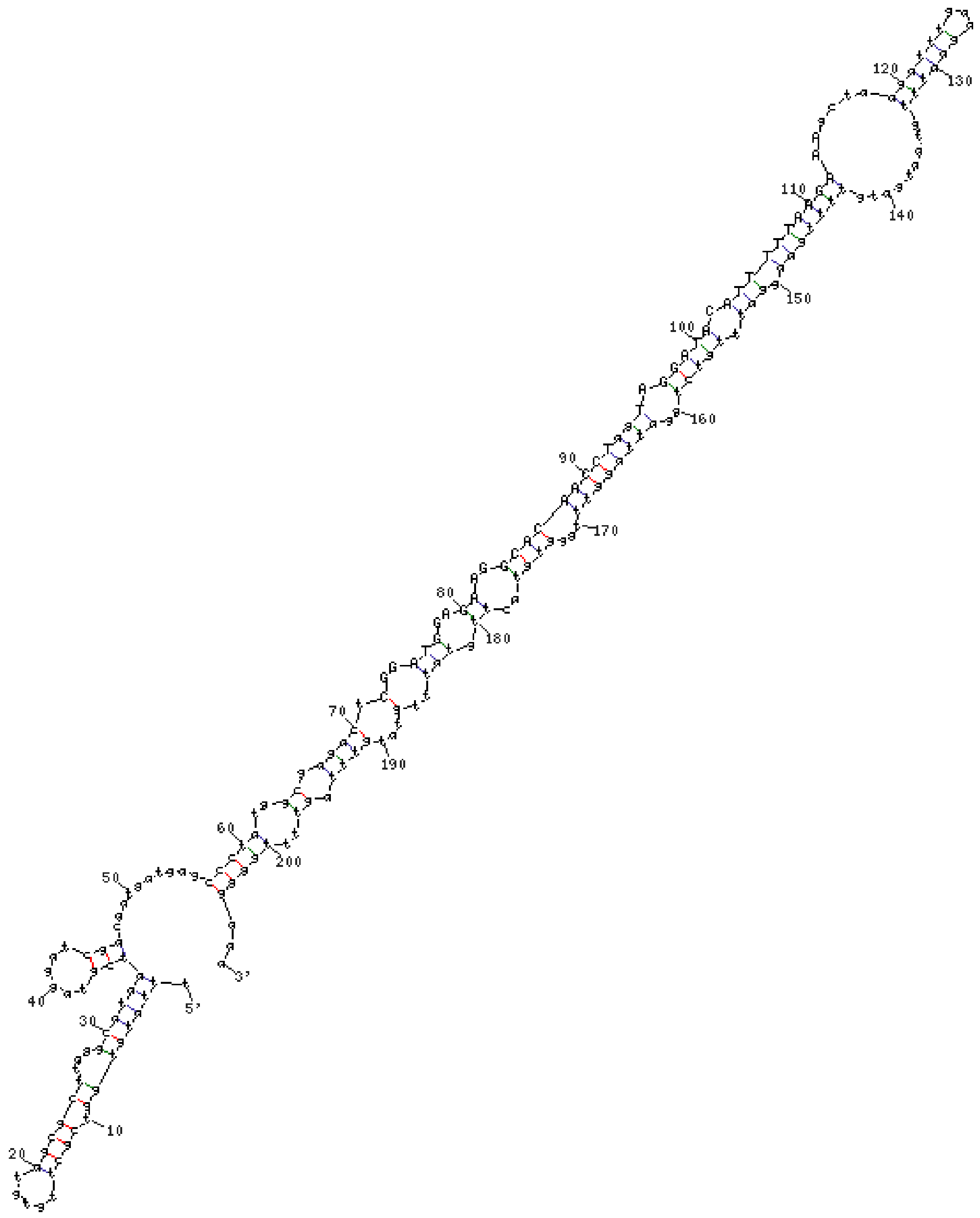

dG = -42.4 [illegible]298

csn-smR47-5p
CGGAUGGAGAAGGCACAACCU

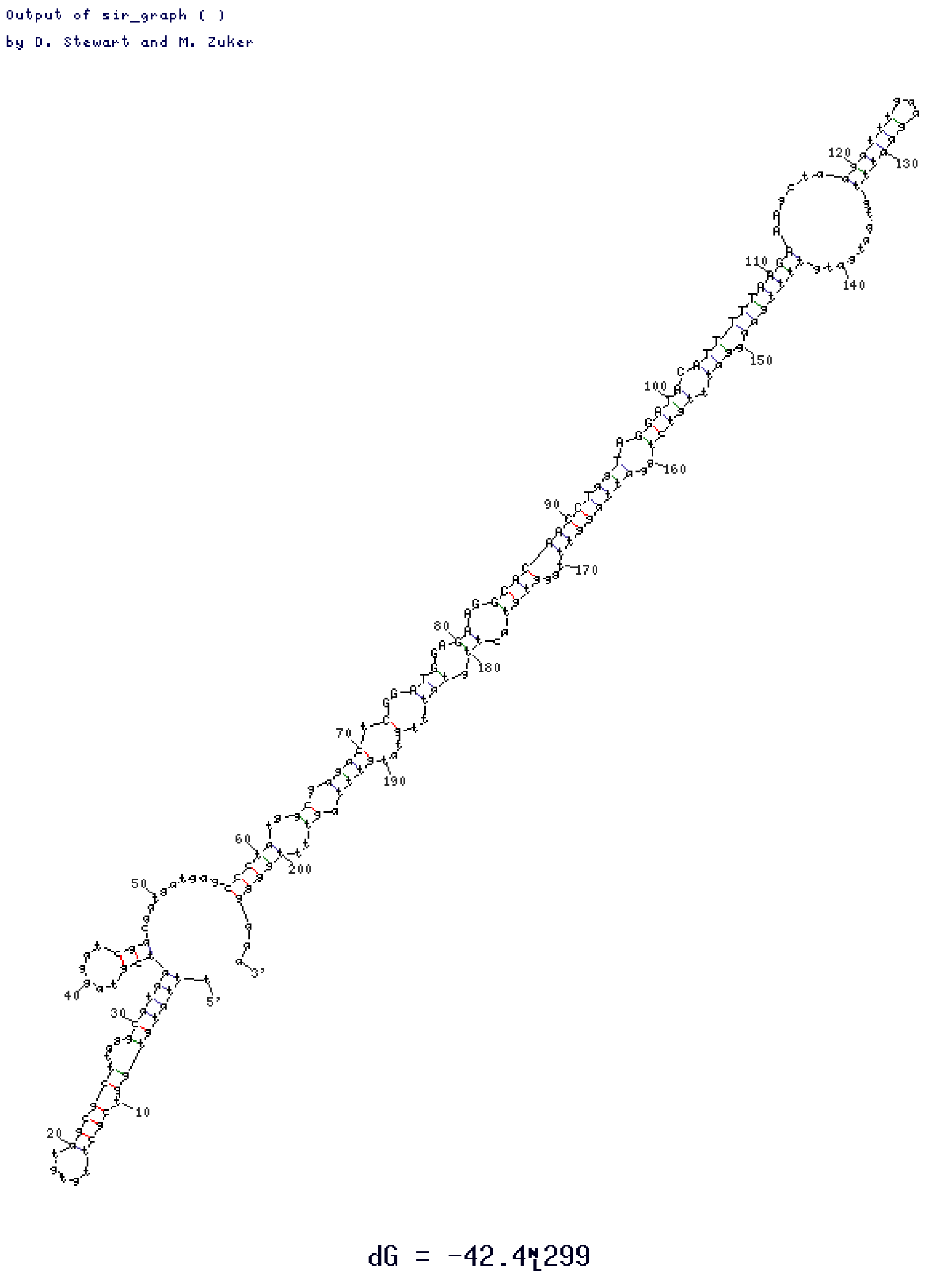

csn-smR47-3p
UAGGAUACAUUUUUAAGAAA

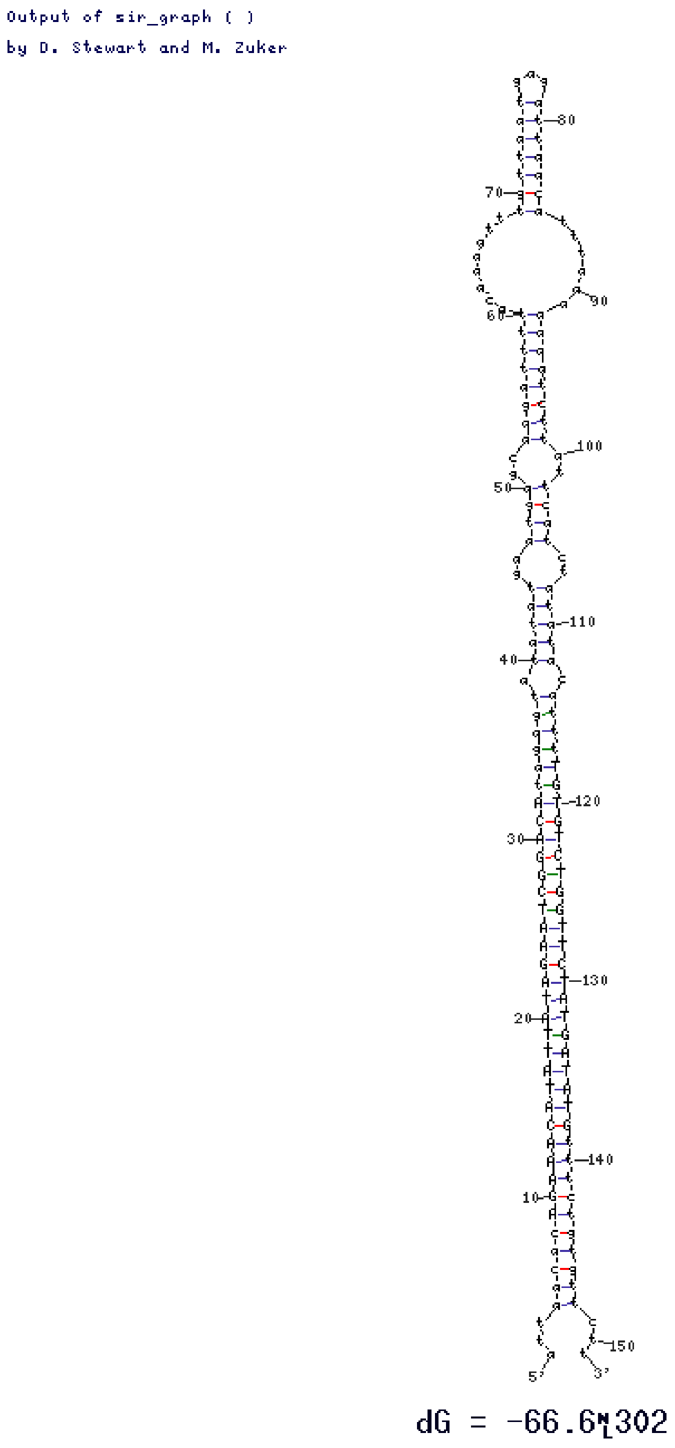

csn-smR49a-5p
AGAAACAUAUUAUAGAAUCGGACA

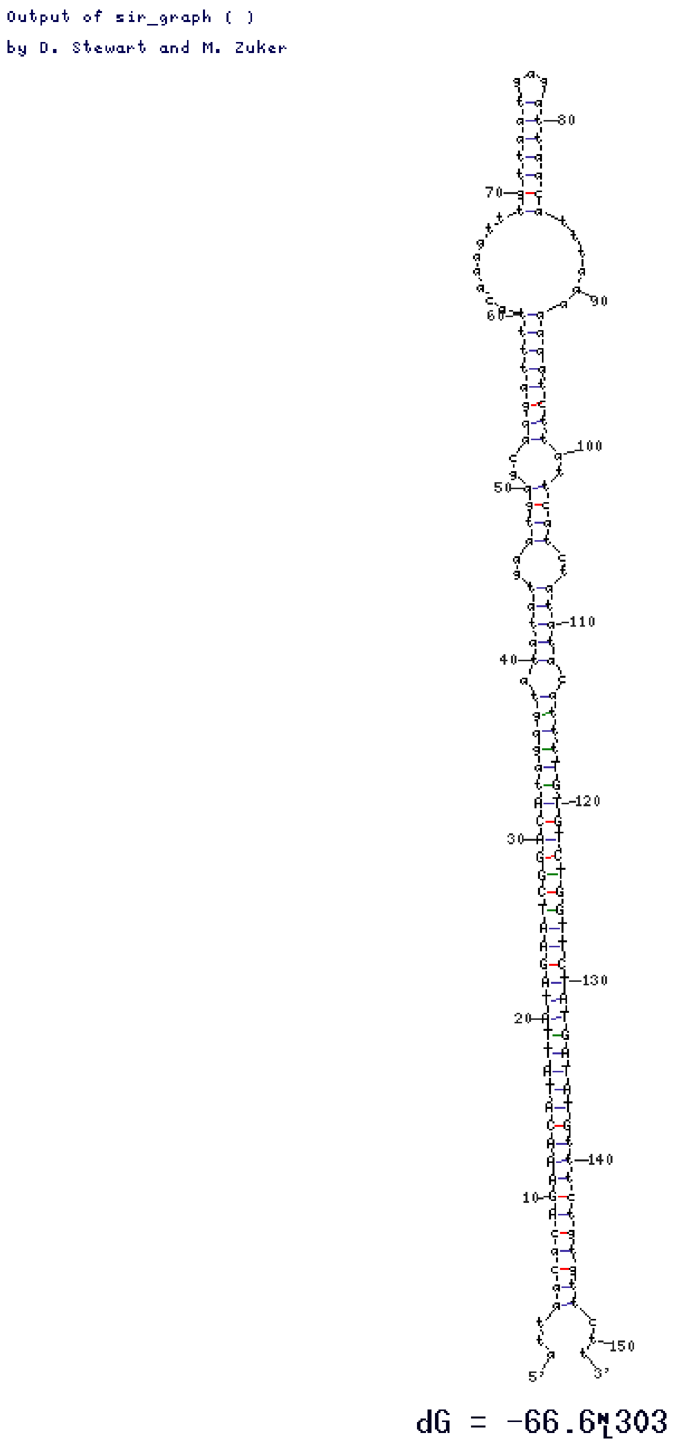

csn-smR49a-3p
UGUGUCUGGUUCUAUGAUAUG

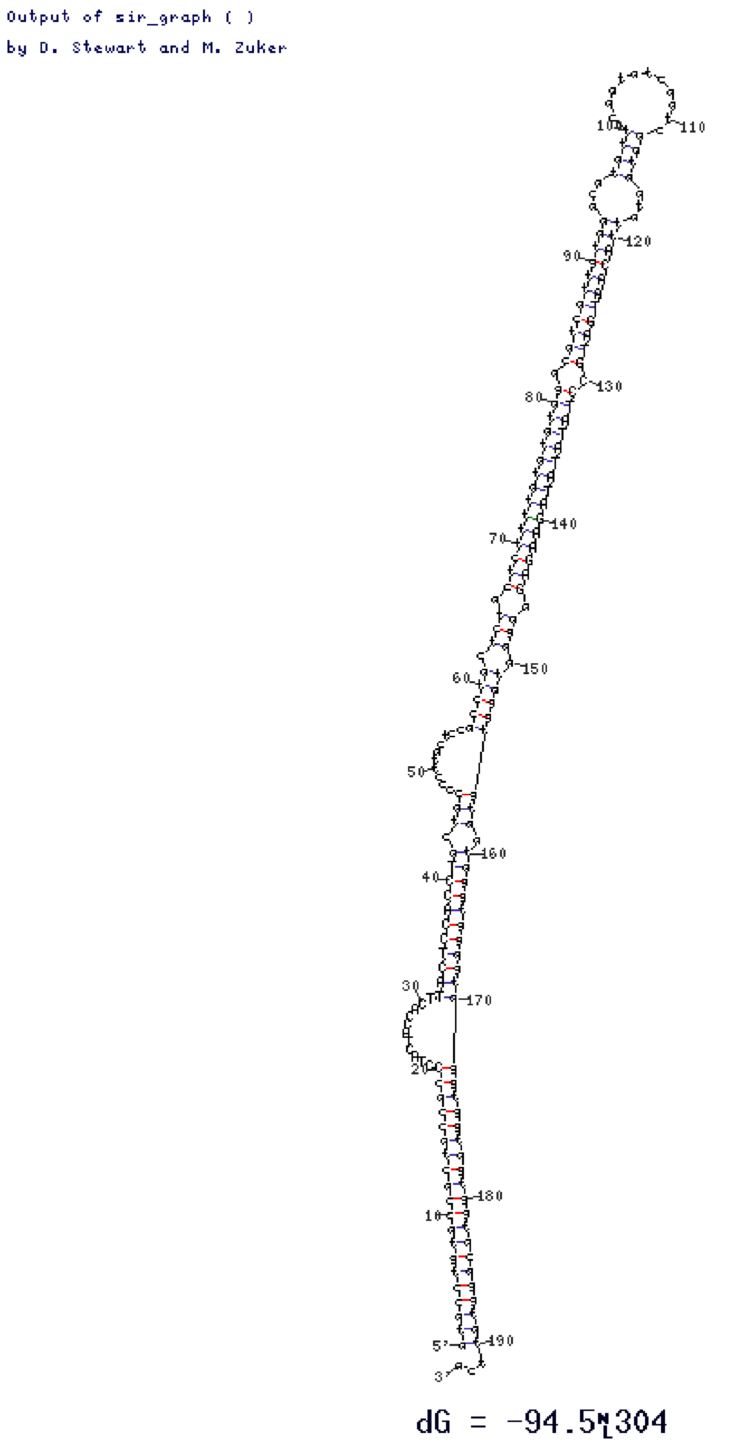

csn-smR50-5p
CCUACUACCACCUACUCCACCU

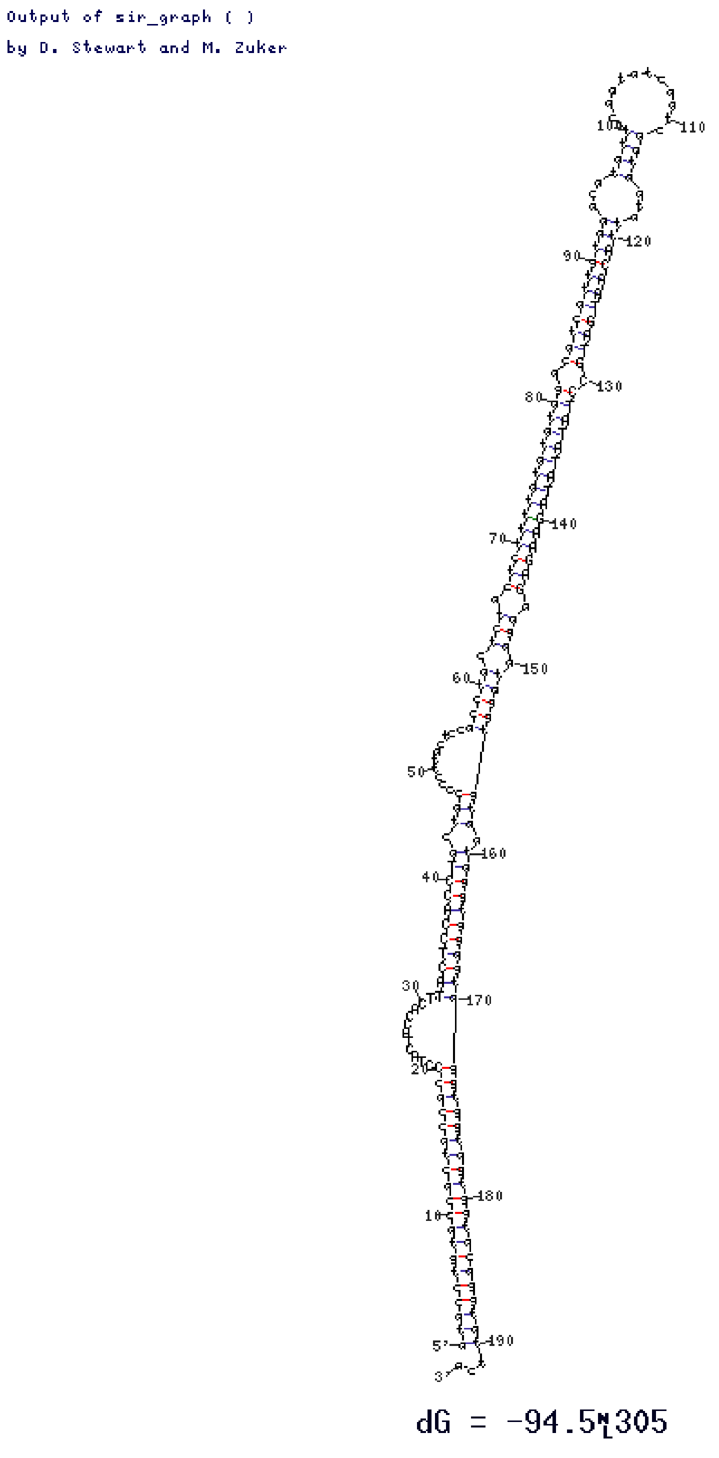

csn-smR50-3p
ACAAUGAUGCCUAUAUAUAGAAGAG

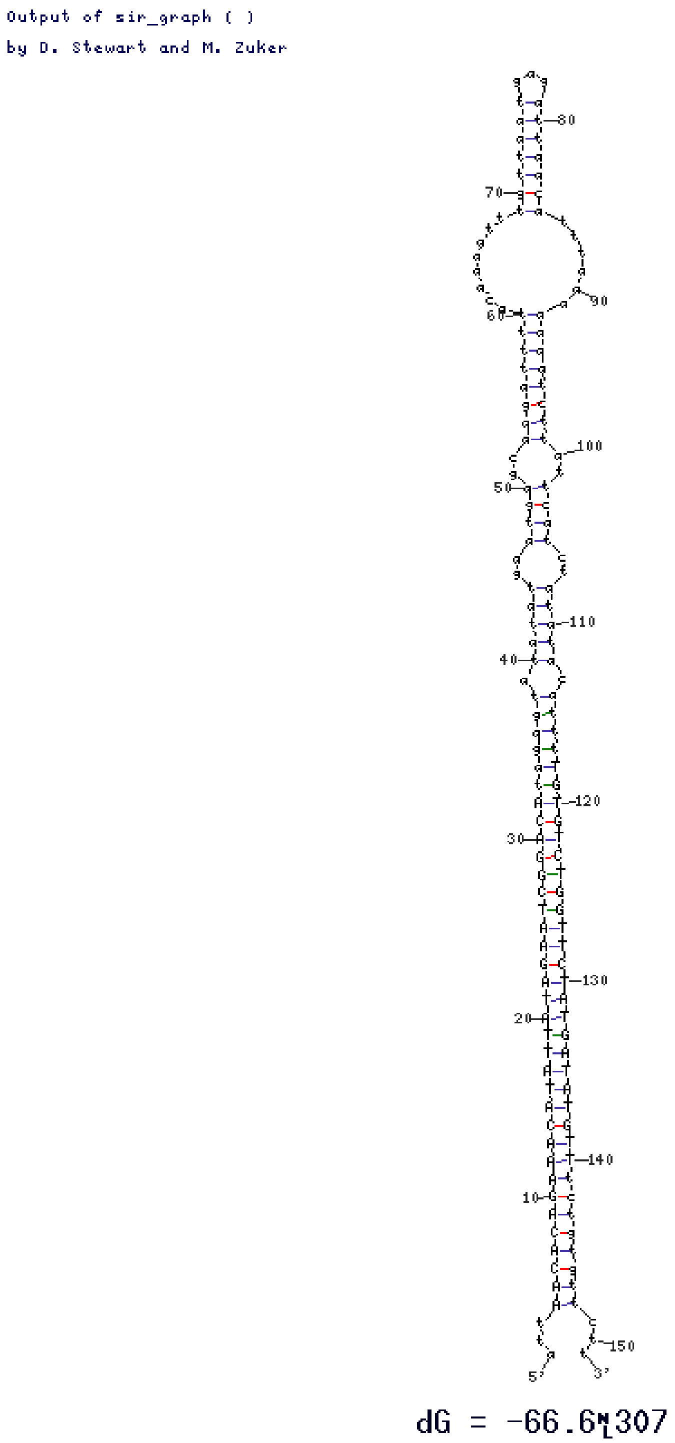

csn-smR49b-3p
UGUGUCUGGUUCUAUGAUAUGUU

Output of sir_graph ( )
by D. Stewart and M. Zuker

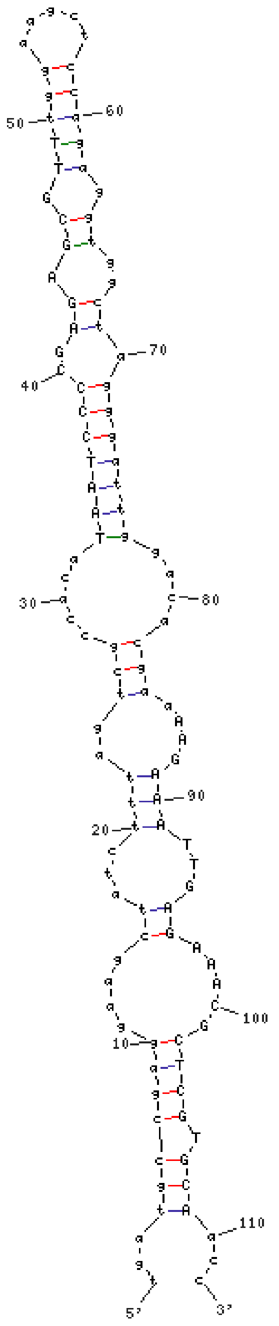

dG = -29.9[illegible]309

csn-smR52-3p
AAGAAAUUGAGAAACGCUCGUGCA

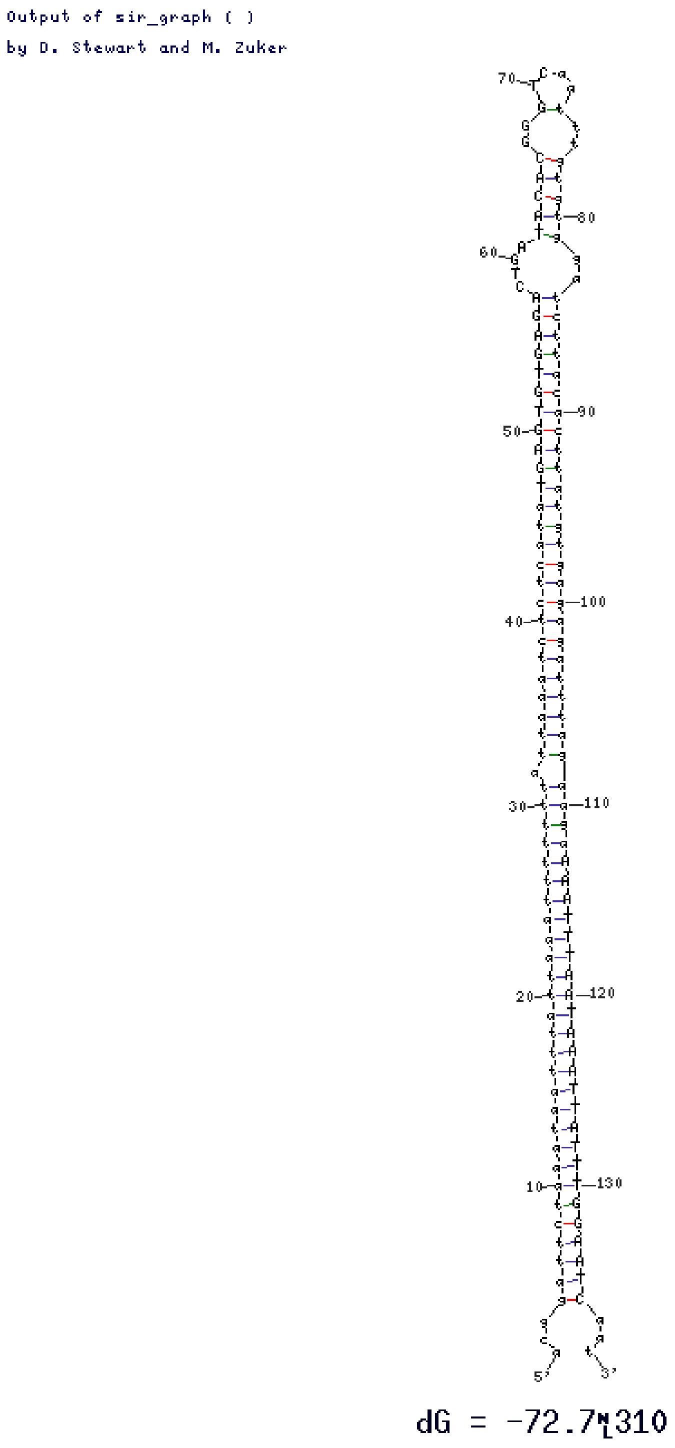

csn-smR53-5p
UGAGUGUGGGACUGAUACACGGGUC

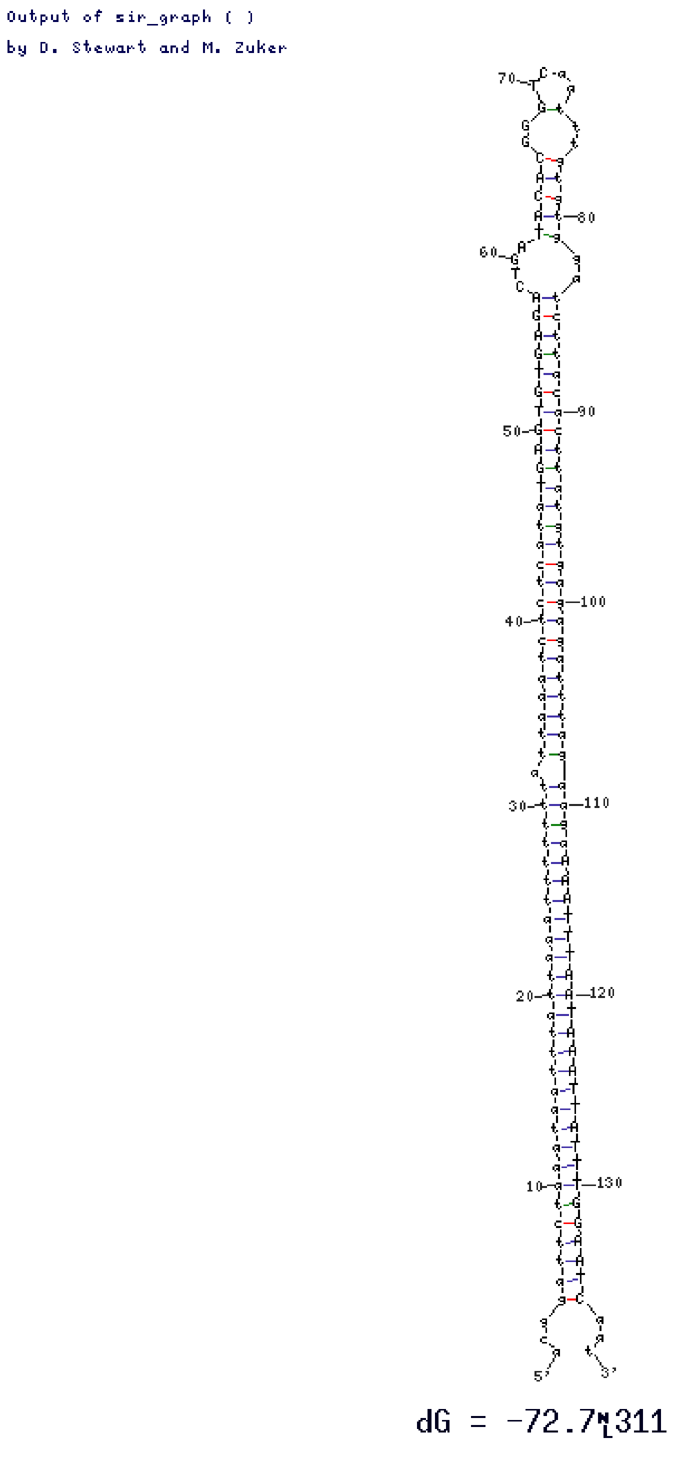

csn-smR53-3p
AAAUUUAAUAAAUUAUUUGGAACC

Output of sir_graph ( )
by D. Stewart and M. Zuker

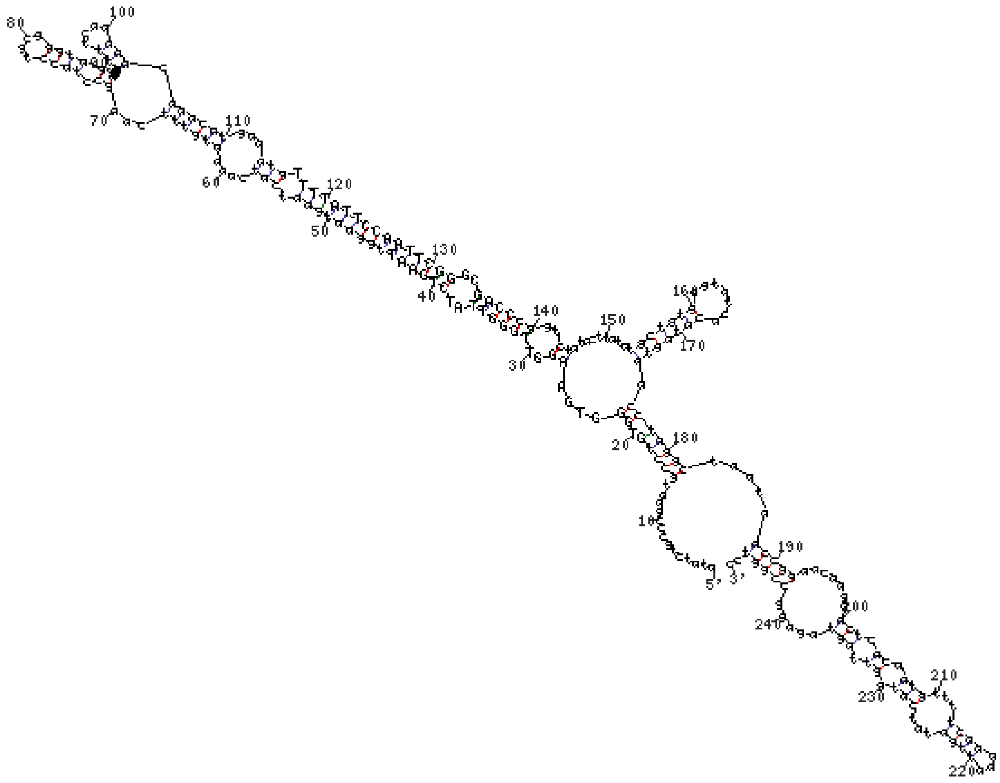

dG = -71.315

csn-smR55-3p
UUUUGUUCCAAUUCGGGCGACC

Output of sir_graph ( )
by D. Stewart and M. Zuker

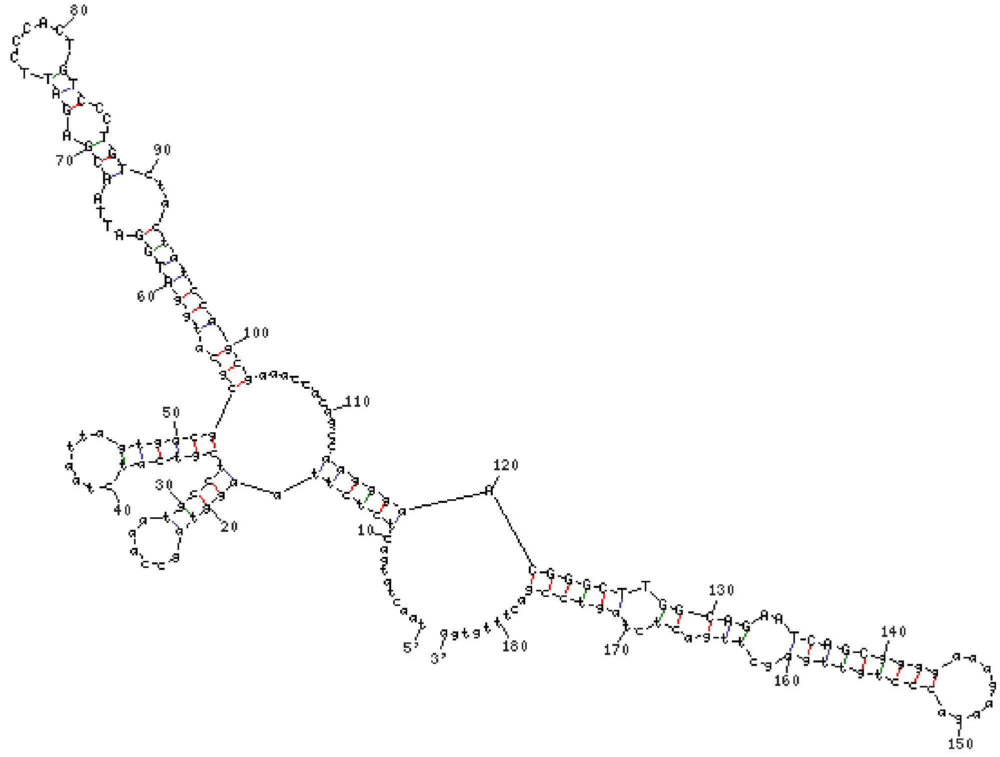

dG = -53.6[illegible]319

csn-smR56
ACGGGCUUGGCAGAAUCAGC

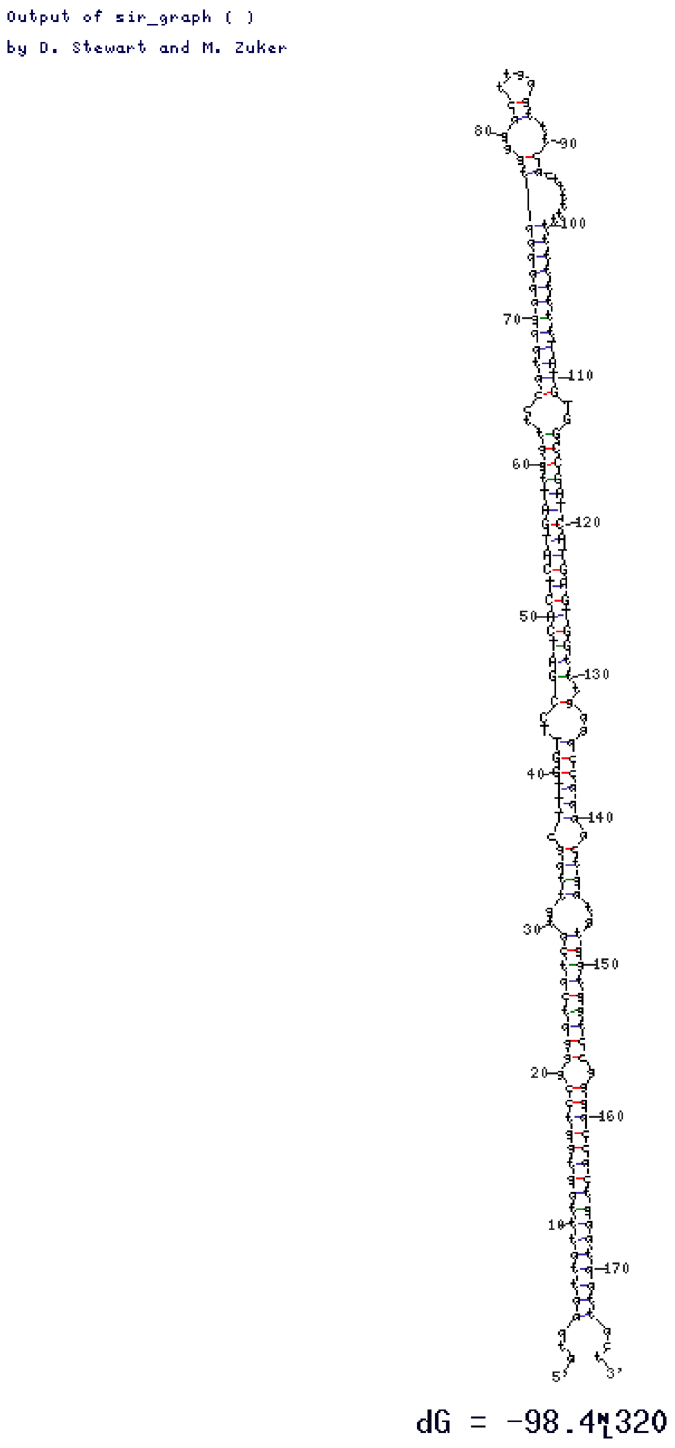

csn-smR57-5p
UUUGGUUCCGAUCACUCAUGAU

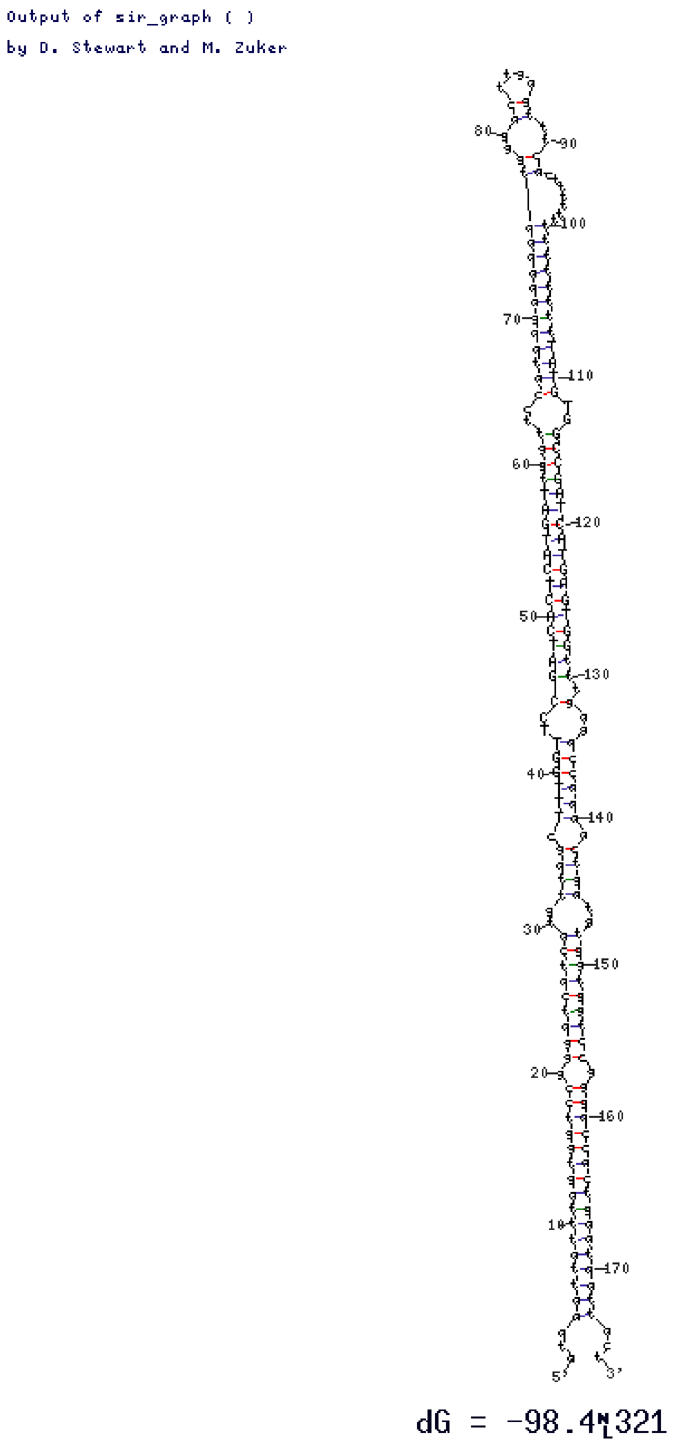

csn-smR57-3p
UAUGUGGCCGAUCAUGAGUGG

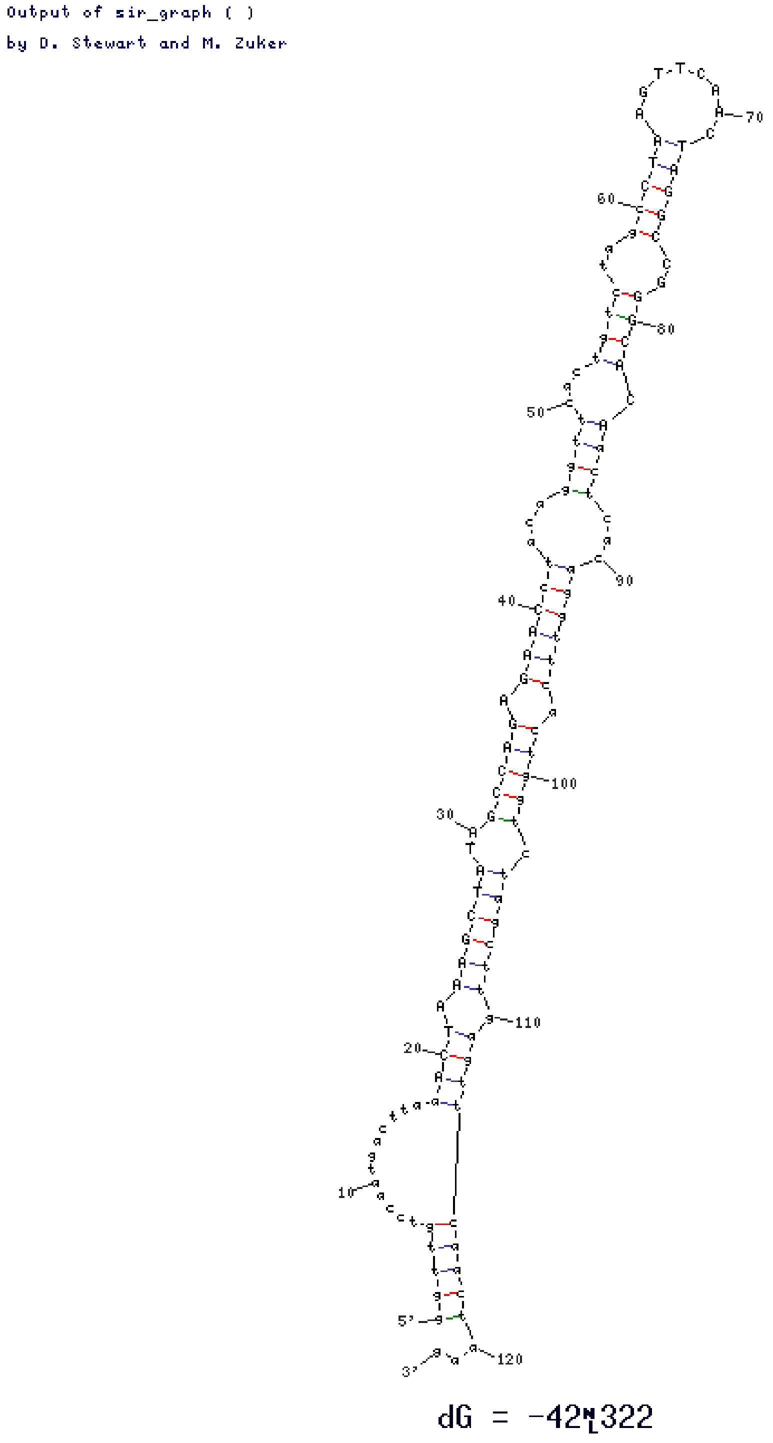

csn-smR58-5p
ACUAAAGCUAUAGCCAGAGAAC

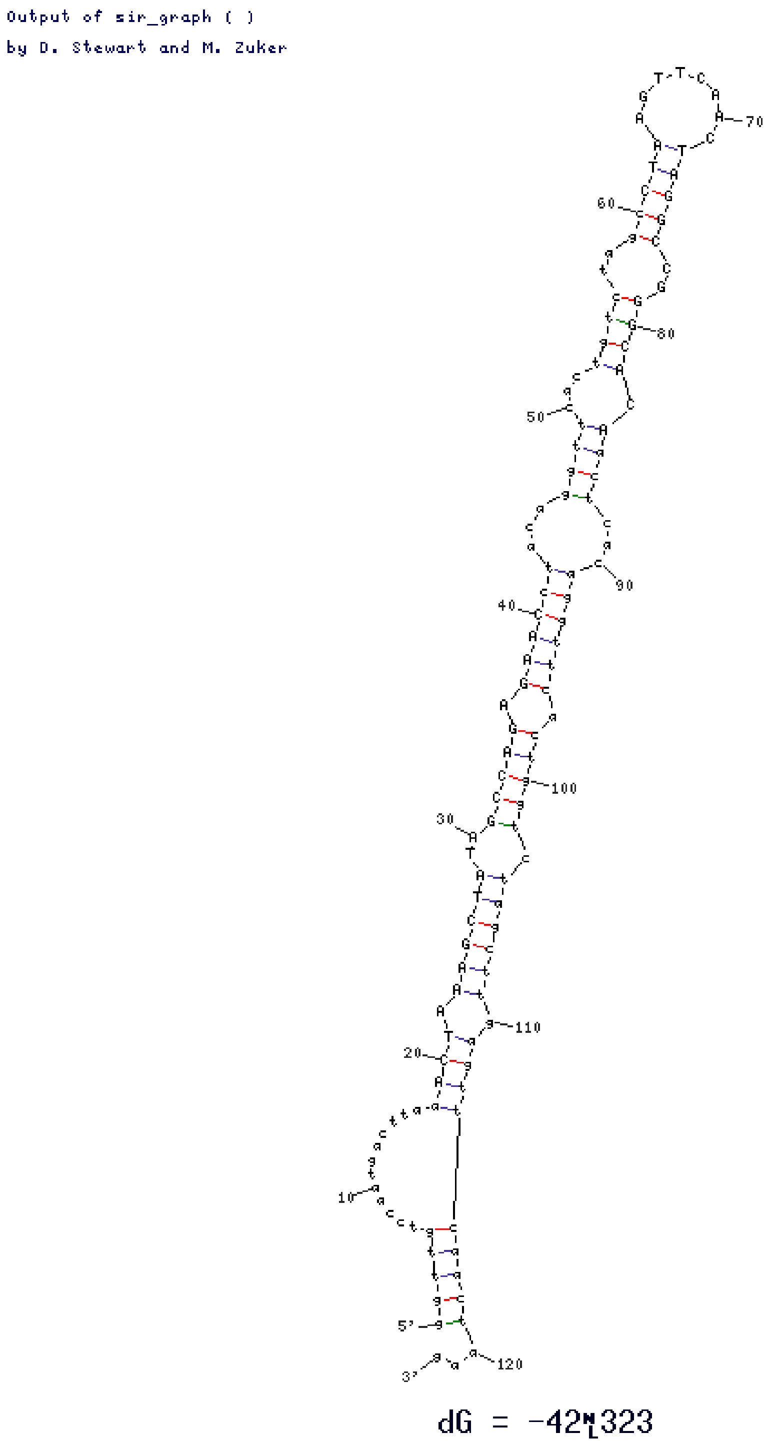

csn-smR58-3p
CUAAGUUCAACUAGGCCGGGCACA

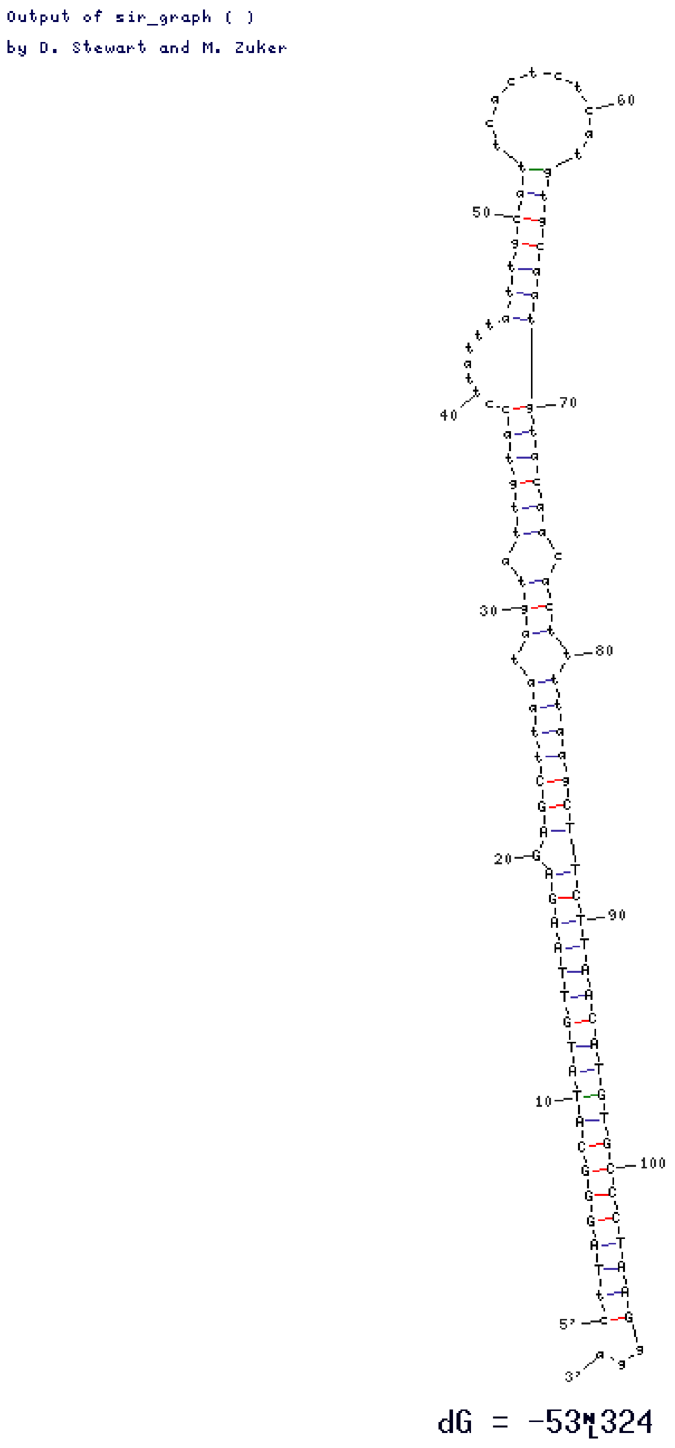

csn-smR59-5p
UAGGGCAUAUGUUAAGAGAGC

Output of sir_graph ( )
by D. Stewart and M. Zuker

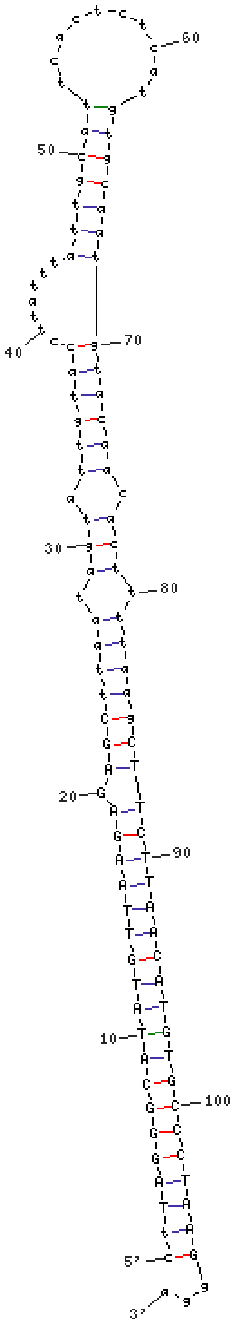

dG = -53[illegible]325

csn-smR59-3p
CUUCUUAACAUGUGCCCUAAG

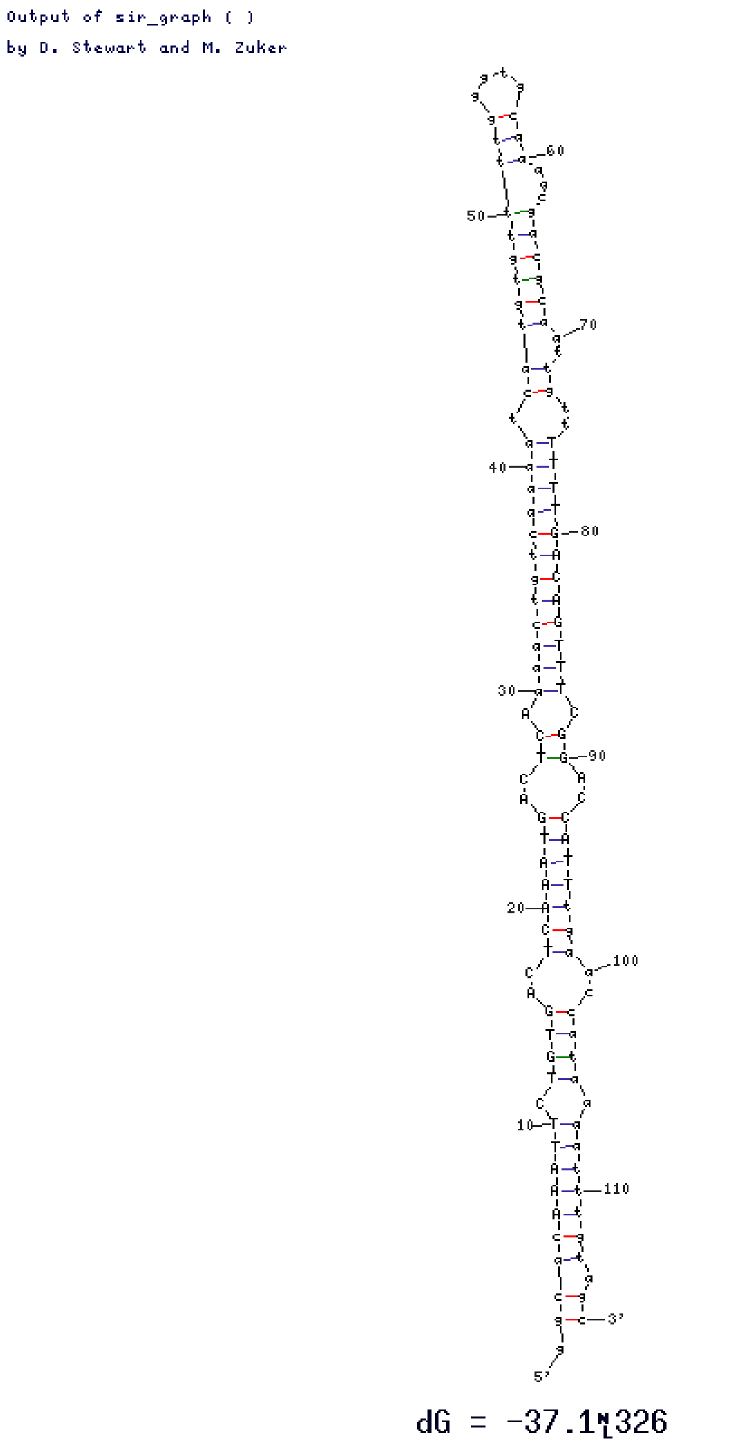

csn-smR60-5p
AAAUUCUGUGACUCAAAUGACUCA

Output of sir_graph ( )
by D. Stewart and M. Zuker

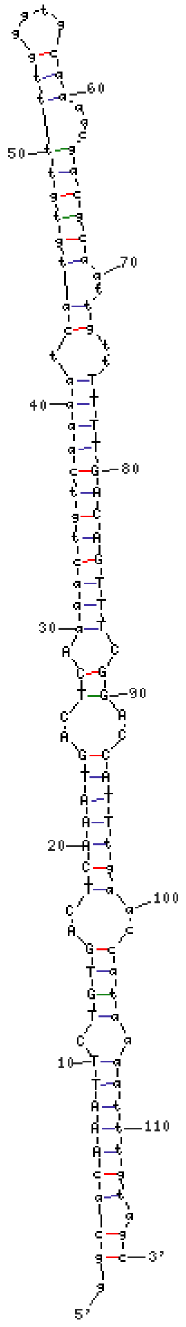

dG = -37.1[illegible]327

csn-smR60-3p
UUUUGACAUUUUCGGACCAUU

Output of sir_graph ( )
by D. Stewart and M. Zuker

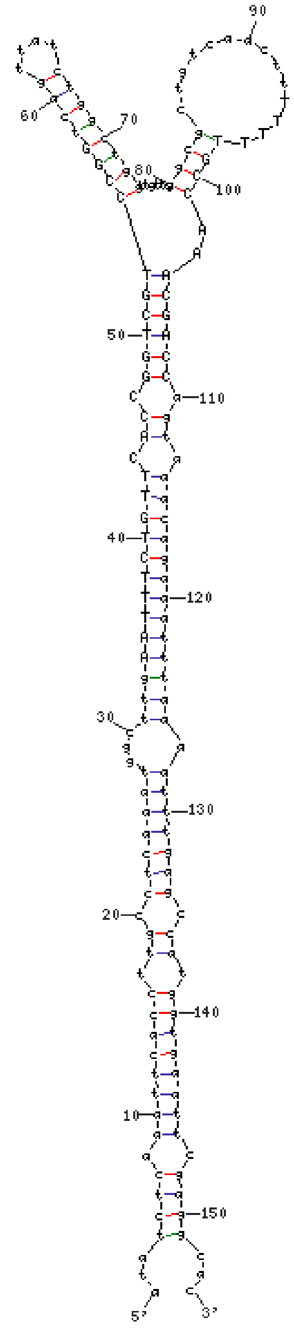

dG = -63[illegible]328

csn-smR61-5p
AAUUUCUGUUCACCGGUCGUCCGG

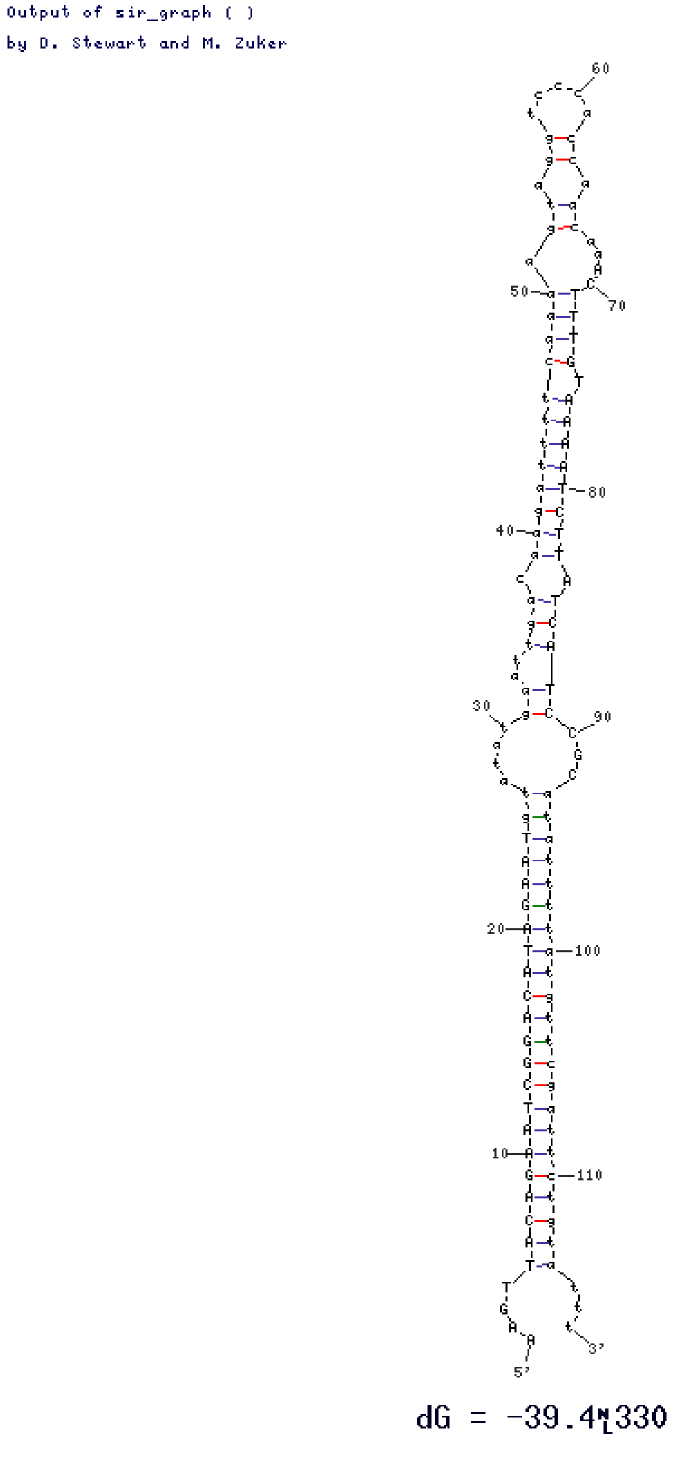

csn-smR62-5p
AAGUUACAGAAUCGGACAUAGAAU

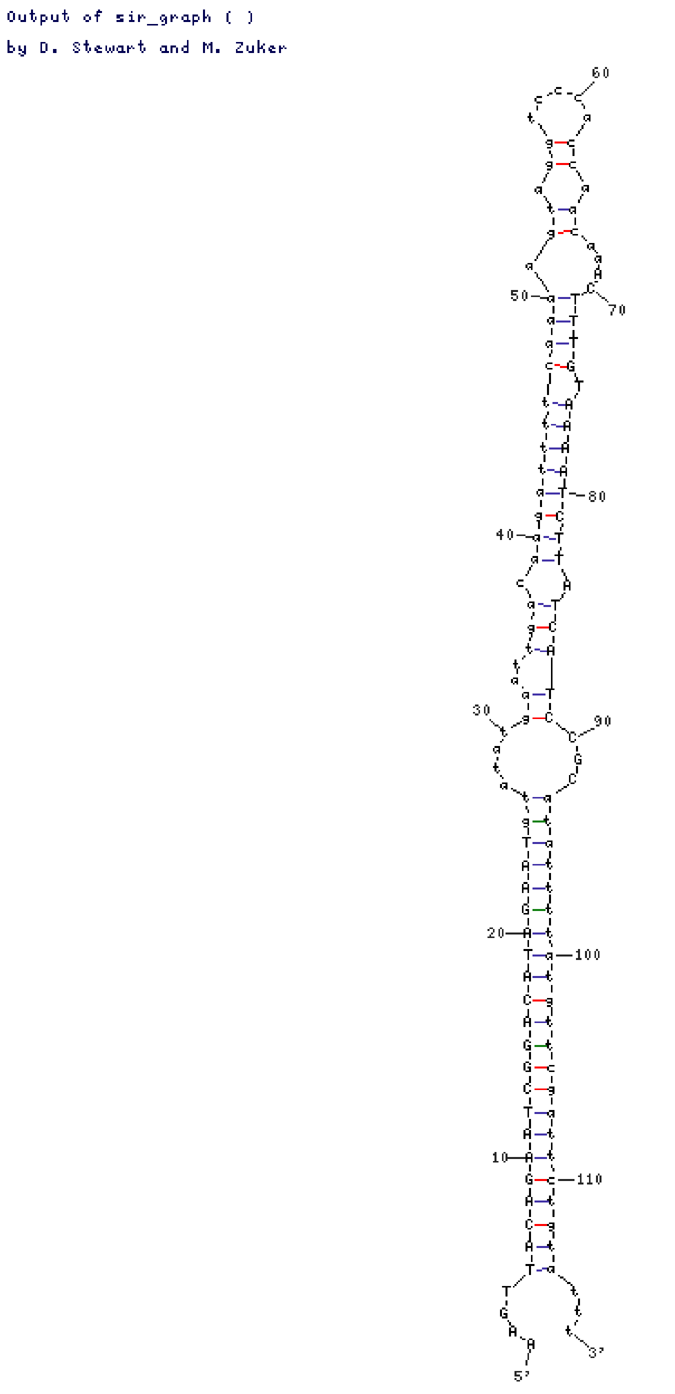

dG = -39.4[illegible]331

csn-smR62-3p
ACUUUGUAAAAUCUUAUCAUCCGC

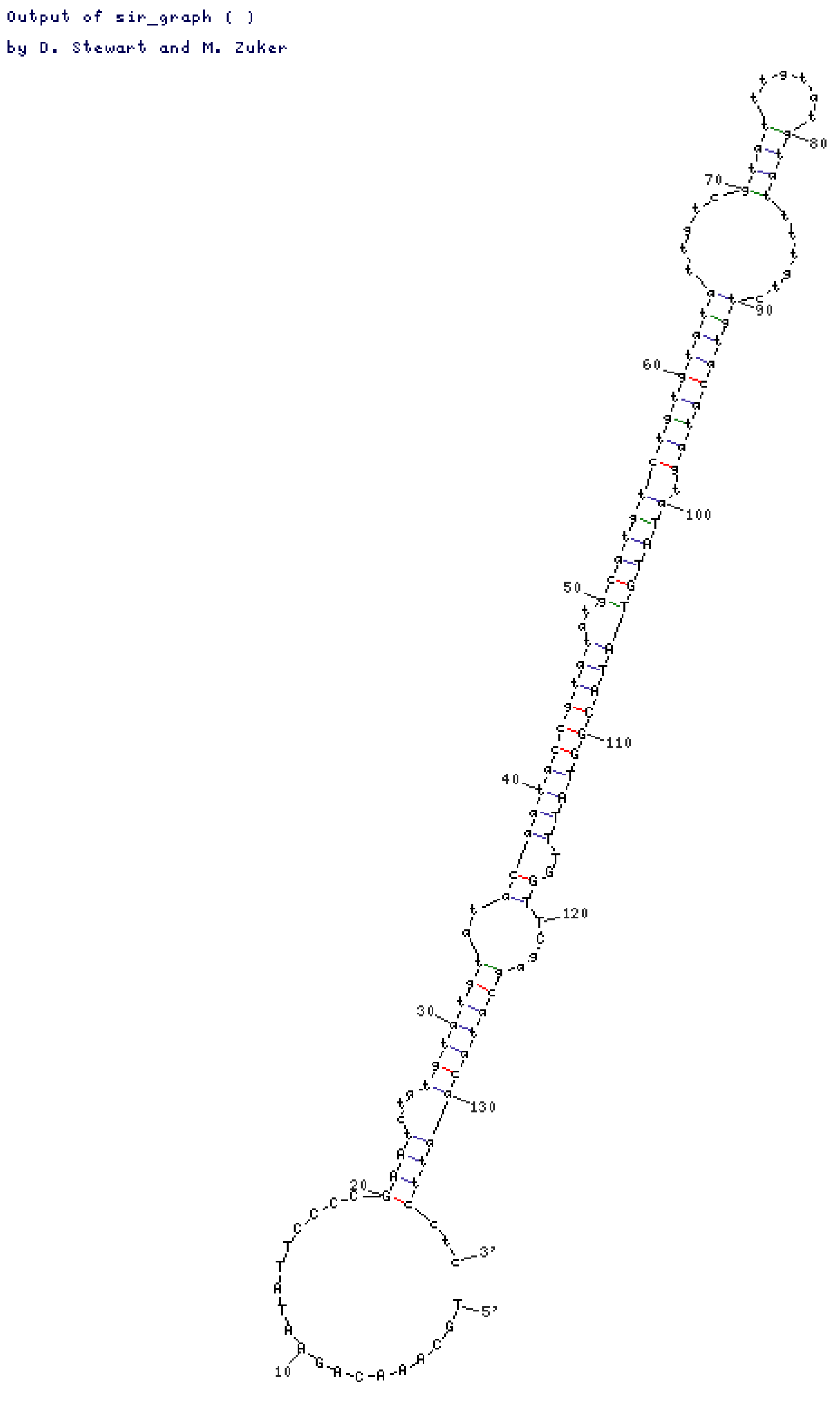

csn-smR63-5p
UGCAAACAGAAUAUUCCCCGAA

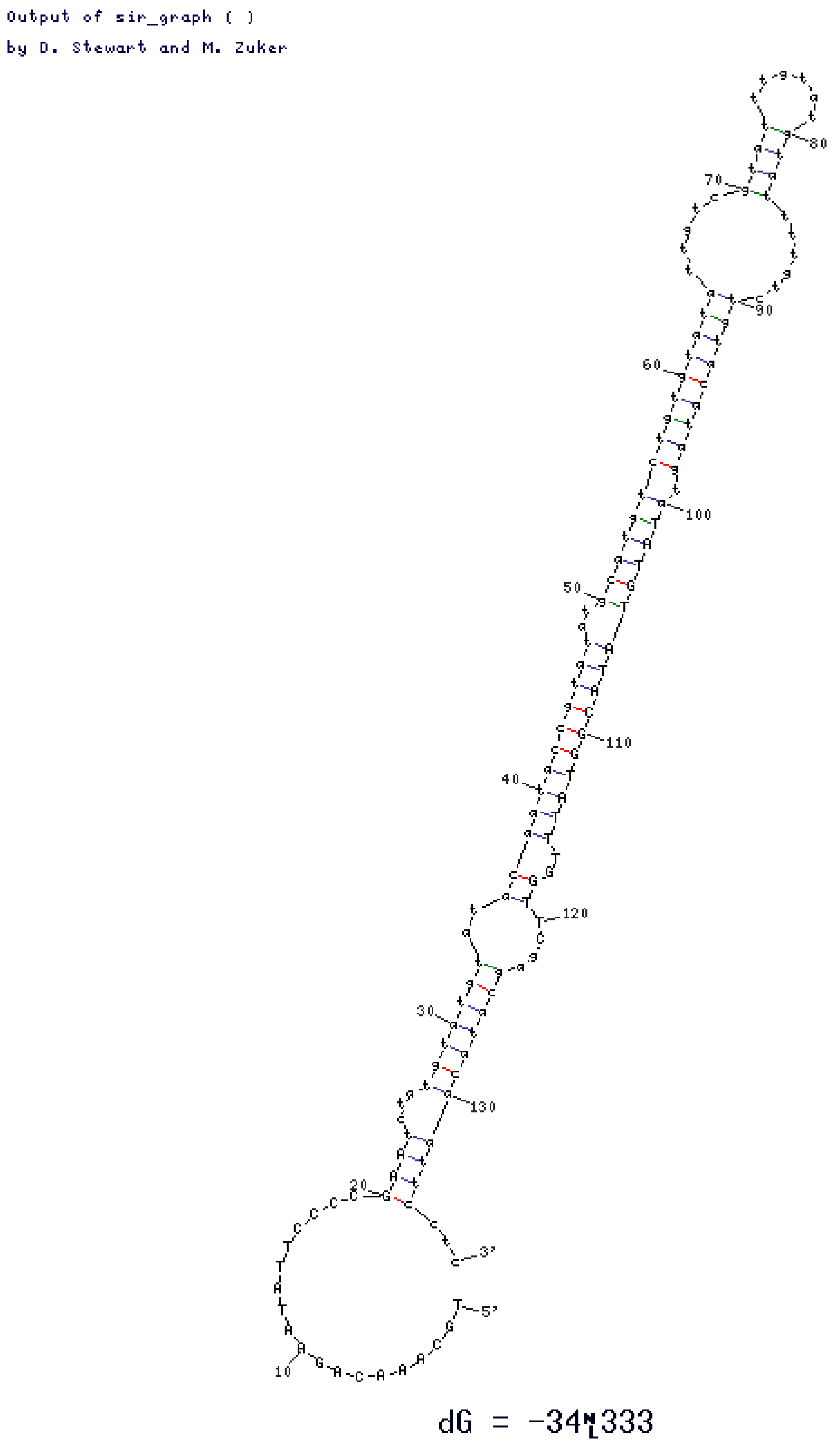

csn-smR63-3p
UAUGUAUACGGUAUUUGGUUC

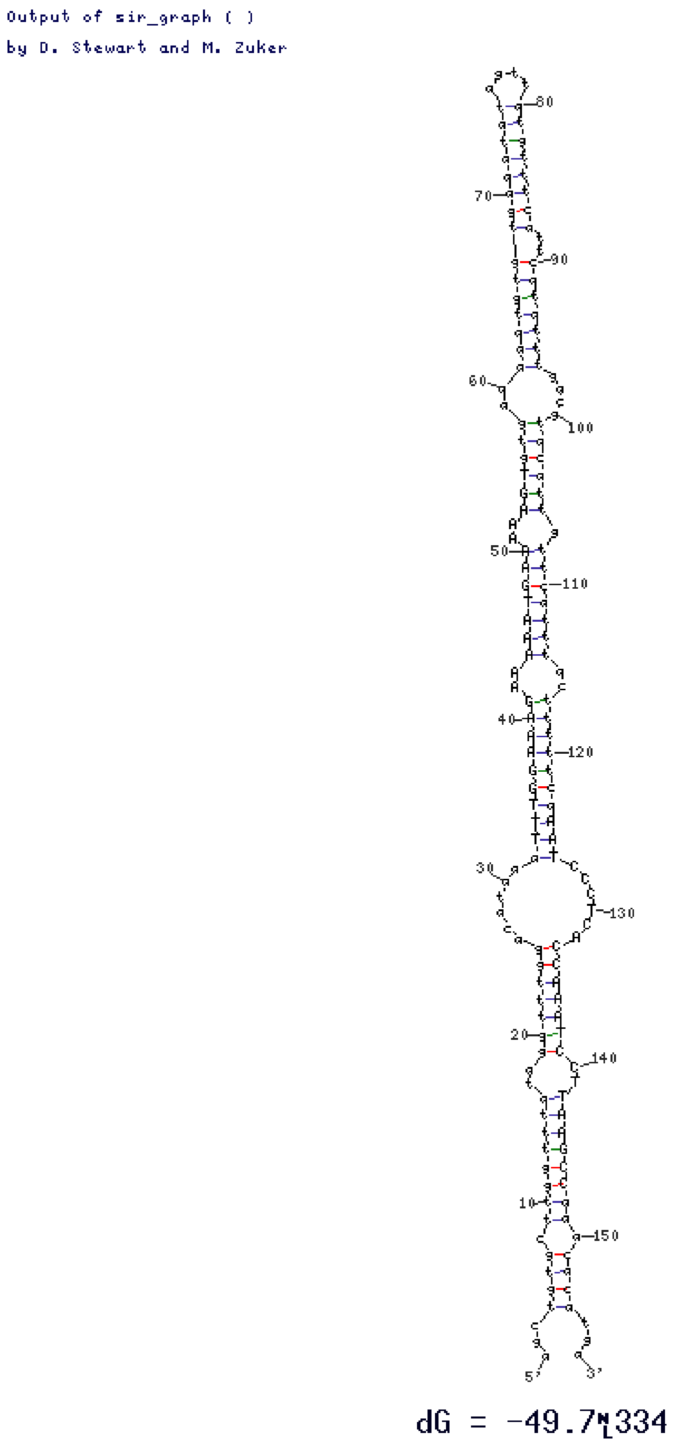

csn-smR64-5p
UUUGGAAAGAUAAAUGAAAAAGU

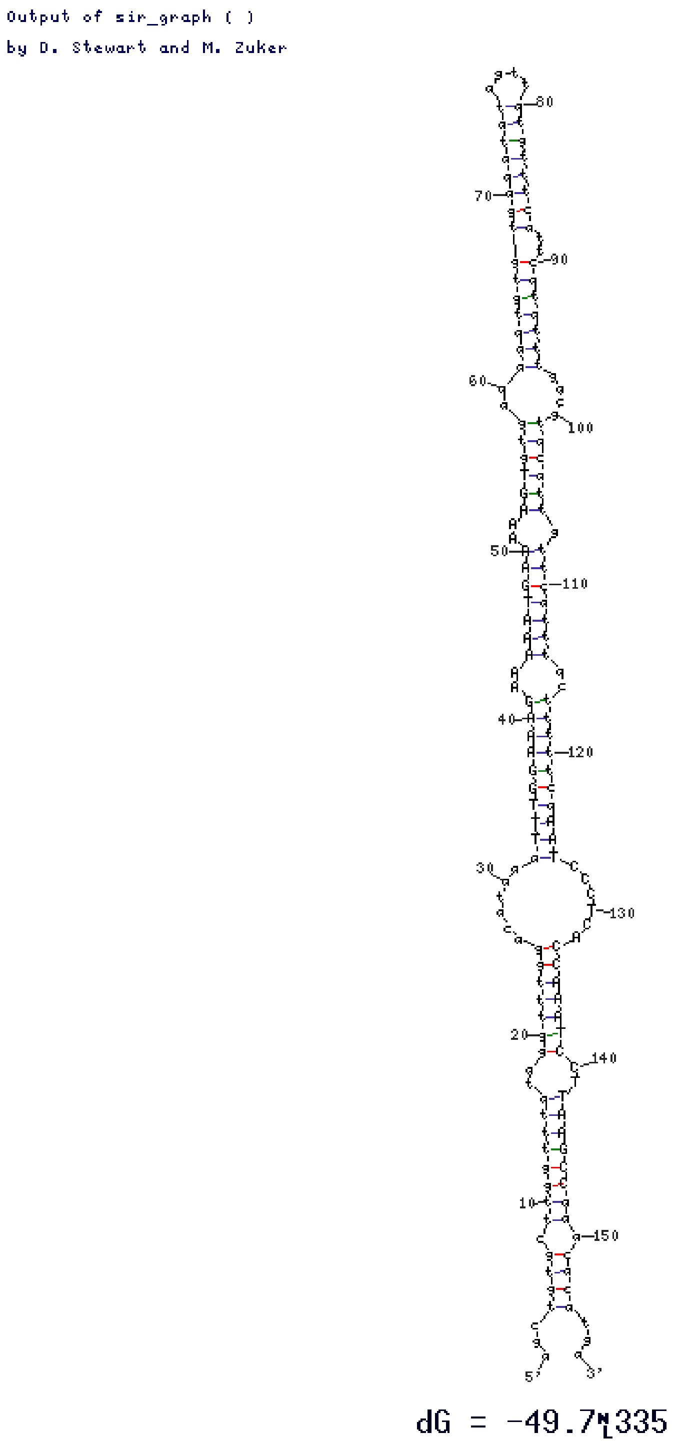

csn-smR64-3p

AAUCCCUCACCAAAUCCUUAAGCC

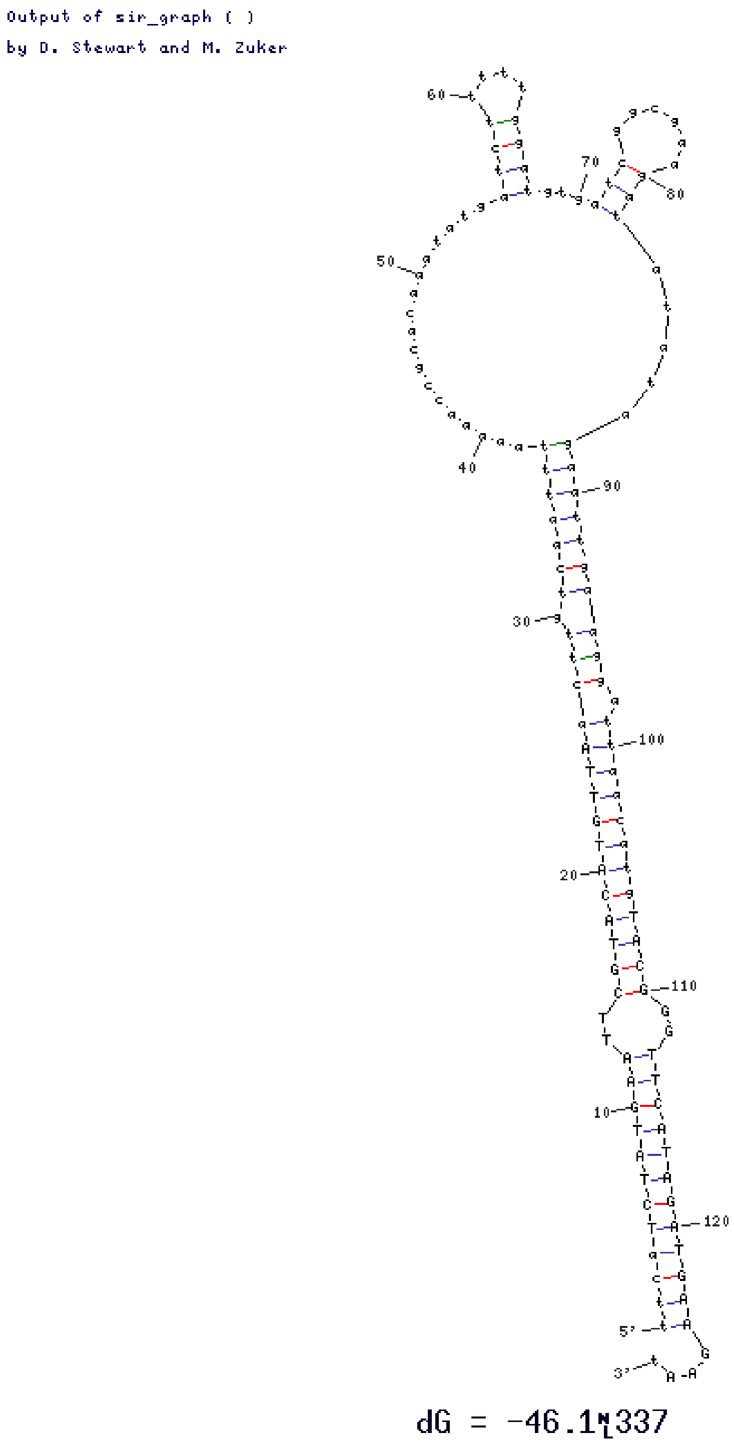

csn-smR65
UACGGGUUCAUAGAUGAAGAA

Output of sir_graph ( )
by D. Stewart and M. Zuker

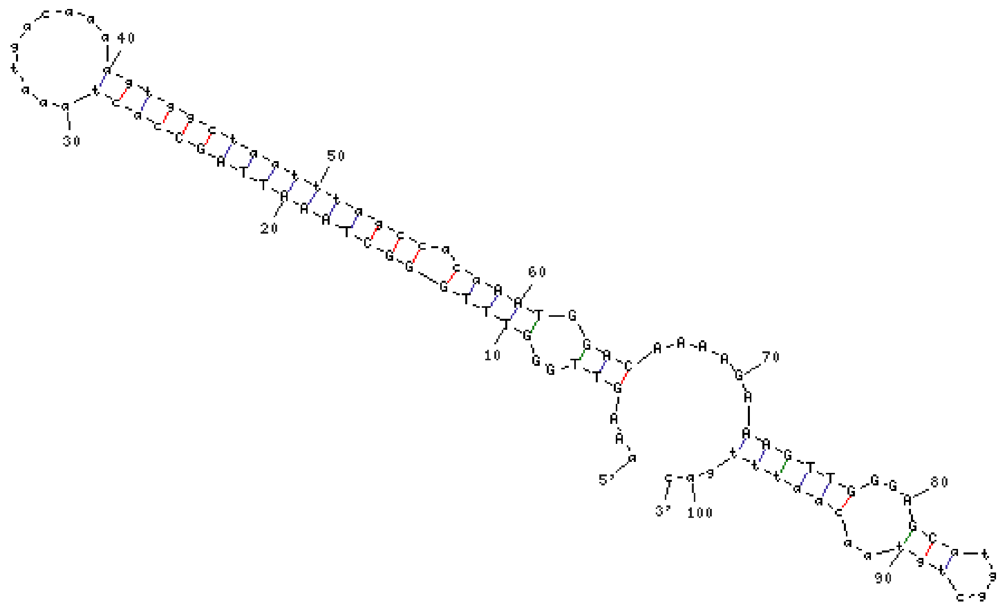

dG = -34.6[illegible]338

csn-smR66-5p
AAGUUGGGUUUGGGCUAAAUUAGC

Output of sir_graph ( )
by D. Stewart and M. Zuker

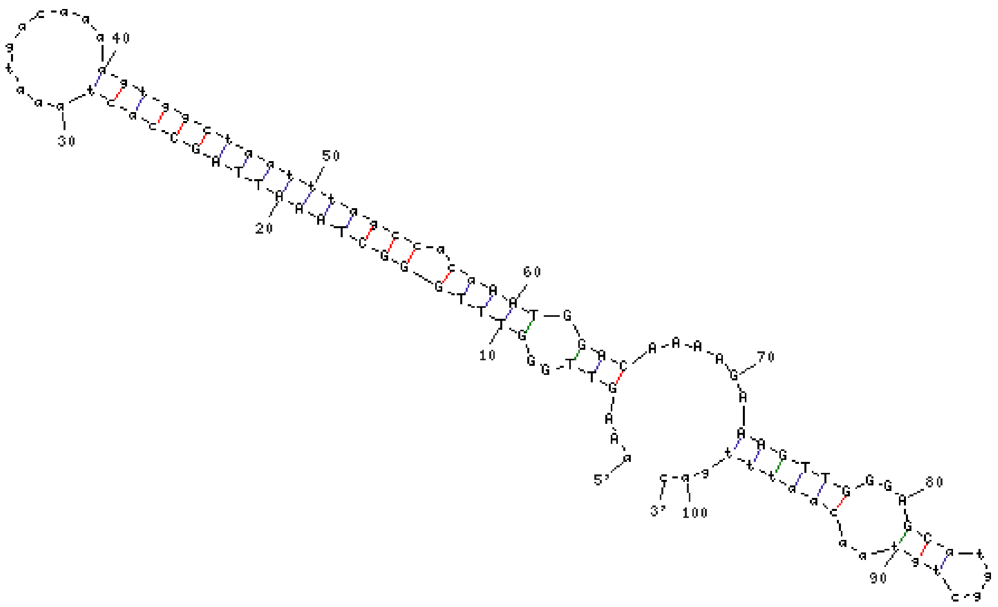

dG = -34.6[illegible]339

csn-smR66-3p
AACGGACAAAAGAAAGUUGGGAGC

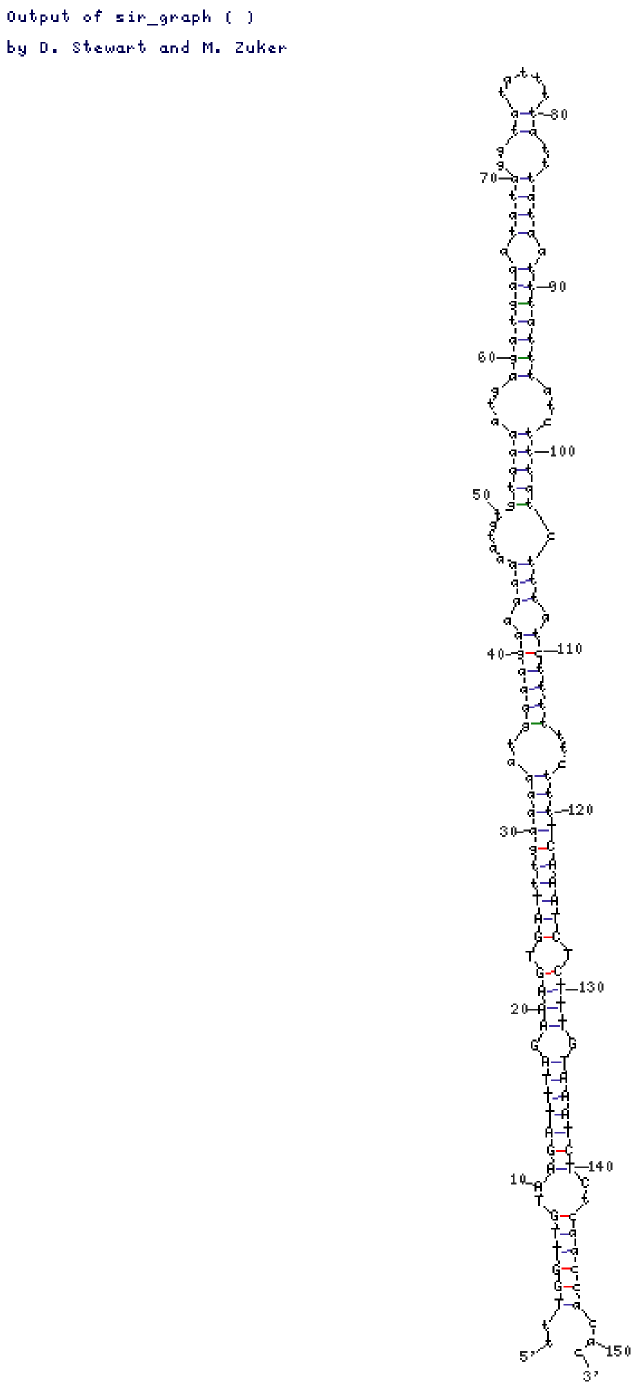

dG = -43.5 [illegible] 341

csn-smR67
UCAAAUCUCUUUGUAAAUCUC

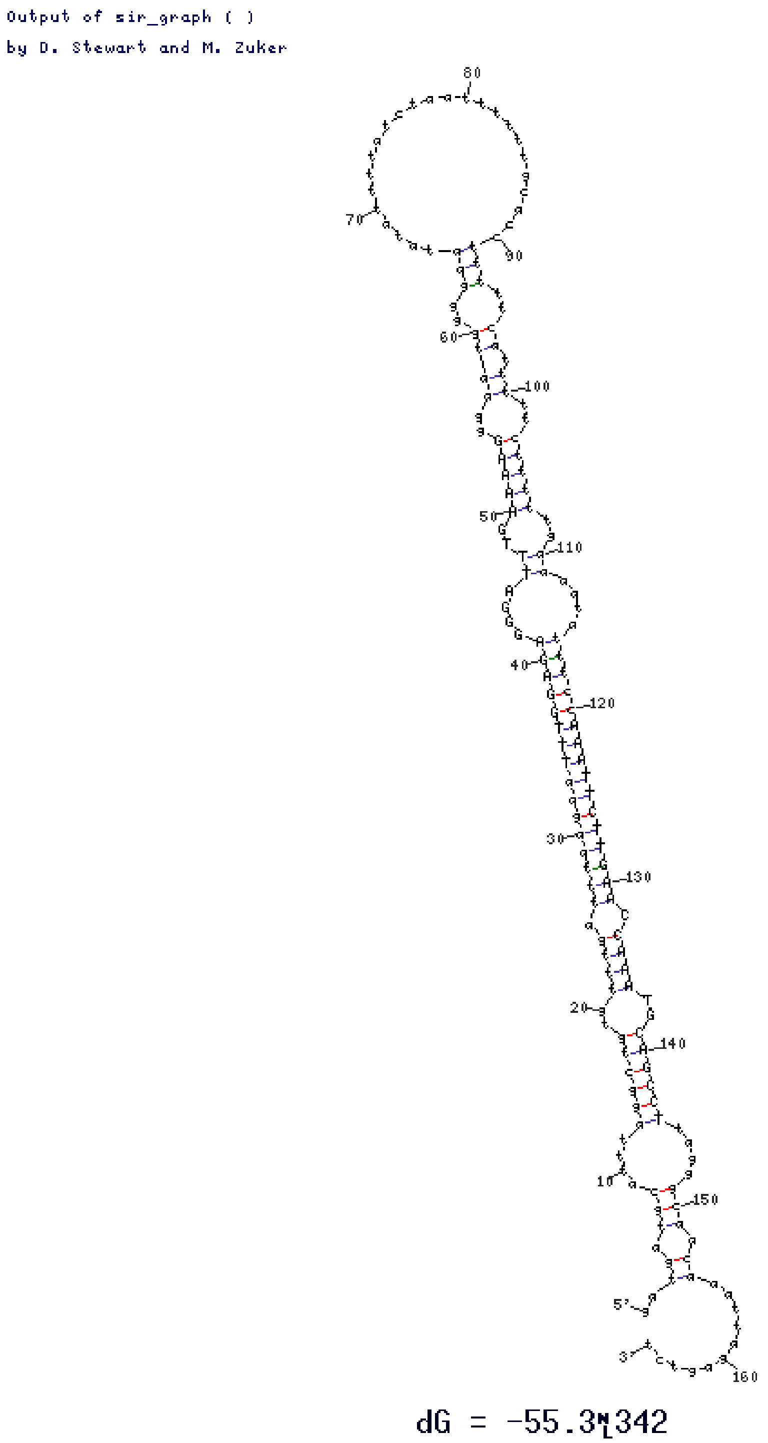

csn-smR68-5p
UUUGGAGAGGGAUUUGGAAAG

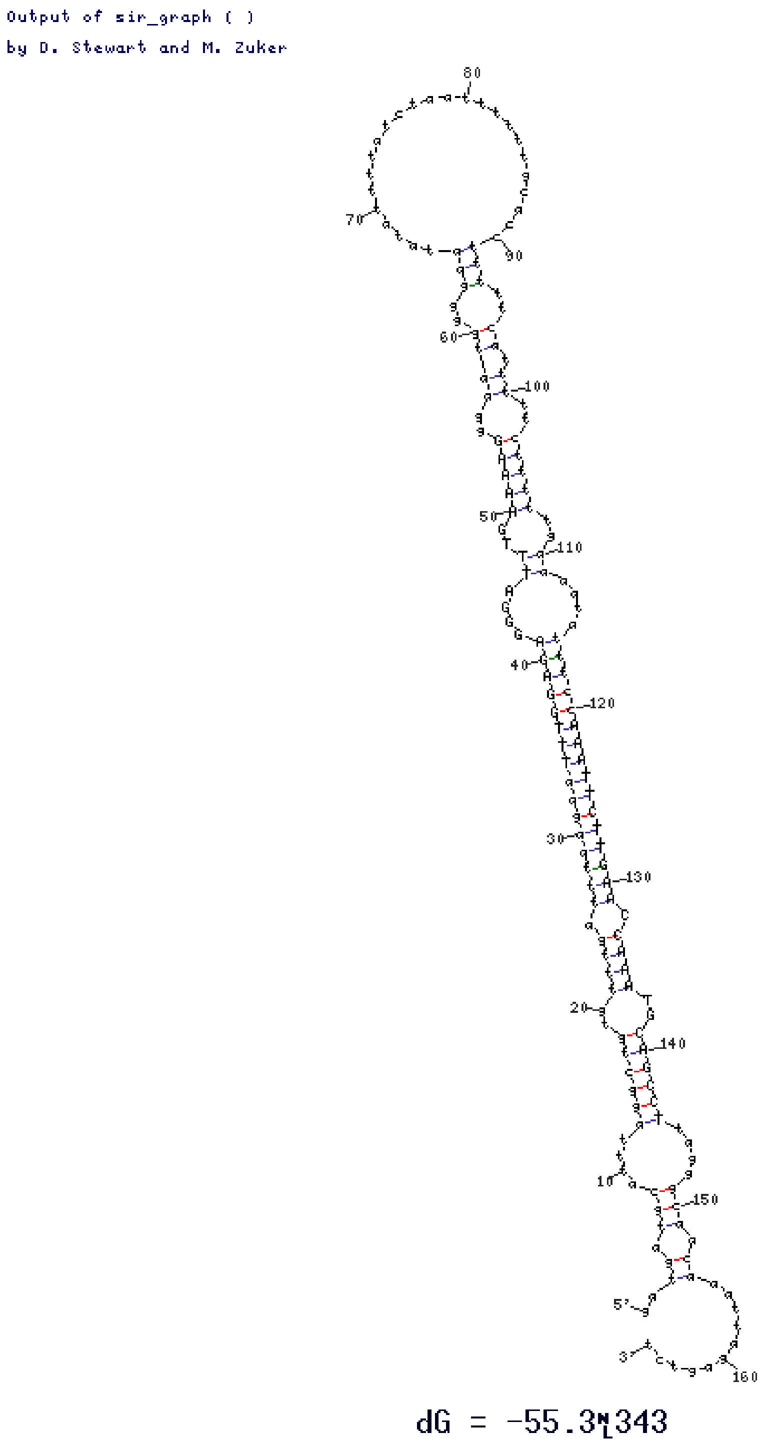

csn-smR68-3p
AAAUUCUUGAACCAAAUGCAGCCU

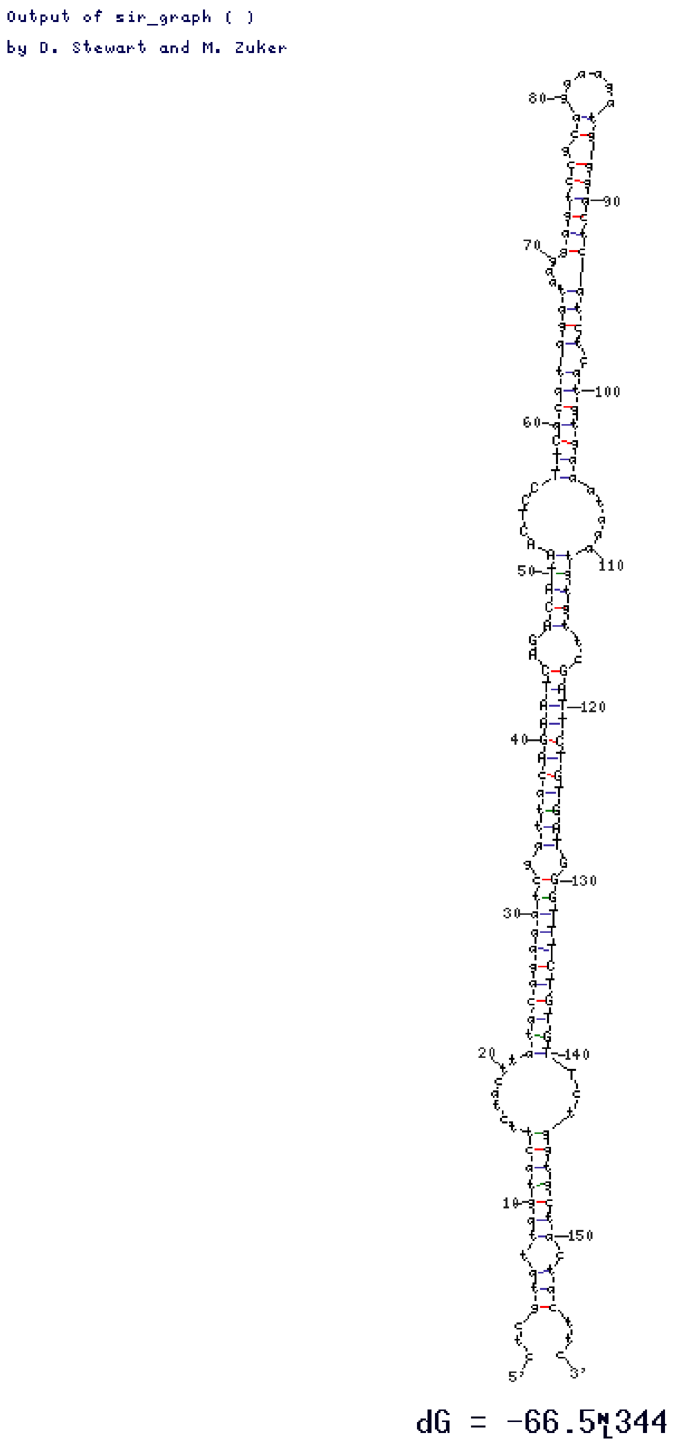

csn-smR69-5p
AGAAUCAGACAUAACUCCUUU

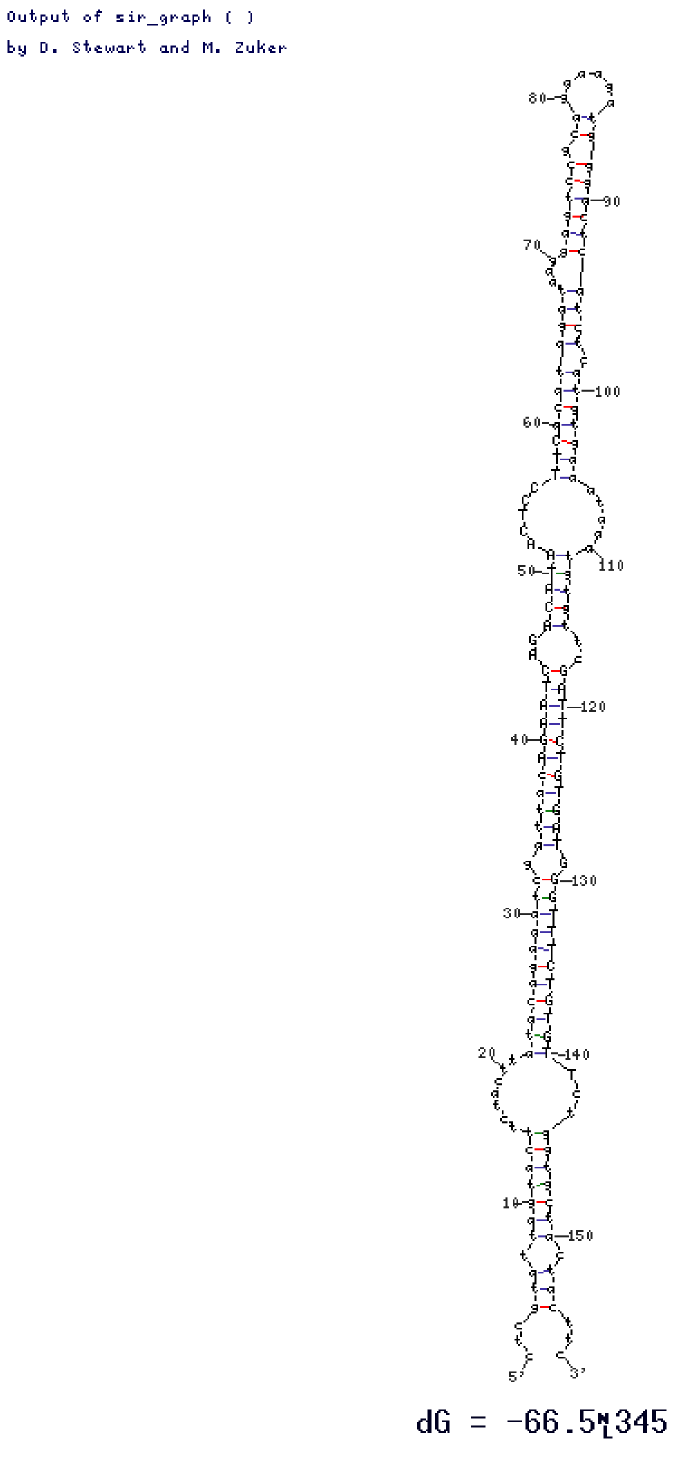

csn-smR69-3p
GAUUCUGUGAUCGGUUUCUGUGUU

Output of sir_graph ( )
by D. Stewart and M. Zuker

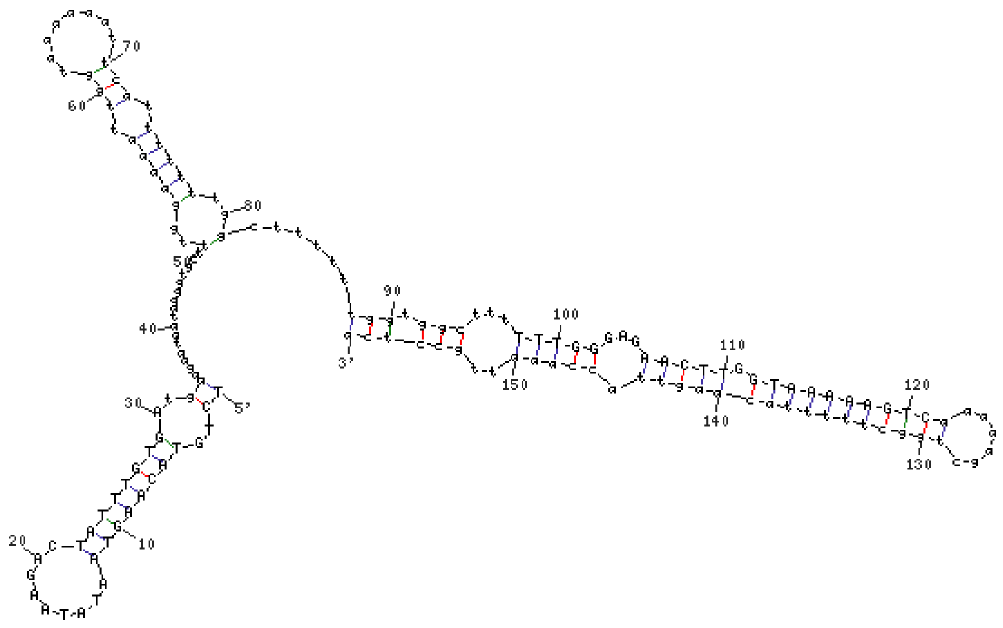

dG = -34.8[illegible]347

csn-smR70-5p
UUUGGGAGAACUUGGUAAAAAGCC

Output of sir_graph ( )
by D. Stewart and M. Zuker

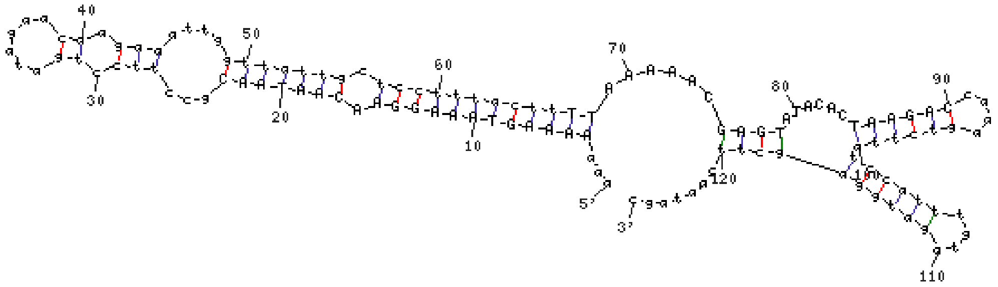

dG = -32.6[illegible]348

csn-smR70-3p
AAAAGUAAAGGAGCAAUAAC

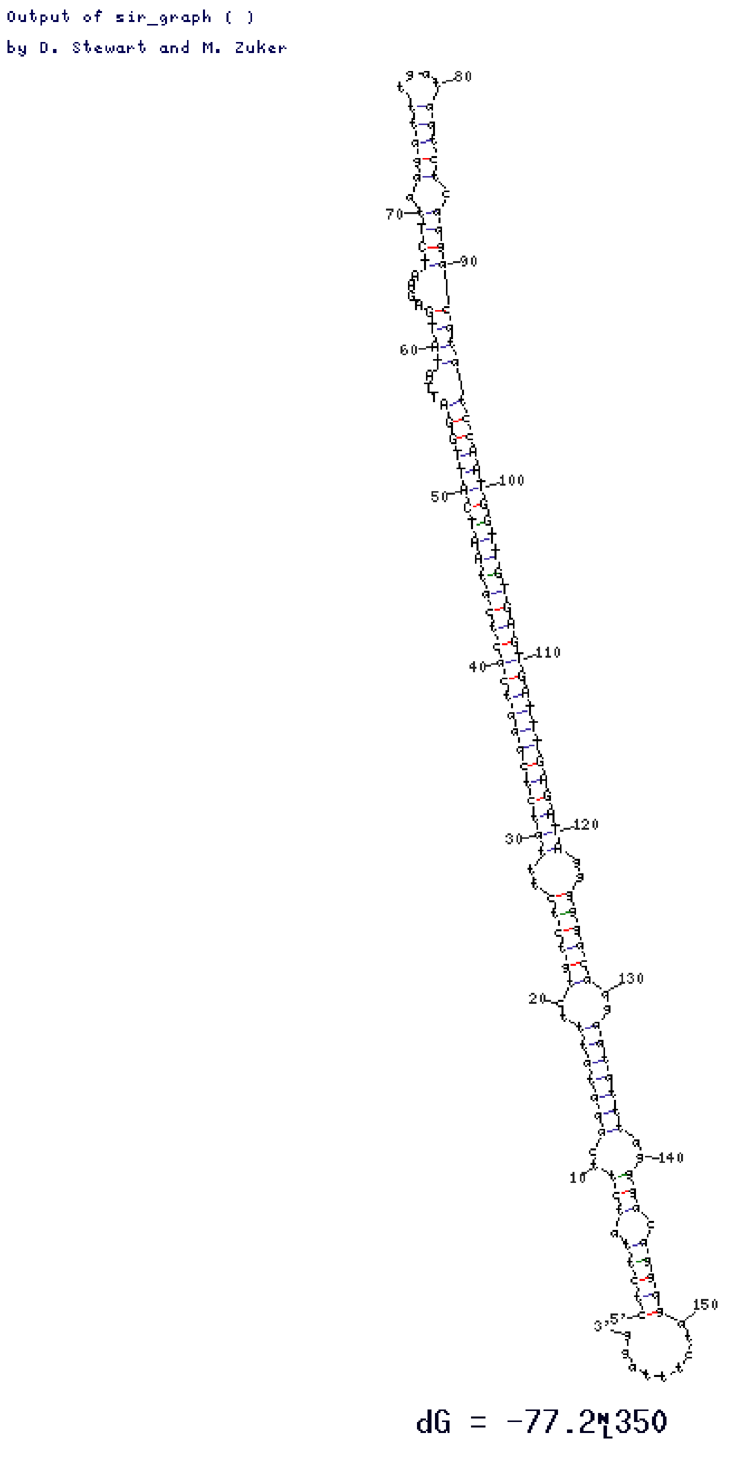

csn-smR71-5p
AAUCAUUGGAUUAUAUGAGAAUCU

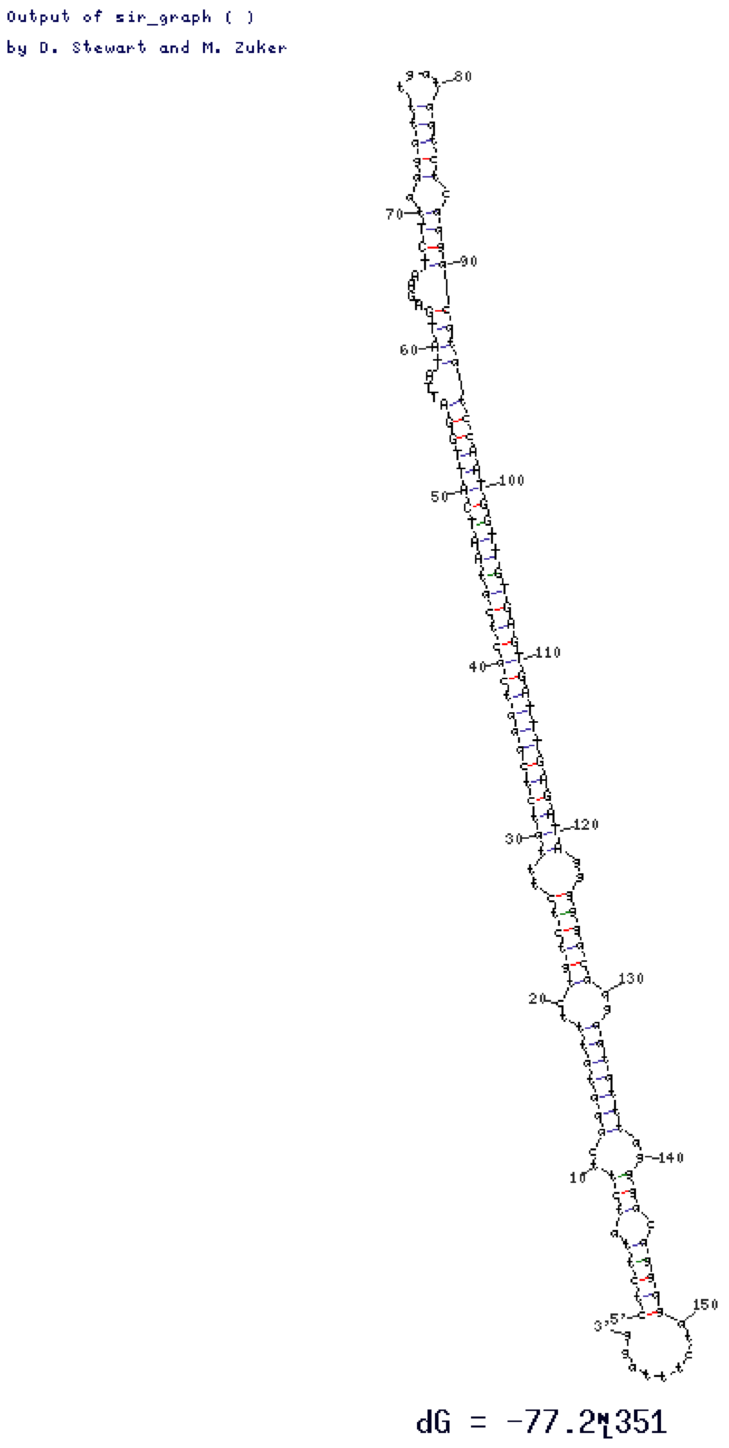

csn-smR71-3p
AAUGGUUGUGAGUGAUUUGAGACA

Output of sir_graph ( )
by D. Stewart and M. Zuker

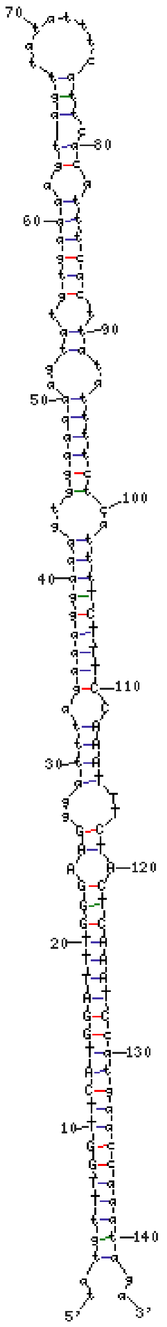

dG = -59.7[illegible]352

csn-smR72-5p
UUGGUUCAUGAAUUUGGGAAG

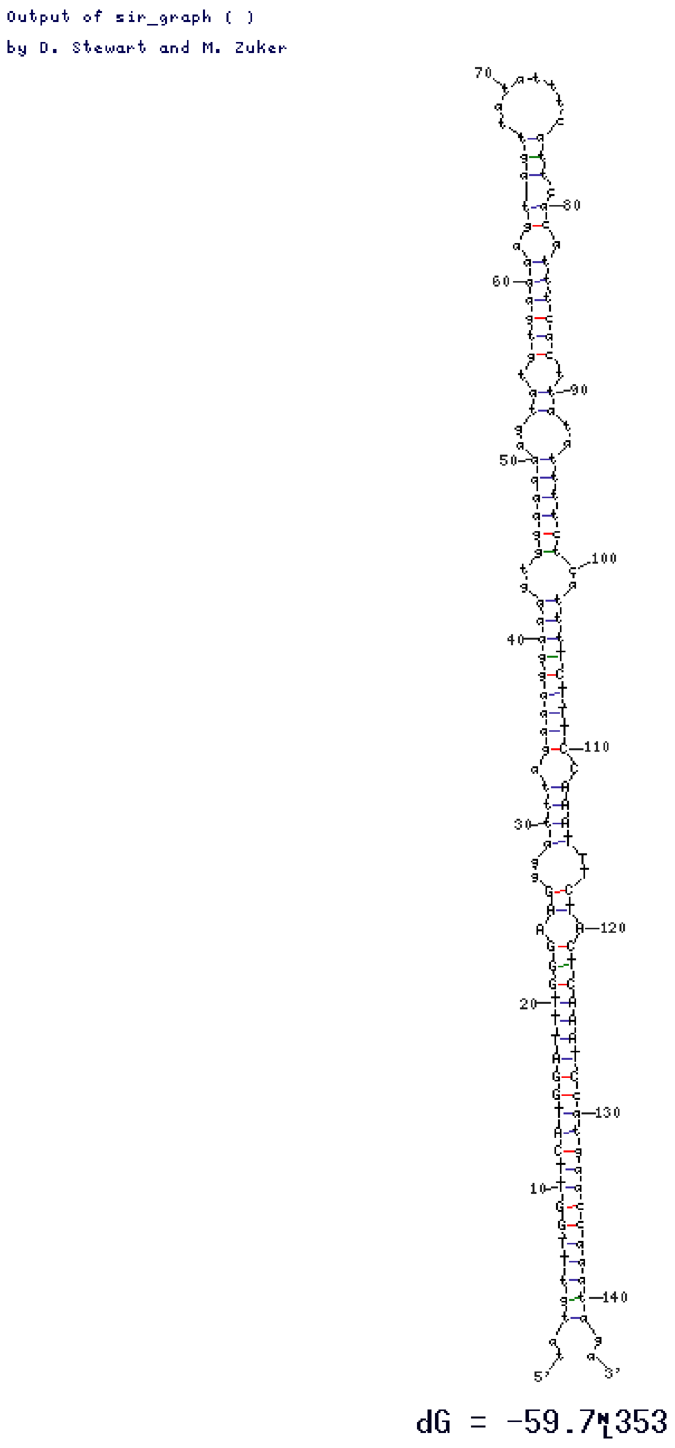

csn-smR72-3p
UCUUUCCAAAUUUCUCCUCAAAUC

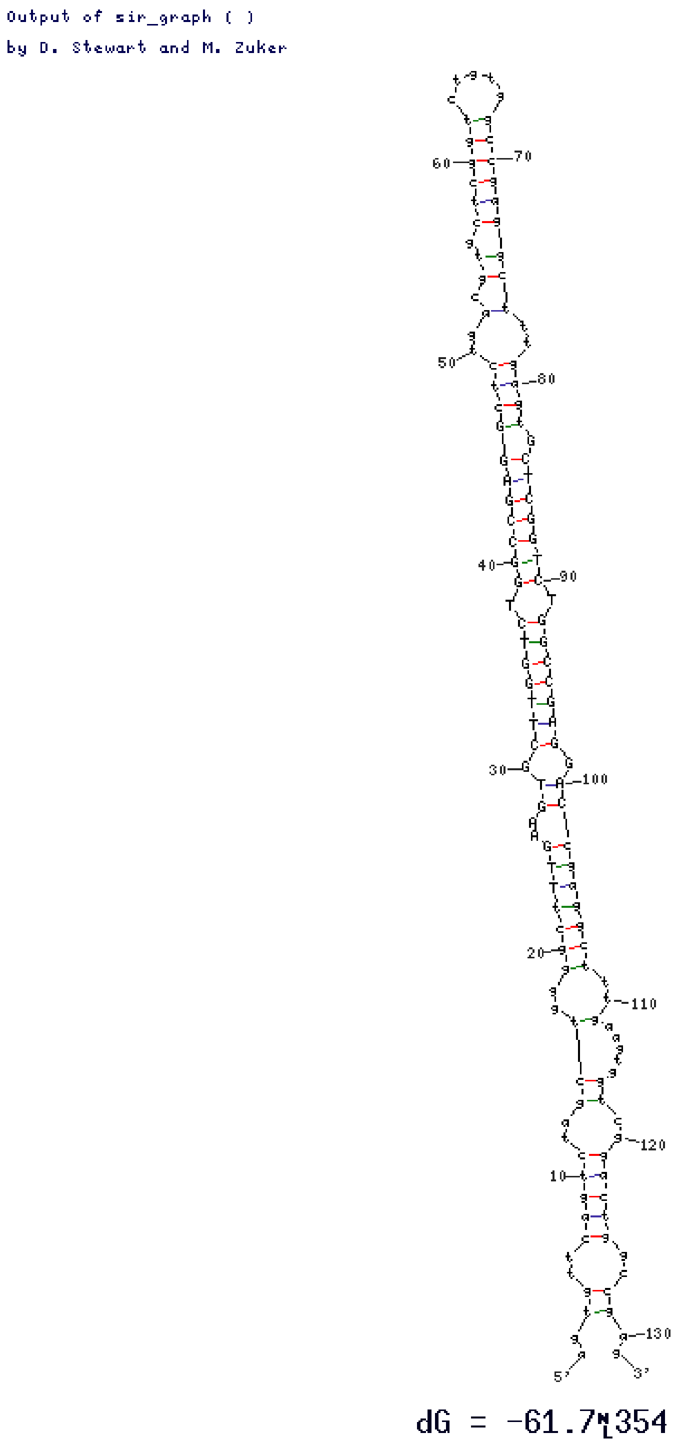

csn-smR73-5p
UUGAAGUGCUUGGUCUGGCCGAGG

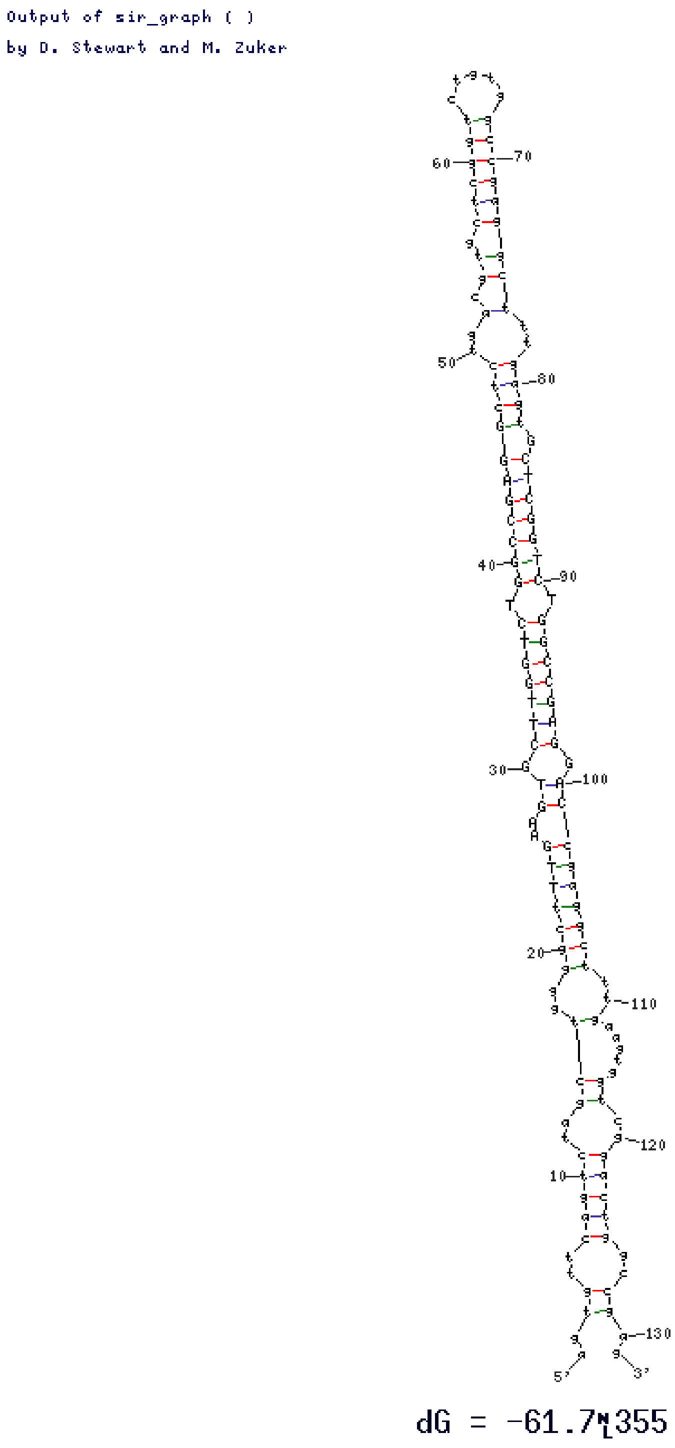

csn-smR73-3p
GCUCGGUCUGGUCGAGGAC

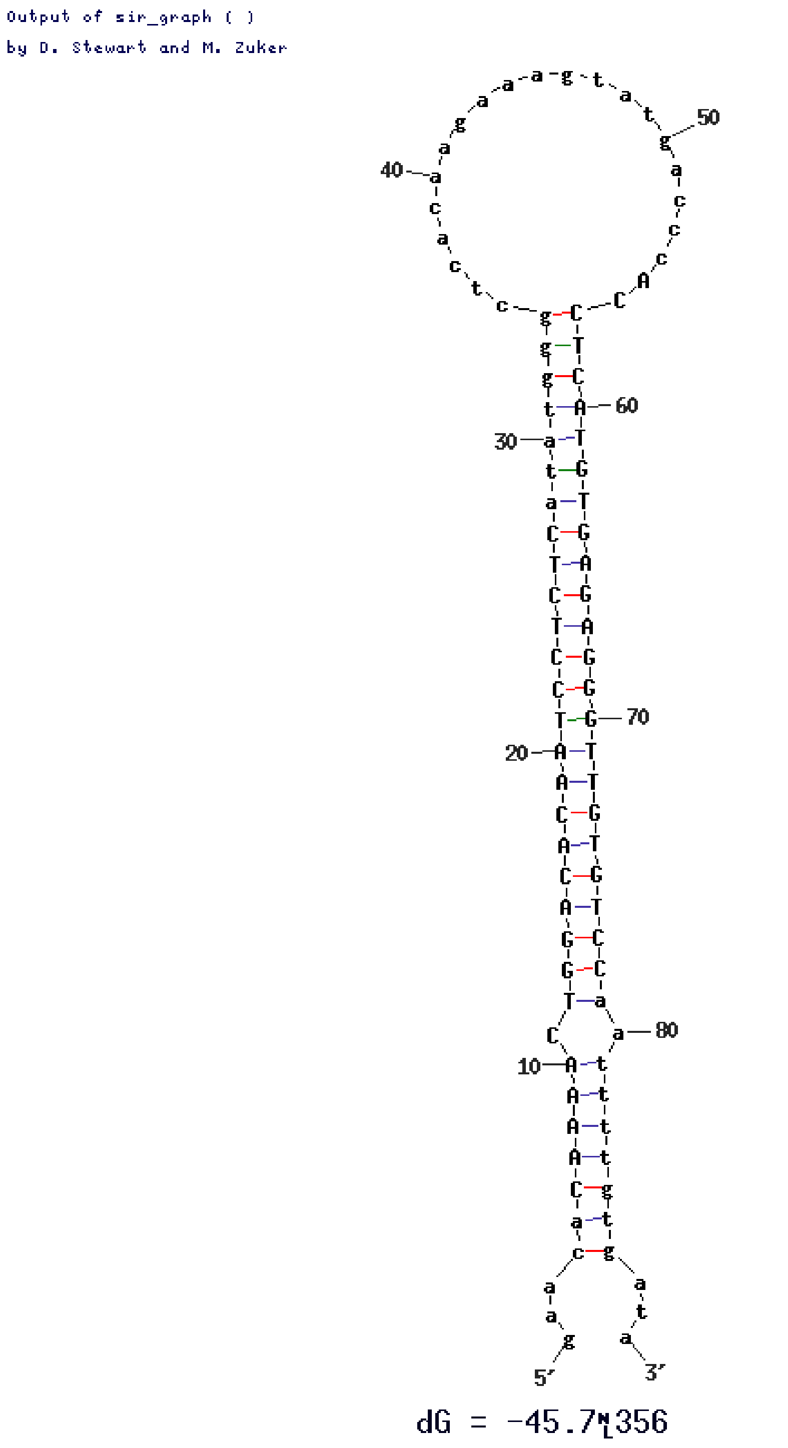

csn-smR74-5p
CAAAACUGGACACAACCCUCUC

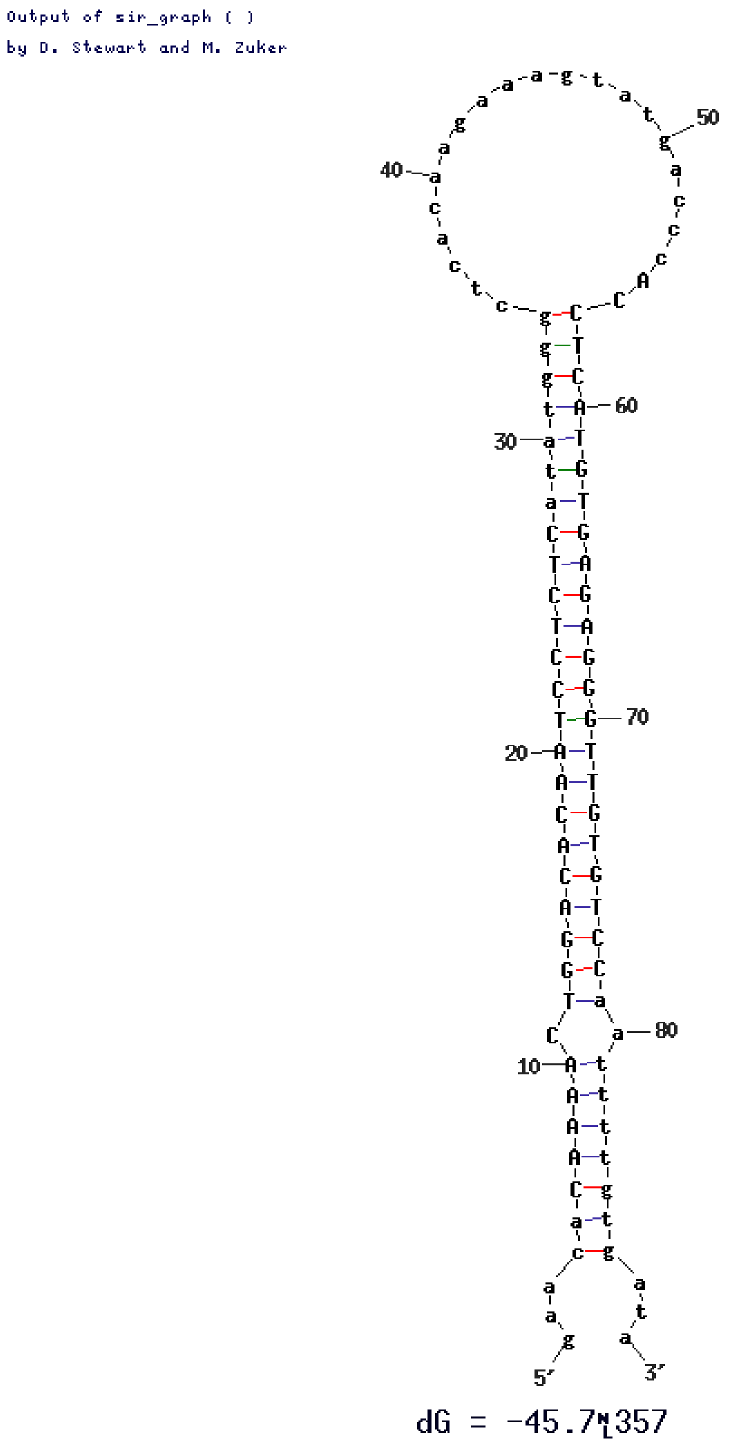

csn-smR74-3p
ACCUCAUGUGAGAGGAUUGUGUCC

Output of sir_graph ( )
by D. Stewart and M. Zuker

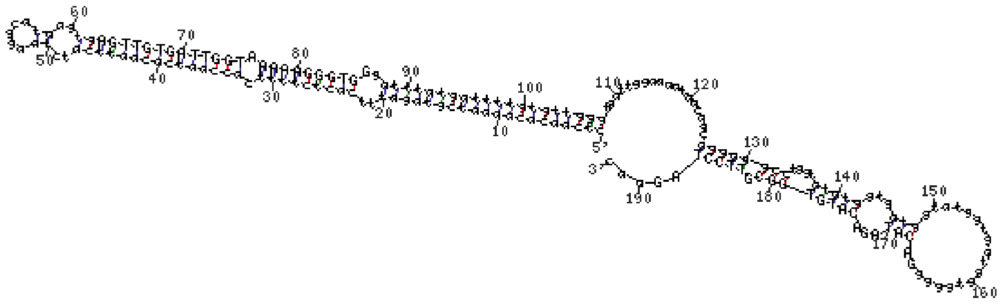

dG = -78.3[illegible]360

csn-smR75
AGUUGUGAUUGGUAAAAGGGUGG

Output of sir_graph ( )
by D. Stewart and M. Zuker

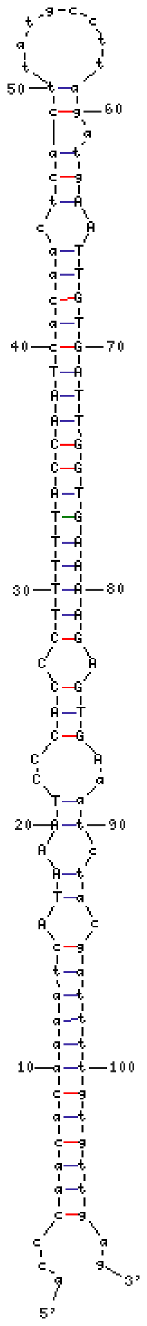

dG = −46.4[illegible]362

csn-smR76-5p
AUAAAUCCCACCCUUUUUACCAAU

Output of sir_graph ( )
by D. Stewart and M. Zuker

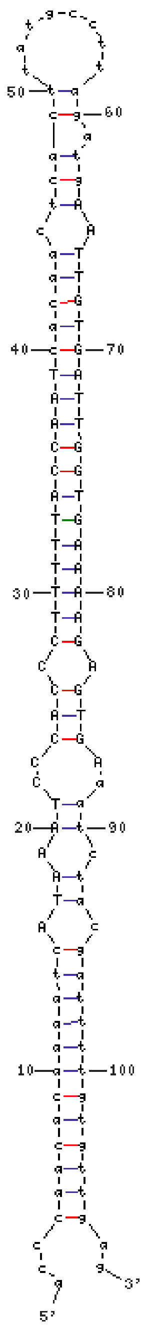

dG = −46.4[illegible]363

csn-smR76-3p
AAUUGUGAUUGGUGAAAAGAGUGA

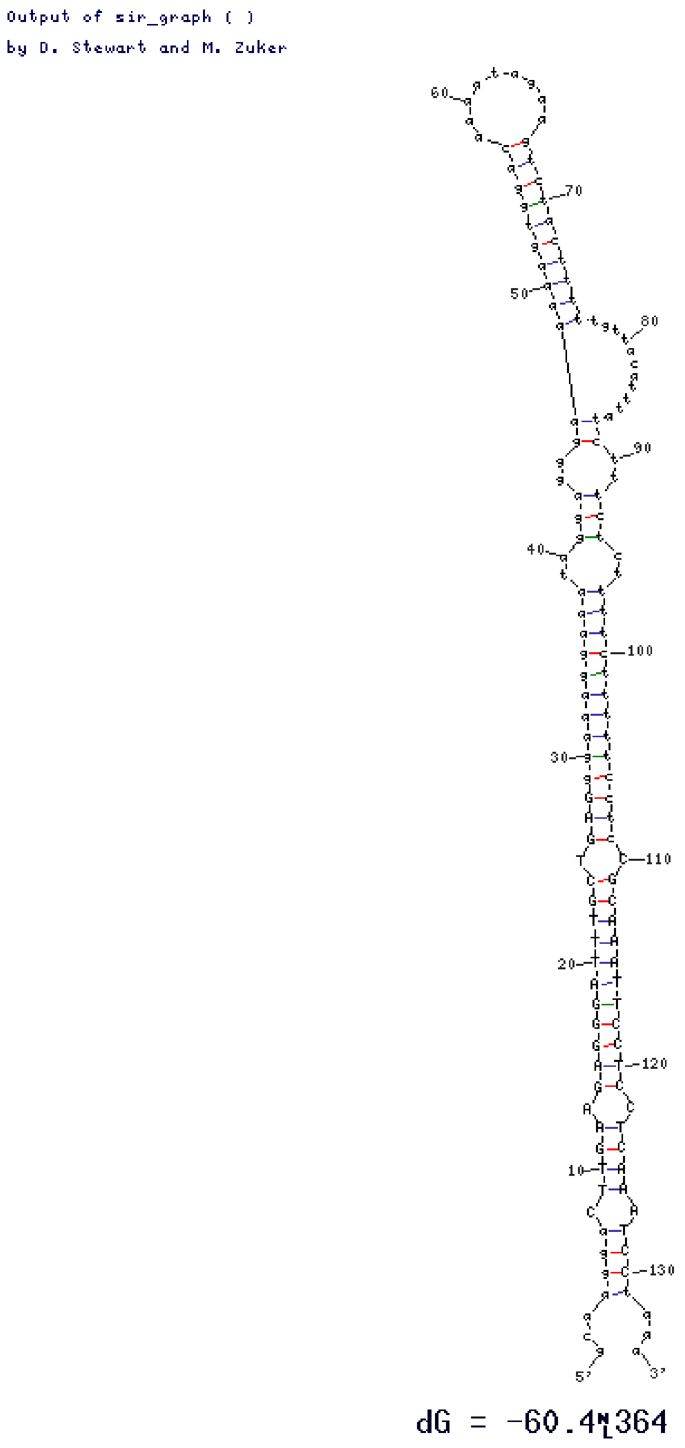

csn-smR77-5p
CUUGAUGAGGGAUUUGCUGAG

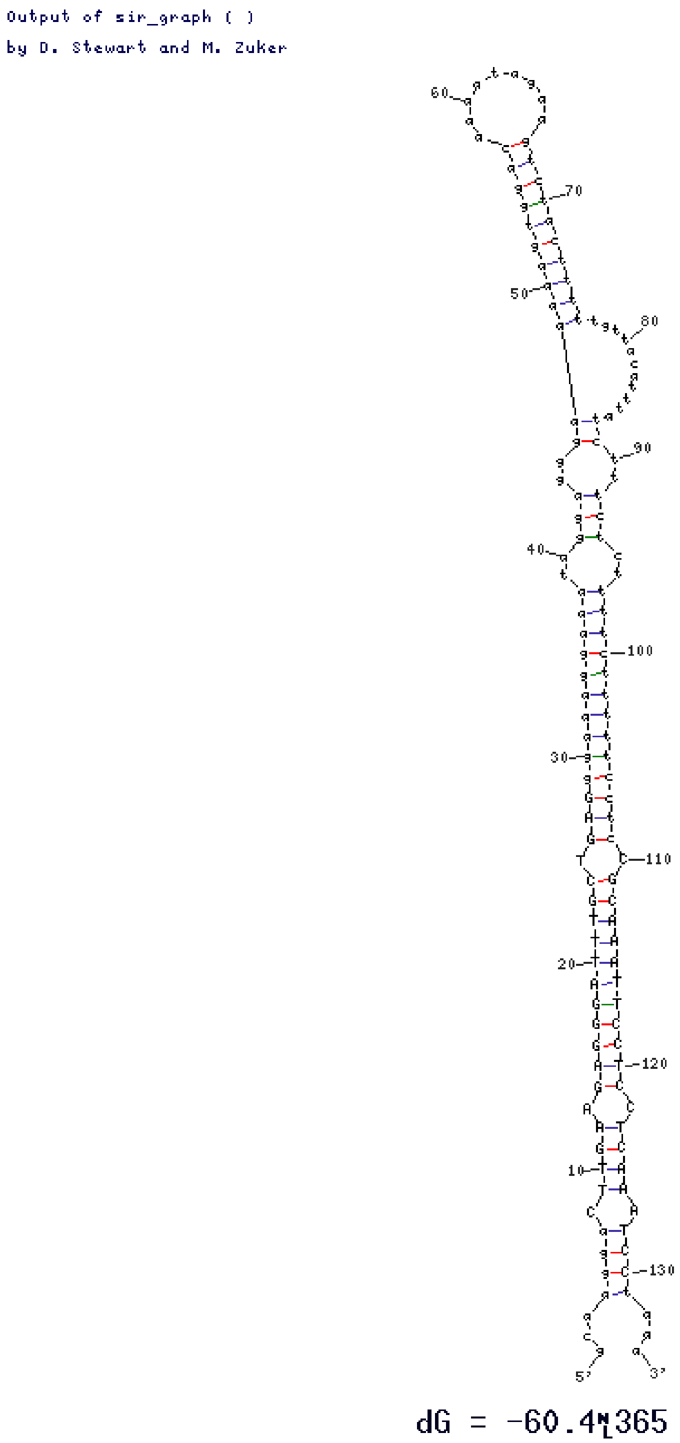

csn-smR77-3p
CGCAAAUUCCUCCUCAAAUCC

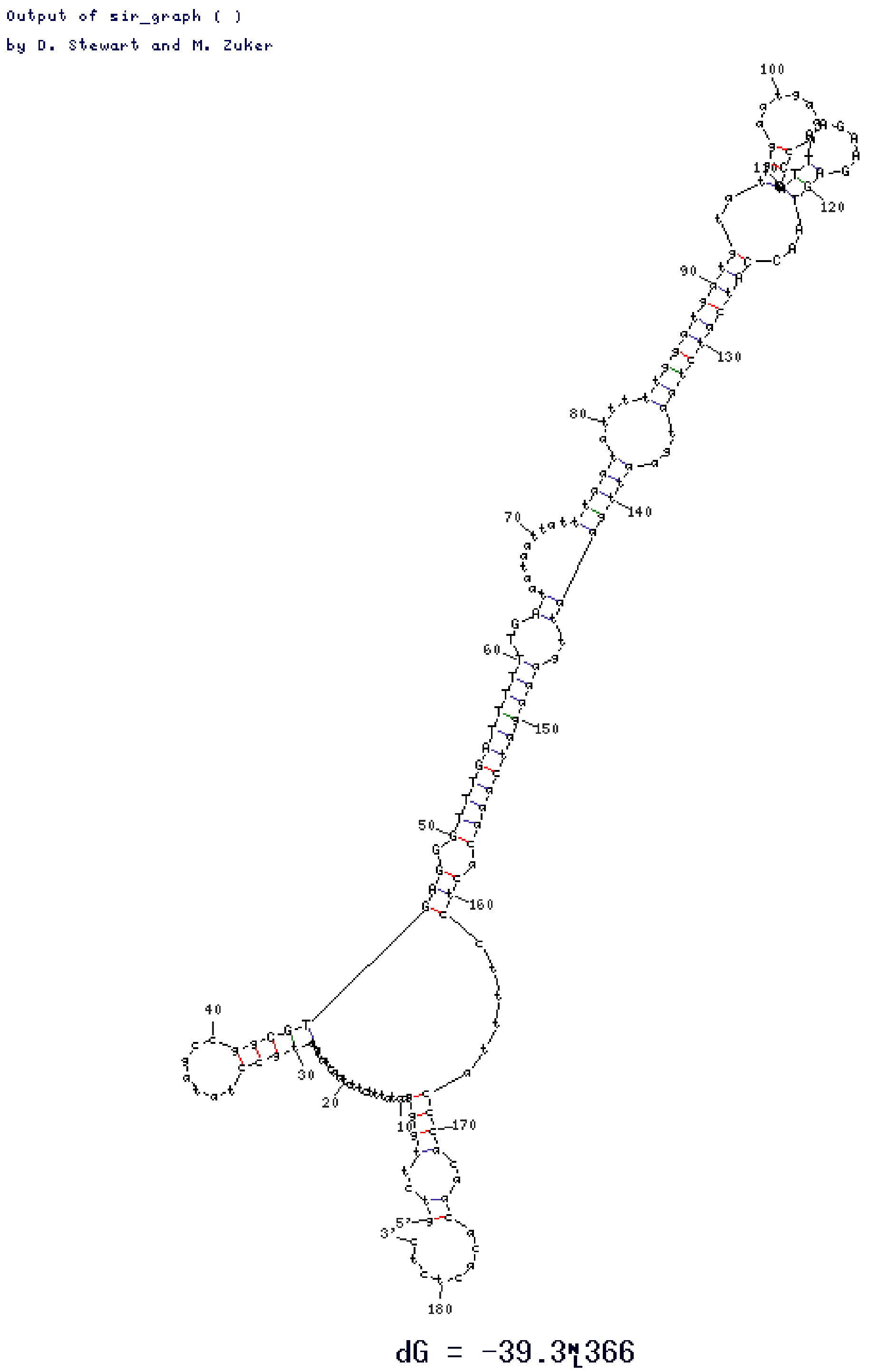

csn-smR78
CGUGAGGGUUUGAUUUUUUGA

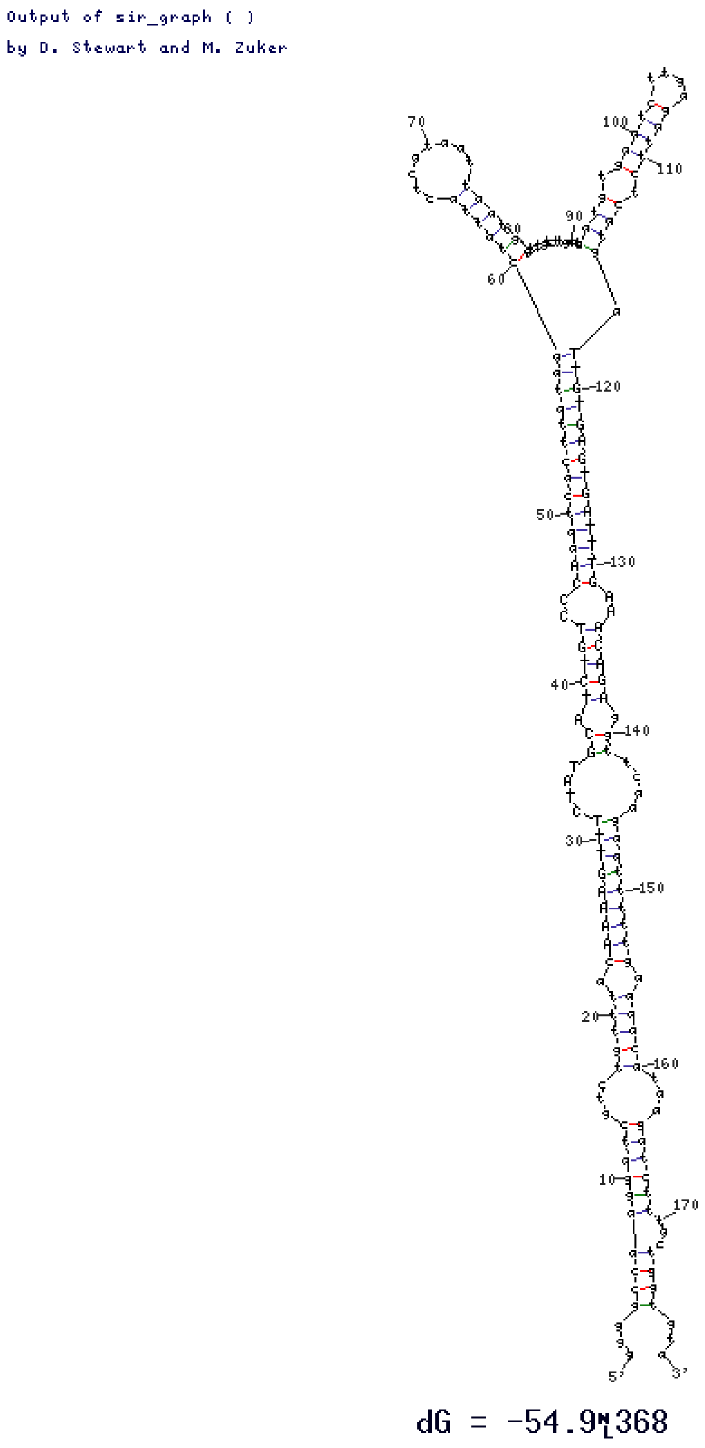

csn-smR79-5p
AAAAGUUUCUAUGCAUCUGUCCCA

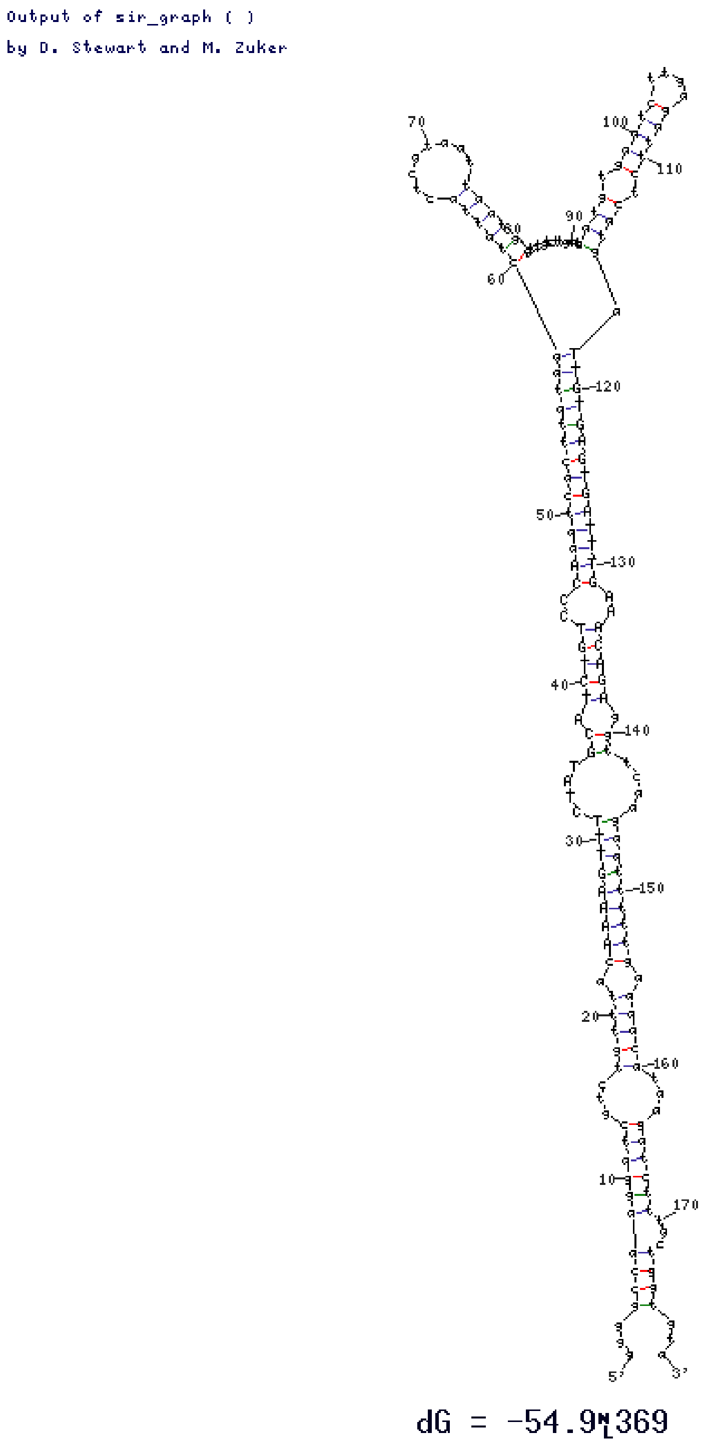

csn-smR79-3p
UUGUGAGUGAUUUGGAACAGA

Output of sir_graph ( )
by D. Stewart and M. Zuker

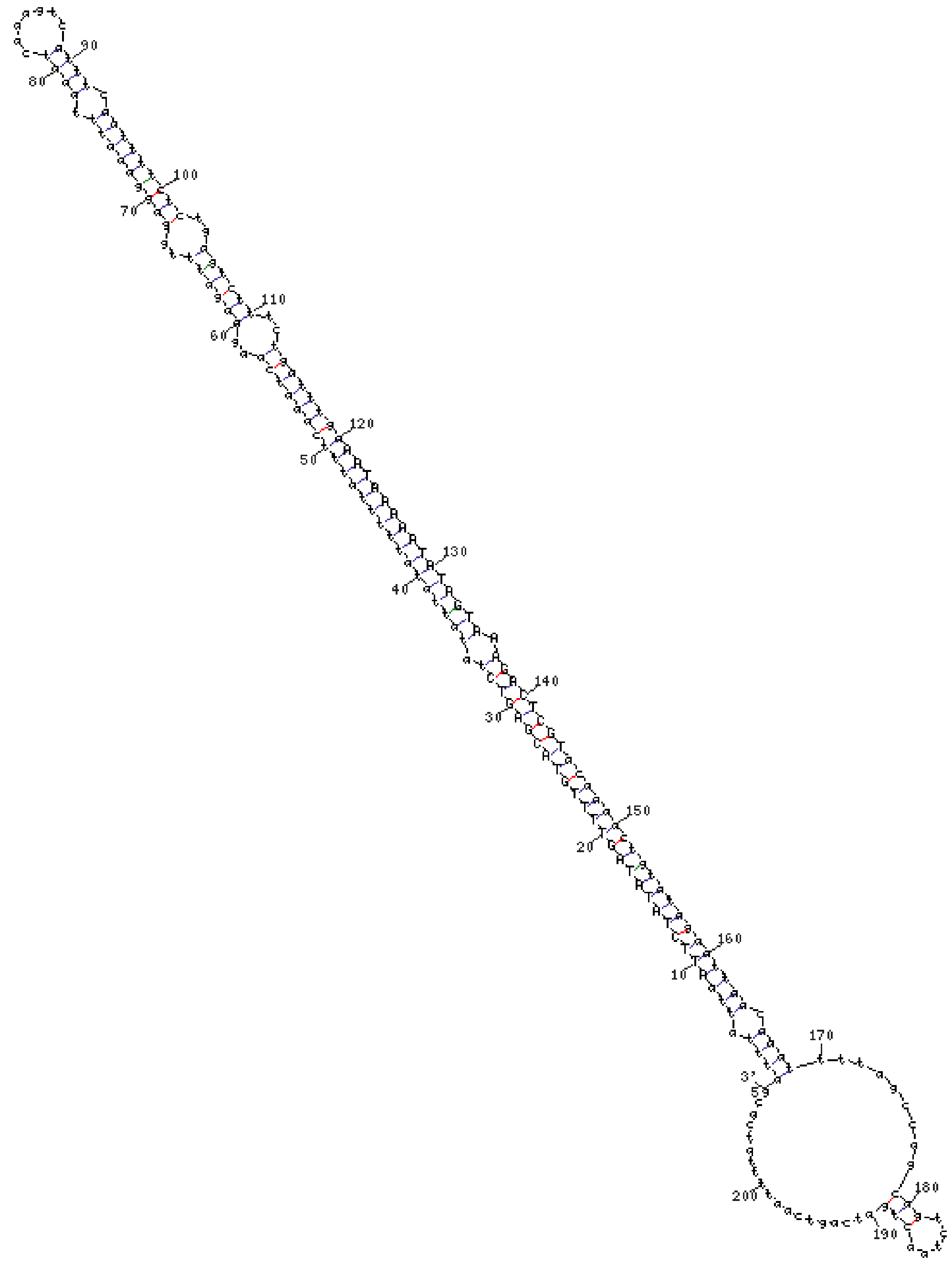

dG = -90.4[illegible]371

csn-smR80
AAUAAAAAUAUAGUAAAGACUCGU

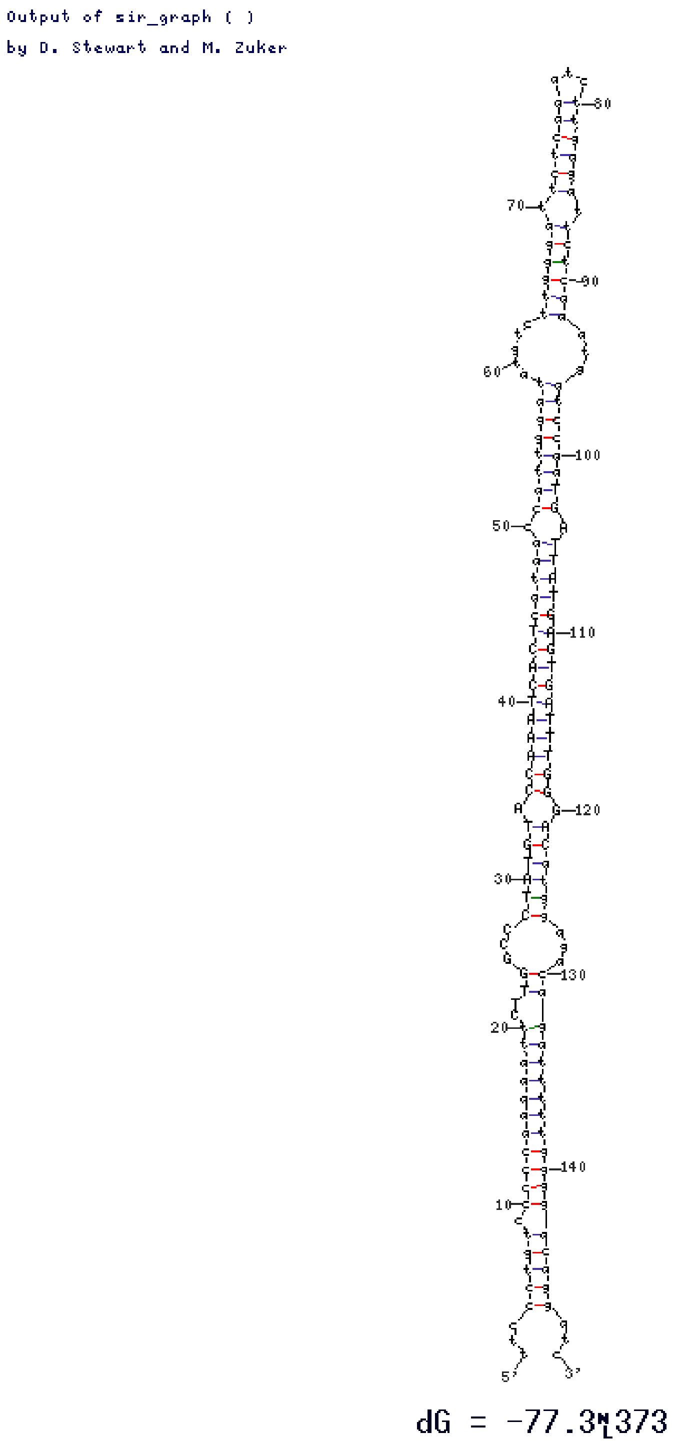

csn-smR81
UGAUUAUGAGUGAUUUGGGAC

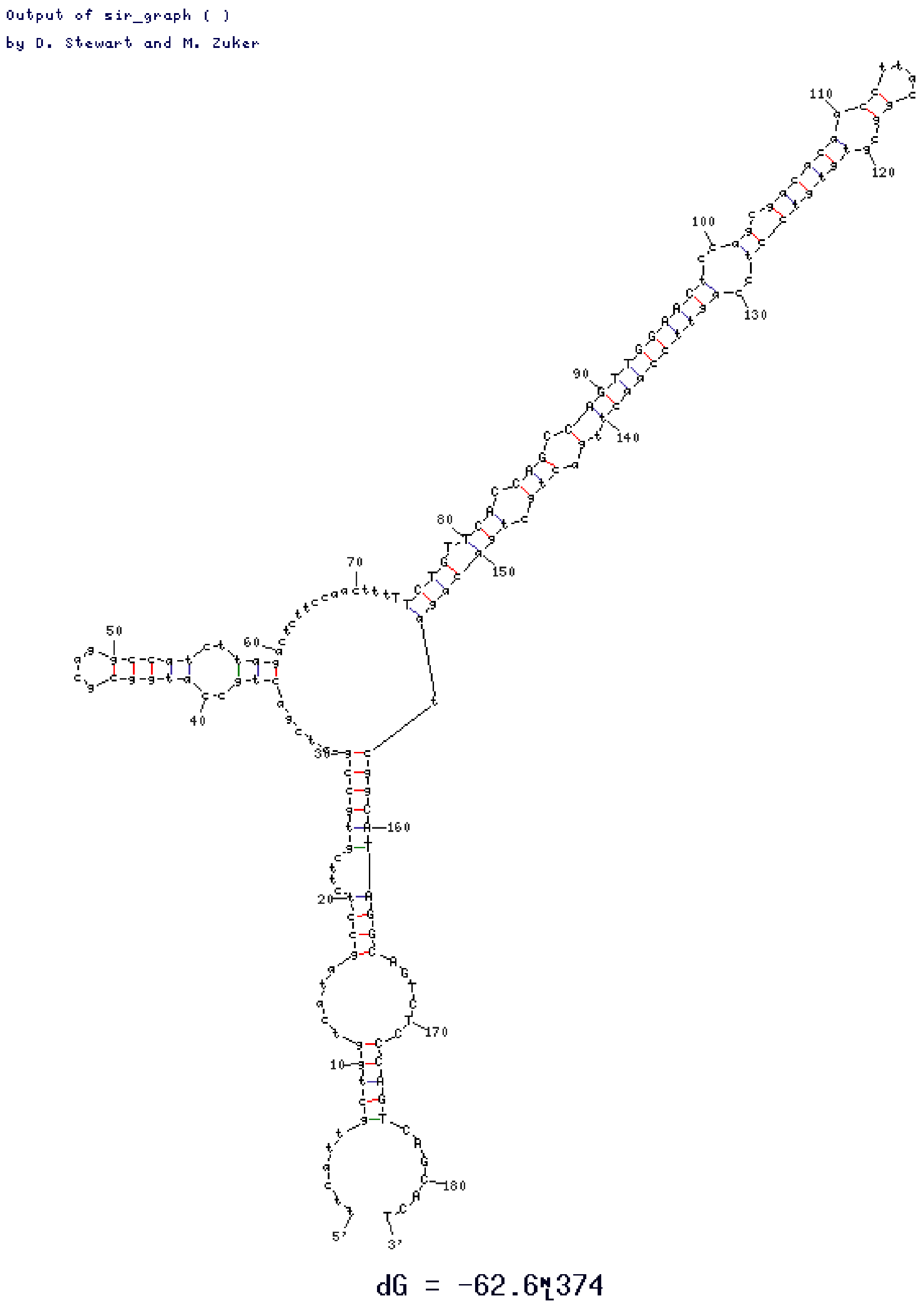

csn-smR82
UUCUGUUCACCAGCCAGUUGGAAC

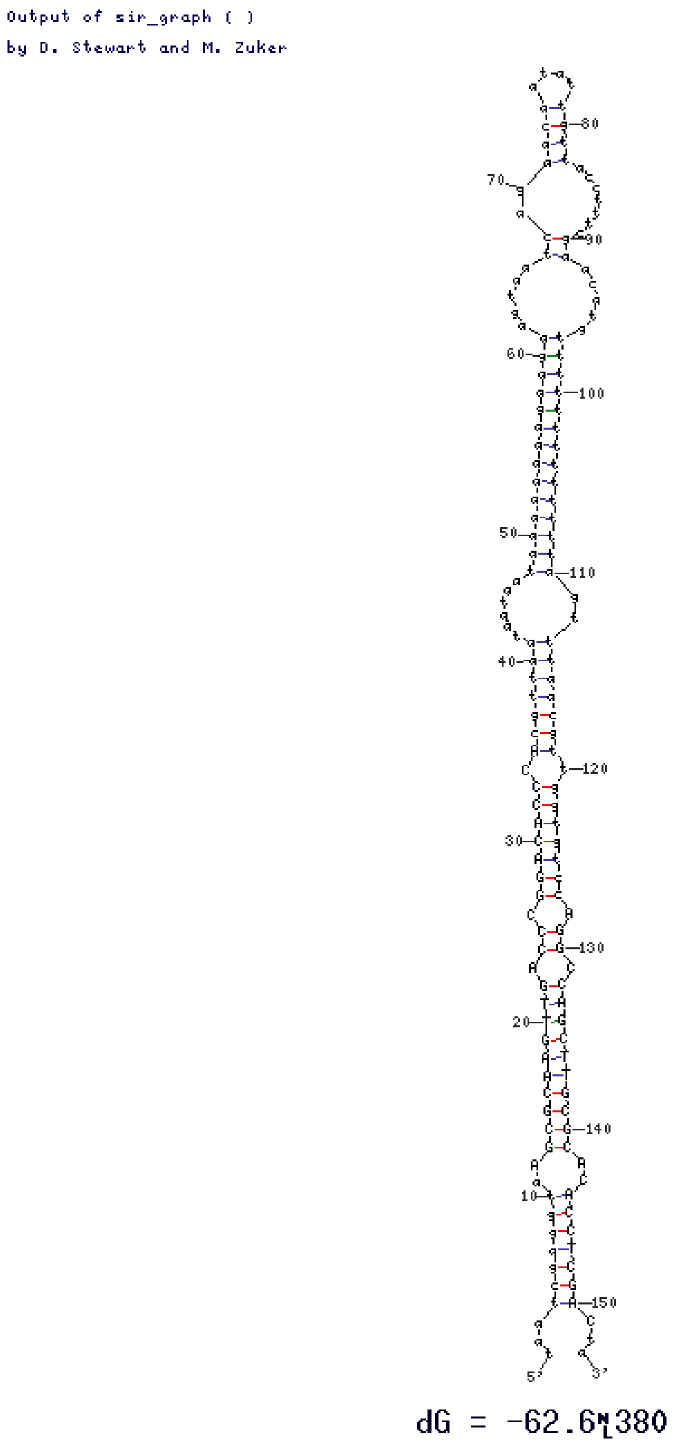

csn-smR84-5p
AGCGCAAGUUGACCCGGACACCCC

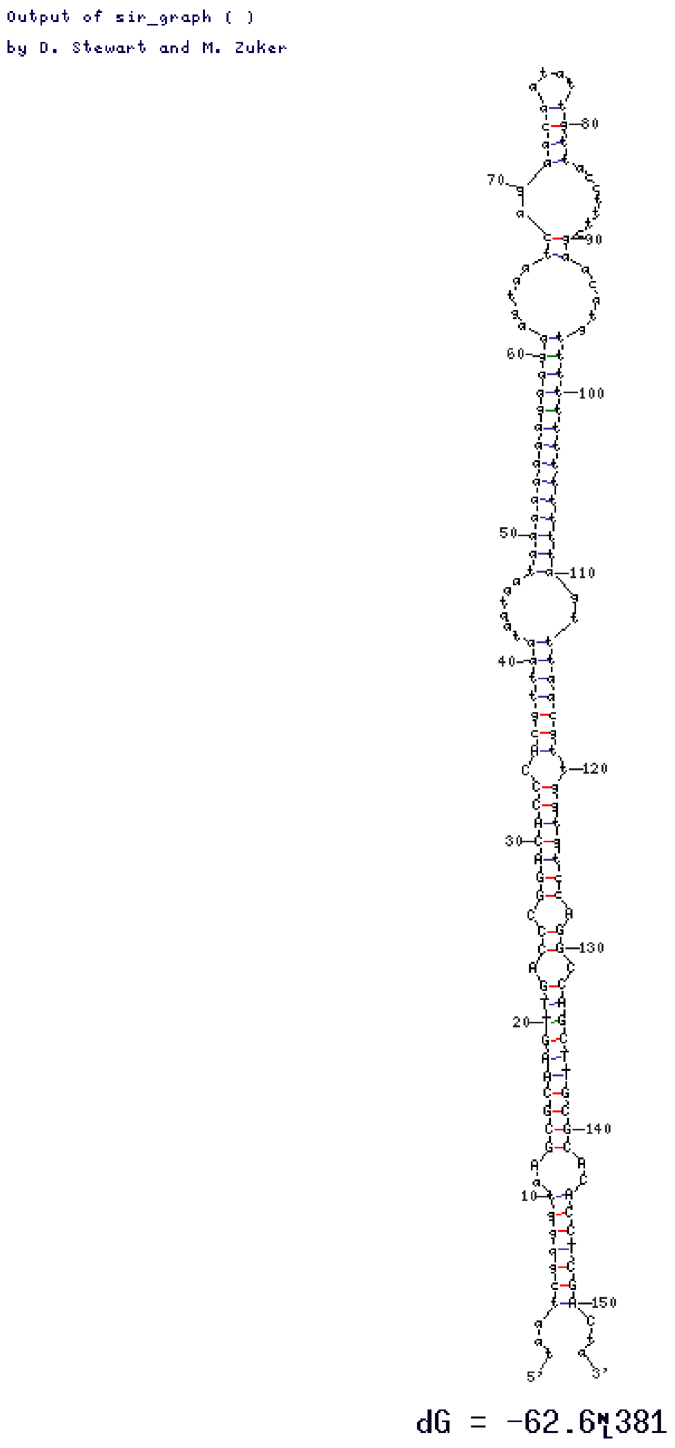

csn-smR84-3p
AGGCCAGCUUGCGCACAUCUCGAC

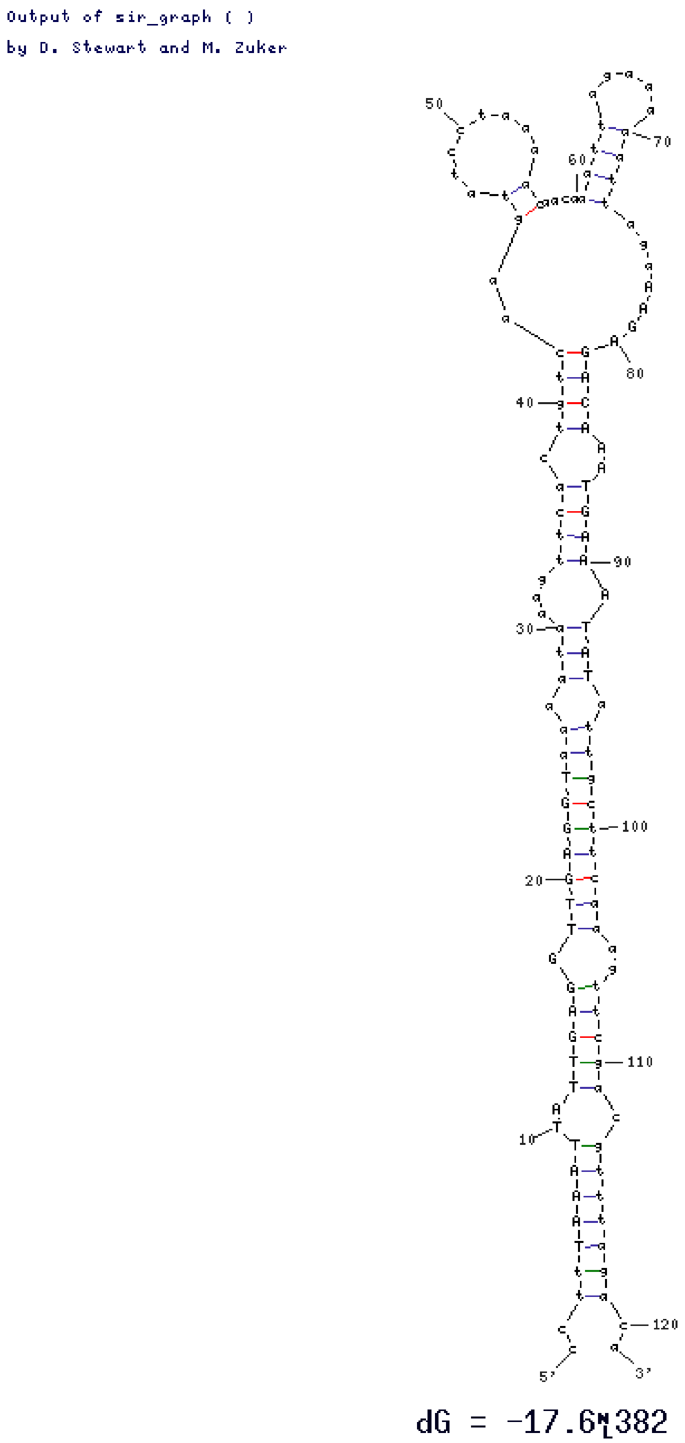

csn-smR85-5p
UAAAAUAUUGAGGUUGAGGU

Figure S1. Mature and precursor sequences and the predicted stem-loop structures of newly identified miRNAs from *Camellia sinensis.*
