# Supplementary material for: Identification and characterization of cold-responsive microRNAs in tea plant (Camellia sinensis) and their targets using high-throughput sequencing and degradome analysis
Source: BMC Plant Biol. 2014 Oct 21;14:271. doi: 10.1186/s12870-014-0271-x (PMC4209041; doi:10.1186/s12870-014-0271-x)
Supplement: Additional file 3: Table S2. — Novel miRNAs and potentially novel miRNAs identified from predicted RNA hairpins in C. sinensis with cold stress. [file 12870_2014_271_MOESM3_ESM.pdf]

Table S2 Novel miRNAs and potentially novel miRNAs identified from predicted RNA hairpins in *C. sinensis* with cold stress

miR_name	miR_seq	Type	Len	Reads	GenomeID	Strand	Start	End	Hairpin Length	Precursors Length	CG%	dG	MFEI	miRNA*
csn-smR1	AUCCGGGUUCUCGAUCAUUCGAGC	3'	24	2	24872_gi|399997300	+	656	919	66	72	52.8	-36.9	1	Y
csn-smR4-5p-1	GAACUGGAGGACACACGCCGUAAG	5'	24	4	76159_gi|319844893	-	1	221	76	102	52	-48.7	0.9	N
csn-smR4-3p-1	UGGAGUUCCAACUGGCUGGGGAGC	3'	24	4	76159_gi|319844893	-	1	221	76	102	52	-48.7	0.9	N
csn-smR4-5p-2	GAACUGGAGGACACACGCCGUAAG	5'	24	3	76159_gi|319888332	-	284	547	93	99	51.5	-49.8	1	N
csn-smR4-3p-2	UGGAGUUCCAACUGGCUGGGGAGC	3'	24	3	76159_gi|319888332	-	284	547	93	99	51.5	-49.8	1	N
csn-smR5-5p-1	GACUUCAUUUGGAUUGAGCCC	5'	21	54	24872_gi|400012797	+	70	330	165	172	42.4	-99.7	1.4	Y
csn-smR5-3p-1	UUCAAUCCGAAUAAAGUCCCC	3'	21	54	24872_gi|400012797	+	70	330	165	172	42.4	-99.7	1.4	Y
csn-smR5-5p-2	GACUUCAUUUGGAUUGAGCCC	5'	21	45	76159_gi|319850607	-	292	552	163	172	43	-94.9	1.3	Y
csn-smR5-3p-2	UUCAAUCCGAAUAAAGUCCCC	3'	21	45	76159_gi|319850607	-	292	552	163	172	43	-94.9	1.3	Y
csn-smR7-5p	ACAGAAAUCAAUCACAGAAUUAGAC	5'	25	4	24872_gi|400018074	-	563	827	129	135	25.2	-52.6	1.5	Y
csn-smR7-3p	UGAUUCUGUAAUUGAUUUCUGUAU	3'	24	4	24872_gi|400018074	-	563	827	129	135	25.2	-52.6	1.5	Y
csn-smR8	CAUAUUAUAGAAUCGGACAUA	5'	21	13	47452_gi|393751706	+	129	389	145	150	24.7	-66.6	1.8	Y
csn-smR9-5p-1	UUUGGAAAGGGAAAUGGAAAAGGU	5'	24	201	24872_gi|400016573	+	223	486	137	147	34	-73.7	1.5	Y
csn-smR9-3p-1	AUUUCCCUUUCCAAAUUCCUUCCC	3'	24	201	24872_gi|400016573	+	223	486	137	147	34	-73.7	1.5	Y
csn-smR9-5p-2	UUUGGAAAGGGAAAUGGAAAAGGU	5'	24	173	47452_gi|393741739	+	423	686	176	177	35.2	-84.6	1.3	N
csn-smR9-3p-2	AAAUUUCACAUAGUUUCACACACC	3'	24	173	47452_gi|393741739	+	423	686	176	177	35.2	-84.6	1.3	N
csn-smR11-1	UUGACAGUUUUGAGCCAUUUG	3'	21	46	76159_gi|319829157	+	1	254	124	128	35.4	-46.7	1	N
csn-smR11-2	UUGACAGUUUUGAGCCAUUUG	3'	21	29	24872_gi|400018477	+	45	305	116	132	35.8	-49.7	1	Y
csn-smR12-1	AUUAGUUUAGAUUGUAGGACC	3'	21	34	76159_gi|319824063	+	351	611	166	205	30.7	-81.7	1.3	N
csn-smR12-2	AUUAGUUUAGAUUGUAGGACC	3'	21	74	76159_gi|319824063	-	281	541	166	201	31.3	-89.8	1.4	Y
csn-smR13	UCUGGUUCUAUGAUAUGUUUC	3'	21	13	47452_gi|393751706	+	129	389	145	150	24.7	-66.6	1.8	Y

csn-smR15-5p	UUUGGAAAAGAAAAUGAAAAAGGU	5'	24	6	24872_gi|399995935	+	1157	1417	180	177	33.9	-75.7	1.2	Y
csn-smR15-3p	UUUUCUUUCCUUUUCCAAAUC	3'	21	6	24872_gi|399995935	+	1157	1417	180	177	33.9	-75.7	1.2	Y
csn-smR17	AUGUCGUUUUGAUACAUUGGAGCC	3'	24	10	24872_gi|399996848	+	246	509	124	135	36.3	-69.7	1.4	N
csn-smR18-5p	AAUGGCUCAAAUGUAUCAAAACGA	5'	24	31	24872_gi|399996848	-	249	512	124	167	32.3	-48.9	0.9	Y
csn-smR18-3p	ACGUCAUUUUGAGACAUUCGAACC	3'	24	31	24872_gi|399996848	-	249	512	124	167	32.3	-48.9	0.9	Y
csn-smR19-5p	AUCCGUCCAAAAGCUAUGAGAGGC	5'	24	4	24872_gi|399996935	+	311	574	151	157	47.1	-68.6	0.9	Y
csn-smR19-3p	CACAGUUUUUGUACUGGACAGCCU	3'	24	4	24872_gi|399996935	+	311	574	151	157	47.1	-68.6	0.9	Y
csn-smR20	AAAAGUUUUACCAAAUGUCUCACA	5'	24	54	24872_gi|399998954	+	1	184	173	176	36.4	-77.9	1.2	N
csn-smR27	AAAAUUUAUGACCCUUGGAUUACC	5'	24	137	24872_gi|400010527	+	5	268	160	174	30.2	-68.1	1.3	Y
csn-smR28	AAAGUUUUGUGACCCUUGGAUUAC	5'	24	92	24872_gi|400010527	-	60	323	156	171	29.2	-53.3	1.1	N
csn-smR29	UUUGGUAGAAAAUUUGGUAUC	3'	21	195	24872_gi|400010809	-	1	193	151	152	30.9	-70.6	1.5	Y
csn-smR30	AUUGUGUGGUUGAGAUUUACUGGC	5'	24	1644	24872_gi|400011477	-	41	304	173	232	35.8	-114.3	1.4	N
csn-smR31-5p	AUUGUGUGGUUGAGAUUUAUUGGC	5'	24	518	24872_gi|400014797	+	1	168	129	152	36.8	-75.6	1.4	Y
csn-smR31-3p	AACCACACAAUGUAACACUCUAGG	3'	24	518	24872_gi|400014797	+	1	168	129	152	36.8	-75.6	1.4	Y
csn-smR32	UUUGGAAAGGGAAAUGGAAAA	5'	21	73	24872_gi|400016573	-	297	557	147	179	33.5	-63.6	1.1	N
csn-smR34-5p	CAAAUGGCUCAAAACUGUCAA	5'	21	8	24872_gi|400018477	-	45	305	118	124	35.5	-33	0.7	Y
csn-smR34-3p	UUUUGACAUUUUCGGGCCAUUUGA	3'	24	8	24872_gi|400018477	-	45	305	118	124	35.5	-33	0.7	Y
csn-smR35	UUUGGUAGAAAAUUUGGGACC	3'	21	181	47452_gi|212378209	-	1	176	151	157	33.8	-62.4	1.2	Y
csn-smR37	AUUGUGUGGUUGAGAUUUAAUGAC	5'	24	668	47452_gi|313605263	+	45	308	169	174	33.1	-72.7	1.3	Y
csn-smR38	GGCUAUGGCGCCGGCCCAGAC	3'	21	3	47452_gi|340628927	+	1	254	72	78	55.1	-31.1	0.7	Y
csn-smR39	UAUGAAAUAUAAACUUCAGGGGGC	3'	24	7	47452_gi|366889485	+	163	426	154	168	34.5	-51.9	0.9	N
csn-smR40-5p	UUCUAGAAGUCCUAUUAGAAGAGC	5'	24	6	47452_gi|366892224	-	37	300	203	209	37.8	-57.8	0.7	N
csn-smR40-3p	CGGGACGAUCAUUAUUUAGAAAGG	3'	24	6	47452_gi|366892224	-	37	300	203	209	37.8	-57.8	0.7	N
csn-smR41	UCGGAUAUCGAUAGCUAUAUGAUC	3'	24	21	47452_gi|366894819	-	21	284	179	264	25	-62.1	0.9	N

csn-smR44-5p	AUGACUCGAAUGUCUCAAAACGAC	5'	24	20	47452_gi|393393589	+	23	286	148	160	33.1	-72.5	1.4	Y
csn-smR44-3p	ACAUUCGAGCUAUUCGGACCACAC	3'	24	20	47452_gi|393393589	+	23	286	148	160	33.1	-72.5	1.4	Y
csn-smR45-5p	AAAAAUGAUUGGUAGCUCGAAUUU	5'	24	30	47452_gi|393393589	-	16	279	126	128	36.4	-66.5	1.4	N
csn-smR45-3p	UGAGACAUUCGAGCCAUUUGAGCC	3'	24	30	47452_gi|393393589	-	16	279	126	128	36.4	-66.5	1.4	N
csn-smR47-5p	CGGAUGGAGAAGGCACAACCU	5'	21	5	47452_gi|393744612	+	121	380	148	207	39.1	-42.4	0.5	N
csn-smR47-3p	UAGGAUACAUUUUUAAGAAA	3'	20	5	47452_gi|393744612	+	121	380	148	207	39.1	-42.4	0.5	N
csn-smR49a-5p	AGAAACAUAUUAUAGAAUCGGACA	5'	24	9	47452_gi|393746364	+	528	791	145	151	24.5	-66.6	1.8	Y
csn-smR49a-3p	UGUGUCUGGUUCUAUGAUAUG	3'	21	9	47452_gi|393746364	+	528	791	145	151	24.5	-66.6	1.8	Y
csn-smR49b	UGUGUCUGGUUCUAUGAUAUGUU	3'	23	5	47452_gi|393751507	+	523	791	145	151	24.5	-66.6	1.8	Y
csn-smR50	ACAAUGAUGCCUAUAUAUAGAAGAG	3'	25	8	47452_gi|393747051	-	18	282	190	193	36.3	-94.5	1.3	N
csn-smR53-5p	UGAGUGUGGGACUGAUACACGGGUC	5'	25	6	47452_gi|393752671	+	67	330	133	139	25.9	-72.7	2	N
csn-smR53-3p	AAAUUUAAUAAAUUAUUUGGAATC	3'	24	6	47452_gi|393752671	+	67	330	133	139	25.9	-72.7	2	N
csn-smR55	UUUUGUUCCAAUUCGGGCGACC	3'	22	35	47452_gi|393756925	-	1	257	118	248	40.3	-71	0.7	Y
csn-smR57-5p	UUUGGUUCCGAUCACUCAUGAU	5'	22	583	76159_gi|319803471	-	527	787	170	174	38.1	-98.4	1.5	Y
csn-smR57-3p	UAUGUGGCCGAUCAUGAGUGG	3'	21	583	76159_gi|319803471	-	527	787	170	174	38.1	-98.4	1.5	Y
csn-smR58	ACUAAAGCUAUAGCCAGAGAAC	5'	22	8	76159_gi|319816168	+	1	160	119	122	45.9	-42	0.7	N
csn-smR59-5p	UAGGGCAUAUGUUAAGAGAGC	5'	21	9	76159_gi|319823336	+	1	143	106	109	35.8	-53	1.4	Y
csn-smR59-3p	CUUCUUAACAUGUGCCCUAAG	3'	21	9	76159_gi|319823336	+	1	143	106	109	35.8	-53	1.4	Y
csn-smR60-5p	AAAUUCUGUGACUCAAAUGACUCA	5'	24	82	76159_gi|319829157	-	1	197	115	116	37.1	-37.1	0.9	N
csn-smR60-3p	UUUUGACAUUUUCGGACCAUU	3'	21	82	76159_gi|319829157	-	1	197	115	116	37.1	-37.1	0.9	N
csn-smR61	AAUUUCUGUUCACCGGUCGUCCGG	5'	24	3	76159_gi|319842121	+	88	351	148	154	43.5	-63	0.9	N
csn-smR62-5p	AAGUUACAGAAUCGGACAUAGAAU	5'	24	5	76159_gi|319844167	+	1	252	110	117	30.8	-39.4	1.1	N
csn-smR62-3p	ACUUUGUAAAAUCUUAUCAUCCGC	3'	24	5	76159_gi|319844167	+	1	252	110	117	30.8	-39.4	1.1	N
csn-smR63	UAUGUAUACGGUAUUUGGUUC	3'	21	7	76159_gi|319847284	+	214	474	115	128	33.6	-34	0.7	N

csn-smR64-5p	UUUGGAAAGAUAAAUGAAAAAGU	5'	23	12	76159_gi|319848059	+	8	270	151	157	30.6	-49.7	1	N
csn-smR64-3p	AAUCCCUCACCAAAUCCUUAAGCC	3'	24	12	76159_gi|319848059	+	8	270	151	157	30.6	-49.7	1	N
csn-smR65	UACGGGUUCAUAGAUGAAGAA	3'	21	794	76159_gi|319850845	+	1	247	124	128	32	-46.1	1.1	Y
csn-smR66-5p	AAGUUGGGUUUGGGCUAAAUUAGC	5'	24	13	76159_gi|319852943	+	1	251	62	101	38.6	-34.6	0.9	N
csn-smR66-3p	AAUGGACAAAAGAAAGUUGGGAGC	3'	24	13	76159_gi|319852943	+	1	251	62	101	38.6	-34.6	0.9	N
csn-smR67	UCAAAUCUCUUUGUAAAUCUC	3'	21	104	76159_gi|319853691	-	453	713	146	151	21.2	-43.5	1.4	Y
csn-smR68-5p	UUUGGAGAGGGAUUUGGAAAG	5'	21	31	76159_gi|319854318	-	1	168	152	165	31.5	-55.3	1.1	N
csn-smR68-3p	AAAUUCUUGAACCAAAUGCAGCCU	3'	24	31	76159_gi|319854318	-	1	168	152	165	31.5	-55.3	1.1	N
csn-smR69-5p	AGAAUCAGACAUAACUCCUUU	5'	21	2	76159_gi|319855438	+	236	499	151	157	38.9	-66.5	1.1	N
csn-smR69-3p	GAUUCUGUGAUCGGUUUCUGUGUU	3'	24	2	76159_gi|319855438	+	236	499	151	157	38.9	-66.5	1.1	N
csn-smR71-5p	AAUCAUUGGAUUAUAUGAGAAUCU	5'	24	44	76159_gi|319863099	+	1	241	149	158	32.9	-77.2	1.5	N
csn-smR71-3p	AAUGGUUGUGAGUGAUUUGAGACA	3'	24	44	76159_gi|319863099	+	1	241	149	158	32.9	-77.2	1.5	N
csn-smR72-5p	UUGGUUCAUGAAUUUGGGAAG	5'	21	17	76159_gi|319863994	-	477	737	139	143	29.4	-59.7	1.4	Y
csn-smR72-3p	UCUUUCCAAAUUUCUCCUCAAAUC	3'	24	17	76159_gi|319863994	-	477	737	139	143	29.4	-59.7	1.4	Y
csn-smR73-5p	UUGAAGUGCUUGGUCUGGCCGAGG	5'	24	2	76159_gi|319870358	-	1	236	127	131	61.8	-61.7	0.8	Y
csn-smR73-3p	GCUCGGUCUGGUCGAGGAC	3'	19	2	76159_gi|319870358	-	1	236	127	131	61.8	-61.7	0.8	Y
csn-smR74-5p	CAAAACUGGACACAACCCUCUC	5'	22	5	76159_gi|319871291	-	16	279	84	83	43.3	-45.7	1.2	Y
csn-smR74-3p	ACCUCAUGUGAGAGGAUUGUGUCC	3'	24	5	76159_gi|319871291	-	16	279	84	83	43.3	-45.7	1.2	Y
csn-smR76-5p	AUAAAUCCCACCCUUUUUACCAAU	5'	24	5	76159_gi|319874140	-	1	165	103	108	36.1	-46.4	1.2	Y
csn-smR76-3p	AAUUGUGAUUGGUGAAAAGAGUGA	3'	24	5	76159_gi|319874140	-	1	165	103	108	36.1	-46.4	1.2	Y
csn-smR77-5p	CUUGAUGAGGGAUUUGCUGAG	5'	21	20	76159_gi|319876865	-	206	466	128	134	37.3	-60.4	1.2	Y
csn-smR77-3p	CGCAAAUUCCUCCUCAAAUCC	3'	21	20	76159_gi|319876865	-	206	466	128	134	37.3	-60.4	1.2	Y
csn-smR79-5p	AAAAGUUUCUAUGCAUCUGUCCCA	5'	24	17	76159_gi|319880415	+	148	408	173	179	33	-54.9	0.9	N
csn-smR79-3p	UUGUGAGUGAUUUGGAACAGA	3'	21	17	76159_gi|319880415	+	148	408	173	179	33	-54.9	0.9	N

csn-smR80	AAUAAAAAUAUAGUAAAGACUCGU	3'	24	46	76159_gi|319880714	+	138	401	169	208	25	-90.4	1.7	N
csn-smR81	UGAUUAUGAGUGAUUUGGGAC	3'	21	23	76159_gi|319885366	+	1932	2192	144	150	40.7	-77.3	1.3	Y
csn-smR84-5p	AGCGCAAGUUGACCCGGACACCCC	5'	24	2	scaffold_8	+	8721753	8722016	147	153	37.9	-62.6	1.1	Y
csn-smR84-3p	AGGCCAGCUUGCGCACAUCUCGAC	3'	24	2	scaffold_8	+	8721753	8722016	147	153	37.9	-62.6	1.1	Y
csn-smR85	UAAAAUAUUGAGGUUGAGGU	5'	20	2	scaffold_8	-	4209255	4209514	117	121	28.1	-17.6	0.5	N

Note: ‘Y’ and ‘N’ with and without express signal
