# Supplementary figures and images for: Identification and characterization of cold-responsive microRNAs in tea plant (Camellia sinensis) and their targets using high-throughput sequencing and degradome analysis
Source: BMC Plant Biol. 2014 Oct 21;14:271. doi: 10.1186/s12870-014-0271-x (PMC4209041; doi:10.1186/s12870-014-0271-x)

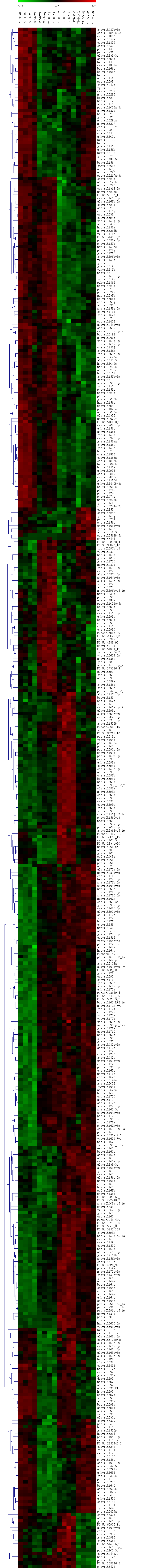

Supplement: Additional file 4: Figure S2. — t-plot for targets of the known miRNAs found in + C and -C libraries of C. sinensis. Signature abundance throughout the length of the transcript is show. Arrows indicate signature consistent with miRNA-directed cleavage. [file 12870_2014_271_MOESM4_ESM.tiff]

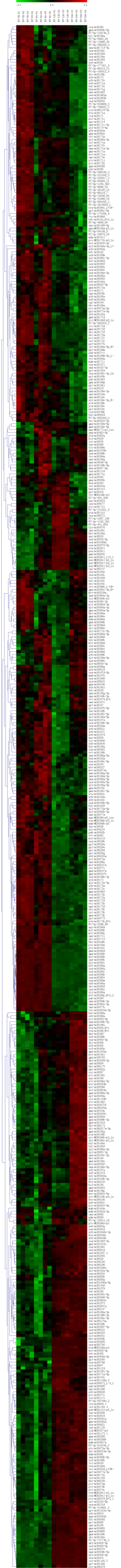

Supplement: Additional file 5: Figure S3. — t-plot for targets of the new miRNA candidates found in + C and -C libraries of C. sinensis. Signature abundance throughout the length of the transcript is show. Arrows indicate signature consistent with miRNA-directed cleavage. [file 12870_2014_271_MOESM5_ESM.tiff]
