# Supplementary material for: Identification and characterization of cold-responsive microRNAs in tea plant (Camellia sinensis) and their targets using high-throughput sequencing and degradome analysis
Source: BMC Plant Biol. 2014 Oct 21;14:271. doi: 10.1186/s12870-014-0271-x (PMC4209041; doi:10.1186/s12870-014-0271-x)
Supplement: Additional file 6: Table S3. — Relative expression analysis of cold-responsive miRNAs in ‘Yingshuang’. The threshold of p value was set 0.01, ***indicated p value less than 0.0001, **indicated p value from 0.0001 to 0.001, *indicated p value from 0.001 to 0.01, respectively. [file 12870_2014_271_MOESM6_ESM.pdf]

Table S3 Relative expression analysis of cold-responsive miRNAs in ‘Yingshuang’

miRNA Family	miRNA Probes	P	YS-CK		YS-4h		YS-12h		YS-24h		Relative fold difference (Log2)					
			Mean	StDev	Mean	StDev	Mean	StDev	Mean	StDev	4h/CK	12h/CK	24h/CK	12h/4h	24h/4h	24h/12h
miR156	mdm-miR156ad	***	399	29	533	44	263	31	302	27	0.51			-0.96	-0.83	
	ptc-MIR156f-p3	***	169	24	234	35	513	14	273	62		1.75		1.19		0.97
	har-miR156c	***	3383	284	3117	181	2587	210	2210	101		-0.34	-0.58		-0.50	
	hci-miR156a	***	499	18	589	24	380	50	354	39	0.31				-0.74	
	vvi-miR156h	***	3130	459	2908	284	2090	79	1961	221			-0.64	-0.47	-0.58	
	ath-miR156j	***	7204	436	5385	818	4405	229	4058	729		-0.68	-0.79			
	bdi-miR156a	***	3444	344	2731	383	2011	189	2316	217		-0.72	-0.54			
	ath-miR156i	**	3126	277	2415	282	1930	126	1879	314		-0.64	-0.69			
	gma-miR156f	**	7057	705	6300	923	4607	352	4559	514		-0.58	-0.59			
	far-miR156b	**	7082	576	5605	794	4609	376	4256	802		-0.59	-0.70			
	cme-miR156g	**	2120	376	1810	273	1158	41	1595	208						
	gma-miR156q	*	464	39	566	26	518	24	459	32	0.36				-0.30	
	aly-miR156e-3p	*	1345	183	1164	182	1714	218	1817	219				0.60	0.66	
	cca-miR156b	*	502	7	607	14	557	35	484	57		0.28				
	osa-miR156l-5p	*	540	37	648	35	503	70	498	34	0.33				-0.38	
	gma-miR156k	*	701	78	788	74	661	47	591	28					-0.41	

	nta-miR156f	*	365	69	580	53	342	34	337	100	0.76			-0.71		
	cme-miR156j	*	687	111	804	84	645	23	559	67					-0.53	
miR159	gma-miR159e-5p	***	321	21	476	57	725	66	630	28	0.64	1.29	1.05	0.66		
	osa-miR159f	***	4477	155	4815	131	5738	276	5510	169	0.14	0.38	0.33	0.24	0.19	
	vvi-miR159a	***	795	75	849	211	284	78	327	15		-1.37	-1.21	-1.50	-1.34	
	ptc-MIR159b-p5_1ss20GA	***	254	29	373	41	643	55	523	71		1.47	1.13	0.85	0.51	
	zma-miR159h-3p	***	1193	193	1180	246	342	30	484	84		-1.69	-1.23	-1.71	-1.26	
	pta-miR159b	***	1644	238	2073	168	926	154	1287	93		-0.74		-1.11	-0.67	
	pta-miR159a	***	2858	54	3149	89	3648	192	3242	121	0.17	0.39	0.21	0.21		
	zma-miR159e-3p	***	1662	130	1698	94	1027	52	1160	179		-0.60		-0.68		
	osa-miR159e	***	3867	101	4089	133	4637	170	4329	110		0.29	0.20	0.17		
	pta-miR159c	***	2210	86	2260	53	2307	98	1940	34			-0.15		-0.22	-0.26
	mdm-miR159a	***	3526	95	3704	93	4202	127	3978	182	0.11	0.28	0.21	0.17		
	osa-miR159c	***	3785	86	4067	96	4671	342	4425	30			0.26		0.12	
	pde-miR159	***	3148	87	3703	236	4337	299	4201	378	0.27	0.49	0.45			
	sbi-miR159b	**	3589	163	3634	241	4564	296	3967	176		0.37		0.32		
	bdi-miR159	*	4514	251	4622	259	5751	226	5200	606		0.38		0.31		
	aly-miR159c-3p_R-1_1ss8TC	*	2225	120	2709	223	2320	48	2367	159	0.33					
miR164	osa-miR164c	***	539	27	777	25	1163	40	992	32	0.58	1.22	0.95	0.64	0.36	-0.27
	bdi-miR164c	***	525	28	731	48	1150	122	906	15	0.54	1.24	0.86	0.70	0.32	

	gma-miR164b	***	275	12	449	44	839	56	589	19	0.77	1.75	1.18	0.98		-0.57
	osa-miR164e	***	603	42	808	23	1237	127	1051	68	0.48	1.14	0.86	0.66	0.38	
	mdm-miR164a	***	240	33	400	18	671	66	482	42		1.63	1.08	0.82		-0.55
	sbi-miR164c	***	646	47	816	60	1278	90	1028	61	0.39		0.73	0.69		0.34
	ath-miR164a	***	525	43	711	43	1065	115	837	66	0.50	1.13	0.74	0.64		-0.39
	ath-miR164c	***	521	35	692	77	1091	73	809	81	0.47	1.18	0.71	0.71		-0.47
	rco-miR164d	*	489	53	754	31	1219	79	814	335	0.69	1.43		0.74		
miR166	csi-miR166a-5p_R+1	***	415	12	387	25	593	45	444	32		0.62		0.67		-0.46
	aly-miR166e-5p	***	495	28	381	21	560	61	437	8				0.60		
	aly-miR166a-5p	**	547	24	405	9	578	87	503	24	-0.38				0.32	
	osa-miR166i-3p	**	9726	1376	8559	1215	5203	836	7477	915		-0.86		-0.72		
	osa-miR166i-3p_2ss6TC14CT	**	7271	338	8444	661	8907	433	9275	615		0.33	0.39			
	zma-miR166k-5p	*	511	158	290	101	131	38	176	79		-1.82				
	bdi-miR166e	*	24273	2467	17433	2046	19964	1537	19560	965						
	bdi-miR166f	*	25103	2835	18492	2788	19263	1710	19988	1587						
miR167	ppt-miR167	***	2174	96	2996	297	3387	184	3299	262	0.51	0.68	0.64			
	osa-miR167d-5p	***	2702	127	3298	97	3564	336	3639	203	0.33	0.44	0.47			
	vvi-miR167d_R+1	***	1784	30	2528	241	2821	241	2651	338	0.55	0.71	0.61			
	tae-miR167b	**	1369	209	1569	272	745	88	1051	175		-0.78		-1.02		
	mdm-miR167h	**	2076	62	2477	95	2759	301	2691	117	0.30		0.41			

	ptc-miR167h-5p	**	3135	432	4149	374	4186	363	4511	218						
	cme-miR167e	*	1506	77	1870	174	2047	88	1900	235		0.52				
	cme-miR167c	*	2777	217	3297	231	3676	344	3558	307		0.44	0.39			
	bna-miR167a	*	1831	262	2231	238	2723	200	2402	401						
miR168	gma-miR168b	***	787	46	619	58	1167	79	1514	90		0.67	1.01	0.96	1.31	0.34
	osa-miR168b	***	518	69	404	4	817	92	1055	60		0.77	1.08	1.07	1.38	
	bna-miR168b	***	669	80	452	51	977	121	1105	84	-0.50	0.66	0.78	1.16	1.28	
	aly-miR168a-3p	***	874	55	664	38	1001	101	1349	119	-0.34		0.68	0.64	1.03	
	cme-miR168	***	837	60	601	64	1188	95	1400	273	-0.43	0.61		1.04	1.24	
	mtr-miR168a	***	1094	138	723	97	1260	75	1652	359				0.85	1.21	
	sof-miR168b	**	377	66	313	46	534	72	633	121				0.83	1.02	
miR171	aqc-miR171f	***	911	36	1159	86	1221	47	1310	94	0.39	0.52	0.59			
	bdi-miR171b	**	1092	47	1253	55	1325	98	1421	76	0.24	0.37	0.45			
	zma-miR171b-3p	**	785	66	956	53	1047	120	1175	75	0.33	0.52	0.65		0.33	
	cca-miR171	**	941	44	1094	39	1058	95	1233	49	0.26		0.46		0.20	
	gma-miR171a	**	591	52	744	60	781	60	796	62	0.36	0.51	0.49			
	zma-miR171c-3p	**	999	68	1160	32	1221	108	1356	117	0.26	0.38	0.51			
	zma-miR171f-3p	*	763	69	912	39	983	130	1049	58			0.53		0.23	
	smo-miR171a	*	504	39	715	69	636	44	634	95	0.53	0.44				
	mtr-miR171c	*	1035	74	1192	101	1254	85	1239	65		0.37	0.33			

miR319	vvi-miR319g	***	2359	124	2416	382	848	72	1090	222		-1.39	-1.06	-1.46	-1.13	
	pta-miR319	***	1518	93	1588	156	1041	77	1084	67		-0.46	-0.42	-0.56	-0.53	
	cme-miR319c	***	2745	153	2925	98	3607	126	3154	119		0.43	0.24	0.30		-0.20
	sly-miR319	***	2994	111	3102	116	3973	176	3439	208		0.44		0.35		
	vun-miR319b	***	2071	302	2349	516	812	82	1029	284		-1.26		-1.48	-1.18	
	cme-miR319a	**	2376	161	2370	92	2808	170	2304	79		0.29		0.25		-0.29
	tcc-miR319	*	509	71	512	174	245	98	204	53			-1.23			
	ppt-miR319c	*	1998	96	2282	73	2473	71	2154	230	0.23	0.36				
miR396	vvi-miR396b_L-1R+3	***	4133	306	6118	707	6397	453	6534	445	0.60	0.66	0.69			
	gma-miR396b-3p	**	393	136	519	192	115	48	111	27		-1.65	-1.76	-2.12	-2.23	
	ptc-miR396e-3p	**	375	178	559	195	149	35	102	28				-1.85	-2.45	
	gma-miR396e	**	4923	577	6394	308	6831	475	6786	386		0.50	0.49			
	mdm-MIR396b-p5	**	464	101	915	140	795	67	802	131	1.01		0.84			
	ath-miR396b	**	1957	439	1970	766	606	136	951	394		-1.60		-1.65		
	osa-miR396g	**	1385	346	1613	536	570	94	705	243		-1.18		-1.44		
	gma-miR396h	**	4742	565	6142	448	6328	530	6601	489	0.41	0.45	0.51			
	nta-miR396a_R+1_1ss21GT	**	4372	250	6259	720	6536	692	6340	863	0.55	0.61				
	nta-MIR396b-p5_1ss19TA	*	1084	268	1884	339	737	225	1142	180				-1.30		
	mdm-miR396a	*	4998	424	6048	311	6158	505	6308	330			0.37			
	pde-miR396	*	1018	477	1858	556	567	90	918	114				-1.65		0.66

	hbr-miR396a	*	3654	428	4804	488	4765	306	4966	498			0.47			
miR408	ath-miR408	***	1130	70	490	22	2108	169	1009	65	-1.14	1.01		2.15	1.05	-1.10
	nta-miR408_R+1	***	380	26	201	28	1223	103	493	68		0.43		2.68	1.29	-1.39
	gma-miR408d	***	1336	133	648	51	2607	299	1297	124	-0.98	1.06		2.04	1.00	-1.04
	sof-miR408e	***	1700	199	731	82	3483	338	1665	298	-1.15	1.11		2.26	1.19	-1.08
	osa-miR408-3p	**	900	406	490	27	2187	109	974	51				2.20	0.99	-1.21
miR474	ptc-miR474a	***	1564	255	691	110	480	62	468	105	-1.12	-1.58	-1.67			
	ptc-miR474b	***	1618	166	762	123	513	80	512	121	-1.02	-1.53	-1.59			
	ptc-miR474c	***	1596	271	737	131	494	60	498	120	-1.05	-1.56	-1.60			
miR529	ppt-miR529g	***	792	152	797	29	402	47	525	47		-0.87		-0.92	-0.58	
	ppt-miR529d	***	507	152	527	32	227	16	251	42				-1.14	-1.05	
	osa-miR529b	***	972	161	815	121	332	70	618	112		-1.43		-1.23		0.85
	ppt-miR529a	***	508	151	524	31	222	37	238	53		-1.07		-1.17	-1.12	
	ppt-miR529e	***	931	219	968	79	529	50	607	33				-0.81	-0.65	
	bdi-miR529	**	1710	206	1357	199	1000	39	962	159		-0.68	-0.76			
	aqc-miR529	**	1434	376	1380	154	750	70	1030	112				-0.81		0.42
miR535	csi-miR535	***	581	98	501	84	233	26	351	45		-1.20	-0.66	-1.04		
	tcc-miR535	**	1240	251	1619	266	791	86	1002	172				-0.97		
	mdm-miR535b	*	1539	346	1613	206	864	70	1221	226				-0.85		
	mdm-miR535d	*	1139	356	1555	270	832	109	1264	207				-0.85		

miR858	cme-miR858	***	264	14	426	25	623	59	766	36		1.37	1.62	0.61	0.85	
	mdm-miR858	***	212	15	403	34	531	41	646	72		1.46	1.69	0.47	0.70	
	ath-miR858a	***	253	20	375	42	517	35	630	120		1.17	1.39	0.53	0.76	
miR1511	mdm-miR1511	**	2200	276	1441	110	1808	170	1544	179	-0.57					
	gma-miR1511	*	2524	484	1706	188	1397	223	1270	361		-0.77				
miR1863	cme-miR1863	***	838	146	576	80	302	40	347	45		-1.35	-1.20	-0.87	-0.71	
	osa-miR1863b	***	970	157	569	138	337	30	332	47		-1.41	-1.48			
	osa-miR1863a	***	830	153	513	122	289	25	299	40		-1.40	-1.40			
	pab-miR1863	**	753	202	722	147	371	40	415	63				-0.89		
miR5368	gma-miR5368	***	684	91	698	76	1330	114	572	73		1.06		0.98		-1.26
	gma-MIR5368-p5_1ss4TC	*	1356	6	1312	120	2100	525	1278	147						
miR6478	ptc-miR6478	**	1557	151	1730	148	1851	111	1364	68		0.33				-0.47
	ptc-miR6478_R+2_1ss21GA	*	1235	127	1474	158	1352	83	1102	122						
	nta-miR482a	*	1316	400	2958	685	1150	342	1707	147	1.21			-1.33		
	aly-miR853-3p	**	1308	263	1301	281	790	126	552	127			-1.19		-1.22	
	ath-miR854a	***	3125	403	1114	298	1010	94	1444	250	-1.46	-1.55	-1.06			
	tae-miR1134	***	831	152	186	38	369	11	777	153		-1.04			2.05	1.01
	hvu-miR1436	*	716	172	271	79	364	36	254	127	-1.34					
	ptc-miR1450	*	58298	7871	44431	5136	41633	2701	41968	2574						
	osa-miR2096-3p	**	683	166	285	82	218	40	193	87	-1.19	-1.53	-1.76			

gma-miR2108b	***	296	29	386	40	596	61	442	37		1.15		0.68		-0.49
peu-miR2916	**	3376	364	2905	269	4352	313	3650	333		0.40		0.57		
ath-miR2936	***	787	161	449	140	202	55	271	58		-1.83	-1.45			
mdm-miR3627a	***	367	103	551	114	261	49	117	28				-1.01	-2.23	
vvi-miR3630-3p	***	631	44	638	41	1442	136	968	140		1.29	0.66	1.22	0.59	-0.63
csi-miR3946	***	3151	541	2652	452	1311	85	1903	203		-1.21	-0.69	-1.00		0.52
osa-miR3979-3p	*	1206	223	823	239	476	63	472	198		-1.22				
gma-miR4403	***	1155	235	469	82	685	70	471	90	-1.24		-1.21			
gma-miR4995	*	2017	296	1968	286	2769	244	1734	218						-0.69
ath-miR5021	***	1490	242	367	117	486	56	1004	99	-1.95	-1.50			1.45	1.00
hvu-miR5049c	**	515	125	420	146	216	49	123	54		-1.13	-1.99		-1.77	
rgl-miR5139	**	5346	484	3634	461	4392	386	3341	433	-0.52		-0.64			
mtr-miR5205b	*	1225	304	670	212	644	46	445	158						
osa-miR5493	*	965	299	356	49	684	146	472	137				0.98		
osa-miR5819	*	1195	398	716	258	345	65	413	140		-1.68				
nta-miR6149a	**	408	48	547	51	663	39	565	74	0.48	0.82				
hbr-miR6173	**	3981	664	4735	614	5065	421	3122	360					-0.60	-0.69
PC-3p-603_509	***	433	9	632	13	813	58	537	41		1.04	0.38	0.44		-0.66
PC-5p-1245_480	***	513	15	550	16	764	71	784	68	0.16	0.68	0.66	0.53	0.51	
PC-5p-283_1050	***	1676	124	1306	97	2520	142	2051	302	-0.32	0.66		0.98	0.66	

PC-5p-314691_3	***	5112	1339	8436	1083	3281	494	2742	447			-1.37	-1.62
PC-5p-19256_60	***	377	28	399	14	564	57	557	50	0.69	0.63	0.55	0.49
PC-3p-5560_85	***	386	8	418	24	590	65	528	49	0.73	0.53	0.55	

Note: *** P<0.0001; ** 0.0001<p<0.001; * 0.001<p<0.01
