# Supplementary material for: Identification and characterization of cold-responsive microRNAs in tea plant (Camellia sinensis) and their targets using high-throughput sequencing and degradome analysis
Source: BMC Plant Biol. 2014 Oct 21;14:271. doi: 10.1186/s12870-014-0271-x (PMC4209041; doi:10.1186/s12870-014-0271-x)
Supplement: Additional file 7: Table S4. — Relative expression analysis of cold-responsive miRNAs in ‘Baiye 1’. The threshold of p value was set 0.01, ***indicated p value less than 0.0001, ** indicated p value from 0.0001 to 0.001, *indicated p value from 0.001 to 0.01, respectively. [file 12870_2014_271_MOESM7_ESM.pdf]

Table S4 Relative expression analysis of cold-responsive miRNAs in ‘Baiye 1’

miRNA Family	miRNA Probes	p	BY-CK		BY-4h		BY-12h		BY-24		Relative fold difference (Log2)					
			Mean	StDev	Mean	StDev	Mean	StDev	Mean	StDev	4h/ CK	12h/ CK	24h/ CK	12h/ 4h	24h/ 4h	24h/ 12h
miR156	bdi-miR156a	***	2067	246	2551	72	1305	64	1832	112		-0.61		-0.91	-0.44	0.47
	far-miR156b	***	5022	254	6014	464	4414	21	5046	222	0.28			-0.42		
	vvi-miR156h	***	2104	151	2524	295	1662	73	1988	126		-0.29		-0.56		0.24
	cme-miR156g	**	1043	113	1407	121	1053	96	1294	78	0.44					
	osa-miR156c-3p	*	776	128	934	753	1843	144	2950	144		1.31	1.97			0.67
	ath-miR156j	*	4917	266	5665	952	4054	235	5128	366		-0.23				0.32
miR159	osa-miR159c	***	3928	223	3233	149	2999	86	2821	167	-0.27	-0.34	-0.44			
	osa-miR159d	***	3990	225	3461	206	3194	100	3071	102		-0.27	-0.34			
	osa-miR159e	**	3742	376	3271	204	3060	61	2858	89					-0.12	
	osa-miR159f	*	4451	211	3864	468	3811	126	3563	111		-0.17	-0.29			
	zma-miR159e-3p	***	1913	231	1836	156	1438	70	1316	77			-0.51		-0.45	
	zma-miR159g-3p	**	429	9	565	128	285	44	465	50				-0.94		
	pta-miR159c	**	2302	353	2089	126	1985	67	1592	72				-0.35	-0.33	
	pta-miR159b	*	2487	275	2409	108	2288	112	2017	76					-0.22	-0.20
	sbi-miR159b	**	3768	320	3356	372	3115	149	2773	81			-0.41		-0.18	
	aly-miR159c-3p_R-1_1ss8TC	*	2938	461	2676	142	2495	157	2183	118					-0.26	

miR166	hbr-miR166b	***	866	128	962	69	418	45	281	34		-1.01	-1.66	-1.15	-1.79	
	hbr-miR166a	**	19643	784	21723	1592	24923	1016	23005	1013		0.40	0.23			
	cme-miR166i	*	16427	1332	17738	781	19802	690	18172	806				0.20		-0.17
	sbi-miR166a	**	18545	1231	19051	1959	16010	301	14363	650			-0.36		-0.41	
	bdi-miR166e	***	23046	1829	23633	967	22004	977	33931	1480			0.56		0.52	0.57
	bdi-miR166f	***	22810	1870	23416	1927	24129	1110	31042	1293			0.45		0.40	0.31
	ath-miR166a	***	14762	1056	16578	910	12625	300	10682	999			-0.45	-0.56	-0.63	
	gma-miR166m	***	12506	488	13275	567	11980	611	9633	531			-0.36		-0.46	-0.35
	gma-miR166u	***	18129	1119	20015	809	22018	767	25012	1413		0.33	0.47	0.18	0.32	
	osa-miR166k-3p	***	10655	691	10141	446	8370	418	7850	122		-0.30	-0.42	-0.24	-0.37	
	osa-miR166e-3p	***	8593	464	8344	1266	6700	447	5119	164		-0.31	-0.73		-0.70	-0.42
	osa-miR166i-3p_2ss6TC14CT	*	11040	950	10755	632	10572	665	8838	388					-0.28	-0.29
	osa-miR166m	*	16077	1305	14996	2053	13228	685	12506	369						
	ppt-miR166m	*	10804	1270	11170	1174	9283	381	8820	352						
	ppt-miR166j	***	12345	924	12522	1765	11241	750	8400	333			-0.54			-0.46
	vvi-MIR166b-p3	***	14743	909	16207	991	16576	514	13208	619					-0.30	-0.37
	csi-miR166a-5p_R+1	**	528	91	617	117	378	6	358	33					-0.81	
	zma-miR166n-5p	**	1260	136	2044	805	1796	192	4058	618		0.58	1.73		1.15	
miR167	ath-miR167d	***	1879	132	2345	129	1469	69	2009	70	0.32	-0.30		-0.62		0.44
	ath-miR167c	***	1677	154	2122	86	1387	58	1861	113	0.35			-0.56		0.41

	bna-miR167a	**	1500	108	1656	225	1175	30	1554	83					0.38	
	osa-miR167d-5p	***	1814	168	1992	174	1497	70	2246	72				-0.36		0.57
	ptc-miR167h-5p	***	2450	132	2780	195	2166	71	2825	106			0.24	-0.32		0.37
	cme-miR167c	***	2119	115	2452	208	1794	60	2297	112				-0.40		0.34
	mdm-miR167h	**	1480	77	1471	110	1190	21	1579	117		-0.25				0.39
	ppt-miR167	*	1855	98	2082	258	1659	95	2095	75			0.22			0.32
	vvi-miR167d_R+1	*	1550	126	1658	124	1424	61	1764	84						0.30
miR168	cme-miR168	***	732	85	823	83	1264	69	1442	52		0.85	1.00	0.68	0.83	
	aly-miR168a-3p	*	570	65	457	205	793	84	1039	52		0.53	0.86			
	gma-miR168b	***	672	59	832	118	1327	69	1451	97		1.04	1.13	0.73	0.82	
	osa-miR168b	*	346	54	301	130	556	68	667	44		0.73	0.91			
	nta-MIR168a-p3	***	605	27	716	104	947	16	958	57		0.70	0.66			
	nta-miR168a	***	654	56	805	212	1278	76	1459	85		1.03	1.17			
	bdi-miR168	***	559	38	496	186	1362	142	1175	62		1.35	1.10			
	aqc-miR168	**	193	21	233	131	304	25	1261	113			2.63			1.99
miR171	cca-miR171	***	2505	120	1620	36	1598	55	983	72	-0.62	-0.58	-1.33		-0.71	-0.75
	mtr-miR171c	***	2527	149	1824	166	1662	71	1015	12	-0.47	-0.53	-1.29		-0.83	-0.76
	mtr-miR171a	***	2125	95	1504	224	1557	50	963	36		-0.38	-1.13			-0.75
	gma-miR171a	***	1523	133	1159	206	1069	50	639	41		-0.45	-1.27		-0.89	-0.82
	bna-miR171g	***	1556	123	1092	187	1099	66	626	6		-0.44	-1.33		-0.83	-0.89

	ptc-miR171k	***	1155	48	849	142	922	34	560	64		-0.27	-1.07			-0.81
	ptc-miR171j	***	636	61	470	88	543	24	309	17			-1.08			-0.90
	htu-miR171a	***	2420	74	1443	164	1637	59	949	100			-1.33		-0.60	-0.84
	bdi-miR171b	***	2522	208	1852	221	1714	39	1169	24	-0.44	-0.49	-1.08		-0.64	-0.59
	aqc-miR171f	***	2364	130	1747	108	1692	43	1082	89	-0.43	-0.41	-1.10		-0.67	-0.69
	ath-miR171b	***	2061	172	1517	140	1312	72	845	43	-0.43	-0.58	-1.28		-0.85	-0.70
	ath-miR171a	***	1632	121	1162	166	1059	57	669	23	-0.56	-1.30			-0.82	-0.74
	zma-miR171c-3p	***	2369	235	1685	191	1662	126	1070	87	-0.49	-0.44	-1.12		-0.63	-0.68
	zma-miR171a-3p	**	1144	142	775	165	882	43	567	32			-1.05			-0.73
	zma-miR171f-3p	*	1864	201	1060	454	1178	110	758	63		-0.60	-1.29			-0.70
	csi-miR171a	**	700	105	572	117	651	27	390	28			-0.89		-0.83	
	smo-miR171b	***	1268	96	837	213	891	50	491	28		-0.45	-1.41			-0.95
miR319	gma-miR319c	***	618	73	688	71	469	45	422	39			-0.59	-0.49	-0.73	
	gma-miR319n	**	407	120	592	234	894	120	1356	73		1.17	1.70			
	ppt-miR319c	*	2383	420	2256	221	1988	127	1621	113					-0.44	-0.31
	cme-miR319c	*	2801	359	2579	231	2517	123	2152	101						-0.24
	cme-miR319a	*	2429	354	2290	295	2059	75	1707	66						-0.28
	sly-miR319	*	3133	317	2838	302	2823	133	2307	216			-0.41			
miR396	nta-MIR396b-p5_1ss19TA	***	2486	197	2097	93	1466	94	1740	105		-0.70	-0.48	-0.45	-0.24	
	gma-miR396b-3p	**	138	47	265	162	366	56	1230	117			3.08		1.68	

	gma-miR396a-3p	***	87	23	88	34	177	33	658	103			2.80		2.86	1.82
	gma-miR396e	**	5712	393	4344	418	4136	233	5279	378	-0.38	-0.42				0.33
	gma-miR396h	*	5375	380	4140	575	4322	236	5379	325						0.29
	aly-miR396a-3p	***	113	15	130	41	148	41	515	45			2.09		1.97	1.73
	mdm-MIR396b-p5	***	1193	150	1187	170	685	27	980	54		-0.74		-0.74		0.45
	mdm-miR396a	*	5404	409	3990	785	4150	148	5112	240		-0.33				0.28
	sbi-miR396e	**	5520	560	4537	564	3992	179	5111	127		-0.42			0.34	
	sbi-miR396d	*	5242	609	4279	873	3602	344	4951	147		-0.49				0.44
	ptc-miR396e-3p	**	176	44	281	172	398	76	1171	103		2.67			1.49	
	vvi-miR396b_L-1R+3	*	5537	443	4492	335	4517	246	5279	292						
	hbr-miR396a	*	4829	250	4099	380	3944	109	4670	259		-0.24			0.22	
	osa-miR396g	*	3242	289	2721	610	2088	92	3020	194		-0.58				0.51
miR474	ptc-miR474a	*	372	40	415	249	1632	193	1132	91		2.20	1.61			-0.59
	ptc-miR474b	*	362	53	446	267	1629	202	1274	69		2.23	1.82			
	ptc-miR474c	*	363	39	437	264	1568	191	1200	132		2.17	1.72			
miR482	pde-miR482c	***	1153	419	1316	332	1349	125	3551	347			1.66		1.46	1.37
	nta-miR482a	**	2477	265	4021	563	3211	196	3324	260	0.71	0.42	0.46			
miR529	aqc-miR529	***	1197	123	1646	103	799	40	1146	74	0.46	-0.52		-0.98	-0.51	0.47
	bdi-miR529	**	1255	140	1351	240	862	22	1262	48					0.51	
	ppt-miR529a	***	542	141	612	177	239	9	316	22		-1.15		-1.30		

	ppt-miR529d	***	525	131	623	156	247	5	307	25		-1.06		-1.29		
	ppt-miR529e	***	1004	181	1232	167	475	12	576	23		-1.03	-0.83	-1.32	-1.13	0.20
	ppt-miR529g	***	780	187	1022	181	420	29	530	35				-1.22	-0.97	
	osa-miR529b	***	1155	241	1705	440	377	43	626	49		-1.57		-2.13	-1.47	0.66
miR535	osa-miR535-5p	***	1972	182	2334	170	2917	54	2721	77		0.61	0.50	0.37		-0.12
	csi-miR535	**	620	46	888	188	473	50	785	15						0.65
	tcc-miR535	**	541	45	795	261	1342	48	1191	100		1.37	1.15			
	aqc-miR535	**	1456	155	1916	304	2588	80	2346	468		0.89				
miR1863	cme-miR1863	***	702	32	537	54	539	24	220	12	-0.39	-0.32	-1.72		-1.33	-1.40
	osa-miR1863b	***	728	60	572	53	580	25	237	23			-1.66		-1.30	-1.39
	osa-miR1863a	***	654	71	523	77	484	18	223	12			-1.60		-1.26	-1.22
	pab-miR1863	***	793	103	661	135	657	154	306	34			-1.44		-1.16	-1.22
miR6478	ptc-miR6478	***	755	44	816	162	1472	329	1467	86			0.99		0.86	
	ptc-miR6478_R+2_1ss21GA	**	676	80	711	186	1143	57	1024	55		0.82	0.62			
	ath-miR165a	***	11215	981	12140	957	9374	462	8012	841			-0.47	-0.34	-0.60	
	ath-miR170	***	2000	241	1472	262	1369	55	938	54			-1.09			-0.61
	aau-MIR172-p3_1ss1AT	**	4344	247	5773	529	5066	241	4637	176	0.43	0.27				
	vvi-miR394a	**	2463	271	2421	215	1987	110	1695	123			-0.51		-0.48	
	aly-miR853-3p	**	777	128	1072	300	782	110	1650	110			1.10			1.04
	ath-miR854a	***	1548	213	1892	370	2302	260	3549	313		0.63	1.24		0.95	0.60

tae-miR1134	*	575	41	799	119	642	31	654	34					
cre-miR1144b	***	455	75	547	182	707	46	2178	89		2.26		2.01	1.57
cre-miR1155	*	1978	248	2184	1029	4610	80	3964	78	1.27	1.03			-0.23
cre-miR1160.3	***	259	44	221	102	901	68	1405	75	1.86	2.43		2.69	0.57
pde-MIR1310-p5_1ss18TC	***	320	43	371	181	1601	147	1567	135	2.38	2.31			
hvu-miR1436	*	284	55	204	229	898	160	1342	154	1.71	2.23			
ptc-miR1450	***	42291	2508	45018	4986	52088	3226	74364	1429	0.36	0.80		0.71	0.45
mdm-miR1511	*	954	143	717	269	1213	62	570	49		-0.74			-1.15
osa-miR1881	*	824	87	894	188	1150	61	1196	69	0.54	0.54			
osa-miR2096-3p	*	179	27	216	206	971	128	860	51	2.48	2.21			
mtr-miR2670f	*	42	23	57	41	133	30	517	90		3.51			1.87
peu-miR2916	***	2158	109	2356	225	2858	201	1921	94	0.46				-0.59
osa-miR2919	***	294	35	317	28	386	67	550	43		0.86		0.78	
ath-miR2936	***	744	106	1028	107	396	68	725	73	-0.86		-1.32	-0.54	0.79
ahy-miR3509-3p	*	505	122	1423	847	2479	161	2979	195	2.37	2.57			
mdm-miR3627a	*	259	47	224	105	430	95	638	28		1.24			
vvi-miR3630-3p	*	481	35	294	94	477	18	290	33					
vvi-miR3633a-3p	***	853	97	904	61	396	21	256	32	-1.07	-1.78	-1.13	-1.84	
csi-miR3946	***	3587	110	4224	418	2485	109	3516	196	-0.49		-0.73		0.49
gma-miR4403	***	445	30	622	184	350	21	988	43		1.13			1.43

gma-miR4993	***	169	34	287	146	1140	118	2121	284		2.82	3.66			0.84
gma-miR4995	***	688	63	785	166	1385	67	1732	197		1.07	1.38		1.18	
ath-miR5021	**	934	70	1152	113	669	92	886	92				-0.72		
hvu-miR5049c	**	217	60	185	140	324	70	1079	120			2.26			1.67
osa-miR5072_L-3_1ss4TA	***	336	24	309	108	973	33	554	36		1.60	0.70			-0.91
osa-miR5076	***	13	5	20	8	411	28	516	38			5.15		4.63	
osa-miR5077	**	3731	302	3337	645	5147	177	3321	117		0.52				-0.65
mtr-miR5205a	**	59	29	95	63	399	125	972	159		2.73	3.95			1.22
osa-miR5493	***	8452	2084	13884	3545	1163	146	4995	199		-2.83		-3.55	-1.47	2.08
mtr-miR5563-5p	*	166	38	276	203	105	19	1324	107			2.91			3.59
gma-miR5783	**	524	249	1251	521	2022	356	2517	303			2.30			
osa-miR5819	***	848	305	1174	599	3547	244	3795	267		2.13	2.20			
hbr-miR6173	***	1946	104	2159	278	2337	102	3397	206		0.32	0.84		0.68	0.52
hvu-miR6186	***	277	75	382	44	370	14	679	87			1.26		0.82	0.80
gma-miR6300	***	3536	300	4705	477	7203	194	4571	412	0.43	1.08	0.40	0.65		-0.68
PC-3p-727746_2	***	182	18	244	20	458	56	515	61			1.44		1.05	
PC-3p-1242_293	***	2342	309	2649	362	4526	110	2725	138		1.00		0.81		-0.75
PC-3p-1887_198	**	2142	172	2240	455	3623	131	2273	166		0.81				-0.69
PC-3p-5560_85	**	572	35	310	88	487	28	323	53			-0.86			
PC-3p-518188_2	**	80	16	94	68	652	206	1184	73		3.00	3.79			

PC-5p-19256_60	***	645	42	418	42	506	32	344	37			-0.95	0.35		-0.65
PC-5p-314691_3	***	4663	183	3713	581	6073	272	7130	577		0.43	0.64	0.75	0.95	
PC-5p-431_684	***	3763	266	4087	551	6651	330	4180	469		0.88		0.74		-0.69
PC-5p-1245_480	***	814	33	520	67	691	53	445	70	-0.65		-0.90			-0.72

Note: *** P<0.0001; ** 0.0001<p<0.001; * 0.001<p<0.01
