# Supplementary material for: Identification and characterization of cold-responsive microRNAs in tea plant (Camellia sinensis) and their targets using high-throughput sequencing and degradome analysis
Source: BMC Plant Biol. 2014 Oct 21;14:271. doi: 10.1186/s12870-014-0271-x (PMC4209041; doi:10.1186/s12870-014-0271-x)
Supplement: Additional file 8: Figure S4. — Microarray analysis of the known and new miRNAs from tea plant cultivar ‘Yingshaung’ treated with cold and cold-free. [file 12870_2014_271_MOESM8_ESM.pdf]

ahy−MIR156a−p5_1ss2GA slicing gi212380427 at nt 564

alignment score=4 , category=2 , p=0.999999999706044

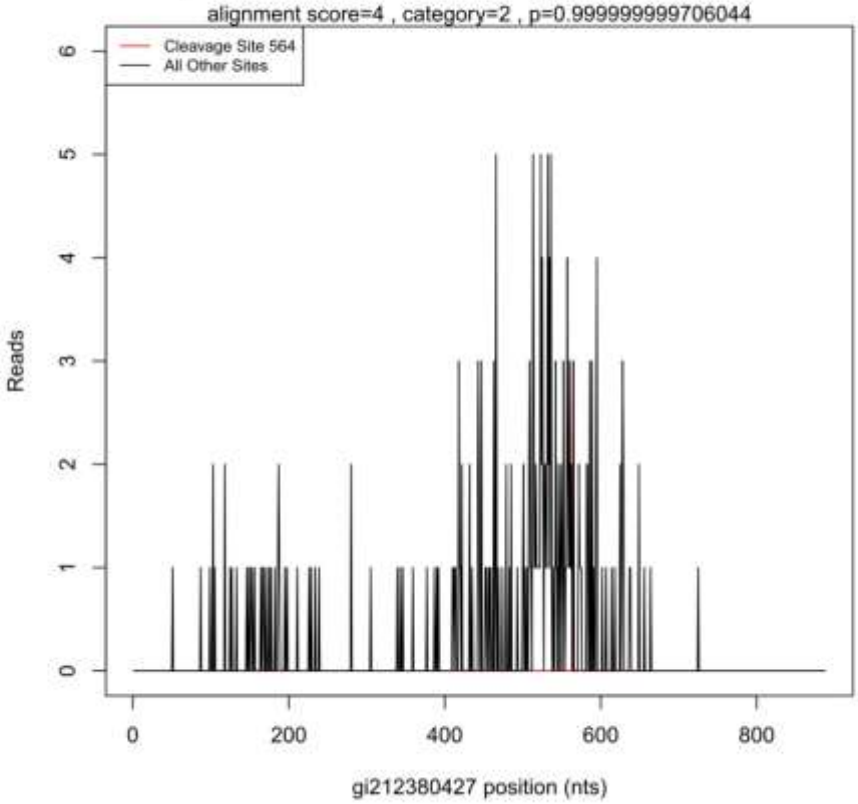

ahy−MIR156a−p5_1ss2GA slicing gi212380702 at nt 194

alignment score=4 , category=2 , p=0.999999999706044

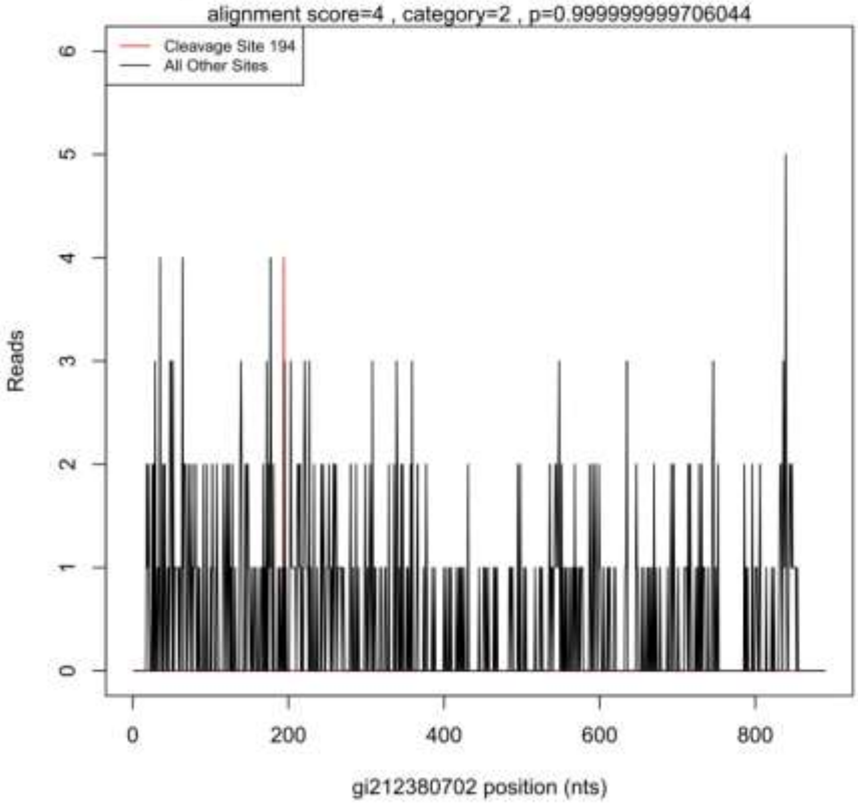

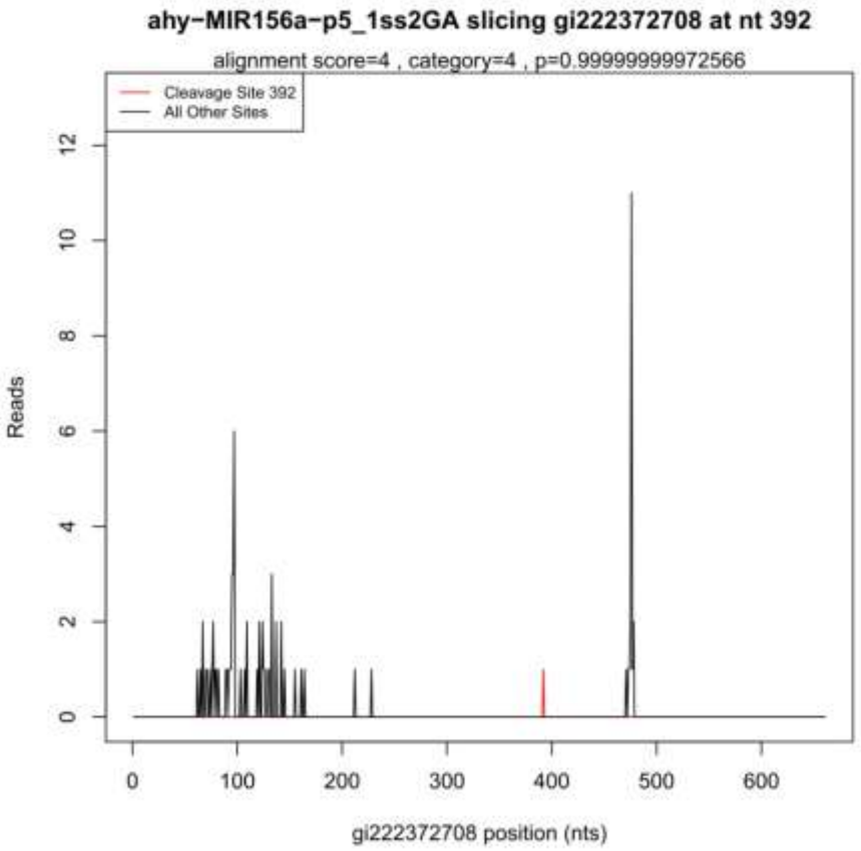
ahy−MIR156a−p5_1ss2GA slicing gi222372708 at nt 392
alignment score=4 , category=4 , p=0.99999999972566
Cleavage Site 392
All Other Sites
Reads
0
2
4
6
8
10
12
0
100
200
300
400
500
600
gi222372708 position (nts)

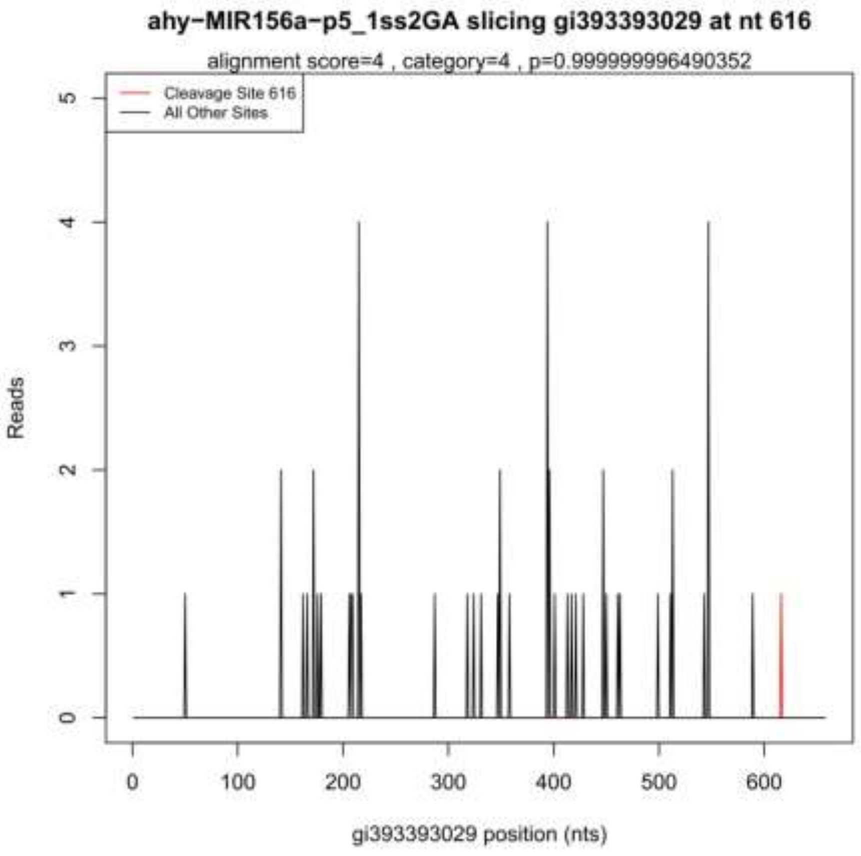
ahy−MIR156a−p5_1ss2GA slicing gi393393029 at nt 616
alignment score=4 , category=4 , p=0.999999996490352
Cleavage Site 616
All Other Sites
Reads
0
1
2
3
4
5
0
100
200
300
400
500
600
gi393393029 position (nts)

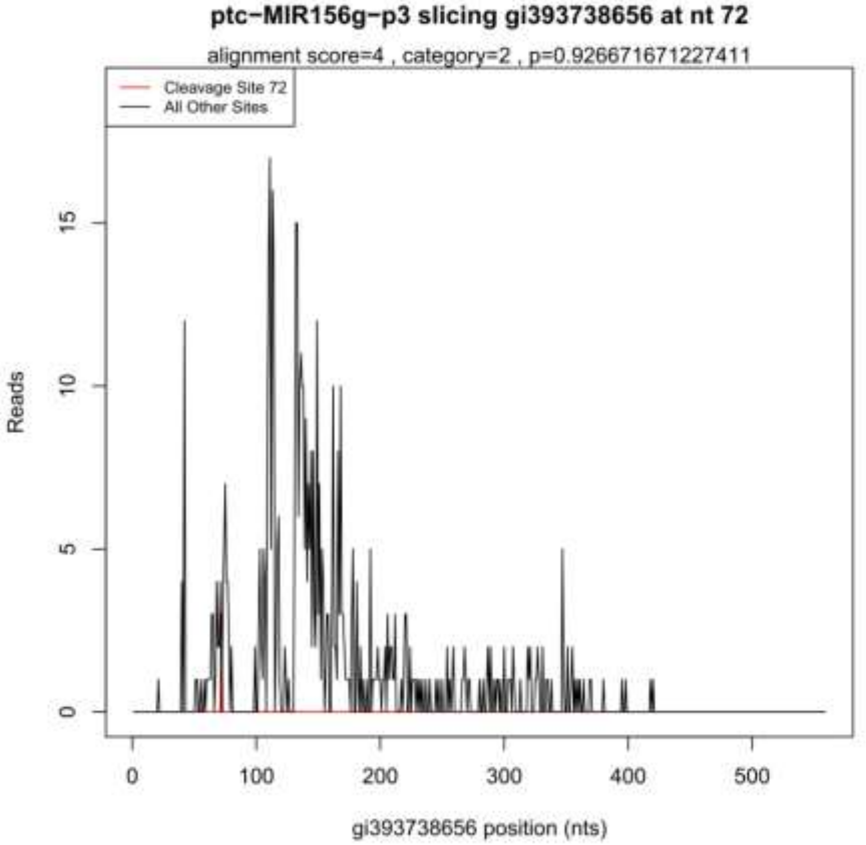
ptc−MIR156g−p3 slicing gi393738656 at nt 72
alignment score=4 , category=2 , p=0.926671671227411
Cleavage Site 72
All Other Sites
Reads
gi393738656 position (nts)

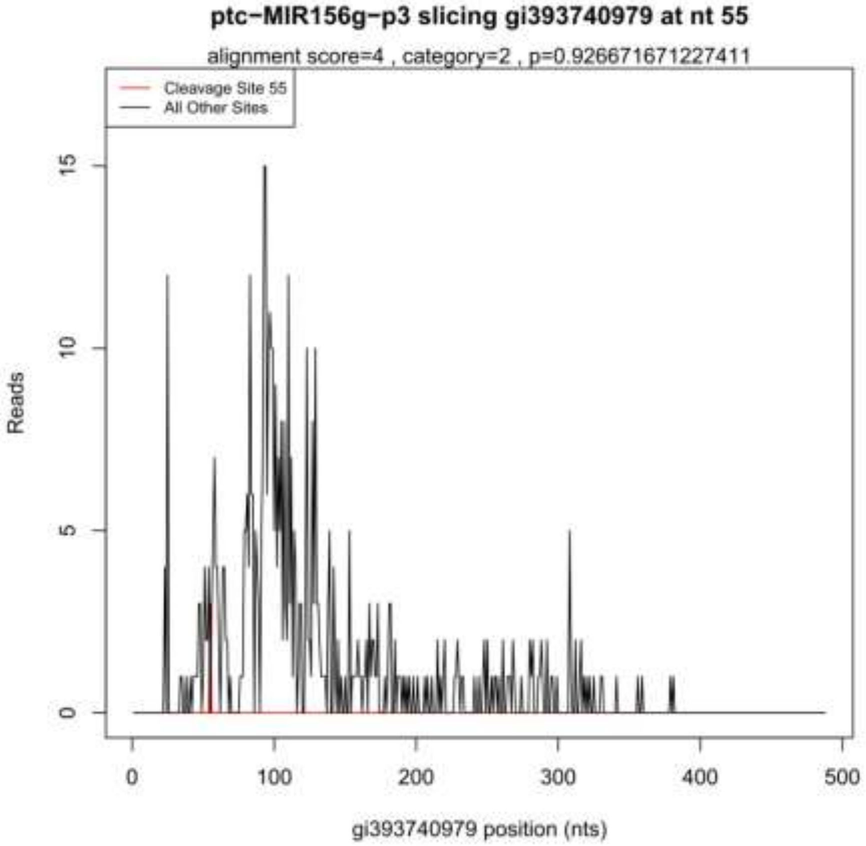
ptc−MIR156g−p3 slicing gi393740979 at nt 55
alignment score=4 , category=2 , p=0.926671671227411
Cleavage Site 55
All Other Sites
Reads
gi393740979 position (nts)

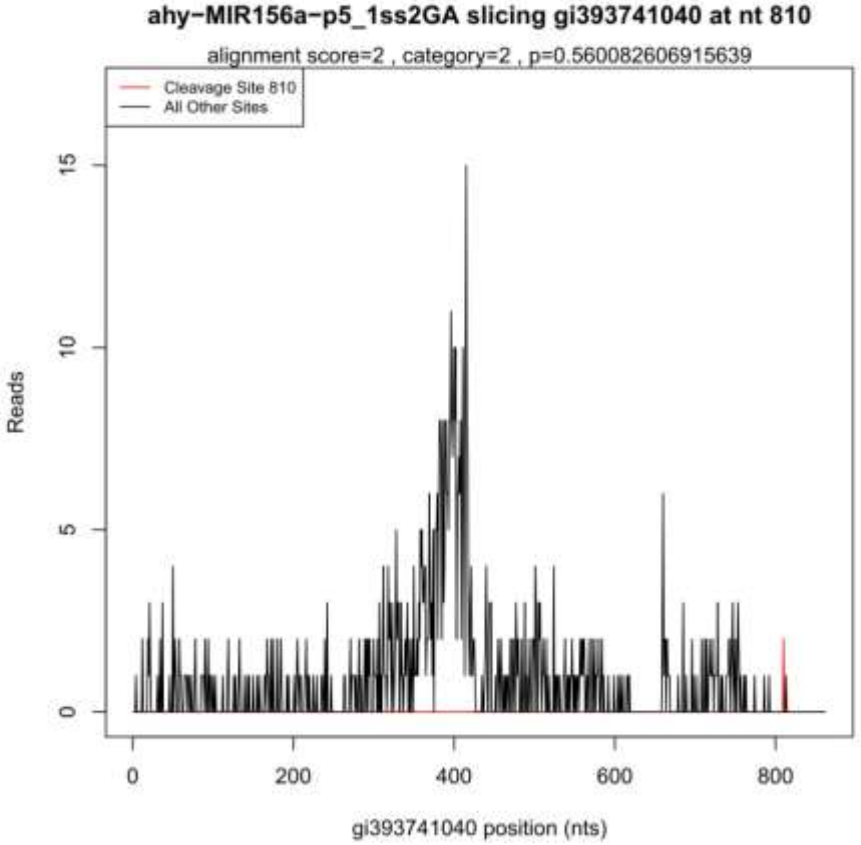
ahy−MIR156a−p5_1ss2GA slicing gi393741040 at nt 810
alignment score=2 , category=2 , p=0.560082606915639
Cleavage Site 810
All Other Sites
Reads
0
5
10
15
0
200
400
600
800
gi393741040 position (nts)

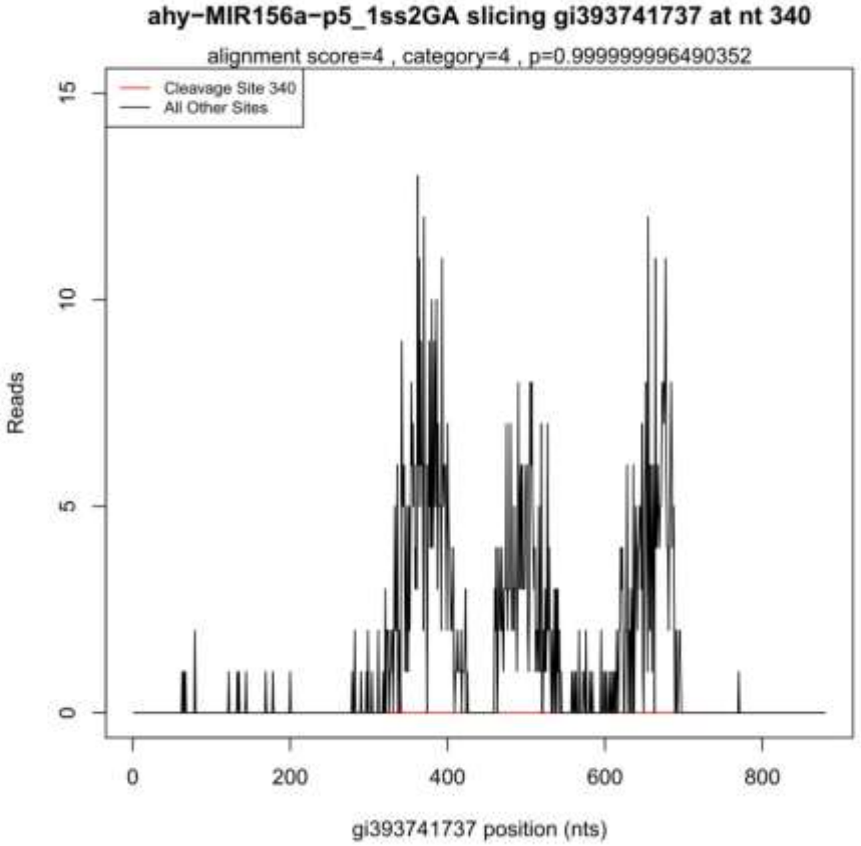
ahy−MIR156a−p5_1ss2GA slicing gi393741737 at nt 340
alignment score=4 , category=4 , p=0.999999996490352
Cleavage Site 340
All Other Sites
Reads
0
5
10
15
0
200
400
600
800
gi393741737 position (nts)

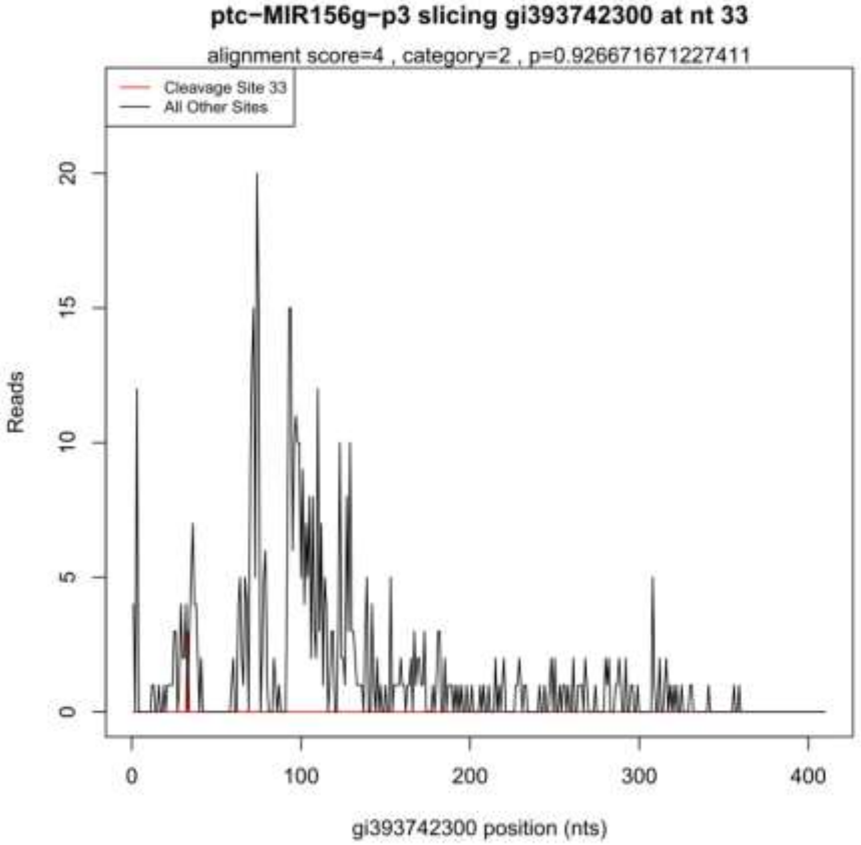
ptc−MIR156g−p3 slicing gi393742300 at nt 33
alignment score=4 , category=2 , p=0.926671671227411
Cleavage Site 33
All Other Sites
Reads
0
5
10
15
20
0
100
200
300
400
gi393742300 position (nts)

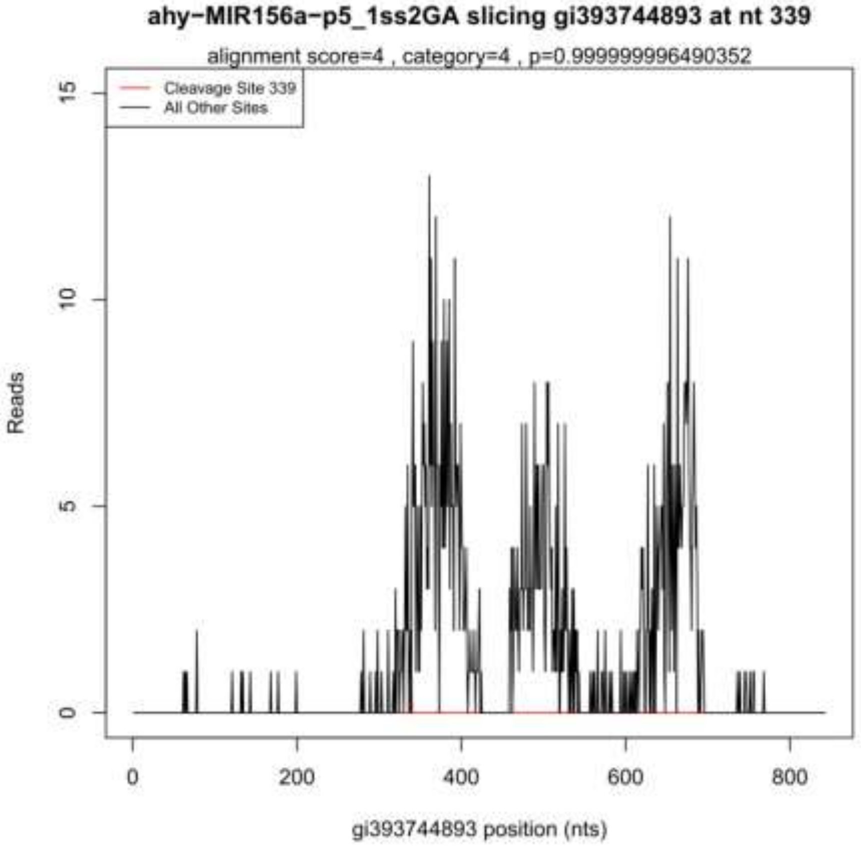
ahy−MIR156a−p5_1ss2GA slicing gi393744893 at nt 339
alignment score=4 , category=4 , p=0.999999996490352
Cleavage Site 339
All Other Sites
Reads
0
5
10
15
0
200
400
600
800
gi393744893 position (nts)

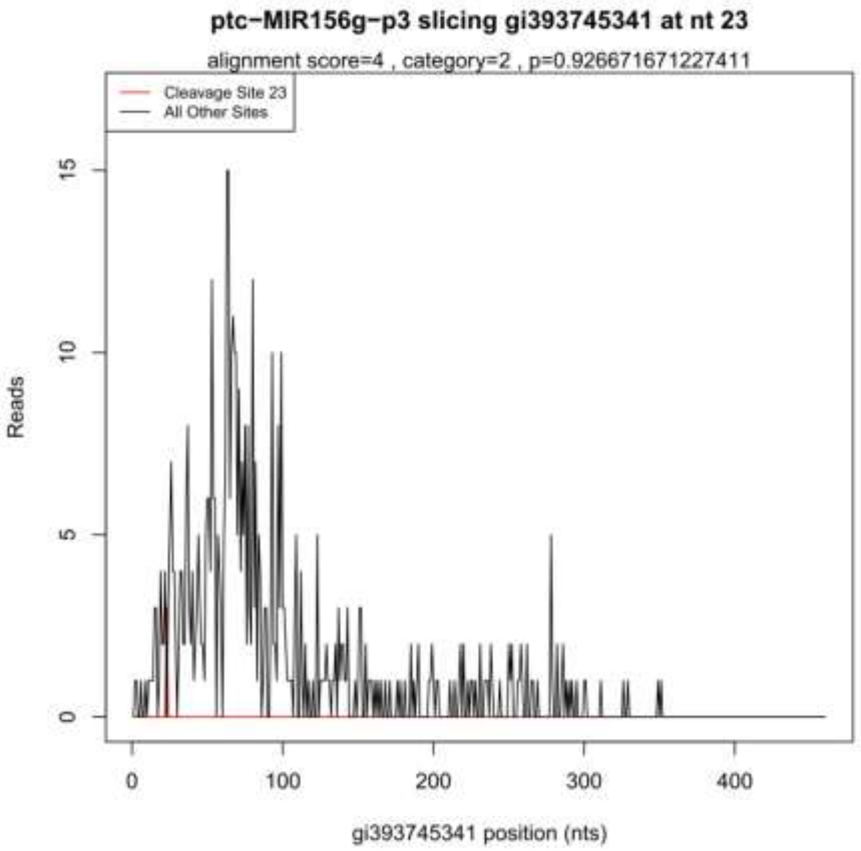
ptc−MIR156g−p3 slicing gi393745341 at nt 23
alignment score=4 , category=2 , p=0.926671671227411
Cleavage Site 23
All Other Sites
Reads
0
5
10
15
0
100
200
300
400
gi393745341 position (nts)

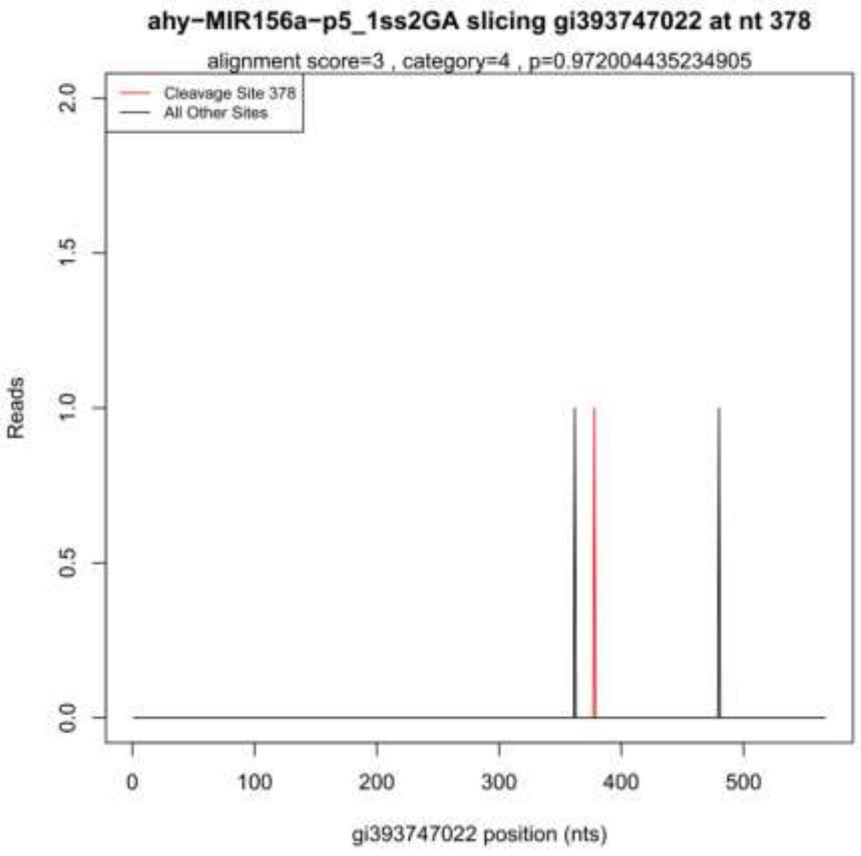
ahy−MIR156a−p5_1ss2GA slicing gi393747022 at nt 378
alignment score=3 , category=4 , p=0.972004435234905
Cleavage Site 378
All Other Sites
Reads
0.0
0.5
1.0
1.5
2.0
0
100
200
300
400
500
gi393747022 position (nts)

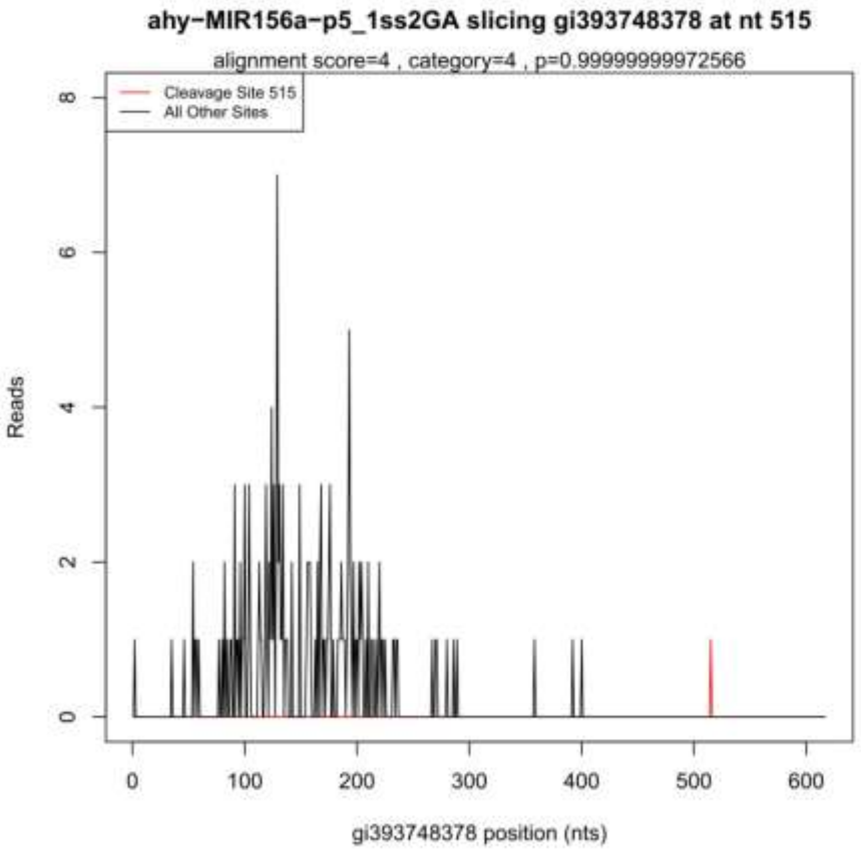
ahy−MIR156a−p5_1ss2GA slicing gi393748378 at nt 515
alignment score=4 , category=4 , p=0.99999999972566
Cleavage Site 515
All Other Sites
Reads
0
2
4
6
8
0
100
200
300
400
500
600
gi393748378 position (nts)

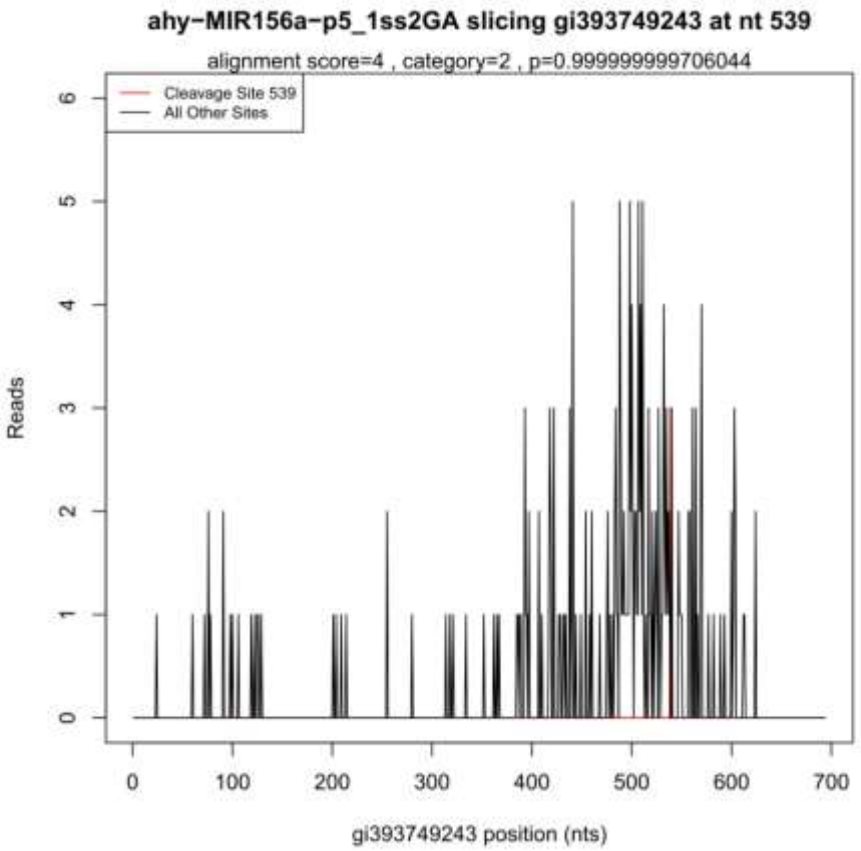
ahy−MIR156a−p5_1ss2GA slicing gi393749243 at nt 539
alignment score=4 , category=2 , p=0.999999999706044
Cleavage Site 539
All Other Sites
Reads
0
1
2
3
4
5
6
0
100
200
300
400
500
600
700
gi393749243 position (nts)

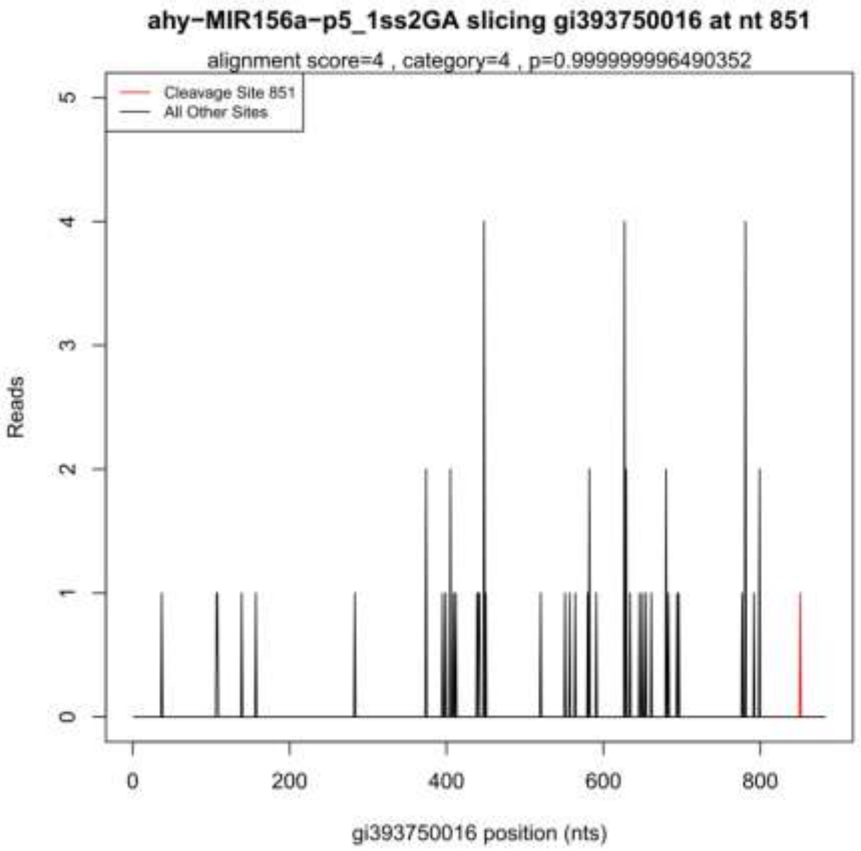
ahy−MIR156a−p5_1ss2GA slicing gi393750016 at nt 851
alignment score=4 , category=4 , p=0.999999996490352
Cleavage Site 851
All Other Sites
Reads
0
1
2
3
4
5
0
200
400
600
800
gi393750016 position (nts)

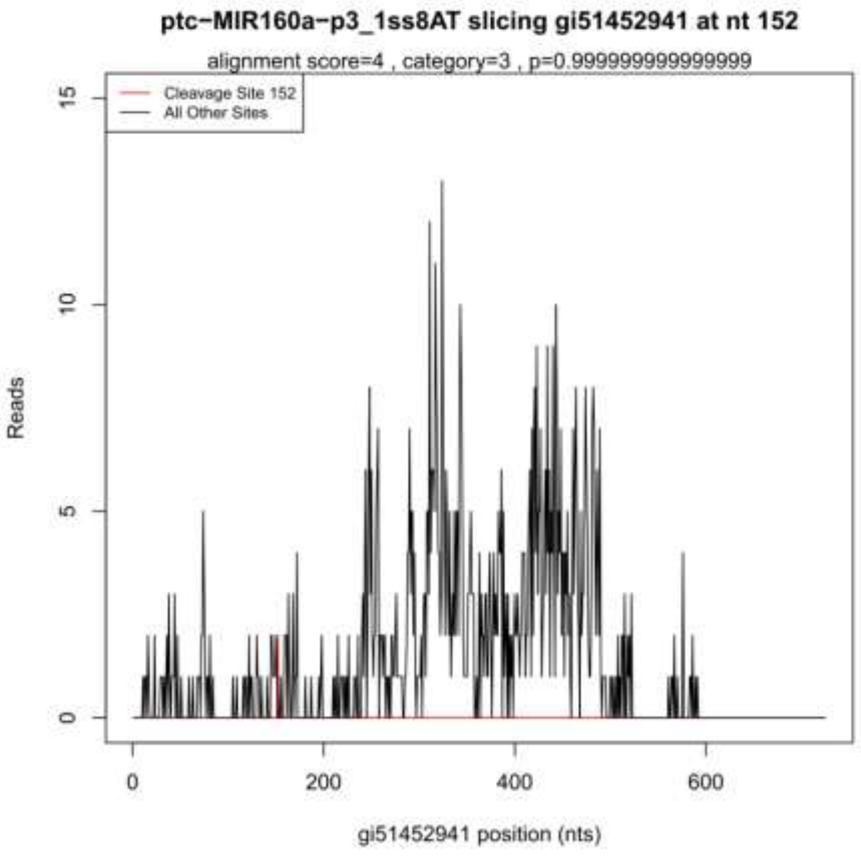
ptc−MIR160a−p3_1ss8AT slicing gi51452941 at nt 152
alignment score=4 , category=3 , p=0.999999999999999
Cleavage Site 152
All Other Sites
Reads
0
5
10
15
0
200
400
600
gi51452941 position (nts)

ptc−MIR160a−p3_1ss8AT slicing gi51453910 at nt 15

alignment score=4 , category=4 , p=1

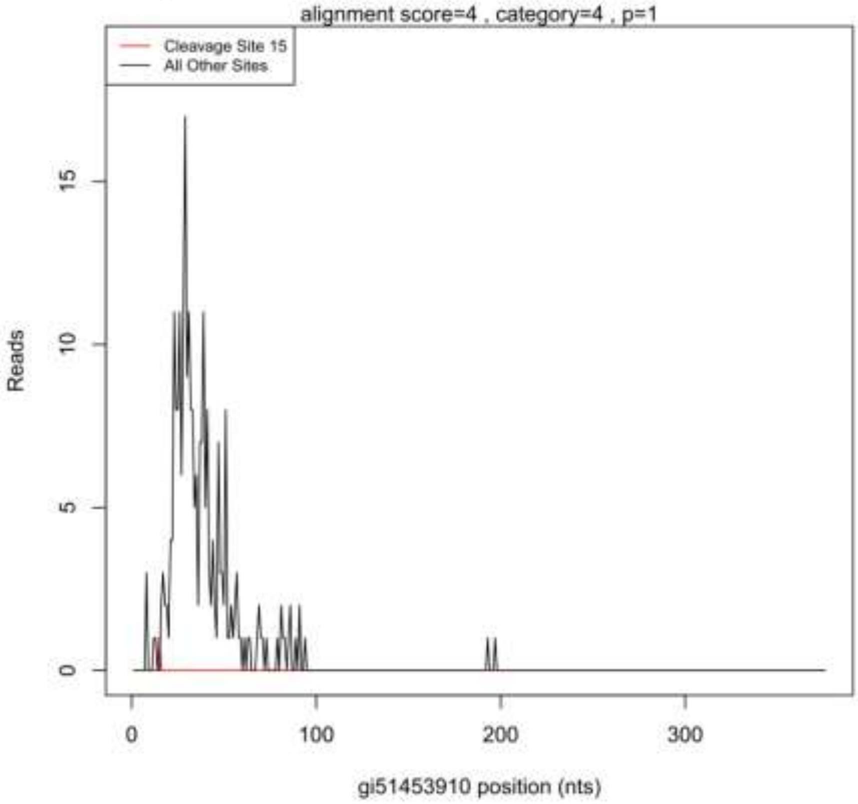

ptc−MIR160a−p3_1ss8AT slicing gi51454085 at nt 76

alignment score=4 , category=4 , p=1

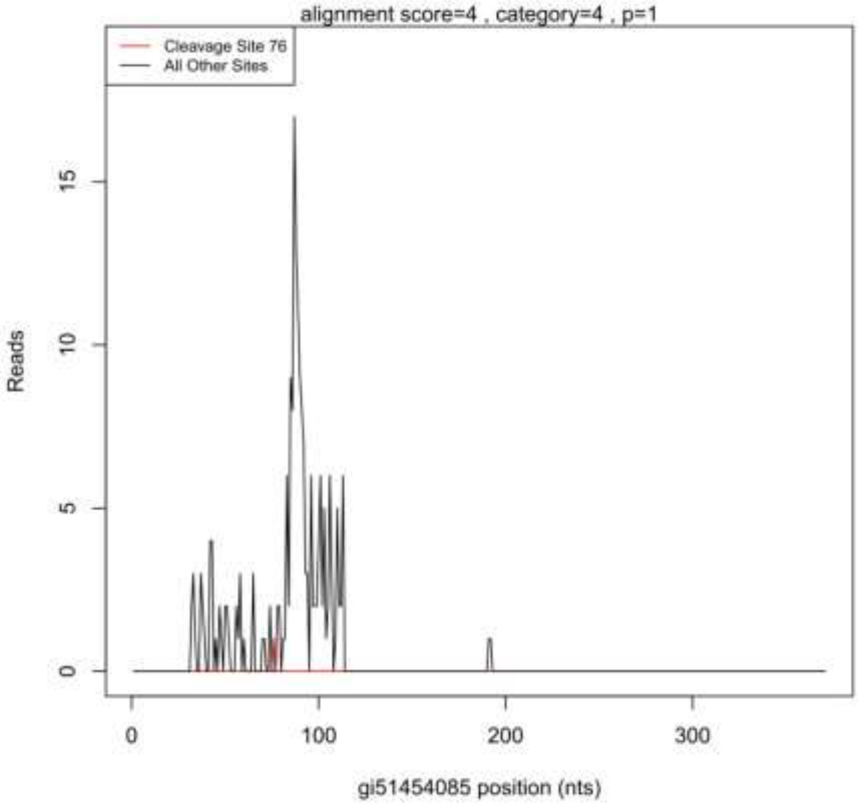

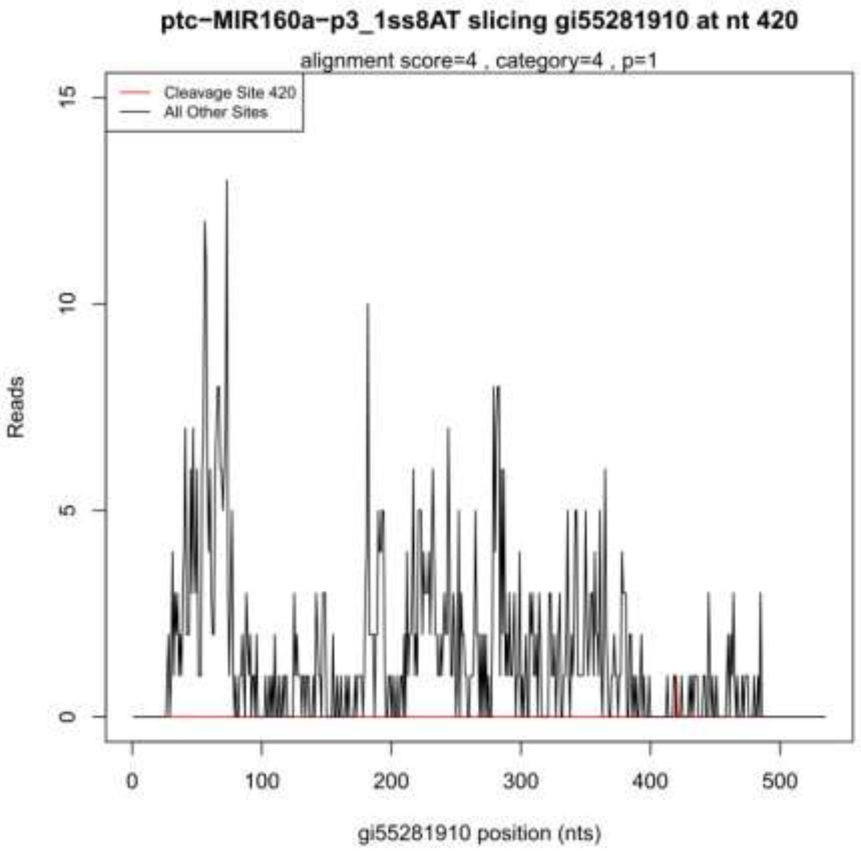
ptc−MIR160a−p3_1ss8AT slicing gi55281910 at nt 420
alignment score=4 , category=4 , p=1
Cleavage Site 420
All Other Sites
Reads
0
5
10
15
0
100
200
300
400
500
gi55281910 position (nts)

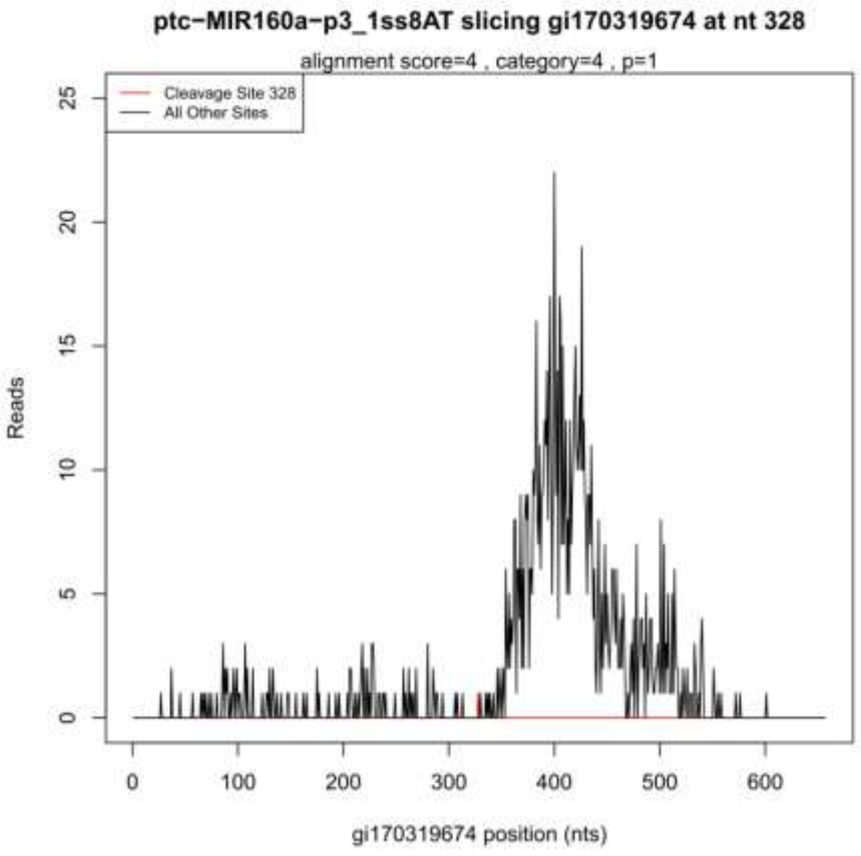
ptc−MIR160a−p3_1ss8AT slicing gi170319674 at nt 328
alignment score=4 , category=4 , p=1
Cleavage Site 328
All Other Sites
Reads
0
5
10
15
20
25
0
100
200
300
400
500
600
gi170319674 position (nts)

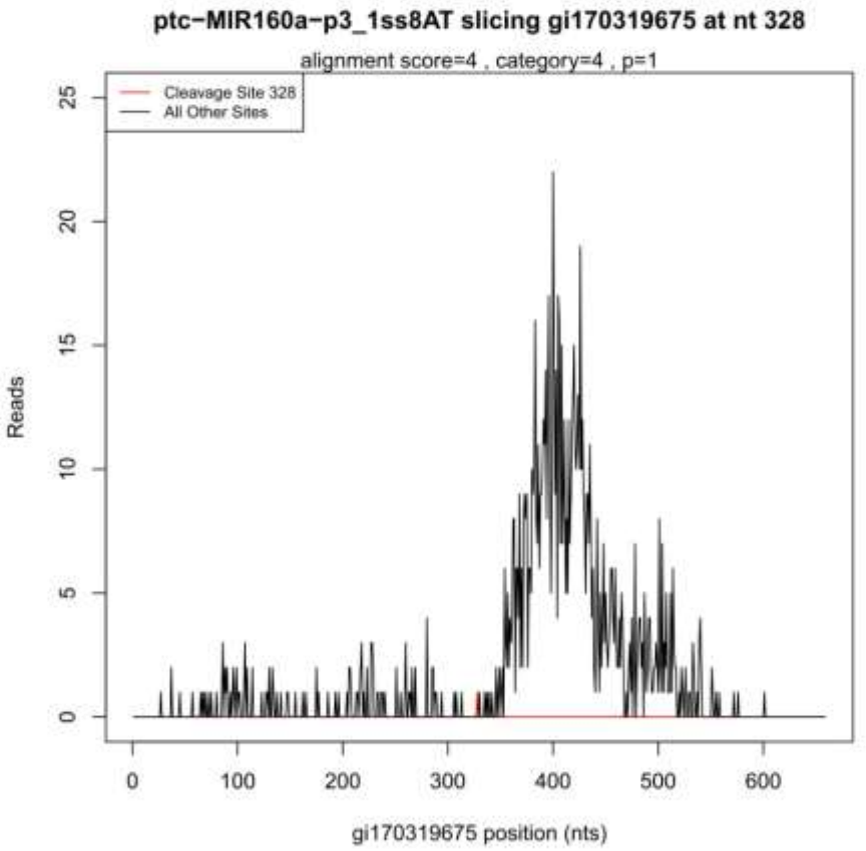
ptc−MIR160a−p3_1ss8AT slicing gi170319675 at nt 328
alignment score=4 , category=4 , p=1
Cleavage Site 328
All Other Sites
Reads
0
5
10
15
20
25
0
100
200
300
400
500
600
gi170319675 position (nts)

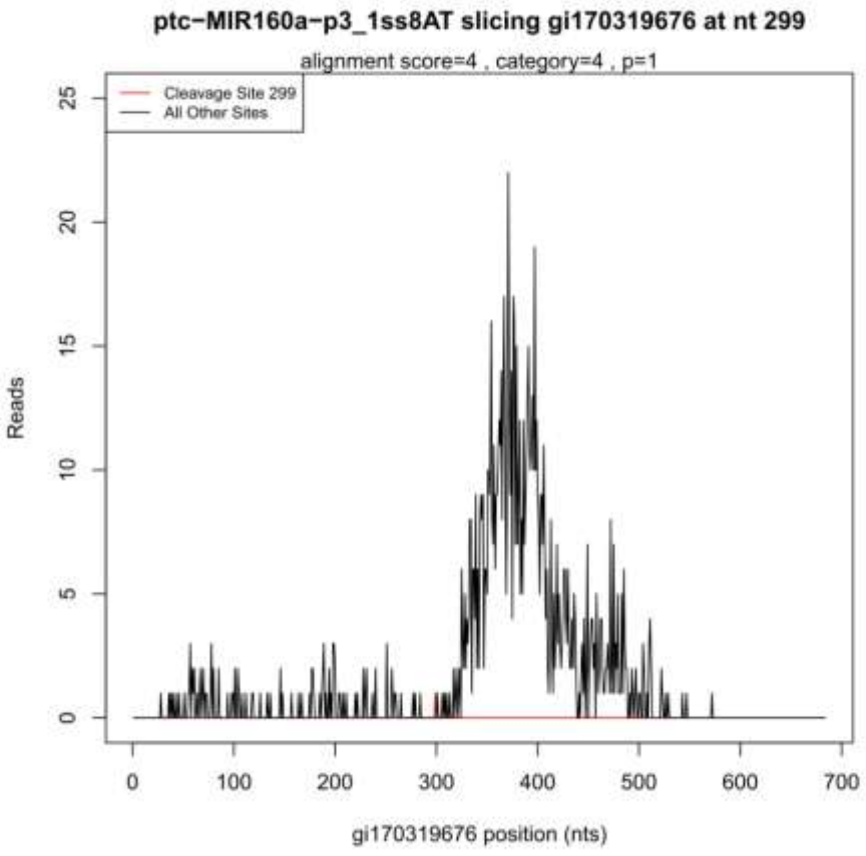
ptc−MIR160a−p3_1ss8AT slicing gi170319676 at nt 299
alignment score=4 , category=4 , p=1
Cleavage Site 299
All Other Sites
Reads
0
5
10
15
20
25
0
100
200
300
400
500
600
700
gi170319676 position (nts)

ptc−MIR160a−p3_1ss8AT slicing gi171355249 at nt 252

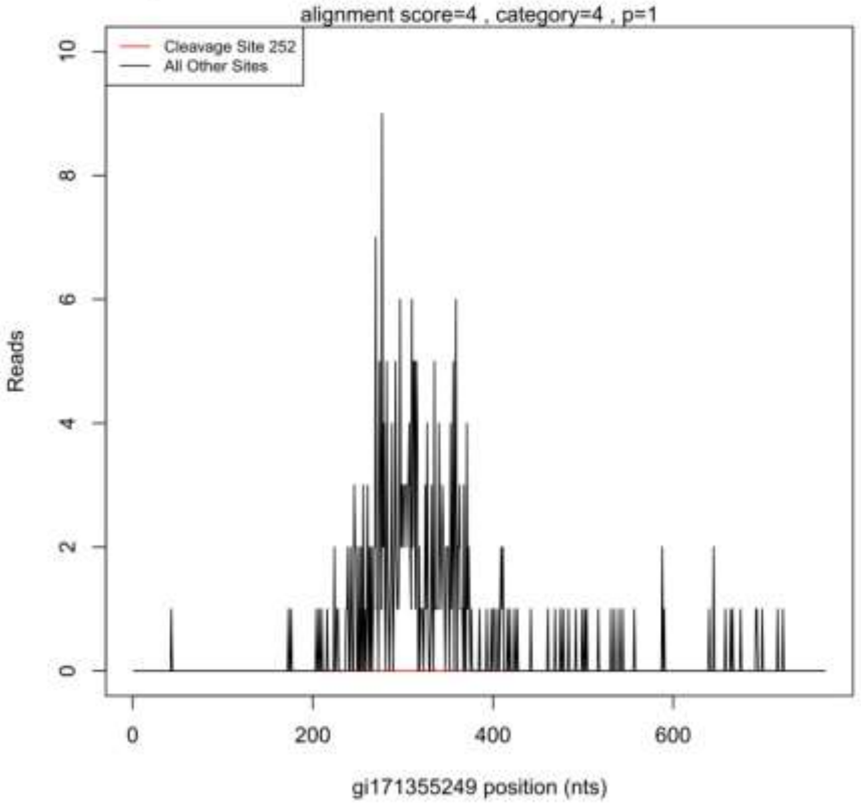

ptc−MIR160a−p3_1ss8AT slicing gi212379345 at nt 177

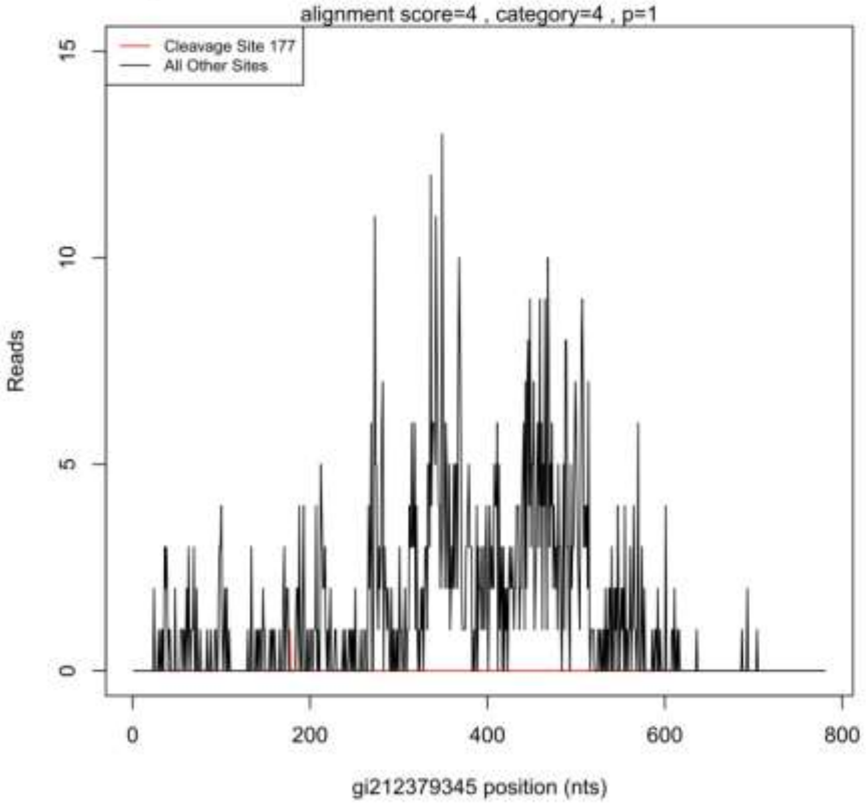

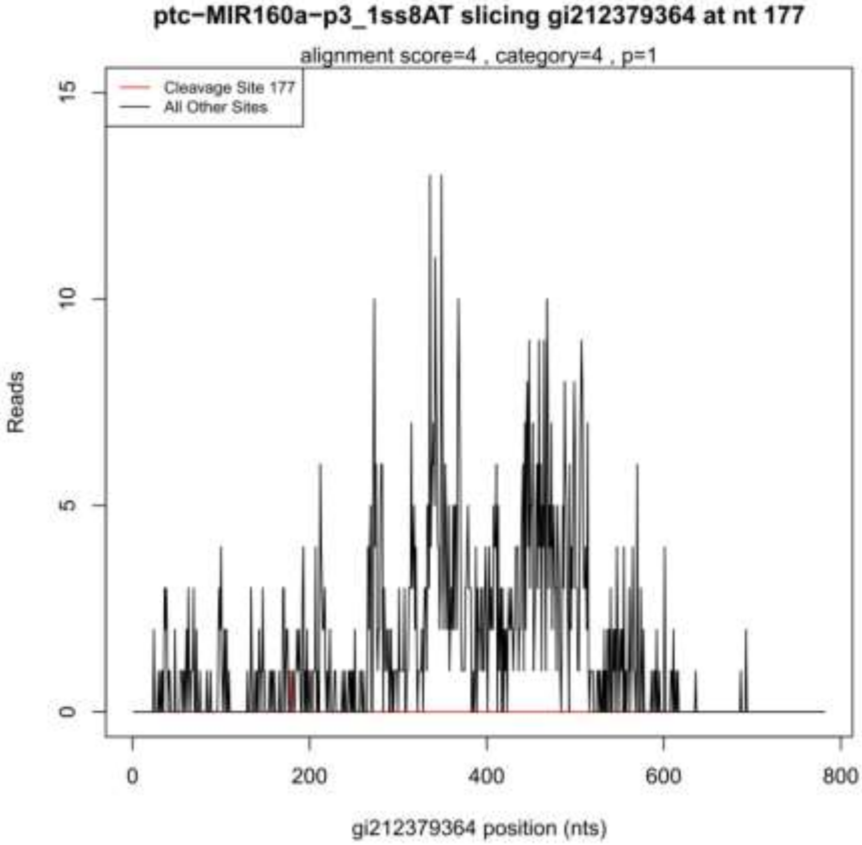
ptc−MIR160a−p3_1ss8AT slicing gi212379364 at nt 177
alignment score=4 , category=4 , p=1
Cleavage Site 177
All Other Sites
Reads
gi212379364 position (nts)

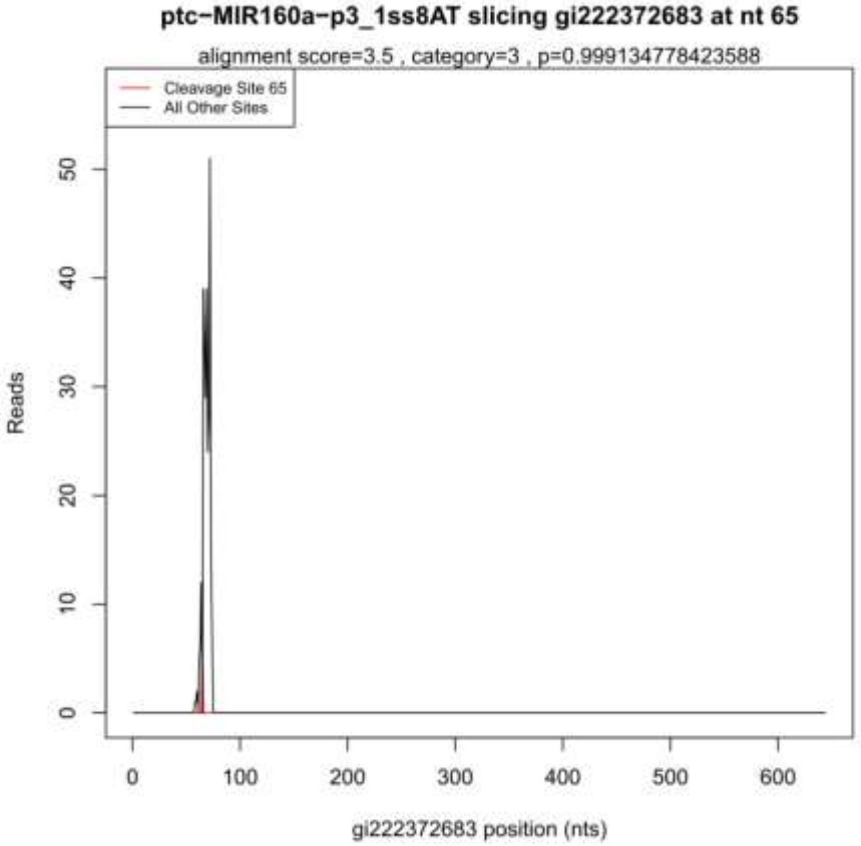
ptc−MIR160a−p3_1ss8AT slicing gi222372683 at nt 65
alignment score=3.5 , category=3 , p=0.999134778423588
Cleavage Site 65
All Other Sites
Reads
gi222372683 position (nts)

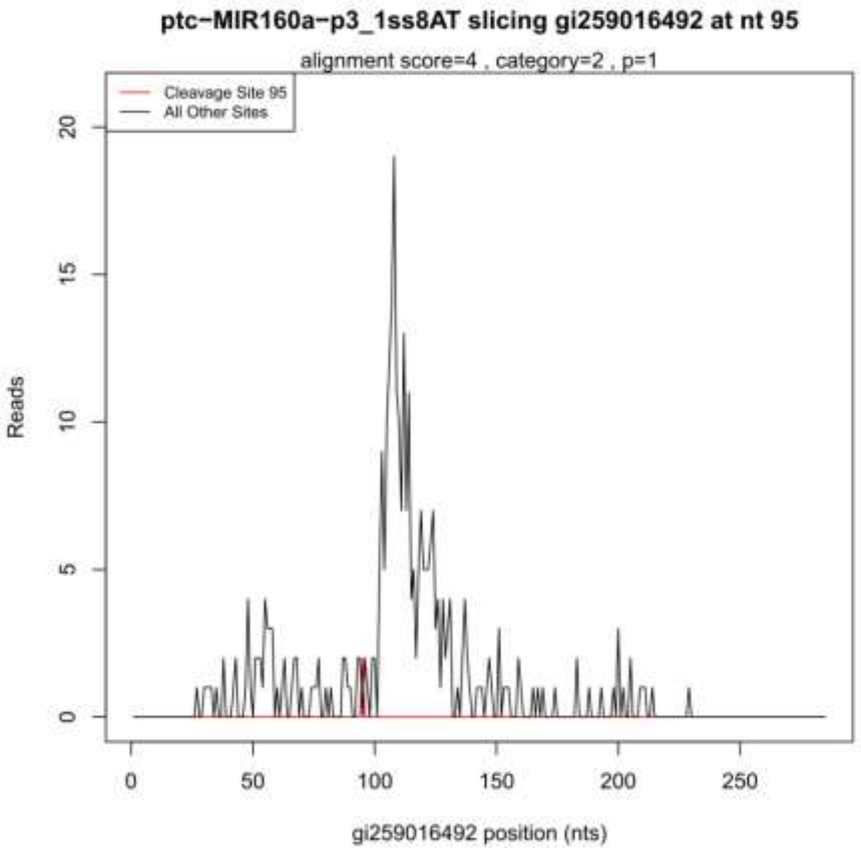
ptc−MIR160a−p3_1ss8AT slicing gi259016492 at nt 95
alignment score=4 , category=2 , p=1
Cleavage Site 95
All Other Sites
Reads
0
5
10
15
20
0
50
100
150
200
250
gi259016492 position (nts)

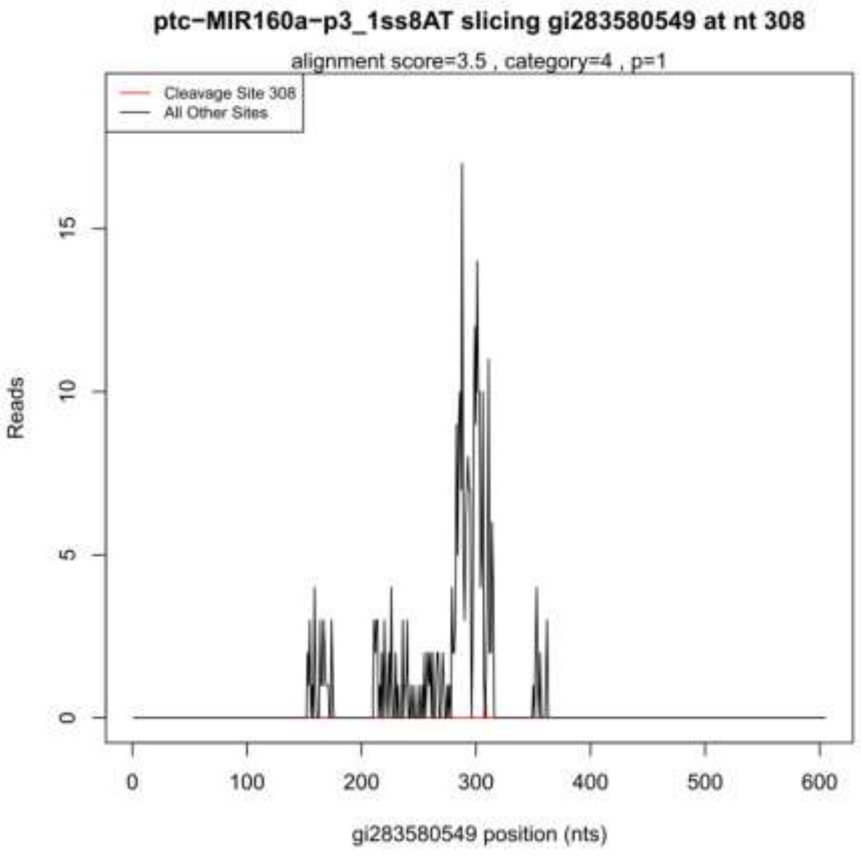
ptc−MIR160a−p3_1ss8AT slicing gi283580549 at nt 308
alignment score=3.5 , category=4 , p=1
Cleavage Site 308
All Other Sites
Reads
0
5
10
15
0
100
200
300
400
500
600
gi283580549 position (nts)

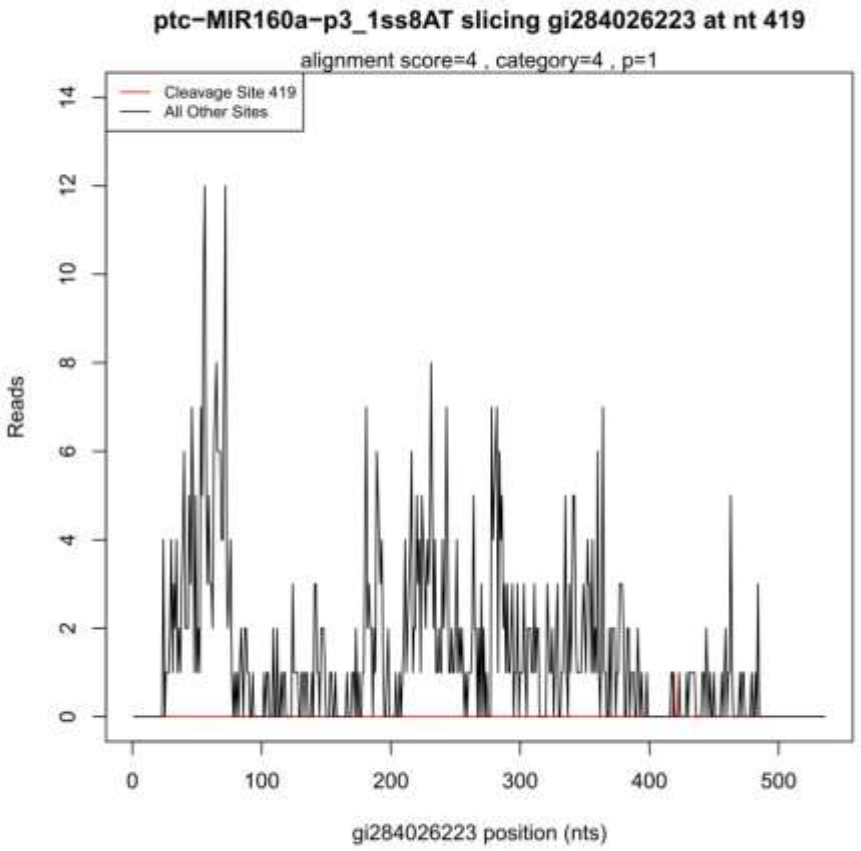
ptc−MIR160a−p3_1ss8AT slicing gi284026223 at nt 419
alignment score=4 , category=4 , p=1
Cleavage Site 419
All Other Sites
Reads
0
2
4
6
8
10
12
14
0
100
200
300
400
500
gi284026223 position (nts)

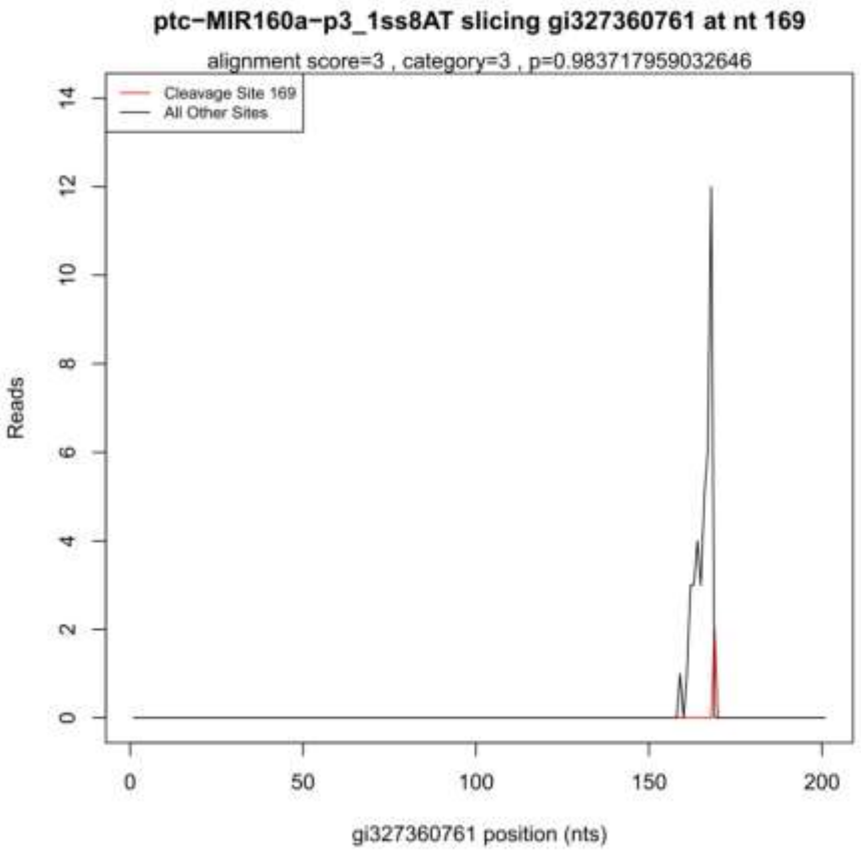
ptc−MIR160a−p3_1ss8AT slicing gi327360761 at nt 169
alignment score=3 , category=3 , p=0.983717959032646
Cleavage Site 169
All Other Sites
Reads
0
2
4
6
8
10
12
14
0
50
100
150
200
gi327360761 position (nts)

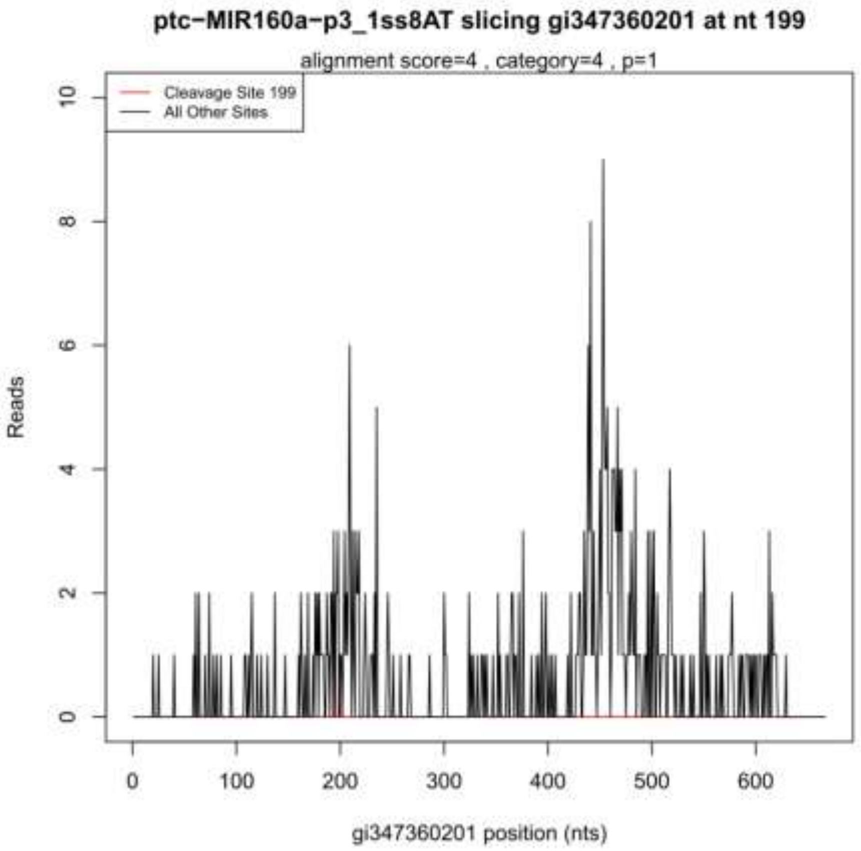
ptc−MIR160a−p3_1ss8AT slicing gi347360201 at nt 199
alignment score=4 , category=4 , p=1
Cleavage Site 199
All Other Sites
Reads
0
2
4
6
8
10
0
100
200
300
400
500
600
gi347360201 position (nts)

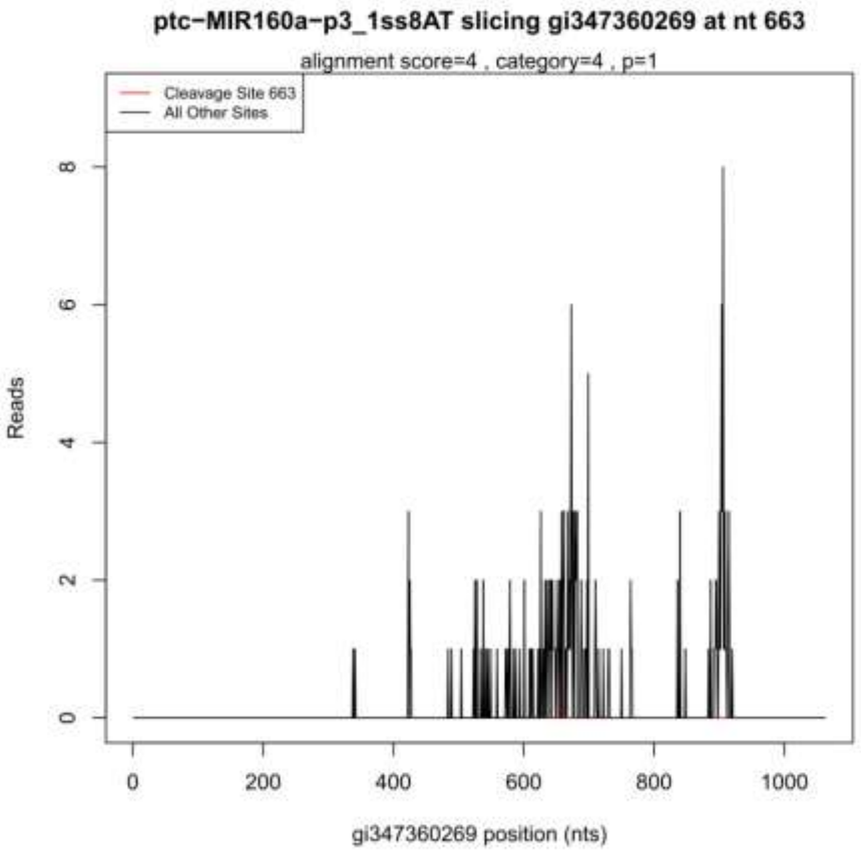
ptc−MIR160a−p3_1ss8AT slicing gi347360269 at nt 663
alignment score=4 , category=4 , p=1
Cleavage Site 663
All Other Sites
Reads
0
2
4
6
8
0
200
400
600
800
1000
gi347360269 position (nts)

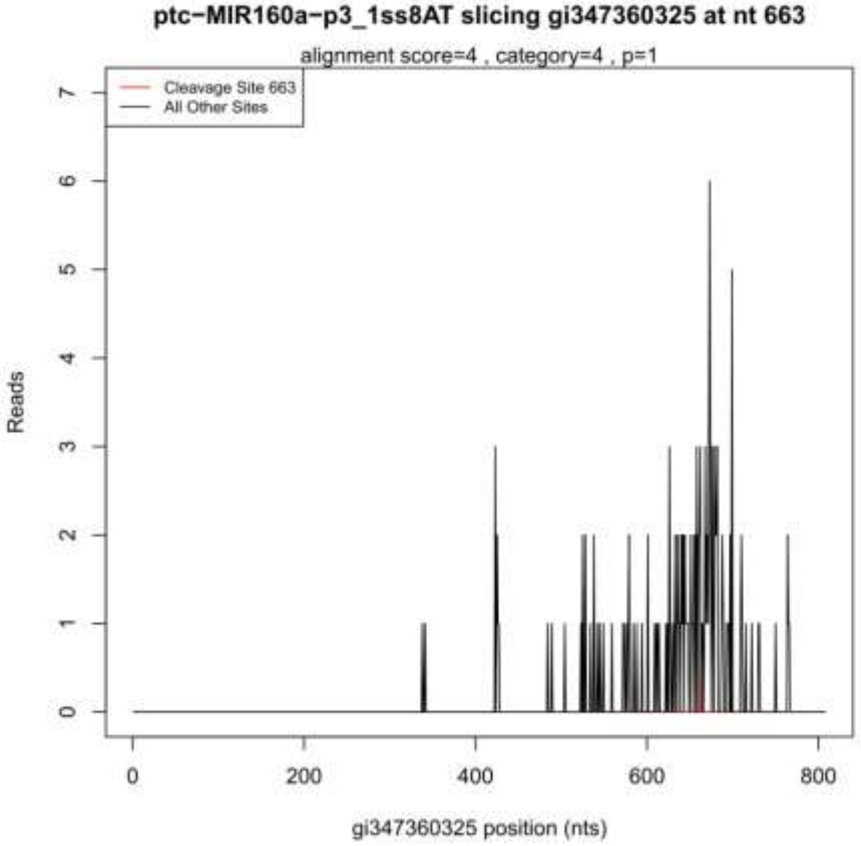
ptc−MIR160a−p3_1ss8AT slicing gi347360325 at nt 663
alignment score=4 , category=4 , p=1
Cleavage Site 663
All Other Sites
Reads
gi347360325 position (nts)

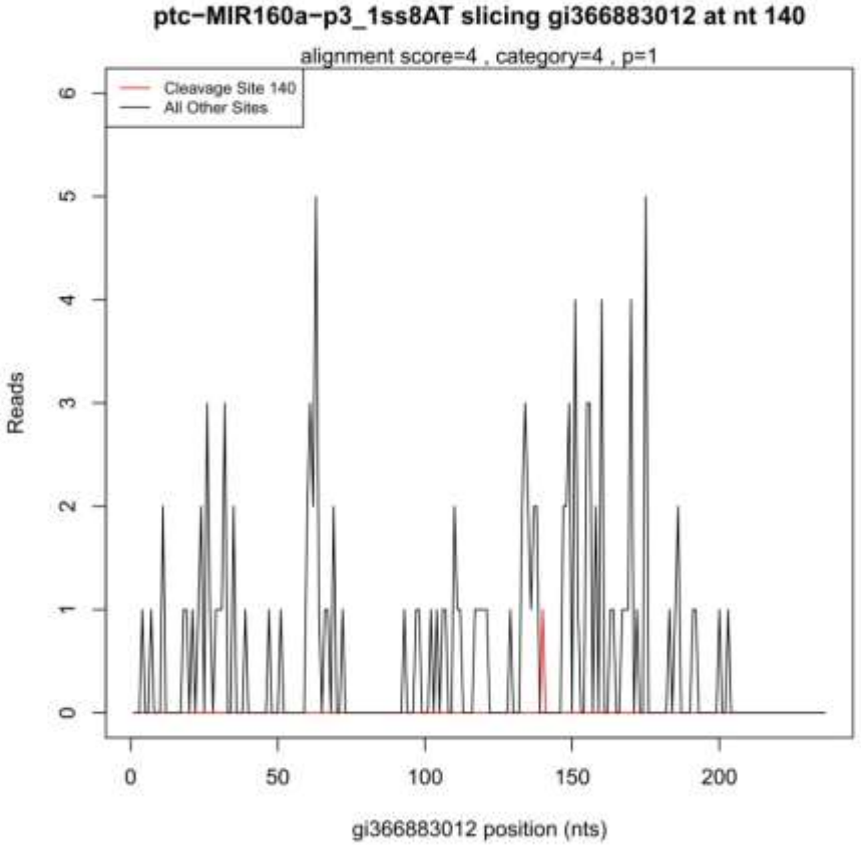
ptc−MIR160a−p3_1ss8AT slicing gi366883012 at nt 140
alignment score=4 , category=4 , p=1
Cleavage Site 140
All Other Sites
Reads
gi366883012 position (nts)

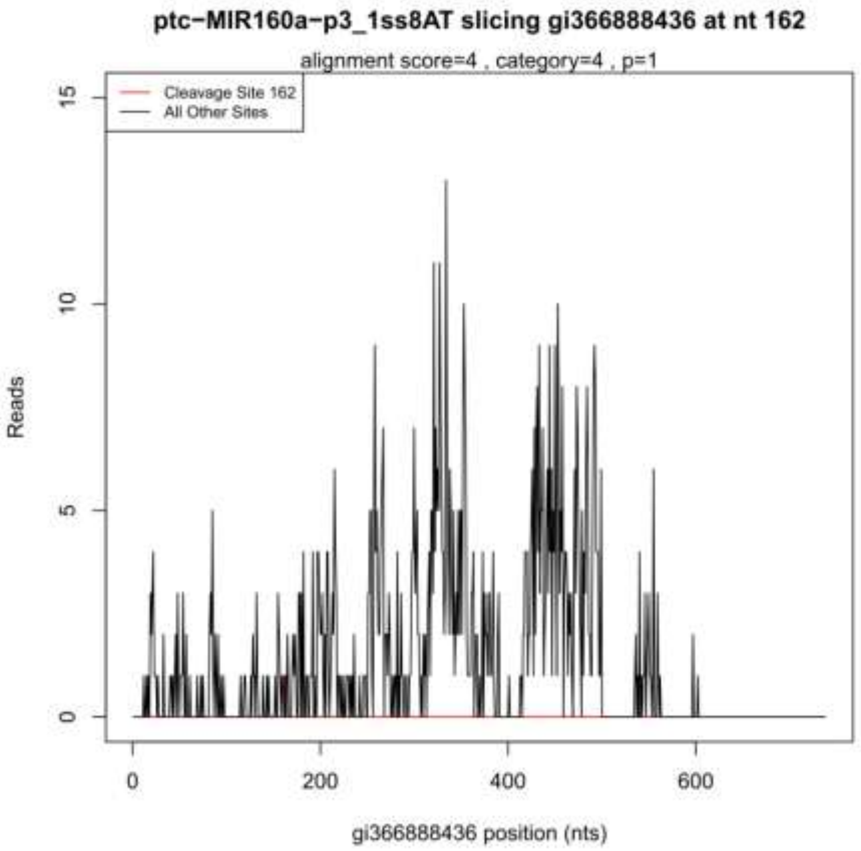

ptc−MIR160a−p3_1ss8AT slicing gi366888436 at nt 162
alignment score=4 , category=4 , p=1
Cleavage Site 162
All Other Sites
Reads
0
5
10
15
0
200
400
600
gi366888436 position (nts)

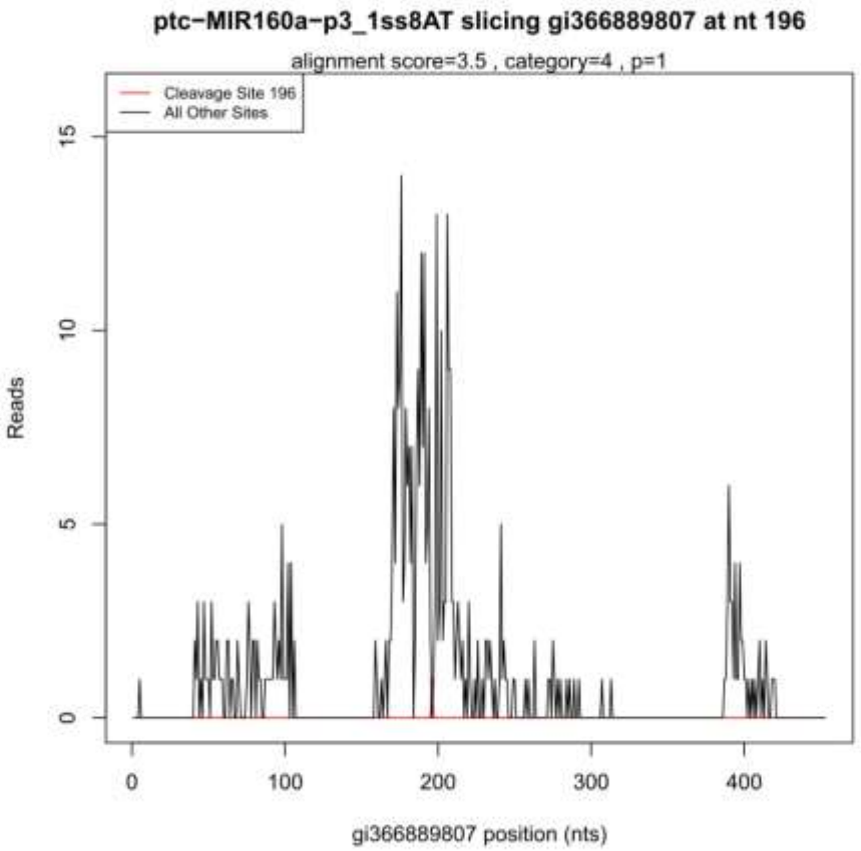

ptc−MIR160a−p3_1ss8AT slicing gi366889807 at nt 196
alignment score=3.5 , category=4 , p=1
Cleavage Site 196
All Other Sites
Reads
0
5
10
15
0
100
200
300
400
gi366889807 position (nts)

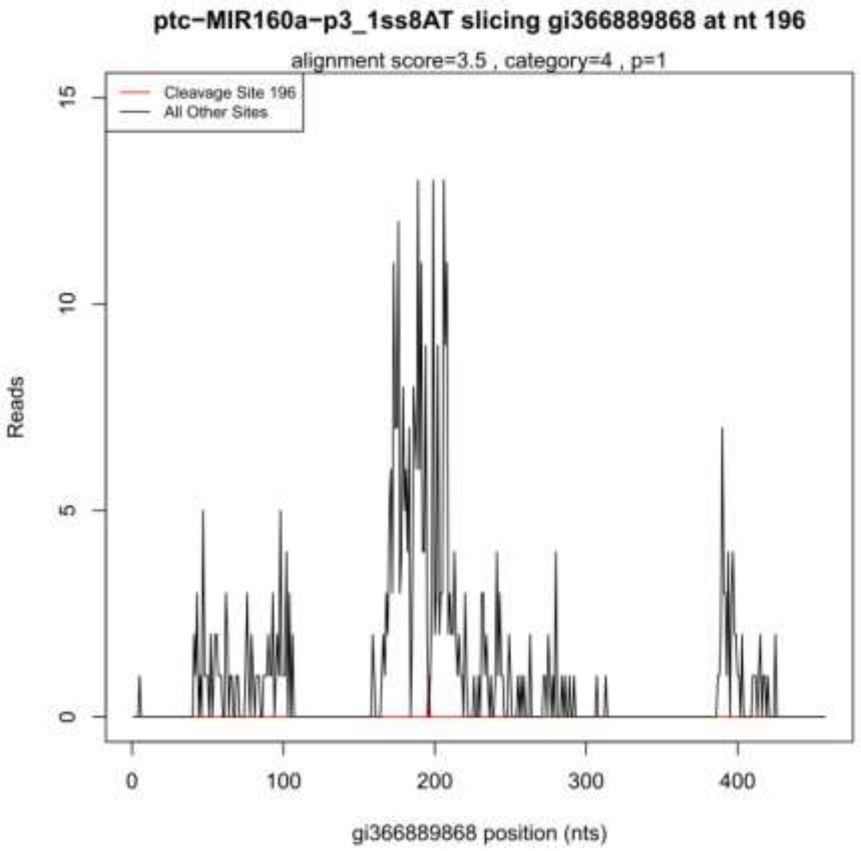
ptc−MIR160a−p3_1ss8AT slicing gi366889868 at nt 196
alignment score=3.5 , category=4 , p=1
Cleavage Site 196
All Other Sites
Reads
0
5
10
15
0
100
200
300
400
gi366889868 position (nts)

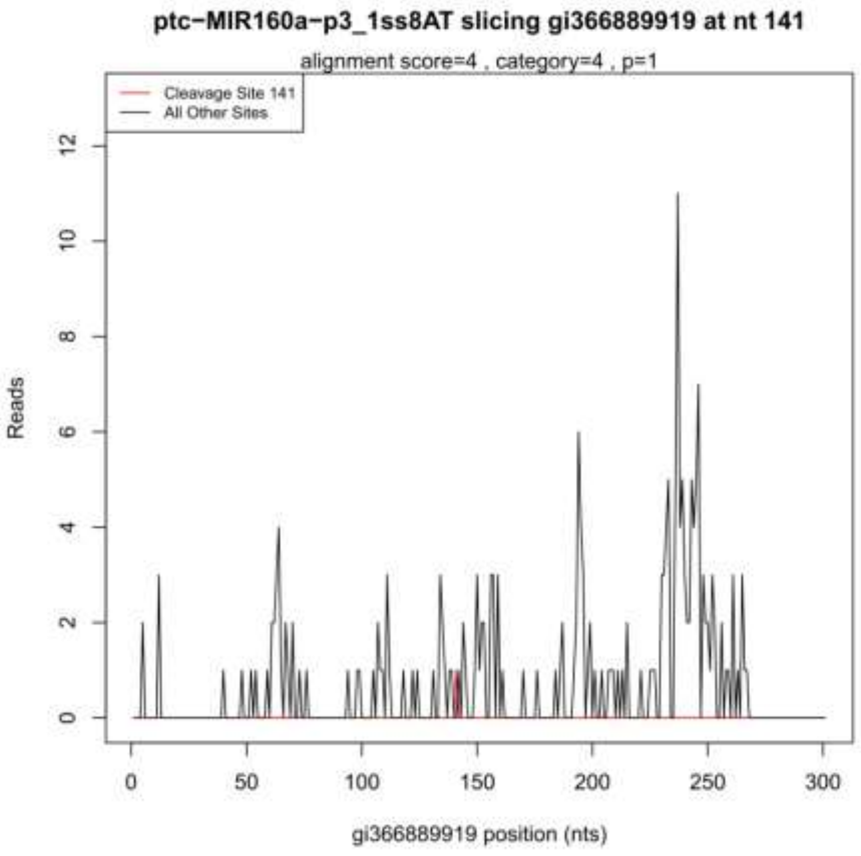
ptc−MIR160a−p3_1ss8AT slicing gi366889919 at nt 141
alignment score=4 , category=4 , p=1
Cleavage Site 141
All Other Sites
Reads
0
2
4
6
8
10
12
0
50
100
150
200
250
300
gi366889919 position (nts)

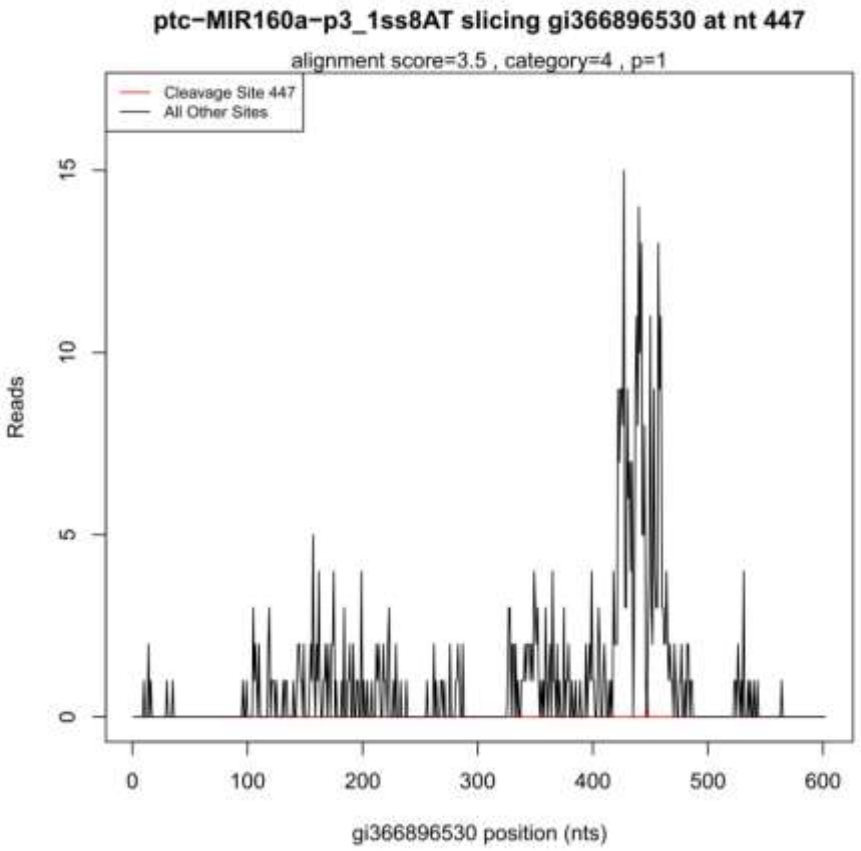

ptc−MIR160a−p3_1ss8AT slicing gi366896530 at nt 447
alignment score=3.5 , category=4 , p=1
Cleavage Site 447
All Other Sites
Reads
0
5
10
15
0
100
200
300
400
500
600
gi366896530 position (nts)

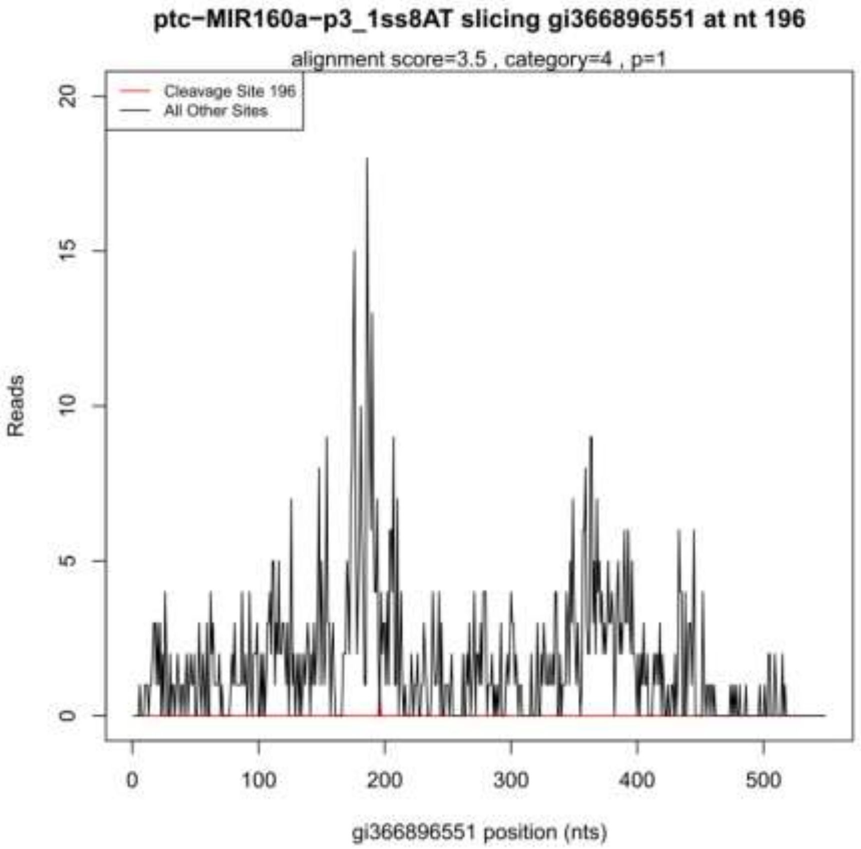

ptc−MIR160a−p3_1ss8AT slicing gi366896551 at nt 196
alignment score=3.5 , category=4 , p=1
Cleavage Site 196
All Other Sites
Reads
0
5
10
15
20
0
100
200
300
400
500
gi366896551 position (nts)

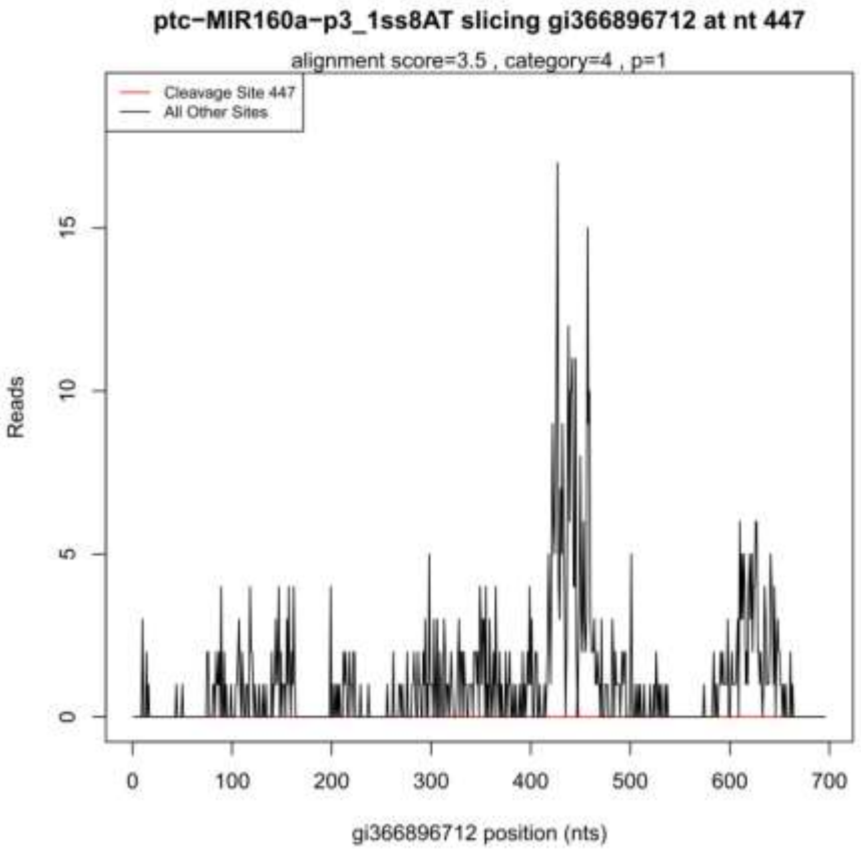

ptc−MIR160a−p3_1ss8AT slicing gi366896712 at nt 447
alignment score=3.5 , category=4 , p=1
Cleavage Site 447
All Other Sites
Reads
0
5
10
15
0
100
200
300
400
500
600
700
gi366896712 position (nts)

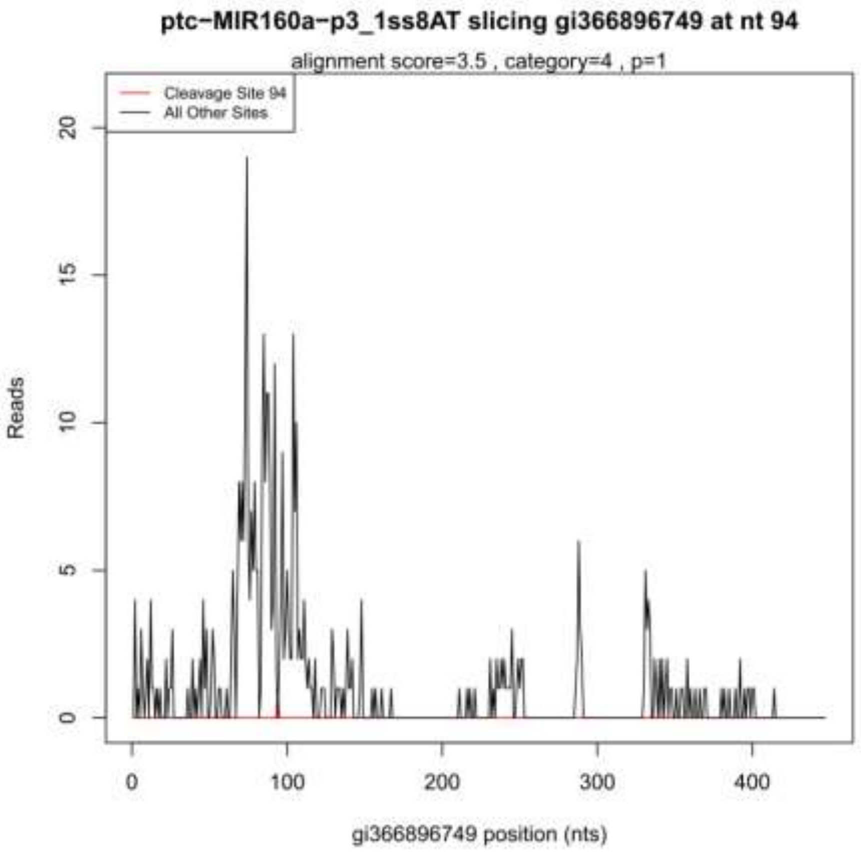

ptc−MIR160a−p3_1ss8AT slicing gi366896749 at nt 94
alignment score=3.5 , category=4 , p=1
Cleavage Site 94
All Other Sites
Reads
0
5
10
15
20
0
100
200
300
400
gi366896749 position (nts)

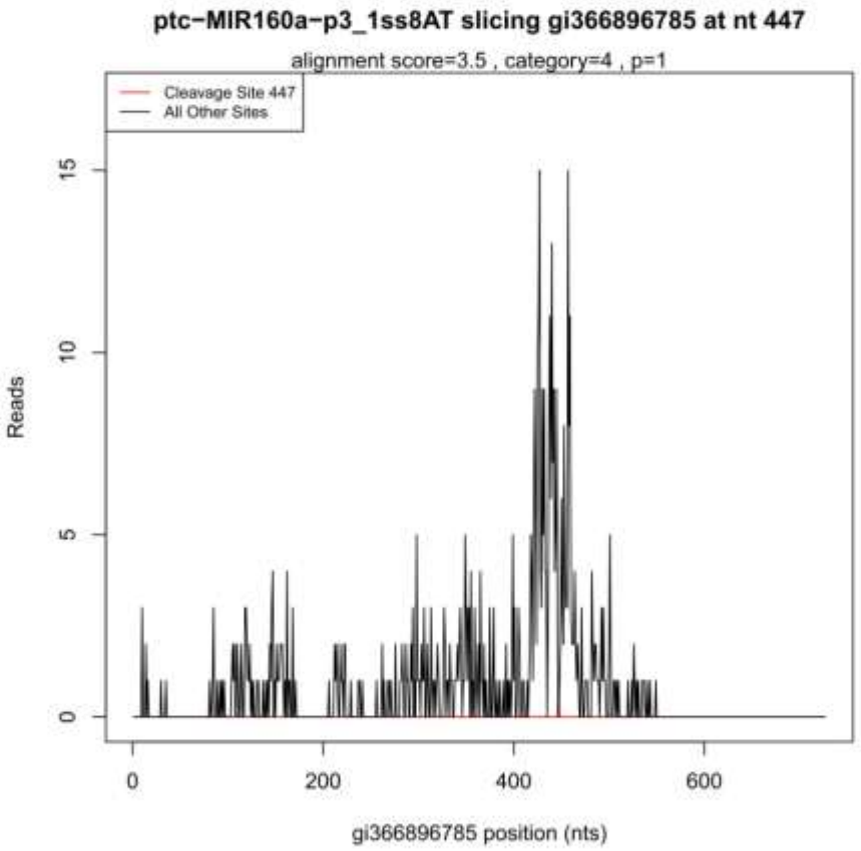

ptc−MIR160a−p3_1ss8AT slicing gi366896785 at nt 447
alignment score=3.5 , category=4 , p=1
Cleavage Site 447
All Other Sites
Reads
0
5
10
15
0
200
400
600
gi366896785 position (nts)

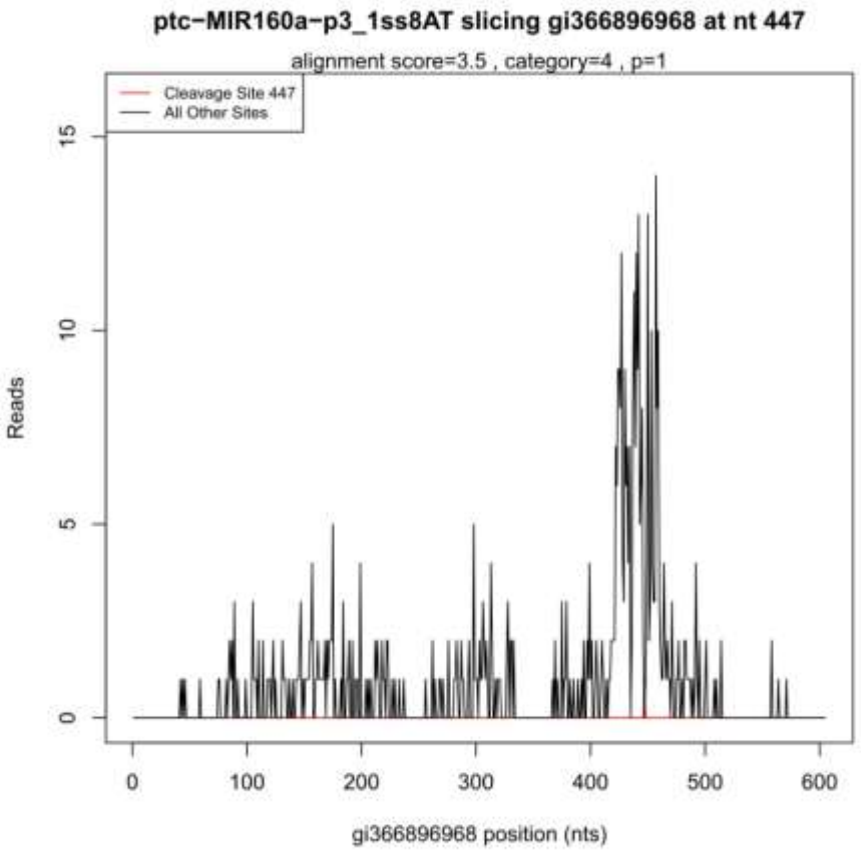

ptc−MIR160a−p3_1ss8AT slicing gi366896968 at nt 447
alignment score=3.5 , category=4 , p=1
Cleavage Site 447
All Other Sites
Reads
0
5
10
15
0
100
200
300
400
500
600
gi366896968 position (nts)

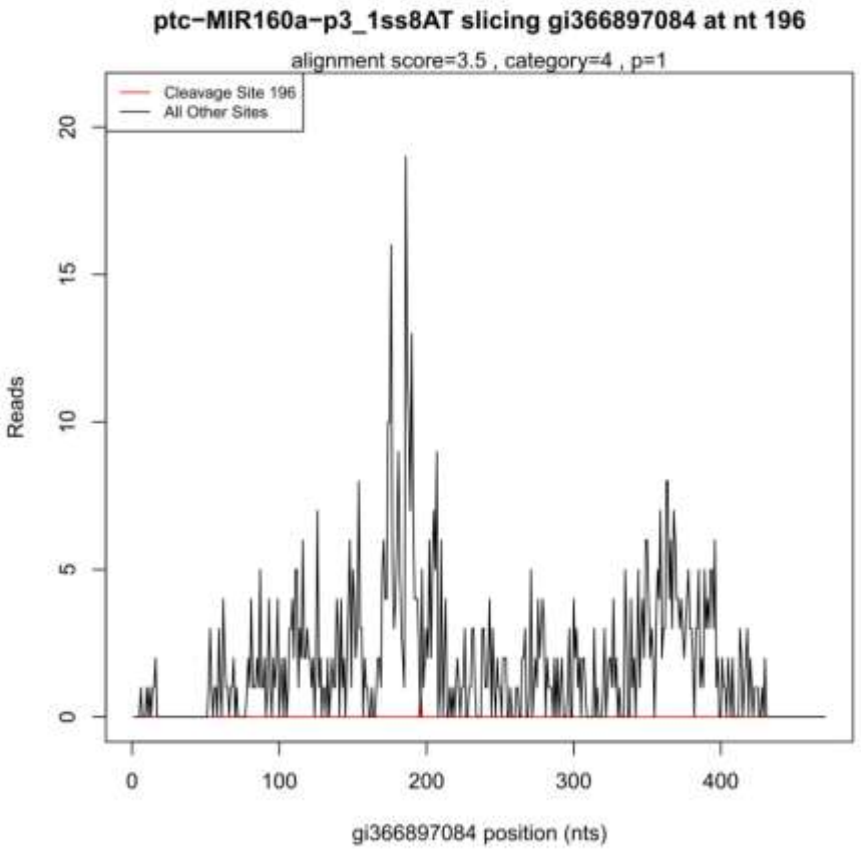
ptc−MIR160a−p3_1ss8AT slicing gi366897084 at nt 196
alignment score=3.5 , category=4 , p=1
Cleavage Site 196
All Other Sites
Reads
0
5
10
15
20
0
100
200
300
400
gi366897084 position (nts)

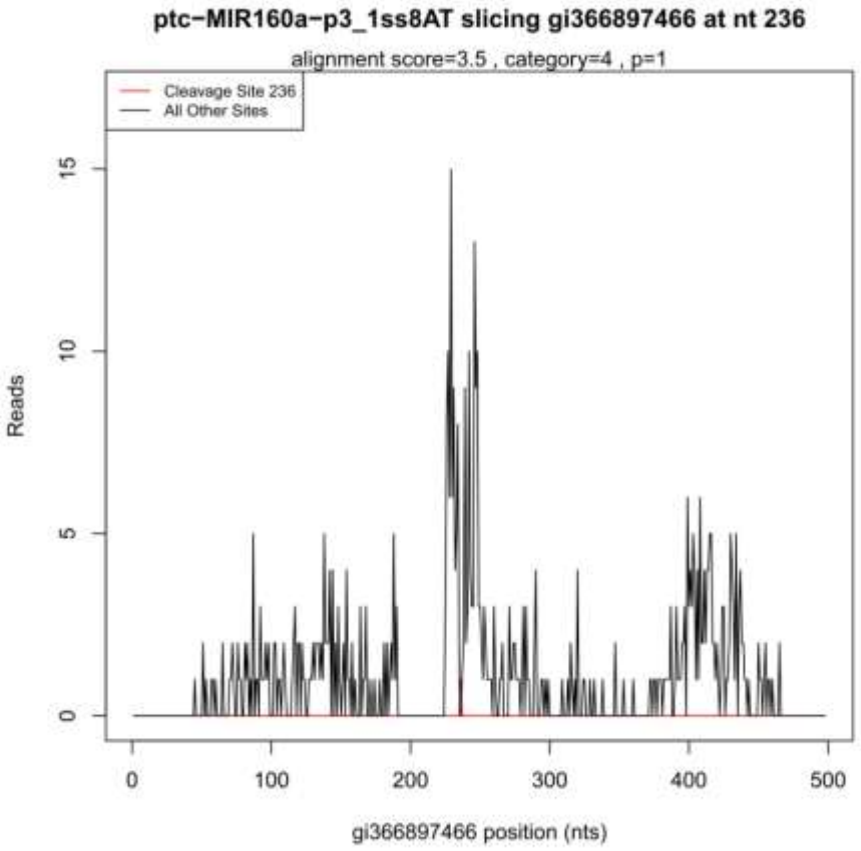
ptc−MIR160a−p3_1ss8AT slicing gi366897466 at nt 236
alignment score=3.5 , category=4 , p=1
Cleavage Site 236
All Other Sites
Reads
0
5
10
15
0
100
200
300
400
500
gi366897466 position (nts)

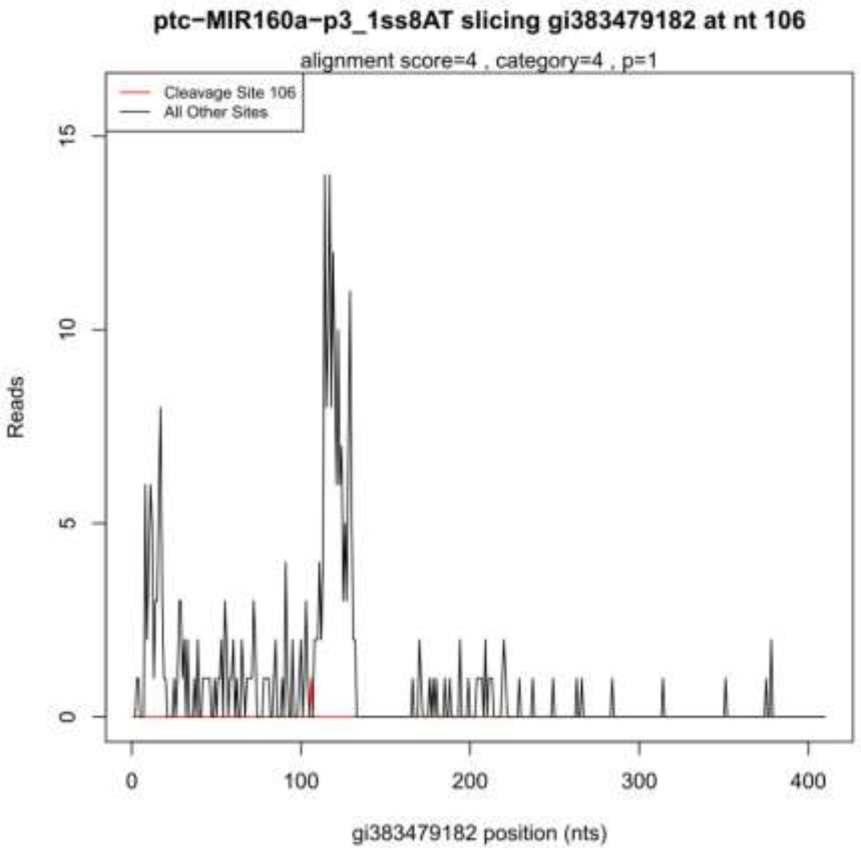
ptc−MIR160a−p3_1ss8AT slicing gi383479182 at nt 106
alignment score=4 , category=4 , p=1
Cleavage Site 106
All Other Sites
Reads
0
5
10
15
0
100
200
300
400
gi383479182 position (nts)

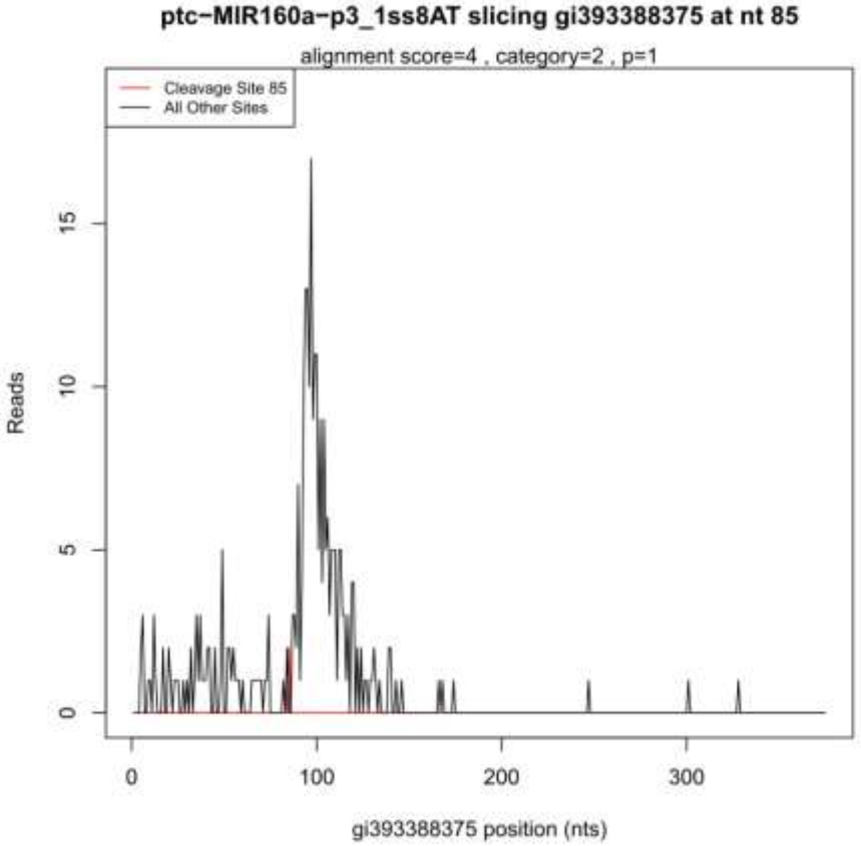
ptc−MIR160a−p3_1ss8AT slicing gi393388375 at nt 85
alignment score=4 , category=2 , p=1
Cleavage Site 85
All Other Sites
Reads
0
5
10
15
0
100
200
300
gi393388375 position (nts)

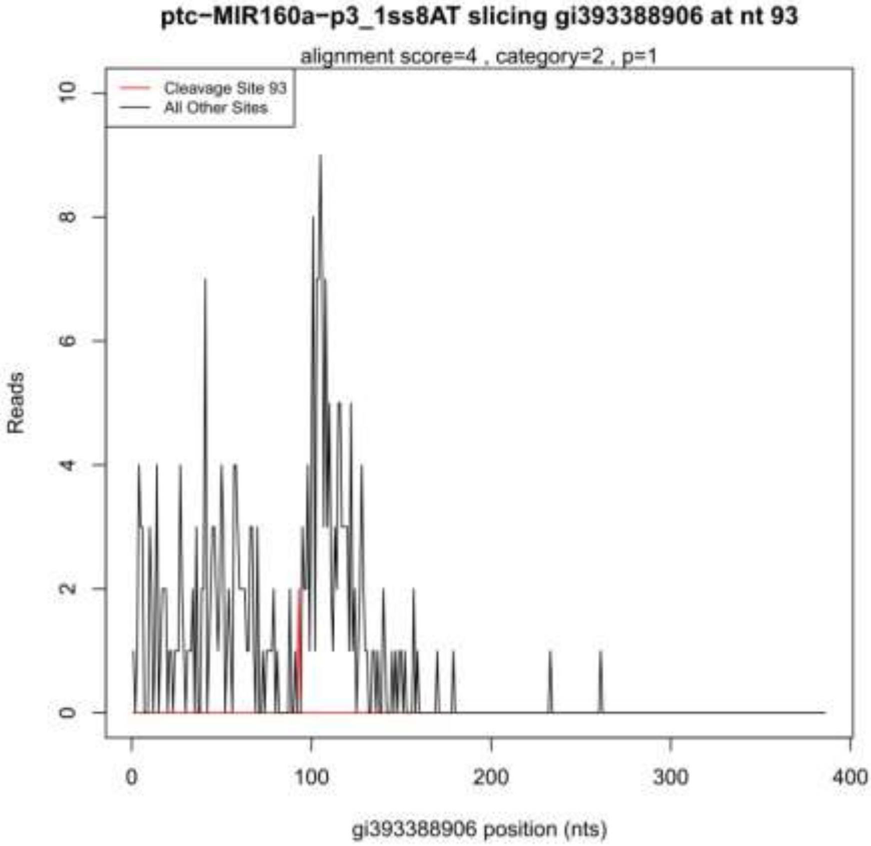
ptc−MIR160a−p3_1ss8AT slicing gi393388906 at nt 93
alignment score=4 , category=2 , p=1
Cleavage Site 93
All Other Sites
Reads
gi393388906 position (nts)

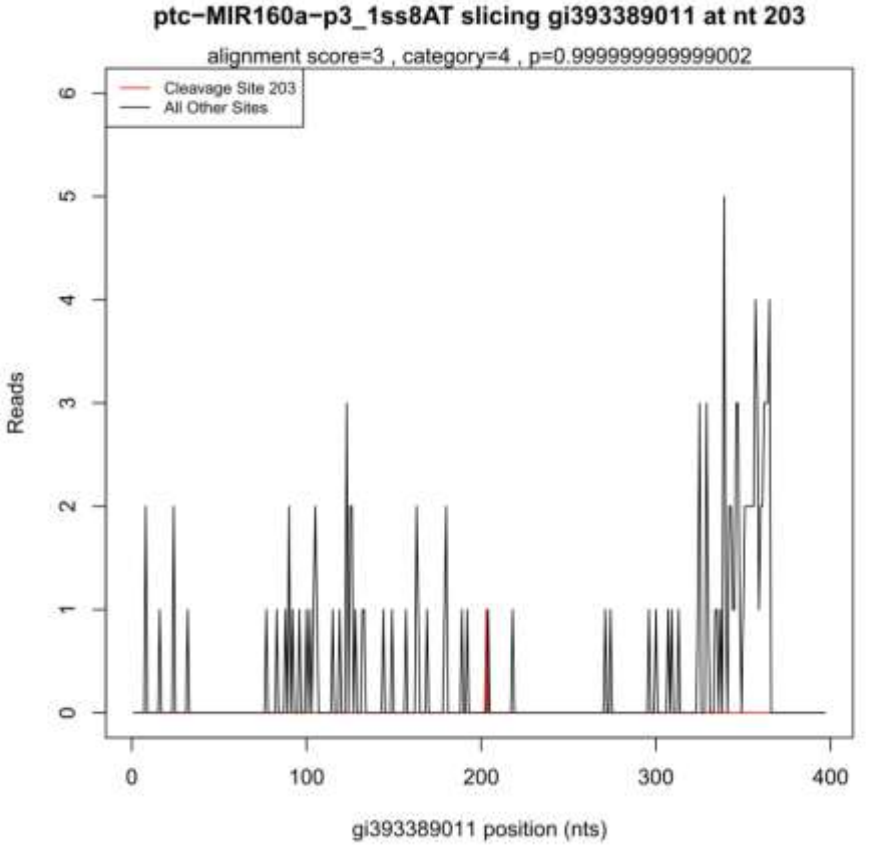
ptc−MIR160a−p3_1ss8AT slicing gi393389011 at nt 203
alignment score=3 , category=4 , p=0.999999999999002
Cleavage Site 203
All Other Sites
Reads
gi393389011 position (nts)

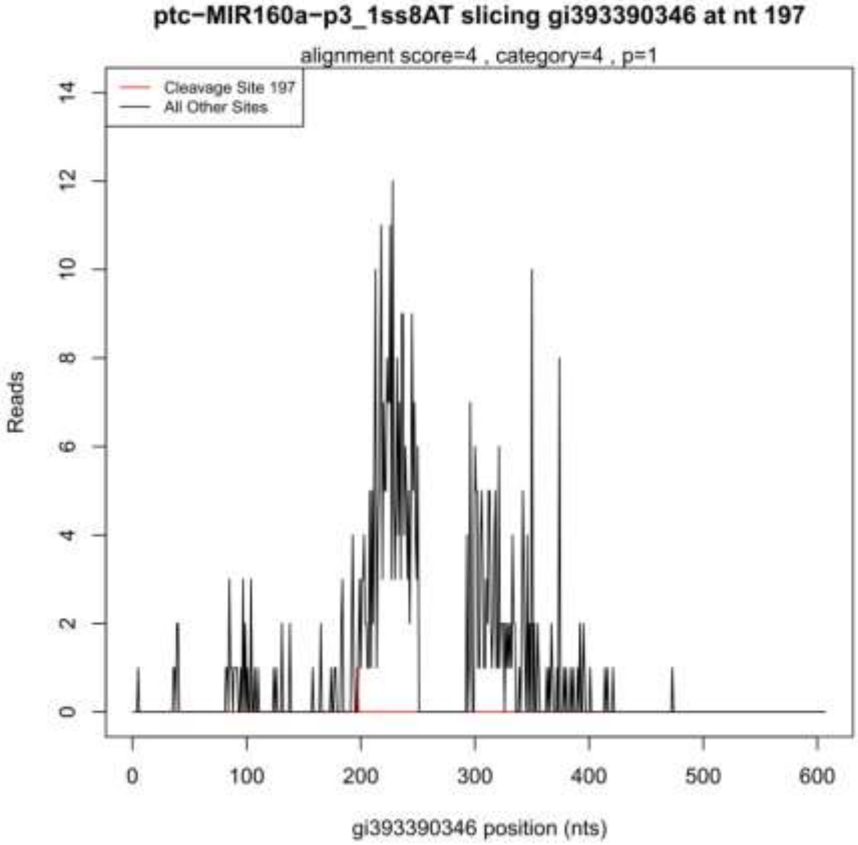
ptc−MIR160a−p3_1ss8AT slicing gi393390346 at nt 197
alignment score=4 , category=4 , p=1
Cleavage Site 197
All Other Sites
Reads
gi393390346 position (nts)
0
2
4
6
8
10
12
14
0
100
200
300
400
500
600

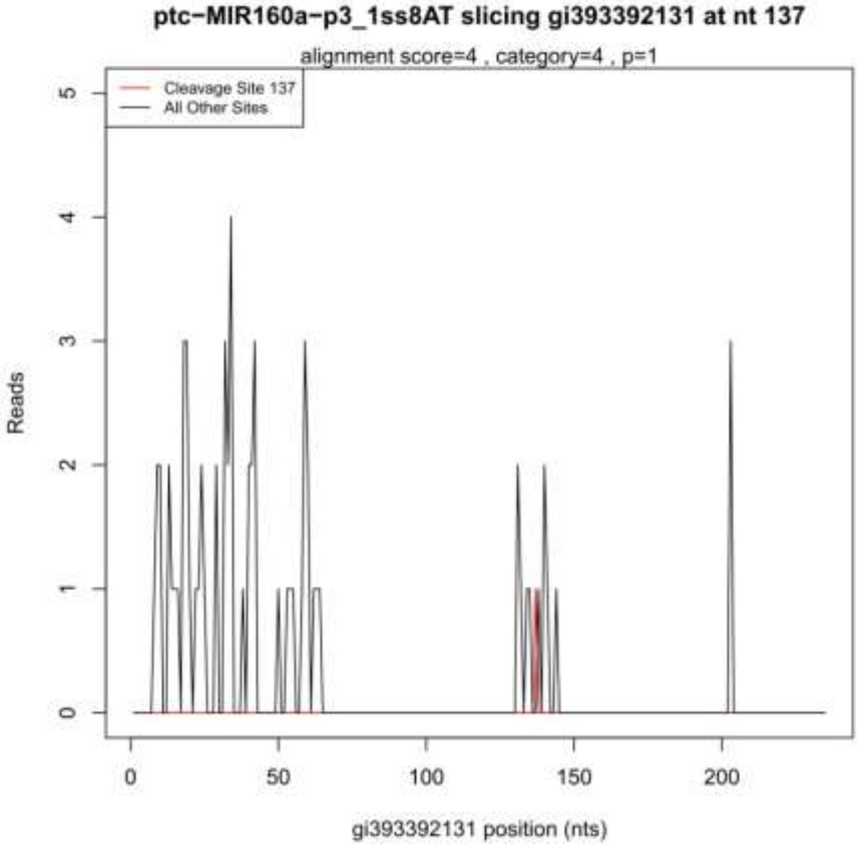
ptc−MIR160a−p3_1ss8AT slicing gi393392131 at nt 137
alignment score=4 , category=4 , p=1
Cleavage Site 137
All Other Sites
Reads
gi393392131 position (nts)
0
1
2
3
4
5
0
50
100
150
200

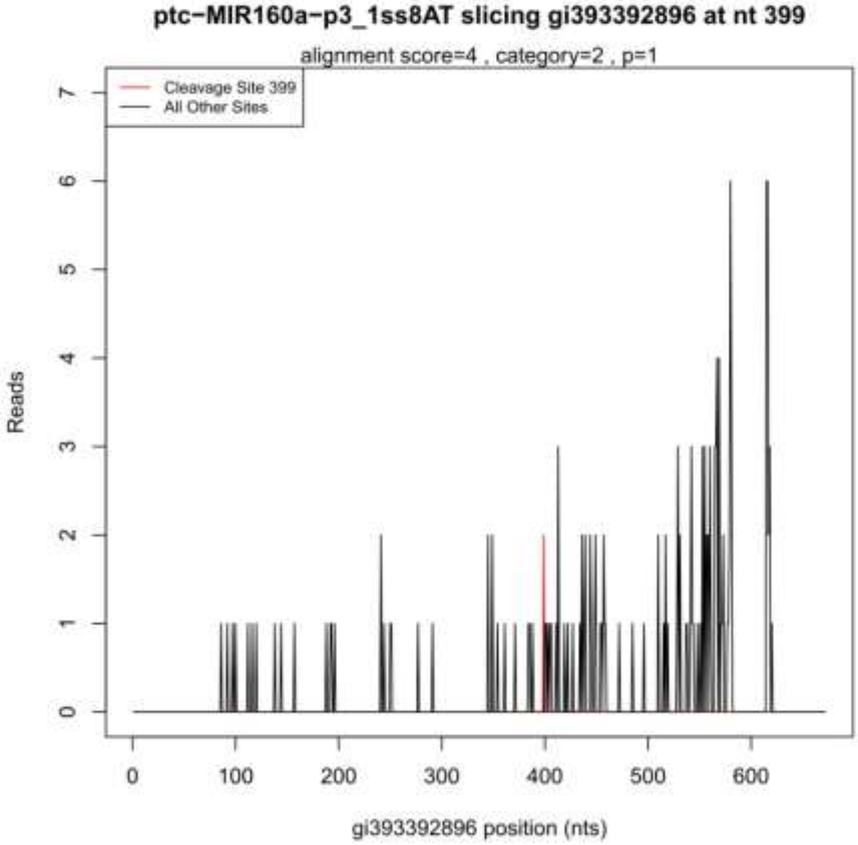

ptc-MIR160a-p3_1ss8AT slicing gi393392896 at nt 399
alignment score=4 , category=2 , p=1
Cleavage Site 399
All Other Sites
Reads
gi393392896 position (nts)

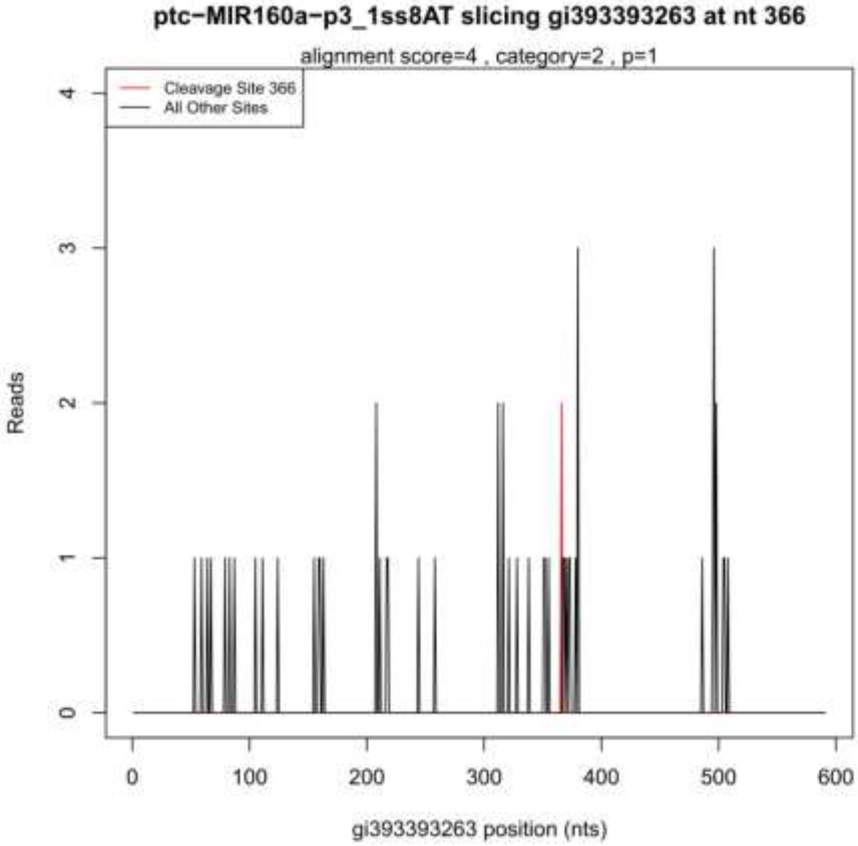

ptc-MIR160a-p3_1ss8AT slicing gi393393263 at nt 366
alignment score=4 , category=2 , p=1
Cleavage Site 366
All Other Sites
Reads
gi393393263 position (nts)

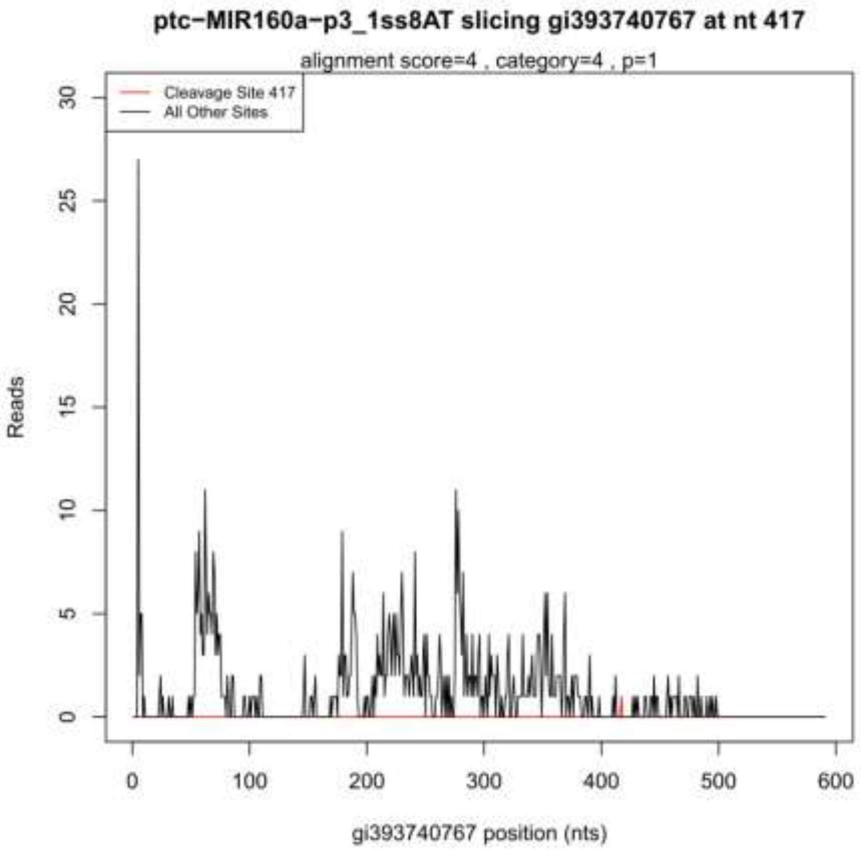
ptc-MIR160a-p3_1ss8AT slicing gi393740767 at nt 417
alignment score=4 , category=4 , p=1
Cleavage Site 417
All Other Sites
Reads
0
5
10
15
20
25
30
0
100
200
300
400
500
600
gi393740767 position (nts)

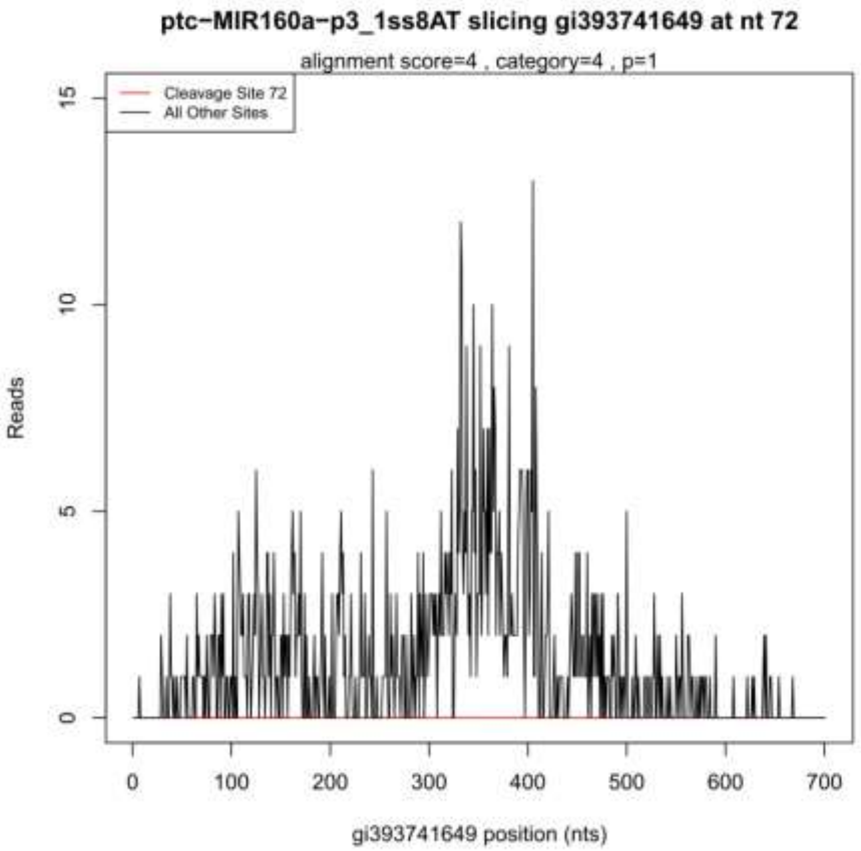
ptc-MIR160a-p3_1ss8AT slicing gi393741649 at nt 72
alignment score=4 , category=4 , p=1
Cleavage Site 72
All Other Sites
Reads
0
5
10
15
0
100
200
300
400
500
600
700
gi393741649 position (nts)

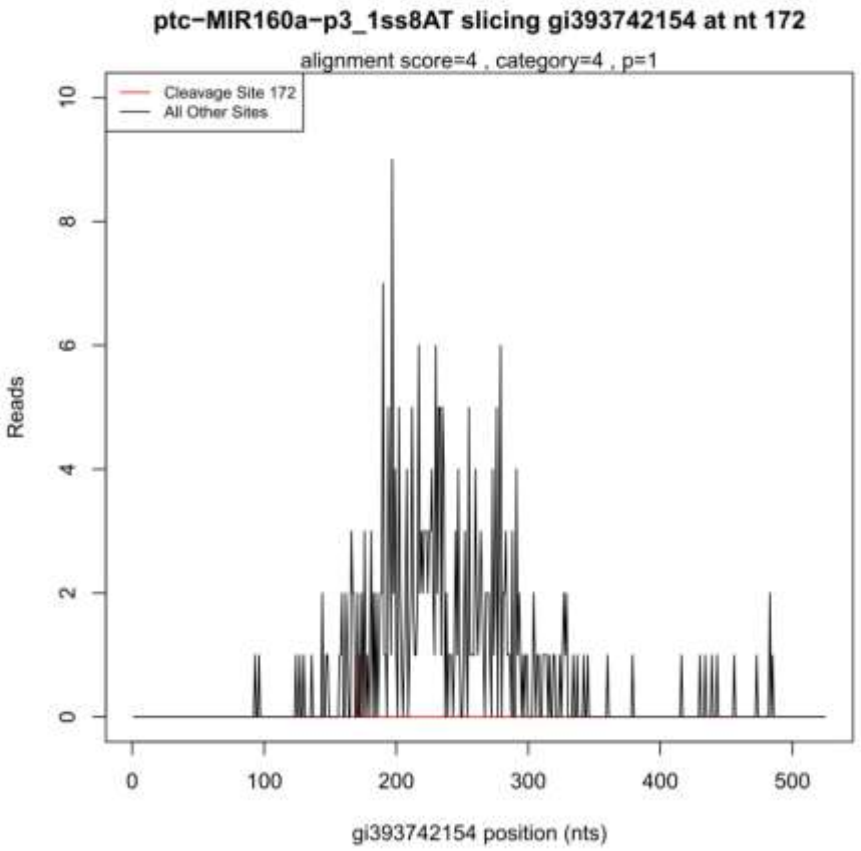

ptc−MIR160a−p3_1ss8AT slicing gi393742154 at nt 172
alignment score=4 , category=4 , p=1
Cleavage Site 172
All Other Sites
Reads
0
2
4
6
8
10
0
100
200
300
400
500
gi393742154 position (nts)

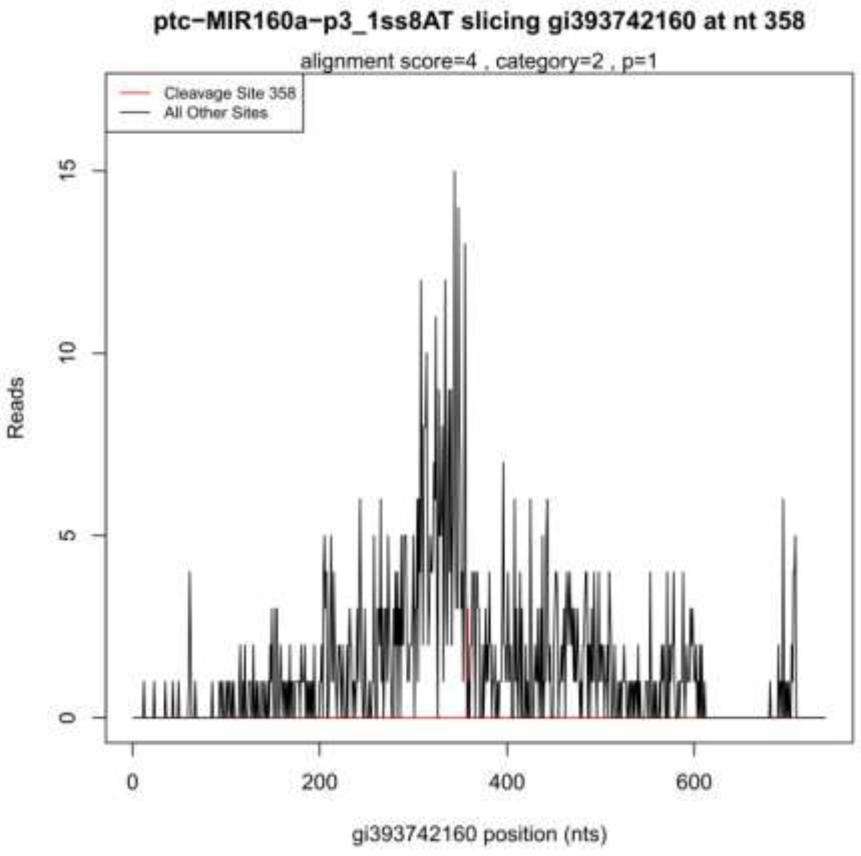

ptc−MIR160a−p3_1ss8AT slicing gi393742160 at nt 358
alignment score=4 , category=2 , p=1
Cleavage Site 358
All Other Sites
Reads
0
5
10
15
0
200
400
600
gi393742160 position (nts)

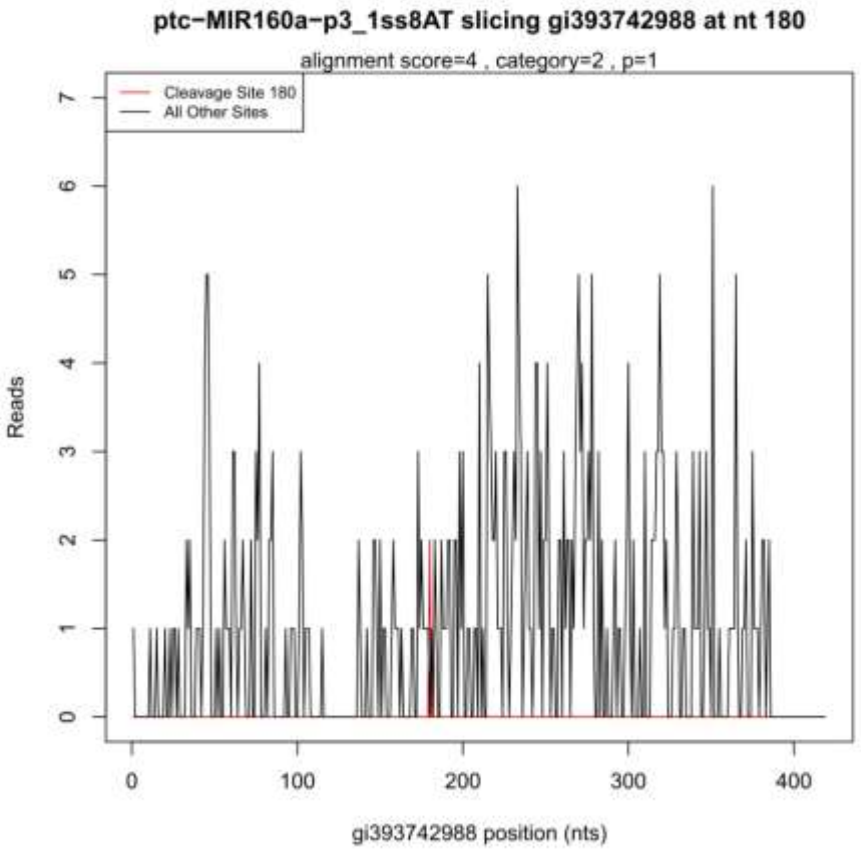
ptc−MIR160a−p3_1ss8AT slicing gi393742988 at nt 180
alignment score=4 , category=2 , p=1
Cleavage Site 180
All Other Sites
Reads
gi393742988 position (nts)

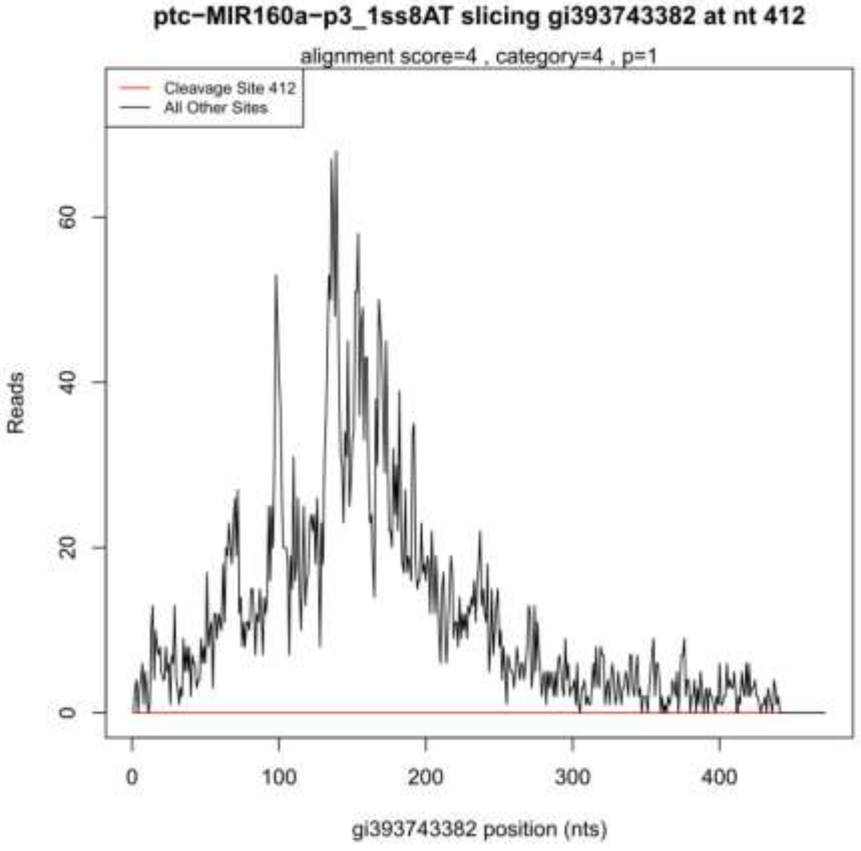
ptc−MIR160a−p3_1ss8AT slicing gi393743382 at nt 412
alignment score=4 , category=4 , p=1
Cleavage Site 412
All Other Sites
Reads
gi393743382 position (nts)

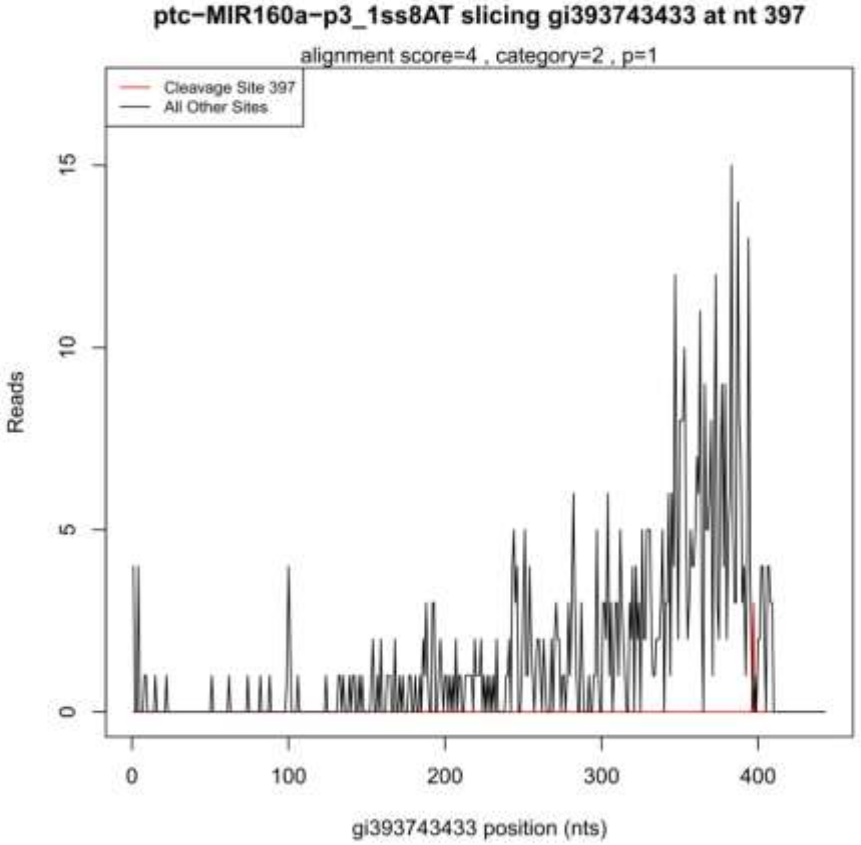
ptc-MIR160a-p3_1ss8AT slicing gi393743433 at nt 397
alignment score=4 , category=2 , p=1
Cleavage Site 397
All Other Sites
Reads
0
5
10
15
0
100
200
300
400
gi393743433 position (nts)

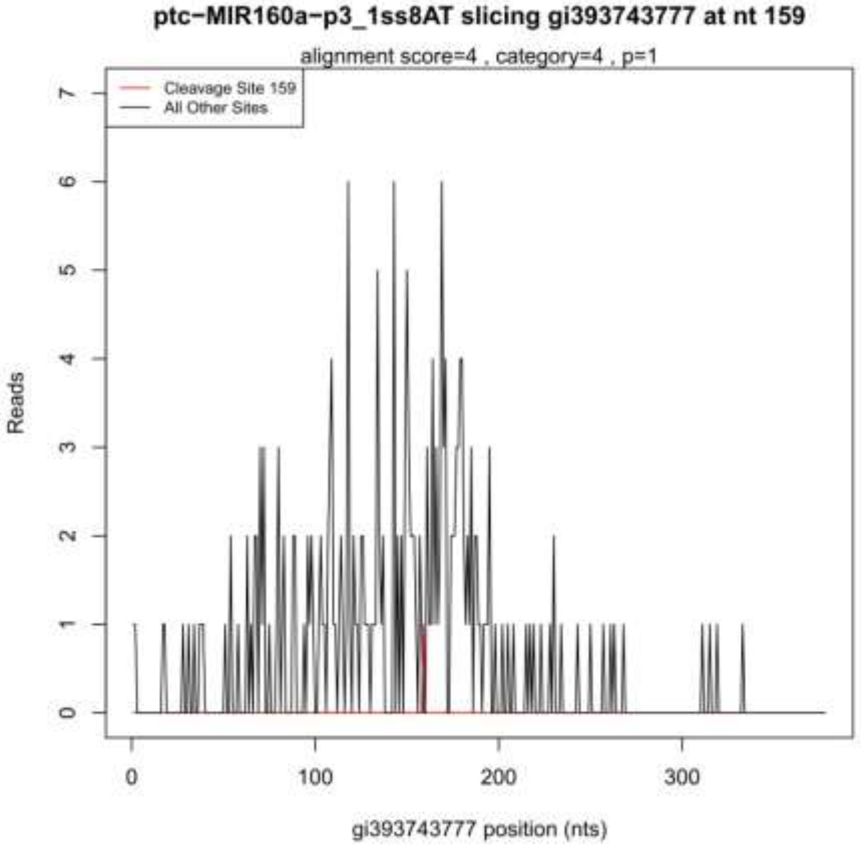
ptc-MIR160a-p3_1ss8AT slicing gi393743777 at nt 159
alignment score=4 , category=4 , p=1
Cleavage Site 159
All Other Sites
Reads
0
1
2
3
4
5
6
7
0
100
200
300
gi393743777 position (nts)

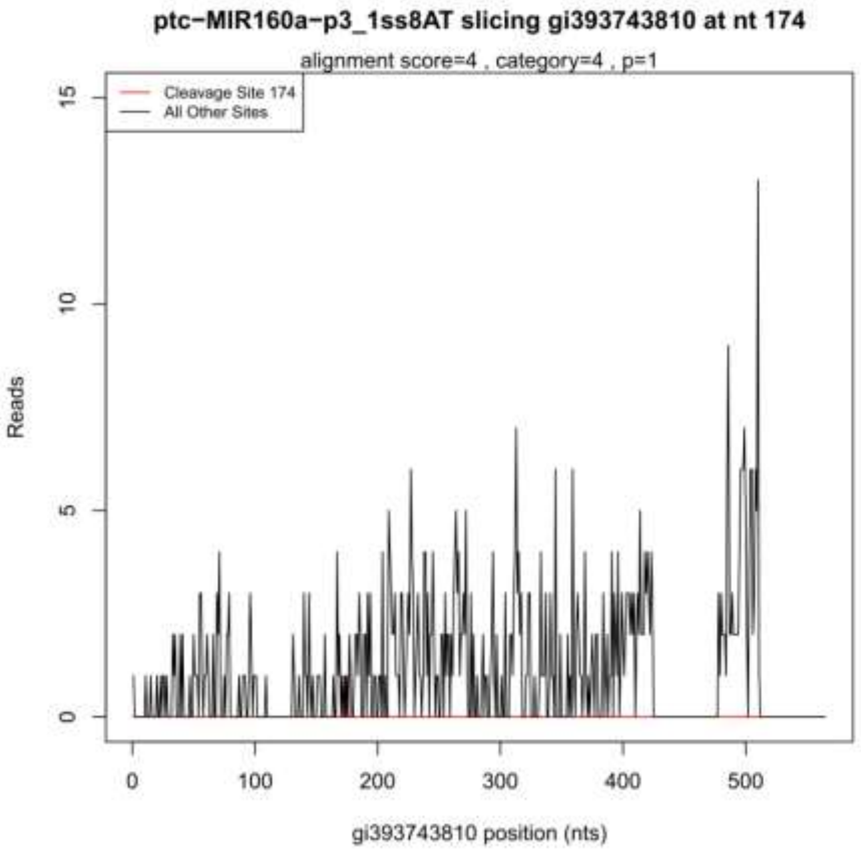
ptc−MIR160a−p3_1ss8AT slicing gi393743810 at nt 174
alignment score=4 , category=4 , p=1
Cleavage Site 174
All Other Sites
Reads
gi393743810 position (nts)

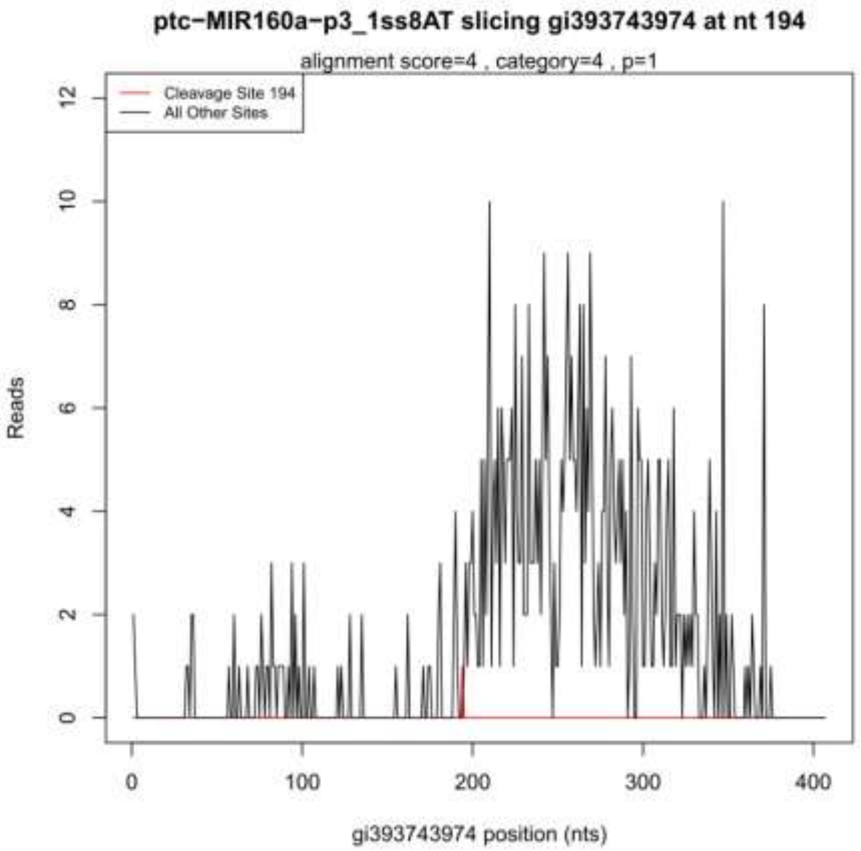
ptc−MIR160a−p3_1ss8AT slicing gi393743974 at nt 194
alignment score=4 , category=4 , p=1
Cleavage Site 194
All Other Sites
Reads
gi393743974 position (nts)

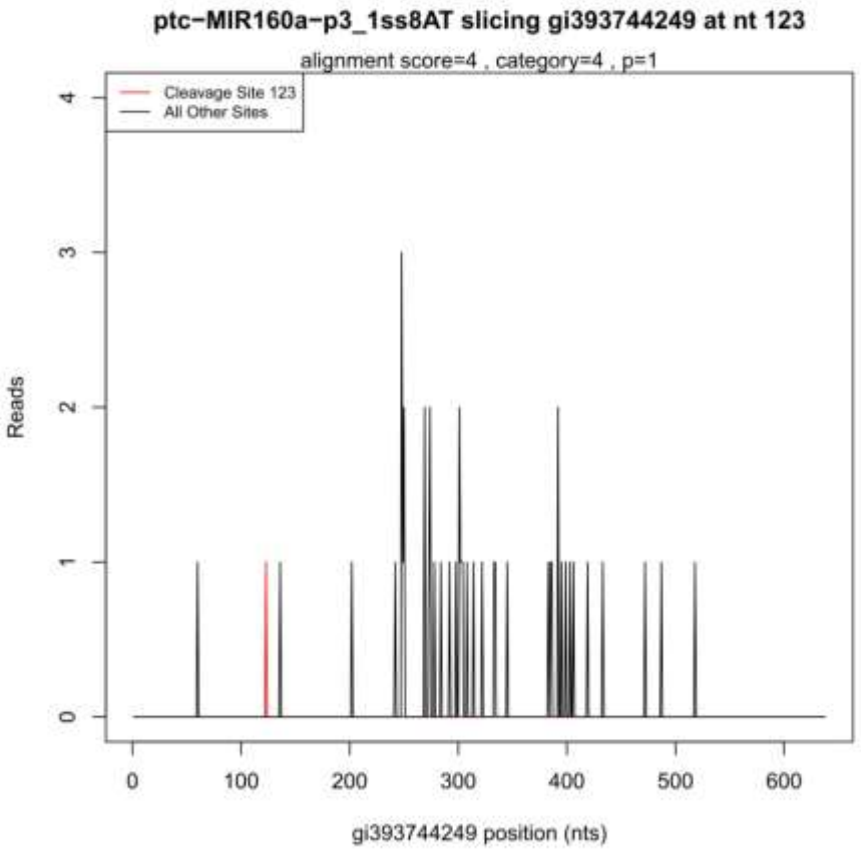
ptc−MIR160a−p3_1ss8AT slicing gi393744249 at nt 123
alignment score=4 , category=4 , p=1
Cleavage Site 123
All Other Sites
Reads
0
1
2
3
4
0
100
200
300
400
500
600
gi393744249 position (nts)

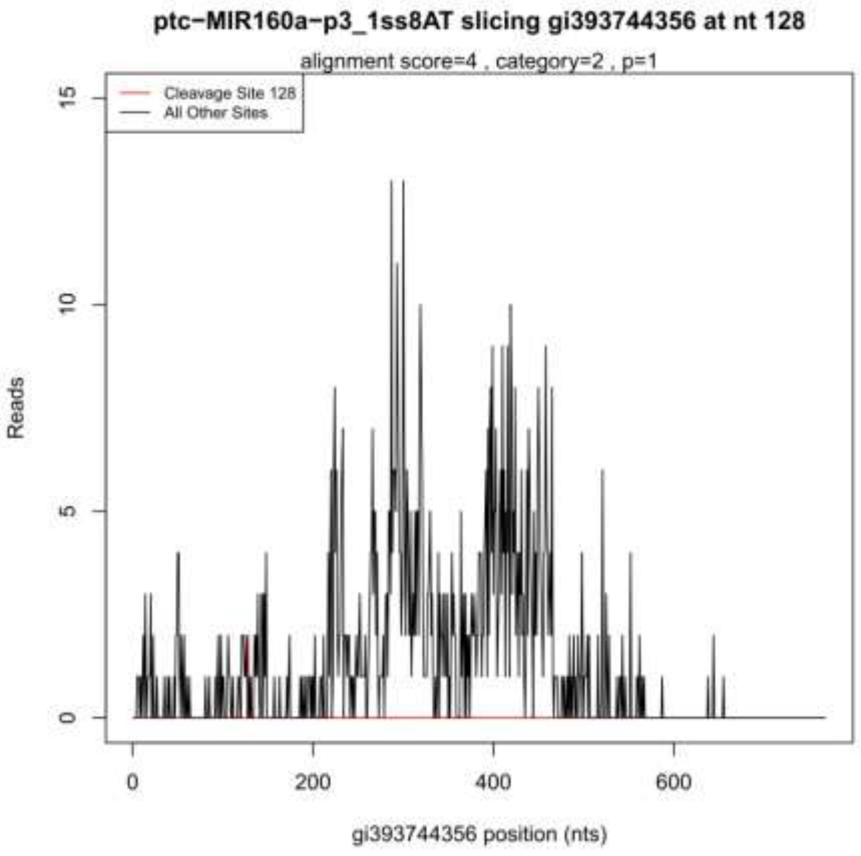
ptc−MIR160a−p3_1ss8AT slicing gi393744356 at nt 128
alignment score=4 , category=2 , p=1
Cleavage Site 128
All Other Sites
Reads
0
5
10
15
0
200
400
600
gi393744356 position (nts)

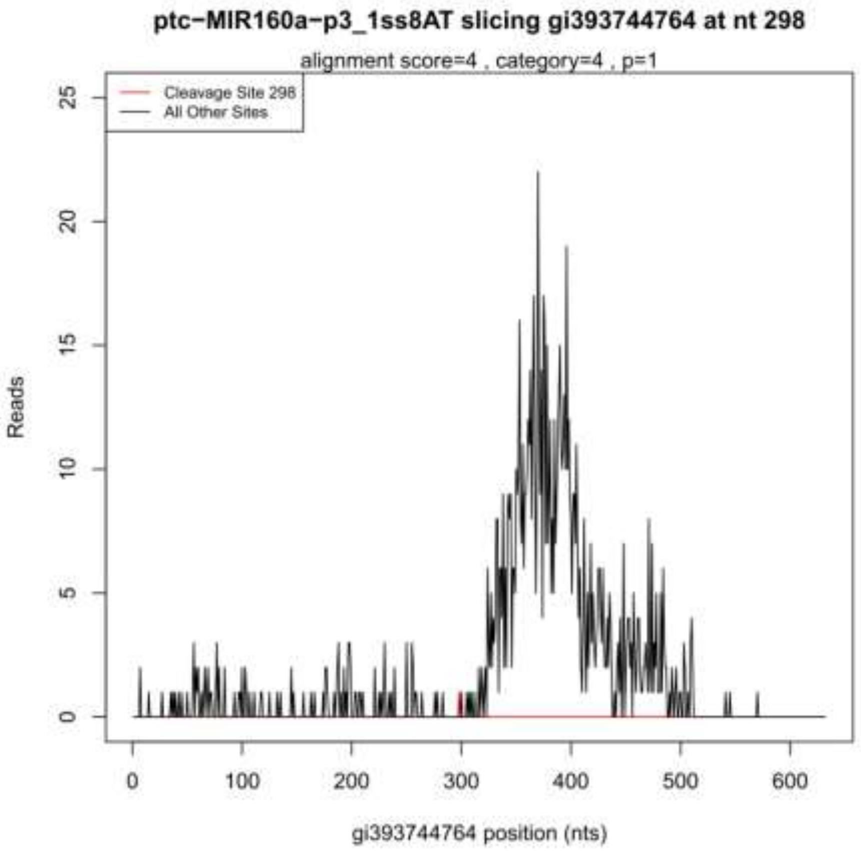
ptc−MIR160a−p3_1ss8AT slicing gi393744764 at nt 298
alignment score=4 , category=4 , p=1
Cleavage Site 298
All Other Sites
Reads
gi393744764 position (nts)

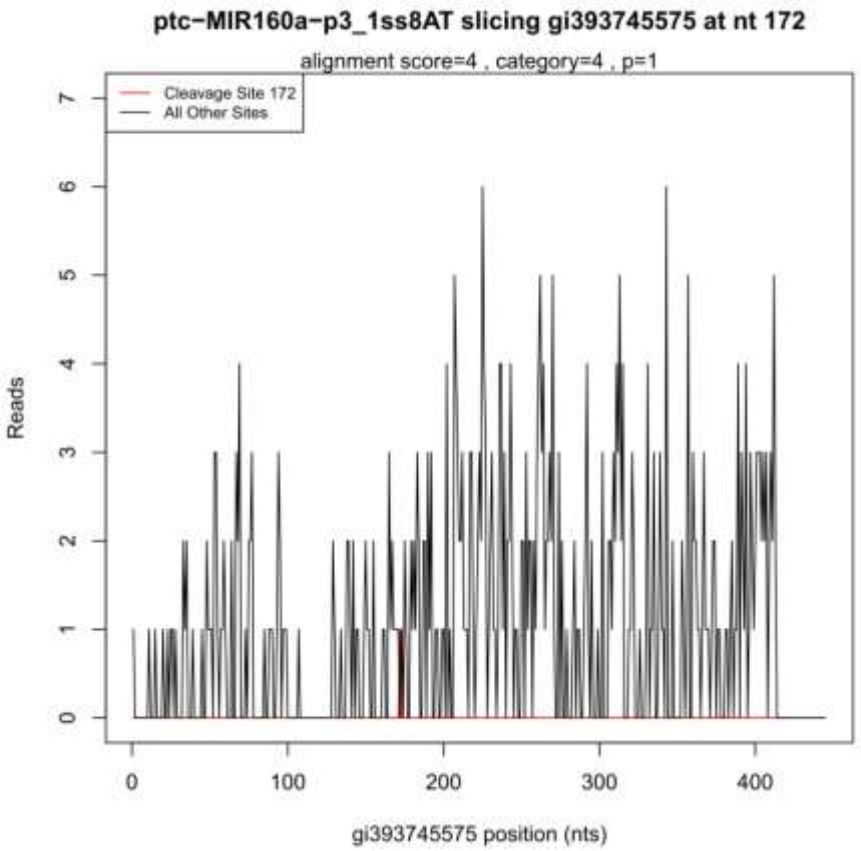
ptc−MIR160a−p3_1ss8AT slicing gi393745575 at nt 172
alignment score=4 , category=4 , p=1
Cleavage Site 172
All Other Sites
Reads
gi393745575 position (nts)

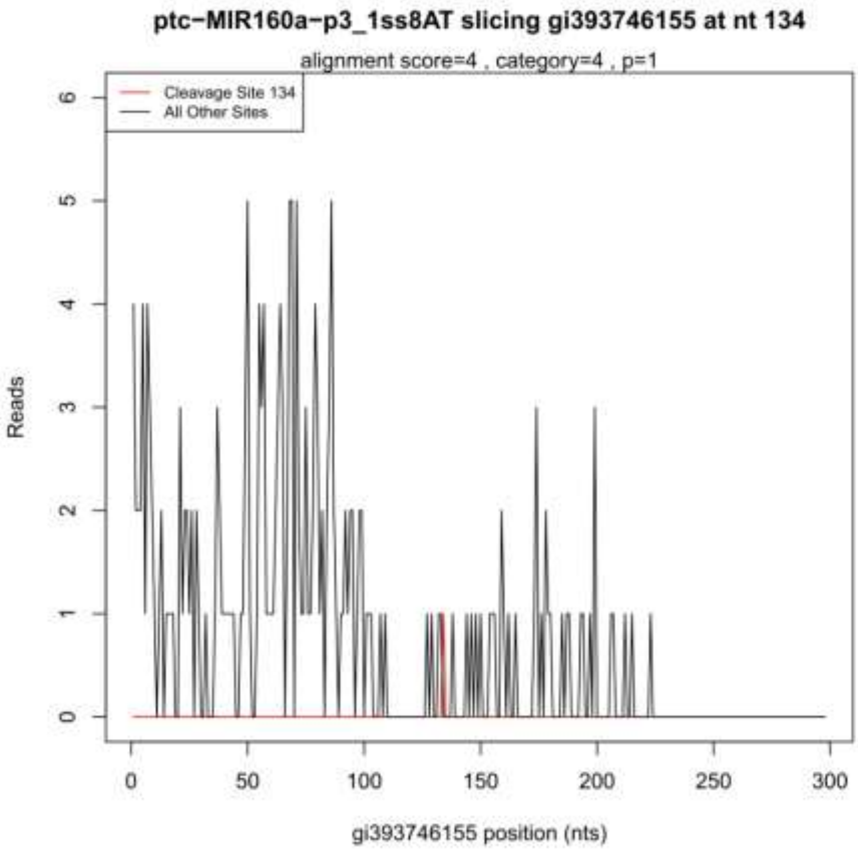

ptc−MIR160a−p3_1ss8AT slicing gi393746155 at nt 134
alignment score=4 , category=4 , p=1
Cleavage Site 134
All Other Sites
Reads
0
1
2
3
4
5
6
0
50
100
150
200
250
300
gi393746155 position (nts)

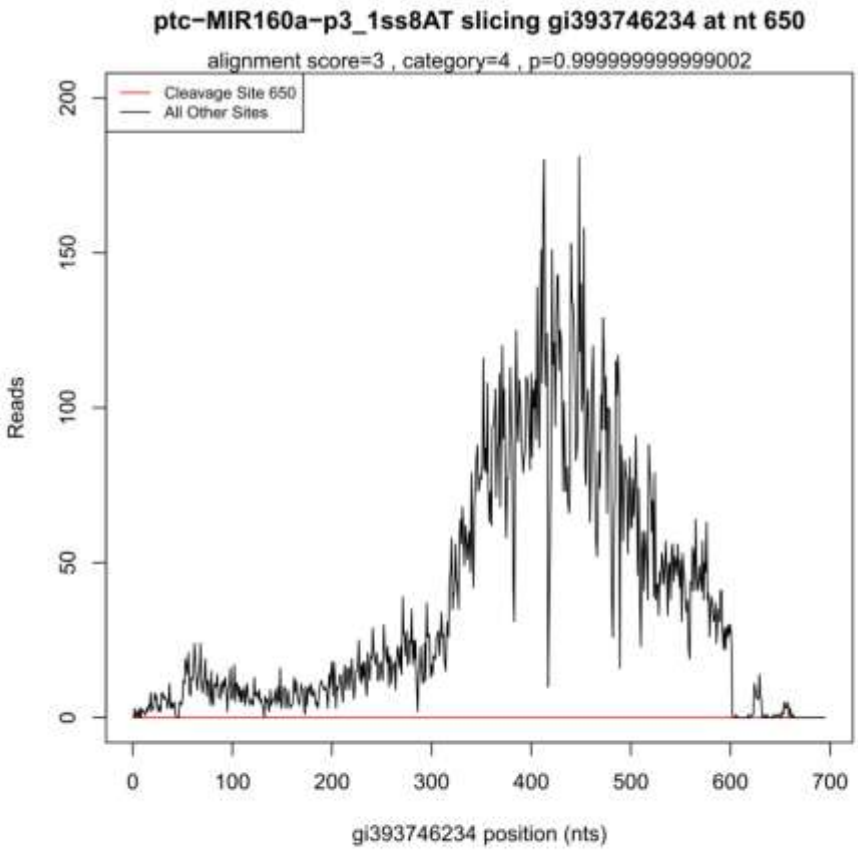

ptc−MIR160a−p3_1ss8AT slicing gi393746234 at nt 650
alignment score=3 , category=4 , p=0.999999999999002
Cleavage Site 650
All Other Sites
Reads
0
50
100
150
200
0
100
200
300
400
500
600
700
gi393746234 position (nts)

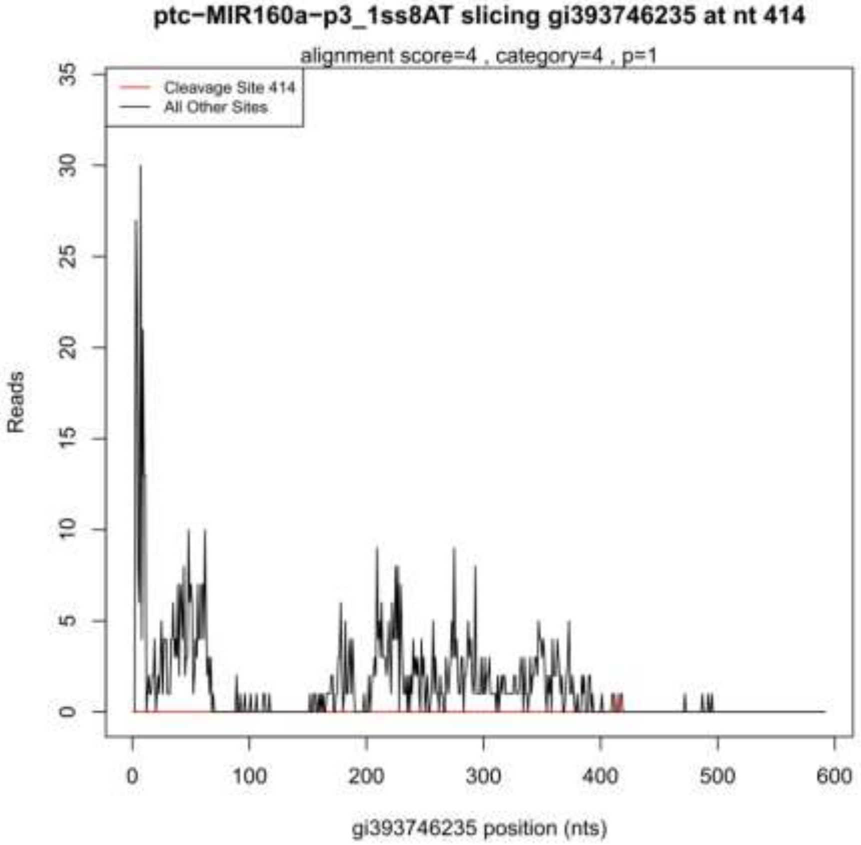
ptc−MIR160a−p3_1ss8AT slicing gi393746235 at nt 414
alignment score=4 , category=4 , p=1
Cleavage Site 414
All Other Sites
Reads
0
5
10
15
20
25
30
35
0
100
200
300
400
500
600
gi393746235 position (nts)

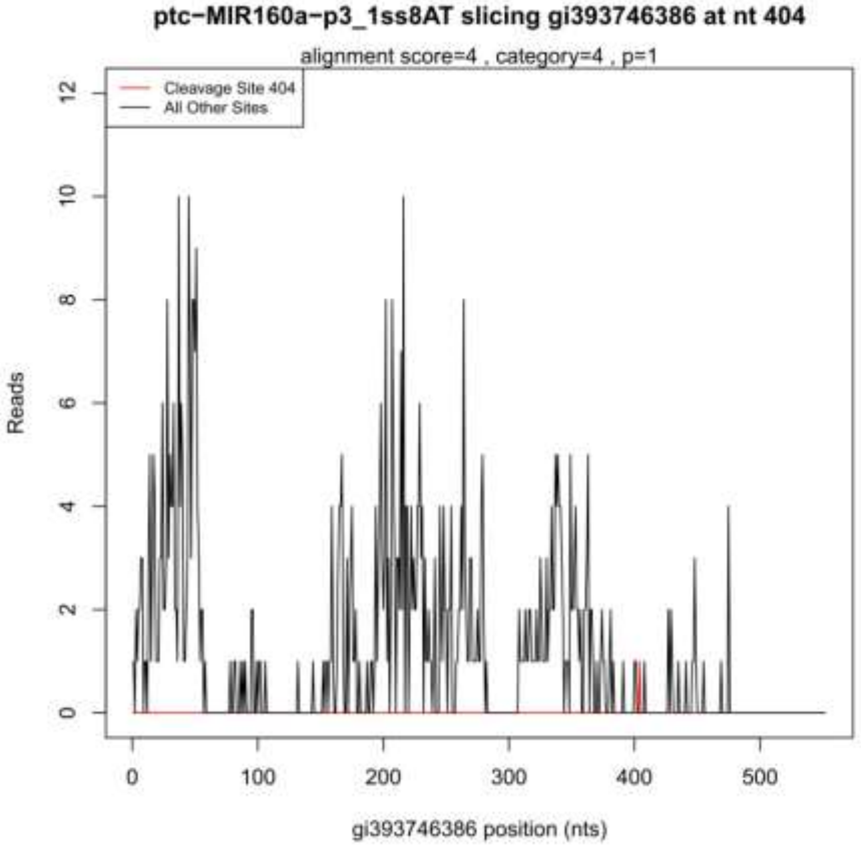
ptc−MIR160a−p3_1ss8AT slicing gi393746386 at nt 404
alignment score=4 , category=4 , p=1
Cleavage Site 404
All Other Sites
Reads
0
2
4
6
8
10
12
0
100
200
300
400
500
gi393746386 position (nts)

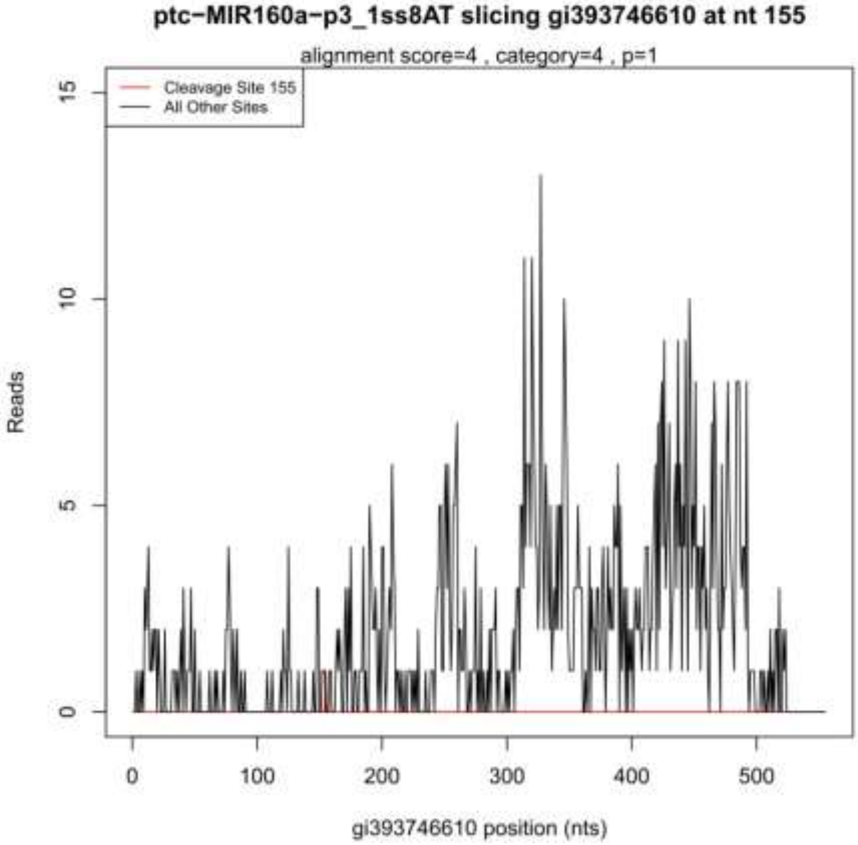
ptc−MIR160a−p3_1ss8AT slicing gi393746610 at nt 155
alignment score=4 , category=4 , p=1
Cleavage Site 155
All Other Sites
Reads
gi393746610 position (nts)

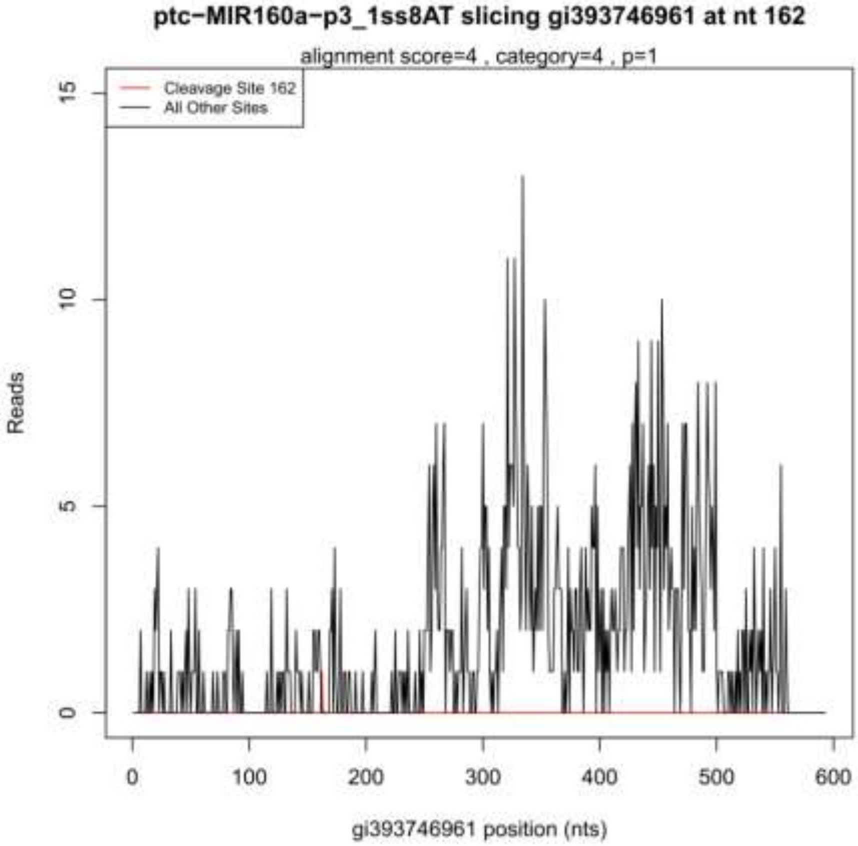
ptc−MIR160a−p3_1ss8AT slicing gi393746961 at nt 162
alignment score=4 , category=4 , p=1
Cleavage Site 162
All Other Sites
Reads
gi393746961 position (nts)

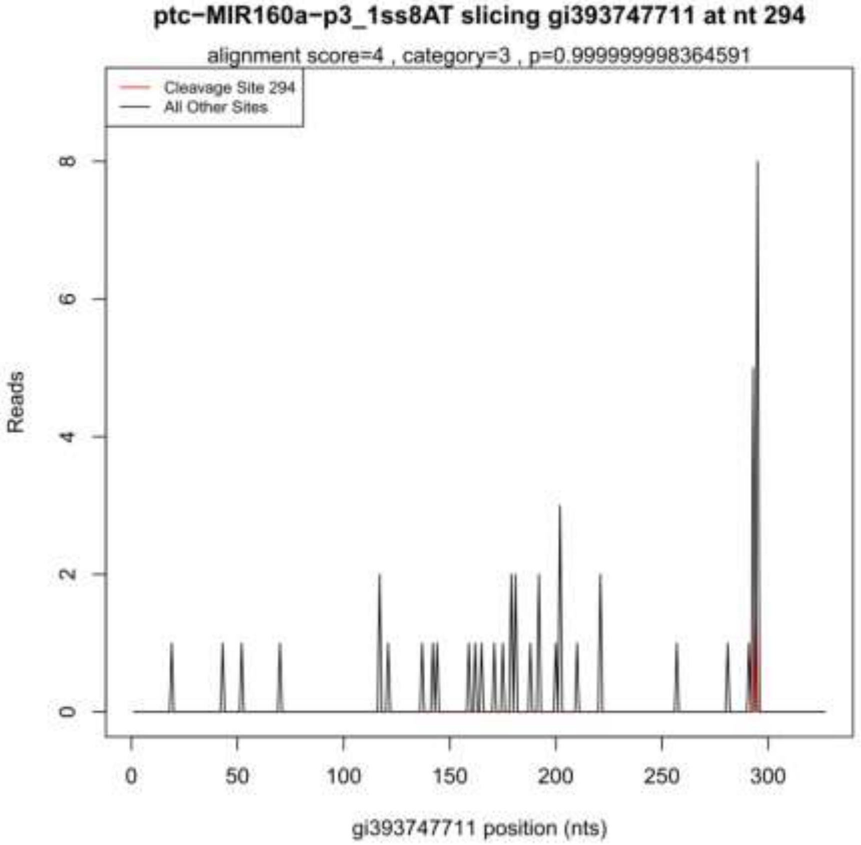
ptc−MIR160a−p3_1ss8AT slicing gi393747711 at nt 294
alignment score=4 , category=3 , p=0.999999998364591
Cleavage Site 294
All Other Sites
Reads
gi393747711 position (nts)

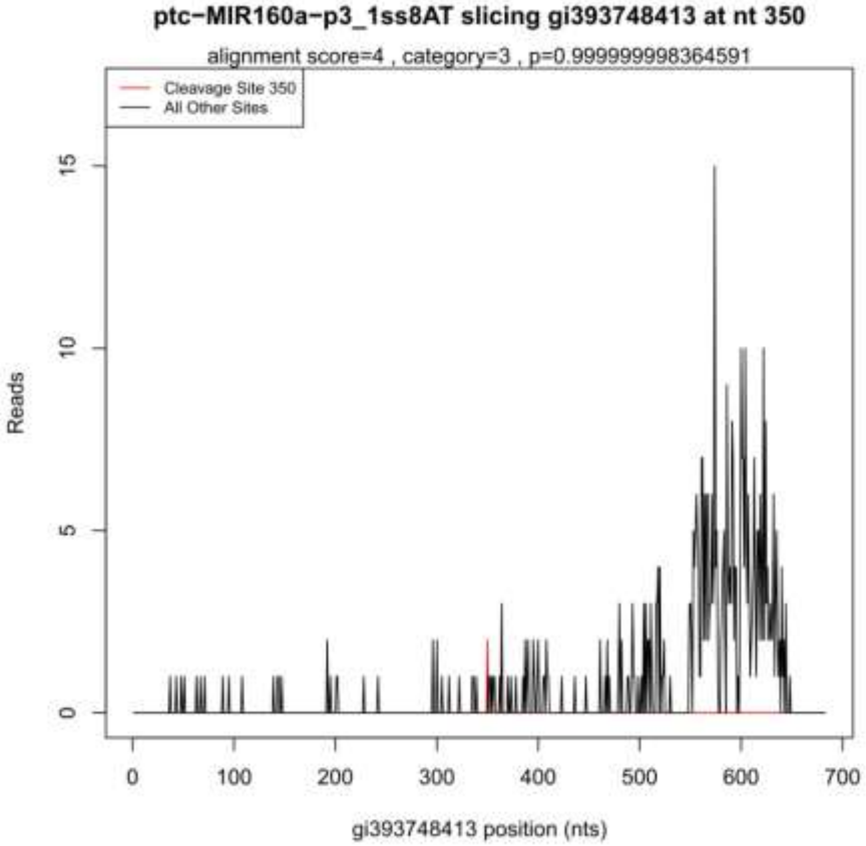
ptc−MIR160a−p3_1ss8AT slicing gi393748413 at nt 350
alignment score=4 , category=3 , p=0.999999998364591
Cleavage Site 350
All Other Sites
Reads
gi393748413 position (nts)

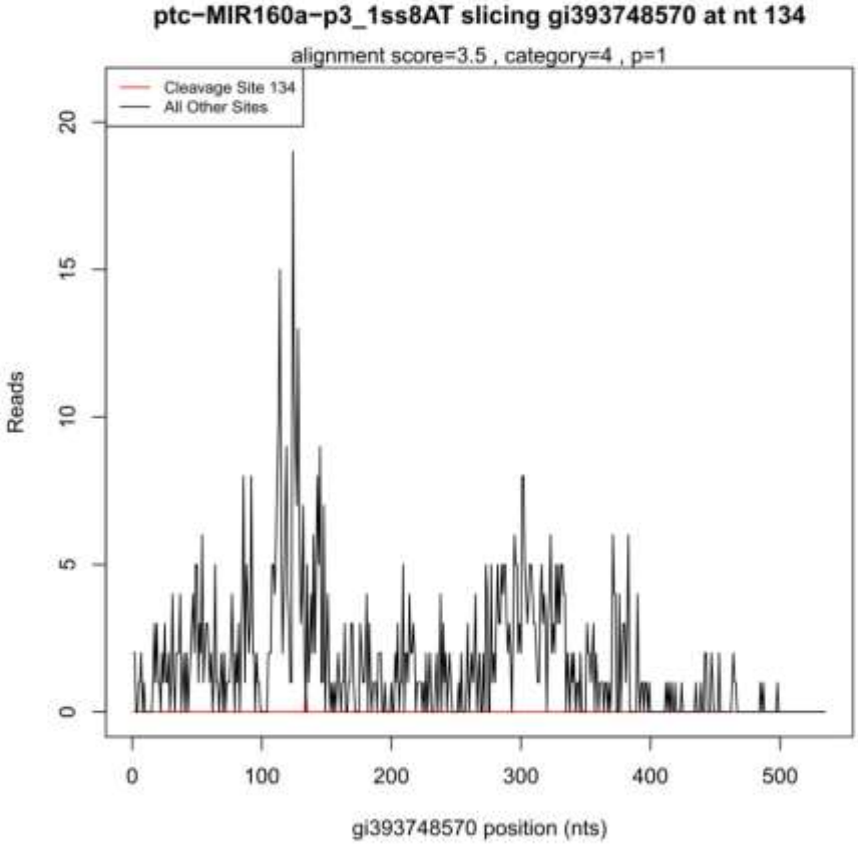
ptc−MIR160a−p3_1ss8AT slicing gi393748570 at nt 134
alignment score=3.5 , category=4 , p=1
Cleavage Site 134
All Other Sites
Reads
0
5
10
15
20
0
100
200
300
400
500
gi393748570 position (nts)

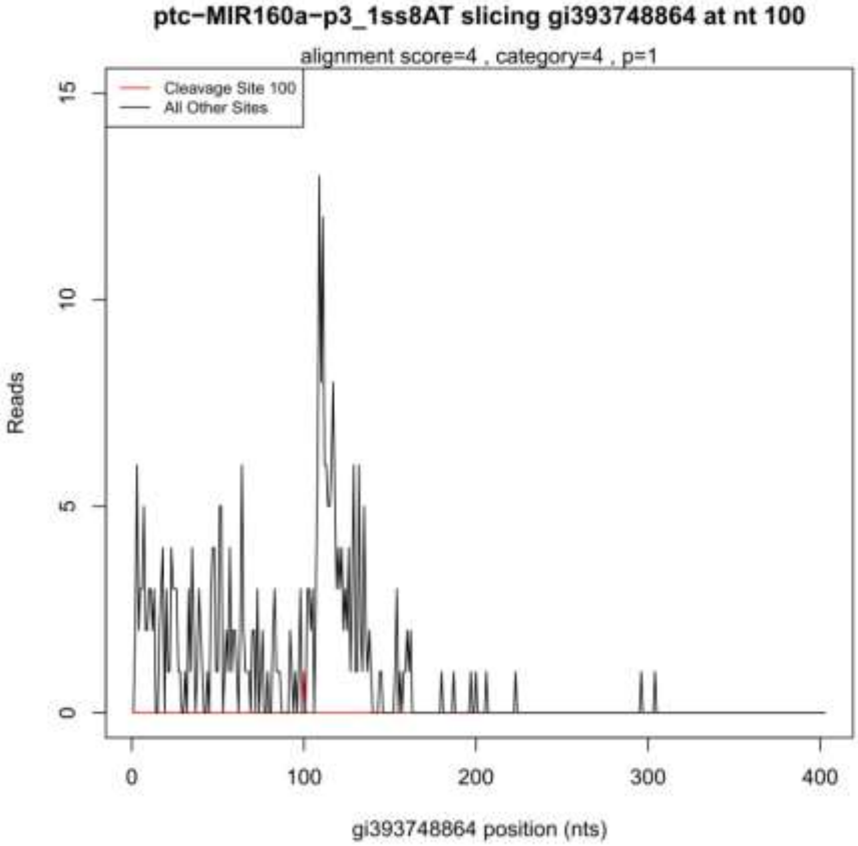
ptc−MIR160a−p3_1ss8AT slicing gi393748864 at nt 100
alignment score=4 , category=4 , p=1
Cleavage Site 100
All Other Sites
Reads
0
5
10
15
0
100
200
300
400
gi393748864 position (nts)

ptc−MIR160a−p3_1ss8AT slicing gi393748894 at nt 403

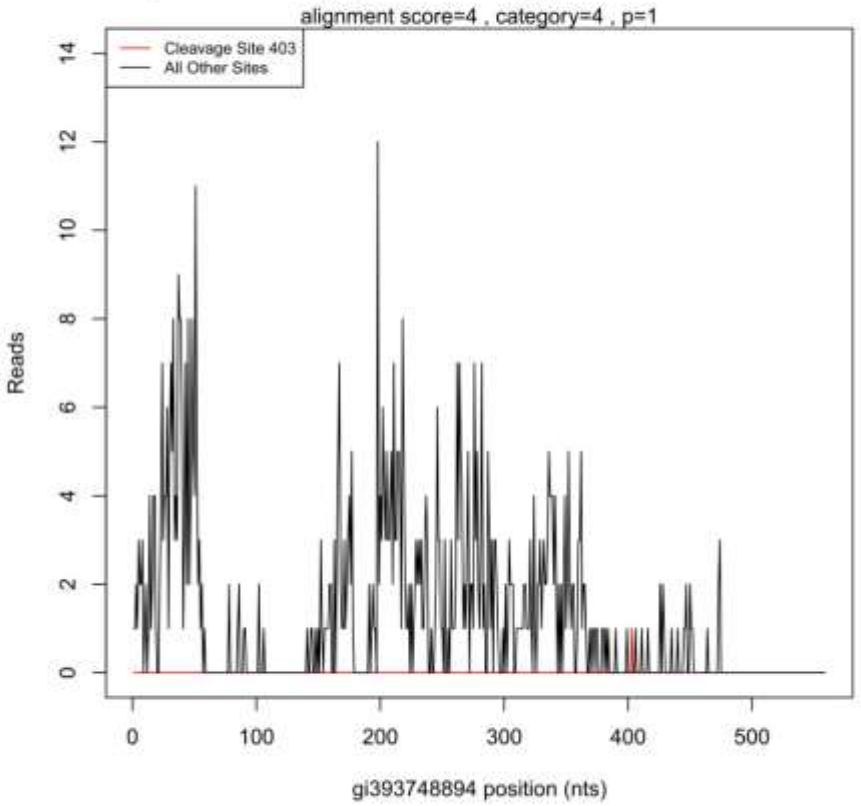

ptc−MIR160a−p3_1ss8AT slicing gi393749487 at nt 350

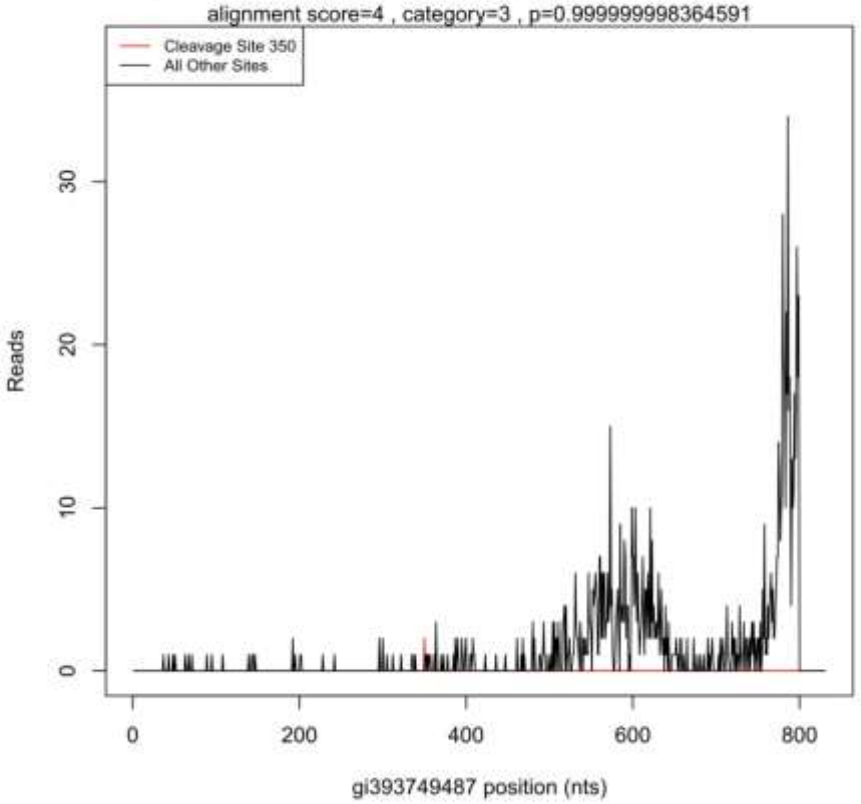

ptc-MIR160a-p3_1ss8AT slicing gi393749631 at nt 389

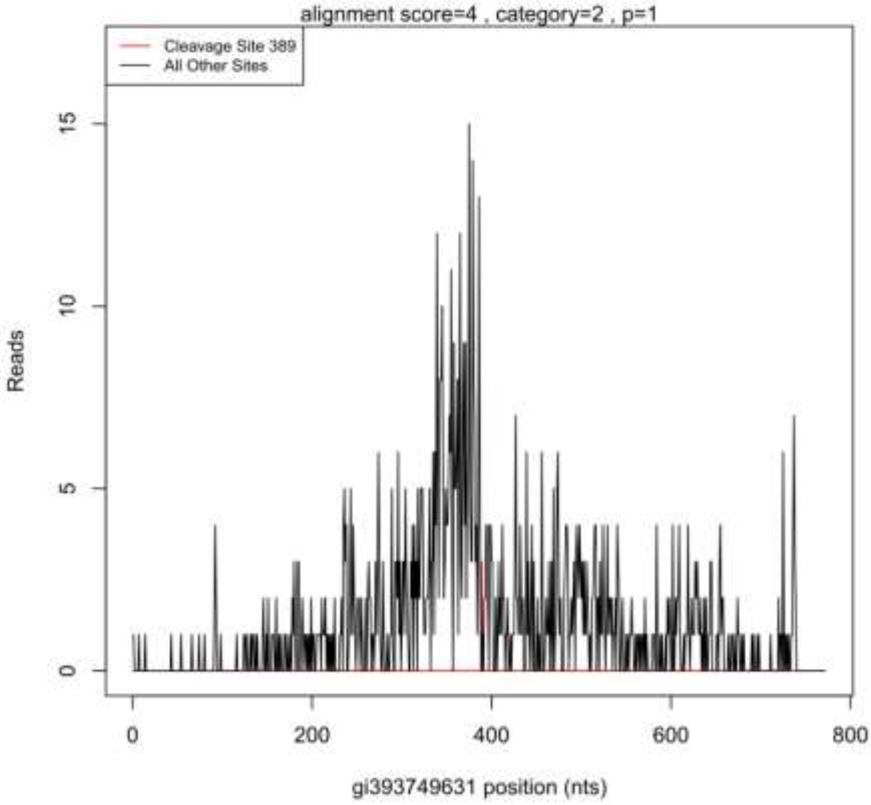

ptc-MIR160a-p3_1ss8AT slicing gi393750300 at nt 400

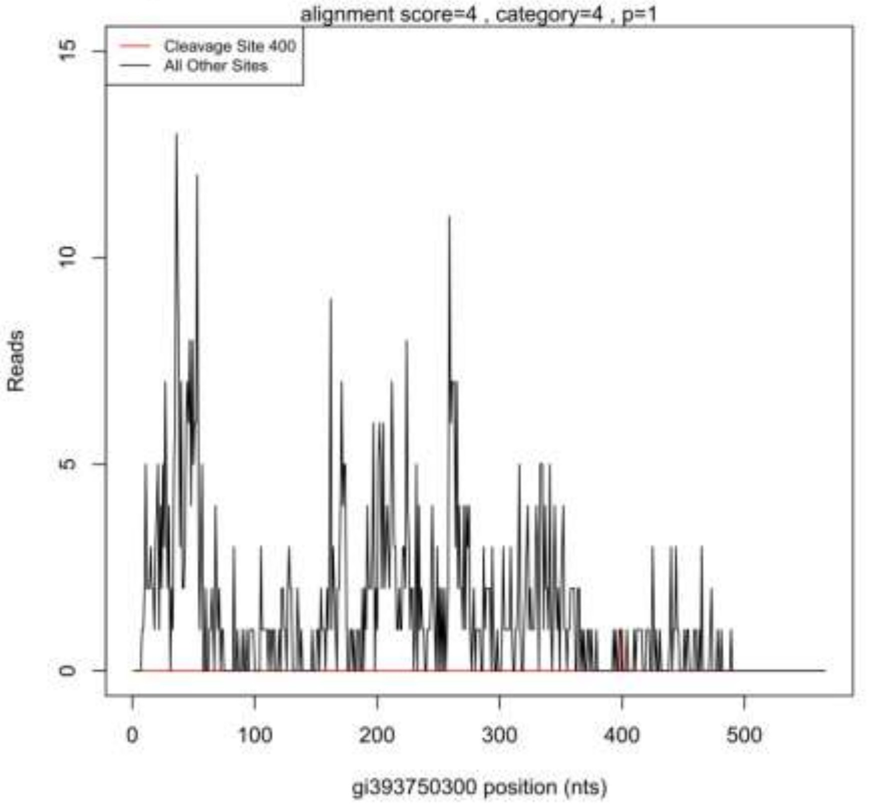

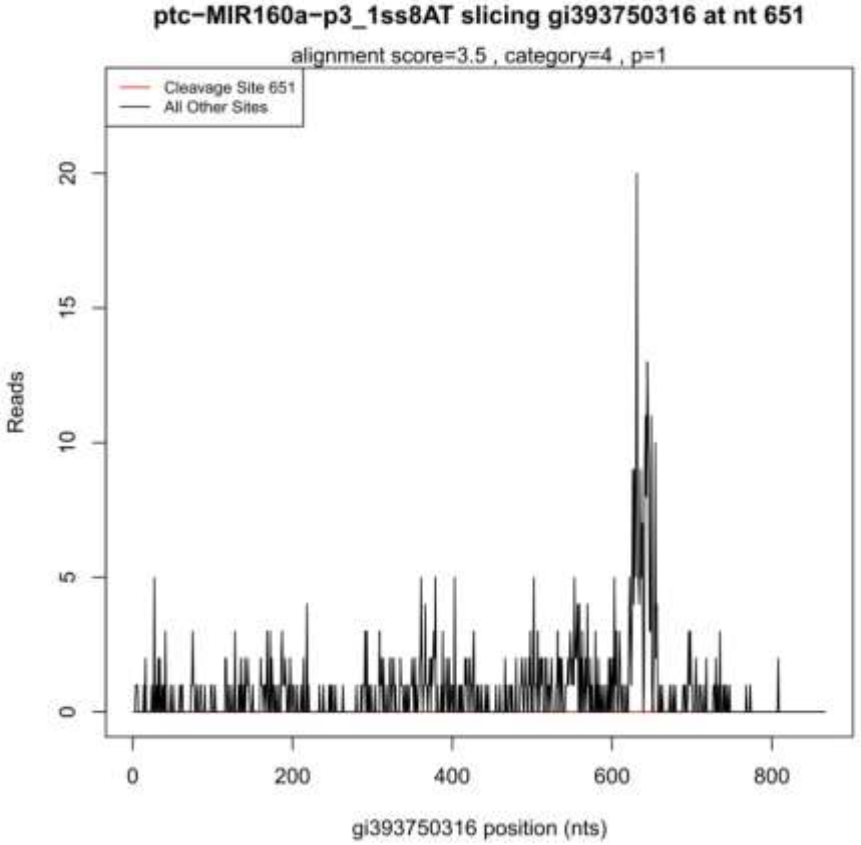
ptc−MIR160a−p3_1ss8AT slicing gi393750316 at nt 651
alignment score=3.5 , category=4 , p=1
Cleavage Site 651
All Other Sites
Reads
0
5
10
15
20
0
200
400
600
800
gi393750316 position (nts)

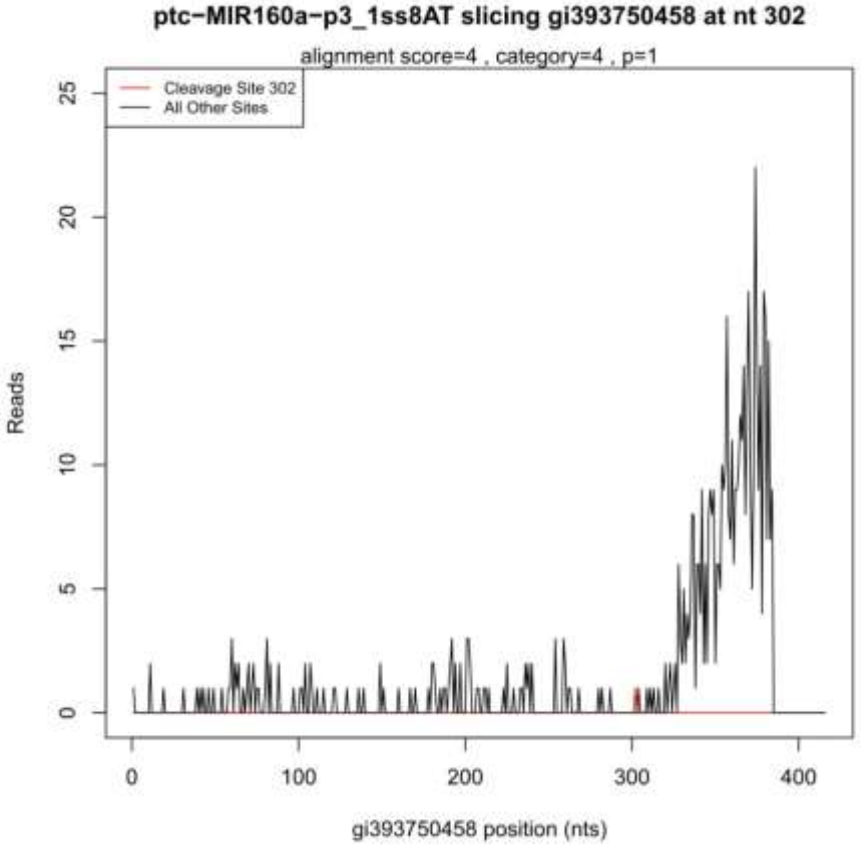
ptc−MIR160a−p3_1ss8AT slicing gi393750458 at nt 302
alignment score=4 , category=4 , p=1
Cleavage Site 302
All Other Sites
Reads
0
5
10
15
20
25
0
100
200
300
400
gi393750458 position (nts)

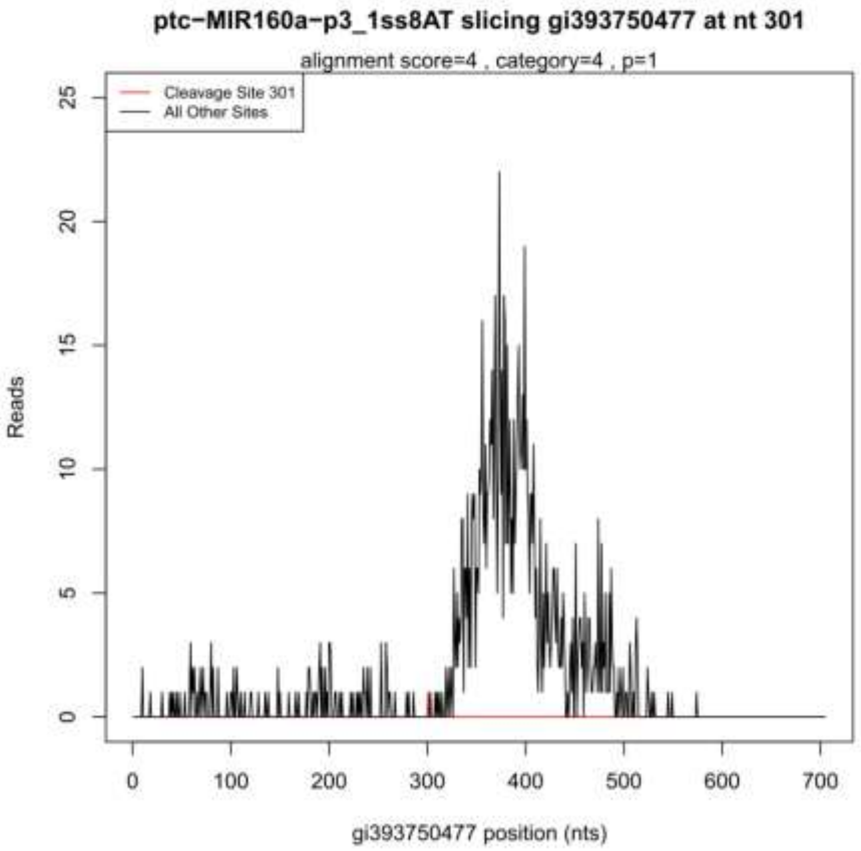
ptc−MIR160a−p3_1ss8AT slicing gi393750477 at nt 301
alignment score=4 , category=4 , p=1
Cleavage Site 301
All Other Sites
Reads
0
5
10
15
20
25
0
100
200
300
400
500
600
700
gi393750477 position (nts)

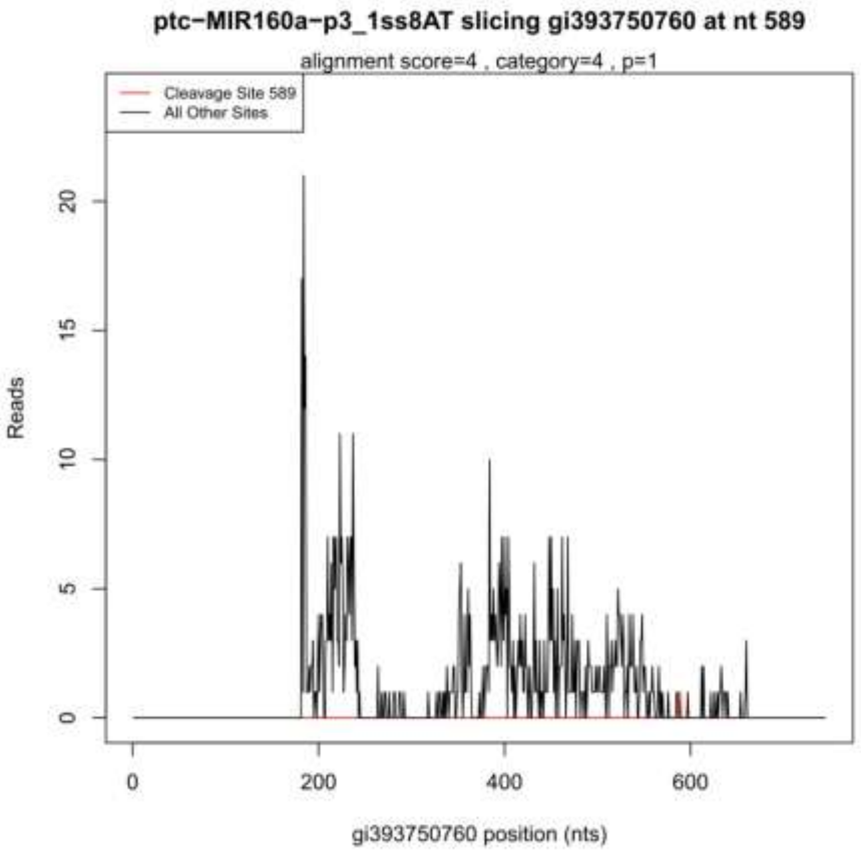
ptc−MIR160a−p3_1ss8AT slicing gi393750760 at nt 589
alignment score=4 , category=4 , p=1
Cleavage Site 589
All Other Sites
Reads
0
5
10
15
20
0
200
400
600
gi393750760 position (nts)

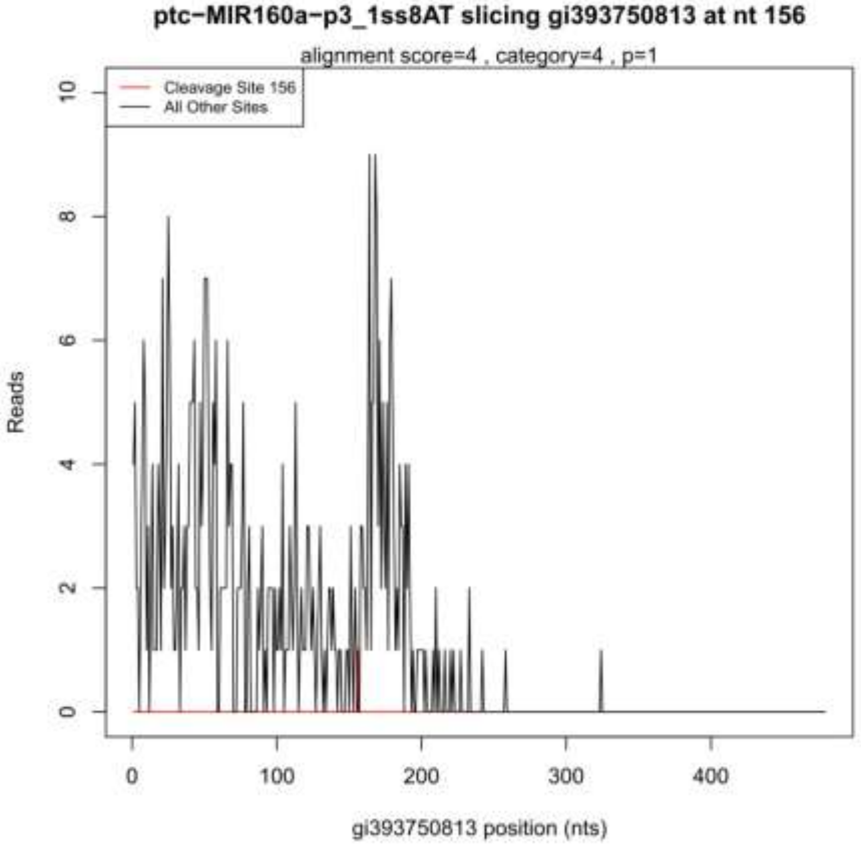

ptc−MIR160a−p3_1ss8AT slicing gi393750813 at nt 156
alignment score=4 , category=4 , p=1
Cleavage Site 156
All Other Sites
Reads
0
2
4
6
8
10
0
100
200
300
400
gi393750813 position (nts)

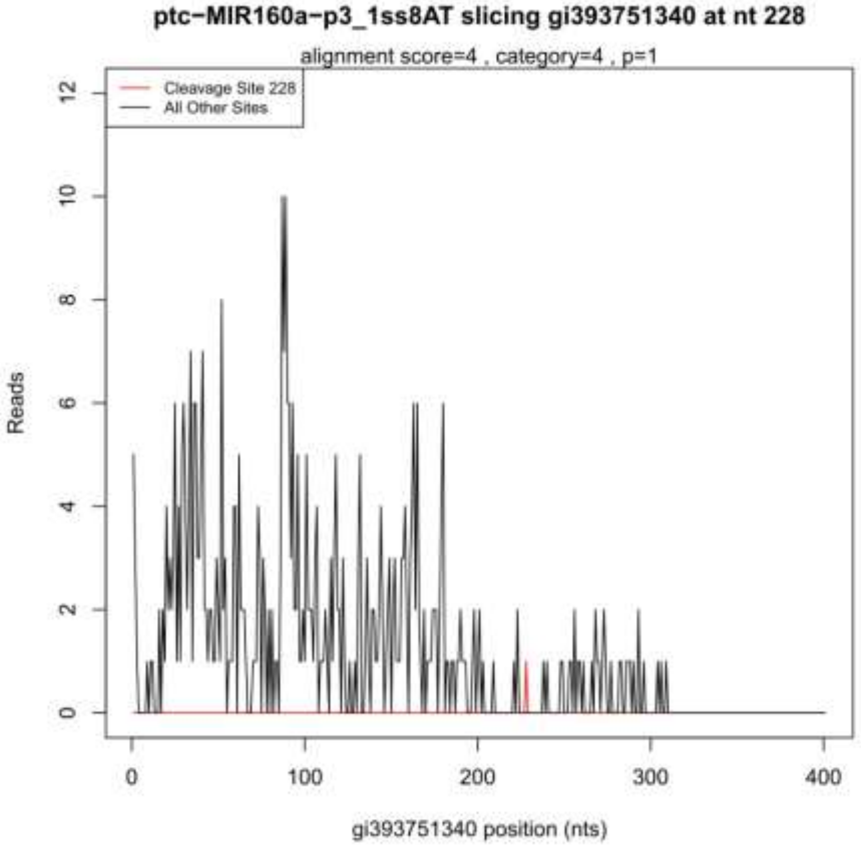

ptc−MIR160a−p3_1ss8AT slicing gi393751340 at nt 228
alignment score=4 , category=4 , p=1
Cleavage Site 228
All Other Sites
Reads
0
2
4
6
8
10
12
0
100
200
300
400
gi393751340 position (nts)

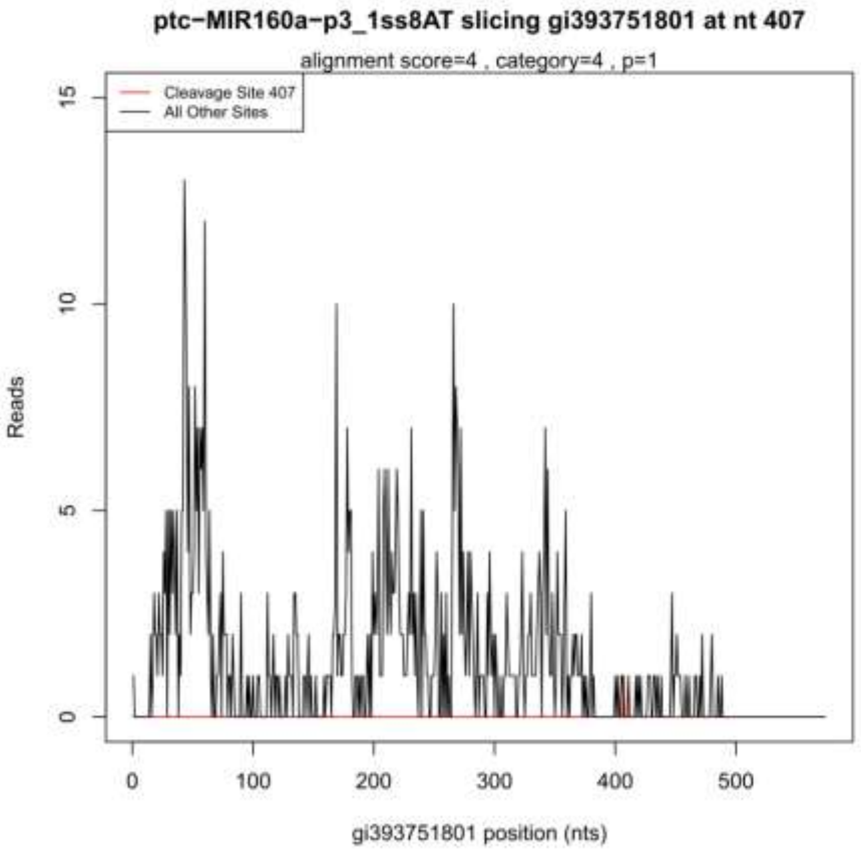

ptc−MIR160a−p3_1ss8AT slicing gi393751801 at nt 407
alignment score=4 , category=4 , p=1
Cleavage Site 407
All Other Sites
Reads
0
5
10
15
0
100
200
300
400
500
gi393751801 position (nts)

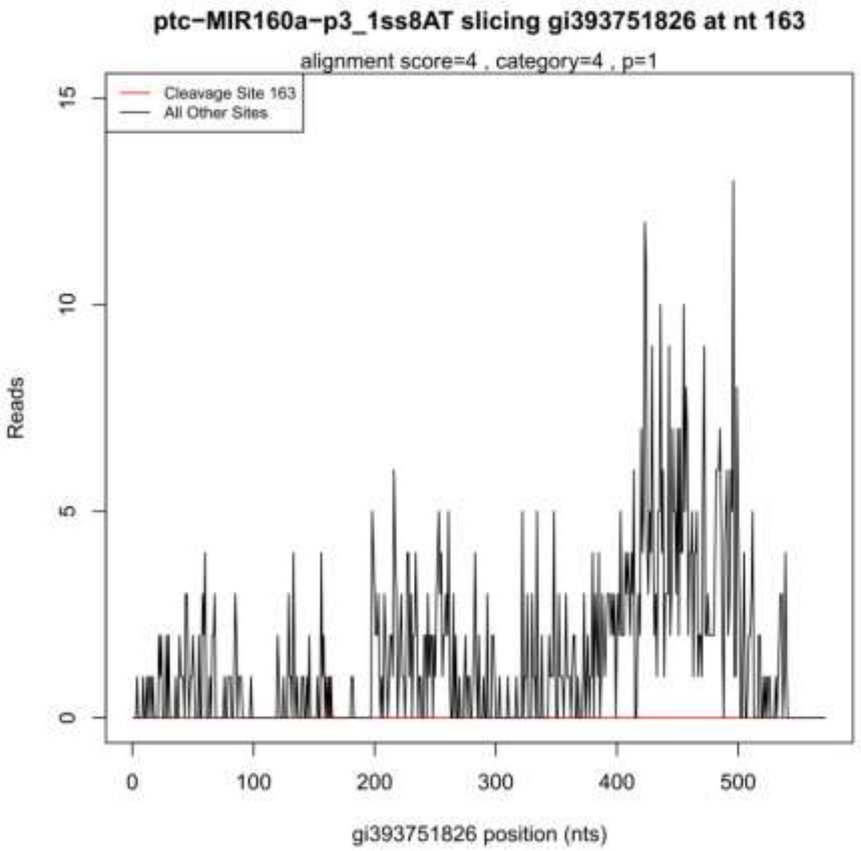

ptc−MIR160a−p3_1ss8AT slicing gi393751826 at nt 163
alignment score=4 , category=4 , p=1
Cleavage Site 163
All Other Sites
Reads
0
5
10
15
0
100
200
300
400
500
gi393751826 position (nts)

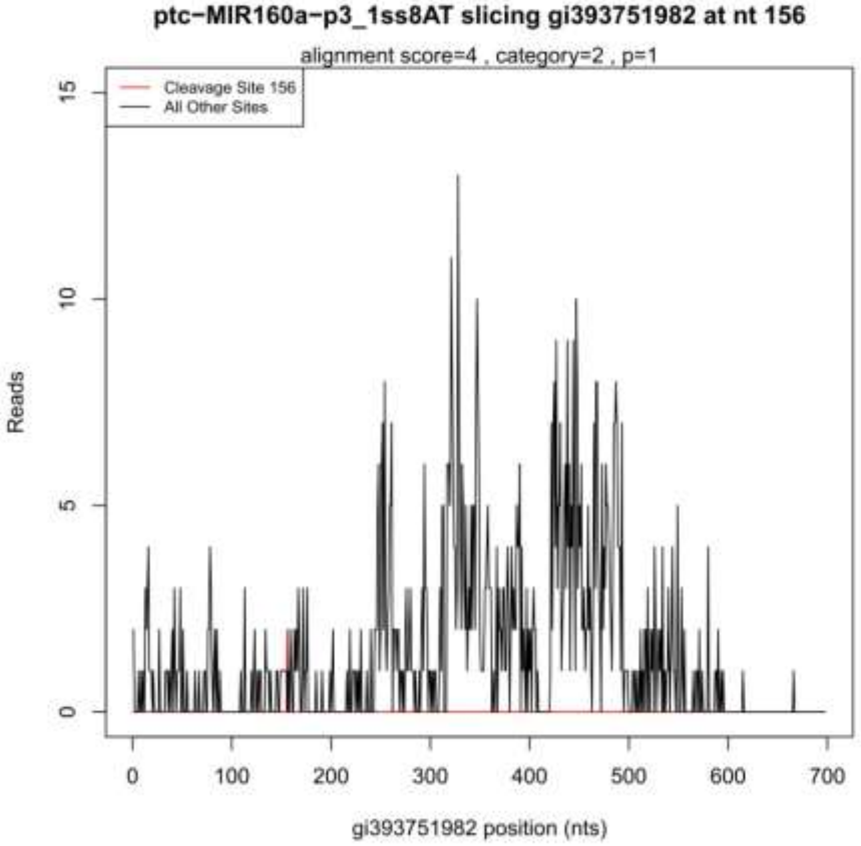
ptc-MIR160a-p3_1ss8AT slicing gi393751982 at nt 156
alignment score=4 , category=2 , p=1
Cleavage Site 156
All Other Sites
Reads
0
5
10
15
0
100
200
300
400
500
600
700
gi393751982 position (nts)

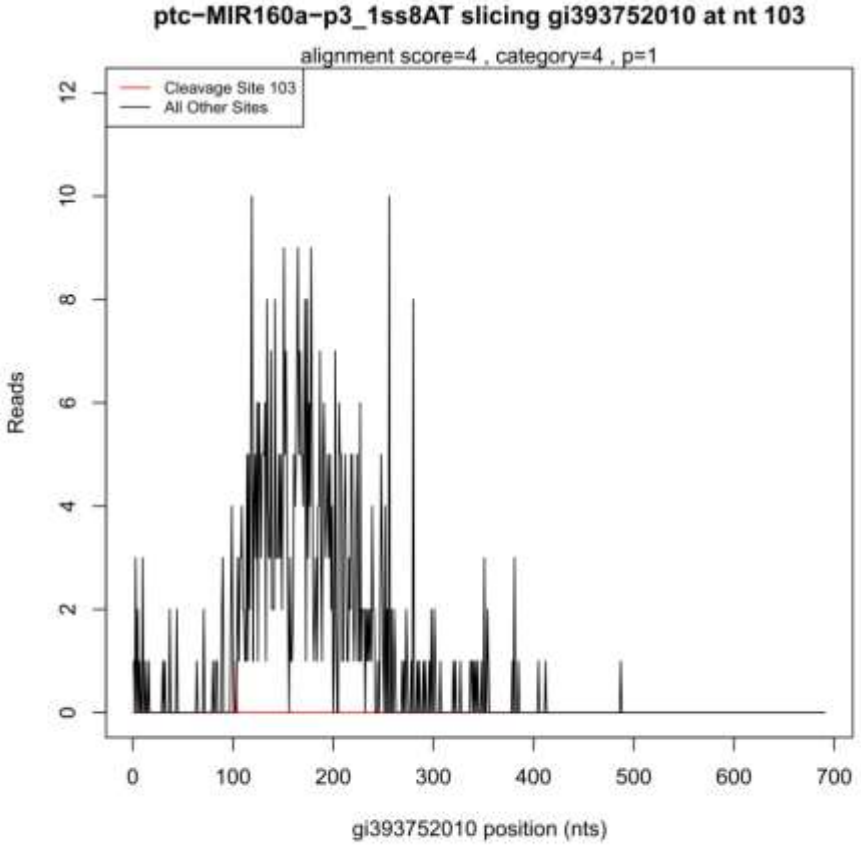
ptc-MIR160a-p3_1ss8AT slicing gi393752010 at nt 103
alignment score=4 , category=4 , p=1
Cleavage Site 103
All Other Sites
Reads
0
2
4
6
8
10
12
0
100
200
300
400
500
600
700
gi393752010 position (nts)

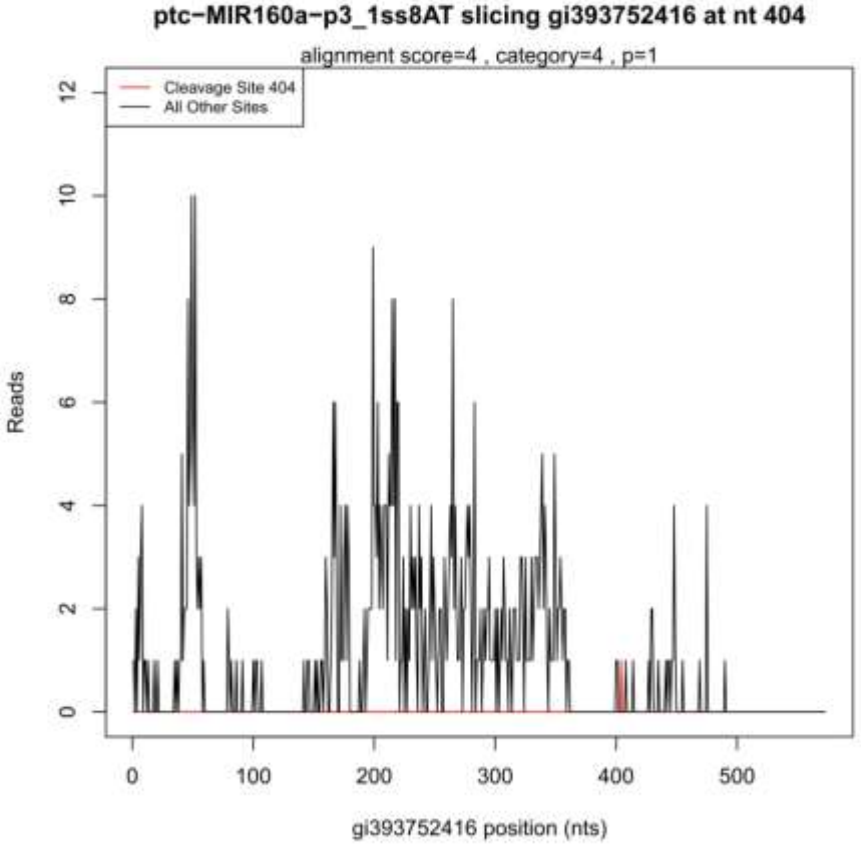
ptc−MIR160a−p3_1ss8AT slicing gi393752416 at nt 404
alignment score=4 , category=4 , p=1
Cleavage Site 404
All Other Sites
Reads
0
2
4
6
8
10
12
0
100
200
300
400
500
gi393752416 position (nts)

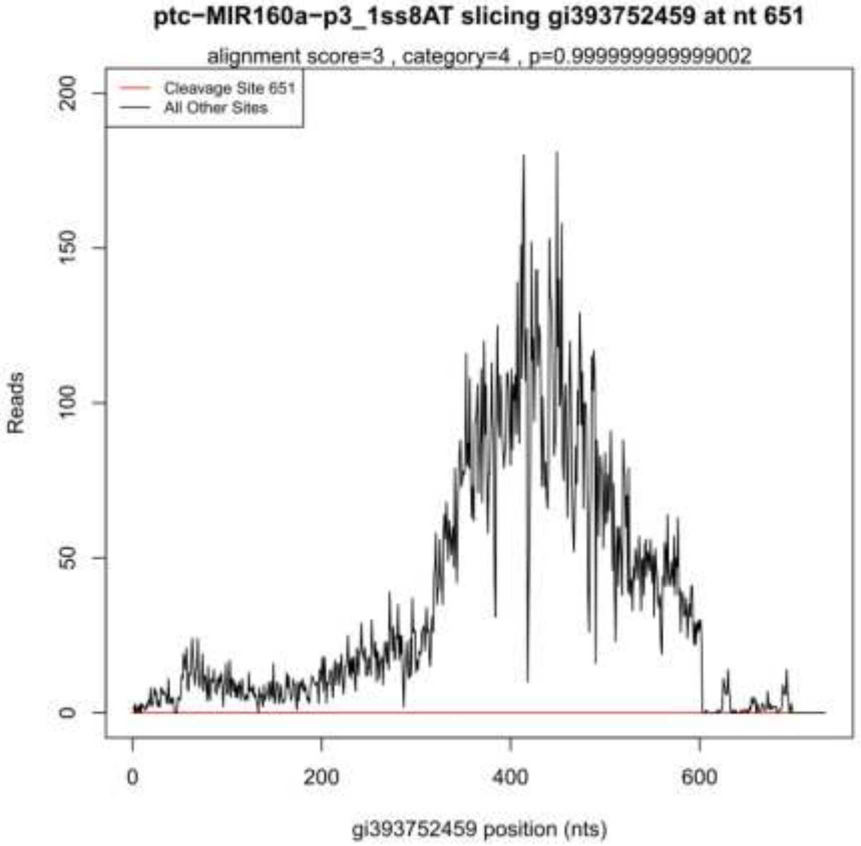
ptc−MIR160a−p3_1ss8AT slicing gi393752459 at nt 651
alignment score=3 , category=4 , p=0.999999999999002
Cleavage Site 651
All Other Sites
Reads
0
50
100
150
200
0
200
400
600
gi393752459 position (nts)

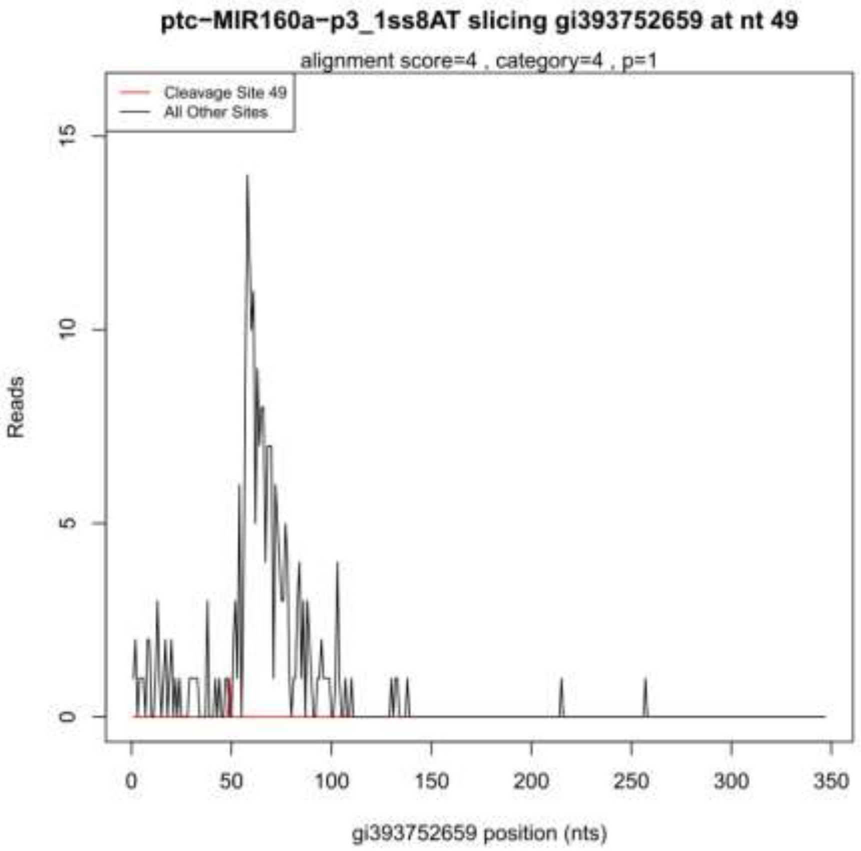

ptc−MIR160a−p3_1ss8AT slicing gi393752659 at nt 49
alignment score=4 , category=4 , p=1
Cleavage Site 49
All Other Sites
Reads
0
5
10
15
0
50
100
150
200
250
300
350
gi393752659 position (nts)

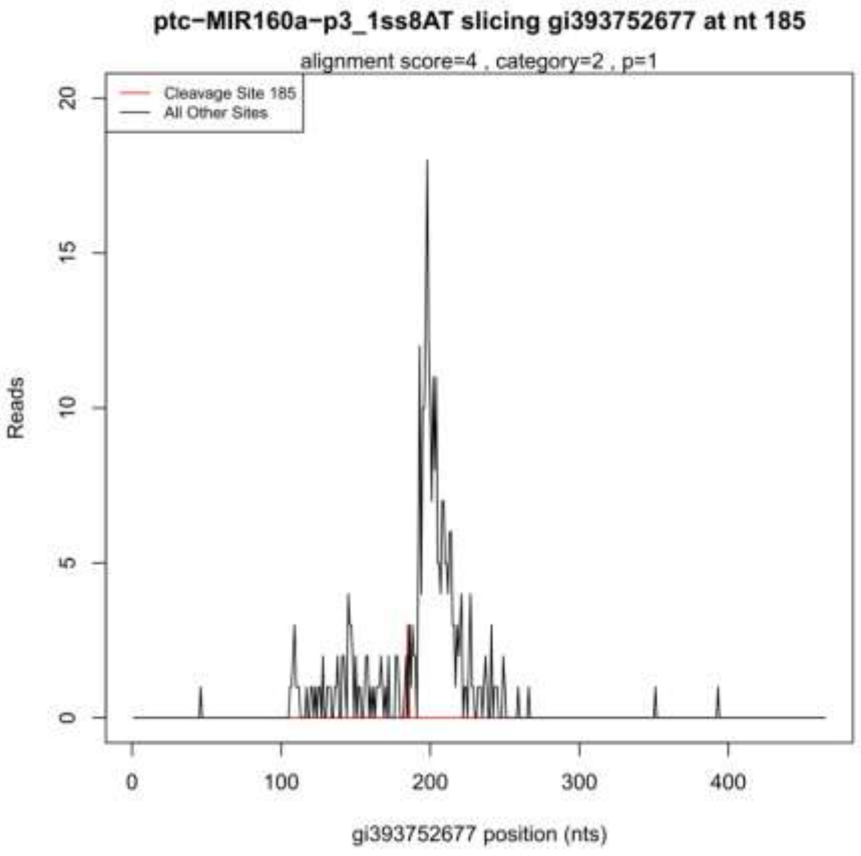

ptc−MIR160a−p3_1ss8AT slicing gi393752677 at nt 185
alignment score=4 , category=2 , p=1
Cleavage Site 185
All Other Sites
Reads
0
5
10
15
20
0
100
200
300
400
gi393752677 position (nts)

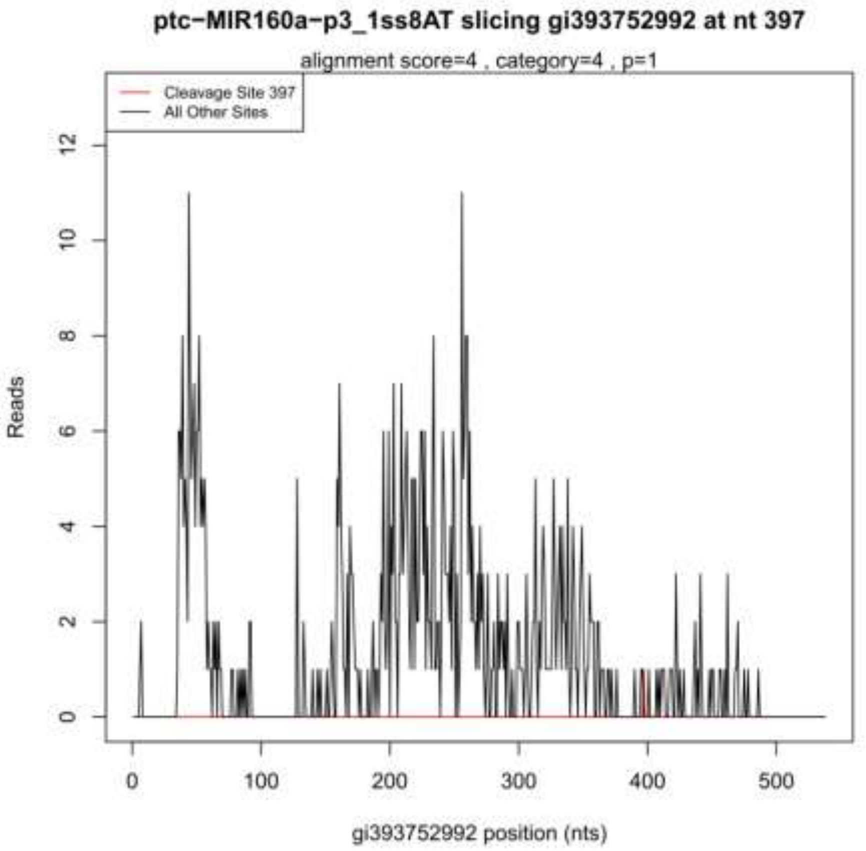

ptc−MIR160a−p3_1ss8AT slicing gi393752992 at nt 397
alignment score=4 , category=4 , p=1
Cleavage Site 397
All Other Sites
Reads
gi393752992 position (nts)

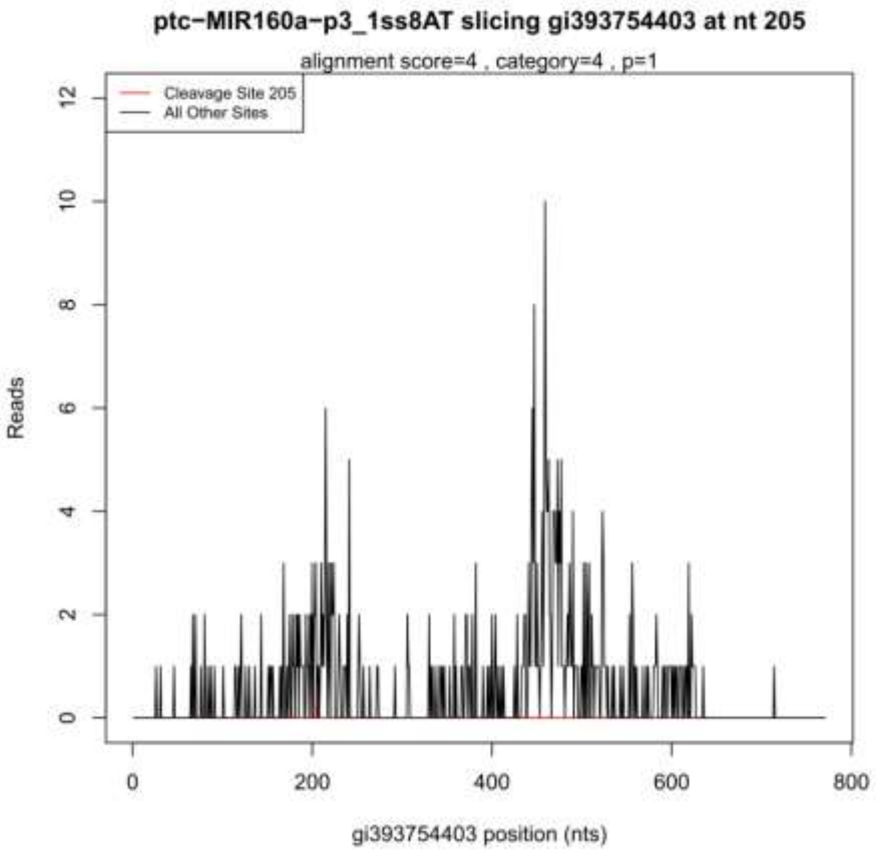

ptc−MIR160a−p3_1ss8AT slicing gi393754403 at nt 205
alignment score=4 , category=4 , p=1
Cleavage Site 205
All Other Sites
Reads
gi393754403 position (nts)

ptc-MIR160a-p3_1ss8AT slicing gi393754523 at nt 403

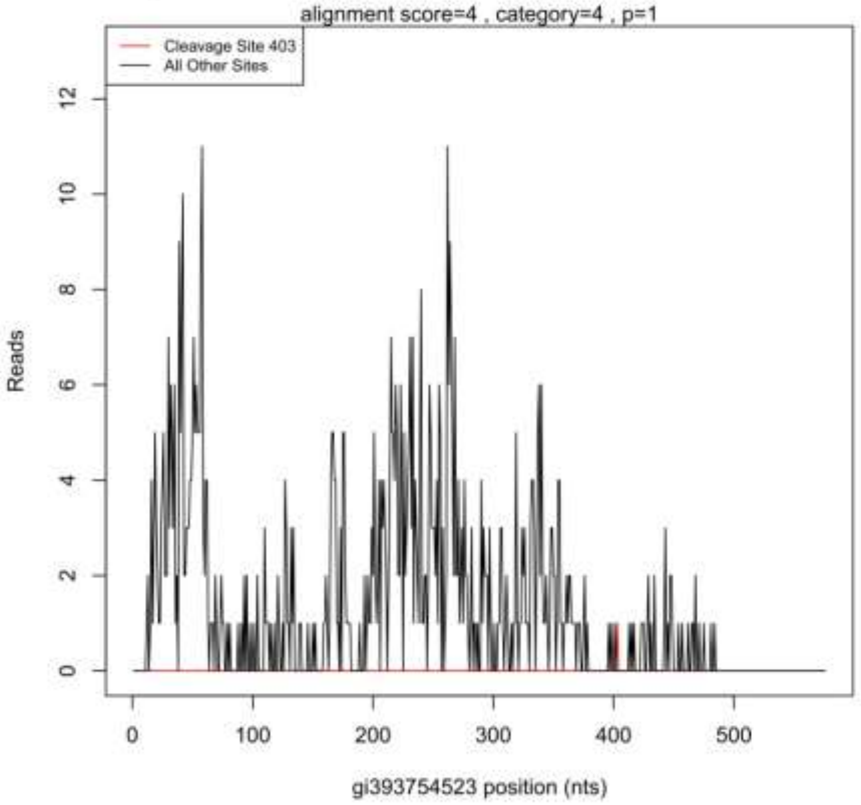

ptc-MIR160a-p3_1ss8AT slicing gi393754558 at nt 416

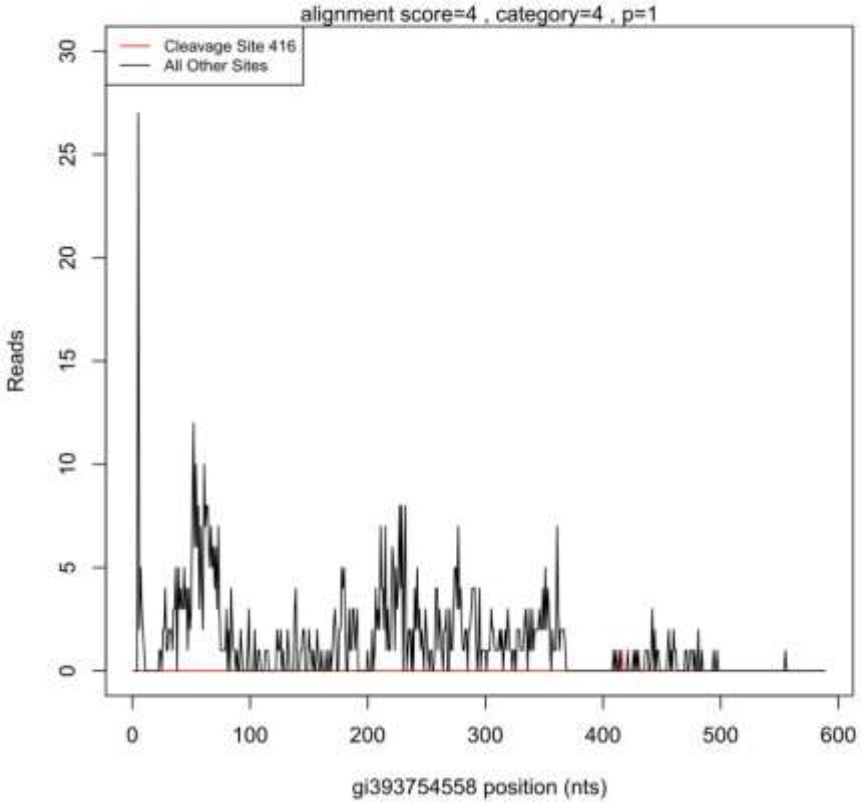

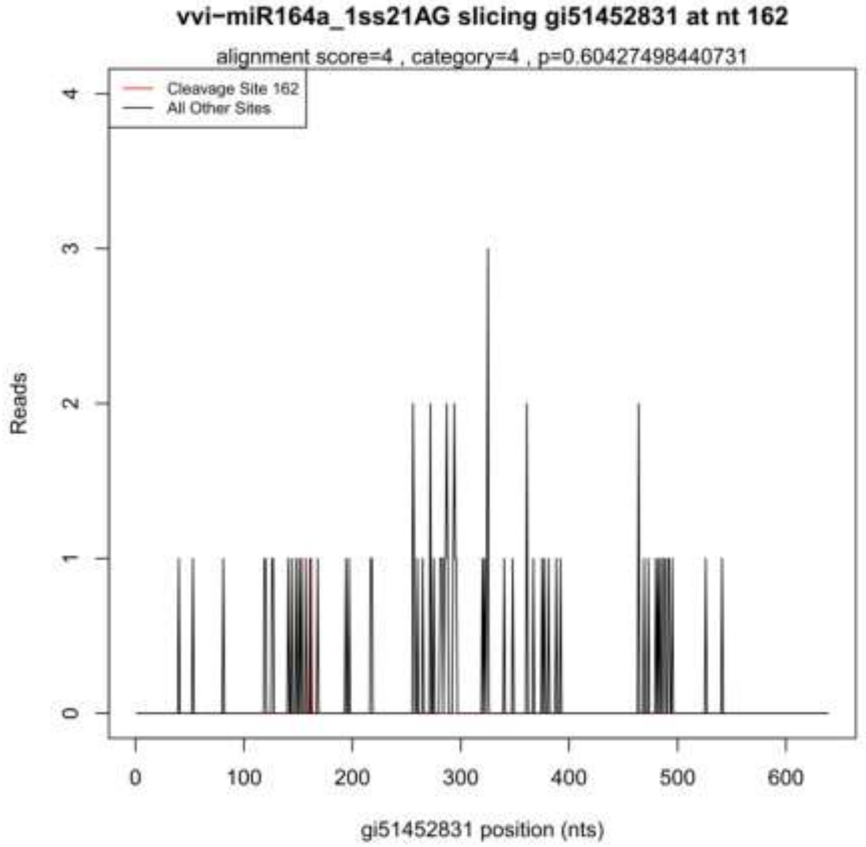
vvi−miR164a_1ss21AG slicing gi51452831 at nt 162
alignment score=4 , category=4 , p=0.60427498440731
Cleavage Site 162
All Other Sites
Reads
0
1
2
3
4
0
100
200
300
400
500
600
gi51452831 position (nts)

A

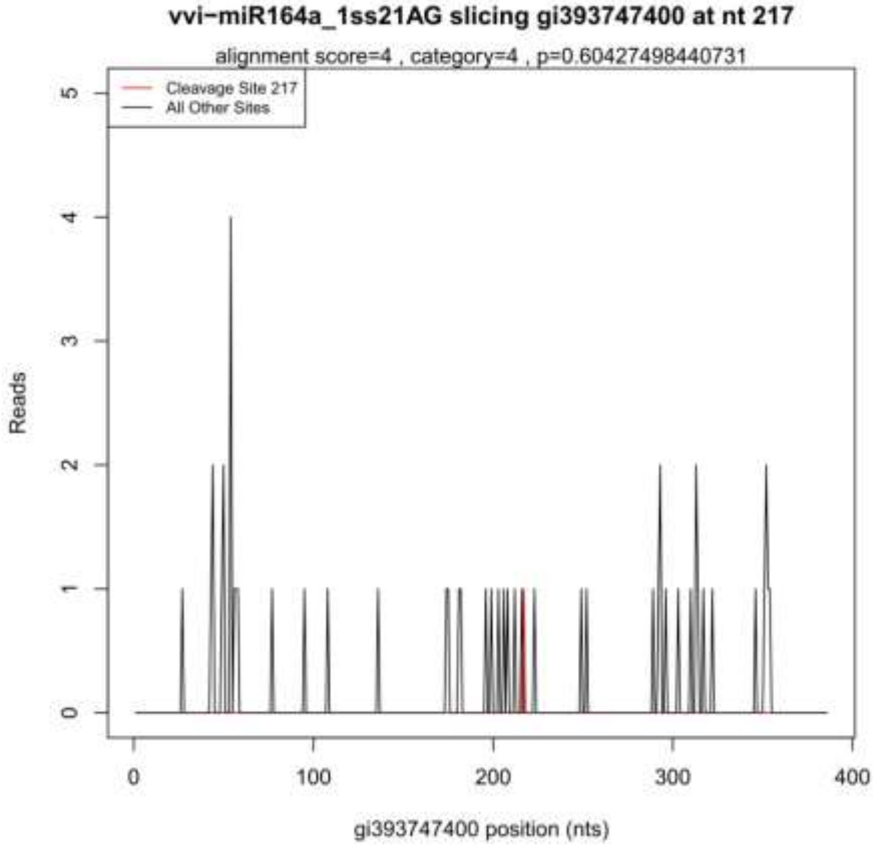
vvi−miR164a_1ss21AG slicing gi393747400 at nt 217
alignment score=4 , category=4 , p=0.60427498440731
Cleavage Site 217
All Other Sites
Reads
0
1
2
3
4
5
0
100
200
300
400
gi393747400 position (nts)

lja-MIR167-p3 slicing gi212379810 at nt 587

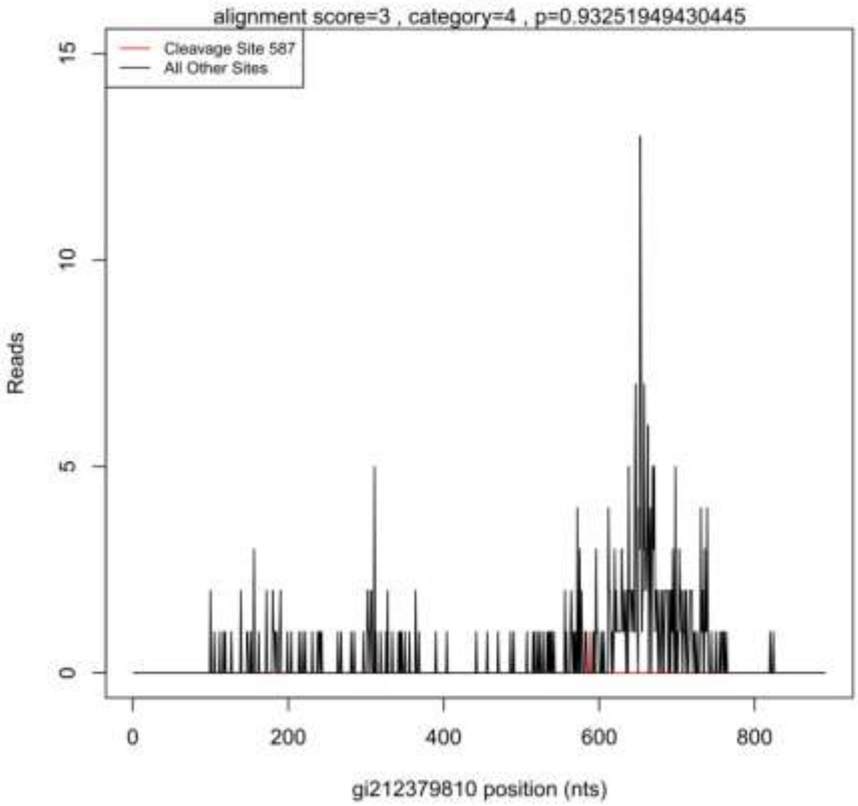

lja-MIR167-p3 slicing gi393741034 at nt 571

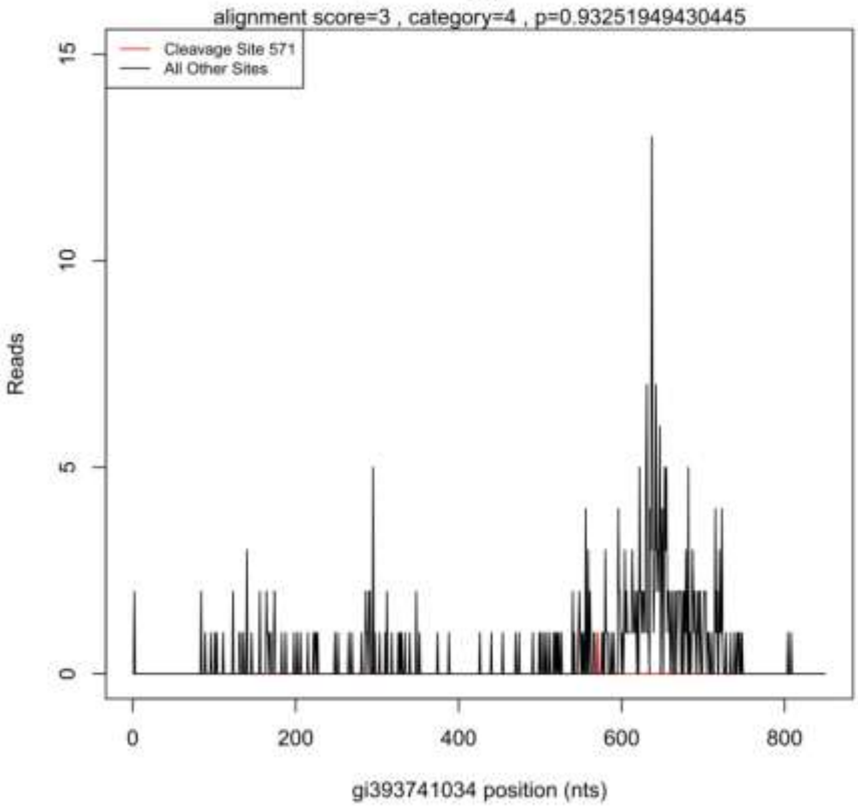

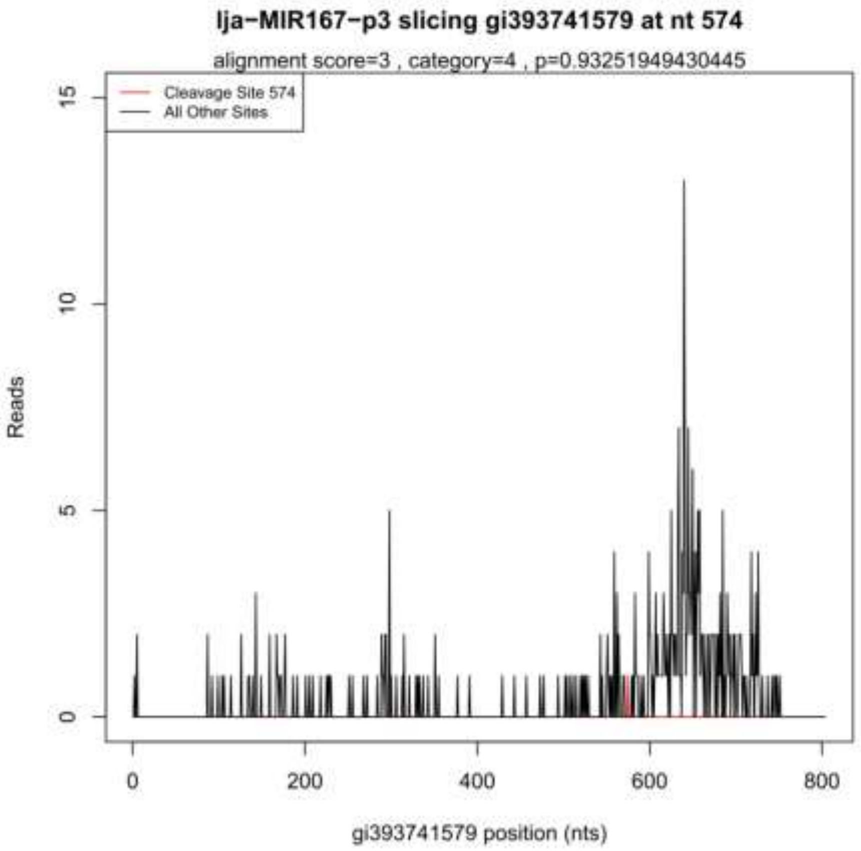
lja−MIR167−p3 slicing gi393741579 at nt 574
alignment score=3 , category=4 , p=0.93251949430445
Cleavage Site 574
All Other Sites
Reads
0
5
10
15
0
200
400
600
800
gi393741579 position (nts)

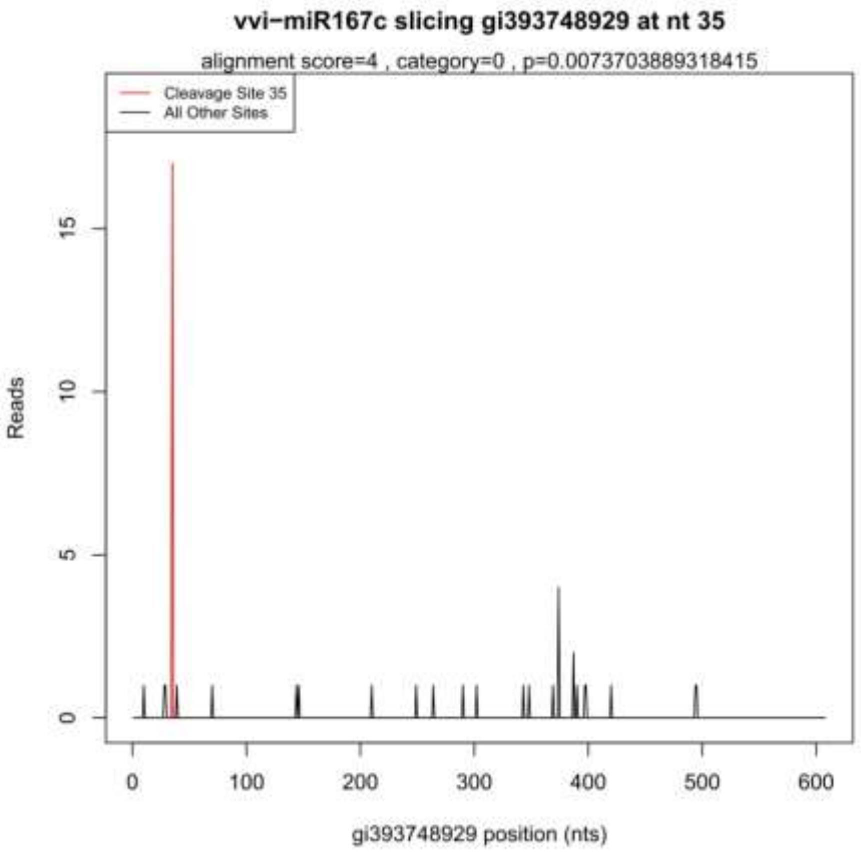
vvi−miR167c slicing gi393748929 at nt 35
alignment score=4 , category=0 , p=0.0073703889318415
Cleavage Site 35
All Other Sites
Reads
0
5
10
15
0
100
200
300
400
500
600
gi393748929 position (nts)

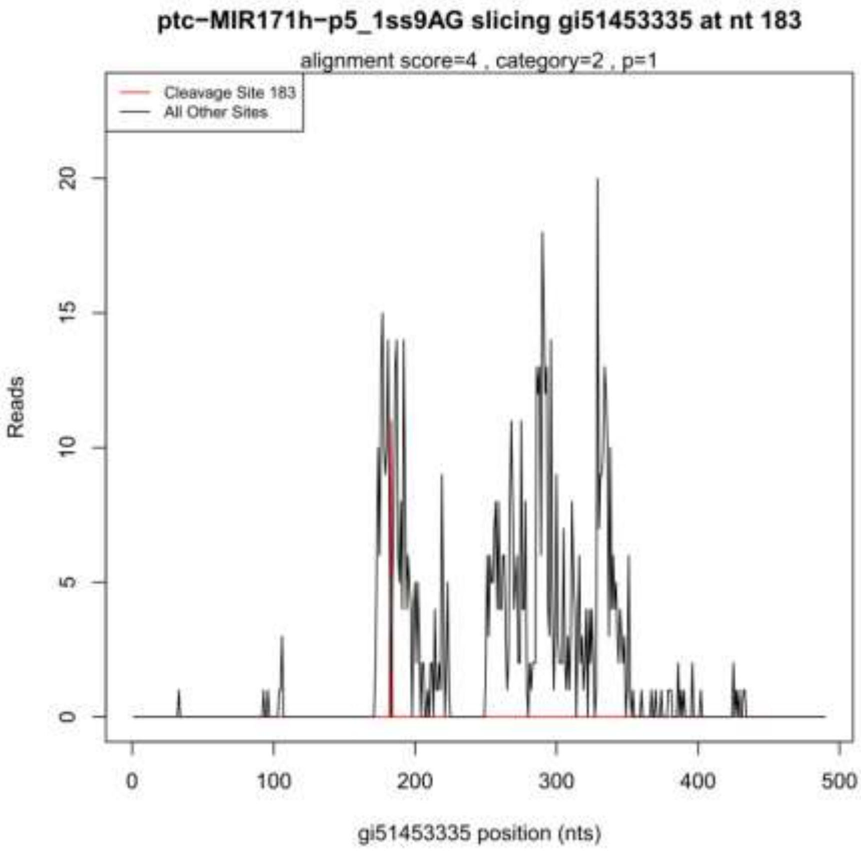

ptc−MIR171h−p5_1ss9AG slicing gi51453335 at nt 183
alignment score=4 , category=2 , p=1
Cleavage Site 183
All Other Sites
Reads
0
5
10
15
20
0
100
200
300
400
500
gi51453335 position (nts)

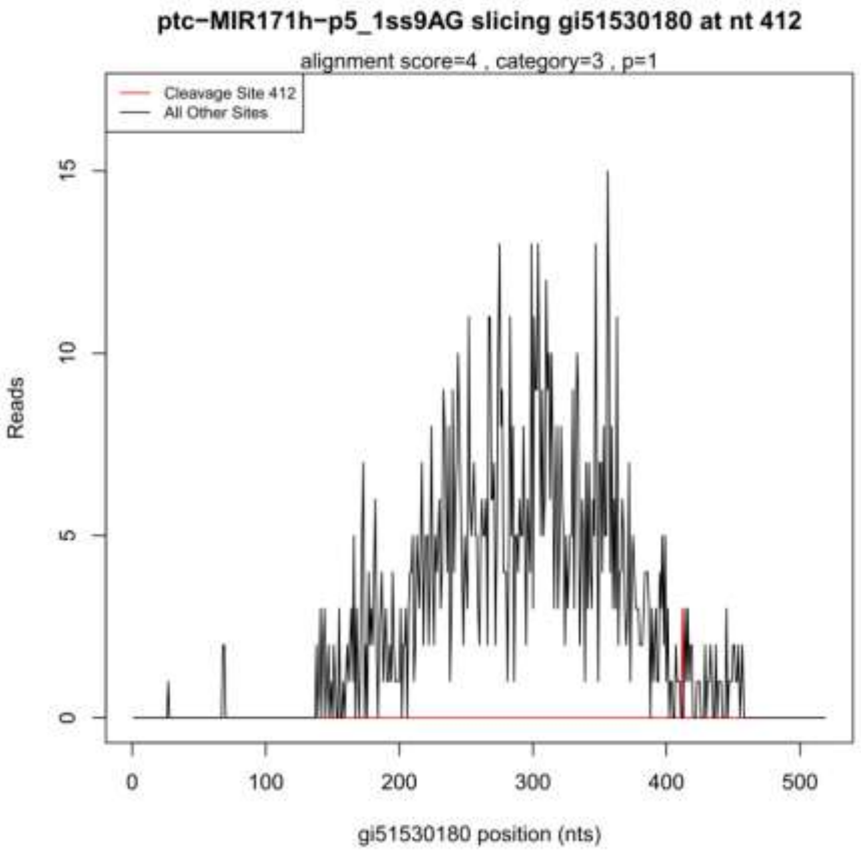

ptc−MIR171h−p5_1ss9AG slicing gi51530180 at nt 412
alignment score=4 , category=3 , p=1
Cleavage Site 412
All Other Sites
Reads
0
5
10
15
0
100
200
300
400
500
gi51530180 position (nts)

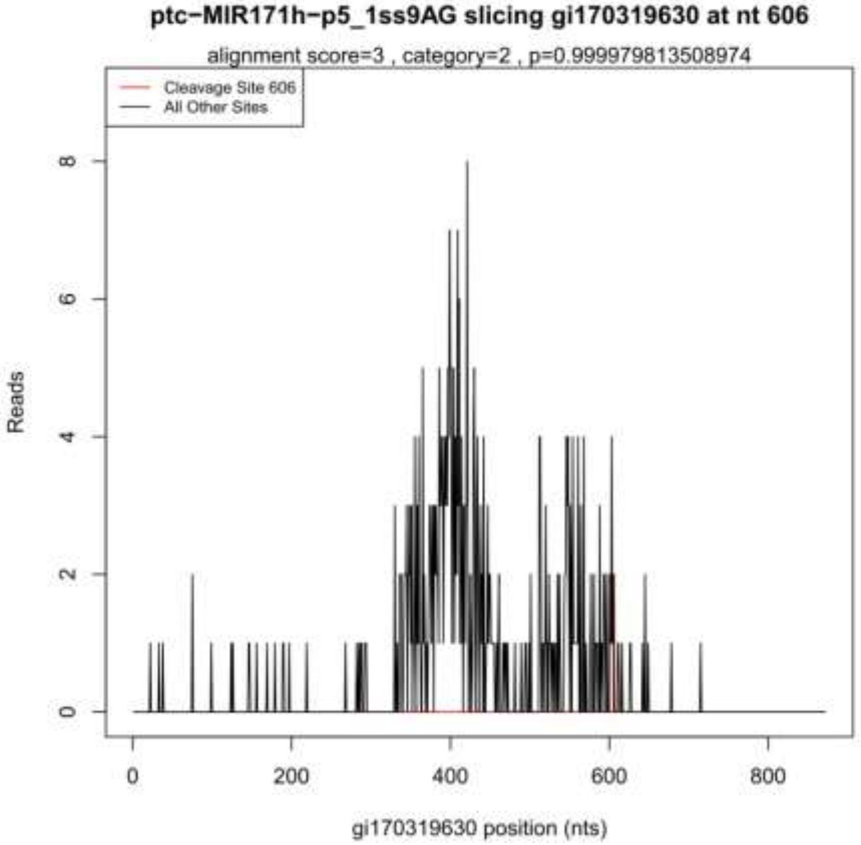
ptc−MIR171h−p5_1ss9AG slicing gi170319630 at nt 606
alignment score=3 , category=2 , p=0.999979813508974
Cleavage Site 606
All Other Sites
Reads
0
2
4
6
8
0
200
400
600
800
gi170319630 position (nts)

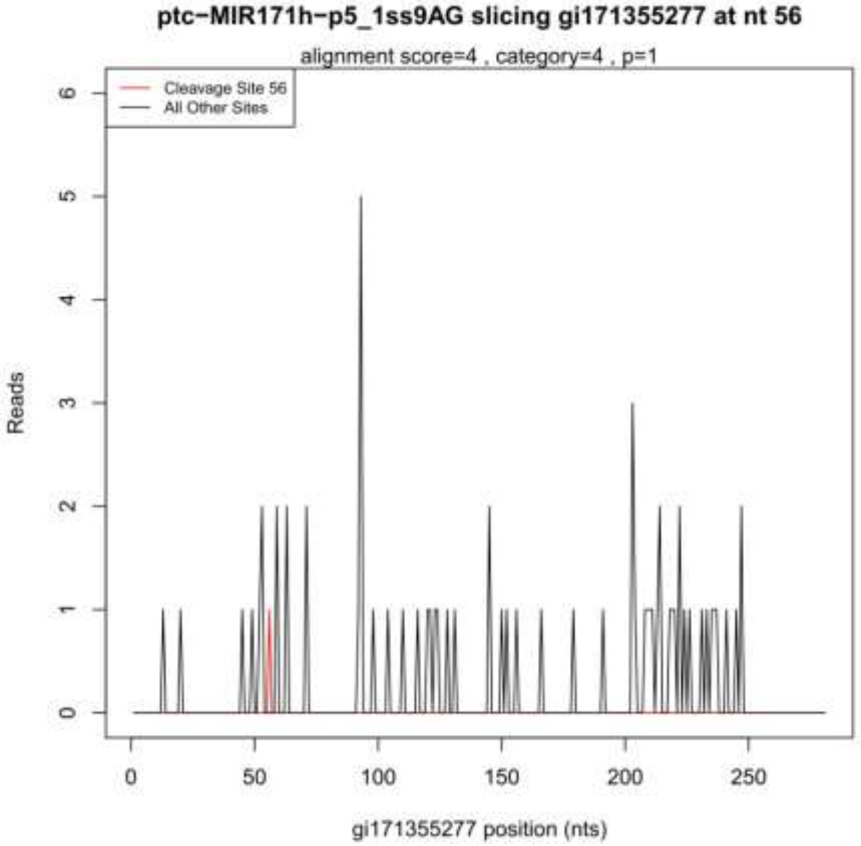
ptc−MIR171h−p5_1ss9AG slicing gi171355277 at nt 56
alignment score=4 , category=4 , p=1
Cleavage Site 56
All Other Sites
Reads
0
1
2
3
4
5
6
0
50
100
150
200
250
gi171355277 position (nts)

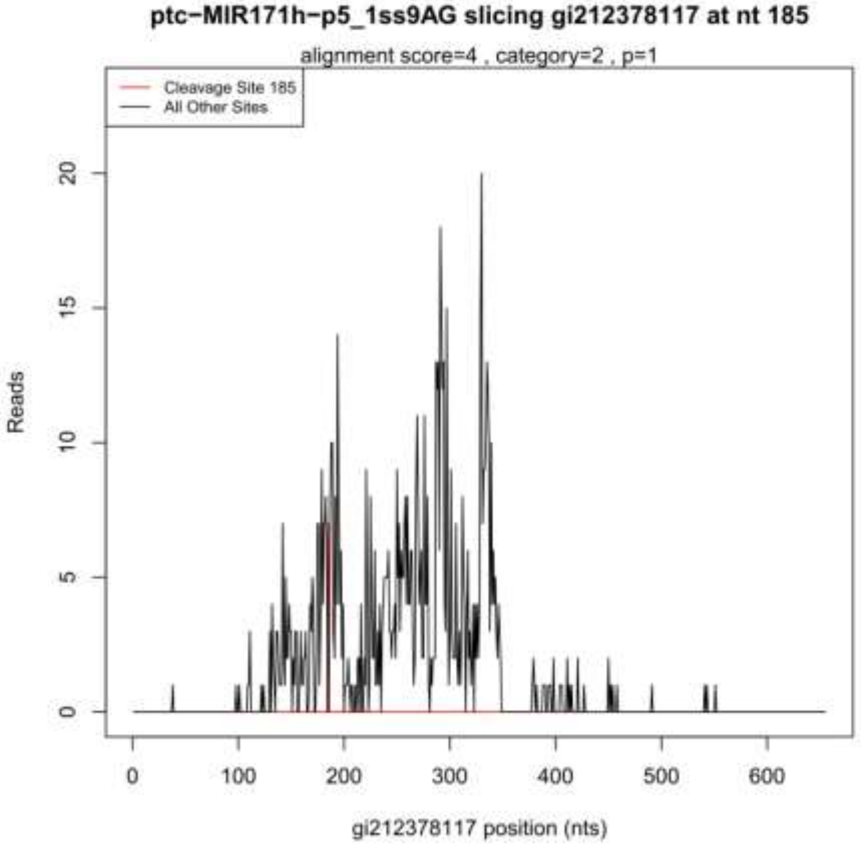
ptc−MIR171h−p5_1ss9AG slicing gi212378117 at nt 185
alignment score=4 , category=2 , p=1
Cleavage Site 185
All Other Sites
Reads
gi212378117 position (nts)

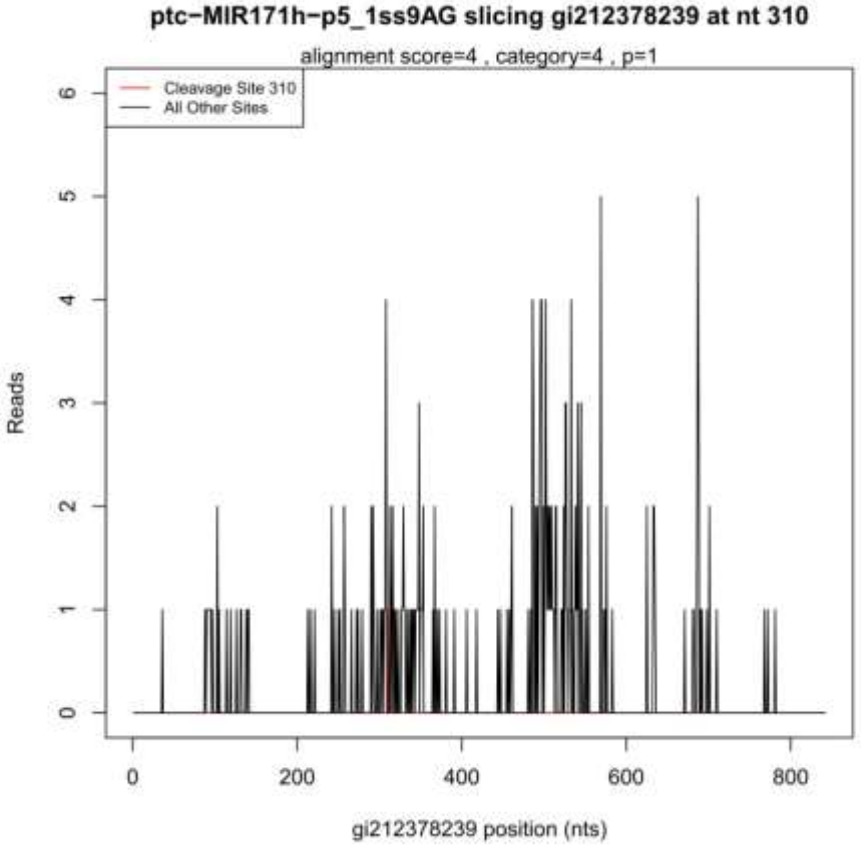
ptc−MIR171h−p5_1ss9AG slicing gi212378239 at nt 310
alignment score=4 , category=4 , p=1
Cleavage Site 310
All Other Sites
Reads
gi212378239 position (nts)

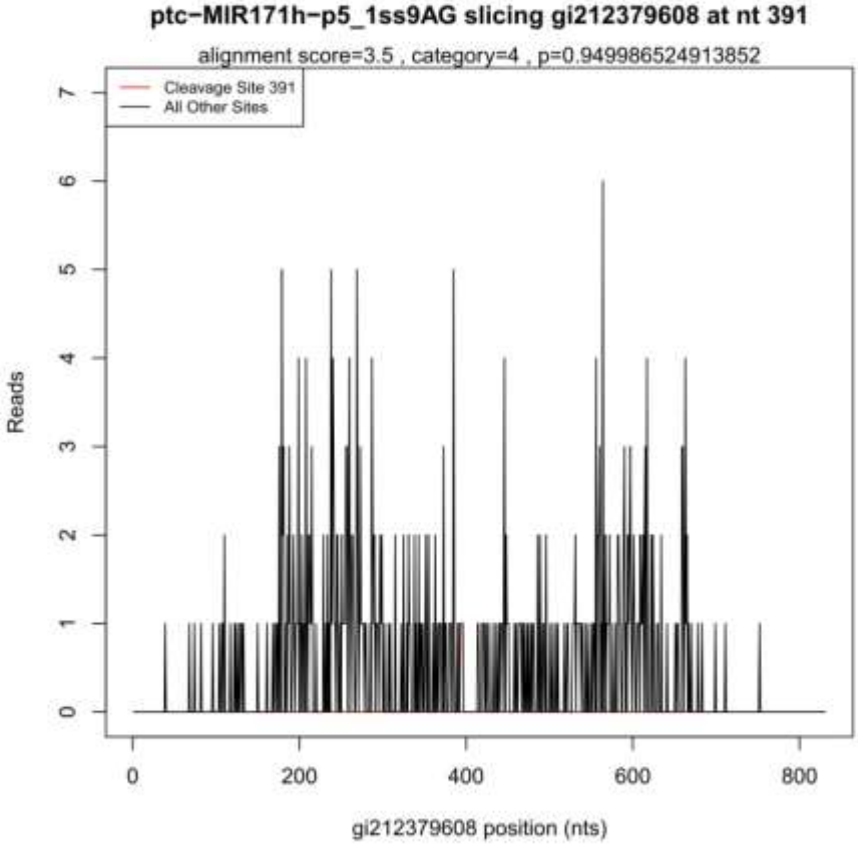
ptc−MIR171h−p5_1ss9AG slicing gi212379608 at nt 391
alignment score=3.5 , category=4 , p=0.949986524913852
Cleavage Site 391
All Other Sites
Reads
gi212379608 position (nts)

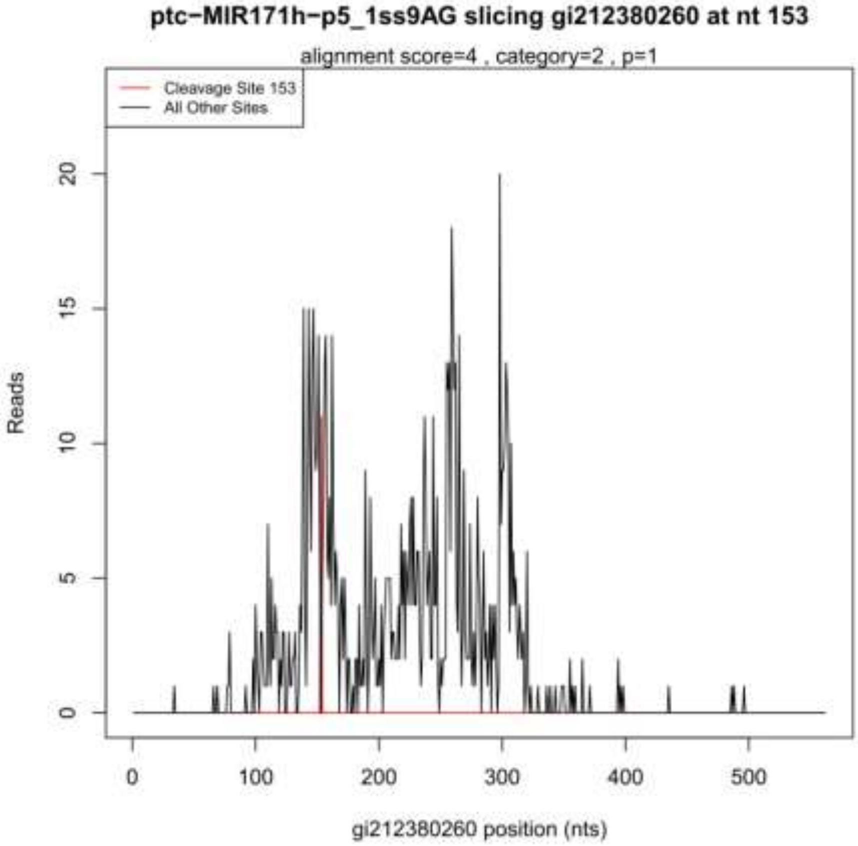
ptc−MIR171h−p5_1ss9AG slicing gi212380260 at nt 153
alignment score=4 , category=2 , p=1
Cleavage Site 153
All Other Sites
Reads
gi212380260 position (nts)

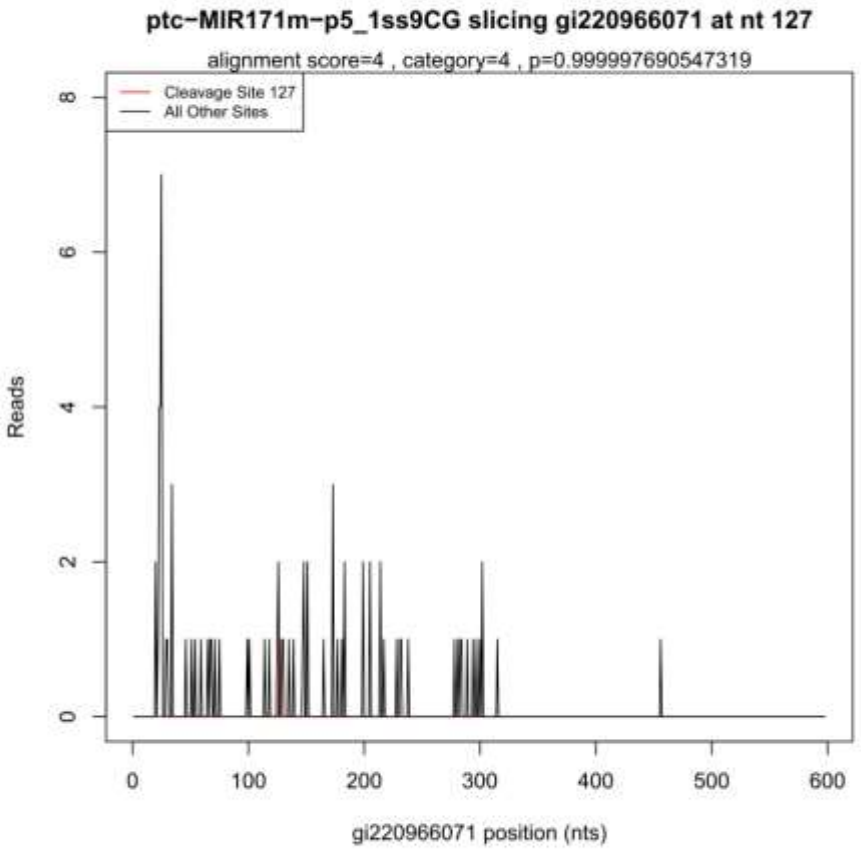
ptc-MIR171m-p5_1ss9CG slicing gi220966071 at nt 127
alignment score=4 , category=4 , p=0.999997690547319
Cleavage Site 127
All Other Sites
Reads
0
2
4
6
8
0
100
200
300
400
500
600
gi220966071 position (nts)

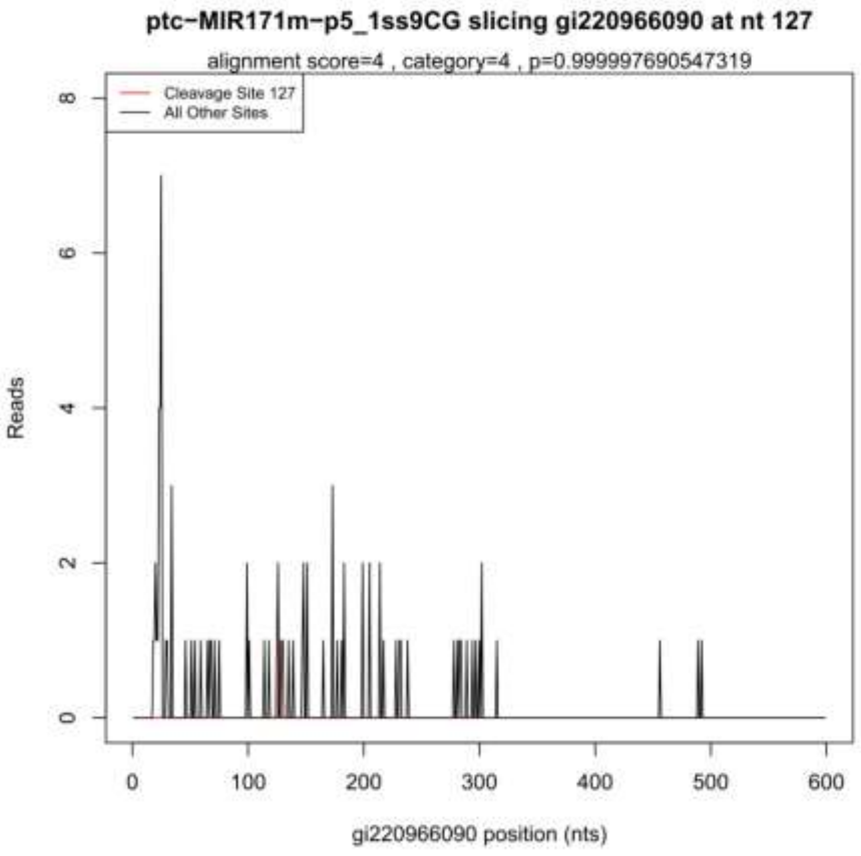
ptc-MIR171m-p5_1ss9CG slicing gi220966090 at nt 127
alignment score=4 , category=4 , p=0.999997690547319
Cleavage Site 127
All Other Sites
Reads
0
2
4
6
8
0
100
200
300
400
500
600
gi220966090 position (nts)

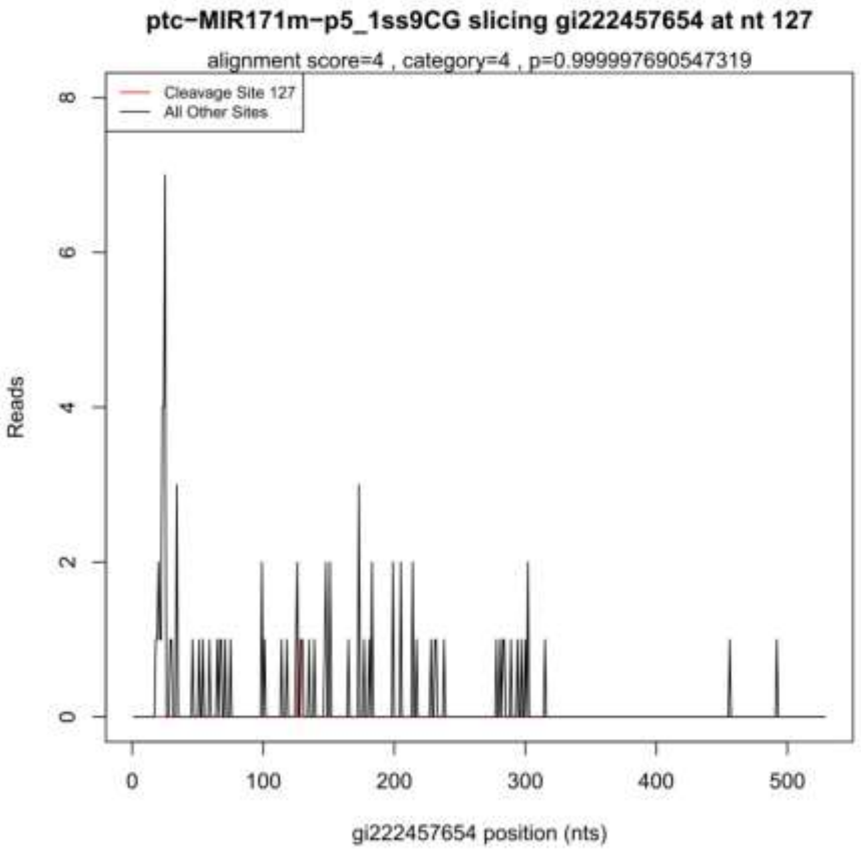
ptc-MIR171m-p5_1ss9CG slicing gi222457654 at nt 127
alignment score=4 , category=4 , p=0.999997690547319
Cleavage Site 127
All Other Sites
Reads
0
2
4
6
8
0
100
200
300
400
500
gi222457654 position (nts)

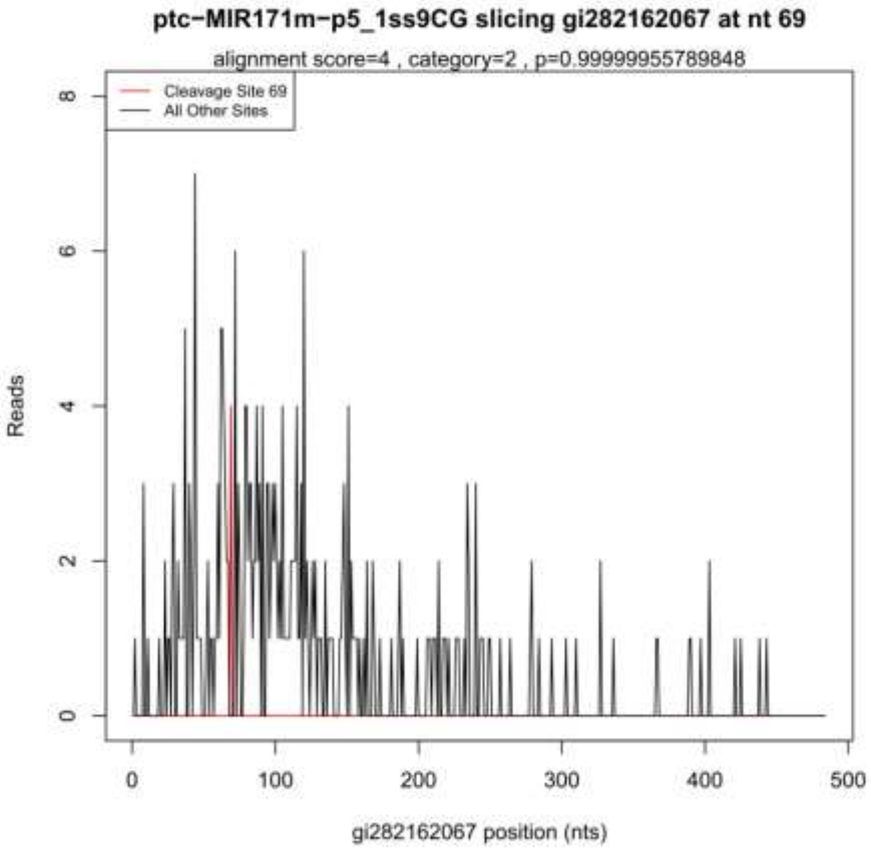
ptc-MIR171m-p5_1ss9CG slicing gi282162067 at nt 69
alignment score=4 , category=2 , p=0.99999955789848
Cleavage Site 69
All Other Sites
Reads
0
2
4
6
8
0
100
200
300
400
500
gi282162067 position (nts)

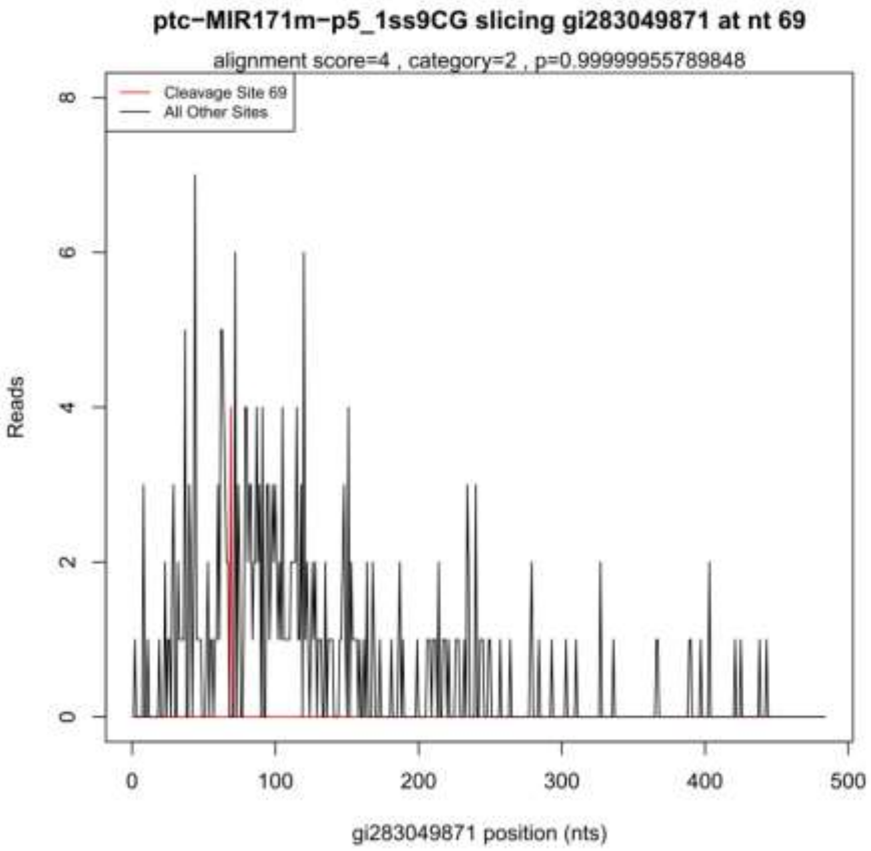
ptc−MIR171m−p5_1ss9CG slicing gi283049871 at nt 69
alignment score=4 , category=2 , p=0.99999955789848
Cleavage Site 69
All Other Sites
Reads
0
2
4
6
8
0
100
200
300
400
500
gi283049871 position (nts)

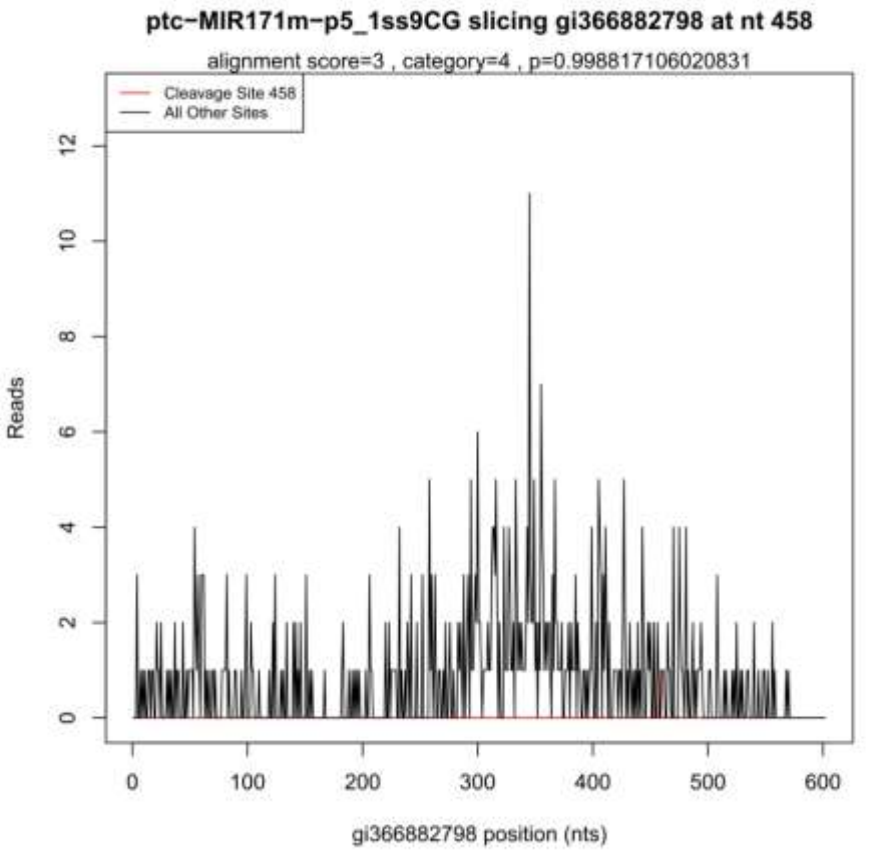
ptc−MIR171m−p5_1ss9CG slicing gi366882798 at nt 458
alignment score=3 , category=4 , p=0.998817106020831
Cleavage Site 458
All Other Sites
Reads
0
2
4
6
8
10
12
0
100
200
300
400
500
600
gi366882798 position (nts)

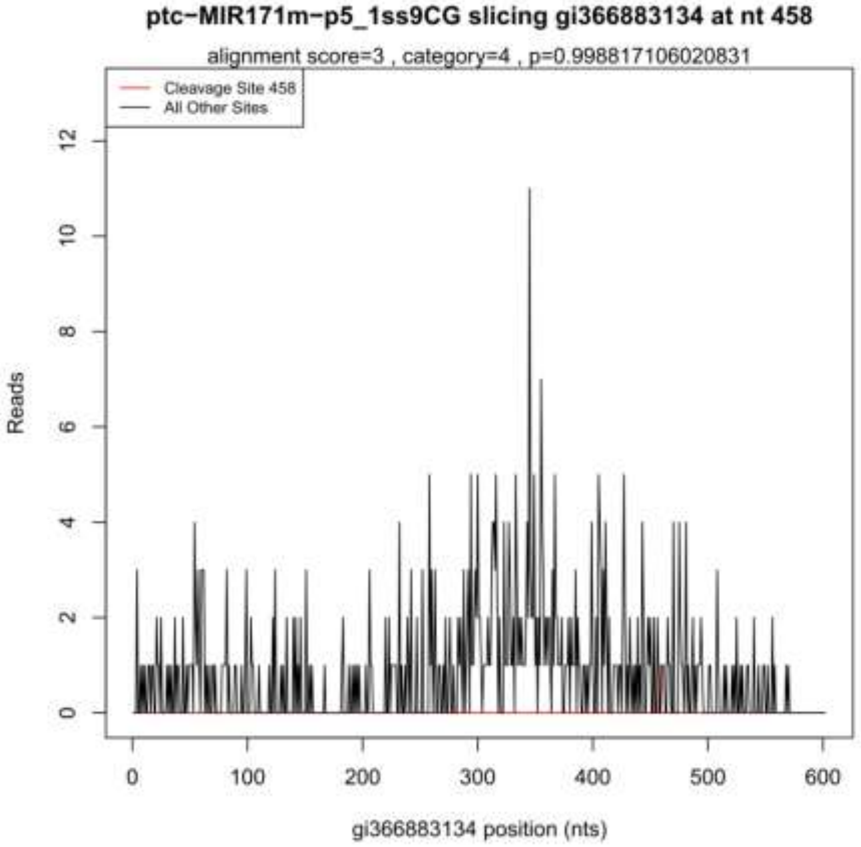
ptc−MIR171m−p5_1ss9CG slicing gi366883134 at nt 458
alignment score=3 , category=4 , p=0.998817106020831
Cleavage Site 458
All Other Sites
Reads
gi366883134 position (nts)

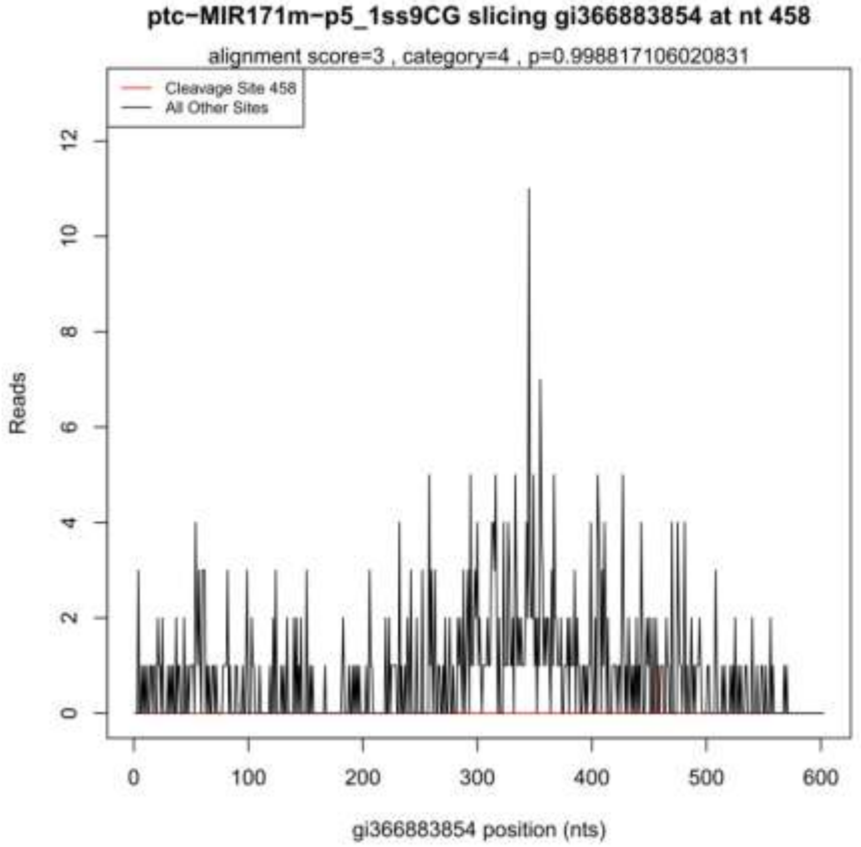
ptc−MIR171m−p5_1ss9CG slicing gi366883854 at nt 458
alignment score=3 , category=4 , p=0.998817106020831
Cleavage Site 458
All Other Sites
Reads
gi366883854 position (nts)

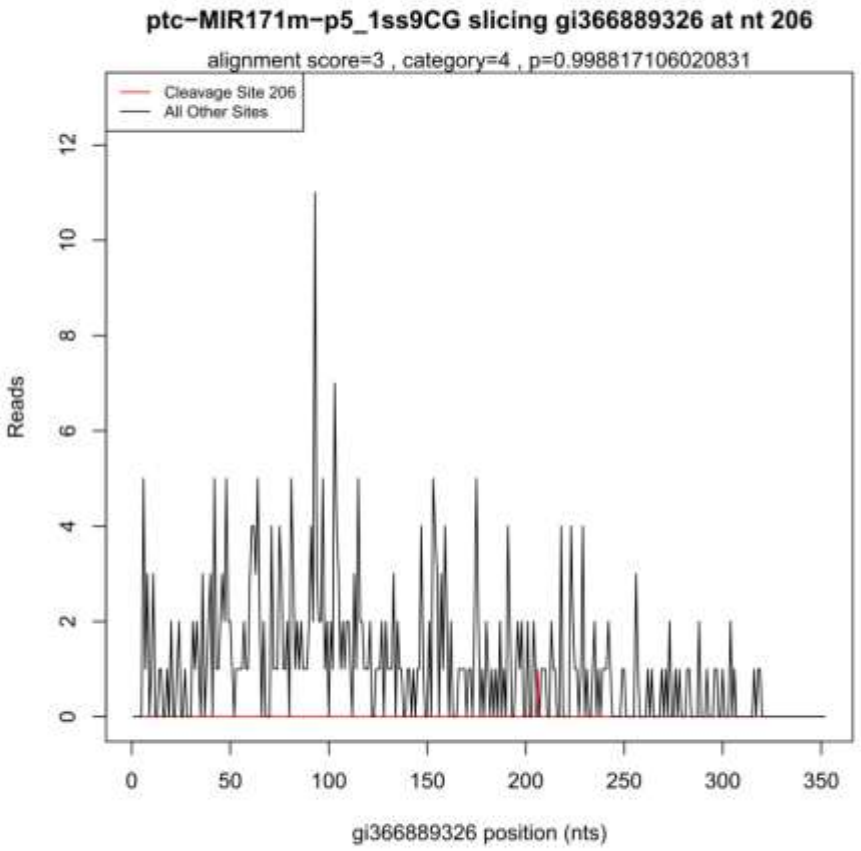
ptc−MIR171m−p5_1ss9CG slicing gi366889326 at nt 206
alignment score=3 , category=4 , p=0.998817106020831
Cleavage Site 206
All Other Sites
Reads
0
2
4
6
8
10
12
0
50
100
150
200
250
300
350
gi366889326 position (nts)

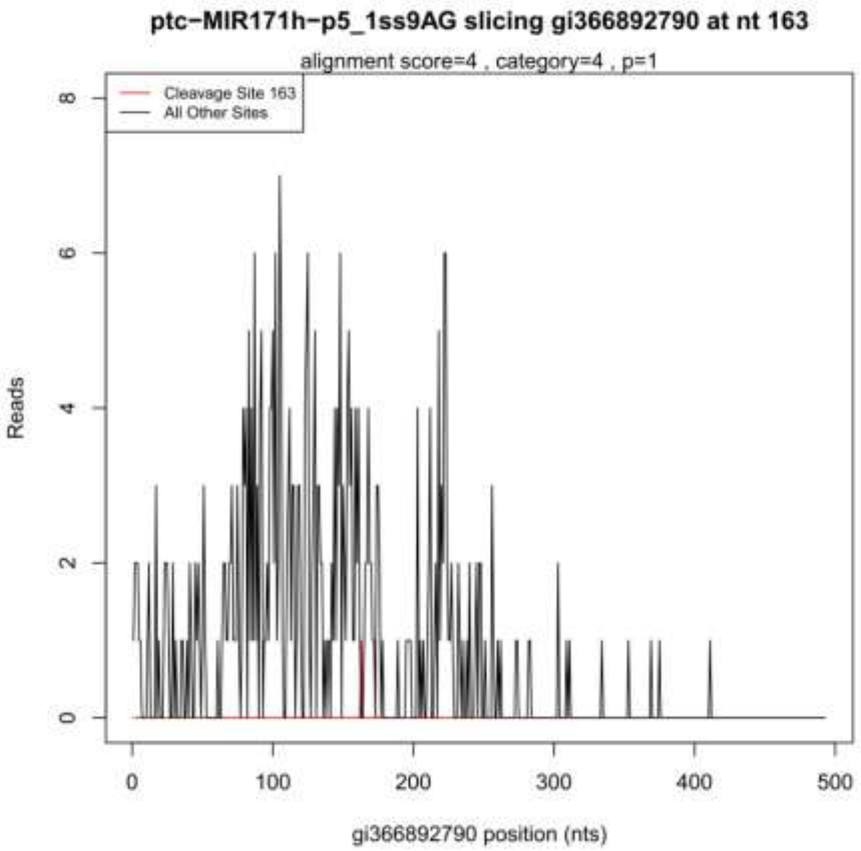
ptc−MIR171h−p5_1ss9AG slicing gi366892790 at nt 163
alignment score=4 , category=4 , p=1
Cleavage Site 163
All Other Sites
Reads
0
2
4
6
8
0
100
200
300
400
500
gi366892790 position (nts)

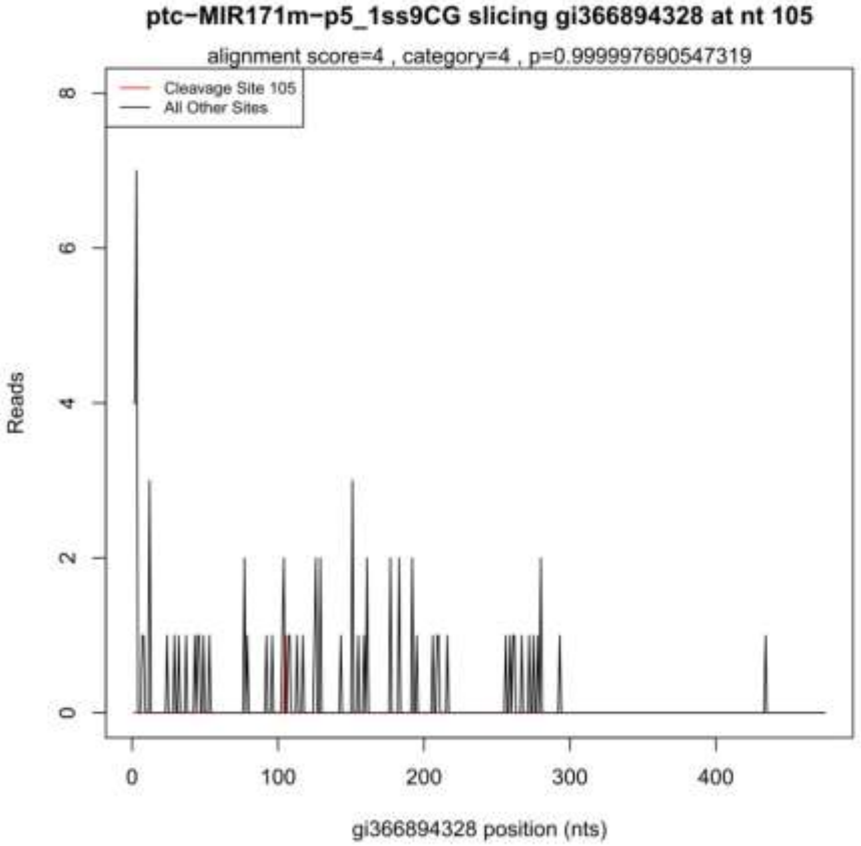
ptc-MIR171m-p5_1ss9CG slicing gi366894328 at nt 105
alignment score=4 , category=4 , p=0.999997690547319
Cleavage Site 105
All Other Sites
Reads
0
2
4
6
8
0
100
200
300
400
gi366894328 position (nts)

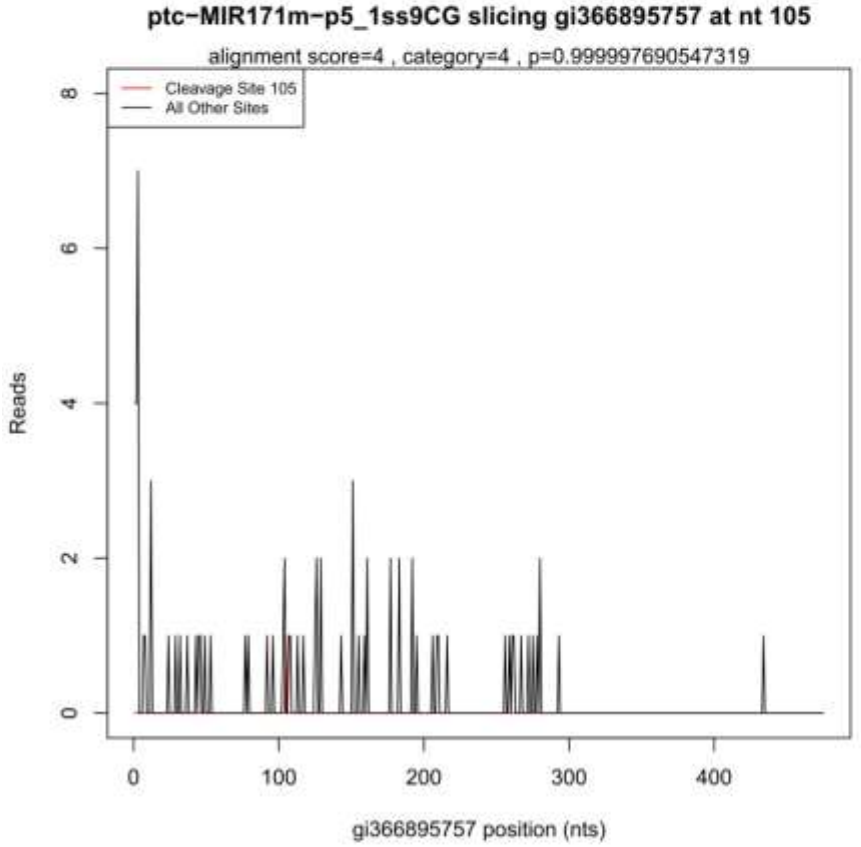
ptc-MIR171m-p5_1ss9CG slicing gi366895757 at nt 105
alignment score=4 , category=4 , p=0.999997690547319
Cleavage Site 105
All Other Sites
Reads
0
2
4
6
8
0
100
200
300
400
gi366895757 position (nts)

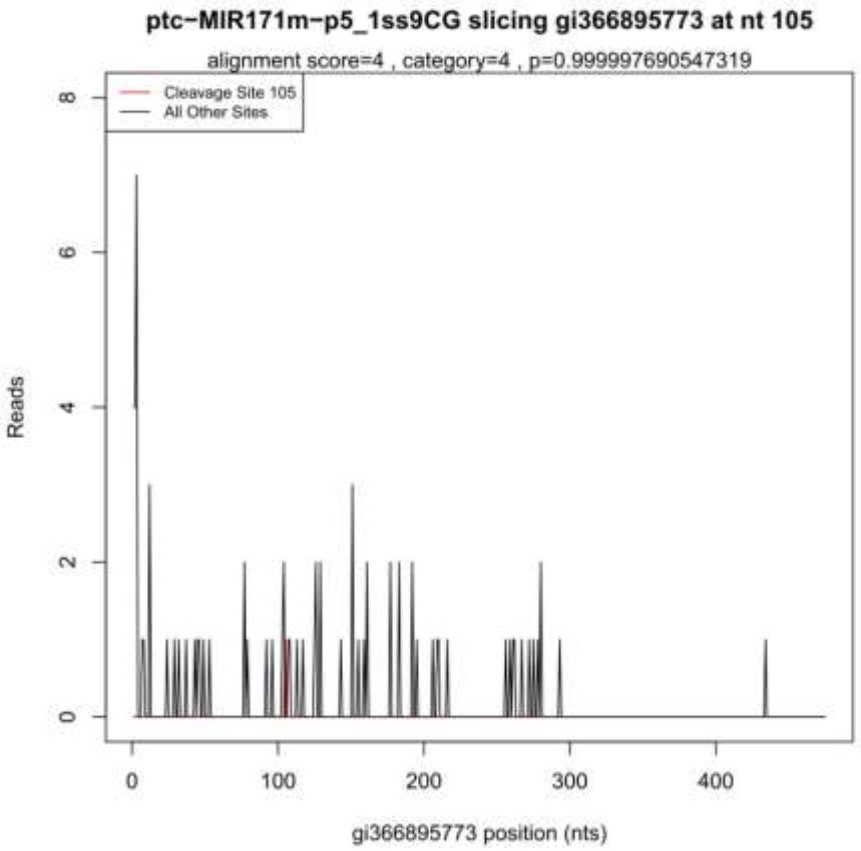
ptc−MIR171m−p5_1ss9CG slicing gi366895773 at nt 105
alignment score=4 , category=4 , p=0.999997690547319
Cleavage Site 105
All Other Sites
Reads
gi366895773 position (nts)

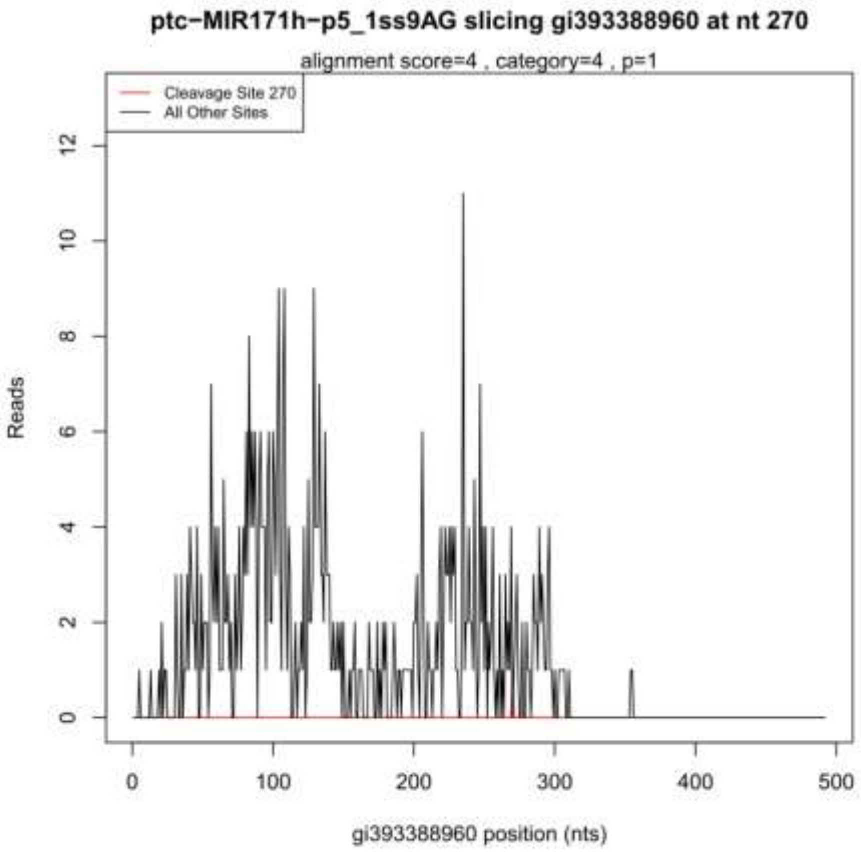
ptc−MIR171h−p5_1ss9AG slicing gi393388960 at nt 270
alignment score=4 , category=4 , p=1
Cleavage Site 270
All Other Sites
Reads
gi393388960 position (nts)

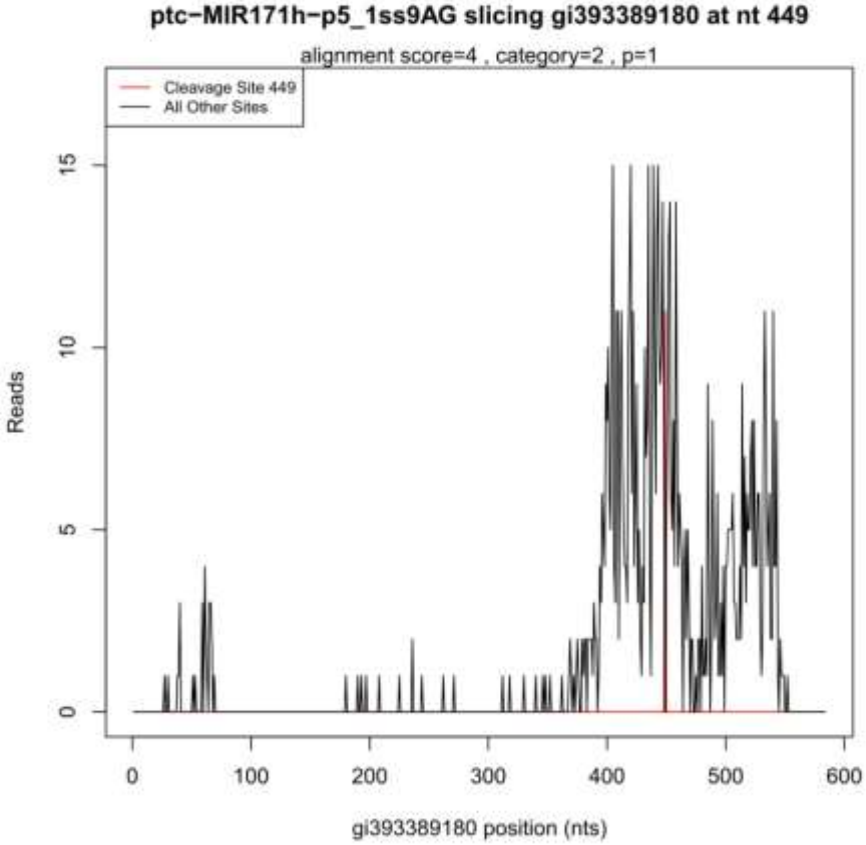
ptc−MIR171h−p5_1ss9AG slicing gi393389180 at nt 449
alignment score=4 , category=2 , p=1
Cleavage Site 449
All Other Sites
Reads
0
5
10
15
0
100
200
300
400
500
600
gi393389180 position (nts)

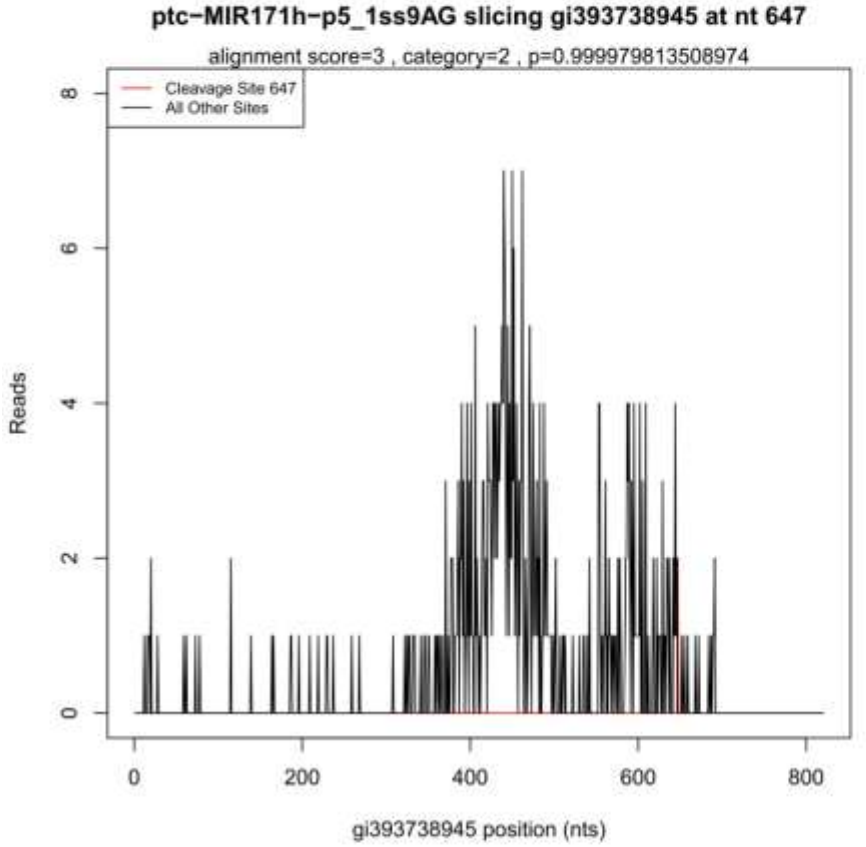
ptc−MIR171h−p5_1ss9AG slicing gi393738945 at nt 647
alignment score=3 , category=2 , p=0.999979813508974
Cleavage Site 647
All Other Sites
Reads
0
2
4
6
8
0
200
400
600
800
gi393738945 position (nts)

ptc−MIR171h−p5_1ss9AG slicing gi393739105 at nt 775

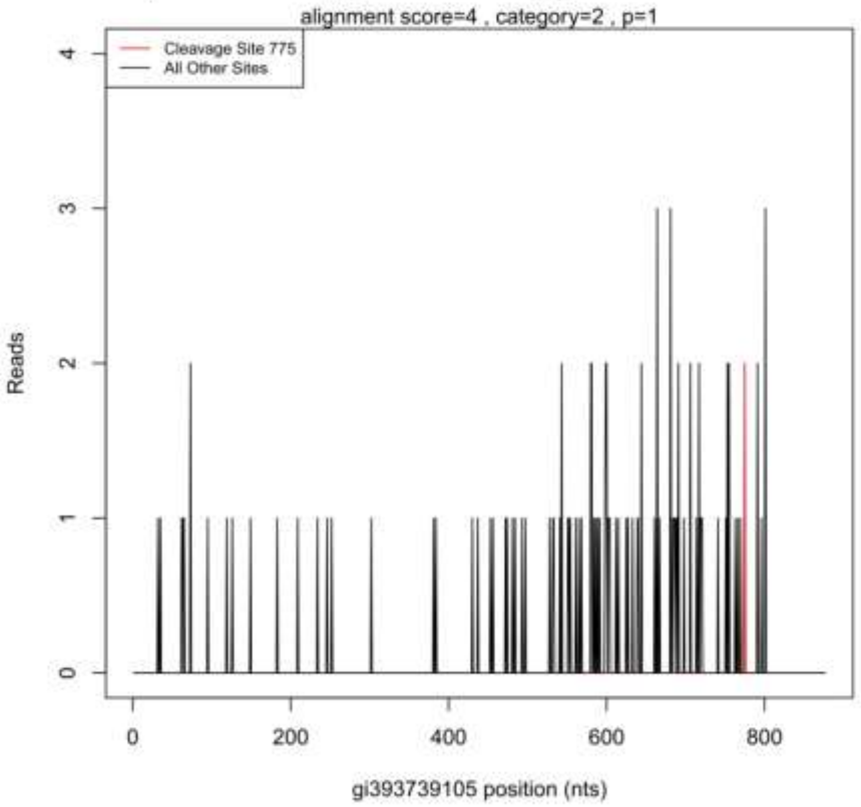

ptc−MIR171m−p5_1ss9CG slicing gi393739664 at nt 311

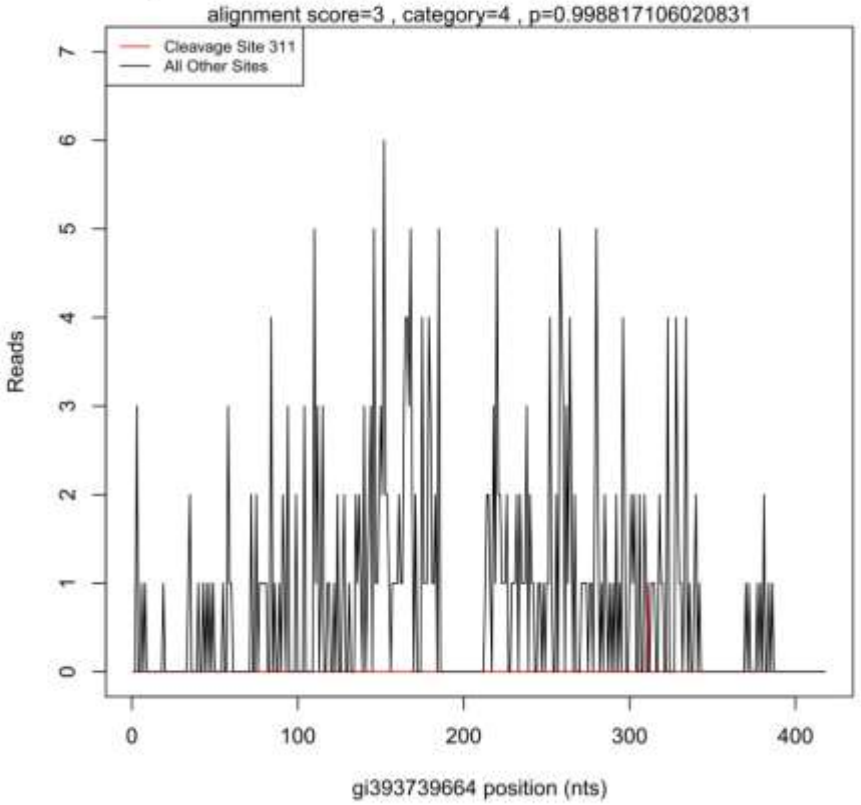

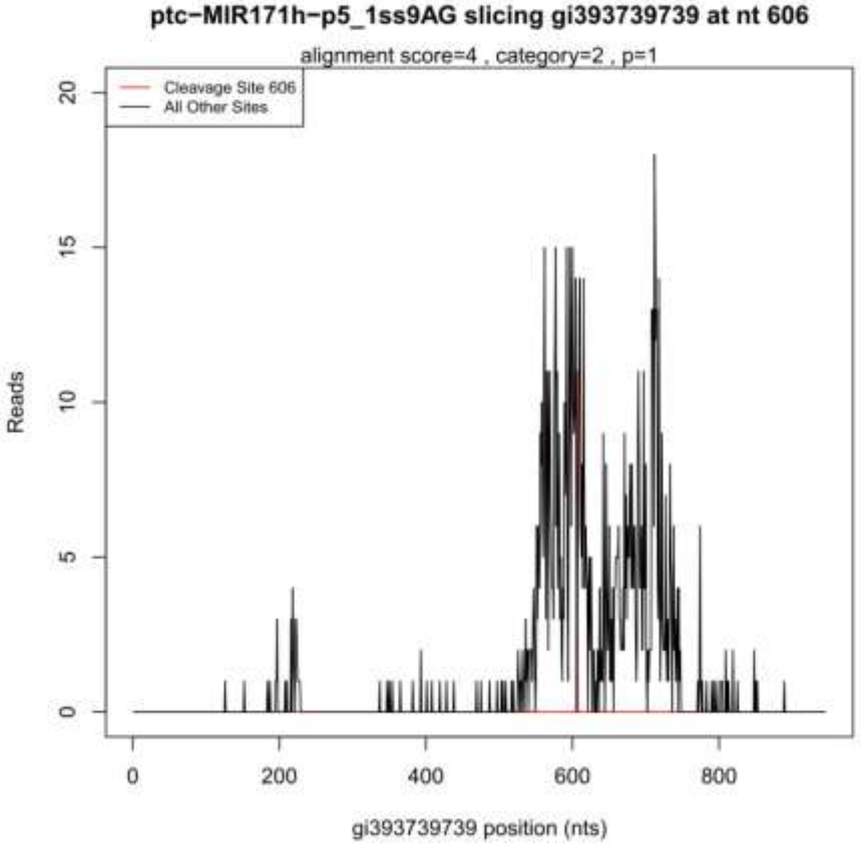

ptc−MIR171h−p5_1ss9AG slicing gi393739739 at nt 606
alignment score=4 , category=2 , p=1
Cleavage Site 606
All Other Sites
Reads
0
5
10
15
20
0
200
400
600
800
gi393739739 position (nts)

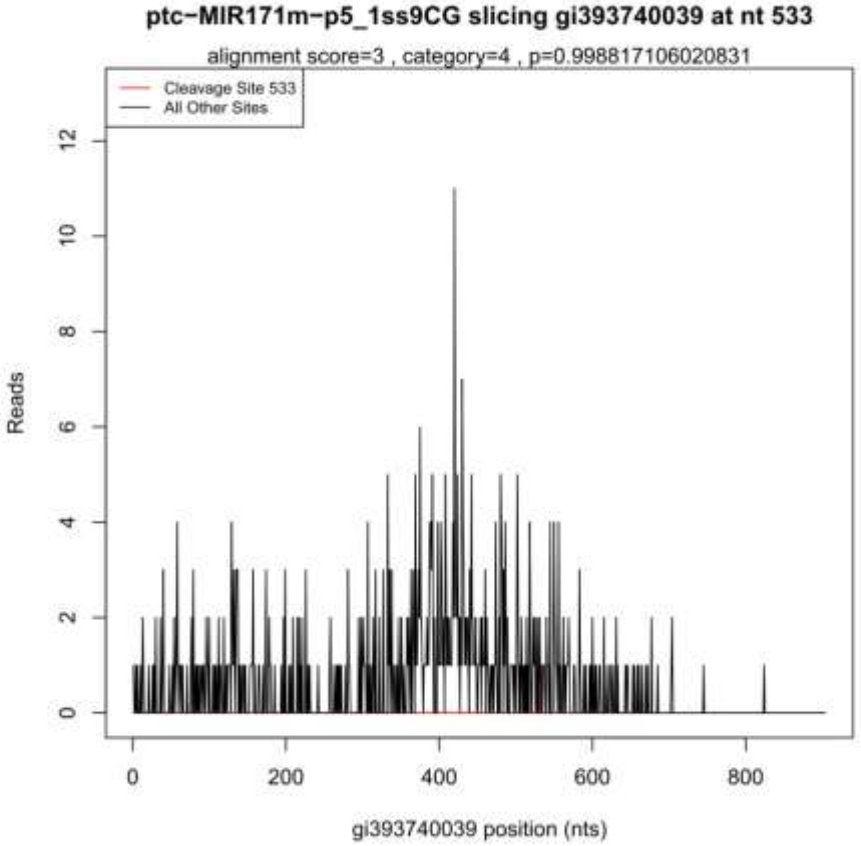

ptc−MIR171m−p5_1ss9CG slicing gi393740039 at nt 533
alignment score=3 , category=4 , p=0.998817106020831
Cleavage Site 533
All Other Sites
Reads
0
2
4
6
8
10
12
0
200
400
600
800
gi393740039 position (nts)

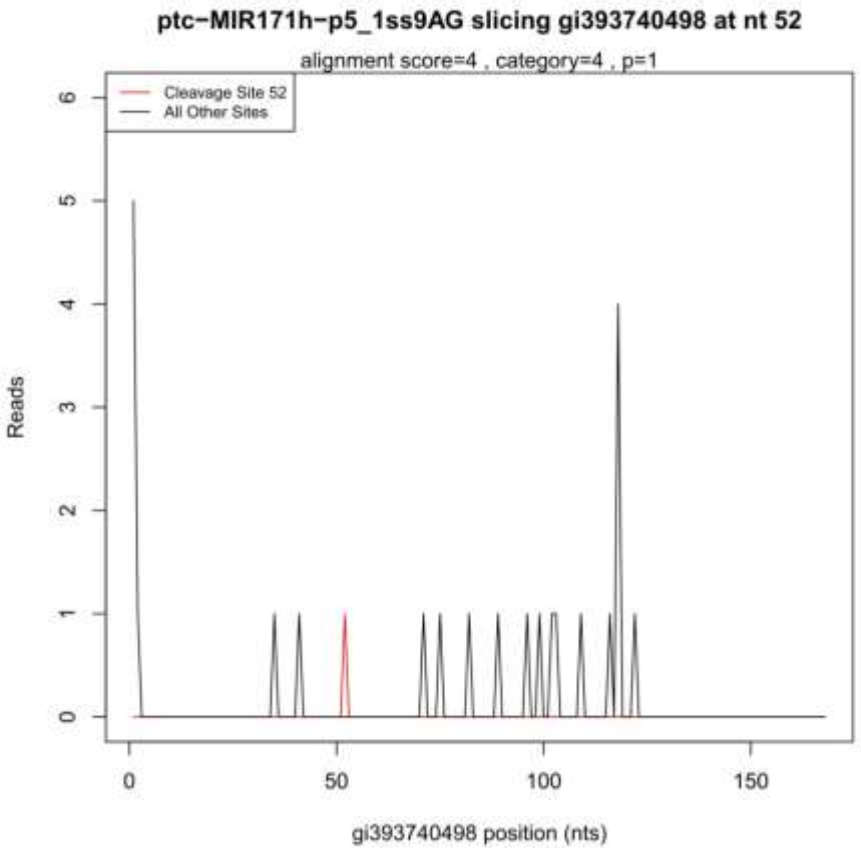
ptc−MIR171h−p5_1ss9AG slicing gi393740498 at nt 52
alignment score=4 , category=4 , p=1
Cleavage Site 52
All Other Sites
Reads
0
1
2
3
4
5
6
0
50
100
150
gi393740498 position (nts)

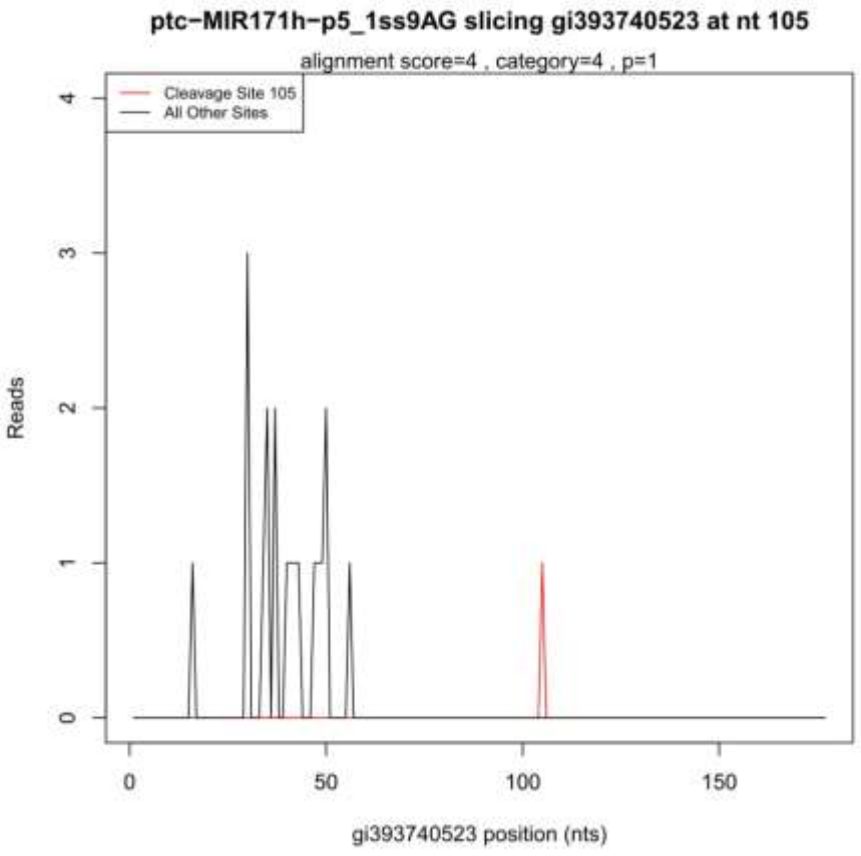
ptc−MIR171h−p5_1ss9AG slicing gi393740523 at nt 105
alignment score=4 , category=4 , p=1
Cleavage Site 105
All Other Sites
Reads
0
1
2
3
4
0
50
100
150
gi393740523 position (nts)

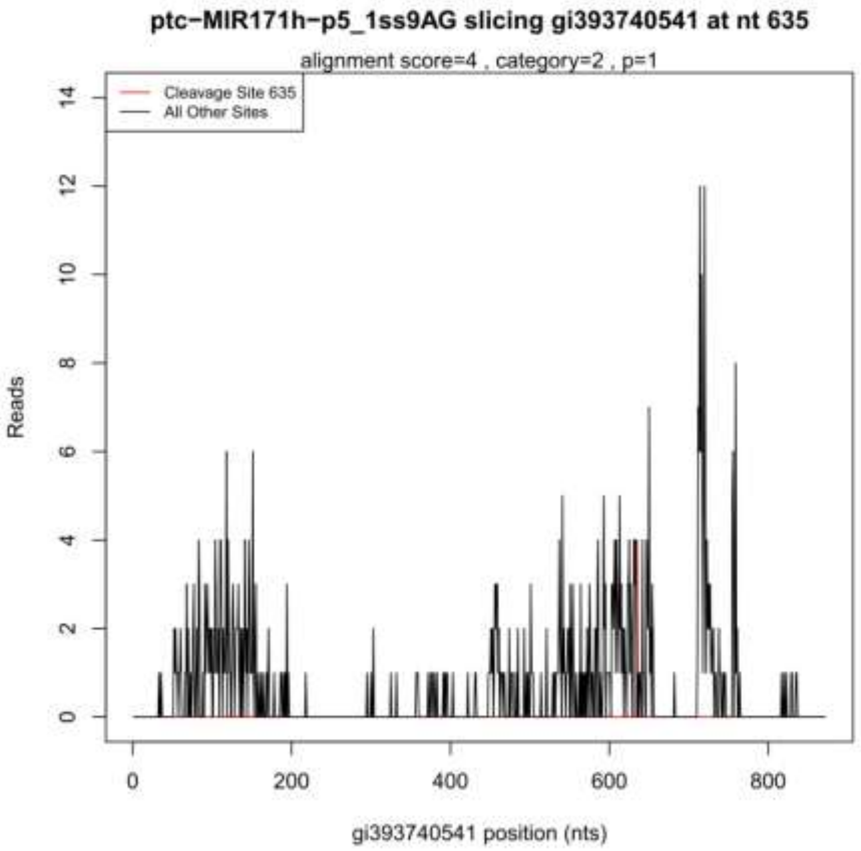
ptc−MIR171h−p5_1ss9AG slicing gi393740541 at nt 635
alignment score=4 , category=2 , p=1
Cleavage Site 635
All Other Sites
Reads
0
2
4
6
8
10
12
14
0
200
400
600
800
gi393740541 position (nts)

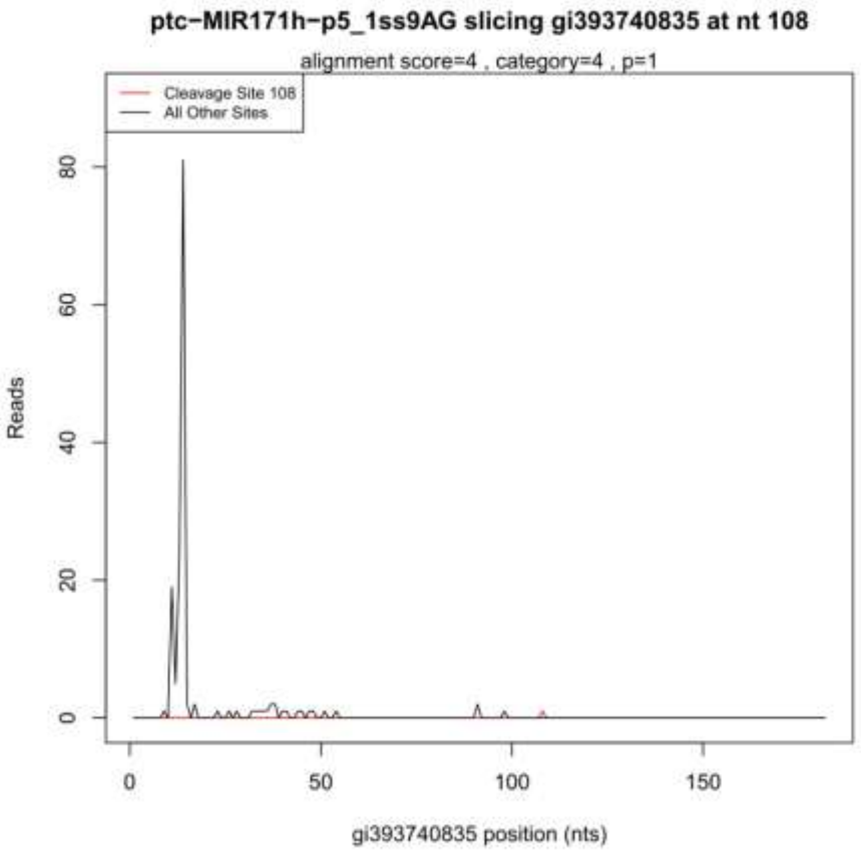
ptc−MIR171h−p5_1ss9AG slicing gi393740835 at nt 108
alignment score=4 , category=4 , p=1
Cleavage Site 108
All Other Sites
Reads
0
20
40
60
80
0
50
100
150
gi393740835 position (nts)

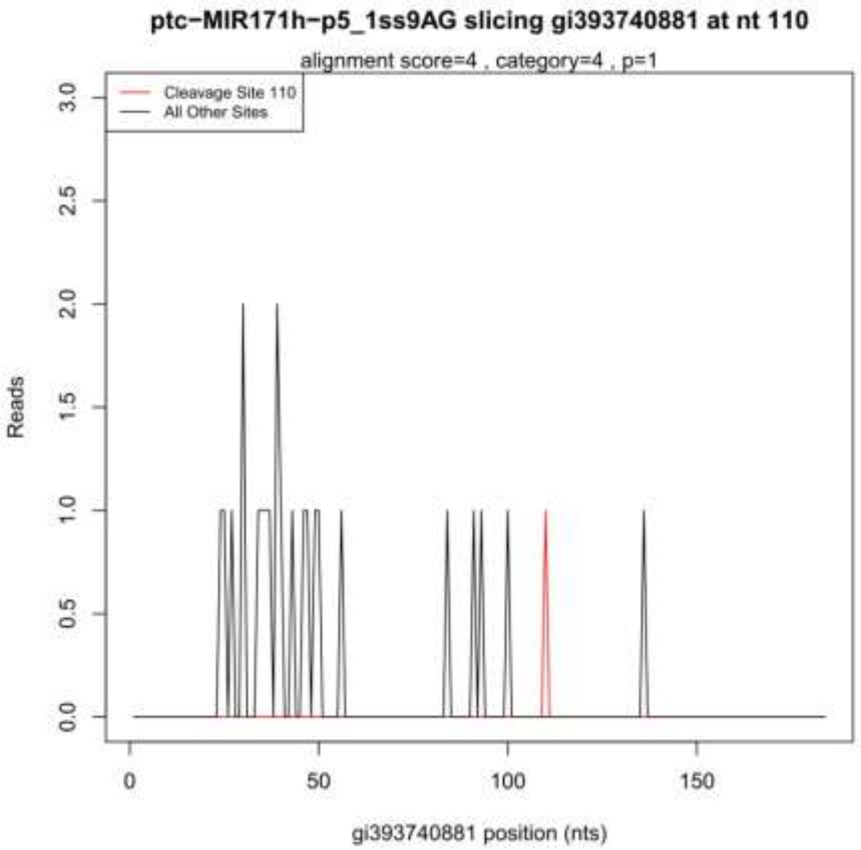
ptc-MIR171h-p5_1ss9AG slicing gi393740881 at nt 110
alignment score=4 , category=4 , p=1
Cleavage Site 110
All Other Sites
Reads
gi393740881 position (nts)

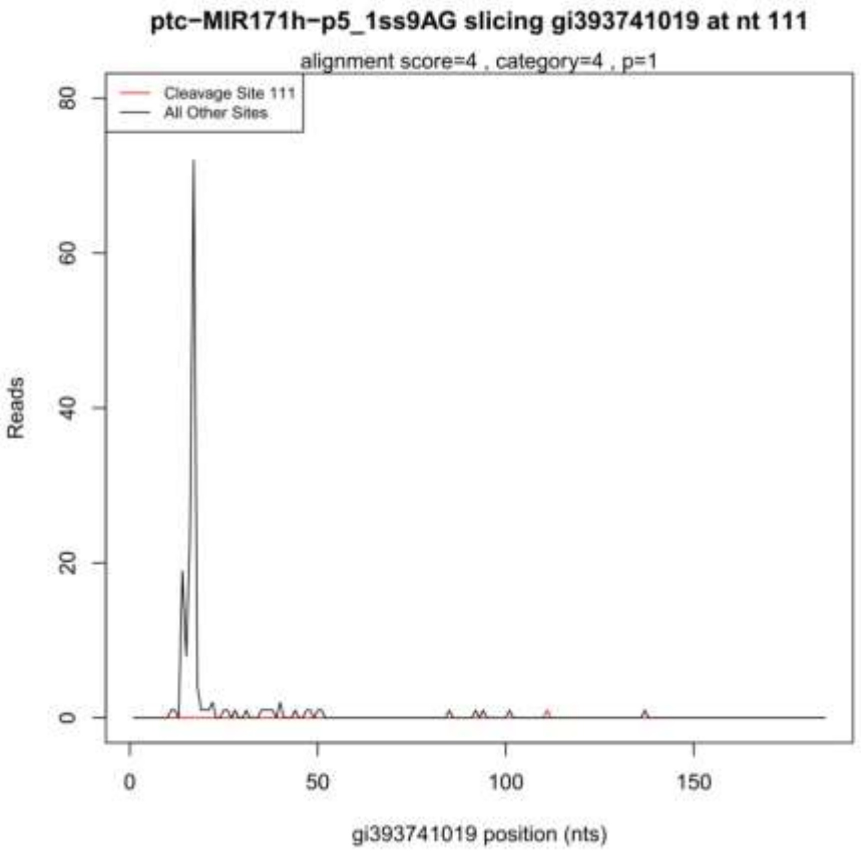
ptc-MIR171h-p5_1ss9AG slicing gi393741019 at nt 111
alignment score=4 , category=4 , p=1
Cleavage Site 111
All Other Sites
Reads
gi393741019 position (nts)

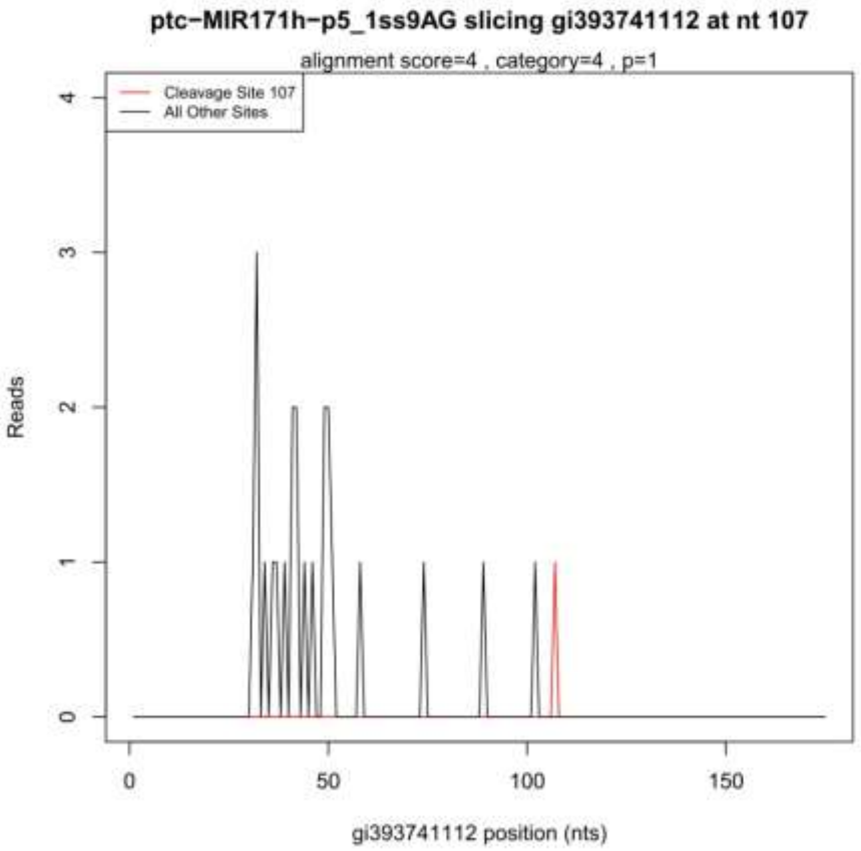
ptc−MIR171h−p5_1ss9AG slicing gi393741112 at nt 107
alignment score=4 , category=4 , p=1
Cleavage Site 107
All Other Sites
Reads
0
1
2
3
4
0
50
100
150
gi393741112 position (nts)

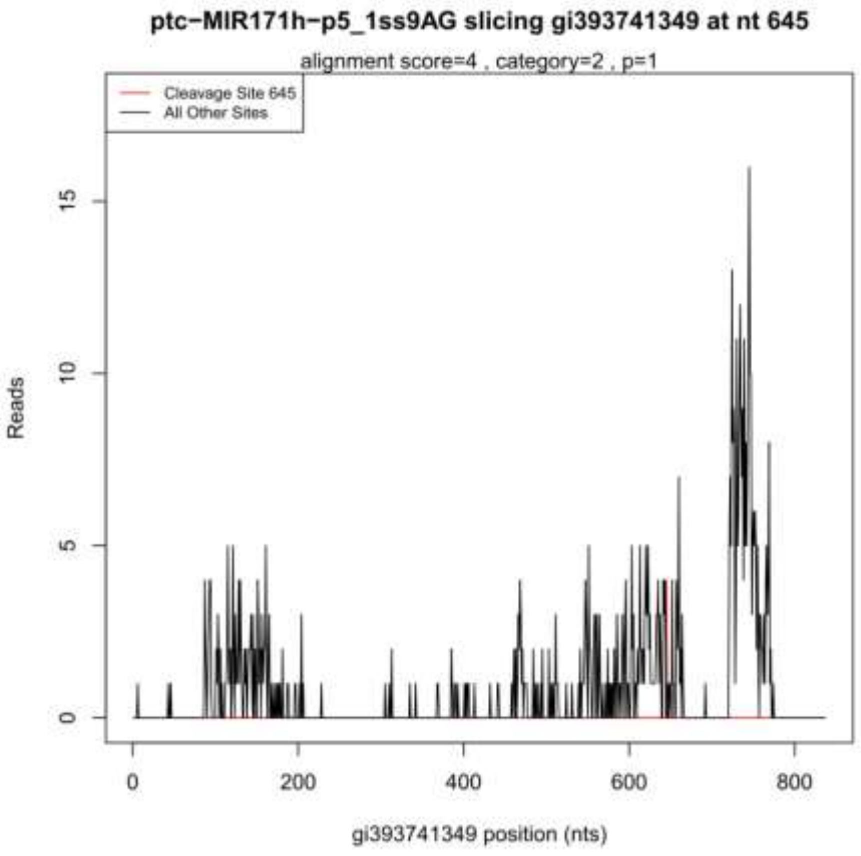
ptc−MIR171h−p5_1ss9AG slicing gi393741349 at nt 645
alignment score=4 , category=2 , p=1
Cleavage Site 645
All Other Sites
Reads
0
5
10
15
0
200
400
600
800
gi393741349 position (nts)

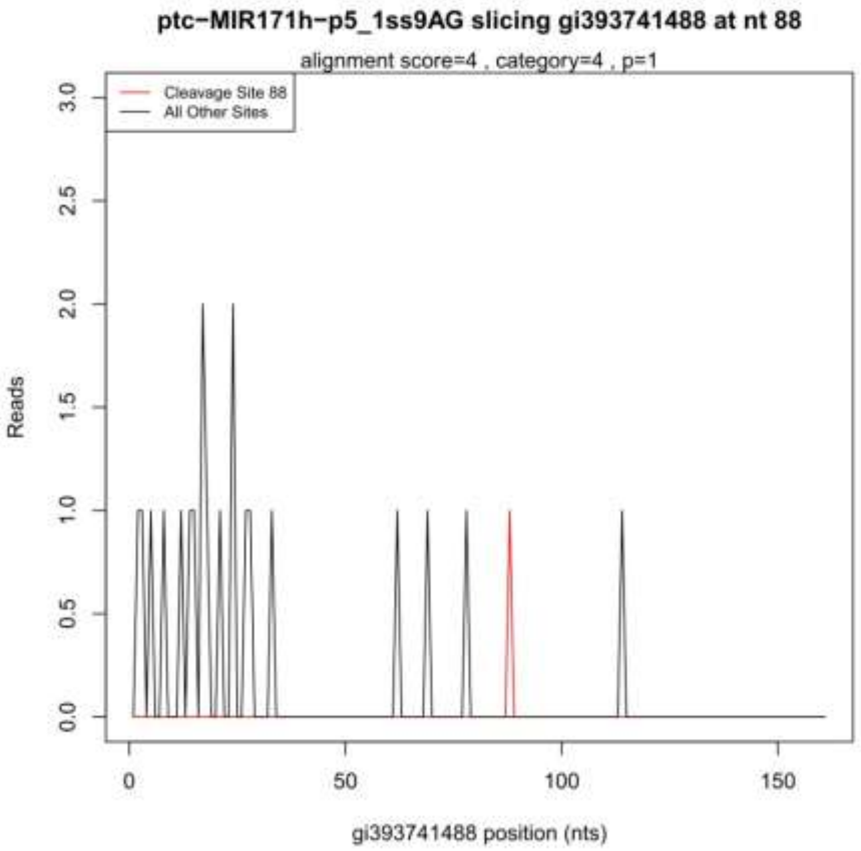

ptc−MIR171h−p5_1ss9AG slicing gi393741488 at nt 88
alignment score=4 , category=4 , p=1
Cleavage Site 88
All Other Sites
Reads
0.0
0.5
1.0
1.5
2.0
2.5
3.0
0
50
100
150
gi393741488 position (nts)

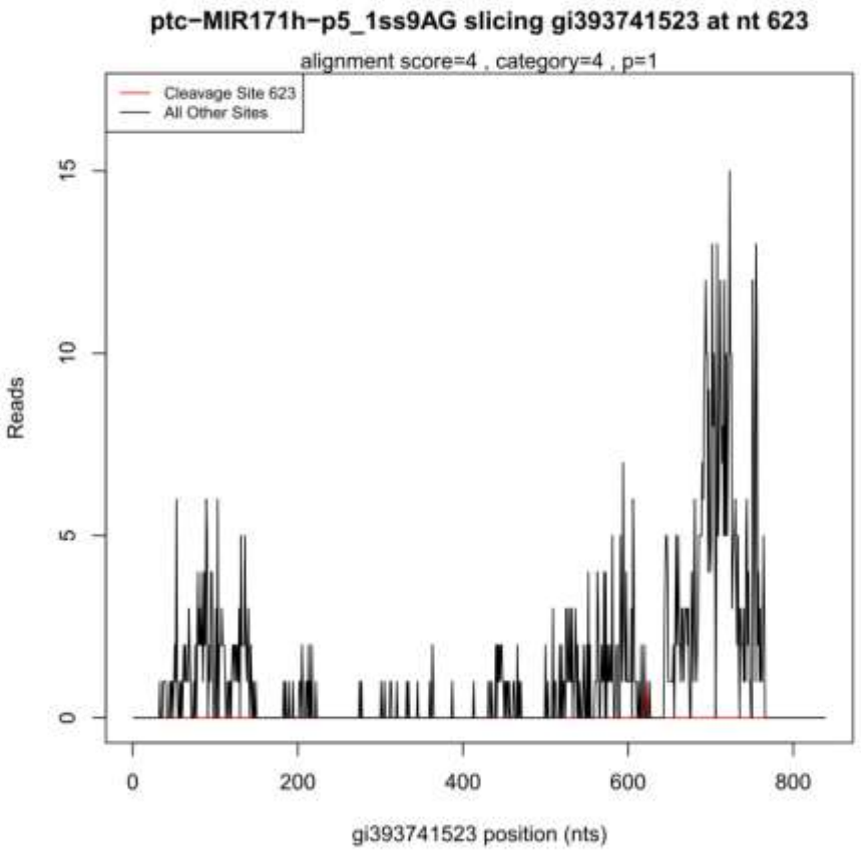

ptc−MIR171h−p5_1ss9AG slicing gi393741523 at nt 623
alignment score=4 , category=4 , p=1
Cleavage Site 623
All Other Sites
Reads
0
5
10
15
0
200
400
600
800
gi393741523 position (nts)

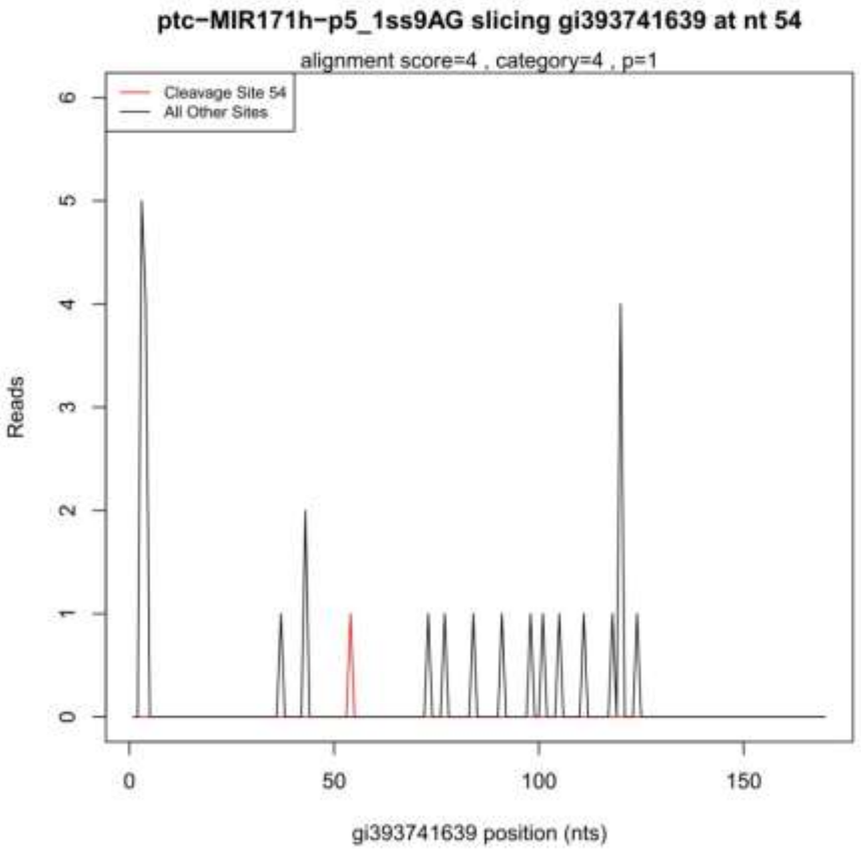

ptc−MIR171h−p5_1ss9AG slicing gi393741639 at nt 54
alignment score=4 , category=4 , p=1
Cleavage Site 54
All Other Sites
Reads
0
1
2
3
4
5
6
0
50
100
150
gi393741639 position (nts)

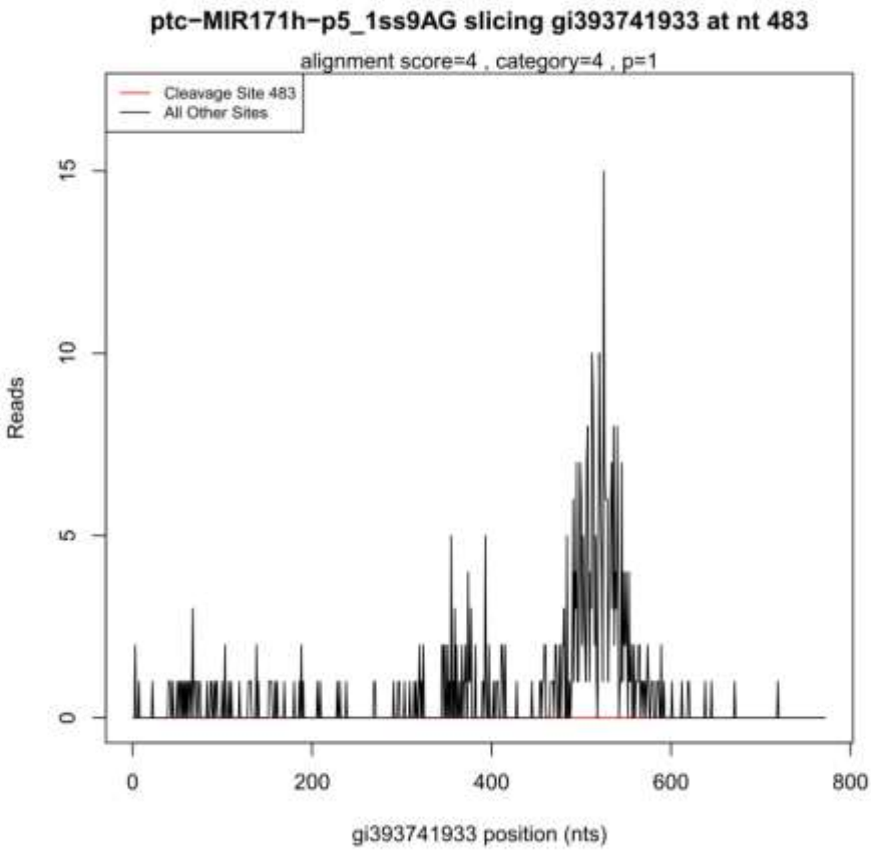

ptc−MIR171h−p5_1ss9AG slicing gi393741933 at nt 483
alignment score=4 , category=4 , p=1
Cleavage Site 483
All Other Sites
Reads
0
5
10
15
0
200
400
600
800
gi393741933 position (nts)

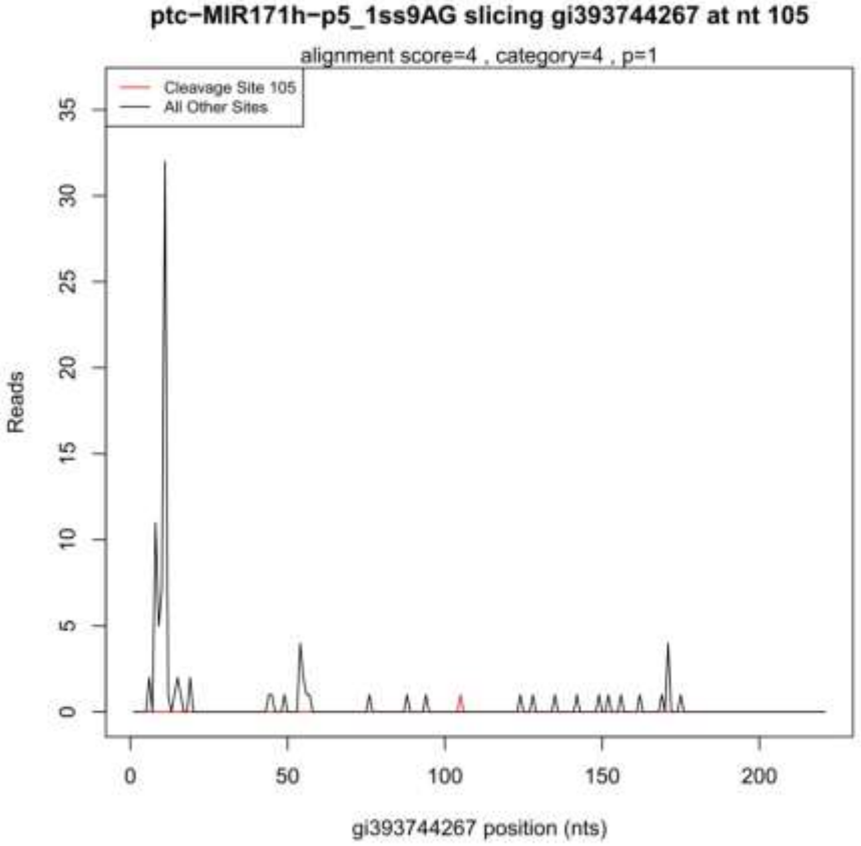

ptc−MIR171h−p5_1ss9AG slicing gi393744267 at nt 105
alignment score=4 , category=4 , p=1
Cleavage Site 105
All Other Sites
Reads
gi393744267 position (nts)

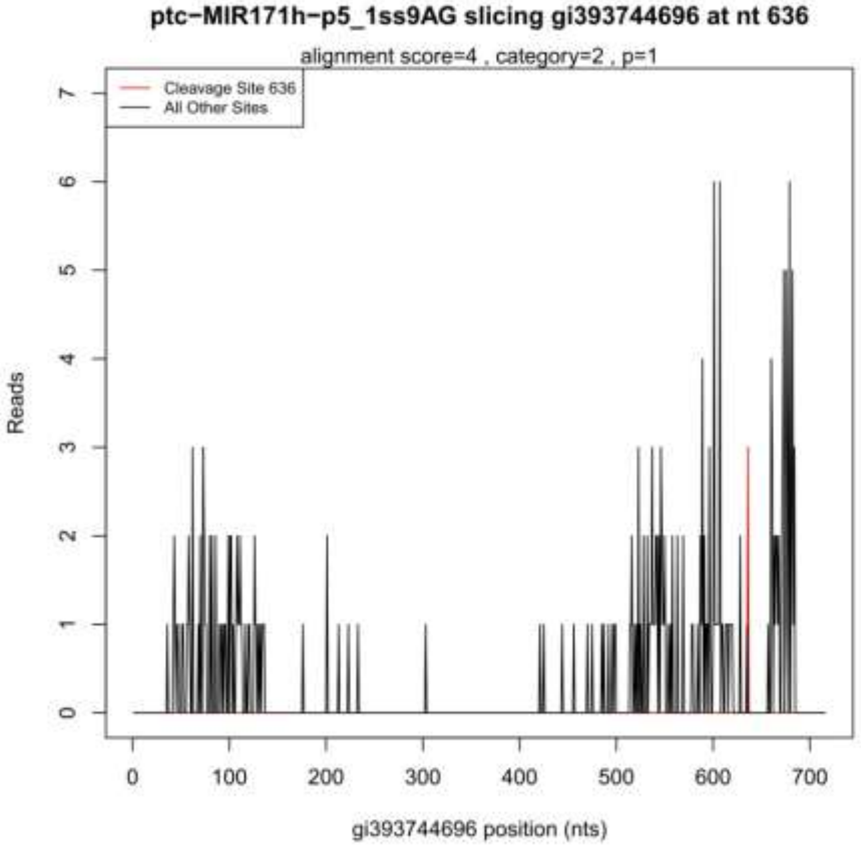

ptc−MIR171h−p5_1ss9AG slicing gi393744696 at nt 636
alignment score=4 , category=2 , p=1
Cleavage Site 636
All Other Sites
Reads
gi393744696 position (nts)

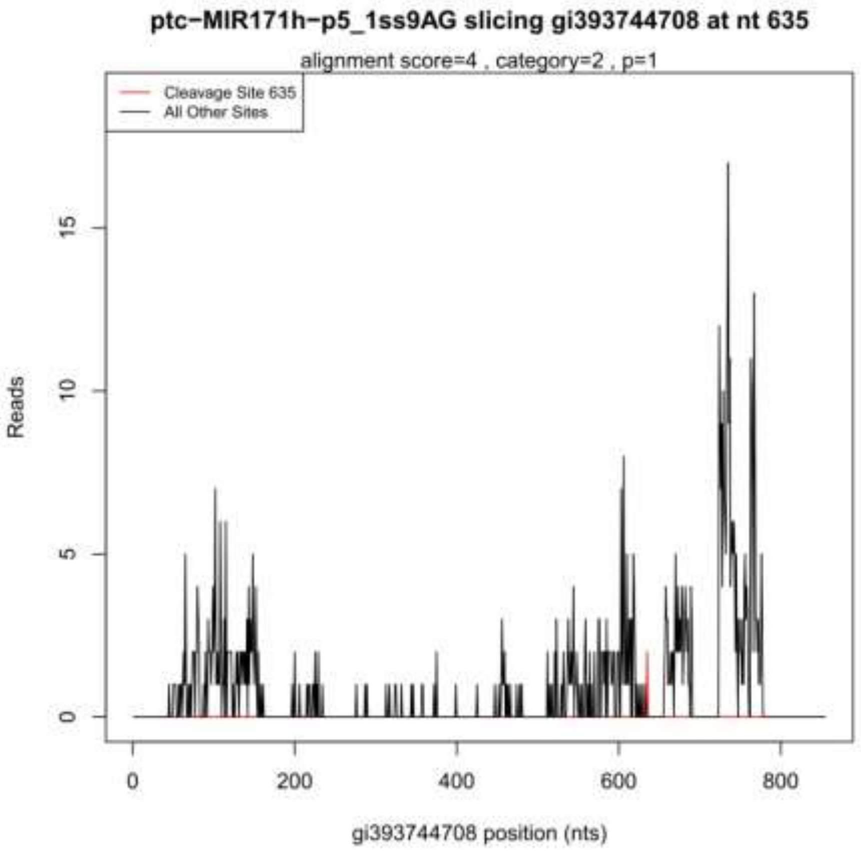
ptc−MIR171h−p5_1ss9AG slicing gi393744708 at nt 635
alignment score=4 , category=2 , p=1
Cleavage Site 635
All Other Sites
Reads
0
5
10
15
0
200
400
600
800
gi393744708 position (nts)

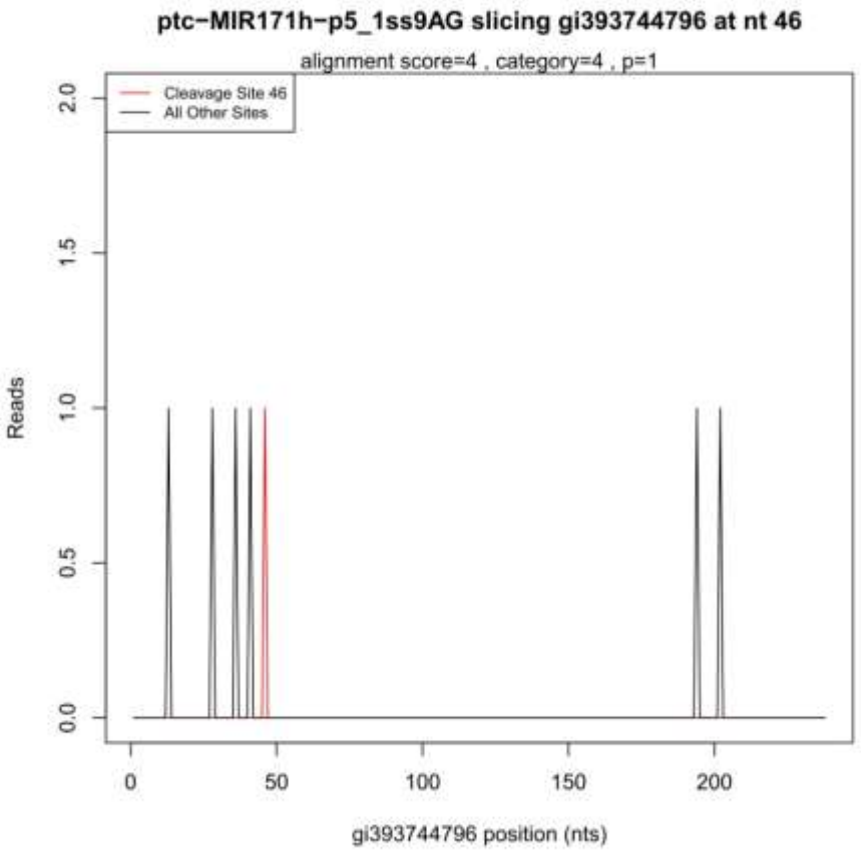
ptc−MIR171h−p5_1ss9AG slicing gi393744796 at nt 46
alignment score=4 , category=4 , p=1
Cleavage Site 46
All Other Sites
Reads
0.0
0.5
1.0
1.5
2.0
0
50
100
150
200
gi393744796 position (nts)

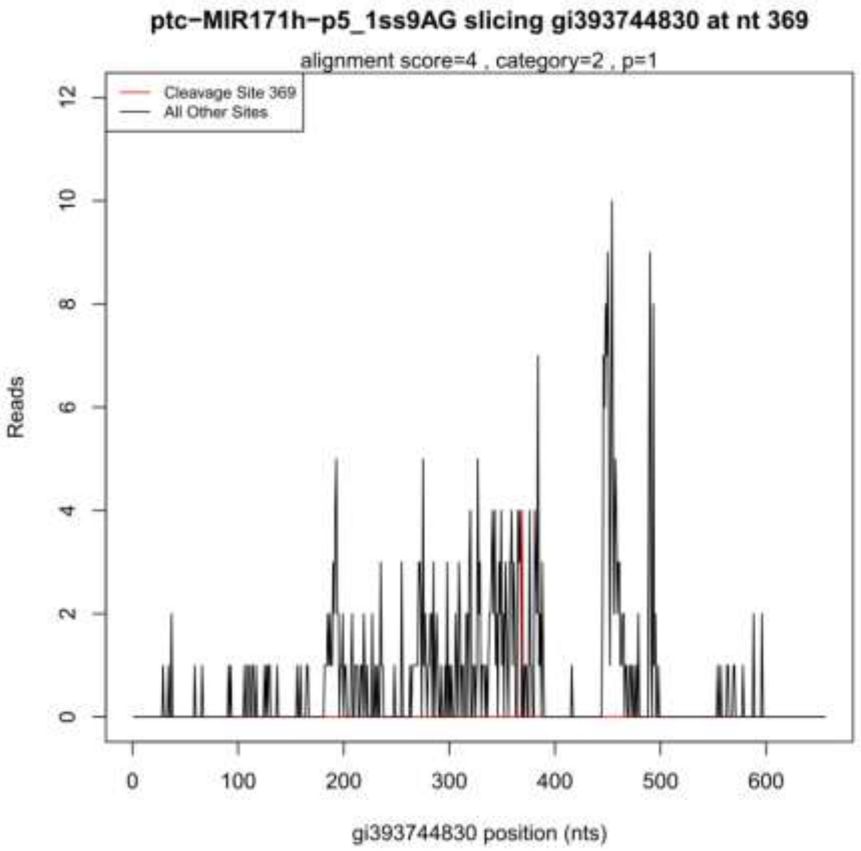
ptc−MIR171h−p5_1ss9AG slicing gi393744830 at nt 369
alignment score=4 , category=2 , p=1
Cleavage Site 369
All Other Sites
Reads
0
2
4
6
8
10
12
0
100
200
300
400
500
600
gi393744830 position (nts)

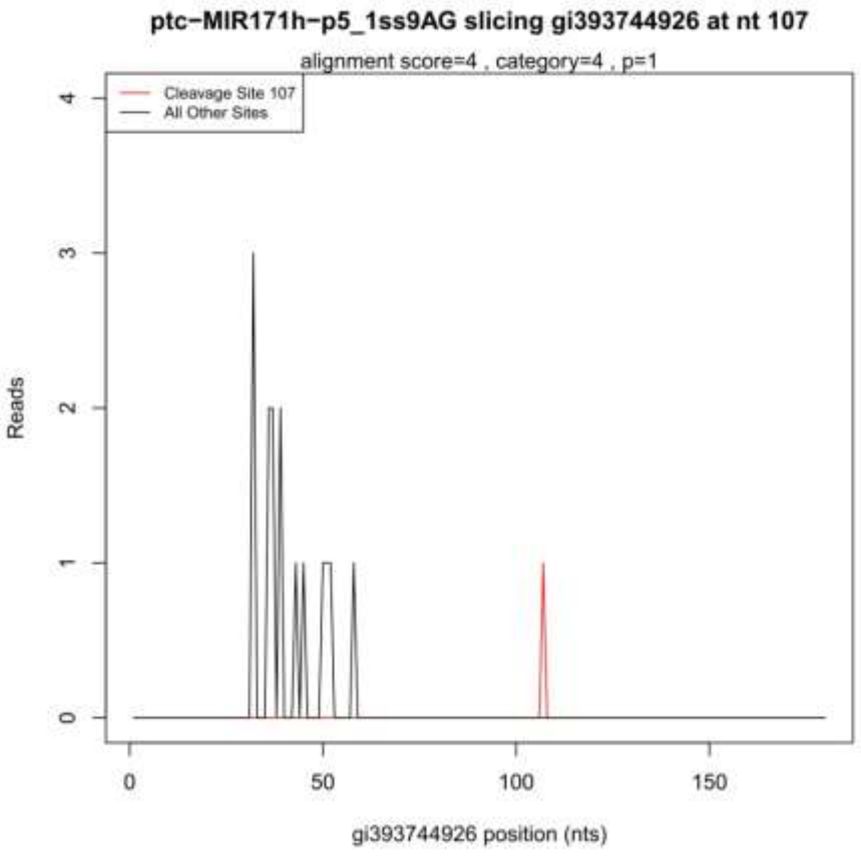
ptc−MIR171h−p5_1ss9AG slicing gi393744926 at nt 107
alignment score=4 , category=4 , p=1
Cleavage Site 107
All Other Sites
Reads
0
1
2
3
4
0
50
100
150
gi393744926 position (nts)

ptc-MIR171h-p5_1ss9AG slicing gi393745964 at nt 432

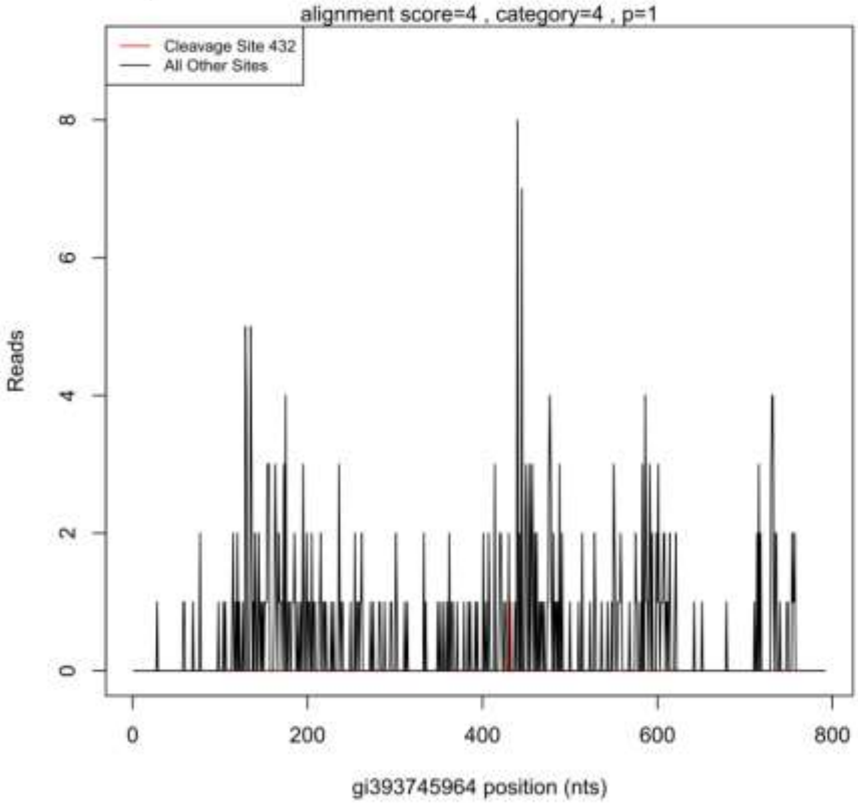

ptc-MIR171h-p5_1ss9AG slicing gi393746120 at nt 107

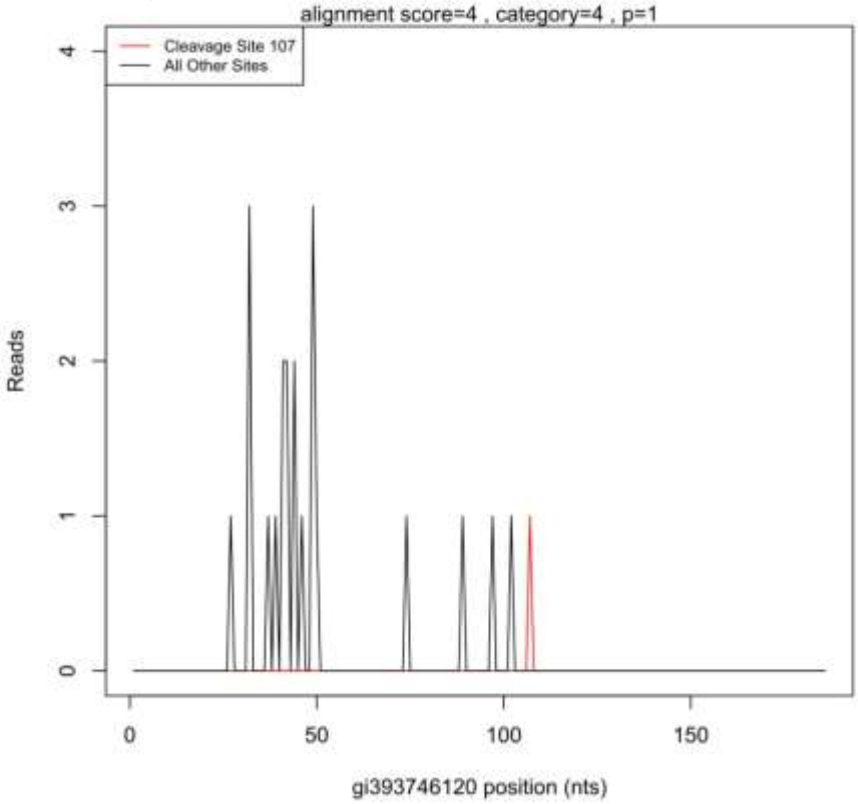

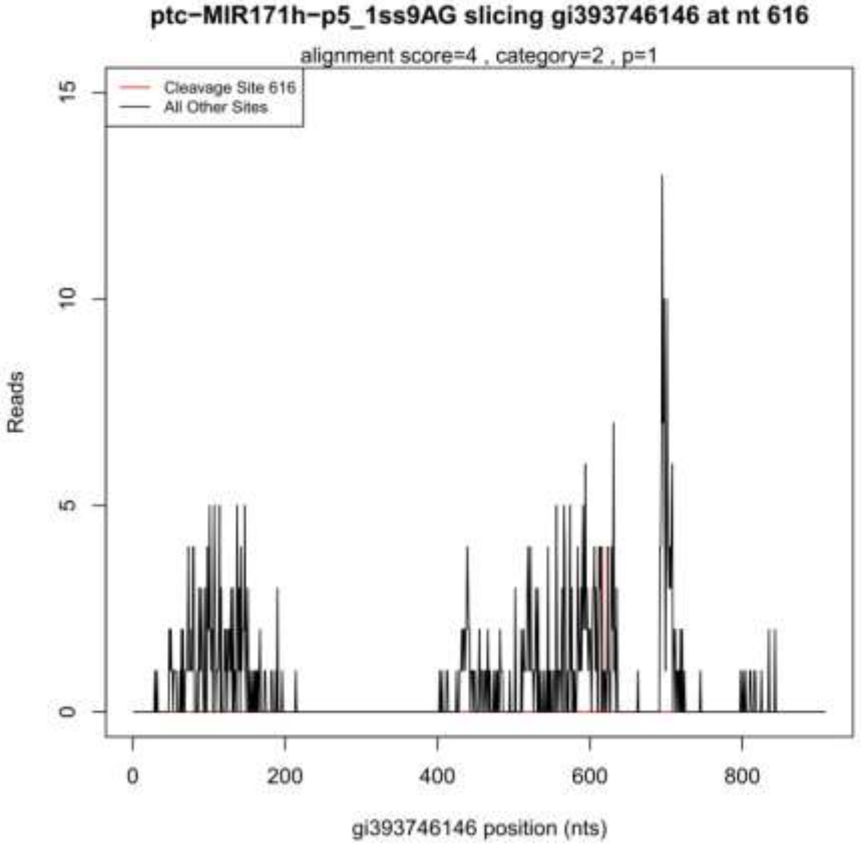

ptc−MIR171h−p5_1ss9AG slicing gi393746146 at nt 616
alignment score=4 , category=2 , p=1
Cleavage Site 616
All Other Sites
Reads
0
5
10
15
0
200
400
600
800
gi393746146 position (nts)

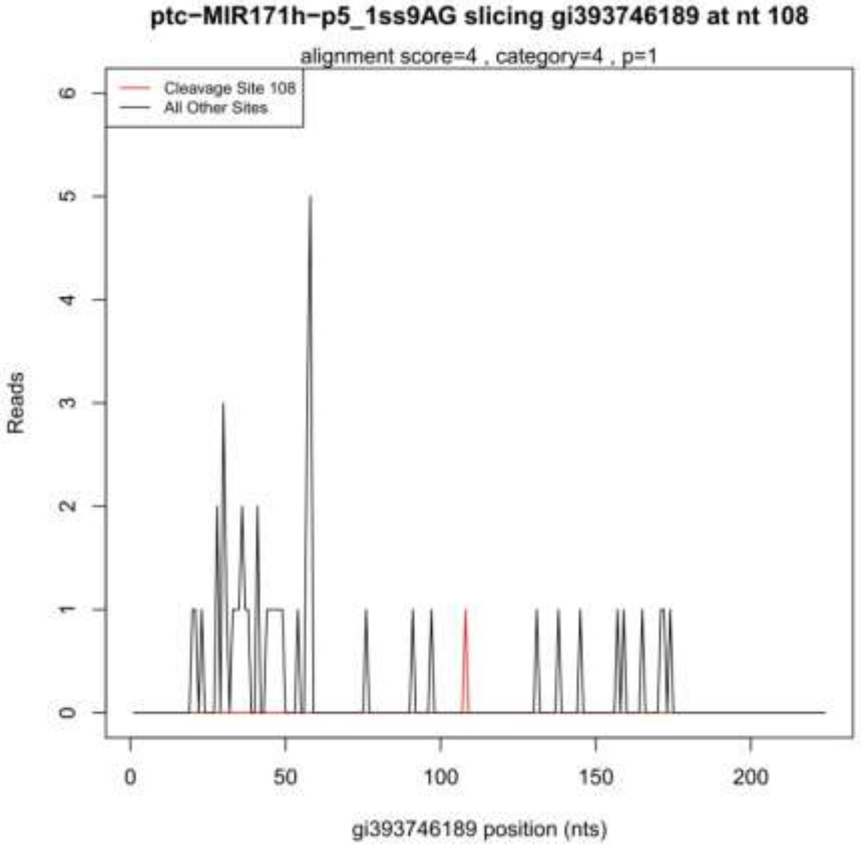

ptc−MIR171h−p5_1ss9AG slicing gi393746189 at nt 108
alignment score=4 , category=4 , p=1
Cleavage Site 108
All Other Sites
Reads
0
1
2
3
4
5
6
0
50
100
150
200
gi393746189 position (nts)

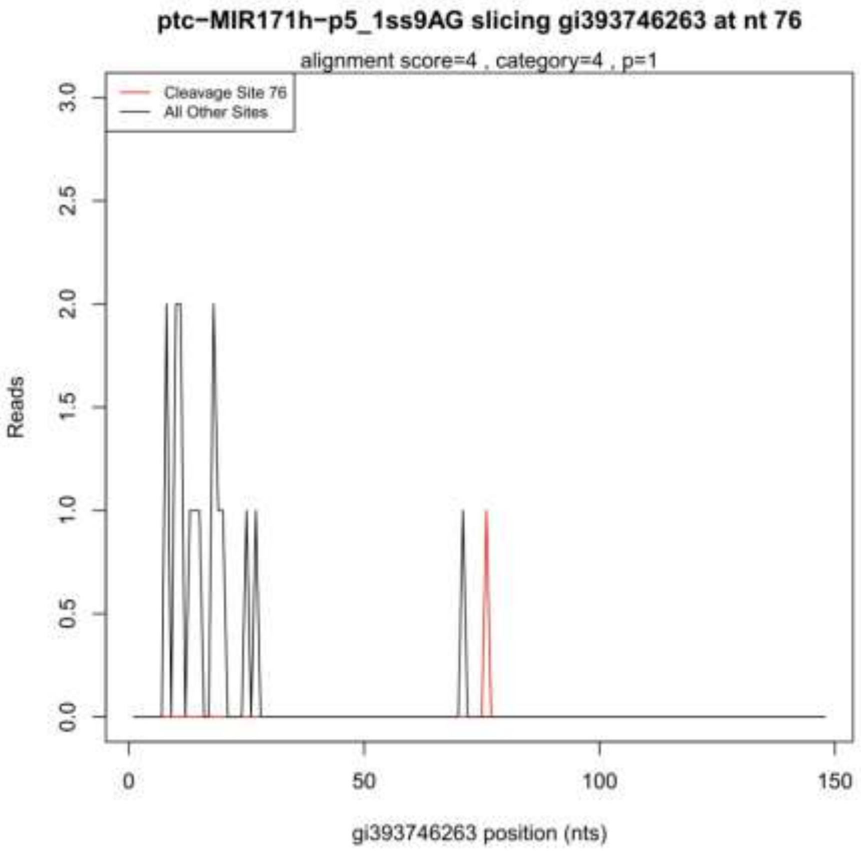

ptc−MIR171h−p5_1ss9AG slicing gi393746263 at nt 76
alignment score=4 , category=4 , p=1
Cleavage Site 76
All Other Sites
Reads
0.0
0.5
1.0
1.5
2.0
2.5
3.0
0
50
100
150
gi393746263 position (nts)

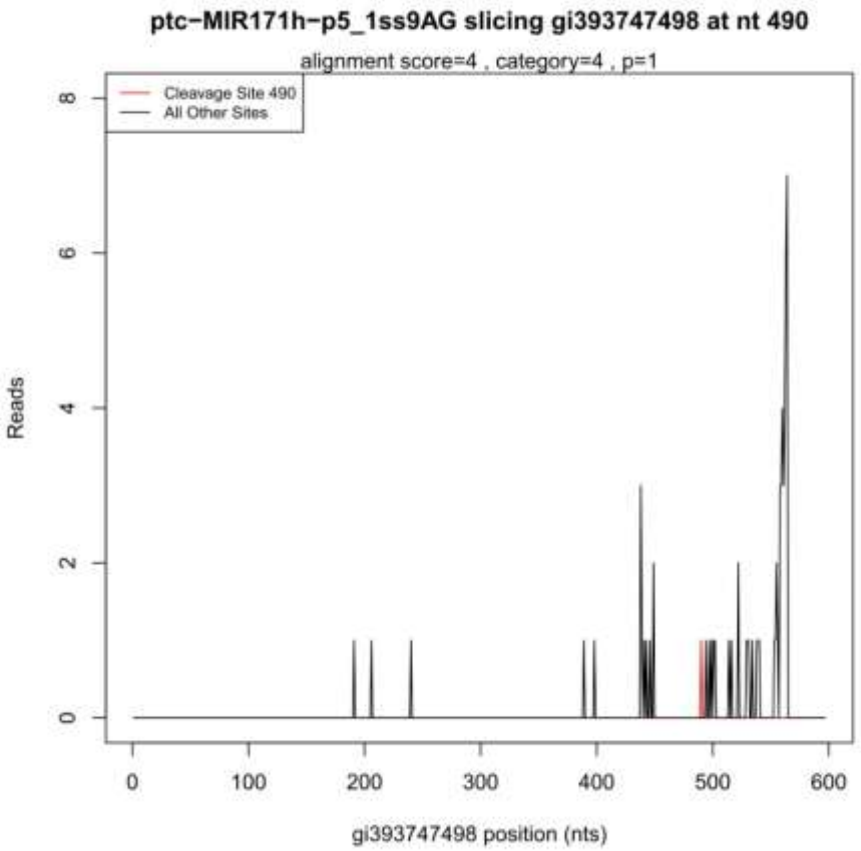

ptc−MIR171h−p5_1ss9AG slicing gi393747498 at nt 490
alignment score=4 , category=4 , p=1
Cleavage Site 490
All Other Sites
Reads
0
2
4
6
8
0
100
200
300
400
500
600
gi393747498 position (nts)

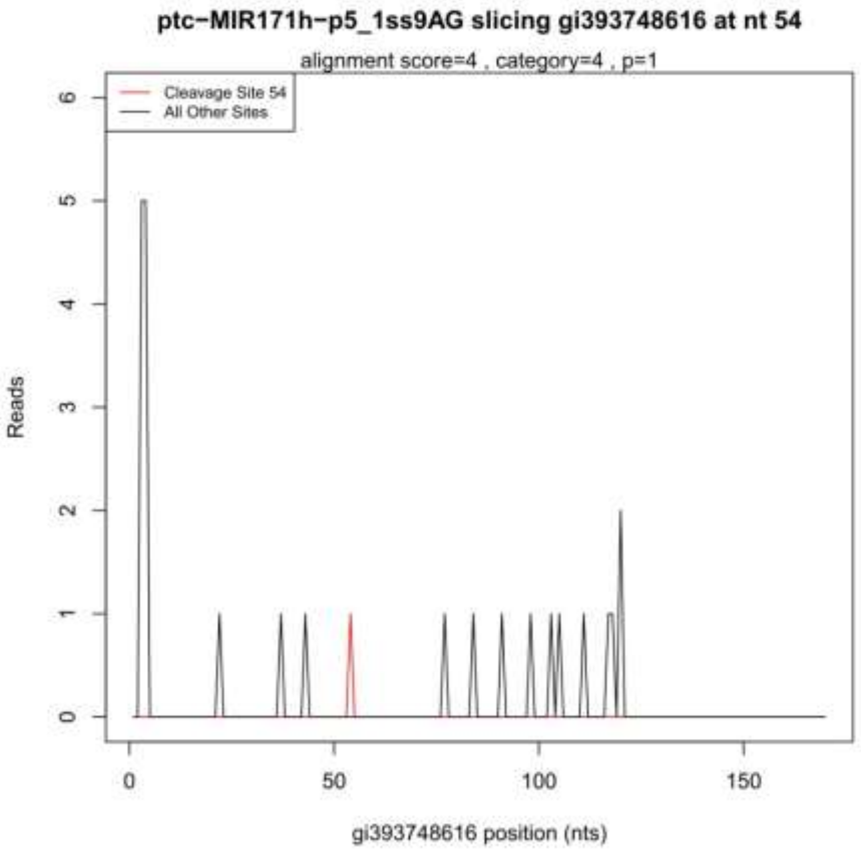
ptc−MIR171h−p5_1ss9AG slicing gi393748616 at nt 54
alignment score=4 , category=4 , p=1
Cleavage Site 54
All Other Sites
Reads
0
1
2
3
4
5
6
0
50
100
150
gi393748616 position (nts)

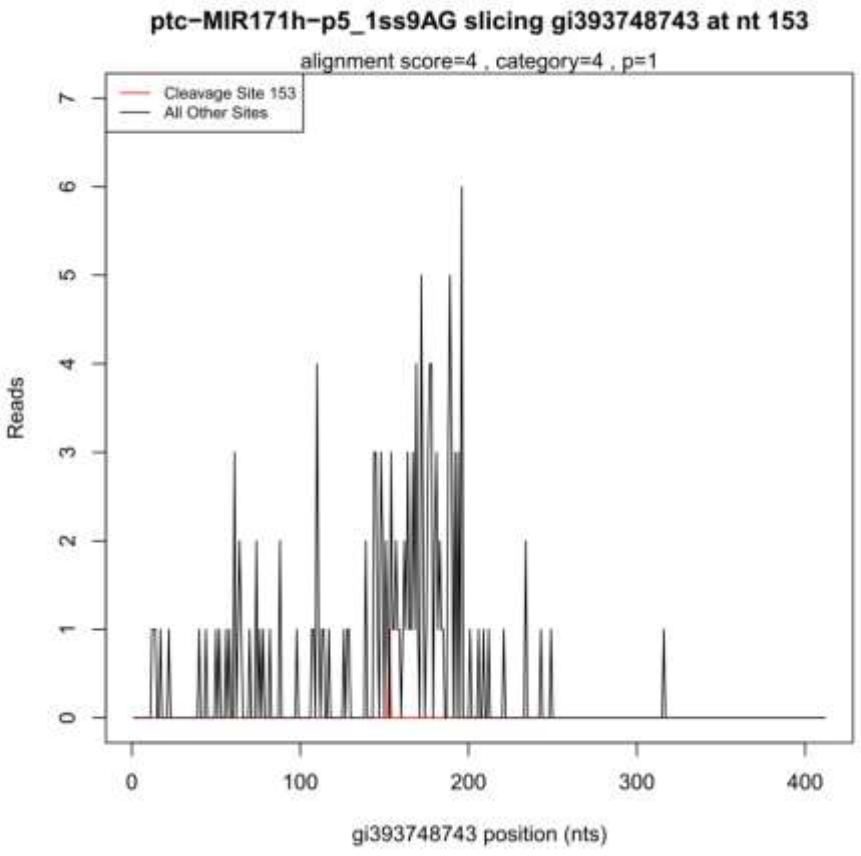
ptc−MIR171h−p5_1ss9AG slicing gi393748743 at nt 153
alignment score=4 , category=4 , p=1
Cleavage Site 153
All Other Sites
Reads
0
1
2
3
4
5
6
7
0
100
200
300
400
gi393748743 position (nts)

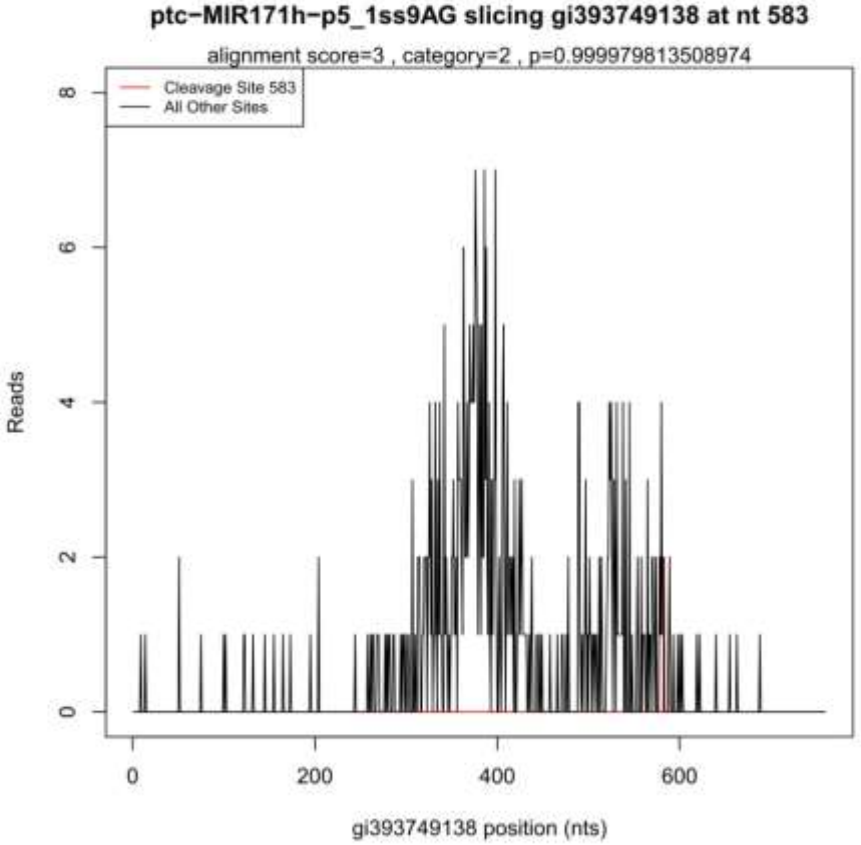
ptc−MIR171h−p5_1ss9AG slicing gi393749138 at nt 583
alignment score=3 , category=2 , p=0.999979813508974
Cleavage Site 583
All Other Sites
Reads
0
2
4
6
8
0
200
400
600
gi393749138 position (nts)

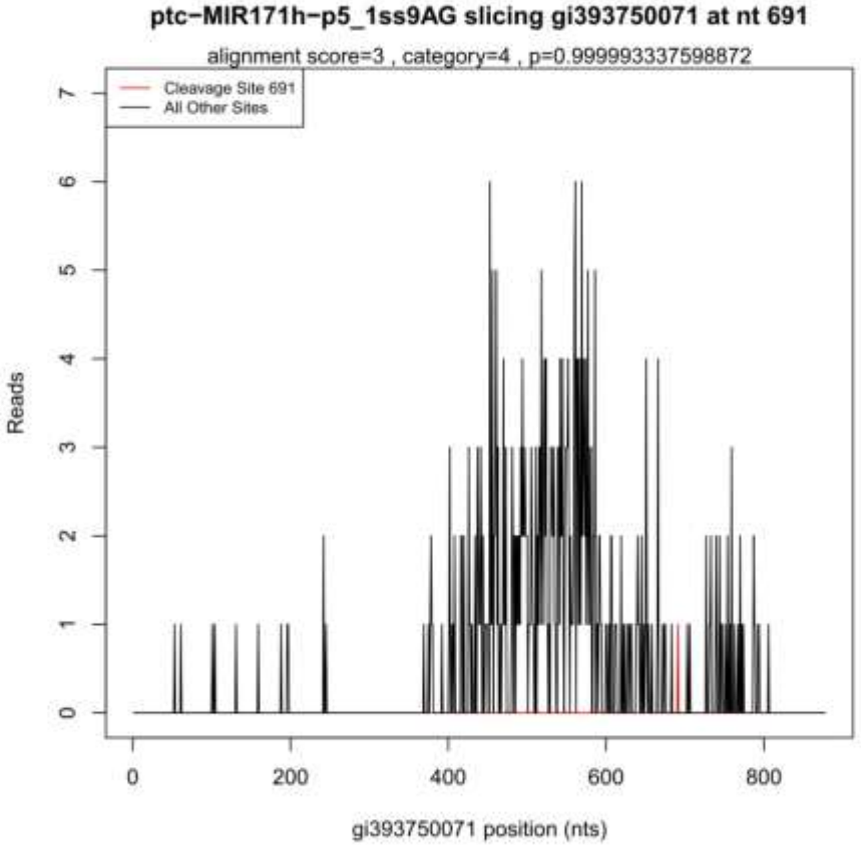
ptc−MIR171h−p5_1ss9AG slicing gi393750071 at nt 691
alignment score=3 , category=4 , p=0.999993337598872
Cleavage Site 691
All Other Sites
Reads
0
1
2
3
4
5
6
7
0
200
400
600
800
gi393750071 position (nts)

ptc−MIR171h−p5_1ss9AG slicing gi393751104 at nt 311

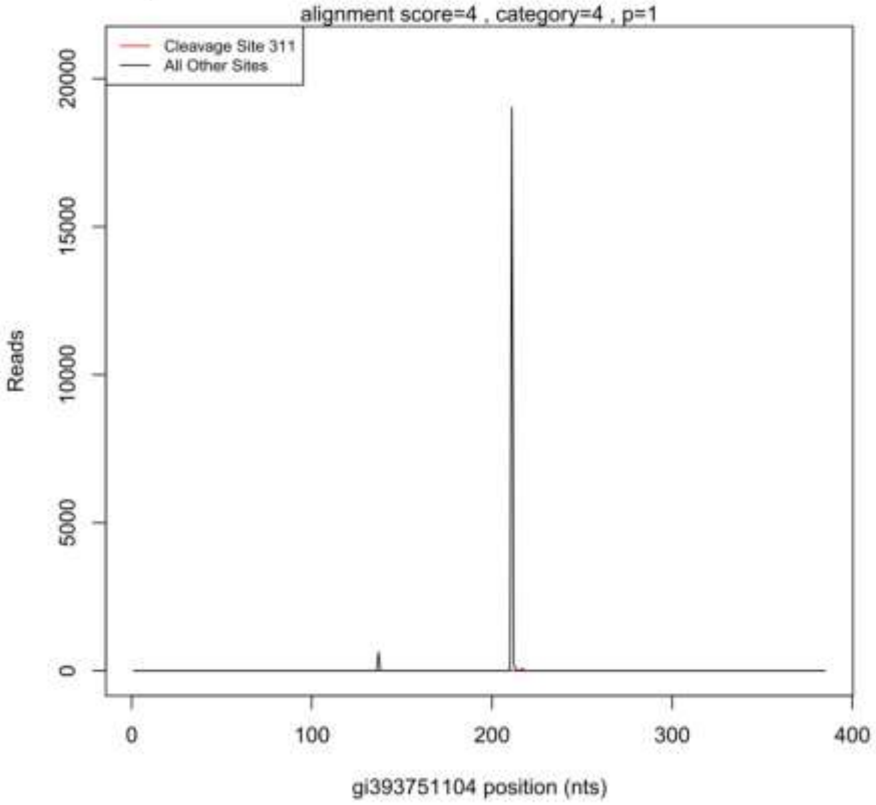

ptc−MIR171h−p5_1ss9AG slicing gi393751380 at nt 92

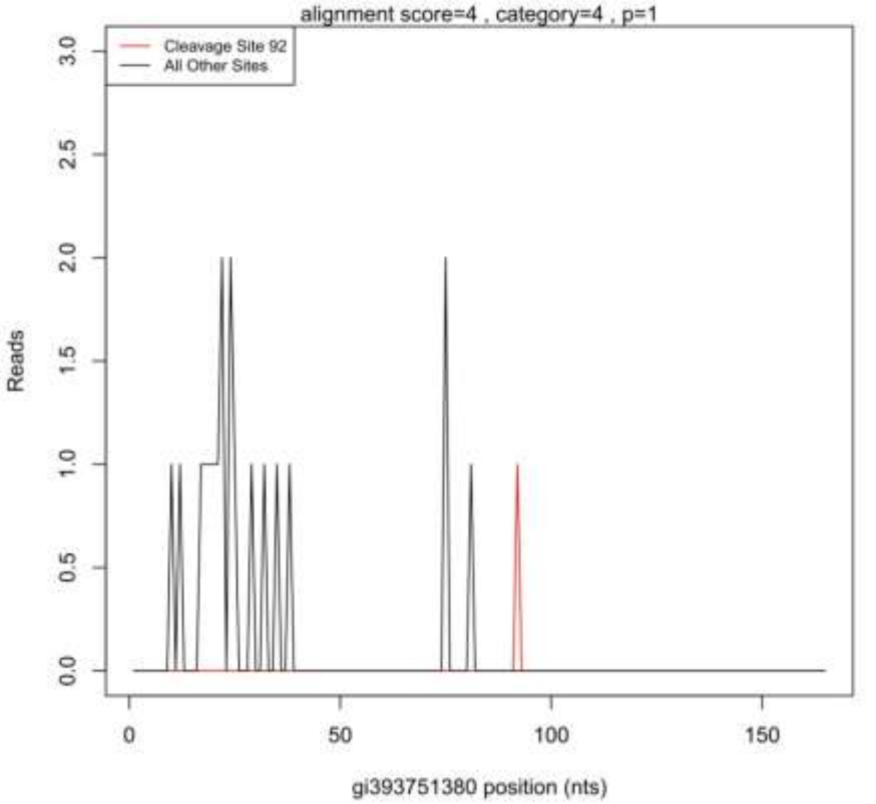

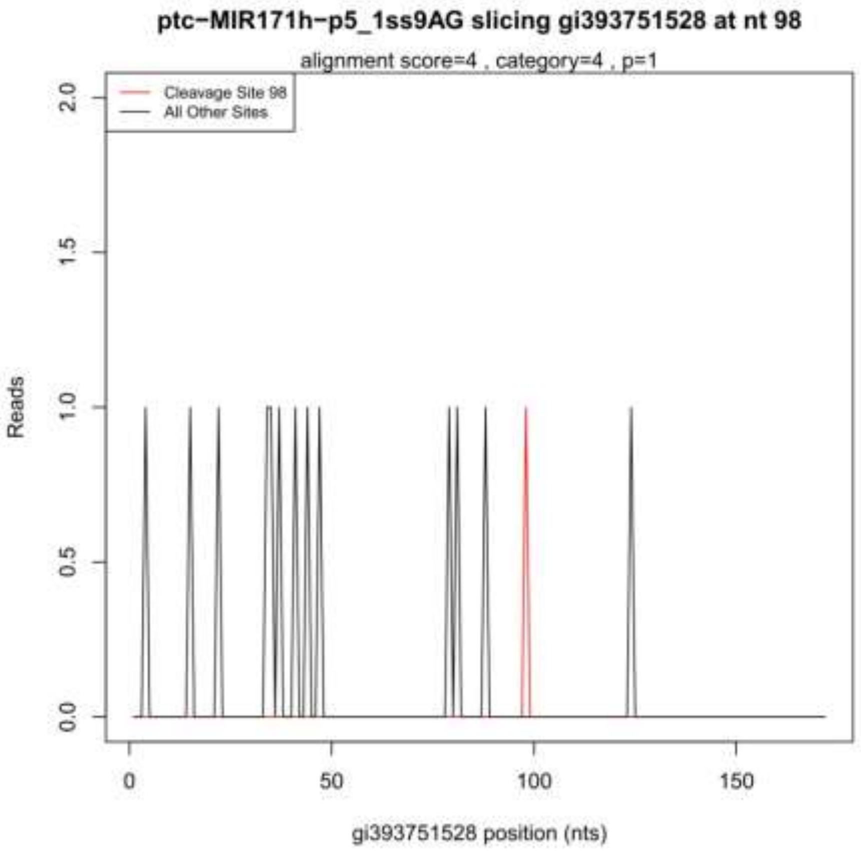
ptc−MIR171h−p5_1ss9AG slicing gi393751528 at nt 98
alignment score=4 , category=4 , p=1
Cleavage Site 98
All Other Sites
Reads
0.0
0.5
1.0
1.5
2.0
0
50
100
150
gi393751528 position (nts)

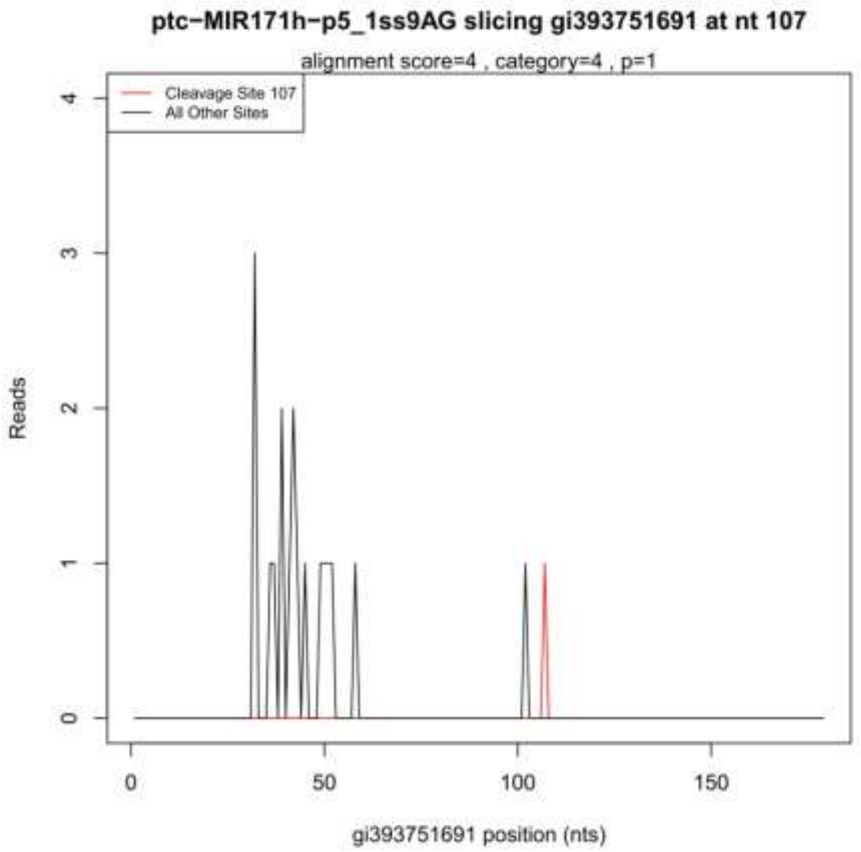
ptc−MIR171h−p5_1ss9AG slicing gi393751691 at nt 107
alignment score=4 , category=4 , p=1
Cleavage Site 107
All Other Sites
Reads
0
1
2
3
4
0
50
100
150
gi393751691 position (nts)

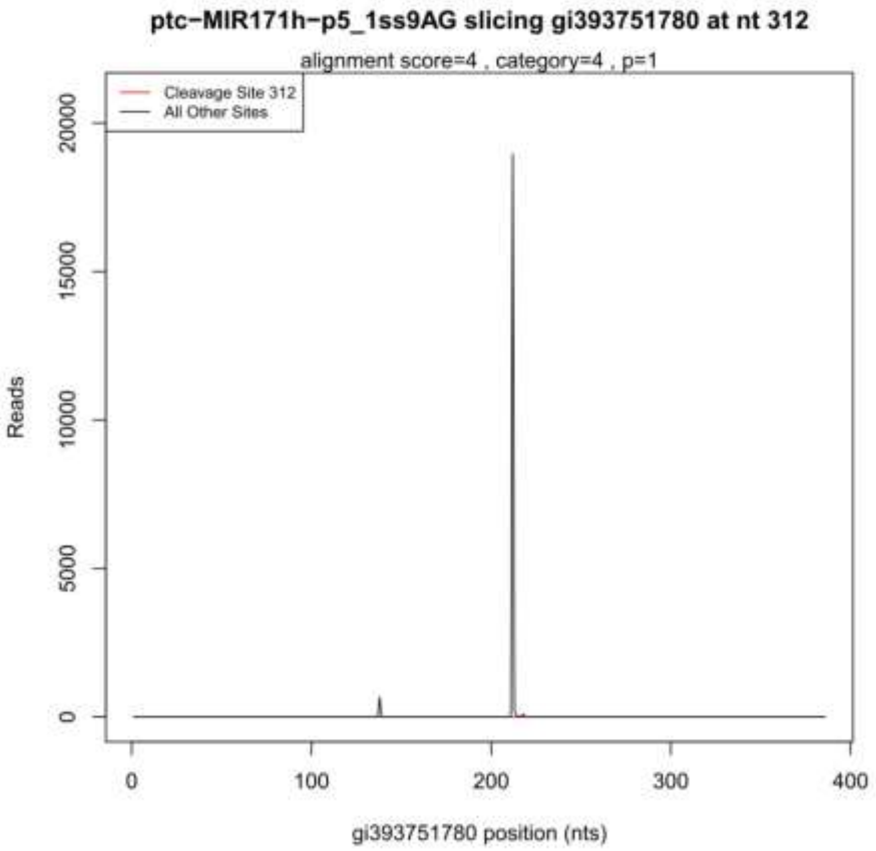

ptc−MIR171h−p5_1ss9AG slicing gi393751780 at nt 312
alignment score=4 , category=4 , p=1
Cleavage Site 312
All Other Sites
Reads
0
5000
10000
15000
20000
0
100
200
300
400
gi393751780 position (nts)

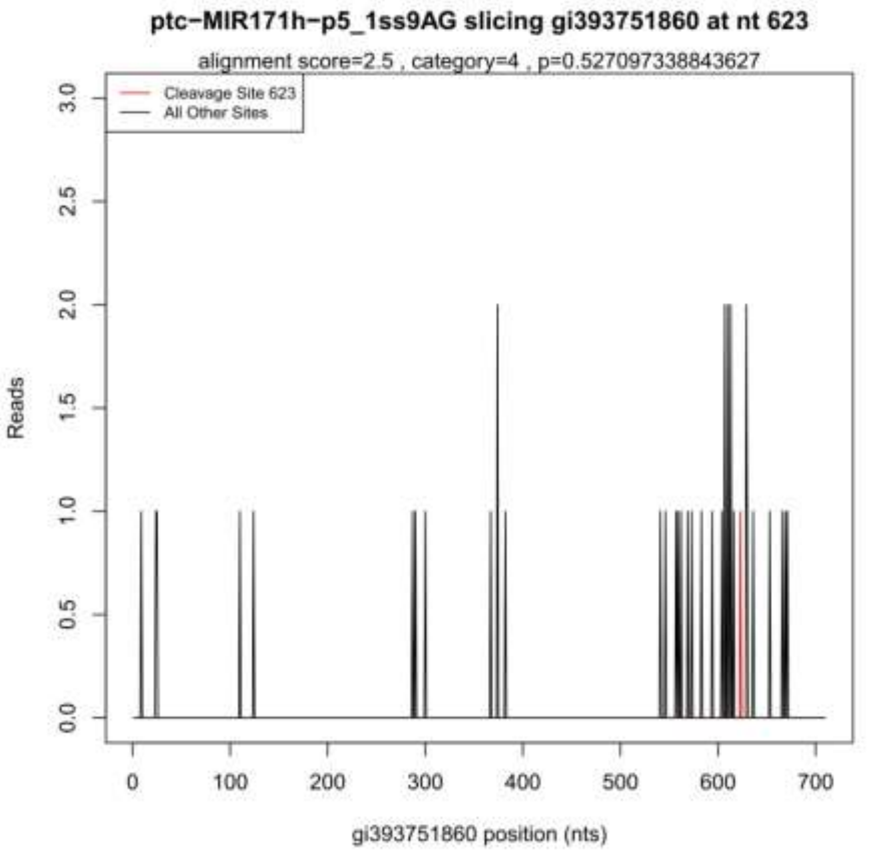

ptc−MIR171h−p5_1ss9AG slicing gi393751860 at nt 623
alignment score=2.5 , category=4 , p=0.527097338843627
Cleavage Site 623
All Other Sites
Reads
0.0
0.5
1.0
1.5
2.0
2.5
3.0
0
100
200
300
400
500
600
700
gi393751860 position (nts)

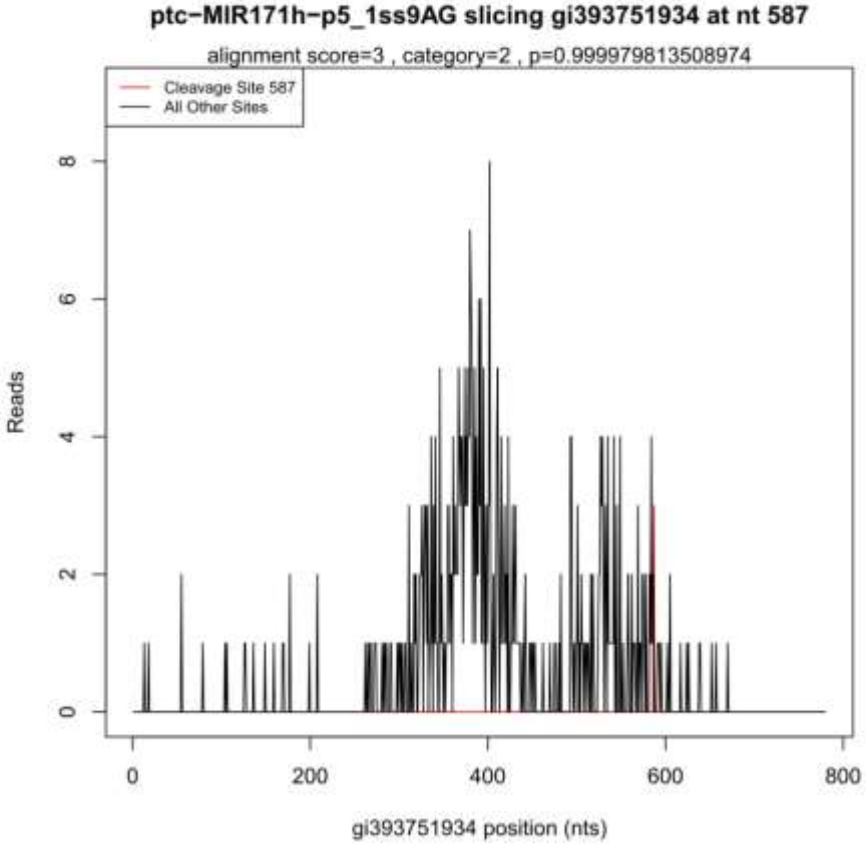
ptc−MIR171h−p5_1ss9AG slicing gi393751934 at nt 587
alignment score=3 , category=2 , p=0.999979813508974
Cleavage Site 587
All Other Sites
Reads
0
2
4
6
8
0
200
400
600
800
gi393751934 position (nts)

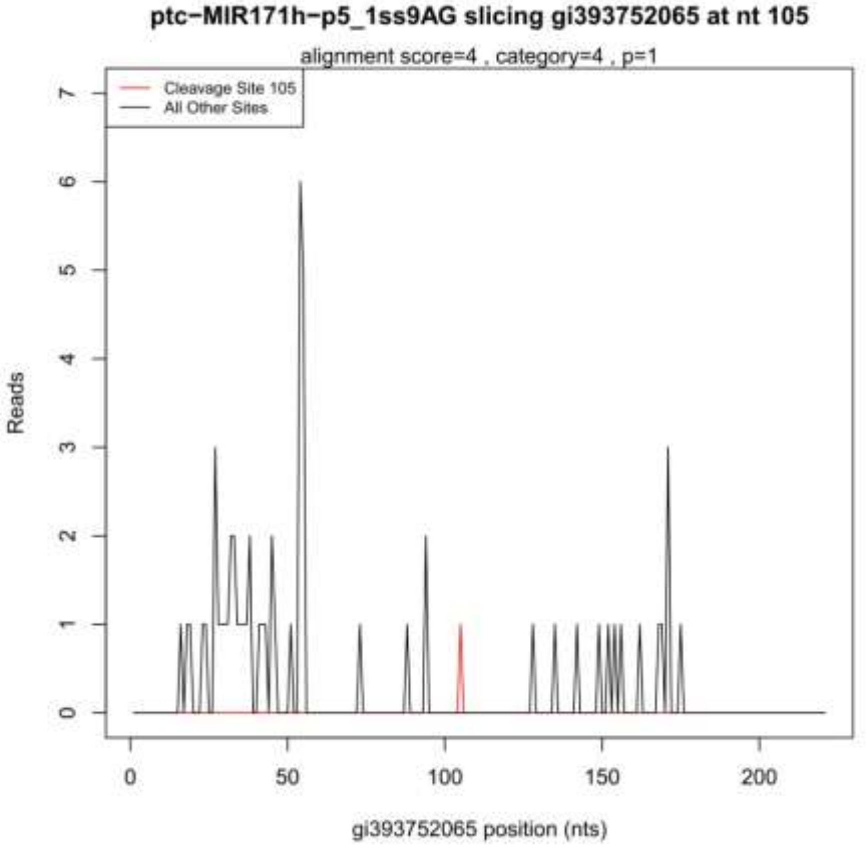
ptc−MIR171h−p5_1ss9AG slicing gi393752065 at nt 105
alignment score=4 , category=4 , p=1
Cleavage Site 105
All Other Sites
Reads
0
1
2
3
4
5
6
7
0
50
100
150
200
gi393752065 position (nts)

ptc−MIR171h−p5_1ss9AG slicing gi393752214 at nt 170

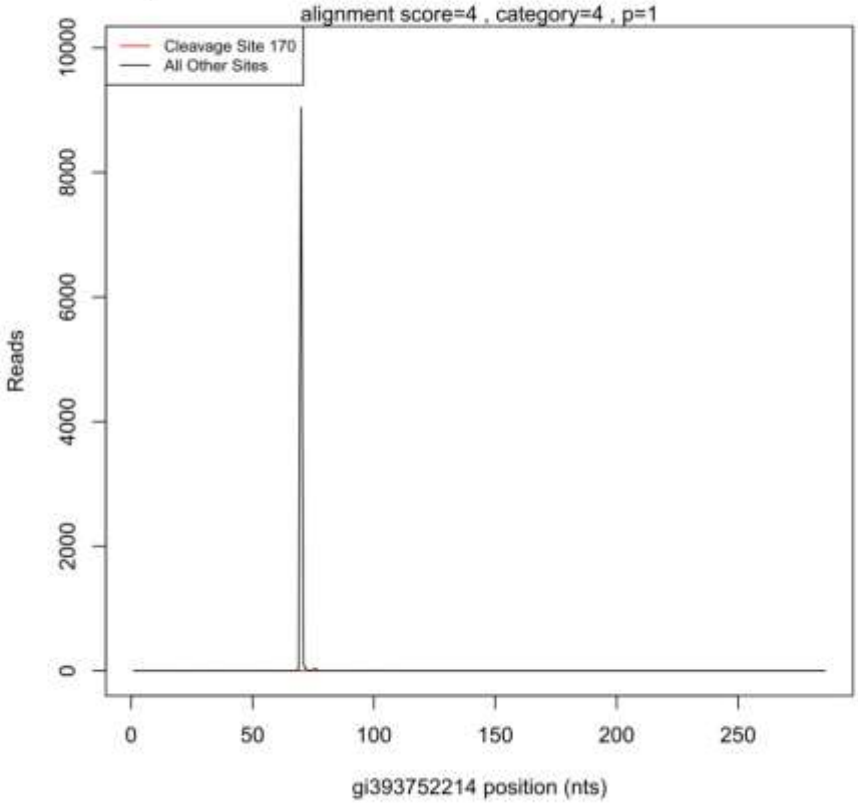

ptc−MIR171h−p5_1ss9AG slicing gi393752441 at nt 108

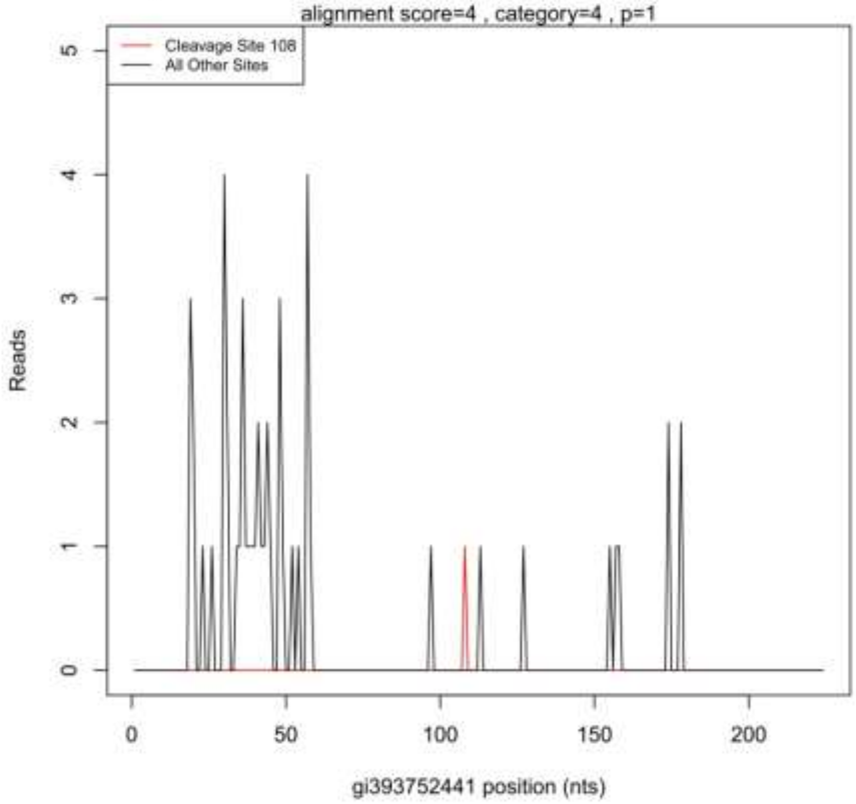

ptc−MIR171h−p5_1ss9AG slicing gi393752884 at nt 641

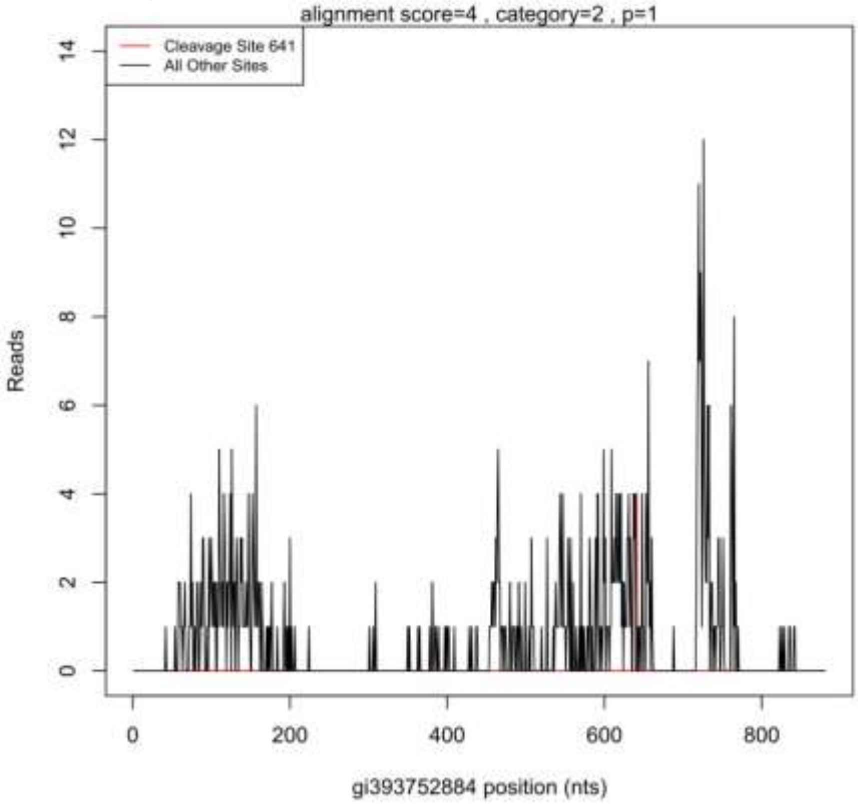

ptc−MIR171h−p5_1ss9AG slicing gi393753024 at nt 769

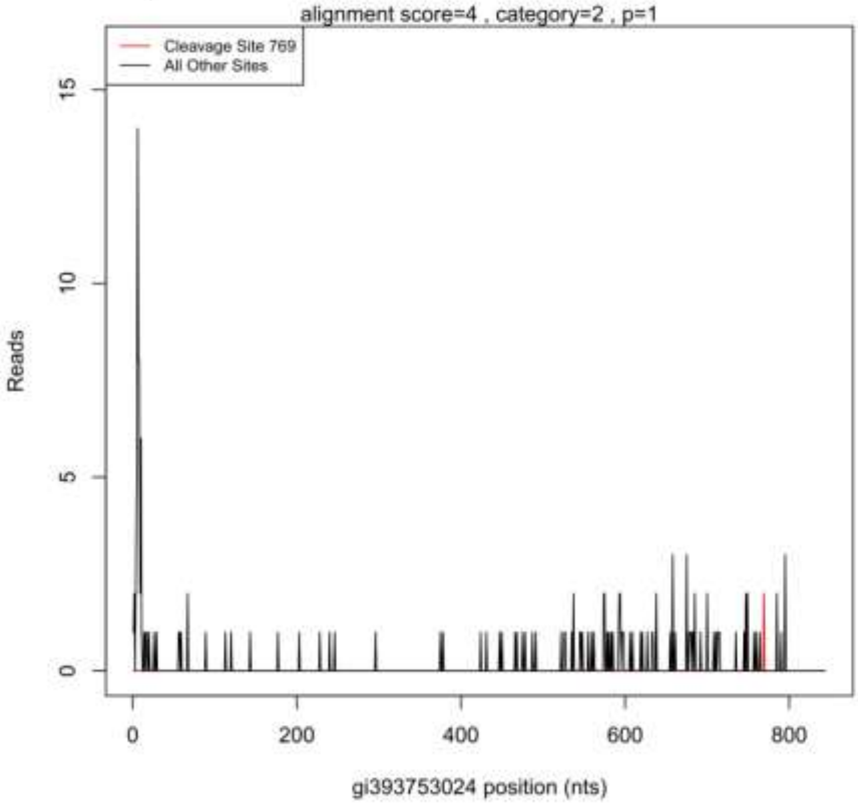

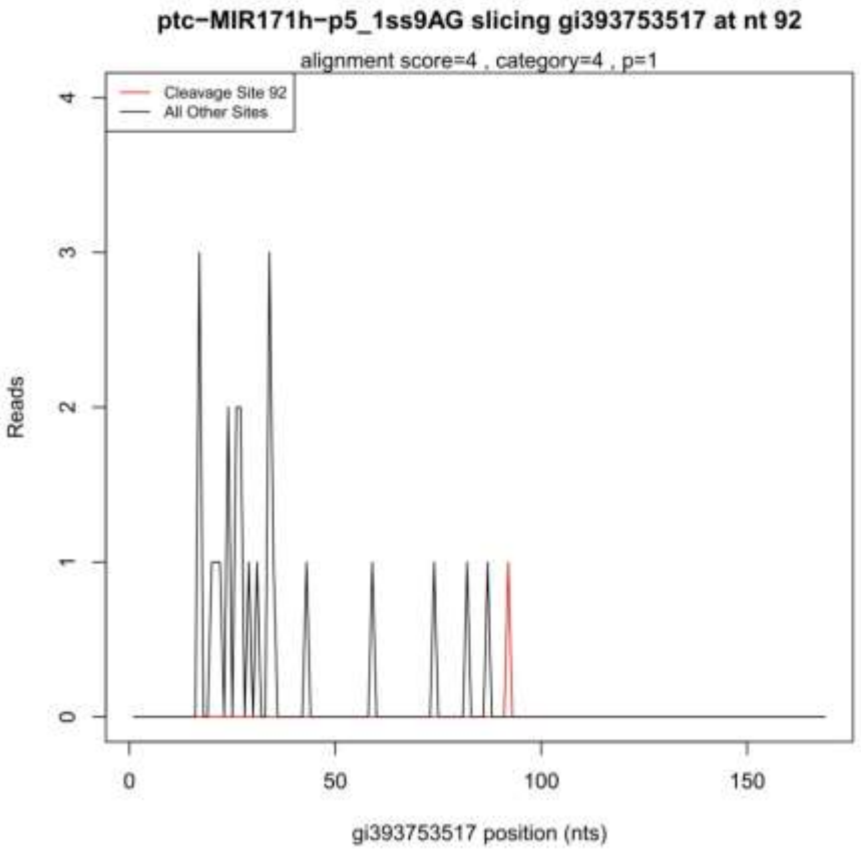
ptc−MIR171h−p5_1ss9AG slicing gi393753517 at nt 92
alignment score=4 , category=4 , p=1
Cleavage Site 92
All Other Sites
Reads
0
1
2
3
4
0
50
100
150
gi393753517 position (nts)

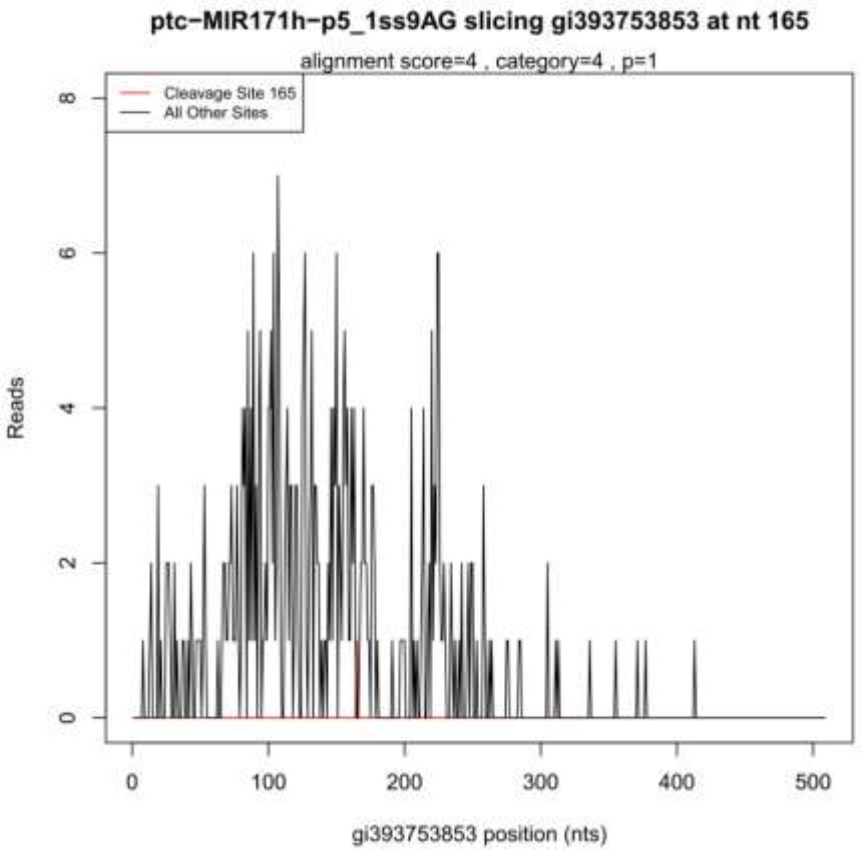
ptc−MIR171h−p5_1ss9AG slicing gi393753853 at nt 165
alignment score=4 , category=4 , p=1
Cleavage Site 165
All Other Sites
Reads
0
2
4
6
8
0
100
200
300
400
500
gi393753853 position (nts)

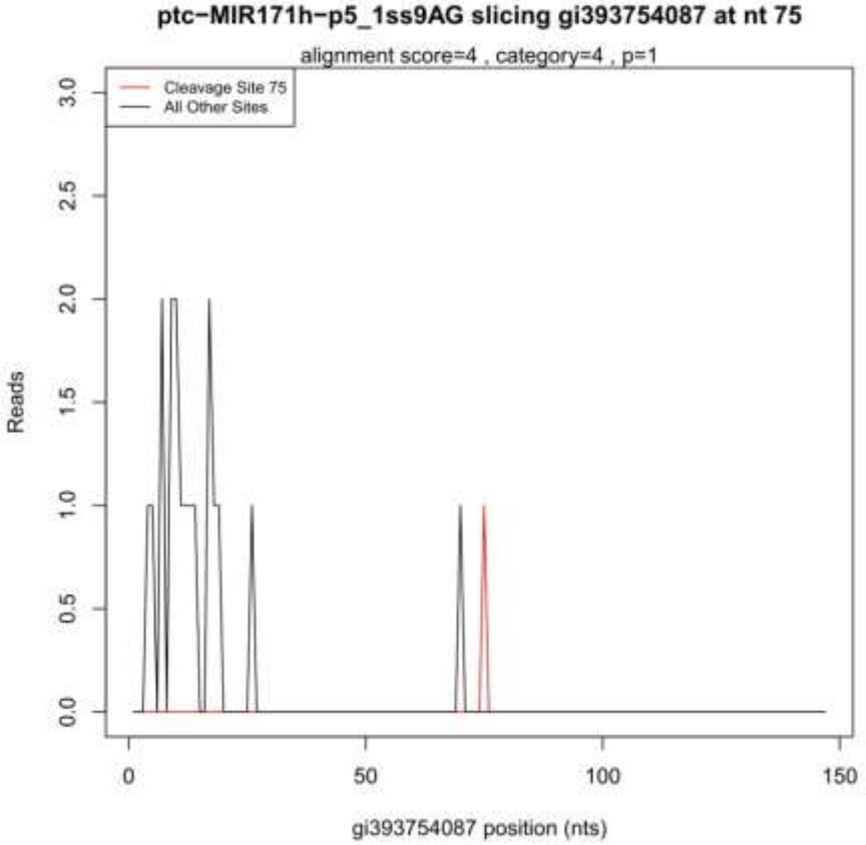
ptc−MIR171h−p5_1ss9AG slicing gi393754087 at nt 75
alignment score=4 , category=4 , p=1
Cleavage Site 75
All Other Sites
Reads
0.0
0.5
1.0
1.5
2.0
2.5
3.0
0
50
100
150
gi393754087 position (nts)

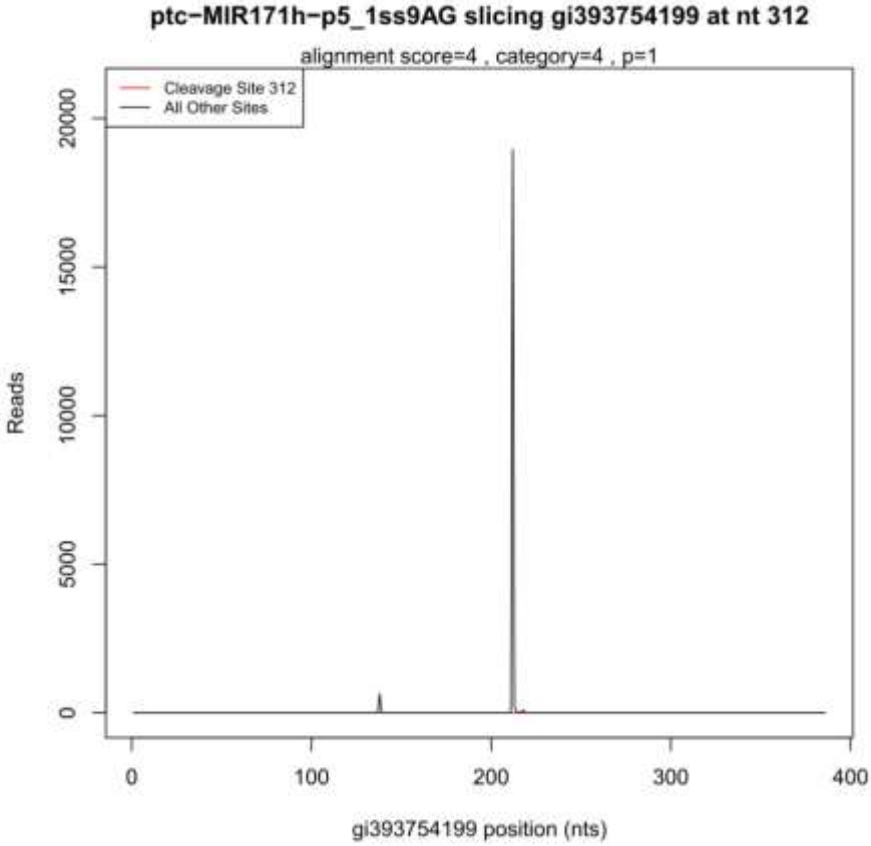
ptc−MIR171h−p5_1ss9AG slicing gi393754199 at nt 312
alignment score=4 , category=4 , p=1
Cleavage Site 312
All Other Sites
Reads
0
5000
10000
15000
20000
0
100
200
300
400
gi393754199 position (nts)

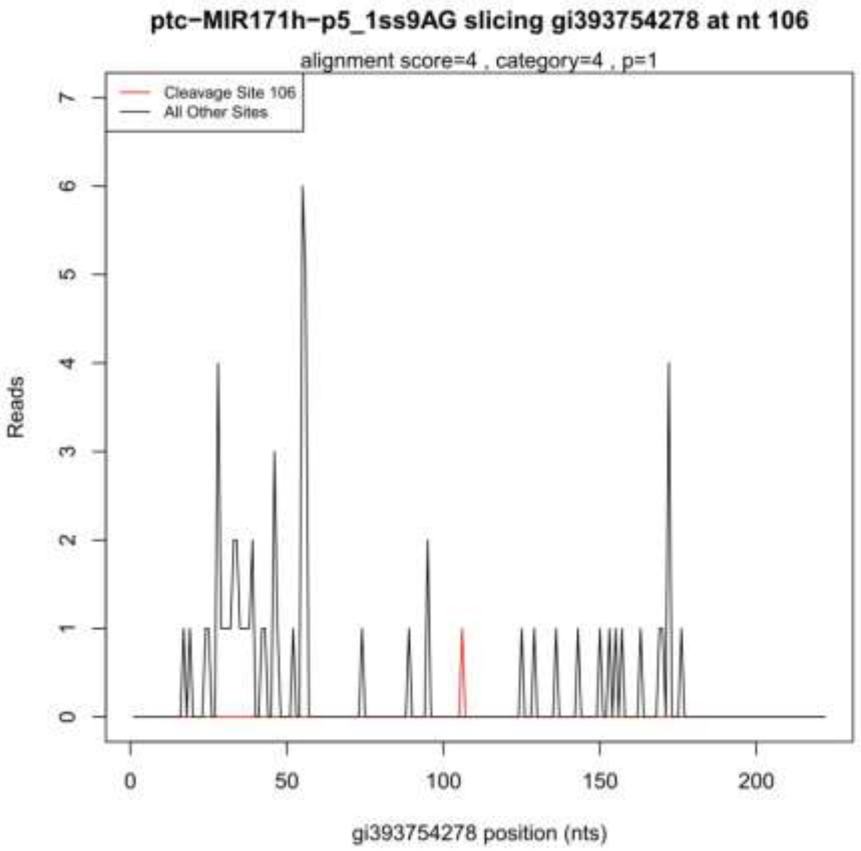
ptc−MIR171h−p5_1ss9AG slicing gi393754278 at nt 106
alignment score=4 , category=4 , p=1
Cleavage Site 106
All Other Sites
Reads
0
1
2
3
4
5
6
7
0
50
100
150
200
gi393754278 position (nts)

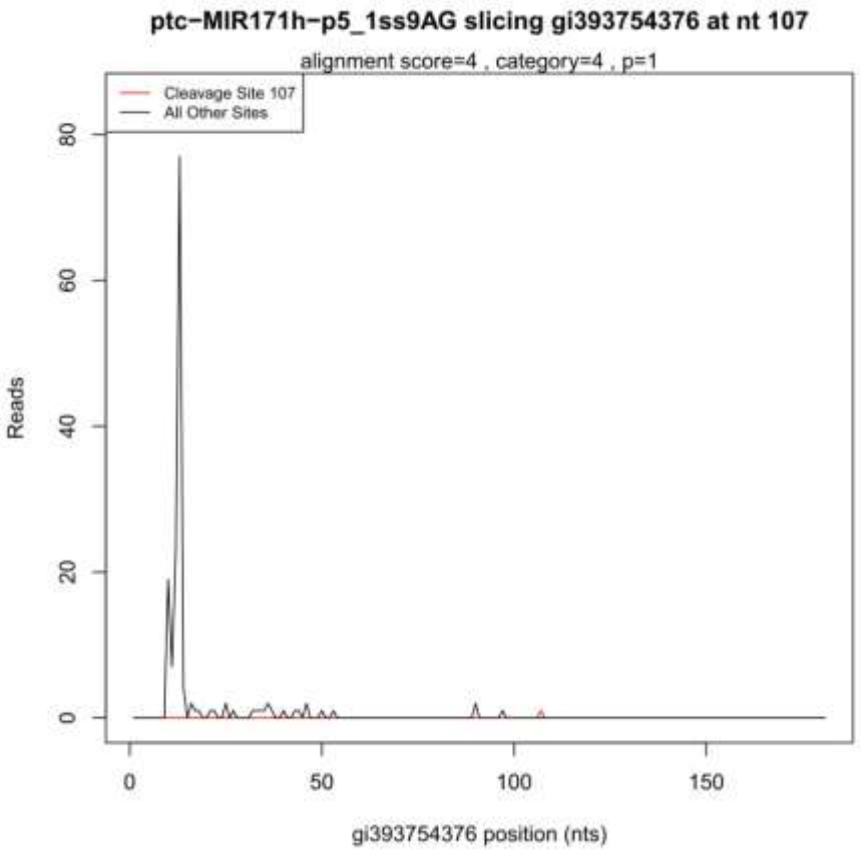
ptc−MIR171h−p5_1ss9AG slicing gi393754376 at nt 107
alignment score=4 , category=4 , p=1
Cleavage Site 107
All Other Sites
Reads
0
20
40
60
80
0
50
100
150
gi393754376 position (nts)

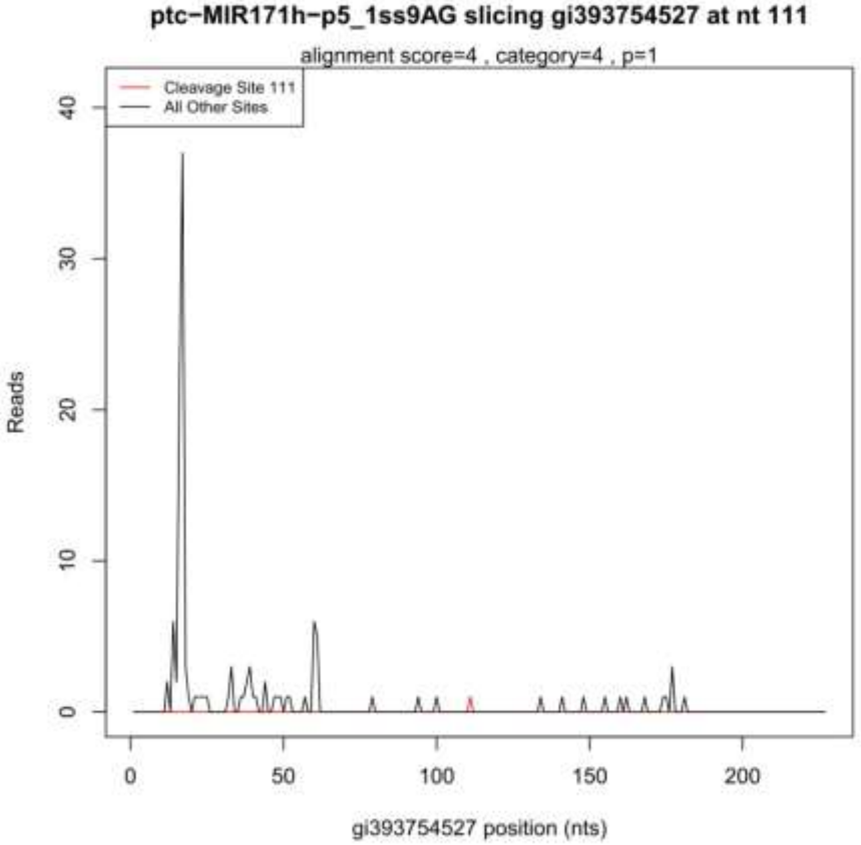
ptc−MIR171h−p5_1ss9AG slicing gi393754527 at nt 111
alignment score=4 , category=4 , p=1
Cleavage Site 111
All Other Sites
Reads
gi393754527 position (nts)

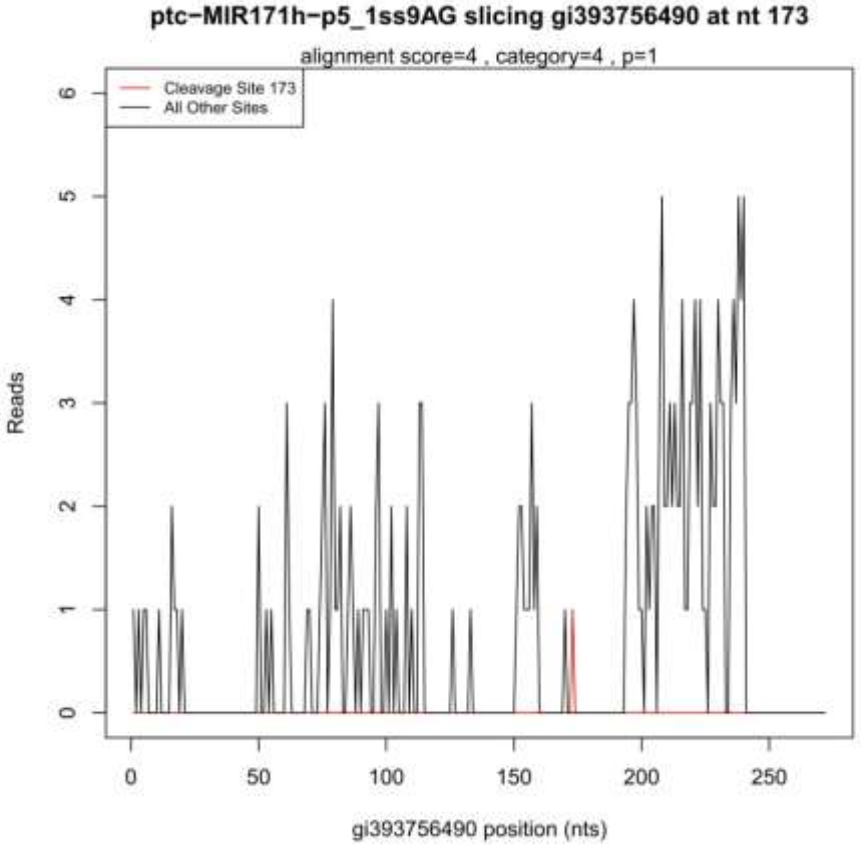
ptc−MIR171h−p5_1ss9AG slicing gi393756490 at nt 173
alignment score=4 , category=4 , p=1
Cleavage Site 173
All Other Sites
Reads
gi393756490 position (nts)

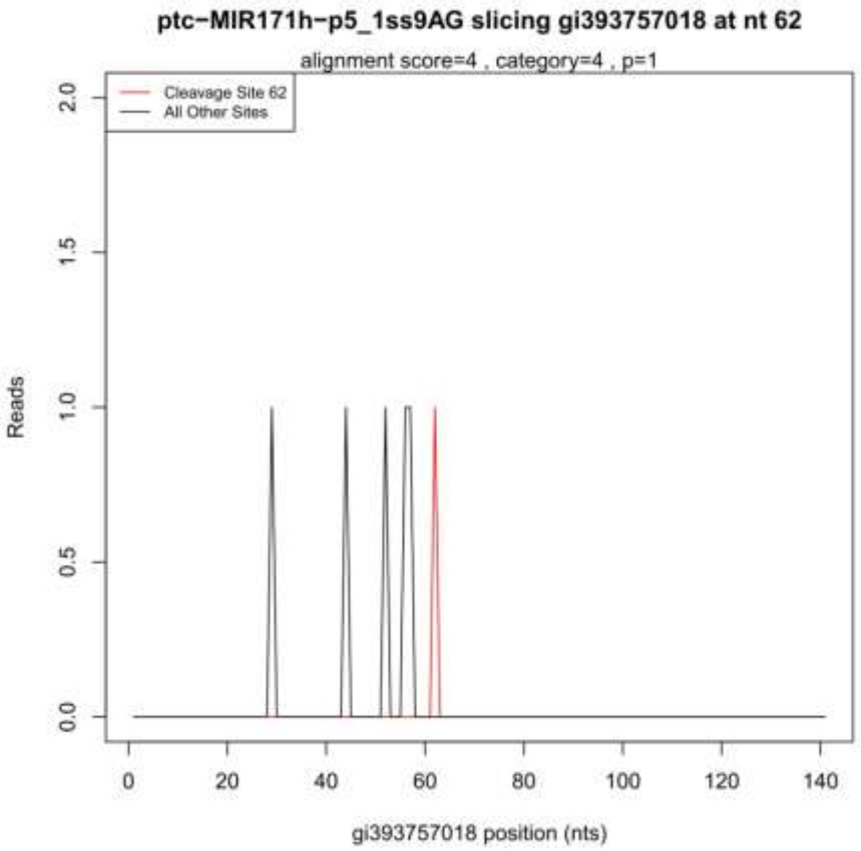

ptc−MIR171h−p5_1ss9AG slicing gi393757018 at nt 62
alignment score=4 , category=4 , p=1
Cleavage Site 62
All Other Sites
Reads
0.0
0.5
1.0
1.5
2.0
0
20
40
60
80
100
120
140
gi393757018 position (nts)

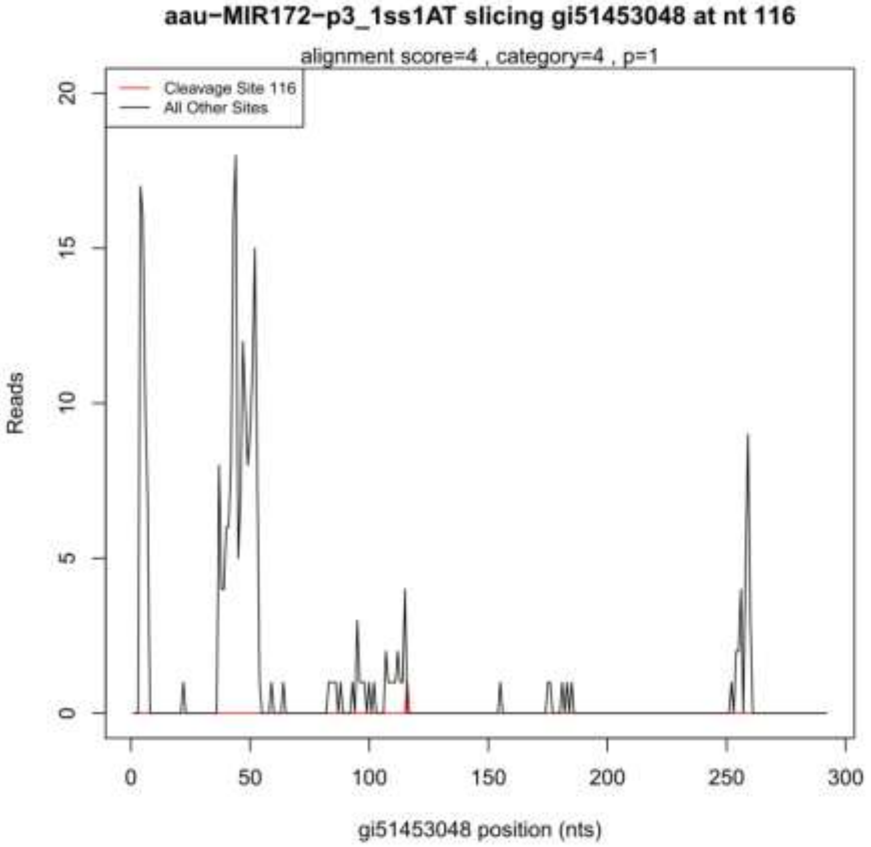

aau−MIR172−p3_1ss1AT slicing gi51453048 at nt 116
alignment score=4 , category=4 , p=1
Cleavage Site 116
All Other Sites
Reads
0
5
10
15
20
0
50
100
150
200
250
300
gi51453048 position (nts)

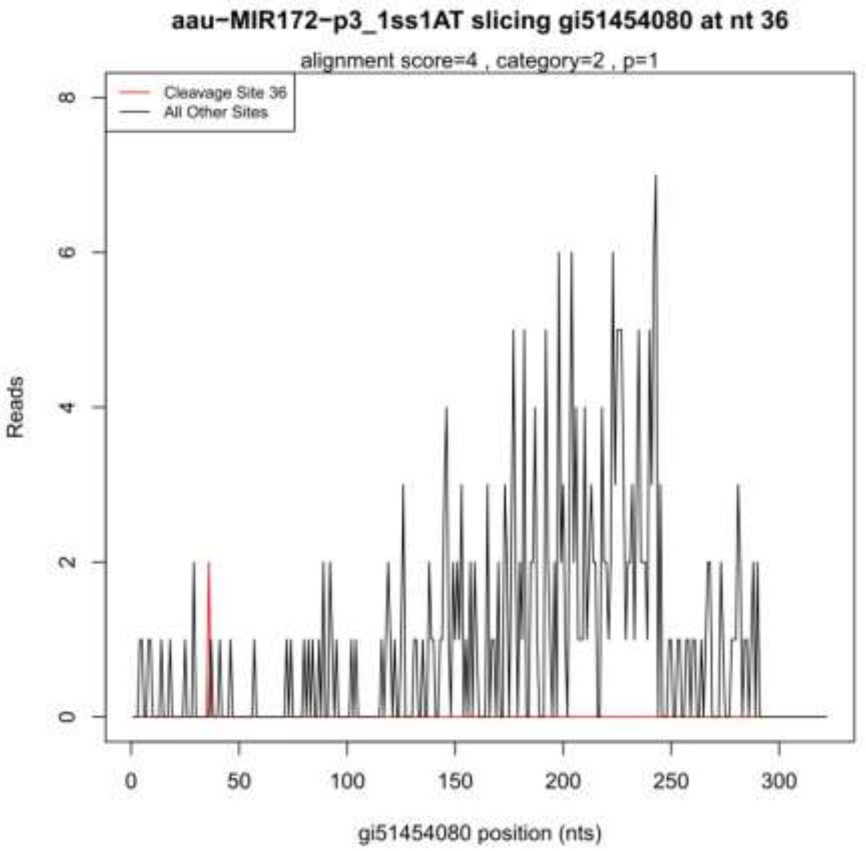
aau-MIR172-p3_1ss1AT slicing gi51454080 at nt 36
alignment score=4 , category=2 , p=1
Cleavage Site 36
All Other Sites
Reads
0
2
4
6
8
0
50
100
150
200
250
300
gi51454080 position (nts)

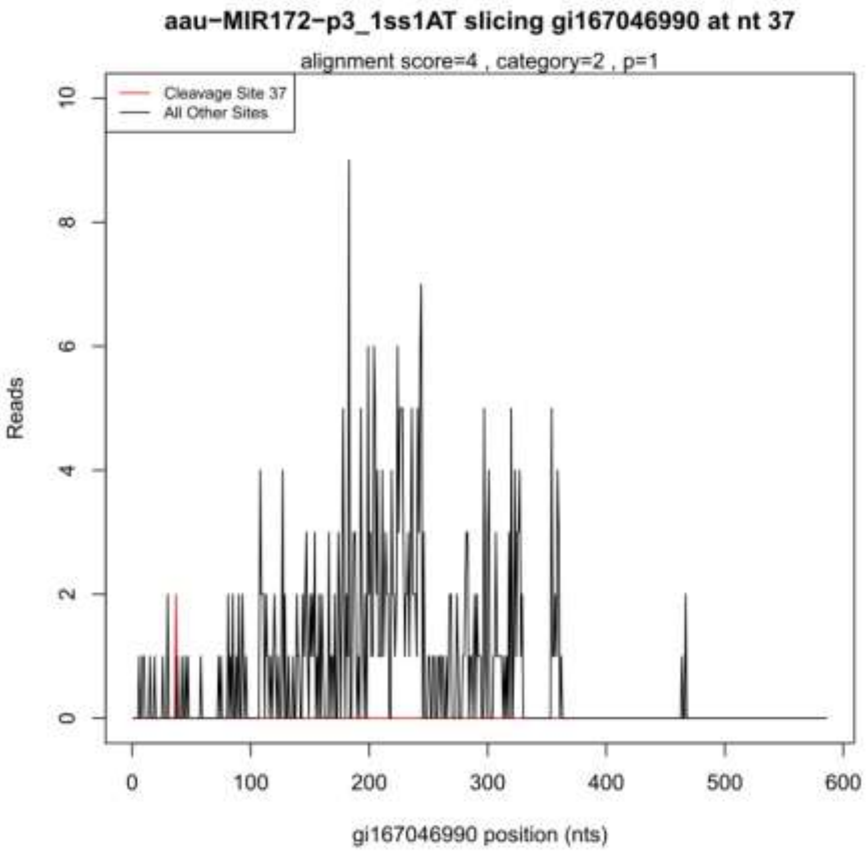
aau-MIR172-p3_1ss1AT slicing gi167046990 at nt 37
alignment score=4 , category=2 , p=1
Cleavage Site 37
All Other Sites
Reads
0
2
4
6
8
10
0
100
200
300
400
500
600
gi167046990 position (nts)

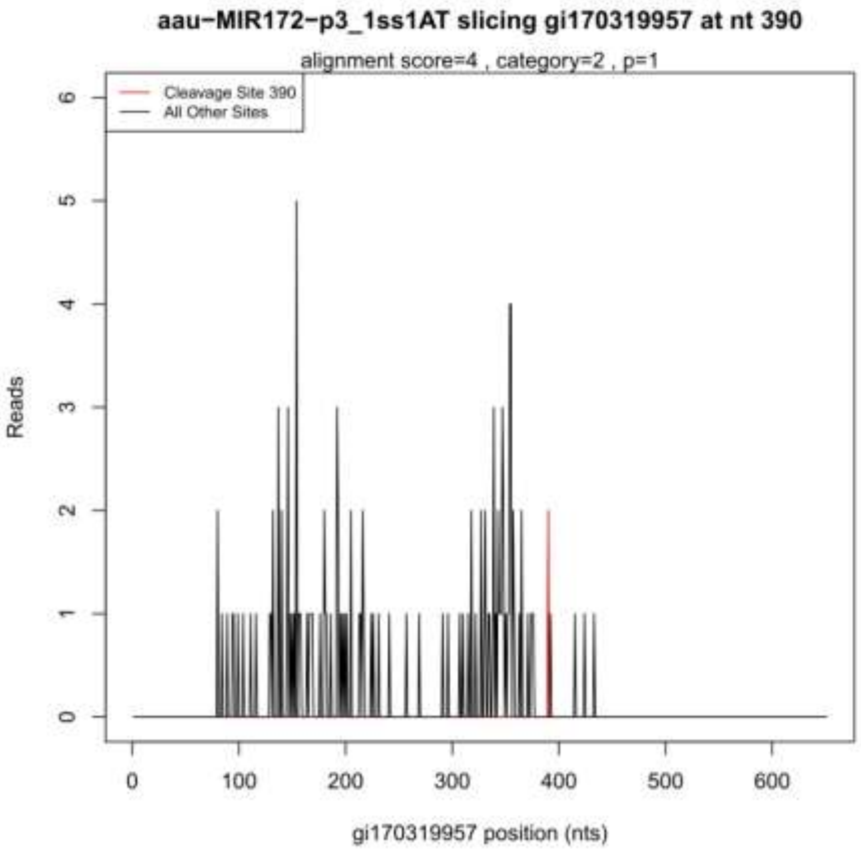
aau−MIR172−p3_1ss1AT slicing gi170319957 at nt 390
alignment score=4 , category=2 , p=1
Cleavage Site 390
All Other Sites
Reads
0
1
2
3
4
5
6
0
100
200
300
400
500
600
gi170319957 position (nts)

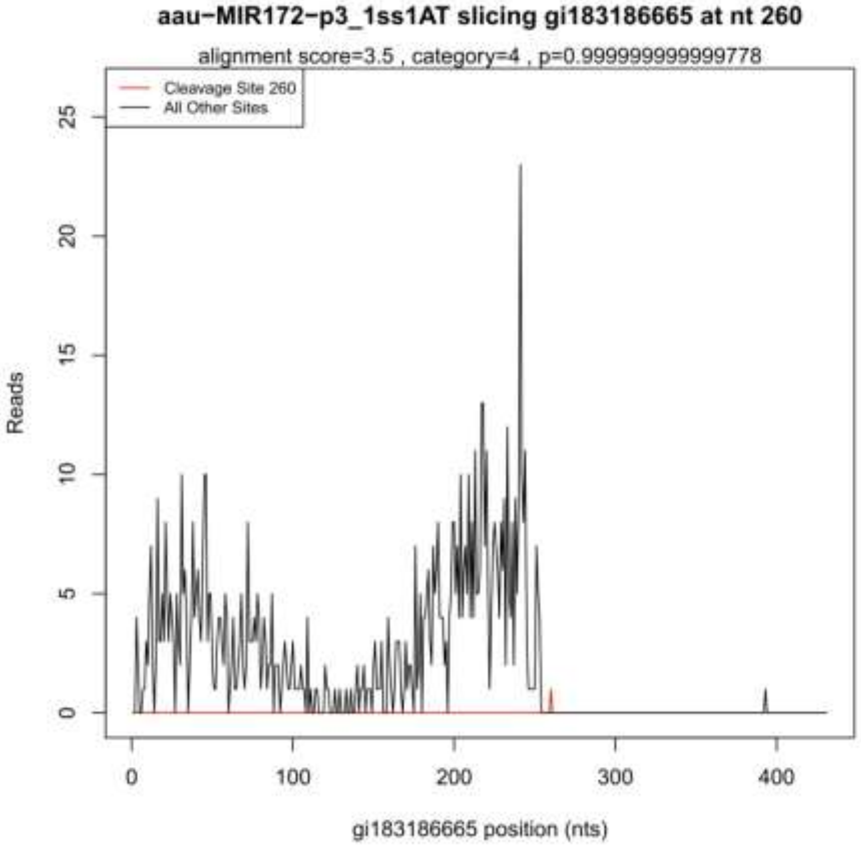
aau−MIR172−p3_1ss1AT slicing gi183186665 at nt 260
alignment score=3.5 , category=4 , p=0.999999999999778
Cleavage Site 260
All Other Sites
Reads
0
5
10
15
20
25
0
100
200
300
400
gi183186665 position (nts)

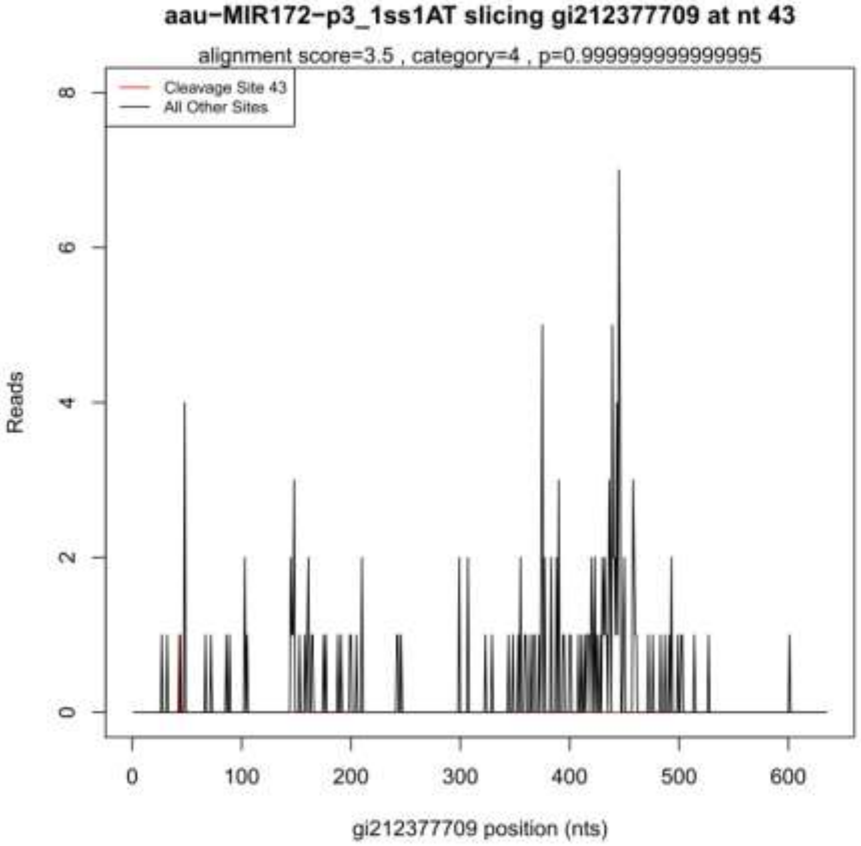
aau−MIR172−p3_1ss1AT slicing gi212377709 at nt 43
alignment score=3.5 , category=4 , p=0.999999999999995
Cleavage Site 43
All Other Sites
Reads
0
2
4
6
8
0
100
200
300
400
500
600
gi212377709 position (nts)

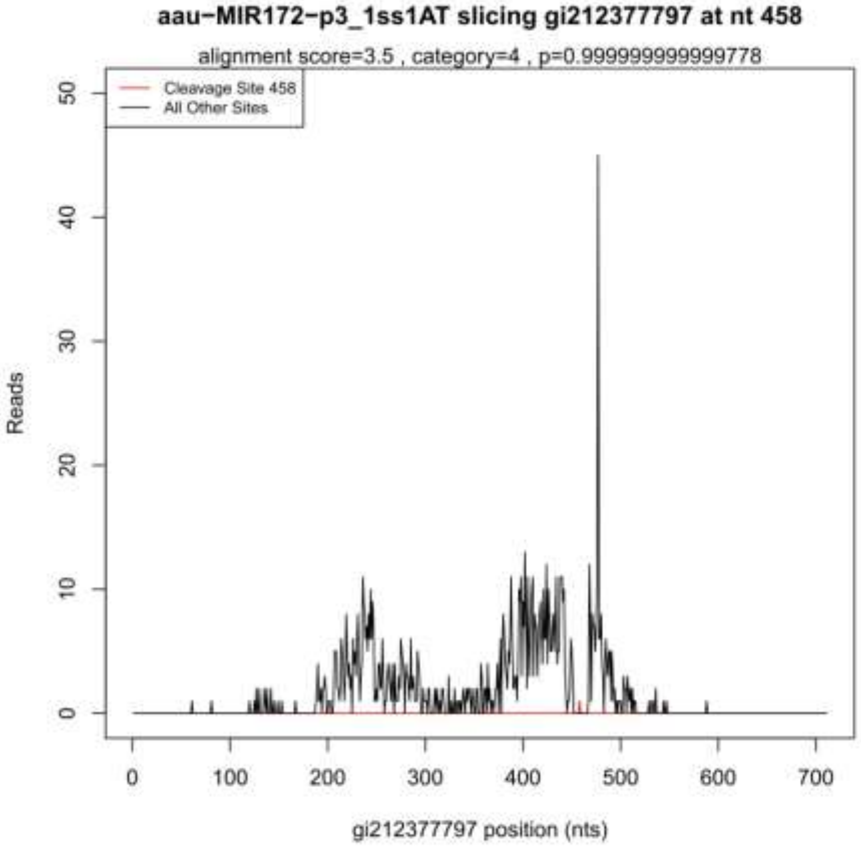
aau−MIR172−p3_1ss1AT slicing gi212377797 at nt 458
alignment score=3.5 , category=4 , p=0.999999999999778
Cleavage Site 458
All Other Sites
Reads
0
10
20
30
40
50
0
100
200
300
400
500
600
700
gi212377797 position (nts)

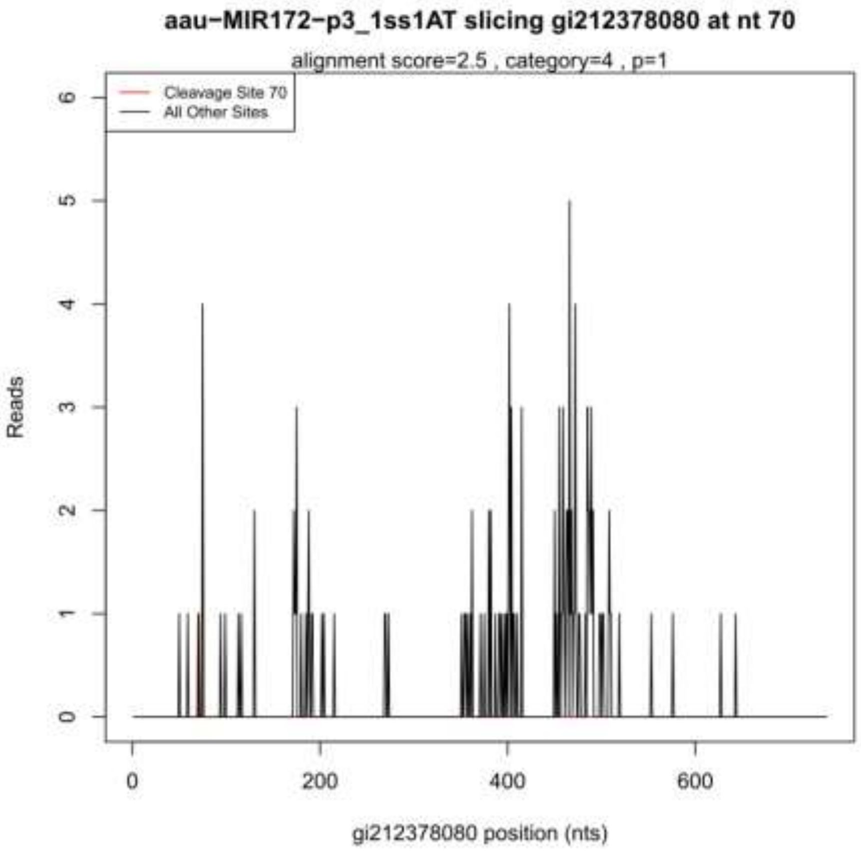
aau−MIR172−p3_1ss1AT slicing gi212378080 at nt 70
alignment score=2.5 , category=4 , p=1
Cleavage Site 70
All Other Sites
Reads
0
1
2
3
4
5
6
0
200
400
600
gi212378080 position (nts)

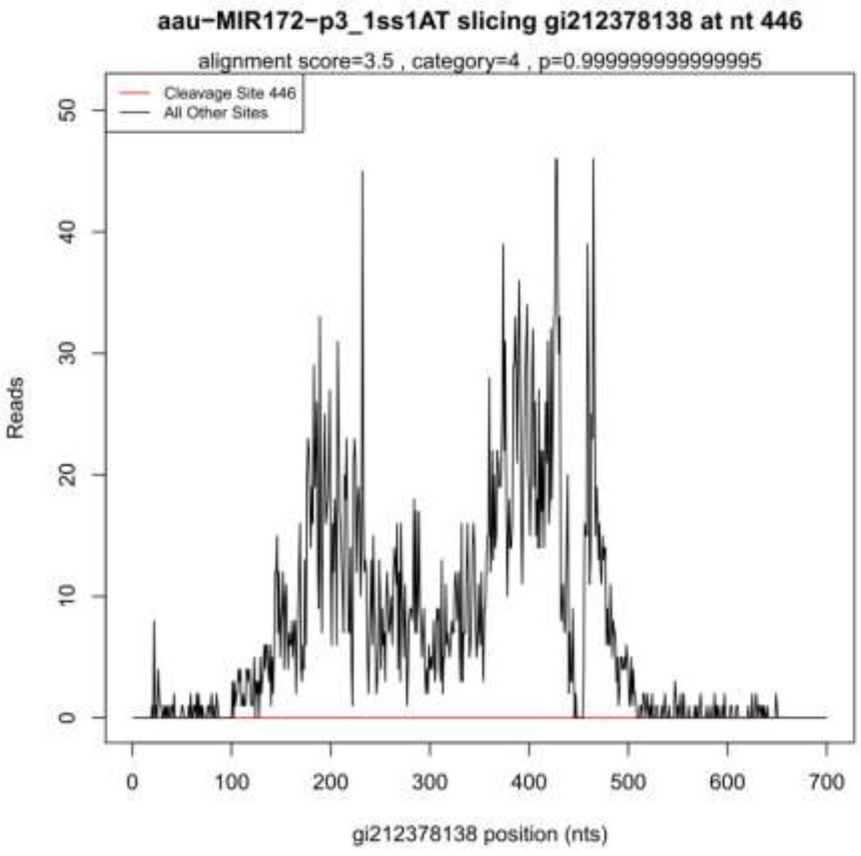
aau−MIR172−p3_1ss1AT slicing gi212378138 at nt 446
alignment score=3.5 , category=4 , p=0.999999999999995
Cleavage Site 446
All Other Sites
Reads
0
10
20
30
40
50
0
100
200
300
400
500
600
700
gi212378138 position (nts)

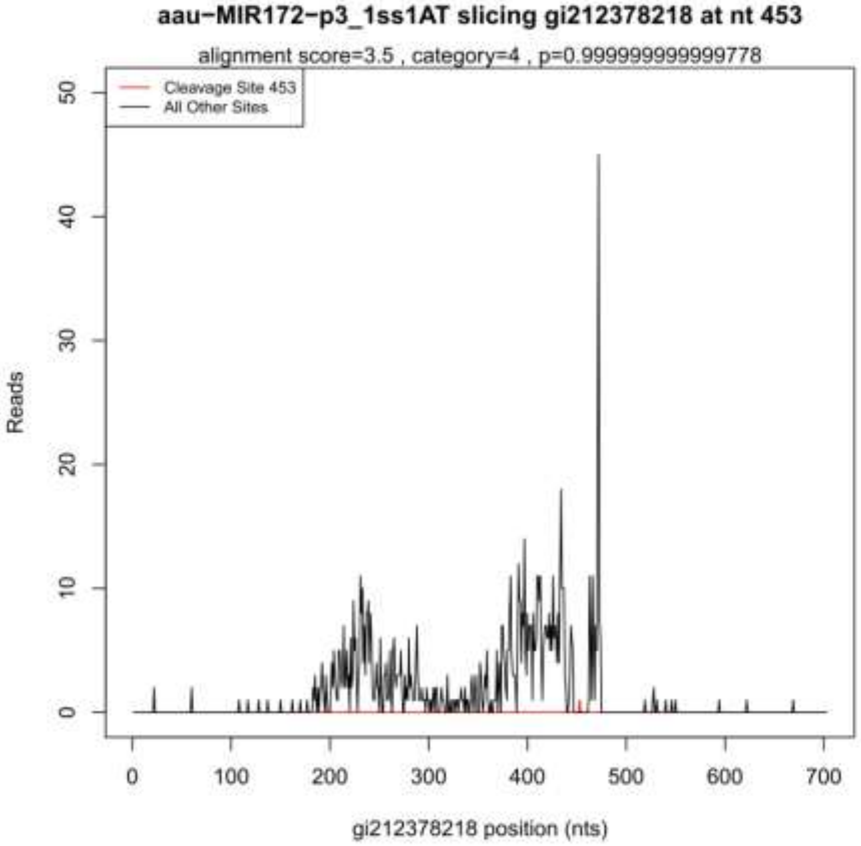
aau−MIR172−p3_1ss1AT slicing gi212378218 at nt 453
alignment score=3.5 , category=4 , p=0.999999999999778
Cleavage Site 453
All Other Sites
Reads
gi212378218 position (nts)

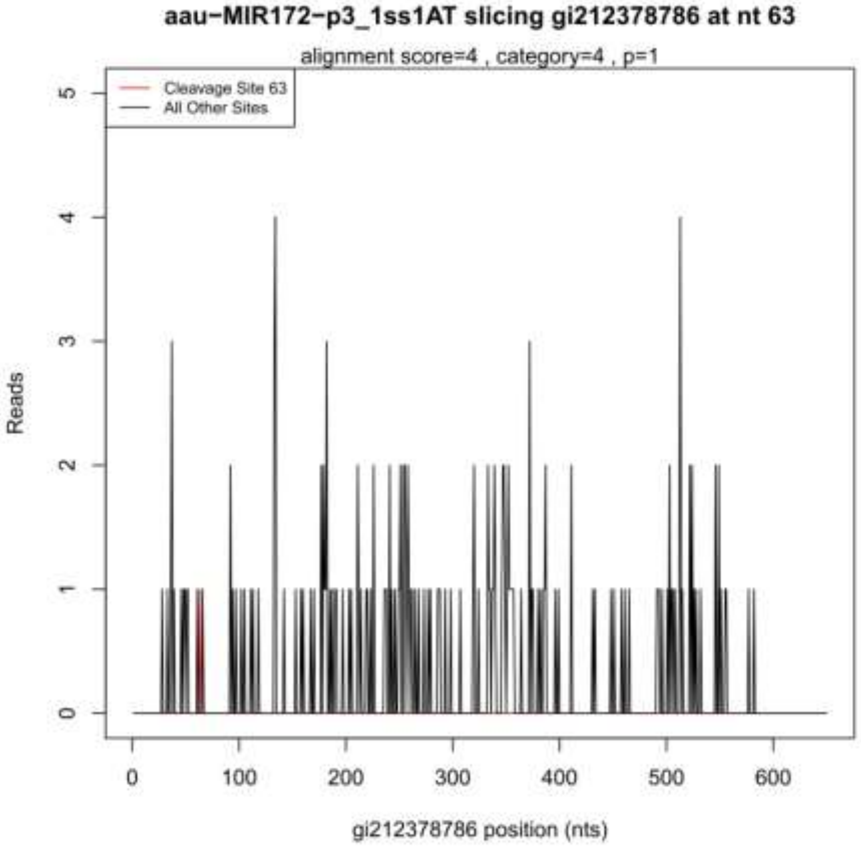
aau−MIR172−p3_1ss1AT slicing gi212378786 at nt 63
alignment score=4 , category=4 , p=1
Cleavage Site 63
All Other Sites
Reads
gi212378786 position (nts)

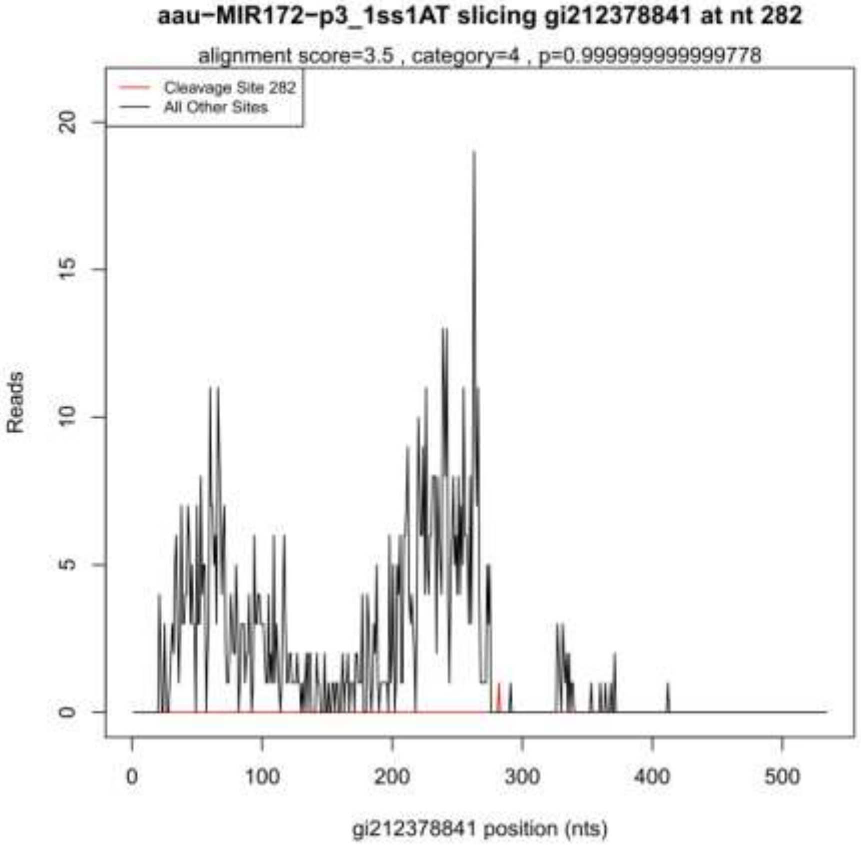
aau−MIR172−p3_1ss1AT slicing gi212378841 at nt 282
alignment score=3.5 , category=4 , p=0.999999999999778
Cleavage Site 282
All Other Sites
Reads
gi212378841 position (nts)

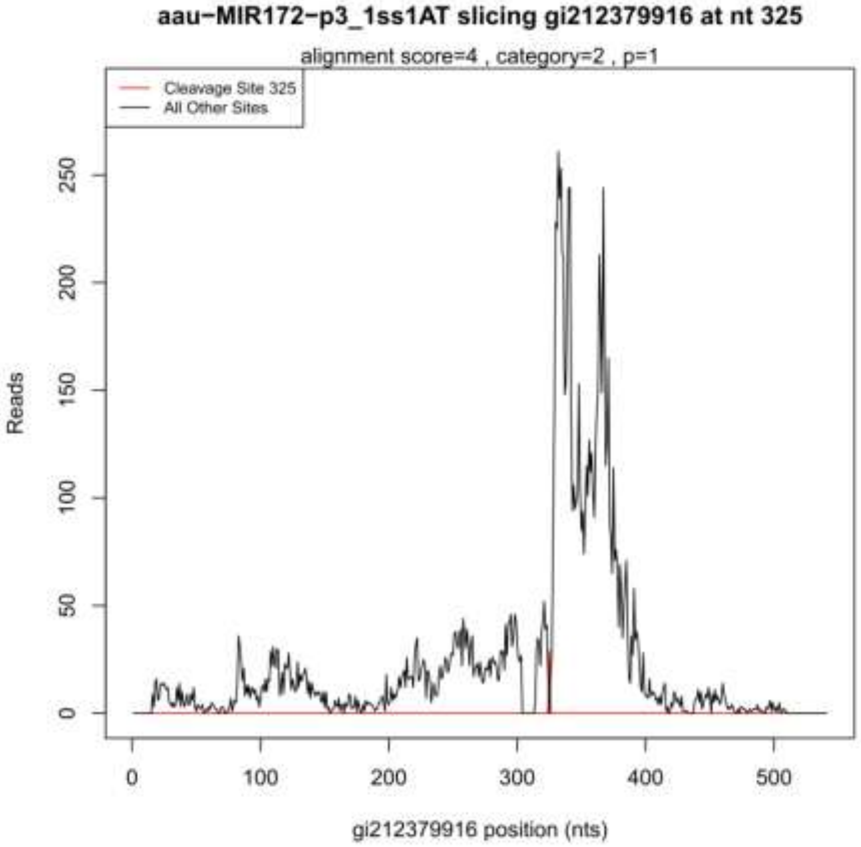
aau−MIR172−p3_1ss1AT slicing gi212379916 at nt 325
alignment score=4 , category=2 , p=1
Cleavage Site 325
All Other Sites
Reads
gi212379916 position (nts)

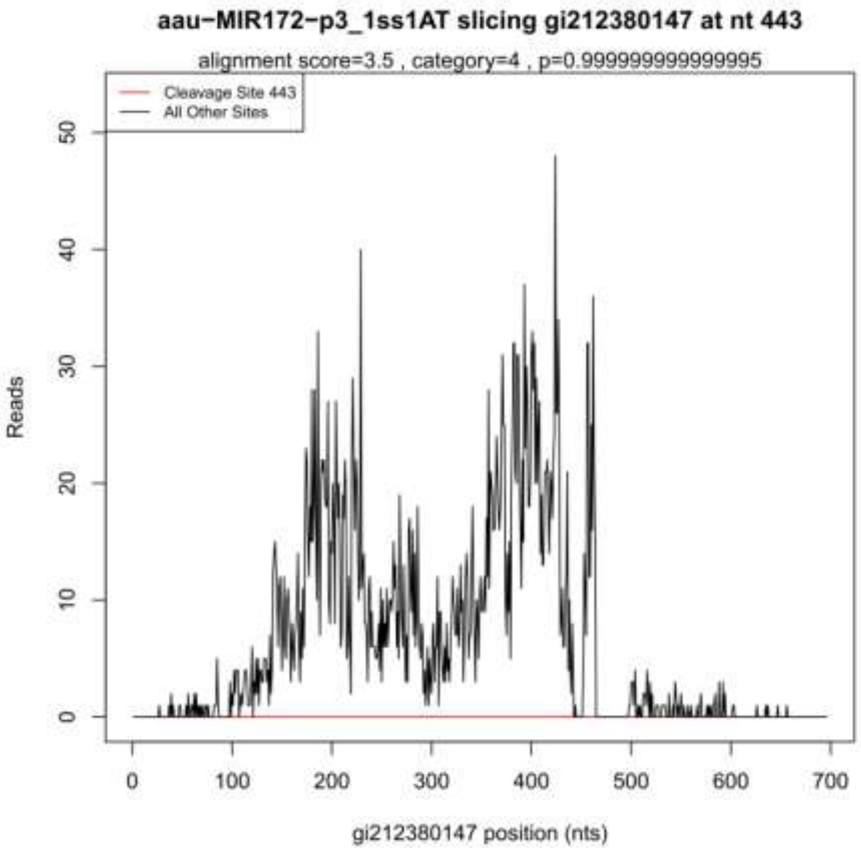
aau−MIR172−p3_1ss1AT slicing gi212380147 at nt 443
alignment score=3.5 , category=4 , p=0.999999999999995
Cleavage Site 443
All Other Sites
Reads
0
10
20
30
40
50
0
100
200
300
400
500
600
700
gi212380147 position (nts)

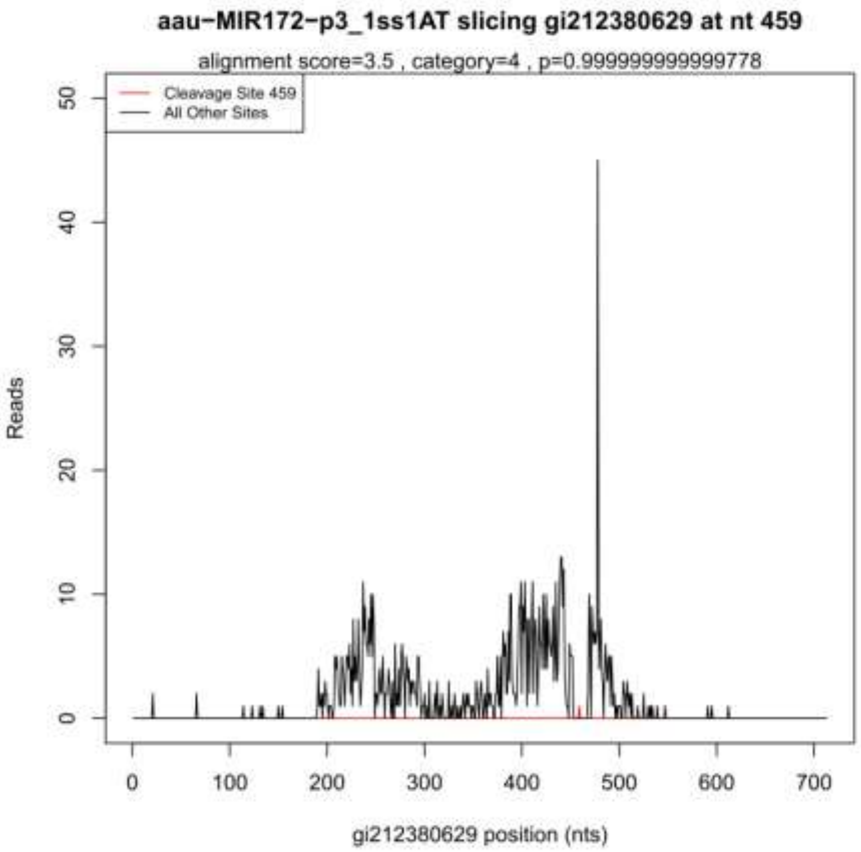
aau−MIR172−p3_1ss1AT slicing gi212380629 at nt 459
alignment score=3.5 , category=4 , p=0.999999999999778
Cleavage Site 459
All Other Sites
Reads
0
10
20
30
40
50
0
100
200
300
400
500
600
700
gi212380629 position (nts)

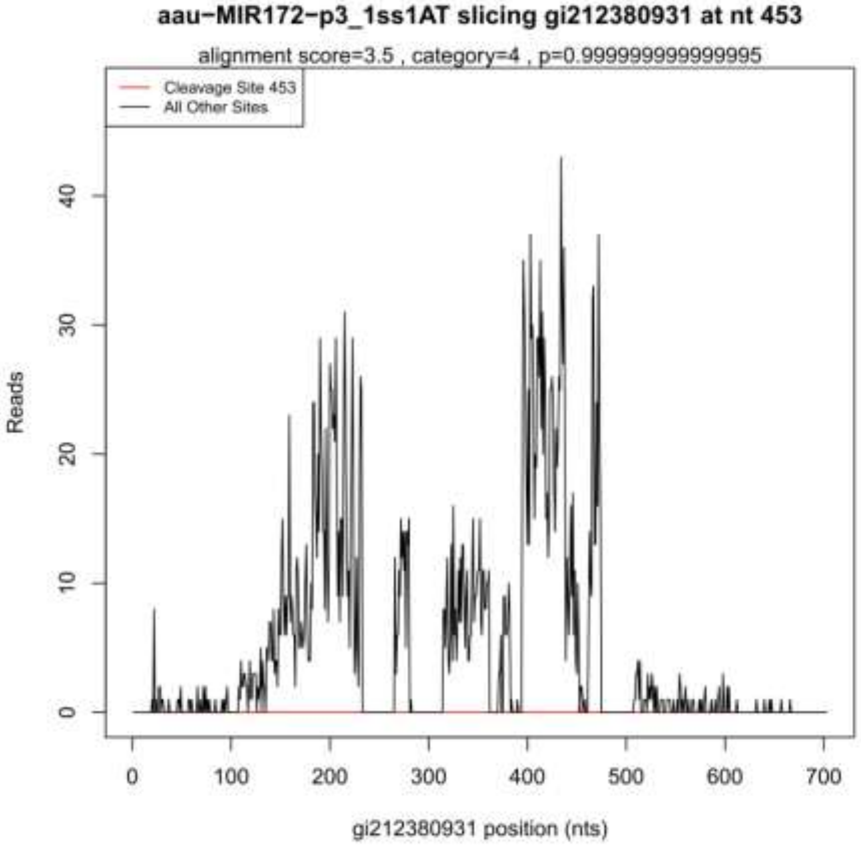

aau−MIR172−p3_1ss1AT slicing gi212380931 at nt 453
alignment score=3.5 , category=4 , p=0.999999999999995
Cleavage Site 453
All Other Sites
Reads
0
10
20
30
40
0
100
200
300
400
500
600
700
gi212380931 position (nts)

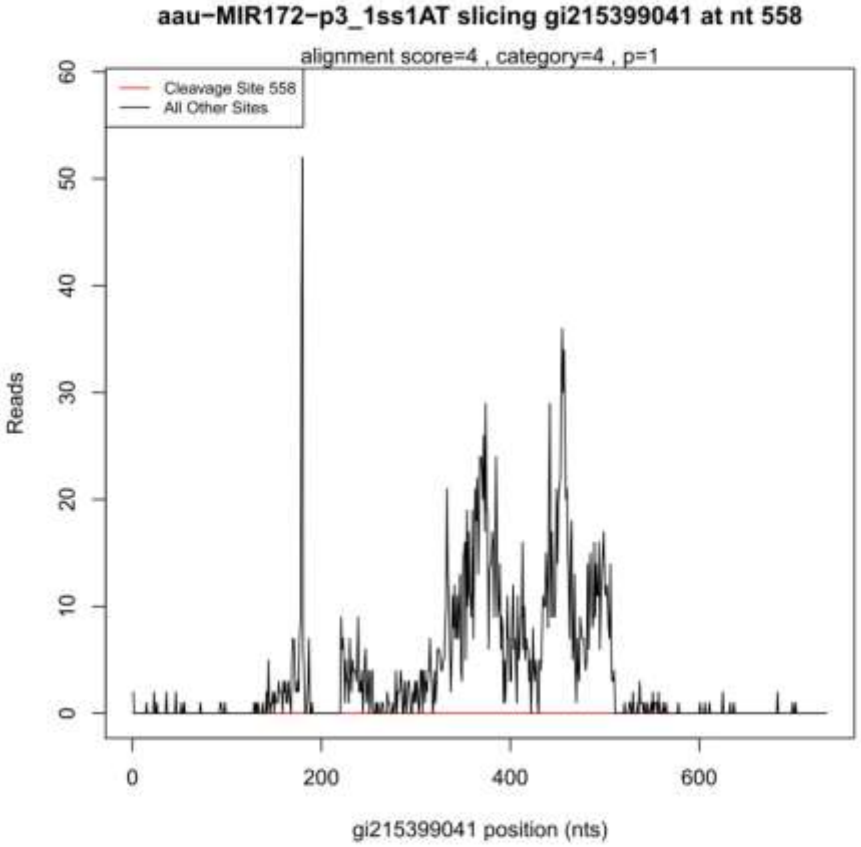

aau−MIR172−p3_1ss1AT slicing gi215399041 at nt 558
alignment score=4 , category=4 , p=1
Cleavage Site 558
All Other Sites
Reads
0
10
20
30
40
50
60
0
200
400
600
gi215399041 position (nts)

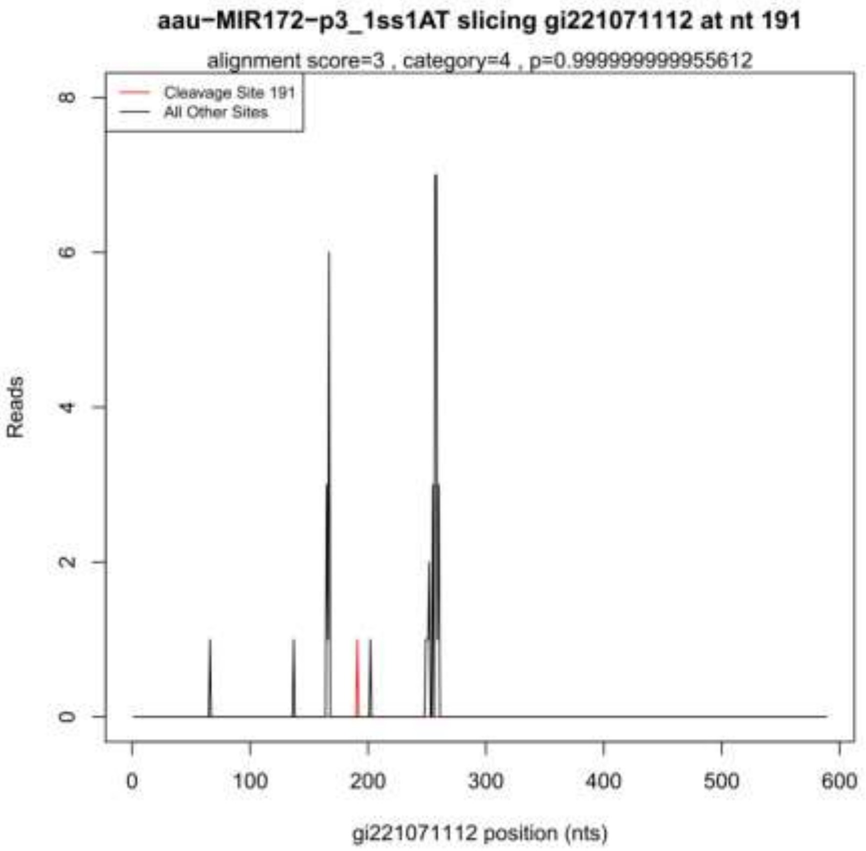
aau−MIR172−p3_1ss1AT slicing gi221071112 at nt 191
alignment score=3 , category=4 , p=0.999999999955612
Cleavage Site 191
All Other Sites
Reads
0
2
4
6
8
0
100
200
300
400
500
600
gi221071112 position (nts)

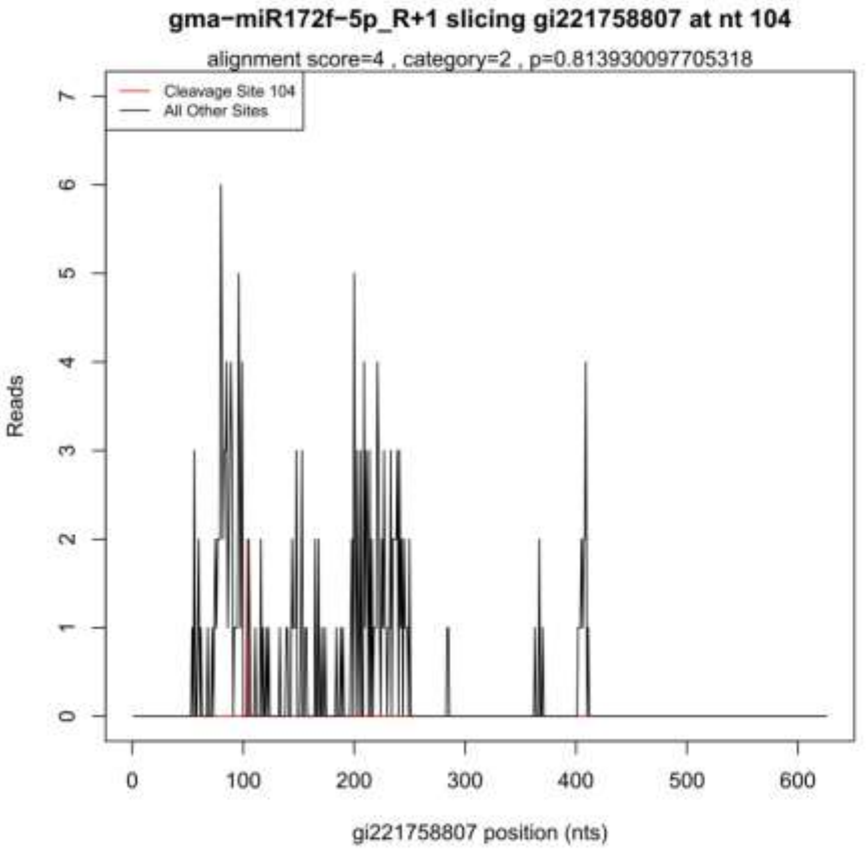
gma−miR172f−5p_R+1 slicing gi221758807 at nt 104
alignment score=4 , category=2 , p=0.813930097705318
Cleavage Site 104
All Other Sites
Reads
0
1
2
3
4
5
6
7
0
100
200
300
400
500
600
gi221758807 position (nts)

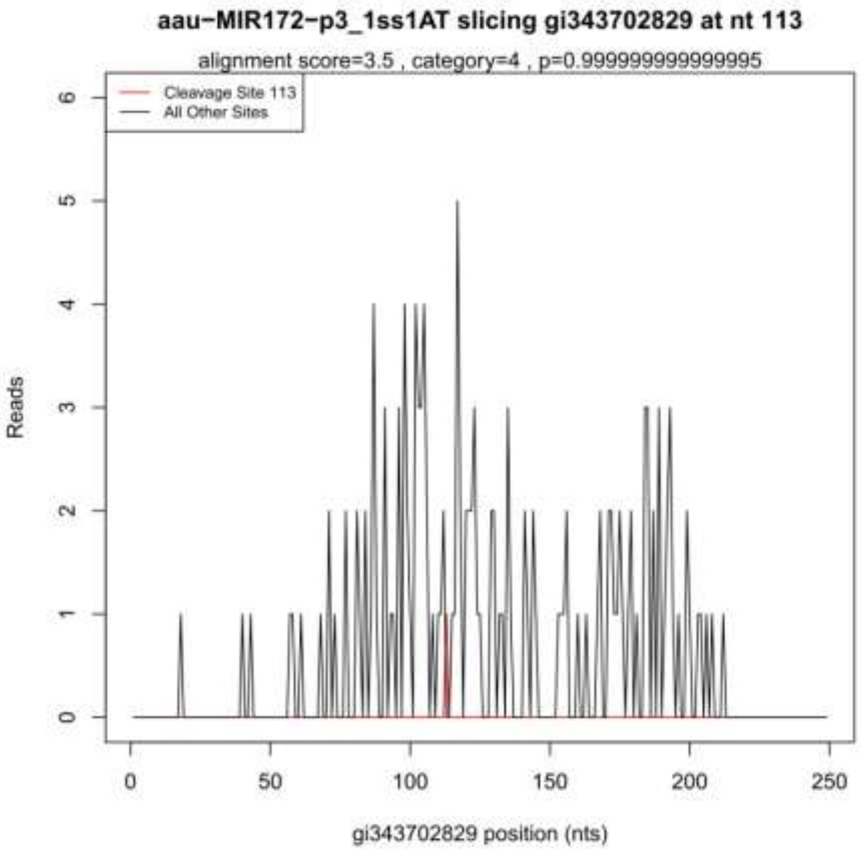
aau−MIR172−p3_1ss1AT slicing gi343702829 at nt 113
alignment score=3.5 , category=4 , p=0.999999999999995
Cleavage Site 113
All Other Sites
Reads
0
1
2
3
4
5
6
0
50
100
150
200
250
gi343702829 position (nts)

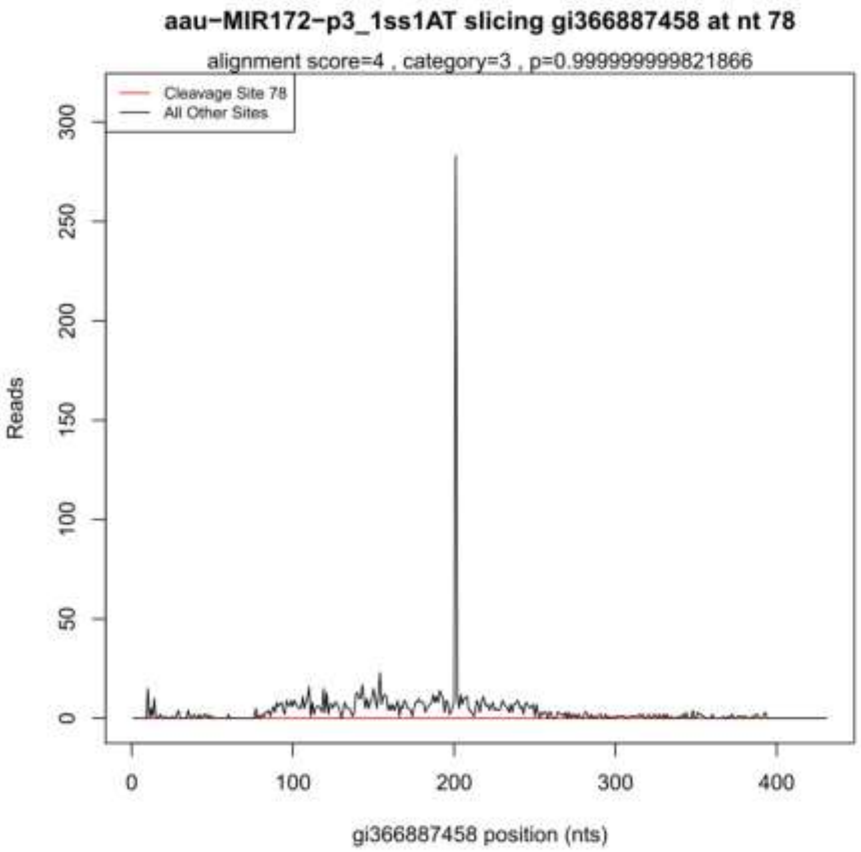
aau−MIR172−p3_1ss1AT slicing gi366887458 at nt 78
alignment score=4 , category=3 , p=0.999999999821866
Cleavage Site 78
All Other Sites
Reads
0
50
100
150
200
250
300
0
100
200
300
400
gi366887458 position (nts)

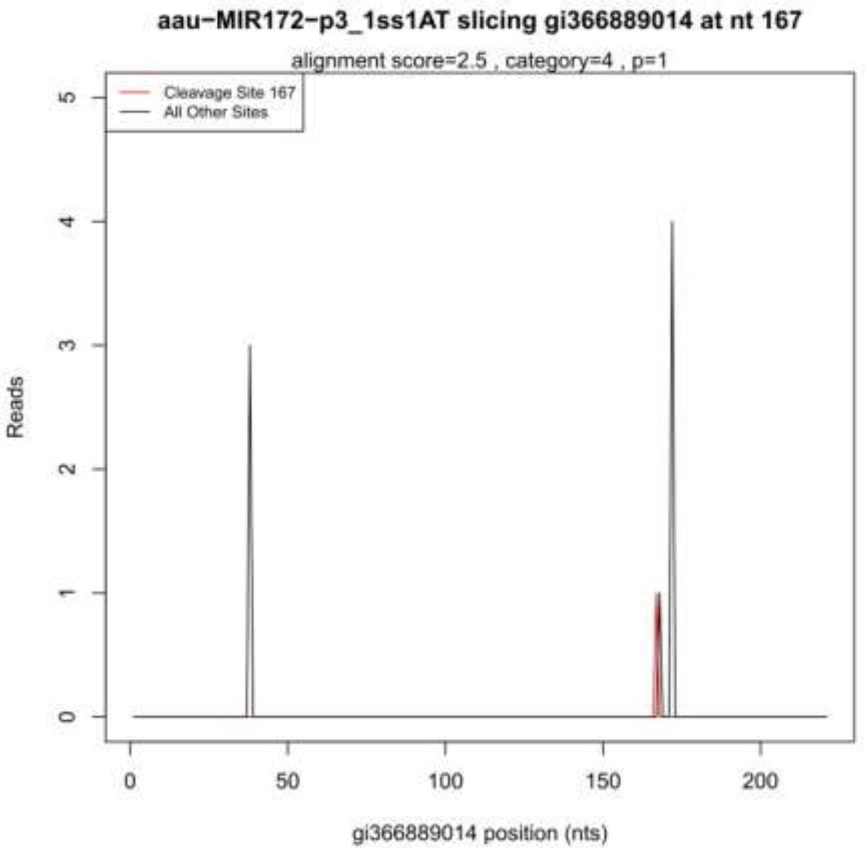

aau−MIR172−p3_1ss1AT slicing gi366889014 at nt 167
alignment score=2.5 , category=4 , p=1
Cleavage Site 167
All Other Sites
Reads
gi366889014 position (nts)

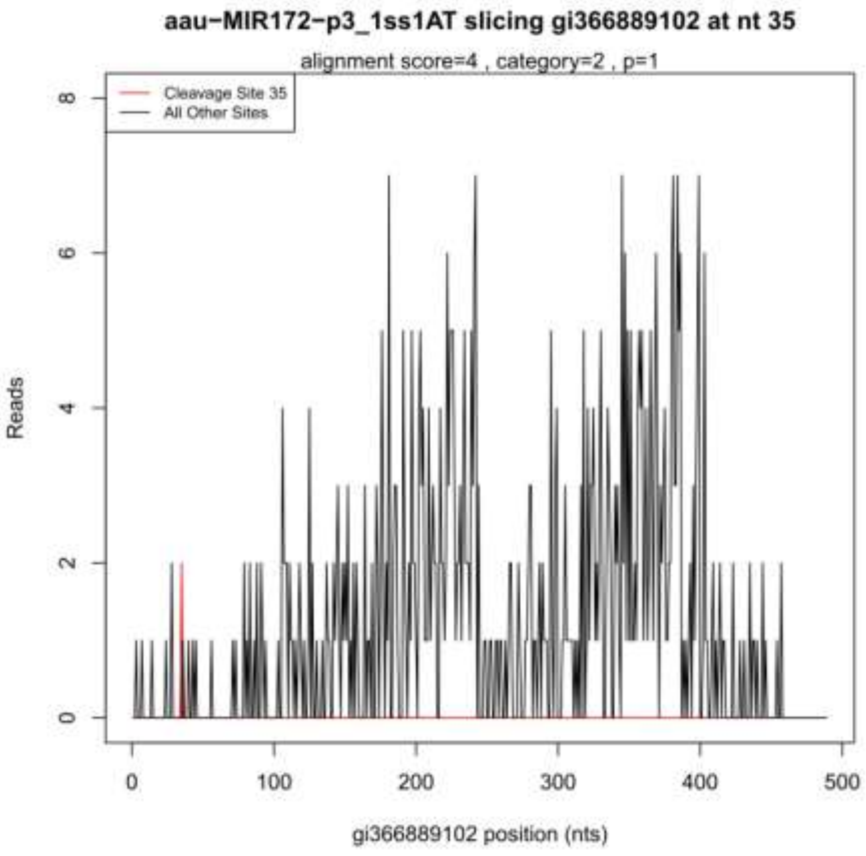

aau−MIR172−p3_1ss1AT slicing gi366889102 at nt 35
alignment score=4 , category=2 , p=1
Cleavage Site 35
All Other Sites
Reads
gi366889102 position (nts)

aau−MIR172−p3_1ss1AT slicing gi366889444 at nt 48

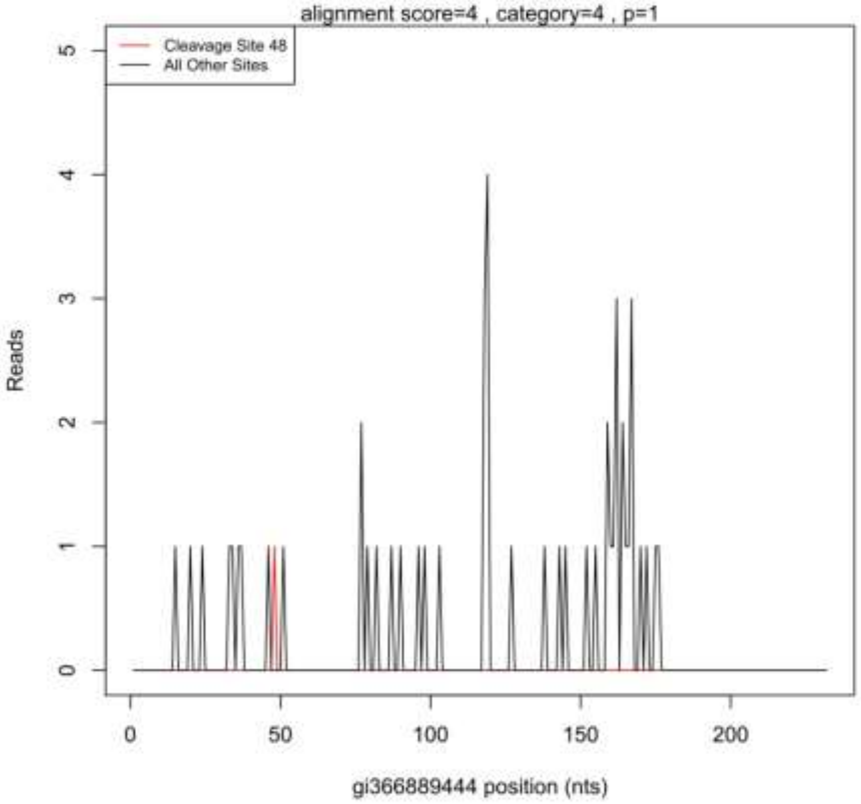

aau−MIR172−p3_1ss1AT slicing gi366889587 at nt 381

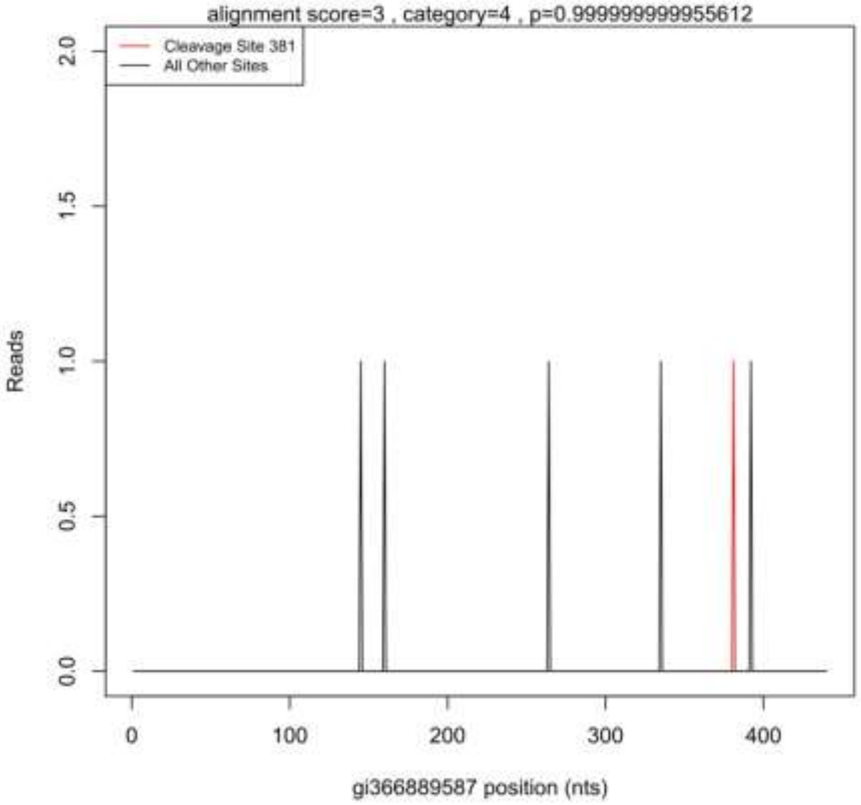

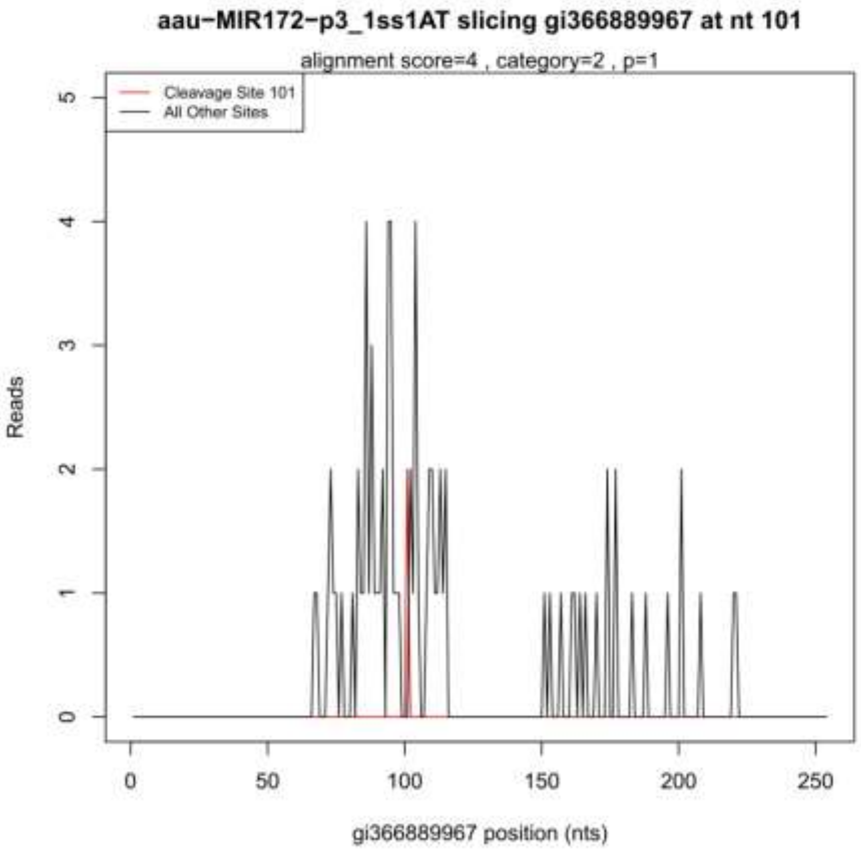

aau−MIR172−p3_1ss1AT slicing gi366889967 at nt 101
alignment score=4 , category=2 , p=1
Cleavage Site 101
All Other Sites
Reads
0
1
2
3
4
5
0
50
100
150
200
250
gi366889967 position (nts)

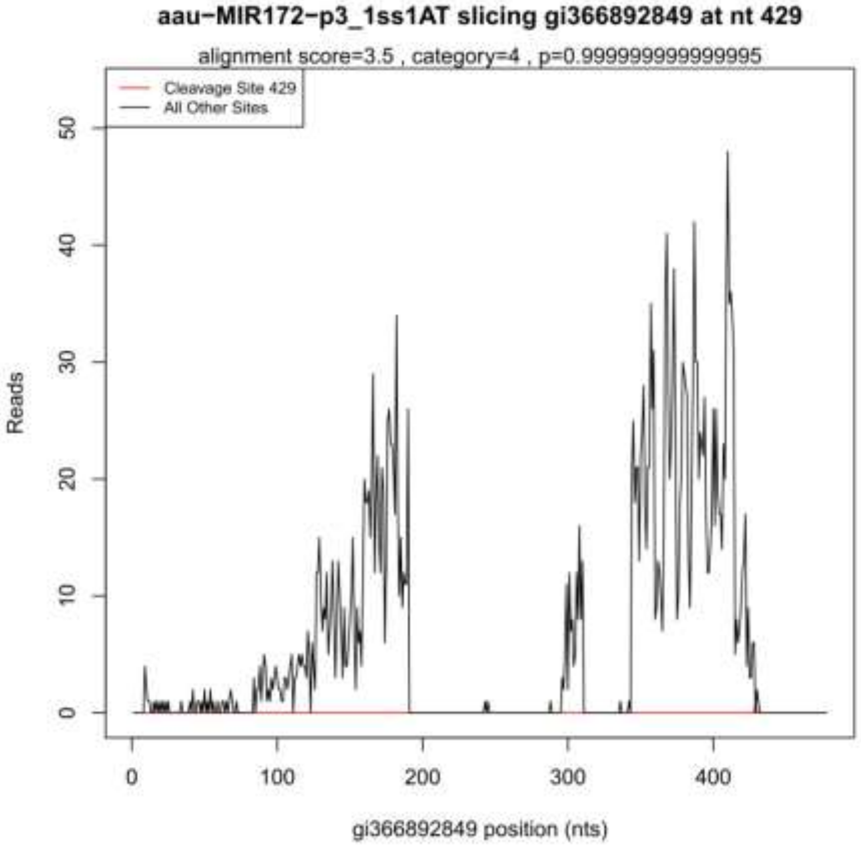

aau−MIR172−p3_1ss1AT slicing gi366892849 at nt 429
alignment score=3.5 , category=4 , p=0.999999999999995
Cleavage Site 429
All Other Sites
Reads
0
10
20
30
40
50
0
100
200
300
400
gi366892849 position (nts)

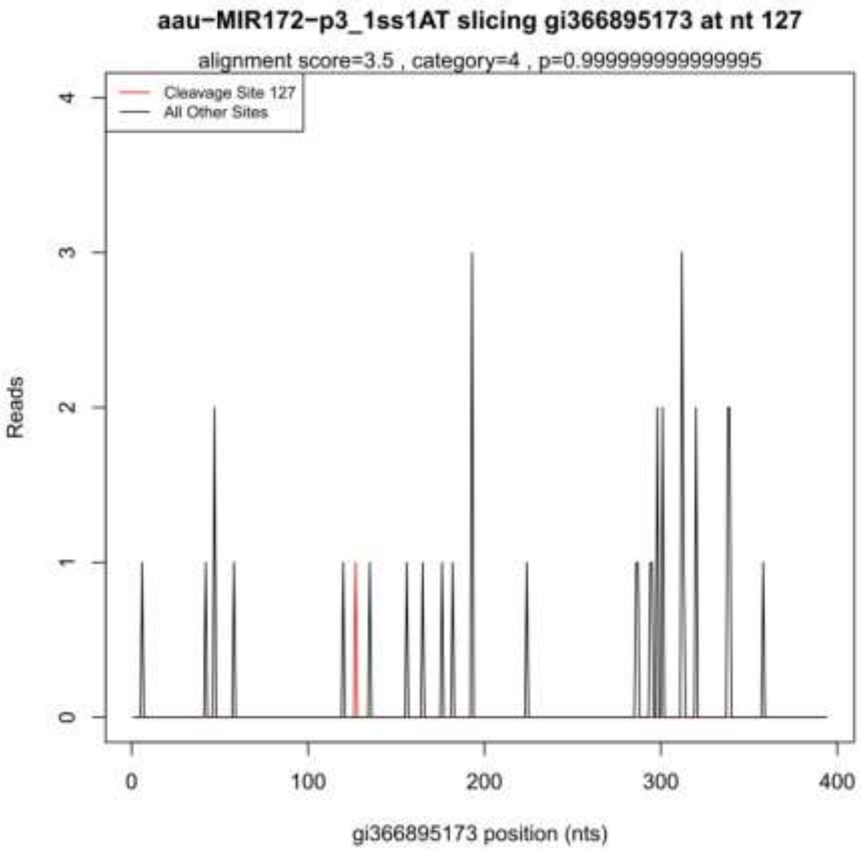

aau−MIR172−p3_1ss1AT slicing gi366895173 at nt 127
alignment score=3.5 , category=4 , p=0.999999999999995
Cleavage Site 127
All Other Sites
Reads
0
1
2
3
4
0
100
200
300
400
gi366895173 position (nts)

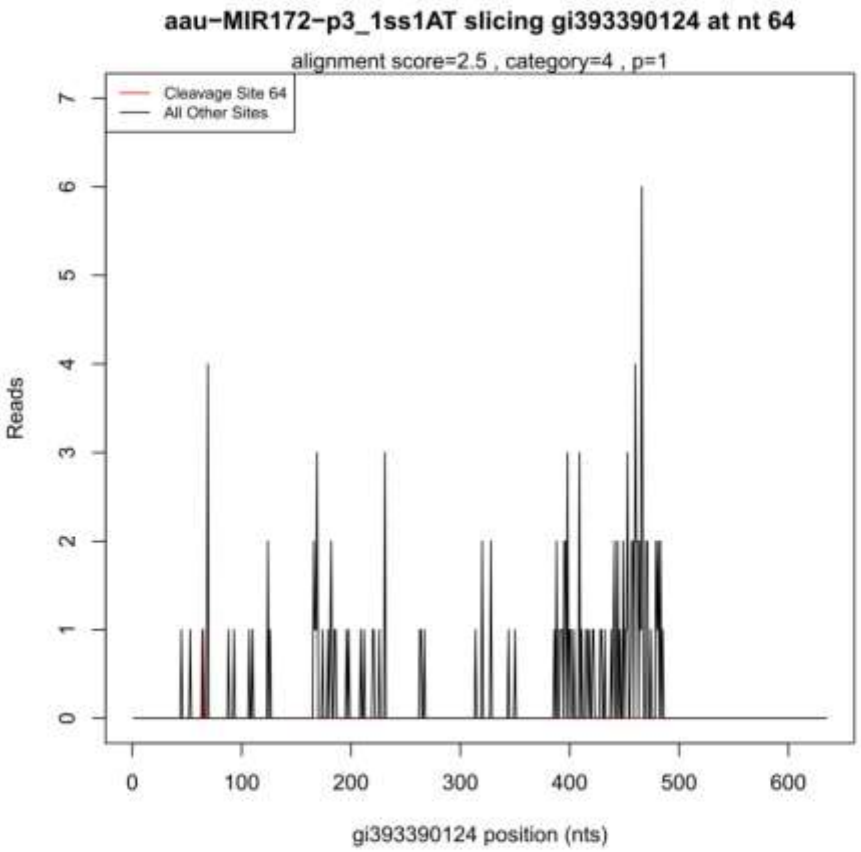

aau−MIR172−p3_1ss1AT slicing gi393390124 at nt 64
alignment score=2.5 , category=4 , p=1
Cleavage Site 64
All Other Sites
Reads
0
1
2
3
4
5
6
7
0
100
200
300
400
500
600
gi393390124 position (nts)

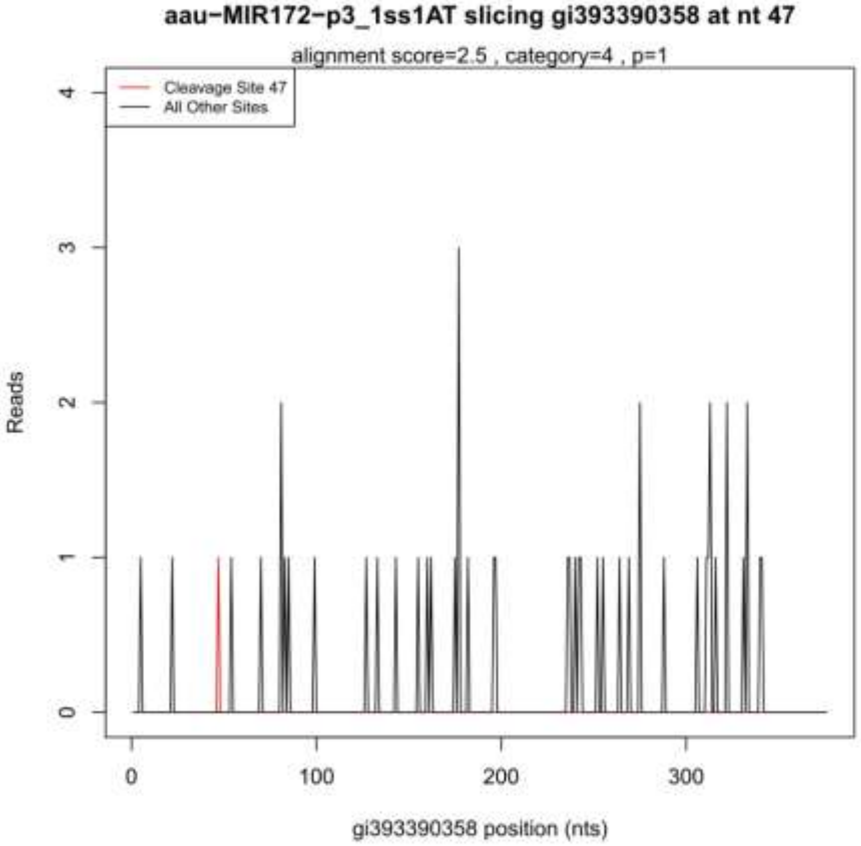
aau−MIR172−p3_1ss1AT slicing gi393390358 at nt 47
alignment score=2.5 , category=4 , p=1
Cleavage Site 47
All Other Sites
Reads
gi393390358 position (nts)

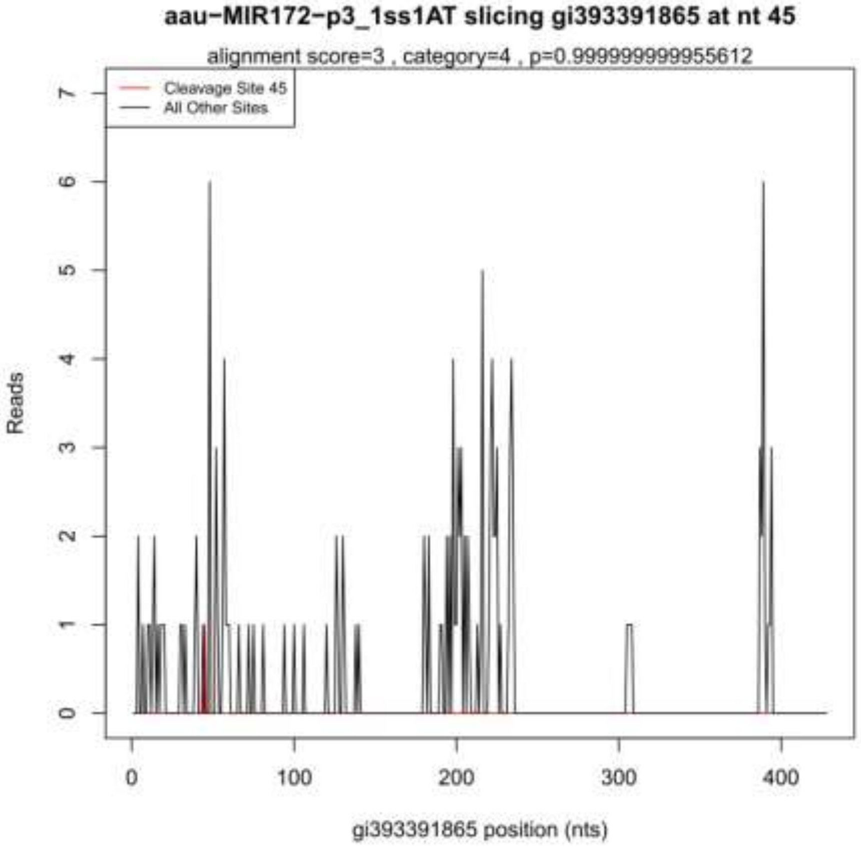
aau−MIR172−p3_1ss1AT slicing gi393391865 at nt 45
alignment score=3 , category=4 , p=0.999999999955612
Cleavage Site 45
All Other Sites
Reads
gi393391865 position (nts)

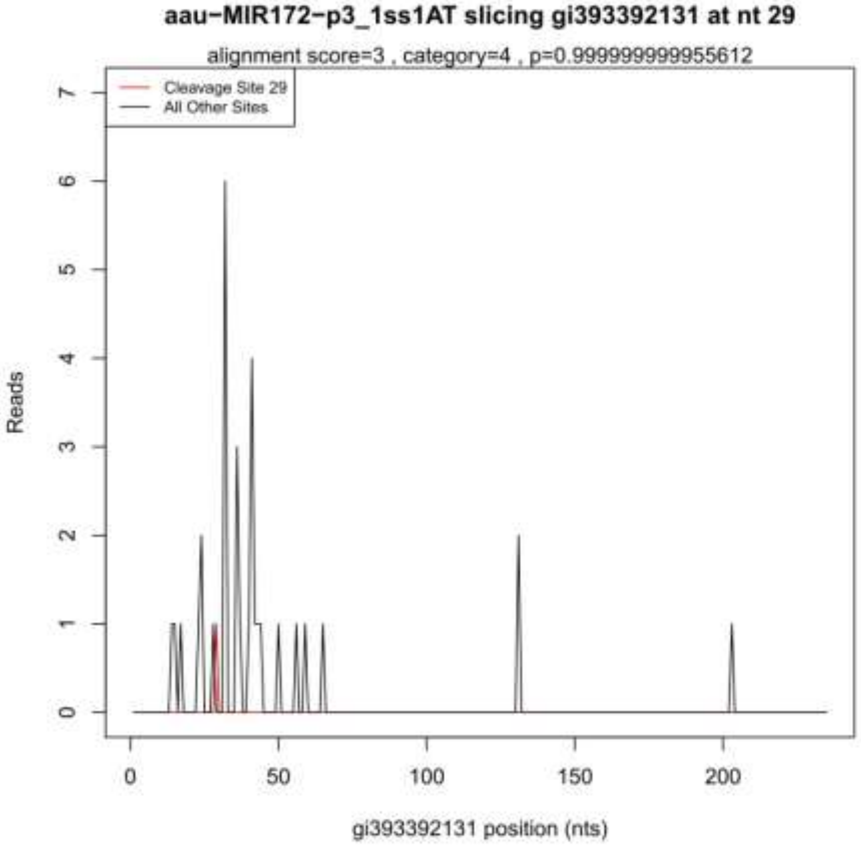
aau−MIR172−p3_1ss1AT slicing gi393392131 at nt 29
alignment score=3 , category=4 , p=0.999999999955612
Cleavage Site 29
All Other Sites
Reads
0
1
2
3
4
5
6
7
0
50
100
150
200
gi393392131 position (nts)

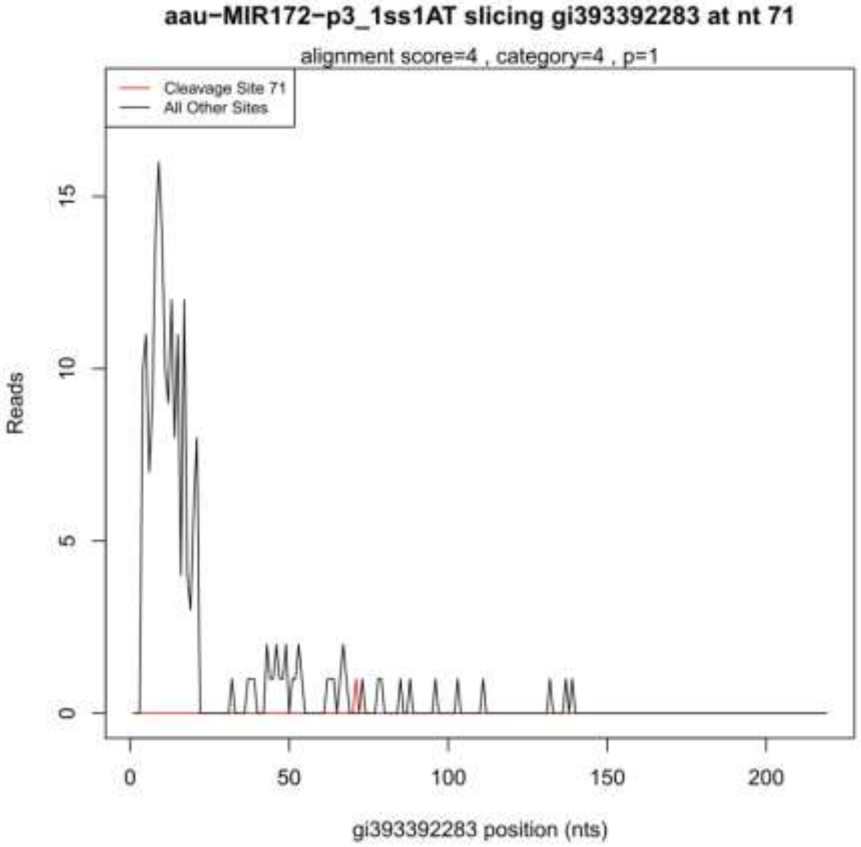
aau−MIR172−p3_1ss1AT slicing gi393392283 at nt 71
alignment score=4 , category=4 , p=1
Cleavage Site 71
All Other Sites
Reads
0
5
10
15
0
50
100
150
200
gi393392283 position (nts)

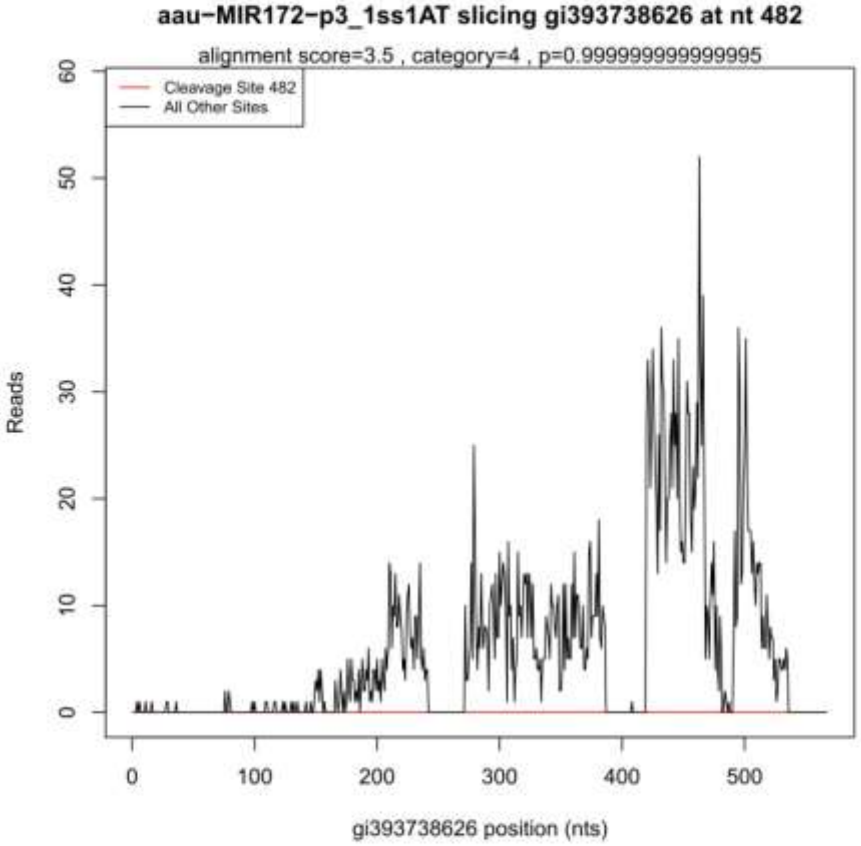
aau−MIR172−p3_1ss1AT slicing gi393738626 at nt 482
alignment score=3.5 , category=4 , p=0.999999999999995
Cleavage Site 482
All Other Sites
Reads
0
10
20
30
40
50
60
0
100
200
300
400
500
gi393738626 position (nts)

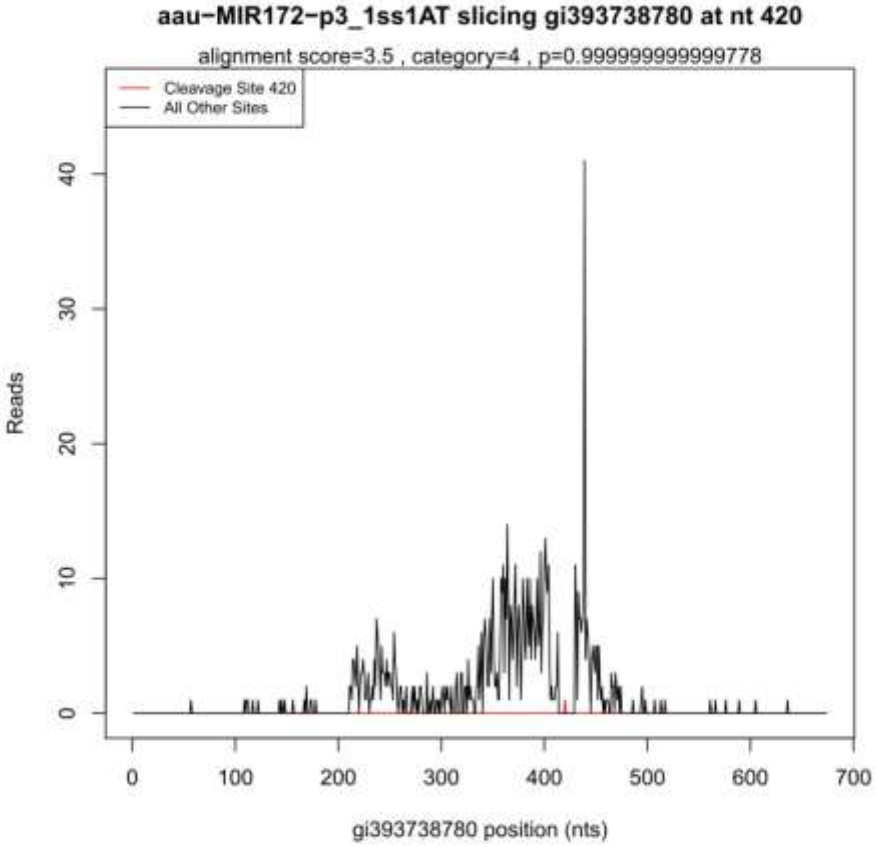
aau−MIR172−p3_1ss1AT slicing gi393738780 at nt 420
alignment score=3.5 , category=4 , p=0.999999999999778
Cleavage Site 420
All Other Sites
Reads
0
10
20
30
40
0
100
200
300
400
500
600
700
gi393738780 position (nts)

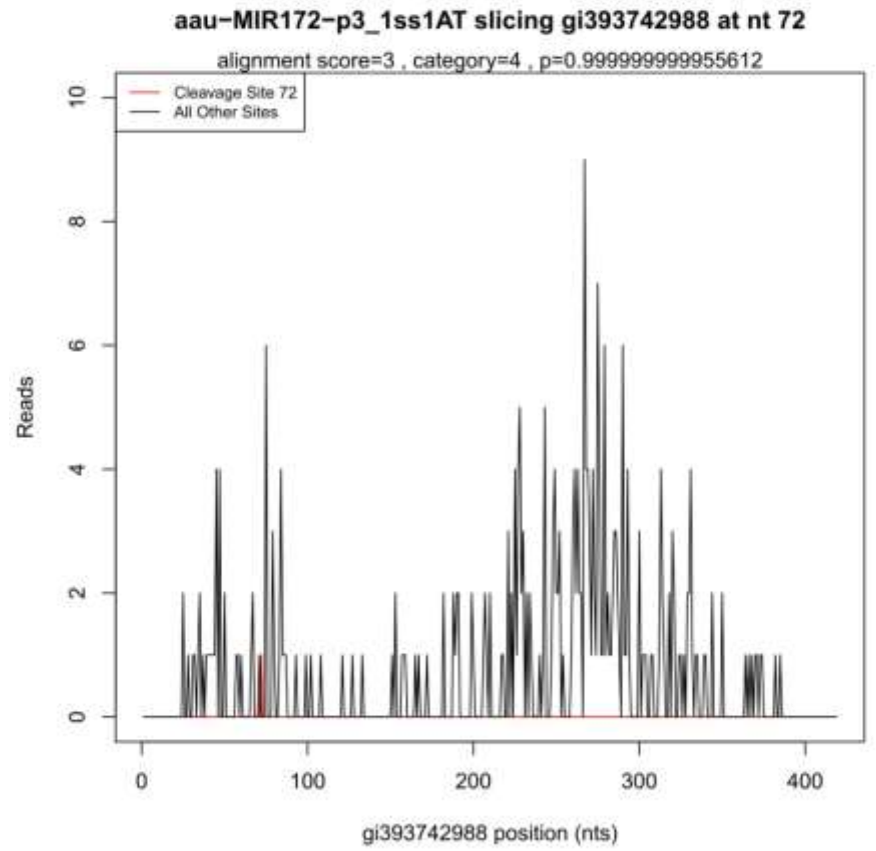
aau−MIR172−p3_1ss1AT slicing gi393742988 at nt 72
alignment score=3 , category=4 , p=0.999999999955612
Cleavage Site 72
All Other Sites
Reads
0
2
4
6
8
10
0
100
200
300
400
gi393742988 position (nts)

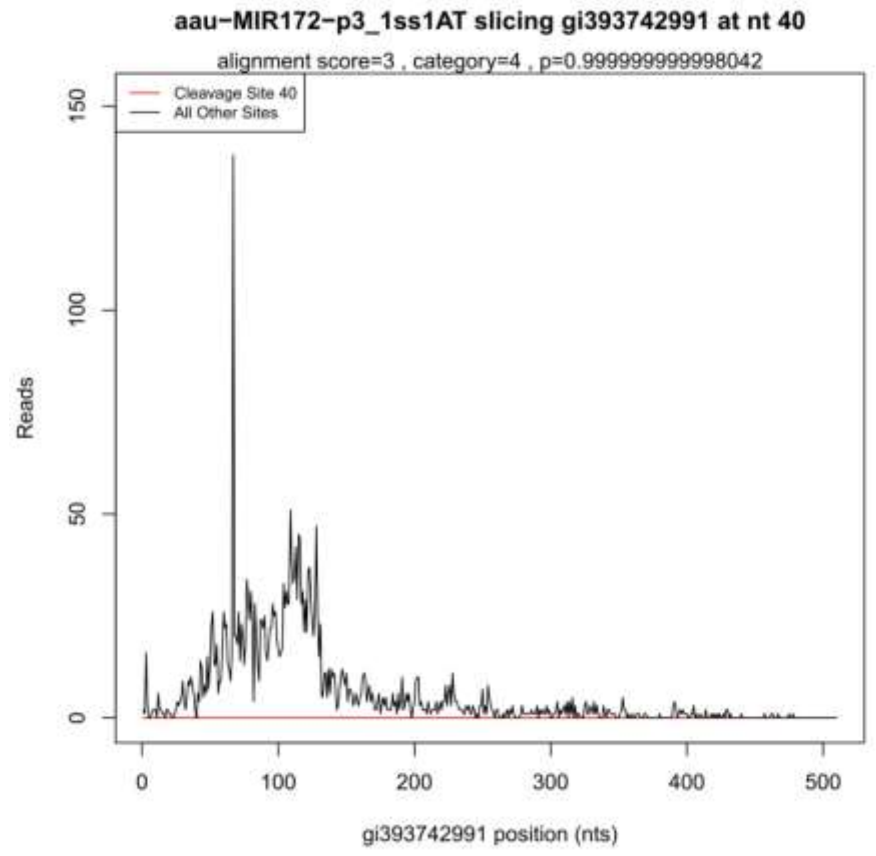
aau−MIR172−p3_1ss1AT slicing gi393742991 at nt 40
alignment score=3 , category=4 , p=0.999999999998042
Cleavage Site 40
All Other Sites
Reads
0
50
100
150
0
100
200
300
400
500
gi393742991 position (nts)

aau−MIR172−p3_1ss1AT slicing gi393743810 at nt 66

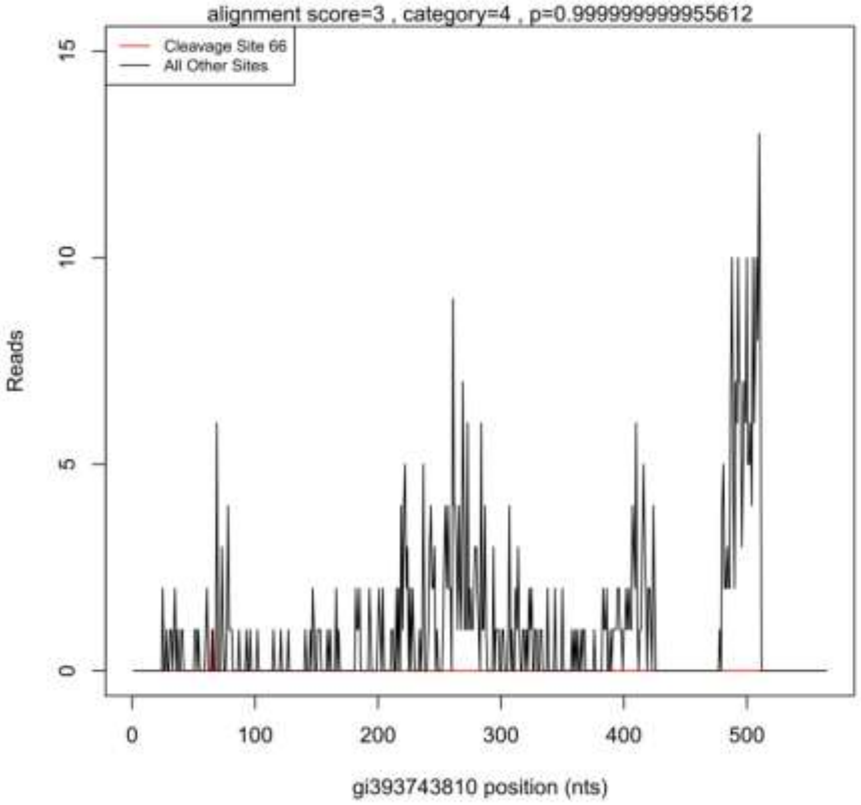

aau−MIR172−p3_1ss1AT slicing gi393744727 at nt 57

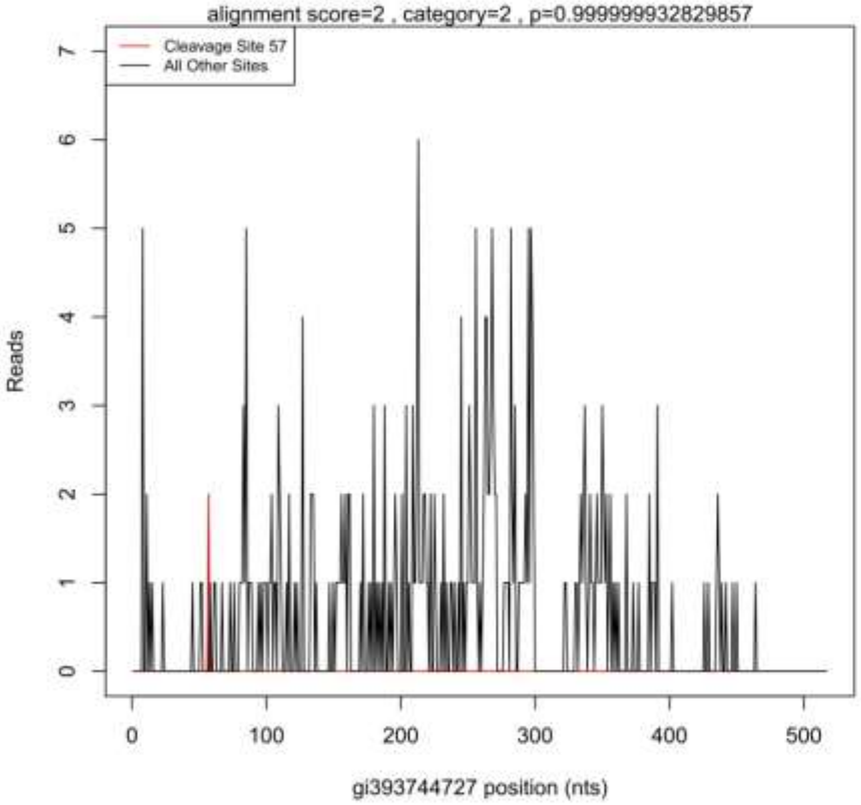

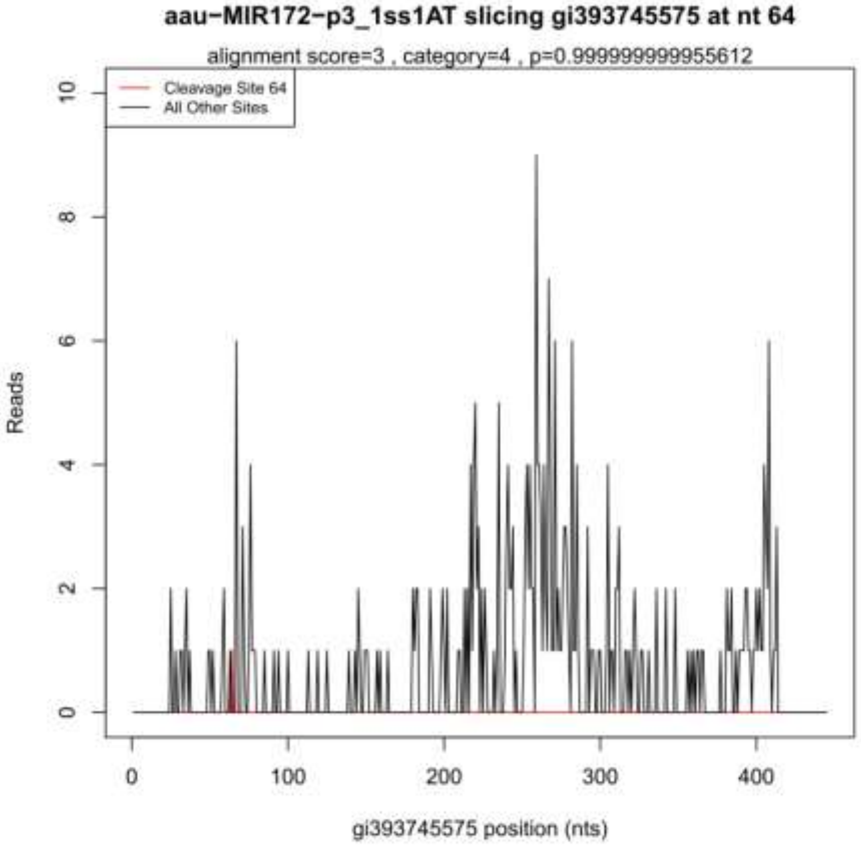
aau−MIR172−p3_1ss1AT slicing gi393745575 at nt 64
alignment score=3 , category=4 , p=0.999999999955612
Cleavage Site 64
All Other Sites
Reads
0
2
4
6
8
10
0
100
200
300
400
gi393745575 position (nts)

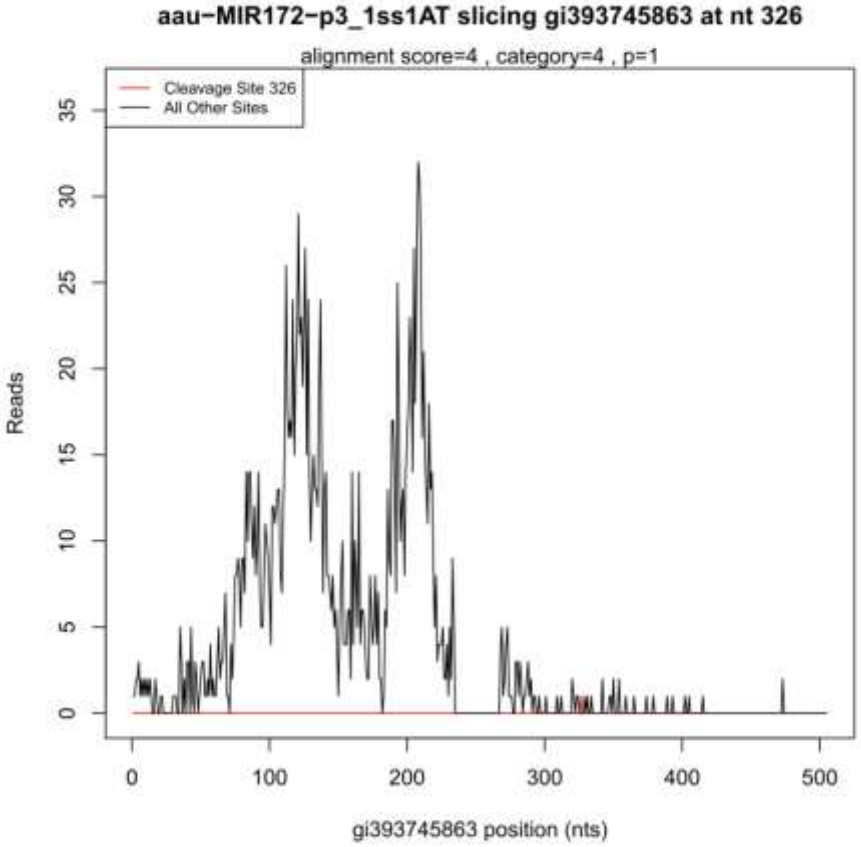
aau−MIR172−p3_1ss1AT slicing gi393745863 at nt 326
alignment score=4 , category=4 , p=1
Cleavage Site 326
All Other Sites
Reads
0
5
10
15
20
25
30
35
0
100
200
300
400
500
gi393745863 position (nts)

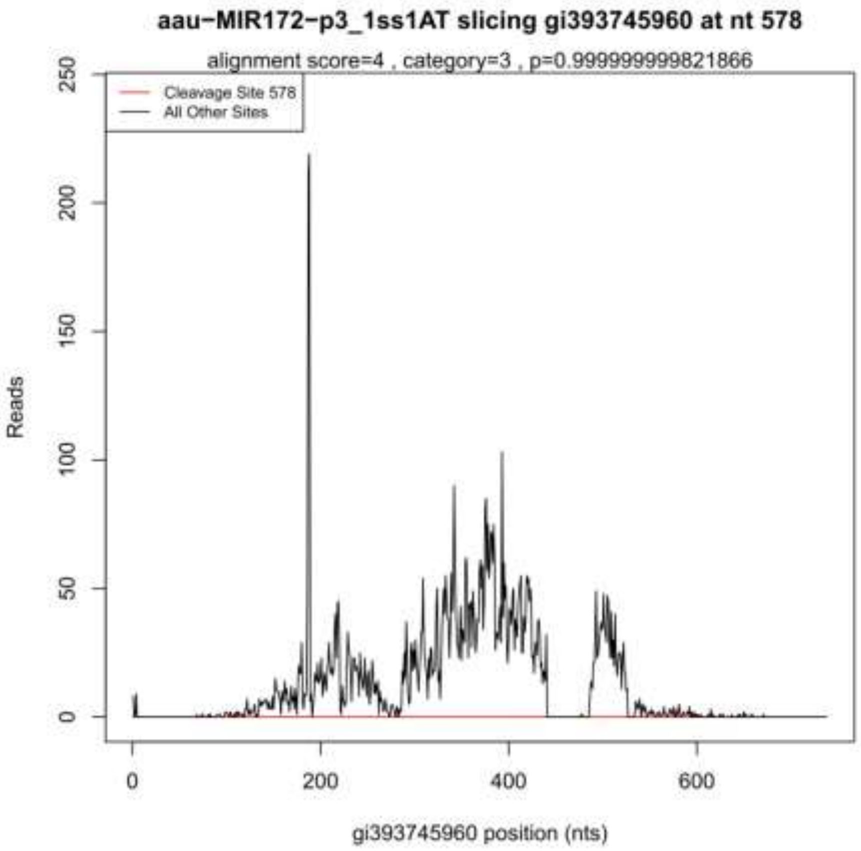
aau−MIR172−p3_1ss1AT slicing gi393745960 at nt 578
alignment score=4 , category=3 , p=0.999999999821866
Cleavage Site 578
All Other Sites
Reads
0
50
100
150
200
250
0
200
400
600
gi393745960 position (nts)

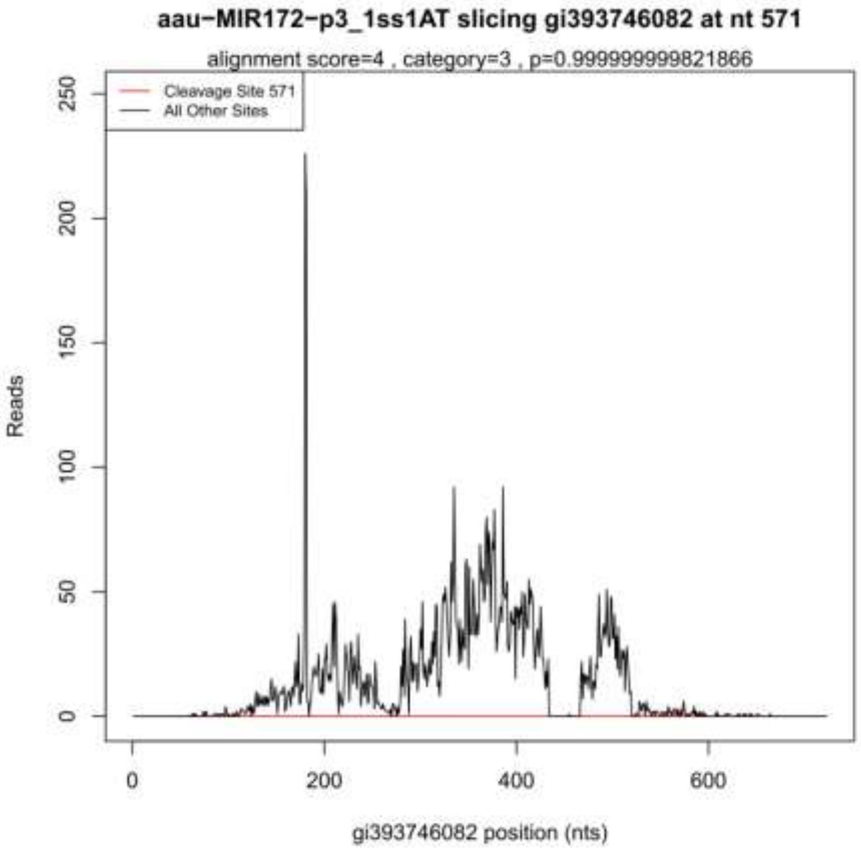
aau−MIR172−p3_1ss1AT slicing gi393746082 at nt 571
alignment score=4 , category=3 , p=0.999999999821866
Cleavage Site 571
All Other Sites
Reads
0
50
100
150
200
250
0
200
400
600
gi393746082 position (nts)

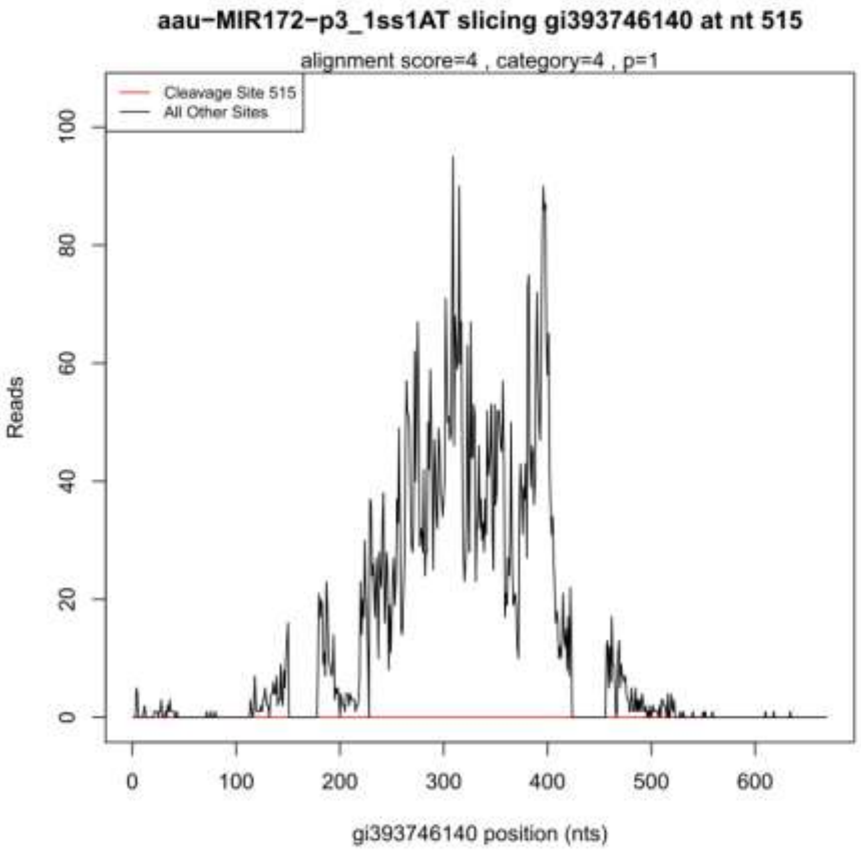
aau−MIR172−p3_1ss1AT slicing gi393746140 at nt 515
alignment score=4 , category=4 , p=1
Cleavage Site 515
All Other Sites
Reads
0
20
40
60
80
100
0
100
200
300
400
500
600
gi393746140 position (nts)

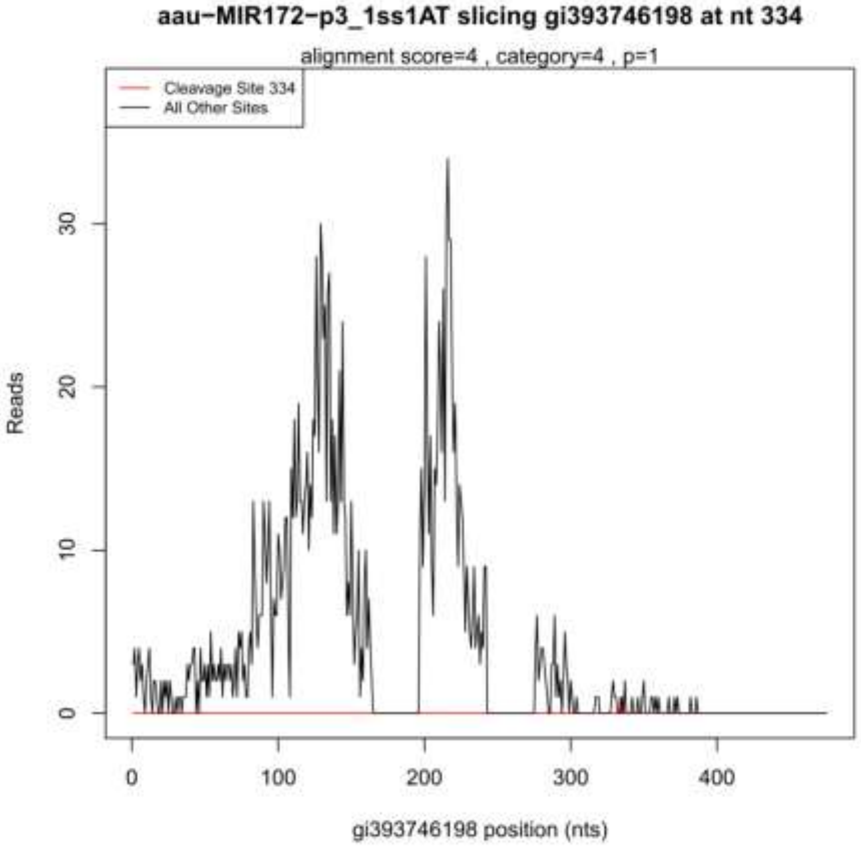
aau−MIR172−p3_1ss1AT slicing gi393746198 at nt 334
alignment score=4 , category=4 , p=1
Cleavage Site 334
All Other Sites
Reads
0
10
20
30
0
100
200
300
400
gi393746198 position (nts)

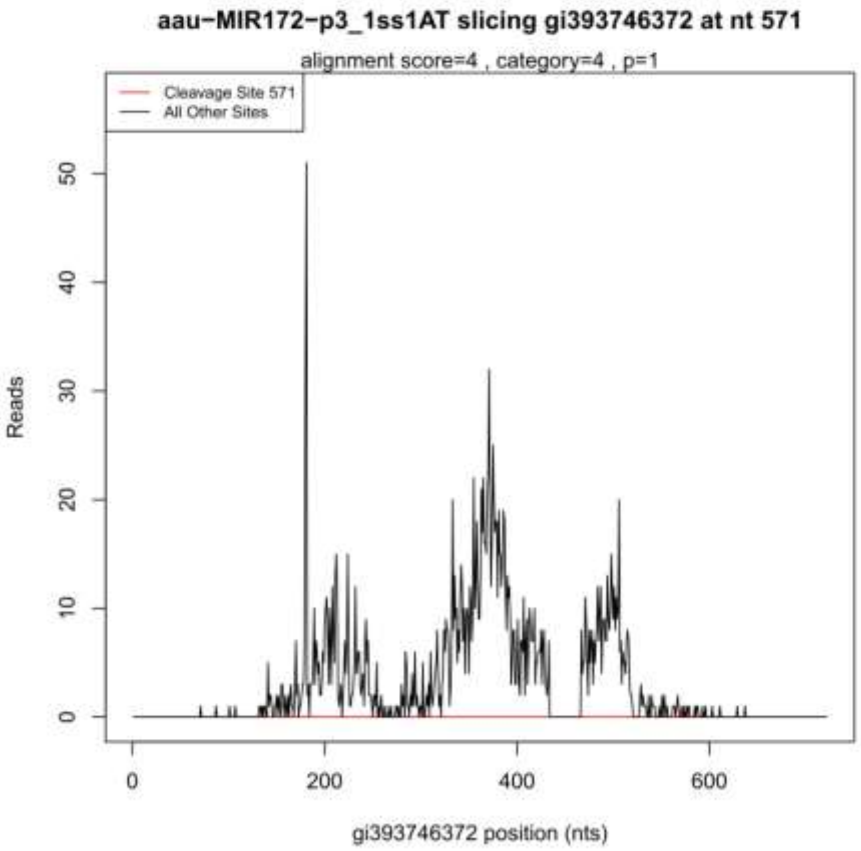
aau−MIR172−p3_1ss1AT slicing gi393746372 at nt 571
alignment score=4 , category=4 , p=1
Cleavage Site 571
All Other Sites
Reads
0
10
20
30
40
50
0
200
400
600
gi393746372 position (nts)

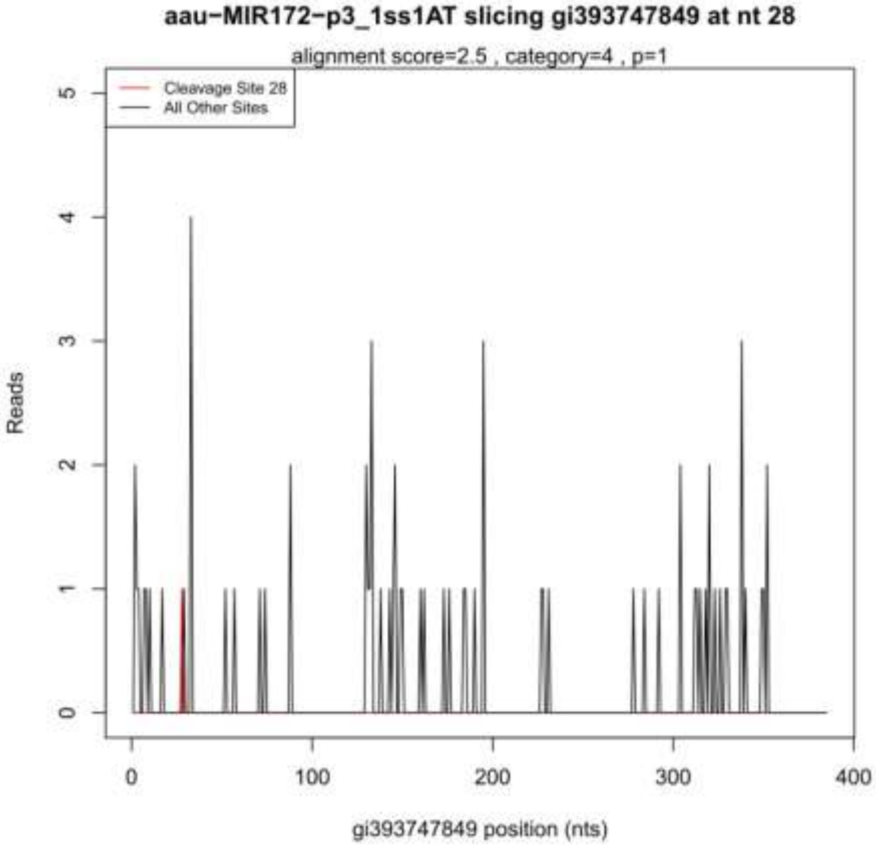
aau−MIR172−p3_1ss1AT slicing gi393747849 at nt 28
alignment score=2.5 , category=4 , p=1
Cleavage Site 28
All Other Sites
Reads
0
1
2
3
4
5
0
100
200
300
400
gi393747849 position (nts)

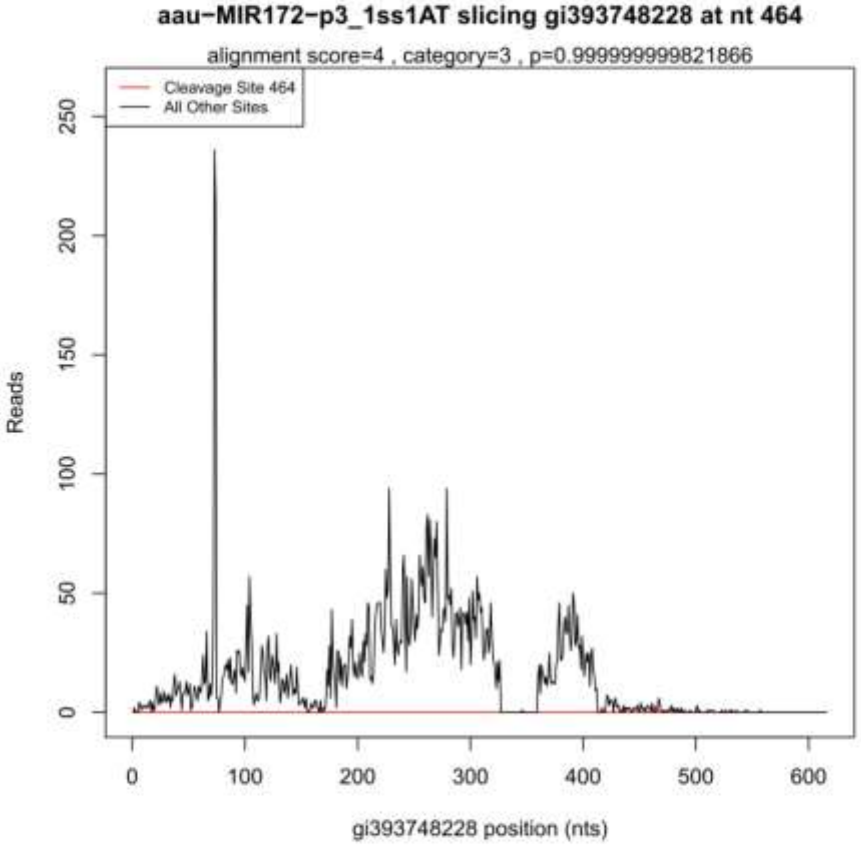
aau−MIR172−p3_1ss1AT slicing gi393748228 at nt 464
alignment score=4 , category=3 , p=0.999999999821866
Cleavage Site 464
All Other Sites
Reads
0
50
100
150
200
250
0
100
200
300
400
500
600
gi393748228 position (nts)

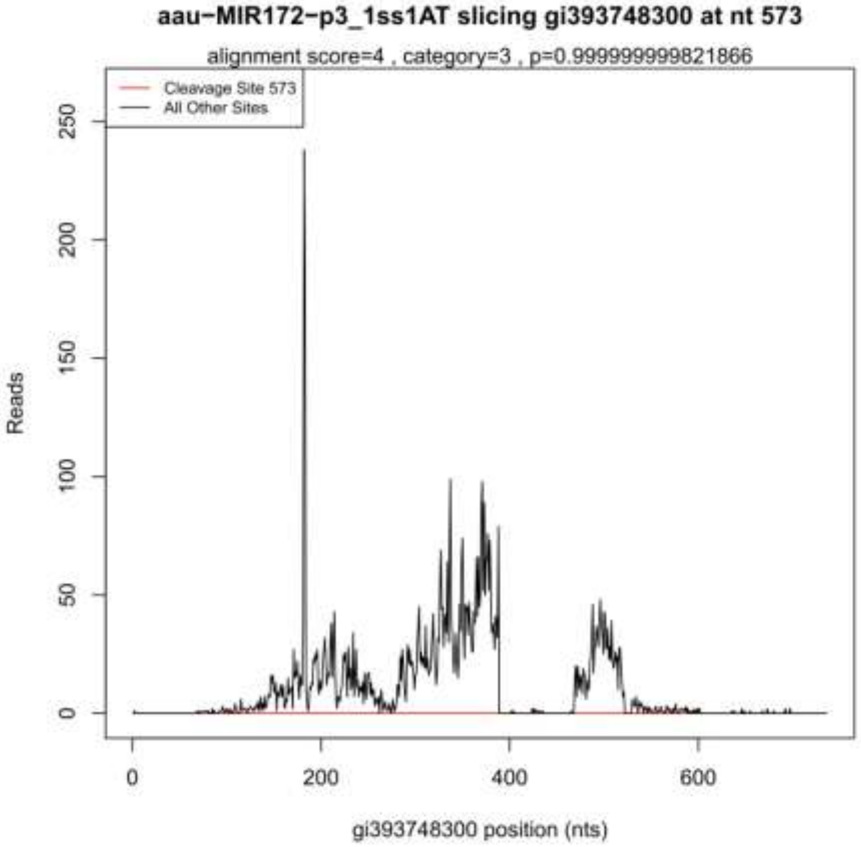
aau−MIR172−p3_1ss1AT slicing gi393748300 at nt 573
alignment score=4 , category=3 , p=0.999999999821866
Cleavage Site 573
All Other Sites
Reads
0
50
100
150
200
250
0
200
400
600
gi393748300 position (nts)

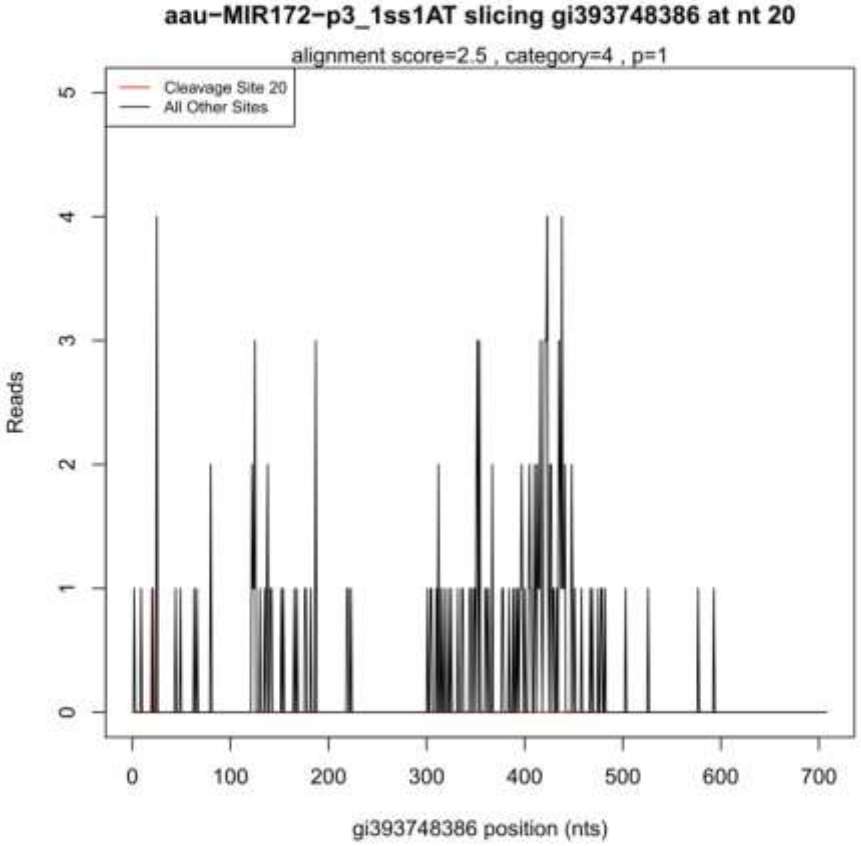
aau−MIR172−p3_1ss1AT slicing gi393748386 at nt 20
alignment score=2.5 , category=4 , p=1
Cleavage Site 20
All Other Sites
Reads
0
1
2
3
4
5
0
100
200
300
400
500
600
700
gi393748386 position (nts)

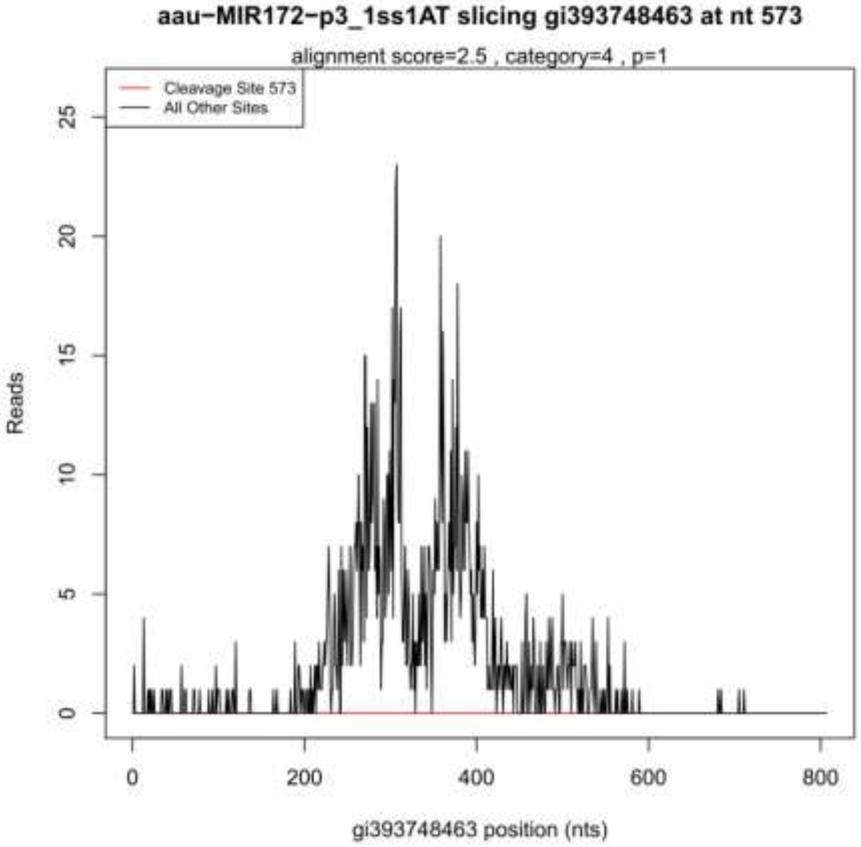
aau−MIR172−p3_1ss1AT slicing gi393748463 at nt 573
alignment score=2.5 , category=4 , p=1
Cleavage Site 573
All Other Sites
Reads
0
5
10
15
20
25
0
200
400
600
800
gi393748463 position (nts)

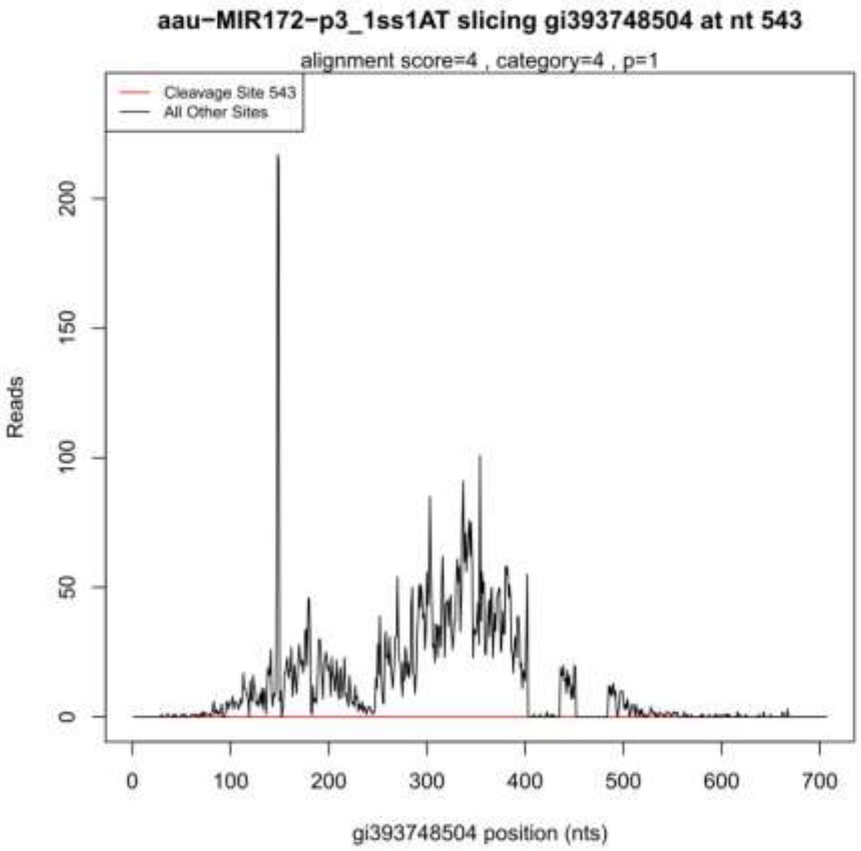

aau−MIR172−p3_1ss1AT slicing gi393748504 at nt 543
alignment score=4 , category=4 , p=1
Cleavage Site 543
All Other Sites
Reads
0
50
100
150
200
0
100
200
300
400
500
600
700
gi393748504 position (nts)

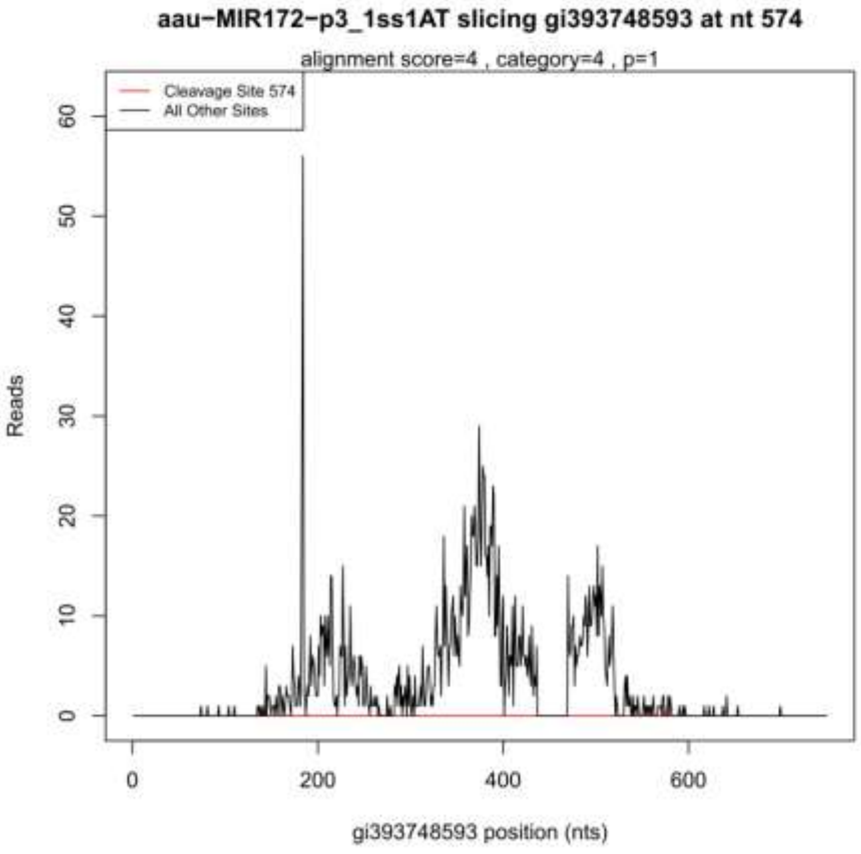

aau−MIR172−p3_1ss1AT slicing gi393748593 at nt 574
alignment score=4 , category=4 , p=1
Cleavage Site 574
All Other Sites
Reads
0
10
20
30
40
50
60
0
200
400
600
gi393748593 position (nts)

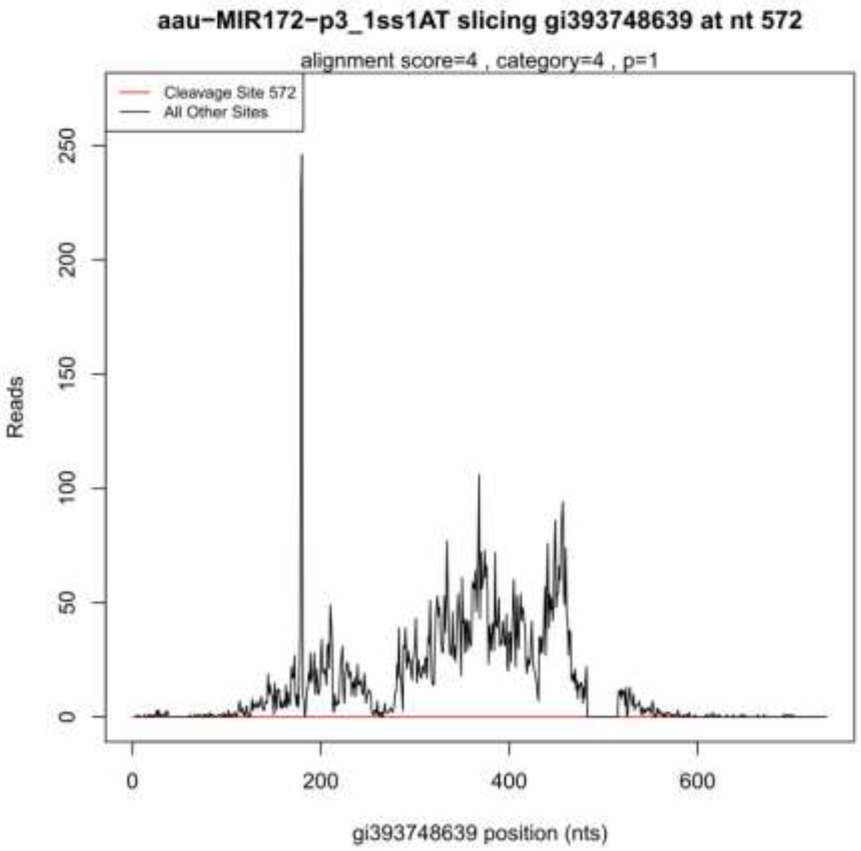
aau−MIR172−p3_1ss1AT slicing gi393748639 at nt 572
alignment score=4 , category=4 , p=1
Cleavage Site 572
All Other Sites
Reads
0
50
100
150
200
250
0
200
400
600
gi393748639 position (nts)

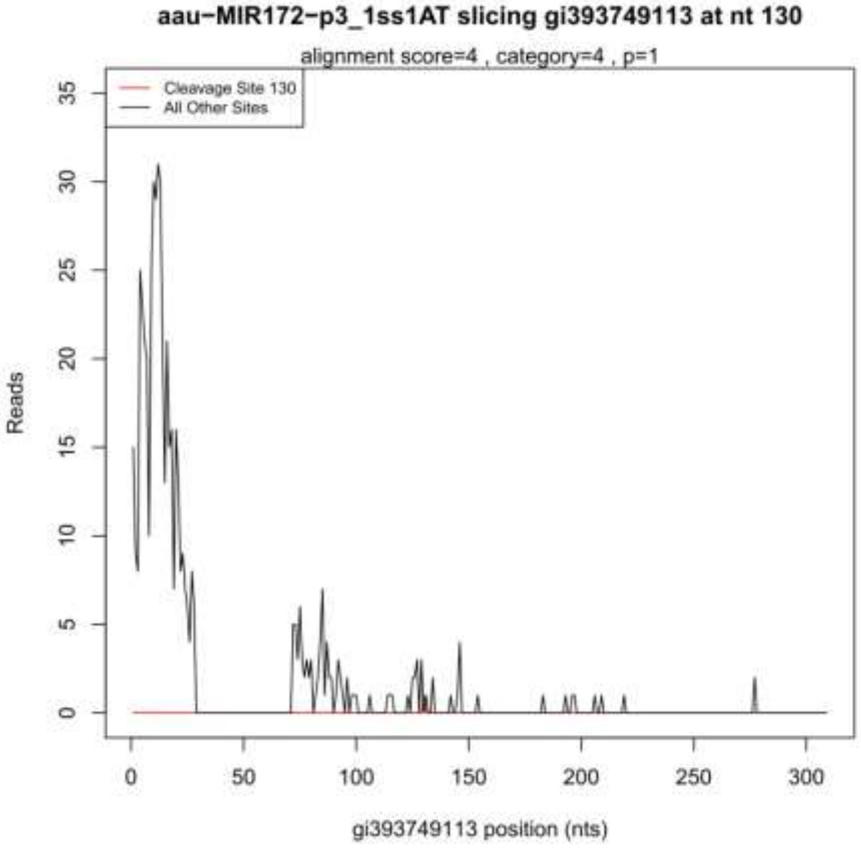
aau−MIR172−p3_1ss1AT slicing gi393749113 at nt 130
alignment score=4 , category=4 , p=1
Cleavage Site 130
All Other Sites
Reads
0
5
10
15
20
25
30
35
0
50
100
150
200
250
300
gi393749113 position (nts)

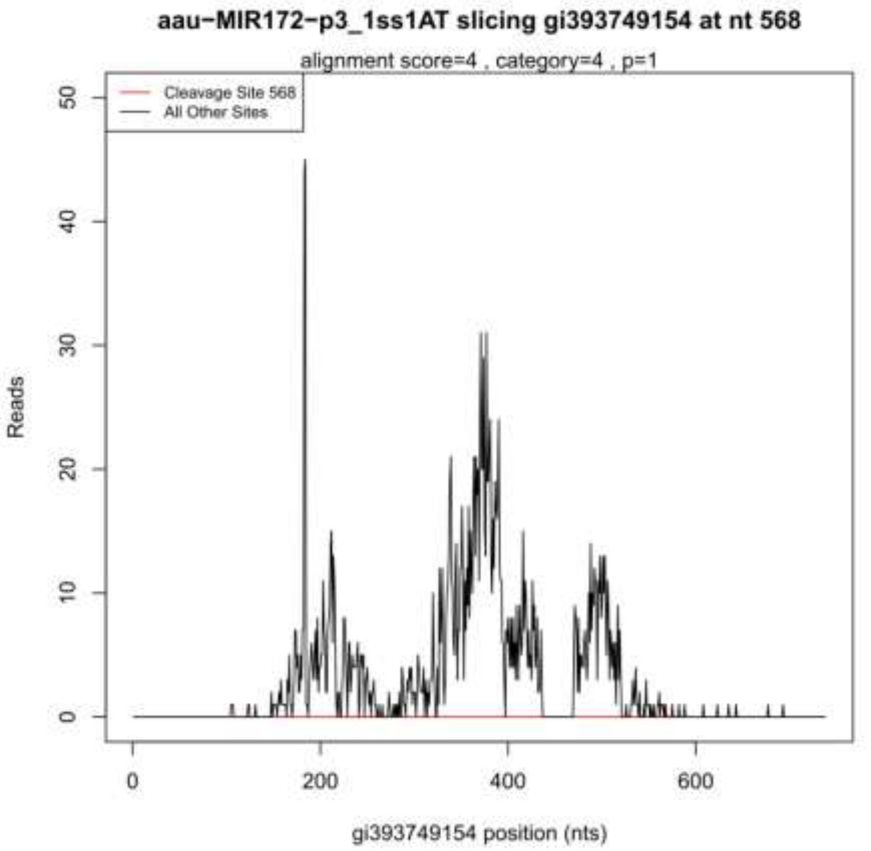
aau−MIR172−p3_1ss1AT slicing gi393749154 at nt 568
alignment score=4 , category=4 , p=1
Cleavage Site 568
All Other Sites
Reads
0
10
20
30
40
50
0
200
400
600
gi393749154 position (nts)

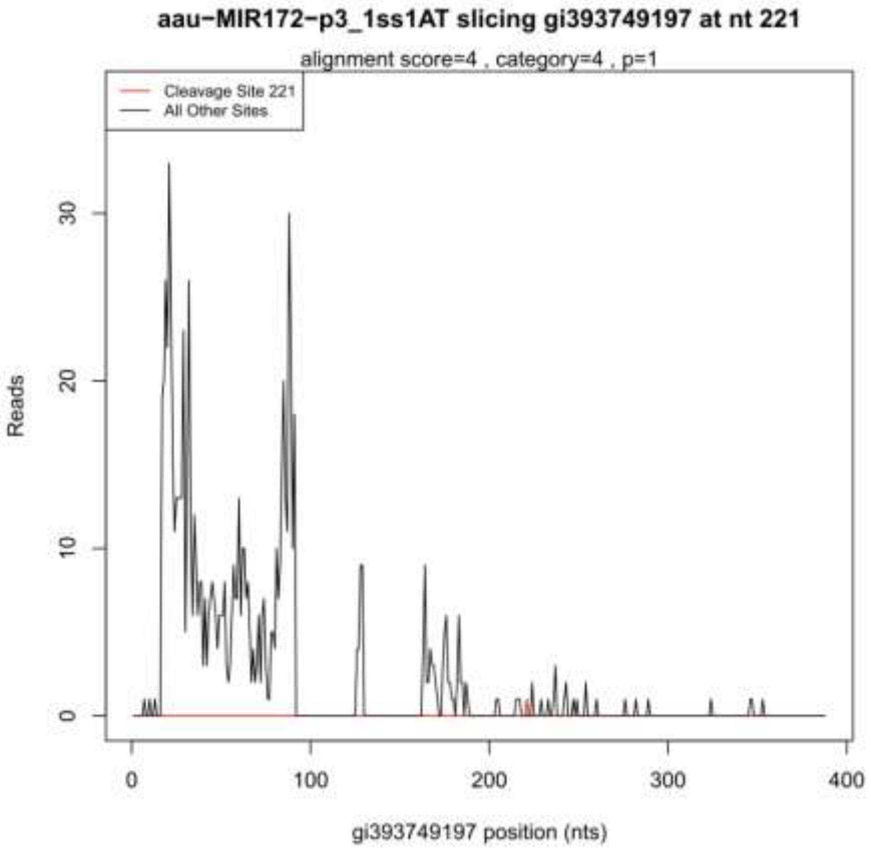
aau−MIR172−p3_1ss1AT slicing gi393749197 at nt 221
alignment score=4 , category=4 , p=1
Cleavage Site 221
All Other Sites
Reads
0
10
20
30
0
100
200
300
400
gi393749197 position (nts)

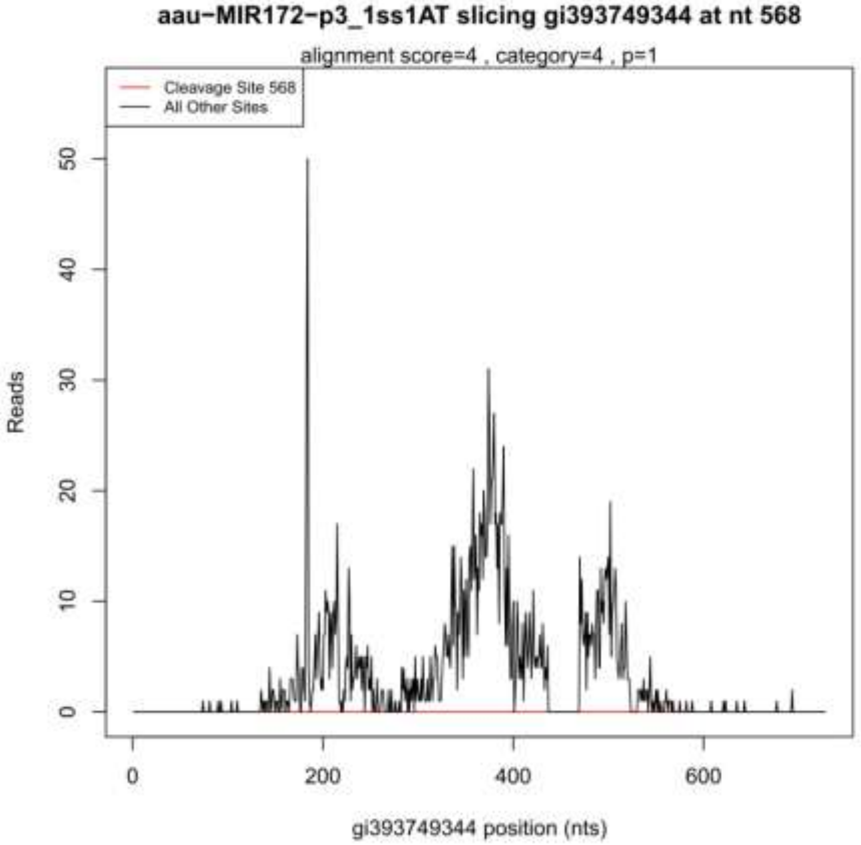
aau−MIR172−p3_1ss1AT slicing gi393749344 at nt 568
alignment score=4 , category=4 , p=1
Cleavage Site 568
All Other Sites
Reads
0
10
20
30
40
50
0
200
400
600
gi393749344 position (nts)

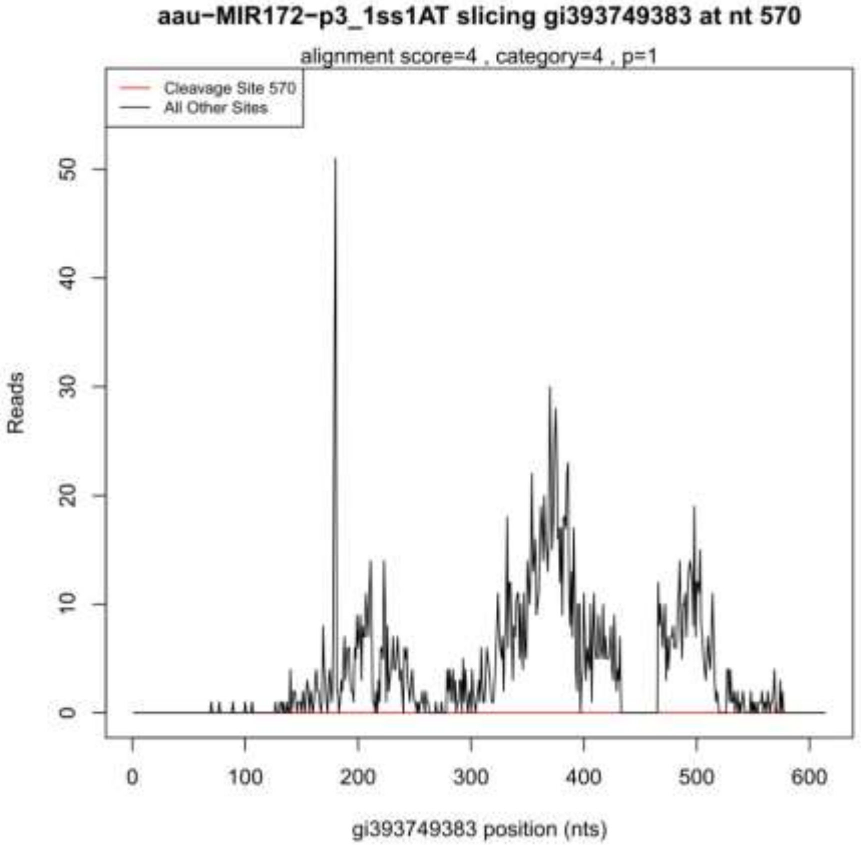
aau−MIR172−p3_1ss1AT slicing gi393749383 at nt 570
alignment score=4 , category=4 , p=1
Cleavage Site 570
All Other Sites
Reads
0
10
20
30
40
50
0
100
200
300
400
500
600
gi393749383 position (nts)

aau−MIR172−p3_1ss1AT slicing gi393749523 at nt 571

alignment score=4 , category=4 , p=1

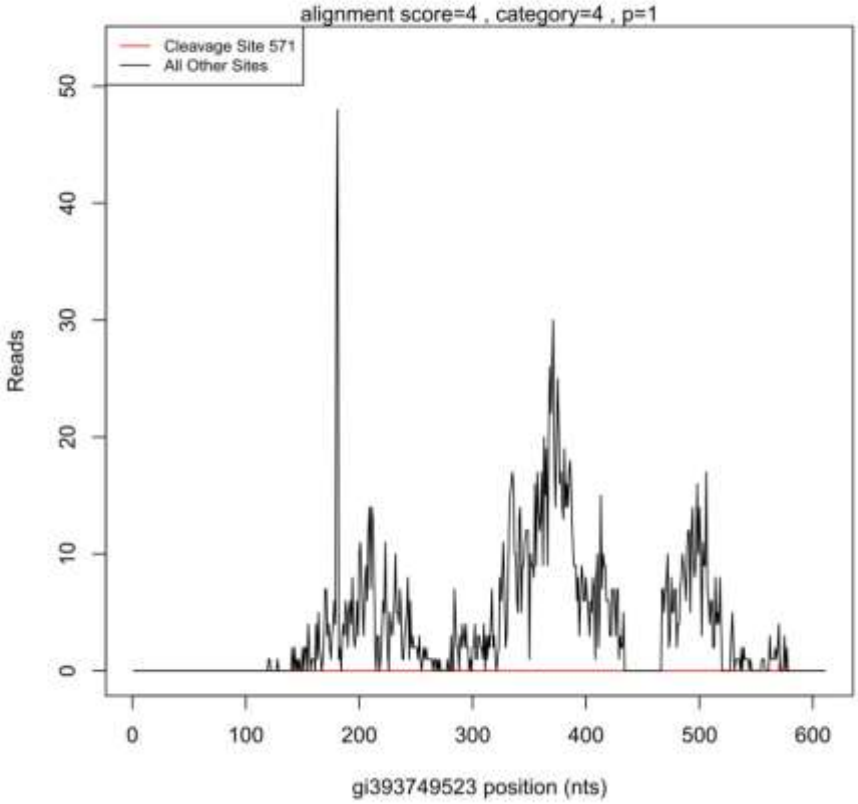

aau−MIR172−p3_1ss1AT slicing gi393749747 at nt 570

alignment score=4 , category=4 , p=1

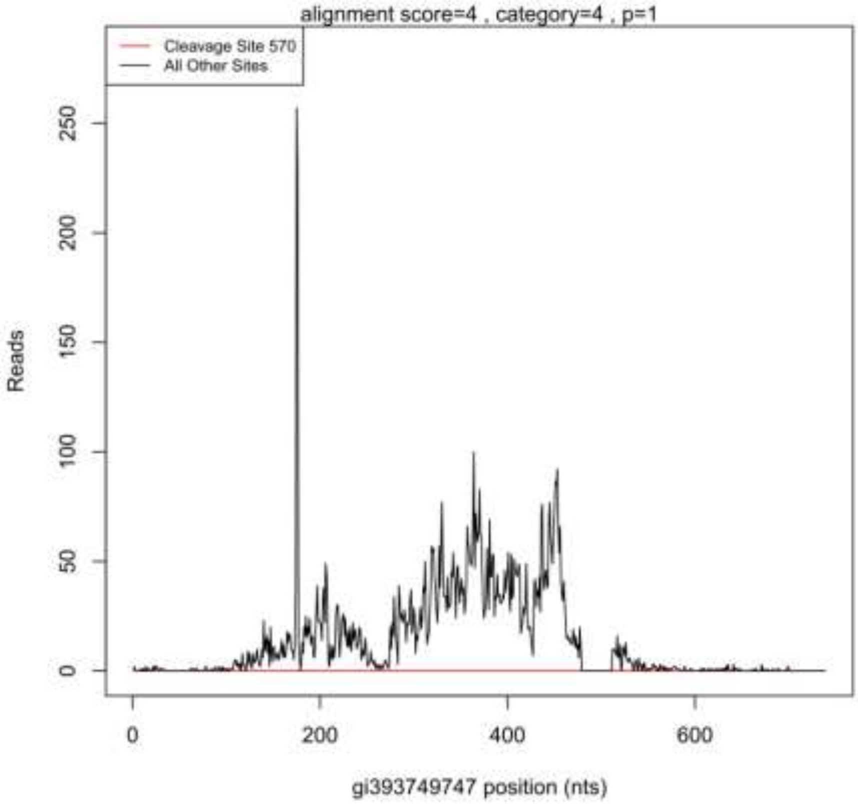

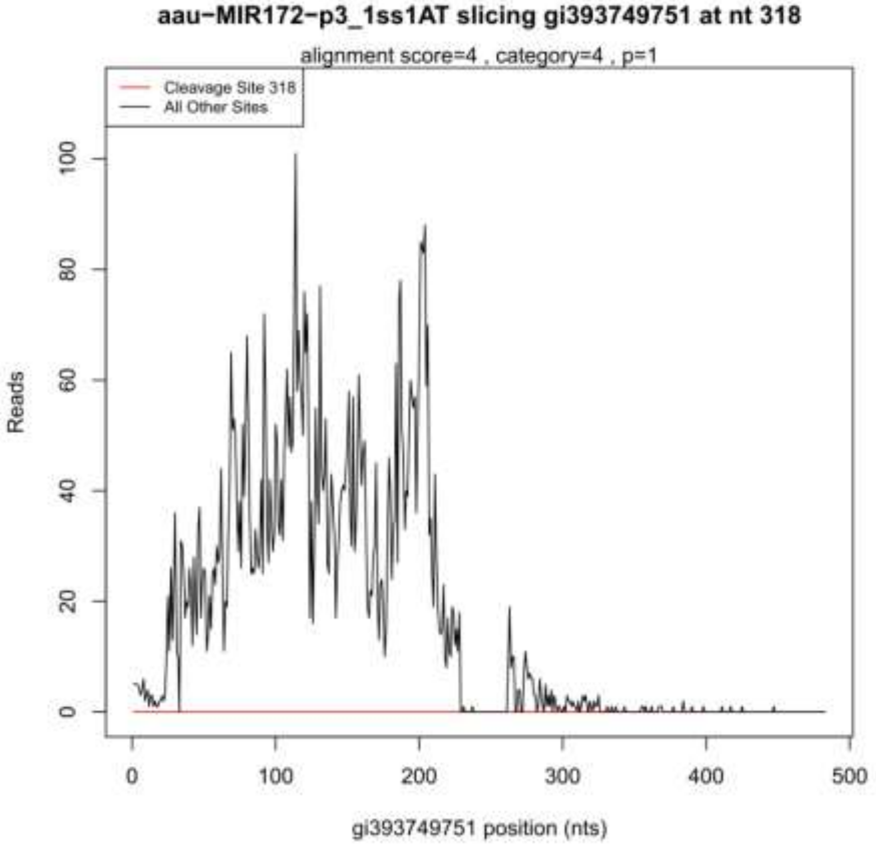
aau−MIR172−p3_1ss1AT slicing gi393749751 at nt 318
alignment score=4 , category=4 , p=1
Cleavage Site 318
All Other Sites
Reads
0
20
40
60
80
100
0
100
200
300
400
500
gi393749751 position (nts)

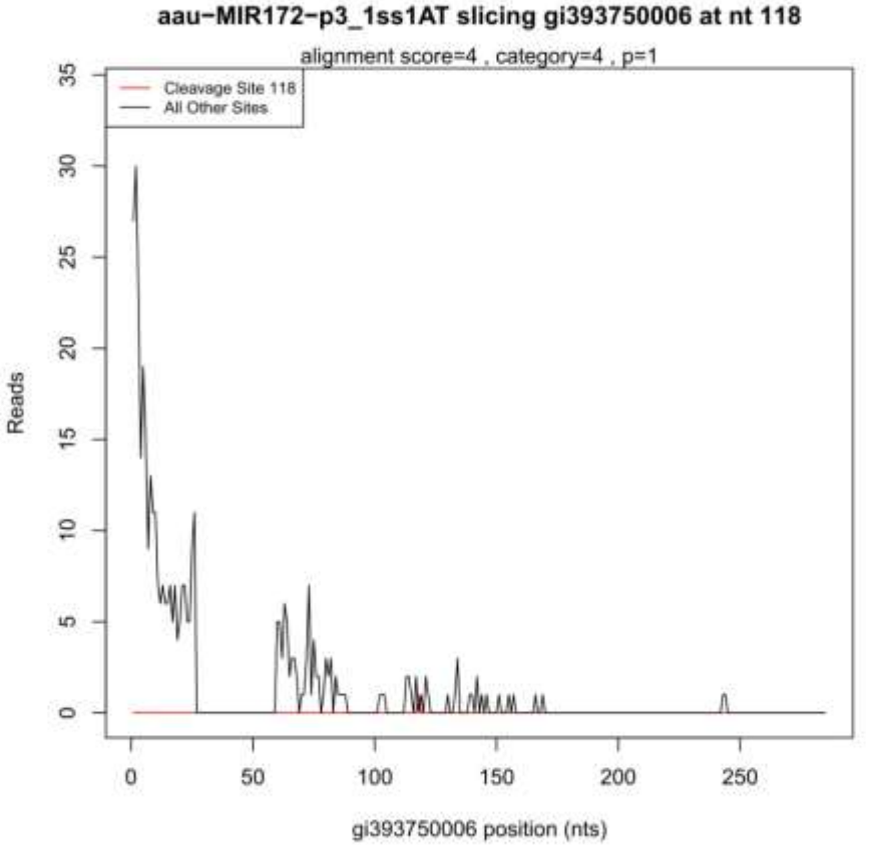
aau−MIR172−p3_1ss1AT slicing gi393750006 at nt 118
alignment score=4 , category=4 , p=1
Cleavage Site 118
All Other Sites
Reads
0
5
10
15
20
25
30
35
0
50
100
150
200
250
gi393750006 position (nts)

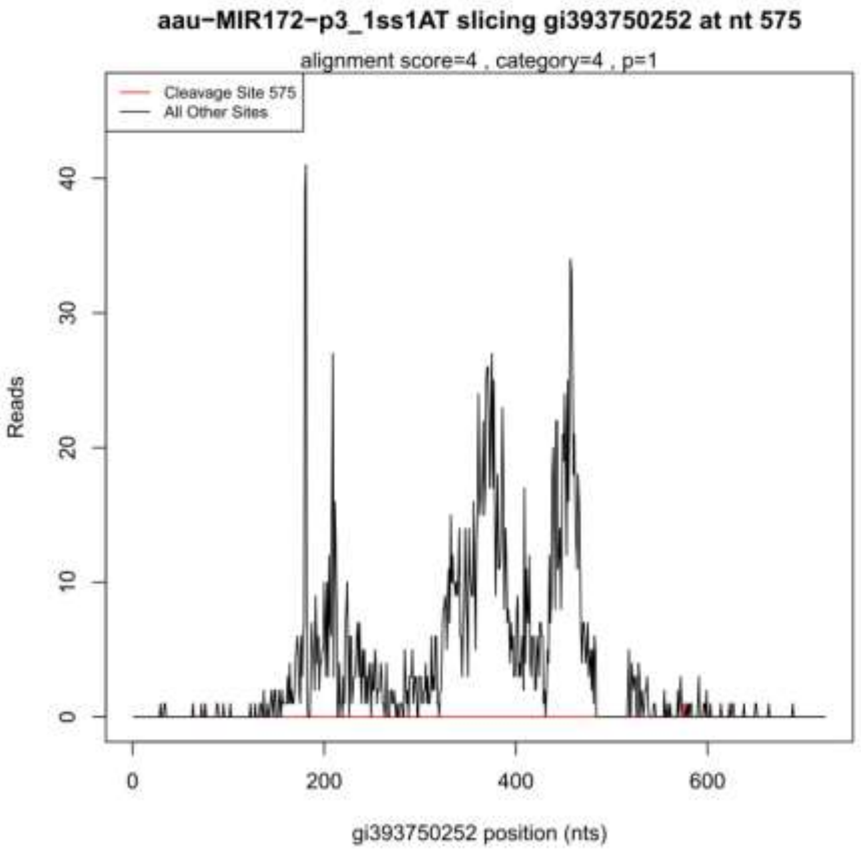

aau−MIR172−p3_1ss1AT slicing gi393750252 at nt 575
alignment score=4 , category=4 , p=1
Cleavage Site 575
All Other Sites
Reads
0
10
20
30
40
0
200
400
600
gi393750252 position (nts)

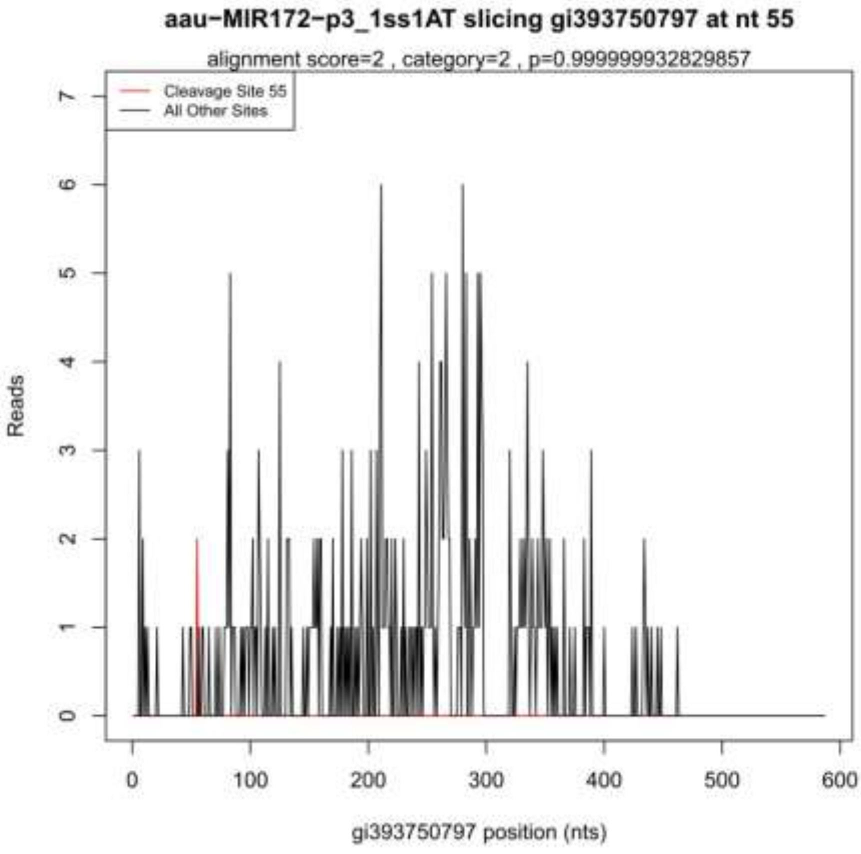

aau−MIR172−p3_1ss1AT slicing gi393750797 at nt 55
alignment score=2 , category=2 , p=0.999999932829857
Cleavage Site 55
All Other Sites
Reads
0
1
2
3
4
5
6
7
0
100
200
300
400
500
600
gi393750797 position (nts)

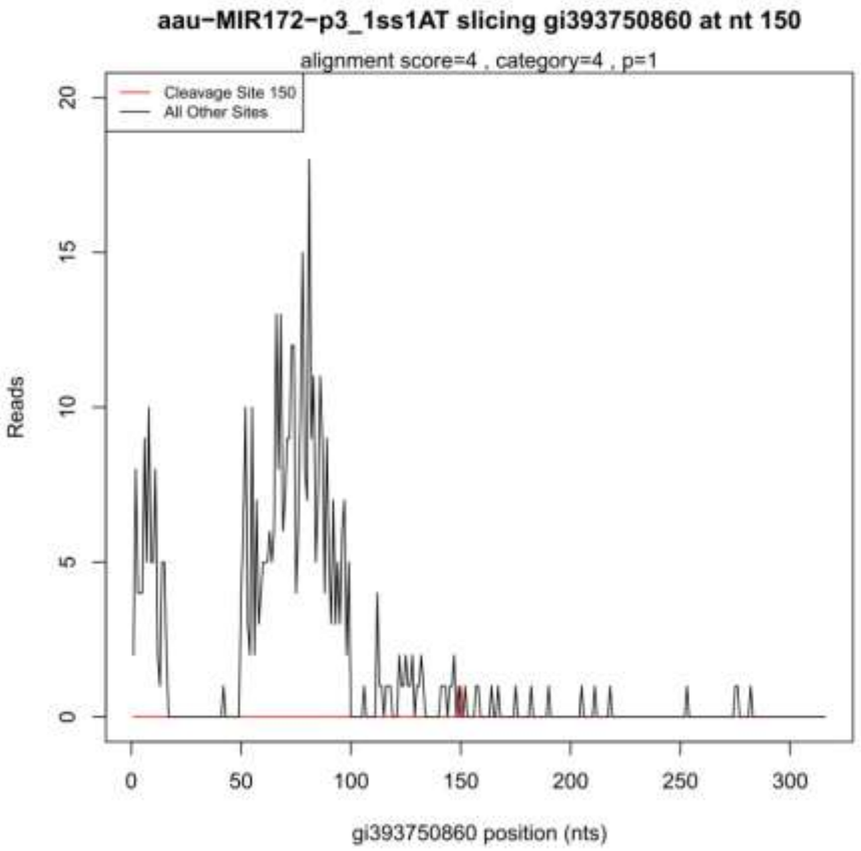
aau−MIR172−p3_1ss1AT slicing gi393750860 at nt 150
alignment score=4 , category=4 , p=1
Cleavage Site 150
All Other Sites
Reads
0
5
10
15
20
0
50
100
150
200
250
300
gi393750860 position (nts)

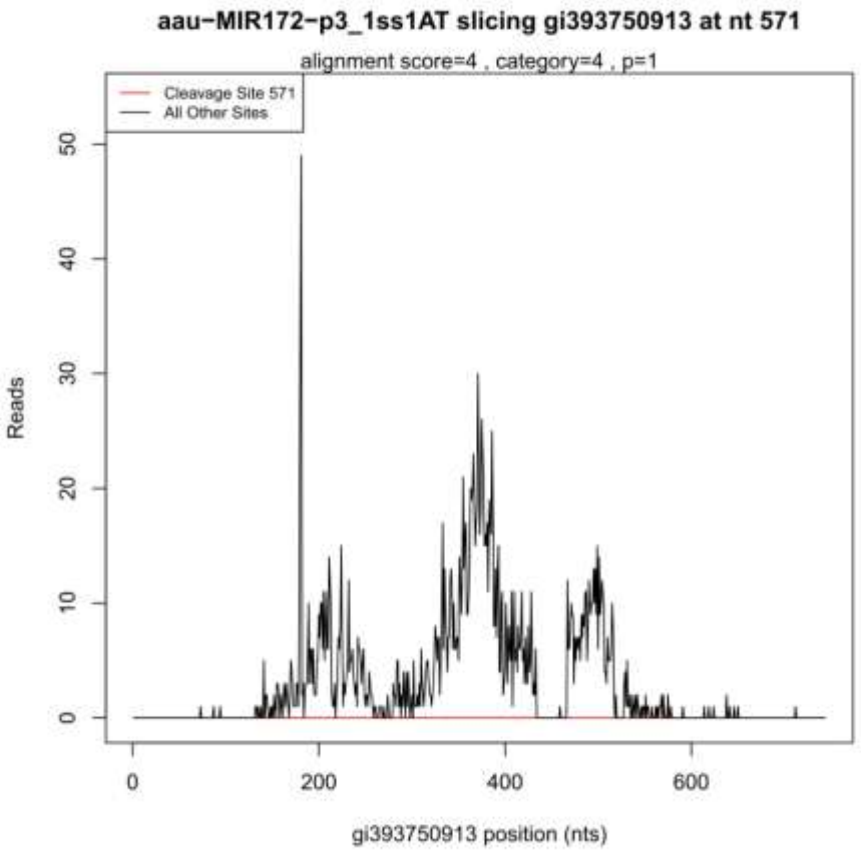
aau−MIR172−p3_1ss1AT slicing gi393750913 at nt 571
alignment score=4 , category=4 , p=1
Cleavage Site 571
All Other Sites
Reads
0
10
20
30
40
50
0
200
400
600
gi393750913 position (nts)

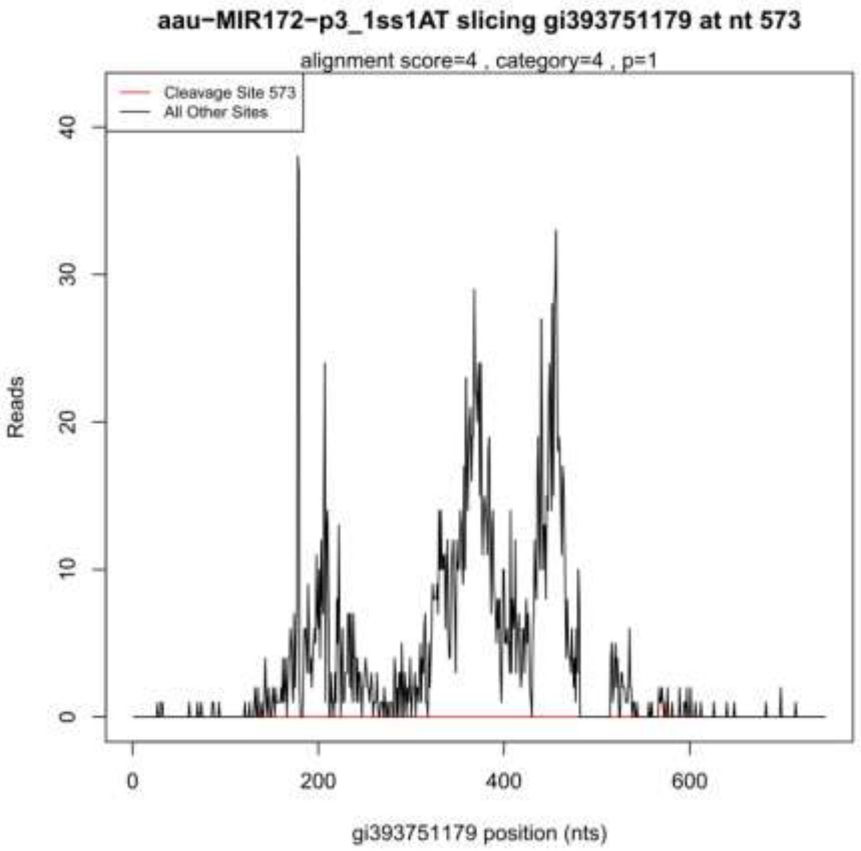
aau−MIR172−p3_1ss1AT slicing gi393751179 at nt 573
alignment score=4 , category=4 , p=1
Cleavage Site 573
All Other Sites
Reads
0
10
20
30
40
0
200
400
600
gi393751179 position (nts)

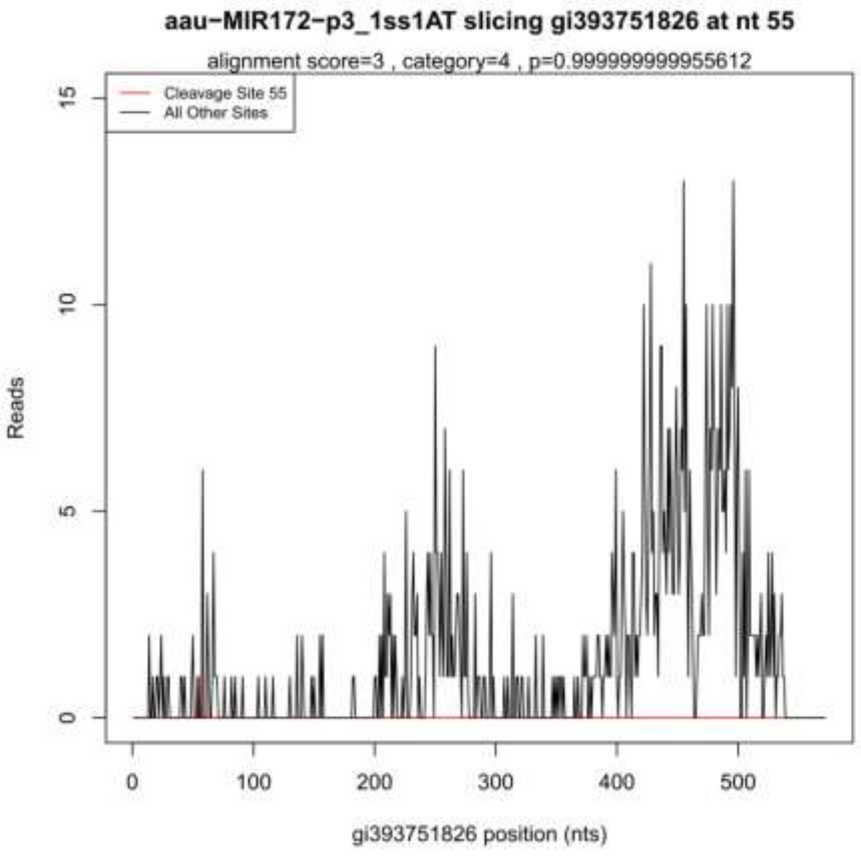
aau−MIR172−p3_1ss1AT slicing gi393751826 at nt 55
alignment score=3 , category=4 , p=0.999999999955612
Cleavage Site 55
All Other Sites
Reads
0
5
10
15
0
100
200
300
400
500
gi393751826 position (nts)

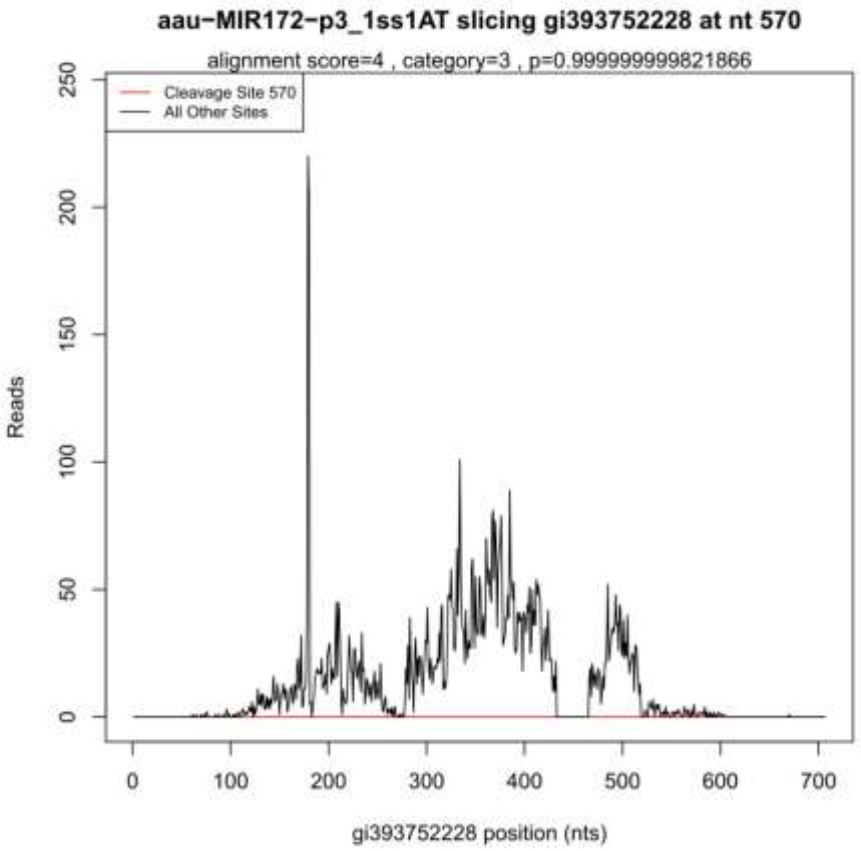

aau−MIR172−p3_1ss1AT slicing gi393752228 at nt 570
alignment score=4 , category=3 , p=0.999999999821866
Cleavage Site 570
All Other Sites
Reads
0
50
100
150
200
250
0
100
200
300
400
500
600
700
gi393752228 position (nts)

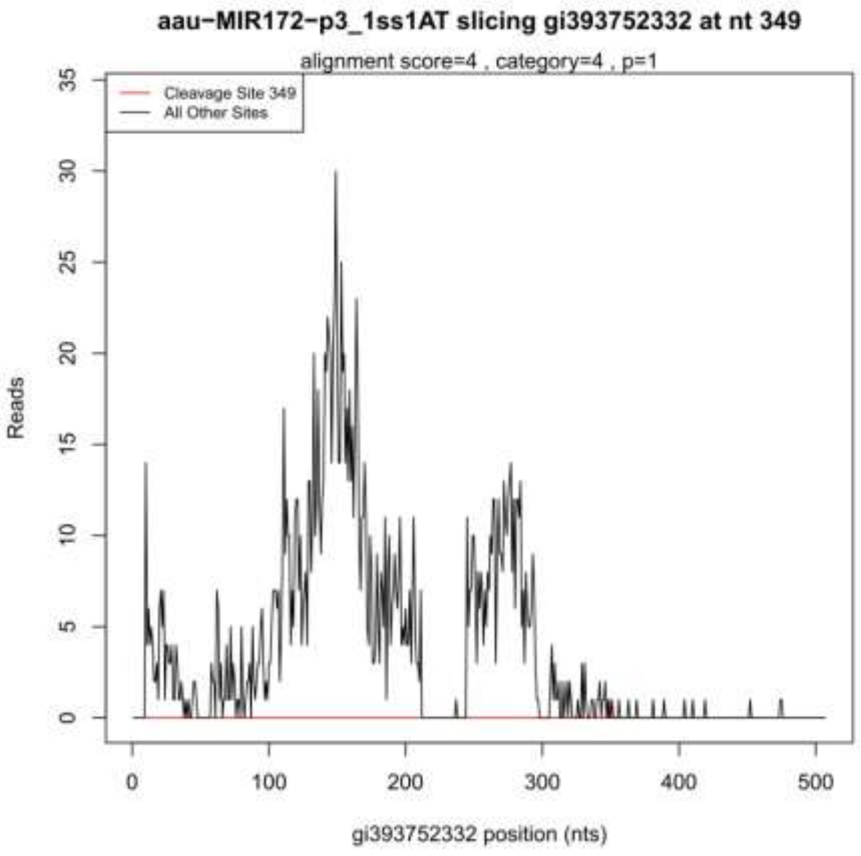

aau−MIR172−p3_1ss1AT slicing gi393752332 at nt 349
alignment score=4 , category=4 , p=1
Cleavage Site 349
All Other Sites
Reads
0
5
10
15
20
25
30
35
0
100
200
300
400
500
gi393752332 position (nts)

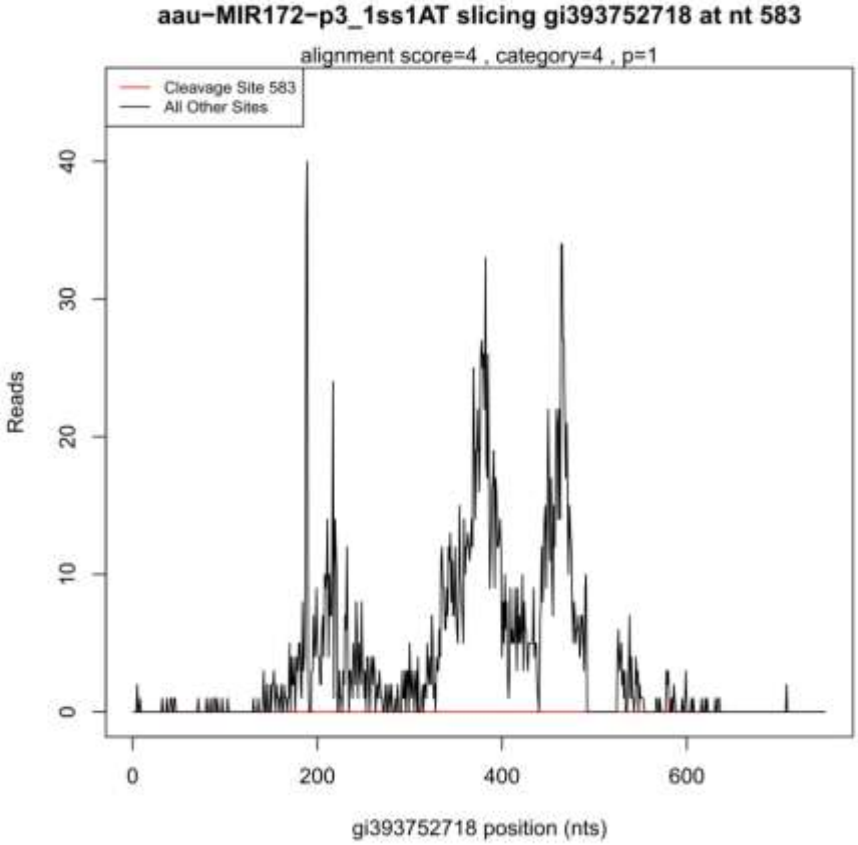
aau−MIR172−p3_1ss1AT slicing gi393752718 at nt 583
alignment score=4 , category=4 , p=1
Cleavage Site 583
All Other Sites
Reads
0
10
20
30
40
0
200
400
600
gi393752718 position (nts)

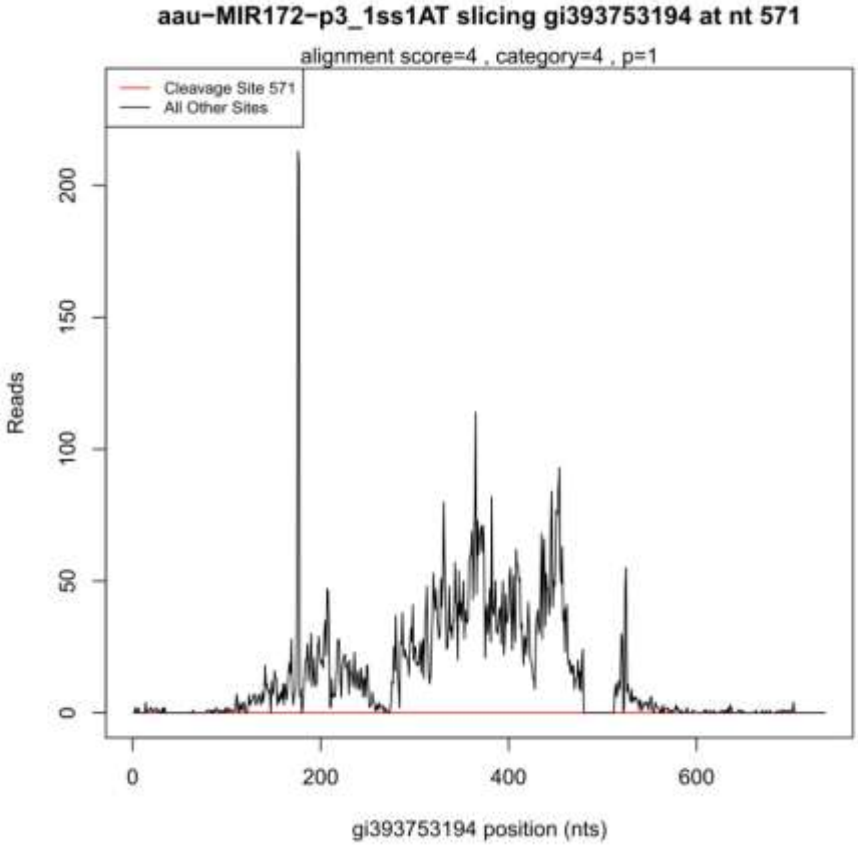
aau−MIR172−p3_1ss1AT slicing gi393753194 at nt 571
alignment score=4 , category=4 , p=1
Cleavage Site 571
All Other Sites
Reads
0
50
100
150
200
0
200
400
600
gi393753194 position (nts)

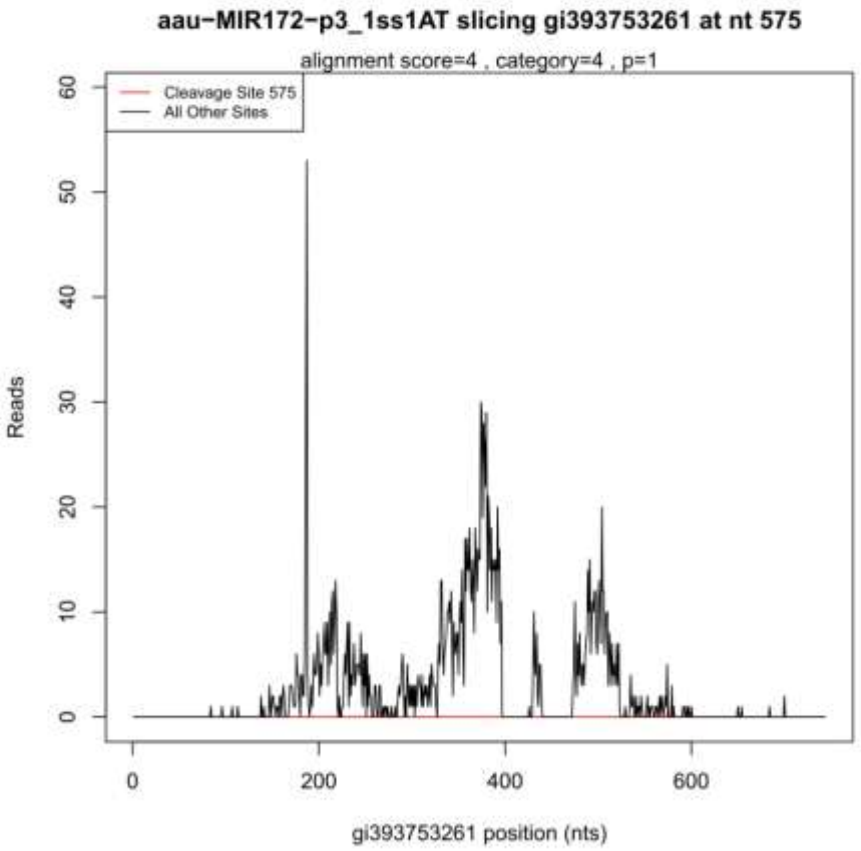
aau−MIR172−p3_1ss1AT slicing gi393753261 at nt 575
alignment score=4 , category=4 , p=1
Cleavage Site 575
All Other Sites
Reads
0
10
20
30
40
50
60
0
200
400
600
gi393753261 position (nts)

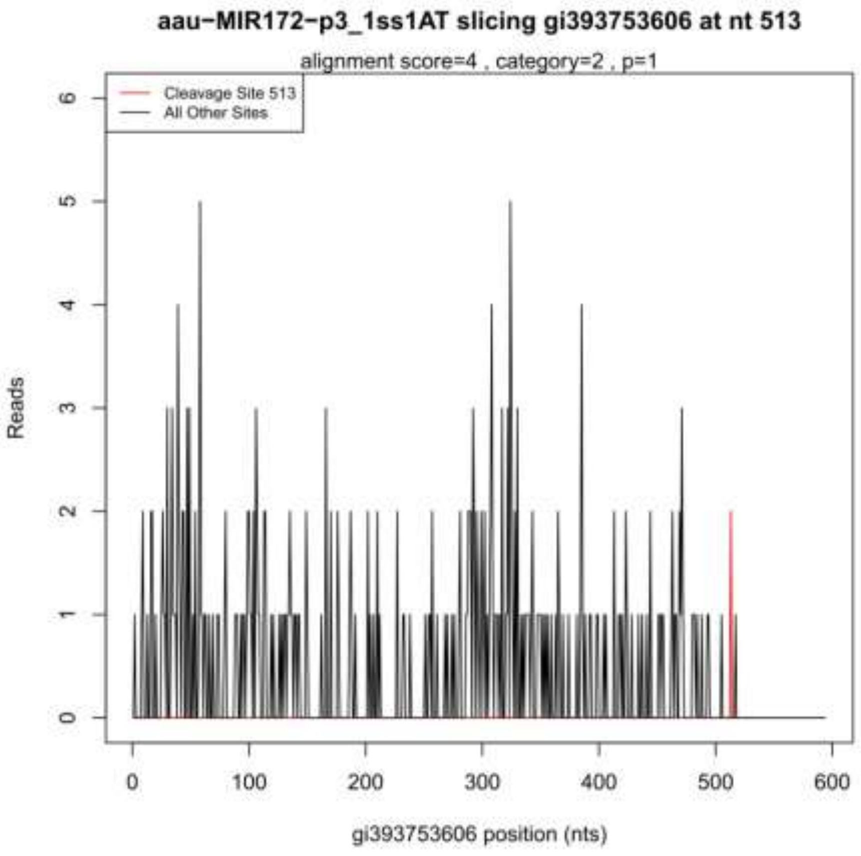
aau−MIR172−p3_1ss1AT slicing gi393753606 at nt 513
alignment score=4 , category=2 , p=1
Cleavage Site 513
All Other Sites
Reads
0
1
2
3
4
5
6
0
100
200
300
400
500
600
gi393753606 position (nts)

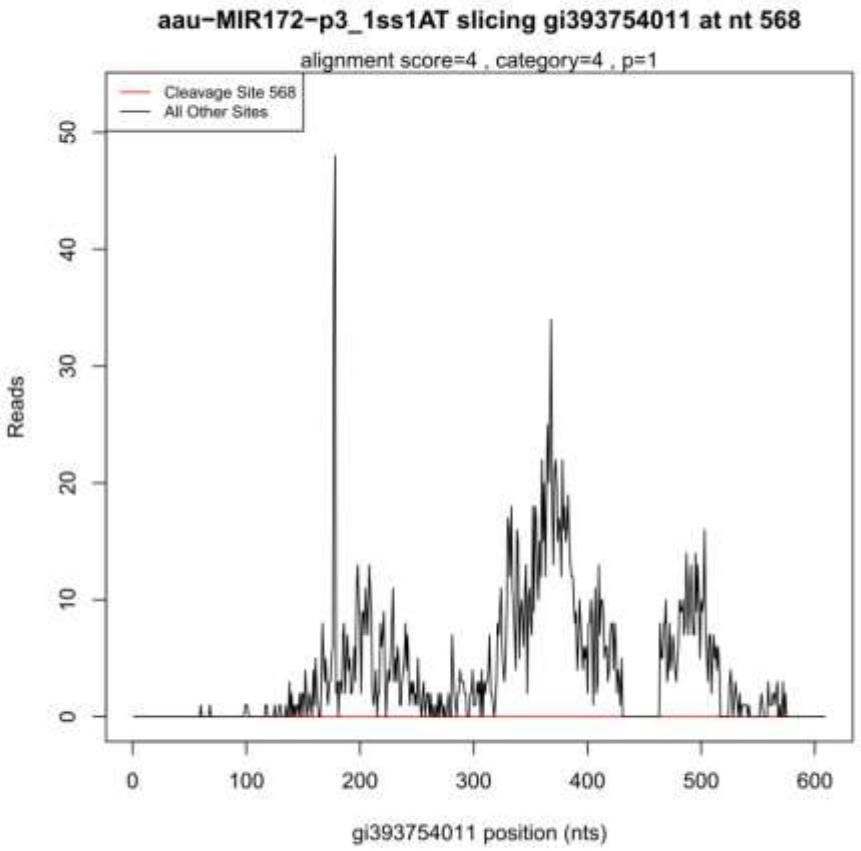
aau−MIR172−p3_1ss1AT slicing gi393754011 at nt 568
alignment score=4 , category=4 , p=1
Cleavage Site 568
All Other Sites
Reads
0
10
20
30
40
50
0
100
200
300
400
500
600
gi393754011 position (nts)

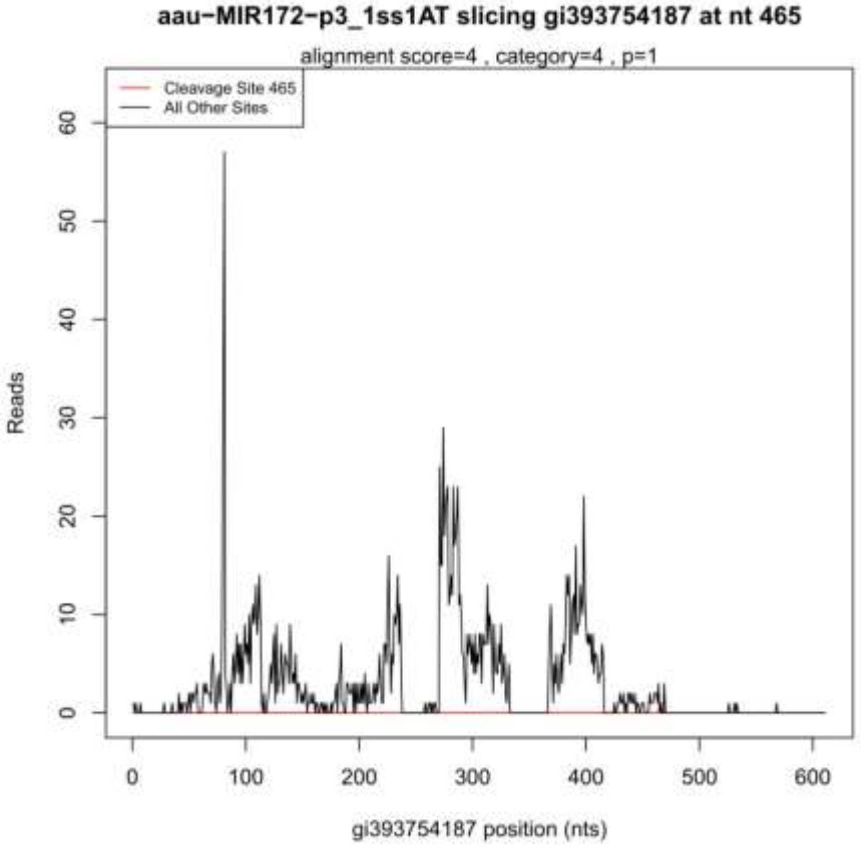
aau−MIR172−p3_1ss1AT slicing gi393754187 at nt 465
alignment score=4 , category=4 , p=1
Cleavage Site 465
All Other Sites
Reads
0
10
20
30
40
50
60
0
100
200
300
400
500
600
gi393754187 position (nts)

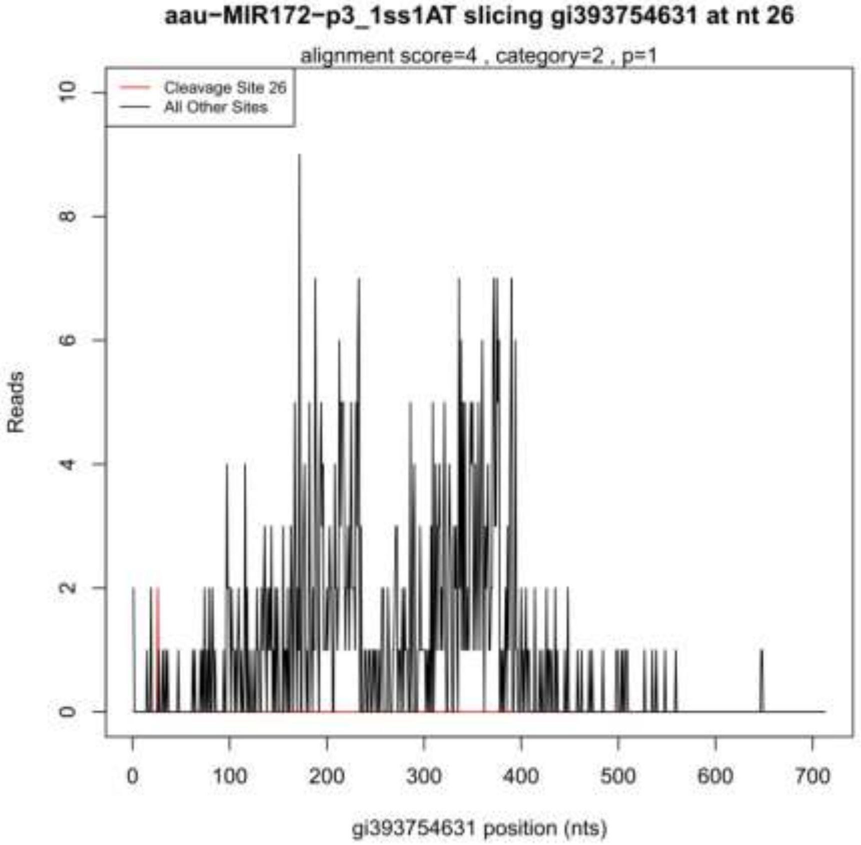

aau-MIR172-p3_1ss1AT slicing gi393754631 at nt 26
alignment score=4 , category=2 , p=1
Cleavage Site 26
All Other Sites
Reads
0
2
4
6
8
10
0
100
200
300
400
500
600
700
gi393754631 position (nts)

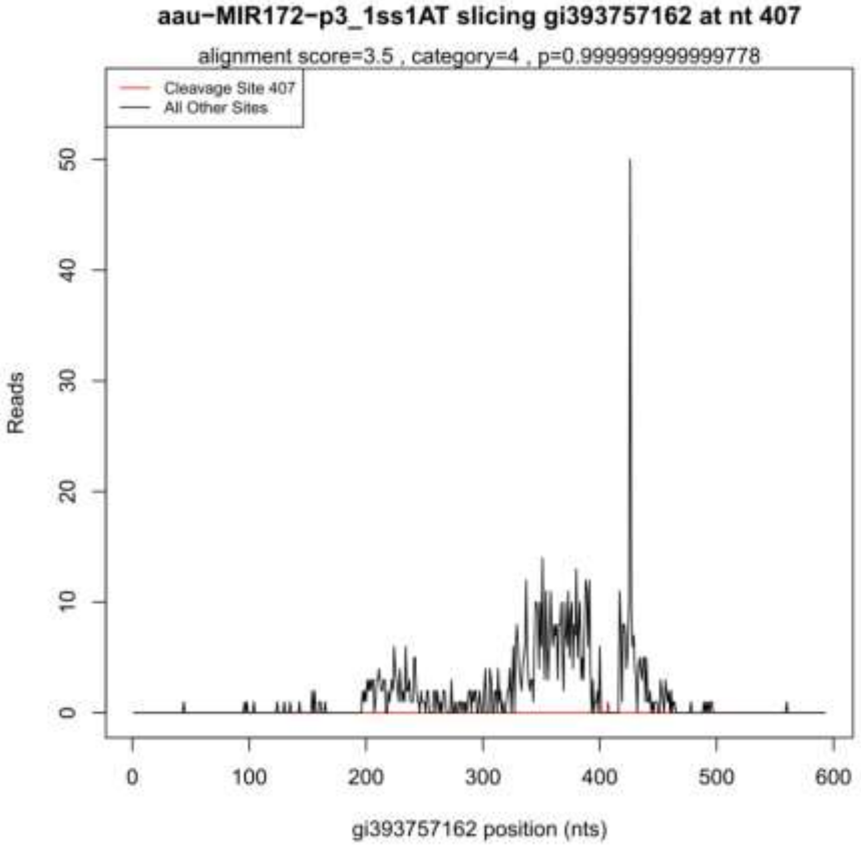

aau-MIR172-p3_1ss1AT slicing gi393757162 at nt 407
alignment score=3.5 , category=4 , p=0.999999999999778
Cleavage Site 407
All Other Sites
Reads
0
10
20
30
40
50
0
100
200
300
400
500
600
gi393757162 position (nts)

ptc−MIR319b−p5_1ss10TA slicing gi51452848 at nt 259

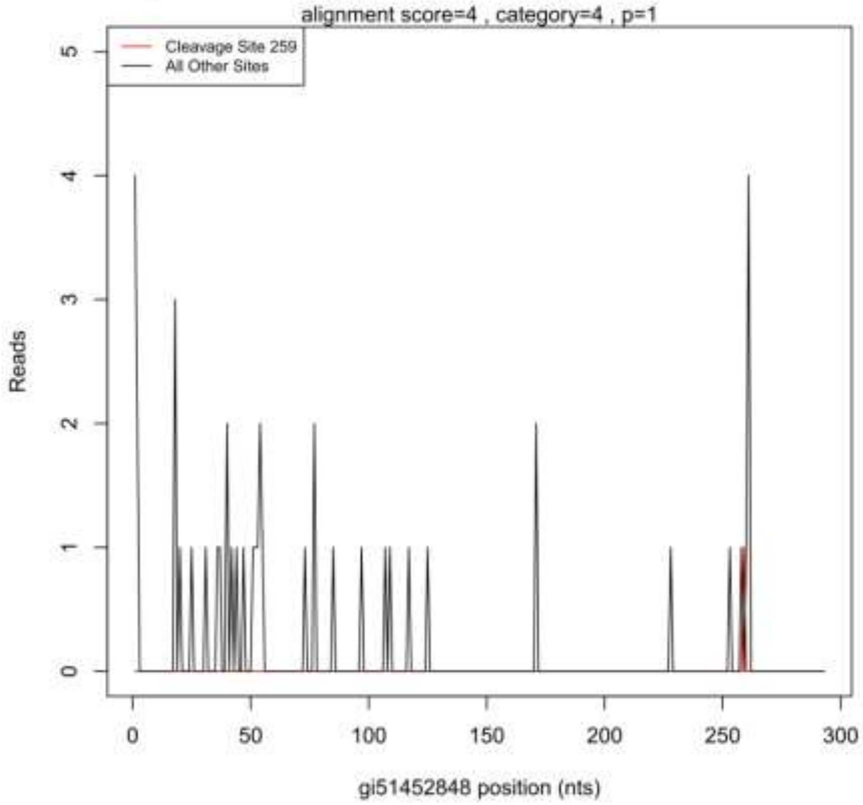

ptc−MIR319b−p5_1ss10TA slicing gi51452849 at nt 140

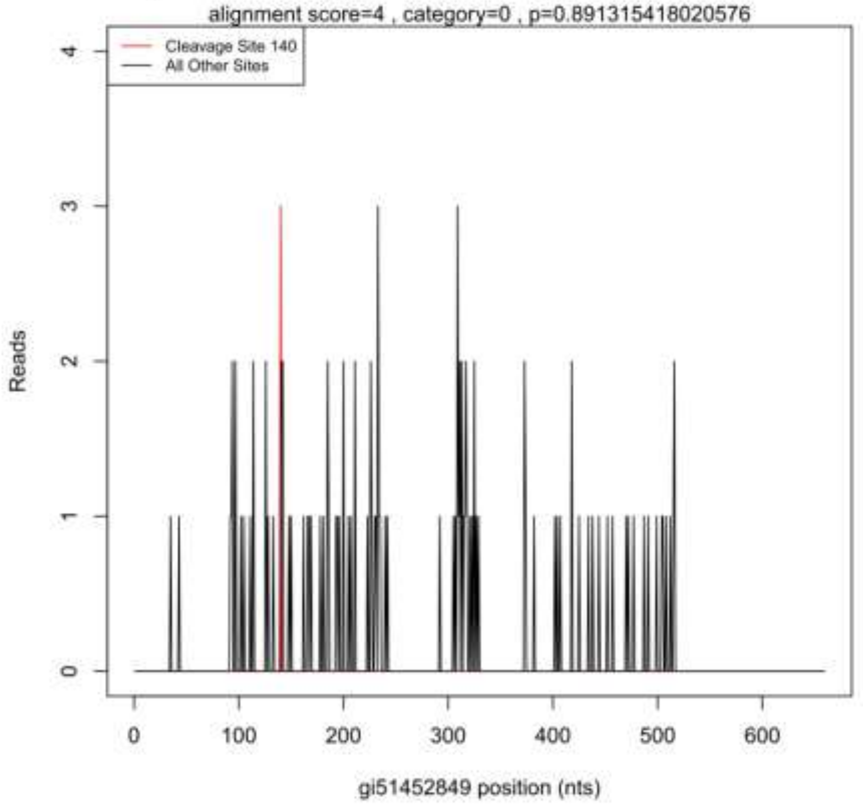

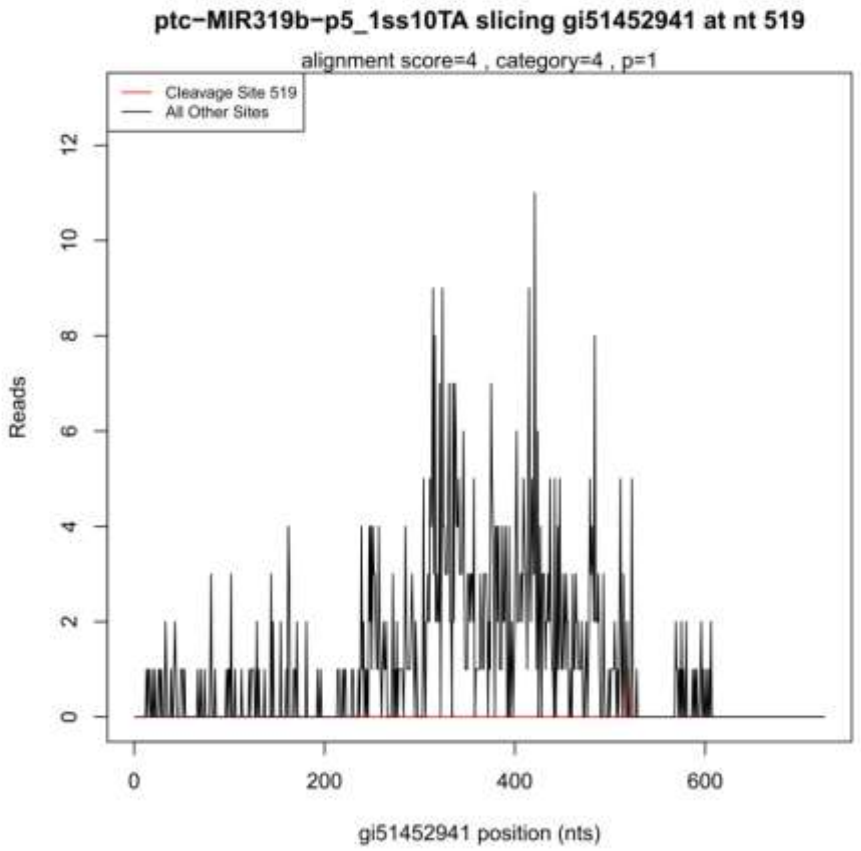
ptc−MIR319b−p5_1ss10TA slicing gi51452941 at nt 519
alignment score=4 , category=4 , p=1
Cleavage Site 519
All Other Sites
Reads
0
2
4
6
8
10
12
0
200
400
600
gi51452941 position (nts)

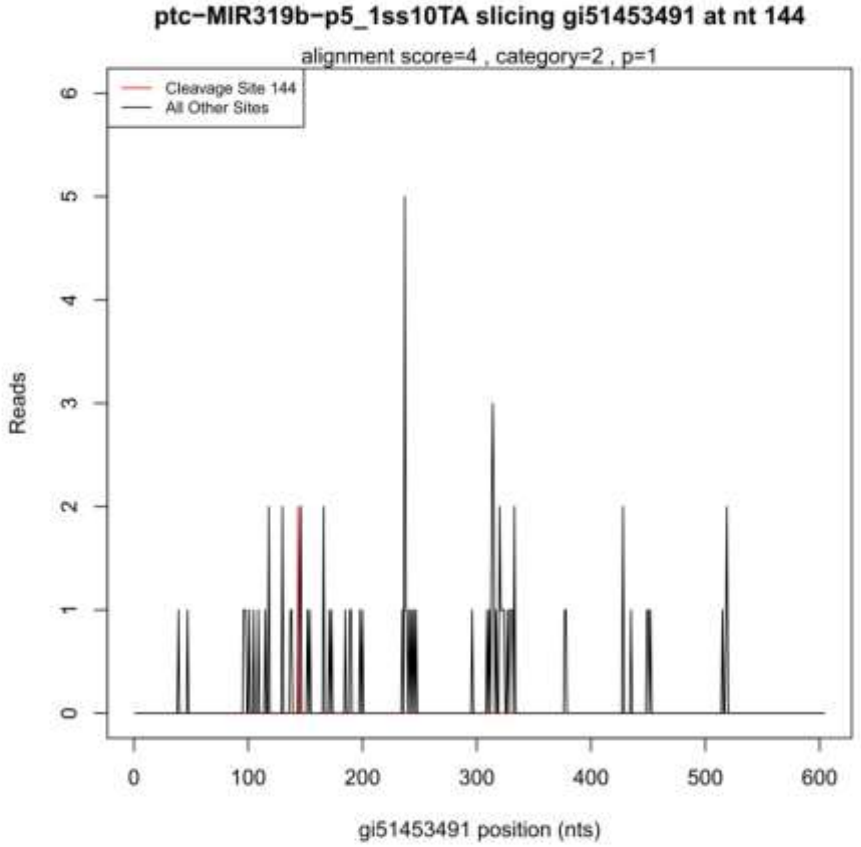
ptc−MIR319b−p5_1ss10TA slicing gi51453491 at nt 144
alignment score=4 , category=2 , p=1
Cleavage Site 144
All Other Sites
Reads
0
1
2
3
4
5
6
0
100
200
300
400
500
600
gi51453491 position (nts)

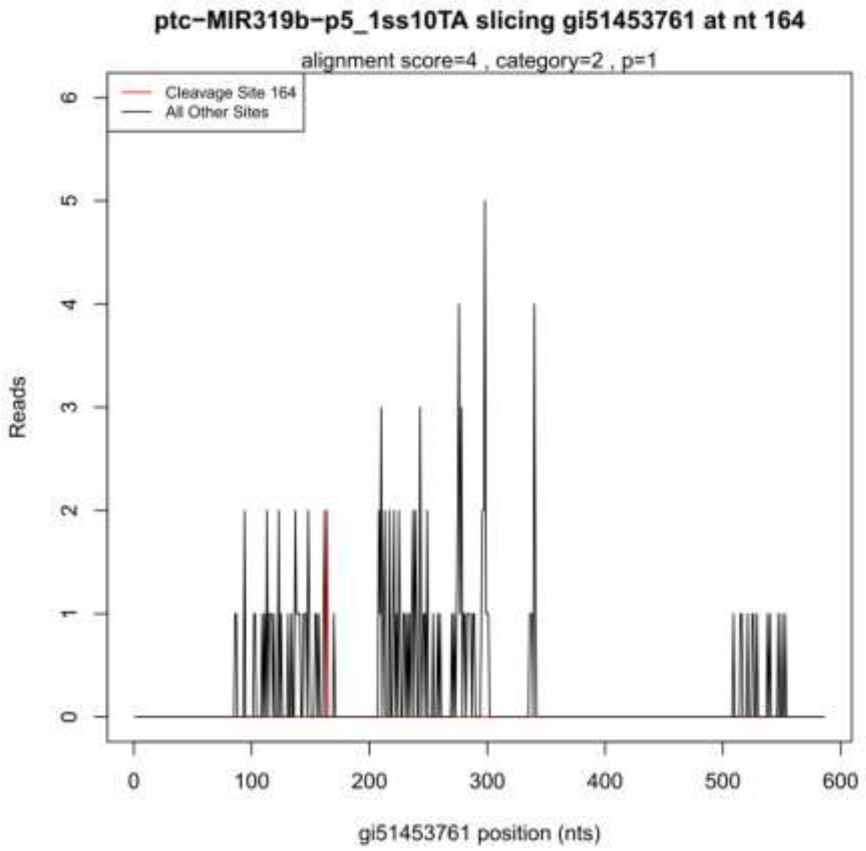

ptc-MIR319b-p5_1ss10TA slicing gi51453761 at nt 164
alignment score=4 , category=2 , p=1
Cleavage Site 164
All Other Sites
Reads
0
1
2
3
4
5
6
0
100
200
300
400
500
600
gi51453761 position (nts)

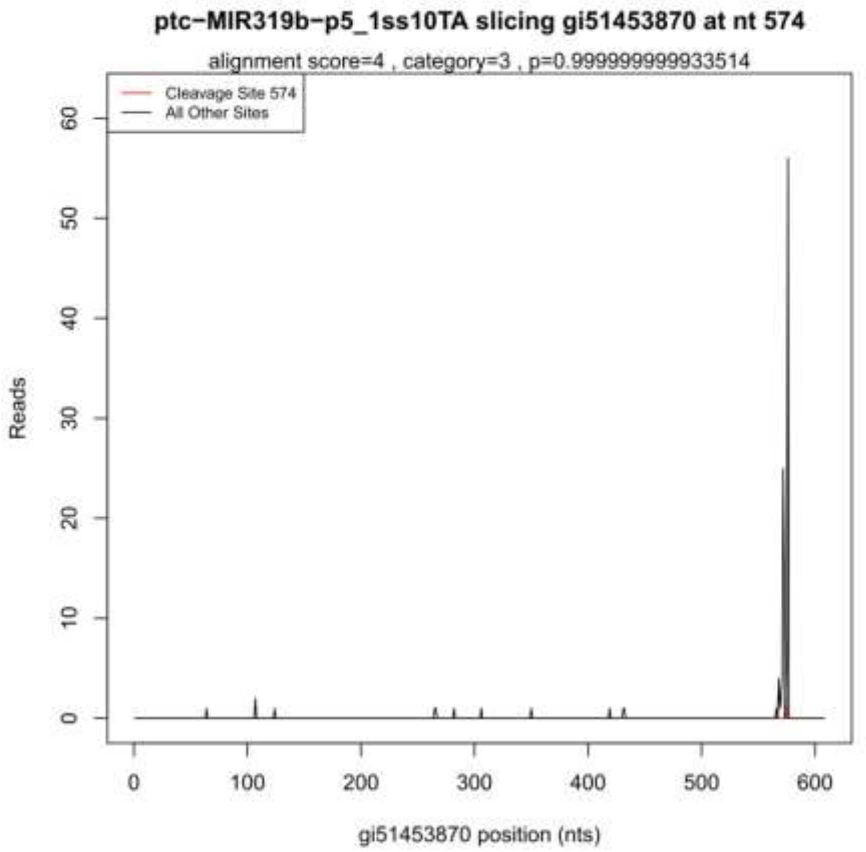

ptc-MIR319b-p5_1ss10TA slicing gi51453870 at nt 574
alignment score=4 , category=3 , p=0.999999999933514
Cleavage Site 574
All Other Sites
Reads
0
10
20
30
40
50
60
0
100
200
300
400
500
600
gi51453870 position (nts)

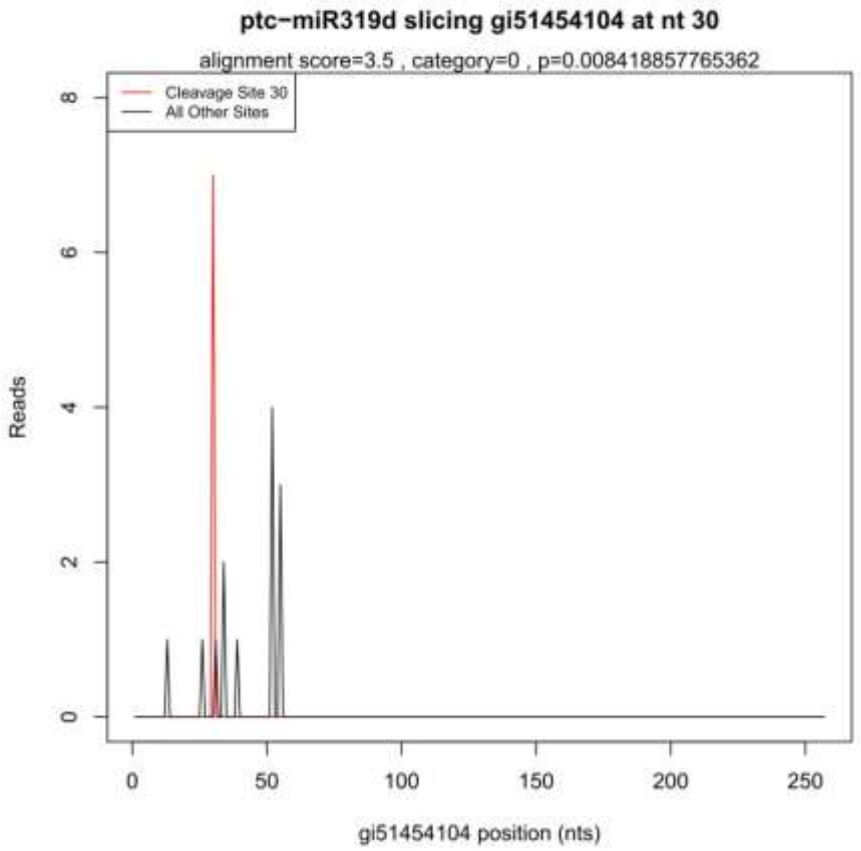
ptc−miR319d slicing gi51454104 at nt 30
alignment score=3.5 , category=0 , p=0.008418857765362
Cleavage Site 30
All Other Sites
Reads
0
2
4
6
8
0
50
100
150
200
250
gi51454104 position (nts)

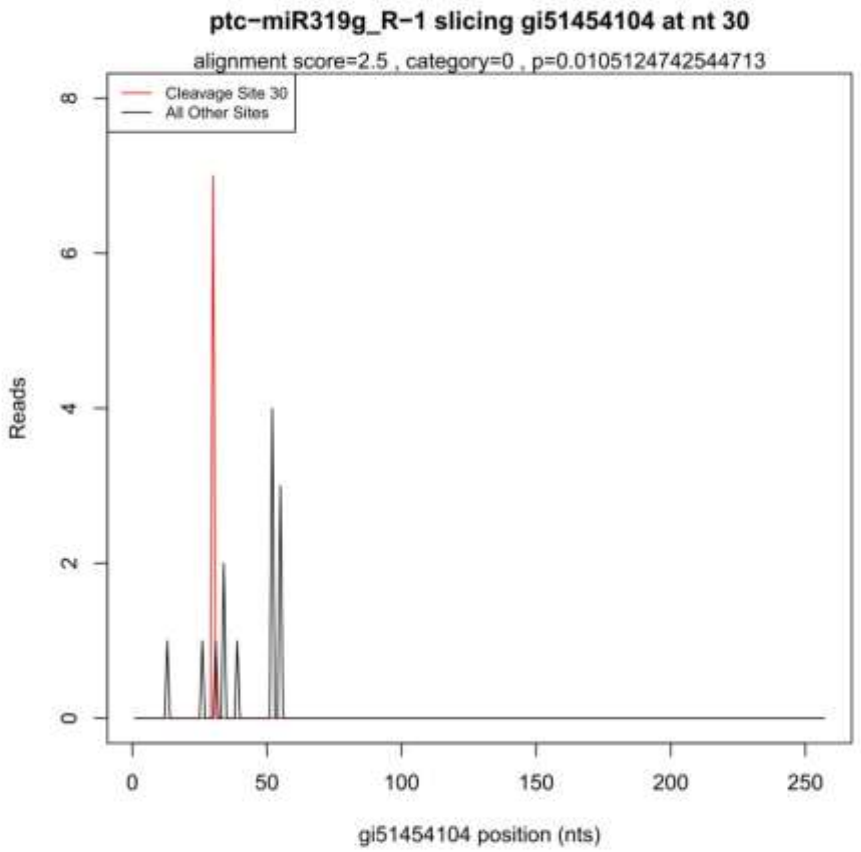
ptc−miR319g_R−1 slicing gi51454104 at nt 30
alignment score=2.5 , category=0 , p=0.0105124742544713
Cleavage Site 30
All Other Sites
Reads
0
2
4
6
8
0
50
100
150
200
250
gi51454104 position (nts)

ptc−MIR319b−p5_1ss10TA slicing gi51530166 at nt 542

alignment score=4 , category=4 , p=1

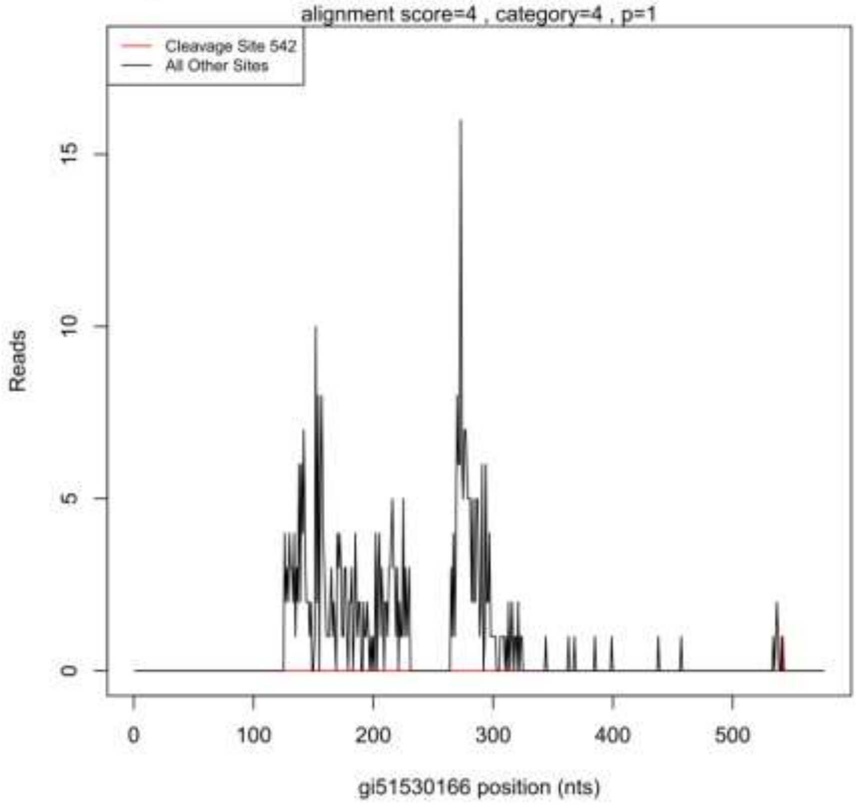

ptc−MIR319b−p5_1ss10TA slicing gi170319482 at nt 124

alignment score=4 , category=4 , p=1

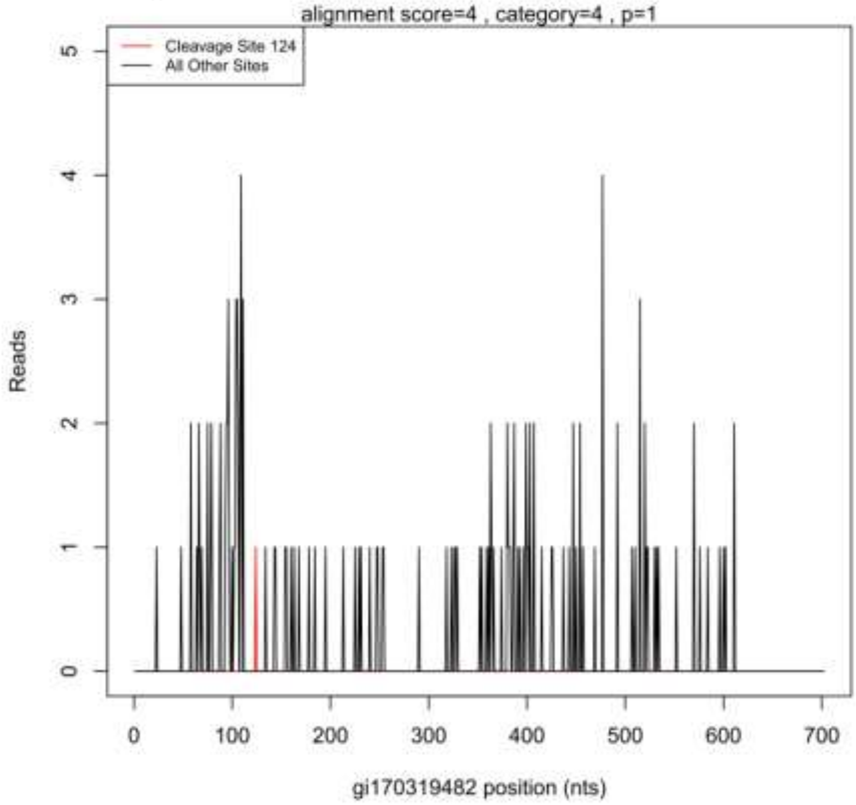

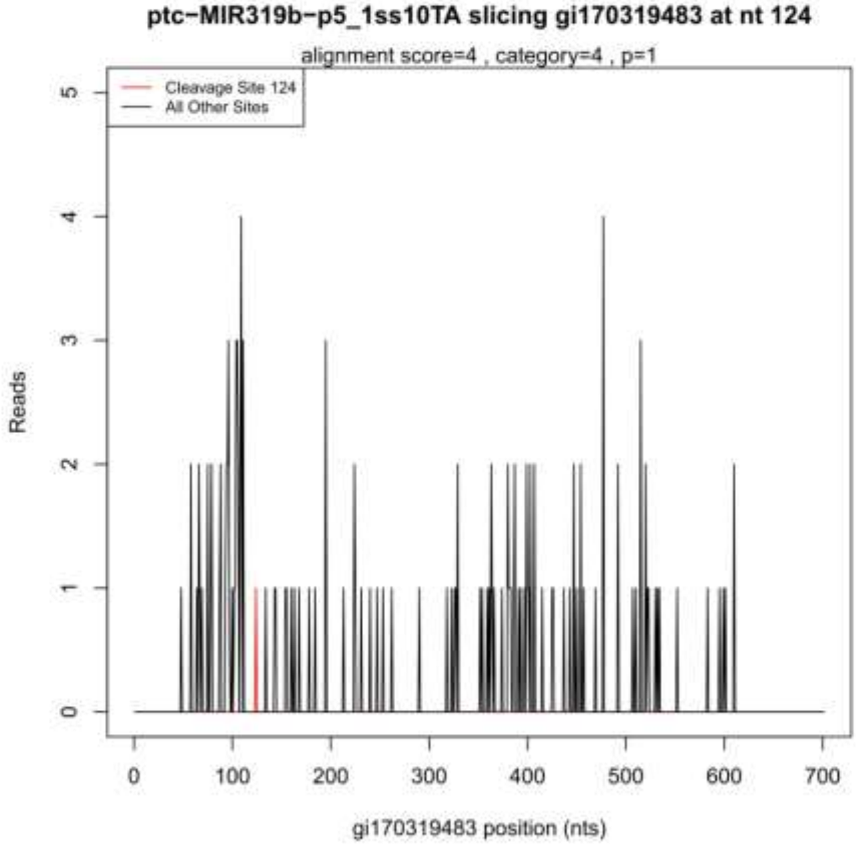
ptc−MIR319b−p5_1ss10TA slicing gi170319483 at nt 124
alignment score=4 , category=4 , p=1
Cleavage Site 124
All Other Sites
Reads
0
1
2
3
4
5
0
100
200
300
400
500
600
700
gi170319483 position (nts)

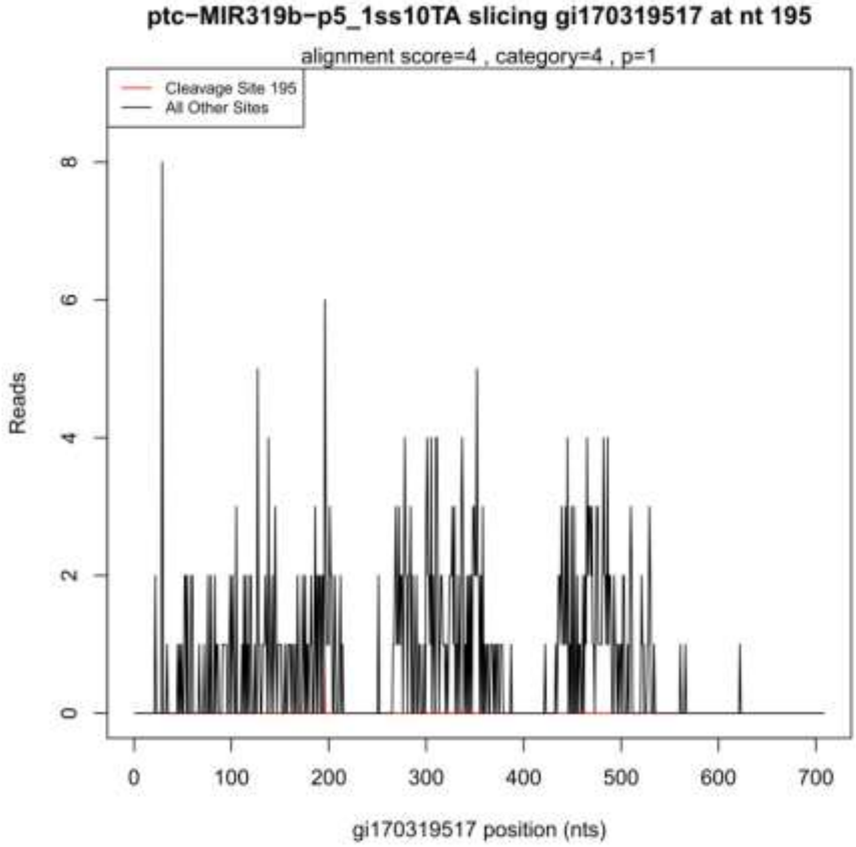
ptc−MIR319b−p5_1ss10TA slicing gi170319517 at nt 195
alignment score=4 , category=4 , p=1
Cleavage Site 195
All Other Sites
Reads
0
2
4
6
8
0
100
200
300
400
500
600
700
gi170319517 position (nts)

ptc−MIR319b−p5_1ss10TA slicing gi170319622 at nt 172

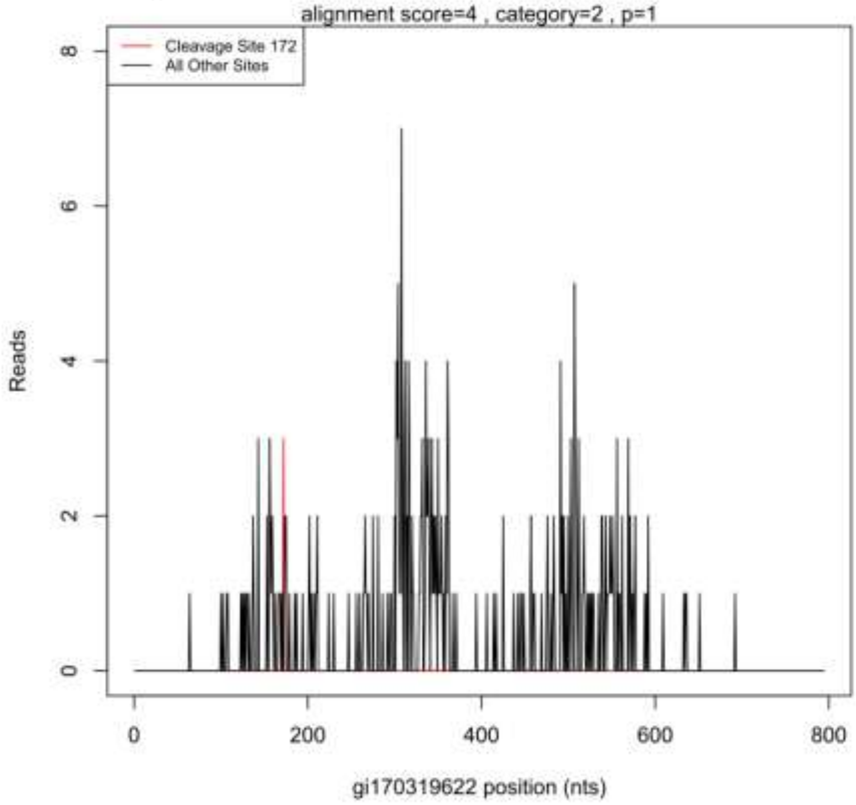

ptc−MIR319b−p5_1ss10TA slicing gi170319718 at nt 181

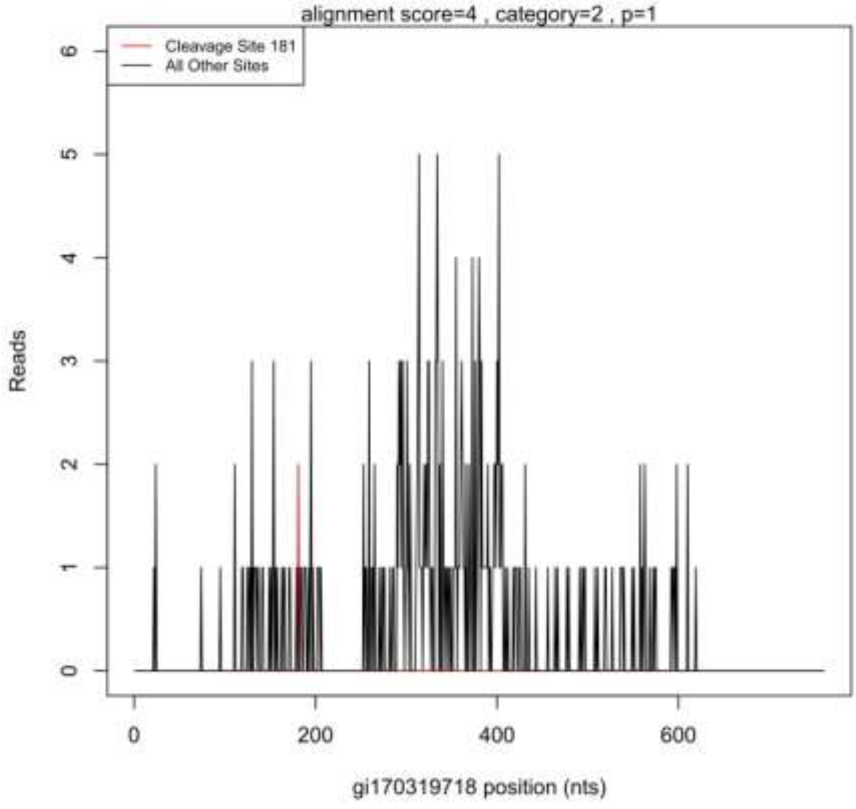

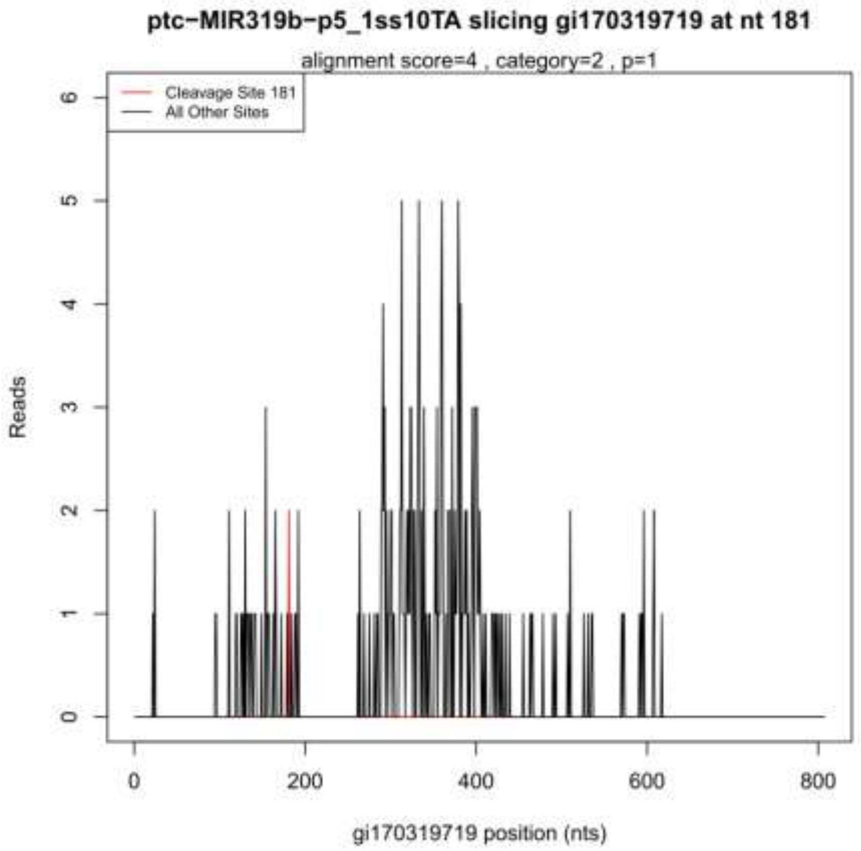
ptc−MIR319b−p5_1ss10TA slicing gi170319719 at nt 181
alignment score=4 , category=2 , p=1
Cleavage Site 181
All Other Sites
Reads
0
1
2
3
4
5
6
0
200
400
600
800
gi170319719 position (nts)

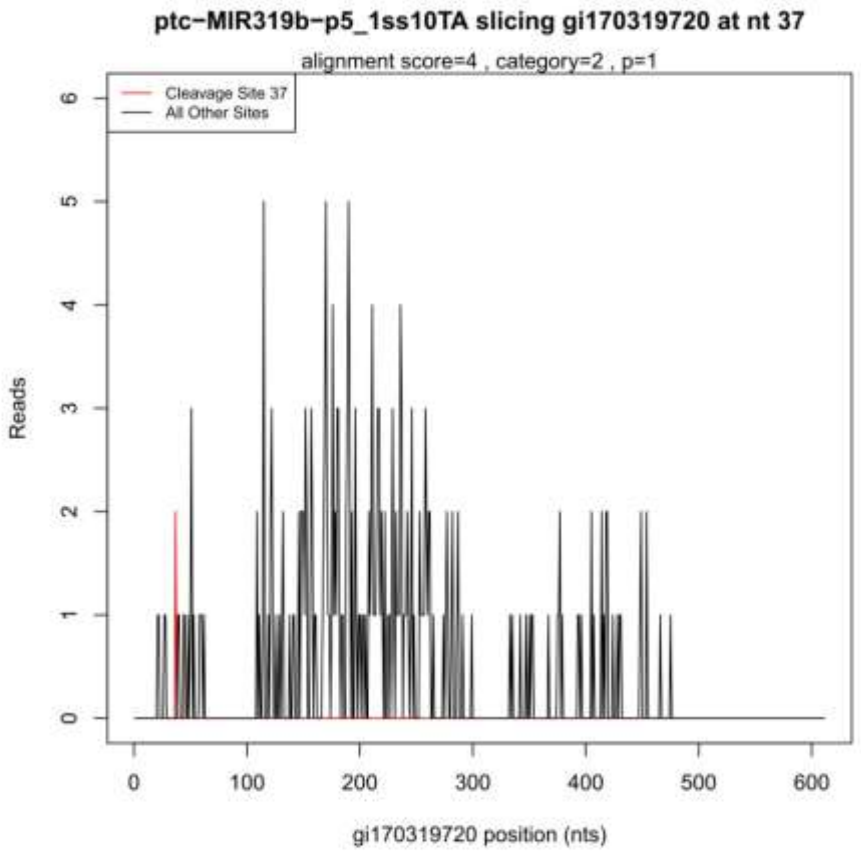
ptc−MIR319b−p5_1ss10TA slicing gi170319720 at nt 37
alignment score=4 , category=2 , p=1
Cleavage Site 37
All Other Sites
Reads
0
1
2
3
4
5
6
0
100
200
300
400
500
600
gi170319720 position (nts)

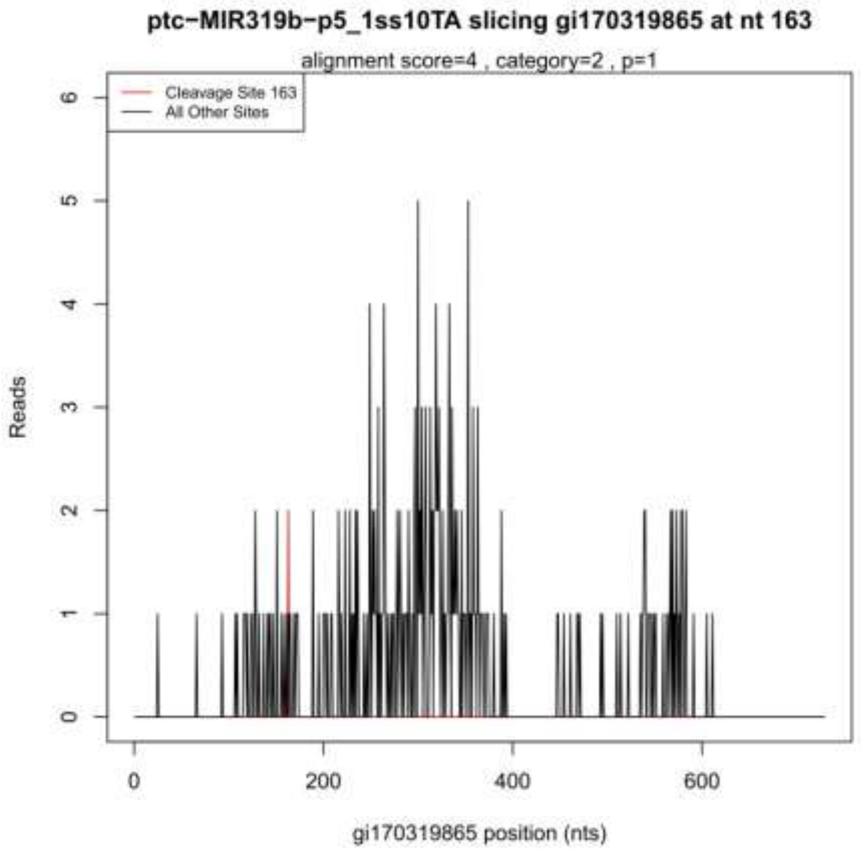

ptc−MIR319b−p5_1ss10TA slicing gi170319865 at nt 163
alignment score=4 , category=2 , p=1
Cleavage Site 163
All Other Sites
Reads
gi170319865 position (nts)

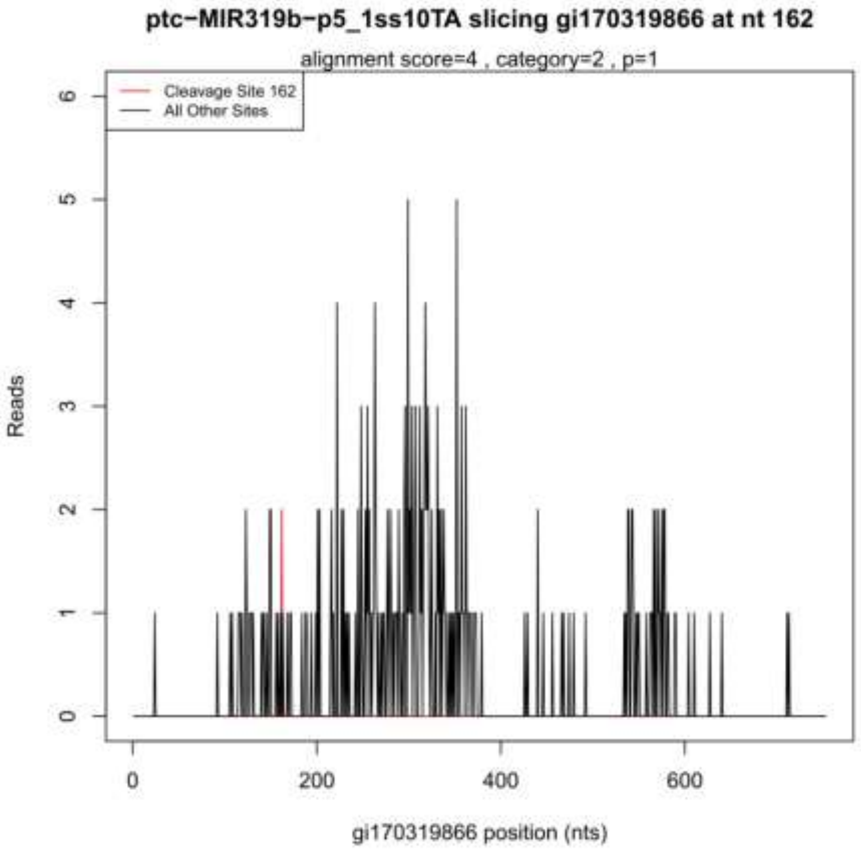

ptc−MIR319b−p5_1ss10TA slicing gi170319866 at nt 162
alignment score=4 , category=2 , p=1
Cleavage Site 162
All Other Sites
Reads
gi170319866 position (nts)

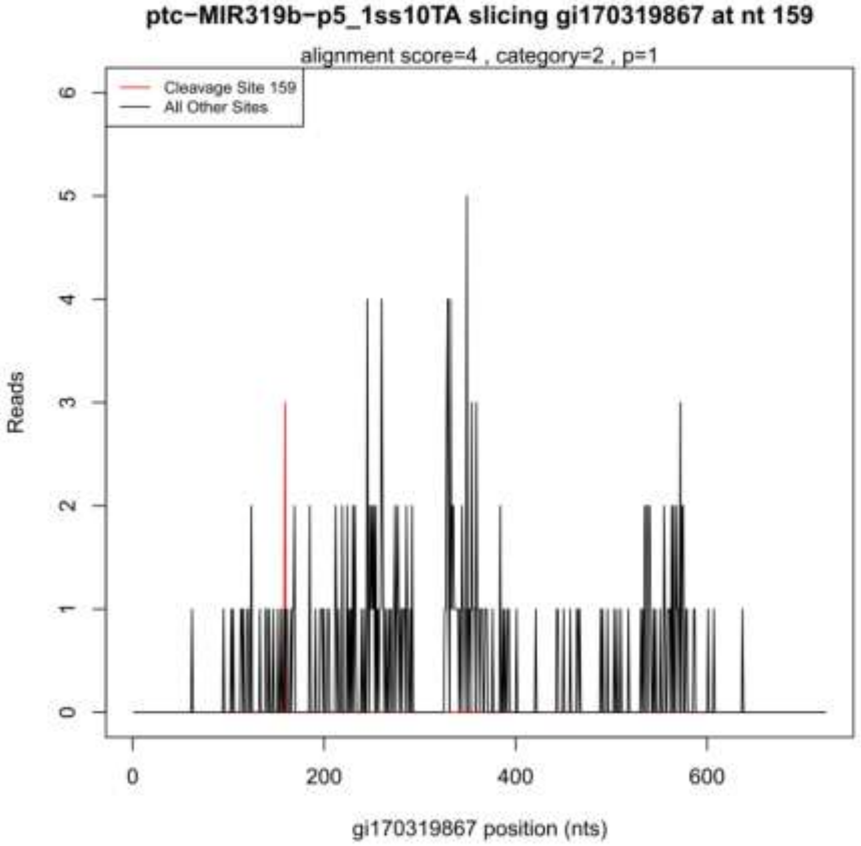

ptc−MIR319b−p5_1ss10TA slicing gi170319867 at nt 159
alignment score=4 , category=2 , p=1
Cleavage Site 159
All Other Sites
Reads
0
1
2
3
4
5
6
0
200
400
600
gi170319867 position (nts)

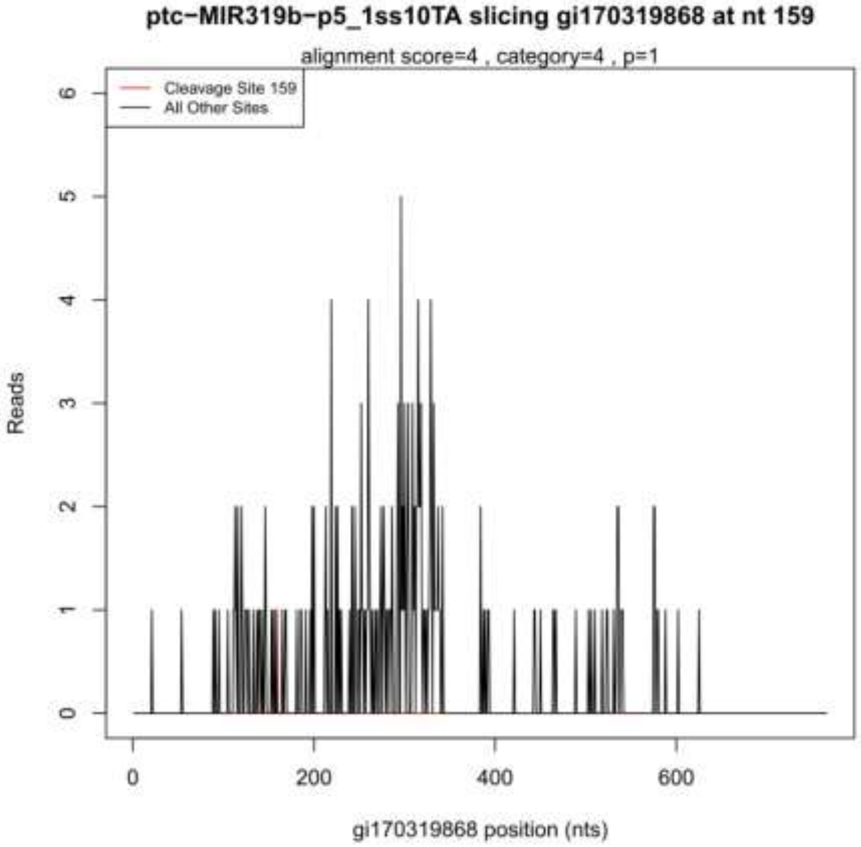

ptc−MIR319b−p5_1ss10TA slicing gi170319868 at nt 159
alignment score=4 , category=4 , p=1
Cleavage Site 159
All Other Sites
Reads
0
1
2
3
4
5
6
0
200
400
600
gi170319868 position (nts)

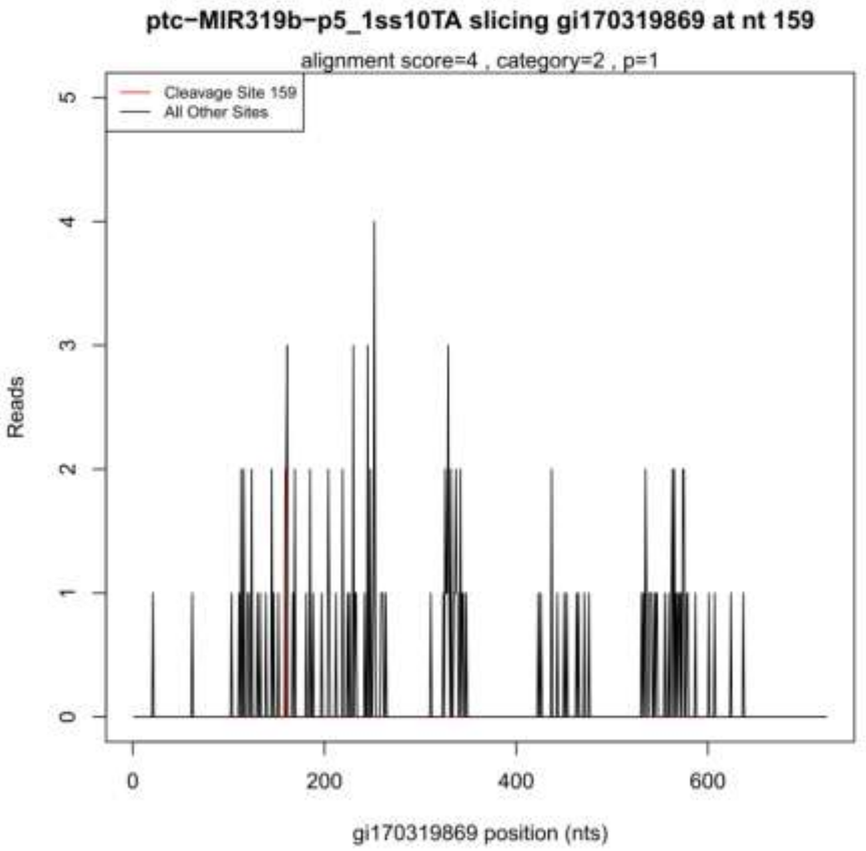
ptc−MIR319b−p5_1ss10TA slicing gi170319869 at nt 159
alignment score=4 , category=2 , p=1
Cleavage Site 159
All Other Sites
Reads
gi170319869 position (nts)

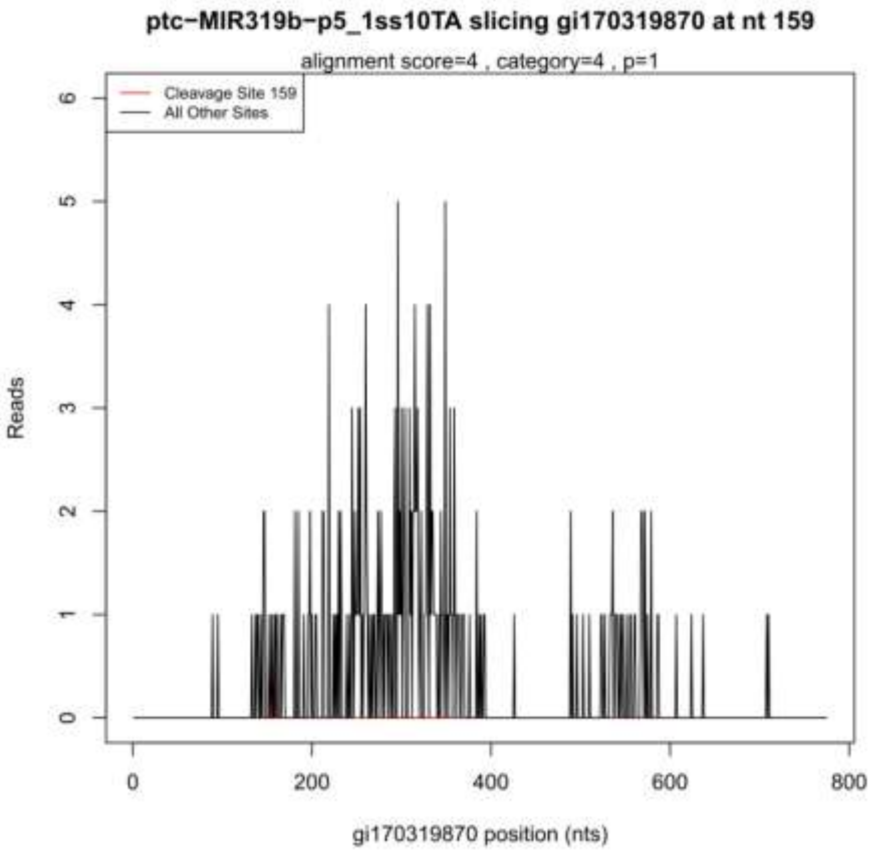
ptc−MIR319b−p5_1ss10TA slicing gi170319870 at nt 159
alignment score=4 , category=4 , p=1
Cleavage Site 159
All Other Sites
Reads
gi170319870 position (nts)

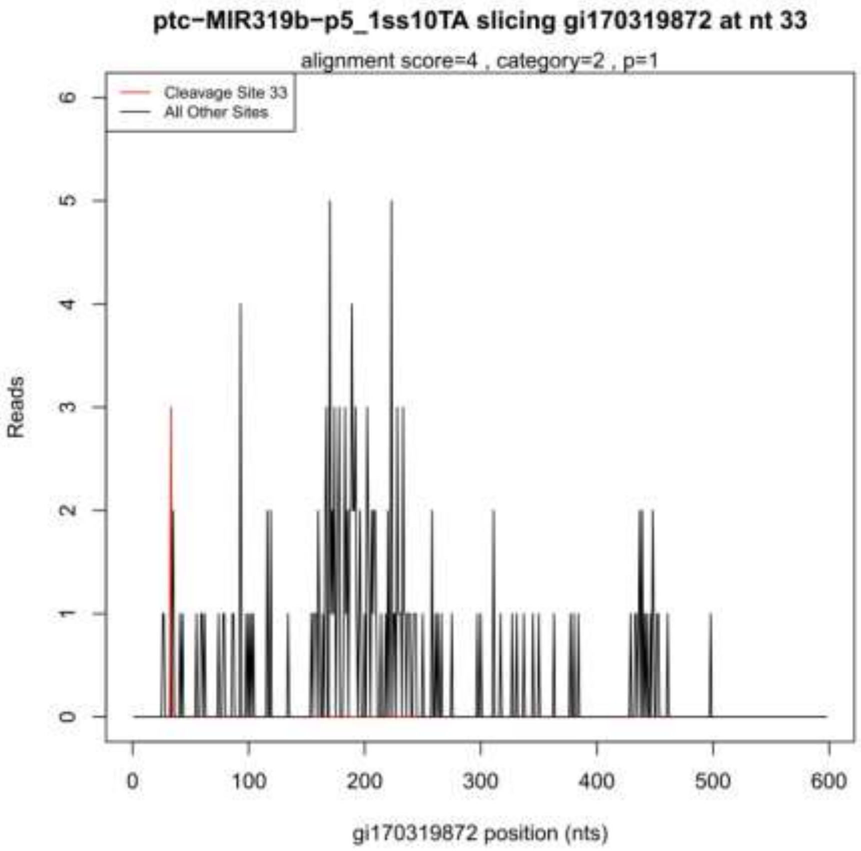

ptc-MIR319b-p5_1ss10TA slicing gi170319872 at nt 33
alignment score=4 , category=2 , p=1
Cleavage Site 33
All Other Sites
Reads
0
1
2
3
4
5
6
0
100
200
300
400
500
600
gi170319872 position (nts)

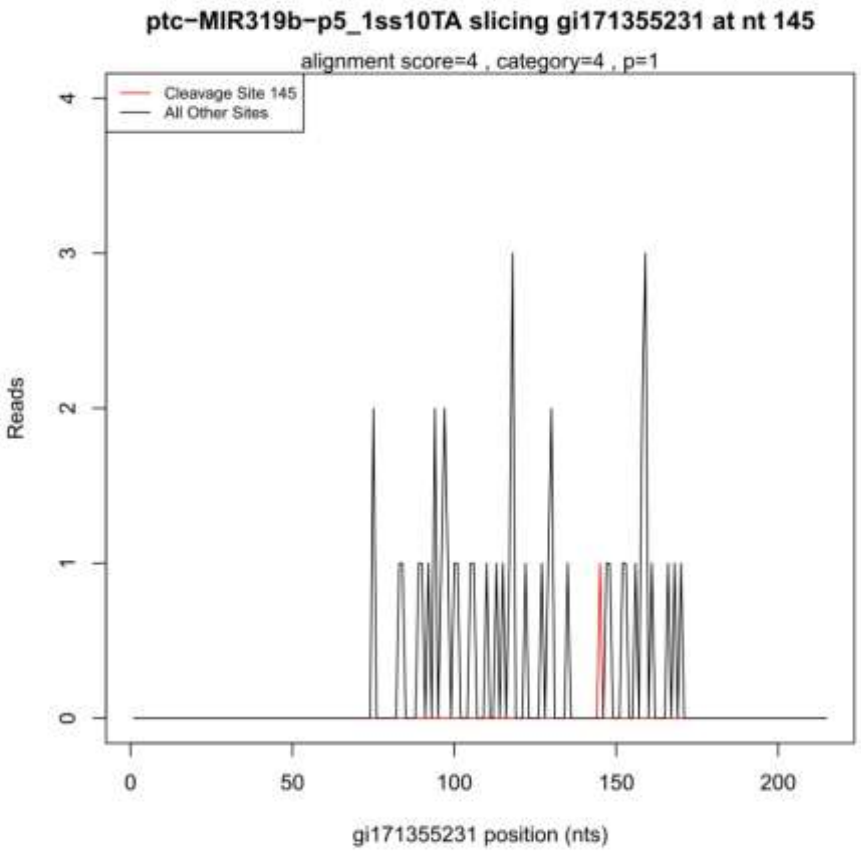

ptc-MIR319b-p5_1ss10TA slicing gi171355231 at nt 145
alignment score=4 , category=4 , p=1
Cleavage Site 145
All Other Sites
Reads
0
1
2
3
4
0
50
100
150
200
gi171355231 position (nts)

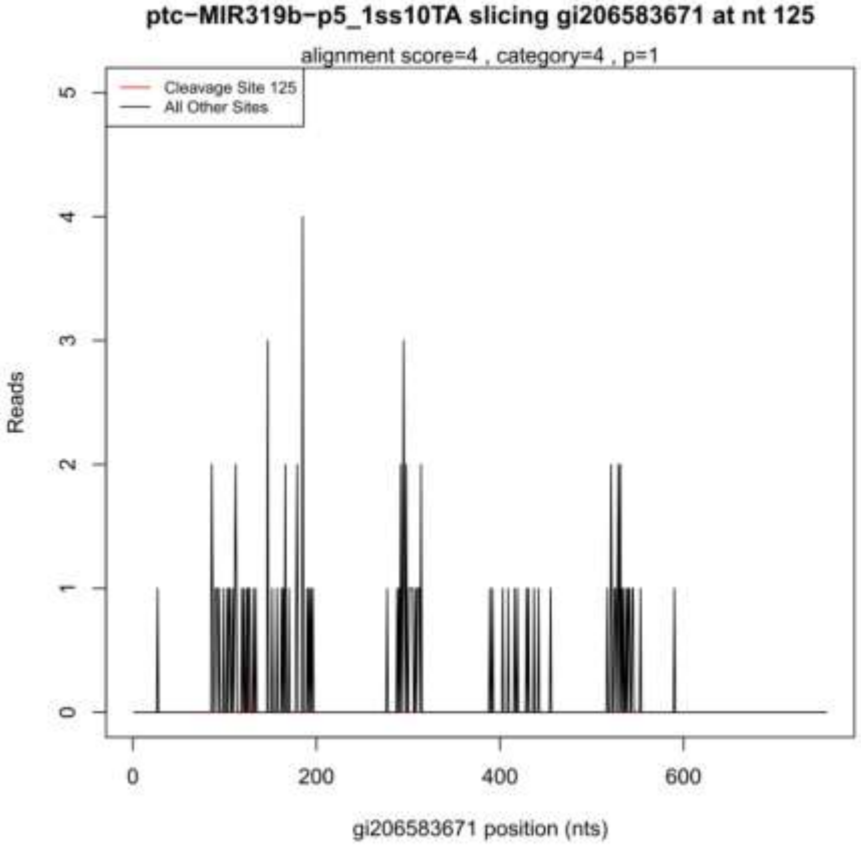

ptc−MIR319b−p5_1ss10TA slicing gi206583671 at nt 125
alignment score=4 , category=4 , p=1
Cleavage Site 125
All Other Sites
Reads
0
1
2
3
4
5
0
200
400
600
gi206583671 position (nts)

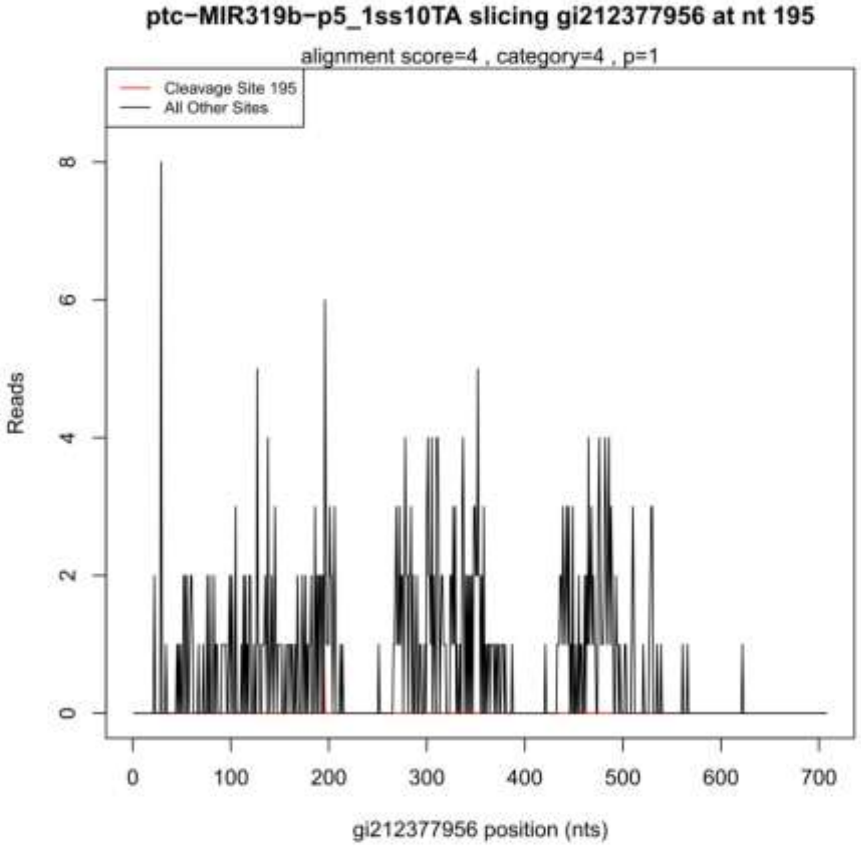

ptc−MIR319b−p5_1ss10TA slicing gi212377956 at nt 195
alignment score=4 , category=4 , p=1
Cleavage Site 195
All Other Sites
Reads
0
2
4
6
8
0
100
200
300
400
500
600
700
gi212377956 position (nts)

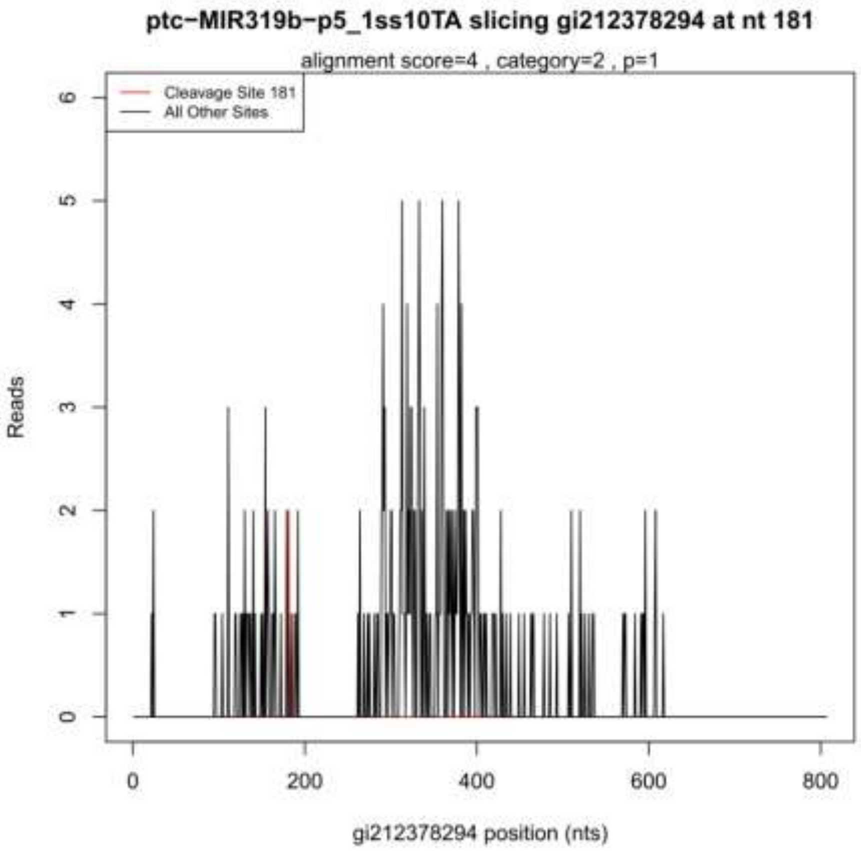
ptc-MIR319b-p5_1ss10TA slicing gi212378294 at nt 181
alignment score=4 , category=2 , p=1
Cleavage Site 181
All Other Sites
Reads
0
1
2
3
4
5
6
0
200
400
600
800
gi212378294 position (nts)

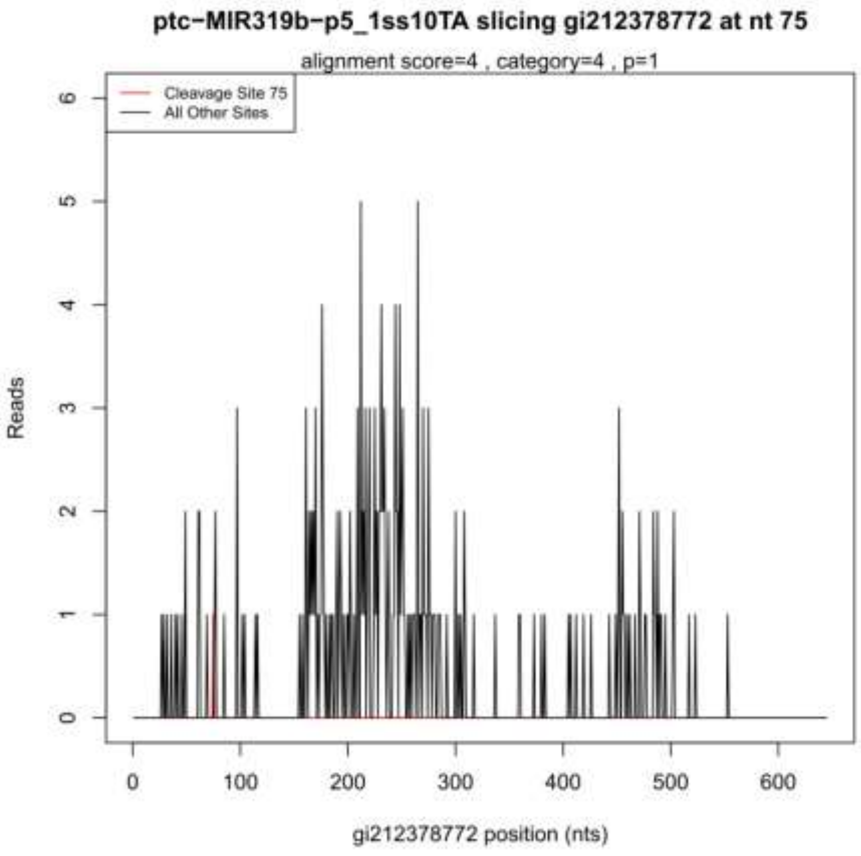
ptc-MIR319b-p5_1ss10TA slicing gi212378772 at nt 75
alignment score=4 , category=4 , p=1
Cleavage Site 75
All Other Sites
Reads
0
1
2
3
4
5
6
0
100
200
300
400
500
600
gi212378772 position (nts)

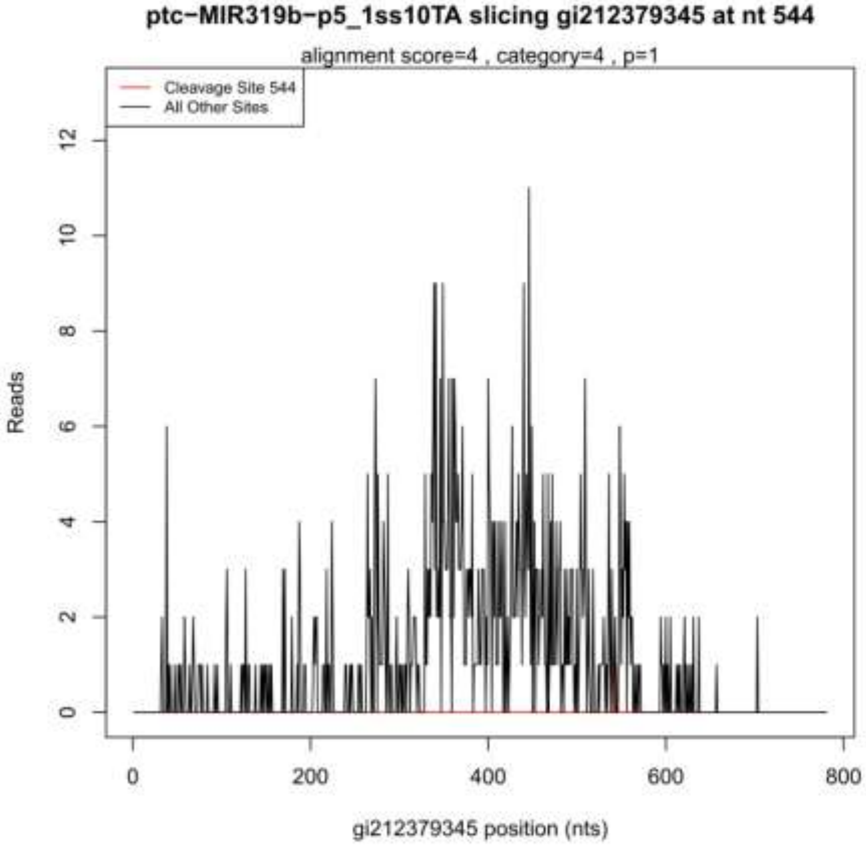
ptc−MIR319b−p5_1ss10TA slicing gi212379345 at nt 544
alignment score=4 , category=4 , p=1
Cleavage Site 544
All Other Sites
Reads
gi212379345 position (nts)

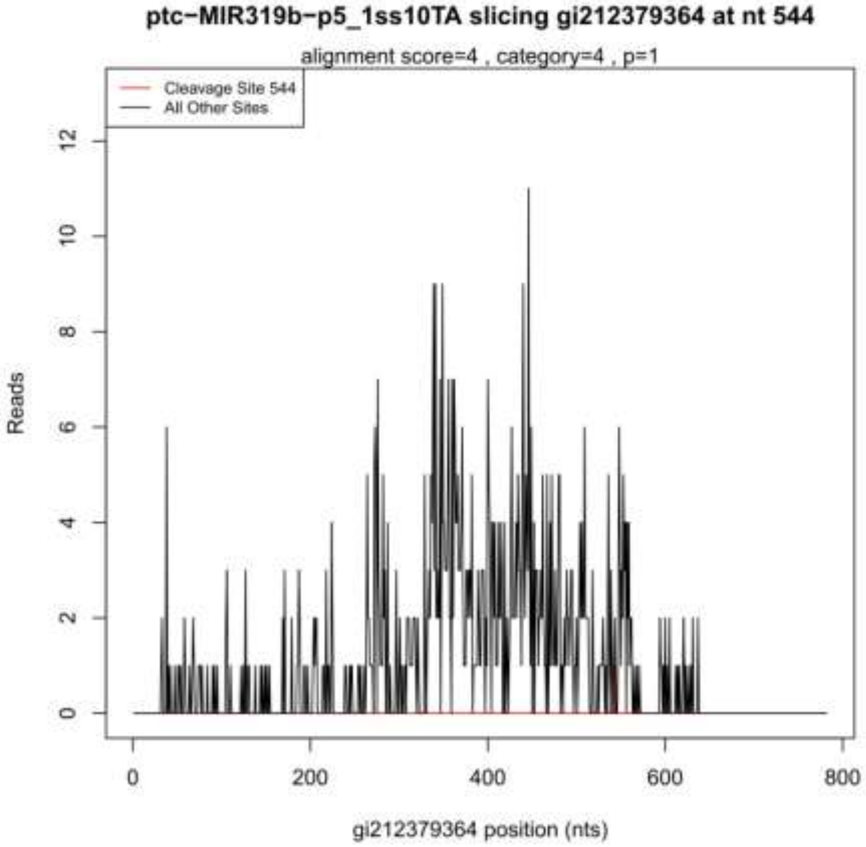
ptc−MIR319b−p5_1ss10TA slicing gi212379364 at nt 544
alignment score=4 , category=4 , p=1
Cleavage Site 544
All Other Sites
Reads
gi212379364 position (nts)

ptc−MIR319b−p5_1ss10TA slicing gi212379569 at nt 228

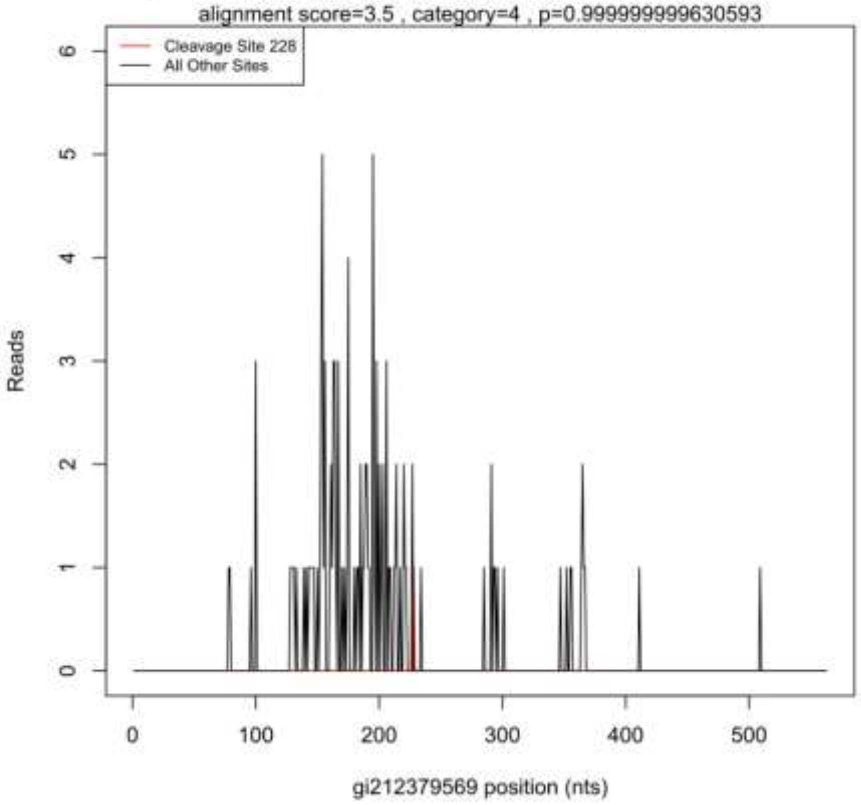

ptc−MIR319b−p5_1ss10TA slicing gi212380194 at nt 124

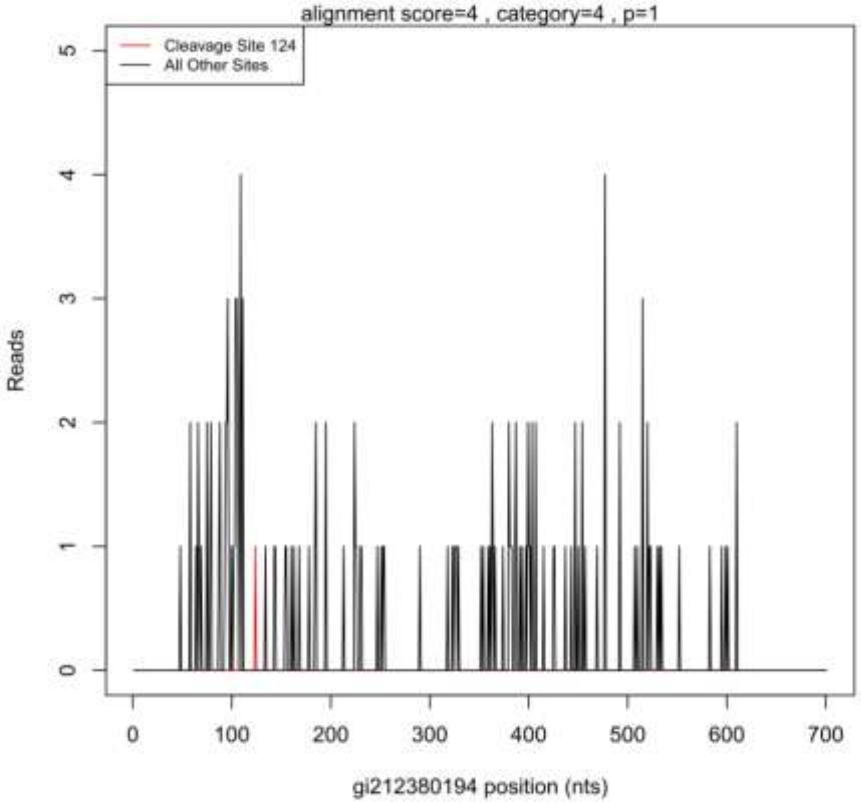

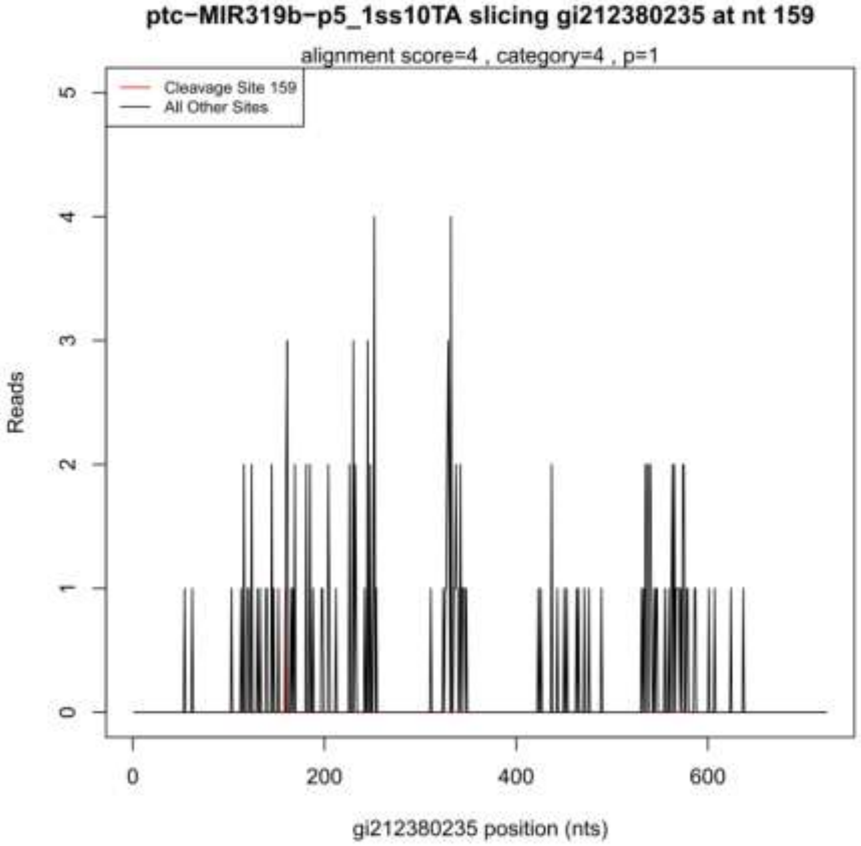

ptc−MIR319b−p5_1ss10TA slicing gi212380235 at nt 159
alignment score=4 , category=4 , p=1
Cleavage Site 159
All Other Sites
Reads
0
1
2
3
4
5
0
200
400
600
gi212380235 position (nts)

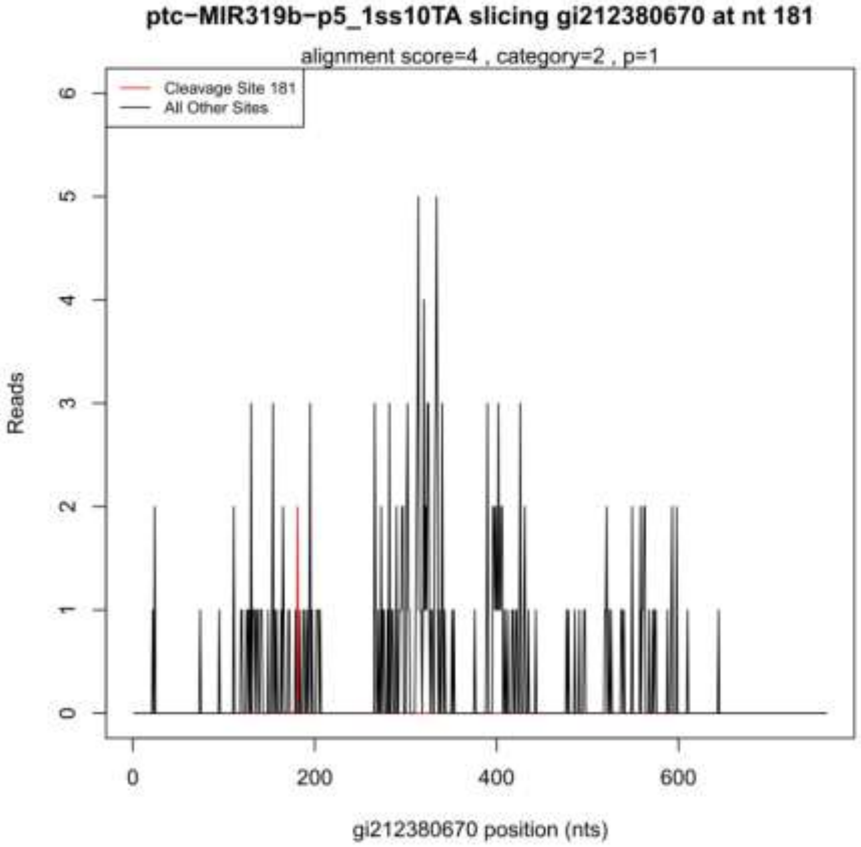

ptc−MIR319b−p5_1ss10TA slicing gi212380670 at nt 181
alignment score=4 , category=2 , p=1
Cleavage Site 181
All Other Sites
Reads
0
1
2
3
4
5
6
0
200
400
600
gi212380670 position (nts)

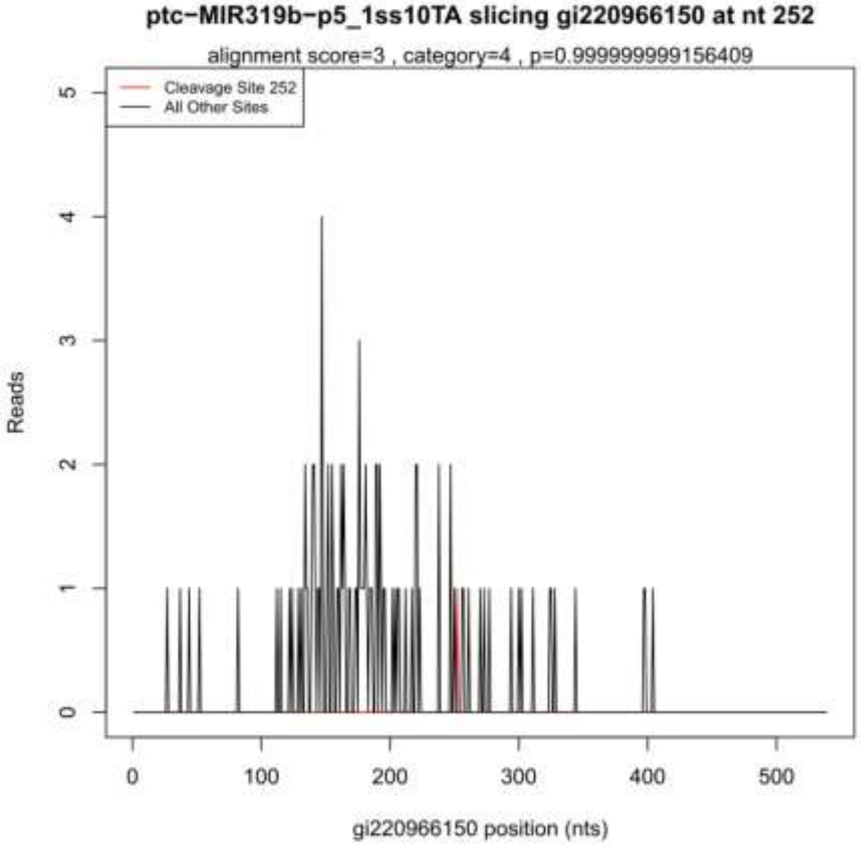

ptc−MIR319b−p5_1ss10TA slicing gi220966150 at nt 252
alignment score=3 , category=4 , p=0.999999999156409
Cleavage Site 252
All Other Sites
Reads
0
1
2
3
4
5
0
100
200
300
400
500
gi220966150 position (nts)

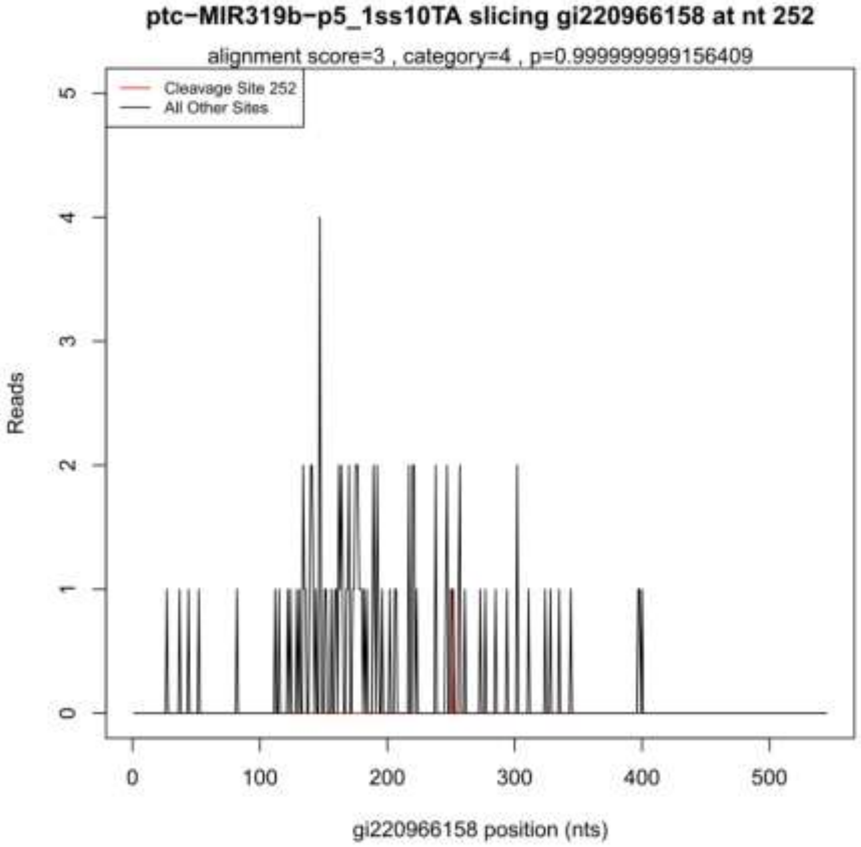

ptc−MIR319b−p5_1ss10TA slicing gi220966158 at nt 252
alignment score=3 , category=4 , p=0.999999999156409
Cleavage Site 252
All Other Sites
Reads
0
1
2
3
4
5
0
100
200
300
400
500
gi220966158 position (nts)

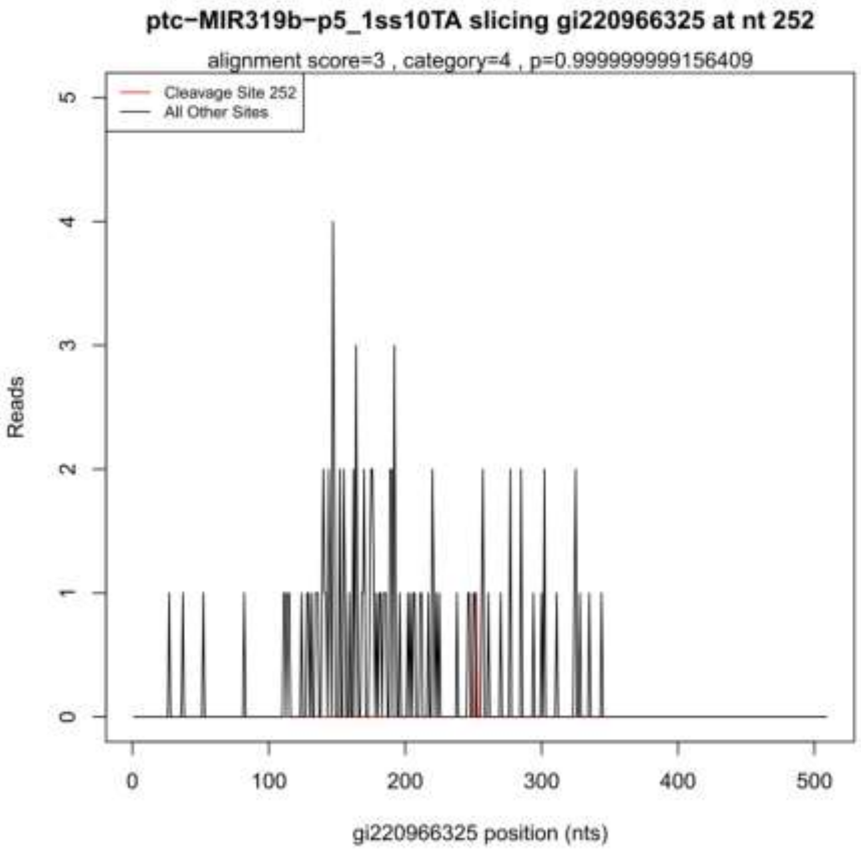
ptc−MIR319b−p5_1ss10TA slicing gi220966325 at nt 252
alignment score=3 , category=4 , p=0.999999999156409
Cleavage Site 252
All Other Sites
Reads
0
1
2
3
4
5
0
100
200
300
400
500
gi220966325 position (nts)

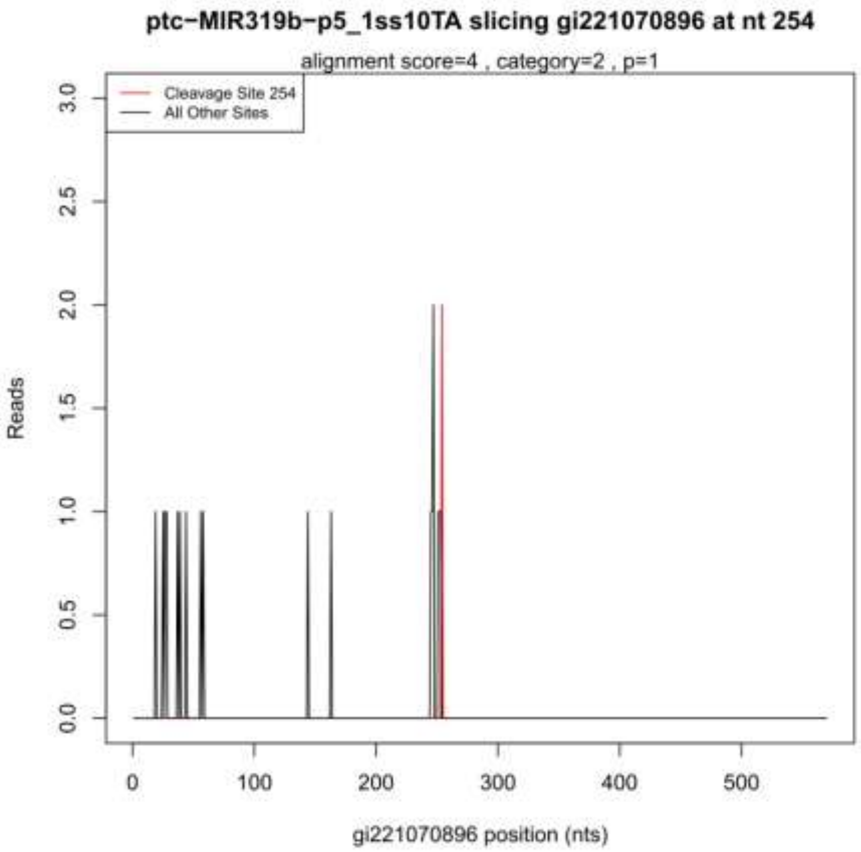
ptc−MIR319b−p5_1ss10TA slicing gi221070896 at nt 254
alignment score=4 , category=2 , p=1
Cleavage Site 254
All Other Sites
Reads
0.0
0.5
1.0
1.5
2.0
2.5
3.0
0
100
200
300
400
500
gi221070896 position (nts)

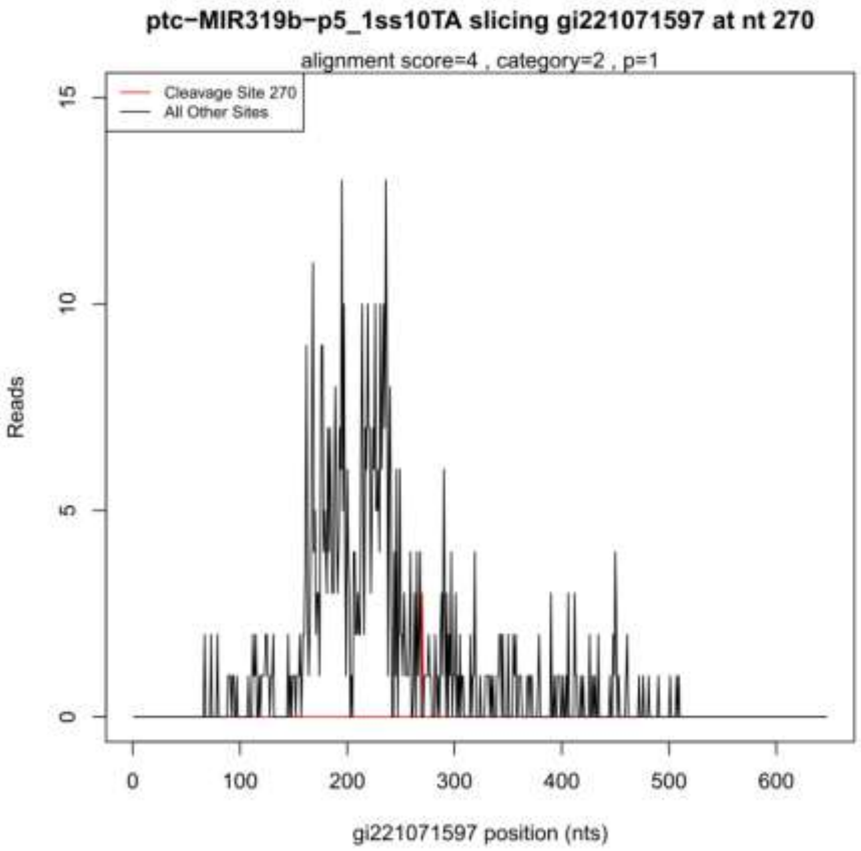
ptc−MIR319b−p5_1ss10TA slicing gi221071597 at nt 270
alignment score=4 , category=2 , p=1
Cleavage Site 270
All Other Sites
Reads
0
5
10
15
0
100
200
300
400
500
600
gi221071597 position (nts)

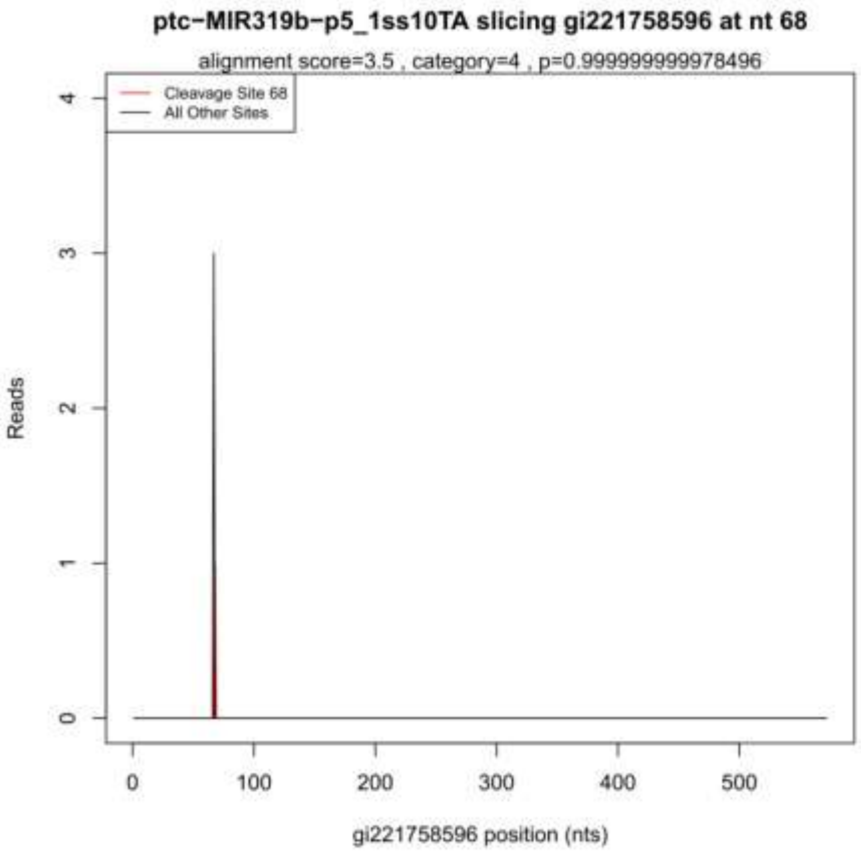
ptc−MIR319b−p5_1ss10TA slicing gi221758596 at nt 68
alignment score=3.5 , category=4 , p=0.999999999978496
Cleavage Site 68
All Other Sites
Reads
0
1
2
3
4
0
100
200
300
400
500
gi221758596 position (nts)

ptc−MIR319b−p5_1ss10TA slicing gi221758896 at nt 102

alignment score=4 , category=2 , p=1

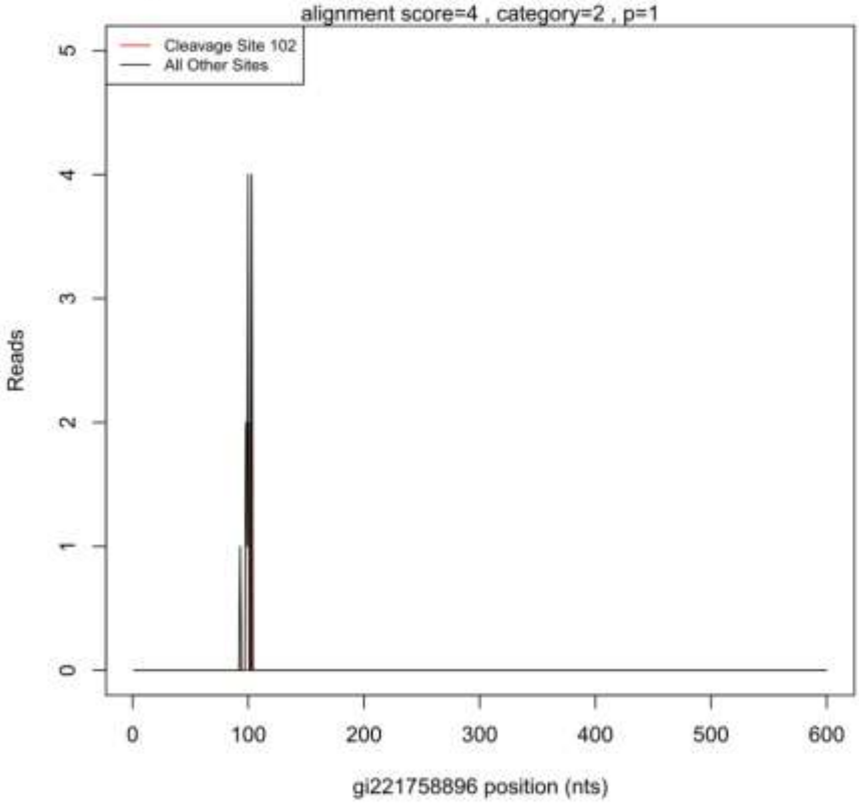

ptc−MIR319b−p5_1ss10TA slicing gi222457788 at nt 338

alignment score=3 , category=4 , p=0.999999999156409

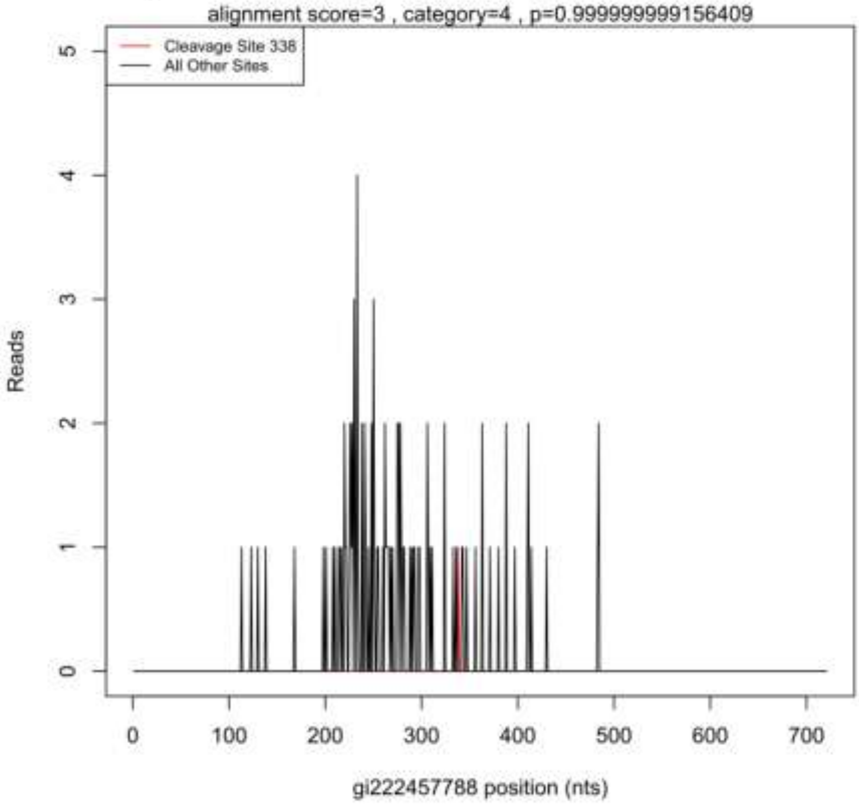

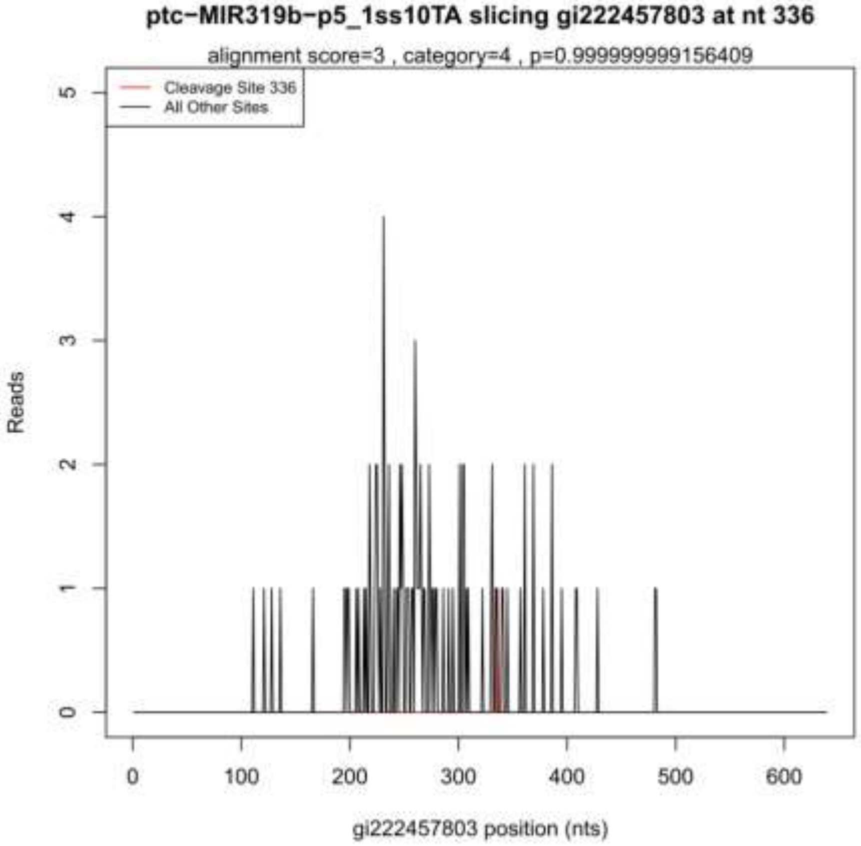
ptc−MIR319b−p5_1ss10TA slicing gi222457803 at nt 336
alignment score=3 , category=4 , p=0.999999999156409
Cleavage Site 336
All Other Sites
Reads
0
1
2
3
4
5
0
100
200
300
400
500
600
gi222457803 position (nts)

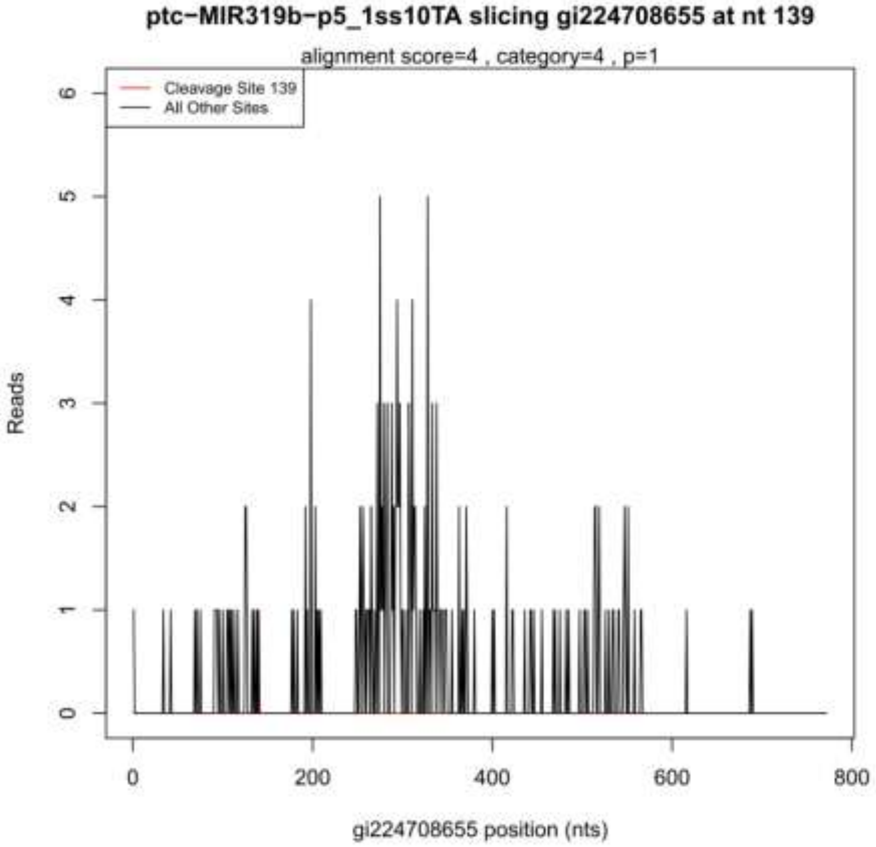
ptc−MIR319b−p5_1ss10TA slicing gi224708655 at nt 139
alignment score=4 , category=4 , p=1
Cleavage Site 139
All Other Sites
Reads
0
1
2
3
4
5
6
0
200
400
600
800
gi224708655 position (nts)

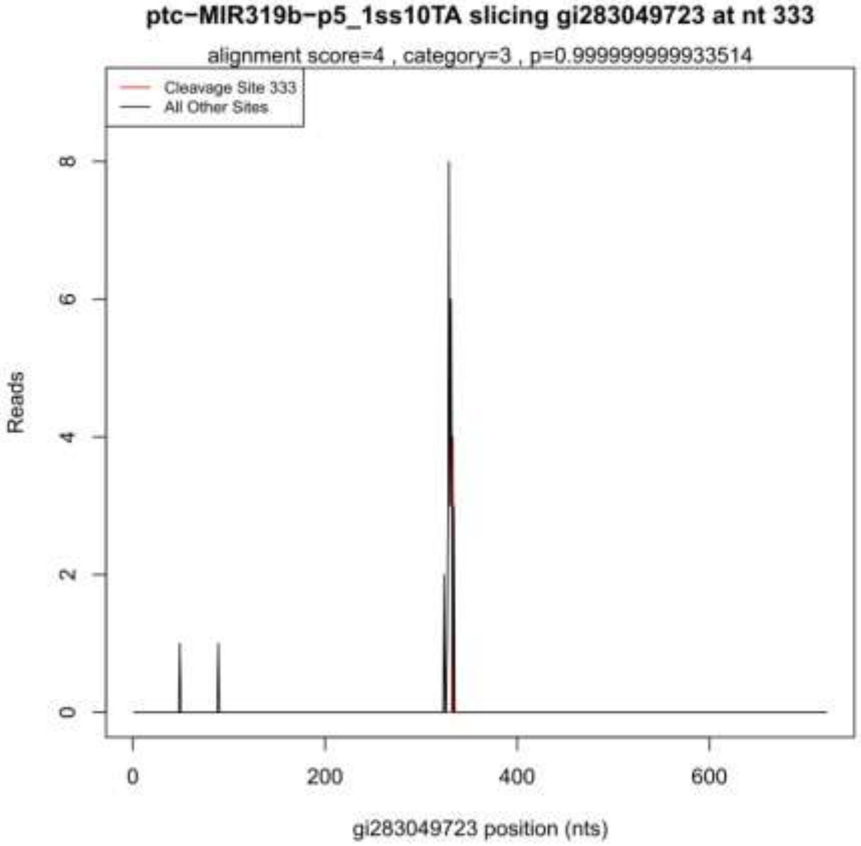
ptc−MIR319b−p5_1ss10TA slicing gi283049723 at nt 333
alignment score=4 , category=3 , p=0.999999999933514
Cleavage Site 333
All Other Sites
Reads
0
2
4
6
8
0
200
400
600
gi283049723 position (nts)

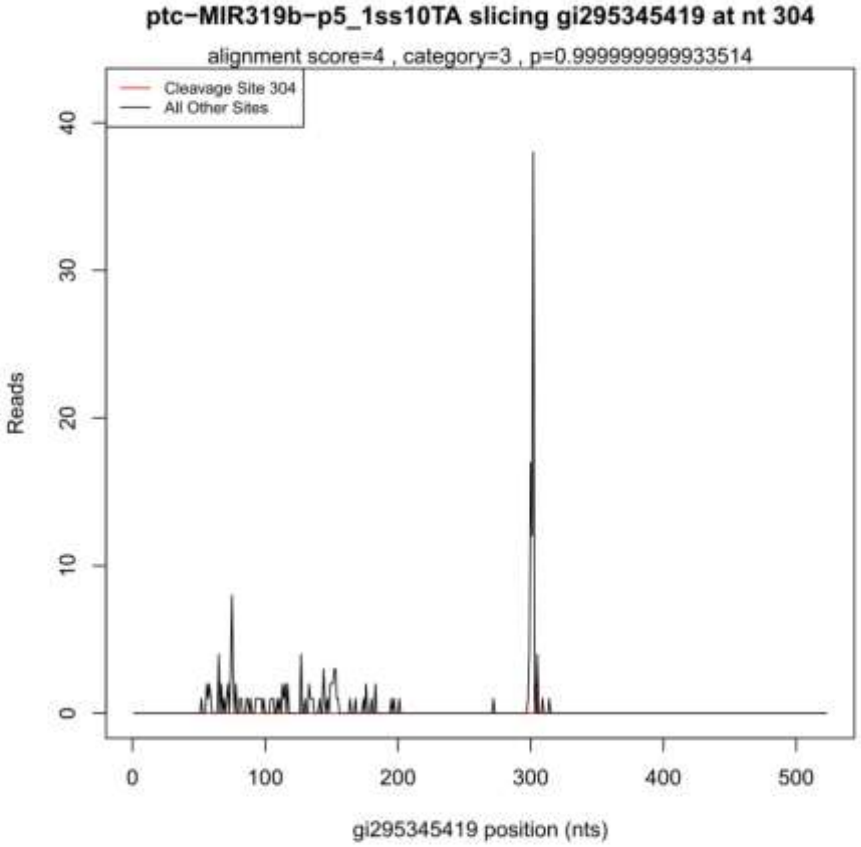
ptc−MIR319b−p5_1ss10TA slicing gi295345419 at nt 304
alignment score=4 , category=3 , p=0.999999999933514
Cleavage Site 304
All Other Sites
Reads
0
10
20
30
40
0
100
200
300
400
500
gi295345419 position (nts)

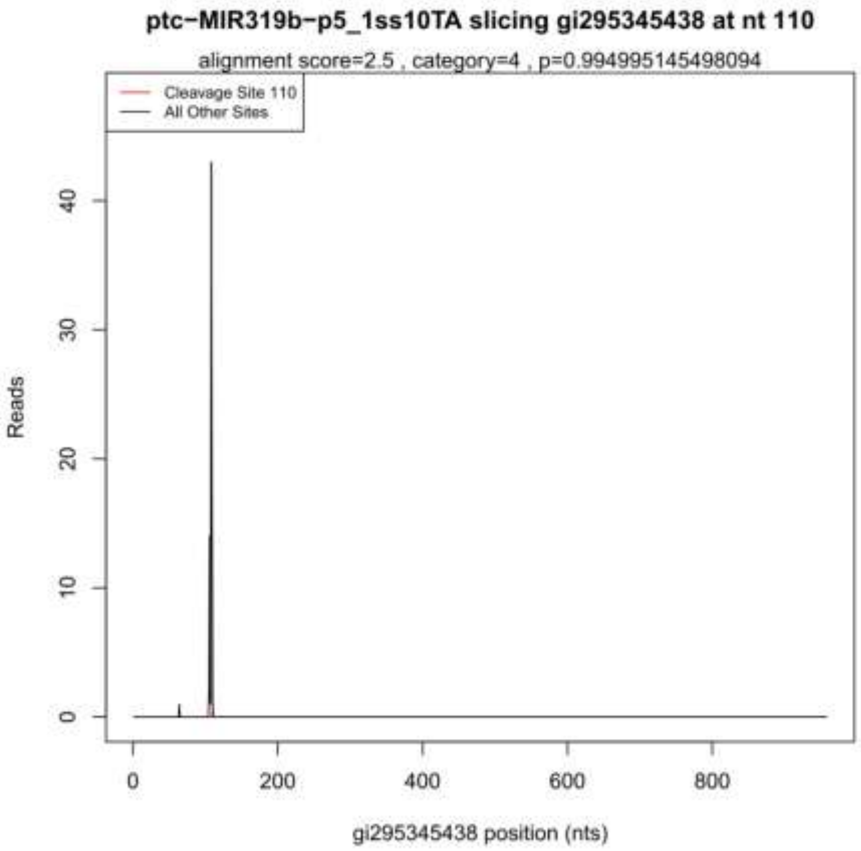

ptc−MIR319b−p5_1ss10TA slicing gi295345438 at nt 110
alignment score=2.5 , category=4 , p=0.994995145498094
Cleavage Site 110
All Other Sites
Reads
0
10
20
30
40
0
200
400
600
800
gi295345438 position (nts)

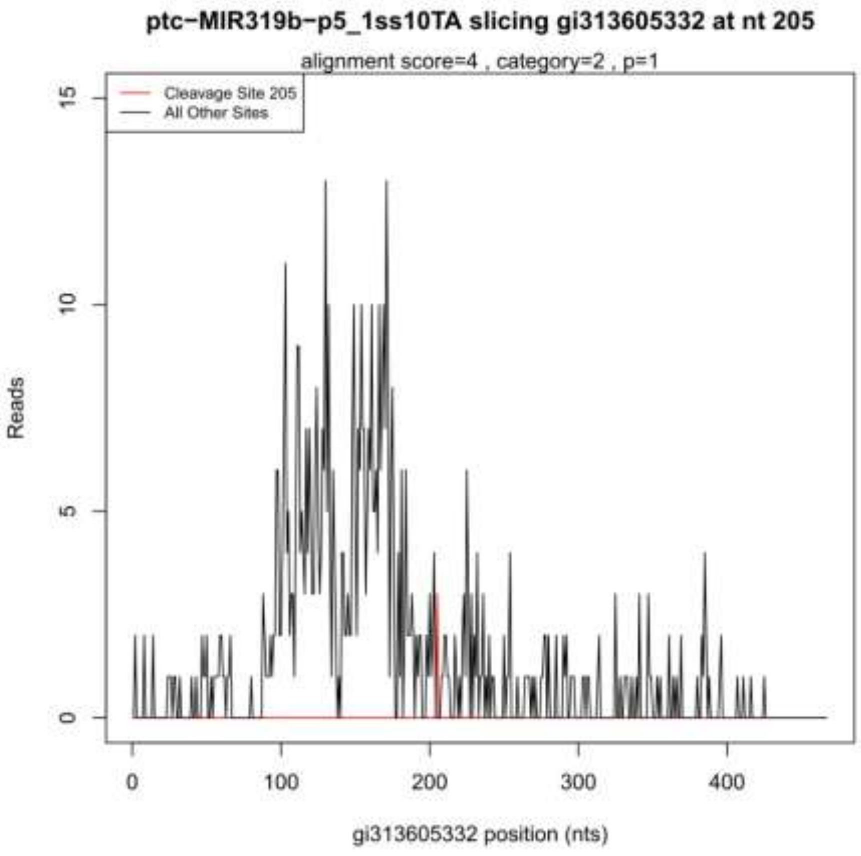

ptc−MIR319b−p5_1ss10TA slicing gi313605332 at nt 205
alignment score=4 , category=2 , p=1
Cleavage Site 205
All Other Sites
Reads
0
5
10
15
0
100
200
300
400
gi313605332 position (nts)

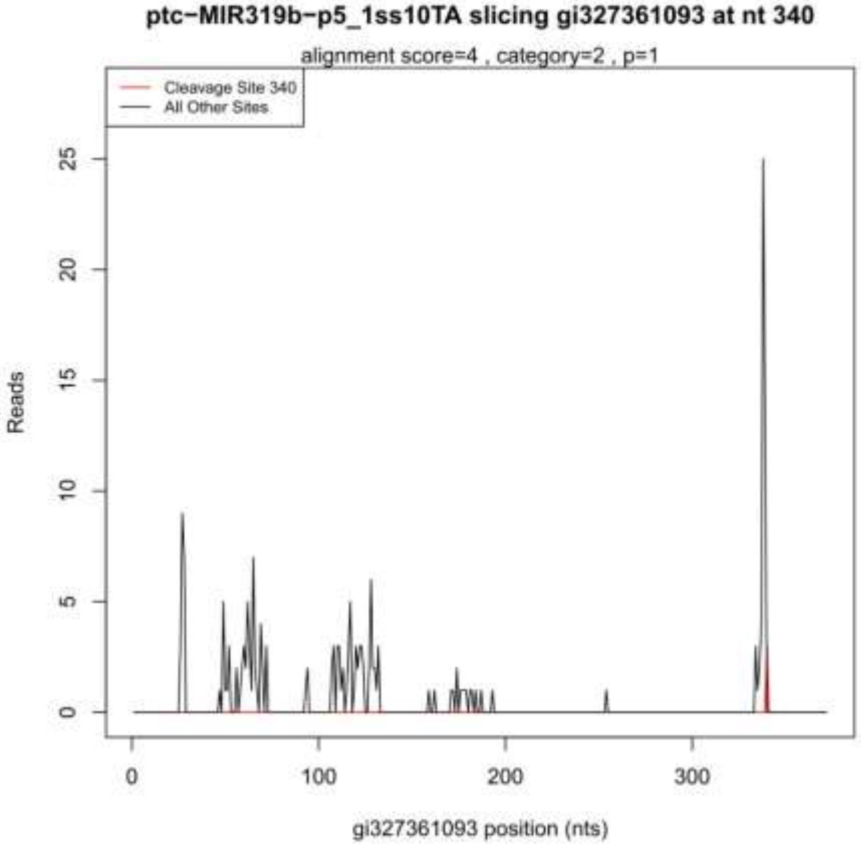

ptc−MIR319b−p5_1ss10TA slicing gi327361093 at nt 340
alignment score=4 , category=2 , p=1
Cleavage Site 340
All Other Sites
Reads
0
5
10
15
20
25
0
100
200
300
gi327361093 position (nts)

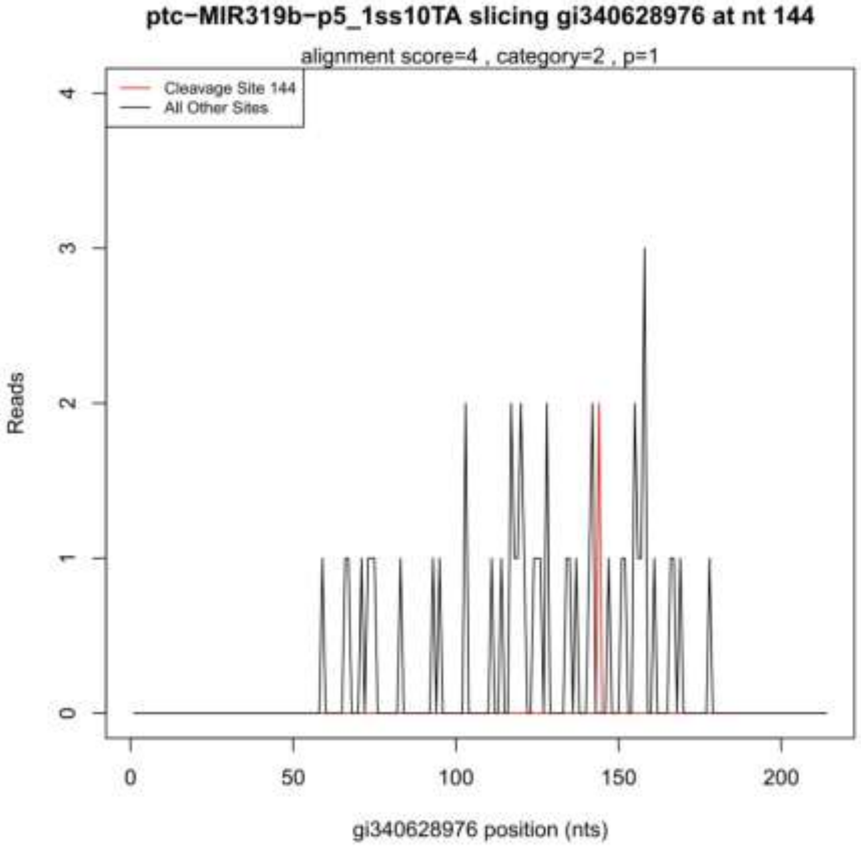

ptc−MIR319b−p5_1ss10TA slicing gi340628976 at nt 144
alignment score=4 , category=2 , p=1
Cleavage Site 144
All Other Sites
Reads
0
1
2
3
4
0
50
100
150
200
gi340628976 position (nts)

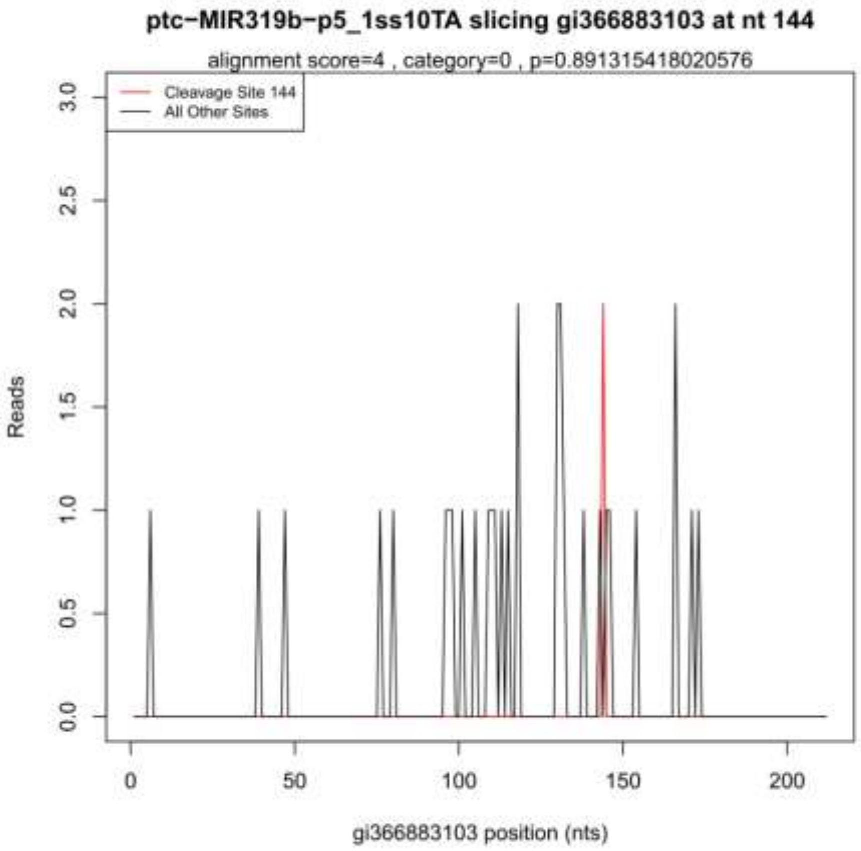
ptc−MIR319b−p5_1ss10TA slicing gi366883103 at nt 144
alignment score=4 , category=0 , p=0.891315418020576
Cleavage Site 144
All Other Sites
Reads
gi366883103 position (nts)

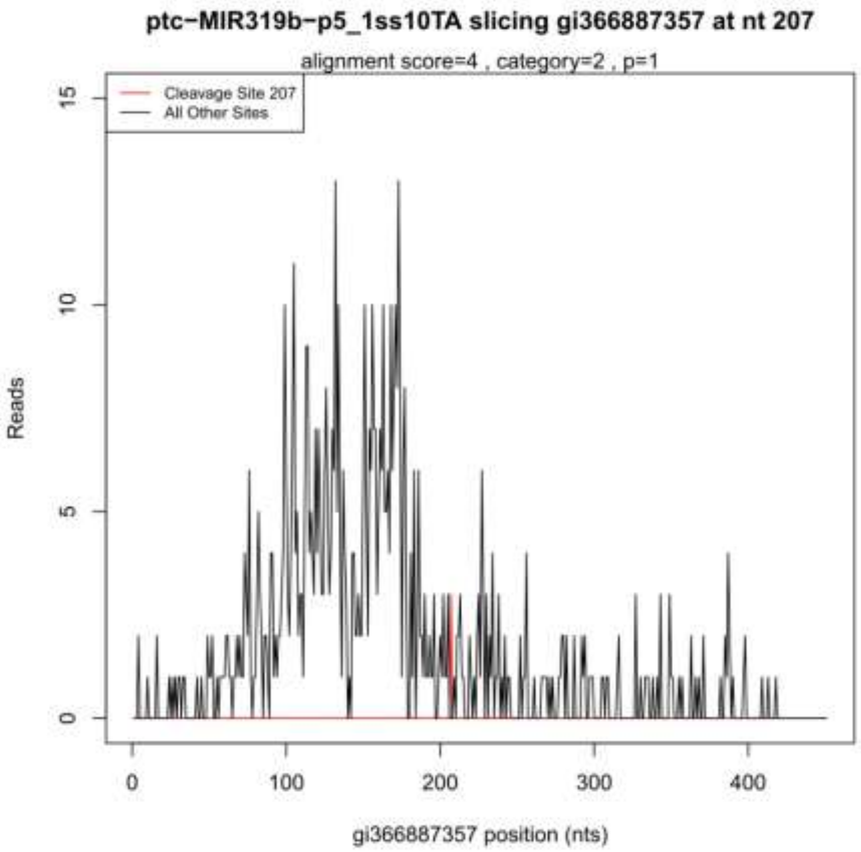
ptc−MIR319b−p5_1ss10TA slicing gi366887357 at nt 207
alignment score=4 , category=2 , p=1
Cleavage Site 207
All Other Sites
Reads
gi366887357 position (nts)

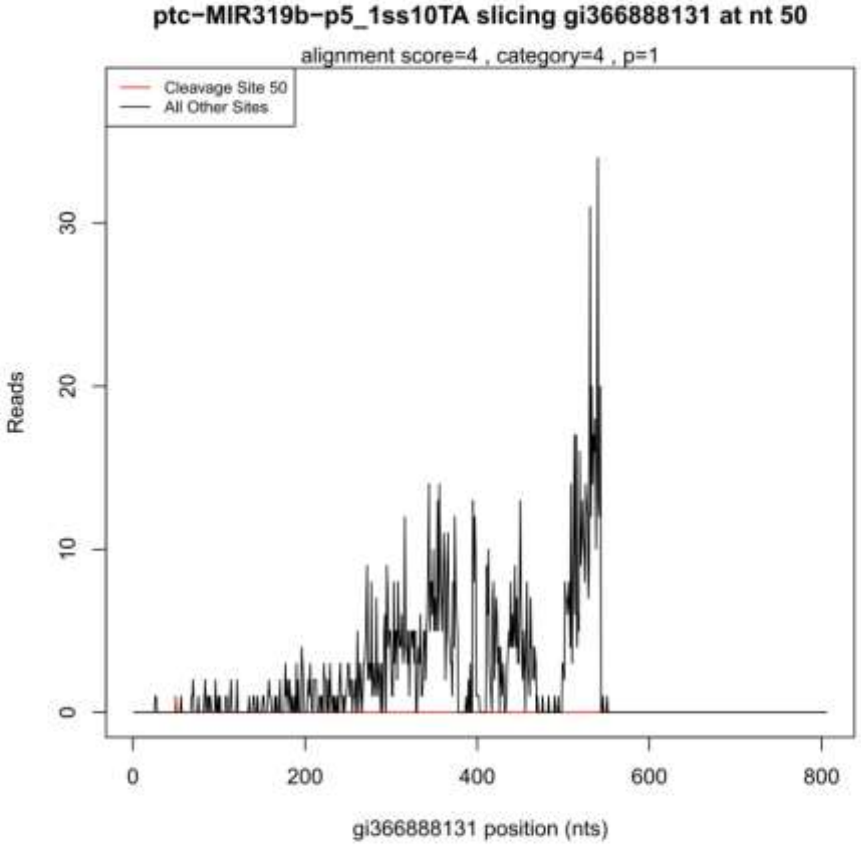
ptc−MIR319b−p5_1ss10TA slicing gi366888131 at nt 50
alignment score=4 , category=4 , p=1
Cleavage Site 50
All Other Sites
Reads
0
10
20
30
0
200
400
600
800
gi366888131 position (nts)

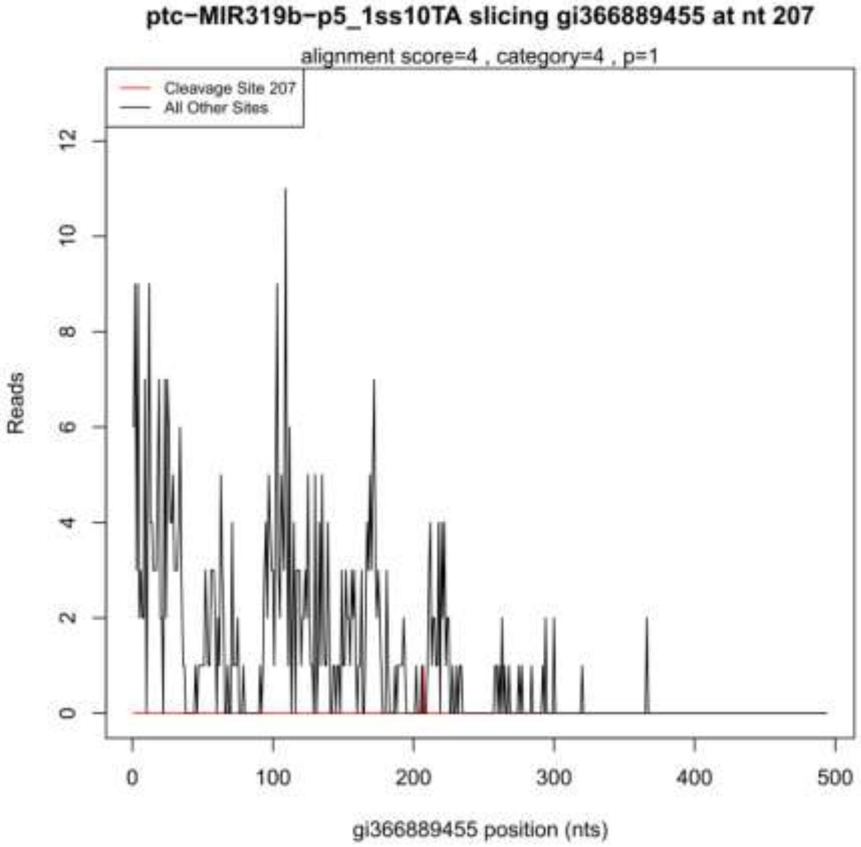
ptc−MIR319b−p5_1ss10TA slicing gi366889455 at nt 207
alignment score=4 , category=4 , p=1
Cleavage Site 207
All Other Sites
Reads
0
2
4
6
8
10
12
0
100
200
300
400
500
gi366889455 position (nts)

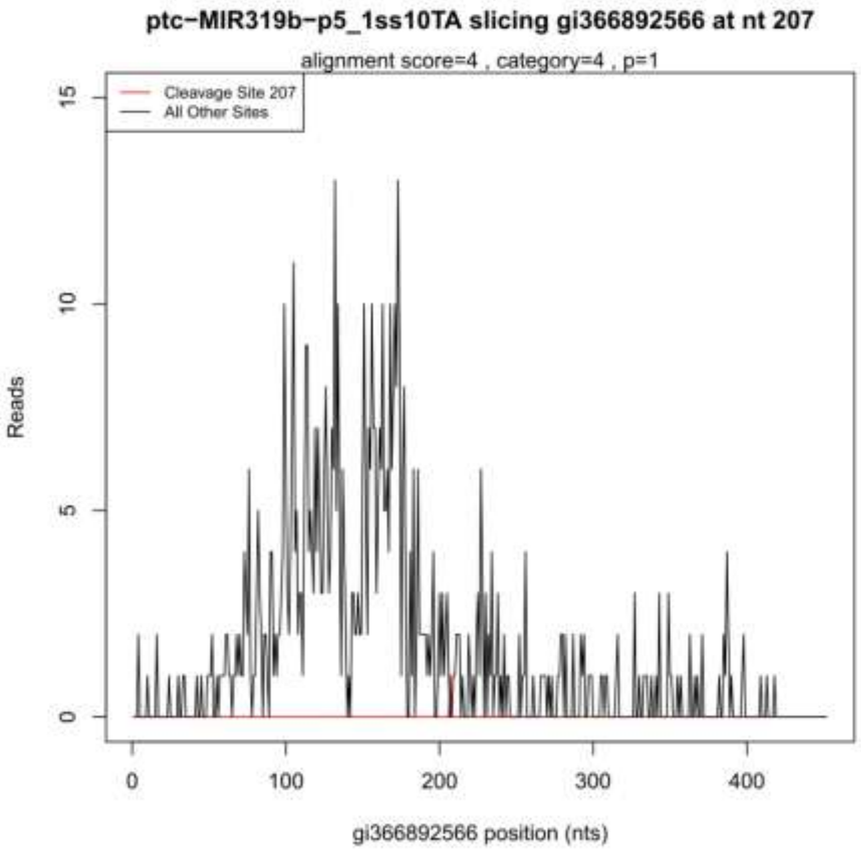

ptc−MIR319b−p5_1ss10TA slicing gi366892566 at nt 207
alignment score=4 , category=4 , p=1
Cleavage Site 207
All Other Sites
Reads
0
5
10
15
0
100
200
300
400
gi366892566 position (nts)

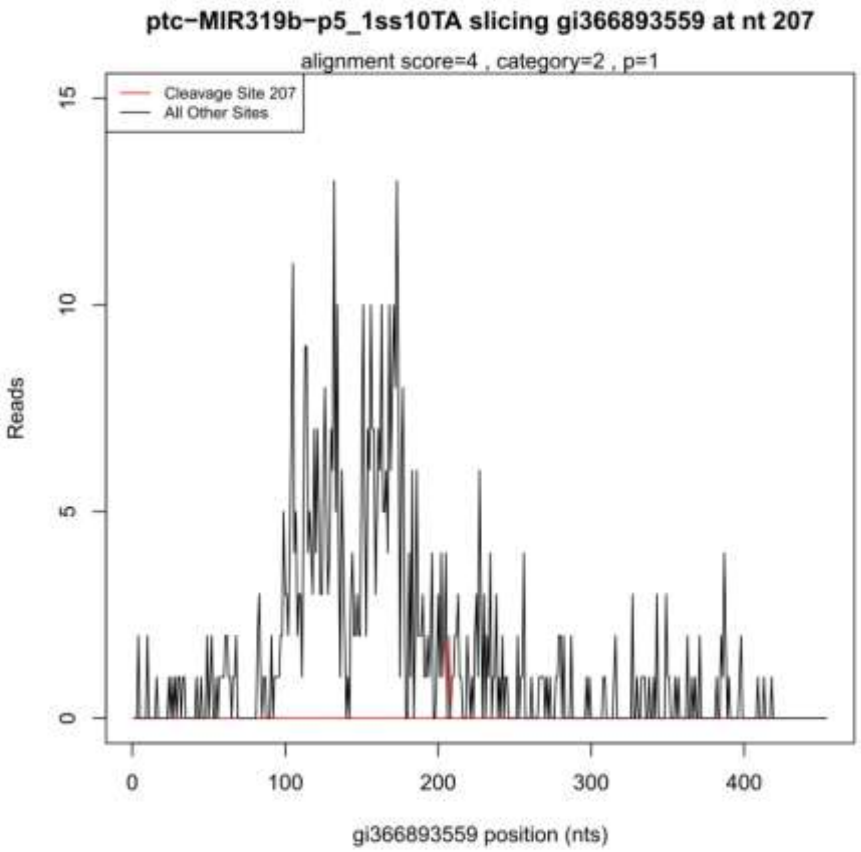

ptc−MIR319b−p5_1ss10TA slicing gi366893559 at nt 207
alignment score=4 , category=2 , p=1
Cleavage Site 207
All Other Sites
Reads
0
5
10
15
0
100
200
300
400
gi366893559 position (nts)

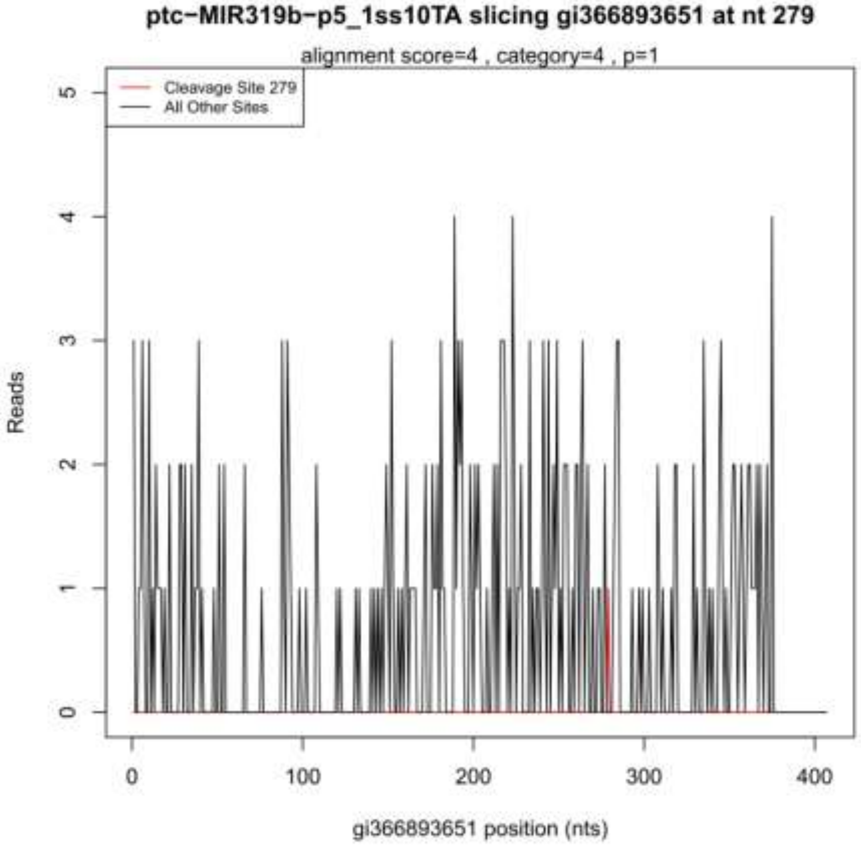
ptc-MIR319b-p5_1ss10TA slicing gi366893651 at nt 279
alignment score=4 , category=4 , p=1
Cleavage Site 279
All Other Sites
Reads
gi366893651 position (nts)

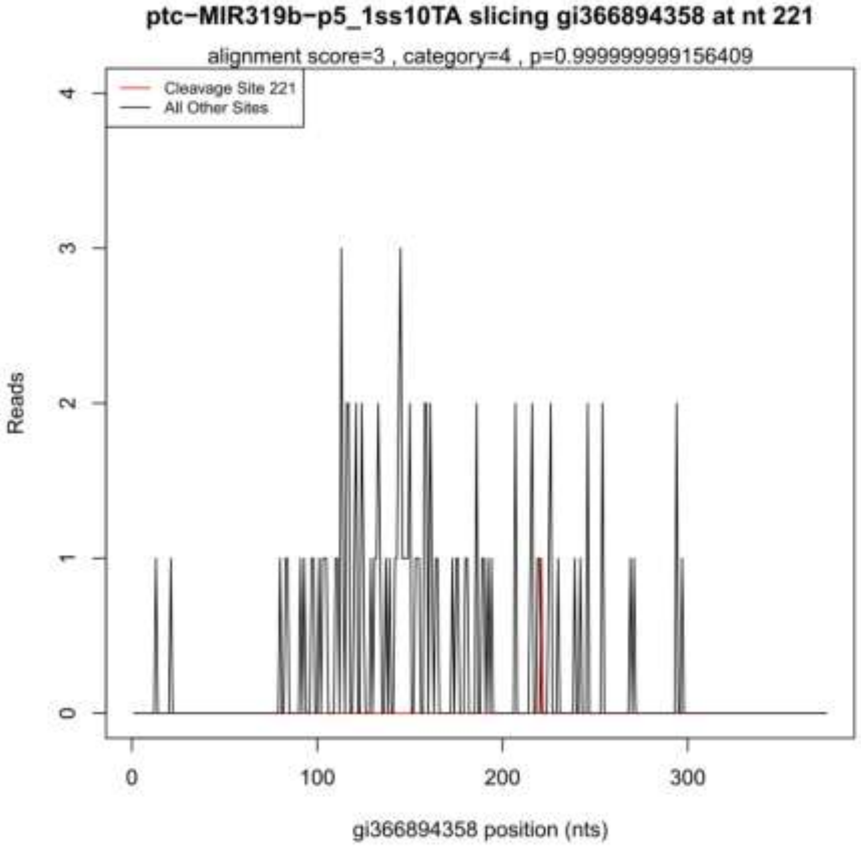
ptc-MIR319b-p5_1ss10TA slicing gi366894358 at nt 221
alignment score=3 , category=4 , p=0.999999999156409
Cleavage Site 221
All Other Sites
Reads
gi366894358 position (nts)

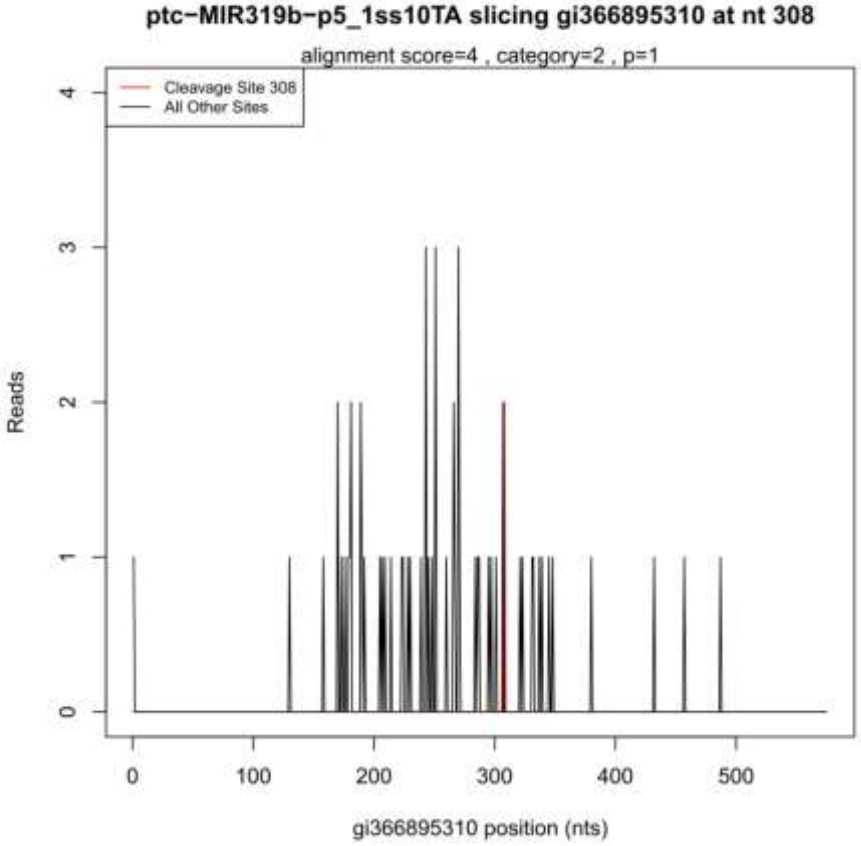

ptc−MIR319b−p5_1ss10TA slicing gi366895310 at nt 308
alignment score=4 , category=2 , p=1
Cleavage Site 308
All Other Sites
Reads
0
1
2
3
4
0
100
200
300
400
500
gi366895310 position (nts)

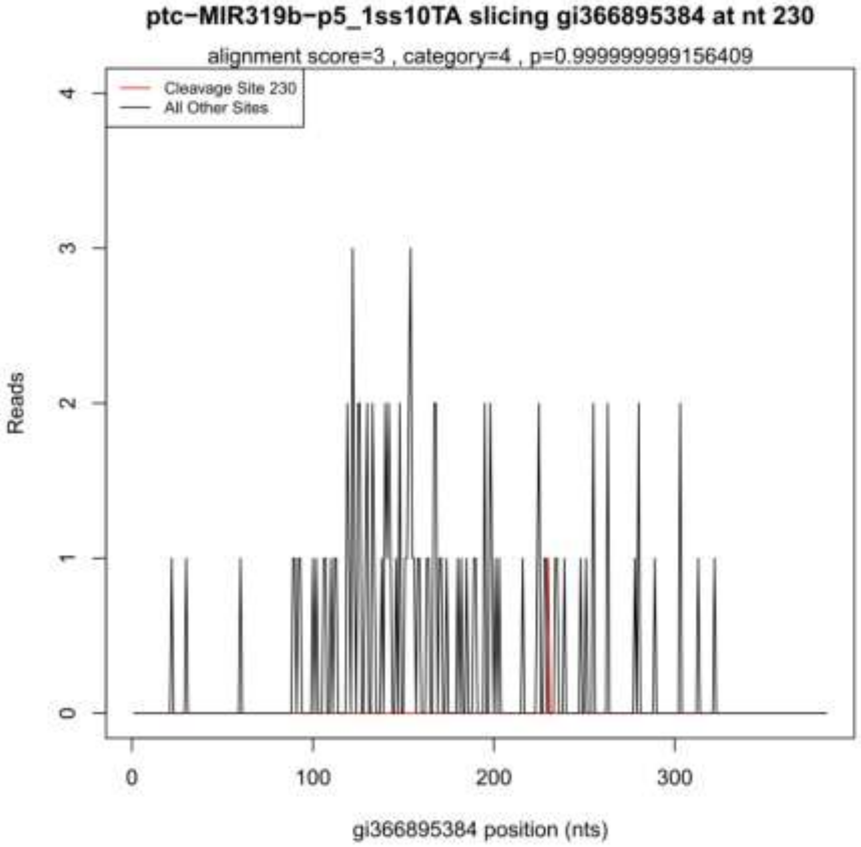

ptc−MIR319b−p5_1ss10TA slicing gi366895384 at nt 230
alignment score=3 , category=4 , p=0.999999999156409
Cleavage Site 230
All Other Sites
Reads
0
1
2
3
4
0
100
200
300
gi366895384 position (nts)

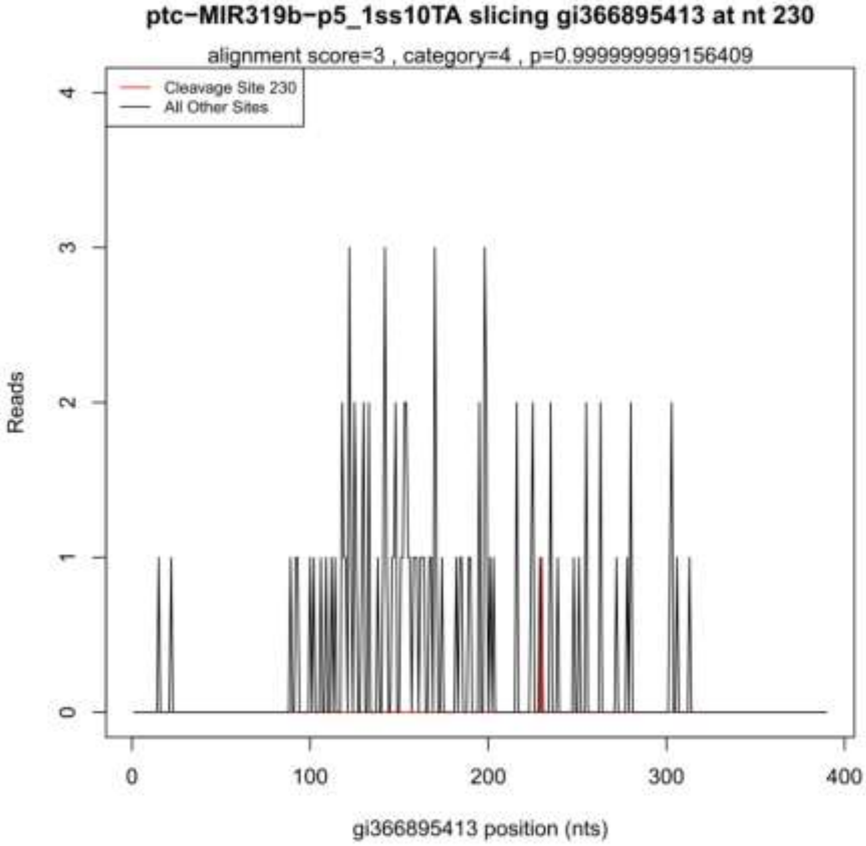
ptc-MIR319b-p5_1ss10TA slicing gi366895413 at nt 230
alignment score=3 , category=4 , p=0.999999999156409
Cleavage Site 230
All Other Sites
Reads
0
1
2
3
4
0
100
200
300
400
gi366895413 position (nts)

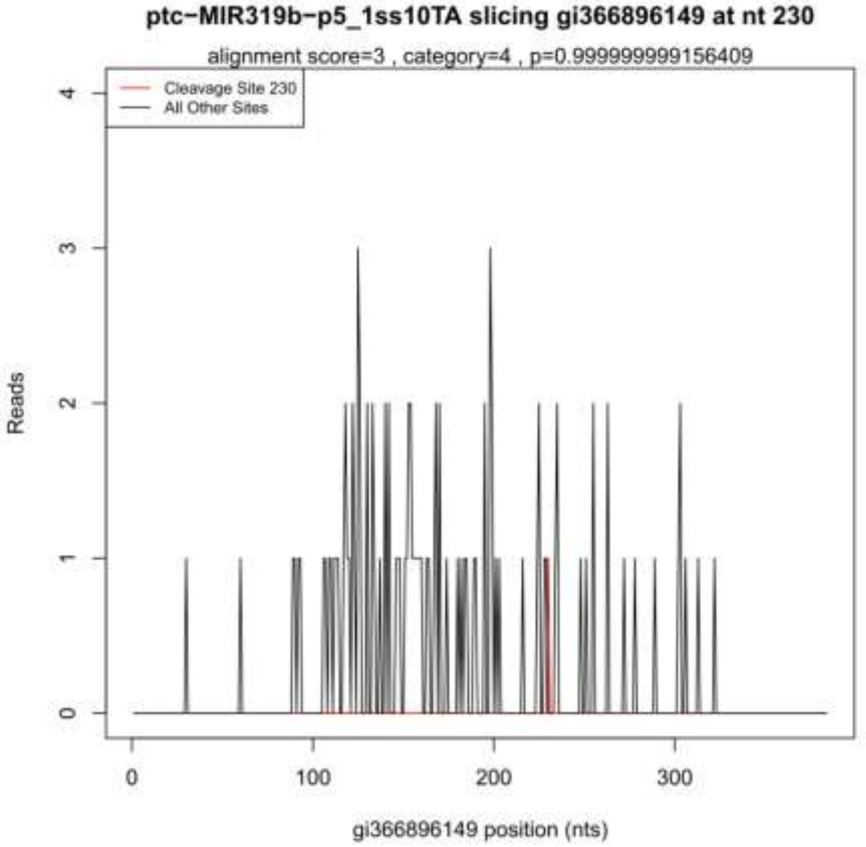
ptc-MIR319b-p5_1ss10TA slicing gi366896149 at nt 230
alignment score=3 , category=4 , p=0.999999999156409
Cleavage Site 230
All Other Sites
Reads
0
1
2
3
4
0
100
200
300
gi366896149 position (nts)

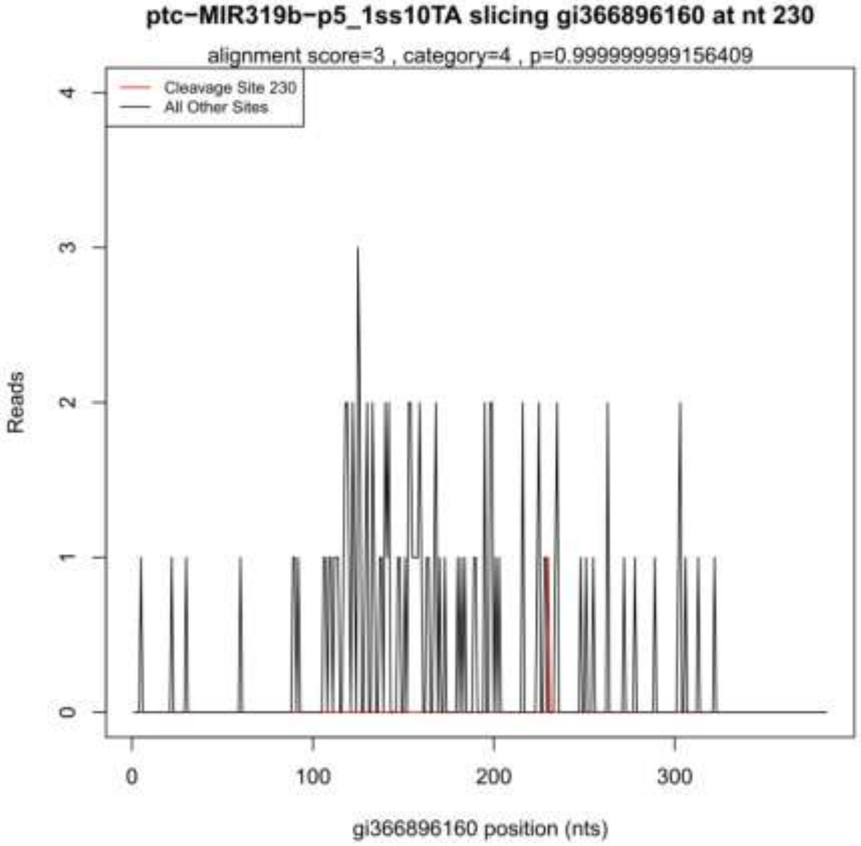
ptc−MIR319b−p5_1ss10TA slicing gi366896160 at nt 230
alignment score=3 , category=4 , p=0.999999999156409
Cleavage Site 230
All Other Sites
Reads
0
1
2
3
4
0
100
200
300
gi366896160 position (nts)

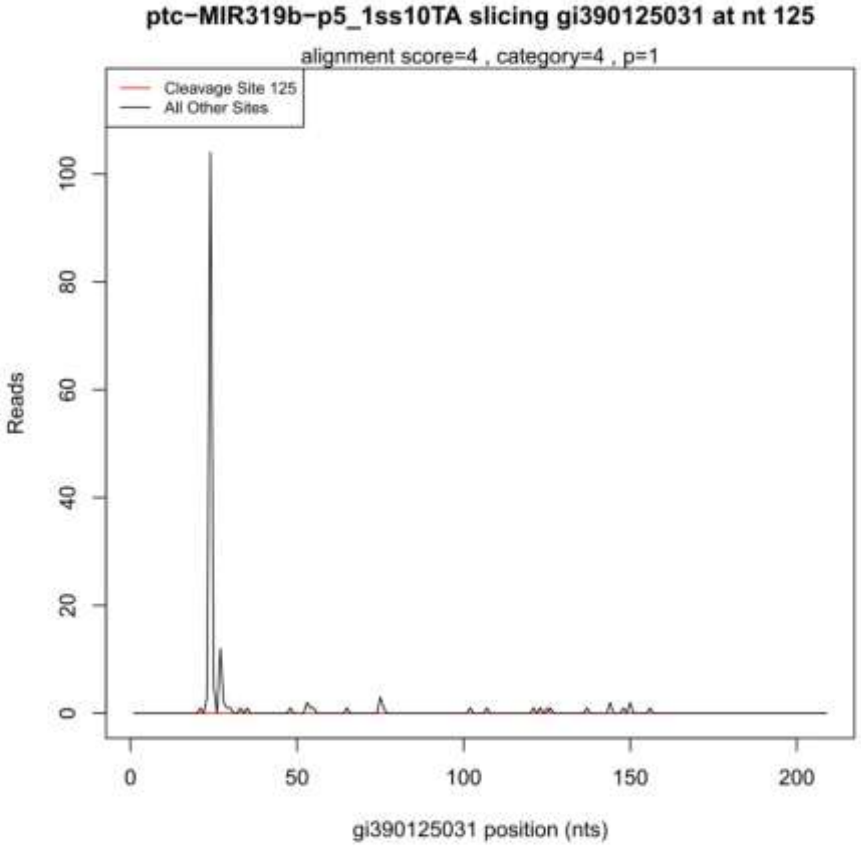
ptc−MIR319b−p5_1ss10TA slicing gi390125031 at nt 125
alignment score=4 , category=4 , p=1
Cleavage Site 125
All Other Sites
Reads
0
20
40
60
80
100
0
50
100
150
200
gi390125031 position (nts)

ptc−MIR319b−p5_1ss10TA slicing gi390125056 at nt 177

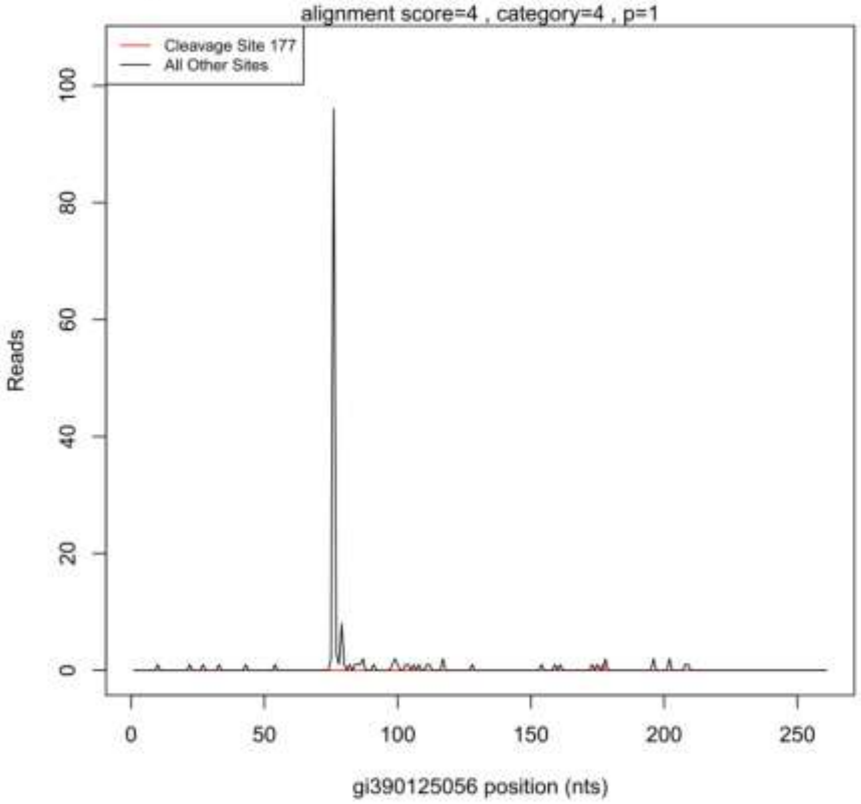

ptc−MIR319b−p5_1ss10TA slicing gi393389193 at nt 142

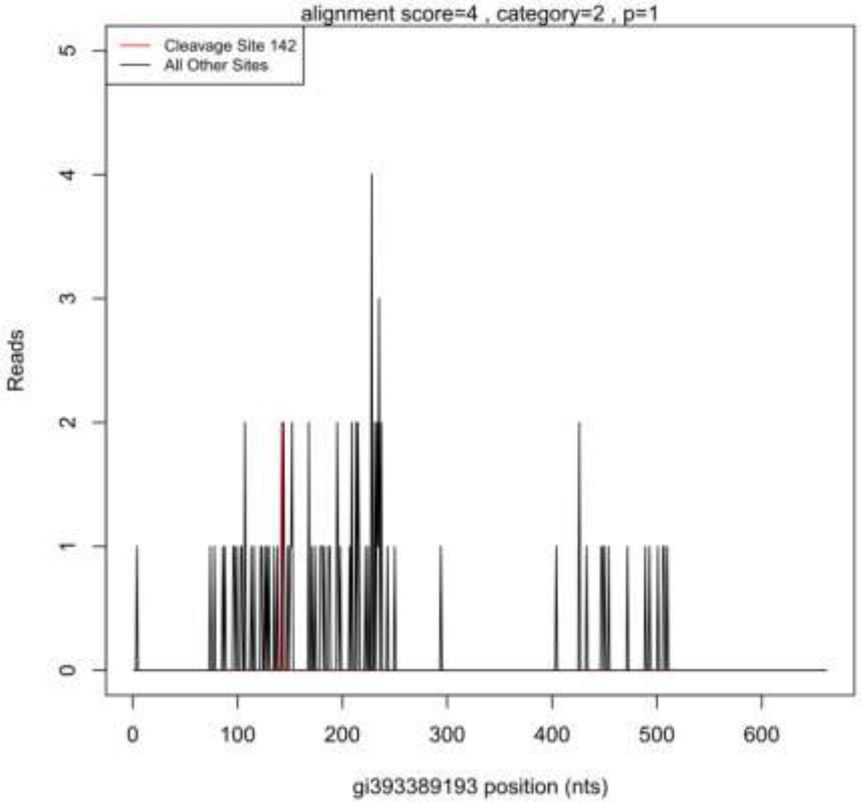

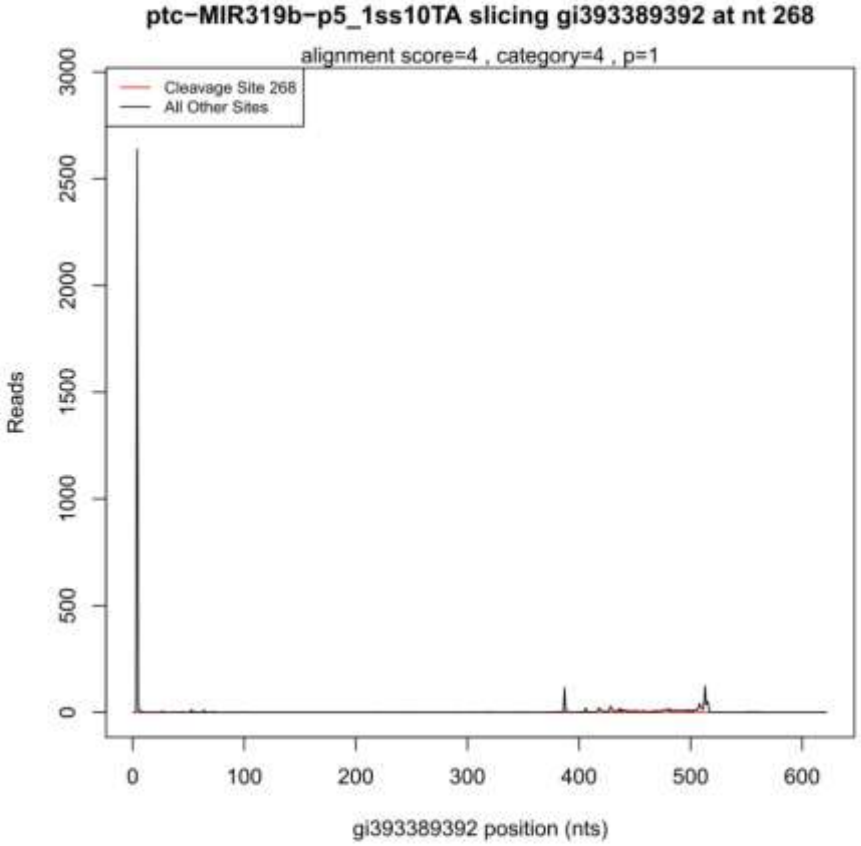
ptc−MIR319b−p5_1ss10TA slicing gi393389392 at nt 268
alignment score=4 , category=4 , p=1
Cleavage Site 268
All Other Sites
Reads
gi393389392 position (nts)

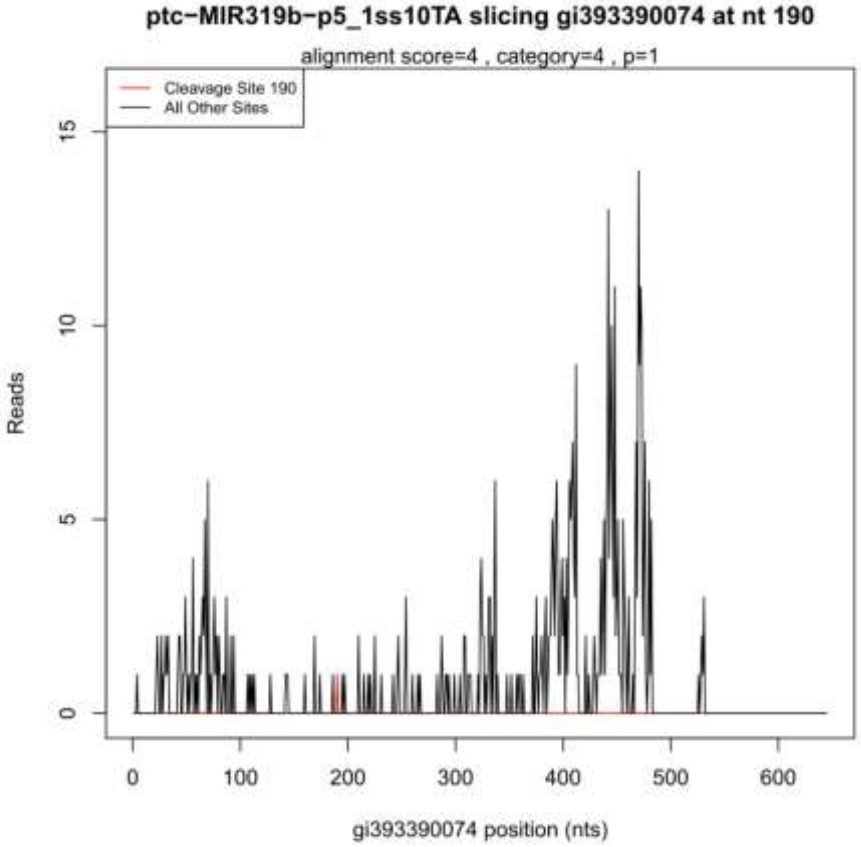
ptc−MIR319b−p5_1ss10TA slicing gi393390074 at nt 190
alignment score=4 , category=4 , p=1
Cleavage Site 190
All Other Sites
Reads
gi393390074 position (nts)

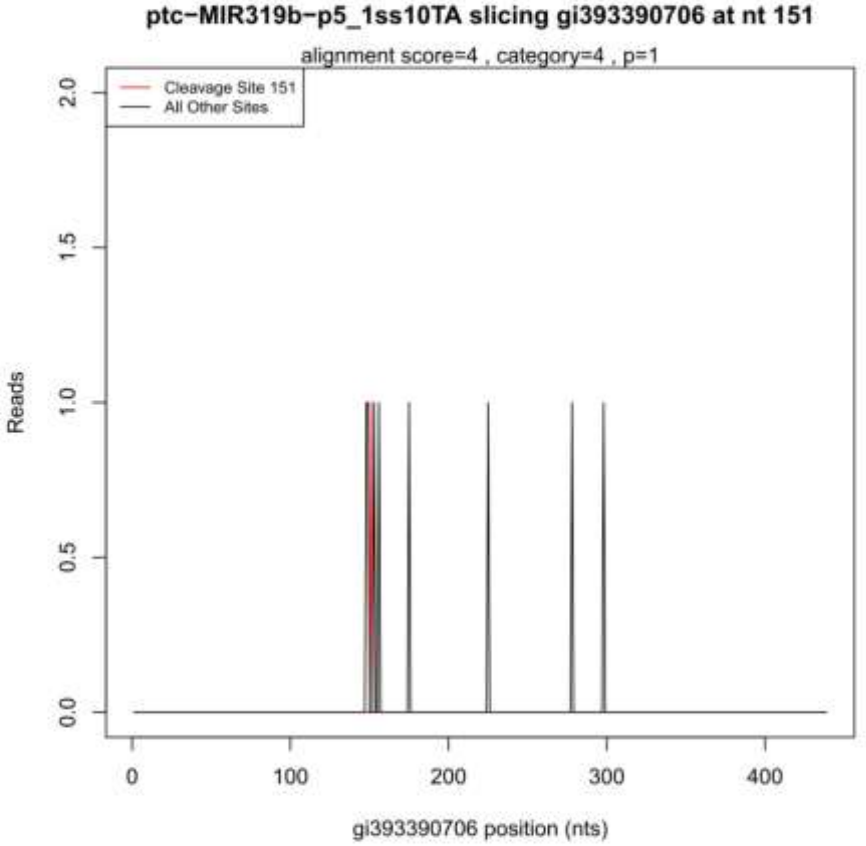
ptc−MIR319b−p5_1ss10TA slicing gi393390706 at nt 151
alignment score=4 , category=4 , p=1
Cleavage Site 151
All Other Sites
Reads
0.0
0.5
1.0
1.5
2.0
0
100
200
300
400
gi393390706 position (nts)

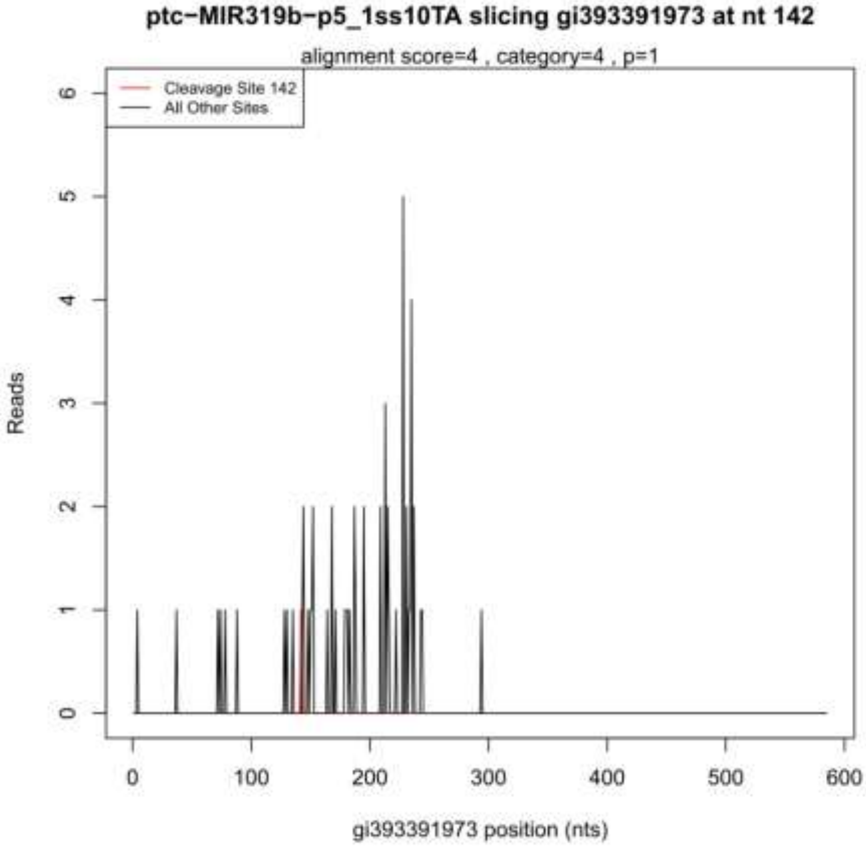
ptc−MIR319b−p5_1ss10TA slicing gi393391973 at nt 142
alignment score=4 , category=4 , p=1
Cleavage Site 142
All Other Sites
Reads
0
1
2
3
4
5
6
0
100
200
300
400
500
600
gi393391973 position (nts)

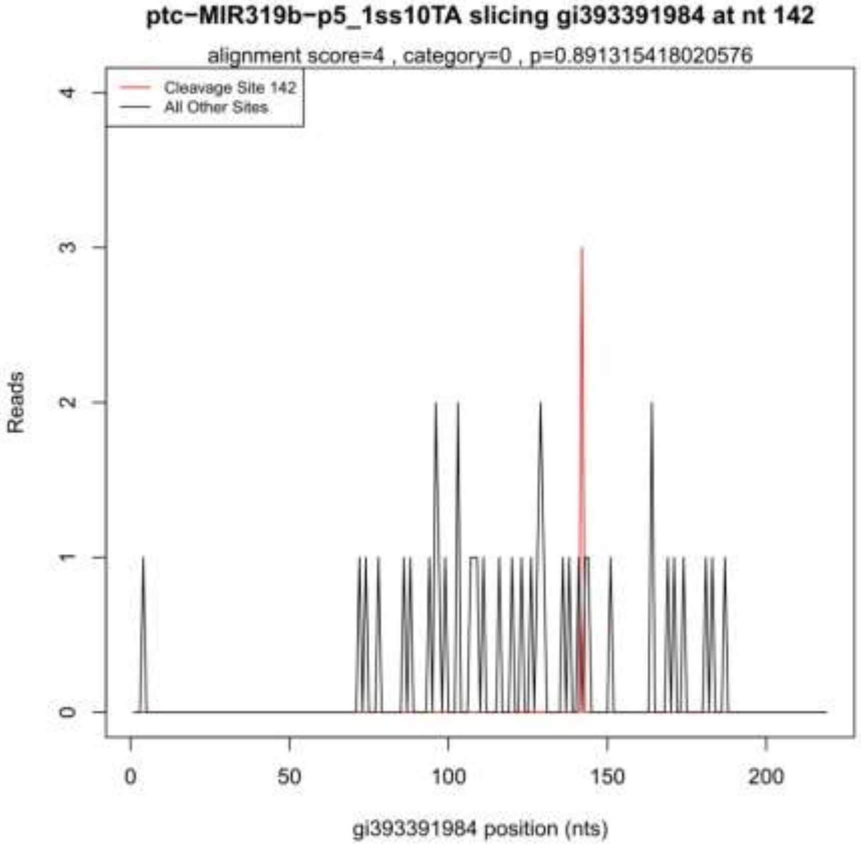
ptc−MIR319b−p5_1ss10TA slicing gi393391984 at nt 142
alignment score=4 , category=0 , p=0.891315418020576
Cleavage Site 142
All Other Sites
Reads
0
1
2
3
4
0
50
100
150
200
gi393391984 position (nts)

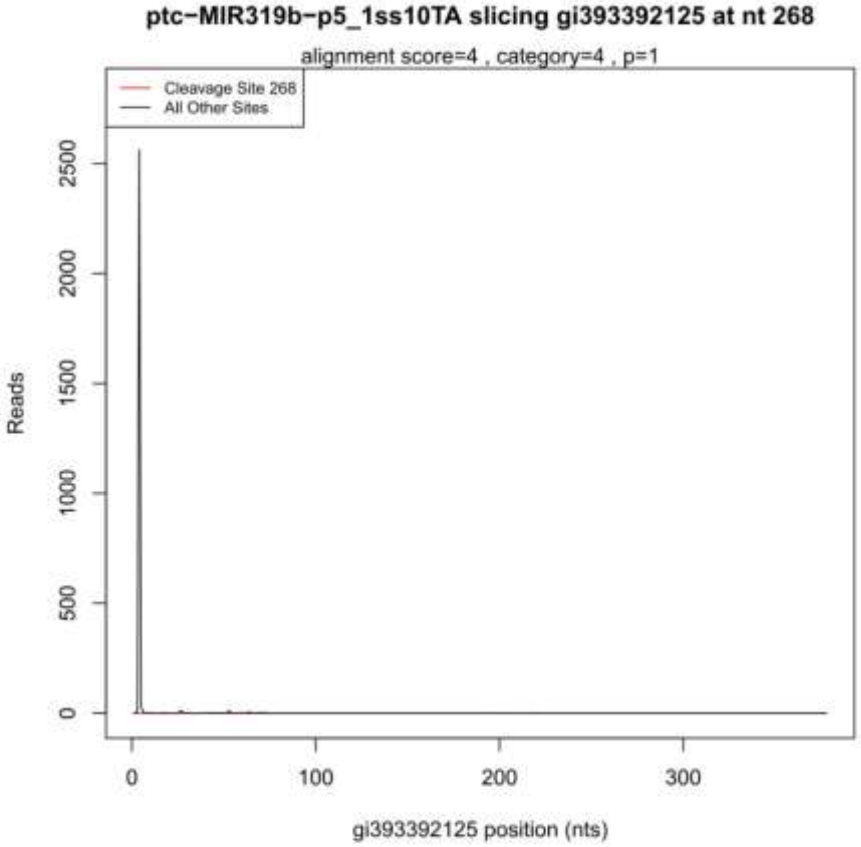
ptc−MIR319b−p5_1ss10TA slicing gi393392125 at nt 268
alignment score=4 , category=4 , p=1
Cleavage Site 268
All Other Sites
Reads
0
500
1000
1500
2000
2500
0
100
200
300
gi393392125 position (nts)

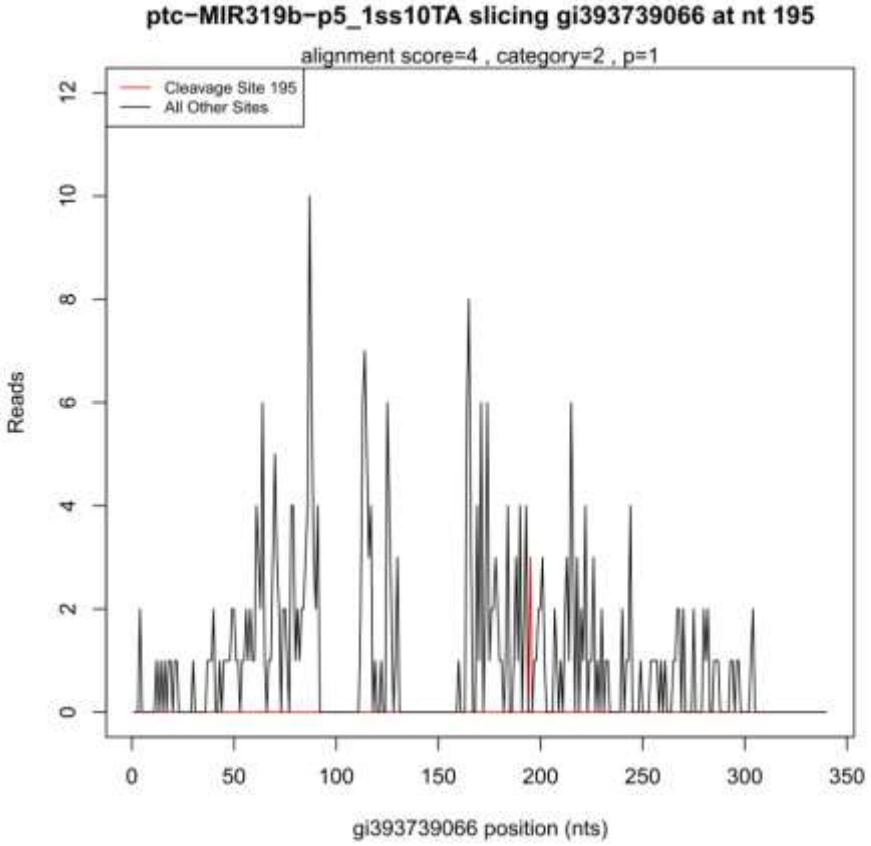
ptc−MIR319b−p5_1ss10TA slicing gi393739066 at nt 195
alignment score=4 , category=2 , p=1
Cleavage Site 195
All Other Sites
Reads
gi393739066 position (nts)

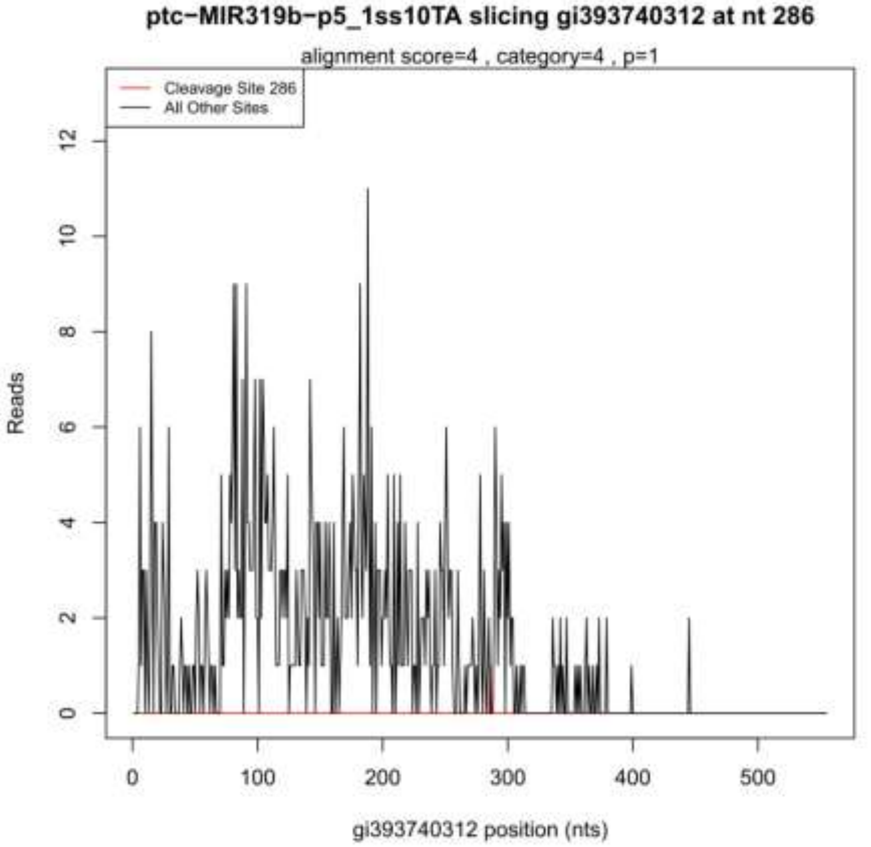
ptc−MIR319b−p5_1ss10TA slicing gi393740312 at nt 286
alignment score=4 , category=4 , p=1
Cleavage Site 286
All Other Sites
Reads
gi393740312 position (nts)

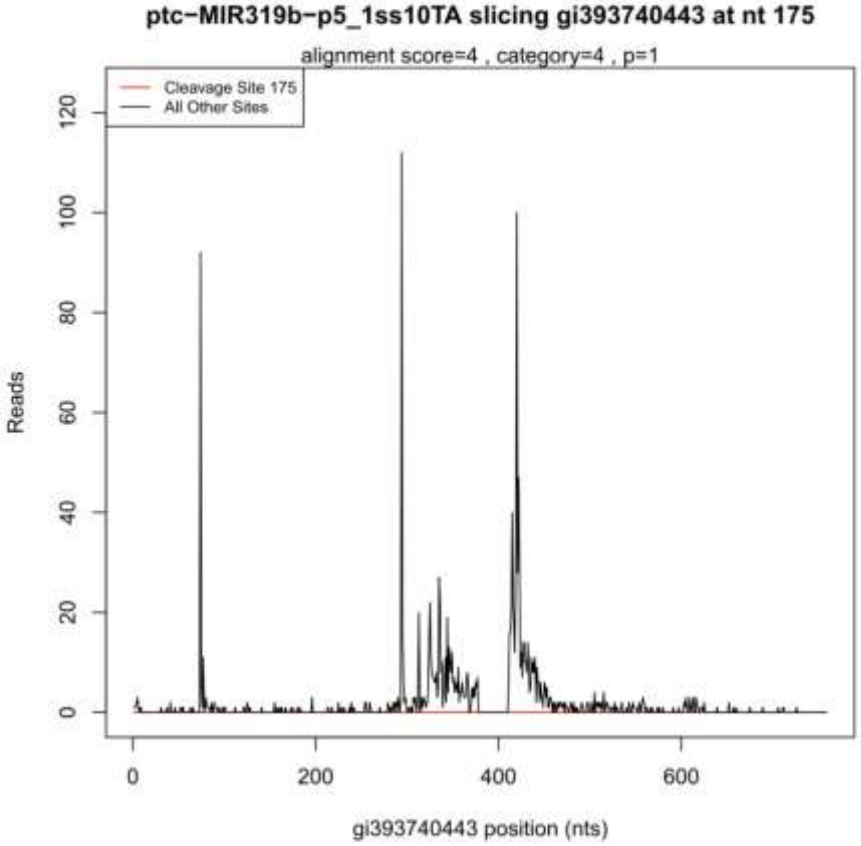

ptc−MIR319b−p5_1ss10TA slicing gi393740443 at nt 175
alignment score=4 , category=4 , p=1
Cleavage Site 175
All Other Sites
Reads
0
20
40
60
80
100
120
0
200
400
600
gi393740443 position (nts)

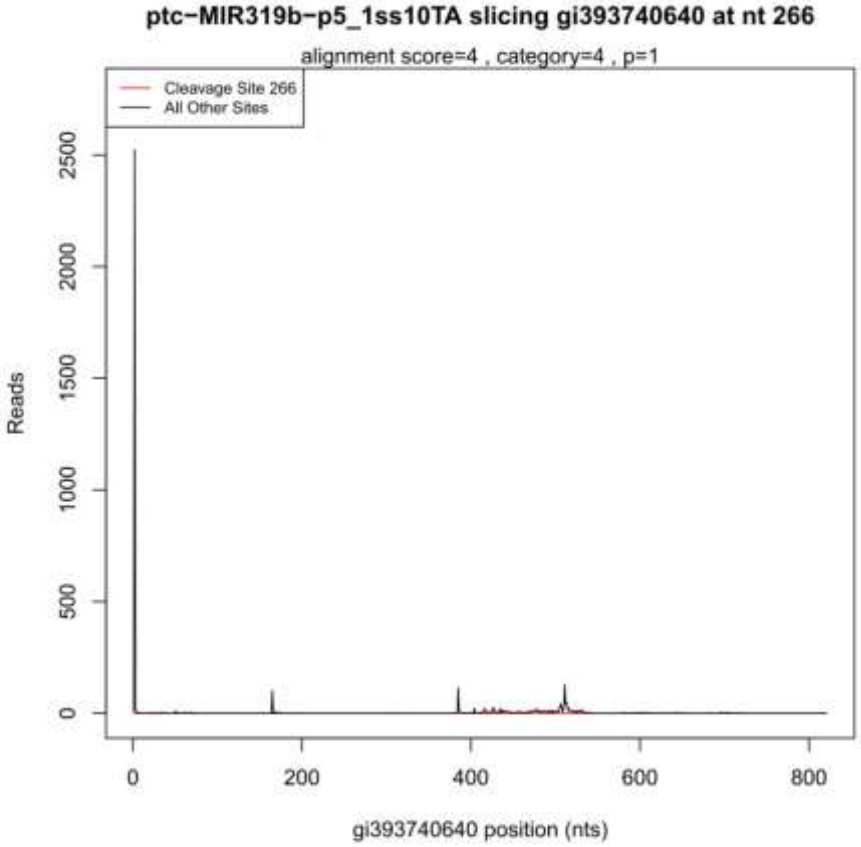

ptc−MIR319b−p5_1ss10TA slicing gi393740640 at nt 266
alignment score=4 , category=4 , p=1
Cleavage Site 266
All Other Sites
Reads
0
500
1000
1500
2000
2500
0
200
400
600
800
gi393740640 position (nts)

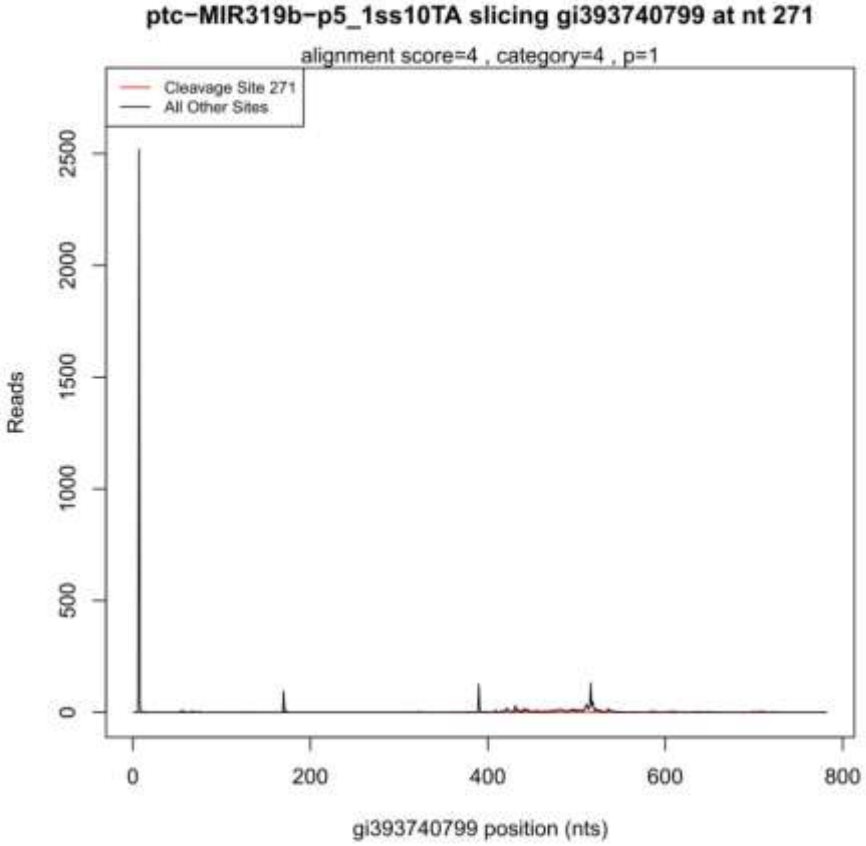
ptc−MIR319b−p5_1ss10TA slicing gi393740799 at nt 271
alignment score=4 , category=4 , p=1
Cleavage Site 271
All Other Sites
Reads
0
500
1000
1500
2000
2500
0
200
400
600
800
gi393740799 position (nts)

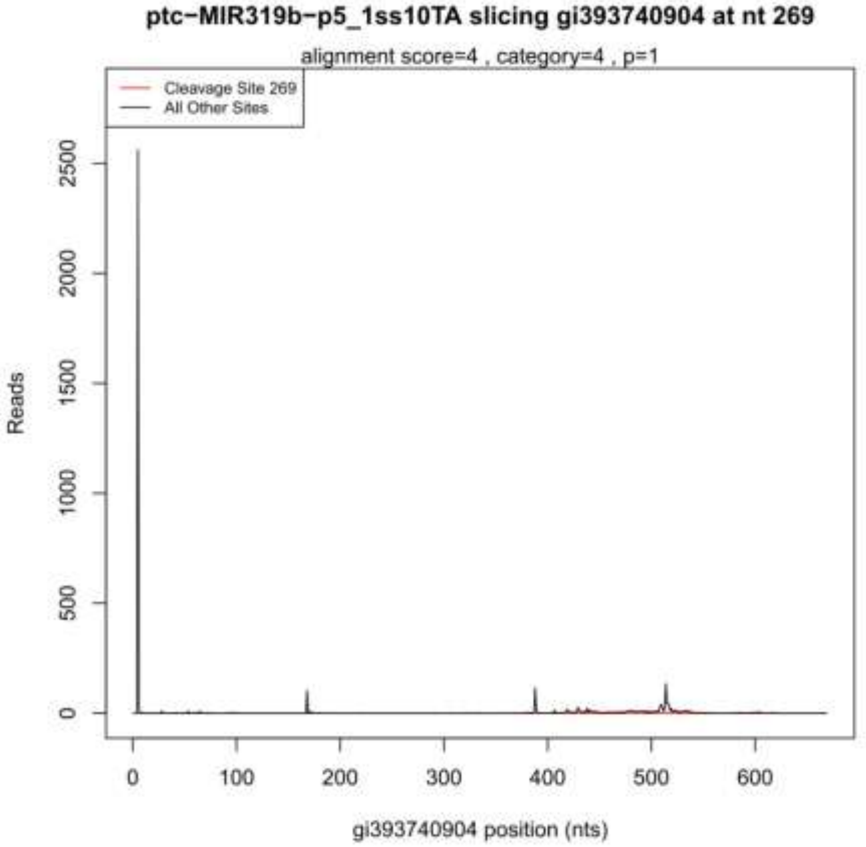
ptc−MIR319b−p5_1ss10TA slicing gi393740904 at nt 269
alignment score=4 , category=4 , p=1
Cleavage Site 269
All Other Sites
Reads
0
500
1000
1500
2000
2500
0
100
200
300
400
500
600
gi393740904 position (nts)

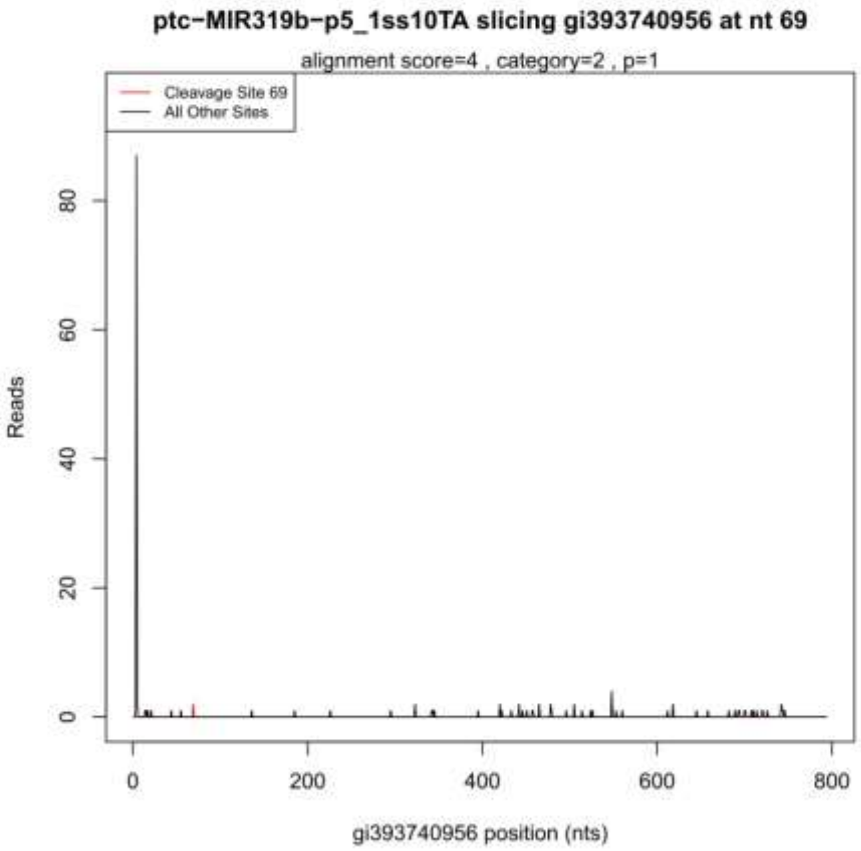
ptc−MIR319b−p5_1ss10TA slicing gi393740956 at nt 69
alignment score=4 , category=2 , p=1
Cleavage Site 69
All Other Sites
Reads
gi393740956 position (nts)
0
20
40
60
80
200
400
600
800

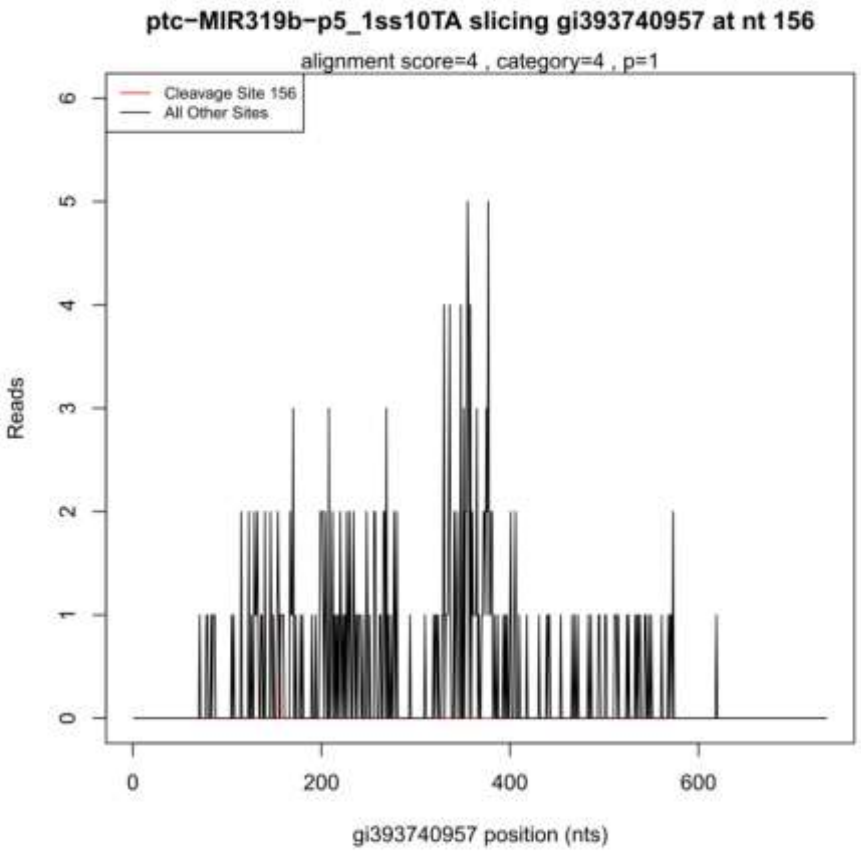
ptc−MIR319b−p5_1ss10TA slicing gi393740957 at nt 156
alignment score=4 , category=4 , p=1
Cleavage Site 156
All Other Sites
Reads
gi393740957 position (nts)
0
1
2
3
4
5
6
200
400
600

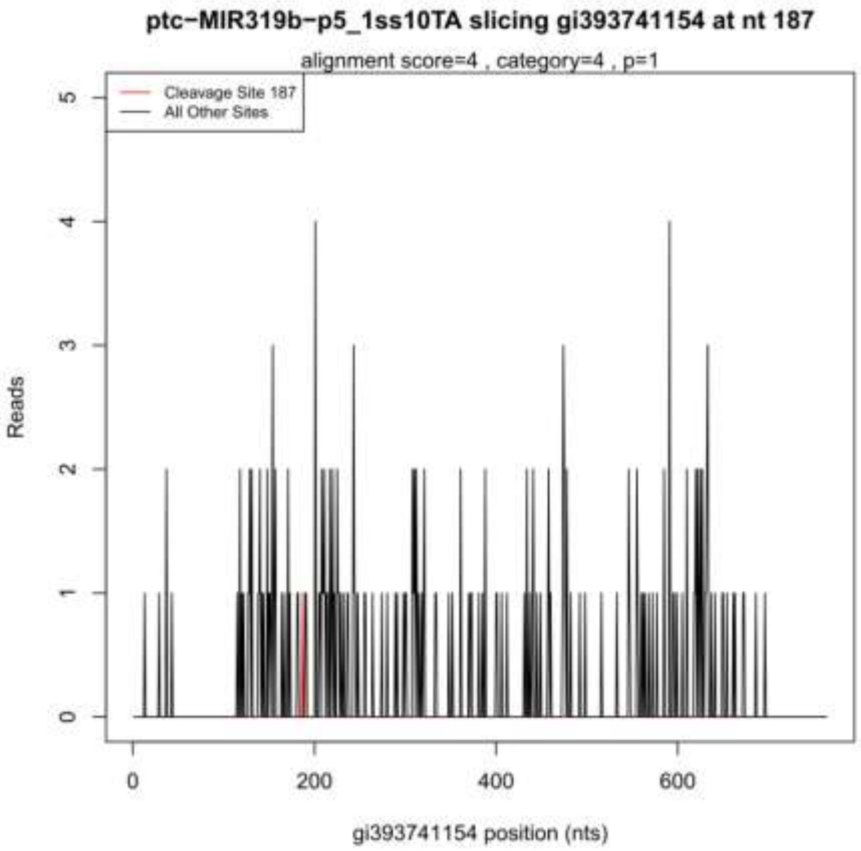

ptc−MIR319b−p5_1ss10TA slicing gi393741154 at nt 187
alignment score=4 , category=4 , p=1
Cleavage Site 187
All Other Sites
Reads
0
1
2
3
4
5
0
200
400
600
gi393741154 position (nts)

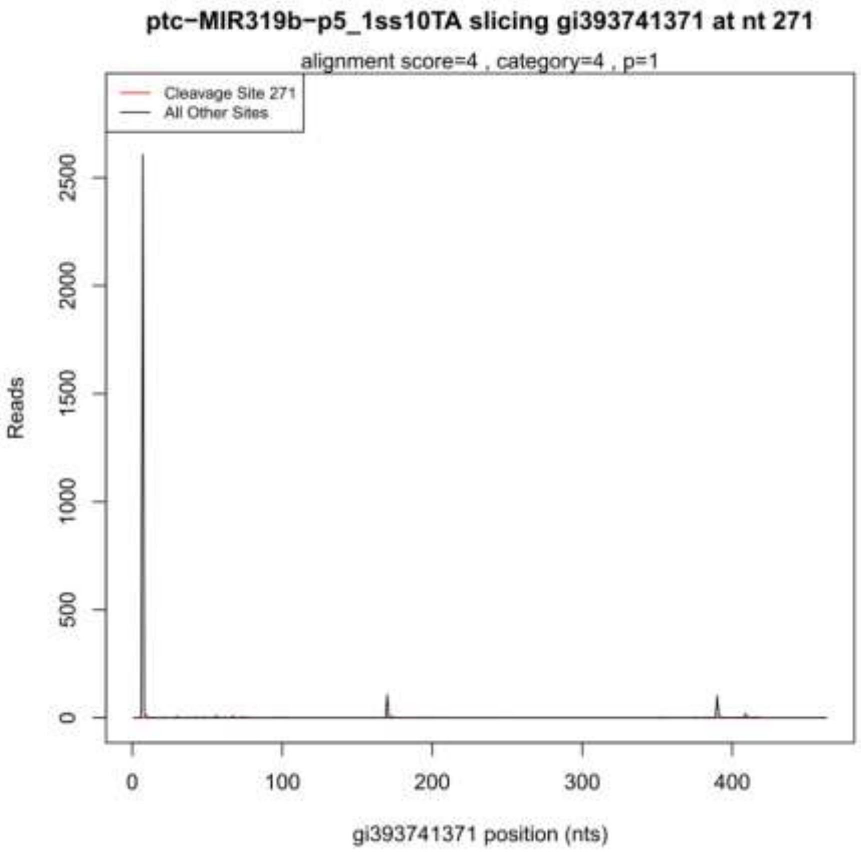

ptc−MIR319b−p5_1ss10TA slicing gi393741371 at nt 271
alignment score=4 , category=4 , p=1
Cleavage Site 271
All Other Sites
Reads
0
500
1000
1500
2000
2500
0
100
200
300
400
gi393741371 position (nts)

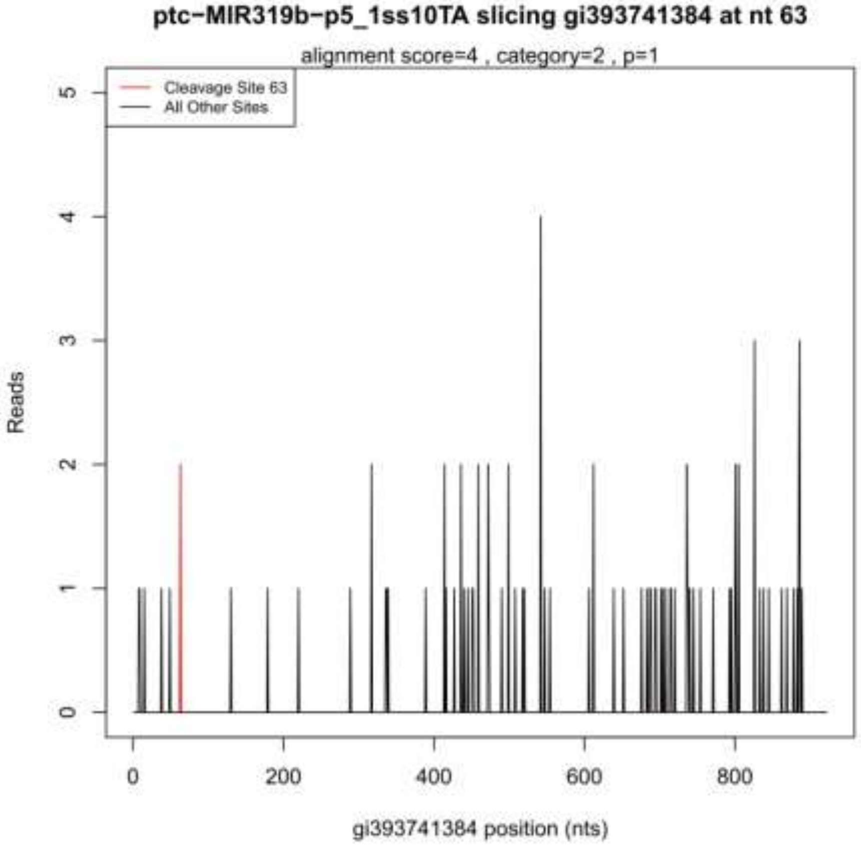

ptc-MIR319b-p5_1ss10TA slicing gi393741384 at nt 63
alignment score=4 , category=2 , p=1
Cleavage Site 63
All Other Sites
Reads
0
1
2
3
4
5
0
200
400
600
800
gi393741384 position (nts)

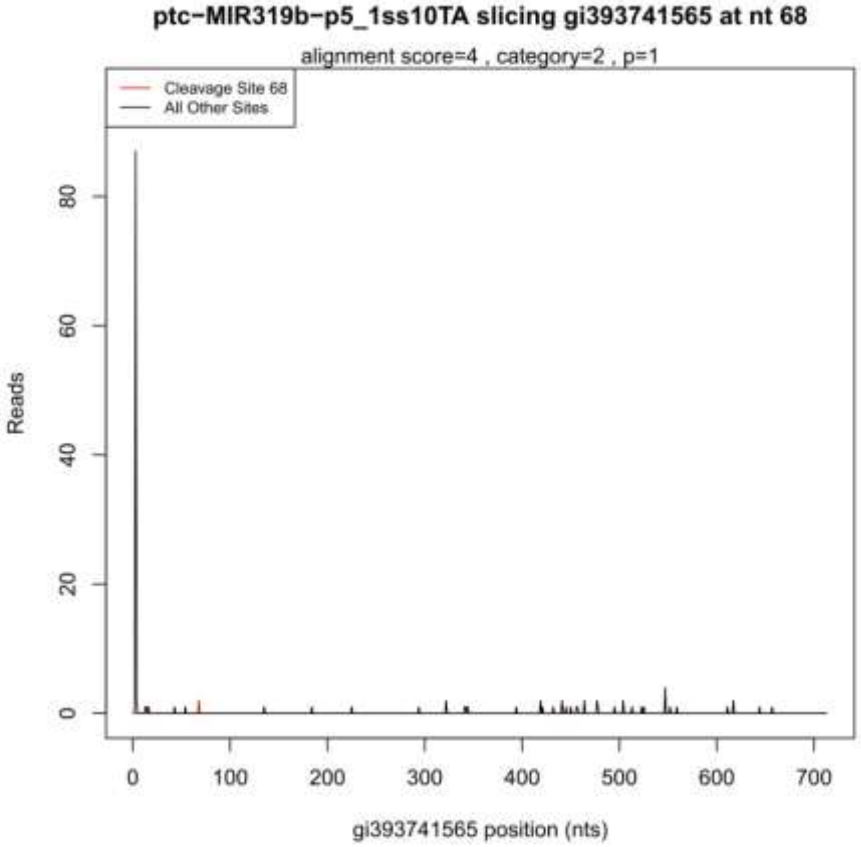

ptc-MIR319b-p5_1ss10TA slicing gi393741565 at nt 68
alignment score=4 , category=2 , p=1
Cleavage Site 68
All Other Sites
Reads
0
20
40
60
80
0
100
200
300
400
500
600
700
gi393741565 position (nts)

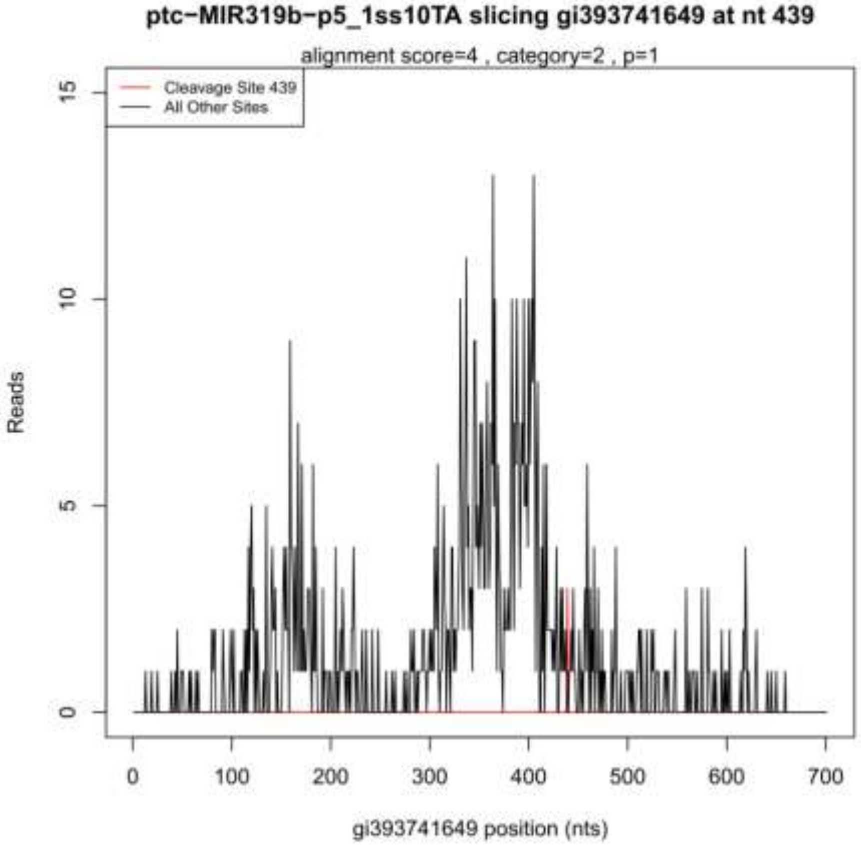
ptc−MIR319b−p5_1ss10TA slicing gi393741649 at nt 439
alignment score=4 , category=2 , p=1
Cleavage Site 439
All Other Sites
Reads
0
5
10
15
0
100
200
300
400
500
600
700
gi393741649 position (nts)

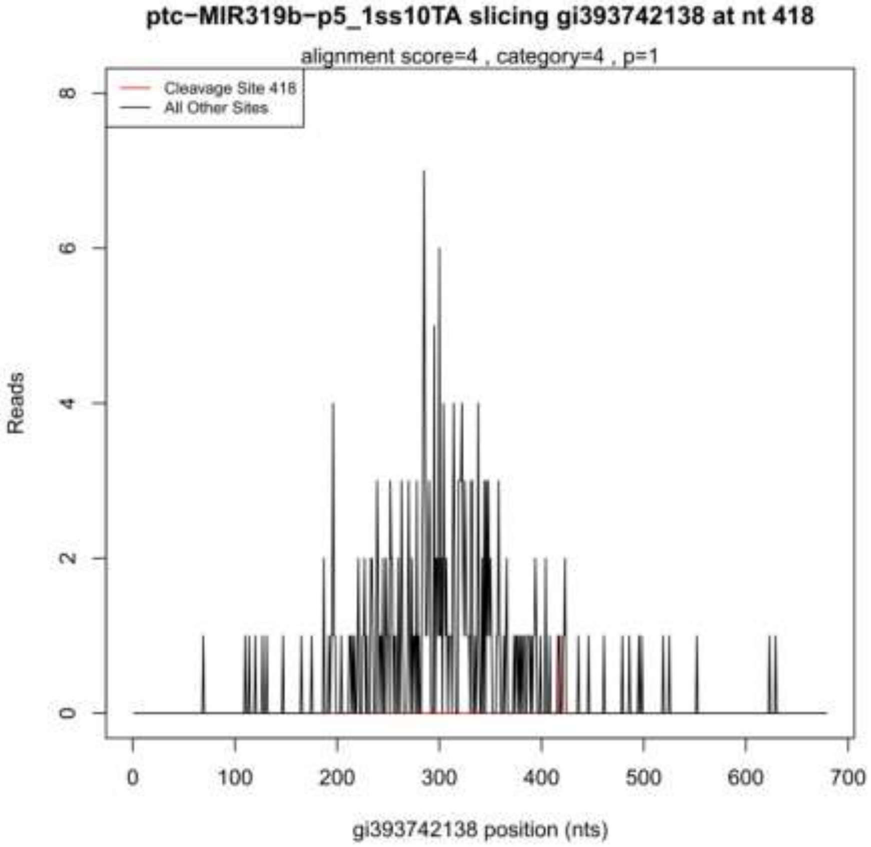
ptc−MIR319b−p5_1ss10TA slicing gi393742138 at nt 418
alignment score=4 , category=4 , p=1
Cleavage Site 418
All Other Sites
Reads
0
2
4
6
8
0
100
200
300
400
500
600
700
gi393742138 position (nts)

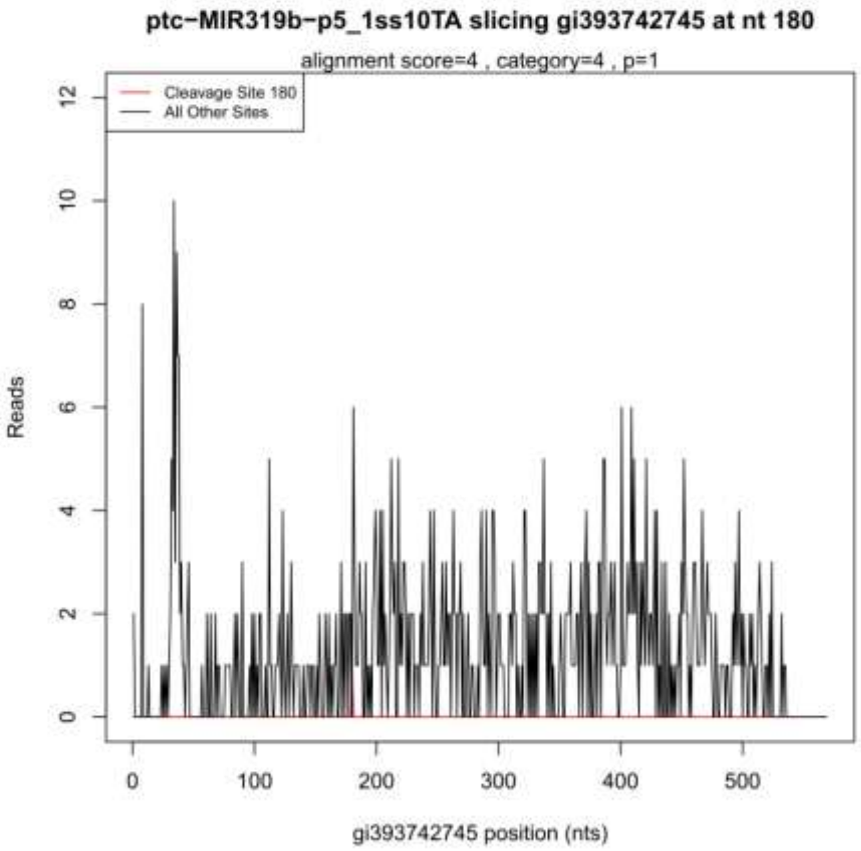

ptc-MIR319b-p5_1ss10TA slicing gi393742745 at nt 180
alignment score=4 , category=4 , p=1
Cleavage Site 180
All Other Sites
Reads
0
2
4
6
8
10
12
0
100
200
300
400
500
gi393742745 position (nts)

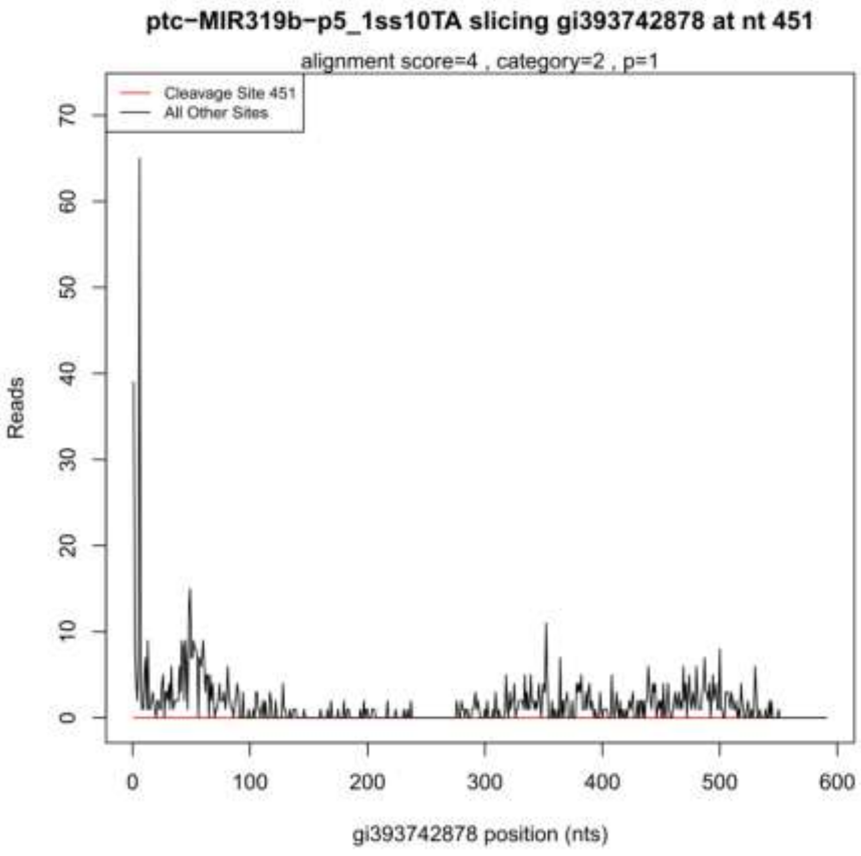

ptc-MIR319b-p5_1ss10TA slicing gi393742878 at nt 451
alignment score=4 , category=2 , p=1
Cleavage Site 451
All Other Sites
Reads
0
10
20
30
40
50
60
70
0
100
200
300
400
500
600
gi393742878 position (nts)

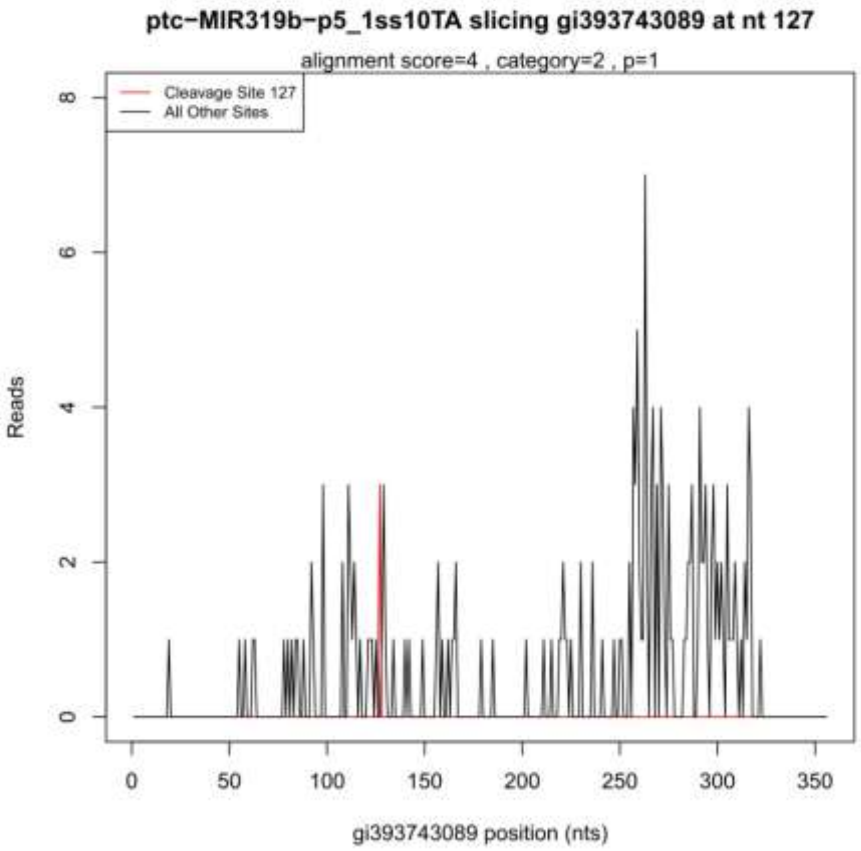
ptc−MIR319b−p5_1ss10TA slicing gi393743089 at nt 127
alignment score=4 , category=2 , p=1
Cleavage Site 127
All Other Sites
Reads
0
2
4
6
8
0
50
100
150
200
250
300
350
gi393743089 position (nts)

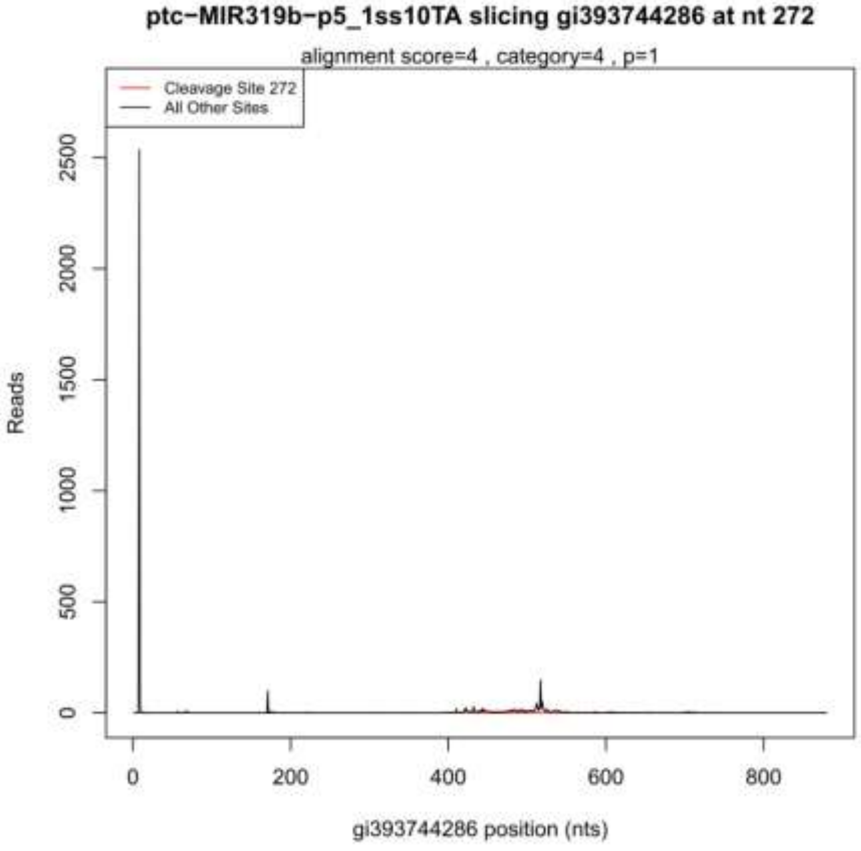
ptc−MIR319b−p5_1ss10TA slicing gi393744286 at nt 272
alignment score=4 , category=4 , p=1
Cleavage Site 272
All Other Sites
Reads
0
500
1000
1500
2000
2500
0
200
400
600
800
gi393744286 position (nts)

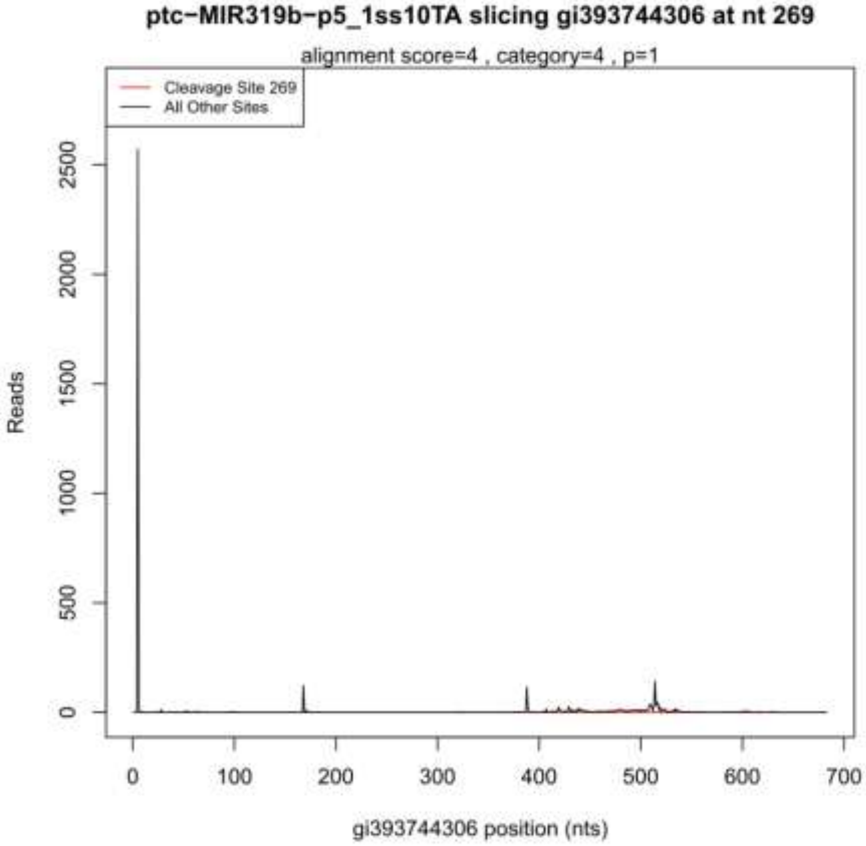

ptc−MIR319b−p5_1ss10TA slicing gi393744306 at nt 269
alignment score=4 , category=4 , p=1
Cleavage Site 269
All Other Sites
Reads
gi393744306 position (nts)

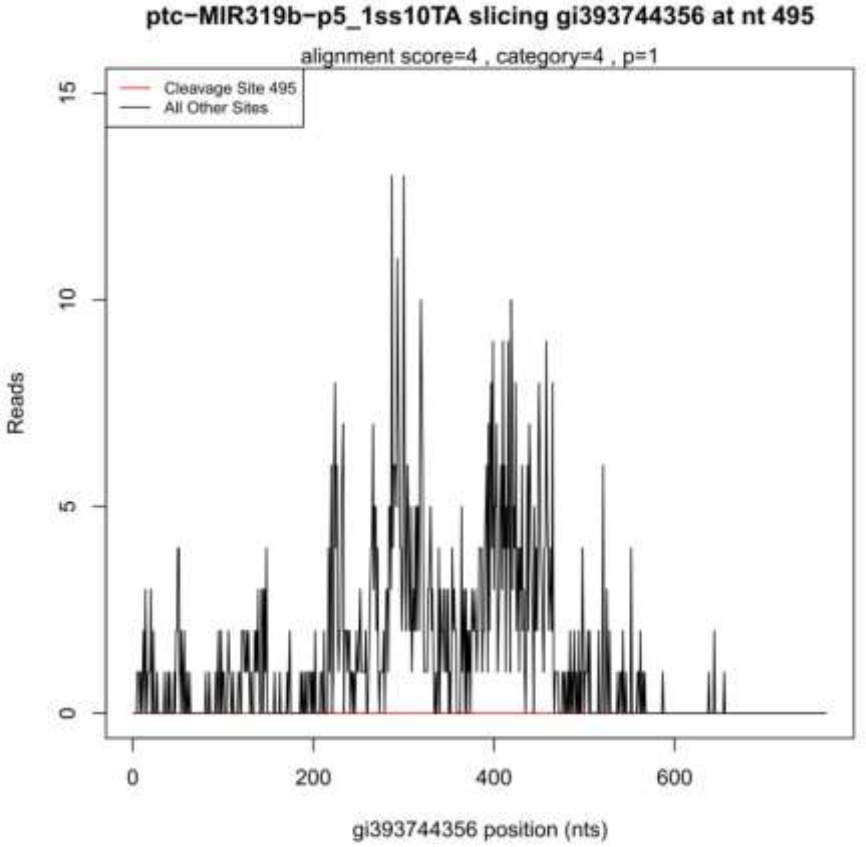

ptc−MIR319b−p5_1ss10TA slicing gi393744356 at nt 495
alignment score=4 , category=4 , p=1
Cleavage Site 495
All Other Sites
Reads
gi393744356 position (nts)

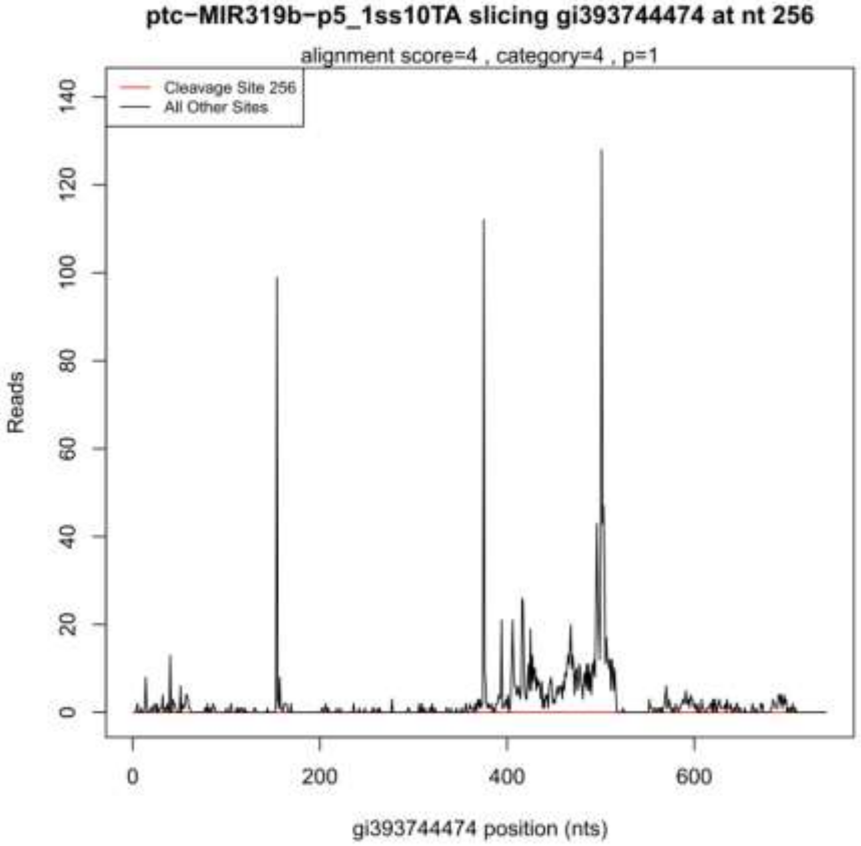
ptc−MIR319b−p5_1ss10TA slicing gi393744474 at nt 256
alignment score=4 , category=4 , p=1
Cleavage Site 256
All Other Sites
Reads
0
20
40
60
80
100
120
140
0
200
400
600
gi393744474 position (nts)

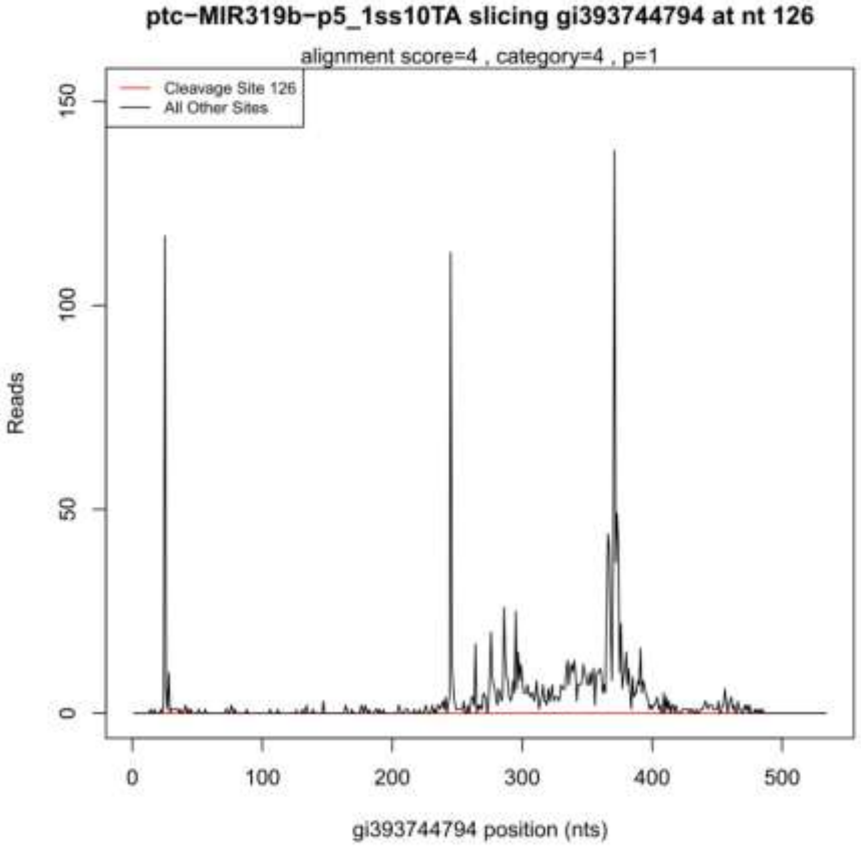
ptc−MIR319b−p5_1ss10TA slicing gi393744794 at nt 126
alignment score=4 , category=4 , p=1
Cleavage Site 126
All Other Sites
Reads
0
50
100
150
0
100
200
300
400
500
gi393744794 position (nts)

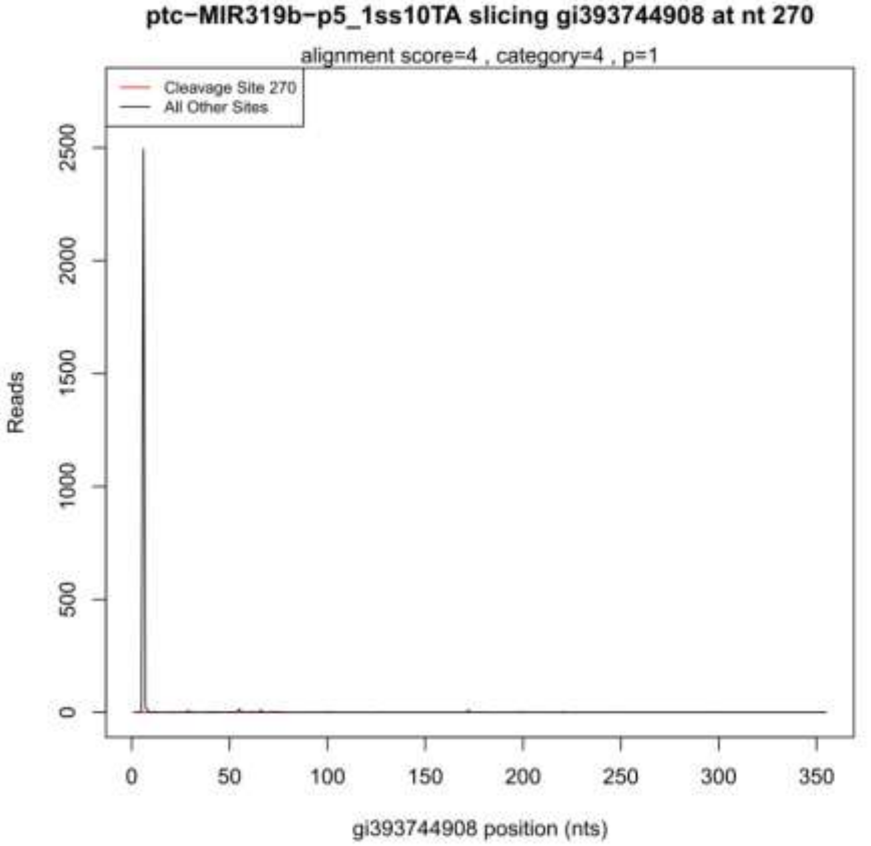
ptc−MIR319b−p5_1ss10TA slicing gi393744908 at nt 270
alignment score=4 , category=4 , p=1
Cleavage Site 270
All Other Sites
Reads
0
500
1000
1500
2000
2500
0
50
100
150
200
250
300
350
gi393744908 position (nts)

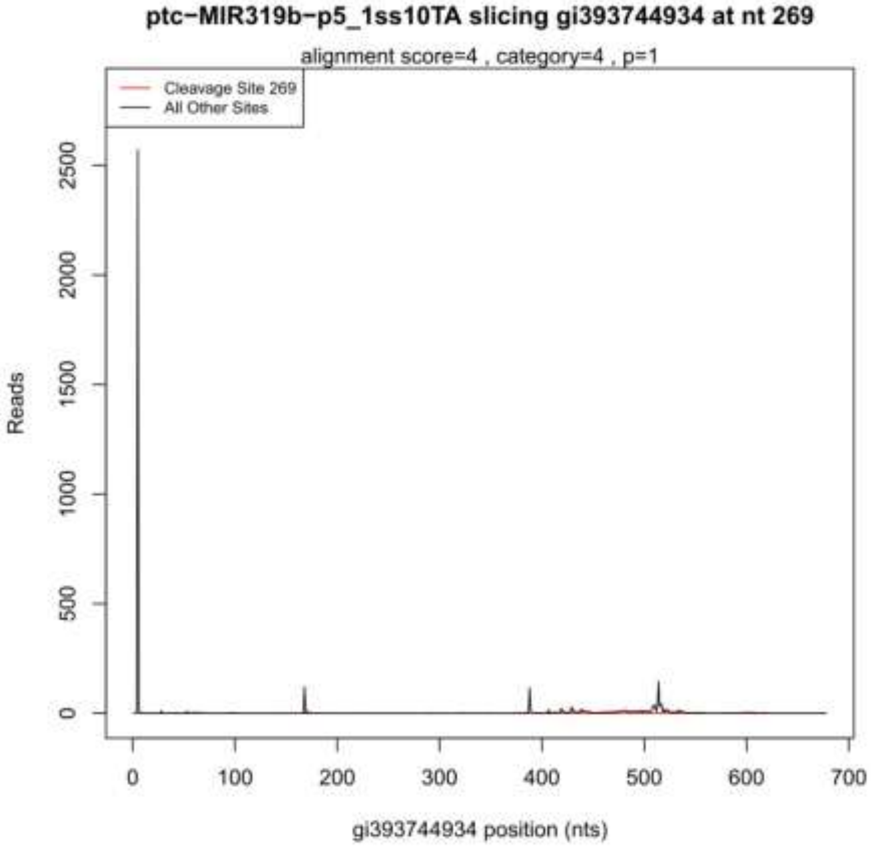
ptc−MIR319b−p5_1ss10TA slicing gi393744934 at nt 269
alignment score=4 , category=4 , p=1
Cleavage Site 269
All Other Sites
Reads
0
500
1000
1500
2000
2500
0
100
200
300
400
500
600
700
gi393744934 position (nts)

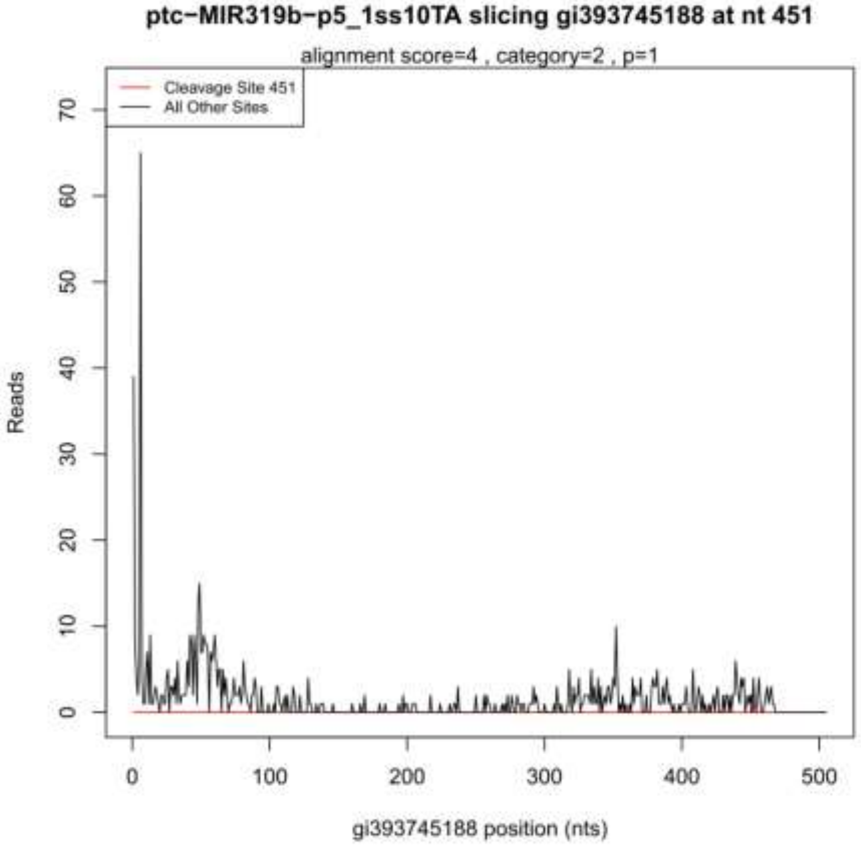
ptc−MIR319b−p5_1ss10TA slicing gi393745188 at nt 451
alignment score=4 , category=2 , p=1
Cleavage Site 451
All Other Sites
Reads
0
10
20
30
40
50
60
70
0
100
200
300
400
500
gi393745188 position (nts)

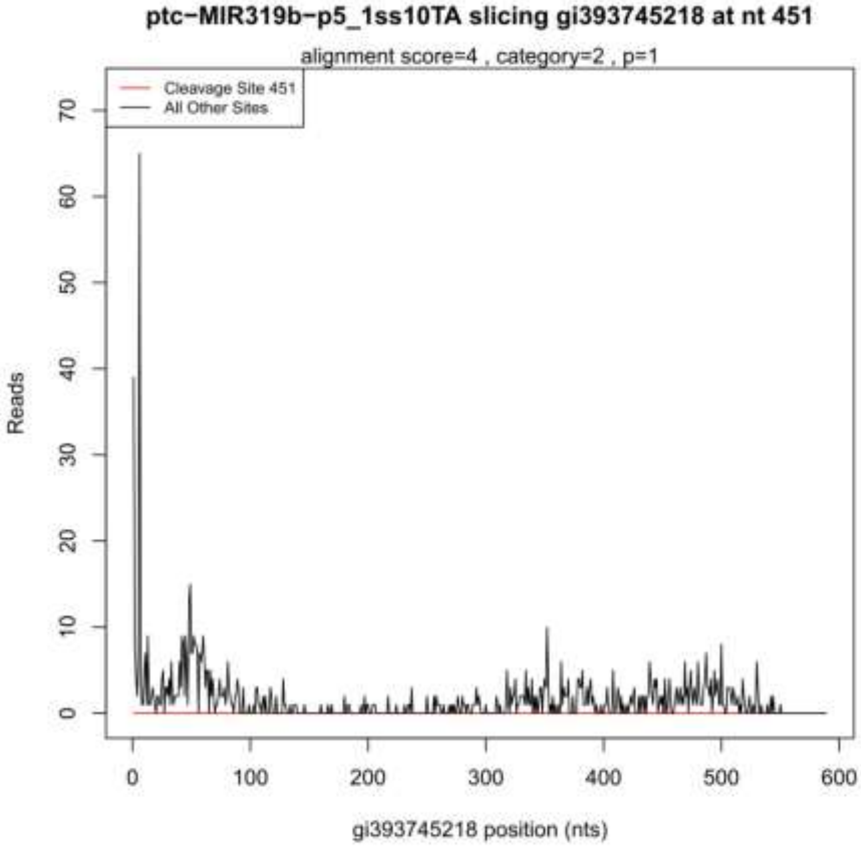
ptc−MIR319b−p5_1ss10TA slicing gi393745218 at nt 451
alignment score=4 , category=2 , p=1
Cleavage Site 451
All Other Sites
Reads
0
10
20
30
40
50
60
70
0
100
200
300
400
500
600
gi393745218 position (nts)

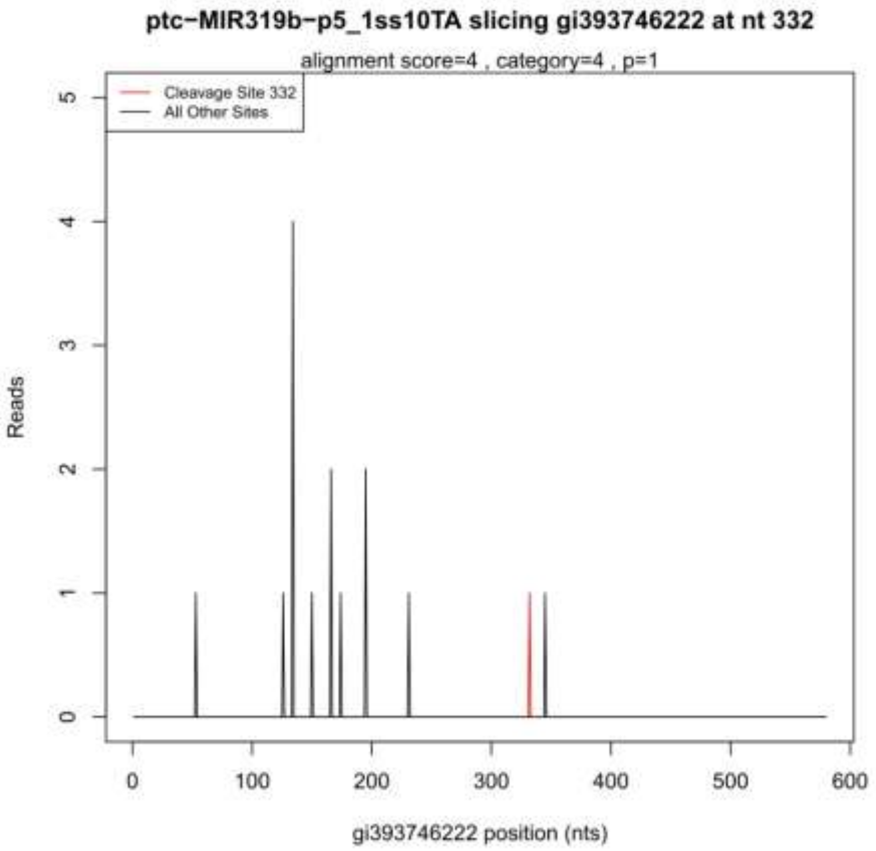
ptc−MIR319b−p5_1ss10TA slicing gi393746222 at nt 332
alignment score=4 , category=4 , p=1
Cleavage Site 332
All Other Sites
Reads
0
1
2
3
4
5
0
100
200
300
400
500
600
gi393746222 position (nts)

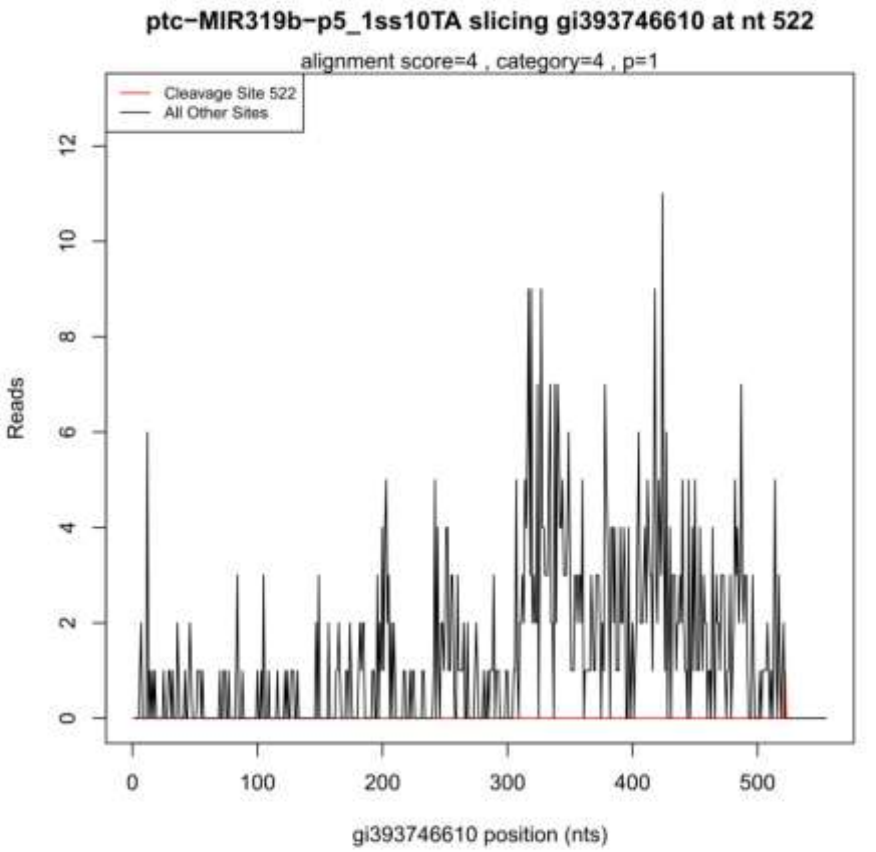
ptc−MIR319b−p5_1ss10TA slicing gi393746610 at nt 522
alignment score=4 , category=4 , p=1
Cleavage Site 522
All Other Sites
Reads
0
2
4
6
8
10
12
0
100
200
300
400
500
gi393746610 position (nts)

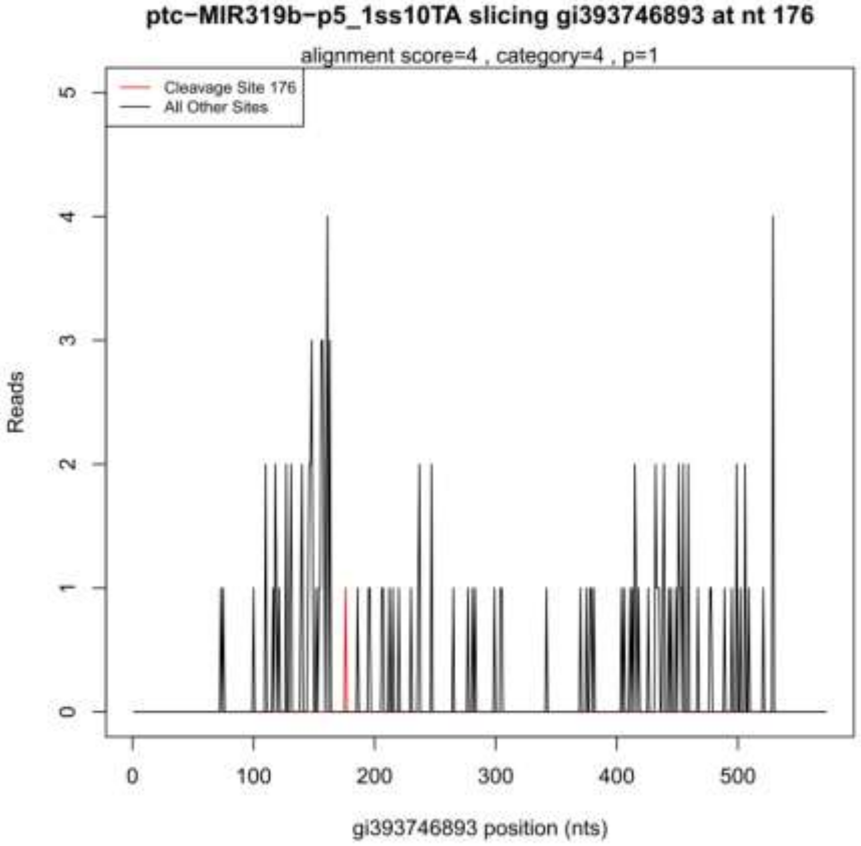
ptc−MIR319b−p5_1ss10TA slicing gi393746893 at nt 176
alignment score=4 , category=4 , p=1
Cleavage Site 176
All Other Sites
Reads
0
1
2
3
4
5
0
100
200
300
400
500
gi393746893 position (nts)

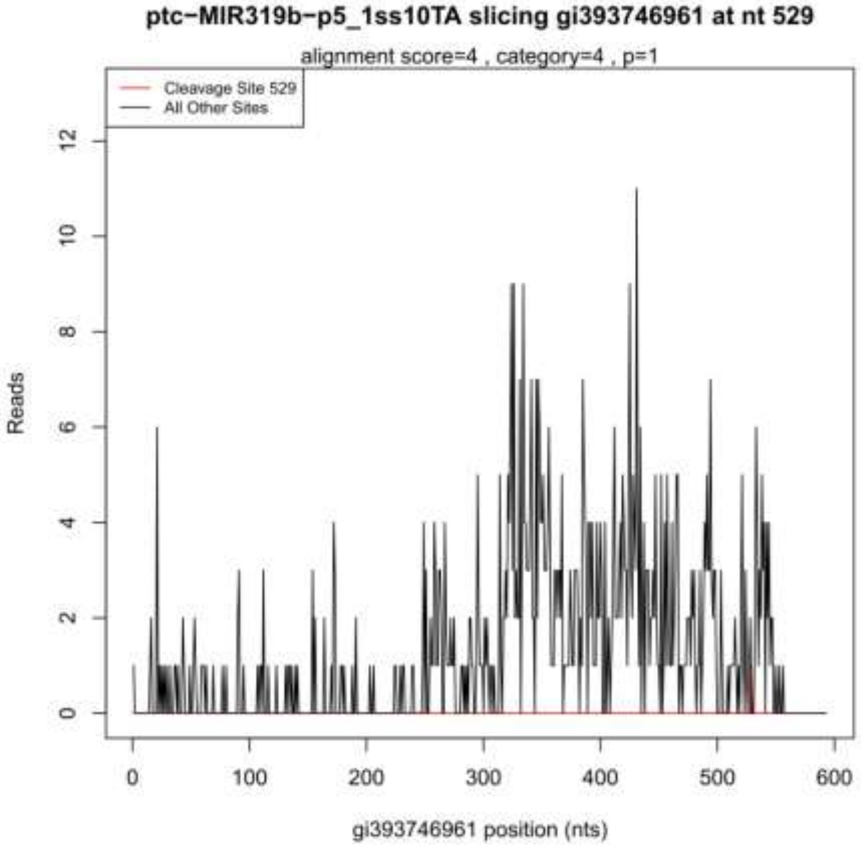
ptc−MIR319b−p5_1ss10TA slicing gi393746961 at nt 529
alignment score=4 , category=4 , p=1
Cleavage Site 529
All Other Sites
Reads
0
2
4
6
8
10
12
0
100
200
300
400
500
600
gi393746961 position (nts)

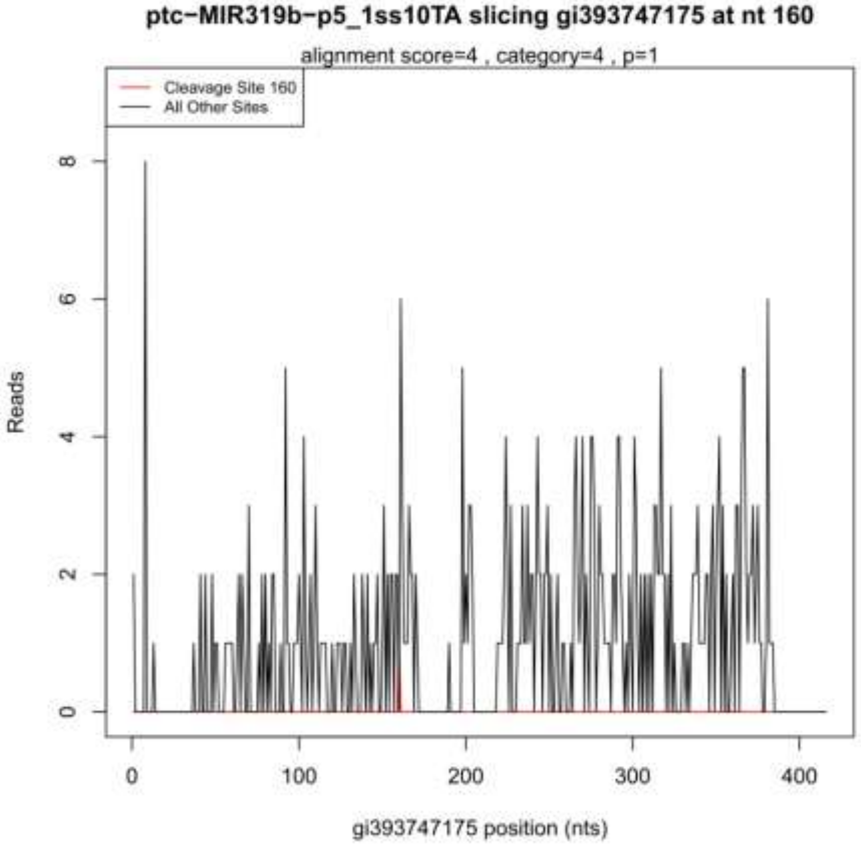

ptc−MIR319b−p5_1ss10TA slicing gi393747175 at nt 160
alignment score=4 , category=4 , p=1
Cleavage Site 160
All Other Sites
Reads
0
2
4
6
8
0
100
200
300
400
gi393747175 position (nts)

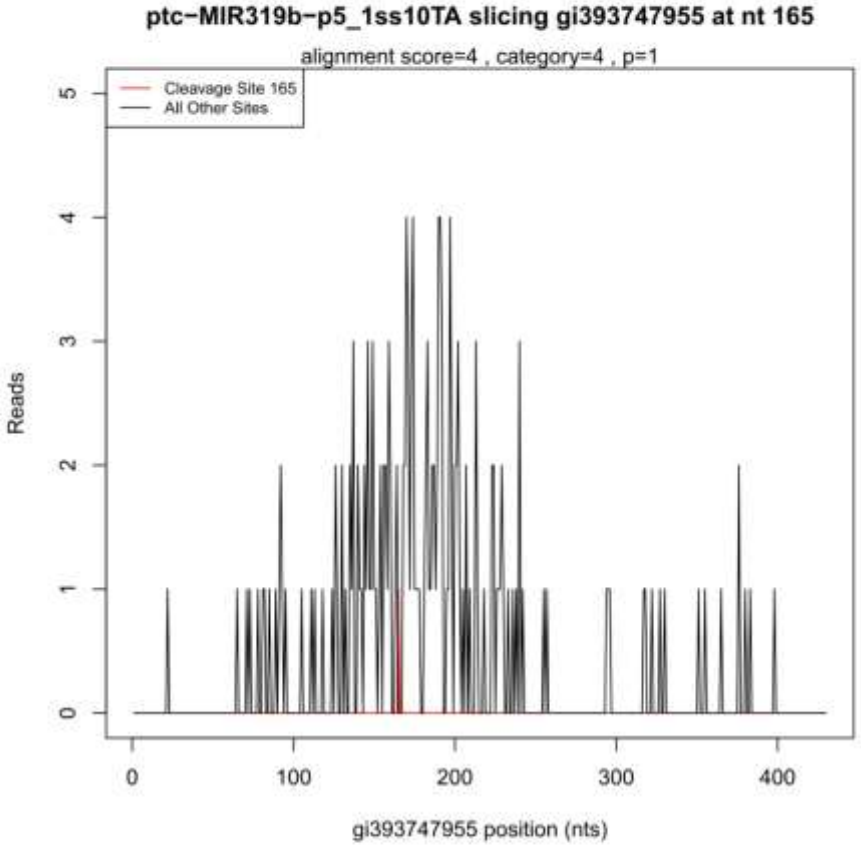

ptc−MIR319b−p5_1ss10TA slicing gi393747955 at nt 165
alignment score=4 , category=4 , p=1
Cleavage Site 165
All Other Sites
Reads
0
1
2
3
4
5
0
100
200
300
400
gi393747955 position (nts)

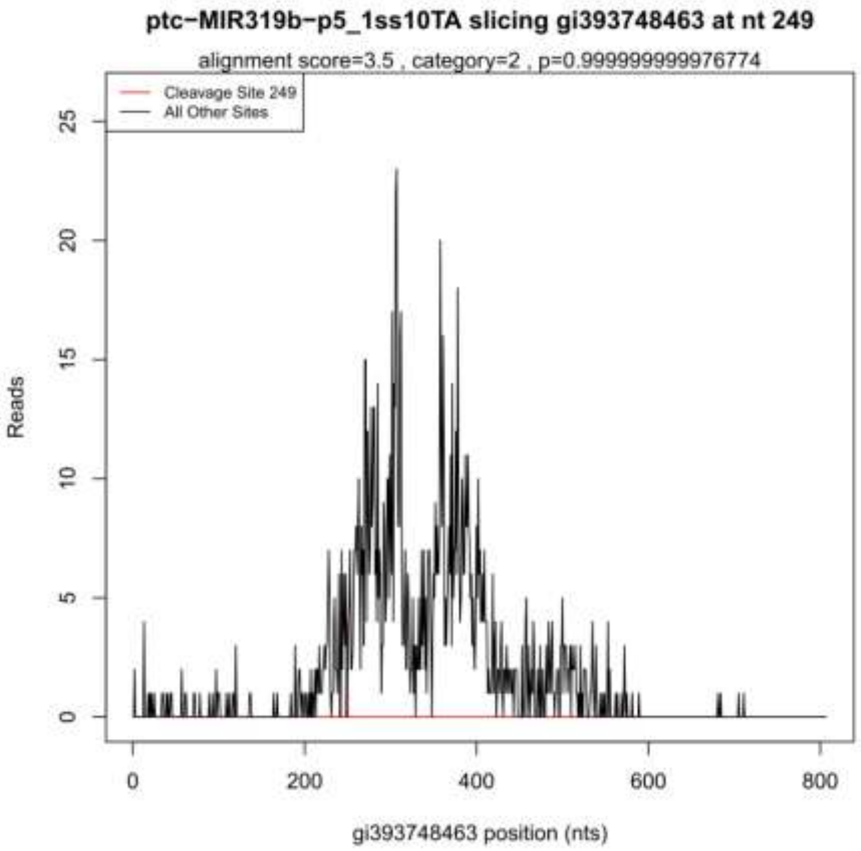
ptc−MIR319b−p5_1ss10TA slicing gi393748463 at nt 249
alignment score=3.5 , category=2 , p=0.999999999976774
Cleavage Site 249
All Other Sites
Reads
gi393748463 position (nts)

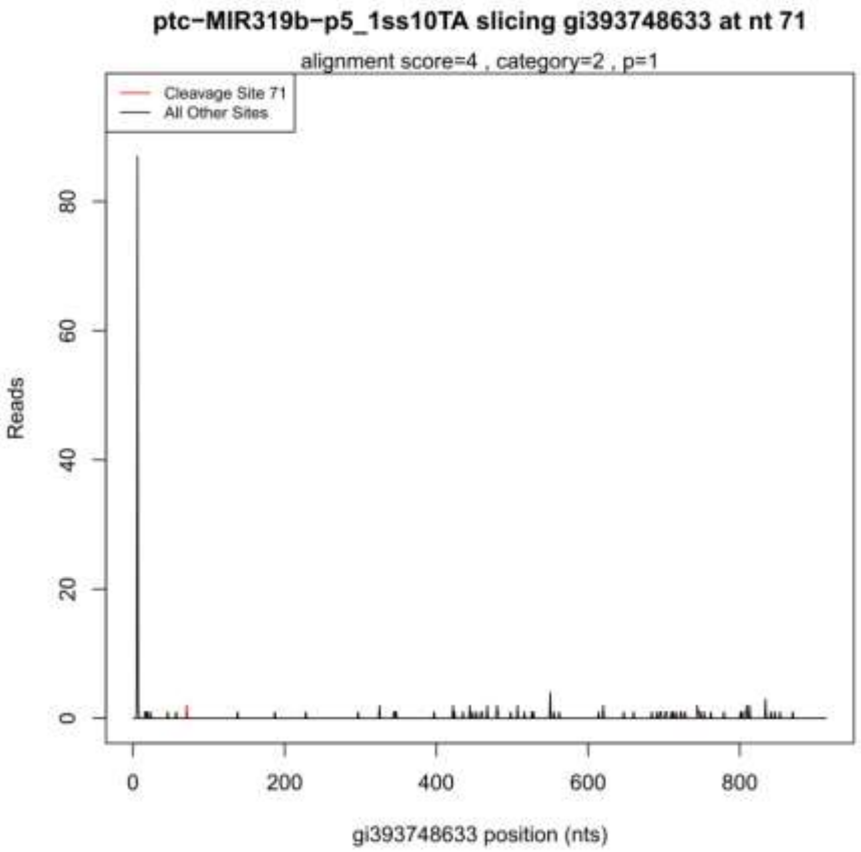
ptc−MIR319b−p5_1ss10TA slicing gi393748633 at nt 71
alignment score=4 , category=2 , p=1
Cleavage Site 71
All Other Sites
Reads
gi393748633 position (nts)

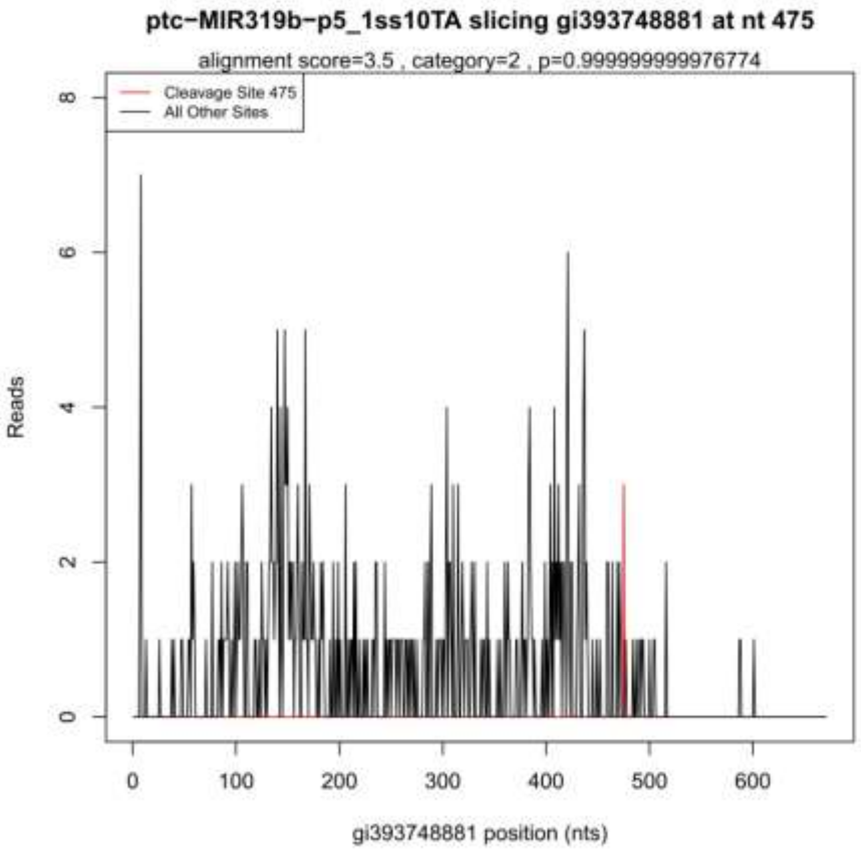
ptc−MIR319b−p5_1ss10TA slicing gi393748881 at nt 475
alignment score=3.5 , category=2 , p=0.999999999976774
Cleavage Site 475
All Other Sites
Reads
gi393748881 position (nts)
0
2
4
6
8
0
100
200
300
400
500
600

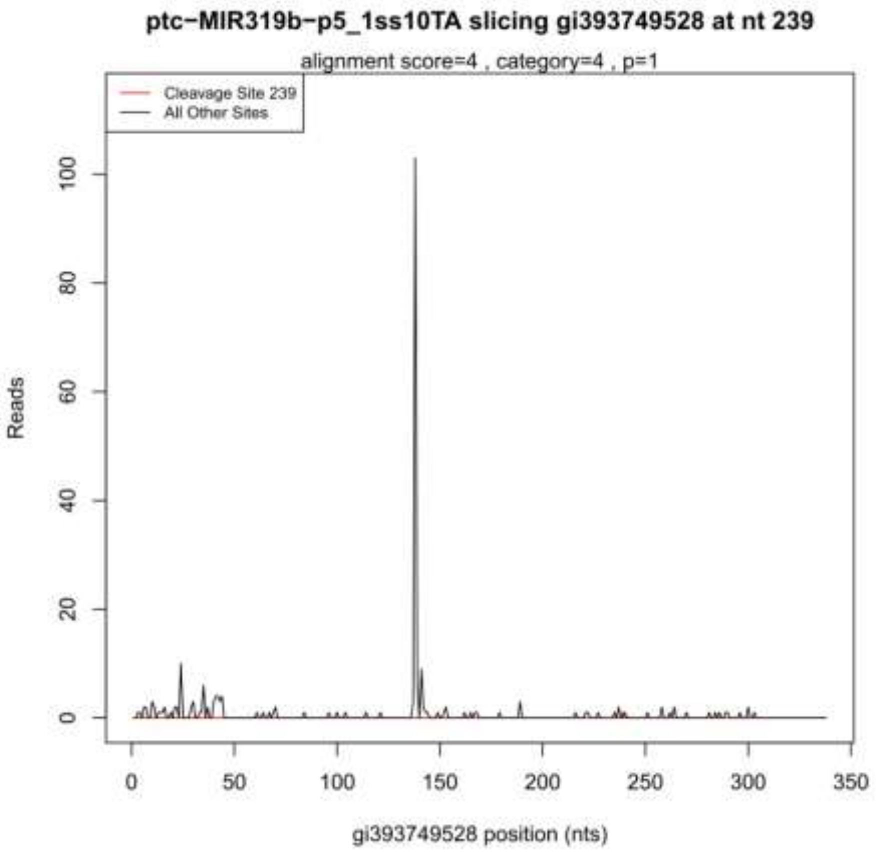
ptc−MIR319b−p5_1ss10TA slicing gi393749528 at nt 239
alignment score=4 , category=4 , p=1
Cleavage Site 239
All Other Sites
Reads
gi393749528 position (nts)
0
20
40
60
80
100
0
50
100
150
200
250
300
350

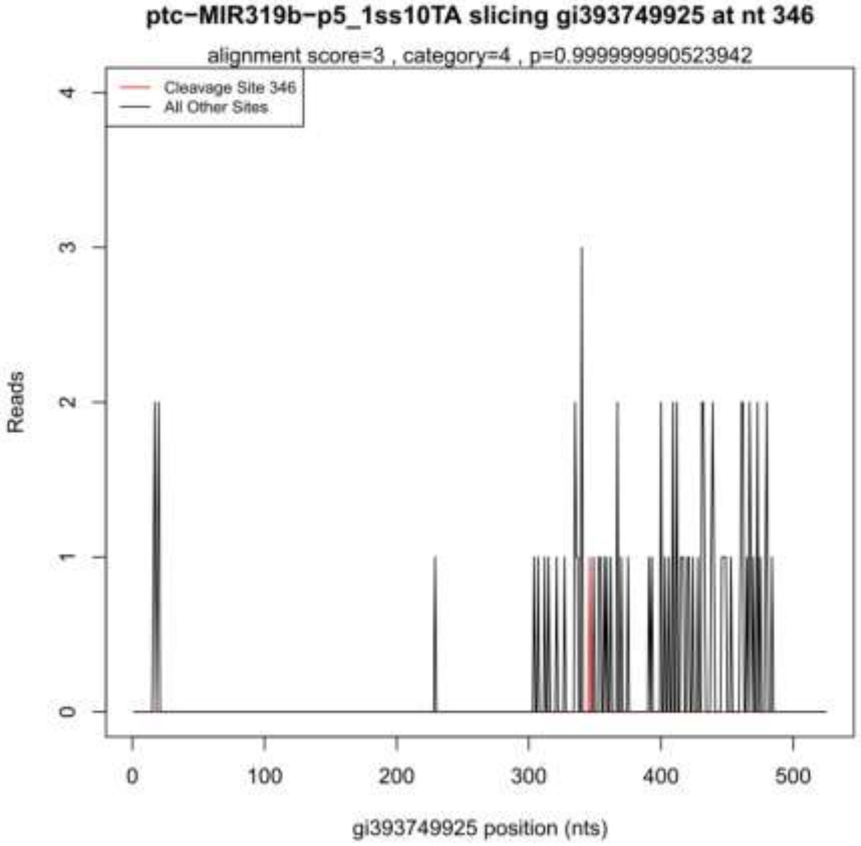

ptc−MIR319b−p5_1ss10TA slicing gi393749925 at nt 346
alignment score=3 , category=4 , p=0.999999990523942
Cleavage Site 346
All Other Sites
Reads
gi393749925 position (nts)

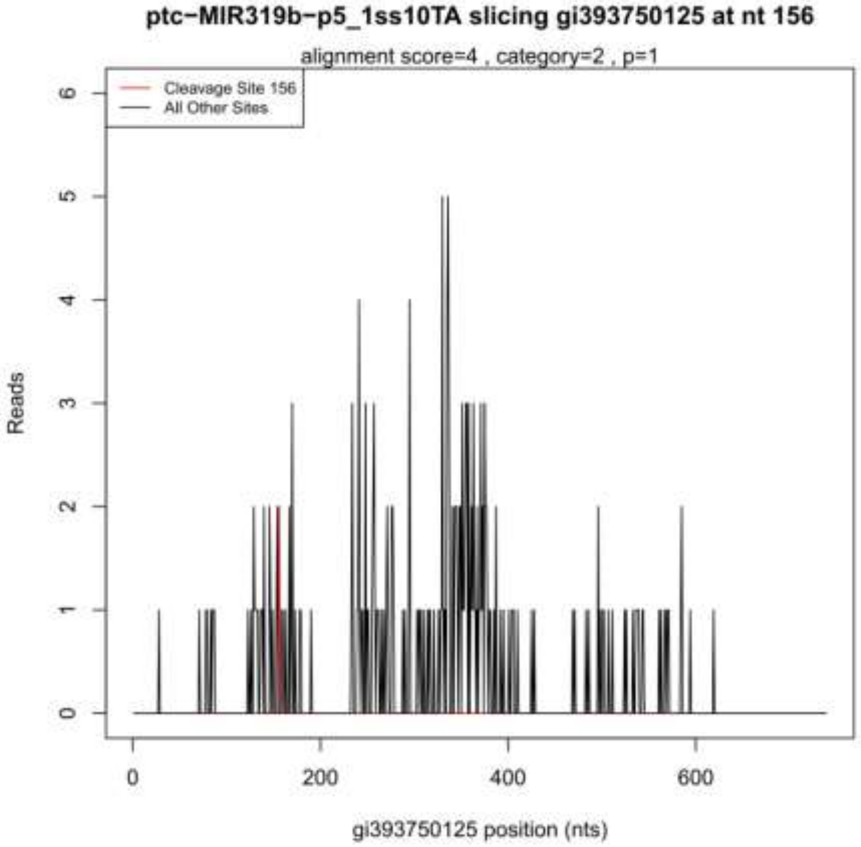

ptc−MIR319b−p5_1ss10TA slicing gi393750125 at nt 156
alignment score=4 , category=2 , p=1
Cleavage Site 156
All Other Sites
Reads
gi393750125 position (nts)

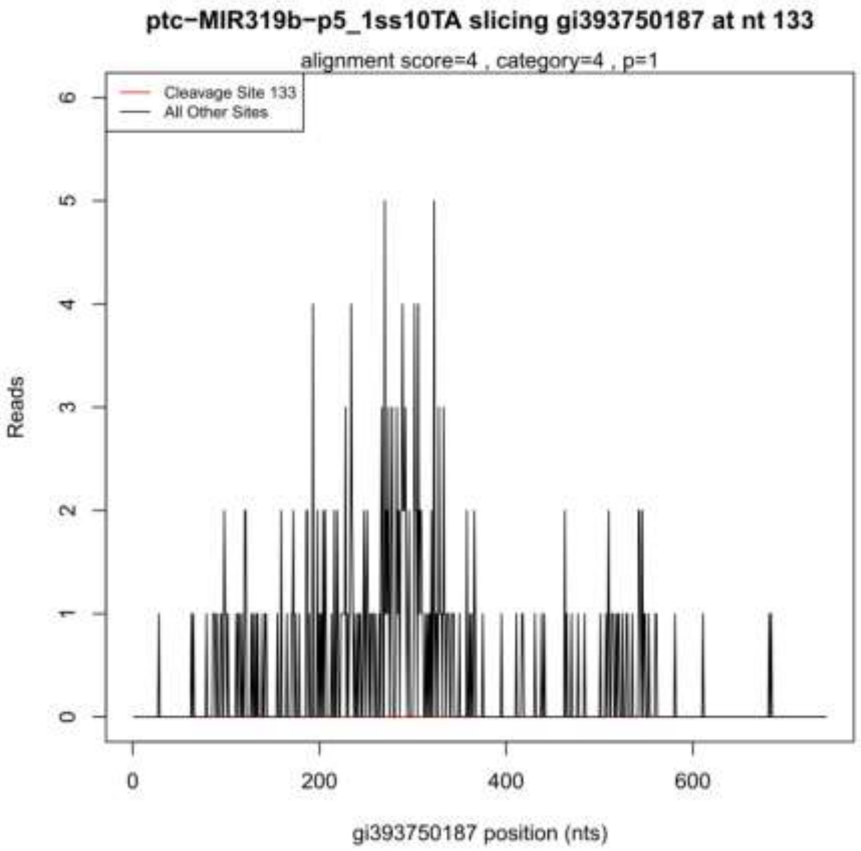
ptc−MIR319b−p5_1ss10TA slicing gi393750187 at nt 133
alignment score=4 , category=4 , p=1
Cleavage Site 133
All Other Sites
Reads
0
1
2
3
4
5
6
0
200
400
600
gi393750187 position (nts)

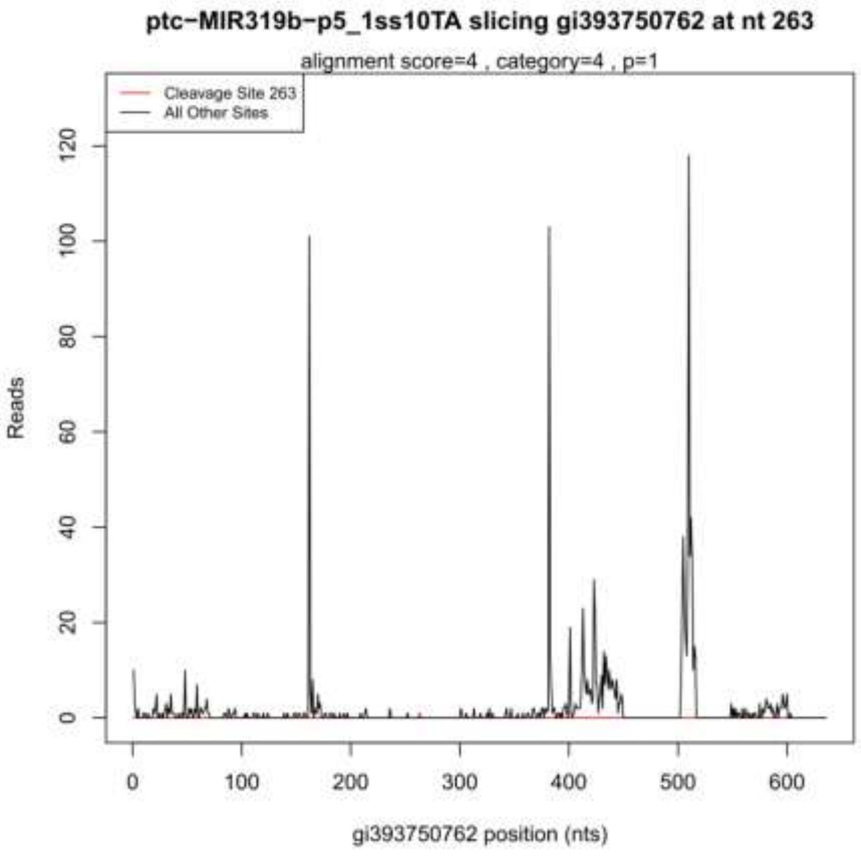
ptc−MIR319b−p5_1ss10TA slicing gi393750762 at nt 263
alignment score=4 , category=4 , p=1
Cleavage Site 263
All Other Sites
Reads
0
20
40
60
80
100
120
0
100
200
300
400
500
600
gi393750762 position (nts)

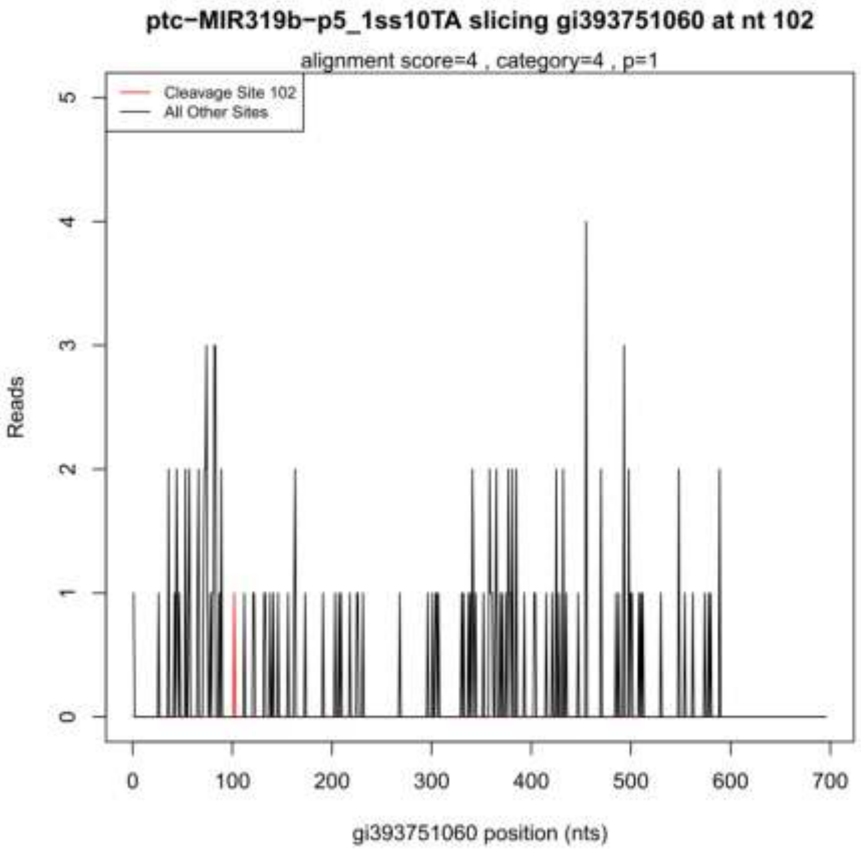
ptc−MIR319b−p5_1ss10TA slicing gi393751060 at nt 102
alignment score=4 , category=4 , p=1
Cleavage Site 102
All Other Sites
Reads
gi393751060 position (nts)
0
1
2
3
4
5
0
100
200
300
400
500
600
700

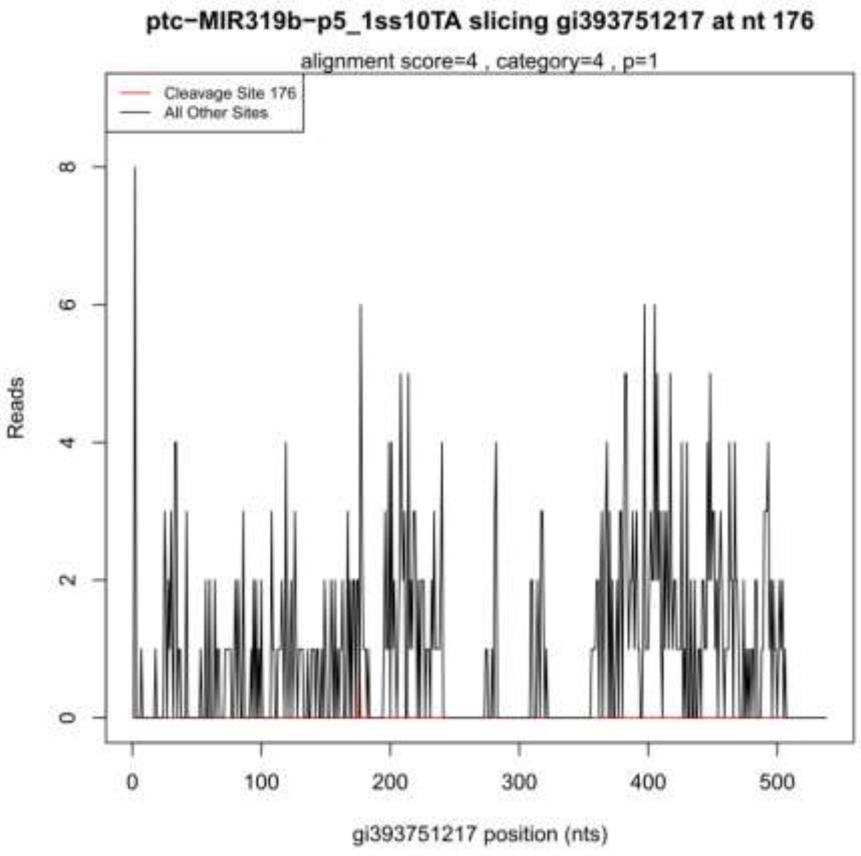
ptc−MIR319b−p5_1ss10TA slicing gi393751217 at nt 176
alignment score=4 , category=4 , p=1
Cleavage Site 176
All Other Sites
Reads
gi393751217 position (nts)
0
2
4
6
8
0
100
200
300
400
500

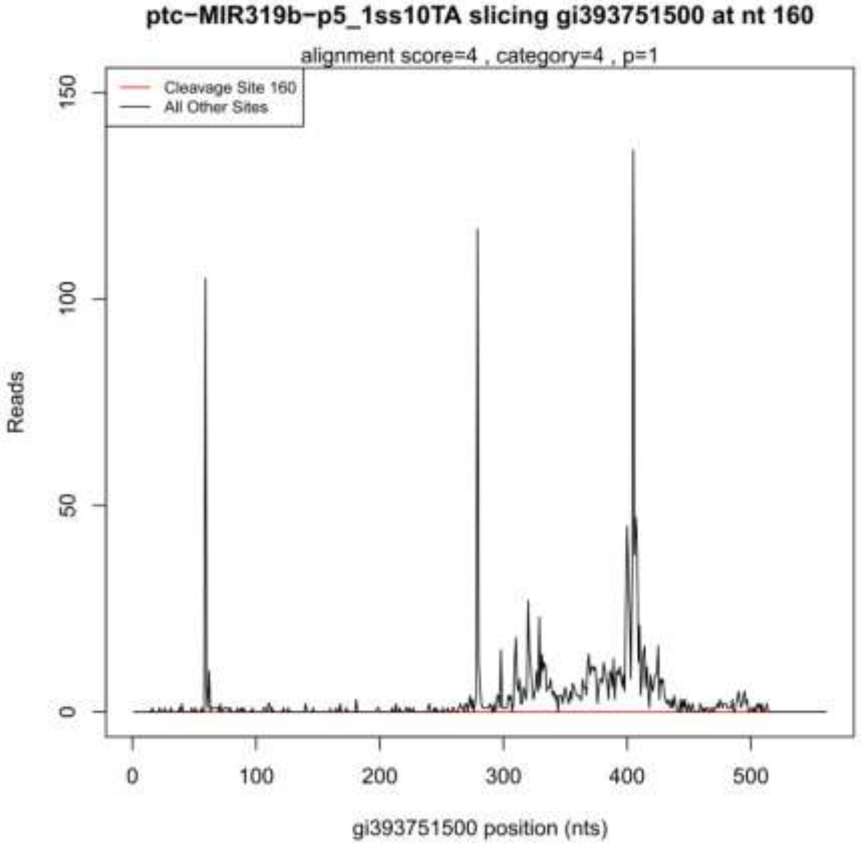
ptc−MIR319b−p5_1ss10TA slicing gi393751500 at nt 160
alignment score=4 , category=4 , p=1
Cleavage Site 160
All Other Sites
Reads
0
50
100
150
0
100
200
300
400
500
gi393751500 position (nts)

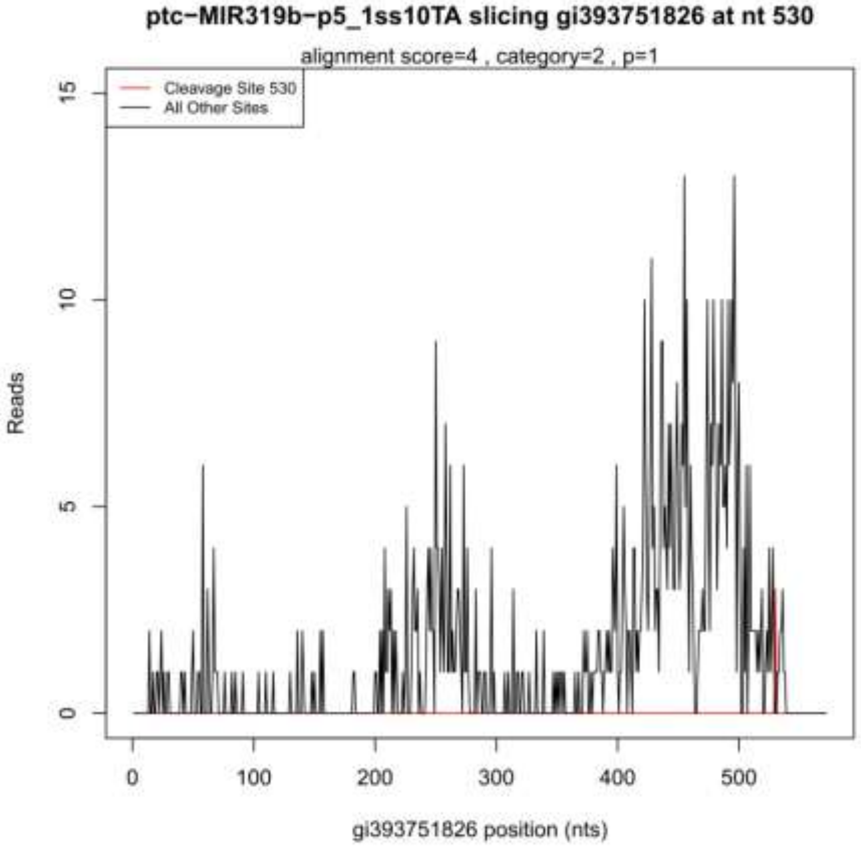
ptc−MIR319b−p5_1ss10TA slicing gi393751826 at nt 530
alignment score=4 , category=2 , p=1
Cleavage Site 530
All Other Sites
Reads
0
5
10
15
0
100
200
300
400
500
gi393751826 position (nts)

ptc-MIR319b-p5_1ss10TA slicing gi393751982 at nt 523

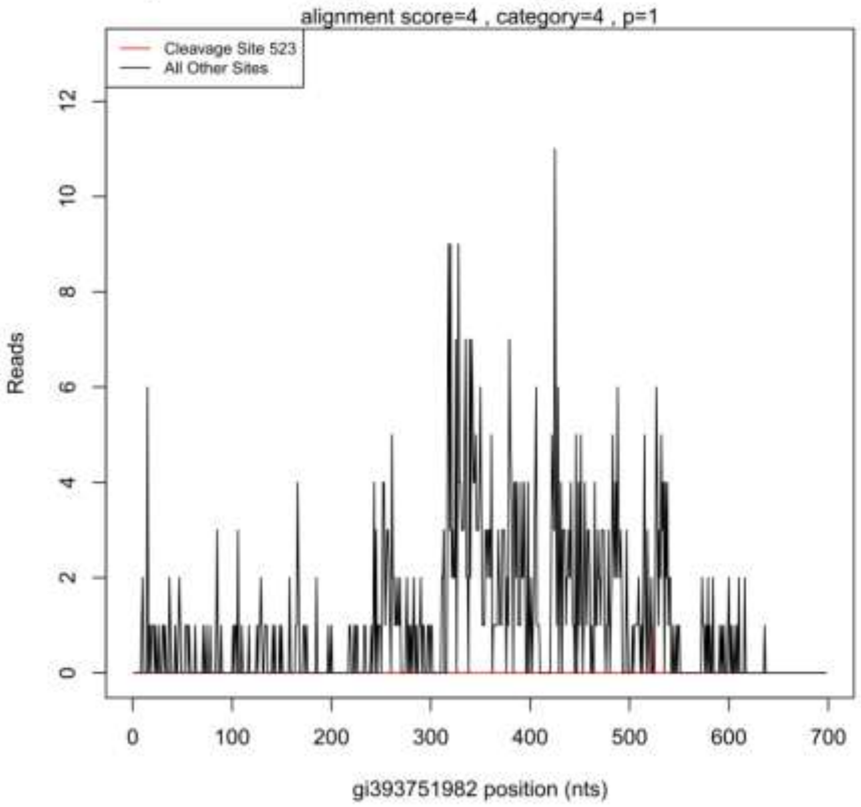

ptc-MIR319b-p5_1ss10TA slicing gi393752334 at nt 150

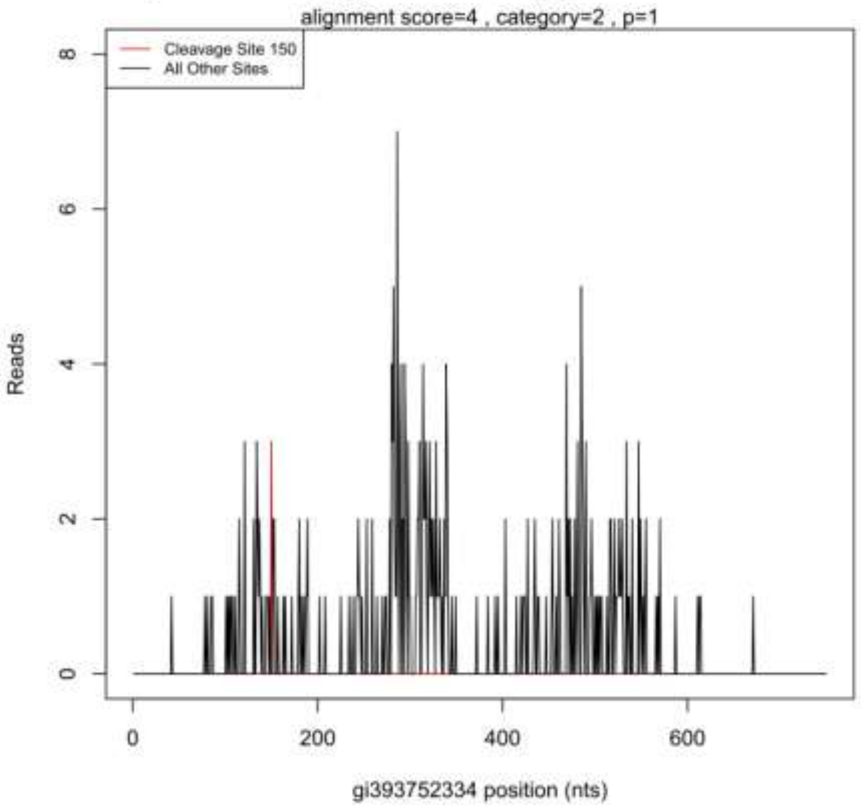

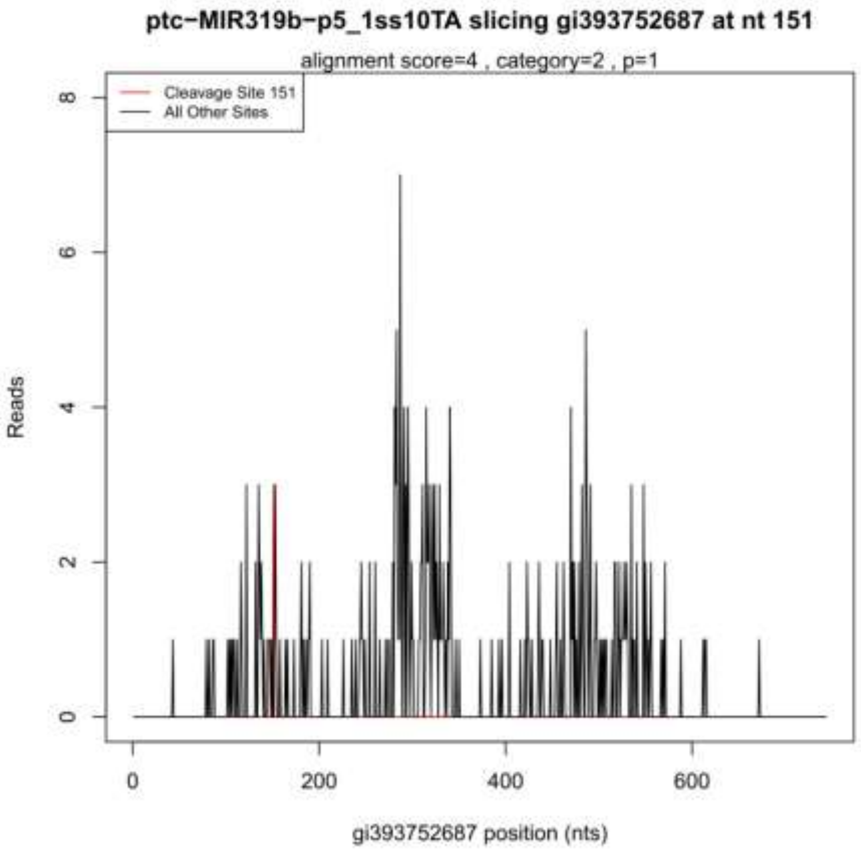
ptc−MIR319b−p5_1ss10TA slicing gi393752687 at nt 151
alignment score=4 , category=2 , p=1
Cleavage Site 151
All Other Sites
Reads
0
2
4
6
8
0
200
400
600
gi393752687 position (nts)

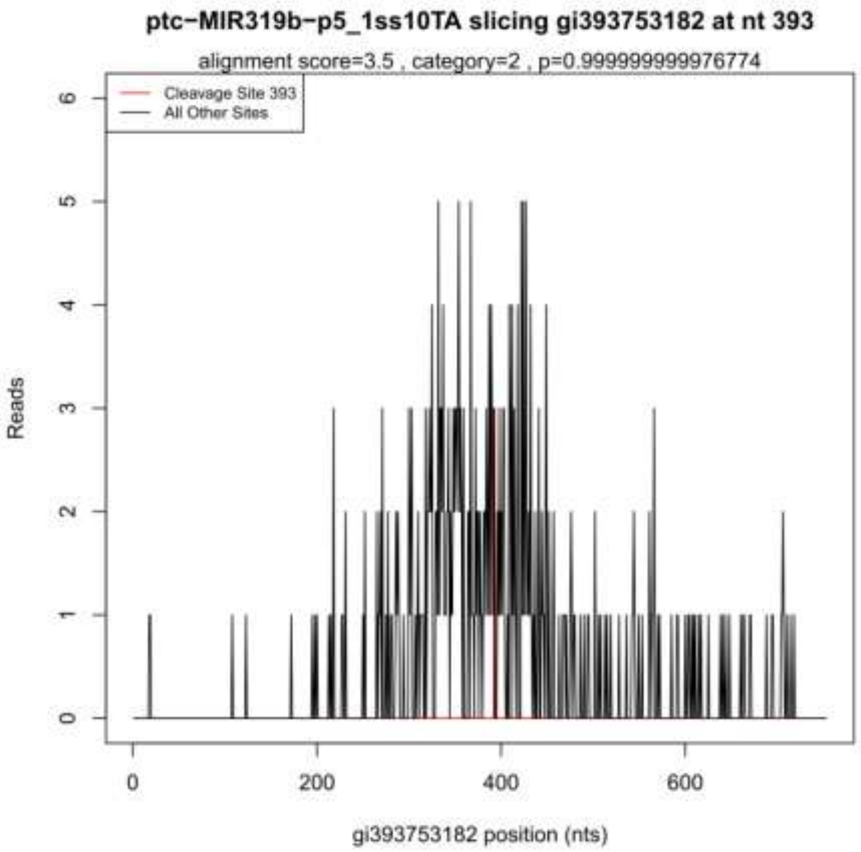
ptc−MIR319b−p5_1ss10TA slicing gi393753182 at nt 393
alignment score=3.5 , category=2 , p=0.999999999976774
Cleavage Site 393
All Other Sites
Reads
0
1
2
3
4
5
6
0
200
400
600
gi393753182 position (nts)

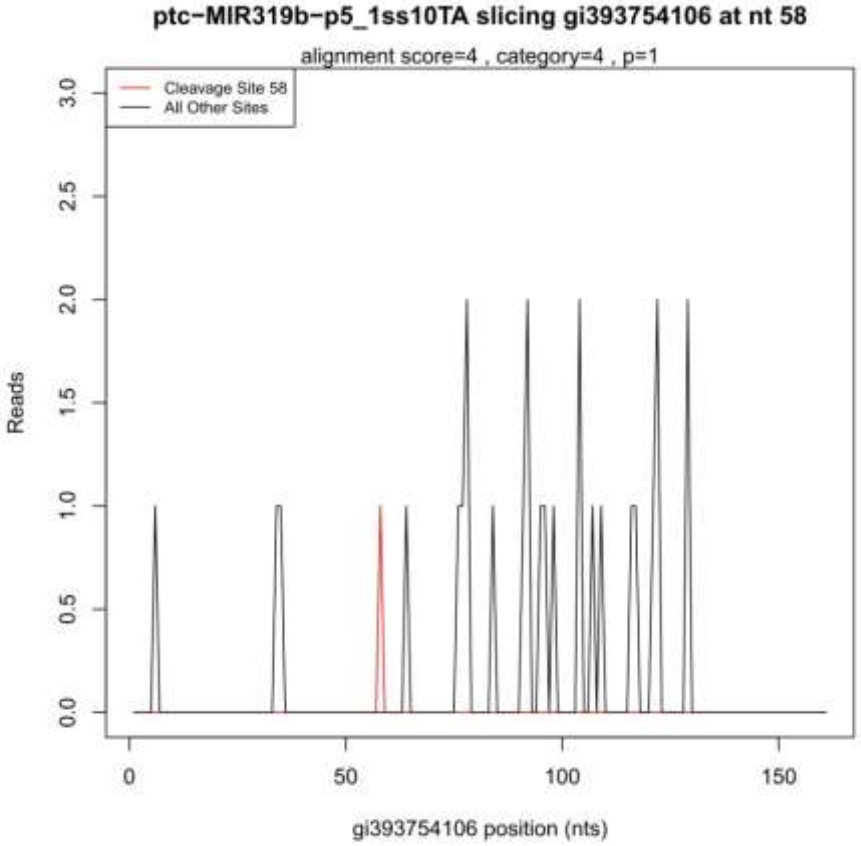

ptc−MIR319b−p5_1ss10TA slicing gi393754106 at nt 58
alignment score=4 , category=4 , p=1
Cleavage Site 58
All Other Sites
Reads
0.0
0.5
1.0
1.5
2.0
2.5
3.0
0
50
100
150
gi393754106 position (nts)

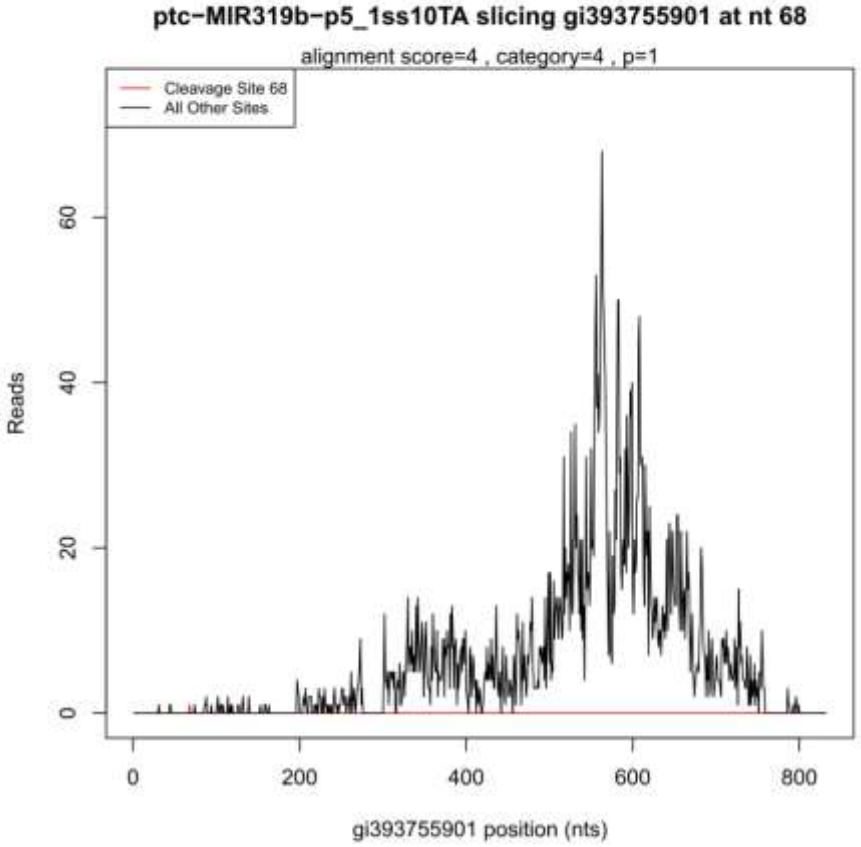

ptc−MIR319b−p5_1ss10TA slicing gi393755901 at nt 68
alignment score=4 , category=4 , p=1
Cleavage Site 68
All Other Sites
Reads
0
20
40
60
0
200
400
600
800
gi393755901 position (nts)

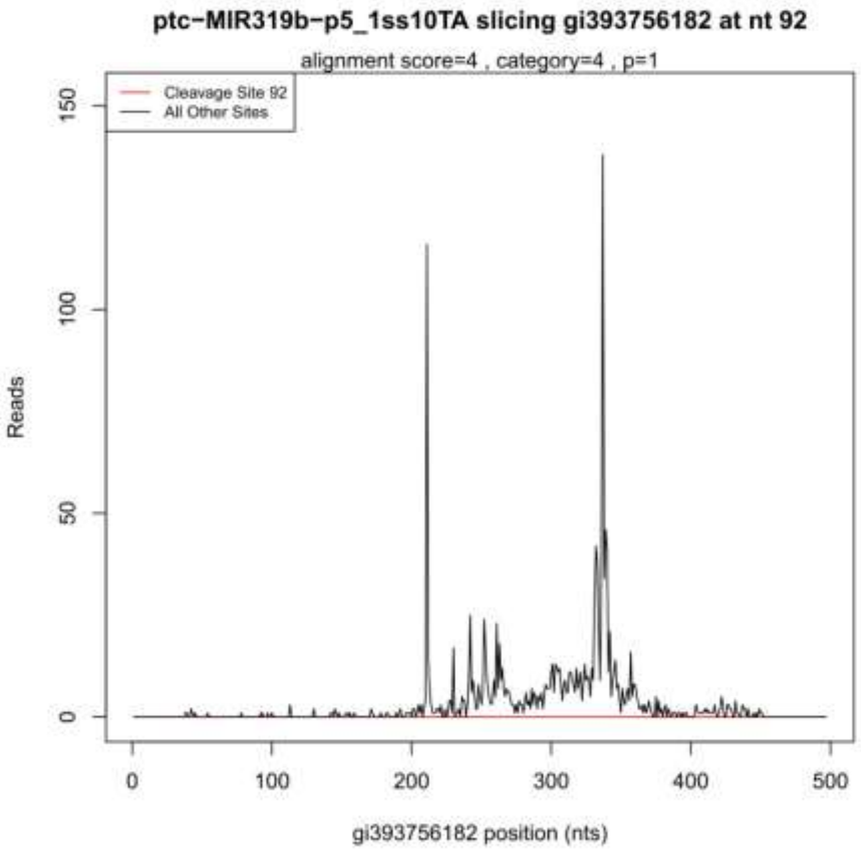
ptc−MIR319b−p5_1ss10TA slicing gi393756182 at nt 92
alignment score=4 , category=4 , p=1
Cleavage Site 92
All Other Sites
Reads
0
50
100
150
0
100
200
300
400
500
gi393756182 position (nts)

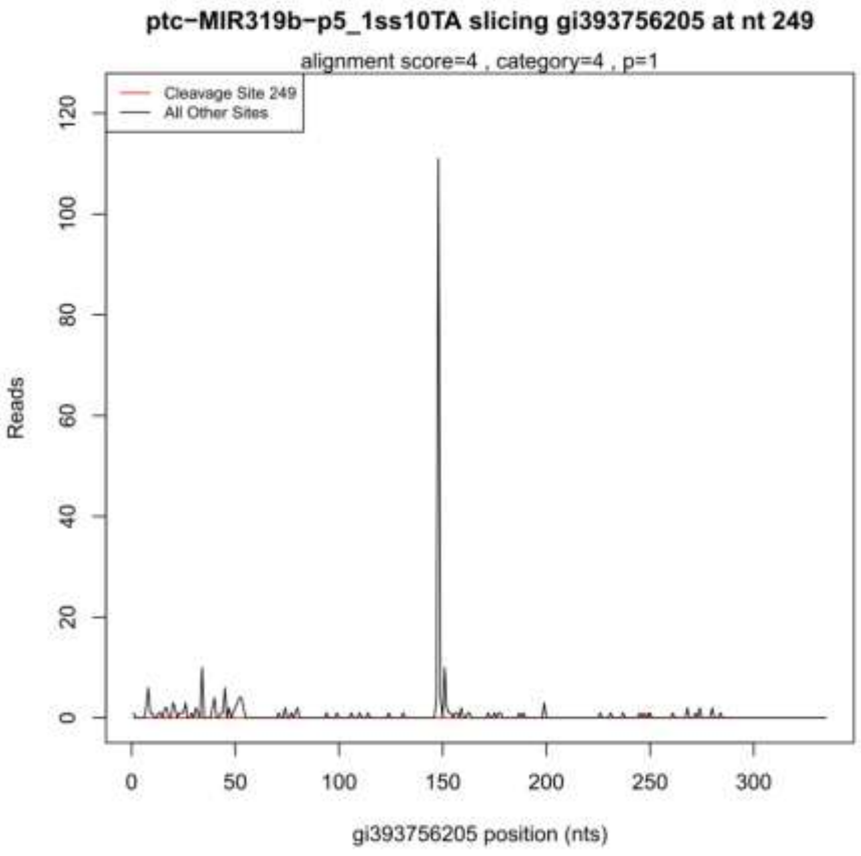
ptc−MIR319b−p5_1ss10TA slicing gi393756205 at nt 249
alignment score=4 , category=4 , p=1
Cleavage Site 249
All Other Sites
Reads
0
20
40
60
80
100
120
0
50
100
150
200
250
300
gi393756205 position (nts)

ptc−MIR319b−p5_1ss10TA slicing gi393756254 at nt 444

alignment score=4 , category=2 , p=1

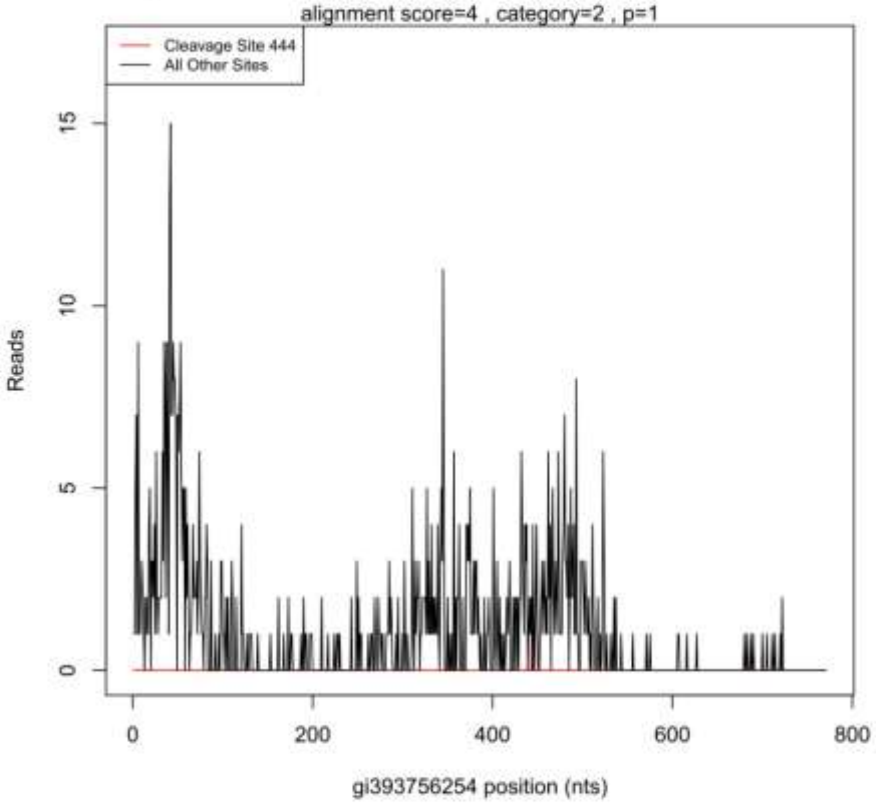

ptc−MIR319b−p5_1ss10TA slicing gi393757025 at nt 814

alignment score=3.5 , category=2 , p=0.999999997215306

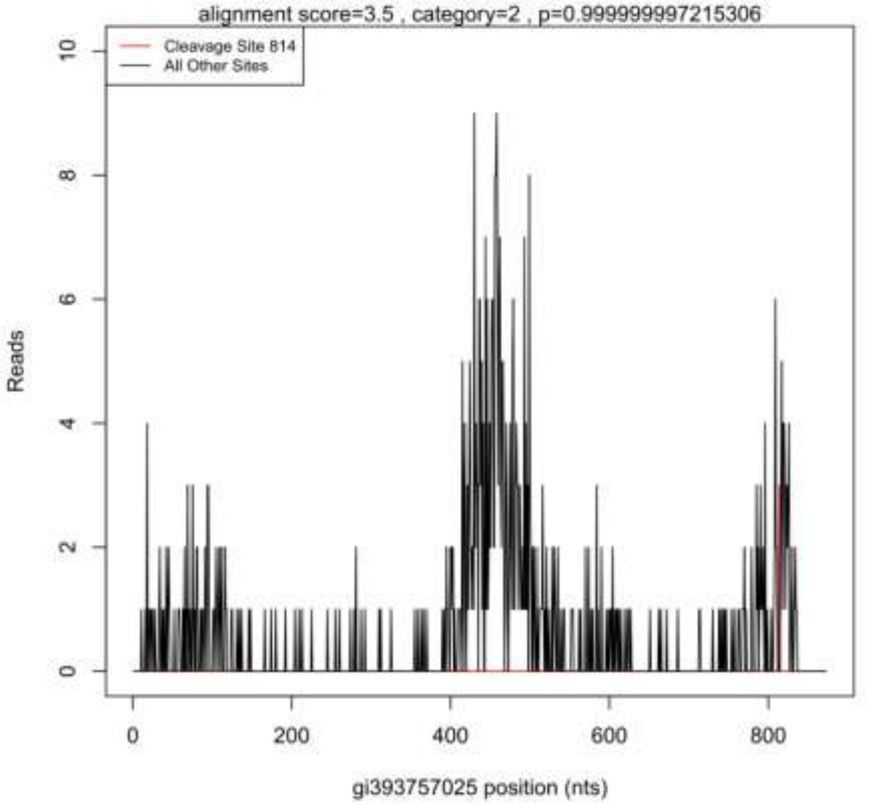

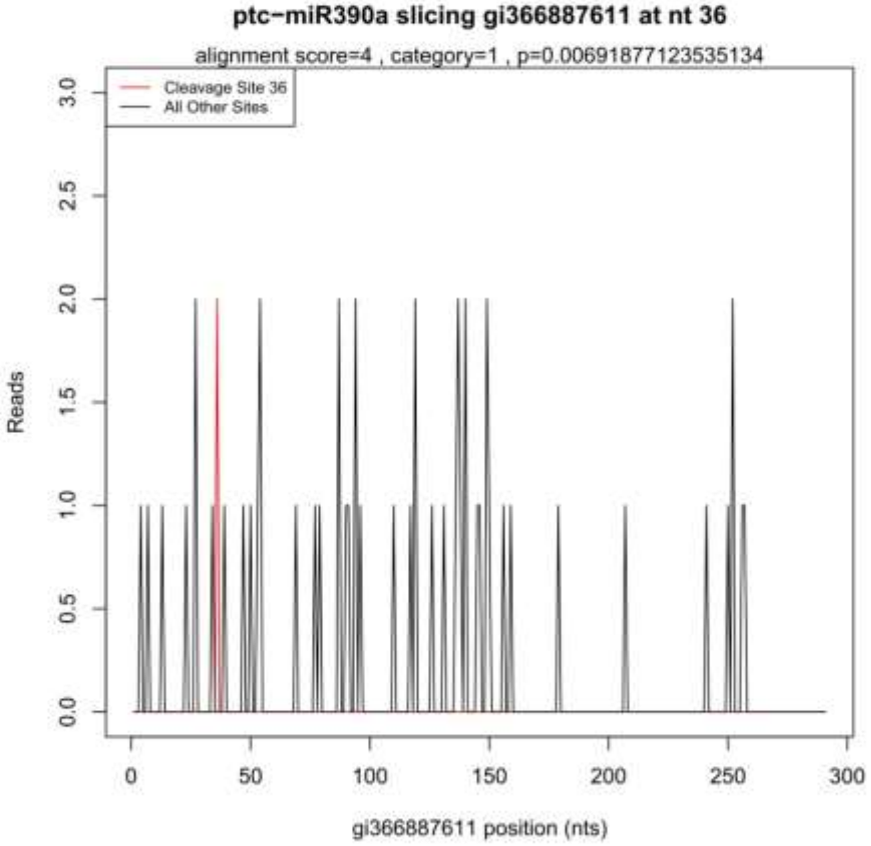

ptc−miR390a slicing gi366887611 at nt 36
alignment score=4 , category=1 , p=0.00691877123535134
Cleavage Site 36
All Other Sites
Reads
0.0
0.5
1.0
1.5
2.0
2.5
3.0
0
50
100
150
200
250
300
gi366887611 position (nts)

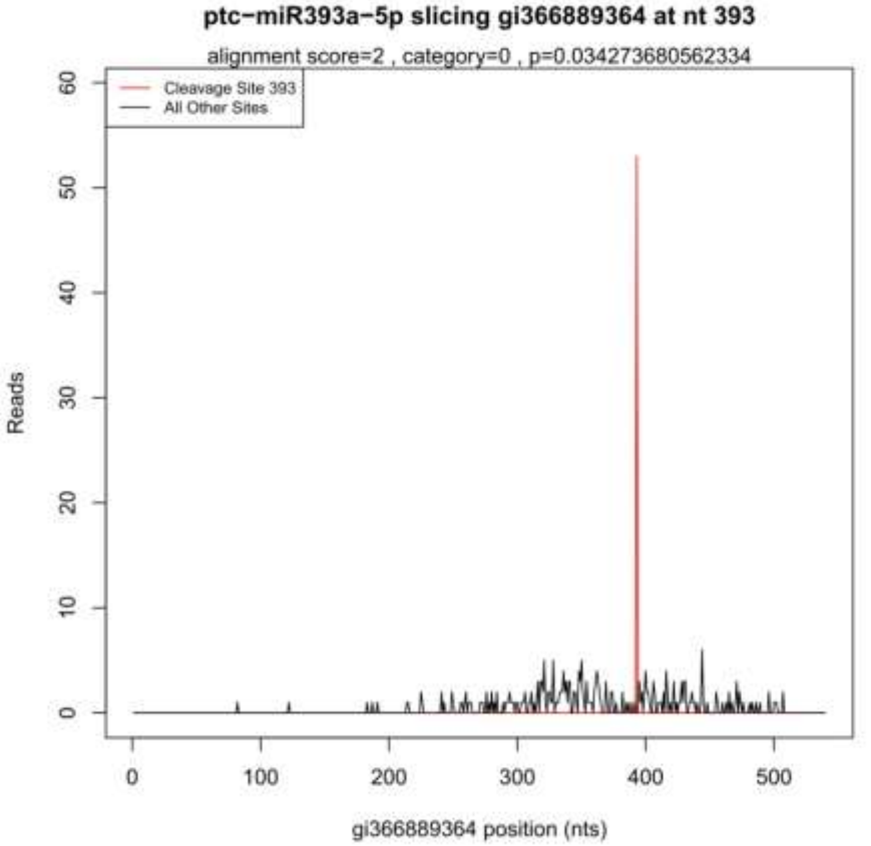

ptc−miR393a−5p slicing gi366889364 at nt 393
alignment score=2 , category=0 , p=0.034273680562334
Cleavage Site 393
All Other Sites
Reads
0
10
20
30
40
50
60
0
100
200
300
400
500
gi366889364 position (nts)

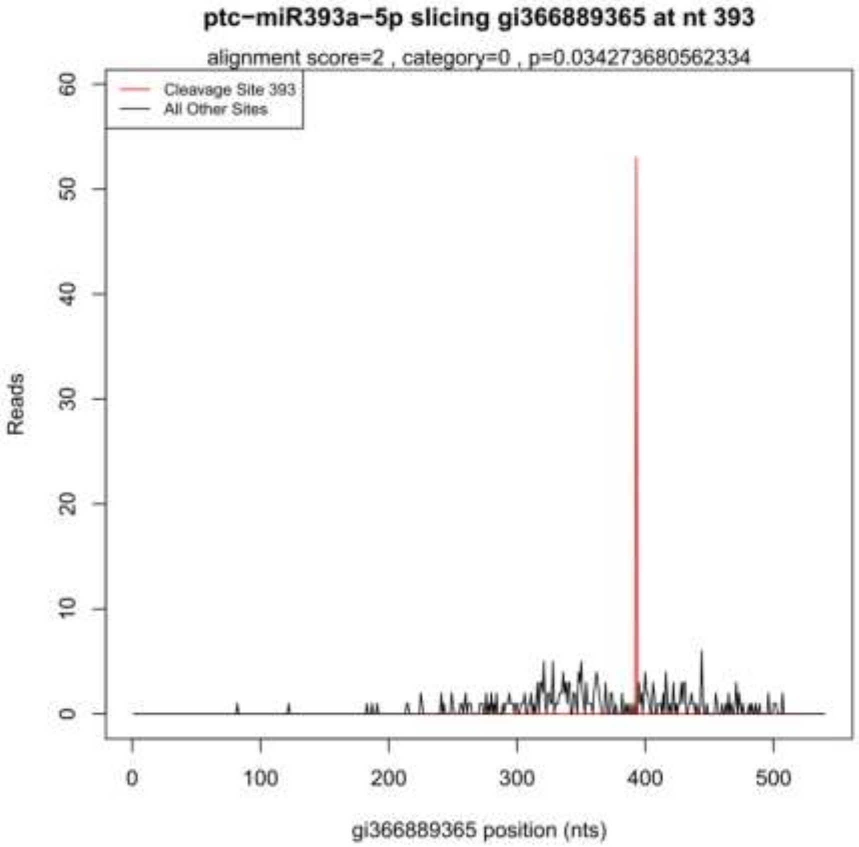
ptc−miR393a−5p slicing gi366889365 at nt 393
alignment score=2 , category=0 , p=0.034273680562334
Cleavage Site 393
All Other Sites
Reads
0
10
20
30
40
50
60
0
100
200
300
400
500
gi366889365 position (nts)

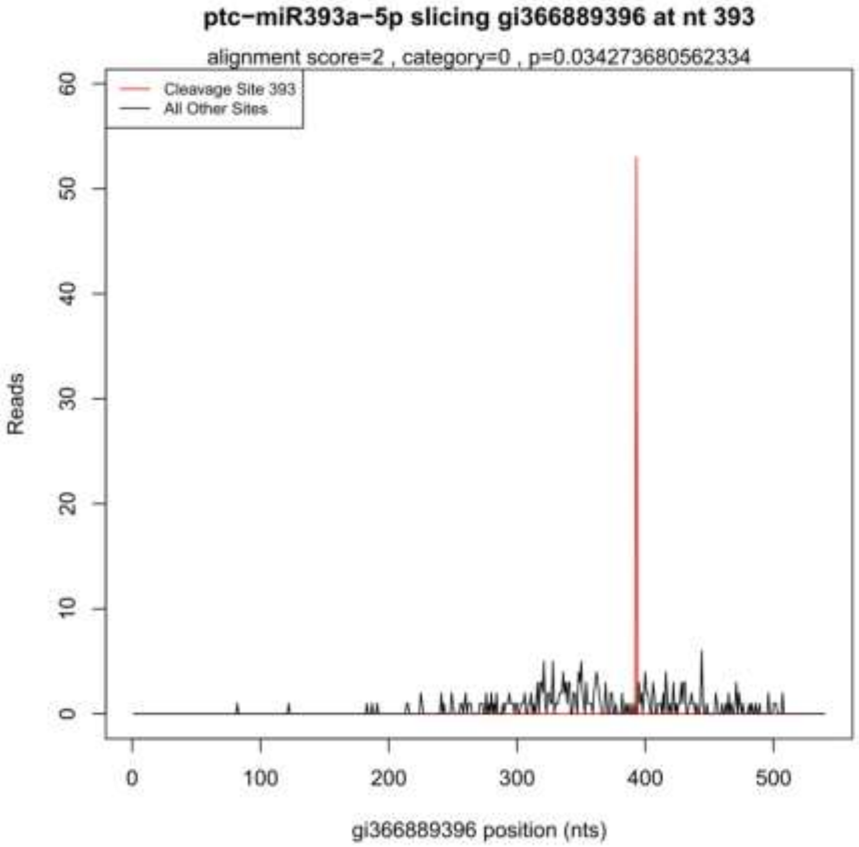
ptc−miR393a−5p slicing gi366889396 at nt 393
alignment score=2 , category=0 , p=0.034273680562334
Cleavage Site 393
All Other Sites
Reads
0
10
20
30
40
50
60
0
100
200
300
400
500
gi366889396 position (nts)

ptc−miR395a_1ss13GA slicing gi212378452 at nt 665

alignment score=3.5 , category=2 , p=0.617464209894666

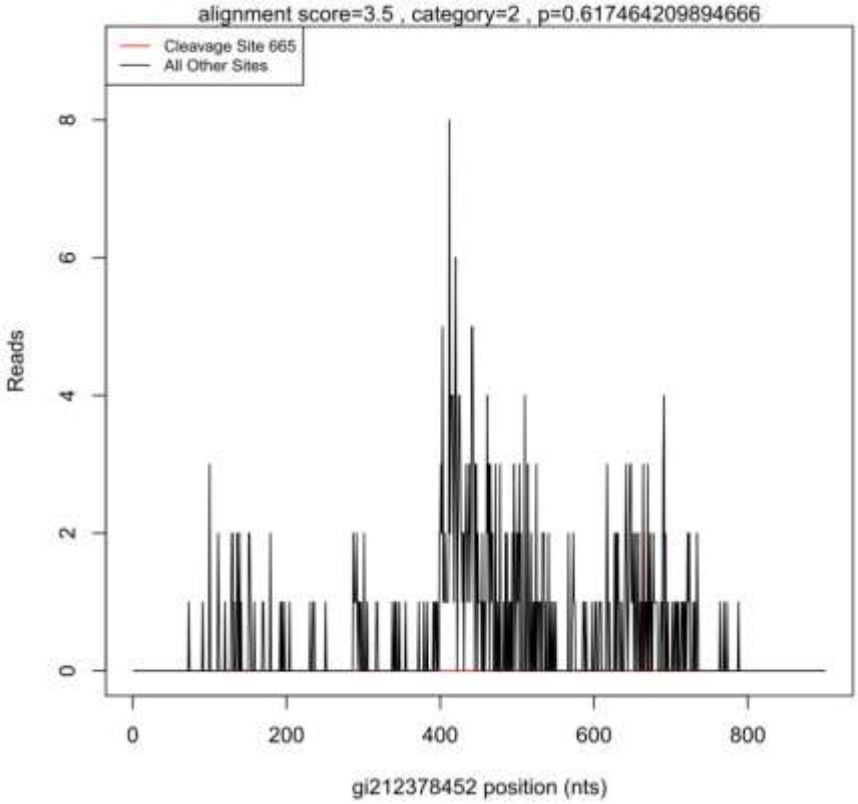

ptc−MIR395a−p5_1ss3GT slicing gi343702799 at nt 105

alignment score=3 , category=2 , p=0.999999993239842

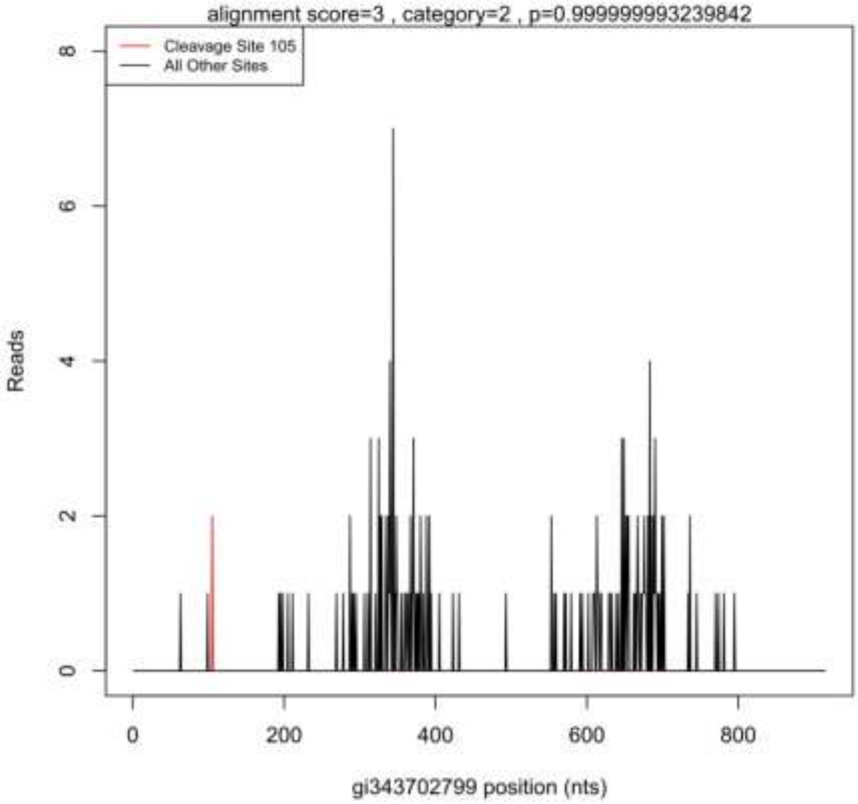

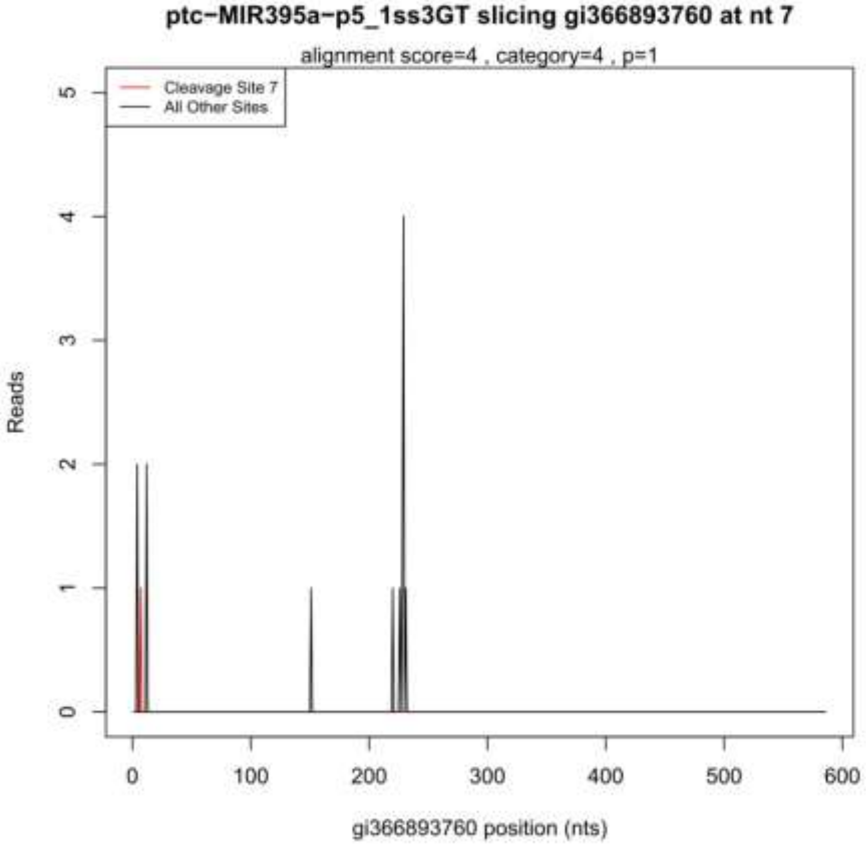
ptc−MIR395a−p5_1ss3GT slicing gi366893760 at nt 7
alignment score=4 , category=4 , p=1
Cleavage Site 7
All Other Sites
Reads
gi366893760 position (nts)

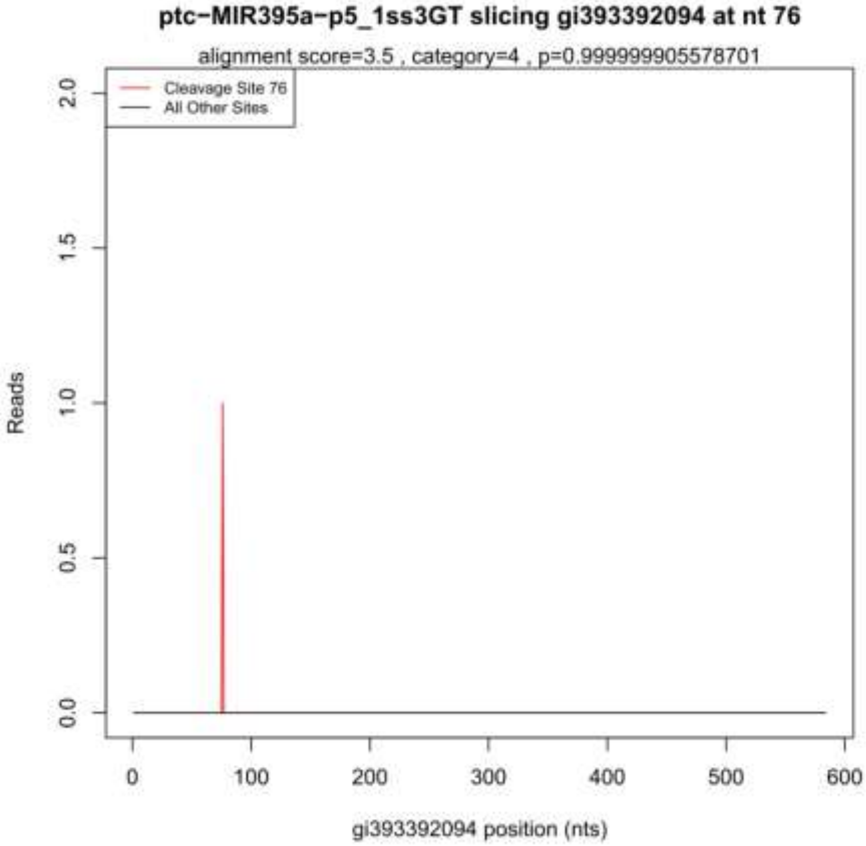
ptc−MIR395a−p5_1ss3GT slicing gi393392094 at nt 76
alignment score=3.5 , category=4 , p=0.999999905578701
Cleavage Site 76
All Other Sites
Reads
gi393392094 position (nts)

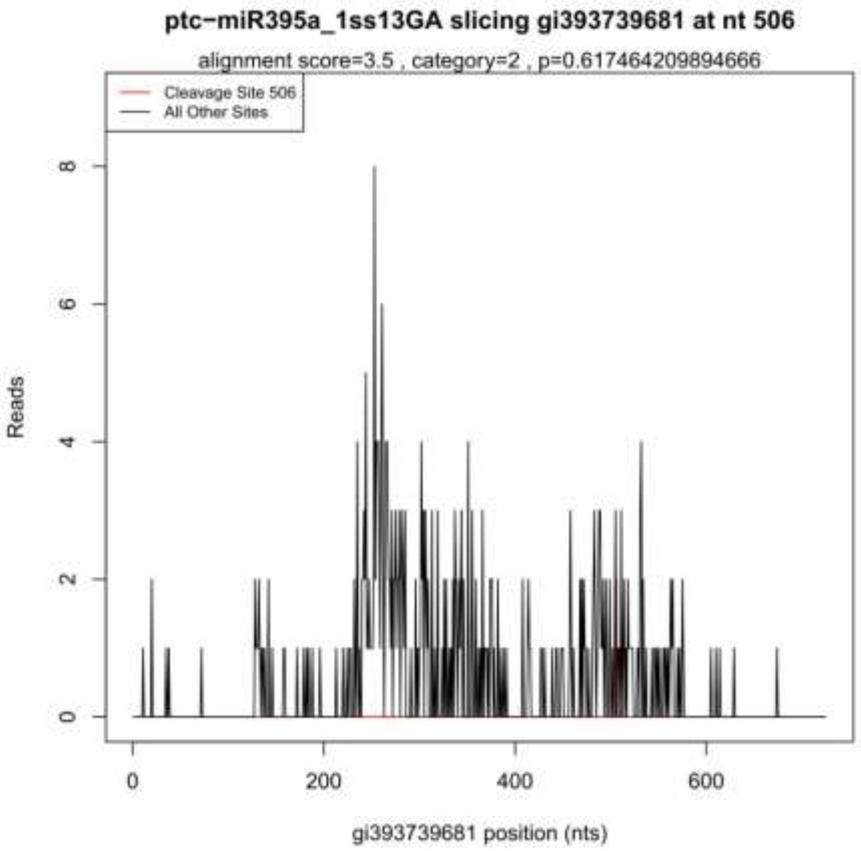
ptc−miR395a_1ss13GA slicing gi393739681 at nt 506
alignment score=3.5 , category=2 , p=0.617464209894666
Cleavage Site 506
All Other Sites
Reads
0
2
4
6
8
0
200
400
600
gi393739681 position (nts)

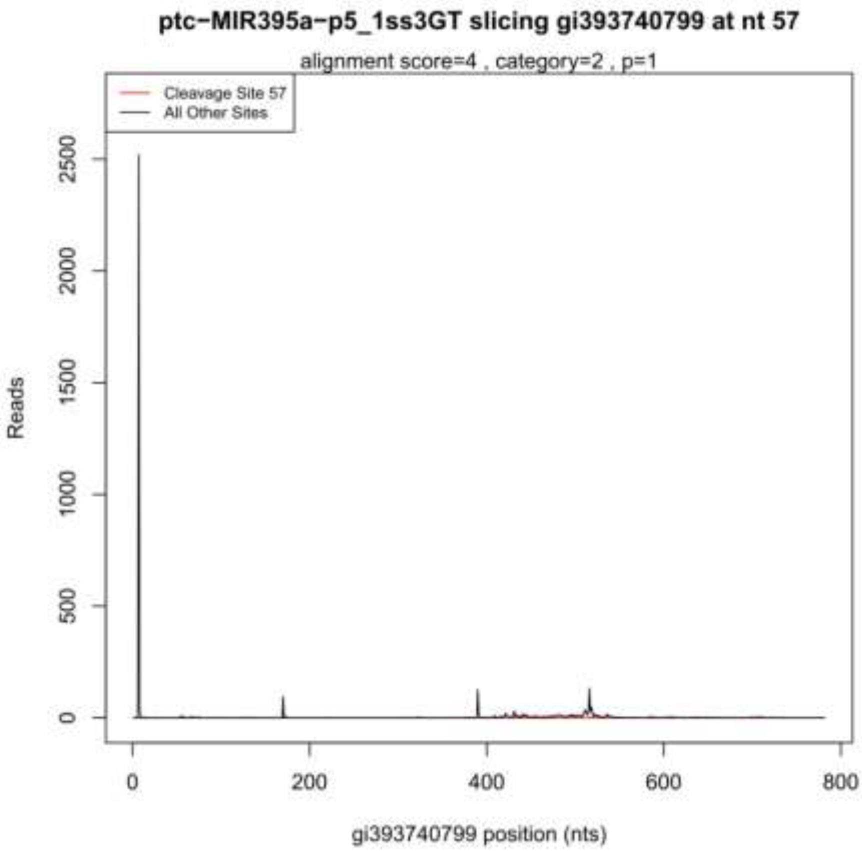
ptc−MIR395a−p5_1ss3GT slicing gi393740799 at nt 57
alignment score=4 , category=2 , p=1
Cleavage Site 57
All Other Sites
Reads
0
500
1000
1500
2000
2500
0
200
400
600
800
gi393740799 position (nts)

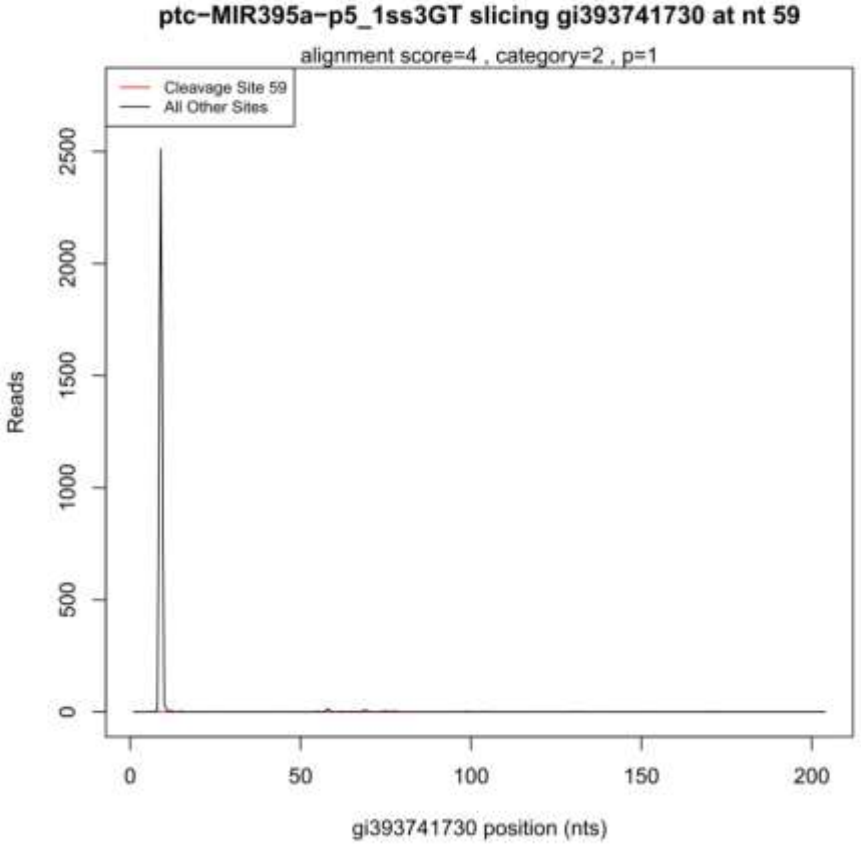
ptc−MIR395a−p5_1ss3GT slicing gi393741730 at nt 59
alignment score=4 , category=2 , p=1
Cleavage Site 59
All Other Sites
Reads
0
500
1000
1500
2000
2500
0
50
100
150
200
gi393741730 position (nts)

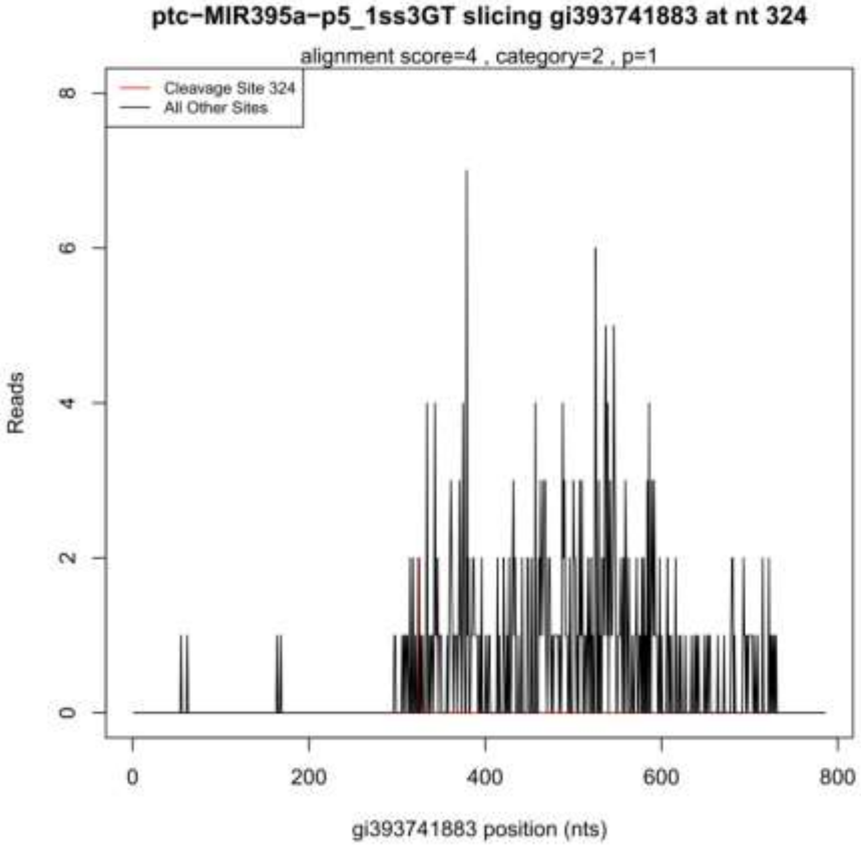
ptc−MIR395a−p5_1ss3GT slicing gi393741883 at nt 324
alignment score=4 , category=2 , p=1
Cleavage Site 324
All Other Sites
Reads
0
2
4
6
8
0
200
400
600
800
gi393741883 position (nts)

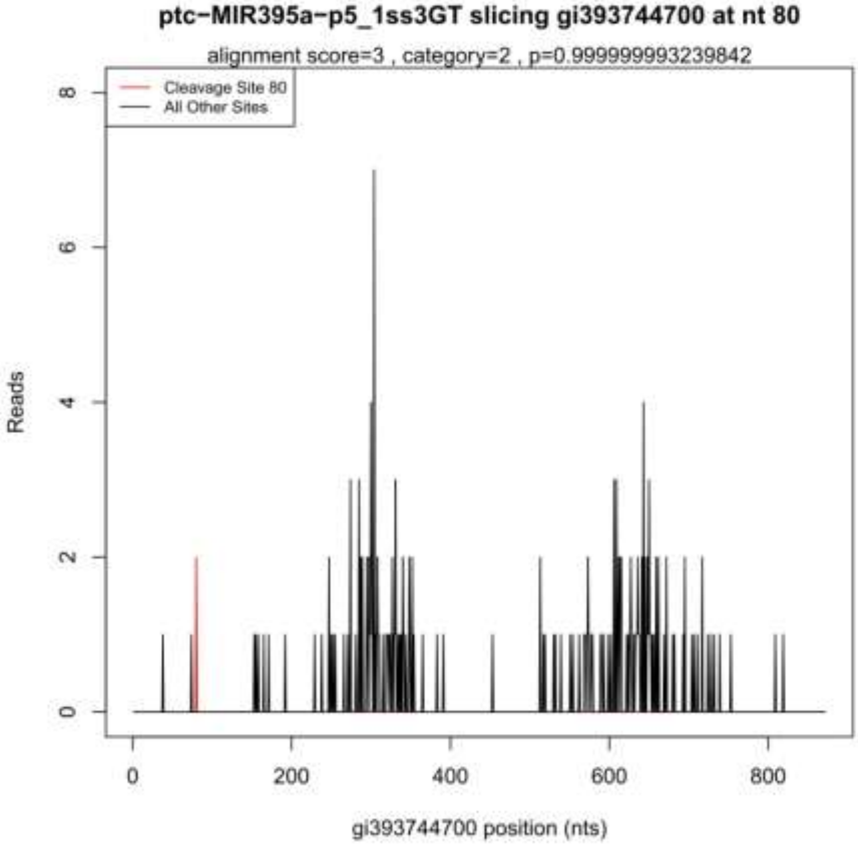
ptc-MIR395a-p5_1ss3GT slicing gi393744700 at nt 80
alignment score=3 , category=2 , p=0.999999993239842
Cleavage Site 80
All Other Sites
Reads
0
2
4
6
8
0
200
400
600
800
gi393744700 position (nts)

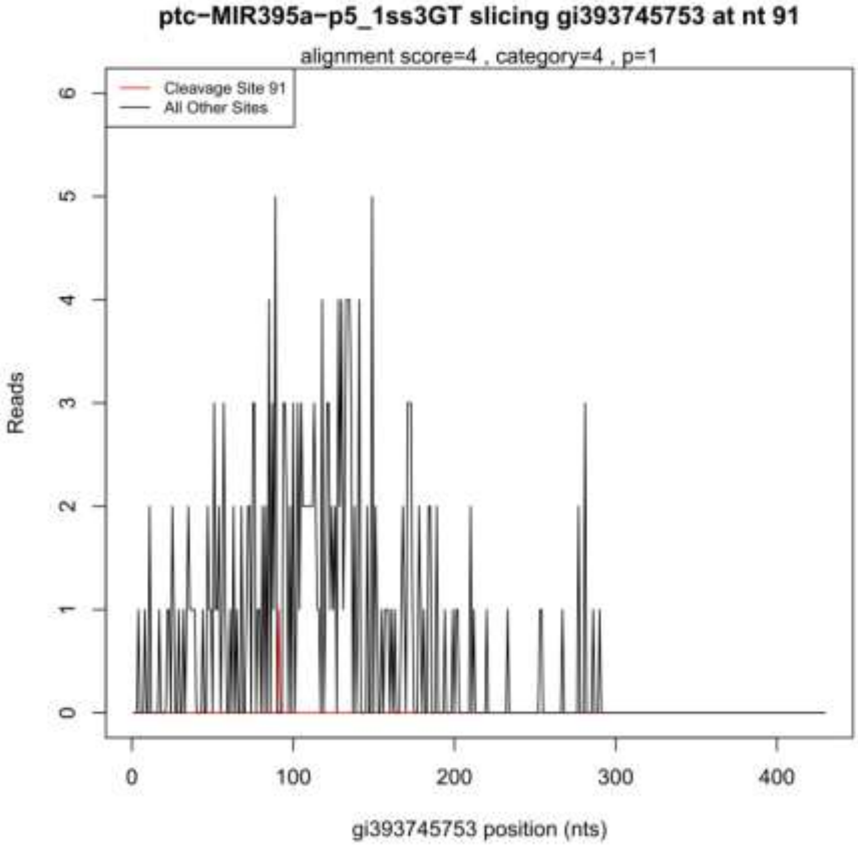
ptc-MIR395a-p5_1ss3GT slicing gi393745753 at nt 91
alignment score=4 , category=4 , p=1
Cleavage Site 91
All Other Sites
Reads
0
1
2
3
4
5
6
0
100
200
300
400
gi393745753 position (nts)

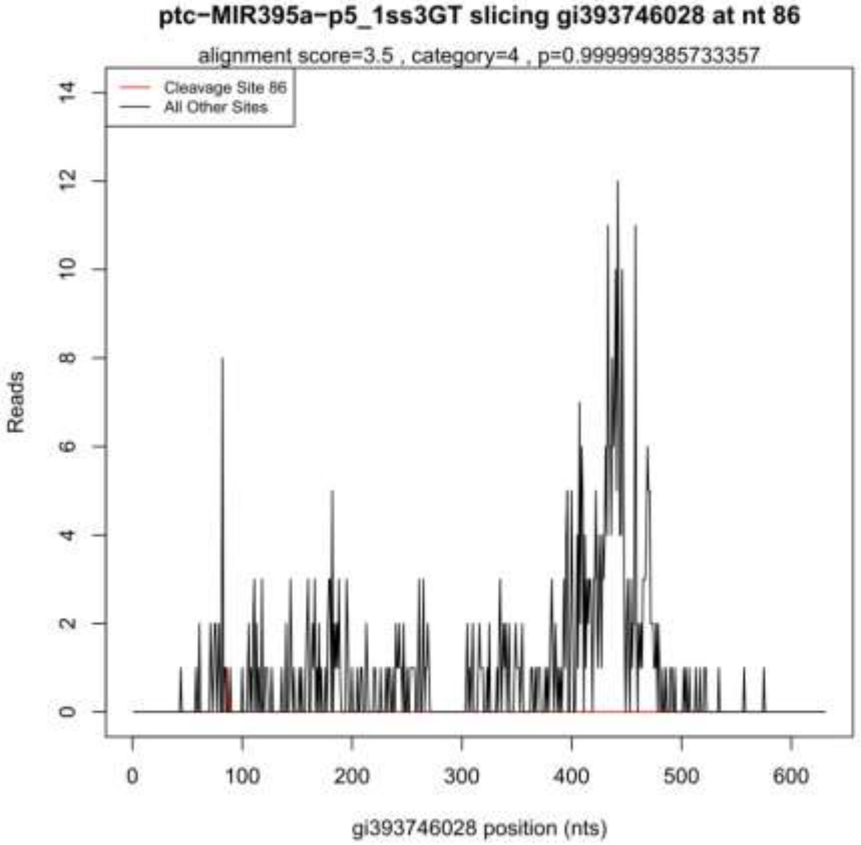
ptc-MIR395a-p5_1ss3GT slicing gi393746028 at nt 86
alignment score=3.5 , category=4 , p=0.999999385733357
Cleavage Site 86
All Other Sites
Reads
0
2
4
6
8
10
12
14
0
100
200
300
400
500
600
gi393746028 position (nts)

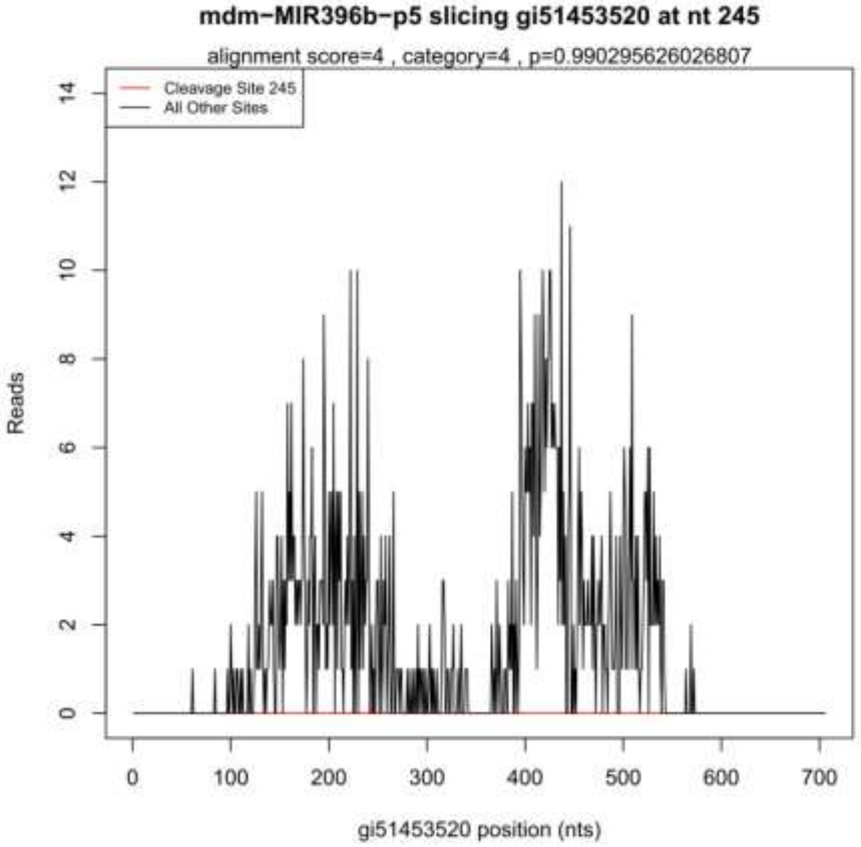
mdm-MIR396b-p5 slicing gi51453520 at nt 245
alignment score=4 , category=4 , p=0.990295626026807
Cleavage Site 245
All Other Sites
Reads
0
2
4
6
8
10
12
14
0
100
200
300
400
500
600
700
gi51453520 position (nts)

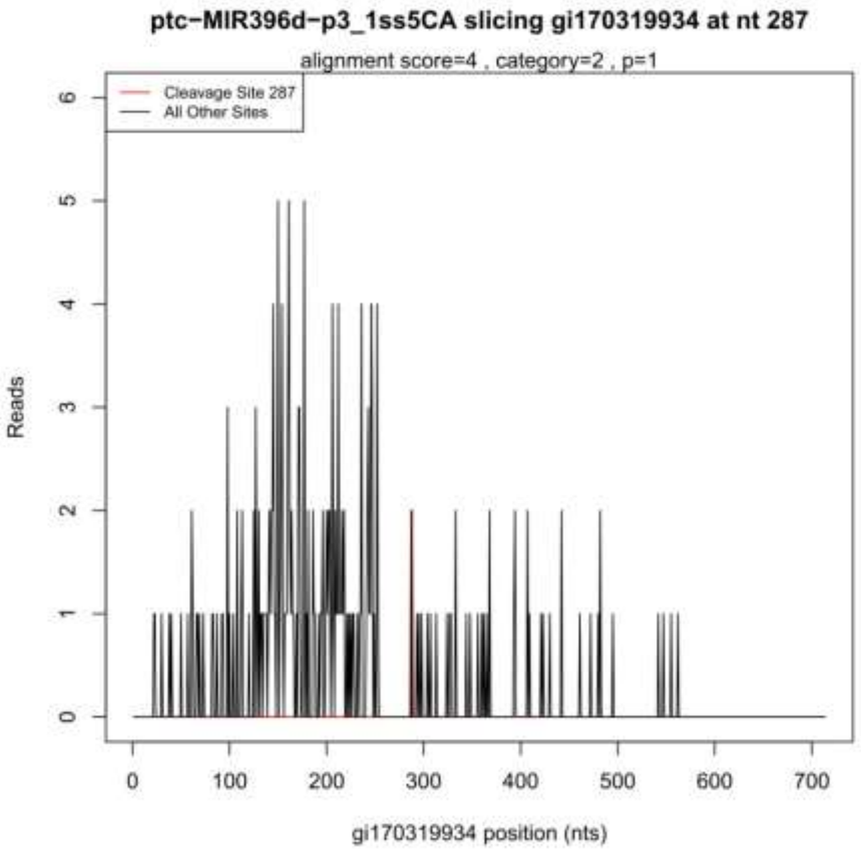
ptc-MIR396d-p3_1ss5CA slicing gi170319934 at nt 287
alignment score=4 , category=2 , p=1
Cleavage Site 287
All Other Sites
Reads
gi170319934 position (nts)

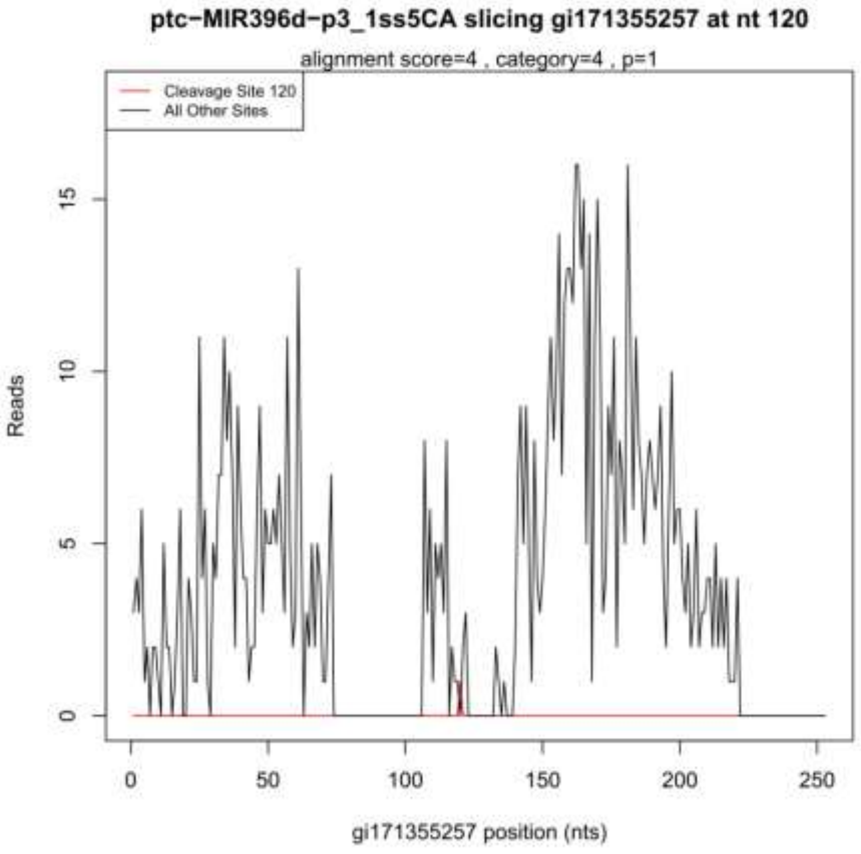
ptc-MIR396d-p3_1ss5CA slicing gi171355257 at nt 120
alignment score=4 , category=4 , p=1
Cleavage Site 120
All Other Sites
Reads
gi171355257 position (nts)

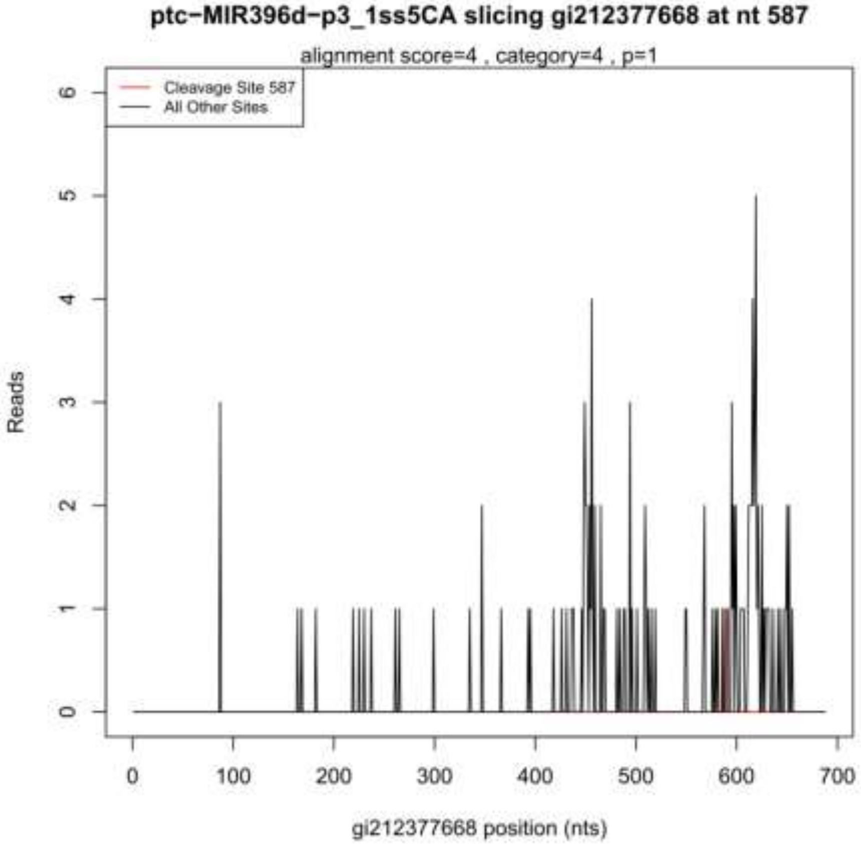

ptc−MIR396d−p3_1ss5CA slicing gi212377668 at nt 587
alignment score=4 , category=4 , p=1
Cleavage Site 587
All Other Sites
Reads
0
1
2
3
4
5
6
0
100
200
300
400
500
600
700
gi212377668 position (nts)

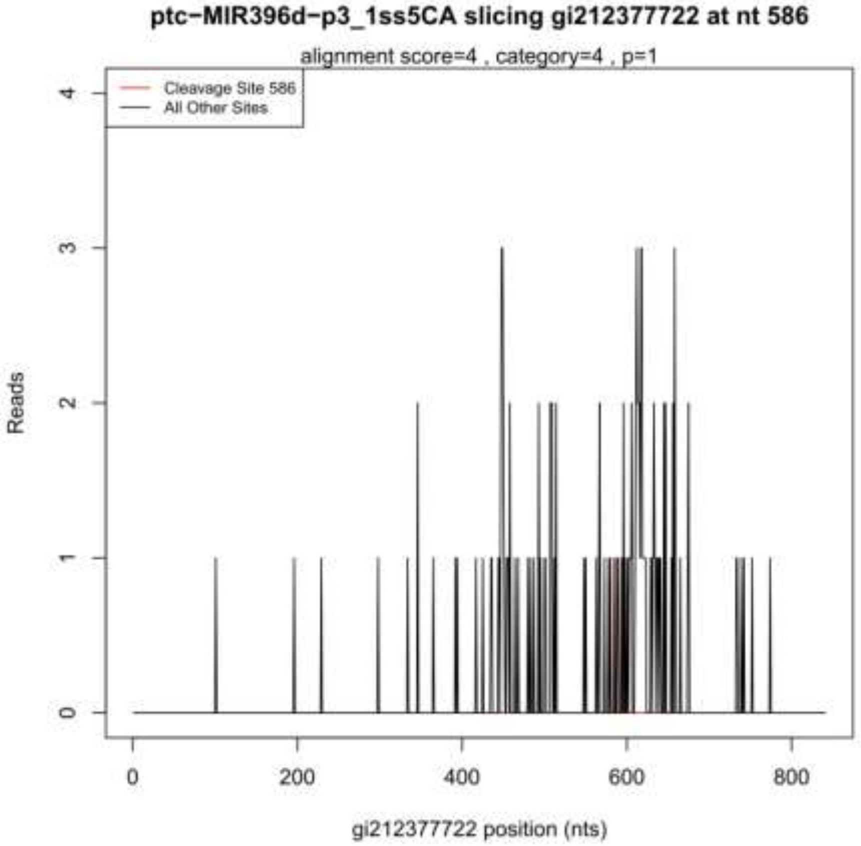

ptc−MIR396d−p3_1ss5CA slicing gi212377722 at nt 586
alignment score=4 , category=4 , p=1
Cleavage Site 586
All Other Sites
Reads
0
1
2
3
4
0
200
400
600
800
gi212377722 position (nts)

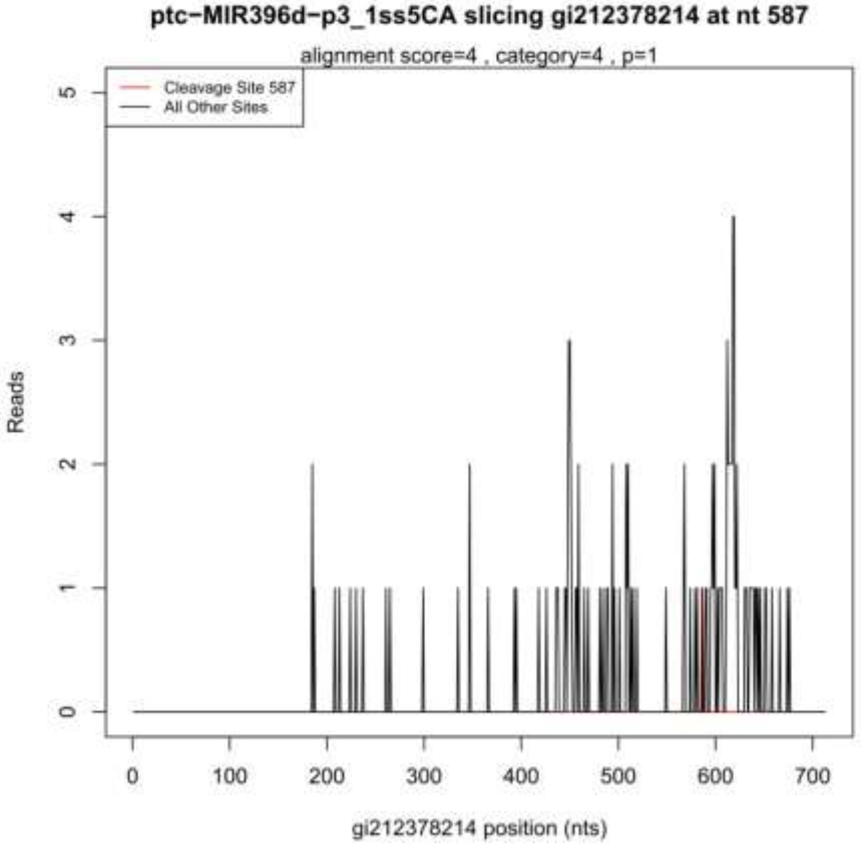
ptc-MIR396d-p3_1ss5CA slicing gi212378214 at nt 587
alignment score=4 , category=4 , p=1
Cleavage Site 587
All Other Sites
Reads
0
1
2
3
4
5
0
100
200
300
400
500
600
700
gi212378214 position (nts)

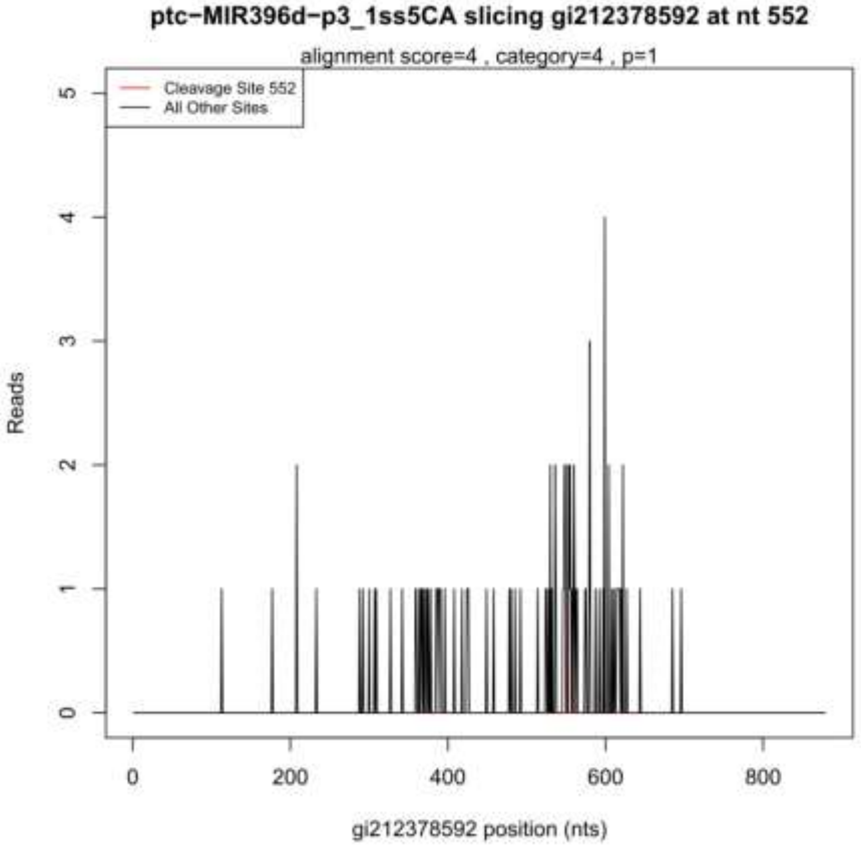
ptc-MIR396d-p3_1ss5CA slicing gi212378592 at nt 552
alignment score=4 , category=4 , p=1
Cleavage Site 552
All Other Sites
Reads
0
1
2
3
4
5
0
200
400
600
800
gi212378592 position (nts)

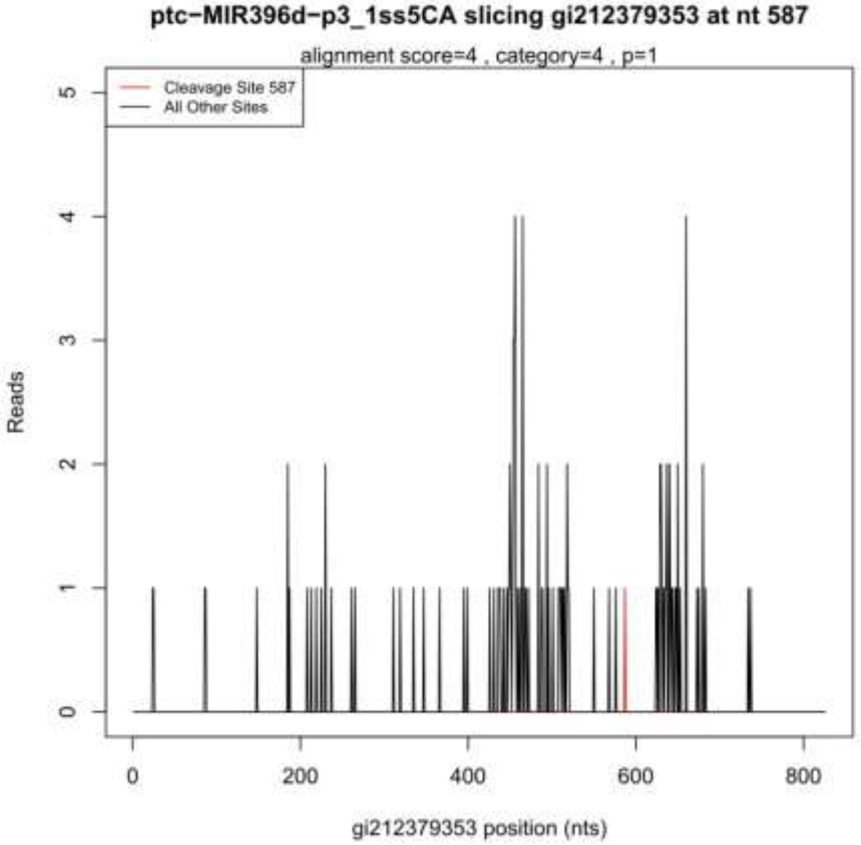
ptc-MIR396d-p3_1ss5CA slicing gi212379353 at nt 587
alignment score=4 , category=4 , p=1
Cleavage Site 587
All Other Sites
Reads
0
1
2
3
4
5
0
200
400
600
800
gi212379353 position (nts)

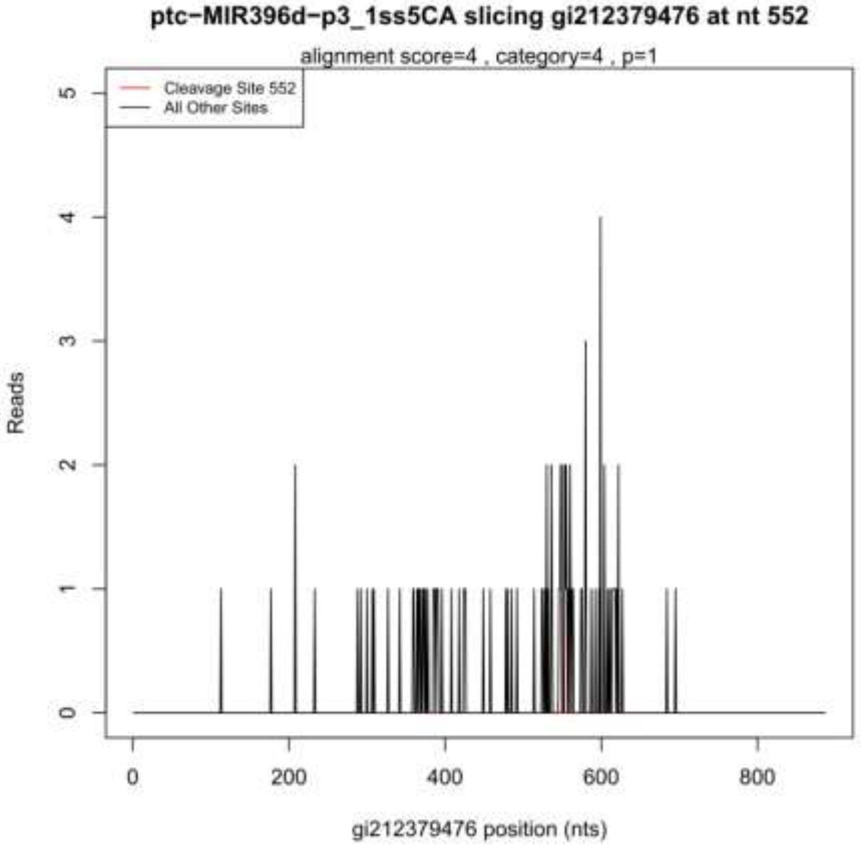
ptc-MIR396d-p3_1ss5CA slicing gi212379476 at nt 552
alignment score=4 , category=4 , p=1
Cleavage Site 552
All Other Sites
Reads
0
1
2
3
4
5
0
200
400
600
800
gi212379476 position (nts)

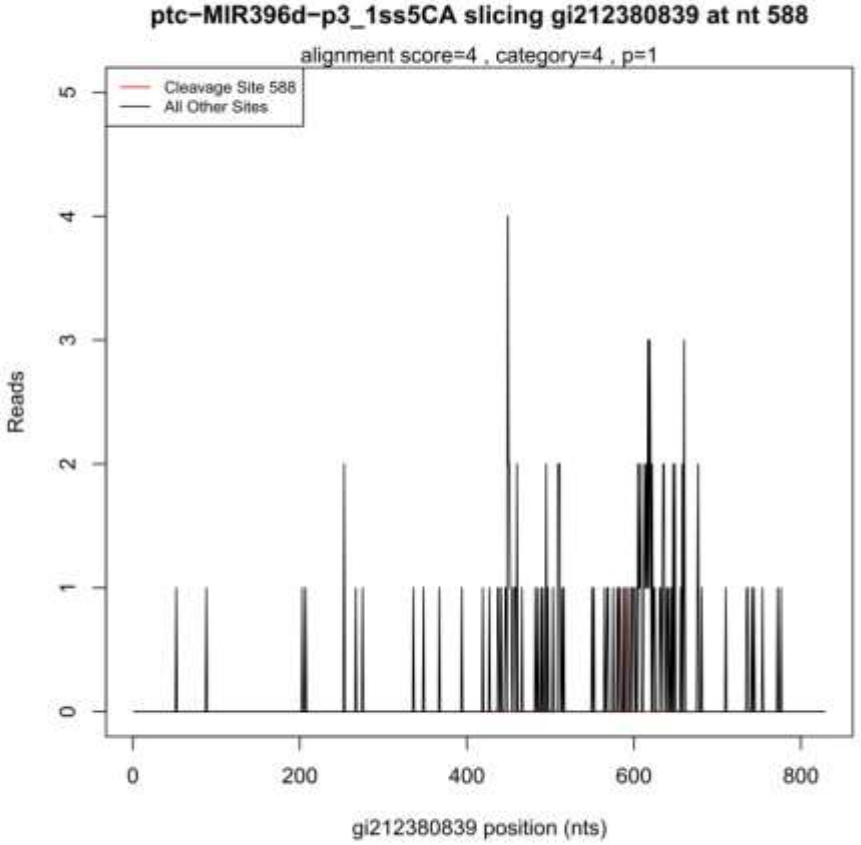
ptc−MIR396d−p3_1ss5CA slicing gi212380839 at nt 588
alignment score=4 , category=4 , p=1
Cleavage Site 588
All Other Sites
Reads
0
1
2
3
4
5
0
200
400
600
800
gi212380839 position (nts)

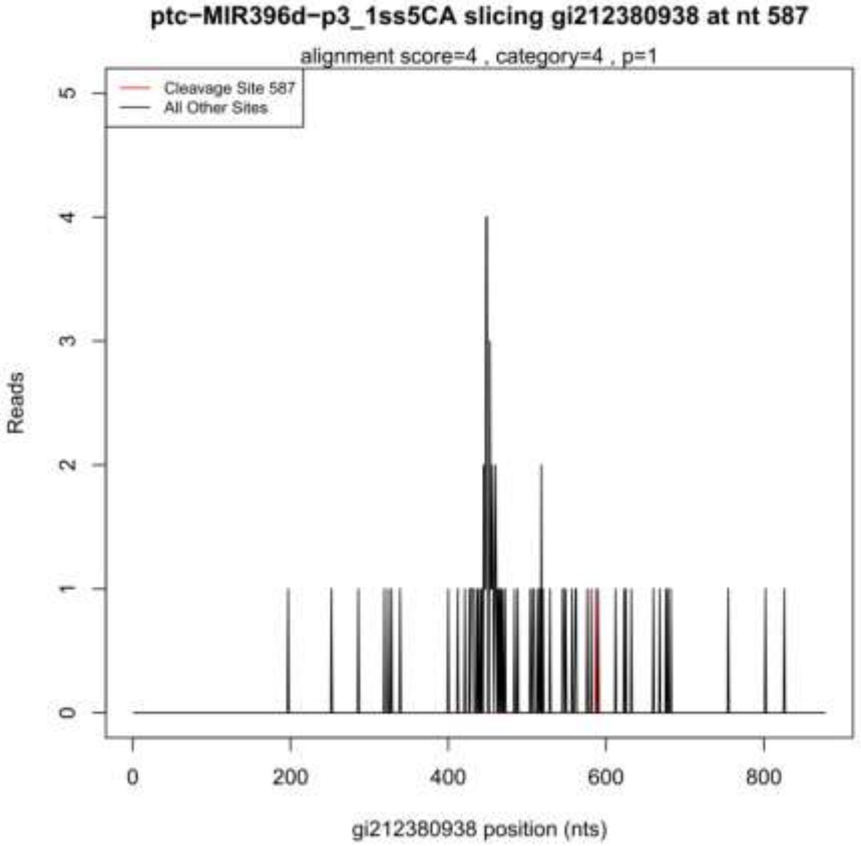
ptc−MIR396d−p3_1ss5CA slicing gi212380938 at nt 587
alignment score=4 , category=4 , p=1
Cleavage Site 587
All Other Sites
Reads
0
1
2
3
4
5
0
200
400
600
800
gi212380938 position (nts)

vvi−miR396b_L−1R+3 slicing gi212381014 at nt 495

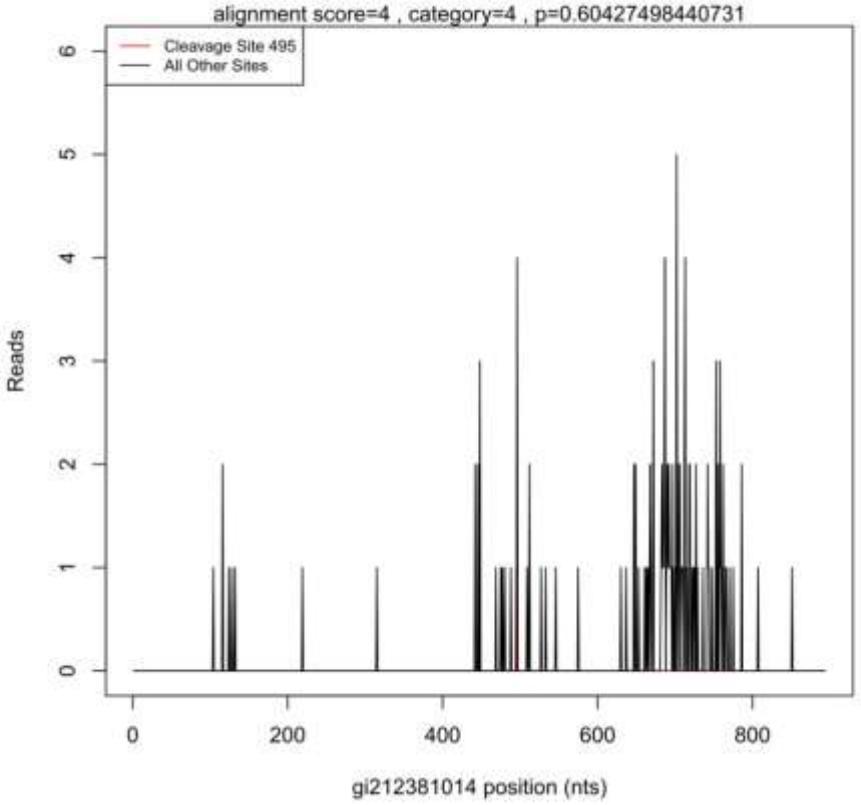

nta−miR396a_R+1_1ss21GT slicing gi212381014 at nt 496

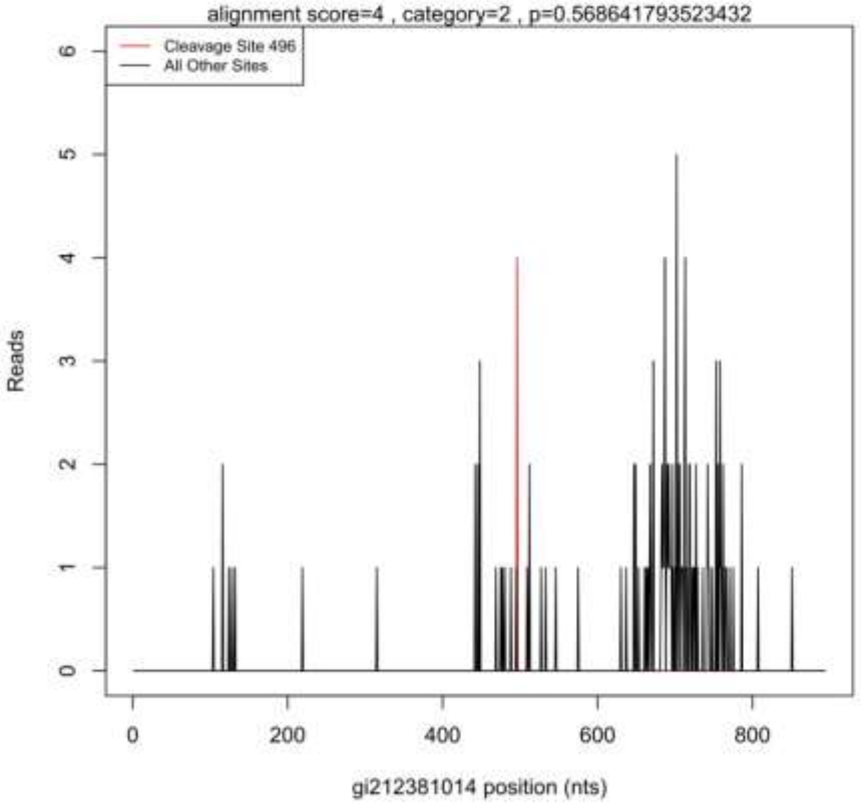

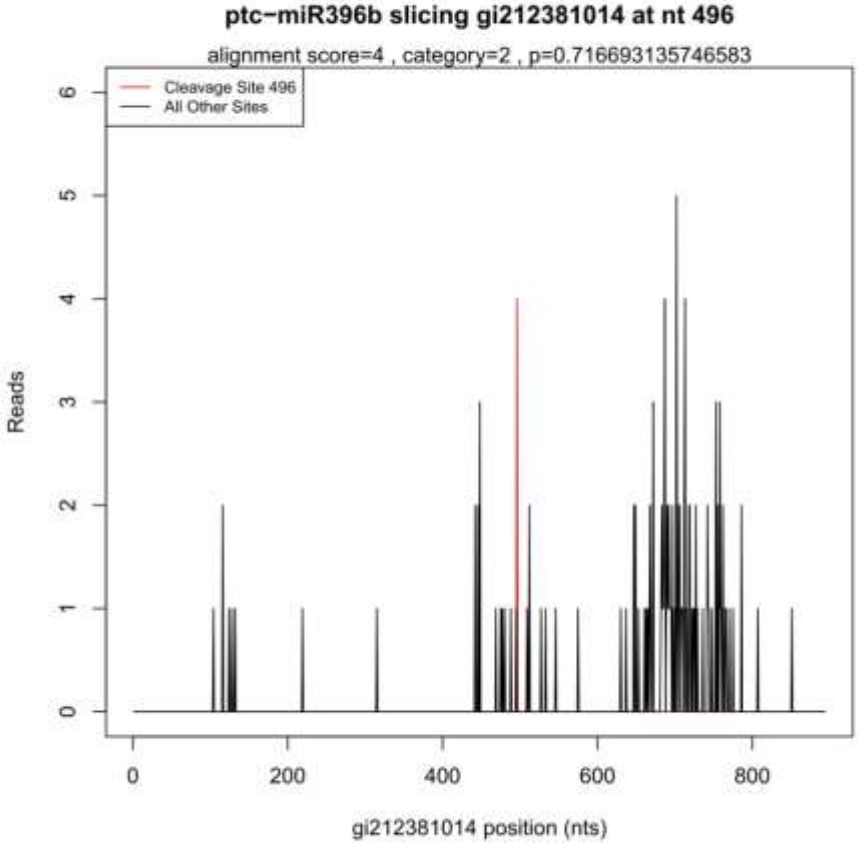

ptc−miR396b slicing gi212381014 at nt 496
alignment score=4 , category=2 , p=0.716693135746583
Cleavage Site 496
All Other Sites
Reads
0
1
2
3
4
5
6
0
200
400
600
800
gi212381014 position (nts)

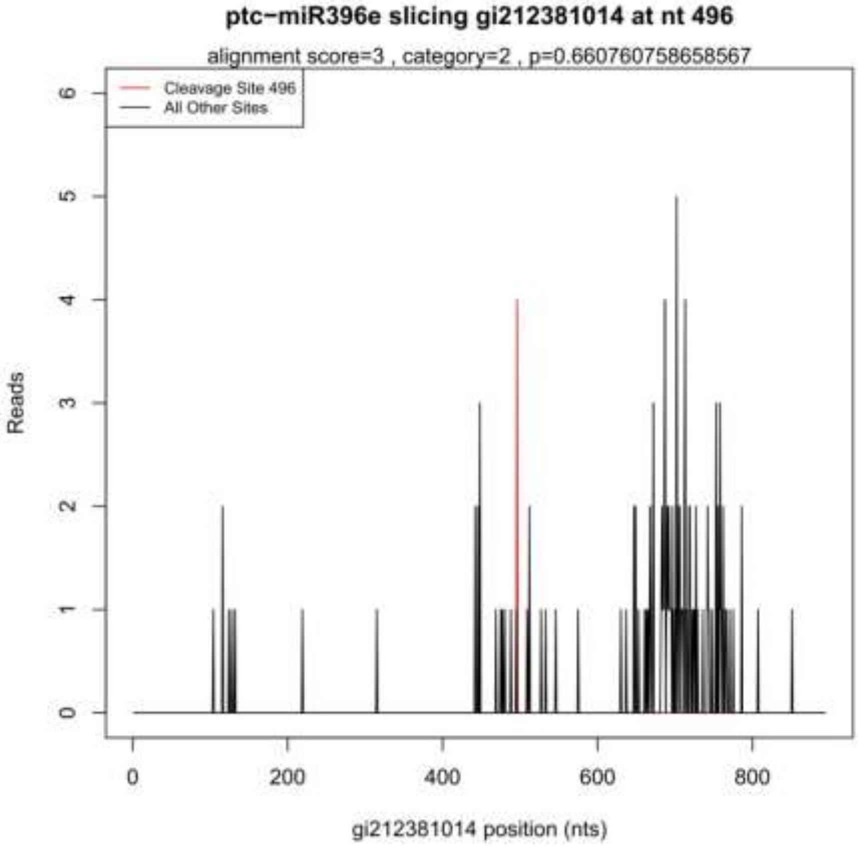

ptc−miR396e slicing gi212381014 at nt 496
alignment score=3 , category=2 , p=0.660760758658567
Cleavage Site 496
All Other Sites
Reads
0
1
2
3
4
5
6
0
200
400
600
800
gi212381014 position (nts)

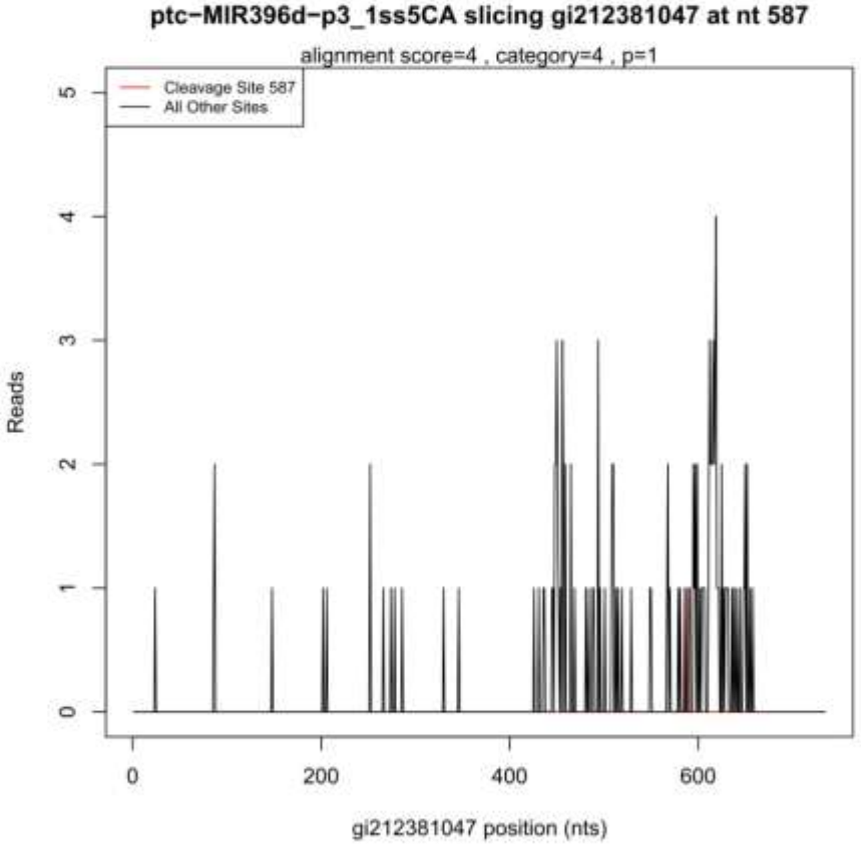
ptc−MIR396d−p3_1ss5CA slicing gi212381047 at nt 587
alignment score=4 , category=4 , p=1
Cleavage Site 587
All Other Sites
Reads
0
1
2
3
4
5
0
200
400
600
gi212381047 position (nts)

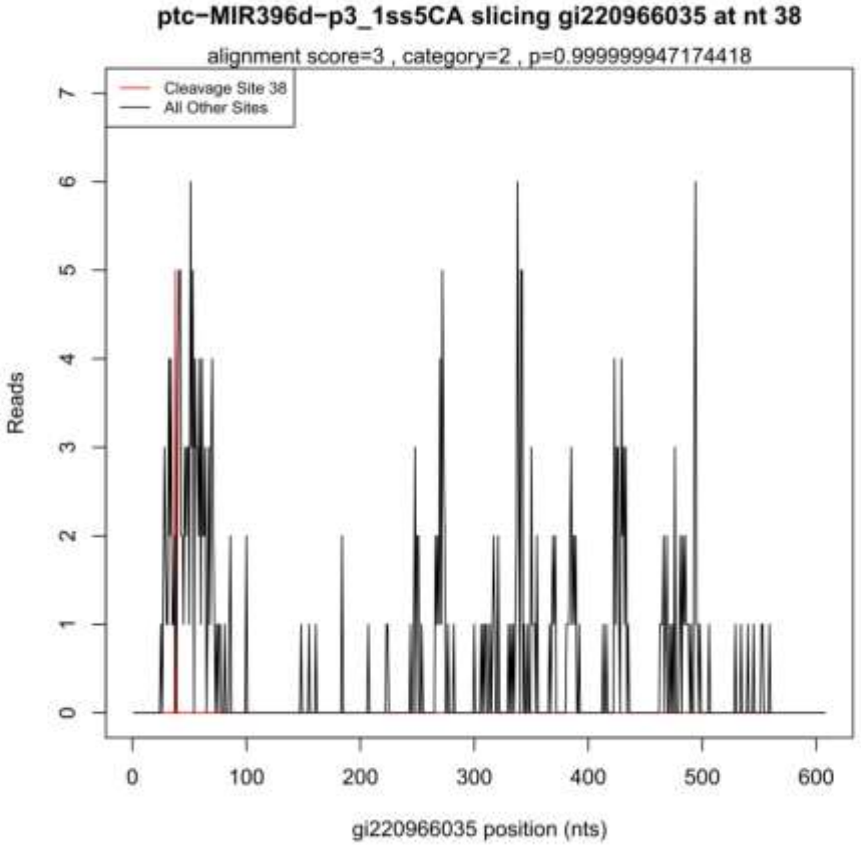
ptc−MIR396d−p3_1ss5CA slicing gi220966035 at nt 38
alignment score=3 , category=2 , p=0.999999947174418
Cleavage Site 38
All Other Sites
Reads
0
1
2
3
4
5
6
7
0
100
200
300
400
500
600
gi220966035 position (nts)

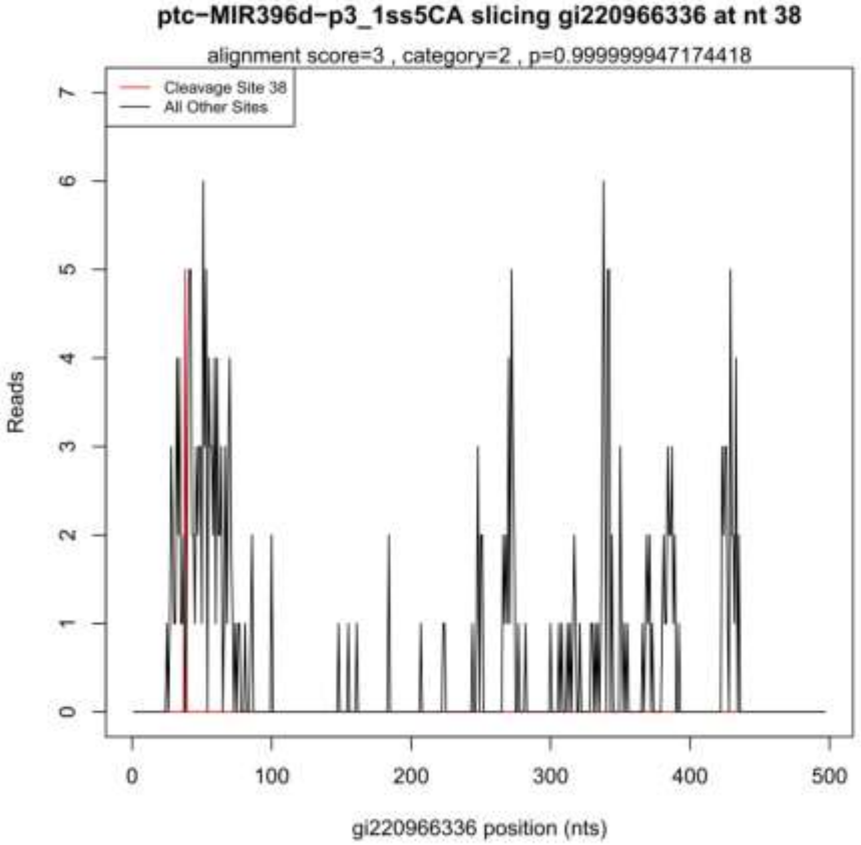
ptc−MIR396d−p3_1ss5CA slicing gi220966336 at nt 38
alignment score=3 , category=2 , p=0.999999947174418
Cleavage Site 38
All Other Sites
Reads
0
1
2
3
4
5
6
7
0
100
200
300
400
500
gi220966336 position (nts)

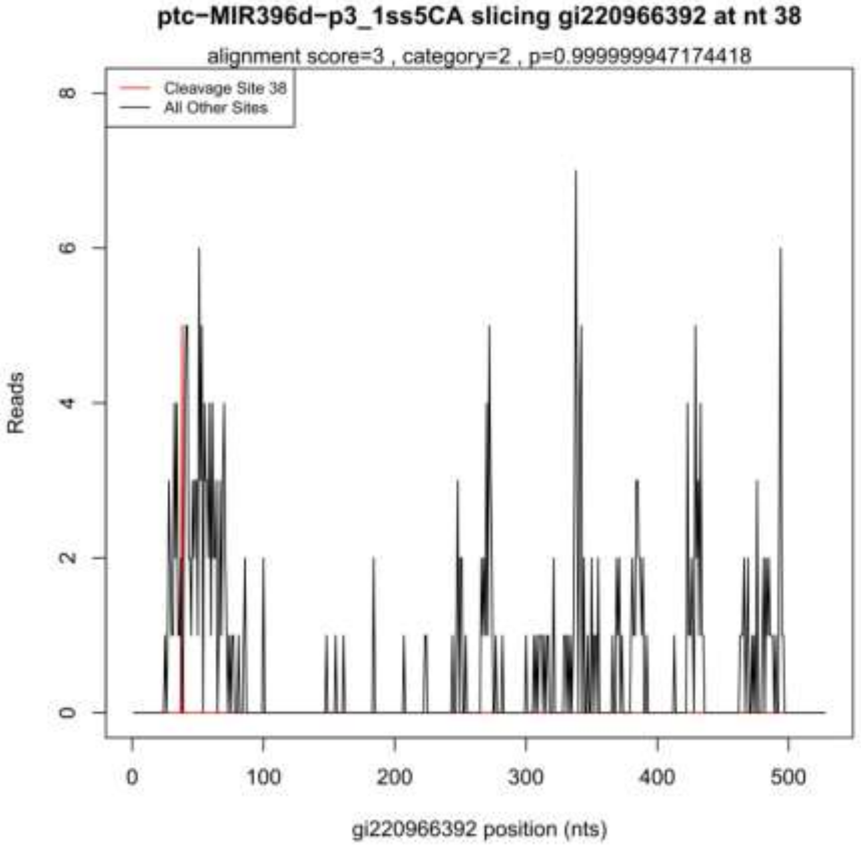
ptc−MIR396d−p3_1ss5CA slicing gi220966392 at nt 38
alignment score=3 , category=2 , p=0.999999947174418
Cleavage Site 38
All Other Sites
Reads
0
2
4
6
8
0
100
200
300
400
500
gi220966392 position (nts)

ptc−MIR396d−p3_1ss5CA slicing gi221071321 at nt 117

alignment score=4 , category=4 , p=1

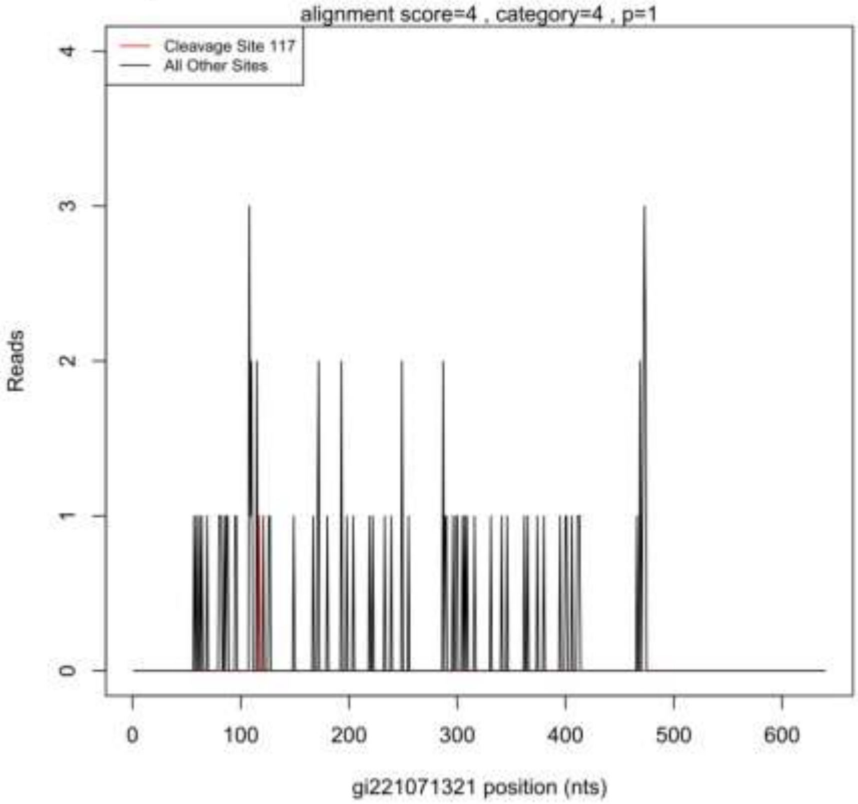

ptc−MIR396d−p3_1ss5CA slicing gi222457813 at nt 121

alignment score=3 , category=2 , p=0.999999947174418

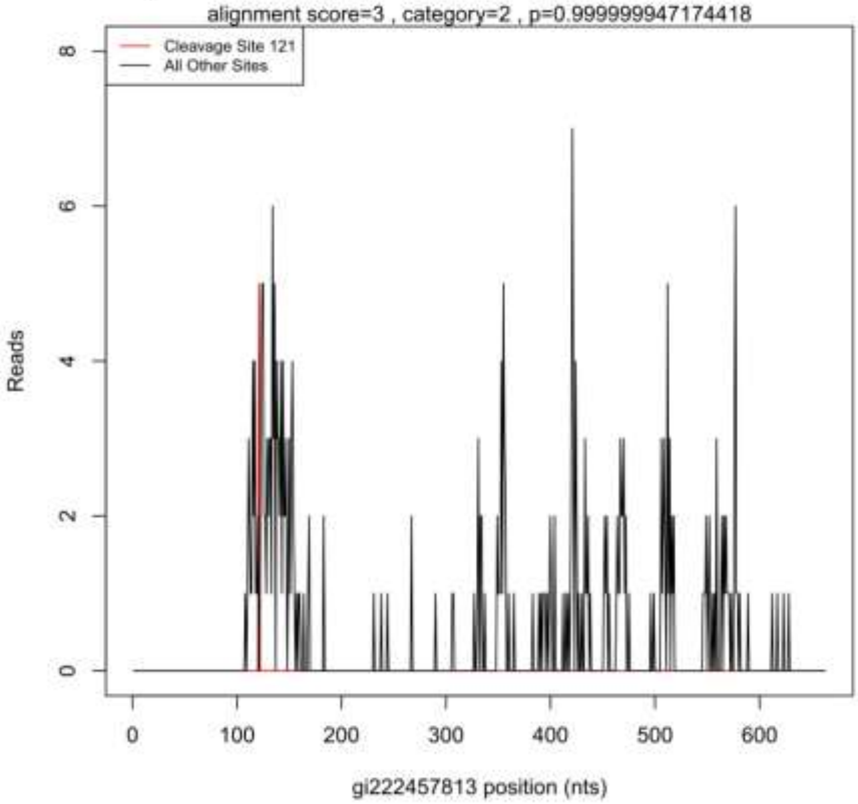

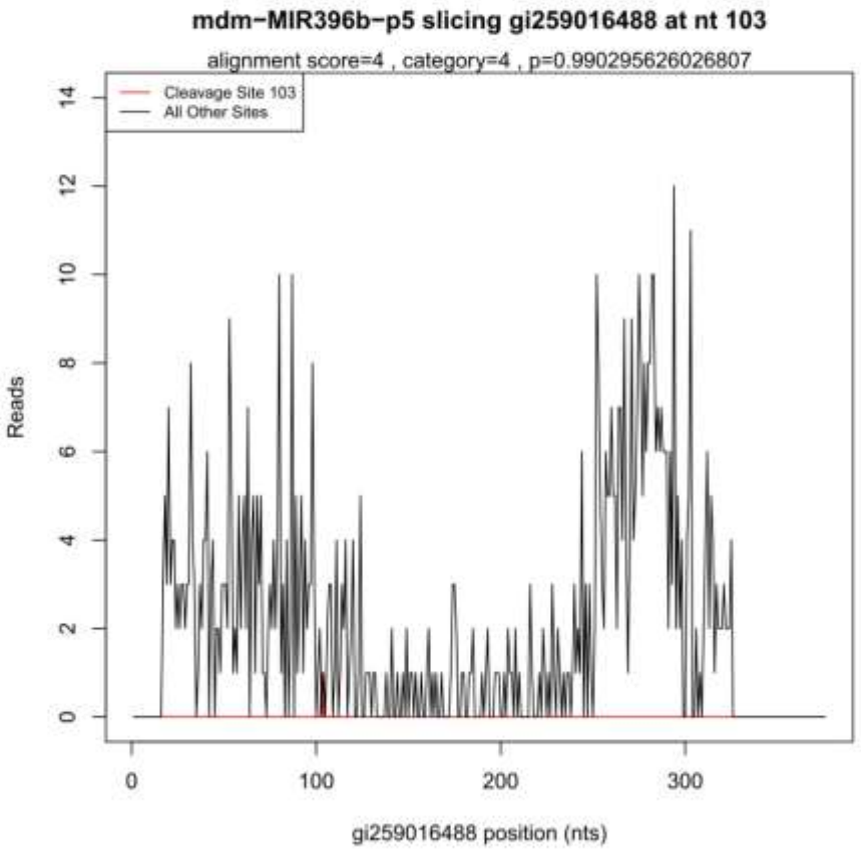
mdm−MIR396b−p5 slicing gi259016488 at nt 103
alignment score=4 , category=4 , p=0.990295626026807
Cleavage Site 103
All Other Sites
Reads
0
2
4
6
8
10
12
14
0
100
200
300
gi259016488 position (nts)

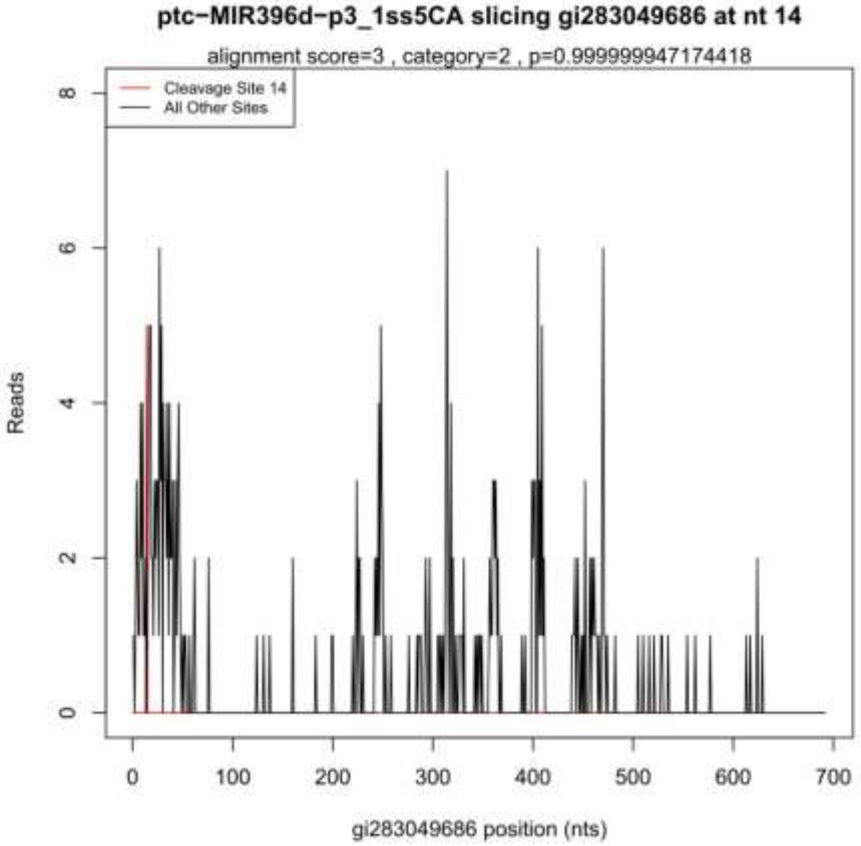
ptc−MIR396d−p3_1ss5CA slicing gi283049686 at nt 14
alignment score=3 , category=2 , p=0.999999947174418
Cleavage Site 14
All Other Sites
Reads
0
2
4
6
8
0
100
200
300
400
500
600
700
gi283049686 position (nts)

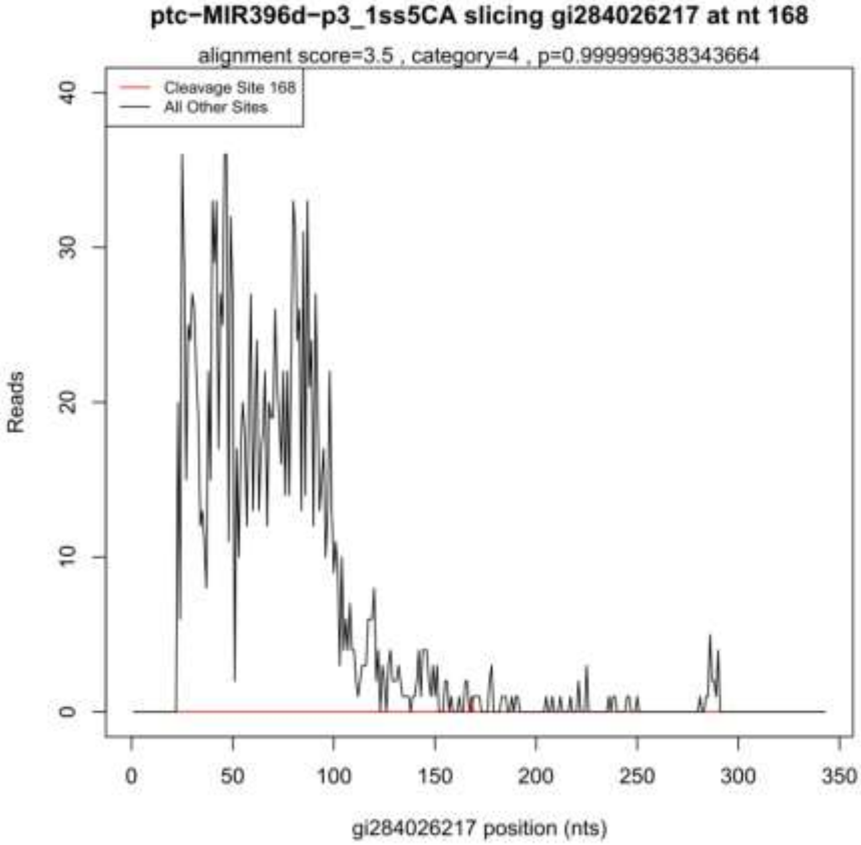
ptc−MIR396d−p3_1ss5CA slicing gi284026217 at nt 168
alignment score=3.5 , category=4 , p=0.999999638343664
Cleavage Site 168
All Other Sites
Reads
gi284026217 position (nts)

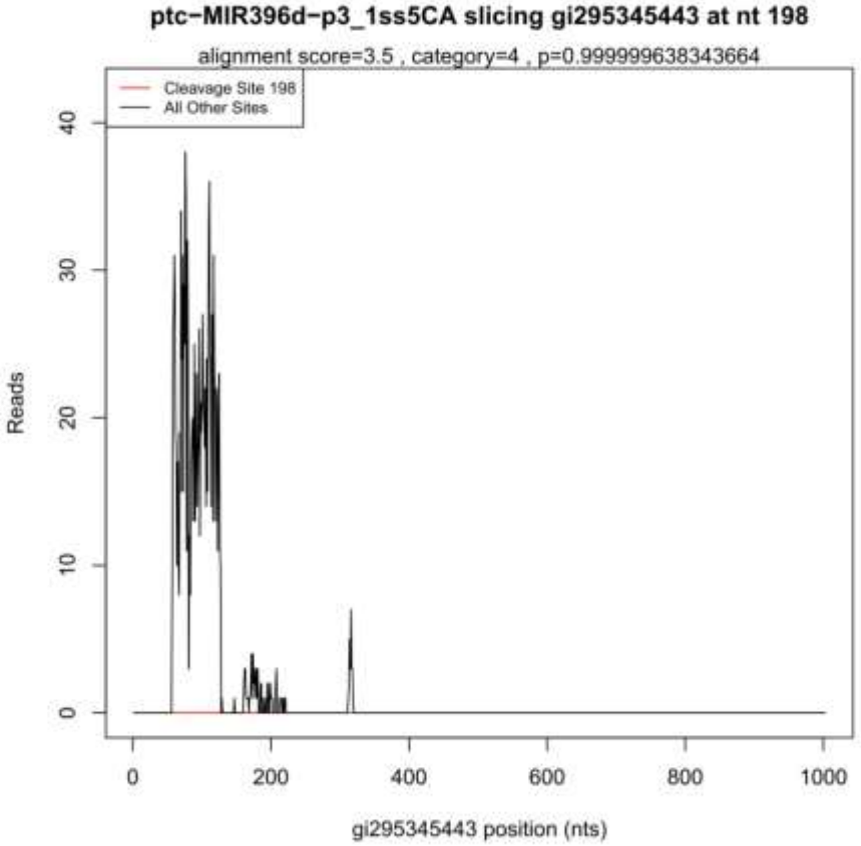
ptc−MIR396d−p3_1ss5CA slicing gi295345443 at nt 198
alignment score=3.5 , category=4 , p=0.999999638343664
Cleavage Site 198
All Other Sites
Reads
gi295345443 position (nts)

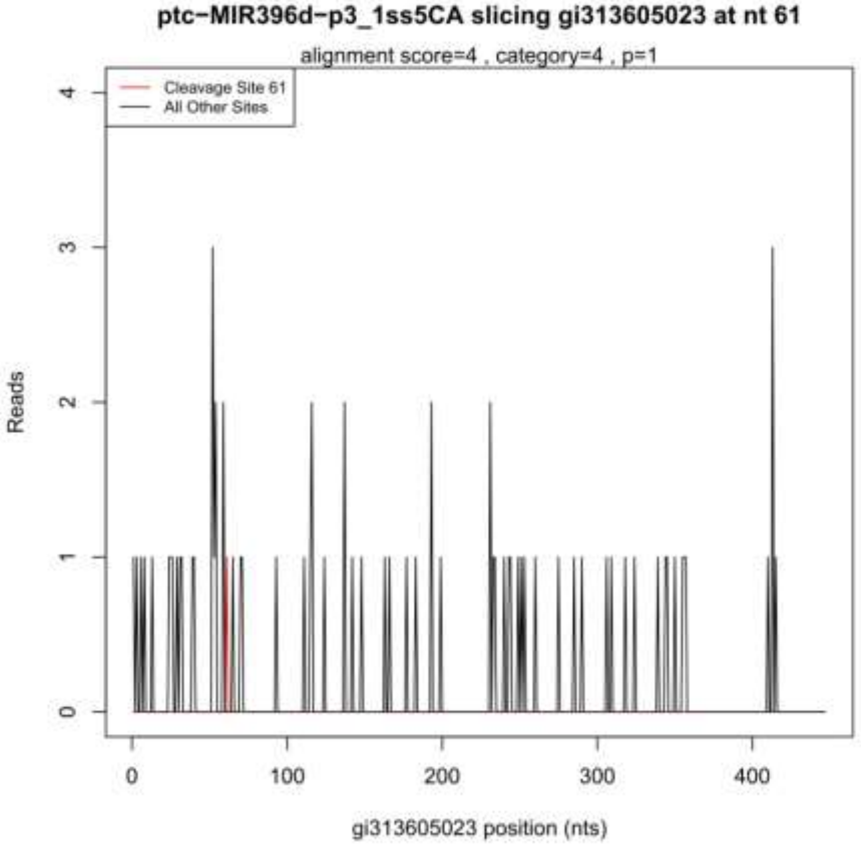
ptc−MIR396d−p3_1ss5CA slicing gi313605023 at nt 61
alignment score=4 , category=4 , p=1
Cleavage Site 61
All Other Sites
Reads
0
1
2
3
4
0
100
200
300
400
gi313605023 position (nts)

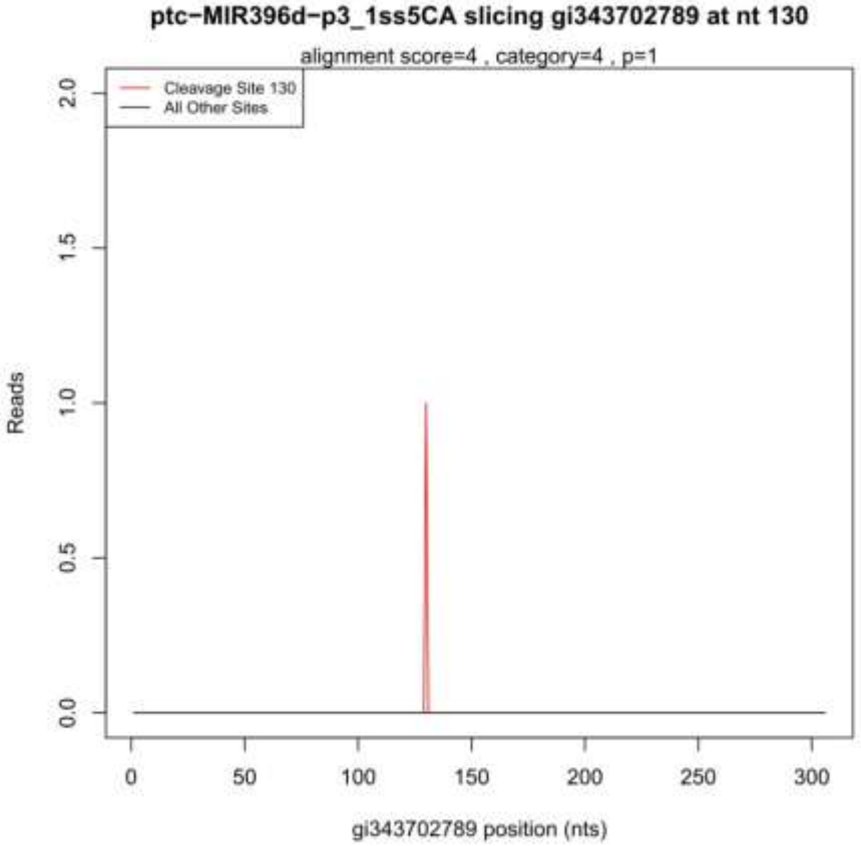
ptc−MIR396d−p3_1ss5CA slicing gi343702789 at nt 130
alignment score=4 , category=4 , p=1
Cleavage Site 130
All Other Sites
Reads
0.0
0.5
1.0
1.5
2.0
0
50
100
150
200
250
300
gi343702789 position (nts)

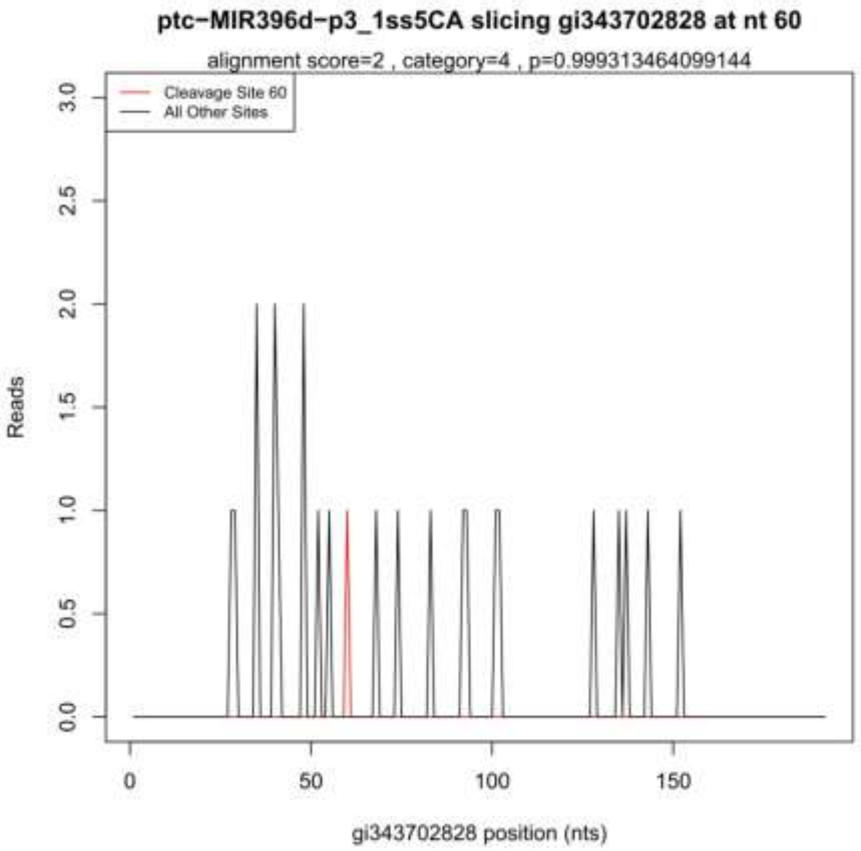
ptc−MIR396d−p3_1ss5CA slicing gi343702828 at nt 60
alignment score=2 , category=4 , p=0.999313464099144
Cleavage Site 60
All Other Sites
Reads
0.0
0.5
1.0
1.5
2.0
2.5
3.0
0
50
100
150
gi343702828 position (nts)

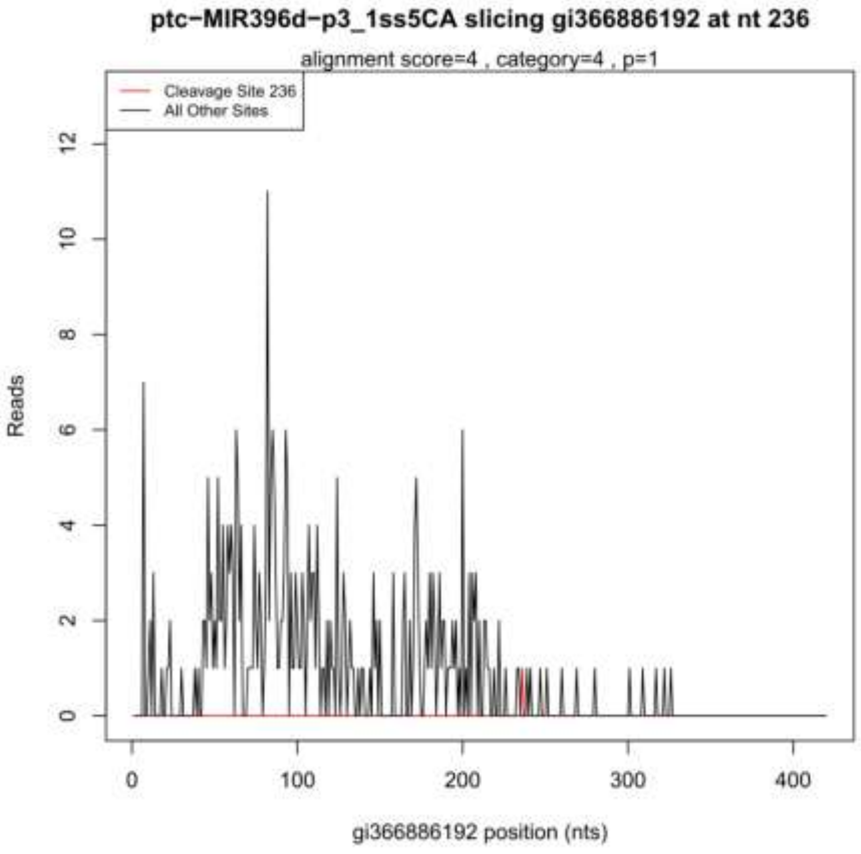
ptc−MIR396d−p3_1ss5CA slicing gi366886192 at nt 236
alignment score=4 , category=4 , p=1
Cleavage Site 236
All Other Sites
Reads
0
2
4
6
8
10
12
0
100
200
300
400
gi366886192 position (nts)

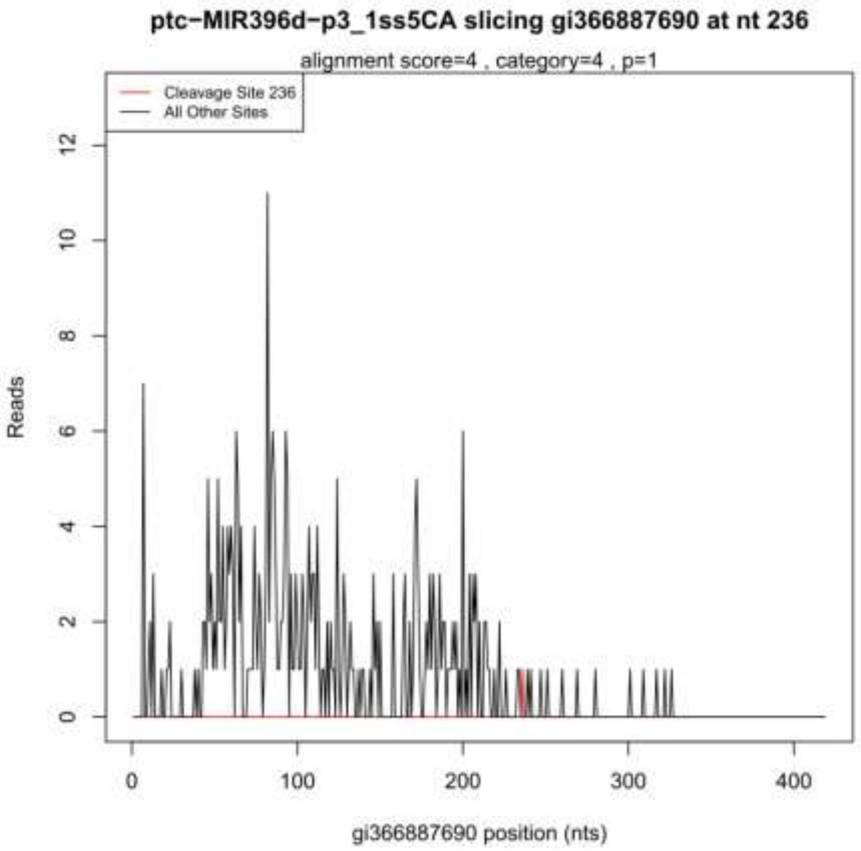
ptc−MIR396d−p3_1ss5CA slicing gi366887690 at nt 236
alignment score=4 , category=4 , p=1
Cleavage Site 236
All Other Sites
Reads
0
2
4
6
8
10
12
0
100
200
300
400
gi366887690 position (nts)

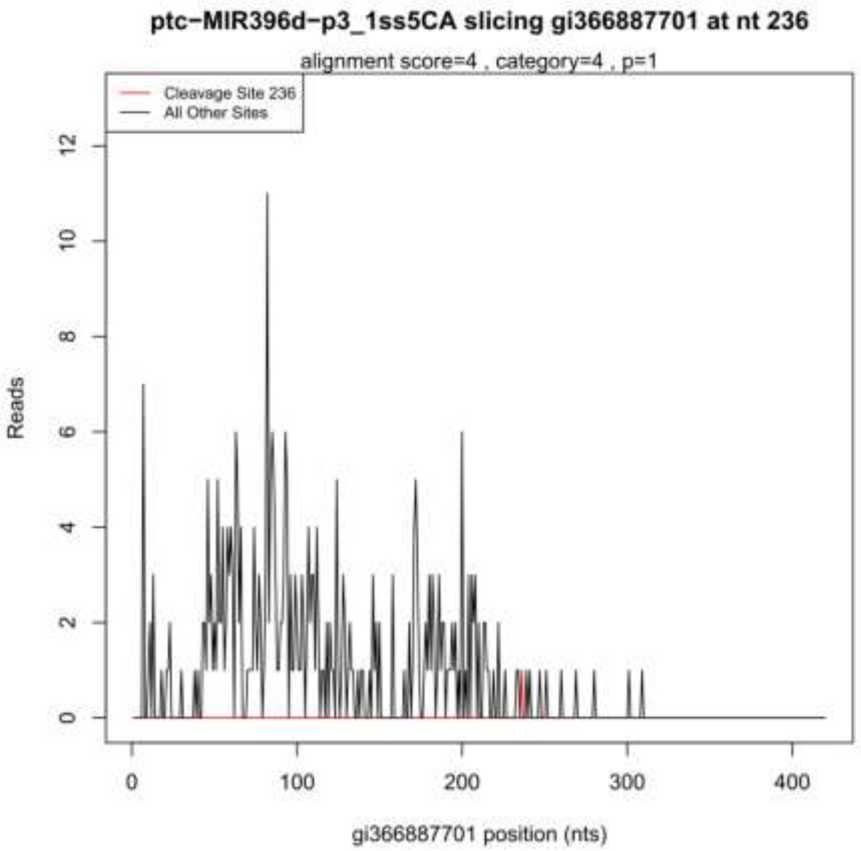
ptc−MIR396d−p3_1ss5CA slicing gi366887701 at nt 236
alignment score=4 , category=4 , p=1
Cleavage Site 236
All Other Sites
Reads
0
2
4
6
8
10
12
0
100
200
300
400
gi366887701 position (nts)

ptc−MIR396d−p3_1ss5CA slicing gi366888481 at nt 146

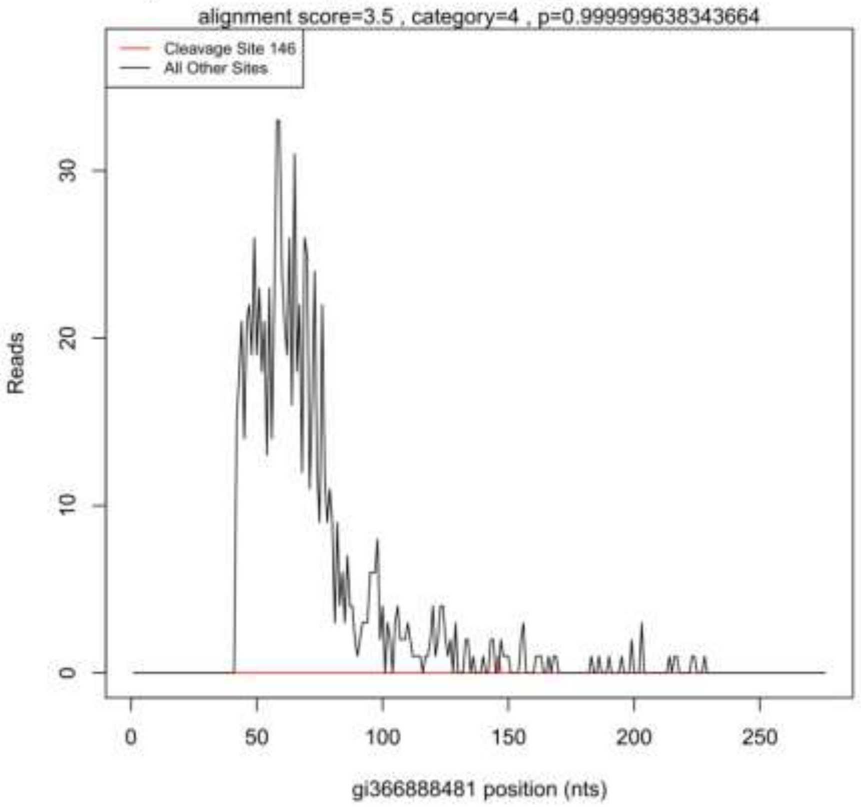

ptc−MIR396d−p3_1ss5CA slicing gi366888652 at nt 277

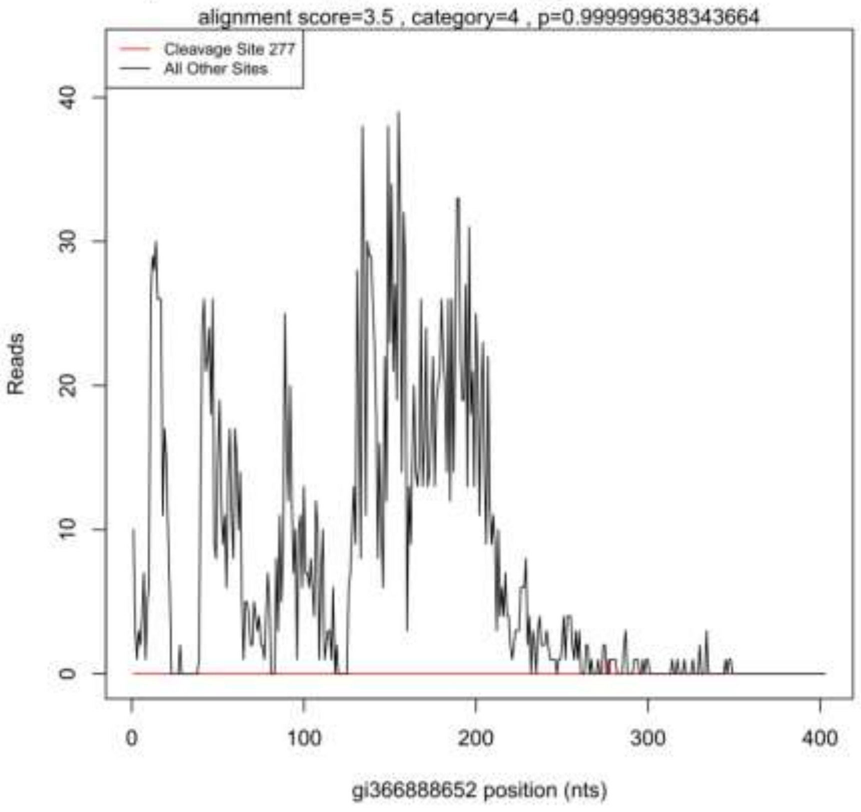

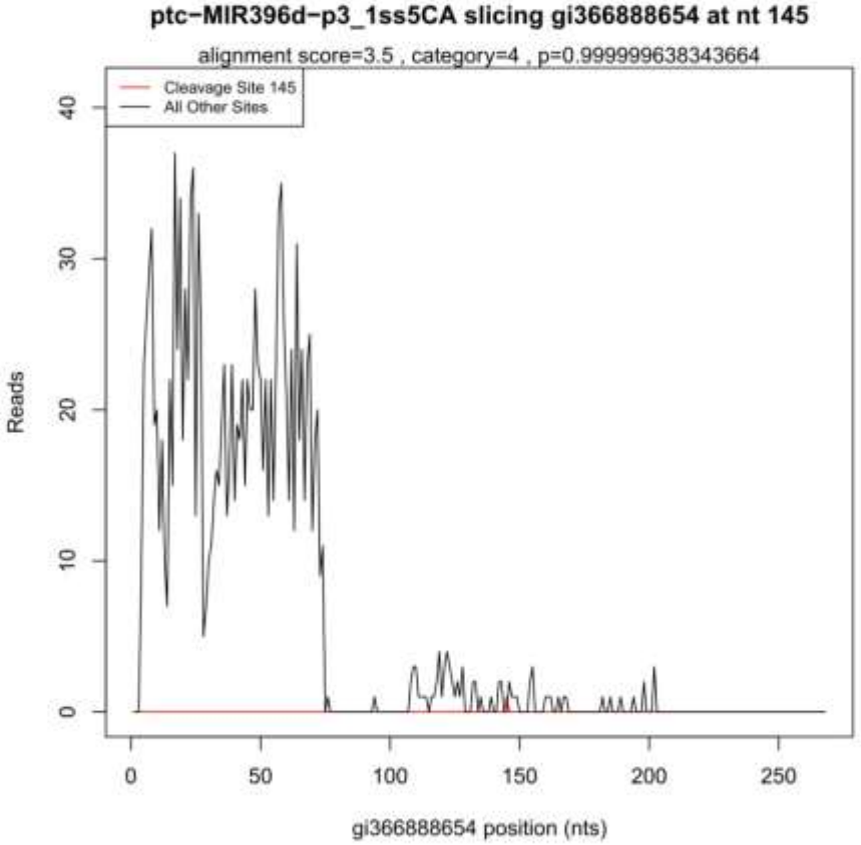

ptc−MIR396d−p3_1ss5CA slicing gi366888654 at nt 145
alignment score=3.5 , category=4 , p=0.999999638343664
Cleavage Site 145
All Other Sites
Reads
0
10
20
30
40
0
50
100
150
200
250
gi366888654 position (nts)

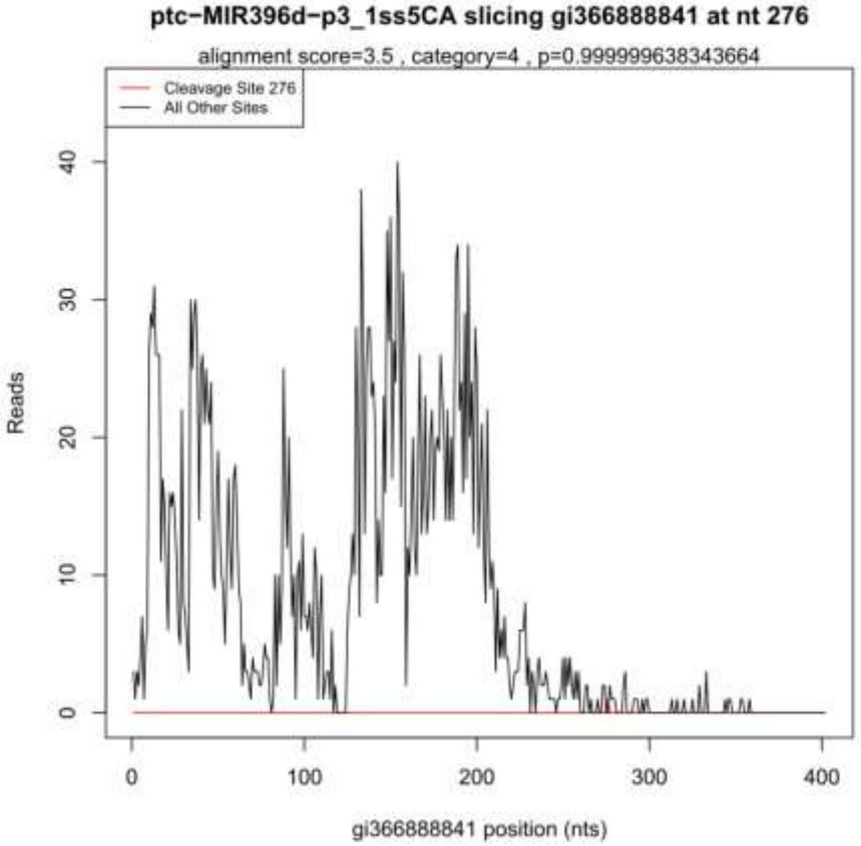

ptc−MIR396d−p3_1ss5CA slicing gi366888841 at nt 276
alignment score=3.5 , category=4 , p=0.999999638343664
Cleavage Site 276
All Other Sites
Reads
0
10
20
30
40
0
100
200
300
400
gi366888841 position (nts)

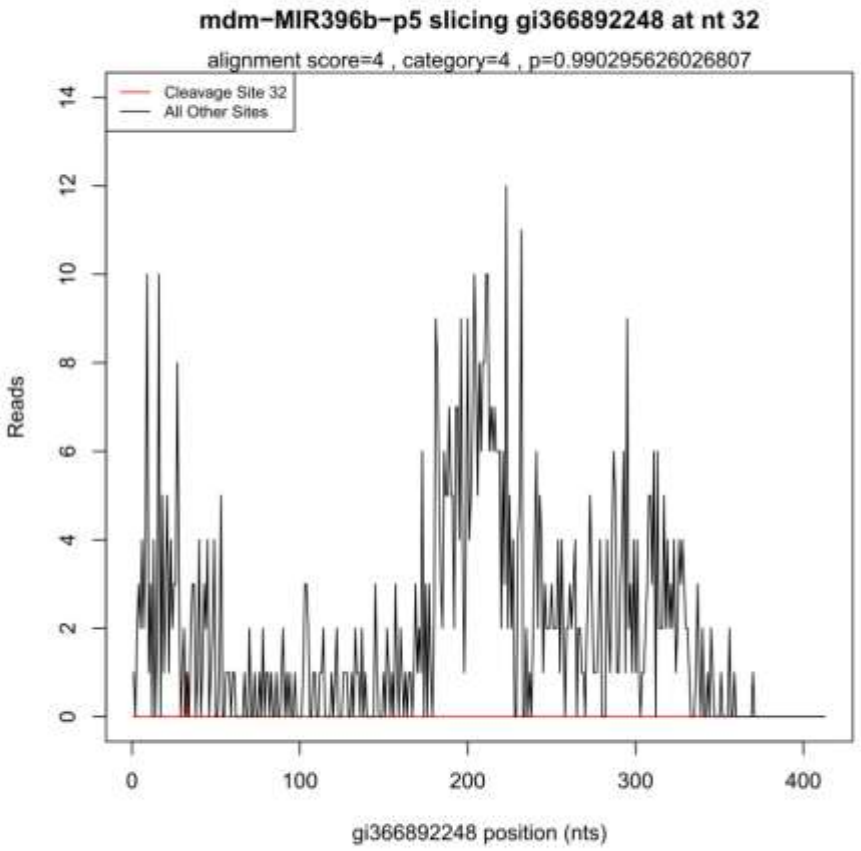
mdm−MIR396b−p5 slicing gi366892248 at nt 32
alignment score=4 , category=4 , p=0.990295626026807
Cleavage Site 32
All Other Sites
Reads
gi366892248 position (nts)
0
2
4
6
8
10
12
14
0
100
200
300
400

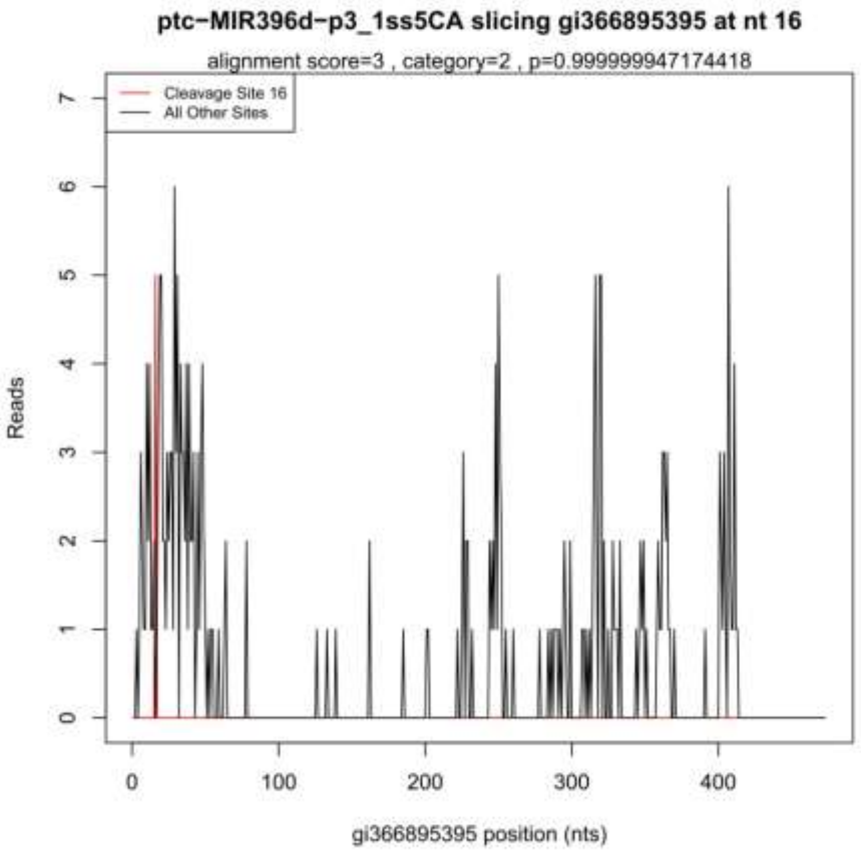
ptc−MIR396d−p3_1ss5CA slicing gi366895395 at nt 16
alignment score=3 , category=2 , p=0.999999947174418
Cleavage Site 16
All Other Sites
Reads
gi366895395 position (nts)
0
1
2
3
4
5
6
7
0
100
200
300
400

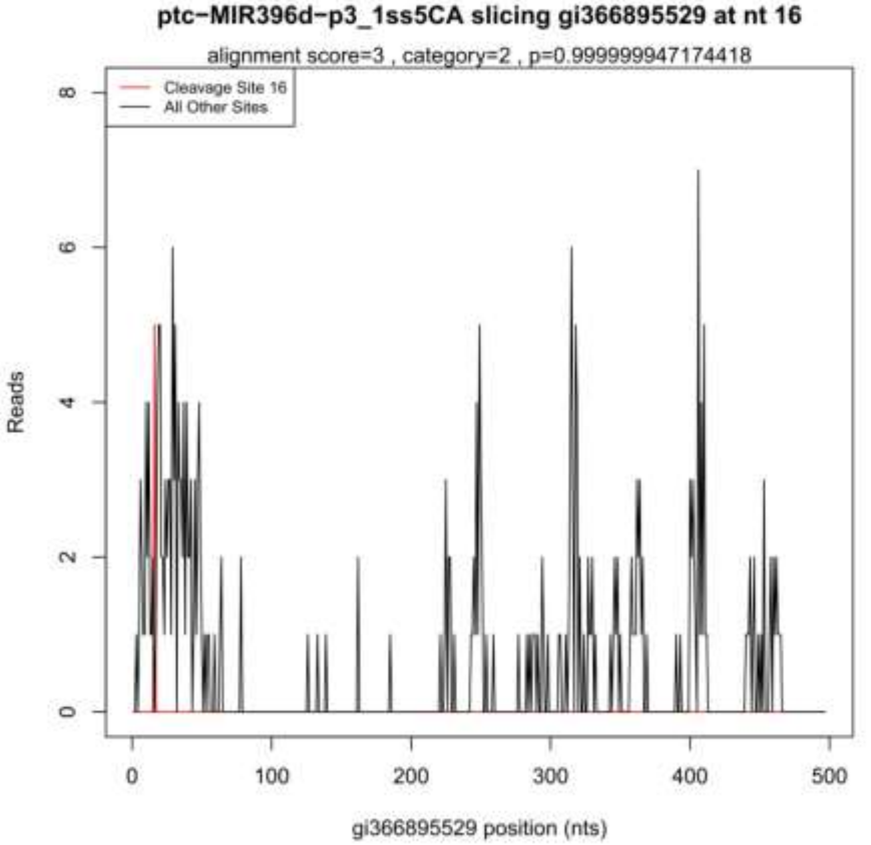

ptc-MIR396d-p3_1ss5CA slicing gi366895529 at nt 16
alignment score=3 , category=2 , p=0.999999947174418
Cleavage Site 16
All Other Sites
Reads
0
2
4
6
8
0
100
200
300
400
500
gi366895529 position (nts)

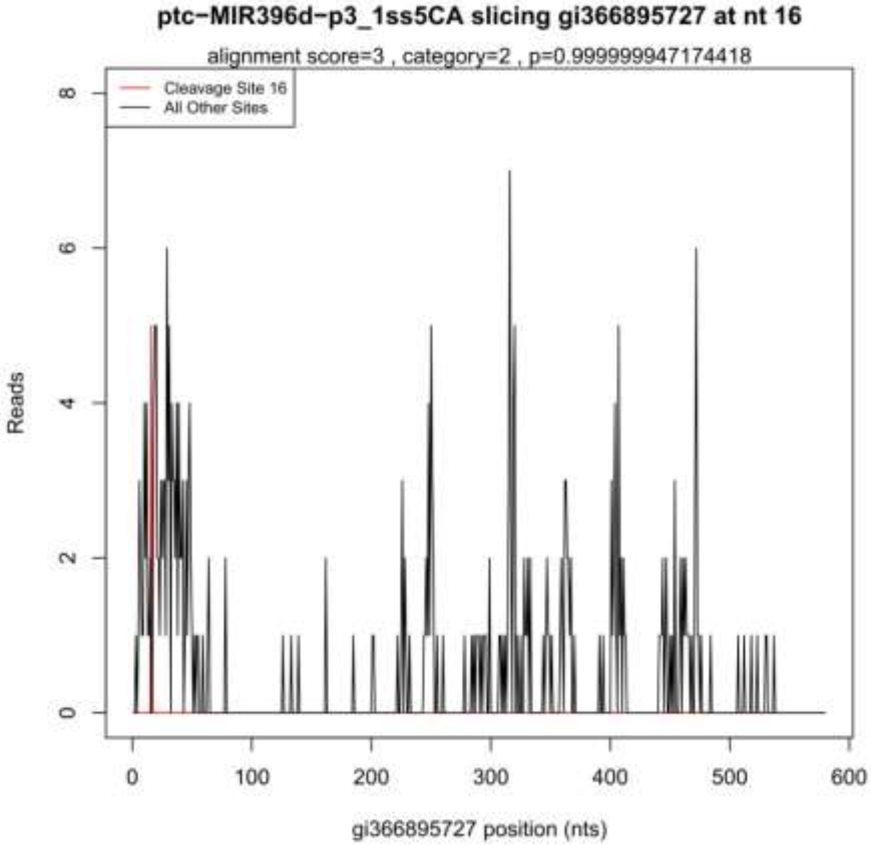

ptc-MIR396d-p3_1ss5CA slicing gi366895727 at nt 16
alignment score=3 , category=2 , p=0.999999947174418
Cleavage Site 16
All Other Sites
Reads
0
2
4
6
8
0
100
200
300
400
500
600
gi366895727 position (nts)

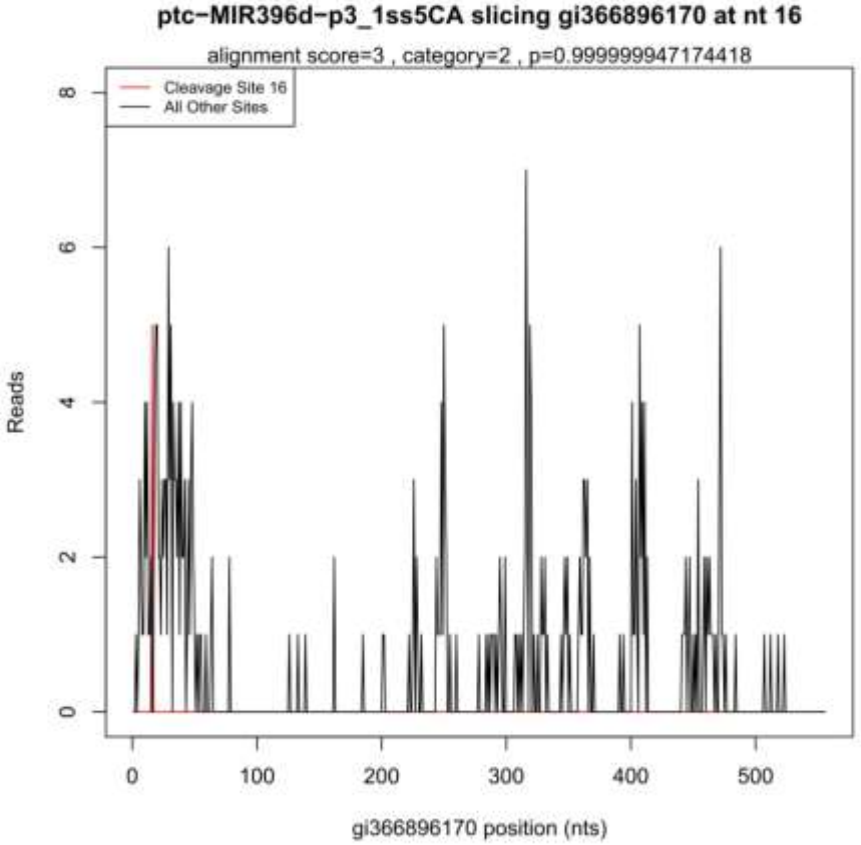

ptc−MIR396d−p3_1ss5CA slicing gi366896170 at nt 16
alignment score=3 , category=2 , p=0.999999947174418
Cleavage Site 16
All Other Sites
Reads
0
2
4
6
8
0
100
200
300
400
500
gi366896170 position (nts)

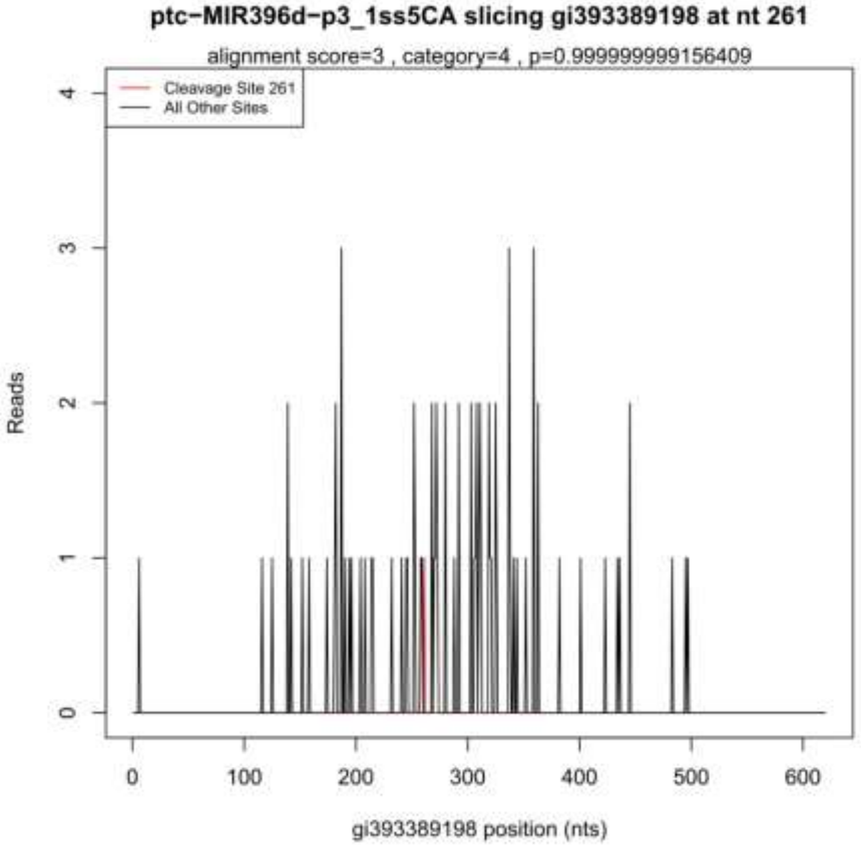

ptc−MIR396d−p3_1ss5CA slicing gi393389198 at nt 261
alignment score=3 , category=4 , p=0.999999999156409
Cleavage Site 261
All Other Sites
Reads
0
1
2
3
4
0
100
200
300
400
500
600
gi393389198 position (nts)

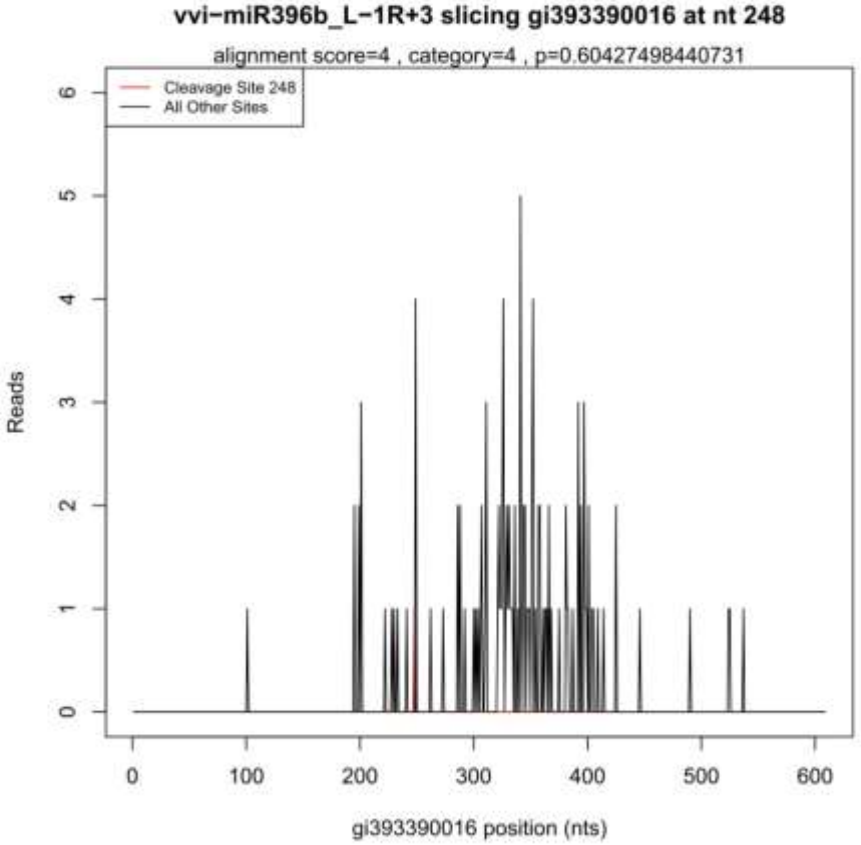
vvi−miR396b_L−1R+3 slicing gi393390016 at nt 248
alignment score=4 , category=4 , p=0.60427498440731
Cleavage Site 248
All Other Sites
Reads
0
1
2
3
4
5
6
0
100
200
300
400
500
600
gi393390016 position (nts)

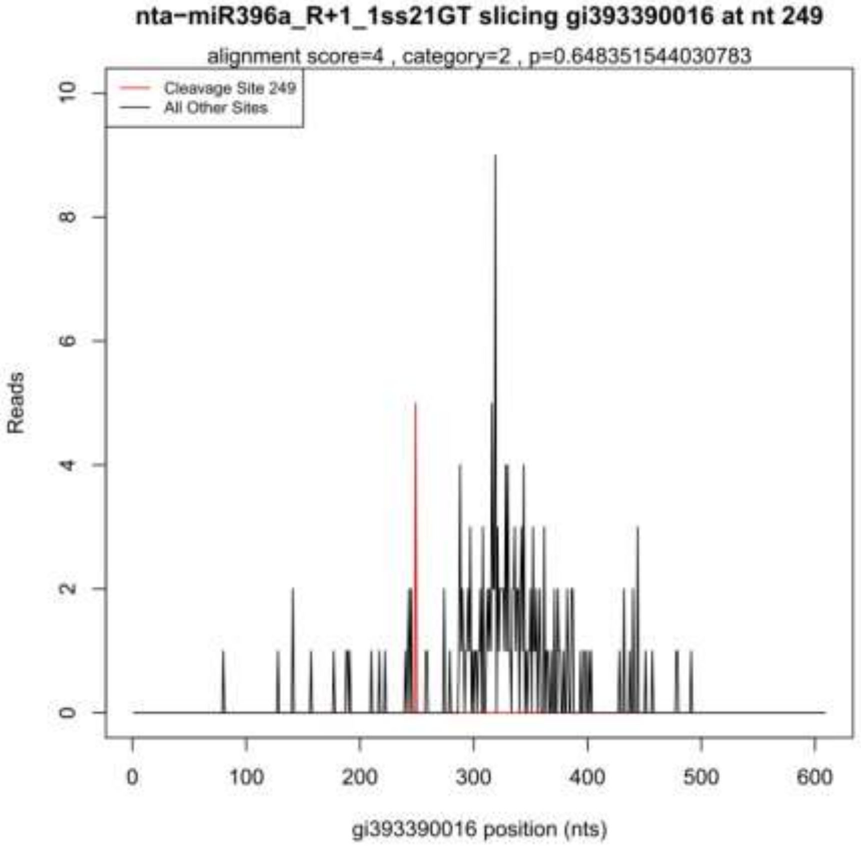
nta−miR396a_R+1_1ss21GT slicing gi393390016 at nt 249
alignment score=4 , category=2 , p=0.648351544030783
Cleavage Site 249
All Other Sites
Reads
0
2
4
6
8
10
0
100
200
300
400
500
600
gi393390016 position (nts)

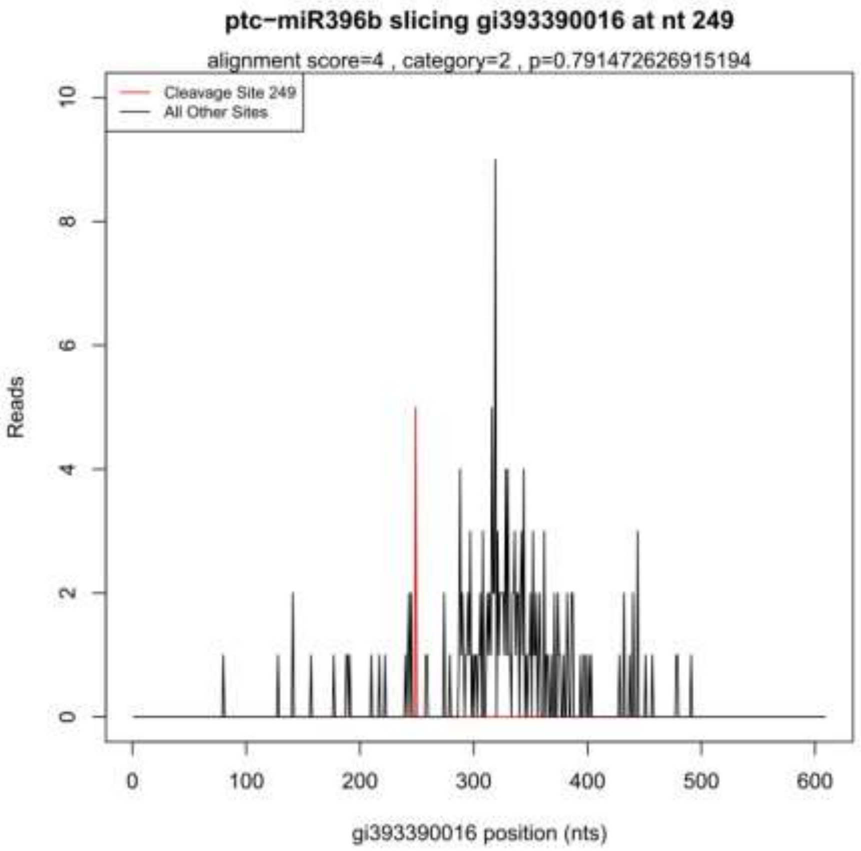
ptc−miR396b slicing gi393390016 at nt 249
alignment score=4 , category=2 , p=0.791472626915194
Cleavage Site 249
All Other Sites
Reads
0
2
4
6
8
10
0
100
200
300
400
500
600
gi393390016 position (nts)

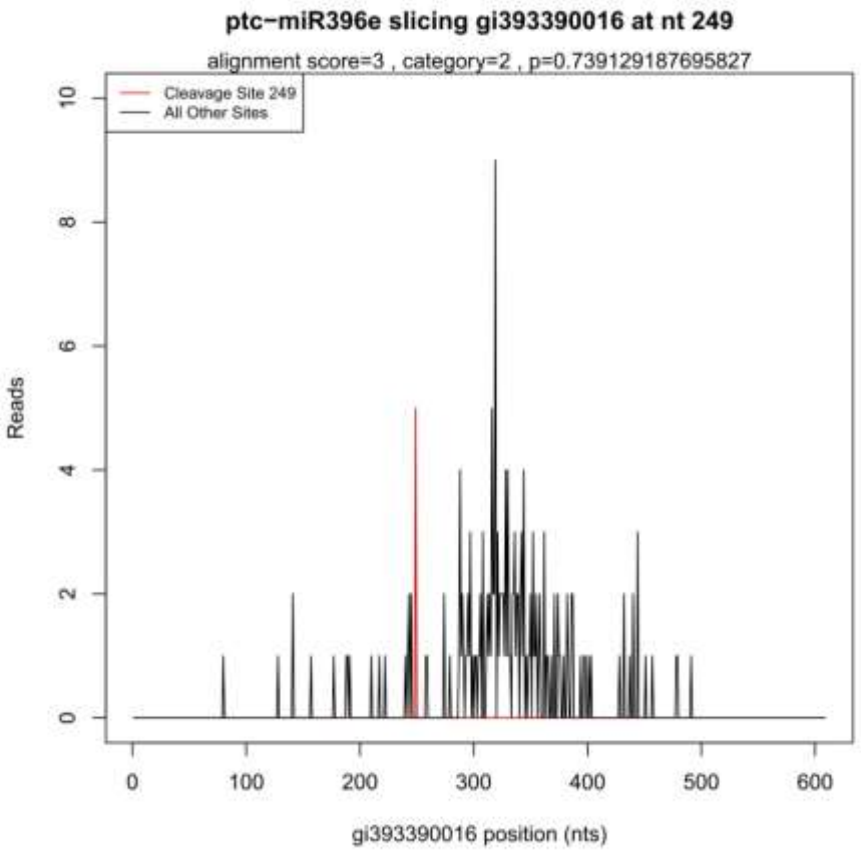
ptc−miR396e slicing gi393390016 at nt 249
alignment score=3 , category=2 , p=0.739129187695827
Cleavage Site 249
All Other Sites
Reads
0
2
4
6
8
10
0
100
200
300
400
500
600
gi393390016 position (nts)

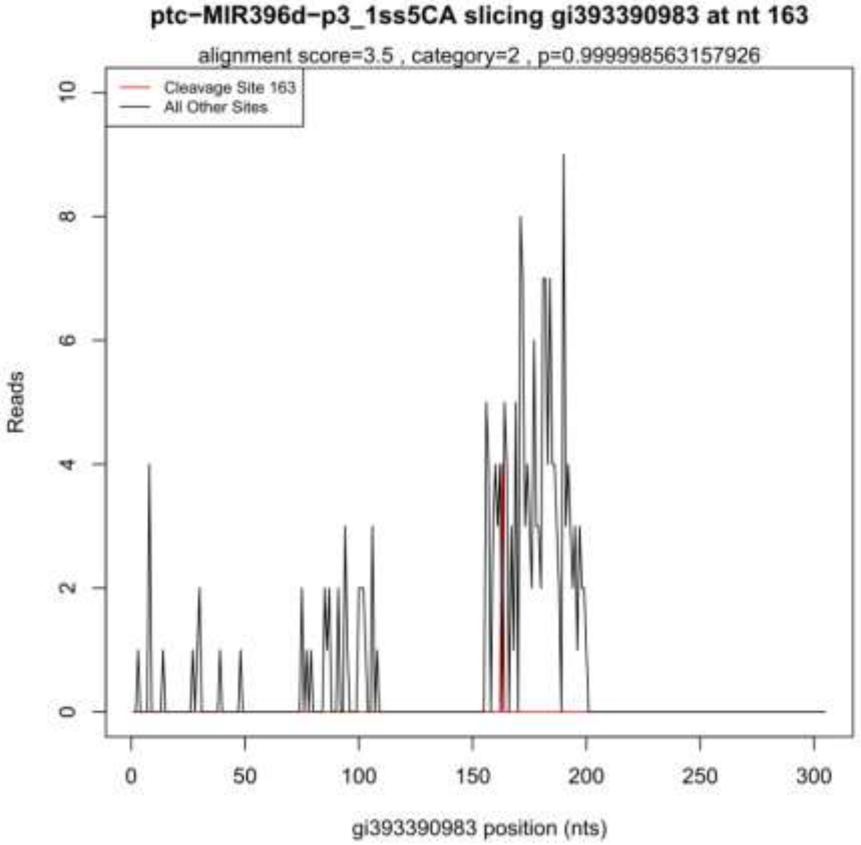
ptc-MIR396d-p3_1ss5CA slicing gi393390983 at nt 163
alignment score=3.5 , category=2 , p=0.999998563157926
Cleavage Site 163
All Other Sites
Reads
0
2
4
6
8
10
0
50
100
150
200
250
300
gi393390983 position (nts)

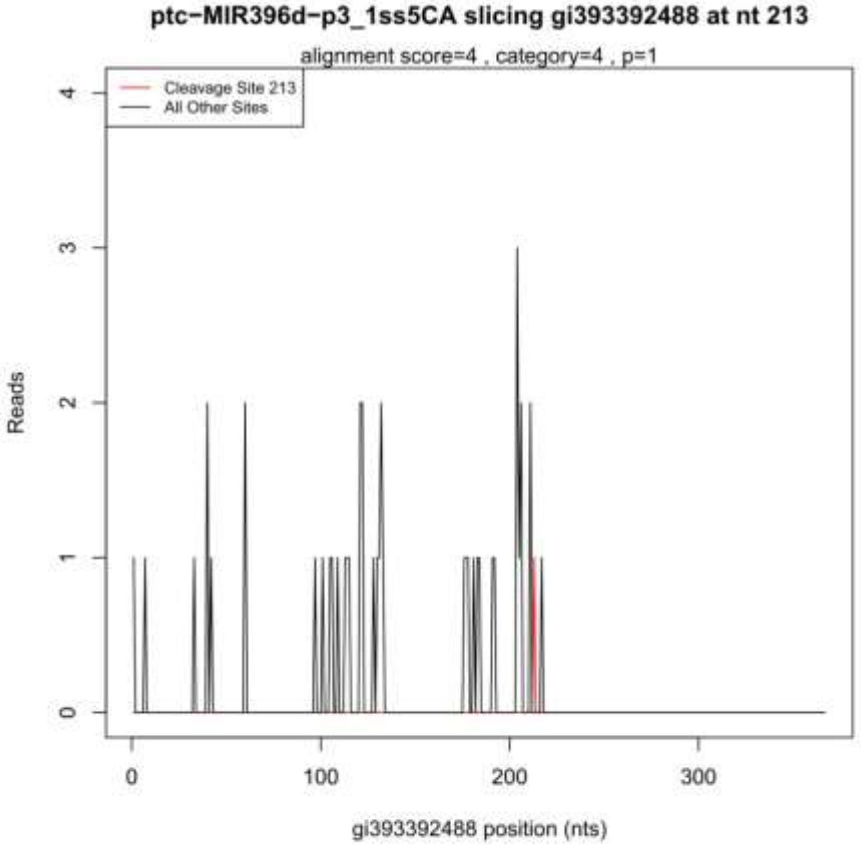
ptc-MIR396d-p3_1ss5CA slicing gi393392488 at nt 213
alignment score=4 , category=4 , p=1
Cleavage Site 213
All Other Sites
Reads
0
1
2
3
4
0
100
200
300
gi393392488 position (nts)

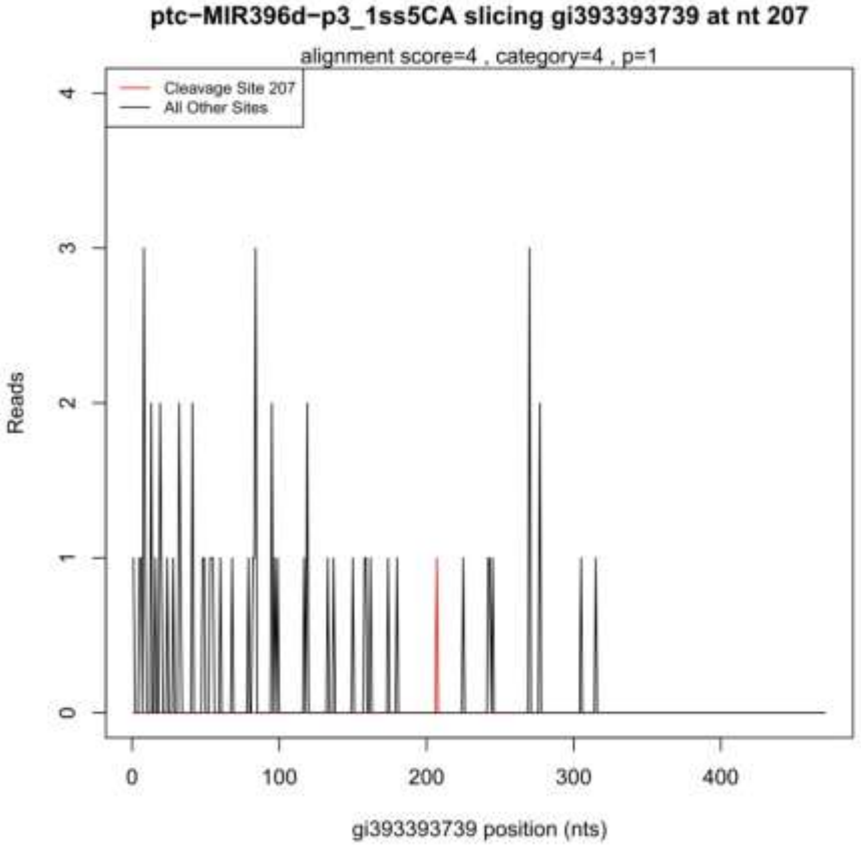
ptc-MIR396d-p3_1ss5CA slicing gi393393739 at nt 207
alignment score=4 , category=4 , p=1
Cleavage Site 207
All Other Sites
Reads
0
1
2
3
4
0
100
200
300
400
gi393393739 position (nts)

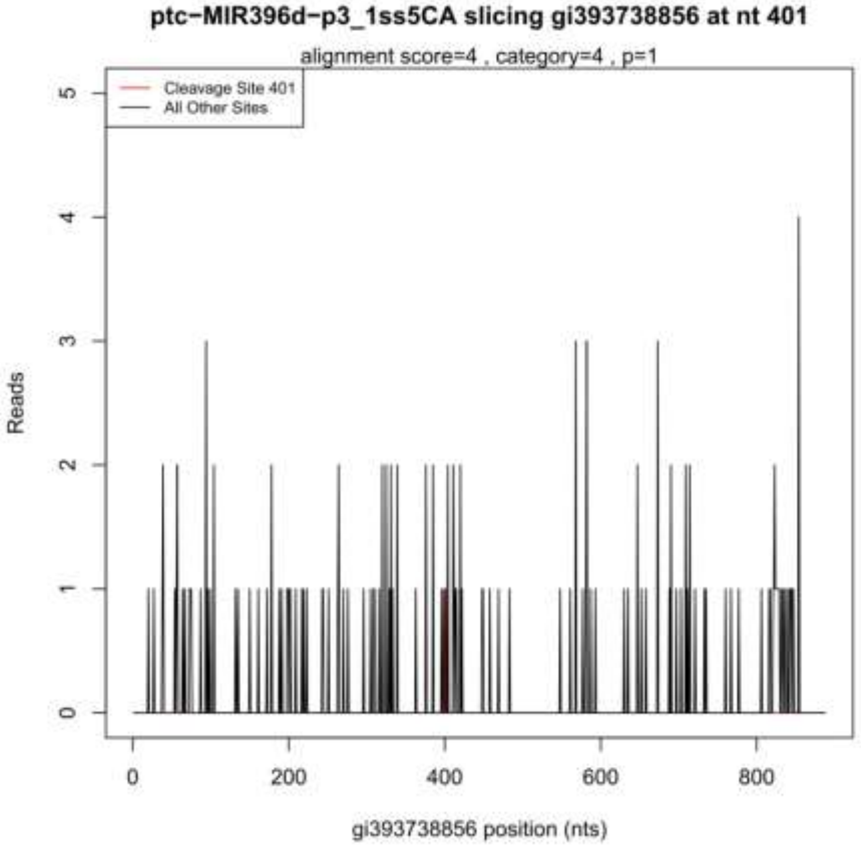
ptc-MIR396d-p3_1ss5CA slicing gi393738856 at nt 401
alignment score=4 , category=4 , p=1
Cleavage Site 401
All Other Sites
Reads
0
1
2
3
4
5
0
200
400
600
800
gi393738856 position (nts)

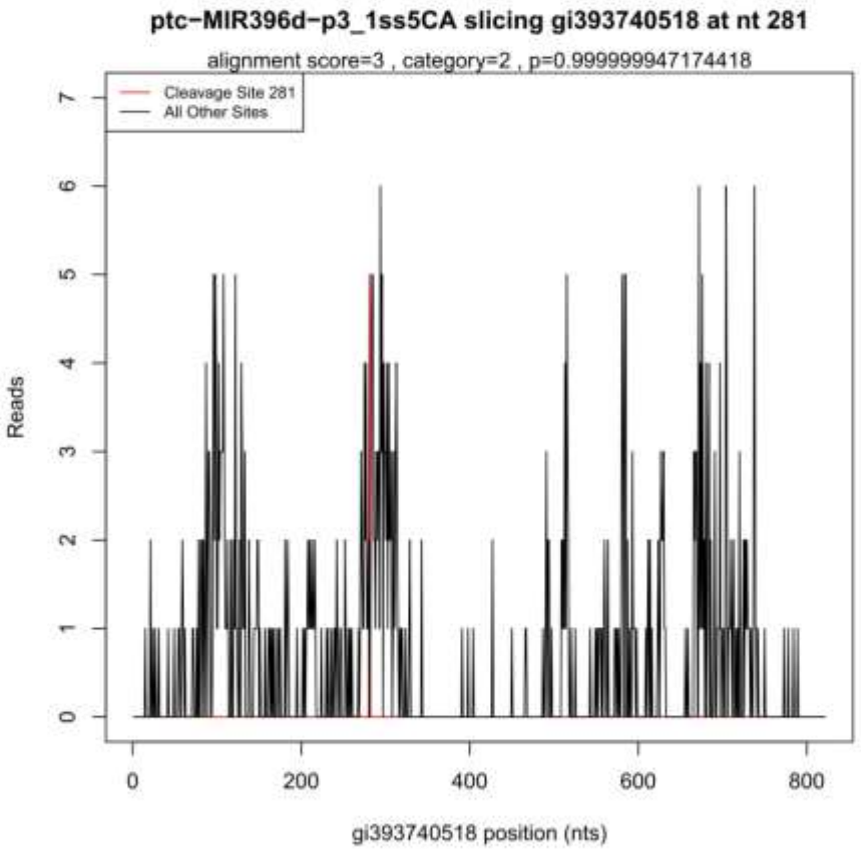

ptc−MIR396d−p3_1ss5CA slicing gi393740518 at nt 281
alignment score=3 , category=2 , p=0.999999947174418
Cleavage Site 281
All Other Sites
Reads
0
1
2
3
4
5
6
7
0
200
400
600
800
gi393740518 position (nts)

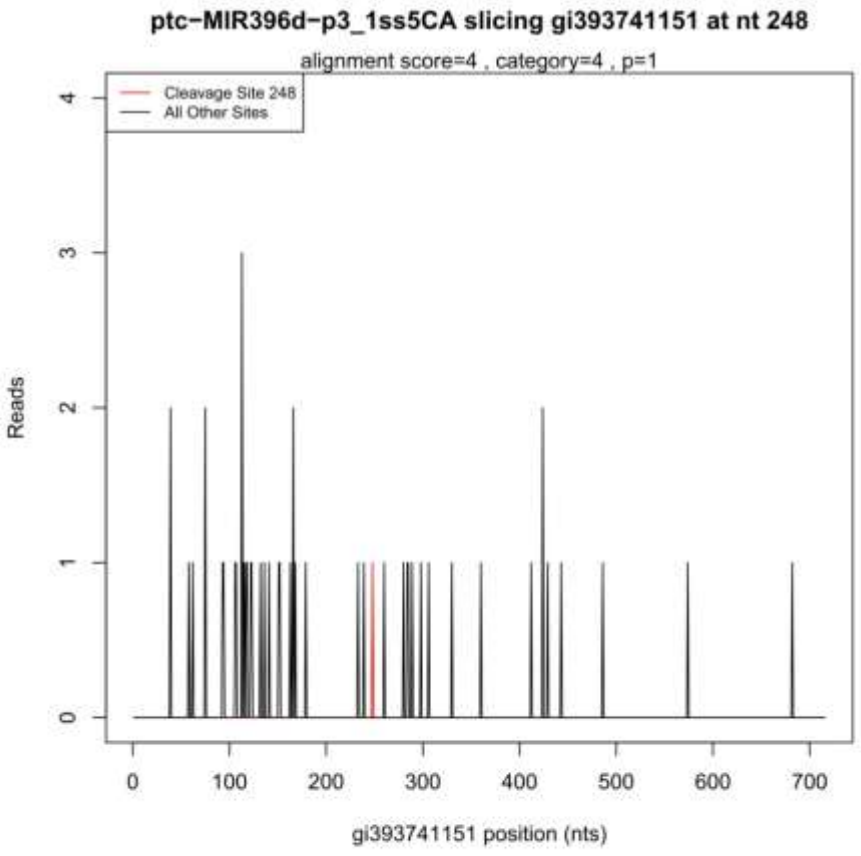

ptc−MIR396d−p3_1ss5CA slicing gi393741151 at nt 248
alignment score=4 , category=4 , p=1
Cleavage Site 248
All Other Sites
Reads
0
1
2
3
4
0
100
200
300
400
500
600
700
gi393741151 position (nts)

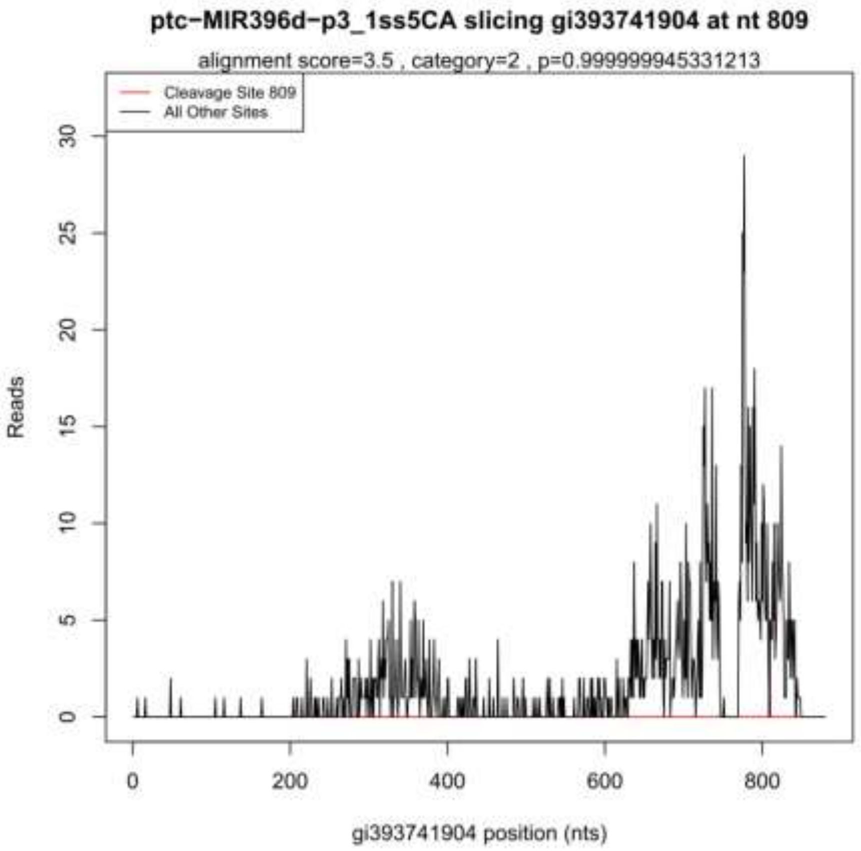

ptc−MIR396d−p3_1ss5CA slicing gi393741904 at nt 809
alignment score=3.5 , category=2 , p=0.999999945331213
Cleavage Site 809
All Other Sites
Reads
0
5
10
15
20
25
30
0
200
400
600
800
gi393741904 position (nts)

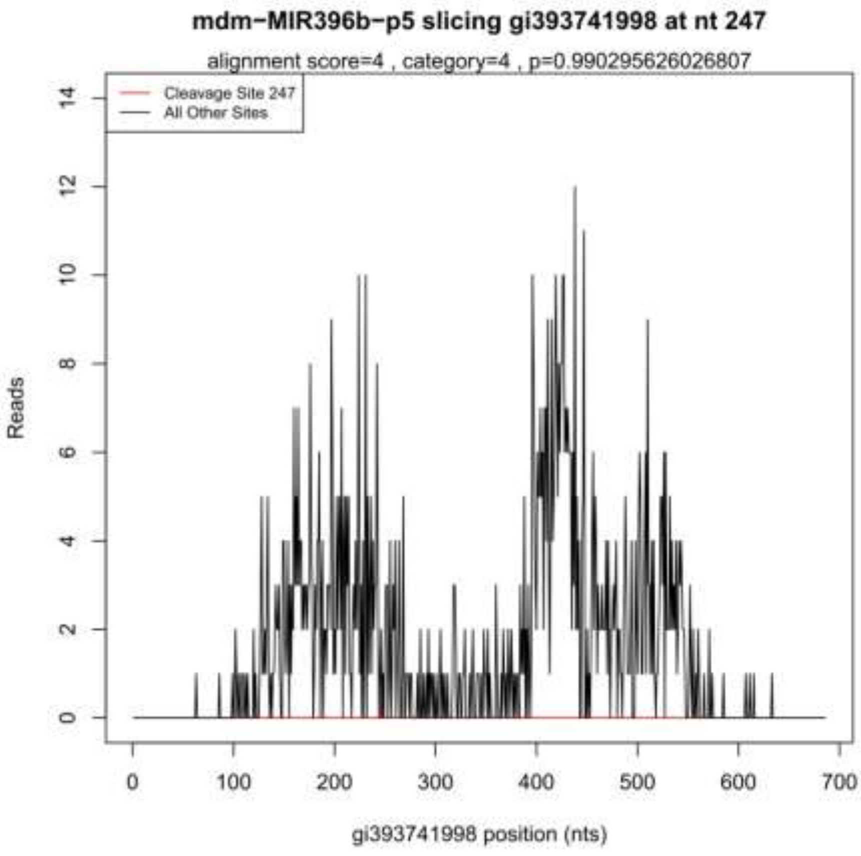

mdm−MIR396b−p5 slicing gi393741998 at nt 247
alignment score=4 , category=4 , p=0.990295626026807
Cleavage Site 247
All Other Sites
Reads
0
2
4
6
8
10
12
14
0
100
200
300
400
500
600
700
gi393741998 position (nts)

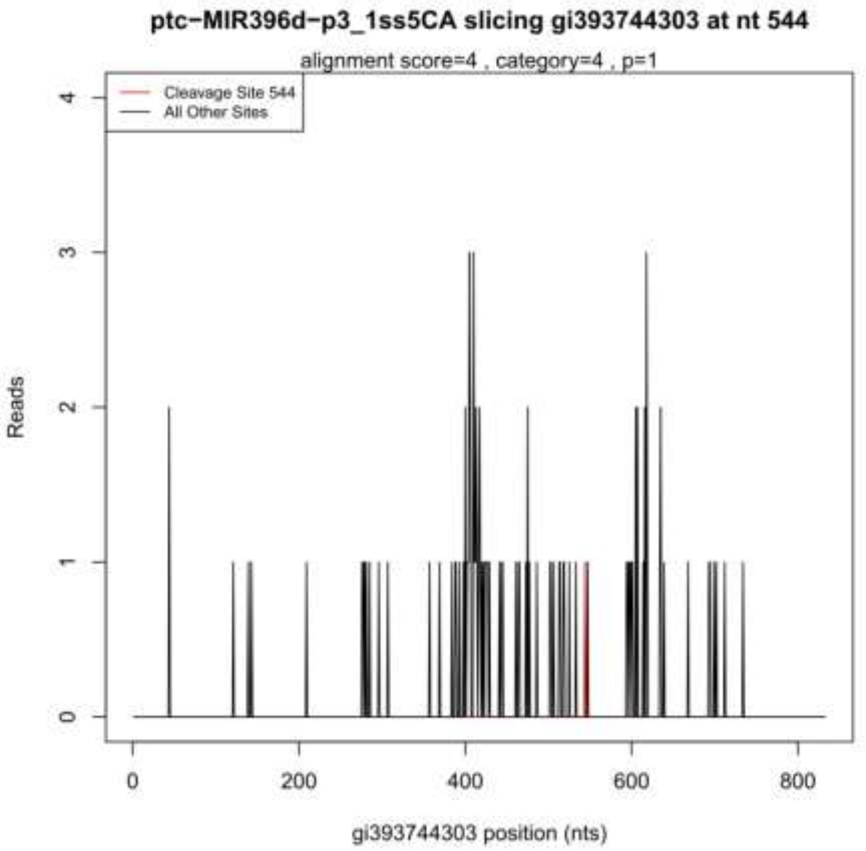

ptc−MIR396d−p3_1ss5CA slicing gi393744303 at nt 544
alignment score=4 , category=4 , p=1
Cleavage Site 544
All Other Sites
Reads
0
1
2
3
4
0
200
400
600
800
gi393744303 position (nts)

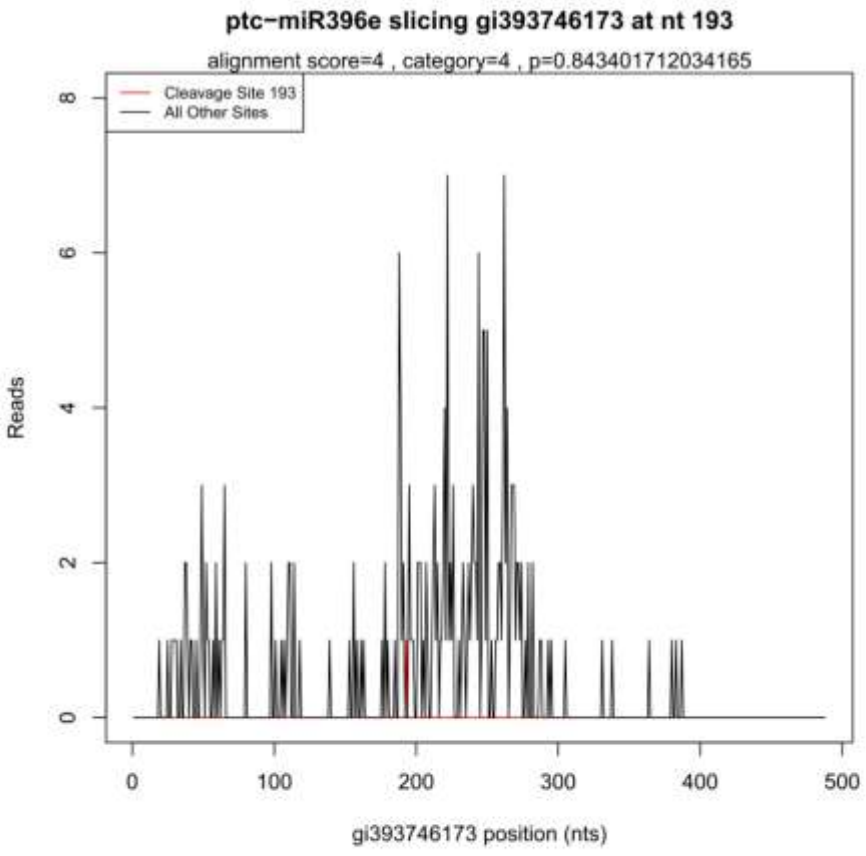

ptc−miR396e slicing gi393746173 at nt 193
alignment score=4 , category=4 , p=0.843401712034165
Cleavage Site 193
All Other Sites
Reads
0
2
4
6
8
0
100
200
300
400
500
gi393746173 position (nts)

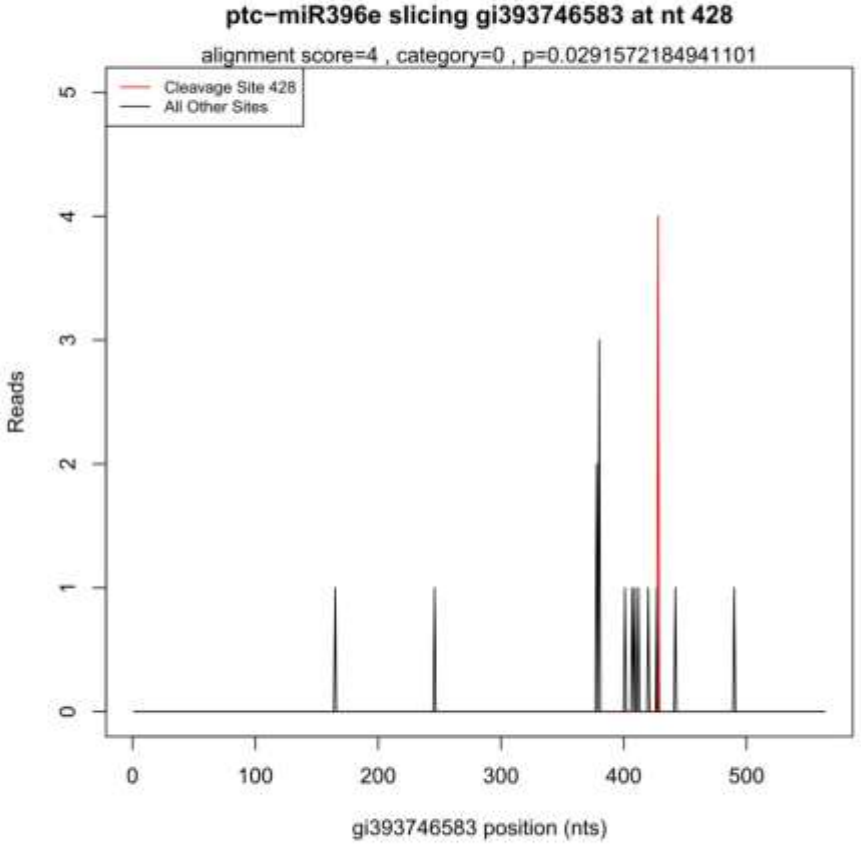
ptc−miR396e slicing gi393746583 at nt 428
alignment score=4 , category=0 , p=0.0291572184941101
Cleavage Site 428
All Other Sites
Reads
0
1
2
3
4
5
0
100
200
300
400
500
gi393746583 position (nts)

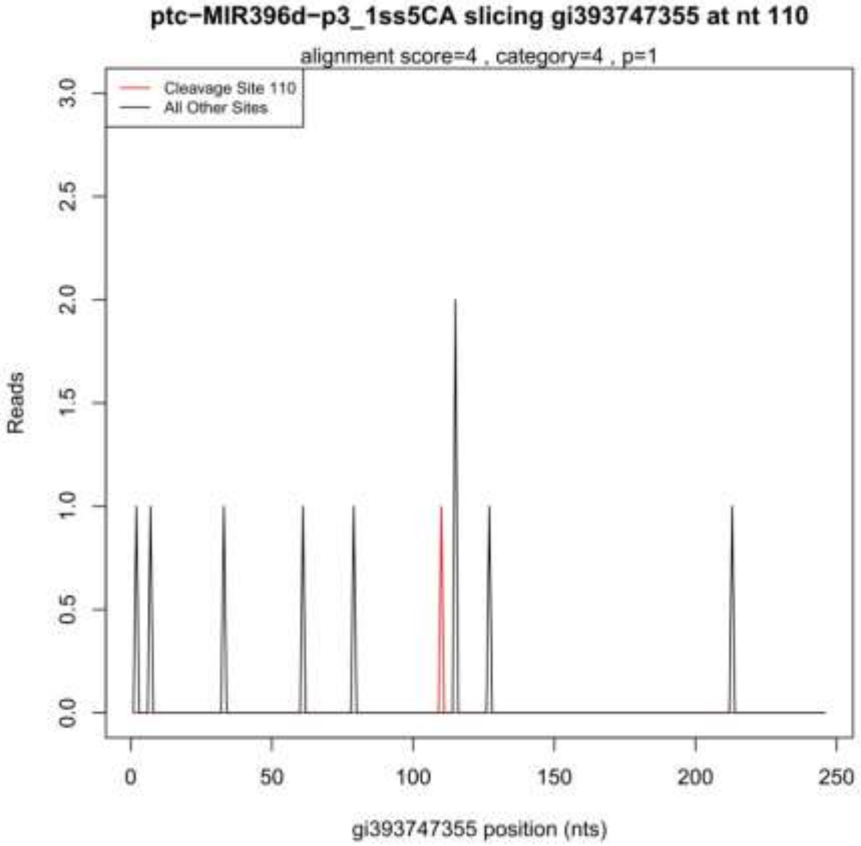
ptc−MIR396d−p3_1ss5CA slicing gi393747355 at nt 110
alignment score=4 , category=4 , p=1
Cleavage Site 110
All Other Sites
Reads
0.0
0.5
1.0
1.5
2.0
2.5
3.0
0
50
100
150
200
250
gi393747355 position (nts)

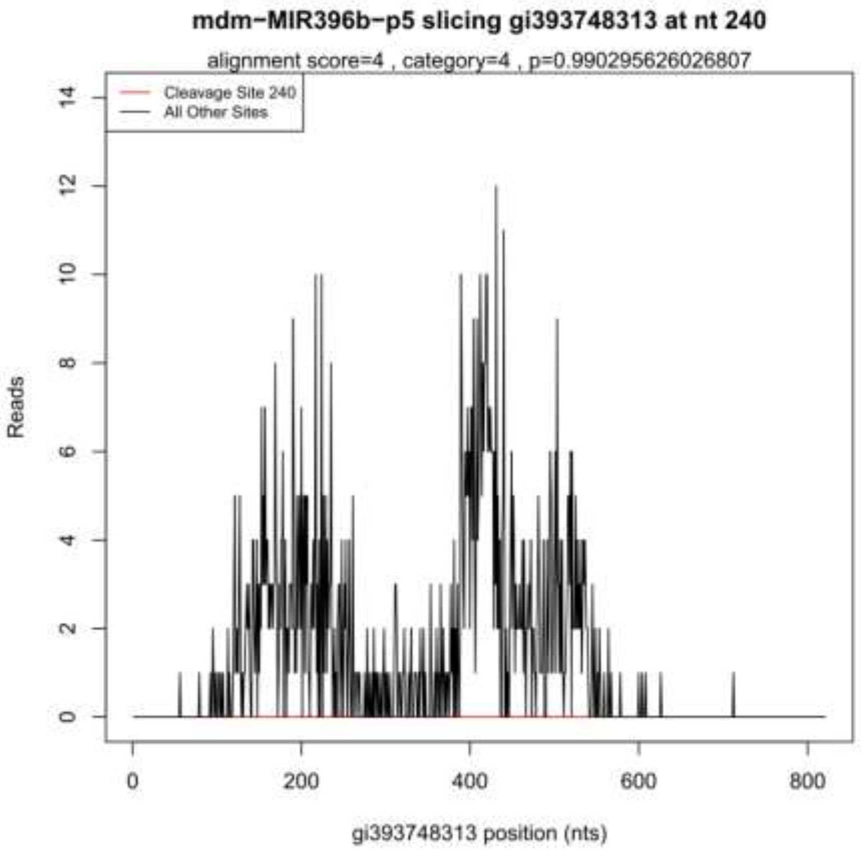
mdm−MIR396b−p5 slicing gi393748313 at nt 240
alignment score=4 , category=4 , p=0.990295626026807
Cleavage Site 240
All Other Sites
Reads
0
2
4
6
8
10
12
14
0
200
400
600
800
gi393748313 position (nts)

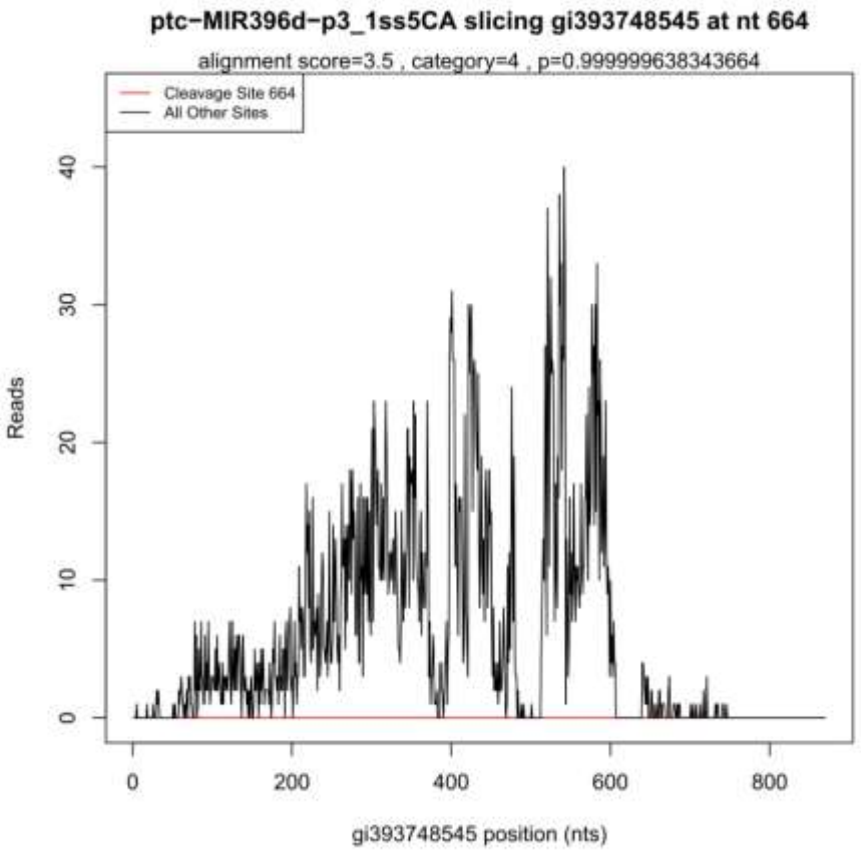
ptc−MIR396d−p3_1ss5CA slicing gi393748545 at nt 664
alignment score=3.5 , category=4 , p=0.999999638343664
Cleavage Site 664
All Other Sites
Reads
0
10
20
30
40
0
200
400
600
800
gi393748545 position (nts)

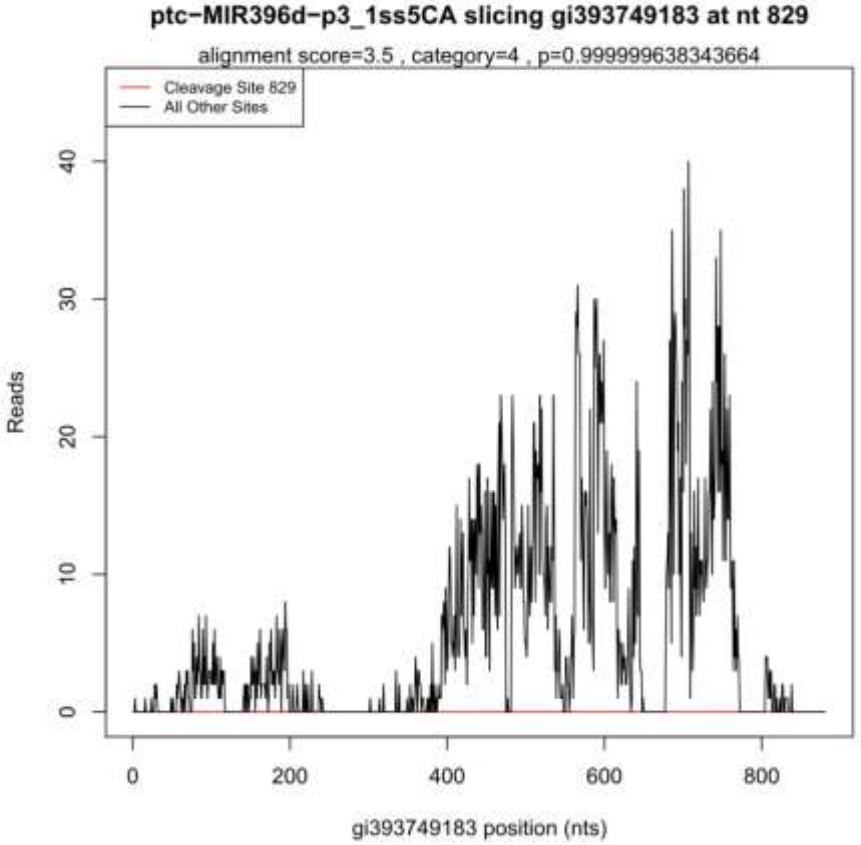

ptc−MIR396d−p3_1ss5CA slicing gi393749183 at nt 829
alignment score=3.5 , category=4 , p=0.999999638343664
Cleavage Site 829
All Other Sites
Reads
0
10
20
30
40
0
200
400
600
800
gi393749183 position (nts)

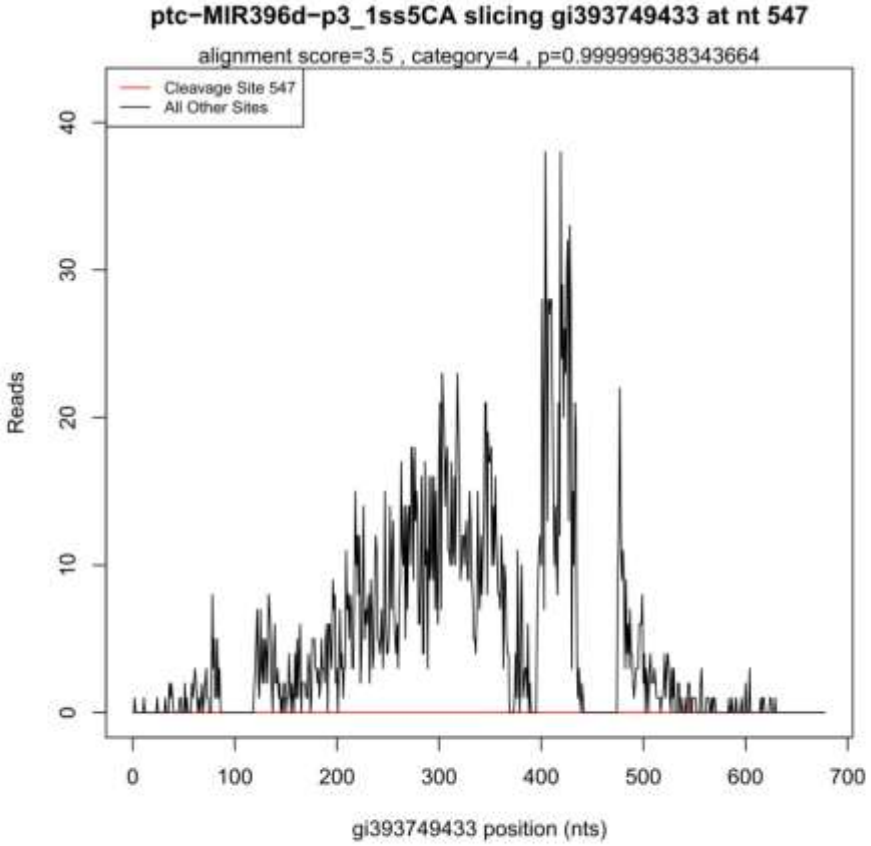

ptc−MIR396d−p3_1ss5CA slicing gi393749433 at nt 547
alignment score=3.5 , category=4 , p=0.999999638343664
Cleavage Site 547
All Other Sites
Reads
0
10
20
30
40
0
100
200
300
400
500
600
700
gi393749433 position (nts)

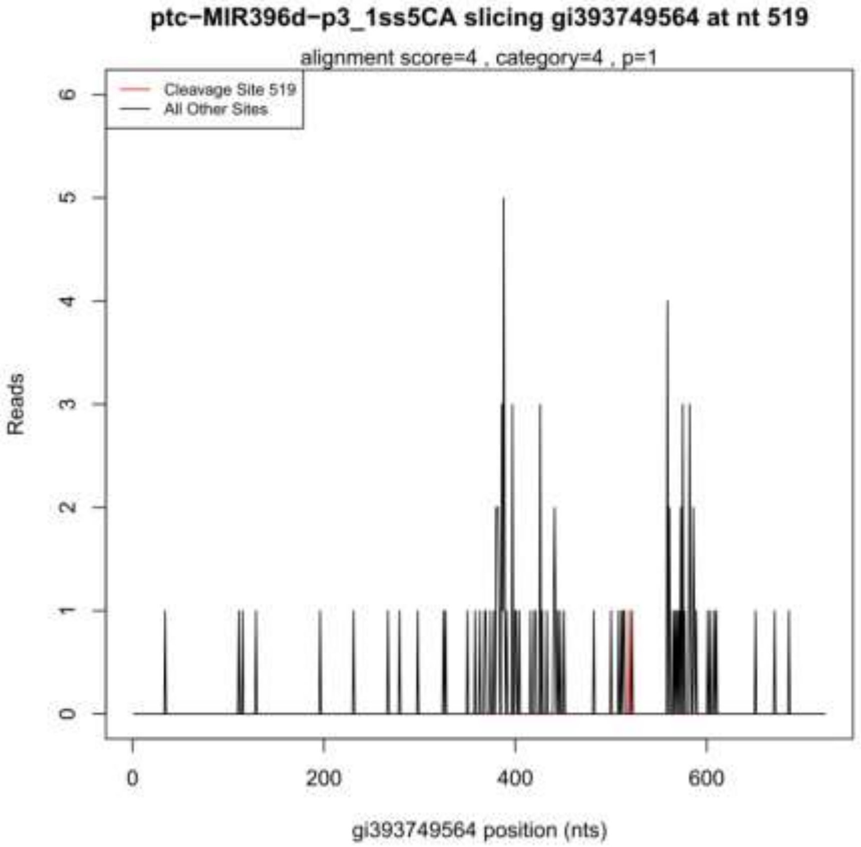
ptc−MIR396d−p3_1ss5CA slicing gi393749564 at nt 519
alignment score=4 , category=4 , p=1
Cleavage Site 519
All Other Sites
Reads
0
1
2
3
4
5
6
0
200
400
600
gi393749564 position (nts)

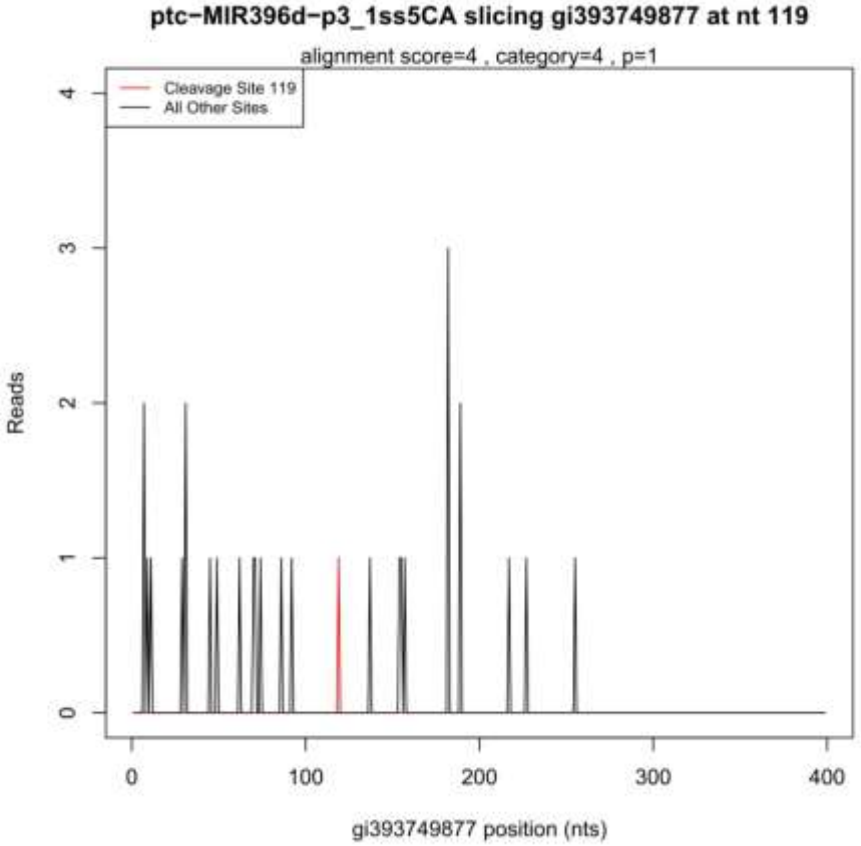
ptc−MIR396d−p3_1ss5CA slicing gi393749877 at nt 119
alignment score=4 , category=4 , p=1
Cleavage Site 119
All Other Sites
Reads
0
1
2
3
4
0
100
200
300
400
gi393749877 position (nts)

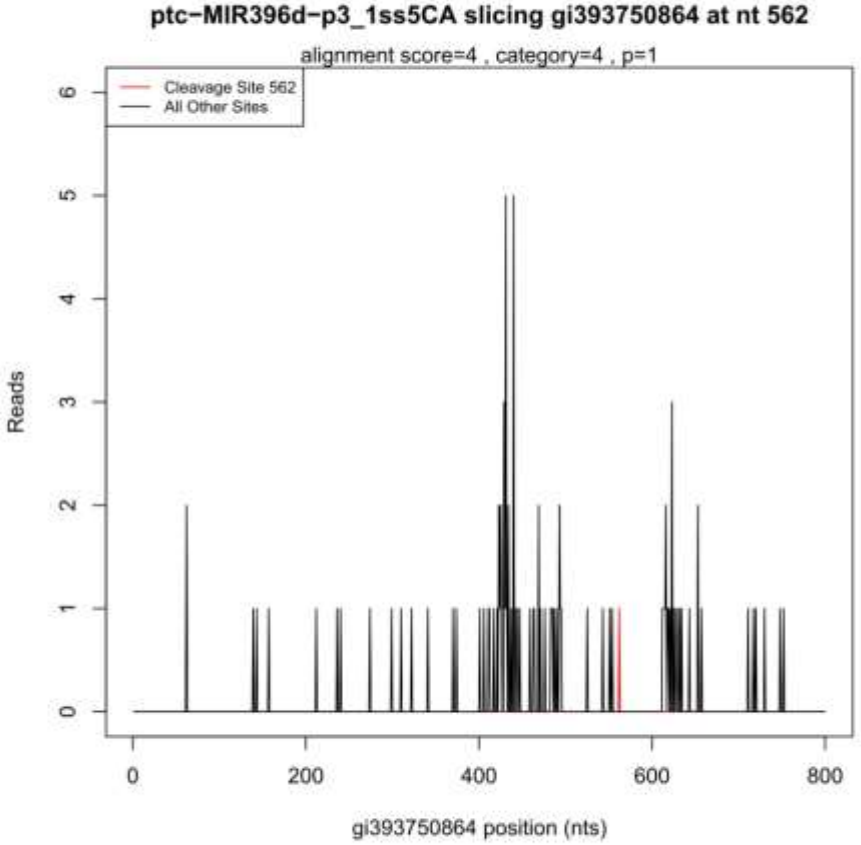
ptc−MIR396d−p3_1ss5CA slicing gi393750864 at nt 562
alignment score=4 , category=4 , p=1
Cleavage Site 562
All Other Sites
Reads
gi393750864 position (nts)
0
1
2
3
4
5
6
0
200
400
600
800

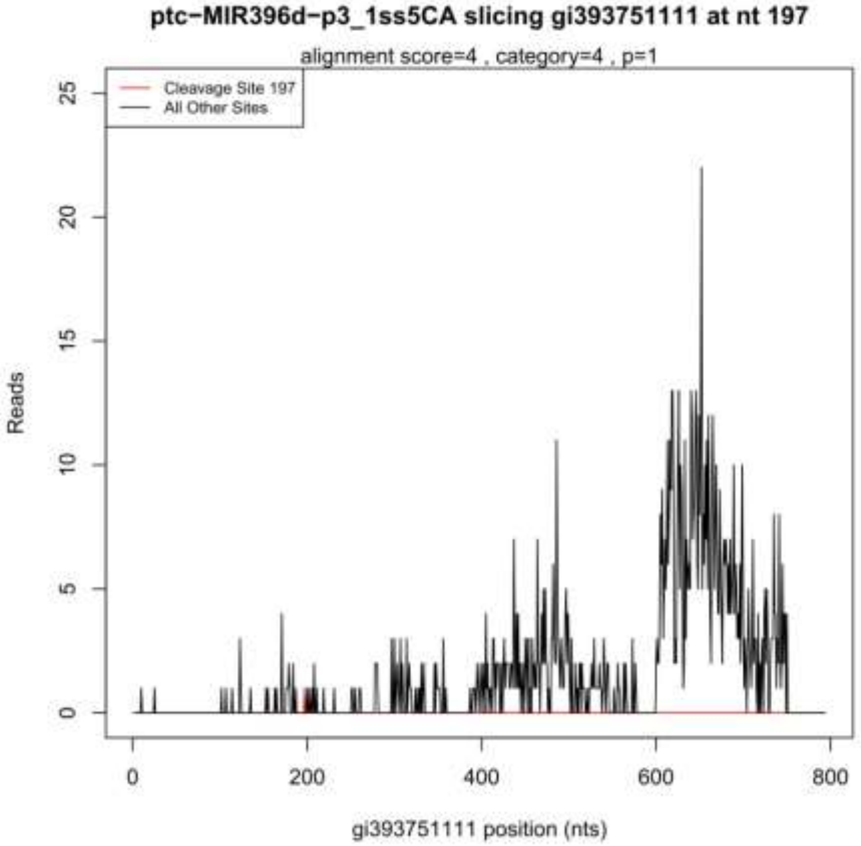
ptc−MIR396d−p3_1ss5CA slicing gi393751111 at nt 197
alignment score=4 , category=4 , p=1
Cleavage Site 197
All Other Sites
Reads
gi393751111 position (nts)
0
5
10
15
20
25
0
200
400
600
800

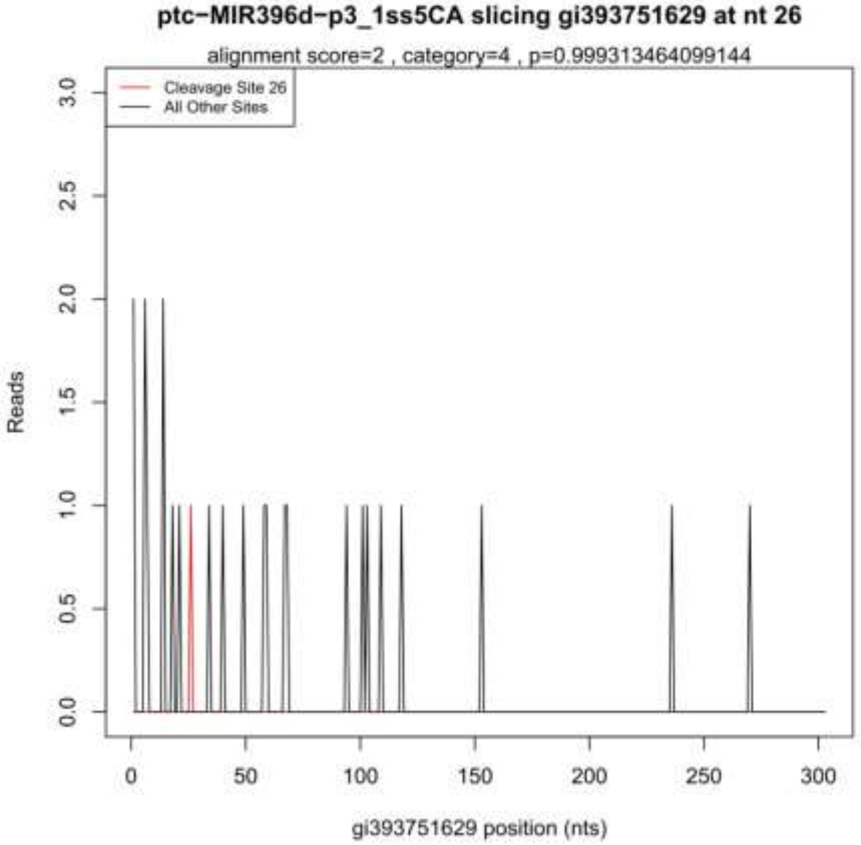
ptc−MIR396d−p3_1ss5CA slicing gi393751629 at nt 26
alignment score=2 , category=4 , p=0.999313464099144
Cleavage Site 26
All Other Sites
Reads
0.0
0.5
1.0
1.5
2.0
2.5
3.0
0
50
100
150
200
250
300
gi393751629 position (nts)

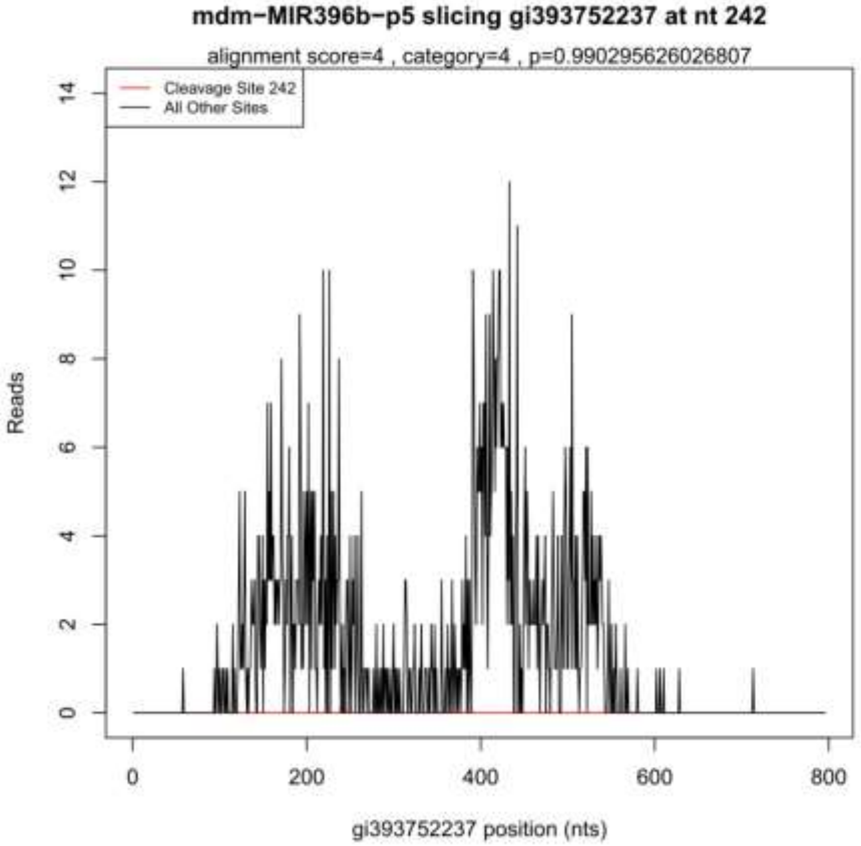
mdm−MIR396b−p5 slicing gi393752237 at nt 242
alignment score=4 , category=4 , p=0.990295626026807
Cleavage Site 242
All Other Sites
Reads
0
2
4
6
8
10
12
14
0
200
400
600
800
gi393752237 position (nts)

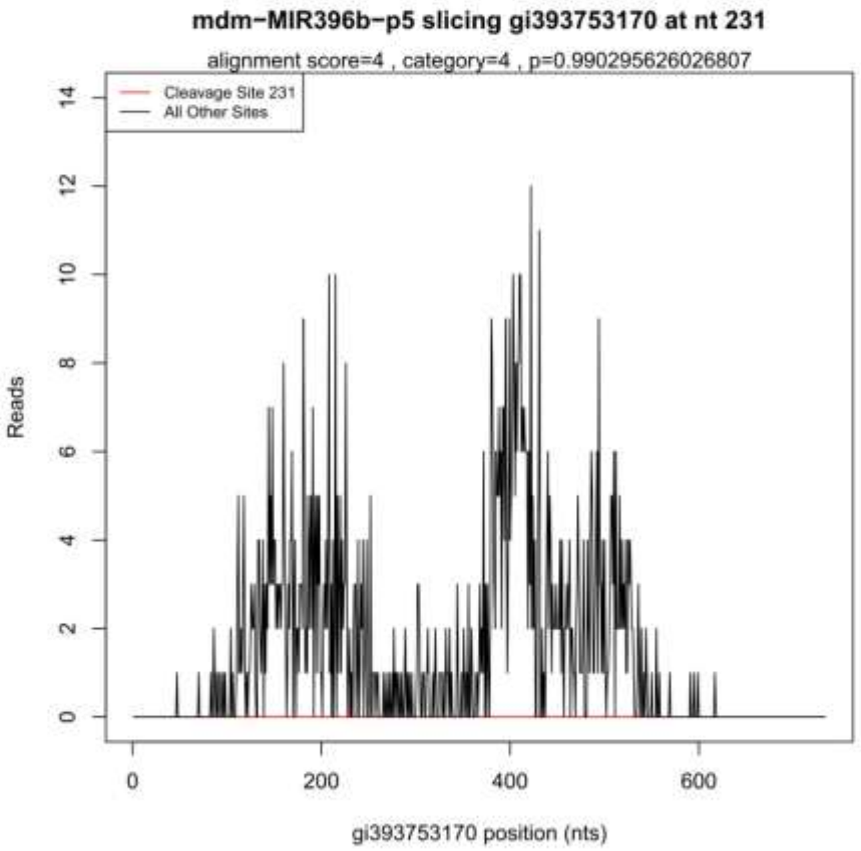
mdm−MIR396b−p5 slicing gi393753170 at nt 231
alignment score=4 , category=4 , p=0.990295626026807
Cleavage Site 231
All Other Sites
Reads
gi393753170 position (nts)

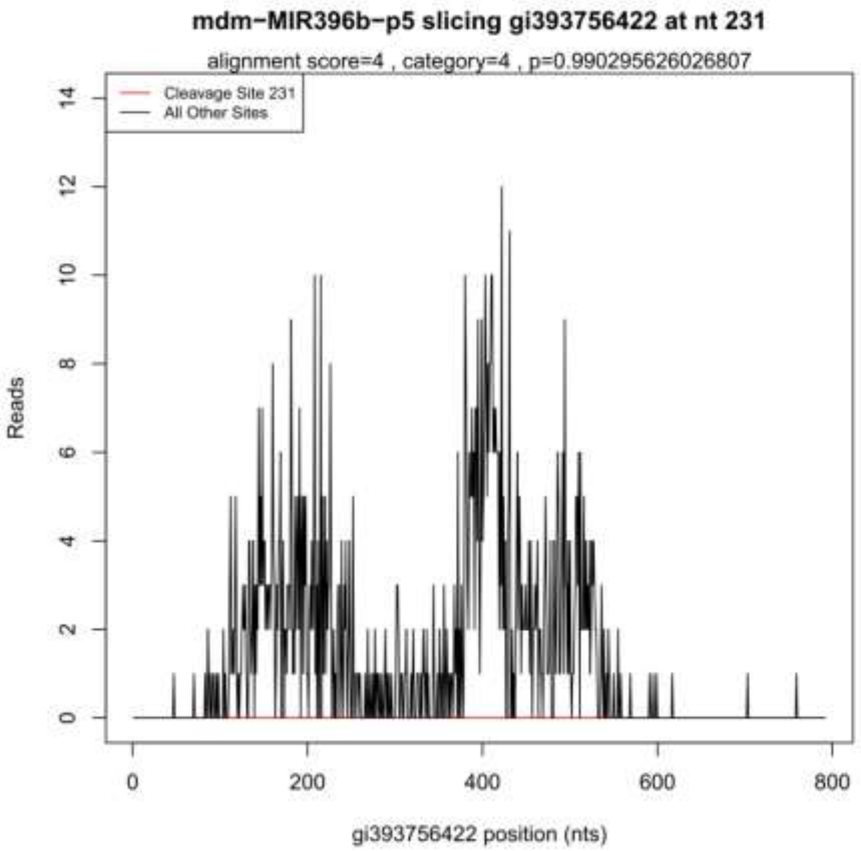
mdm−MIR396b−p5 slicing gi393756422 at nt 231
alignment score=4 , category=4 , p=0.990295626026807
Cleavage Site 231
All Other Sites
Reads
gi393756422 position (nts)

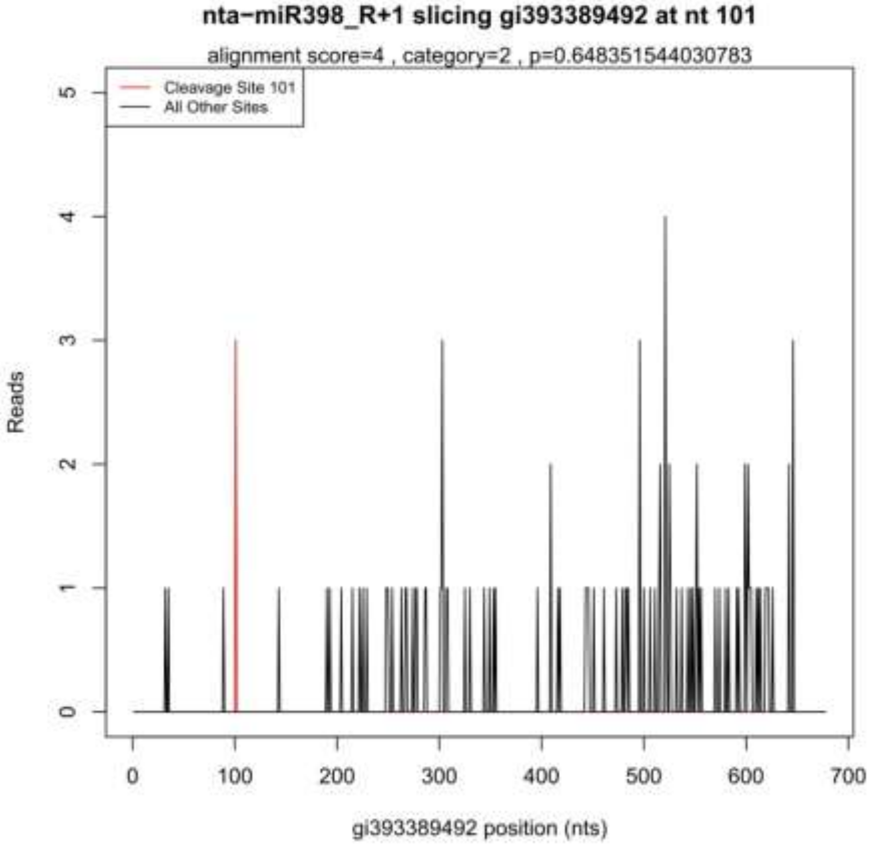
nta−miR398_R+1 slicing gi393389492 at nt 101
alignment score=4 , category=2 , p=0.648351544030783
Cleavage Site 101
All Other Sites
Reads
0
1
2
3
4
5
0
100
200
300
400
500
600
700
gi393389492 position (nts)

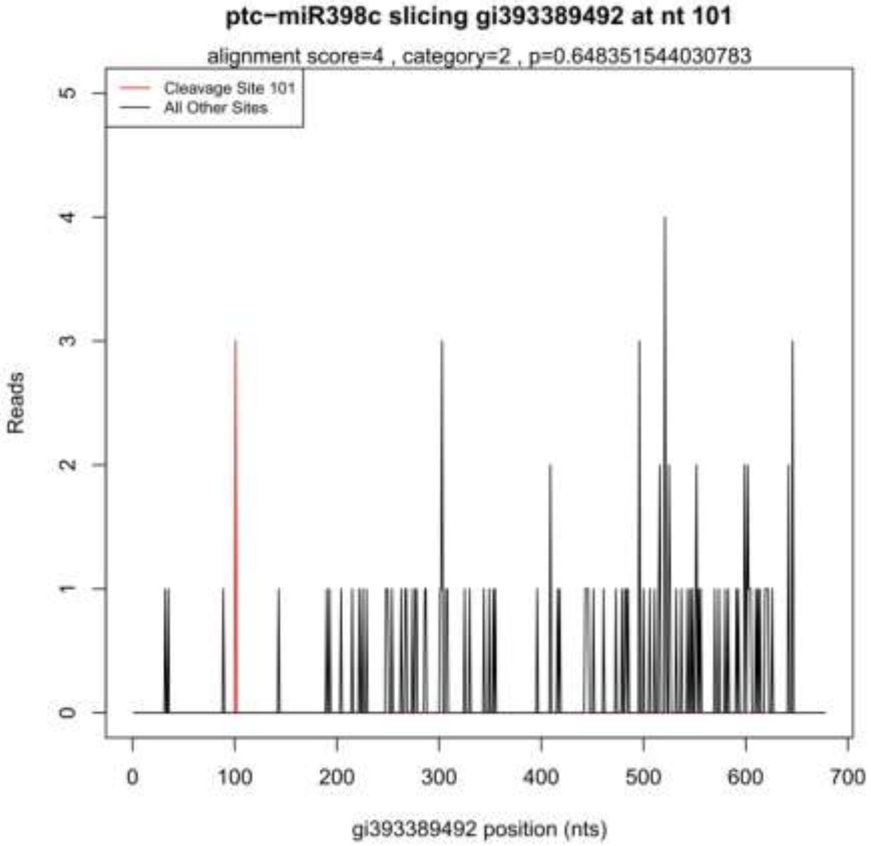
ptc−miR398c slicing gi393389492 at nt 101
alignment score=4 , category=2 , p=0.648351544030783
Cleavage Site 101
All Other Sites
Reads
0
1
2
3
4
5
0
100
200
300
400
500
600
700
gi393389492 position (nts)

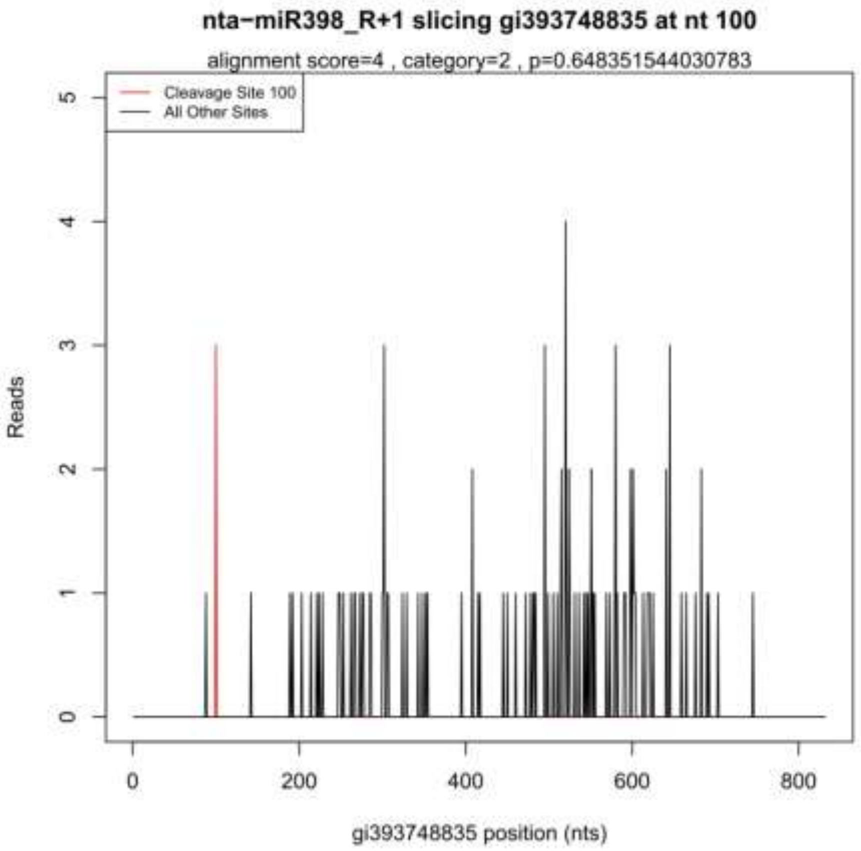

nta−miR398_R+1 slicing gi393748835 at nt 100
alignment score=4 , category=2 , p=0.648351544030783
Cleavage Site 100
All Other Sites
Reads
0
1
2
3
4
5
0
200
400
600
800
gi393748835 position (nts)

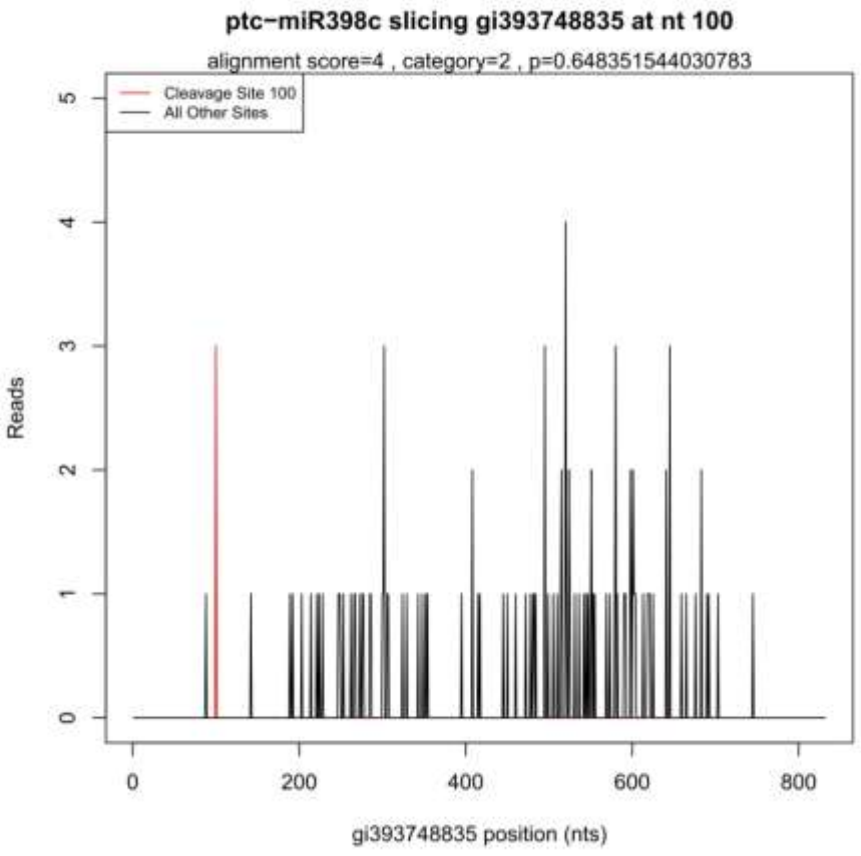

ptc−miR398c slicing gi393748835 at nt 100
alignment score=4 , category=2 , p=0.648351544030783
Cleavage Site 100
All Other Sites
Reads
0
1
2
3
4
5
0
200
400
600
800
gi393748835 position (nts)

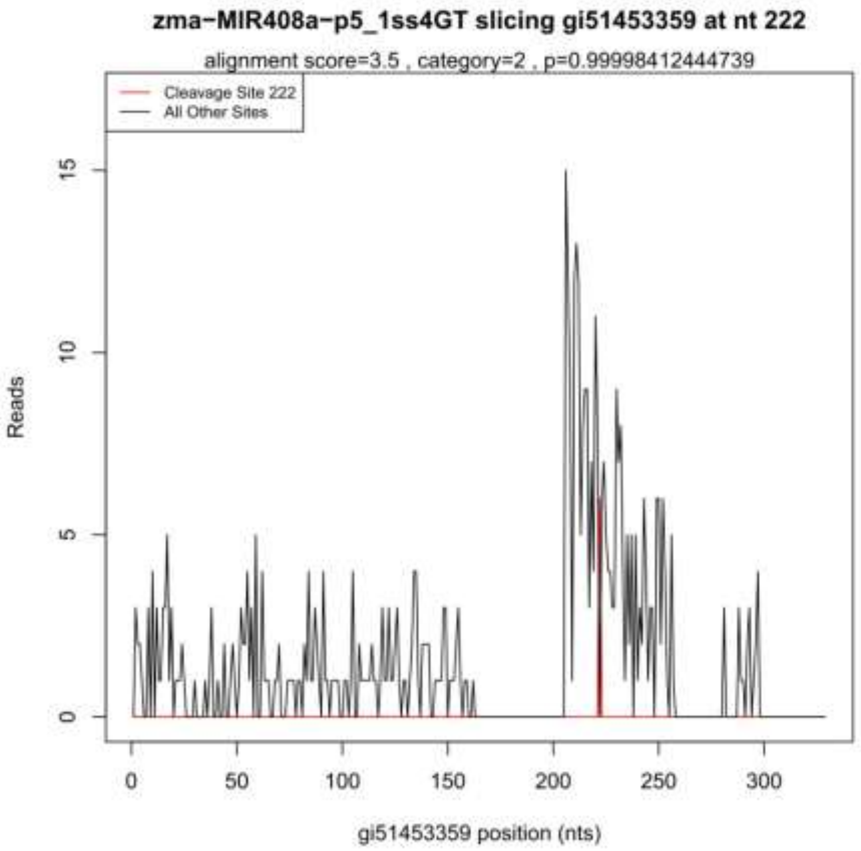
zma−MIR408a−p5_1ss4GT slicing gi51453359 at nt 222
alignment score=3.5 , category=2 , p=0.99998412444739
Cleavage Site 222
All Other Sites
Reads
0
5
10
15
0
50
100
150
200
250
300
gi51453359 position (nts)

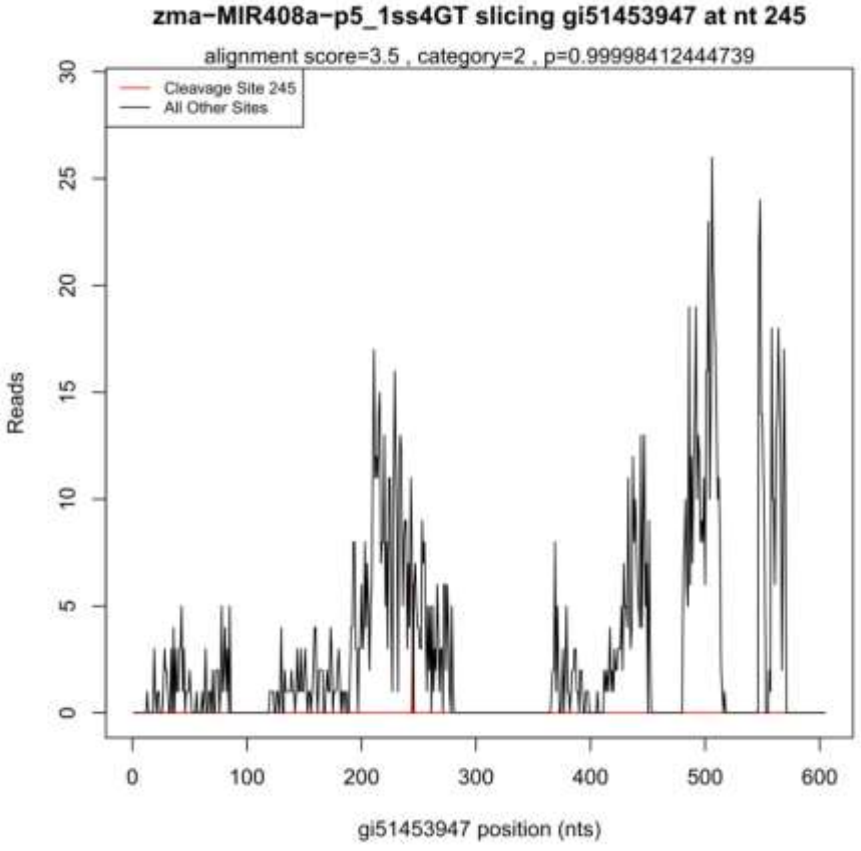
zma−MIR408a−p5_1ss4GT slicing gi51453947 at nt 245
alignment score=3.5 , category=2 , p=0.99998412444739
Cleavage Site 245
All Other Sites
Reads
0
5
10
15
20
25
30
0
100
200
300
400
500
600
gi51453947 position (nts)

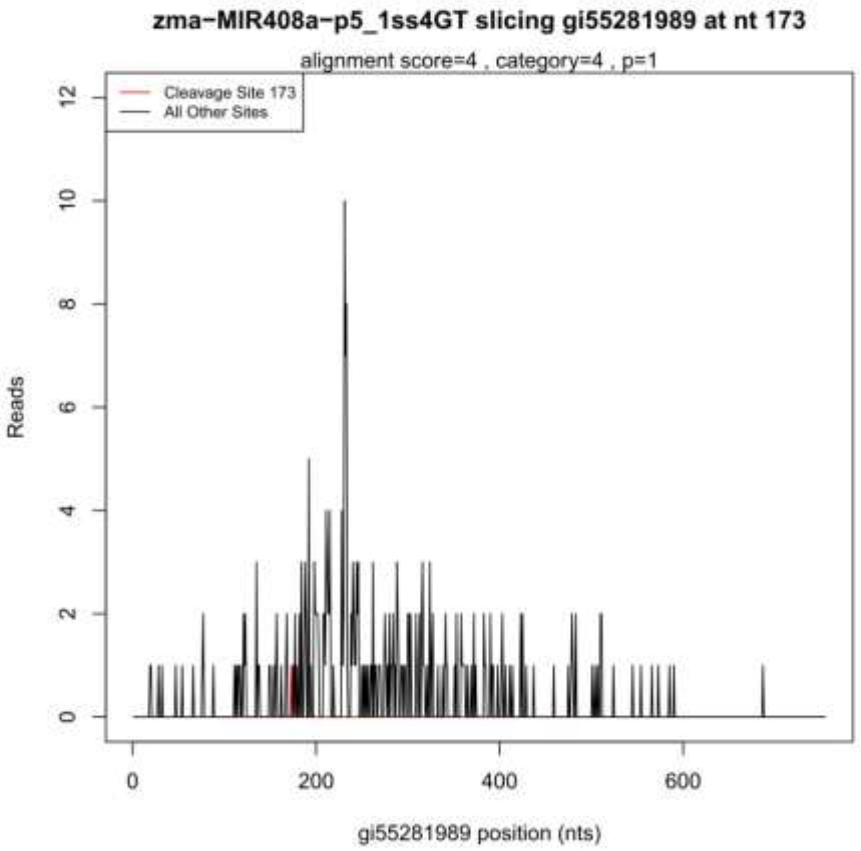
zma−MIR408a−p5_1ss4GT slicing gi55281989 at nt 173
alignment score=4 , category=4 , p=1
Cleavage Site 173
All Other Sites
Reads
0
2
4
6
8
10
12
0
200
400
600
gi55281989 position (nts)

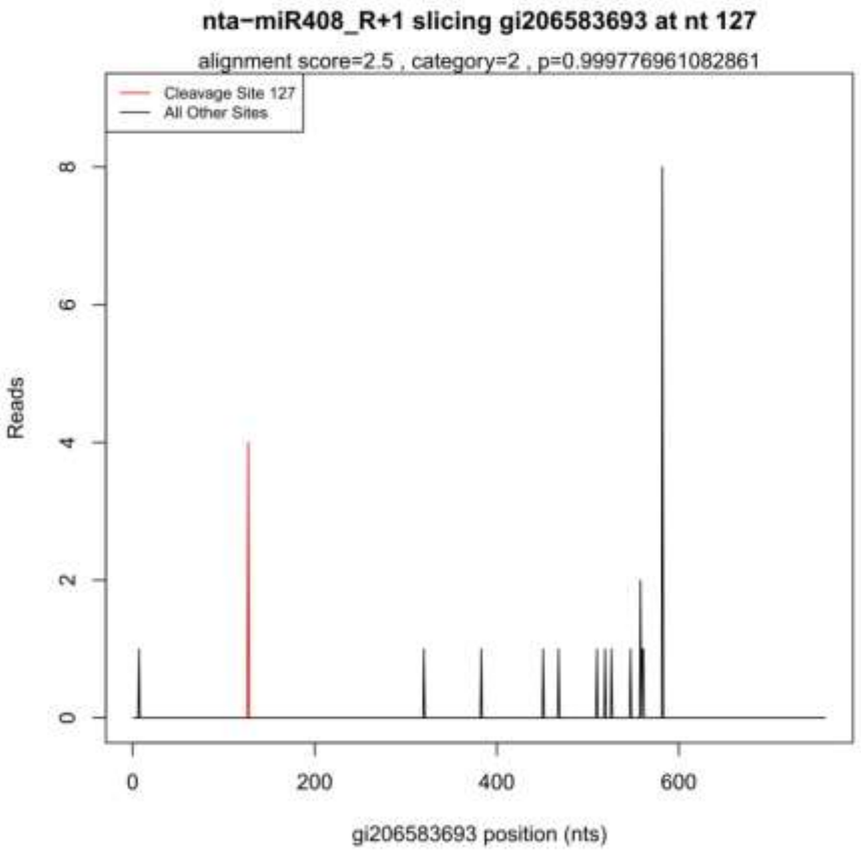
nta−miR408_R+1 slicing gi206583693 at nt 127
alignment score=2.5 , category=2 , p=0.999776961082861
Cleavage Site 127
All Other Sites
Reads
0
2
4
6
8
0
200
400
600
gi206583693 position (nts)

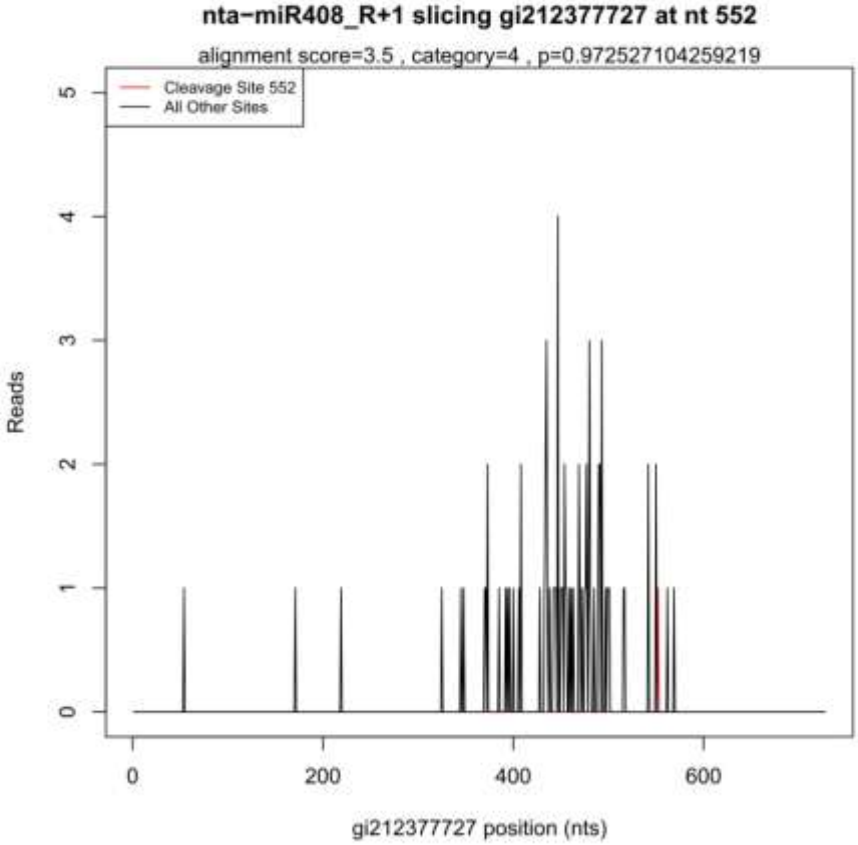
nta−miR408_R+1 slicing gi212377727 at nt 552
alignment score=3.5 , category=4 , p=0.972527104259219
Cleavage Site 552
All Other Sites
Reads
gi212377727 position (nts)
0
200
400
600
1
2
3
4
5

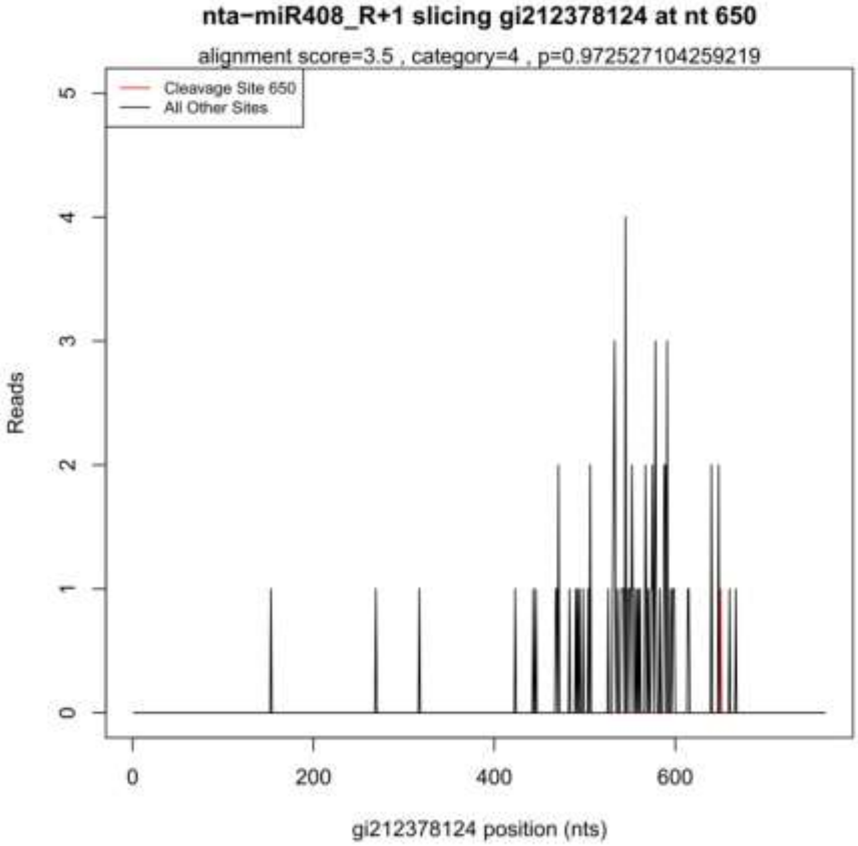
nta−miR408_R+1 slicing gi212378124 at nt 650
alignment score=3.5 , category=4 , p=0.972527104259219
Cleavage Site 650
All Other Sites
Reads
gi212378124 position (nts)
0
200
400
600
1
2
3
4
5

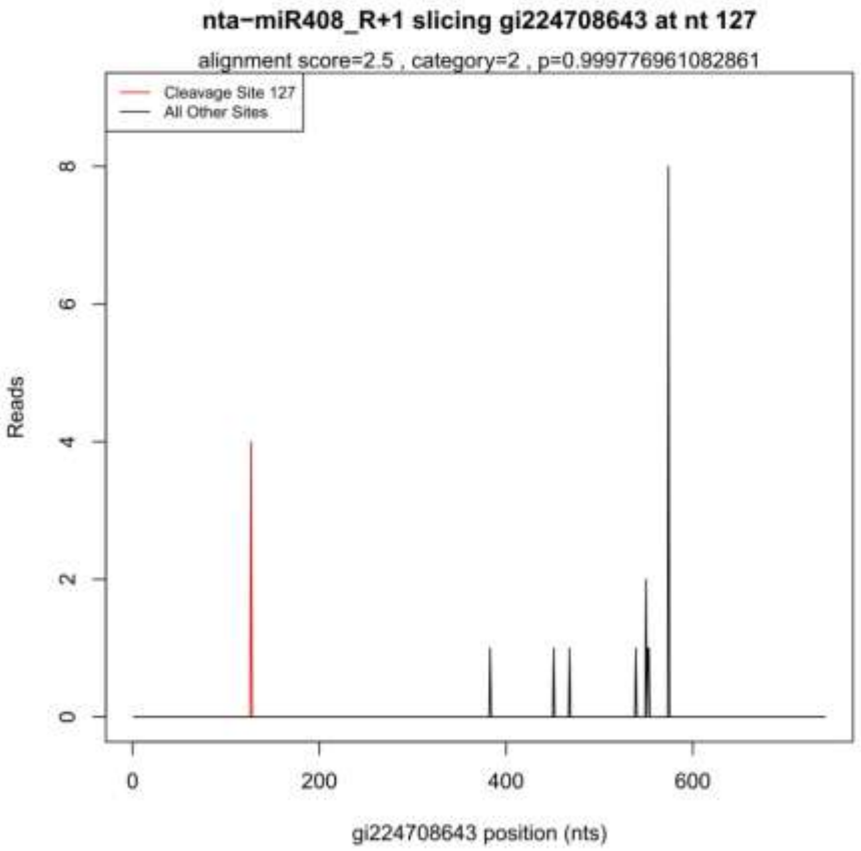
nta−miR408_R+1 slicing gi224708643 at nt 127
alignment score=2.5 , category=2 , p=0.999776961082861
Cleavage Site 127
All Other Sites
Reads
0
2
4
6
8
0
200
400
600
gi224708643 position (nts)

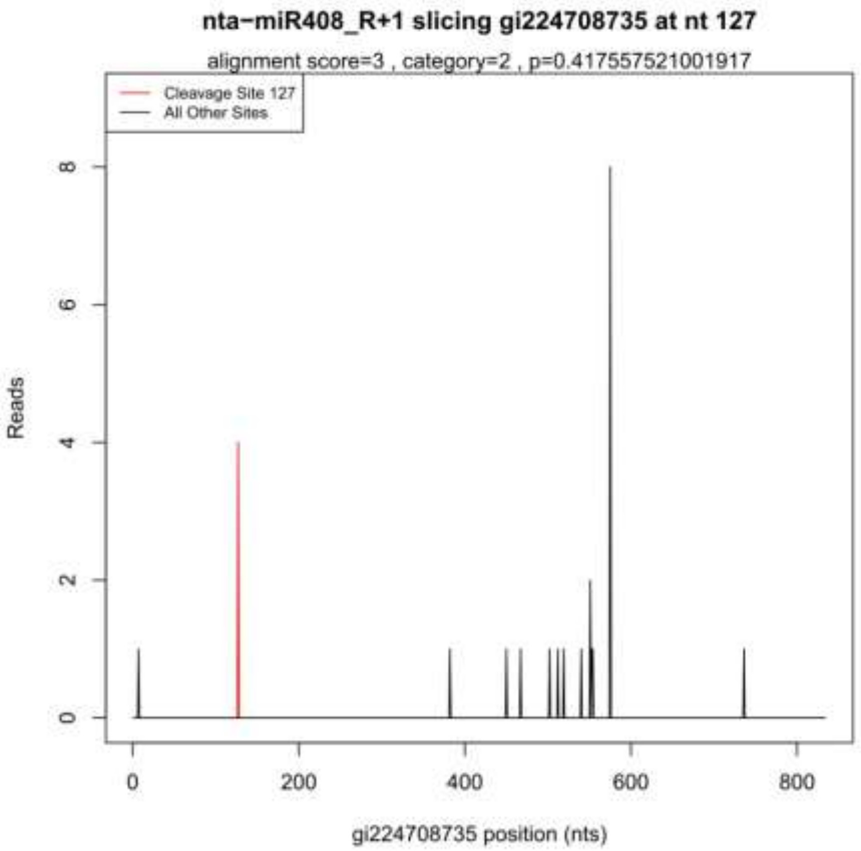
nta−miR408_R+1 slicing gi224708735 at nt 127
alignment score=3 , category=2 , p=0.417557521001917
Cleavage Site 127
All Other Sites
Reads
0
2
4
6
8
0
200
400
600
800
gi224708735 position (nts)

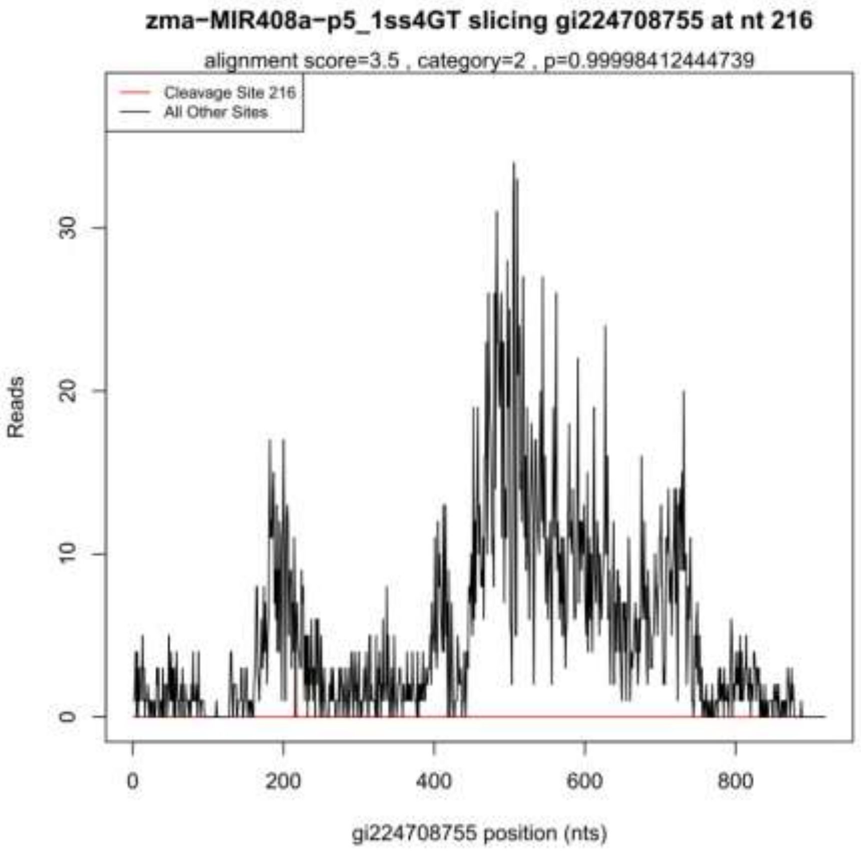
zma−MIR408a−p5_1ss4GT slicing gi224708755 at nt 216
alignment score=3.5 , category=2 , p=0.99998412444739
Cleavage Site 216
All Other Sites
Reads
0
10
20
30
0
200
400
600
800
gi224708755 position (nts)

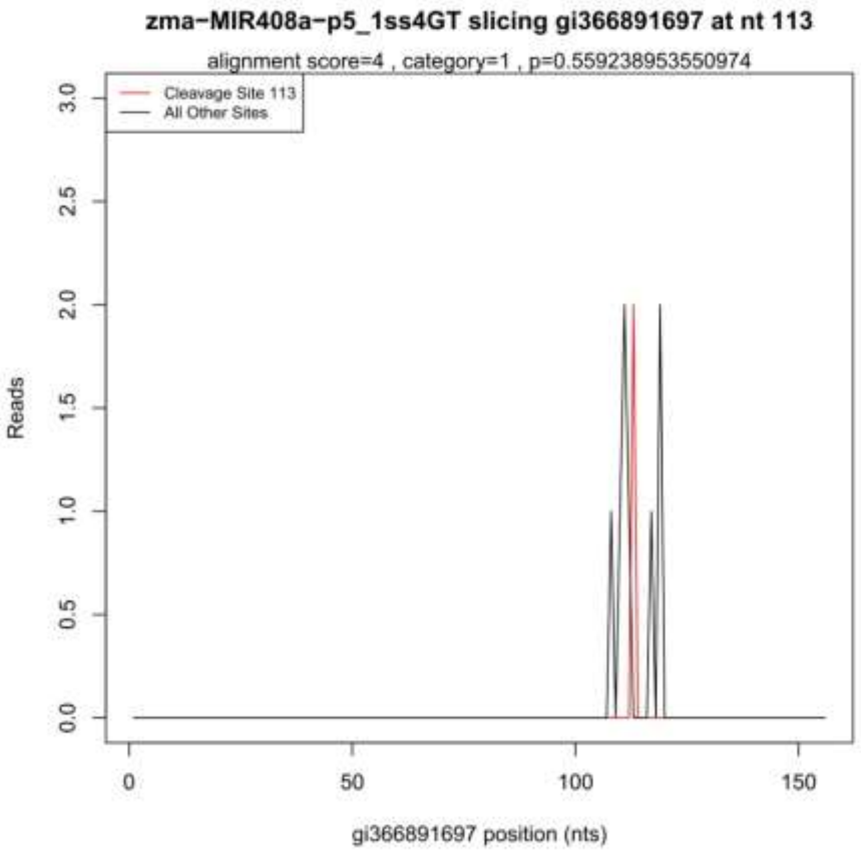
zma−MIR408a−p5_1ss4GT slicing gi366891697 at nt 113
alignment score=4 , category=1 , p=0.559238953550974
Cleavage Site 113
All Other Sites
Reads
0.0
0.5
1.0
1.5
2.0
2.5
3.0
0
50
100
150
gi366891697 position (nts)

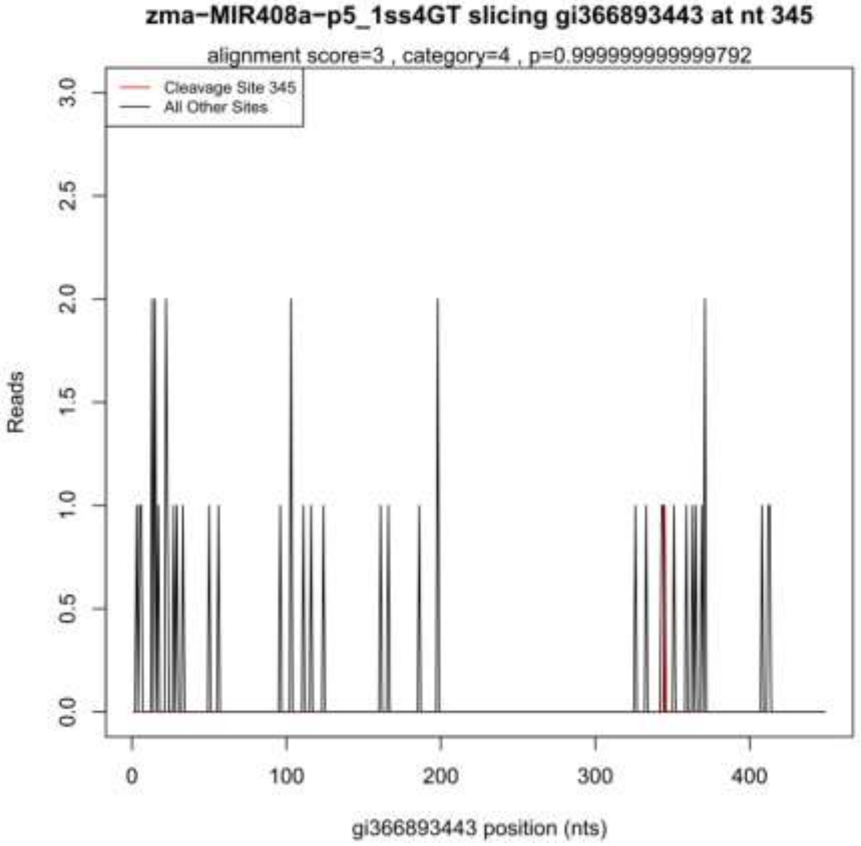

zma−MIR408a−p5_1ss4GT slicing gi366893443 at nt 345
alignment score=3 , category=4 , p=0.999999999999792
Cleavage Site 345
All Other Sites
Reads
gi366893443 position (nts)

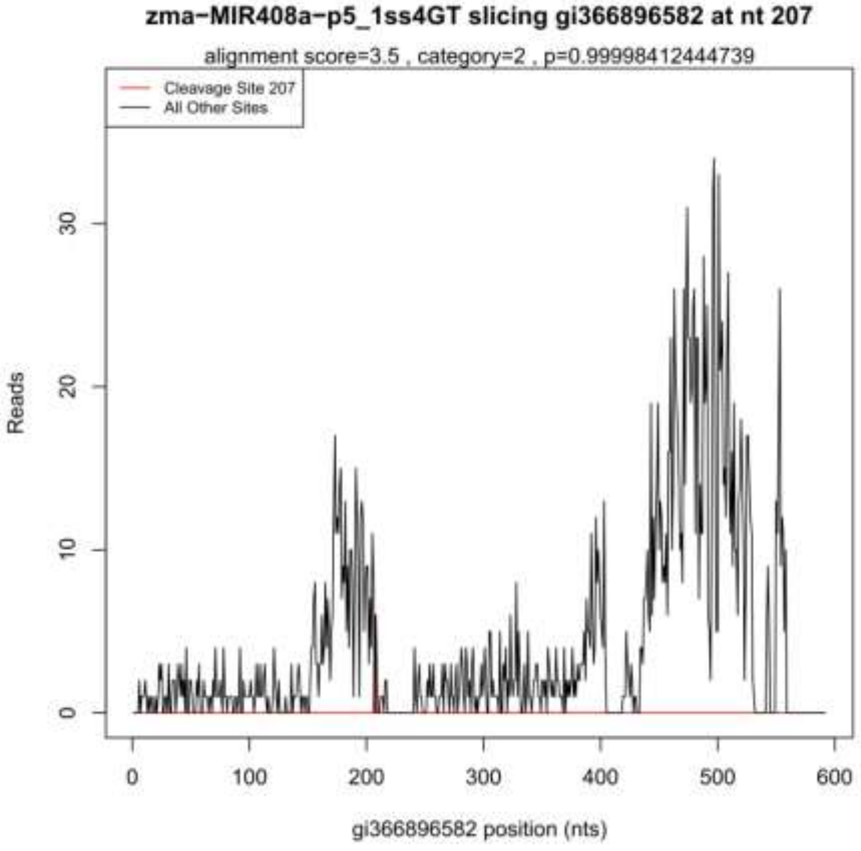

zma−MIR408a−p5_1ss4GT slicing gi366896582 at nt 207
alignment score=3.5 , category=2 , p=0.99998412444739
Cleavage Site 207
All Other Sites
Reads
gi366896582 position (nts)

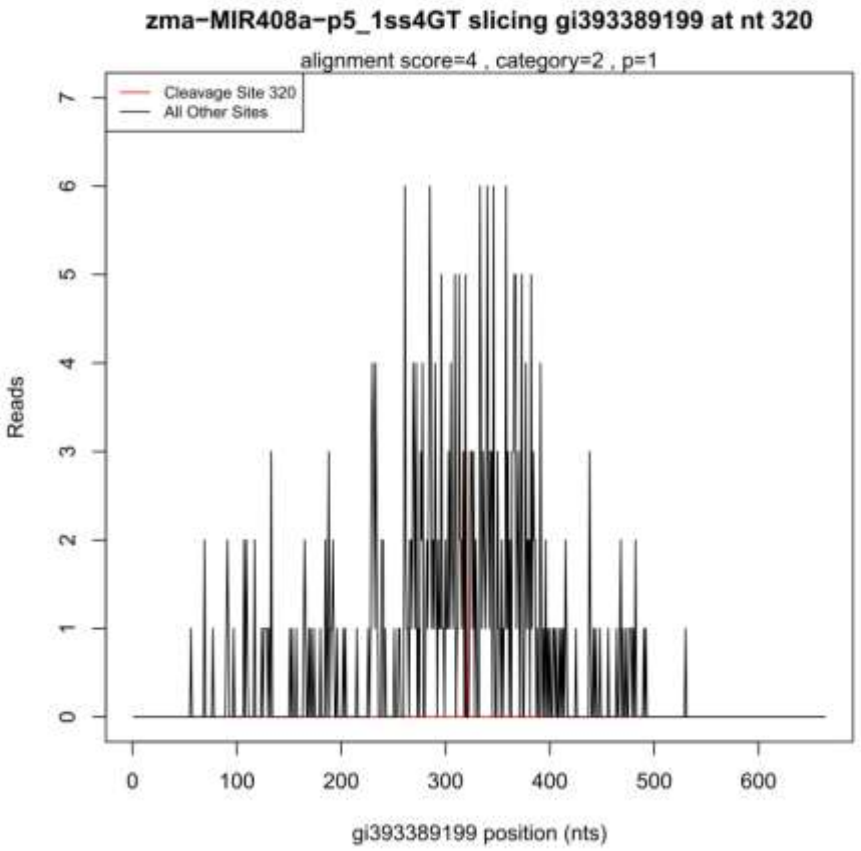
zma−MIR408a−p5_1ss4GT slicing gi393389199 at nt 320
alignment score=4 , category=2 , p=1
Cleavage Site 320
All Other Sites
Reads
gi393389199 position (nts)

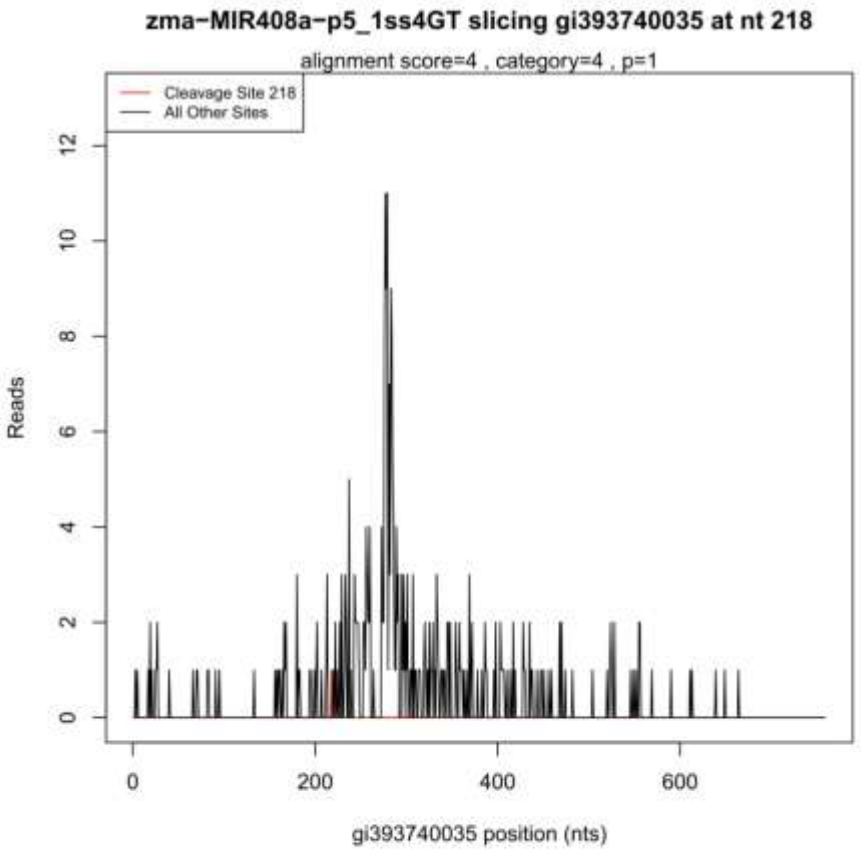
zma−MIR408a−p5_1ss4GT slicing gi393740035 at nt 218
alignment score=4 , category=4 , p=1
Cleavage Site 218
All Other Sites
Reads
gi393740035 position (nts)

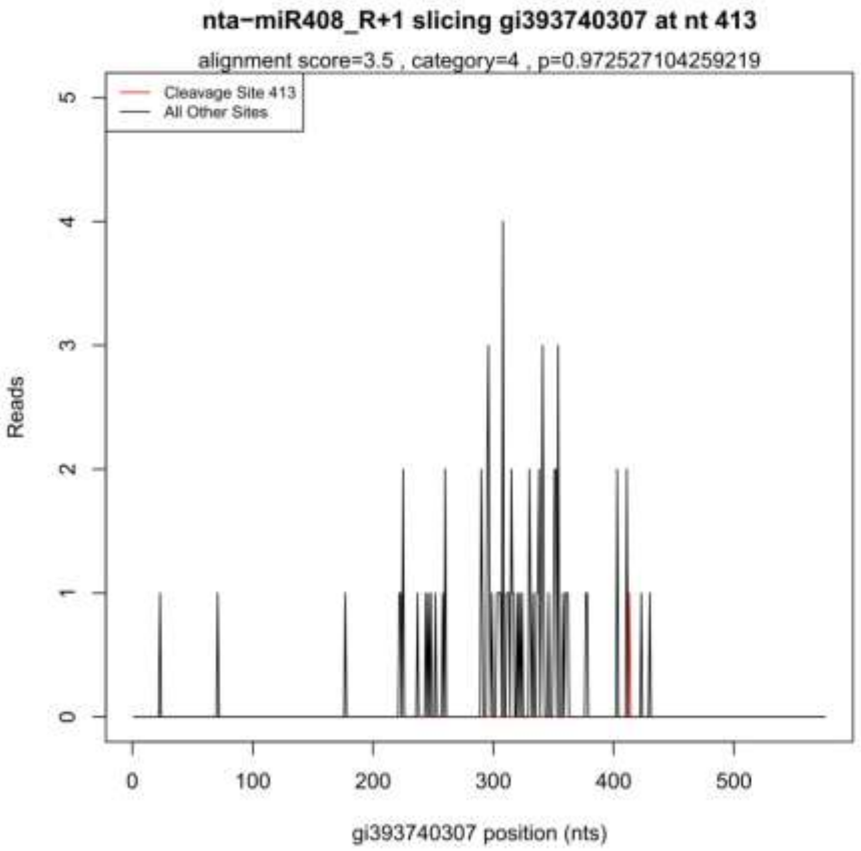
nta−miR408_R+1 slicing gi393740307 at nt 413
alignment score=3.5 , category=4 , p=0.972527104259219
Cleavage Site 413
All Other Sites
Reads
0
1
2
3
4
5
0
100
200
300
400
500
gi393740307 position (nts)

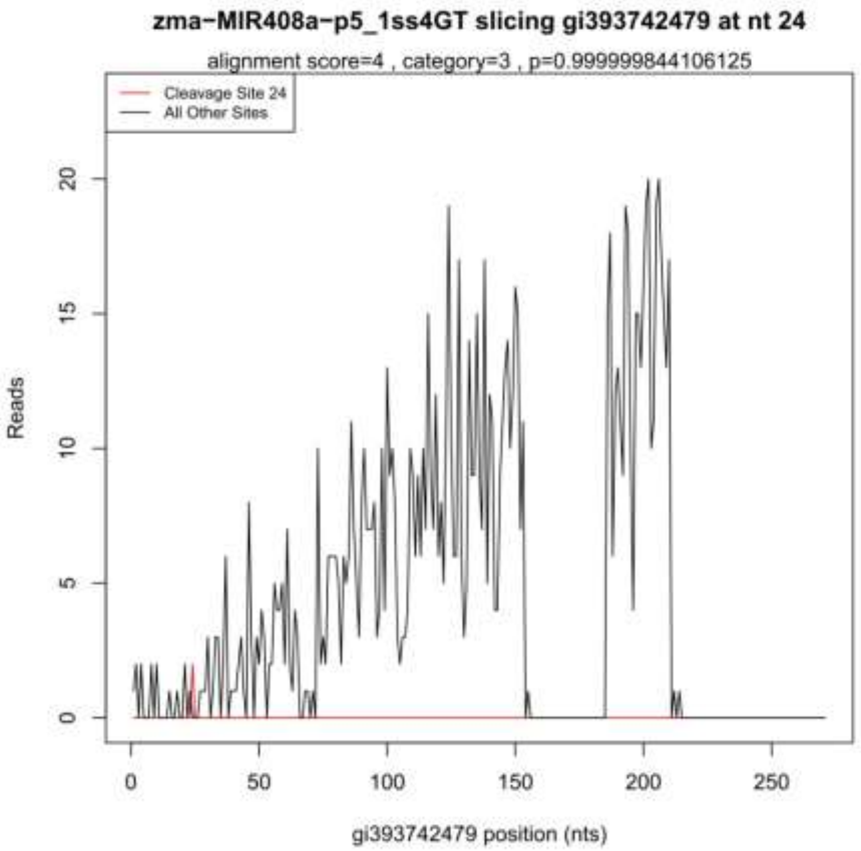
zma−MIR408a−p5_1ss4GT slicing gi393742479 at nt 24
alignment score=4 , category=3 , p=0.999999844106125
Cleavage Site 24
All Other Sites
Reads
0
5
10
15
20
0
50
100
150
200
250
gi393742479 position (nts)

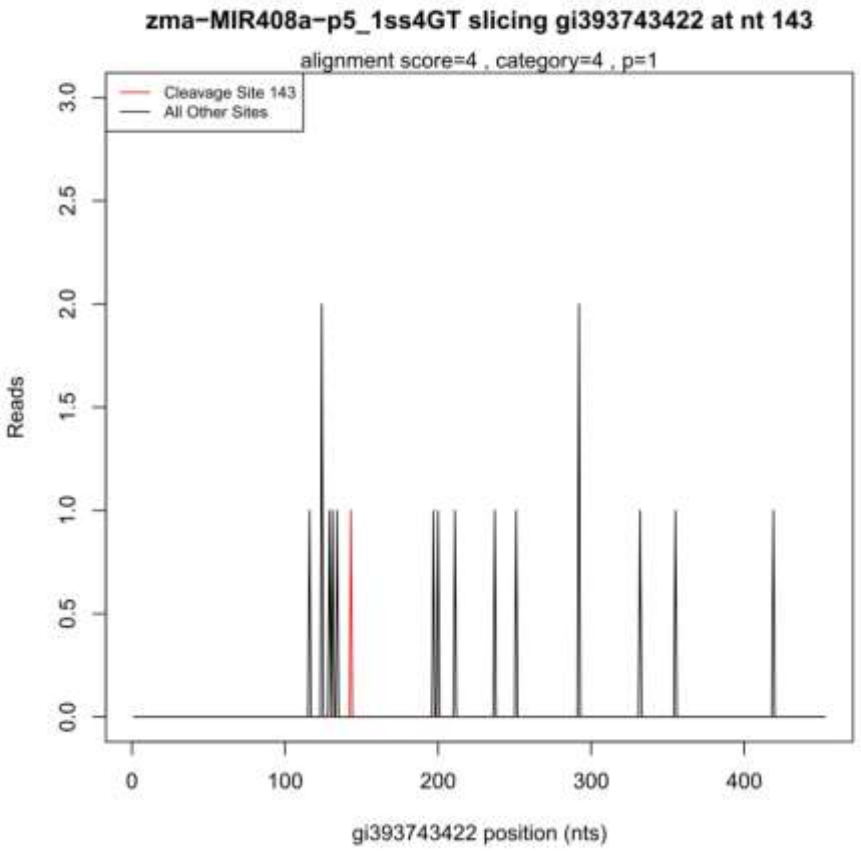
zma−MIR408a−p5_1ss4GT slicing gi393743422 at nt 143
alignment score=4 , category=4 , p=1
Cleavage Site 143
All Other Sites
Reads
0.0
0.5
1.0
1.5
2.0
2.5
3.0
0
100
200
300
400
gi393743422 position (nts)

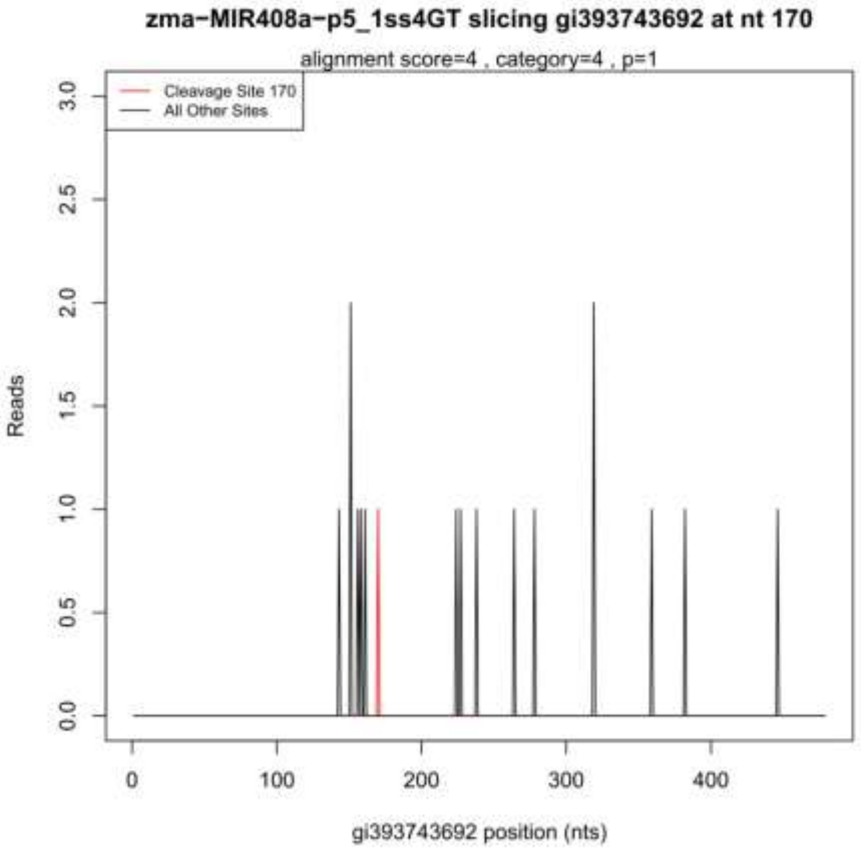
zma−MIR408a−p5_1ss4GT slicing gi393743692 at nt 170
alignment score=4 , category=4 , p=1
Cleavage Site 170
All Other Sites
Reads
0.0
0.5
1.0
1.5
2.0
2.5
3.0
0
100
200
300
400
gi393743692 position (nts)

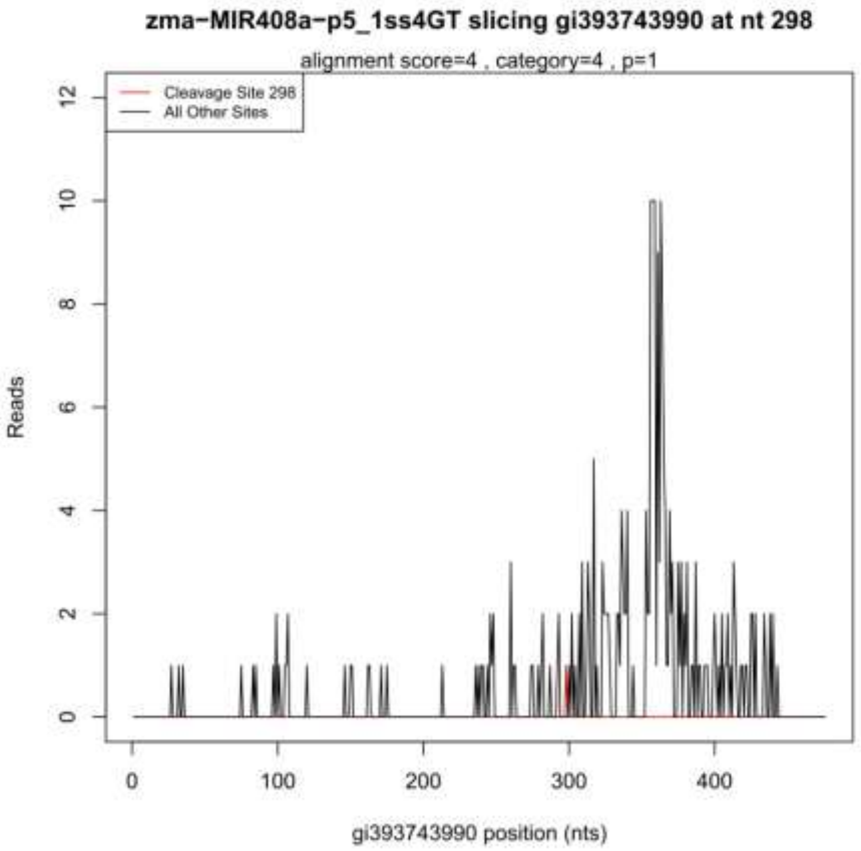
zma−MIR408a−p5_1ss4GT slicing gi393743990 at nt 298
alignment score=4 , category=4 , p=1
Cleavage Site 298
All Other Sites
Reads
gi393743990 position (nts)

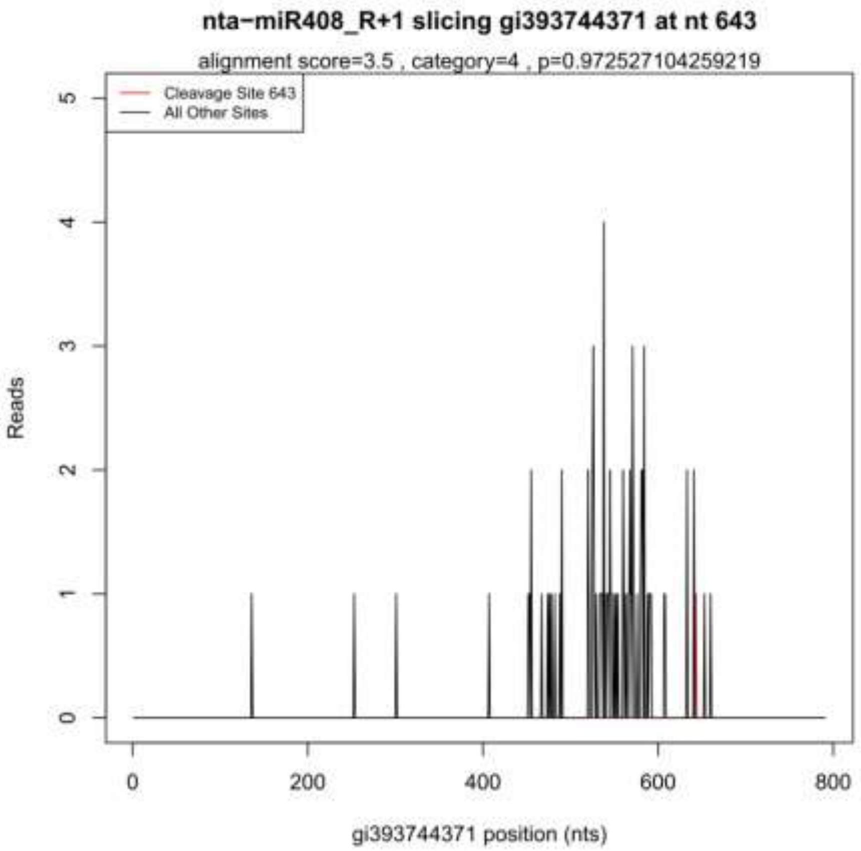
nta−miR408_R+1 slicing gi393744371 at nt 643
alignment score=3.5 , category=4 , p=0.972527104259219
Cleavage Site 643
All Other Sites
Reads
gi393744371 position (nts)

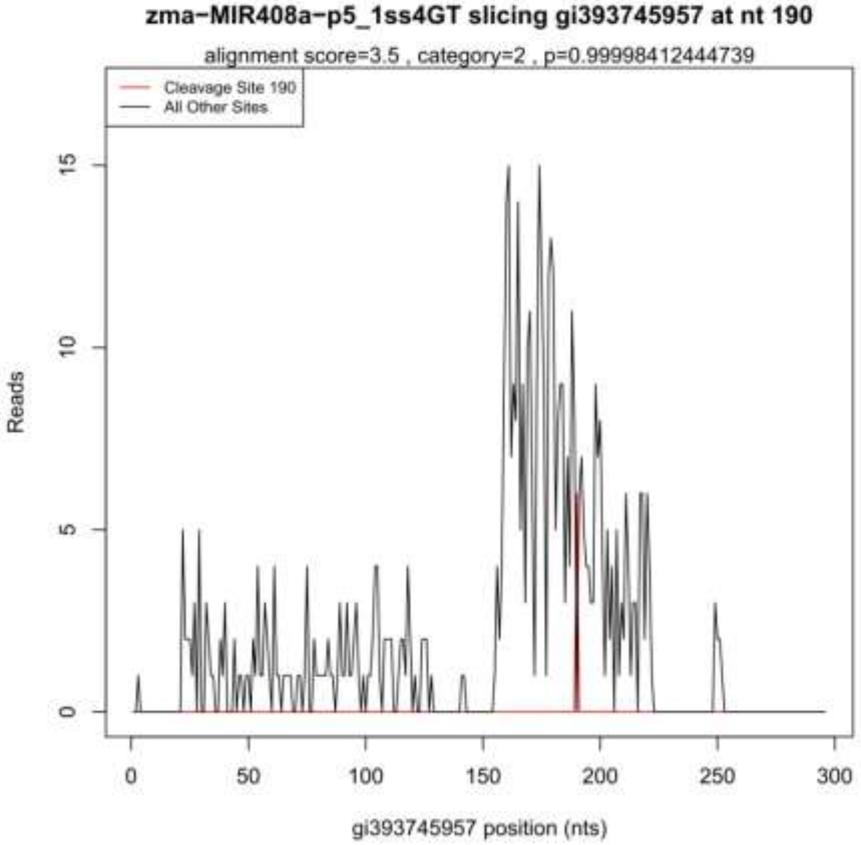
zma−MIR408a−p5_1ss4GT slicing gi393745957 at nt 190
alignment score=3.5 , category=2 , p=0.99998412444739
Cleavage Site 190
All Other Sites
Reads
0
5
10
15
0
50
100
150
200
250
300
gi393745957 position (nts)

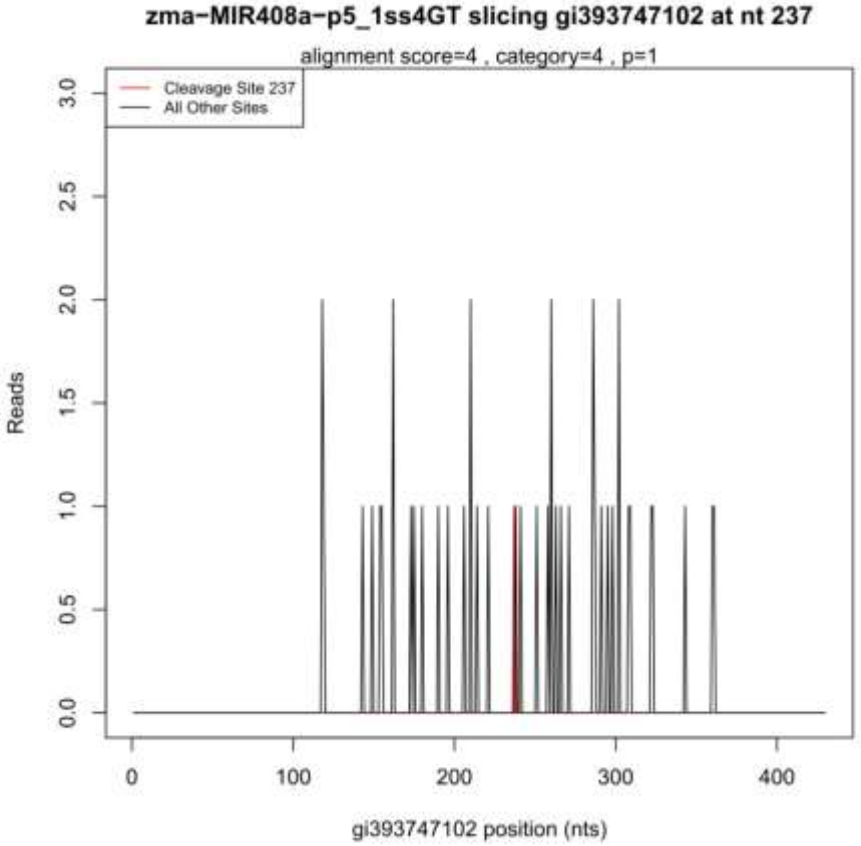
zma−MIR408a−p5_1ss4GT slicing gi393747102 at nt 237
alignment score=4 , category=4 , p=1
Cleavage Site 237
All Other Sites
Reads
0.0
0.5
1.0
1.5
2.0
2.5
3.0
0
100
200
300
400
gi393747102 position (nts)

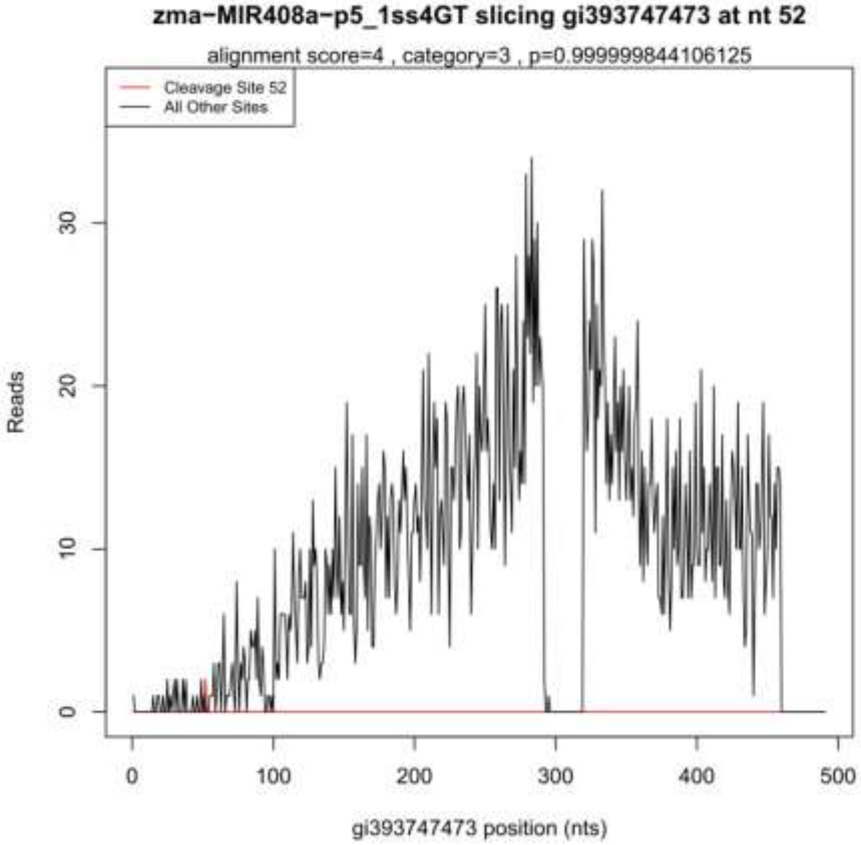

zma-MIR408a-p5_1ss4GT slicing gi393747473 at nt 52
alignment score=4 , category=3 , p=0.999999844106125
Cleavage Site 52
All Other Sites
Reads
0
10
20
30
0
100
200
300
400
500
gi393747473 position (nts)

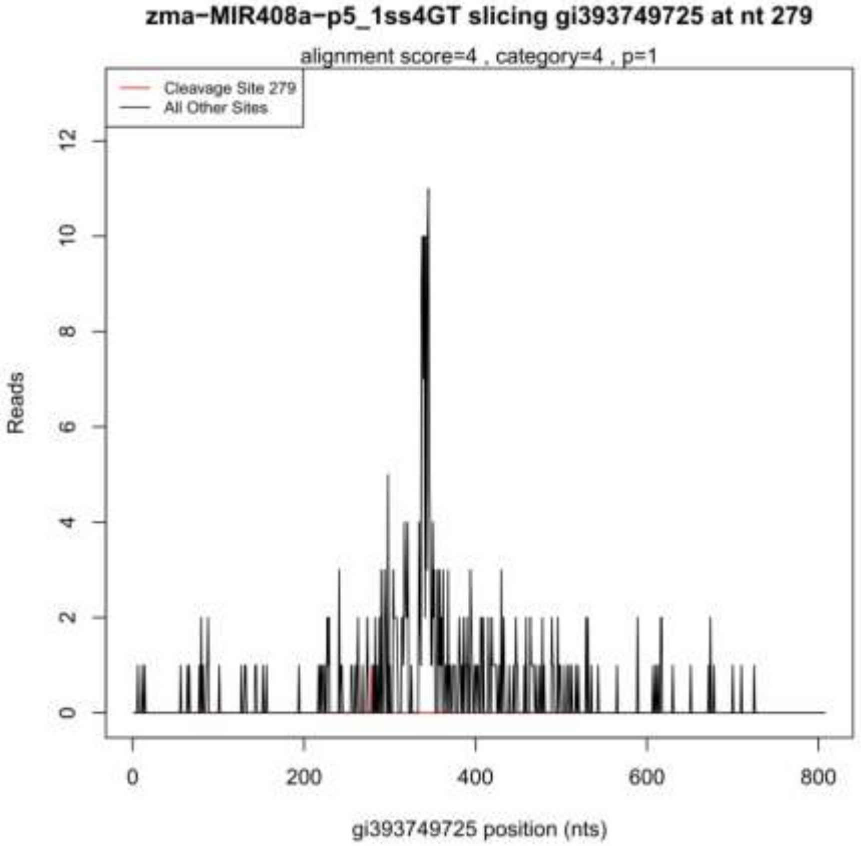

zma-MIR408a-p5_1ss4GT slicing gi393749725 at nt 279
alignment score=4 , category=4 , p=1
Cleavage Site 279
All Other Sites
Reads
0
2
4
6
8
10
12
0
200
400
600
800
gi393749725 position (nts)

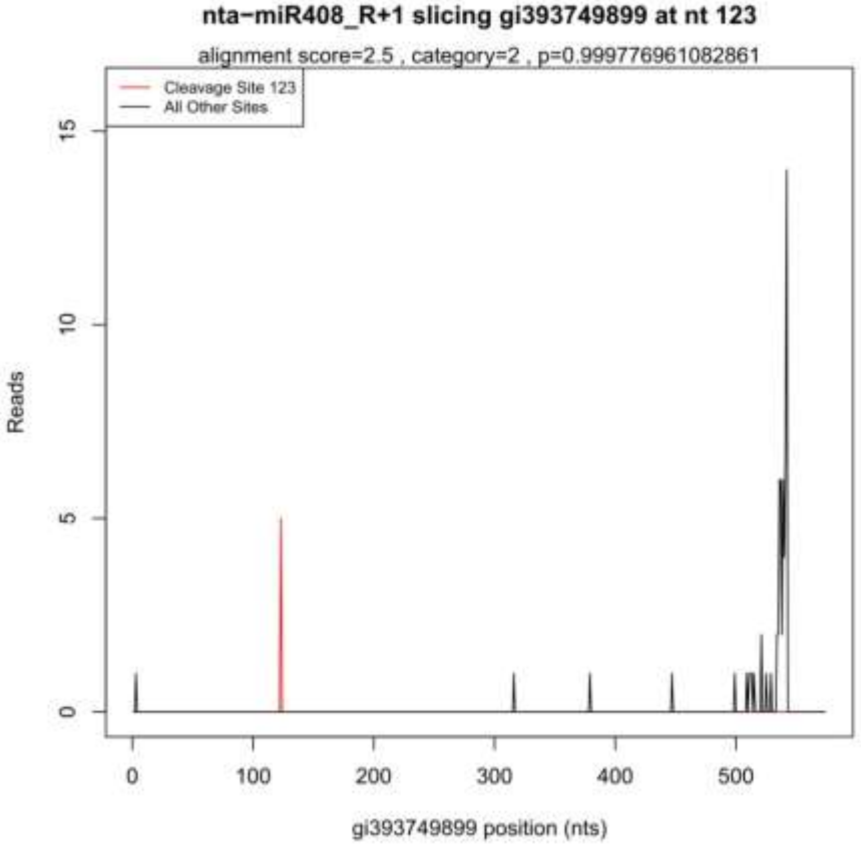

nta−miR408_R+1 slicing gi393749899 at nt 123
alignment score=2.5 , category=2 , p=0.999776961082861
Cleavage Site 123
All Other Sites
Reads
0
5
10
15
0
100
200
300
400
500
gi393749899 position (nts)

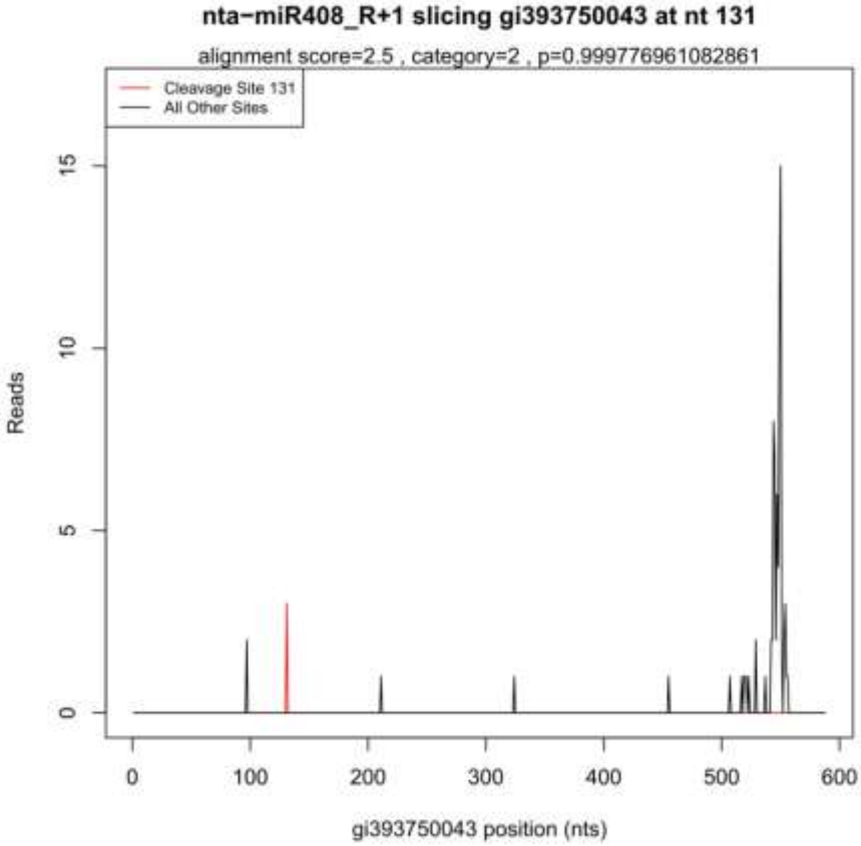

nta−miR408_R+1 slicing gi393750043 at nt 131
alignment score=2.5 , category=2 , p=0.999776961082861
Cleavage Site 131
All Other Sites
Reads
0
5
10
15
0
100
200
300
400
500
600
gi393750043 position (nts)

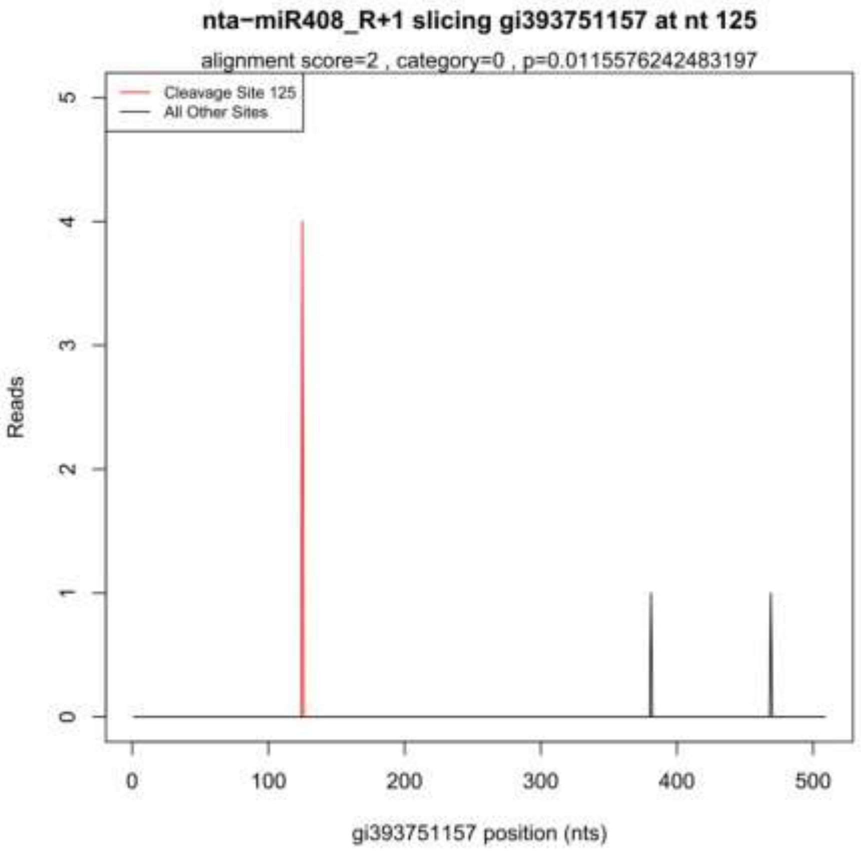
nta−miR408_R+1 slicing gi393751157 at nt 125
alignment score=2 , category=0 , p=0.0115576242483197
Cleavage Site 125
All Other Sites
Reads
0
1
2
3
4
5
0
100
200
300
400
500
gi393751157 position (nts)

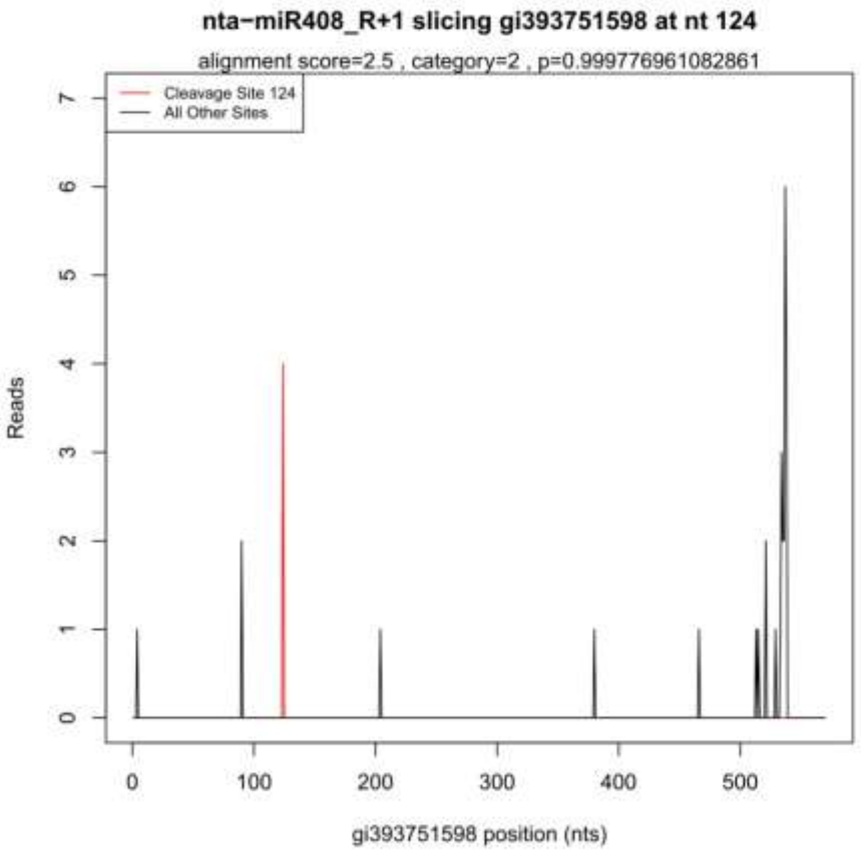
nta−miR408_R+1 slicing gi393751598 at nt 124
alignment score=2.5 , category=2 , p=0.999776961082861
Cleavage Site 124
All Other Sites
Reads
0
1
2
3
4
5
6
7
0
100
200
300
400
500
gi393751598 position (nts)

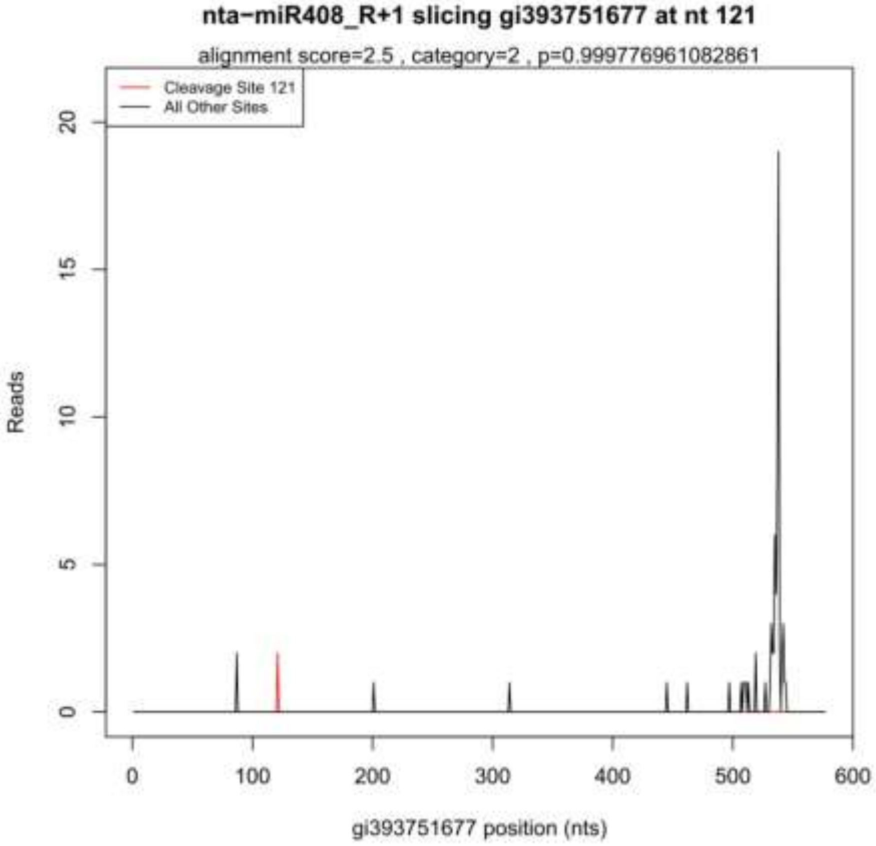
nta−miR408_R+1 slicing gi393751677 at nt 121
alignment score=2.5 , category=2 , p=0.999776961082861
Cleavage Site 121
All Other Sites
Reads
gi393751677 position (nts)

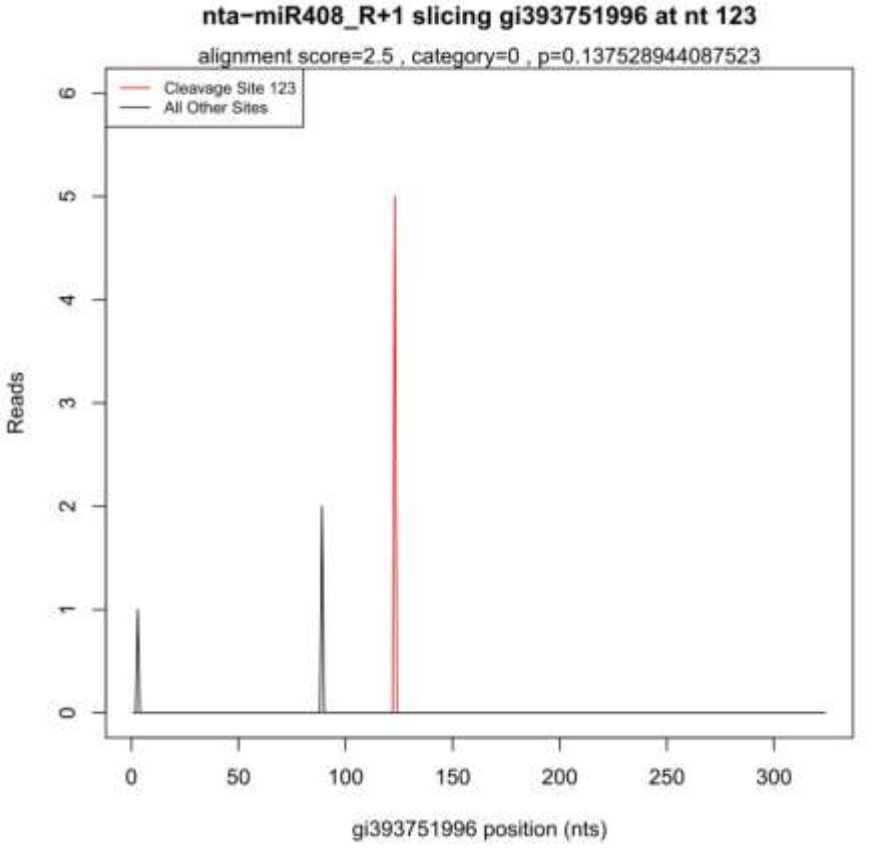
nta−miR408_R+1 slicing gi393751996 at nt 123
alignment score=2.5 , category=0 , p=0.137528944087523
Cleavage Site 123
All Other Sites
Reads
gi393751996 position (nts)

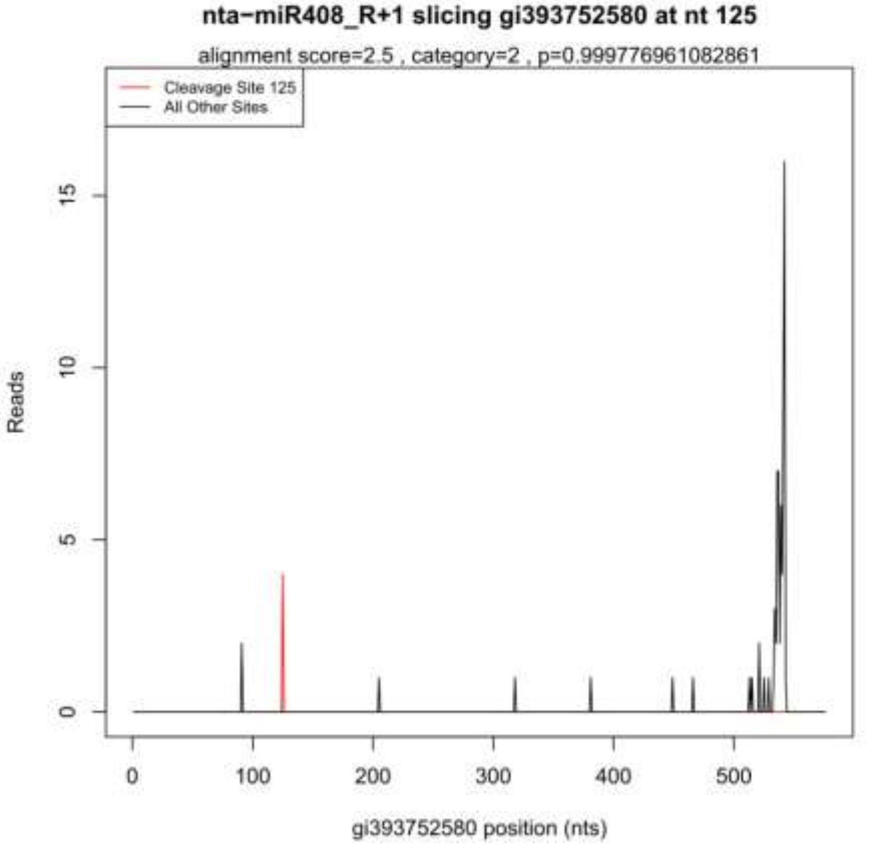

nta−miR408_R+1 slicing gi393752580 at nt 125
alignment score=2.5 , category=2 , p=0.999776961082861
Cleavage Site 125
All Other Sites
Reads
0
5
10
15
0
100
200
300
400
500
gi393752580 position (nts)

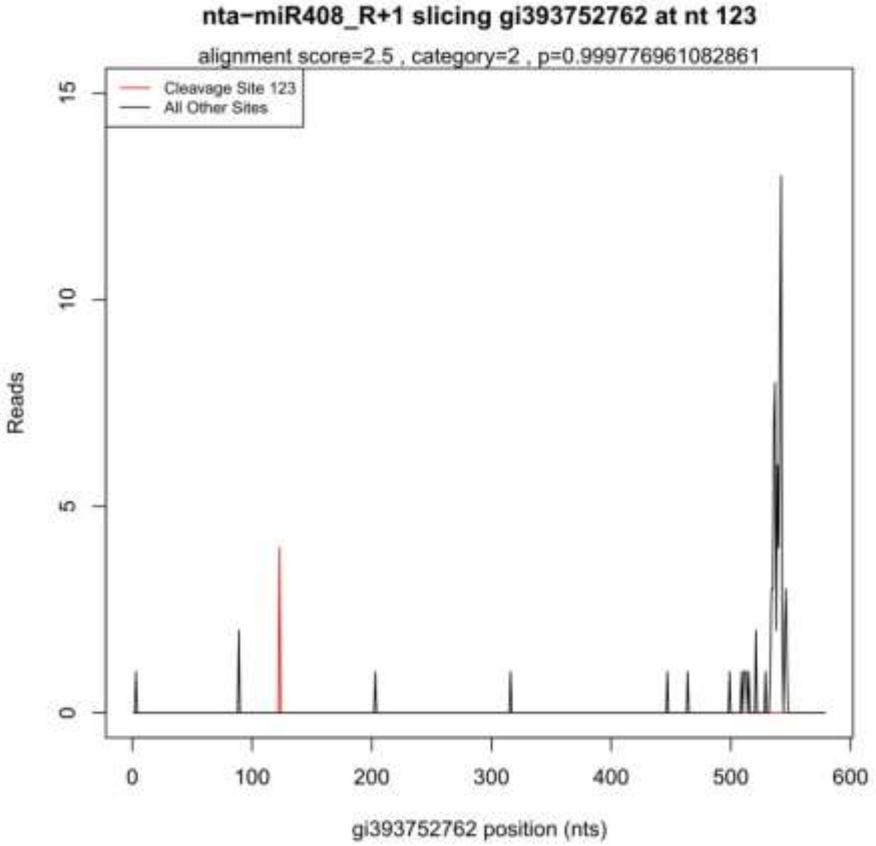

nta−miR408_R+1 slicing gi393752762 at nt 123
alignment score=2.5 , category=2 , p=0.999776961082861
Cleavage Site 123
All Other Sites
Reads
0
5
10
15
0
100
200
300
400
500
600
gi393752762 position (nts)

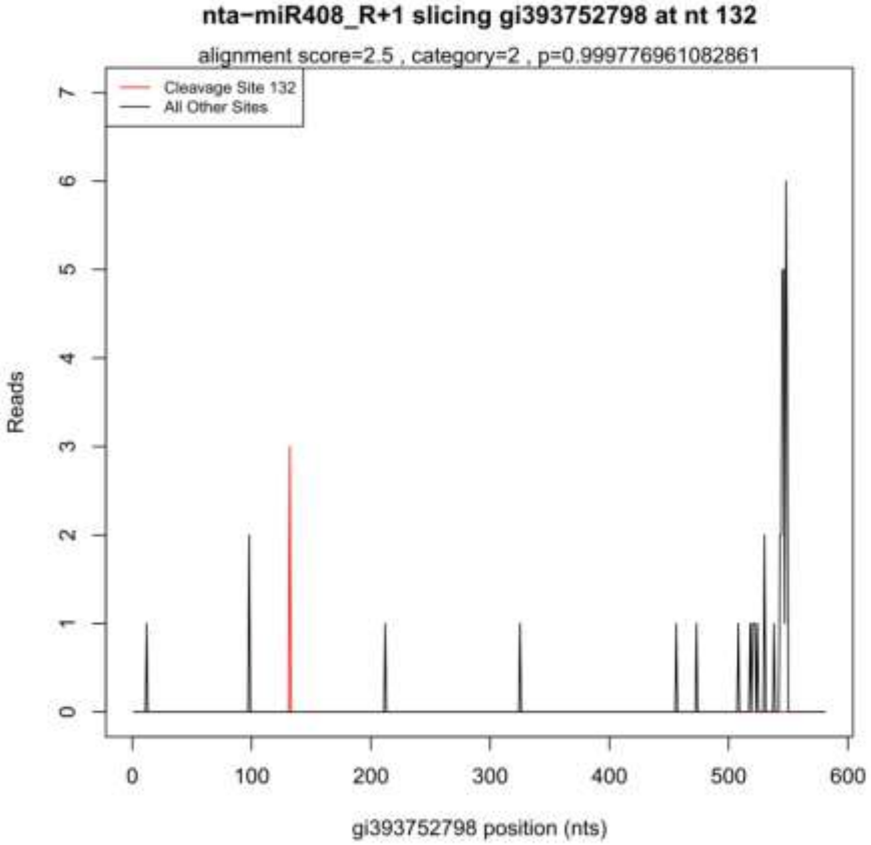
nta−miR408_R+1 slicing gi393752798 at nt 132
alignment score=2.5 , category=2 , p=0.999776961082861
Cleavage Site 132
All Other Sites
Reads
0
1
2
3
4
5
6
7
0
100
200
300
400
500
600
gi393752798 position (nts)

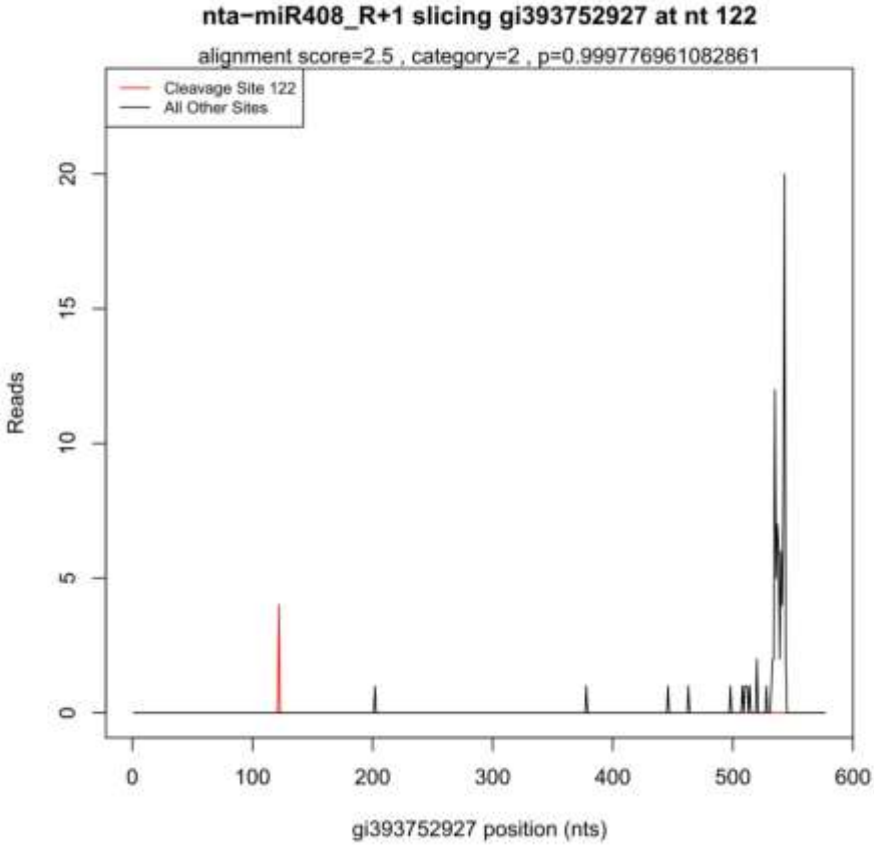
nta−miR408_R+1 slicing gi393752927 at nt 122
alignment score=2.5 , category=2 , p=0.999776961082861
Cleavage Site 122
All Other Sites
Reads
0
5
10
15
20
0
100
200
300
400
500
600
gi393752927 position (nts)

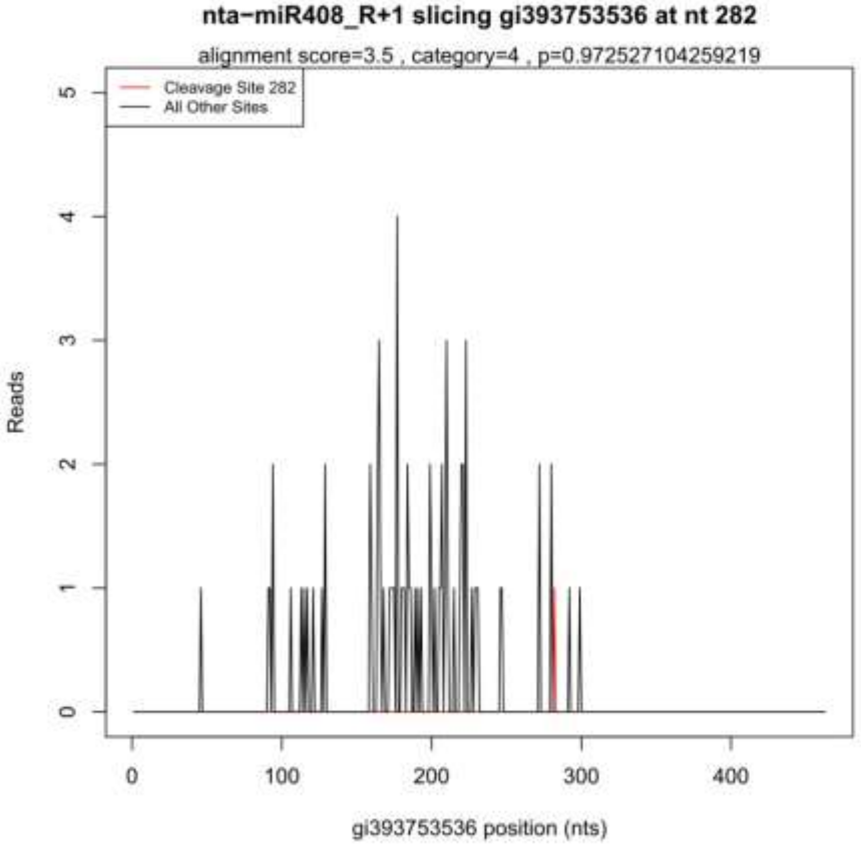
nta−miR408_R+1 slicing gi393753536 at nt 282
alignment score=3.5 , category=4 , p=0.972527104259219
Cleavage Site 282
All Other Sites
Reads
0
1
2
3
4
5
0
100
200
300
400
gi393753536 position (nts)

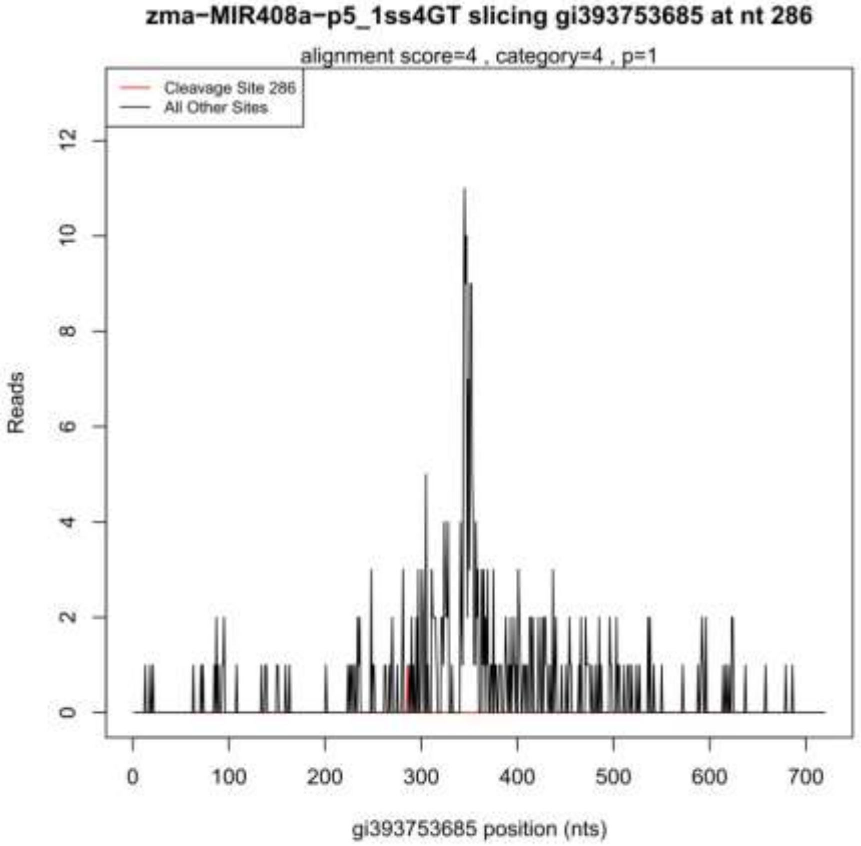
zma−MIR408a−p5_1ss4GT slicing gi393753685 at nt 286
alignment score=4 , category=4 , p=1
Cleavage Site 286
All Other Sites
Reads
0
2
4
6
8
10
12
0
100
200
300
400
500
600
700
gi393753685 position (nts)

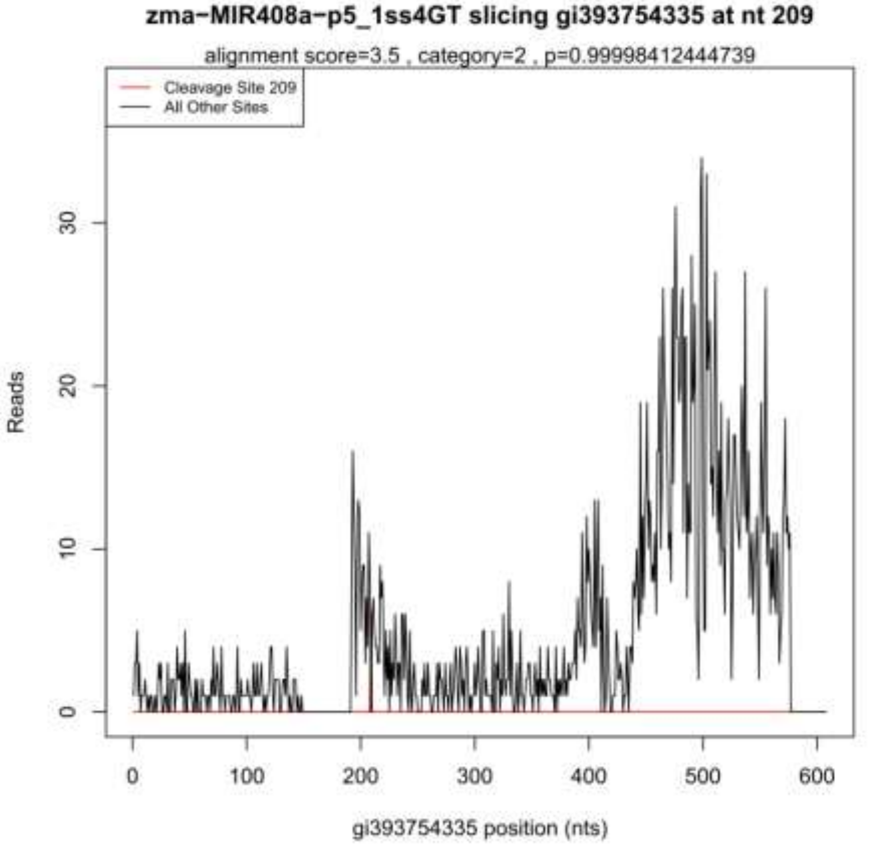
zma−MIR408a−p5_1ss4GT slicing gi393754335 at nt 209
alignment score=3.5 , category=2 , p=0.99998412444739
Cleavage Site 209
All Other Sites
Reads
0
10
20
30
0
100
200
300
400
500
600
gi393754335 position (nts)

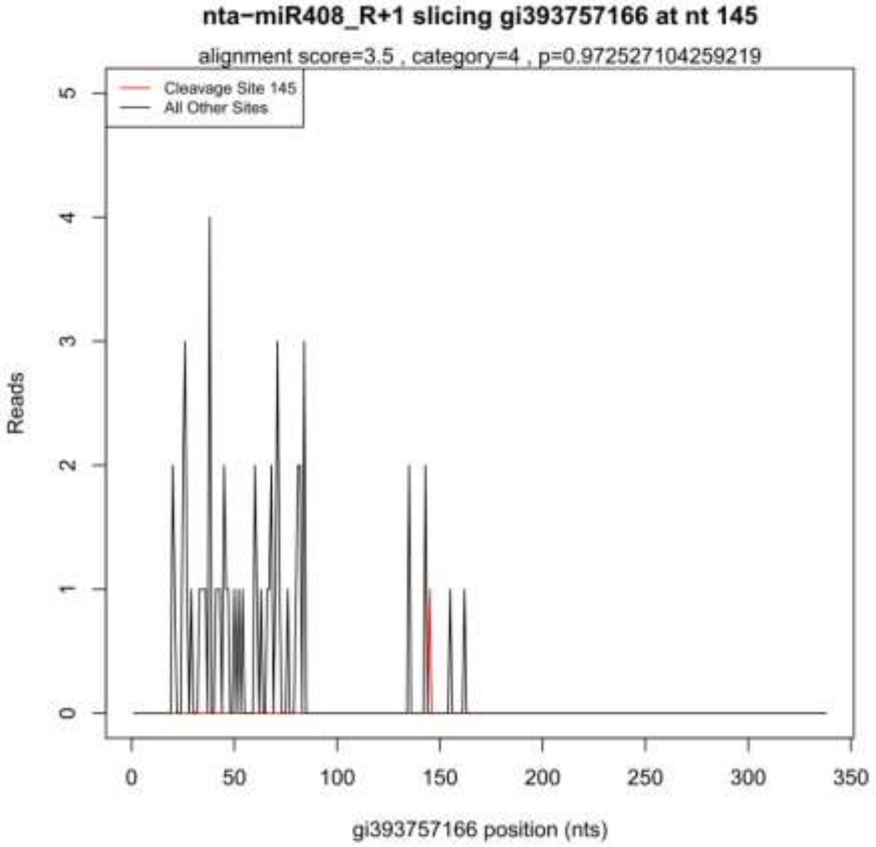
nta−miR408_R+1 slicing gi393757166 at nt 145
alignment score=3.5 , category=4 , p=0.972527104259219
Cleavage Site 145
All Other Sites
Reads
0
1
2
3
4
5
0
50
100
150
200
250
300
350
gi393757166 position (nts)

Figure S4 t-plot for targets of the known miRNA found in +C and –C libraries of *camellia sinensis*. Signature abundance throughout the length of the transcript is show. Arrows indicate signature consistent with miRNA-directed cleavage.
