# Supplementary material for: Identification and characterization of cold-responsive microRNAs in tea plant (Camellia sinensis) and their targets using high-throughput sequencing and degradome analysis
Source: BMC Plant Biol. 2014 Oct 21;14:271. doi: 10.1186/s12870-014-0271-x (PMC4209041; doi:10.1186/s12870-014-0271-x)
Supplement: Additional file 9: Figure S5. — Microarray analysis of the known and new miRNAs from tea plant cultivar ‘Baiye 1’ treated with cold and cold-free. [file 12870_2014_271_MOESM9_ESM.pdf]

PC-5p-92106_7 slicing gi393749713 at nt 116

alignment score=4 , category=3 , p=0.328669572257198

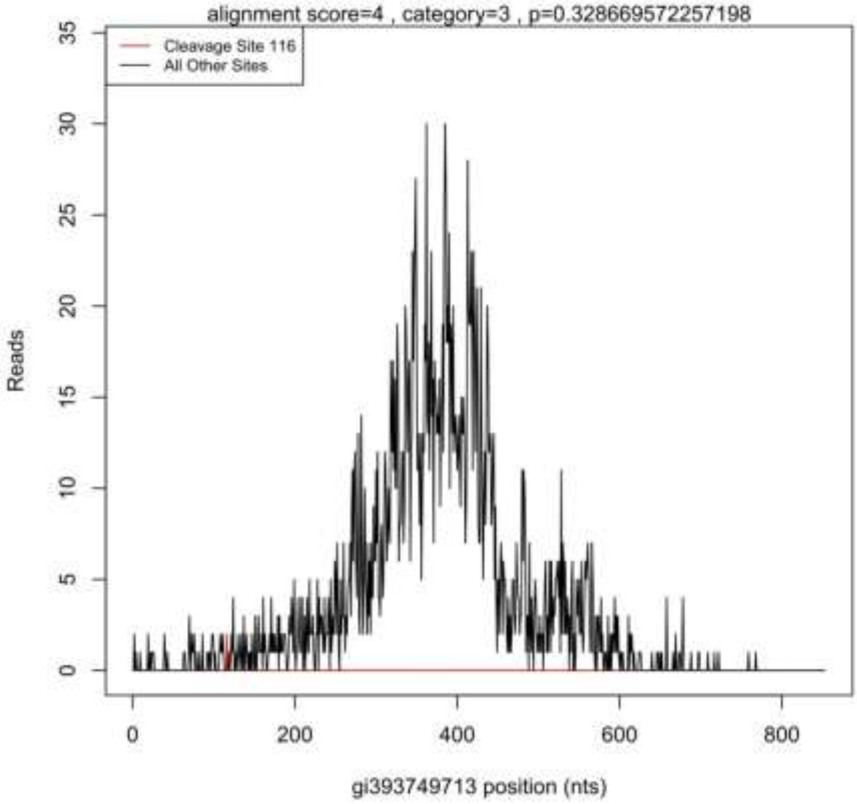

PC-5p-92106_7 slicing gi393748405 at nt 116

alignment score=4 , category=3 , p=0.328669572257198

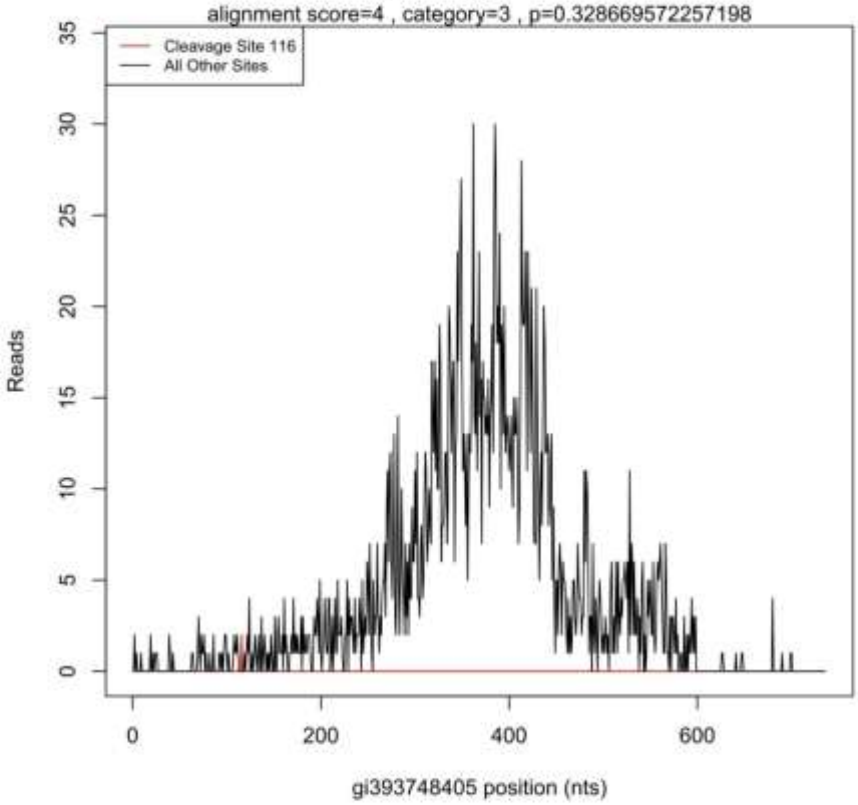

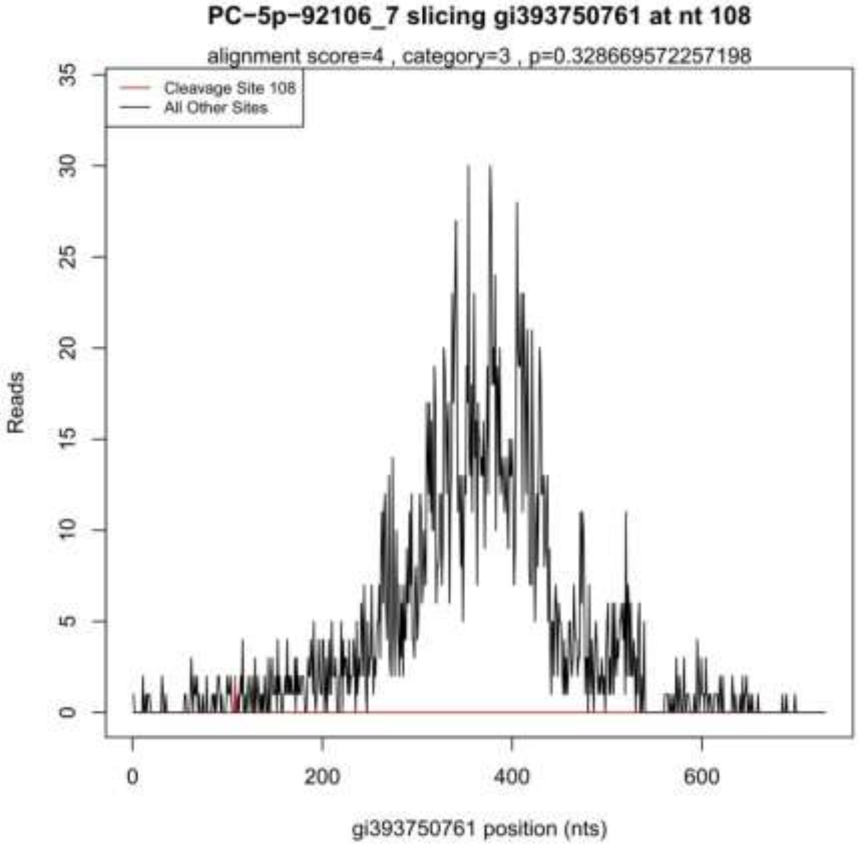
PC−5p−92106_7 slicing gi393750761 at nt 108
alignment score=4 , category=3 , p=0.328669572257198
Cleavage Site 108
All Other Sites
Reads
0
5
10
15
20
25
30
35
0
200
400
600
gi393750761 position (nts)

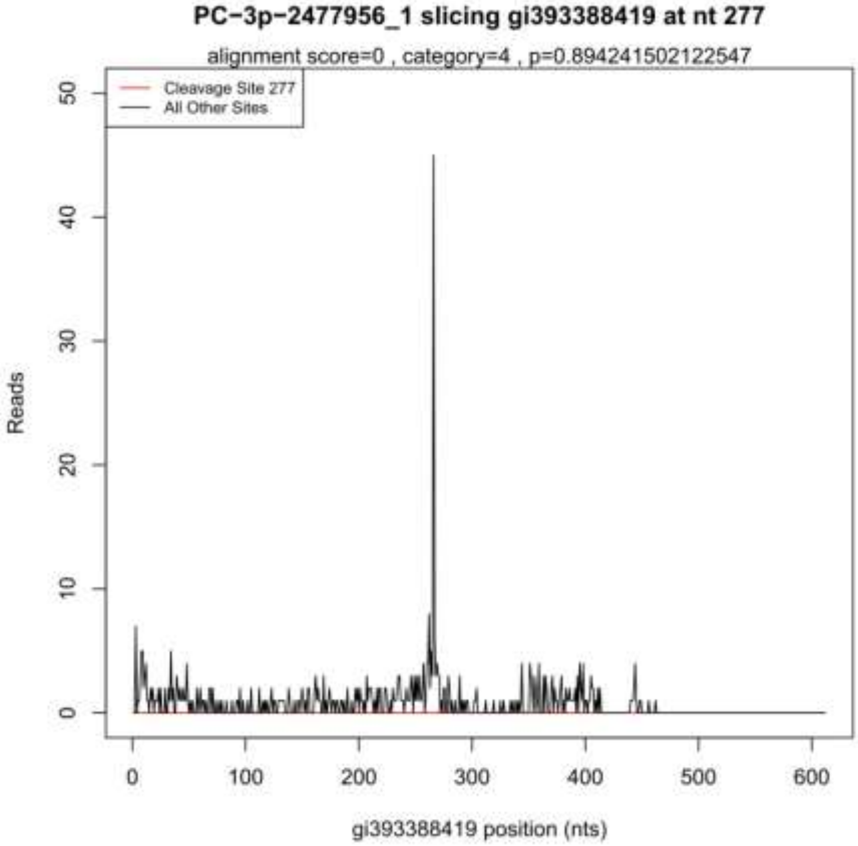
PC−3p−2477956_1 slicing gi393388419 at nt 277
alignment score=0 , category=4 , p=0.894241502122547
Cleavage Site 277
All Other Sites
Reads
0
10
20
30
40
50
0
100
200
300
400
500
600
gi393388419 position (nts)

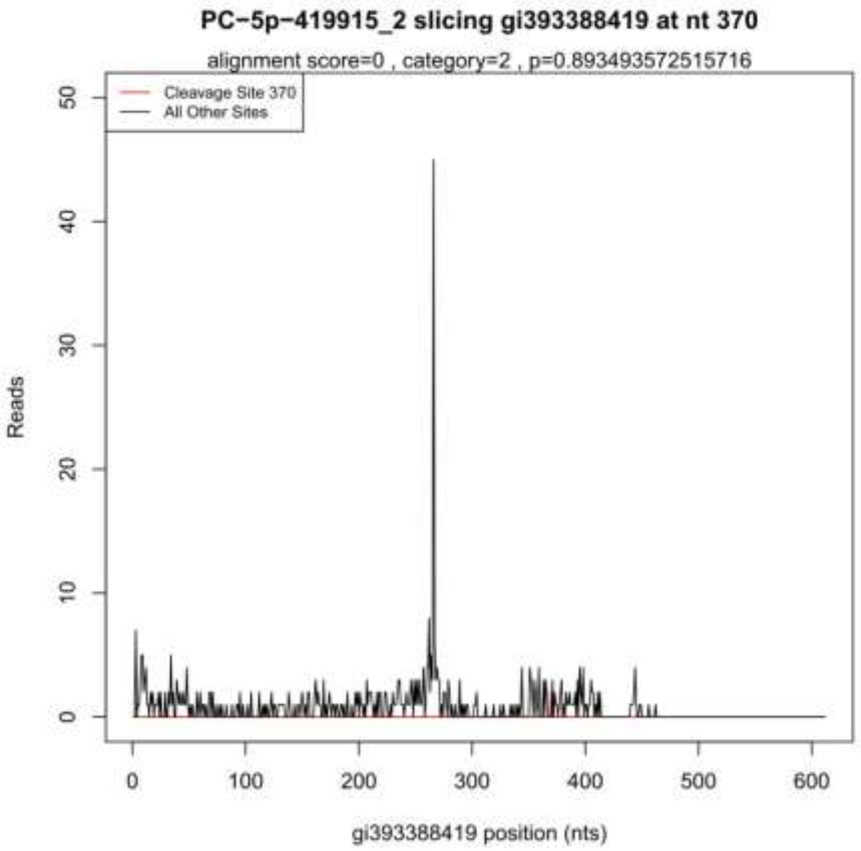
PC−5p−419915_2 slicing gi393388419 at nt 370
alignment score=0 , category=2 , p=0.893493572515716
Cleavage Site 370
All Other Sites
Reads
0
10
20
30
40
50
0
100
200
300
400
500
600
gi393388419 position (nts)

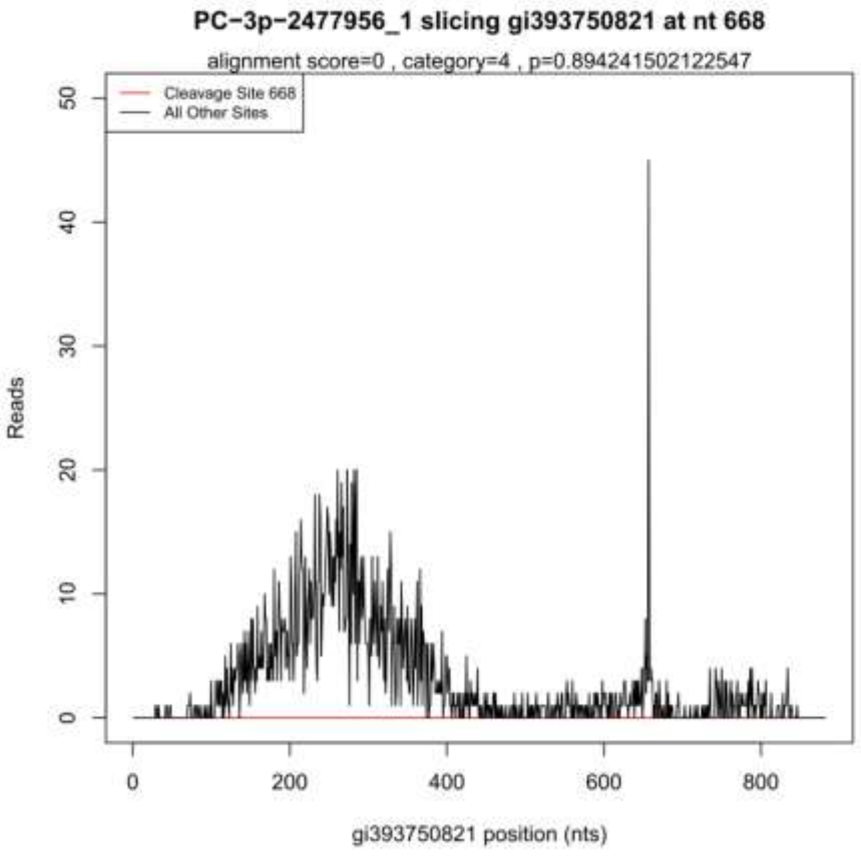
PC−3p−2477956_1 slicing gi393750821 at nt 668
alignment score=0 , category=4 , p=0.894241502122547
Cleavage Site 668
All Other Sites
Reads
0
10
20
30
40
50
0
200
400
600
800
gi393750821 position (nts)

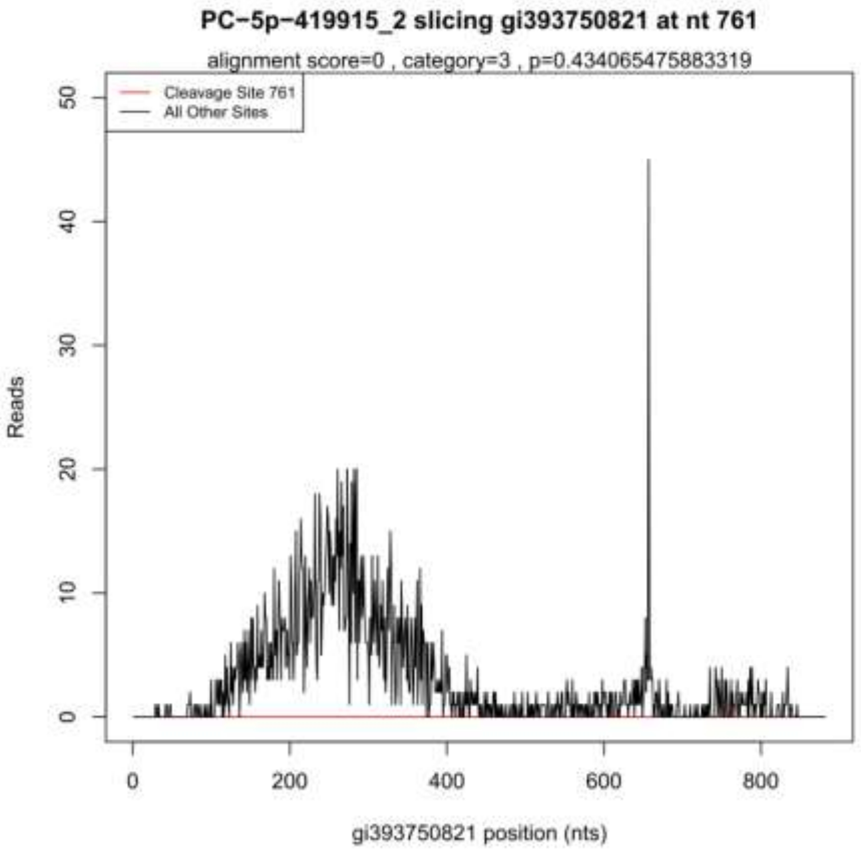
PC−5p−419915_2 slicing gi393750821 at nt 761
alignment score=0 , category=3 , p=0.434065475883319
Cleavage Site 761
All Other Sites
Reads
0
10
20
30
40
50
0
200
400
600
800
gi393750821 position (nts)

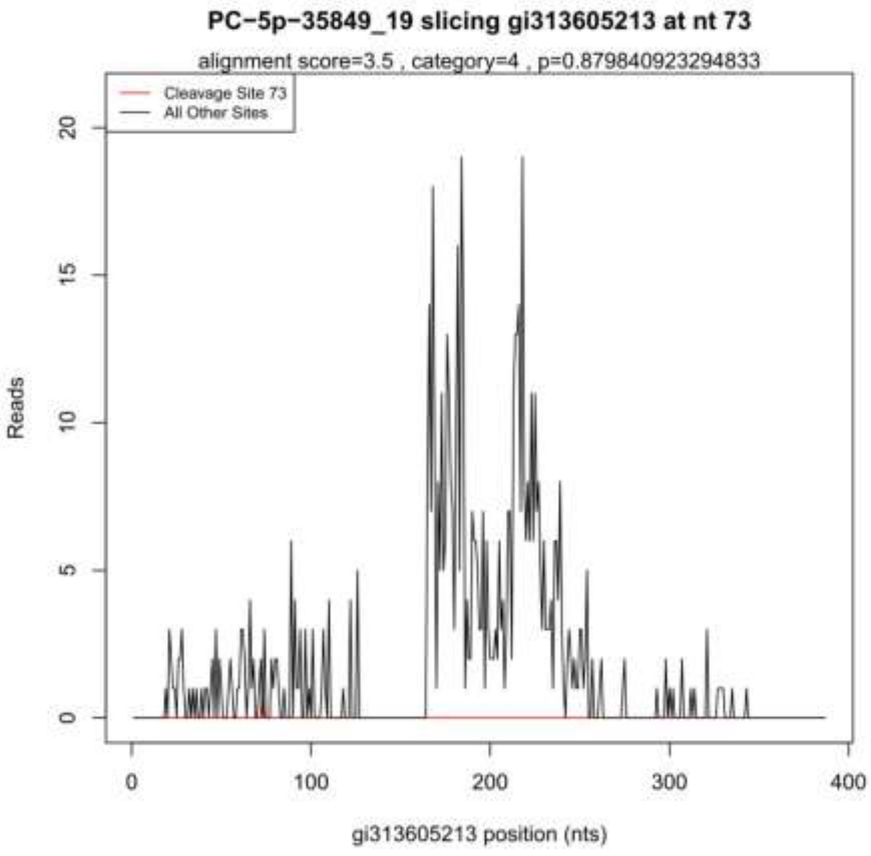
PC−5p−35849_19 slicing gi313605213 at nt 73
alignment score=3.5 , category=4 , p=0.879840923294833
Cleavage Site 73
All Other Sites
Reads
0
5
10
15
20
0
100
200
300
400
gi313605213 position (nts)

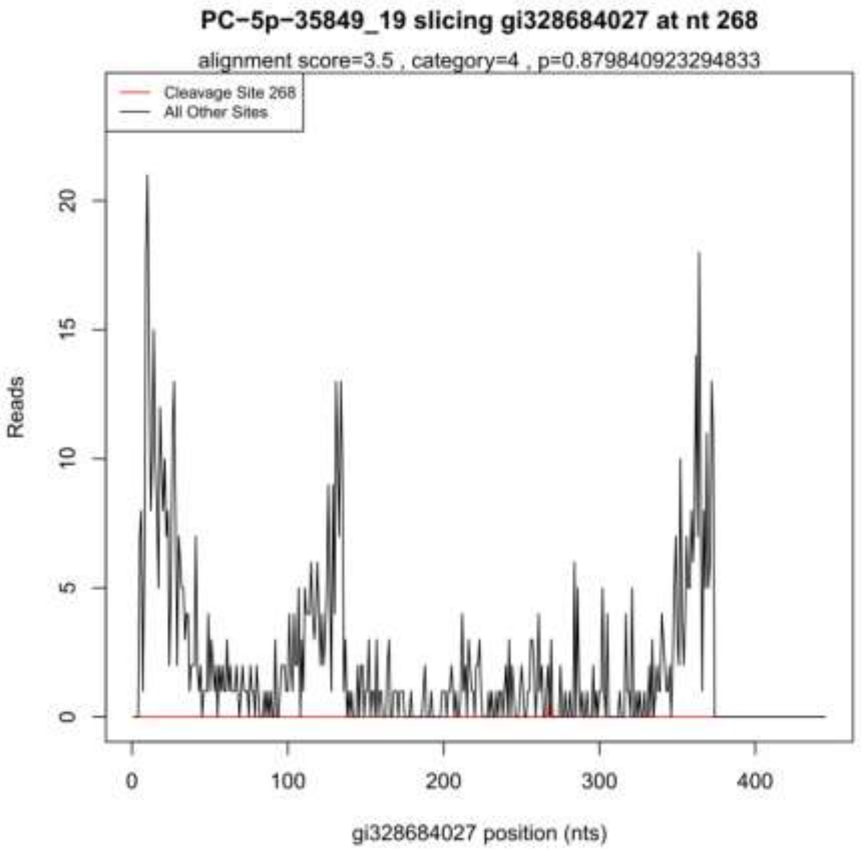
PC−5p−35849_19 slicing gi328684027 at nt 268
alignment score=3.5 , category=4 , p=0.879840923294833
Cleavage Site 268
All Other Sites
Reads
0
5
10
15
20
0
100
200
300
400
gi328684027 position (nts)

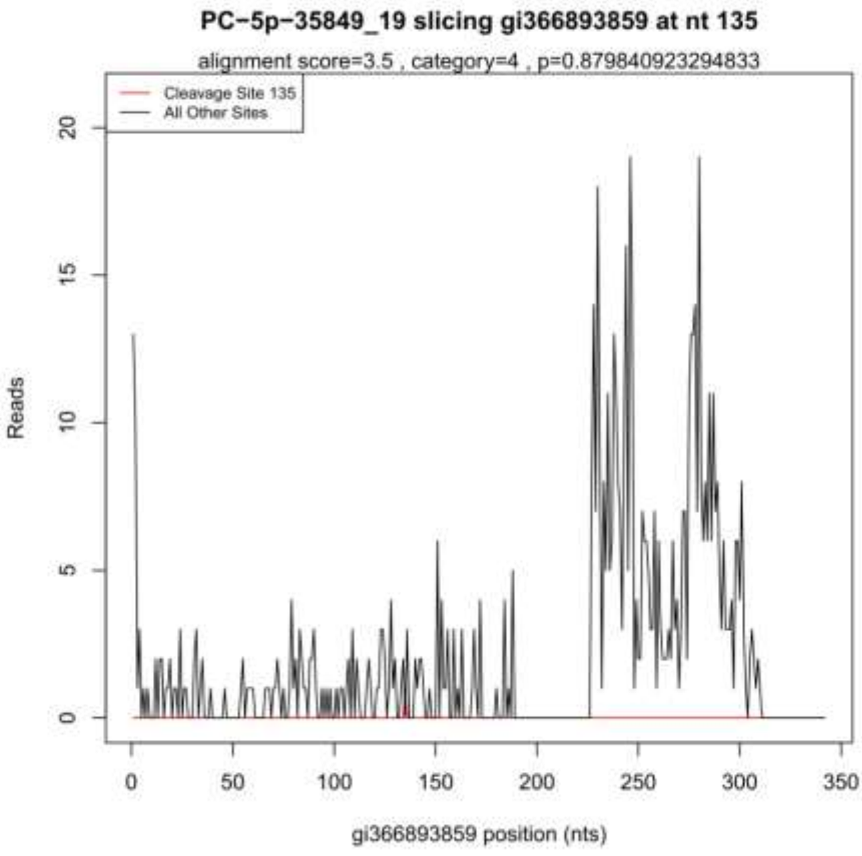
PC−5p−35849_19 slicing gi366893859 at nt 135
alignment score=3.5 , category=4 , p=0.879840923294833
Cleavage Site 135
All Other Sites
Reads
0
5
10
15
20
0
50
100
150
200
250
300
350
gi366893859 position (nts)

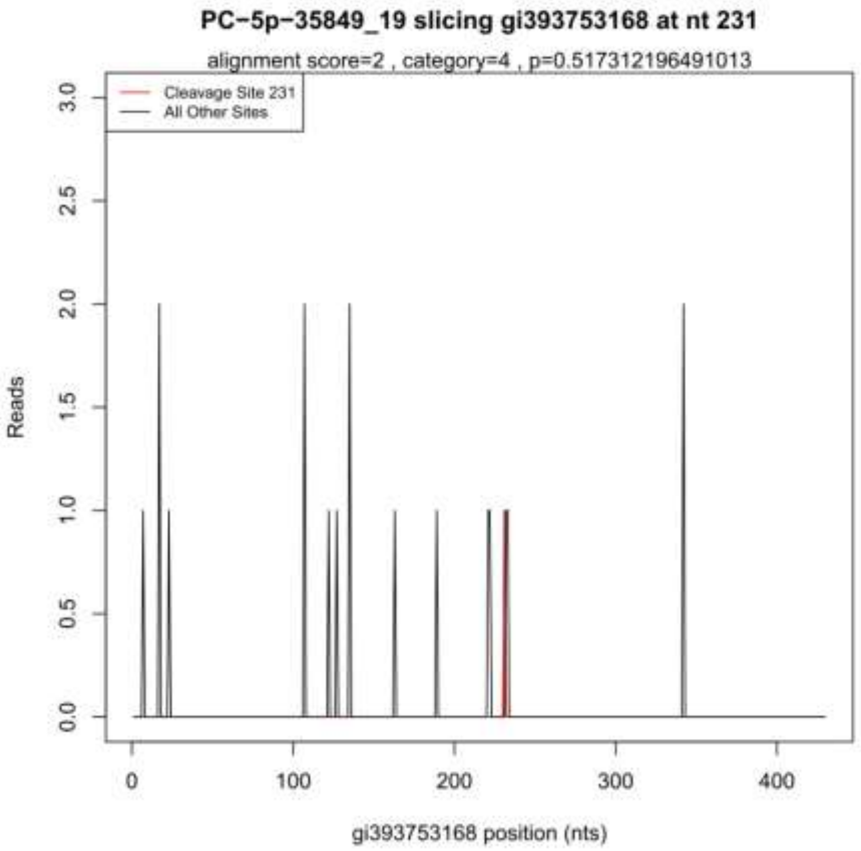
PC−5p−35849_19 slicing gi393753168 at nt 231
alignment score=2 , category=4 , p=0.517312196491013
Cleavage Site 231
All Other Sites
Reads
gi393753168 position (nts)

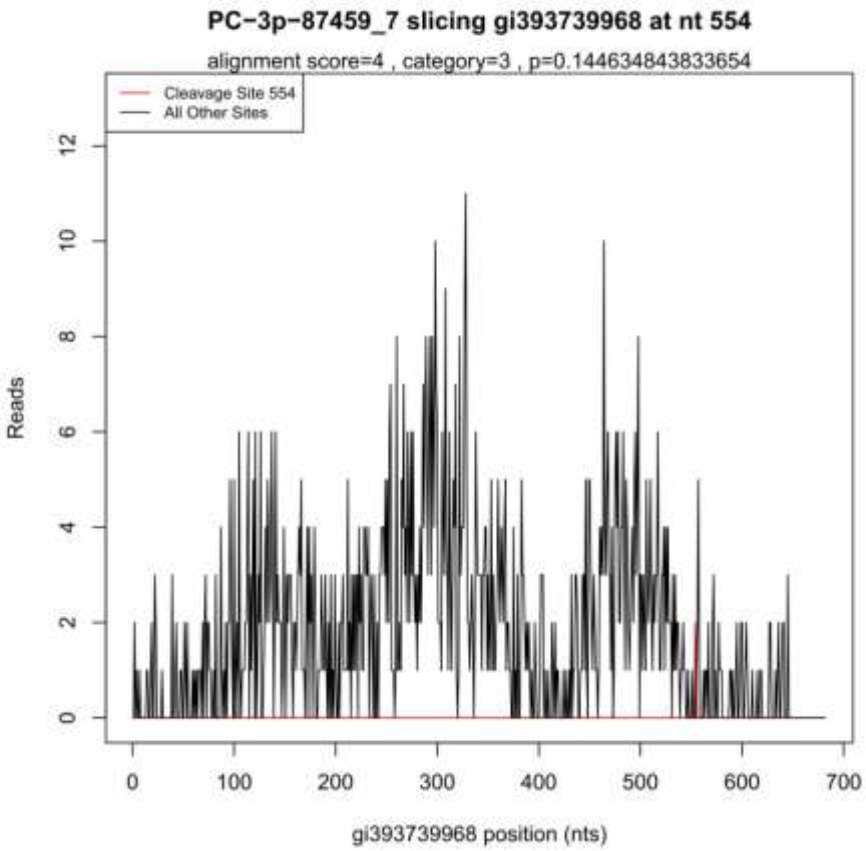
PC−3p−87459_7 slicing gi393739968 at nt 554
alignment score=4 , category=3 , p=0.144634843833654
Cleavage Site 554
All Other Sites
Reads
gi393739968 position (nts)

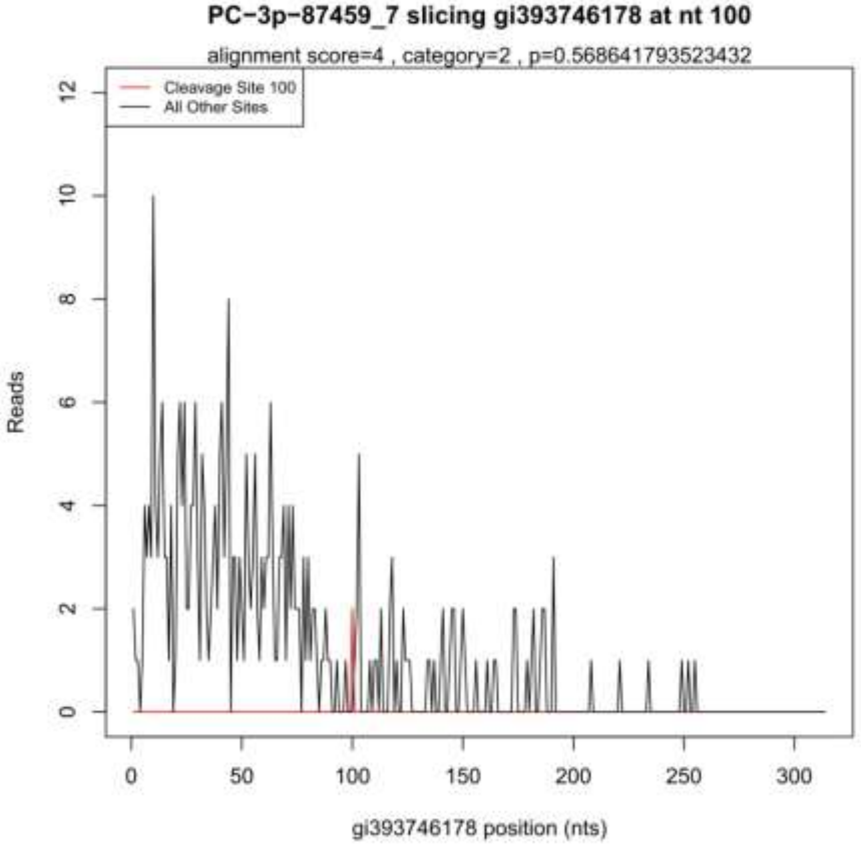
PC-3p-87459_7 slicing gi393746178 at nt 100
alignment score=4 , category=2 , p=0.568641793523432
Cleavage Site 100
All Other Sites
Reads
0
2
4
6
8
10
12
0
50
100
150
200
250
300
gi393746178 position (nts)

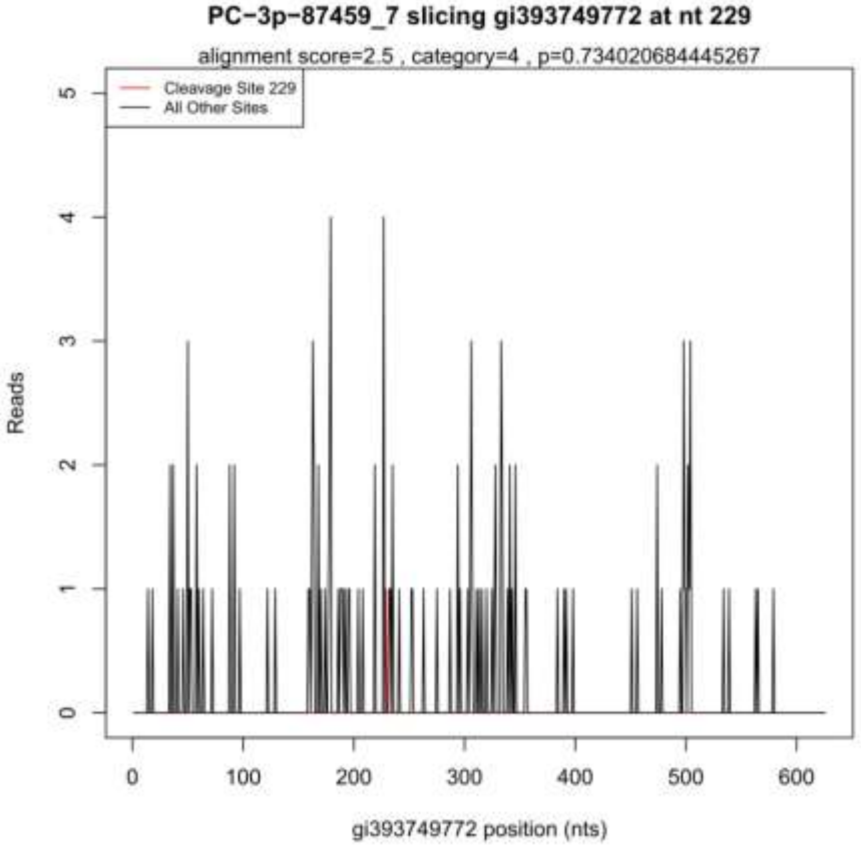
PC-3p-87459_7 slicing gi393749772 at nt 229
alignment score=2.5 , category=4 , p=0.734020684445267
Cleavage Site 229
All Other Sites
Reads
0
1
2
3
4
5
0
100
200
300
400
500
600
gi393749772 position (nts)

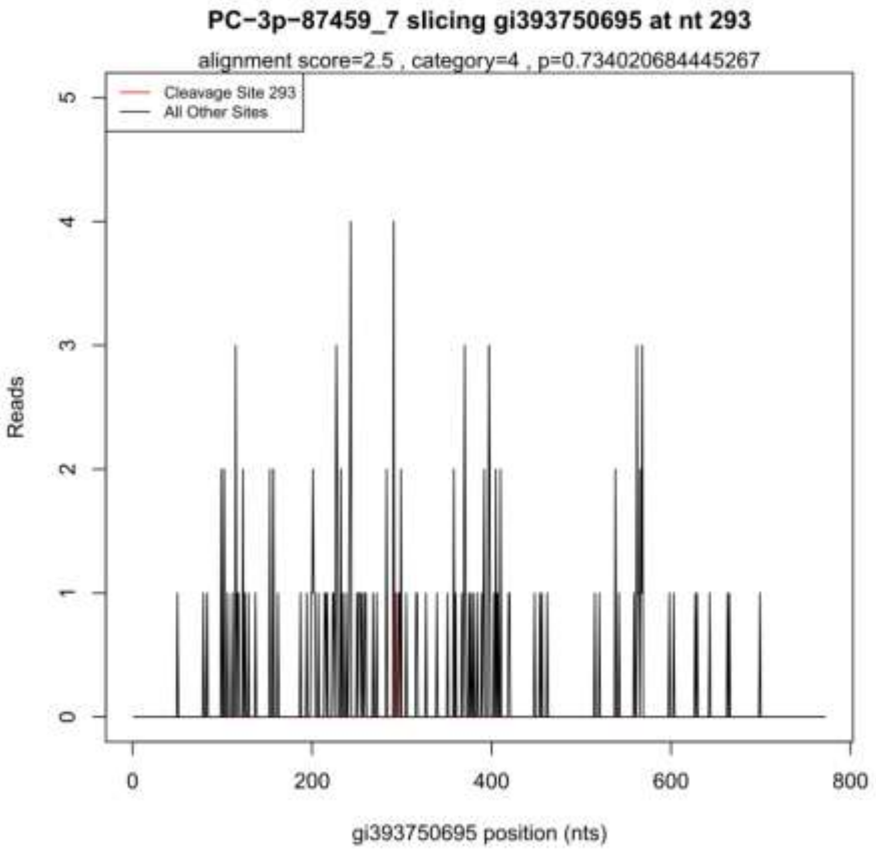
PC−3p−87459_7 slicing gi393750695 at nt 293
alignment score=2.5 , category=4 , p=0.734020684445267
Cleavage Site 293
All Other Sites
Reads
0
1
2
3
4
5
0
200
400
600
800
gi393750695 position (nts)

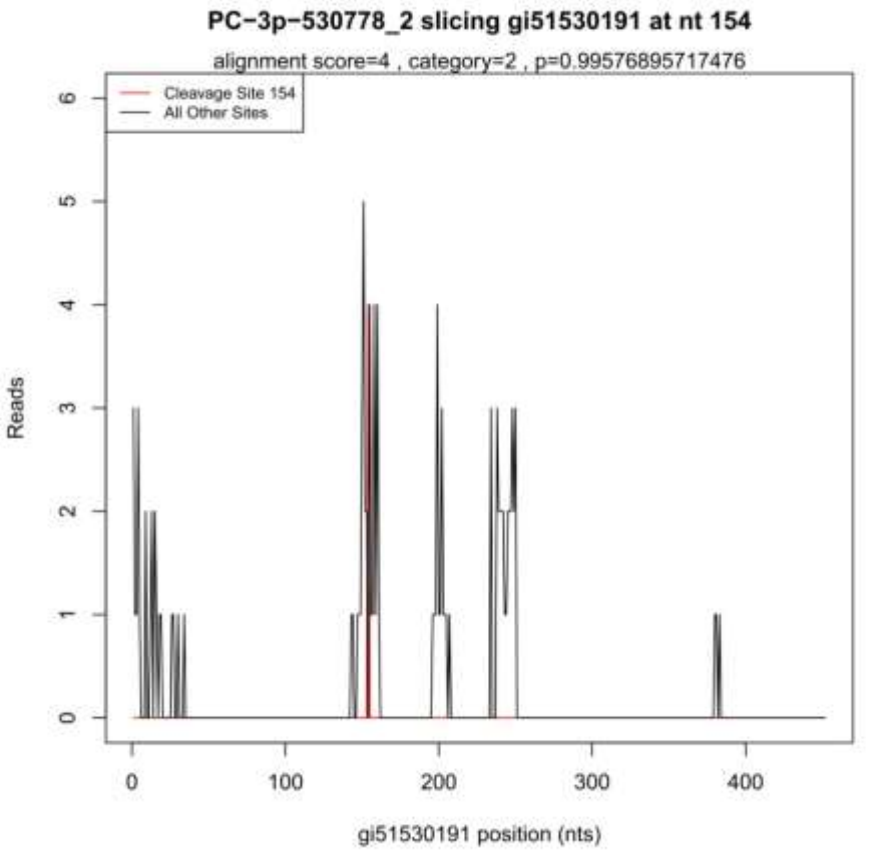
PC−3p−530778_2 slicing gi51530191 at nt 154
alignment score=4 , category=2 , p=0.99576895717476
Cleavage Site 154
All Other Sites
Reads
0
1
2
3
4
5
6
0
100
200
300
400
gi51530191 position (nts)

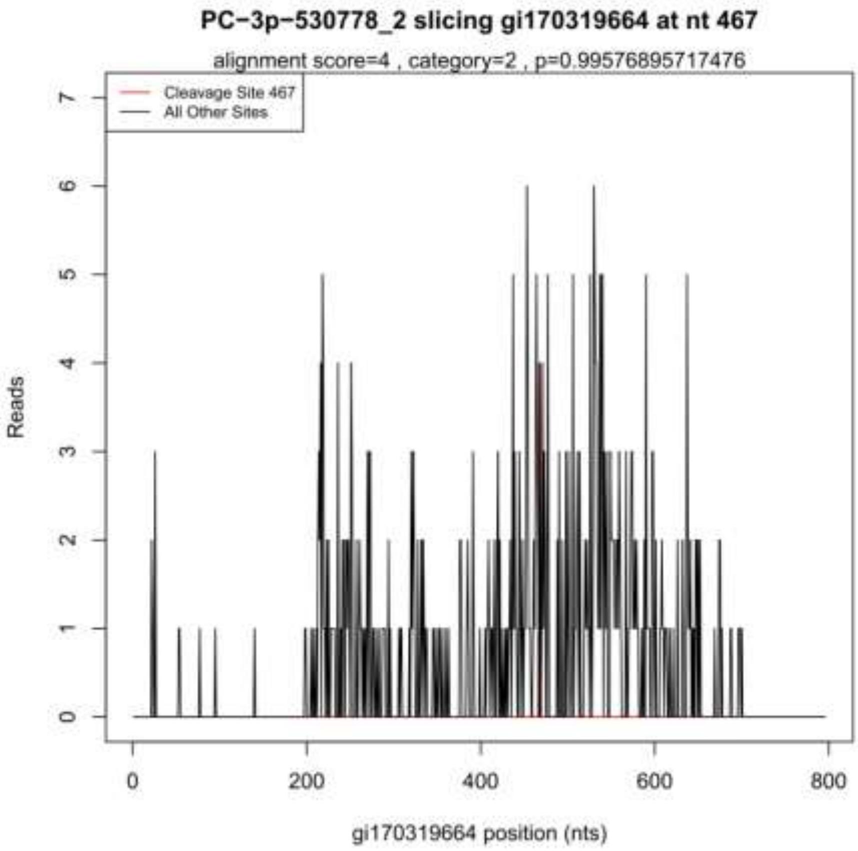
PC−3p−530778_2 slicing gi170319664 at nt 467
alignment score=4 , category=2 , p=0.99576895717476
Cleavage Site 467
All Other Sites
Reads
gi170319664 position (nts)
0
200
400
600
800
1
2
3
4
5
6
7

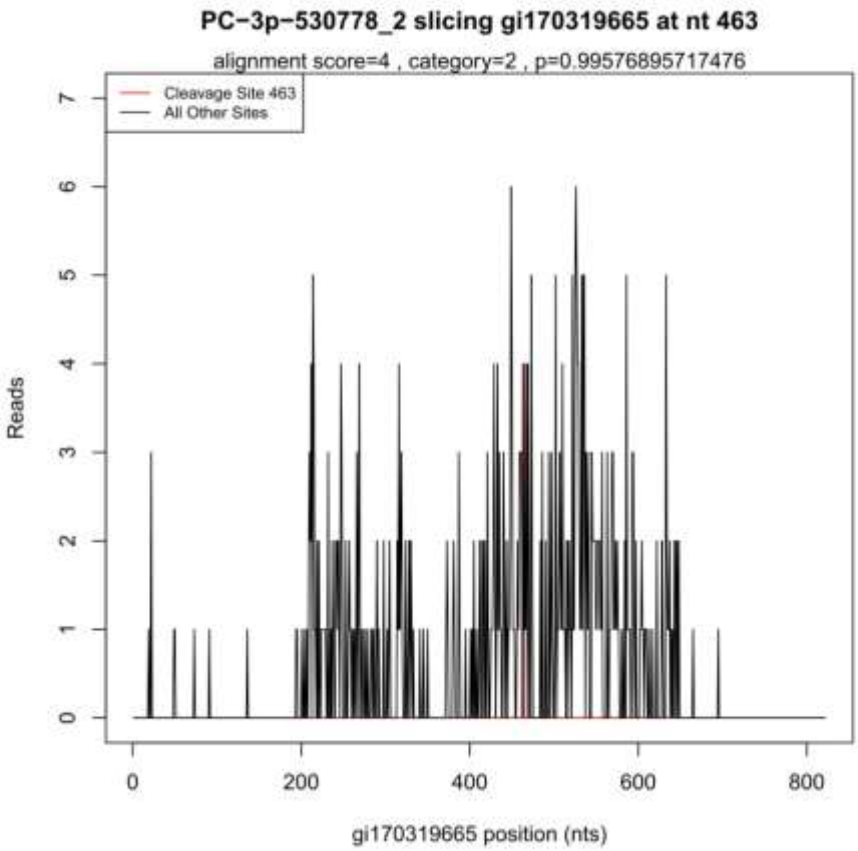
PC−3p−530778_2 slicing gi170319665 at nt 463
alignment score=4 , category=2 , p=0.99576895717476
Cleavage Site 463
All Other Sites
Reads
gi170319665 position (nts)
0
200
400
600
800
1
2
3
4
5
6
7

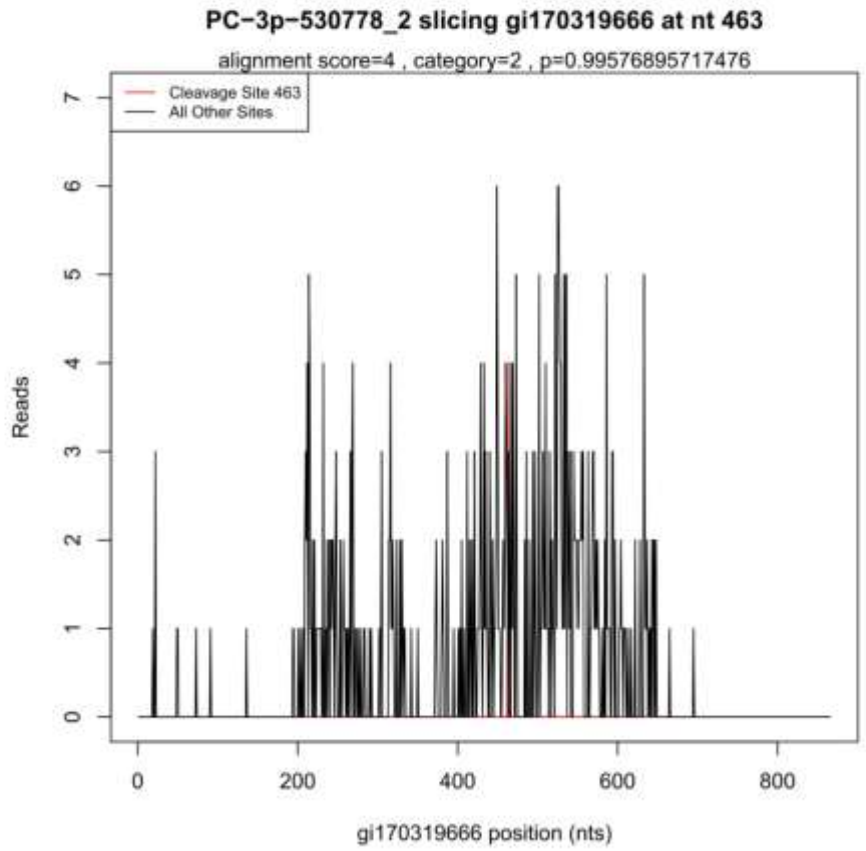
PC−3p−530778_2 slicing gi170319666 at nt 463
alignment score=4 , category=2 , p=0.99576895717476
Cleavage Site 463
All Other Sites
Reads
0
1
2
3
4
5
6
7
0
200
400
600
800
gi170319666 position (nts)

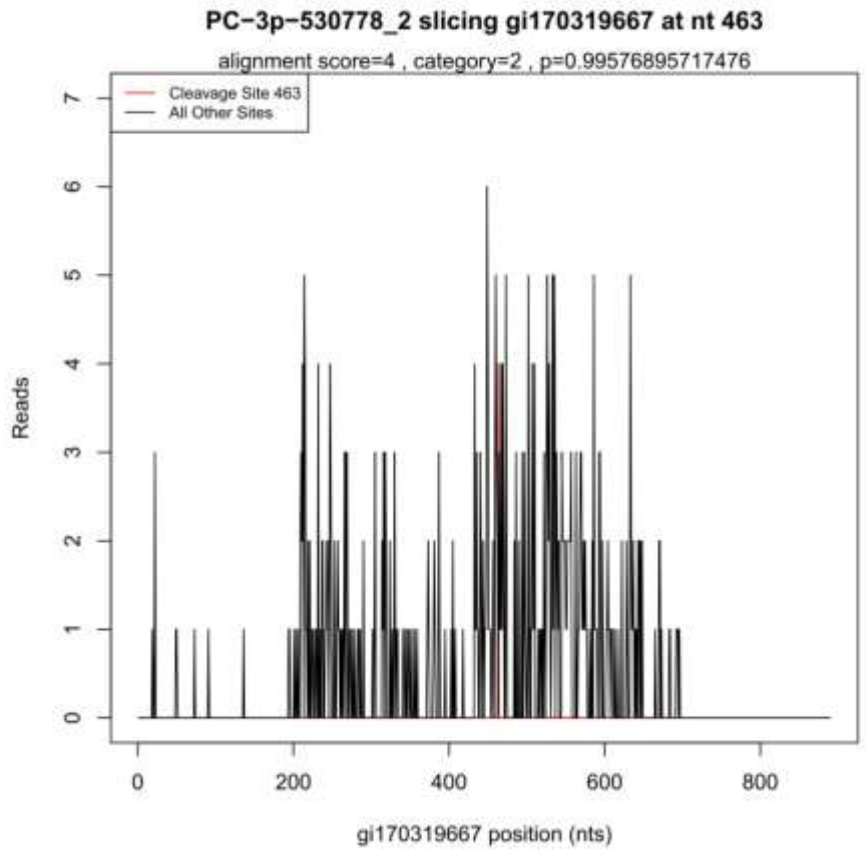
PC−3p−530778_2 slicing gi170319667 at nt 463
alignment score=4 , category=2 , p=0.99576895717476
Cleavage Site 463
All Other Sites
Reads
0
1
2
3
4
5
6
7
0
200
400
600
800
gi170319667 position (nts)

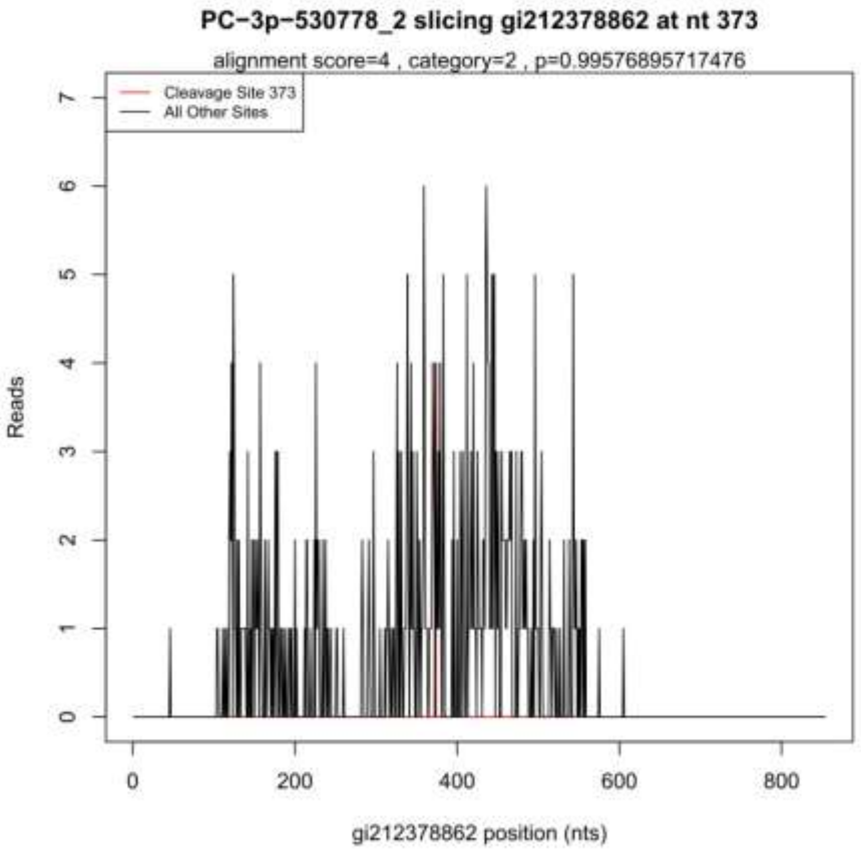

PC-3p-530778_2 slicing gi212378862 at nt 373
alignment score=4 , category=2 , p=0.99576895717476
Cleavage Site 373
All Other Sites
Reads
gi212378862 position (nts)

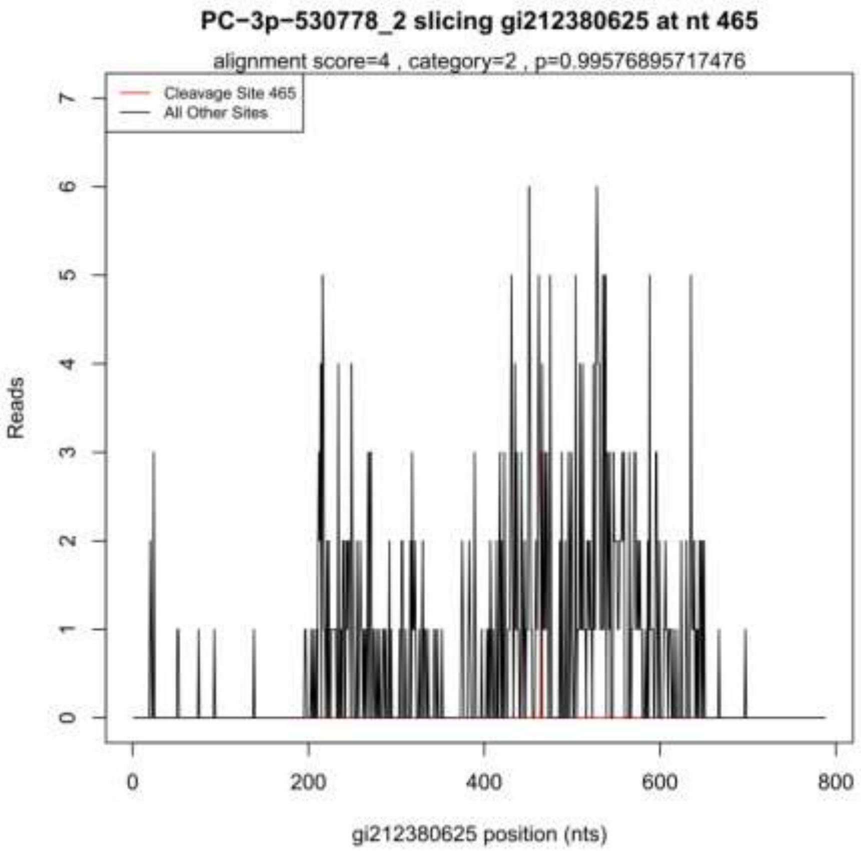

PC-3p-530778_2 slicing gi212380625 at nt 465
alignment score=4 , category=2 , p=0.99576895717476
Cleavage Site 465
All Other Sites
Reads
gi212380625 position (nts)

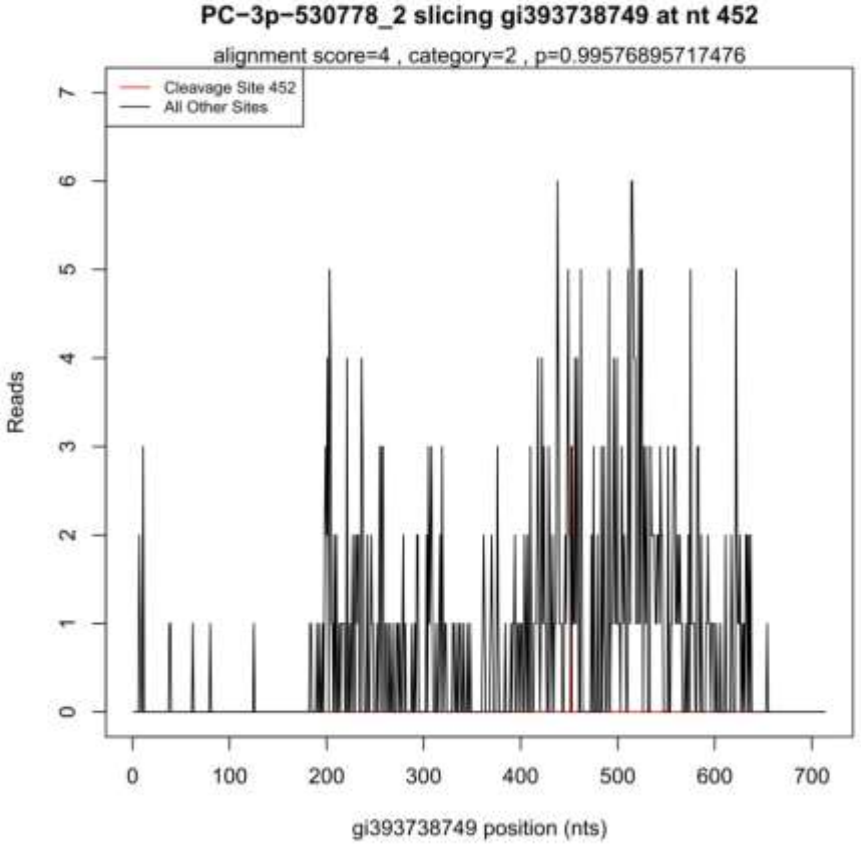
PC−3p−530778_2 slicing gi393738749 at nt 452
alignment score=4 , category=2 , p=0.99576895717476
Cleavage Site 452
All Other Sites
Reads
gi393738749 position (nts)

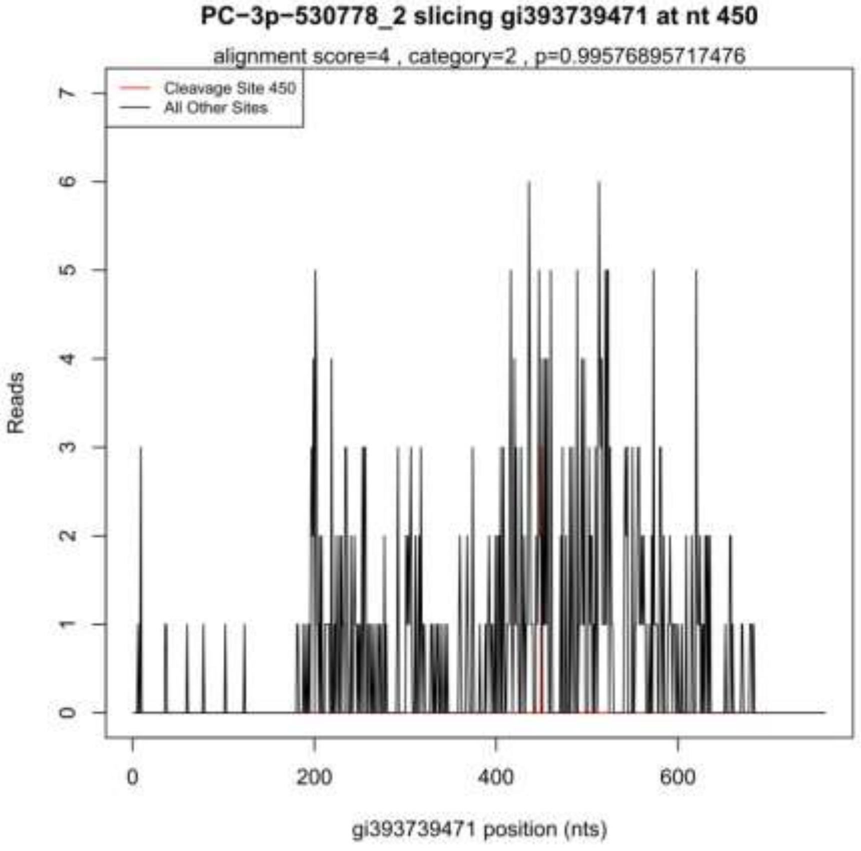
PC−3p−530778_2 slicing gi393739471 at nt 450
alignment score=4 , category=2 , p=0.99576895717476
Cleavage Site 450
All Other Sites
Reads
gi393739471 position (nts)

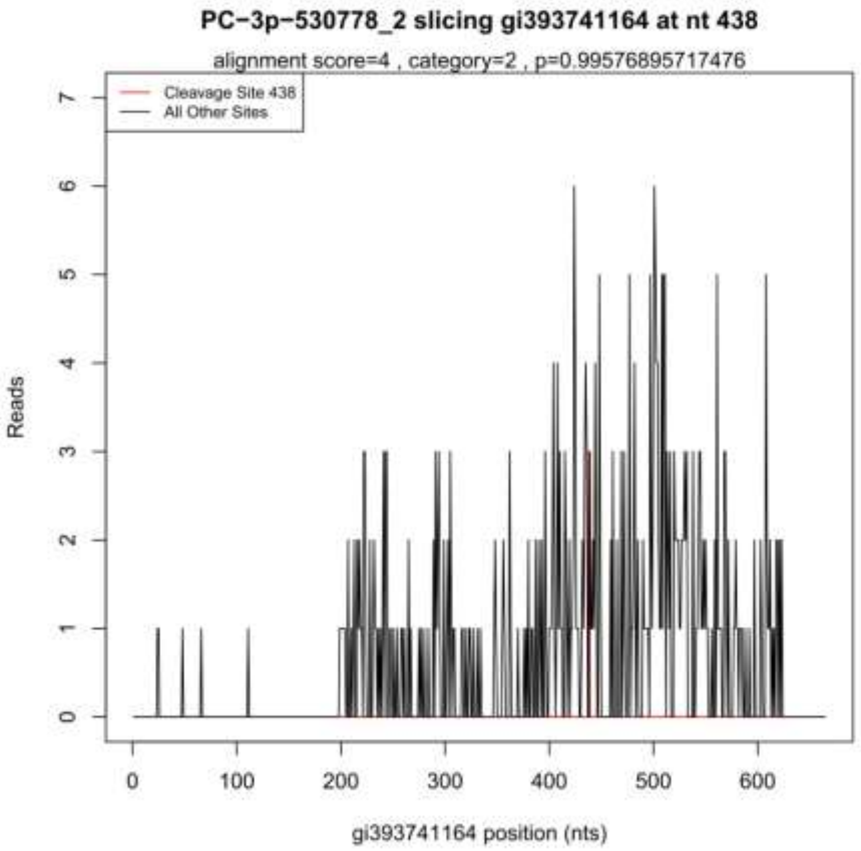
PC-3p-530778_2 slicing gi393741164 at nt 438
alignment score=4 , category=2 , p=0.99576895717476
Cleavage Site 438
All Other Sites
Reads
gi393741164 position (nts)

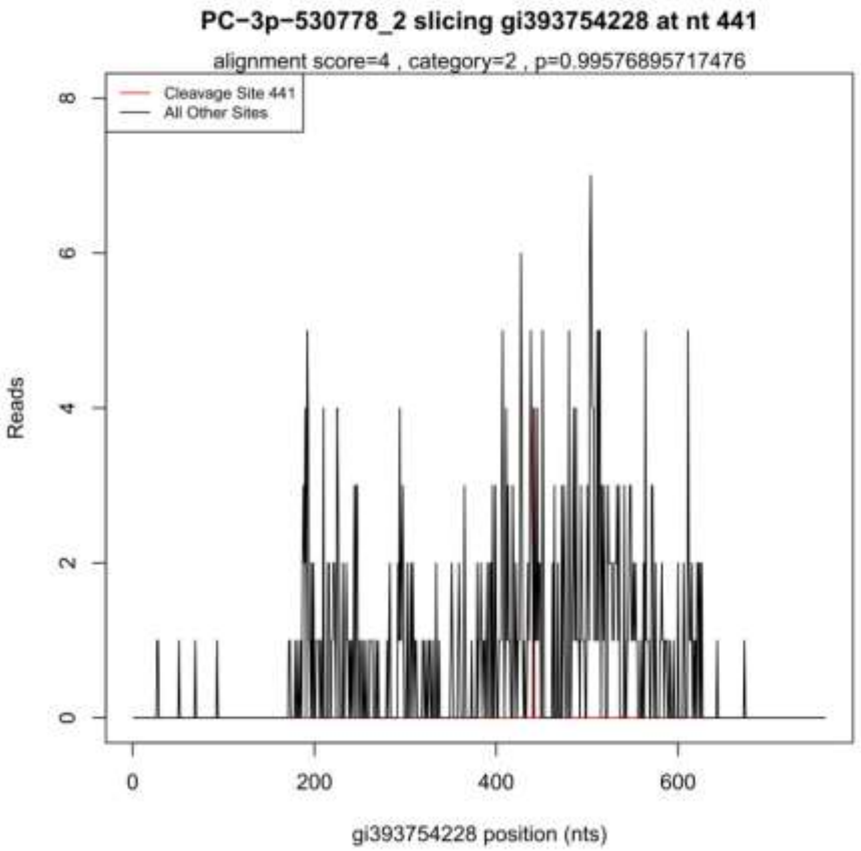
PC-3p-530778_2 slicing gi393754228 at nt 441
alignment score=4 , category=2 , p=0.99576895717476
Cleavage Site 441
All Other Sites
Reads
gi393754228 position (nts)

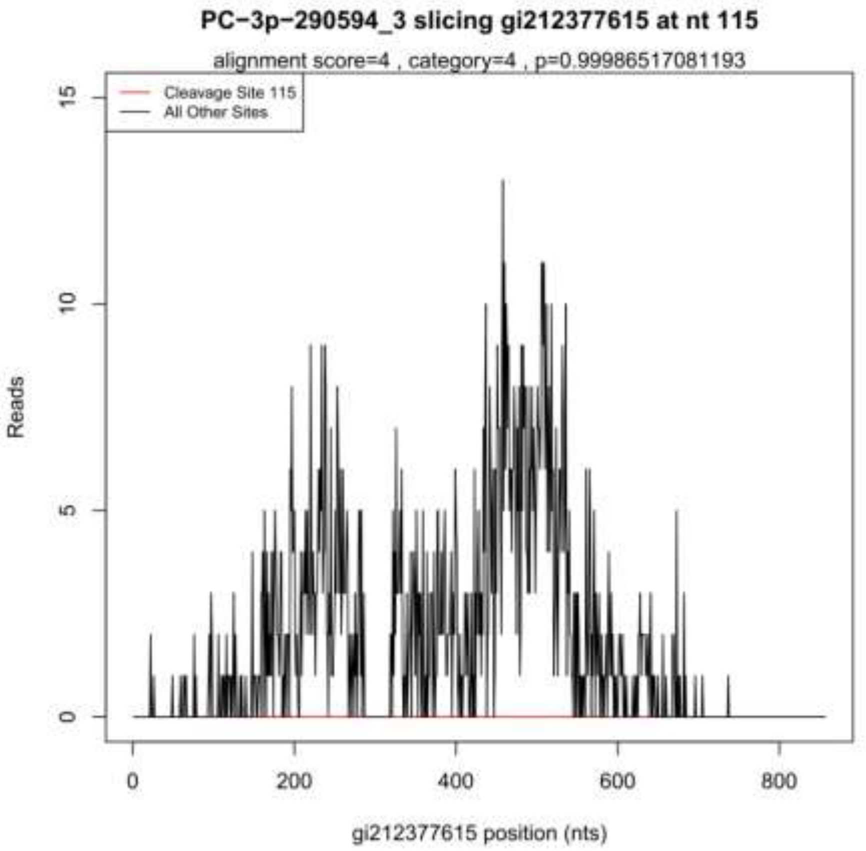

PC−3p−290594_3 slicing gi212377615 at nt 115
alignment score=4 , category=4 , p=0.99986517081193
Cleavage Site 115
All Other Sites
Reads
0
5
10
15
0
200
400
600
800
gi212377615 position (nts)

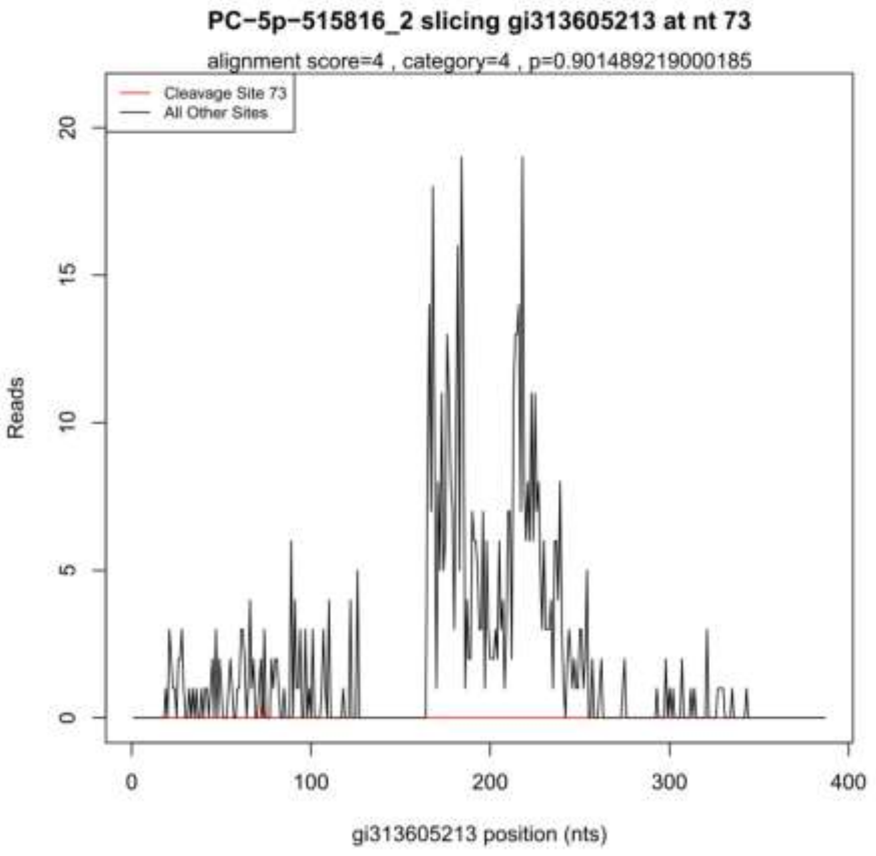

PC−5p−515816_2 slicing gi313605213 at nt 73
alignment score=4 , category=4 , p=0.901489219000185
Cleavage Site 73
All Other Sites
Reads
0
5
10
15
20
0
100
200
300
400
gi313605213 position (nts)

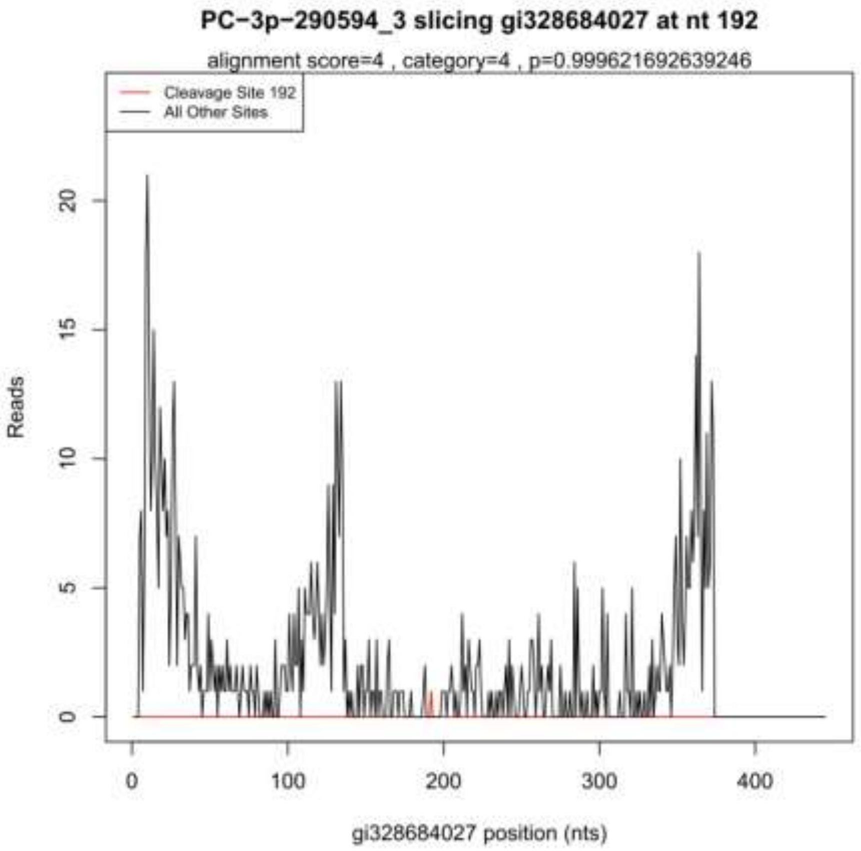
PC−3p−290594_3 slicing gi328684027 at nt 192
alignment score=4 , category=4 , p=0.999621692639246
Cleavage Site 192
All Other Sites
Reads
0
5
10
15
20
0
100
200
300
400
gi328684027 position (nts)

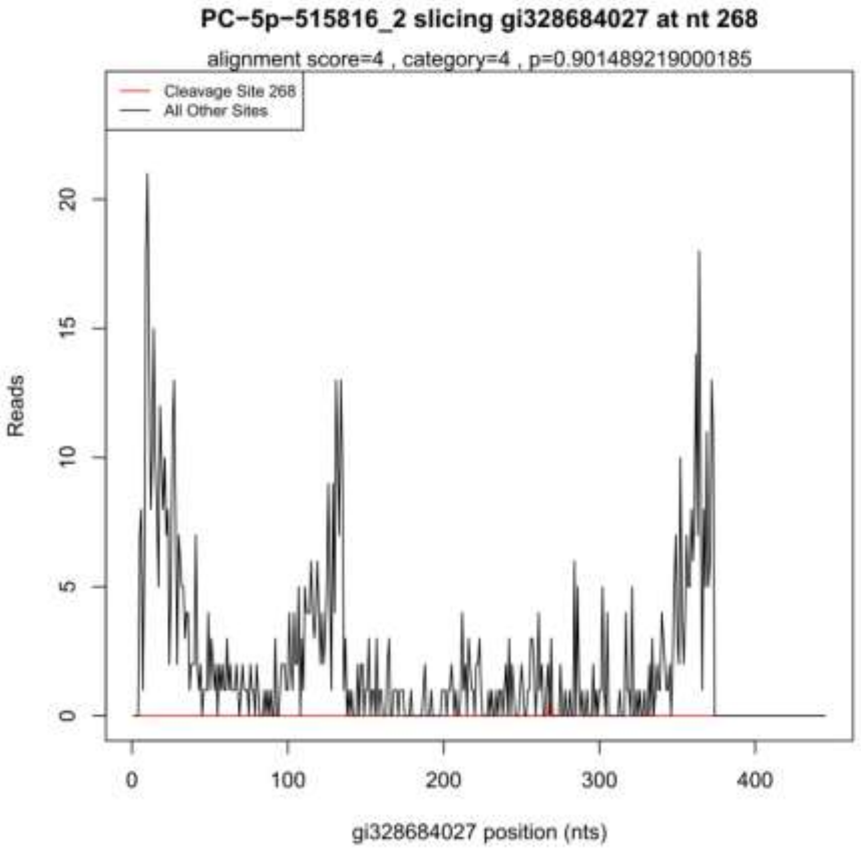
PC−5p−515816_2 slicing gi328684027 at nt 268
alignment score=4 , category=4 , p=0.901489219000185
Cleavage Site 268
All Other Sites
Reads
0
5
10
15
20
0
100
200
300
400
gi328684027 position (nts)

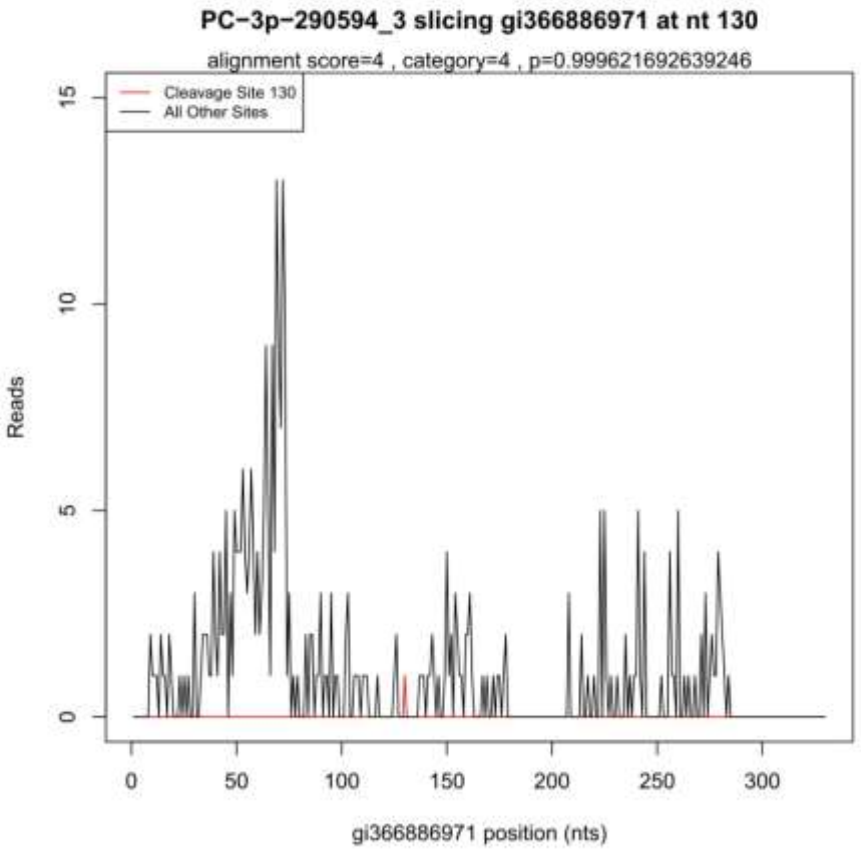
PC−3p−290594_3 slicing gi366886971 at nt 130
alignment score=4 , category=4 , p=0.999621692639246
Cleavage Site 130
All Other Sites
Reads
0
5
10
15
0
50
100
150
200
250
300
gi366886971 position (nts)

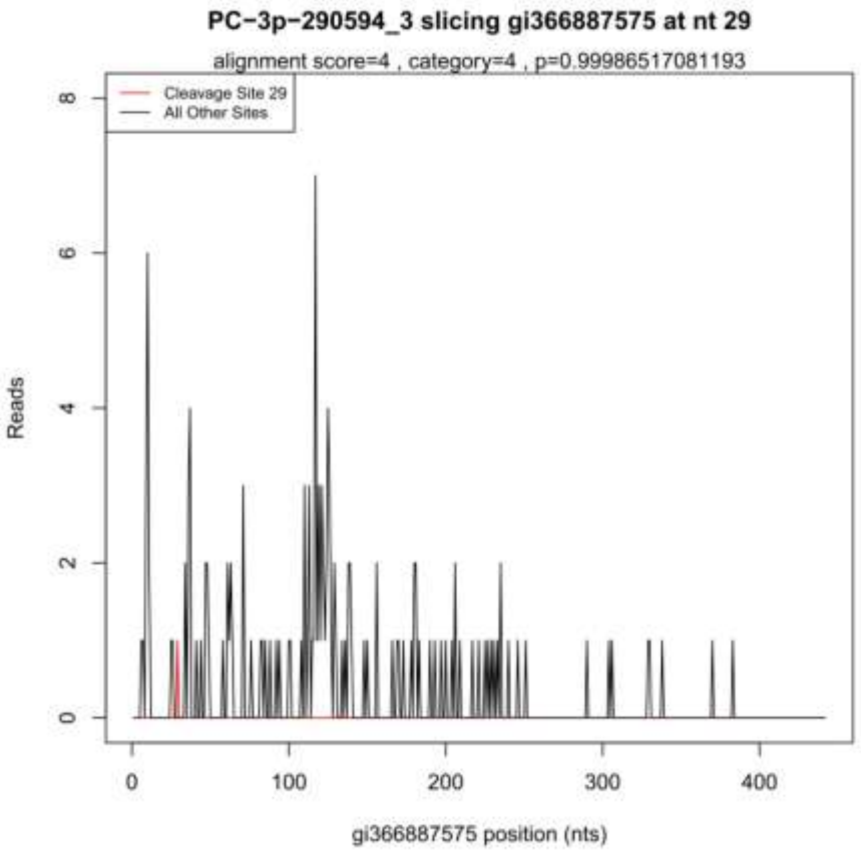
PC−3p−290594_3 slicing gi366887575 at nt 29
alignment score=4 , category=4 , p=0.99986517081193
Cleavage Site 29
All Other Sites
Reads
0
2
4
6
8
0
100
200
300
400
gi366887575 position (nts)

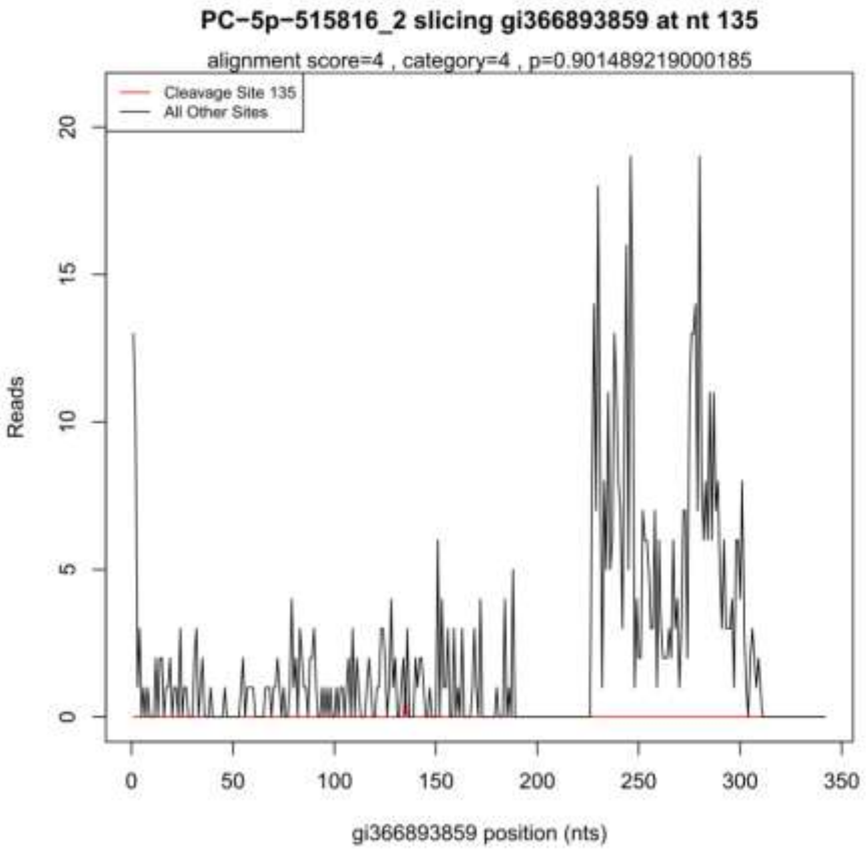
PC−5p−515816_2 slicing gi366893859 at nt 135
alignment score=4 , category=4 , p=0.901489219000185
Cleavage Site 135
All Other Sites
Reads
gi366893859 position (nts)

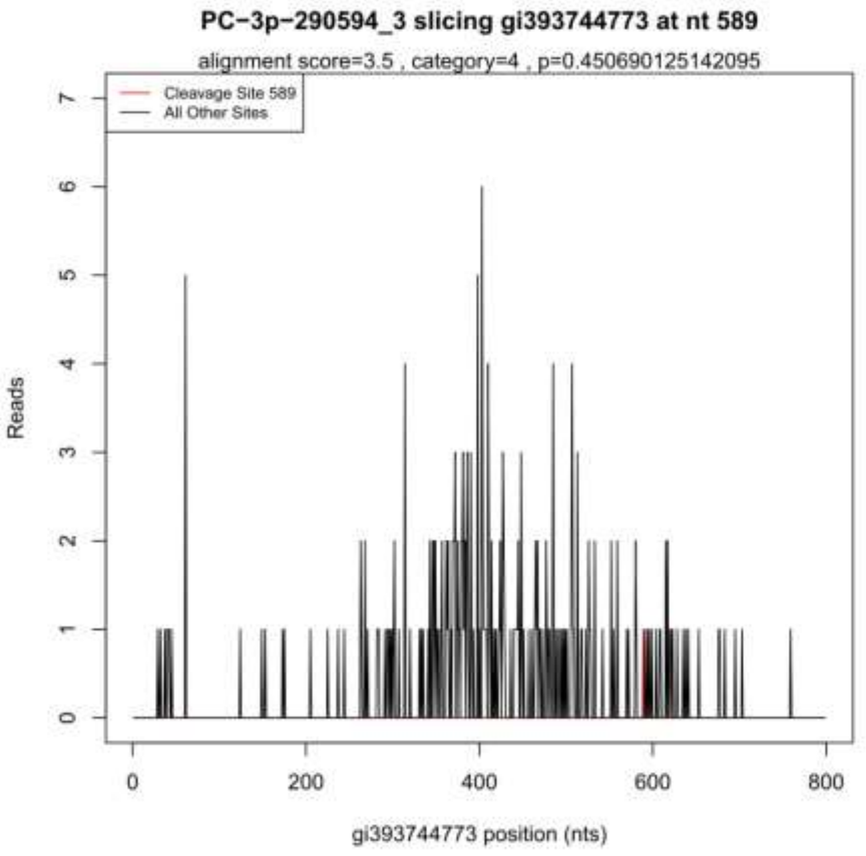
PC−3p−290594_3 slicing gi393744773 at nt 589
alignment score=3.5 , category=4 , p=0.450690125142095
Cleavage Site 589
All Other Sites
Reads
gi393744773 position (nts)

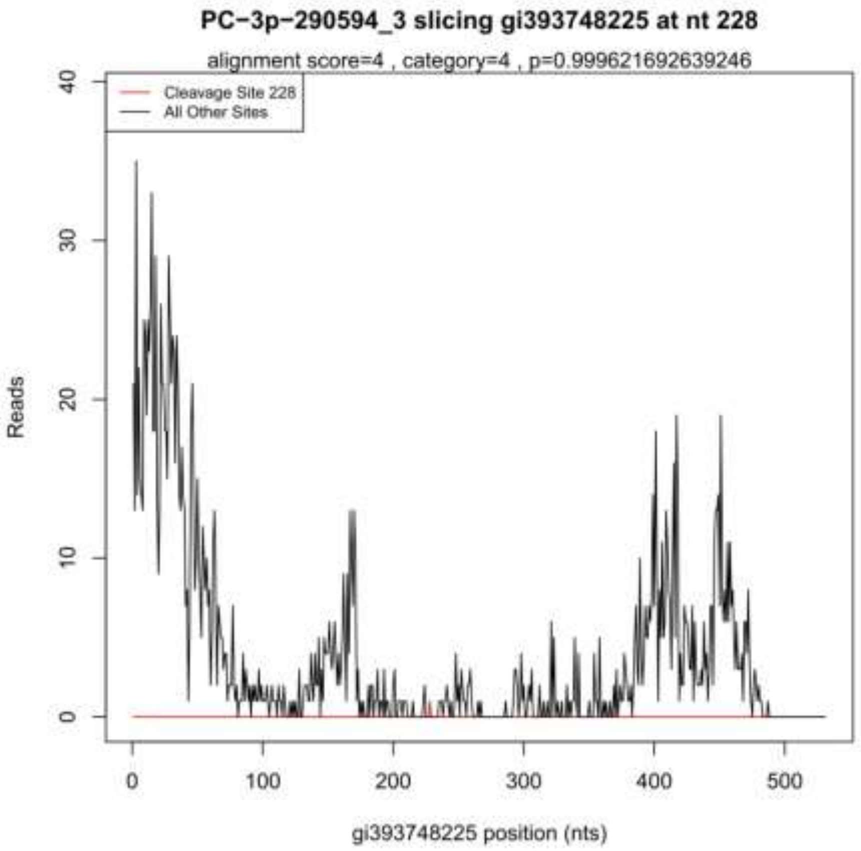
PC−3p−290594_3 slicing gi393748225 at nt 228
alignment score=4 , category=4 , p=0.999621692639246
Cleavage Site 228
All Other Sites
Reads
0
10
20
30
40
0
100
200
300
400
500
gi393748225 position (nts)

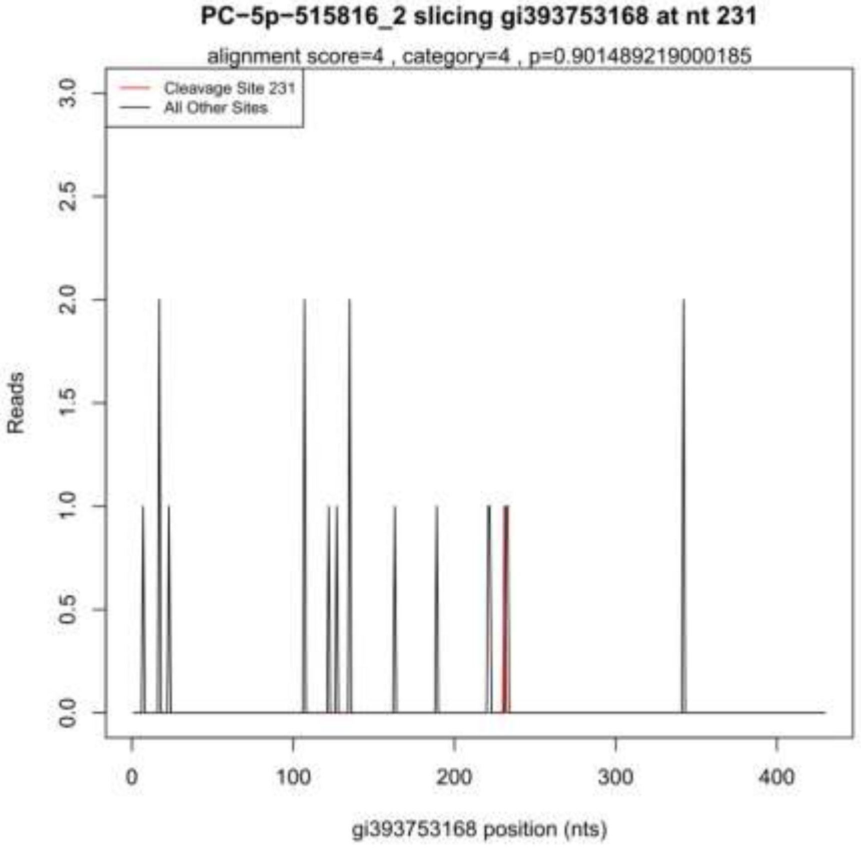
PC−5p−515816_2 slicing gi393753168 at nt 231
alignment score=4 , category=4 , p=0.901489219000185
Cleavage Site 231
All Other Sites
Reads
0.0
0.5
1.0
1.5
2.0
2.5
3.0
0
100
200
300
400
gi393753168 position (nts)

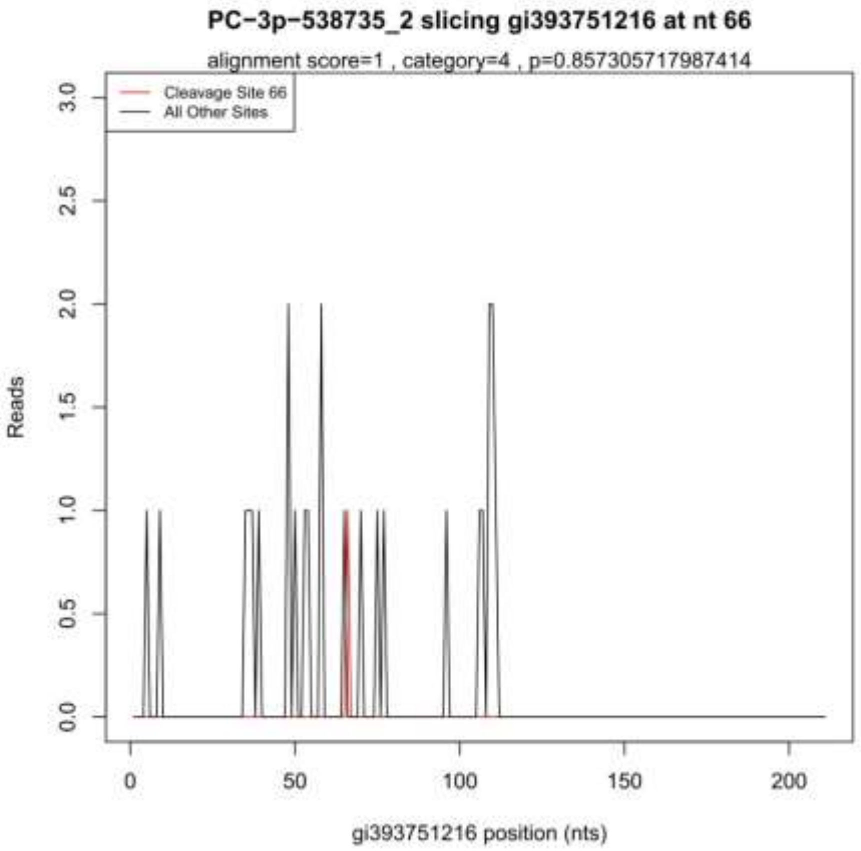
PC−3p−538735_2 slicing gi393751216 at nt 66
alignment score=1 , category=4 , p=0.857305717987414
Cleavage Site 66
All Other Sites
Reads
gi393751216 position (nts)

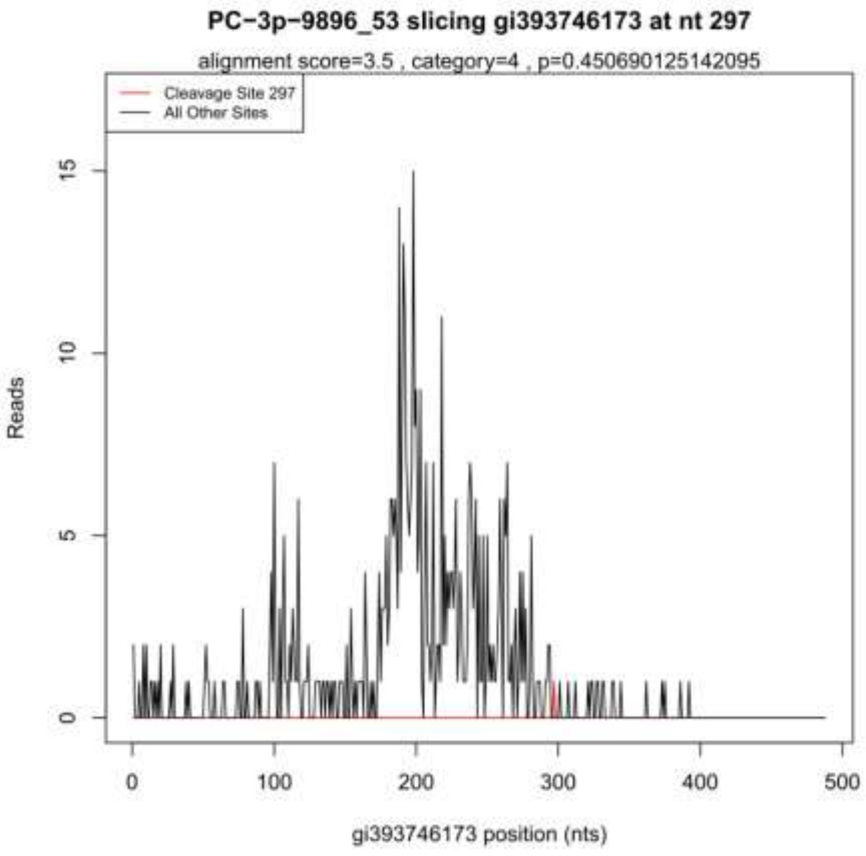
PC−3p−9896_53 slicing gi393746173 at nt 297
alignment score=3.5 , category=4 , p=0.450690125142095
Cleavage Site 297
All Other Sites
Reads
gi393746173 position (nts)

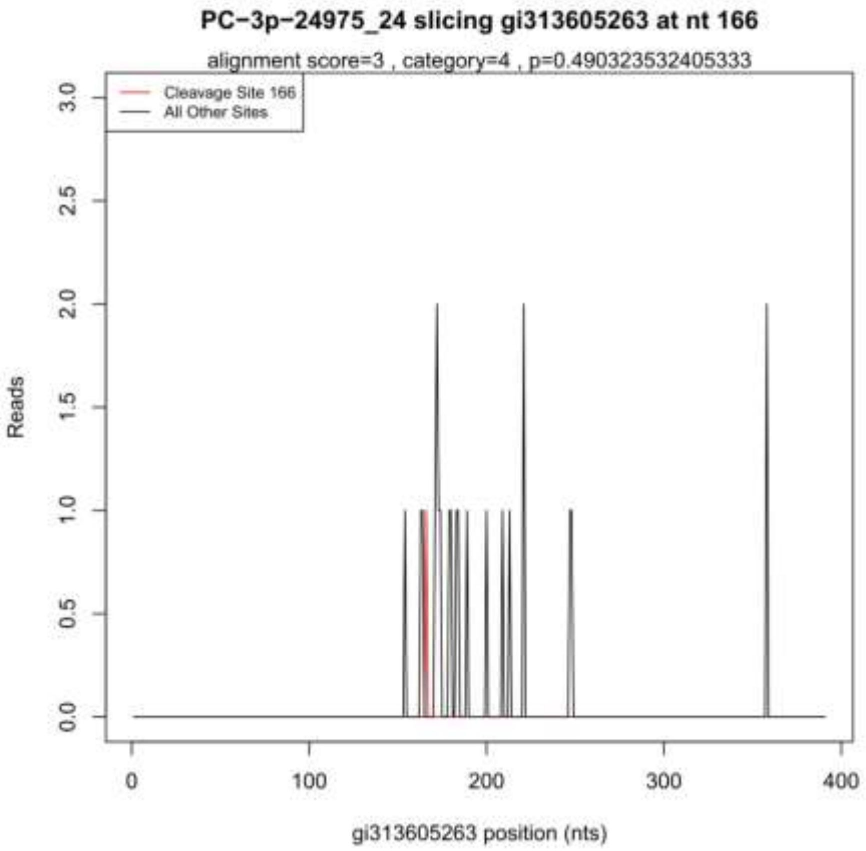
PC−3p−24975_24 slicing gi313605263 at nt 166
alignment score=3 , category=4 , p=0.490323532405333
Cleavage Site 166
All Other Sites
Reads
gi313605263 position (nts)

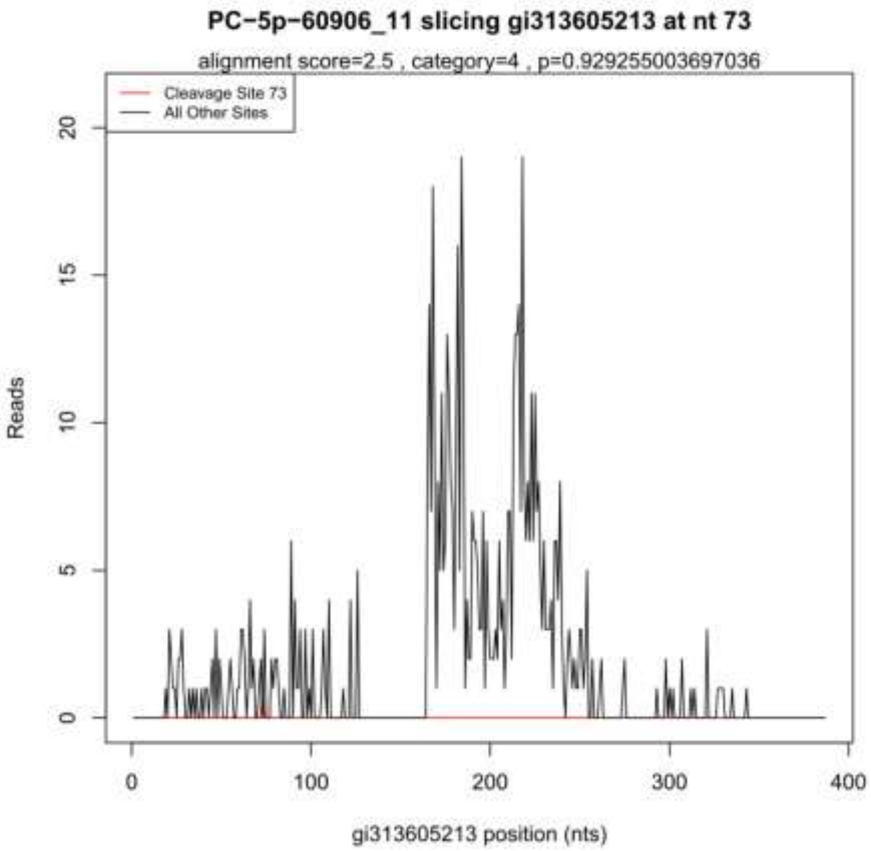
PC−5p−60906_11 slicing gi313605213 at nt 73
alignment score=2.5 , category=4 , p=0.929255003697036
Cleavage Site 73
All Other Sites
Reads
gi313605213 position (nts)

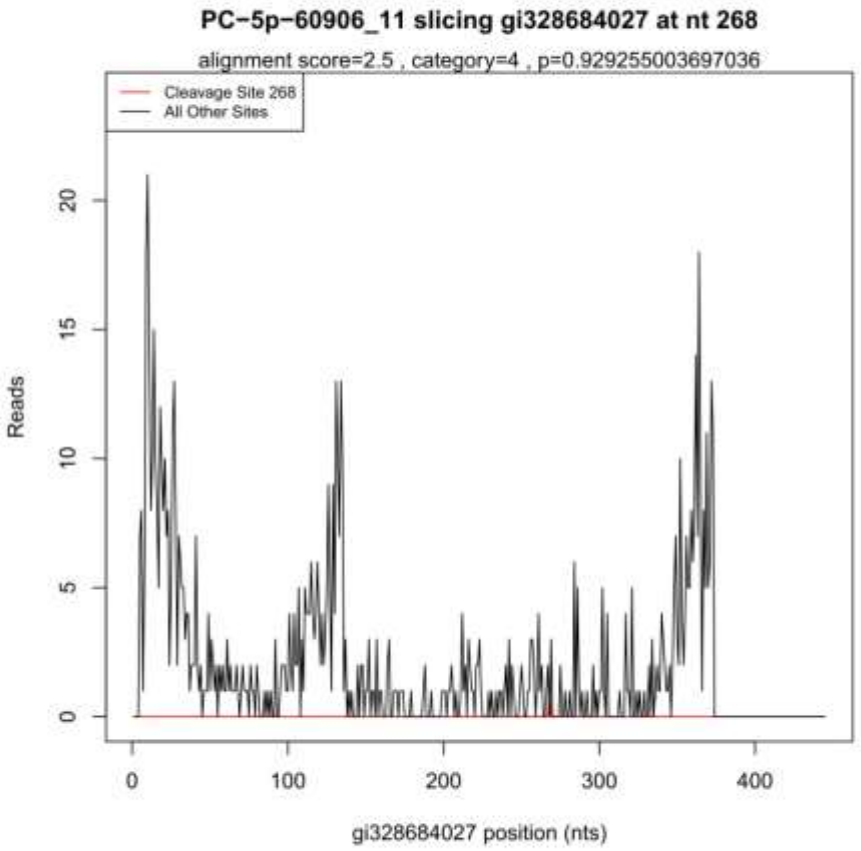
PC−5p−60906_11 slicing gi328684027 at nt 268
alignment score=2.5 , category=4 , p=0.929255003697036
Cleavage Site 268
All Other Sites
Reads
0
5
10
15
20
0
100
200
300
400
gi328684027 position (nts)

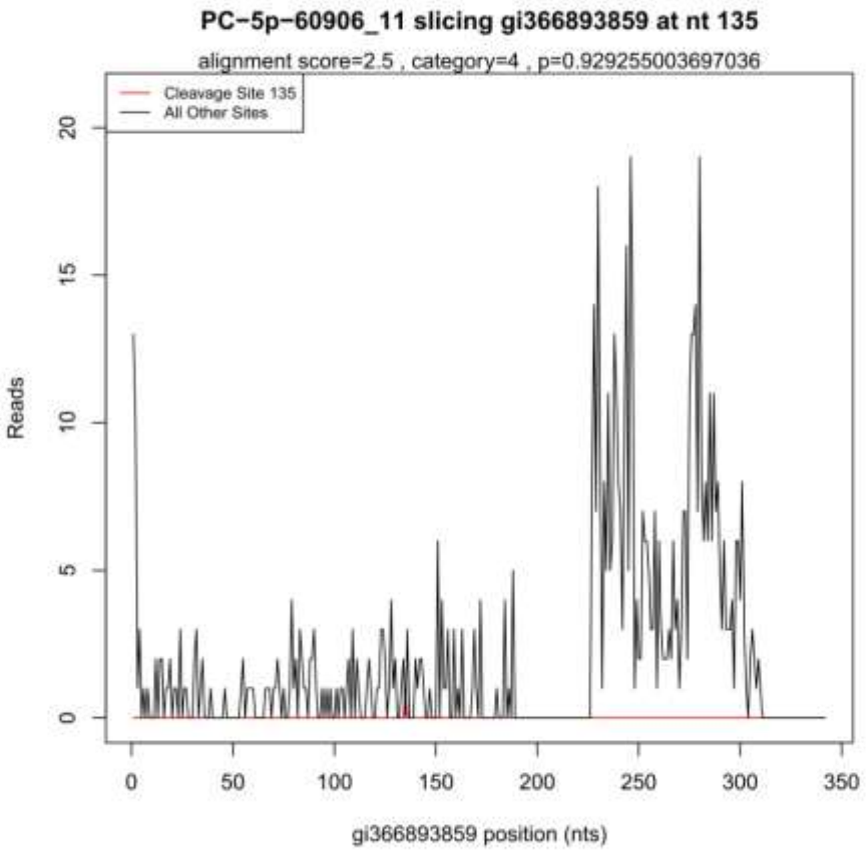
PC−5p−60906_11 slicing gi366893859 at nt 135
alignment score=2.5 , category=4 , p=0.929255003697036
Cleavage Site 135
All Other Sites
Reads
0
5
10
15
20
0
50
100
150
200
250
300
350
gi366893859 position (nts)

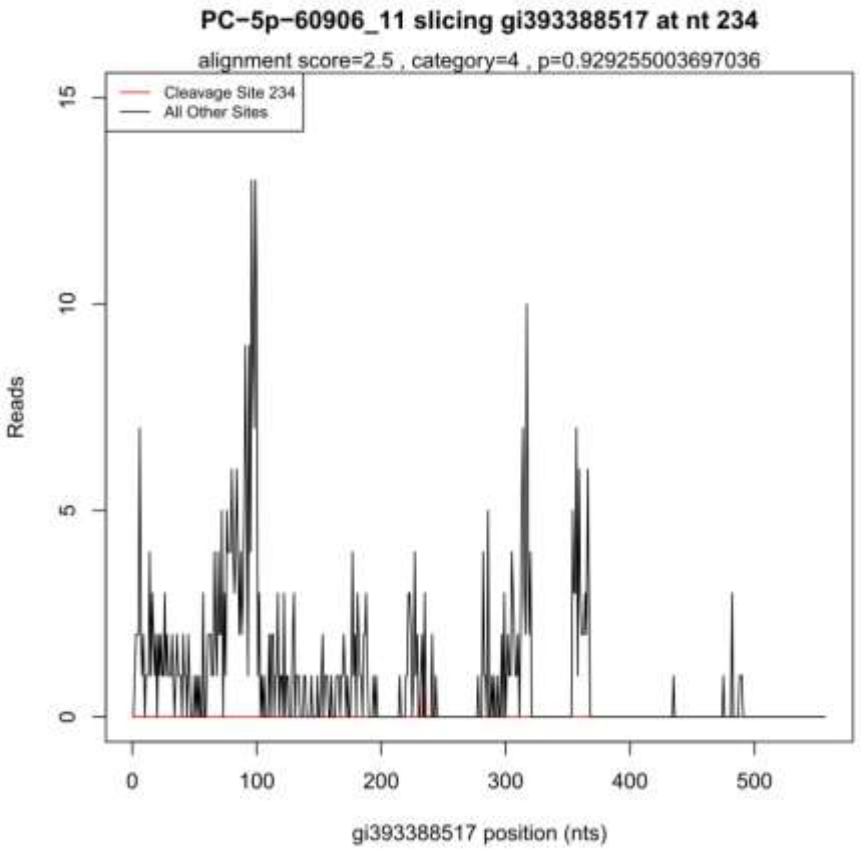
PC−5p−60906_11 slicing gi393388517 at nt 234
alignment score=2.5 , category=4 , p=0.929255003697036
Cleavage Site 234
All Other Sites
Reads
gi393388517 position (nts)

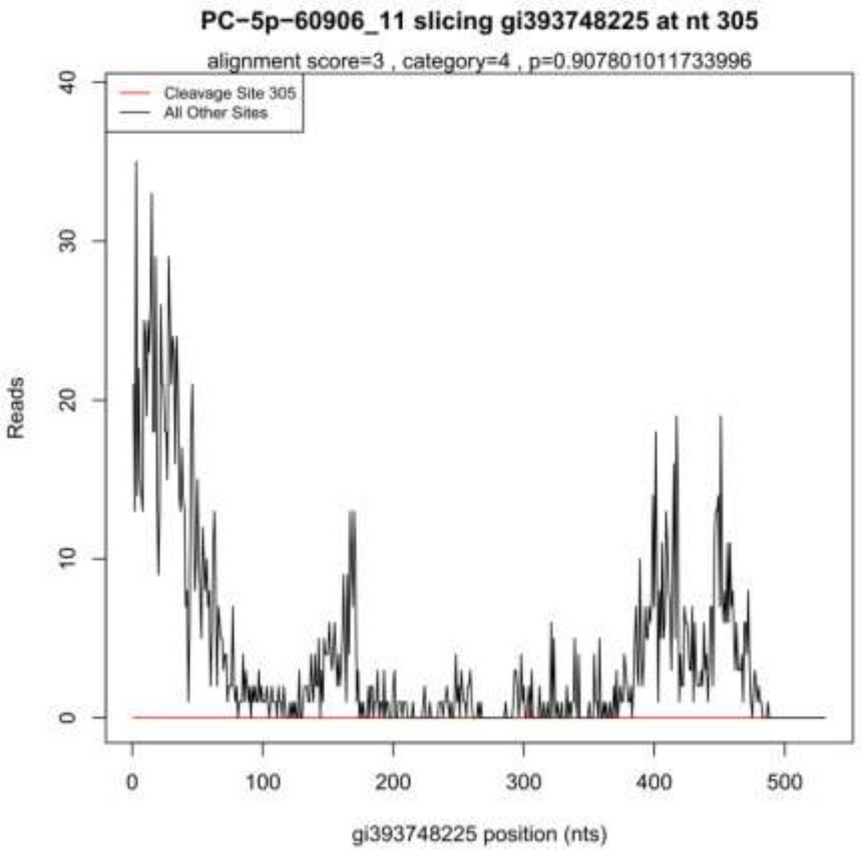
PC−5p−60906_11 slicing gi393748225 at nt 305
alignment score=3 , category=4 , p=0.907801011733996
Cleavage Site 305
All Other Sites
Reads
gi393748225 position (nts)

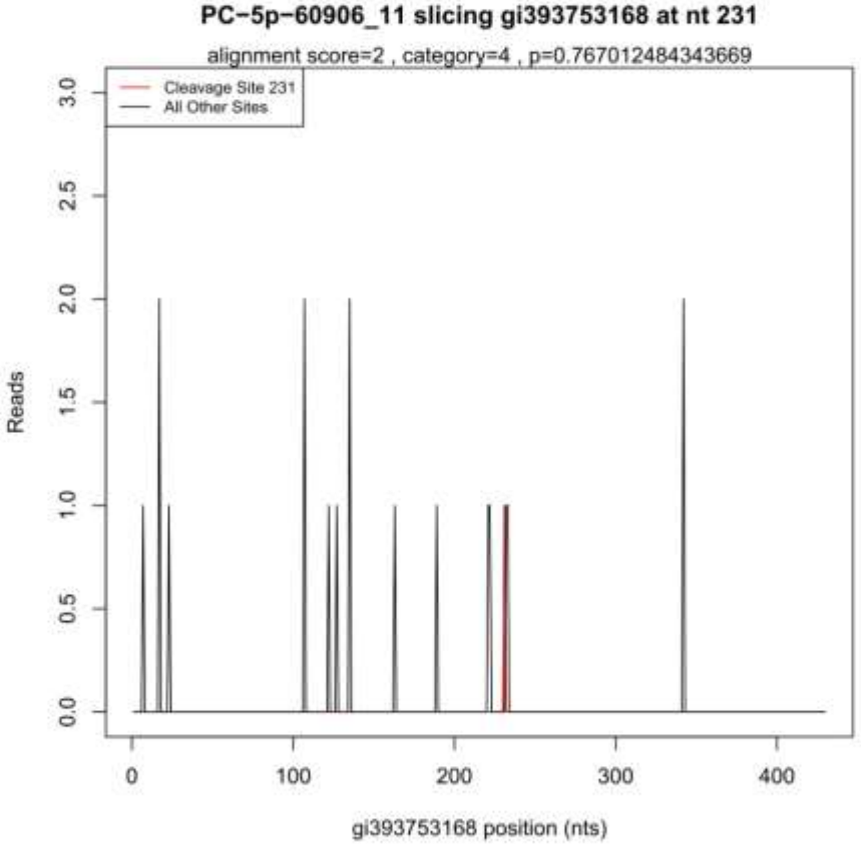
PC−5p−60906_11 slicing gi393753168 at nt 231
alignment score=2 , category=4 , p=0.767012484343669
Cleavage Site 231
All Other Sites
Reads
gi393753168 position (nts)

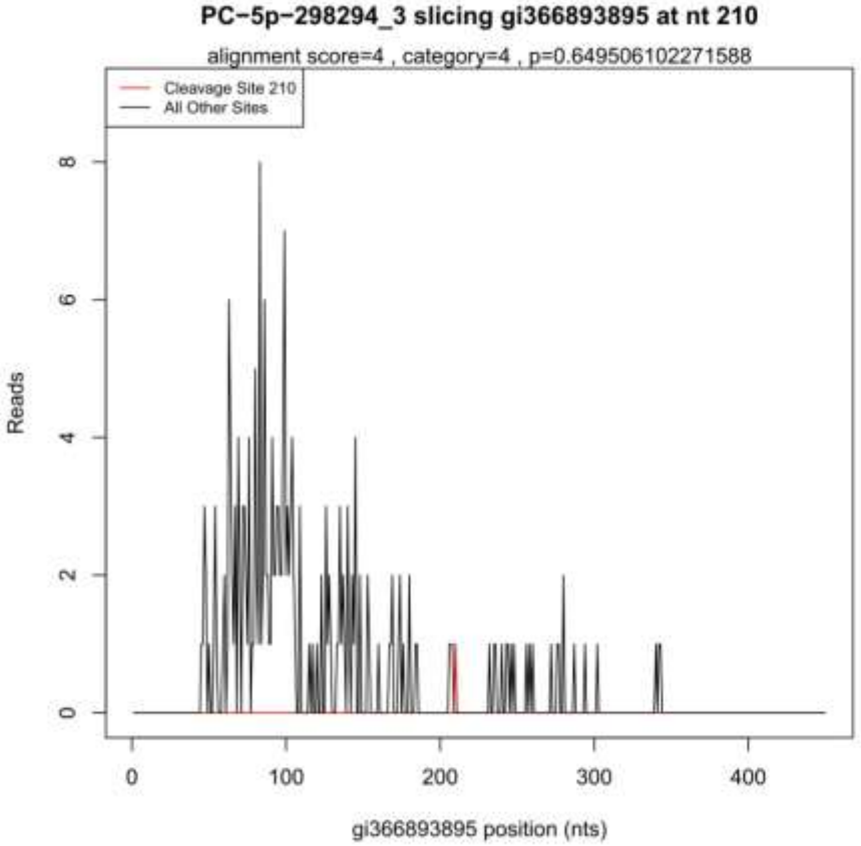
PC−5p−298294_3 slicing gi366893895 at nt 210
alignment score=4 , category=4 , p=0.649506102271588
Cleavage Site 210
All Other Sites
Reads
gi366893895 position (nts)

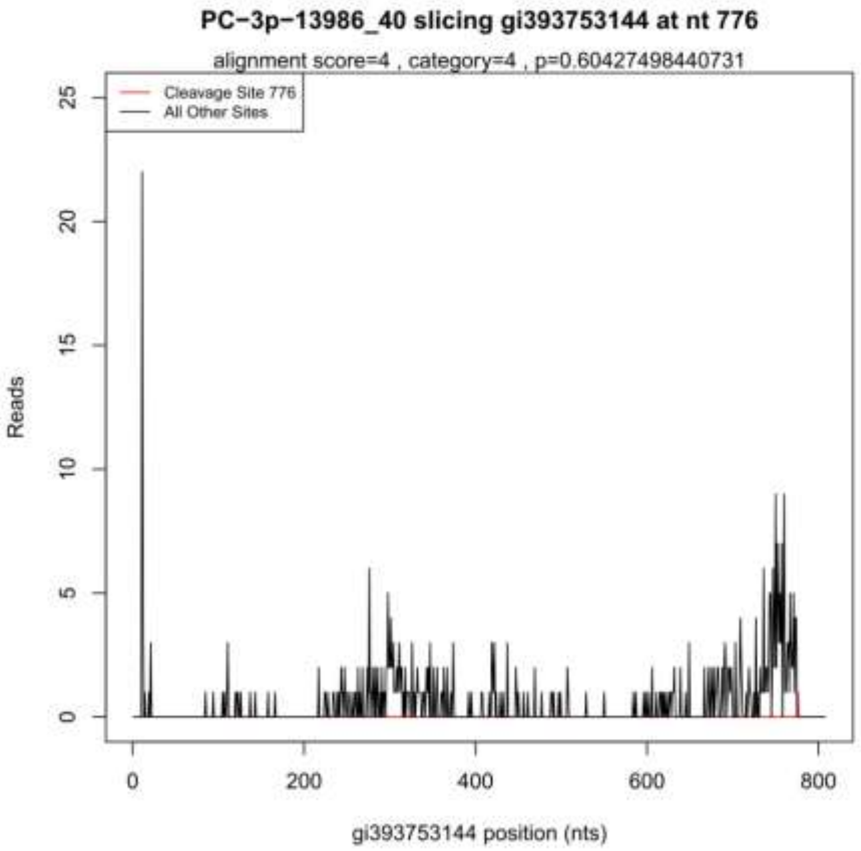
PC−3p−13986_40 slicing gi393753144 at nt 776
alignment score=4 , category=4 , p=0.60427498440731
Cleavage Site 776
All Other Sites
Reads
gi393753144 position (nts)

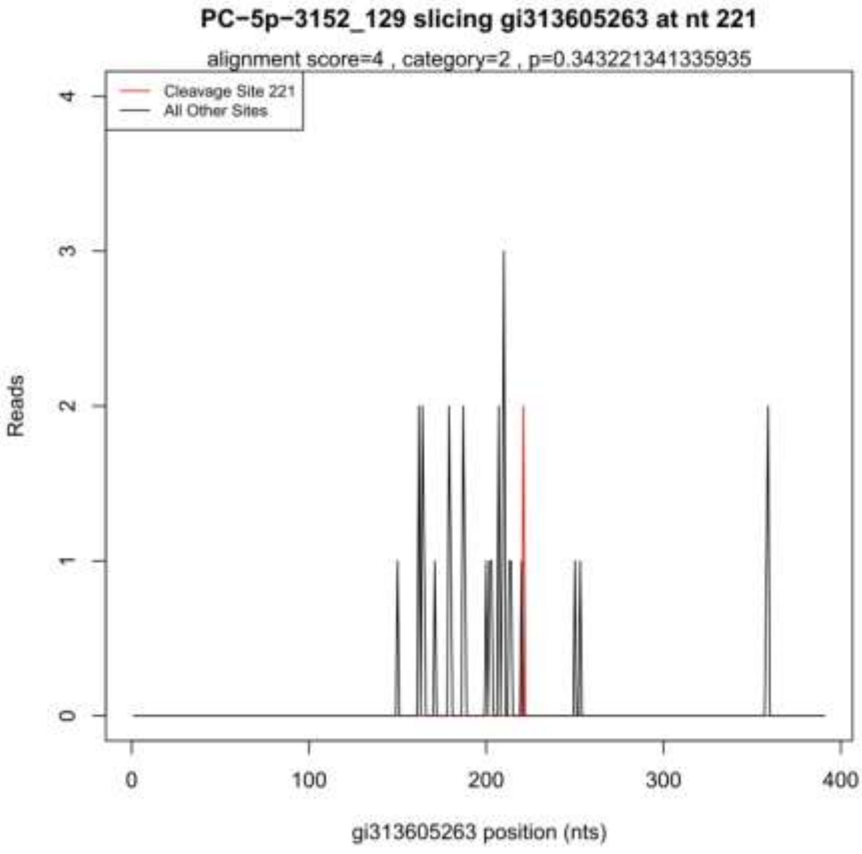
PC−5p−3152_129 slicing gi313605263 at nt 221
alignment score=4 , category=2 , p=0.343221341335935
Cleavage Site 221
All Other Sites
Reads
gi313605263 position (nts)

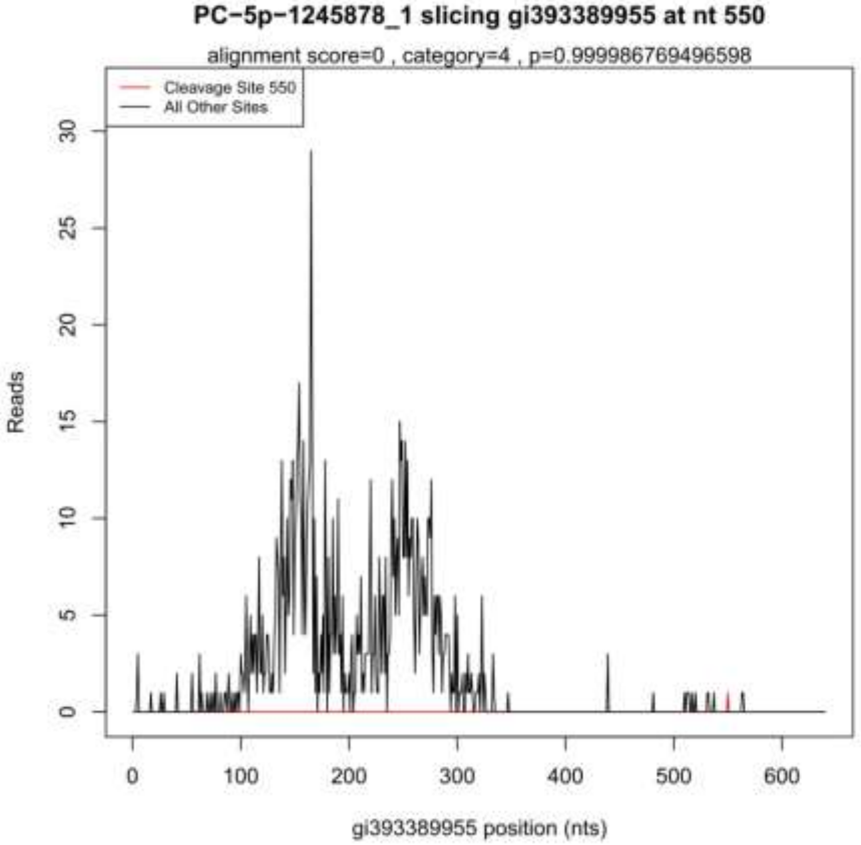
PC−5p−1245878_1 slicing gi393389955 at nt 550
alignment score=0 , category=4 , p=0.999986769496598
Cleavage Site 550
All Other Sites
Reads
gi393389955 position (nts)

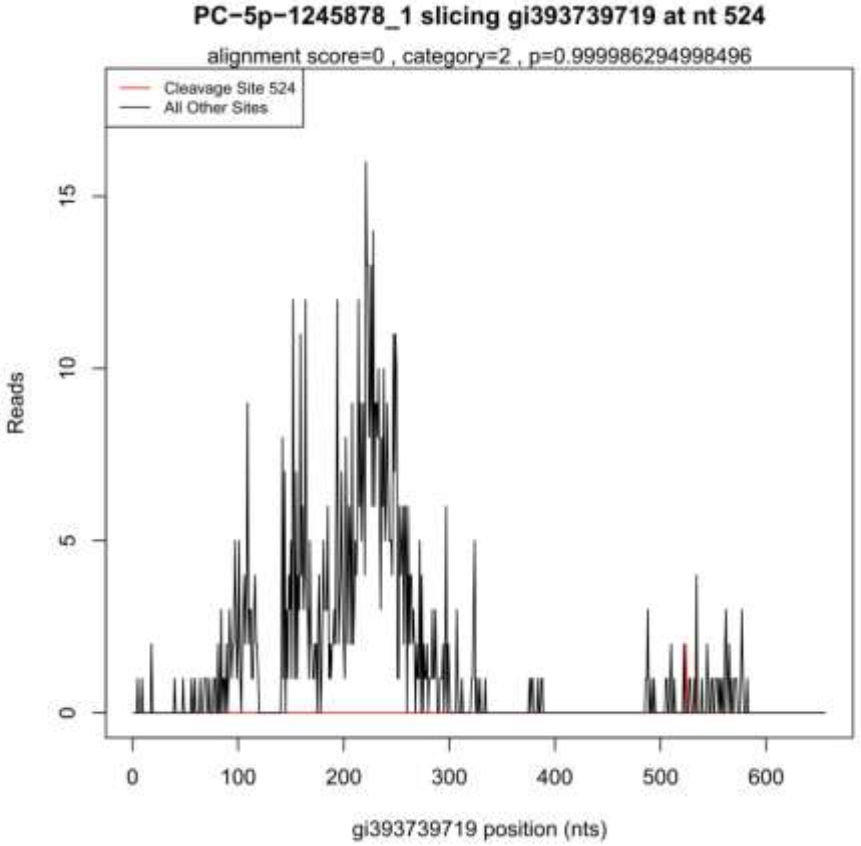
PC−5p−1245878_1 slicing gi393739719 at nt 524
alignment score=0 , category=2 , p=0.999986294998496
Cleavage Site 524
All Other Sites
Reads
gi393739719 position (nts)

PC−5p−1245878_1 slicing gi393744366 at nt 526

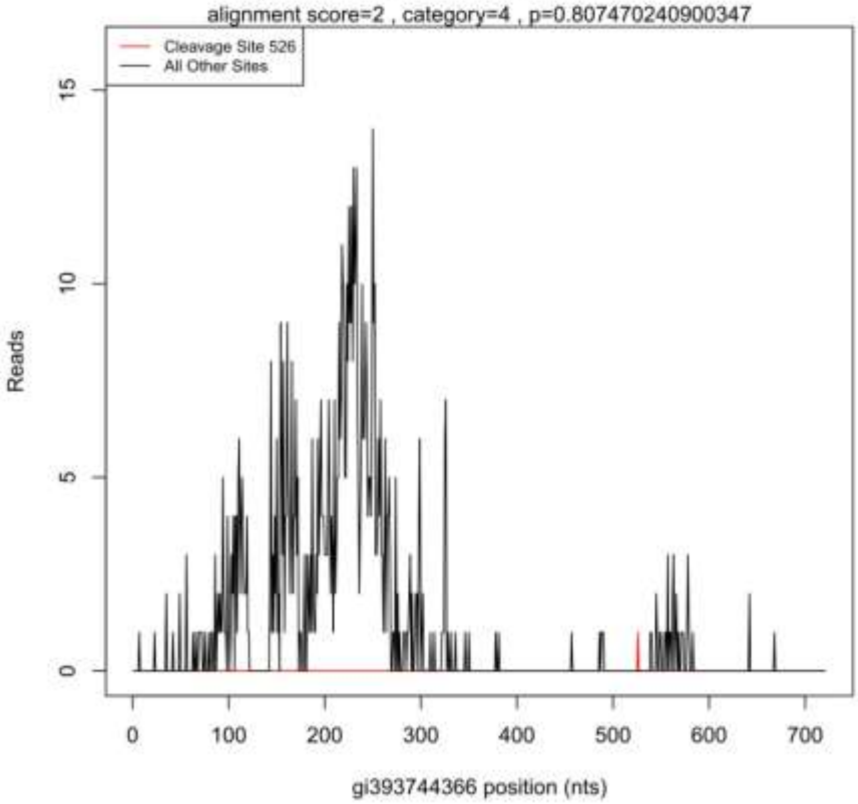

PC−5p−1245878_1 slicing gi393748367 at nt 521

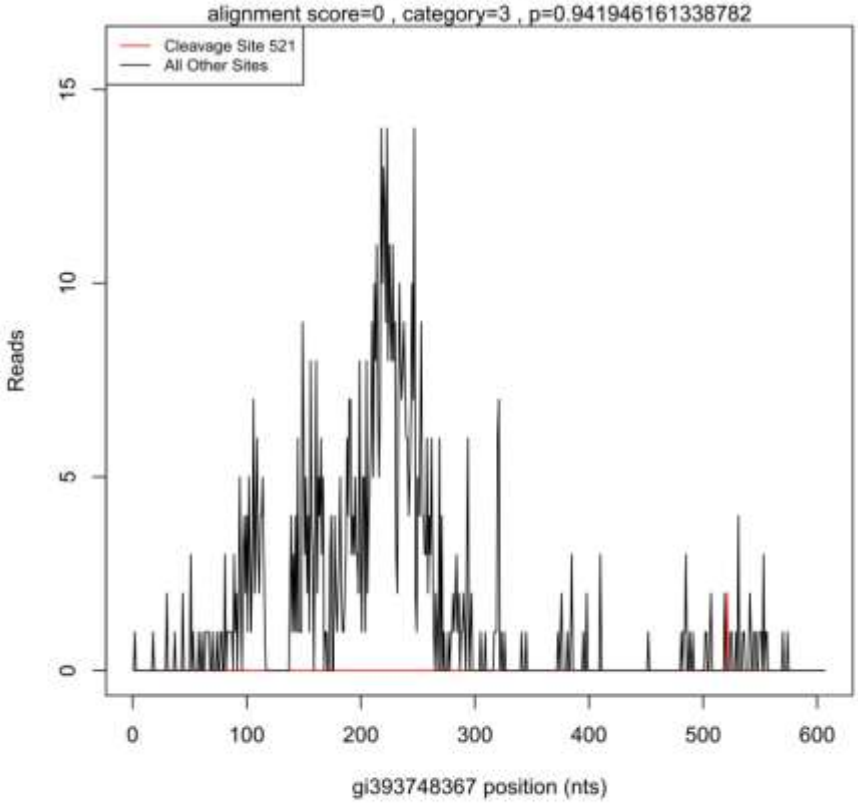

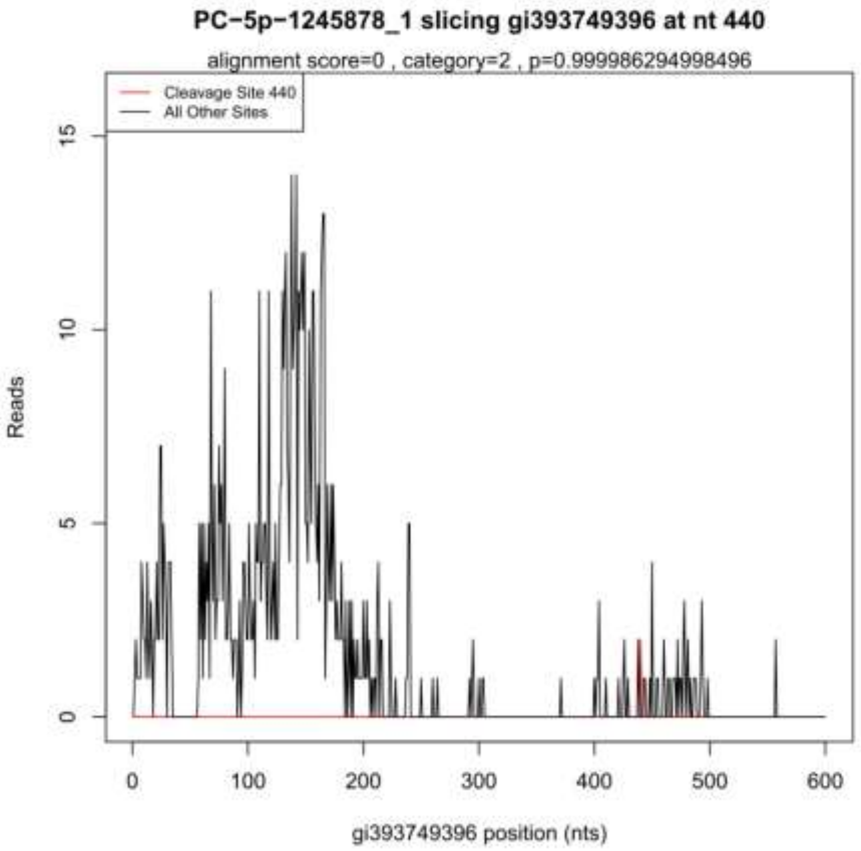

PC−5p−1245878_1 slicing gi393749396 at nt 440
alignment score=0 , category=2 , p=0.999986294998496
Cleavage Site 440
All Other Sites
Reads
0
5
10
15
0
100
200
300
400
500
600
gi393749396 position (nts)

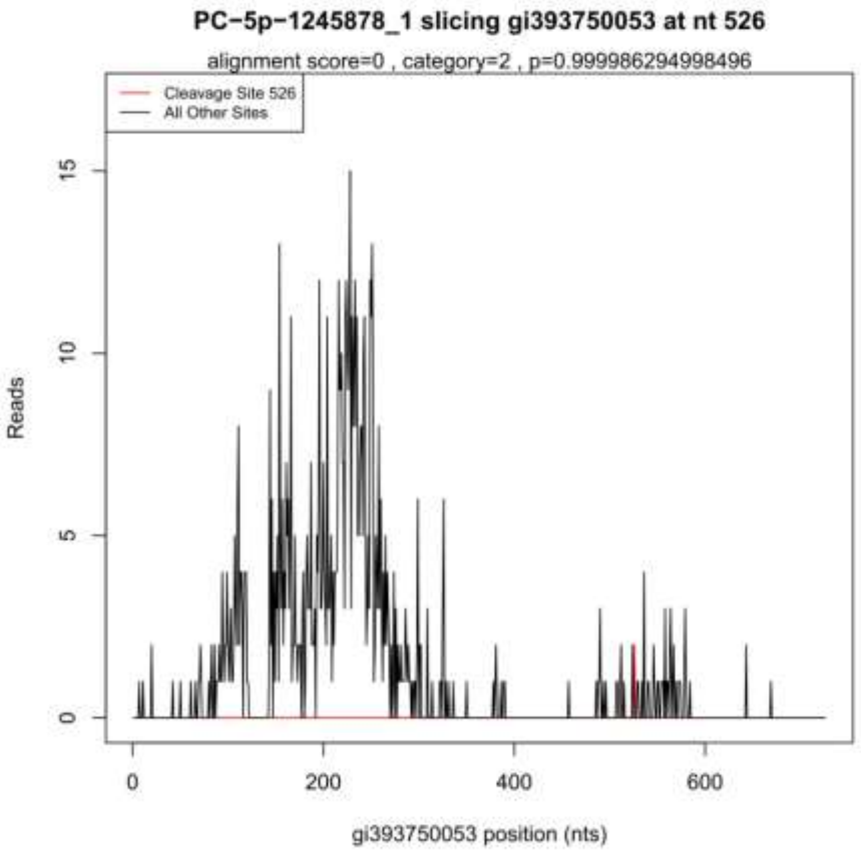

PC−5p−1245878_1 slicing gi393750053 at nt 526
alignment score=0 , category=2 , p=0.999986294998496
Cleavage Site 526
All Other Sites
Reads
0
5
10
15
0
200
400
600
gi393750053 position (nts)

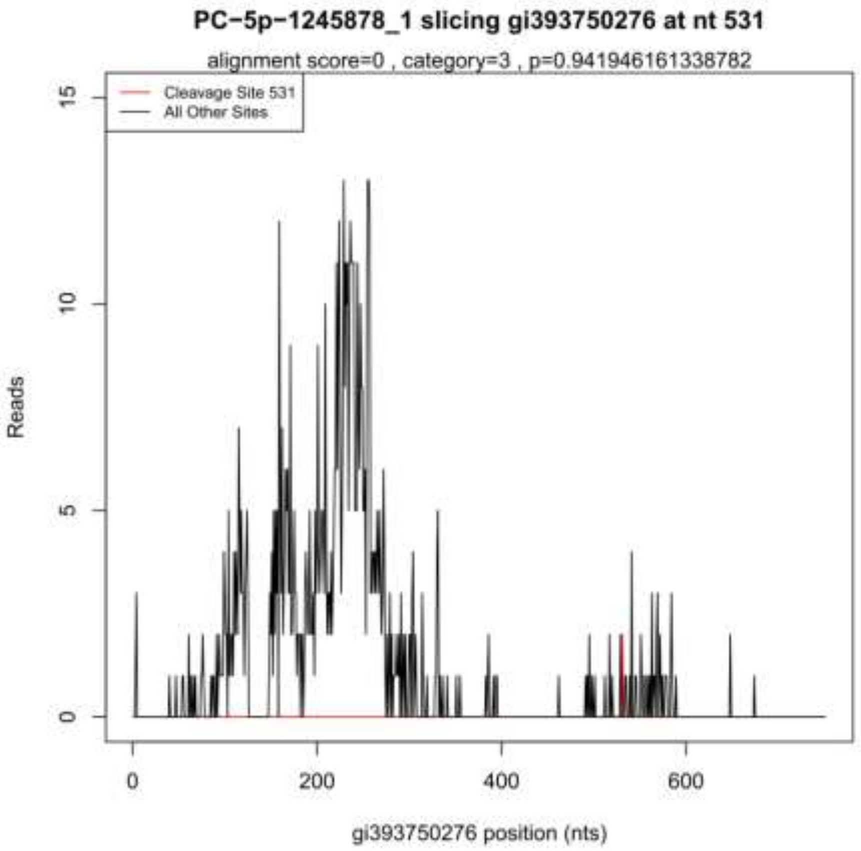
PC−5p−1245878_1 slicing gi393750276 at nt 531
alignment score=0 , category=3 , p=0.941946161338782
Cleavage Site 531
All Other Sites
Reads
0
5
10
15
0
200
400
600
gi393750276 position (nts)

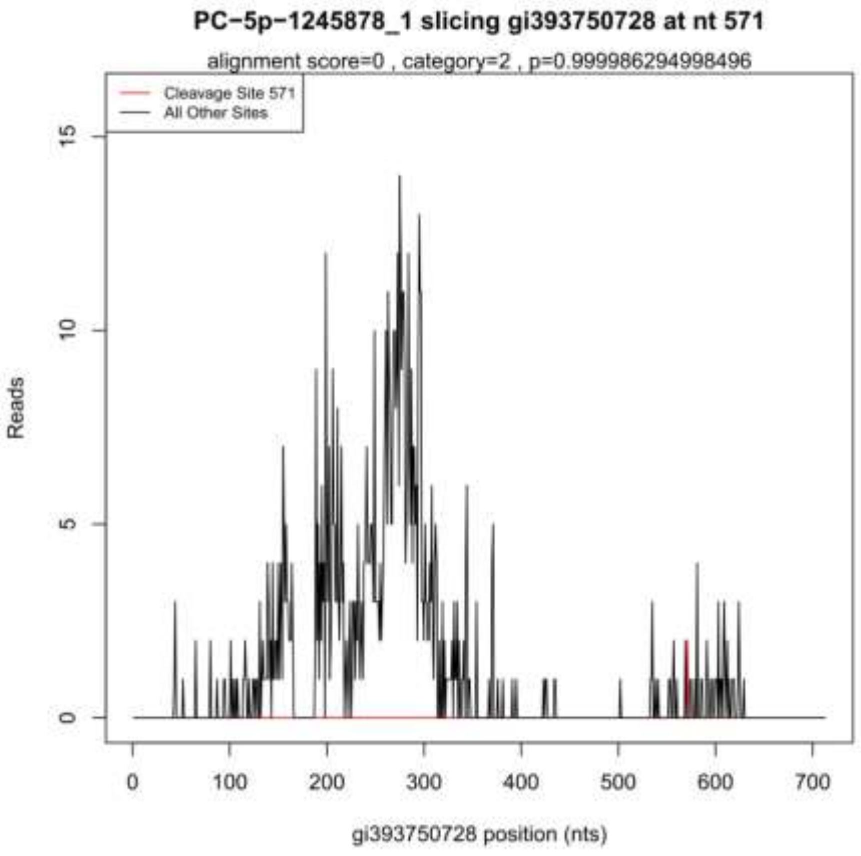
PC−5p−1245878_1 slicing gi393750728 at nt 571
alignment score=0 , category=2 , p=0.999986294998496
Cleavage Site 571
All Other Sites
Reads
0
5
10
15
0
100
200
300
400
500
600
700
gi393750728 position (nts)

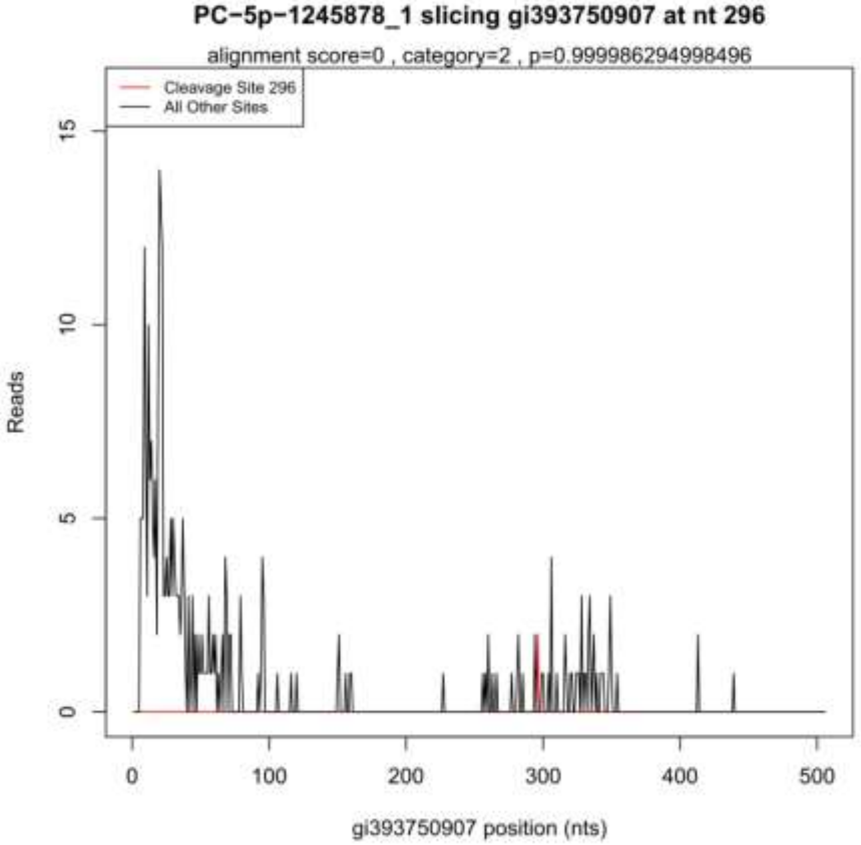
PC−5p−1245878_1 slicing gi393750907 at nt 296
alignment score=0 , category=2 , p=0.999986294998496
Cleavage Site 296
All Other Sites
Reads
0
5
10
15
0
100
200
300
400
500
gi393750907 position (nts)

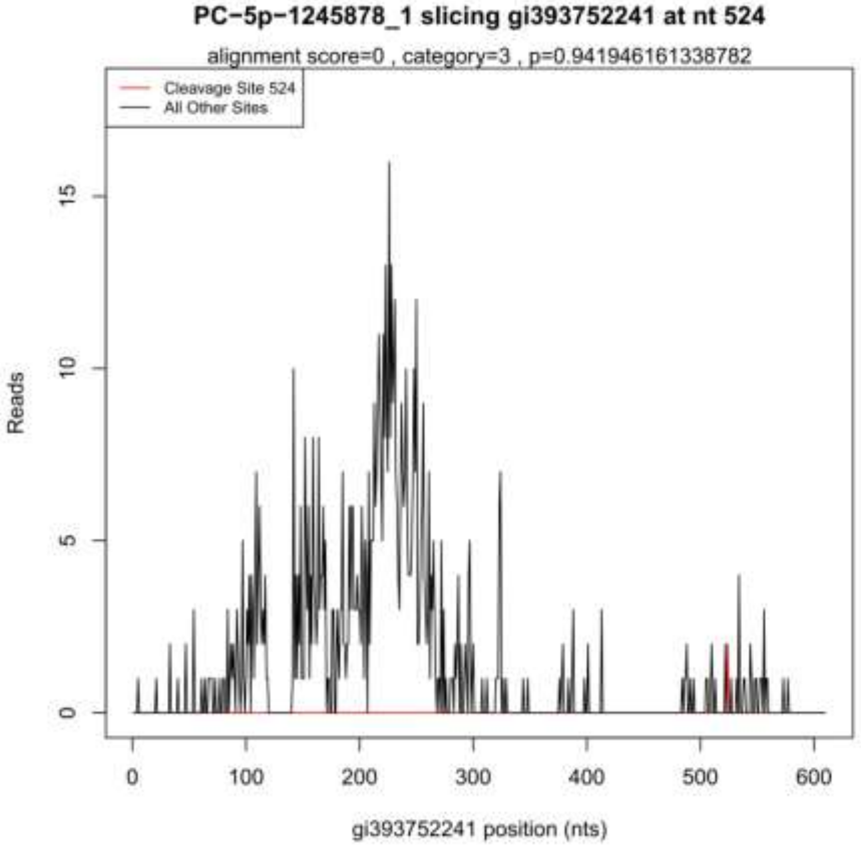
PC−5p−1245878_1 slicing gi393752241 at nt 524
alignment score=0 , category=3 , p=0.941946161338782
Cleavage Site 524
All Other Sites
Reads
0
5
10
15
0
100
200
300
400
500
600
gi393752241 position (nts)

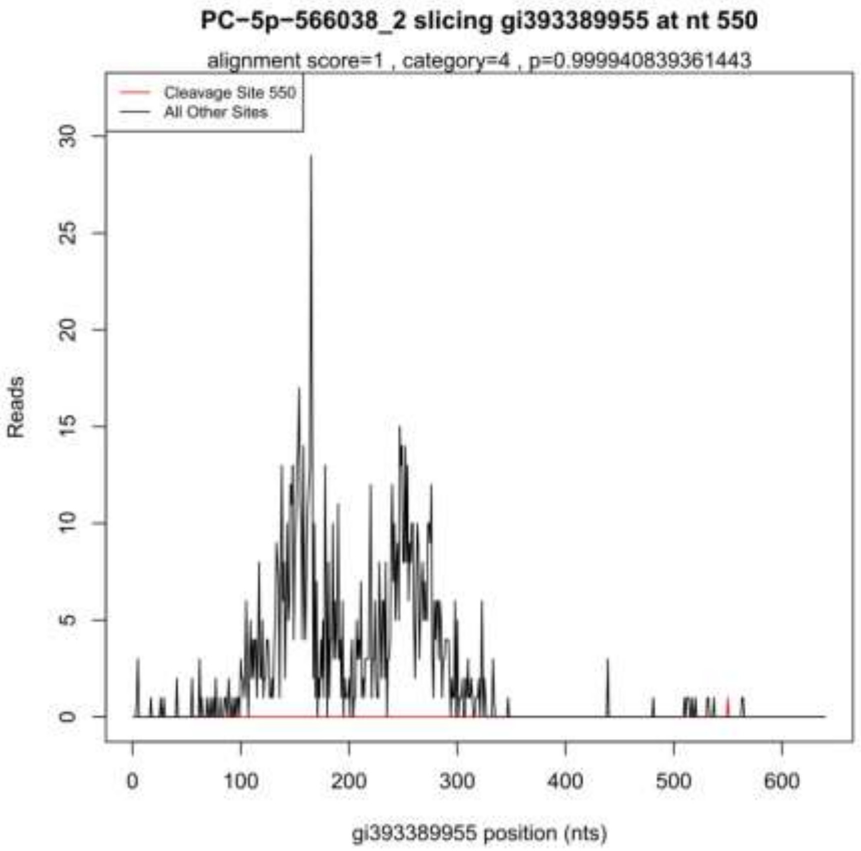
PC−5p−566038_2 slicing gi393389955 at nt 550
alignment score=1 , category=4 , p=0.999940839361443
Cleavage Site 550
All Other Sites
Reads
gi393389955 position (nts)

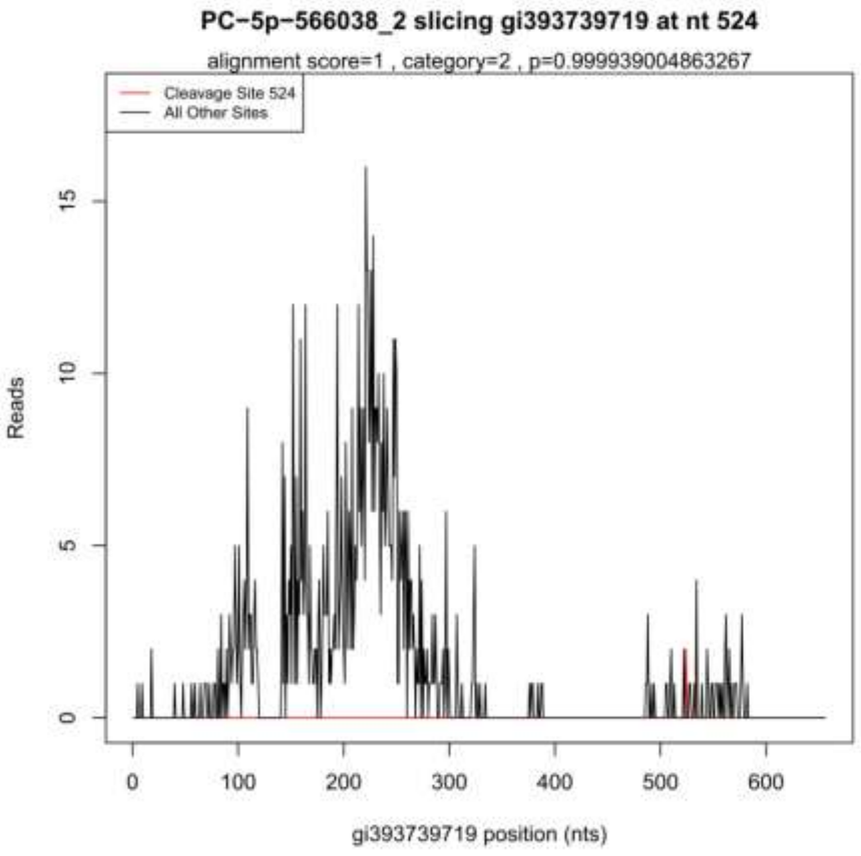
PC−5p−566038_2 slicing gi393739719 at nt 524
alignment score=1 , category=2 , p=0.999939004863267
Cleavage Site 524
All Other Sites
Reads
gi393739719 position (nts)

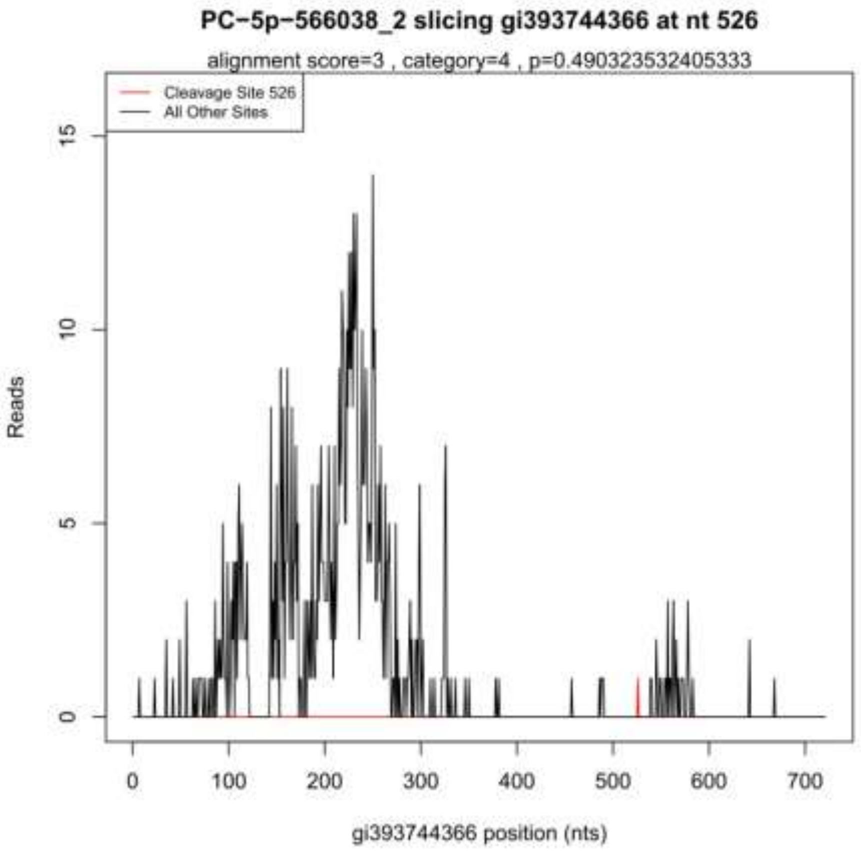

PC-5p-566038_2 slicing gi393744366 at nt 526
alignment score=3 , category=4 , p=0.490323532405333
Cleavage Site 526
All Other Sites
Reads
gi393744366 position (nts)

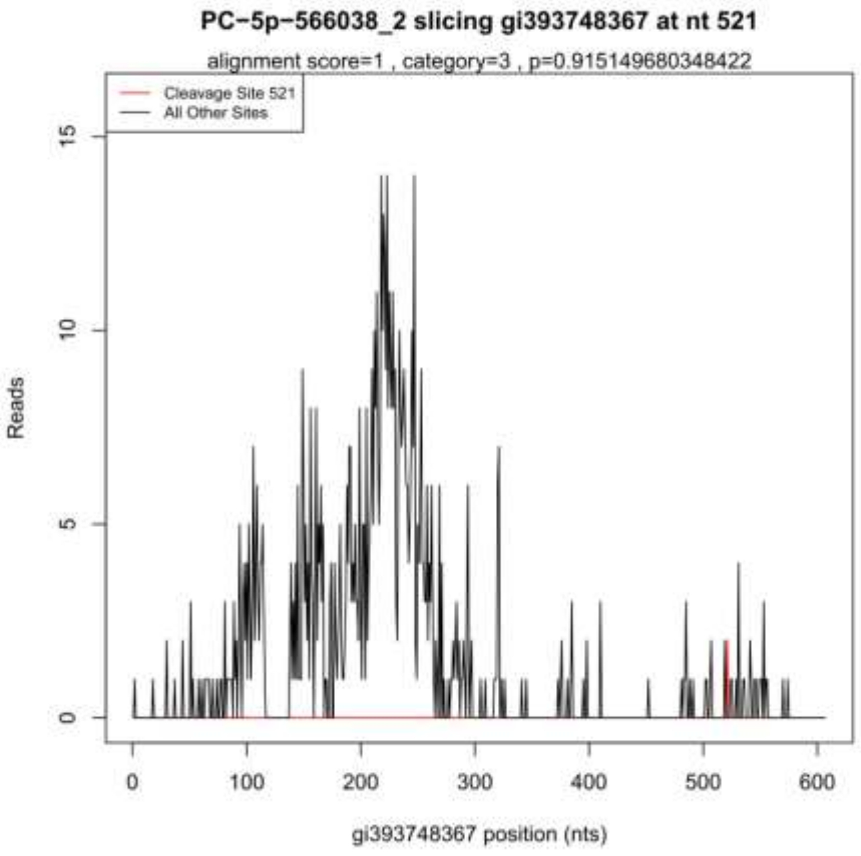

PC-5p-566038_2 slicing gi393748367 at nt 521
alignment score=1 , category=3 , p=0.915149680348422
Cleavage Site 521
All Other Sites
Reads
gi393748367 position (nts)

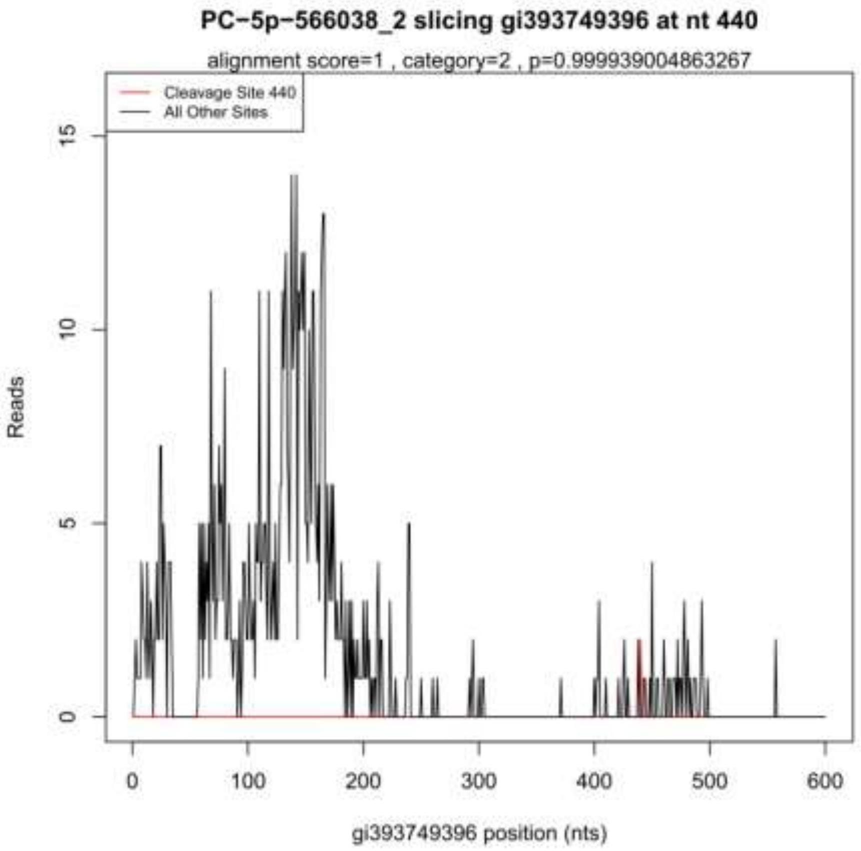
PC−5p−566038_2 slicing gi393749396 at nt 440
alignment score=1 , category=2 , p=0.999939004863267
Cleavage Site 440
All Other Sites
Reads
0
5
10
15
0
100
200
300
400
500
600
gi393749396 position (nts)

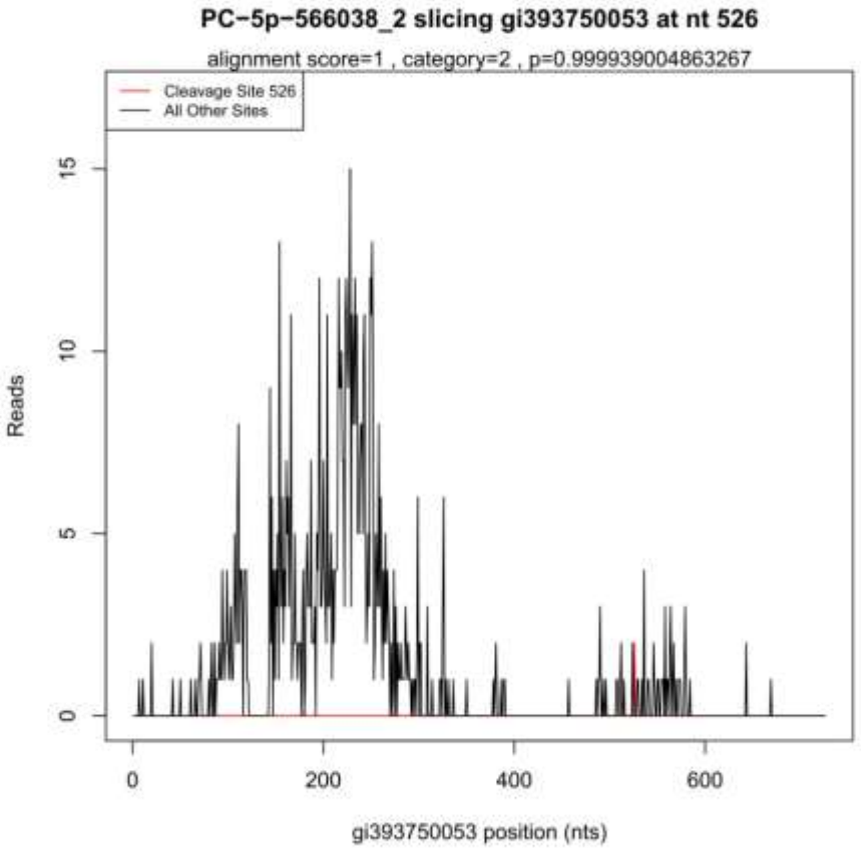
PC−5p−566038_2 slicing gi393750053 at nt 526
alignment score=1 , category=2 , p=0.999939004863267
Cleavage Site 526
All Other Sites
Reads
0
5
10
15
0
200
400
600
gi393750053 position (nts)

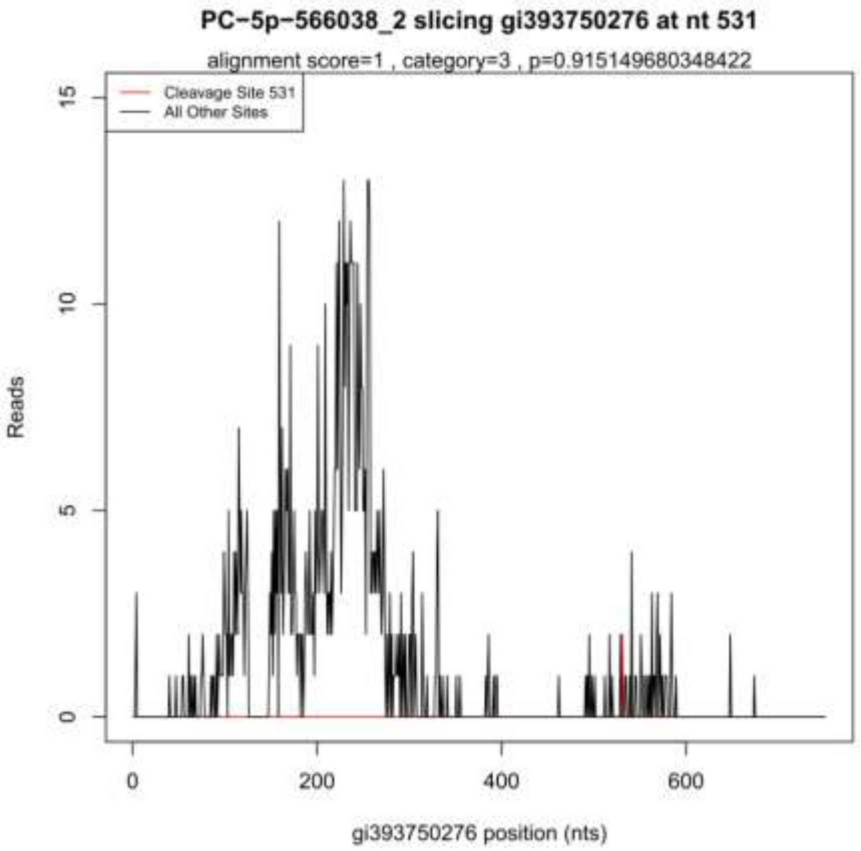
PC−5p−566038_2 slicing gi393750276 at nt 531
alignment score=1 , category=3 , p=0.915149680348422
Cleavage Site 531
All Other Sites
Reads
0
5
10
15
0
200
400
600
gi393750276 position (nts)

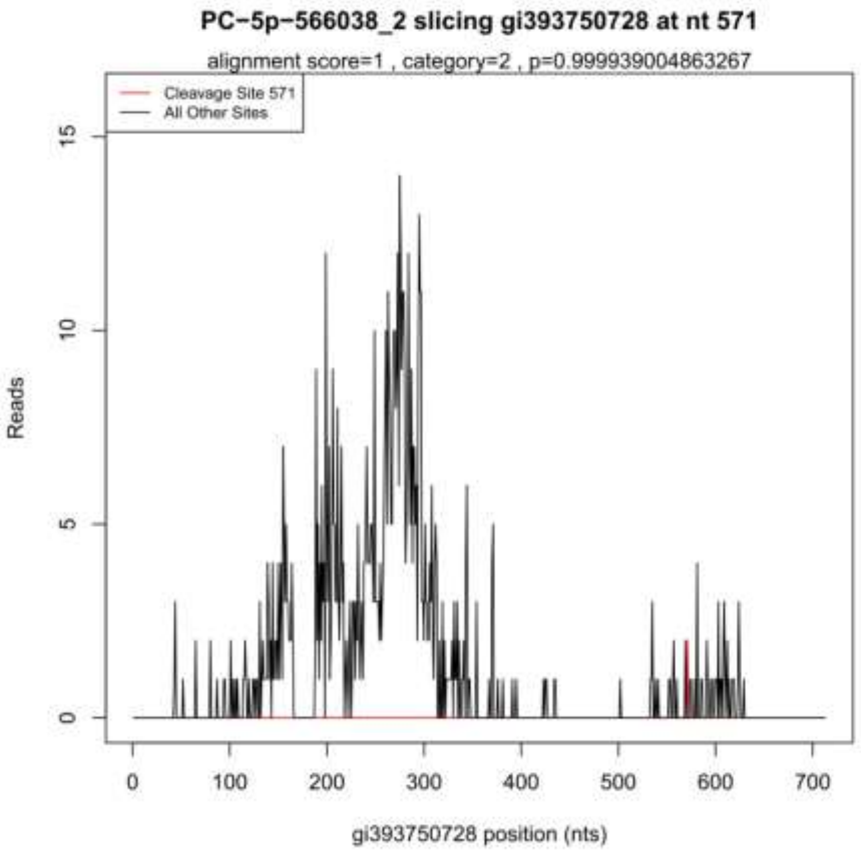
PC−5p−566038_2 slicing gi393750728 at nt 571
alignment score=1 , category=2 , p=0.999939004863267
Cleavage Site 571
All Other Sites
Reads
0
5
10
15
0
100
200
300
400
500
600
700
gi393750728 position (nts)

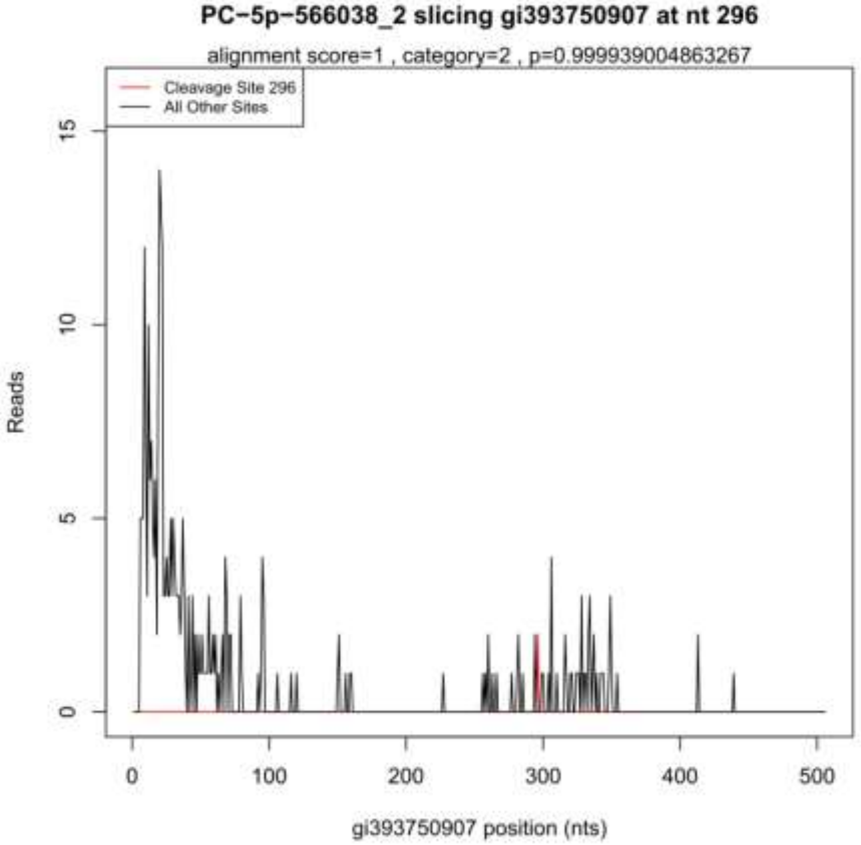
PC−5p−566038_2 slicing gi393750907 at nt 296
alignment score=1 , category=2 , p=0.999939004863267
Cleavage Site 296
All Other Sites
Reads
0
5
10
15
0
100
200
300
400
500
gi393750907 position (nts)

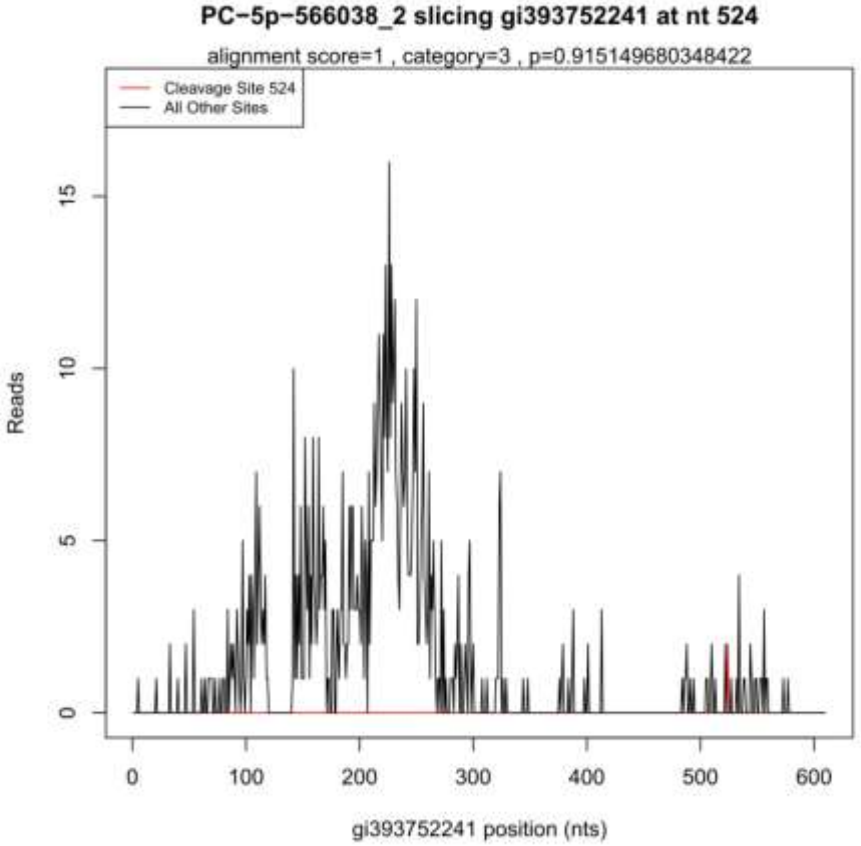
PC−5p−566038_2 slicing gi393752241 at nt 524
alignment score=1 , category=3 , p=0.915149680348422
Cleavage Site 524
All Other Sites
Reads
0
5
10
15
0
100
200
300
400
500
600
gi393752241 position (nts)

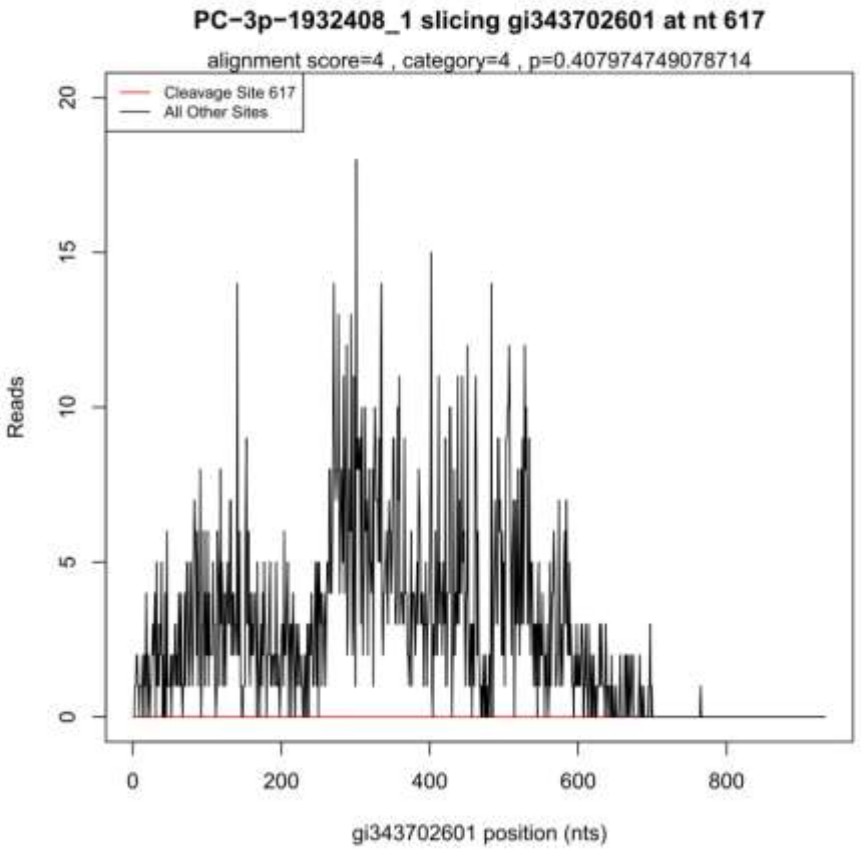
PC−3p−1932408_1 slicing gi343702601 at nt 617
alignment score=4 , category=4 , p=0.407974749078714
Cleavage Site 617
All Other Sites
Reads
gi343702601 position (nts)
0
5
10
15
20
0
200
400
600
800

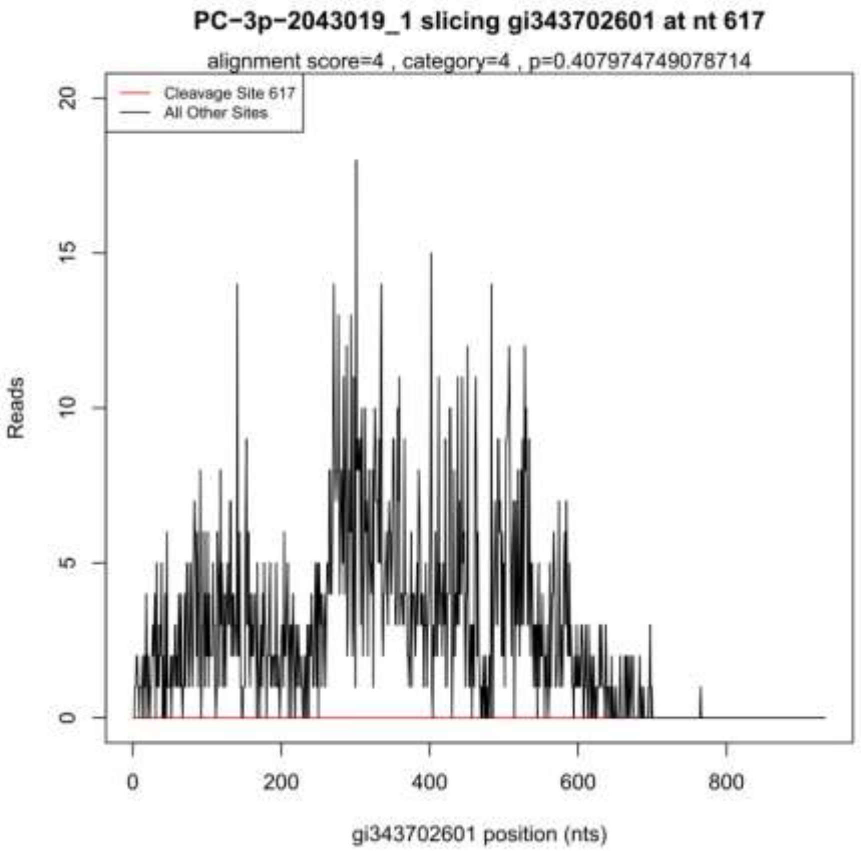
PC−3p−2043019_1 slicing gi343702601 at nt 617
alignment score=4 , category=4 , p=0.407974749078714
Cleavage Site 617
All Other Sites
Reads
gi343702601 position (nts)
0
5
10
15
20
0
200
400
600
800

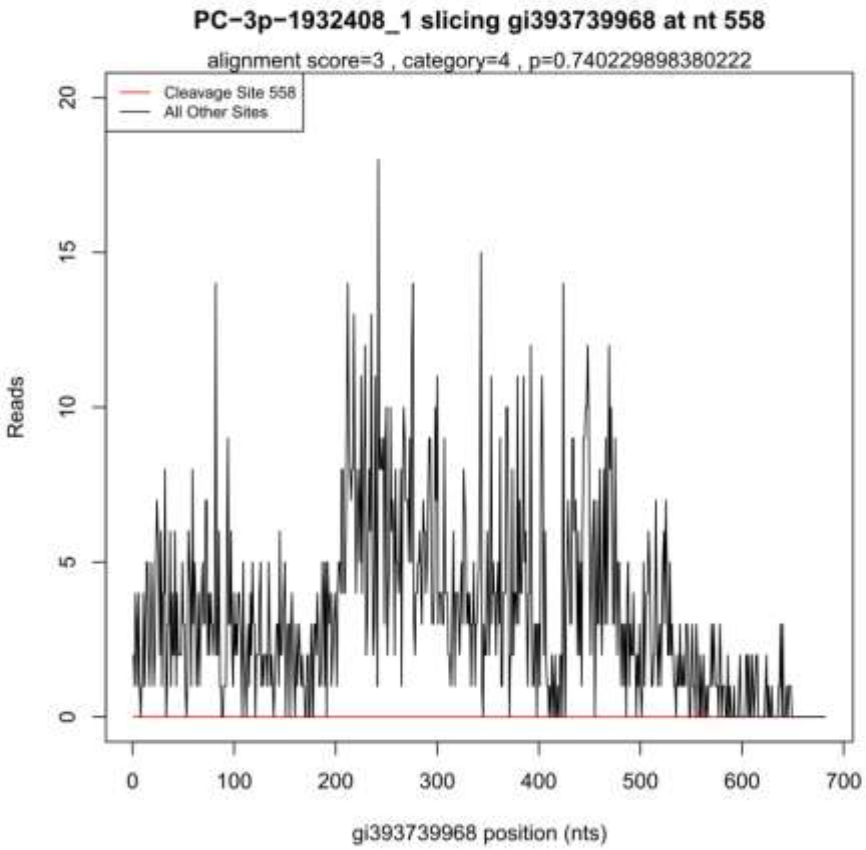
PC−3p−1932408_1 slicing gi393739968 at nt 558
alignment score=3 , category=4 , p=0.740229898380222
Cleavage Site 558
All Other Sites
Reads
gi393739968 position (nts)

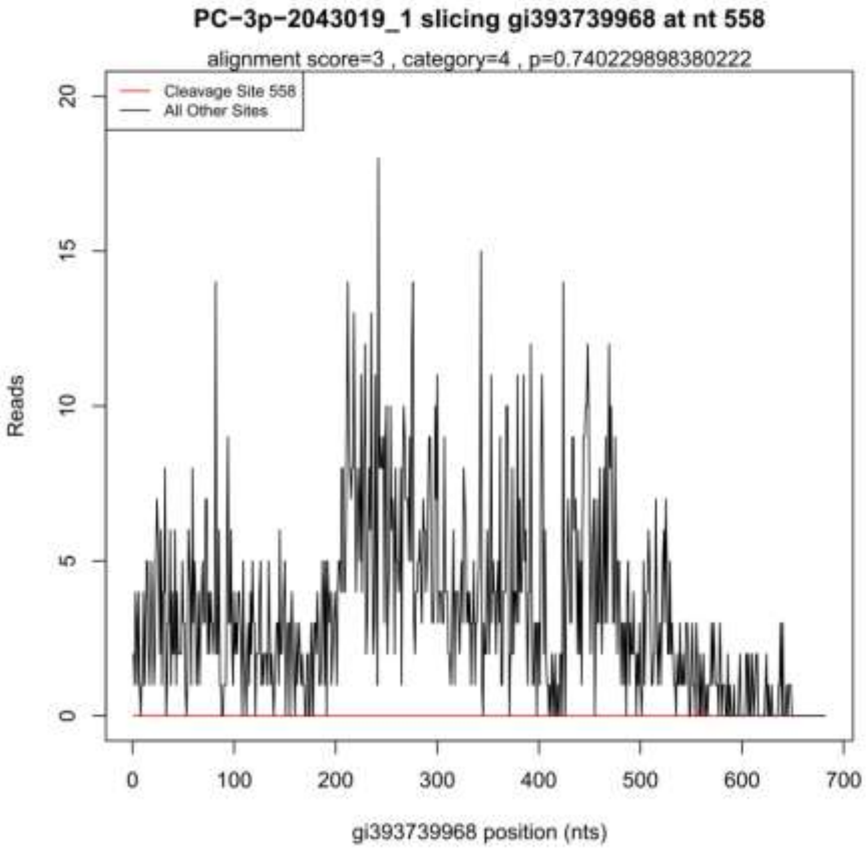
PC−3p−2043019_1 slicing gi393739968 at nt 558
alignment score=3 , category=4 , p=0.740229898380222
Cleavage Site 558
All Other Sites
Reads
gi393739968 position (nts)

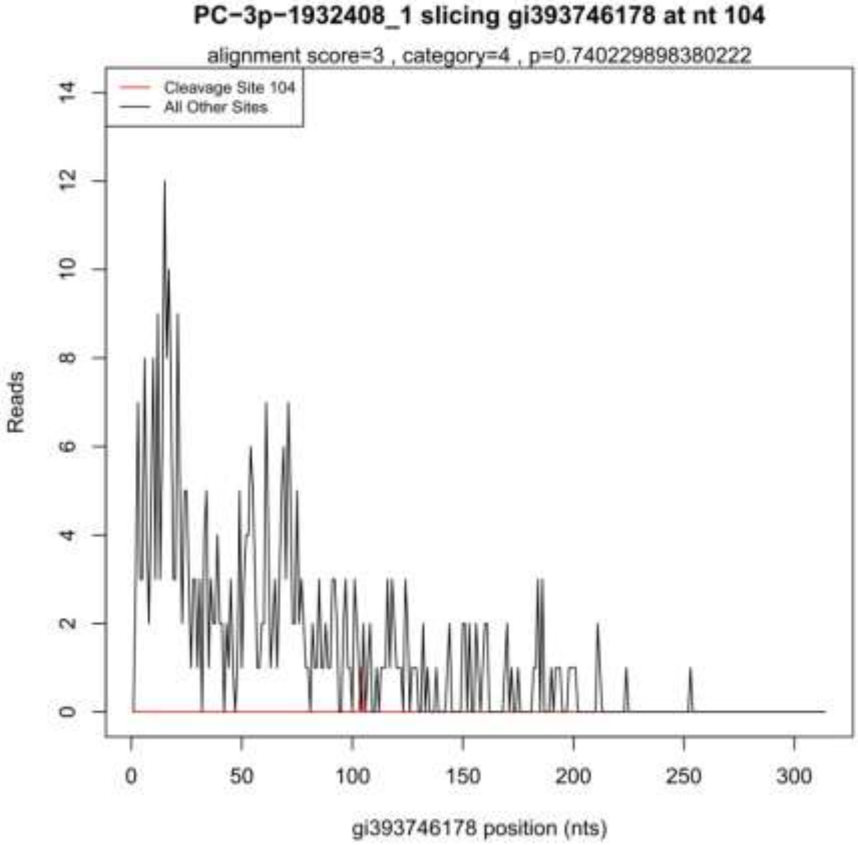
PC−3p−1932408_1 slicing gi393746178 at nt 104
alignment score=3 , category=4 , p=0.740229898380222
Cleavage Site 104
All Other Sites
Reads
gi393746178 position (nts)

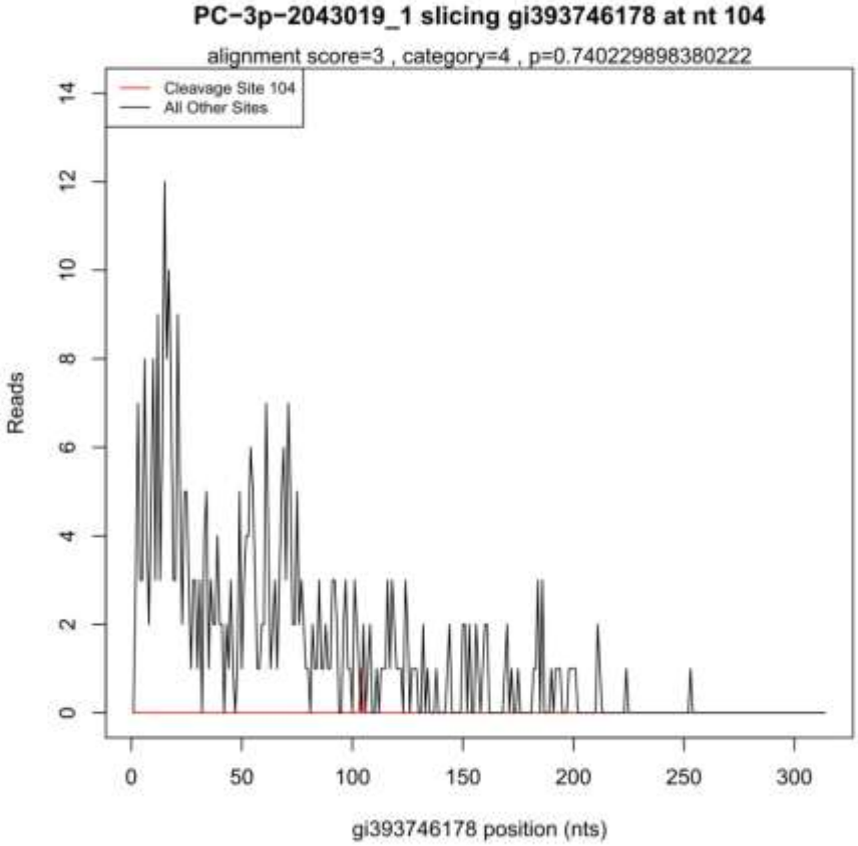
PC−3p−2043019_1 slicing gi393746178 at nt 104
alignment score=3 , category=4 , p=0.740229898380222
Cleavage Site 104
All Other Sites
Reads
gi393746178 position (nts)

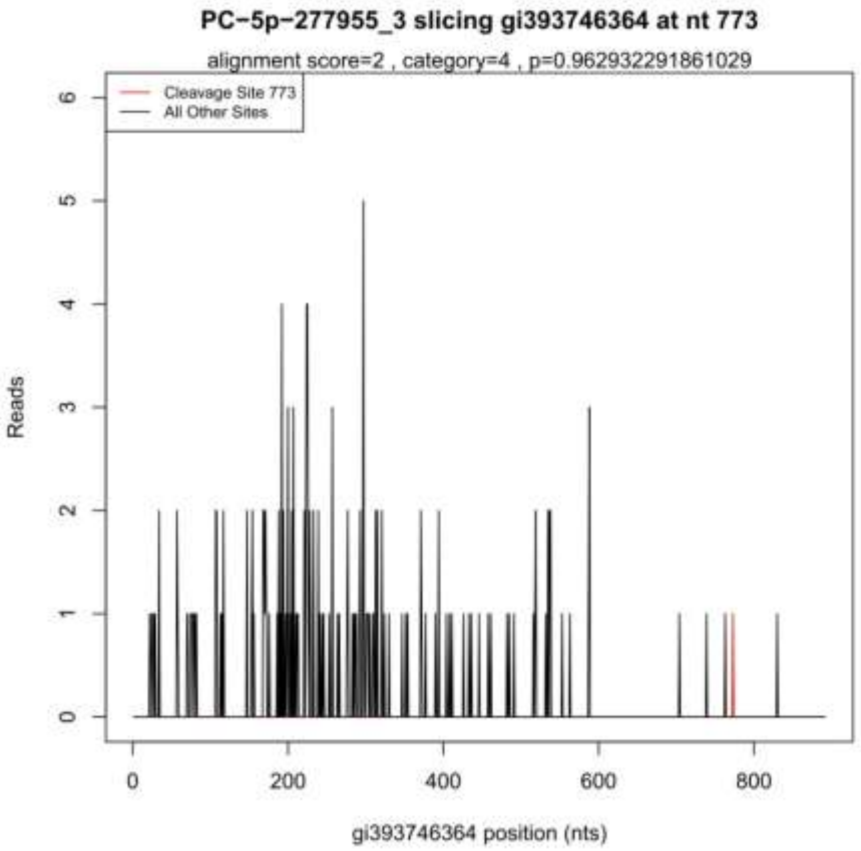
PC−5p−277955_3 slicing gi393746364 at nt 773
alignment score=2 , category=4 , p=0.962932291861029
Cleavage Site 773
All Other Sites
Reads
0
1
2
3
4
5
6
0
200
400
600
800
gi393746364 position (nts)

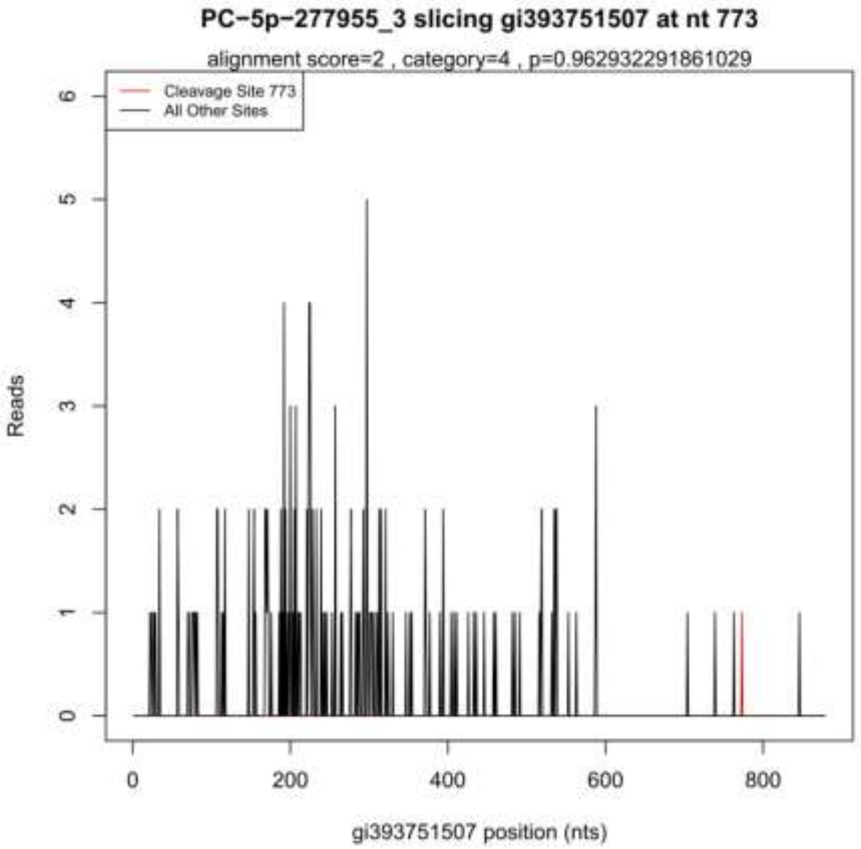
PC−5p−277955_3 slicing gi393751507 at nt 773
alignment score=2 , category=4 , p=0.962932291861029
Cleavage Site 773
All Other Sites
Reads
0
1
2
3
4
5
6
0
200
400
600
800
gi393751507 position (nts)

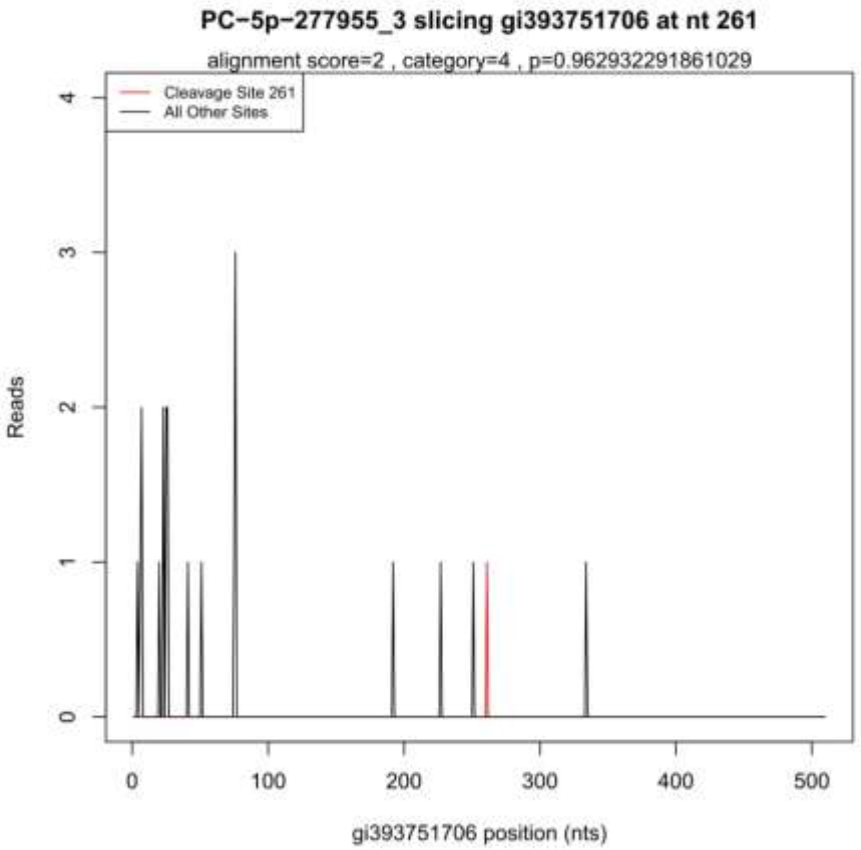
PC−5p−277955_3 slicing gi393751706 at nt 261
alignment score=2 , category=4 , p=0.962932291861029
Cleavage Site 261
All Other Sites
Reads
gi393751706 position (nts)

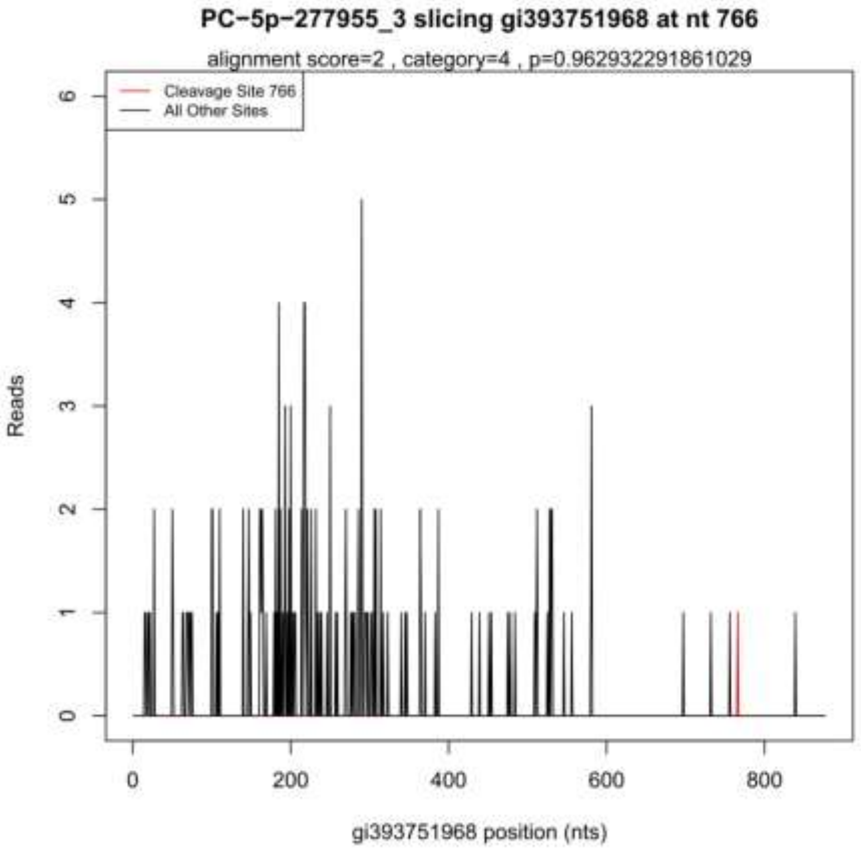
PC−5p−277955_3 slicing gi393751968 at nt 766
alignment score=2 , category=4 , p=0.962932291861029
Cleavage Site 766
All Other Sites
Reads
gi393751968 position (nts)

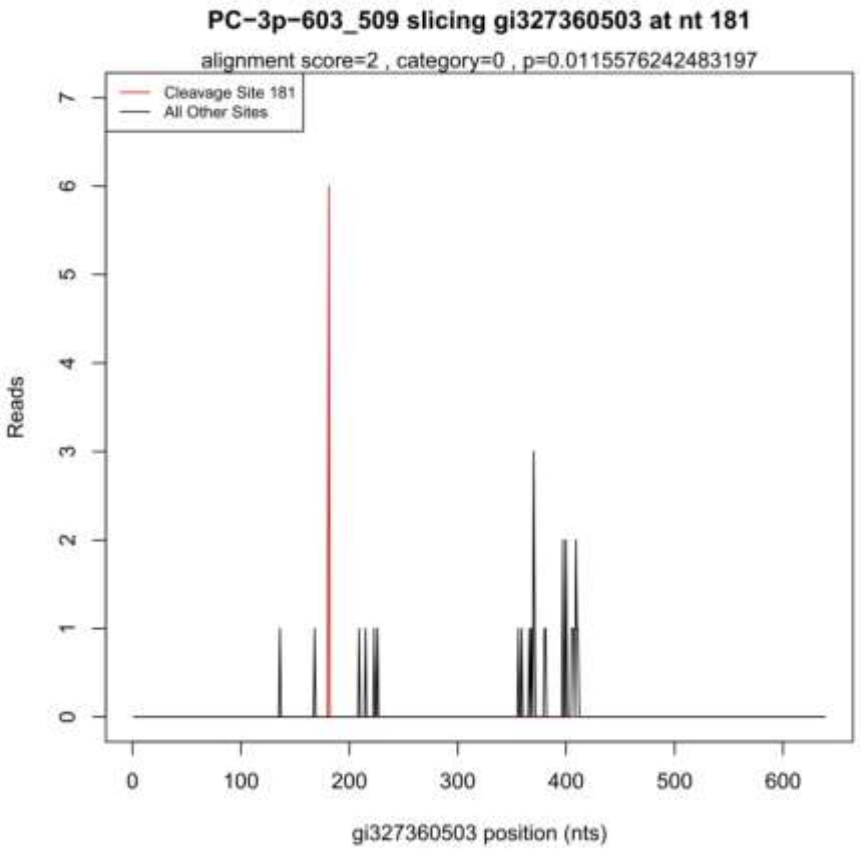
PC−3p−603_509 slicing gi327360503 at nt 181
alignment score=2 , category=0 , p=0.0115576242483197
Cleavage Site 181
All Other Sites
Reads
gi327360503 position (nts)

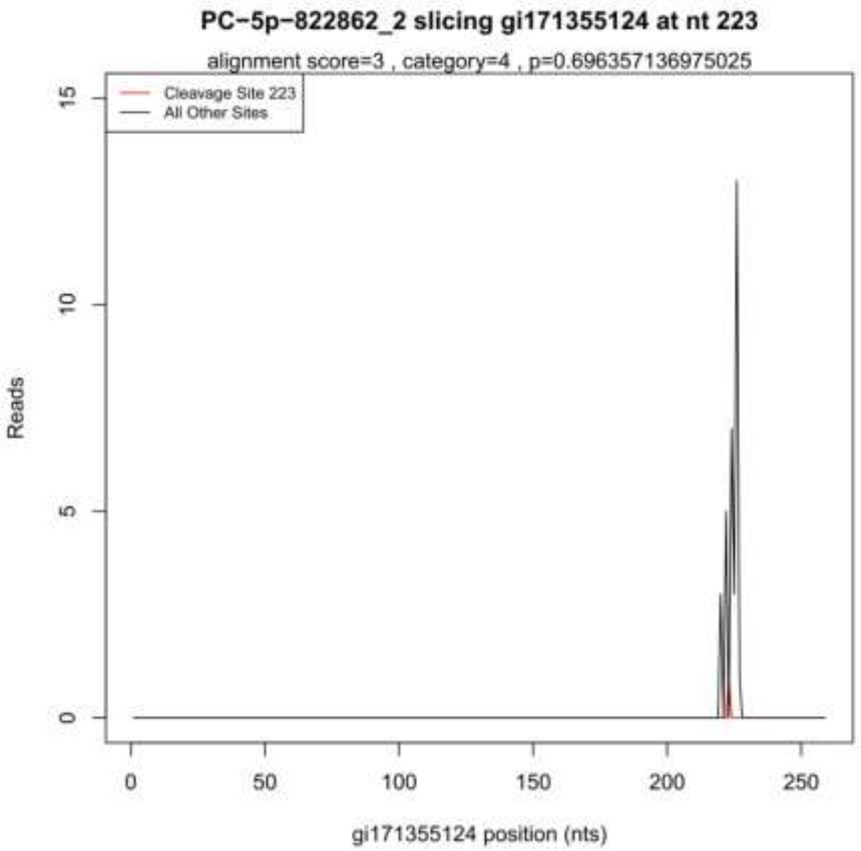
PC−5p−822862_2 slicing gi171355124 at nt 223
alignment score=3 , category=4 , p=0.696357136975025
Cleavage Site 223
All Other Sites
Reads
gi171355124 position (nts)

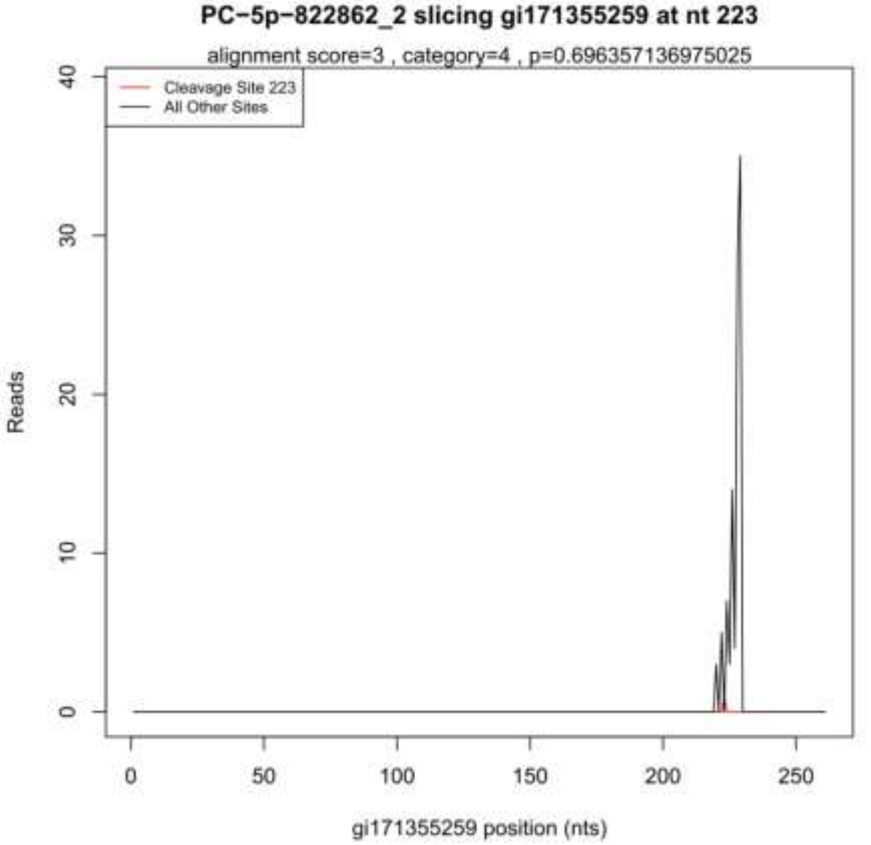
PC−5p−822862_2 slicing gi171355259 at nt 223
alignment score=3 , category=4 , p=0.696357136975025
Cleavage Site 223
All Other Sites
Reads
gi171355259 position (nts)

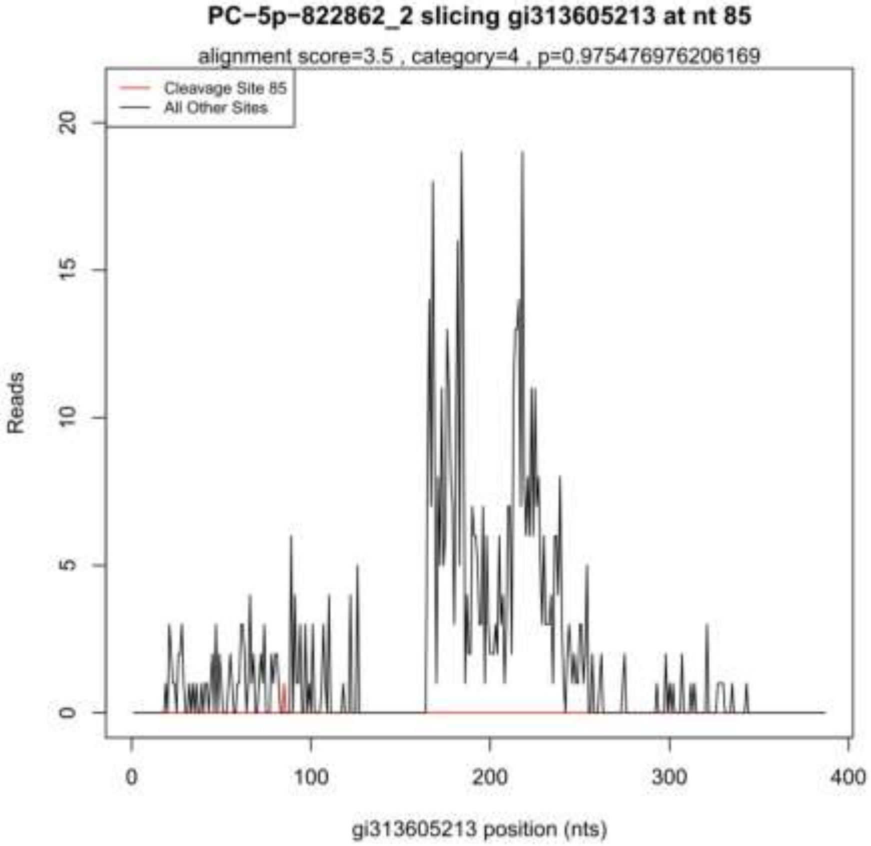
PC−5p−822862_2 slicing gi313605213 at nt 85
alignment score=3.5 , category=4 , p=0.975476976206169
Cleavage Site 85
All Other Sites
Reads
gi313605213 position (nts)

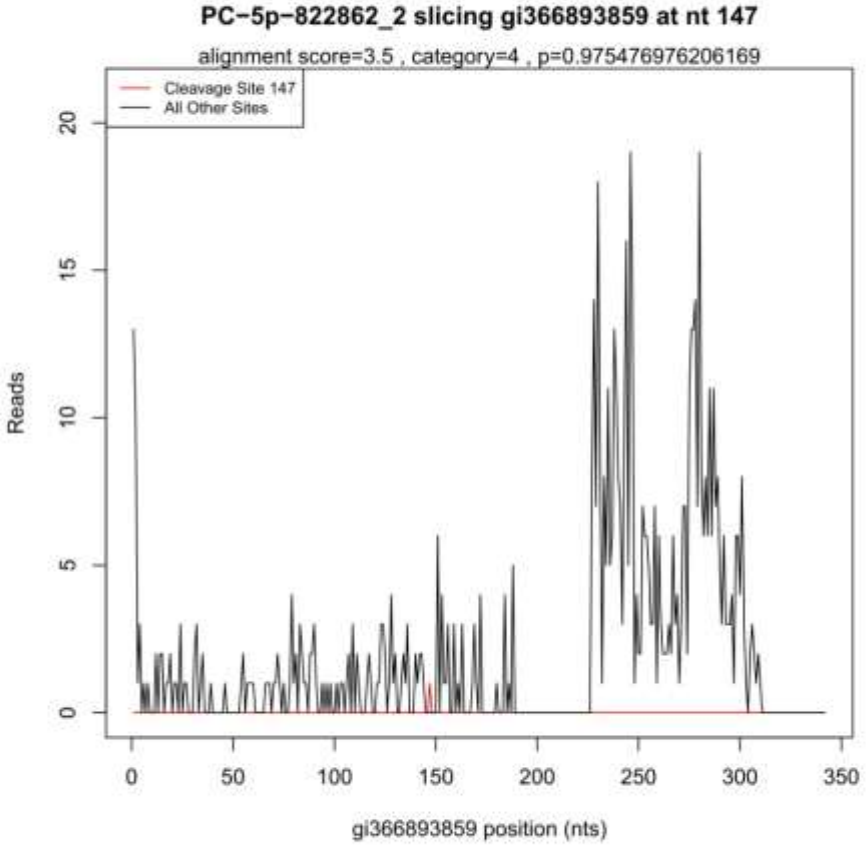
PC−5p−822862_2 slicing gi366893859 at nt 147
alignment score=3.5 , category=4 , p=0.975476976206169
Cleavage Site 147
All Other Sites
Reads
gi366893859 position (nts)

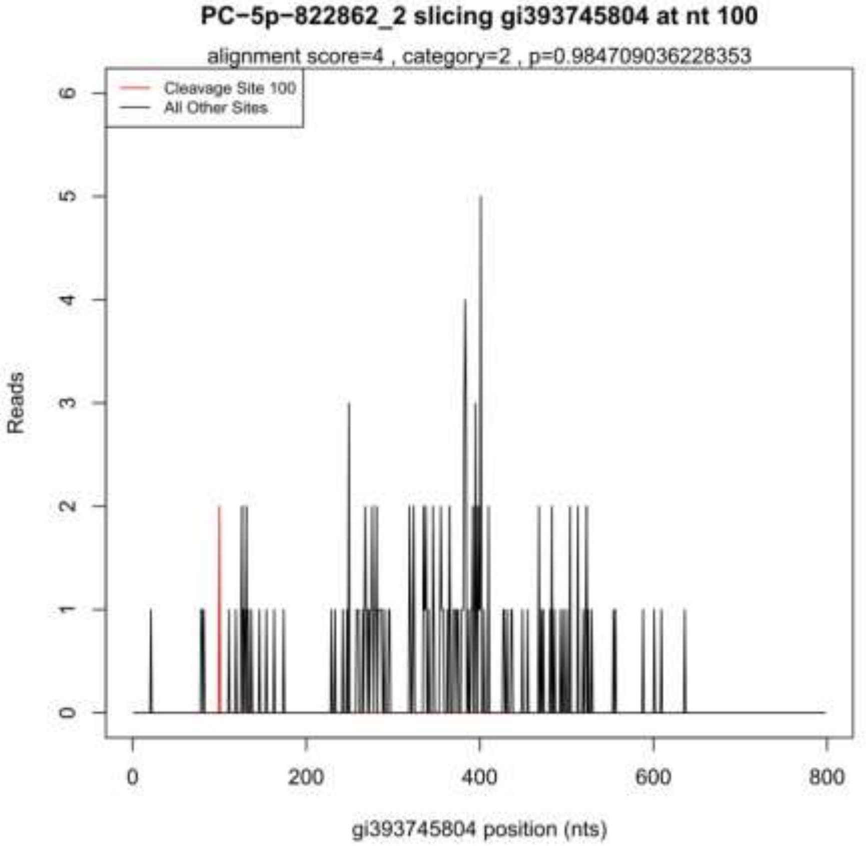
PC−5p−822862_2 slicing gi393745804 at nt 100
alignment score=4 , category=2 , p=0.984709036228353
Cleavage Site 100
All Other Sites
Reads
gi393745804 position (nts)

PC−3p−985144_1 slicing gi393388419 at nt 276

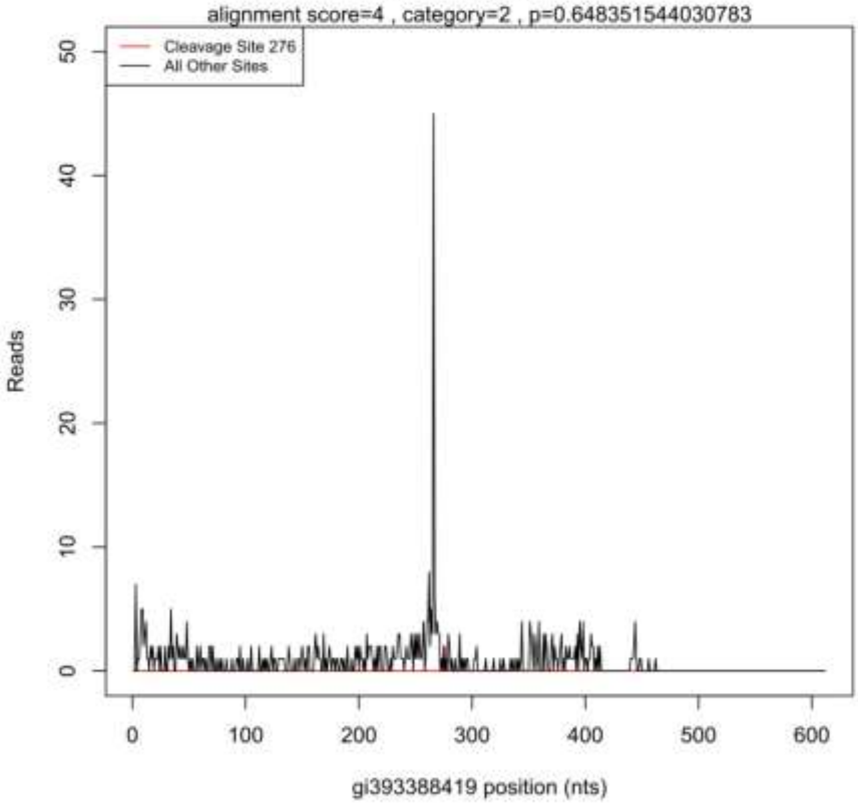

PC−5p−1575706_1 slicing gi393388419 at nt 355

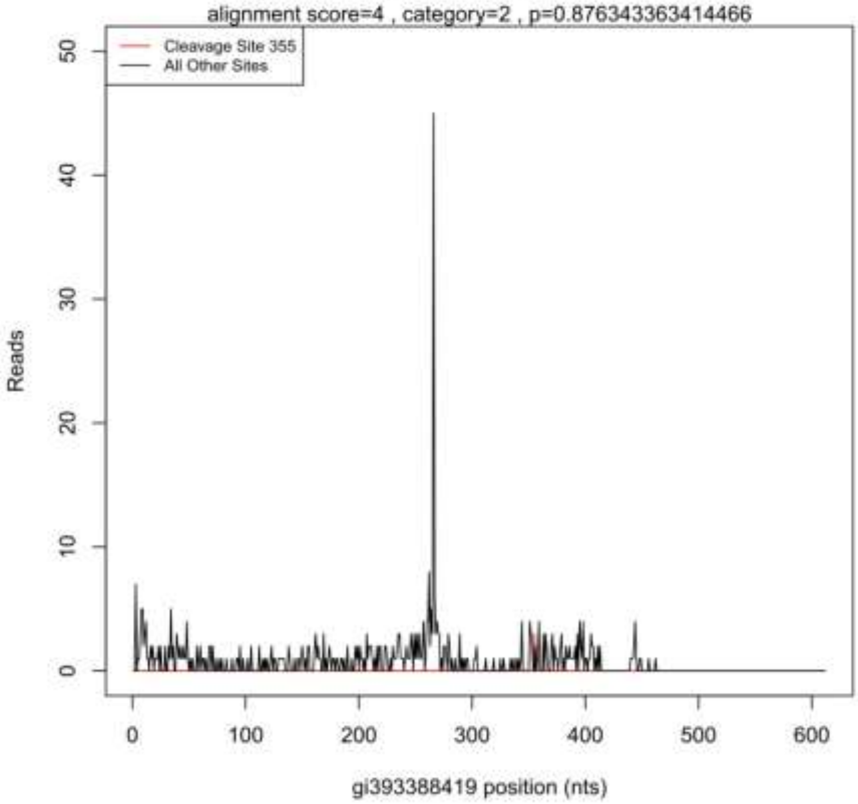

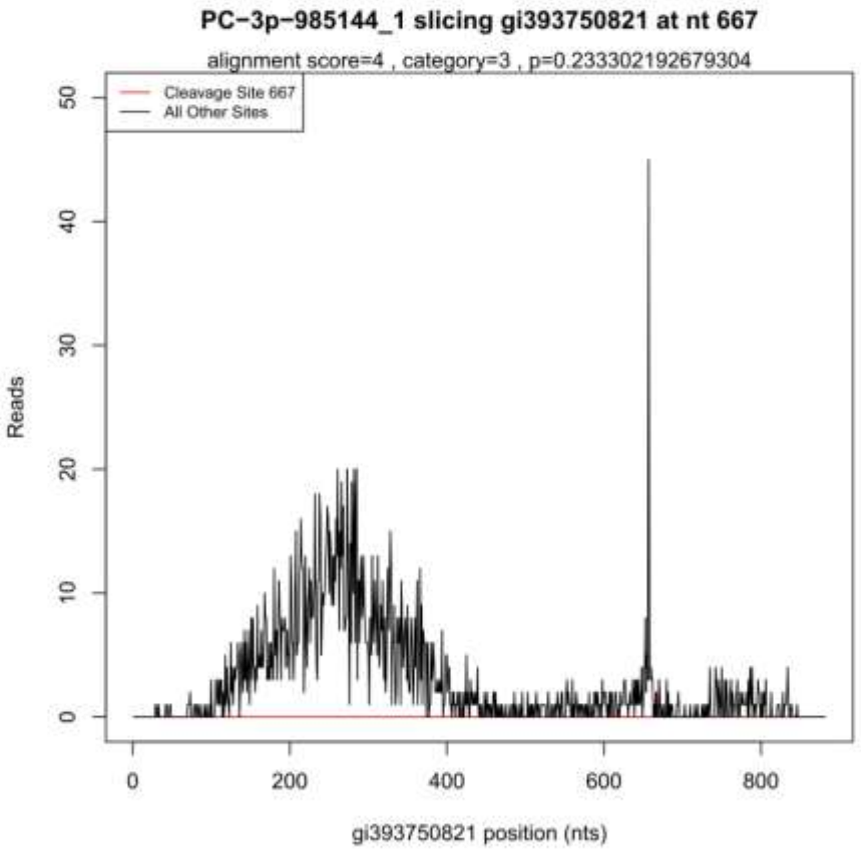
PC−3p−985144_1 slicing gi393750821 at nt 667
alignment score=4 , category=3 , p=0.233302192679304
Cleavage Site 667
All Other Sites
Reads
0
10
20
30
40
50
0
200
400
600
800
gi393750821 position (nts)

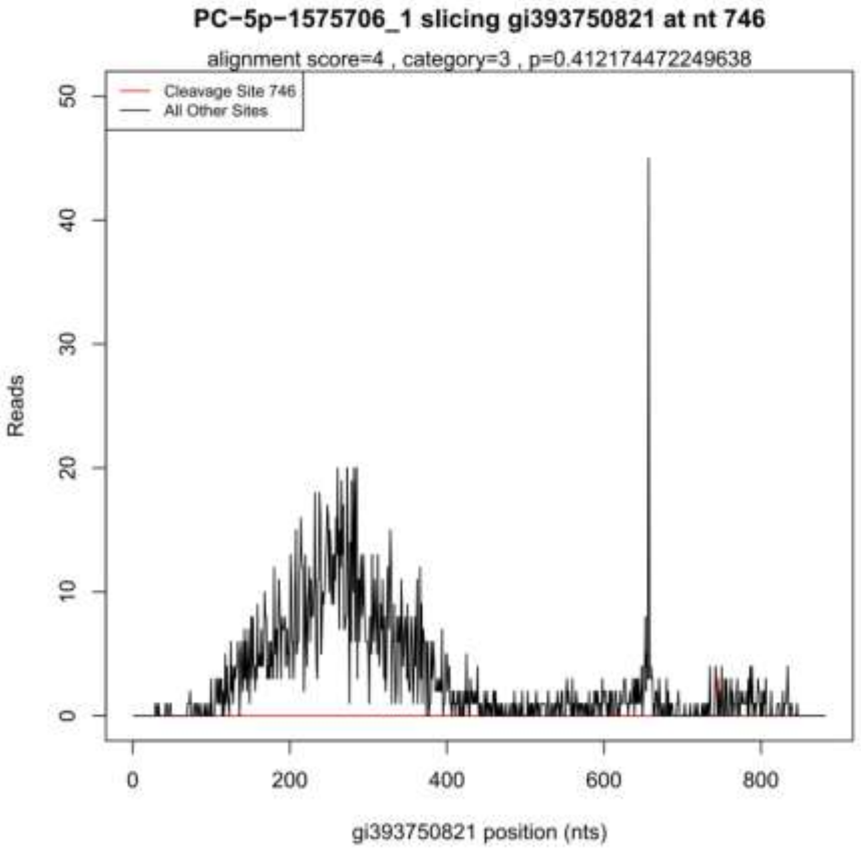
PC−5p−1575706_1 slicing gi393750821 at nt 746
alignment score=4 , category=3 , p=0.412174472249638
Cleavage Site 746
All Other Sites
Reads
0
10
20
30
40
50
0
200
400
600
800
gi393750821 position (nts)

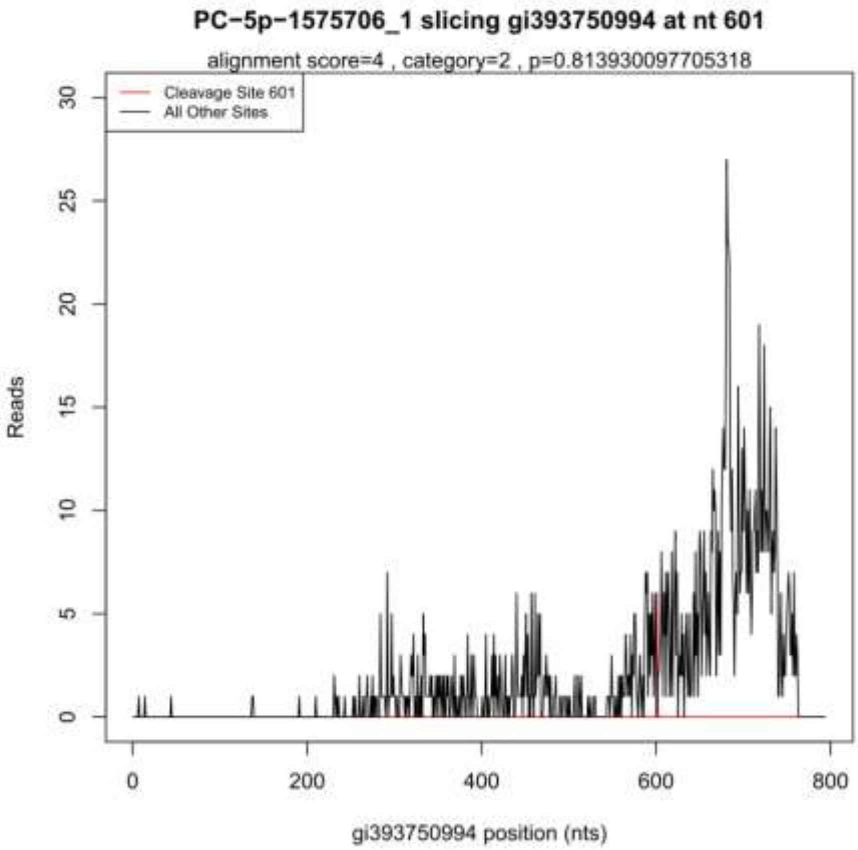

PC−5p−1575706_1 slicing gi393750994 at nt 601
alignment score=4 , category=2 , p=0.813930097705318
Cleavage Site 601
All Other Sites
Reads
gi393750994 position (nts)

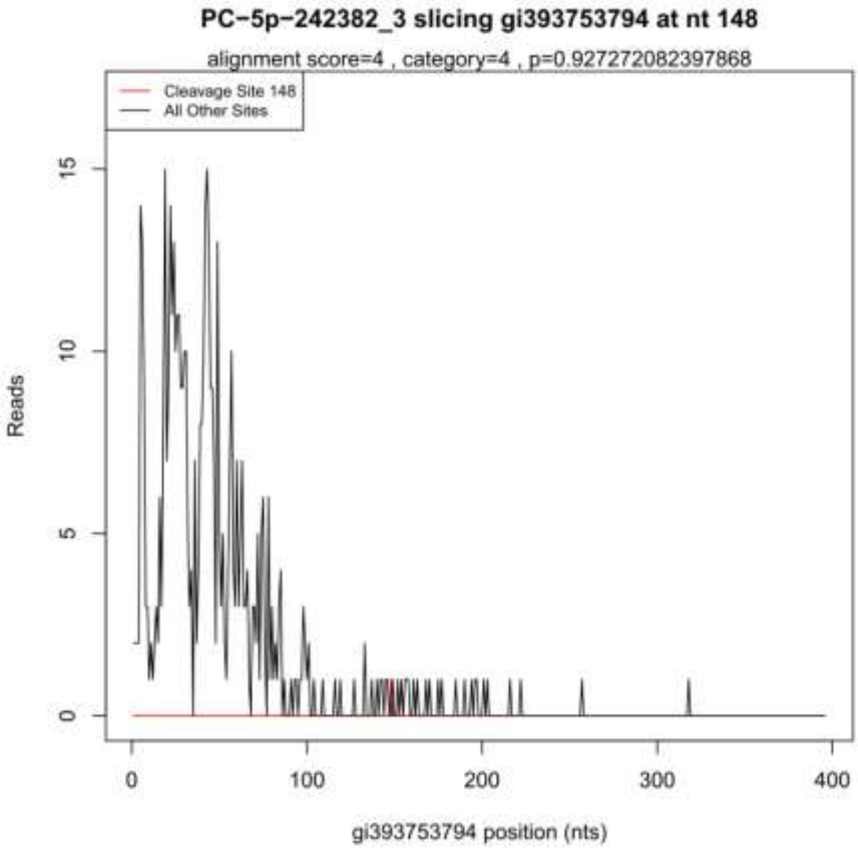

PC−5p−242382_3 slicing gi393753794 at nt 148
alignment score=4 , category=4 , p=0.927272082397868
Cleavage Site 148
All Other Sites
Reads
gi393753794 position (nts)

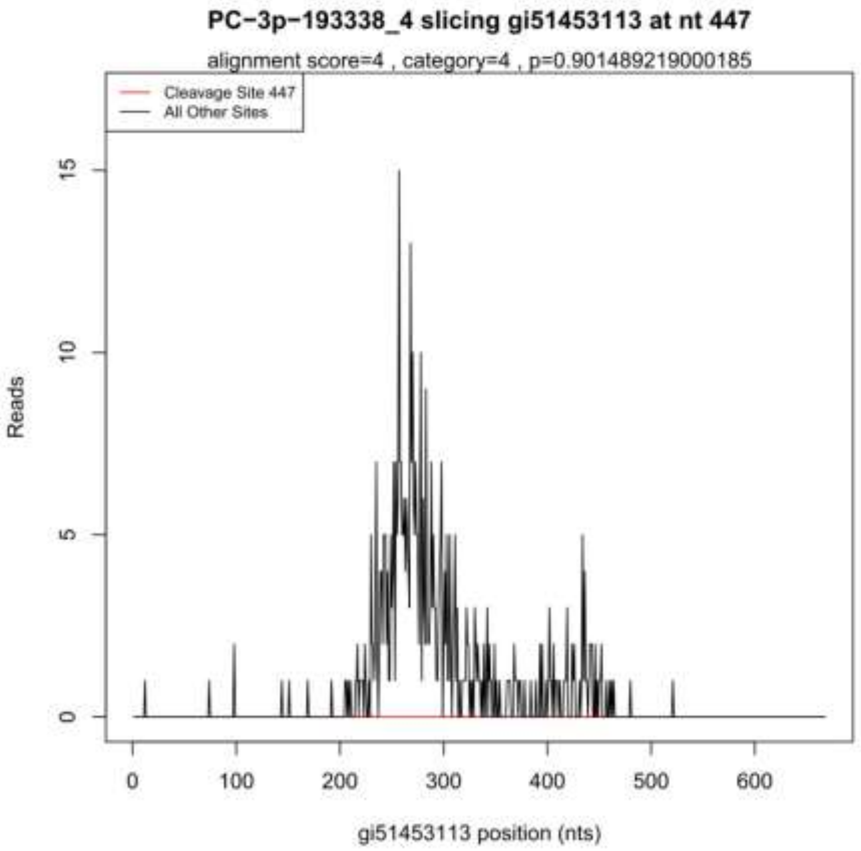
PC-3p-193338_4 slicing gi51453113 at nt 447
alignment score=4 , category=4 , p=0.901489219000185
Cleavage Site 447
All Other Sites
Reads
gi51453113 position (nts)

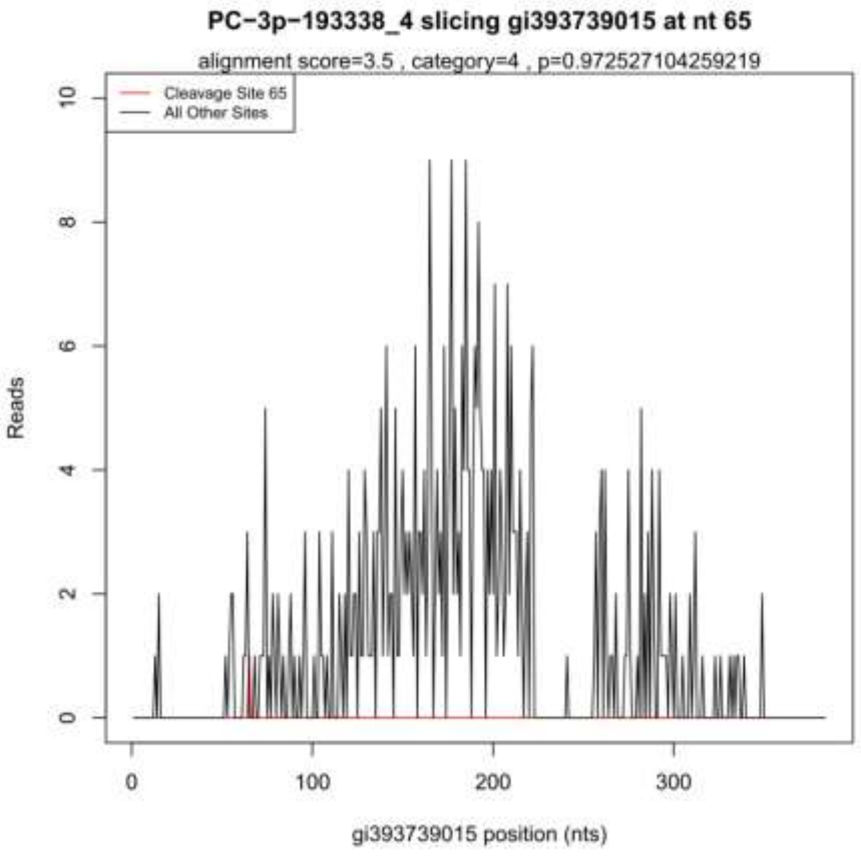
PC-3p-193338_4 slicing gi393739015 at nt 65
alignment score=3.5 , category=4 , p=0.972527104259219
Cleavage Site 65
All Other Sites
Reads
gi393739015 position (nts)

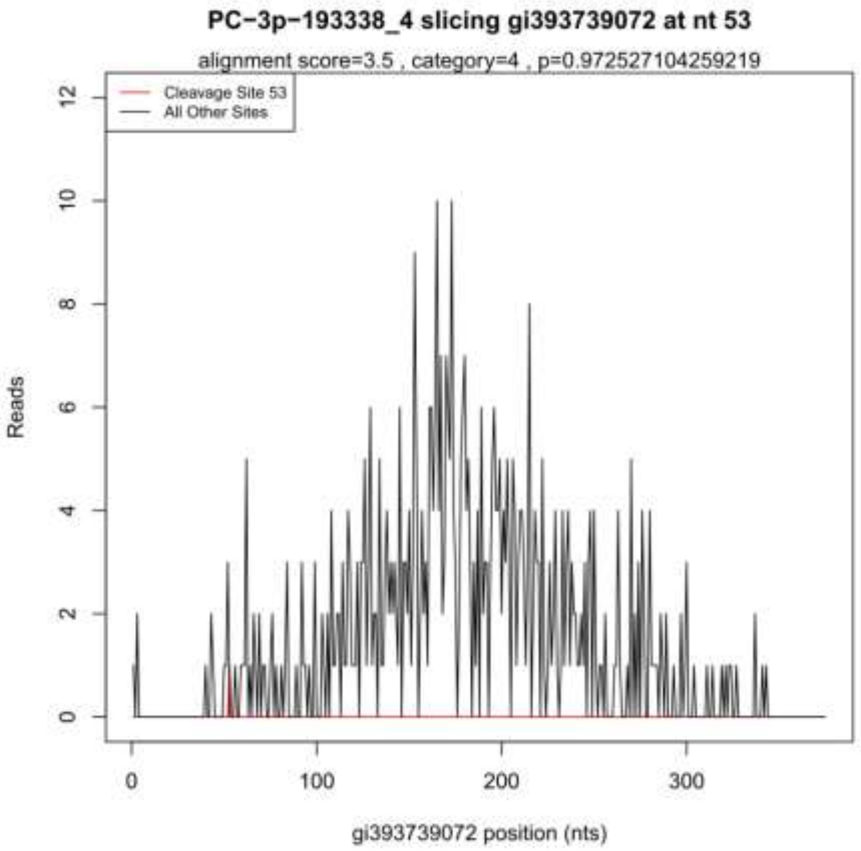
PC−3p−193338_4 slicing gi393739072 at nt 53
alignment score=3.5 , category=4 , p=0.972527104259219
Cleavage Site 53
All Other Sites
Reads
gi393739072 position (nts)

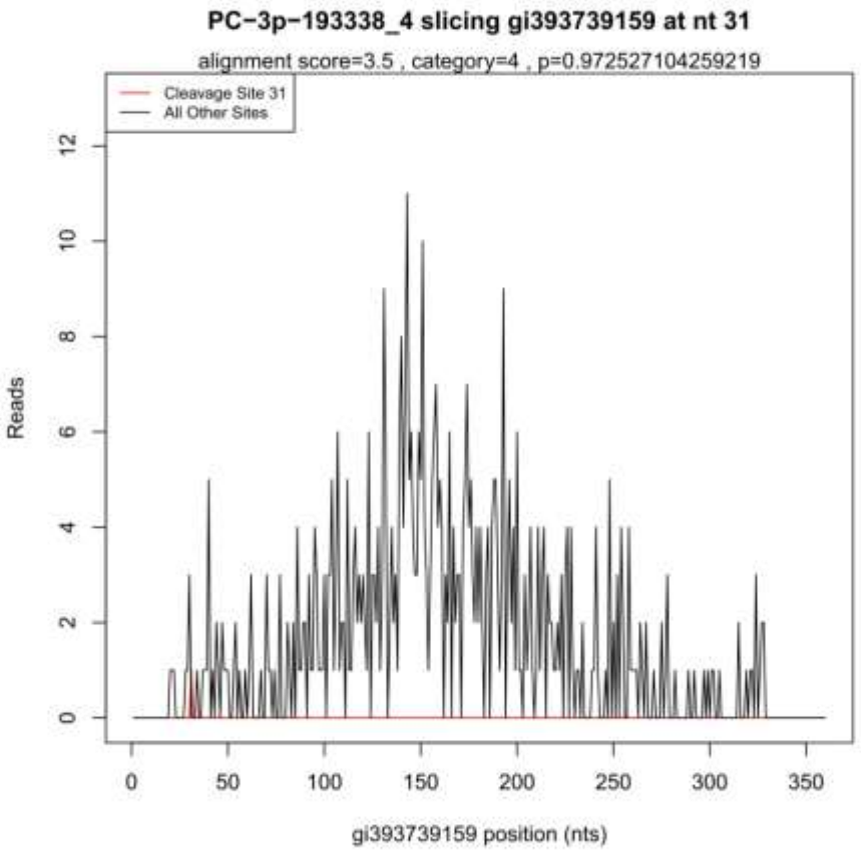
PC−3p−193338_4 slicing gi393739159 at nt 31
alignment score=3.5 , category=4 , p=0.972527104259219
Cleavage Site 31
All Other Sites
Reads
gi393739159 position (nts)

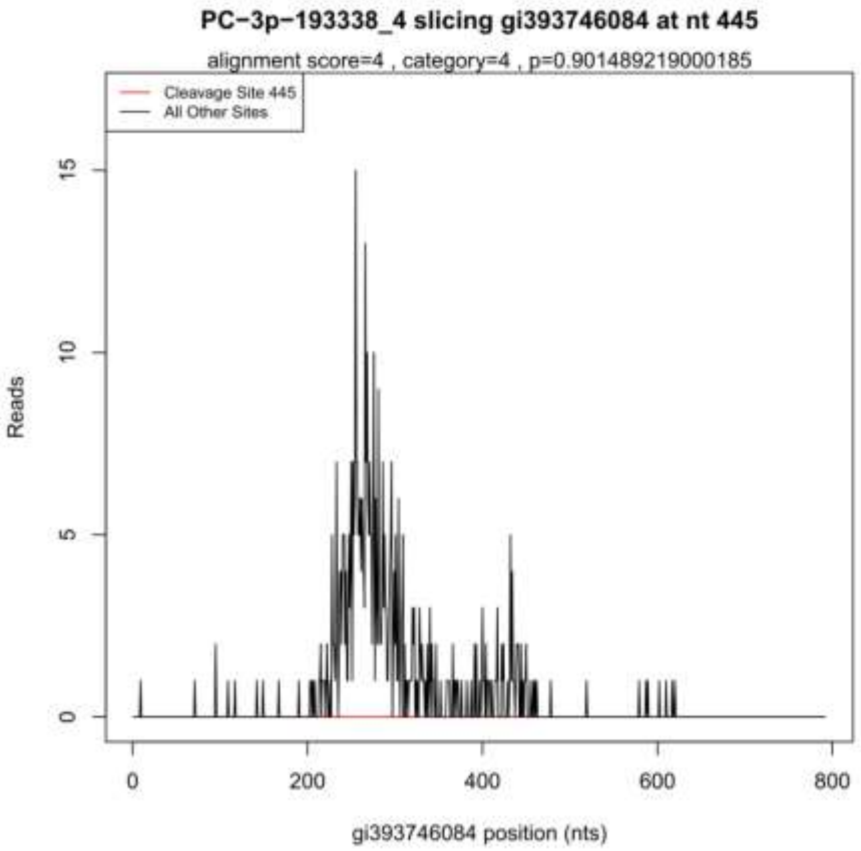
PC−3p−193338_4 slicing gi393746084 at nt 445
alignment score=4 , category=4 , p=0.901489219000185
Cleavage Site 445
All Other Sites
Reads
gi393746084 position (nts)
0
5
10
15
0
200
400
600
800

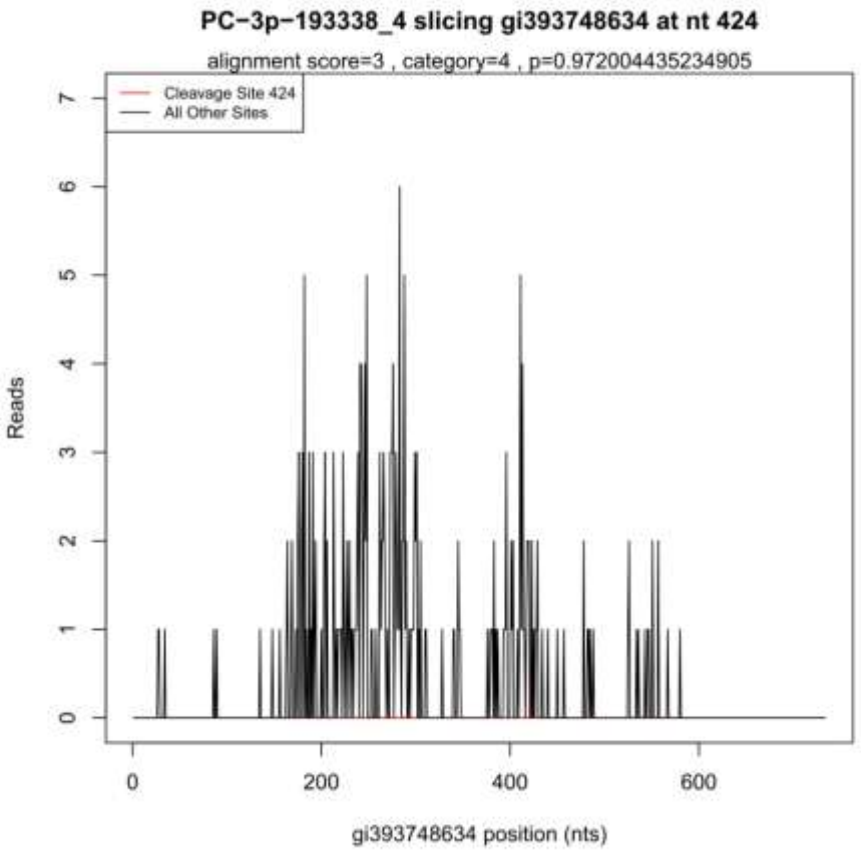
PC−3p−193338_4 slicing gi393748634 at nt 424
alignment score=3 , category=4 , p=0.972004435234905
Cleavage Site 424
All Other Sites
Reads
gi393748634 position (nts)
0
1
2
3
4
5
6
7
0
200
400
600

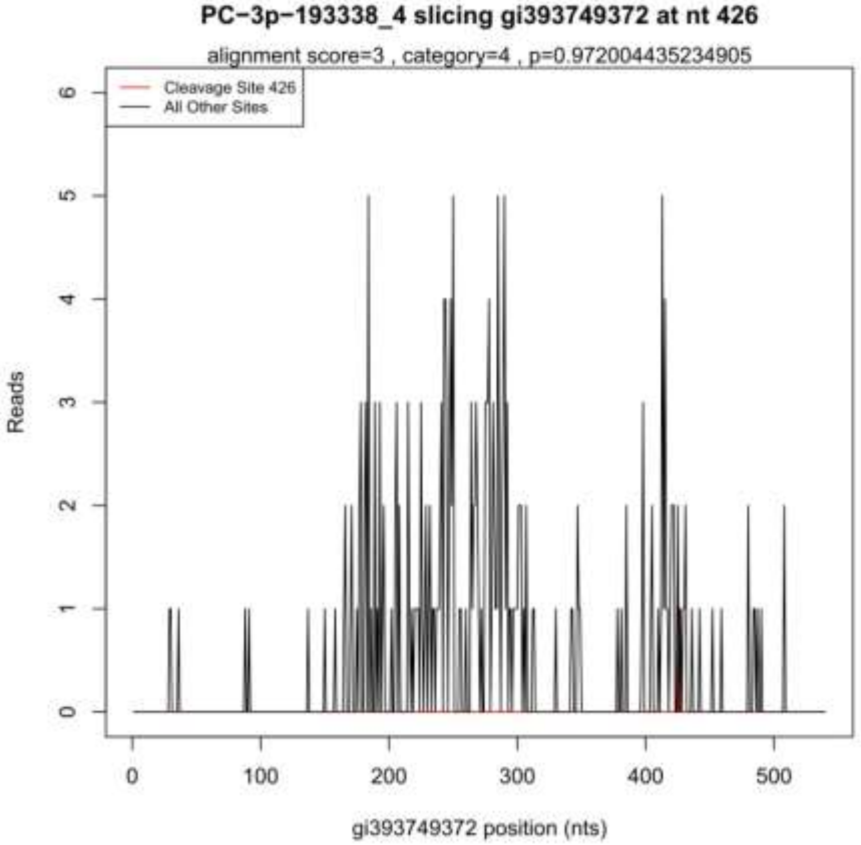
PC−3p−193338_4 slicing gi393749372 at nt 426
alignment score=3 , category=4 , p=0.972004435234905
Cleavage Site 426
All Other Sites
Reads
0
1
2
3
4
5
6
0
100
200
300
400
500
gi393749372 position (nts)

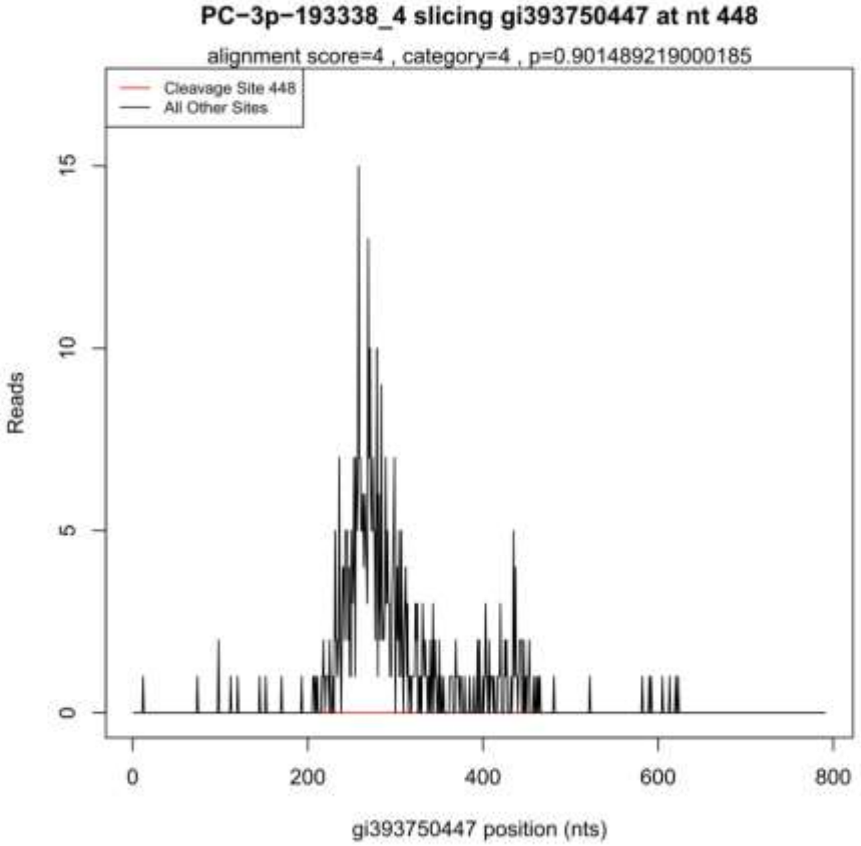
PC−3p−193338_4 slicing gi393750447 at nt 448
alignment score=4 , category=4 , p=0.901489219000185
Cleavage Site 448
All Other Sites
Reads
0
5
10
15
0
200
400
600
800
gi393750447 position (nts)

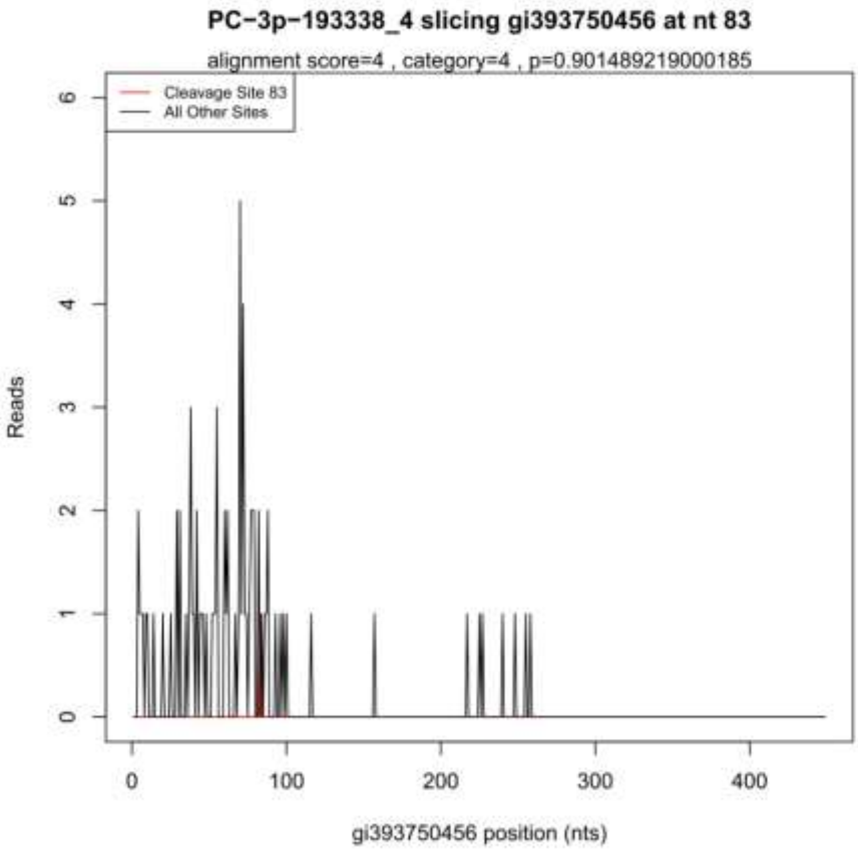
PC−3p−193338_4 slicing gi393750456 at nt 83
alignment score=4 , category=4 , p=0.901489219000185
Cleavage Site 83
All Other Sites
Reads
gi393750456 position (nts)

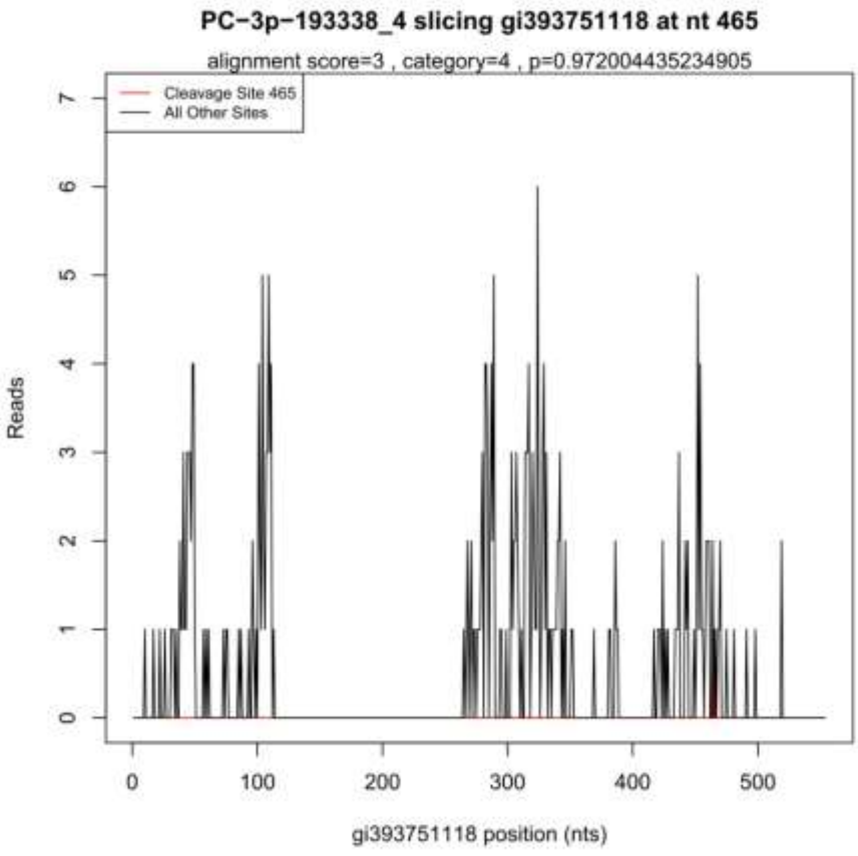
PC−3p−193338_4 slicing gi393751118 at nt 465
alignment score=3 , category=4 , p=0.972004435234905
Cleavage Site 465
All Other Sites
Reads
gi393751118 position (nts)

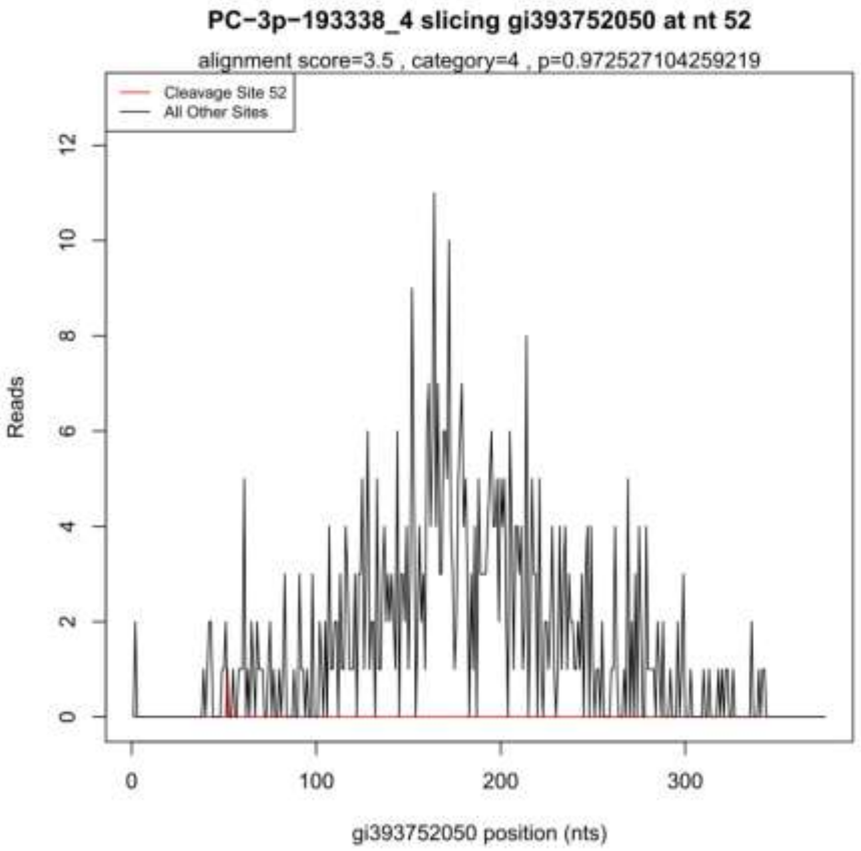
PC-3p-193338_4 slicing gi393752050 at nt 52
alignment score=3.5 , category=4 , p=0.972527104259219
Cleavage Site 52
All Other Sites
Reads
0
2
4
6
8
10
12
0
100
200
300
gi393752050 position (nts)

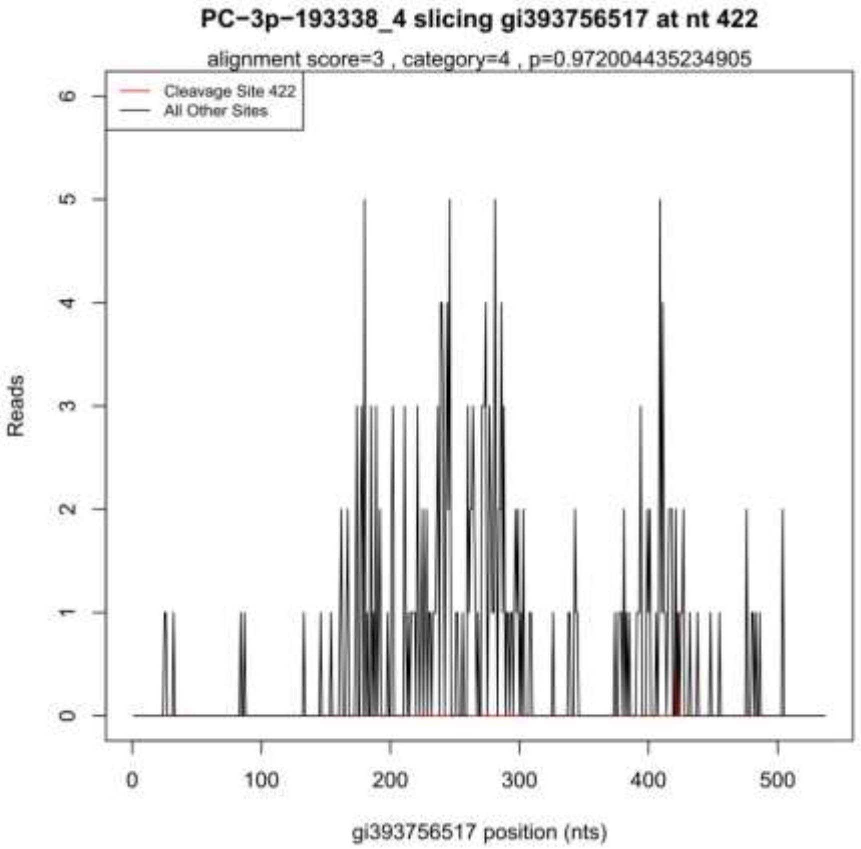
PC-3p-193338_4 slicing gi393756517 at nt 422
alignment score=3 , category=4 , p=0.972004435234905
Cleavage Site 422
All Other Sites
Reads
0
1
2
3
4
5
6
0
100
200
300
400
500
gi393756517 position (nts)

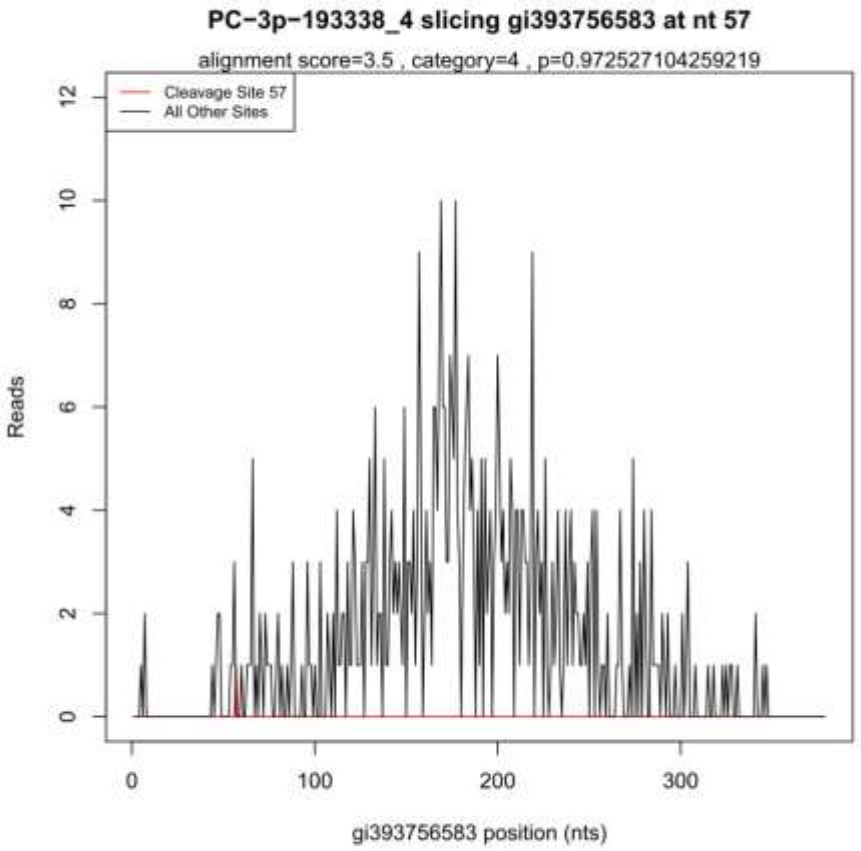
PC−3p−193338_4 slicing gi393756583 at nt 57
alignment score=3.5 , category=4 , p=0.972527104259219
Cleavage Site 57
All Other Sites
Reads
0
2
4
6
8
10
12
0
100
200
300
gi393756583 position (nts)

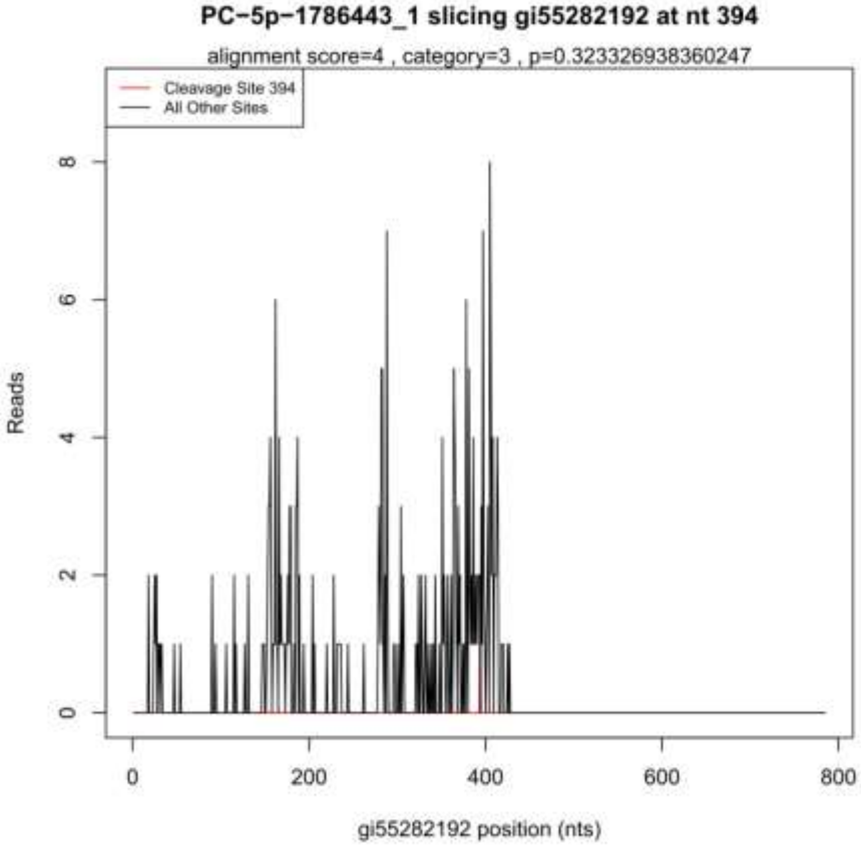
PC−5p−1786443_1 slicing gi55282192 at nt 394
alignment score=4 , category=3 , p=0.323326938360247
Cleavage Site 394
All Other Sites
Reads
0
2
4
6
8
0
200
400
600
800
gi55282192 position (nts)

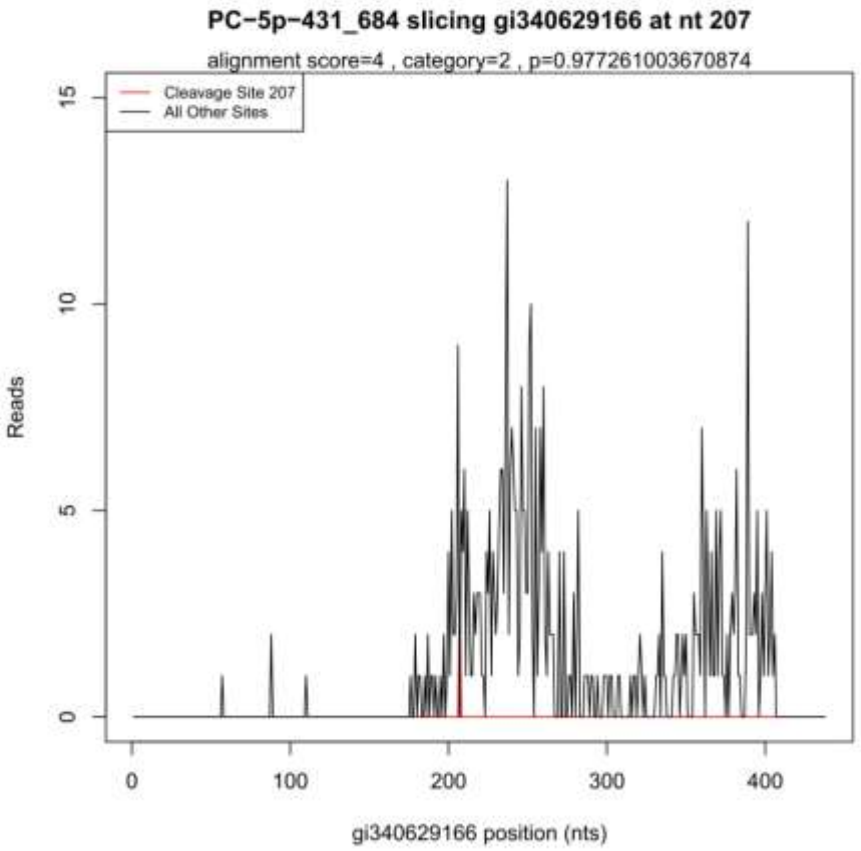
PC−5p−431_684 slicing gi340629166 at nt 207
alignment score=4 , category=2 , p=0.977261003670874
Cleavage Site 207
All Other Sites
Reads
0
5
10
15
0
100
200
300
400
gi340629166 position (nts)

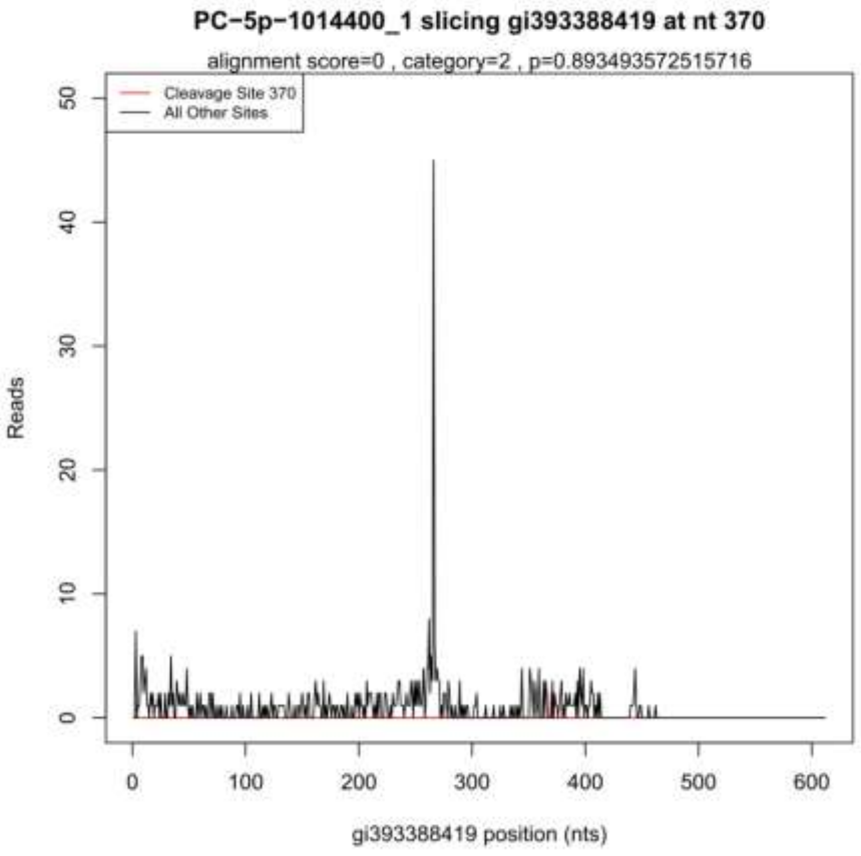
PC−5p−1014400_1 slicing gi393388419 at nt 370
alignment score=0 , category=2 , p=0.893493572515716
Cleavage Site 370
All Other Sites
Reads
0
10
20
30
40
50
0
100
200
300
400
500
600
gi393388419 position (nts)

PC−5p−1014400_1 slicing gi393750821 at nt 761

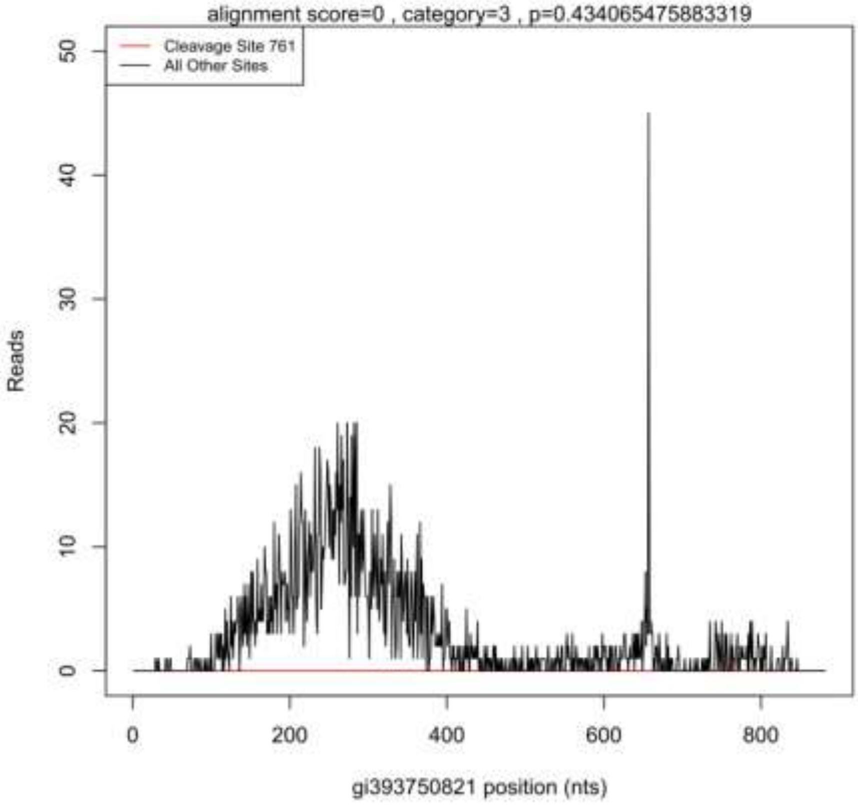

PC−3p−238382_3 slicing gi88605435 at nt 183

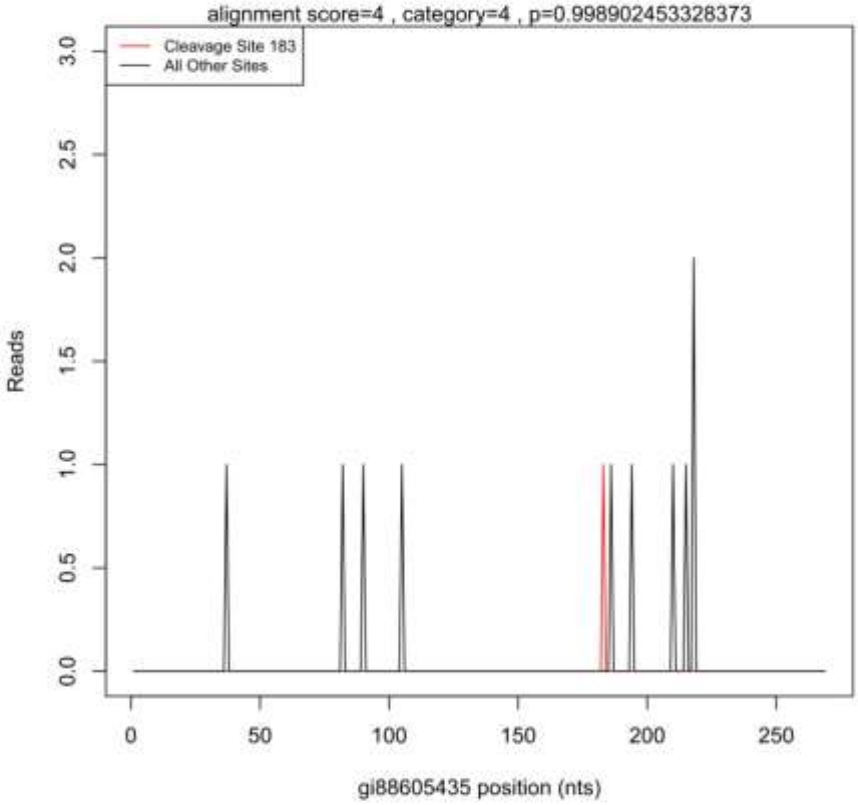

PC−3p−238382_3 slicing gi393738770 at nt 473

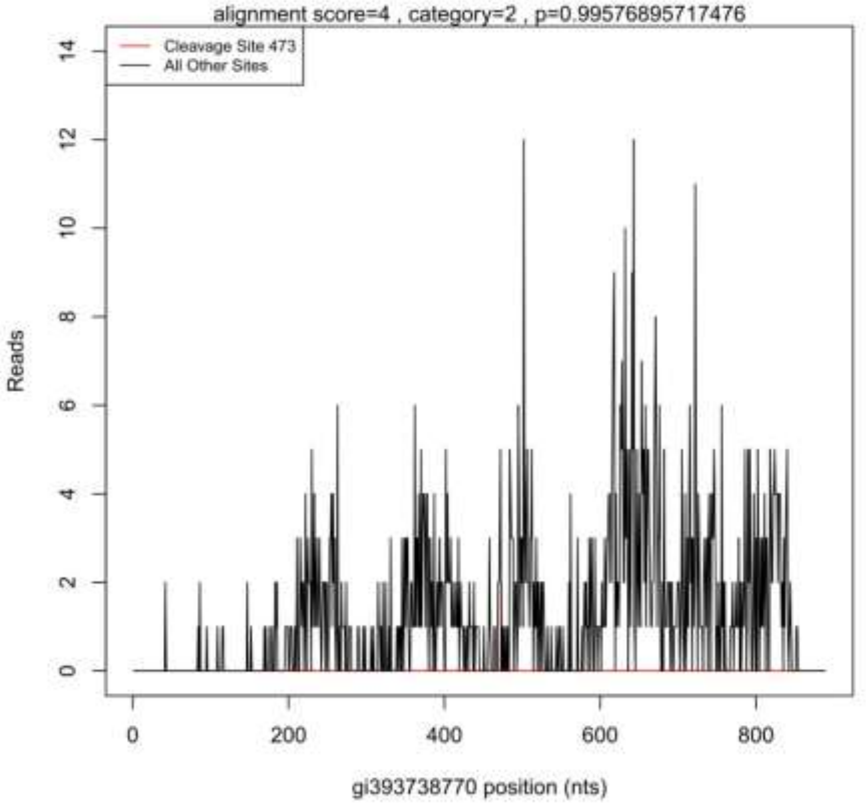

PC−3p−238382_3 slicing gi393745887 at nt 361

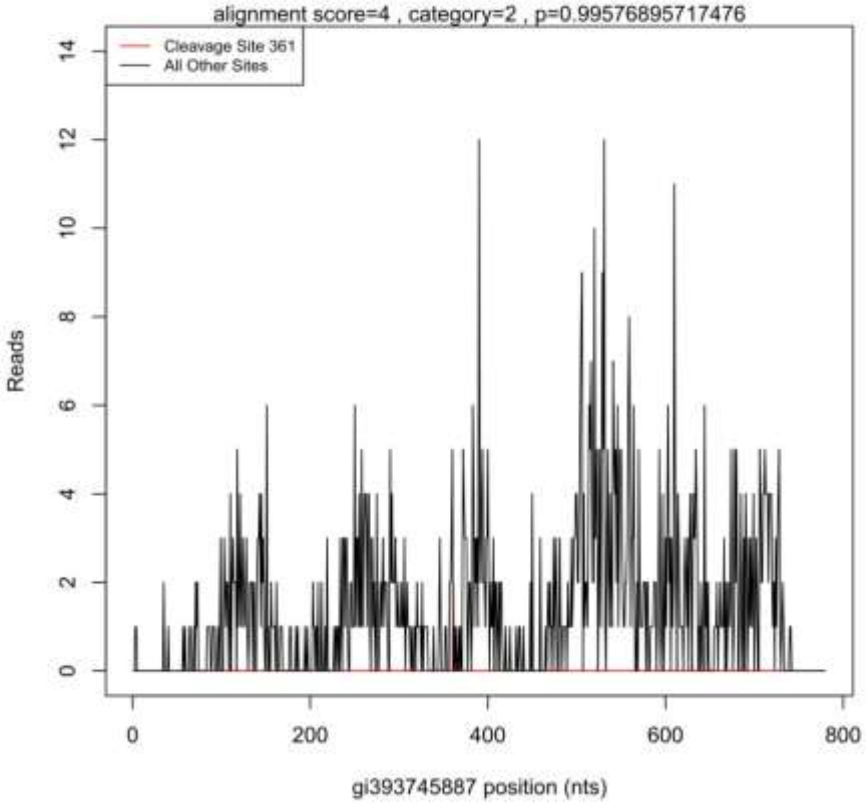

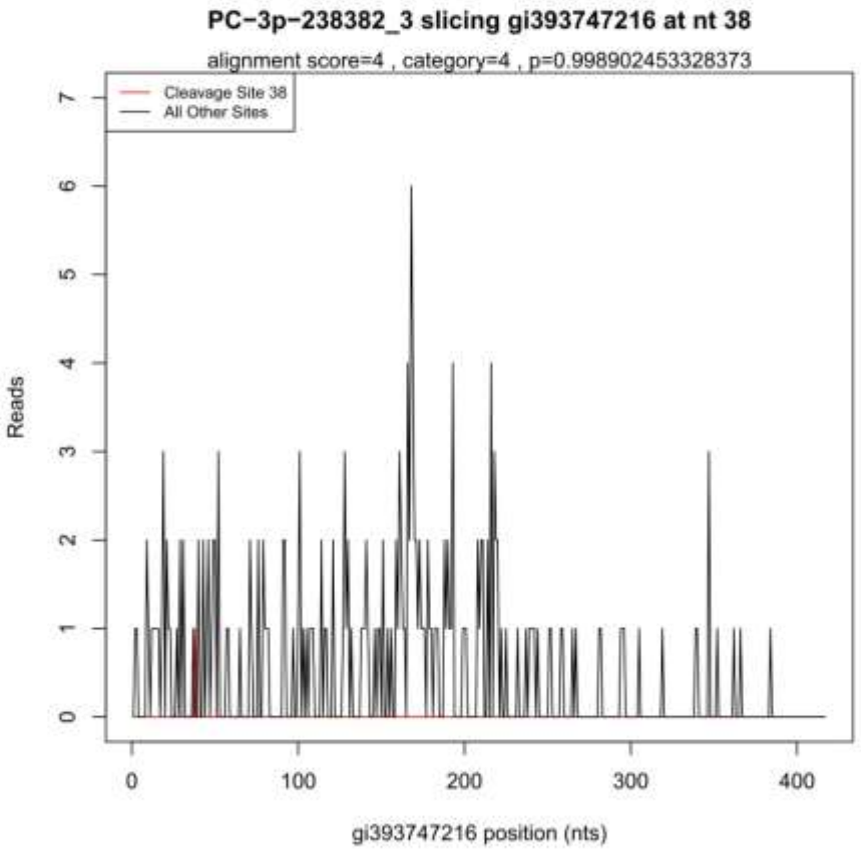
PC−3p−238382_3 slicing gi393747216 at nt 38
alignment score=4 , category=4 , p=0.998902453328373
Cleavage Site 38
All Other Sites
Reads
gi393747216 position (nts)

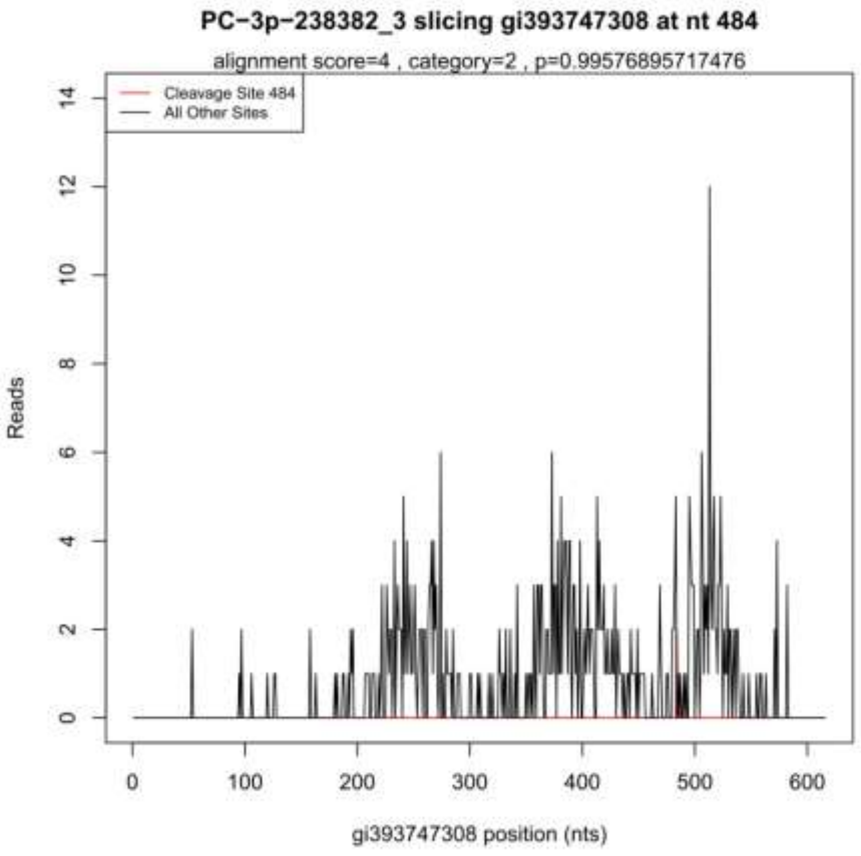
PC−3p−238382_3 slicing gi393747308 at nt 484
alignment score=4 , category=2 , p=0.99576895717476
Cleavage Site 484
All Other Sites
Reads
gi393747308 position (nts)

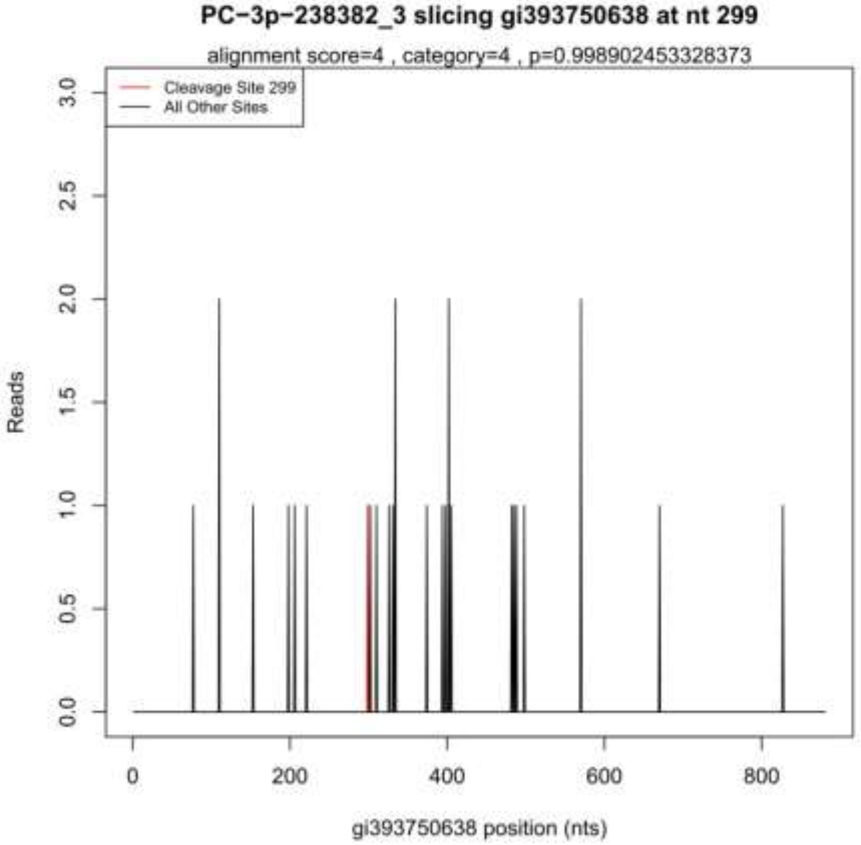
PC−3p−238382_3 slicing gi393750638 at nt 299
alignment score=4 , category=4 , p=0.998902453328373
Cleavage Site 299
All Other Sites
Reads
0.0
0.5
1.0
1.5
2.0
2.5
3.0
0
200
400
600
800
gi393750638 position (nts)

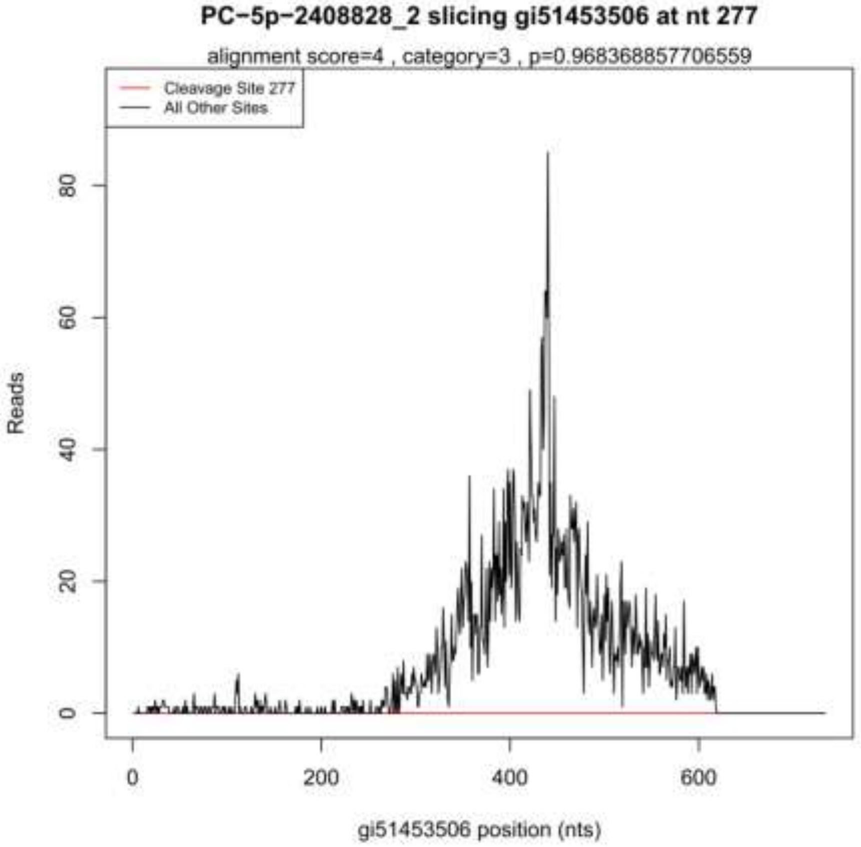
PC−5p−2408828_2 slicing gi51453506 at nt 277
alignment score=4 , category=3 , p=0.968368857706559
Cleavage Site 277
All Other Sites
Reads
0
20
40
60
80
0
200
400
600
gi51453506 position (nts)

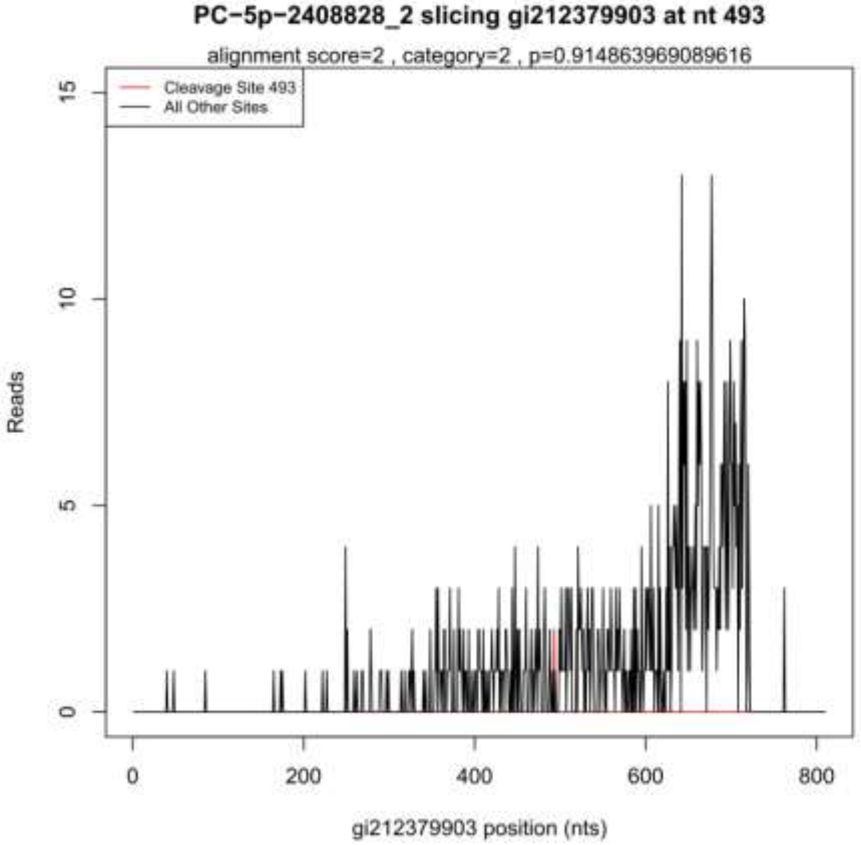
PC−5p−2408828_2 slicing gi212379903 at nt 493
alignment score=2 , category=2 , p=0.914863969089616
Cleavage Site 493
All Other Sites
Reads
0
5
10
15
0
200
400
600
800
gi212379903 position (nts)

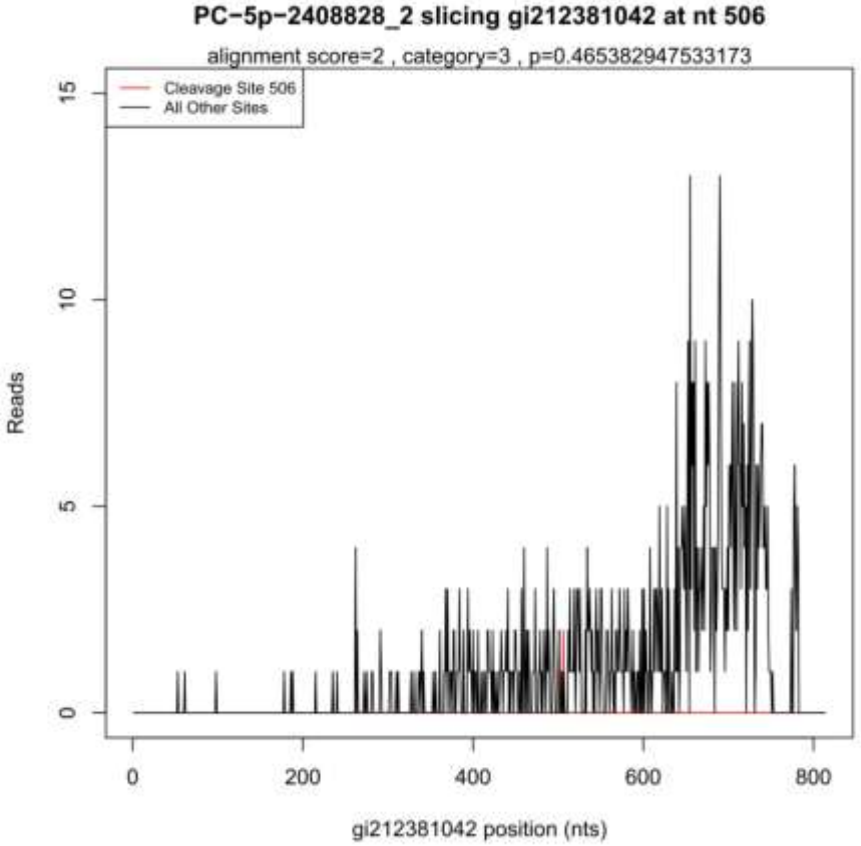
PC−5p−2408828_2 slicing gi212381042 at nt 506
alignment score=2 , category=3 , p=0.465382947533173
Cleavage Site 506
All Other Sites
Reads
0
5
10
15
0
200
400
600
800
gi212381042 position (nts)

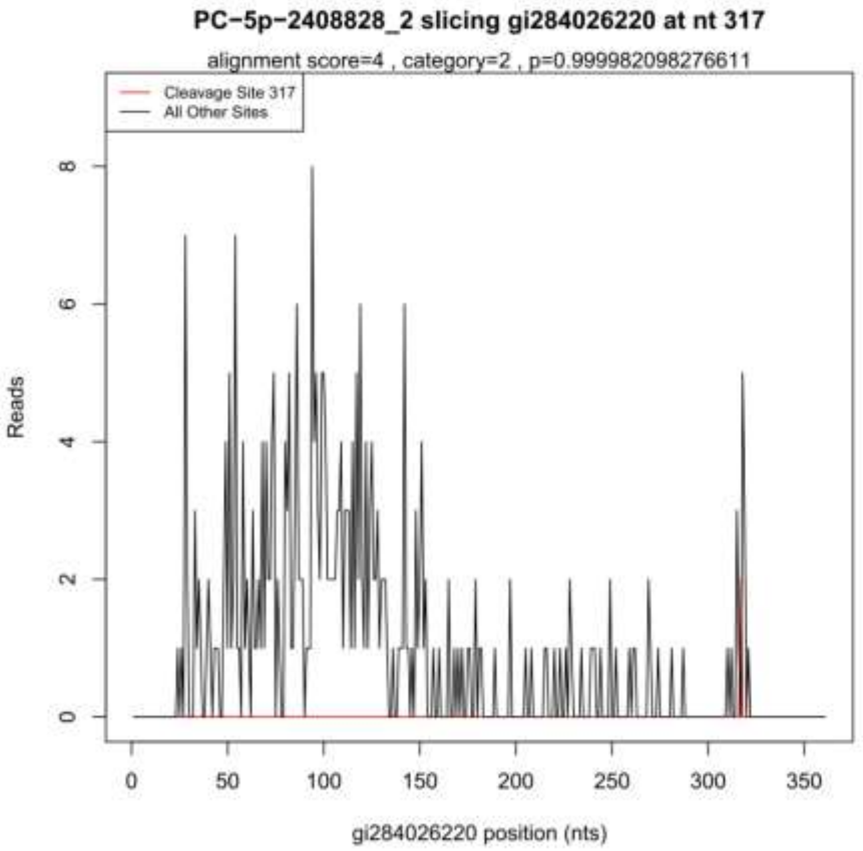
PC−5p−2408828_2 slicing gi284026220 at nt 317
alignment score=4 , category=2 , p=0.999982098276611
Cleavage Site 317
All Other Sites
Reads
0
2
4
6
8
0
50
100
150
200
250
300
350
gi284026220 position (nts)

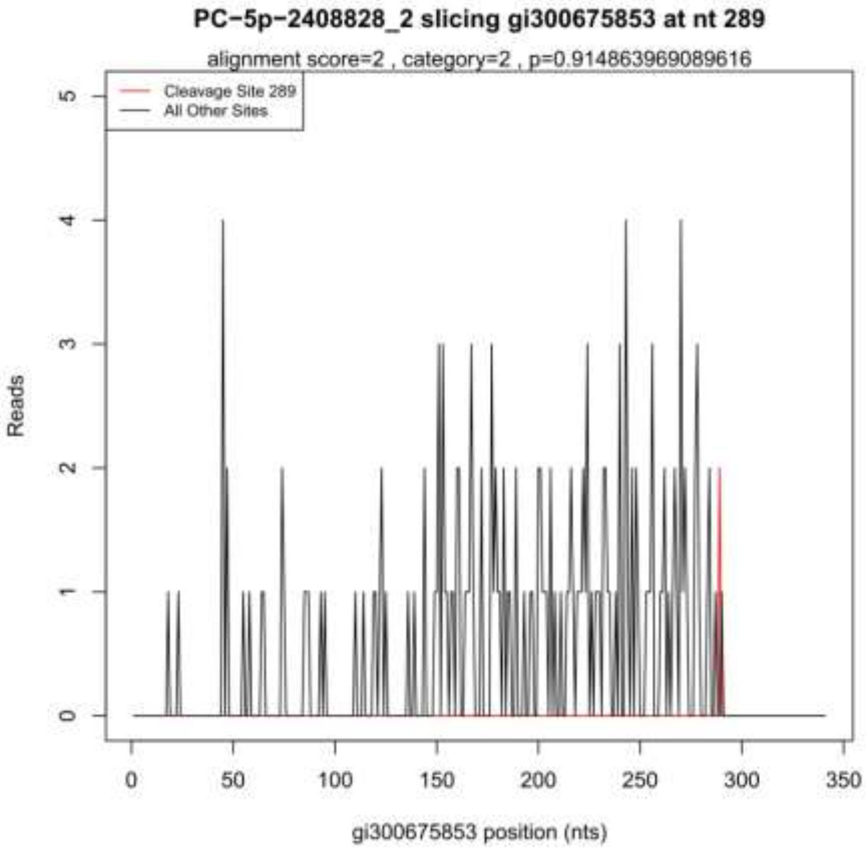
PC−5p−2408828_2 slicing gi300675853 at nt 289
alignment score=2 , category=2 , p=0.914863969089616
Cleavage Site 289
All Other Sites
Reads
0
1
2
3
4
5
0
50
100
150
200
250
300
350
gi300675853 position (nts)

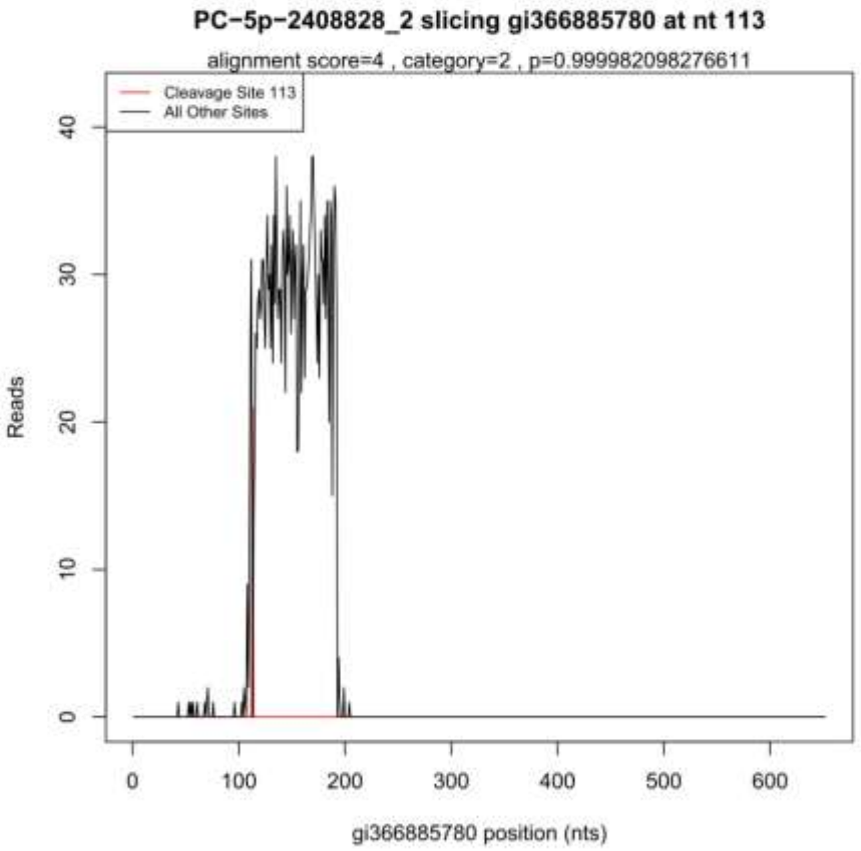
PC−5p−2408828_2 slicing gi366885780 at nt 113
alignment score=4 , category=2 , p=0.999982098276611
Cleavage Site 113
All Other Sites
Reads
gi366885780 position (nts)

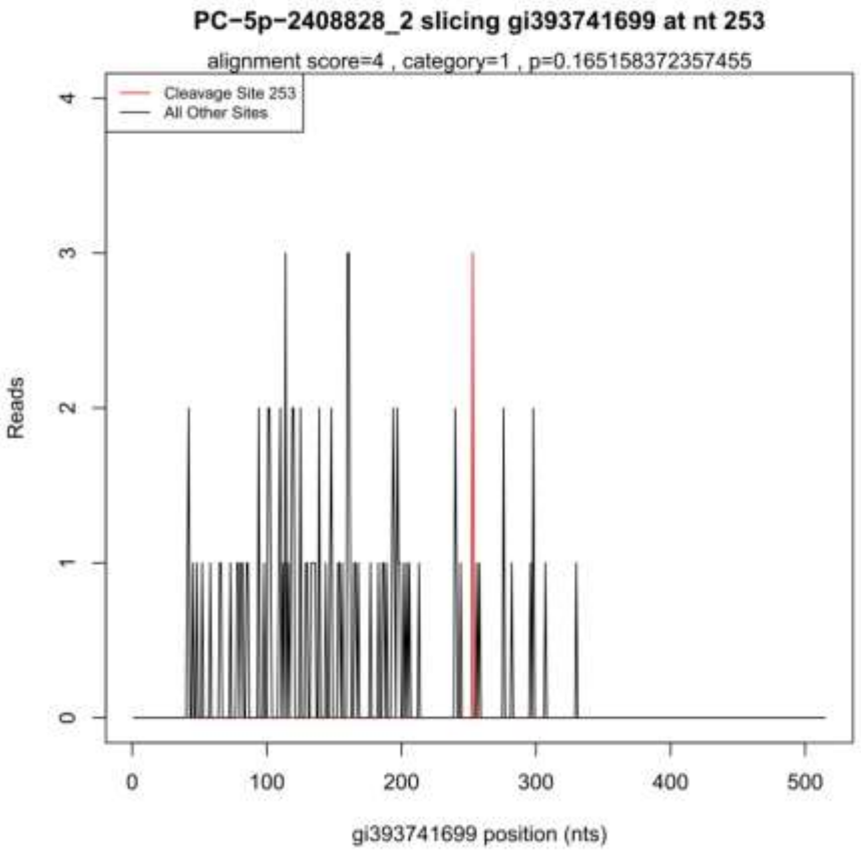
PC−5p−2408828_2 slicing gi393741699 at nt 253
alignment score=4 , category=1 , p=0.165158372357455
Cleavage Site 253
All Other Sites
Reads
gi393741699 position (nts)

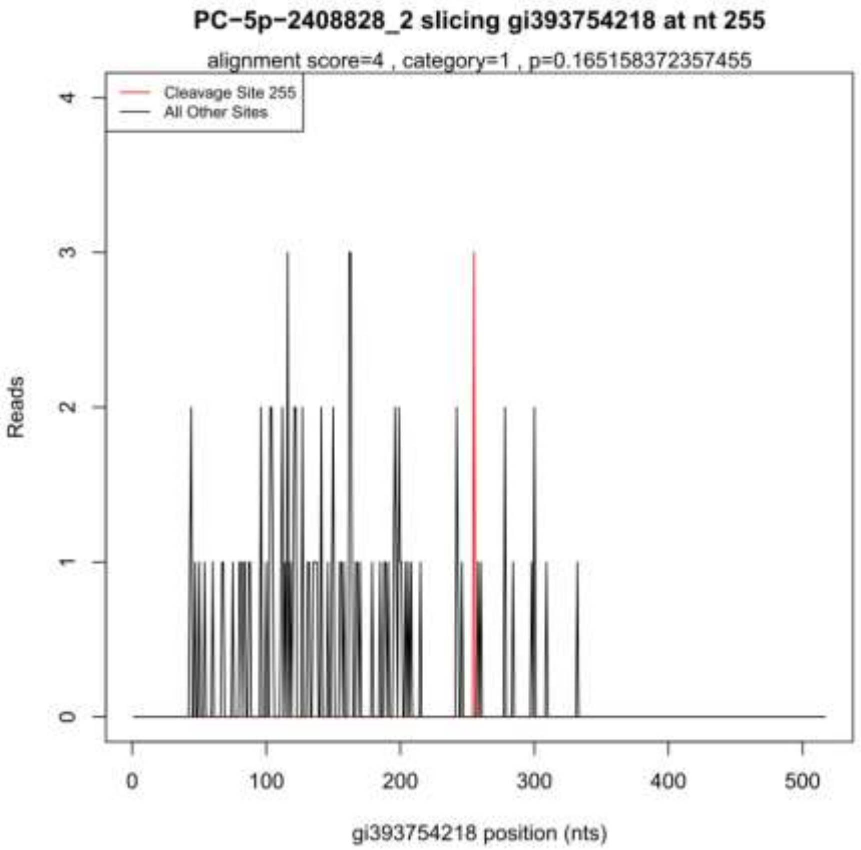
PC−5p−2408828_2 slicing gi393754218 at nt 255
alignment score=4 , category=1 , p=0.165158372357455
Cleavage Site 255
All Other Sites
Reads
gi393754218 position (nts)
0
1
2
3
4
0
100
200
300
400
500

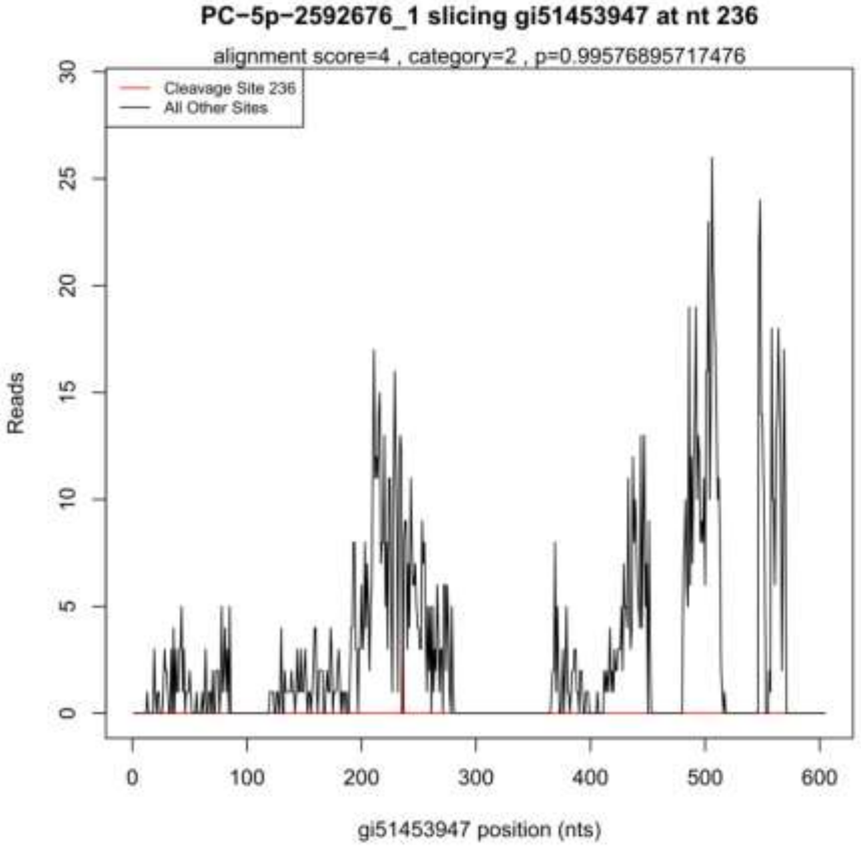
PC−5p−2592676_1 slicing gi51453947 at nt 236
alignment score=4 , category=2 , p=0.99576895717476
Cleavage Site 236
All Other Sites
Reads
gi51453947 position (nts)
0
5
10
15
20
25
30
0
100
200
300
400
500
600

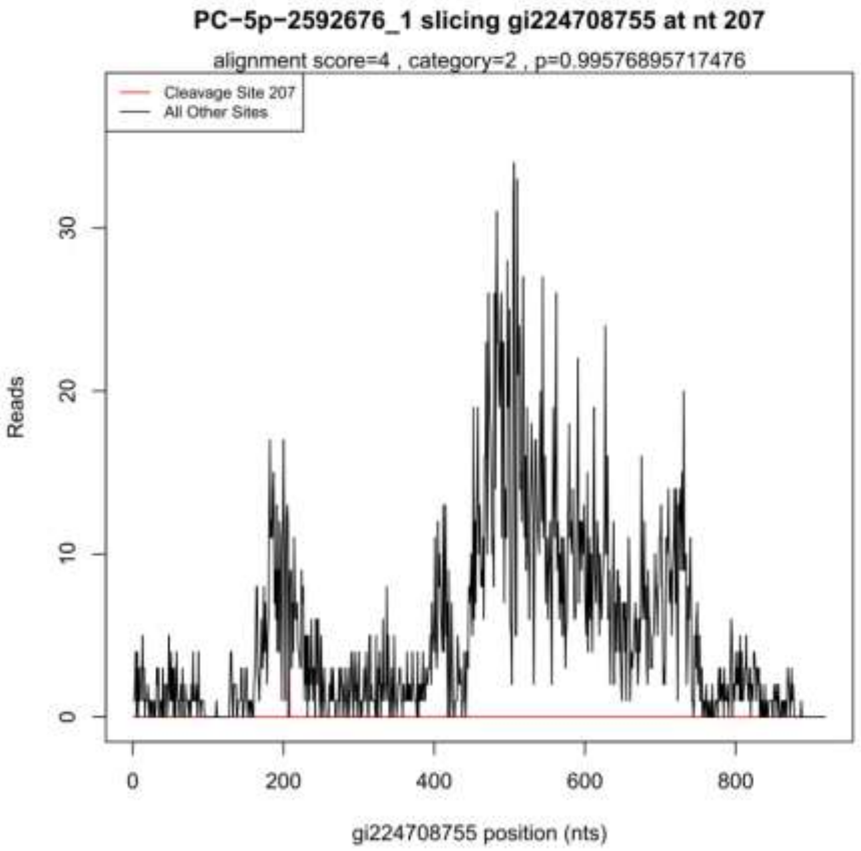
PC−5p−2592676_1 slicing gi224708755 at nt 207
alignment score=4 , category=2 , p=0.99576895717476
Cleavage Site 207
All Other Sites
Reads
0
10
20
30
0
200
400
600
800
gi224708755 position (nts)

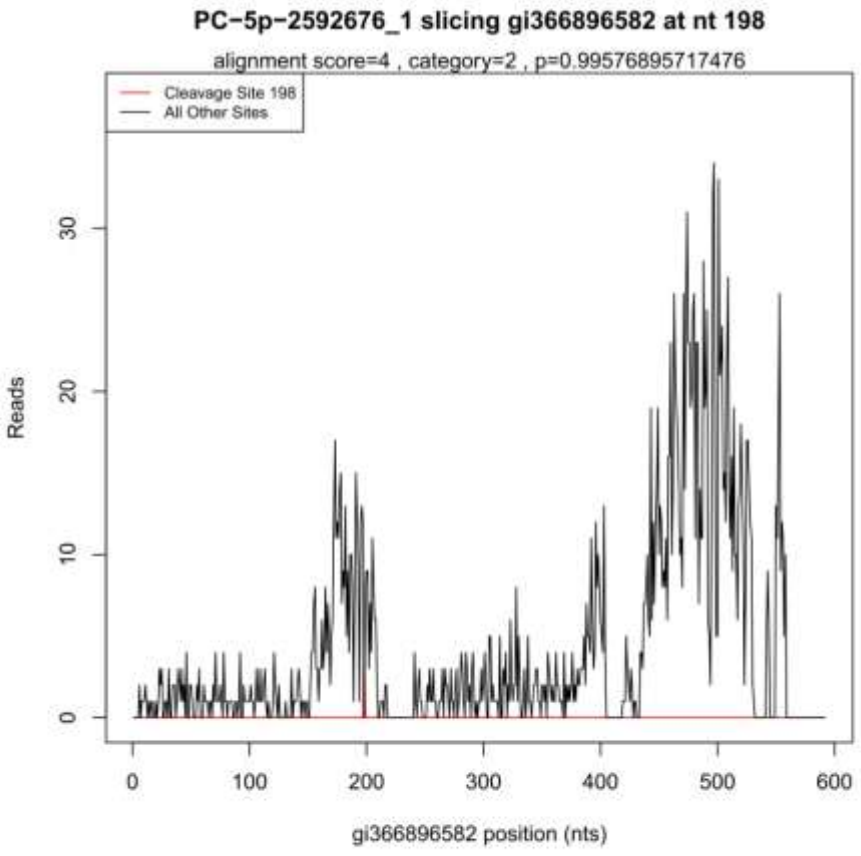
PC−5p−2592676_1 slicing gi366896582 at nt 198
alignment score=4 , category=2 , p=0.99576895717476
Cleavage Site 198
All Other Sites
Reads
0
10
20
30
0
100
200
300
400
500
600
gi366896582 position (nts)

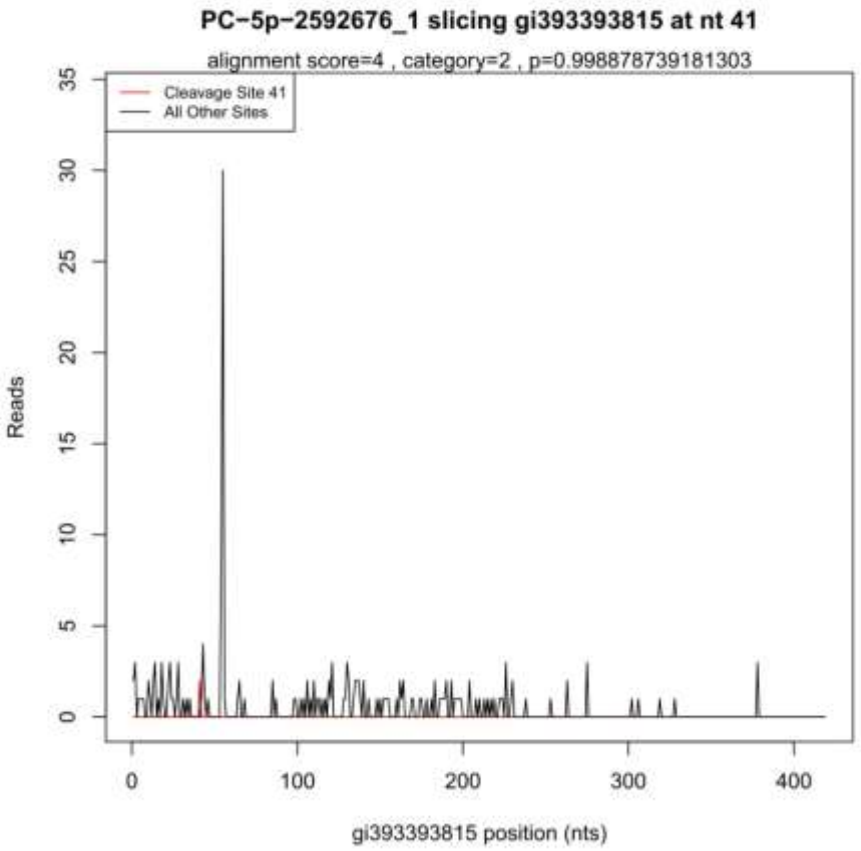
PC−5p−2592676_1 slicing gi393393815 at nt 41
alignment score=4 , category=2 , p=0.998878739181303
Cleavage Site 41
All Other Sites
Reads
gi393393815 position (nts)

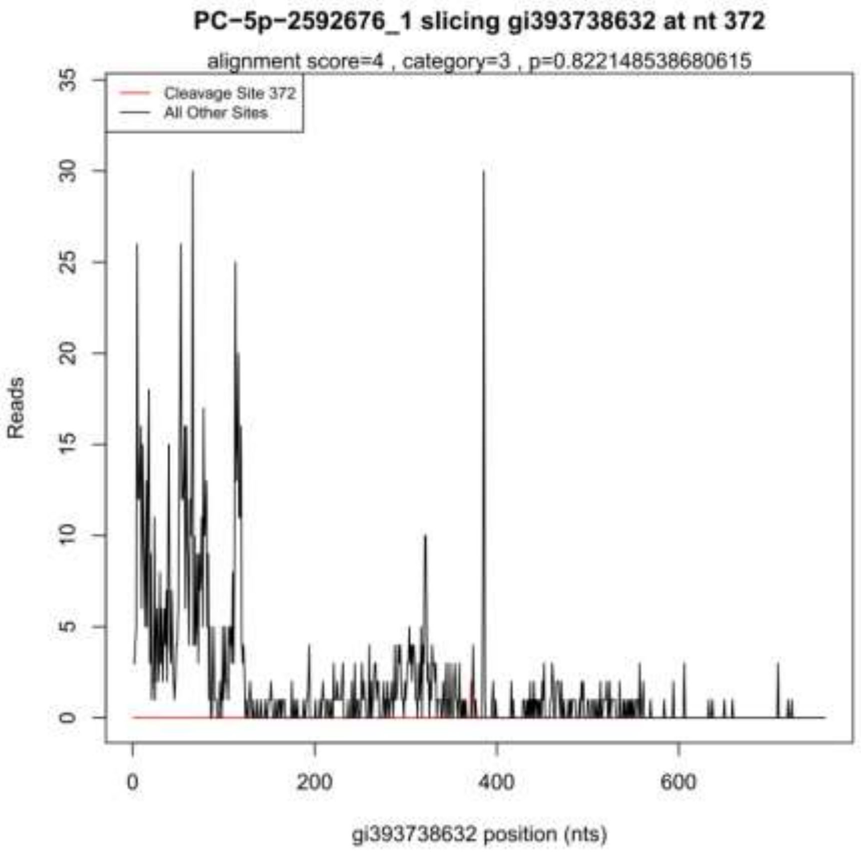
PC−5p−2592676_1 slicing gi393738632 at nt 372
alignment score=4 , category=3 , p=0.822148538680615
Cleavage Site 372
All Other Sites
Reads
gi393738632 position (nts)

PC−5p−2592676_1 slicing gi393738980 at nt 475

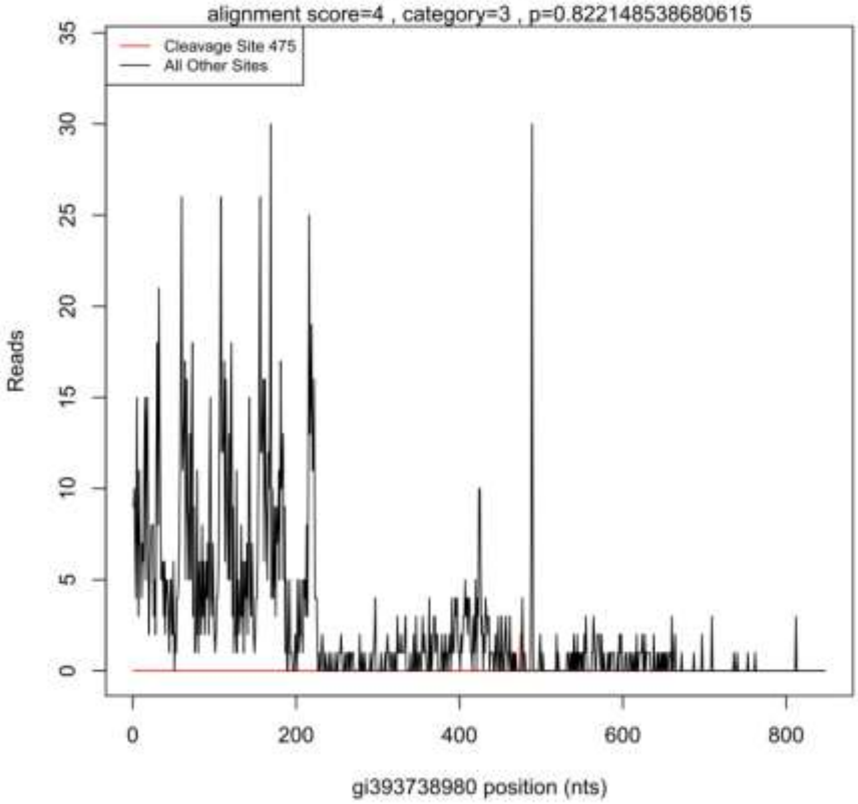

PC−5p−2592676_1 slicing gi393745957 at nt 181

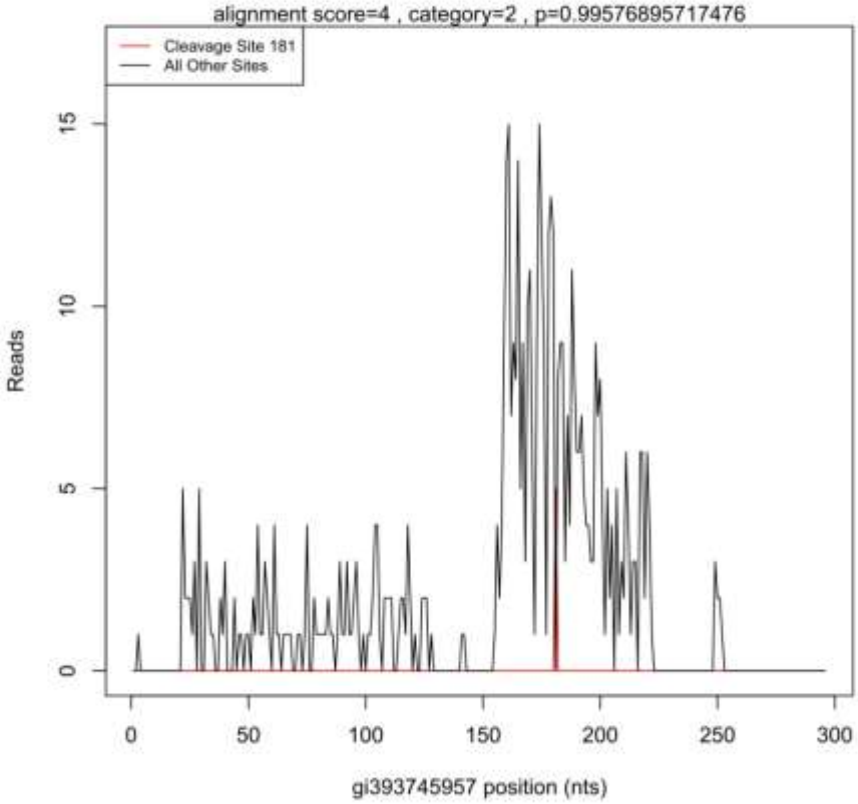

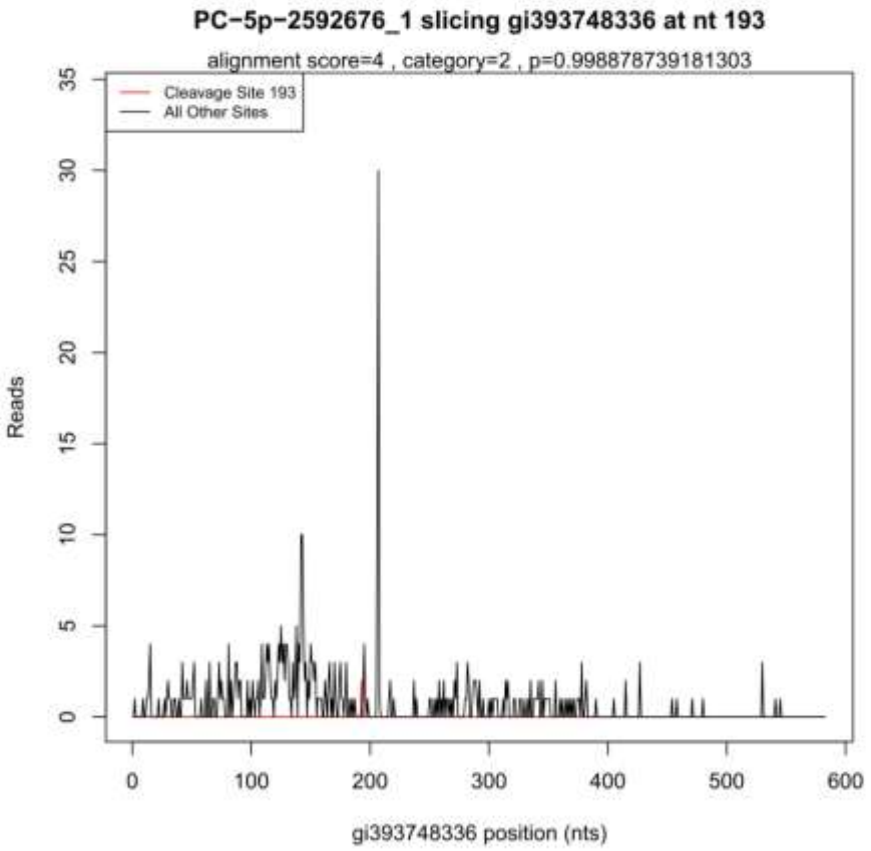

PC−5p−2592676_1 slicing gi393748336 at nt 193
alignment score=4 , category=2 , p=0.998878739181303
Cleavage Site 193
All Other Sites
Reads
gi393748336 position (nts)

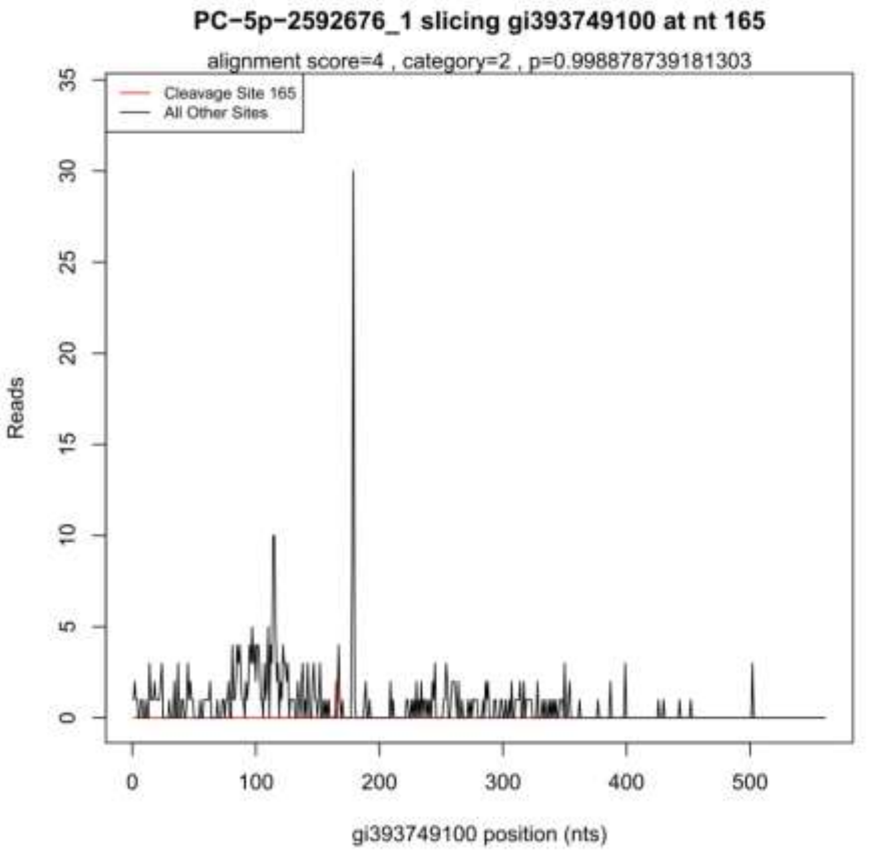

PC−5p−2592676_1 slicing gi393749100 at nt 165
alignment score=4 , category=2 , p=0.998878739181303
Cleavage Site 165
All Other Sites
Reads
gi393749100 position (nts)

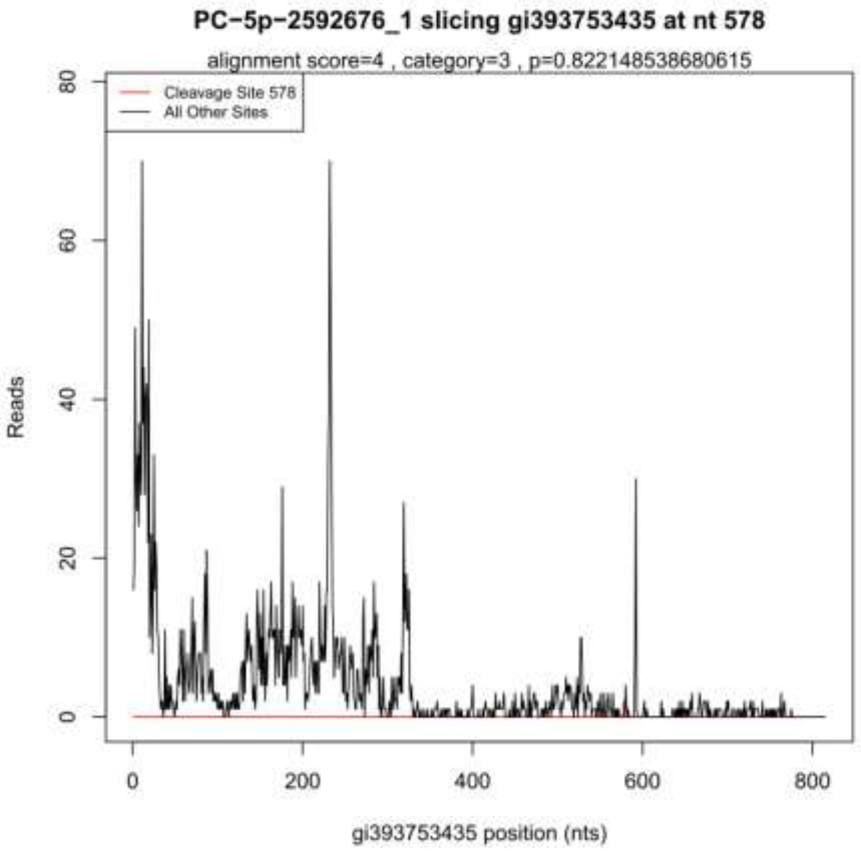
PC−5p−2592676_1 slicing gi393753435 at nt 578
alignment score=4 , category=3 , p=0.822148538680615
Cleavage Site 578
All Other Sites
Reads
0
20
40
60
80
0
200
400
600
800
gi393753435 position (nts)

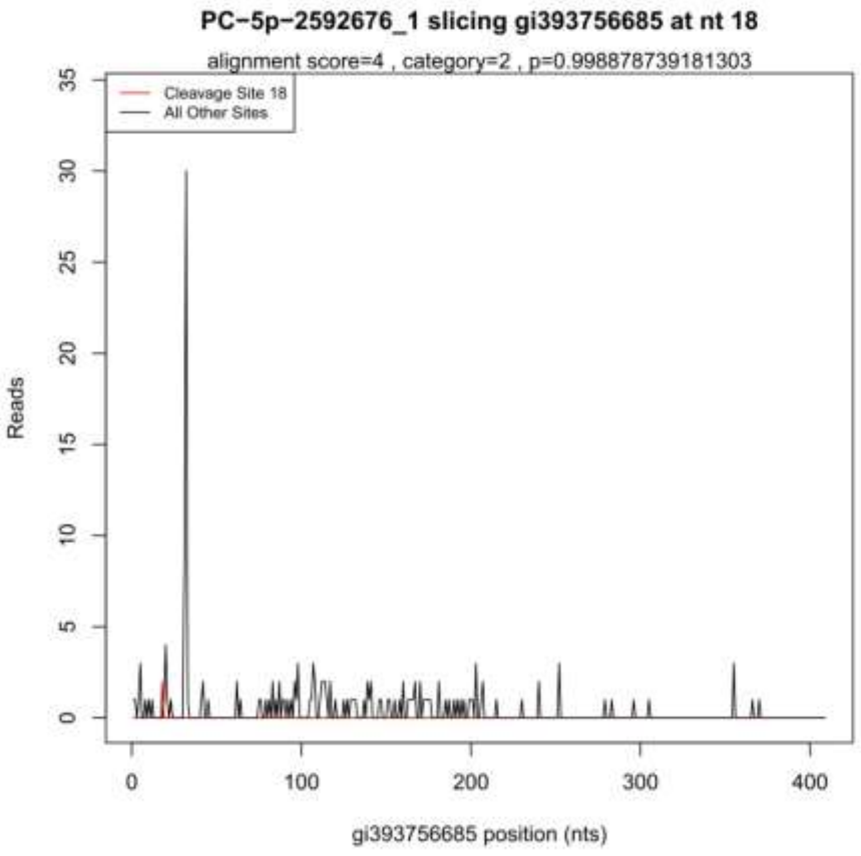
PC−5p−2592676_1 slicing gi393756685 at nt 18
alignment score=4 , category=2 , p=0.998878739181303
Cleavage Site 18
All Other Sites
Reads
0
5
10
15
20
25
30
35
0
100
200
300
400
gi393756685 position (nts)

PC-5p-2845188_1 slicing gi51452794 at nt 347

alignment score=2 , category=2 , p=0.999947464412046

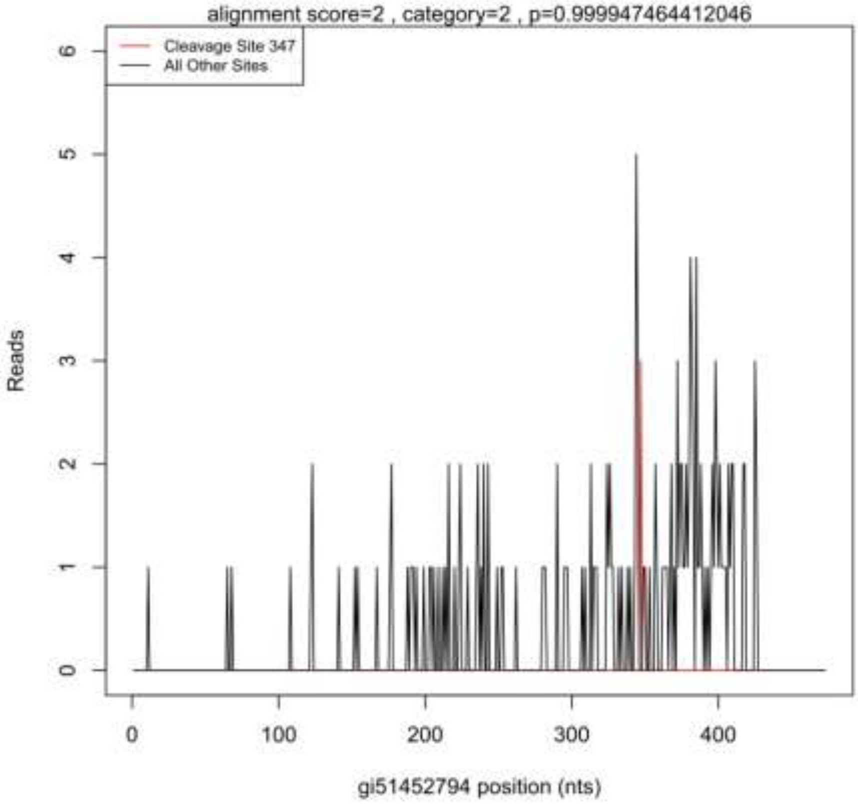

PC-5p-2845188_1 slicing gi51530274 at nt 214

alignment score=0 , category=2 , p=1

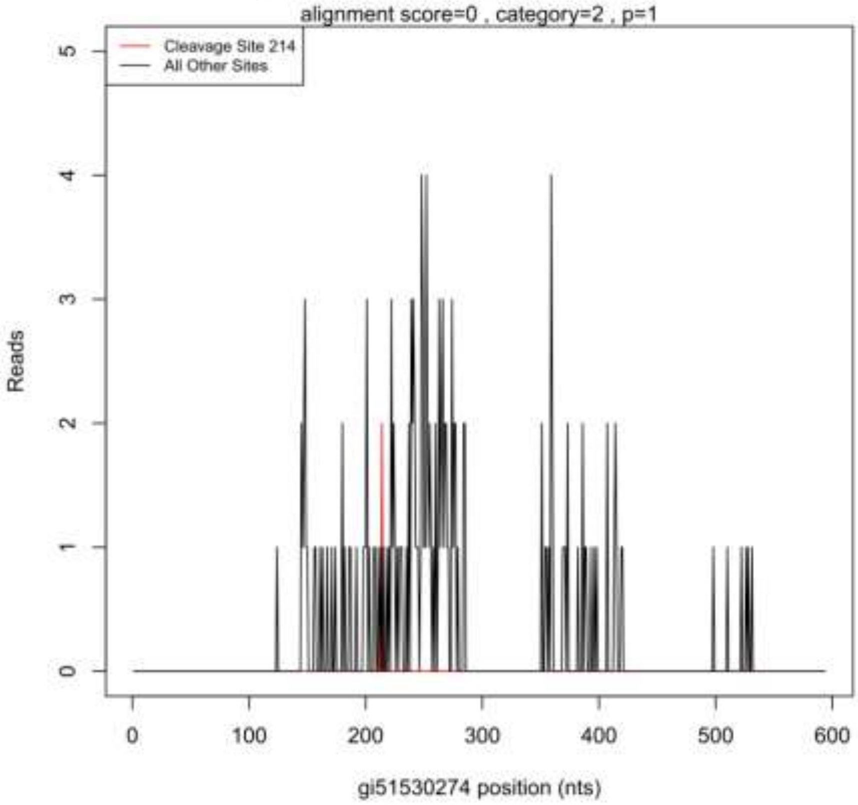

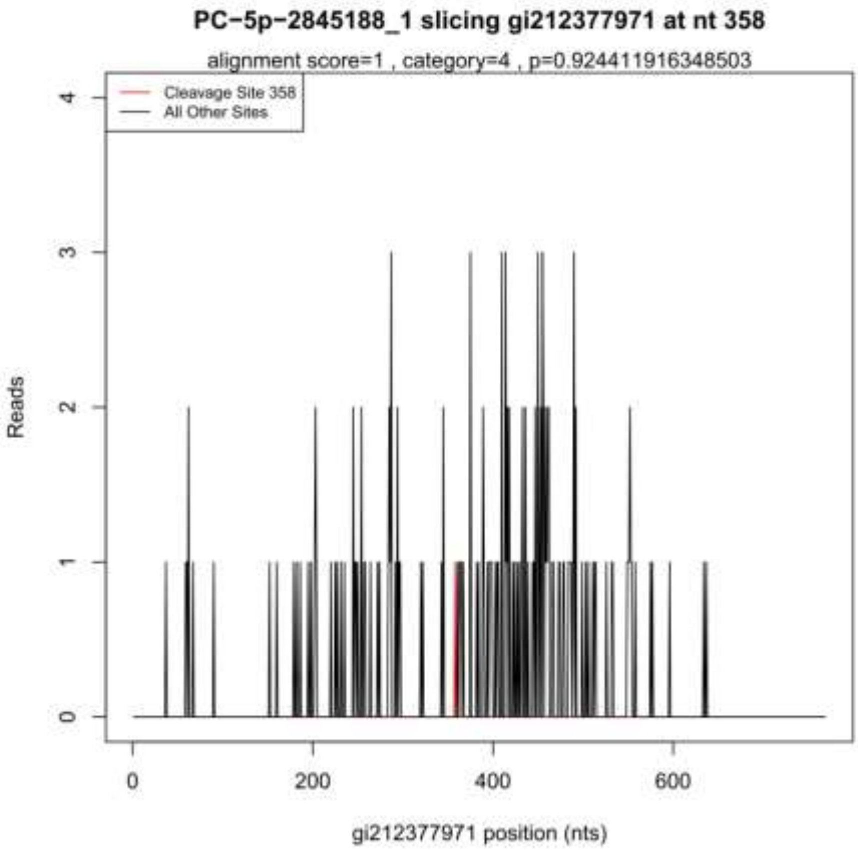
PC−5p−2845188_1 slicing gi212377971 at nt 358
alignment score=1 , category=4 , p=0.924411916348503
Cleavage Site 358
All Other Sites
Reads
gi212377971 position (nts)

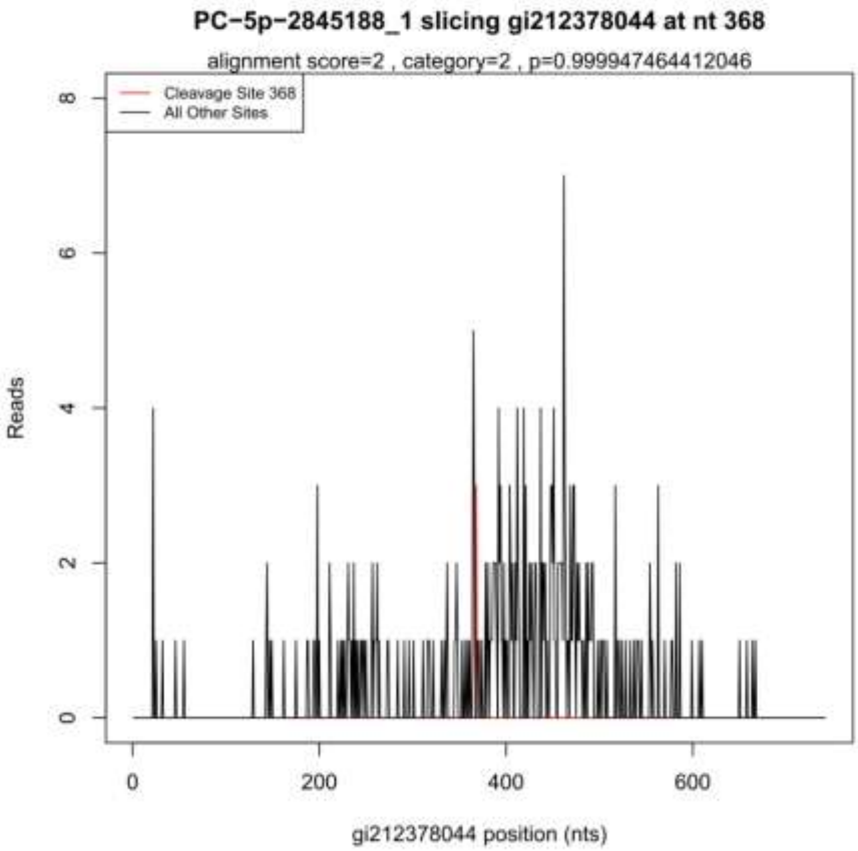
PC−5p−2845188_1 slicing gi212378044 at nt 368
alignment score=2 , category=2 , p=0.999947464412046
Cleavage Site 368
All Other Sites
Reads
gi212378044 position (nts)

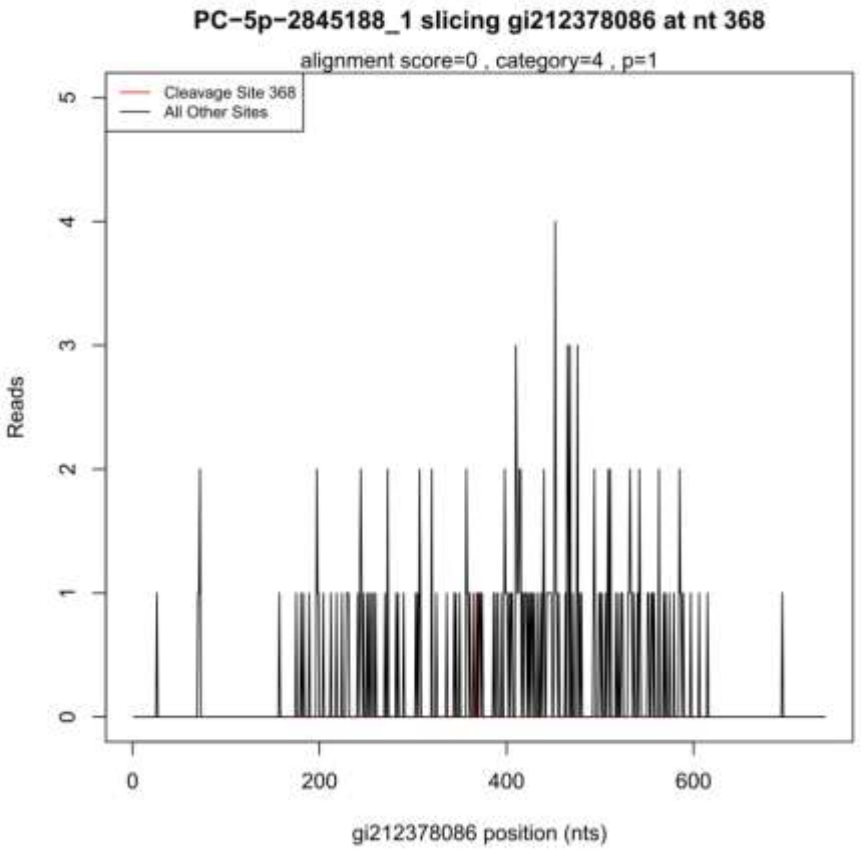
PC−5p−2845188_1 slicing gi212378086 at nt 368
alignment score=0 , category=4 , p=1
Cleavage Site 368
All Other Sites
Reads
0
1
2
3
4
5
0
200
400
600
gi212378086 position (nts)

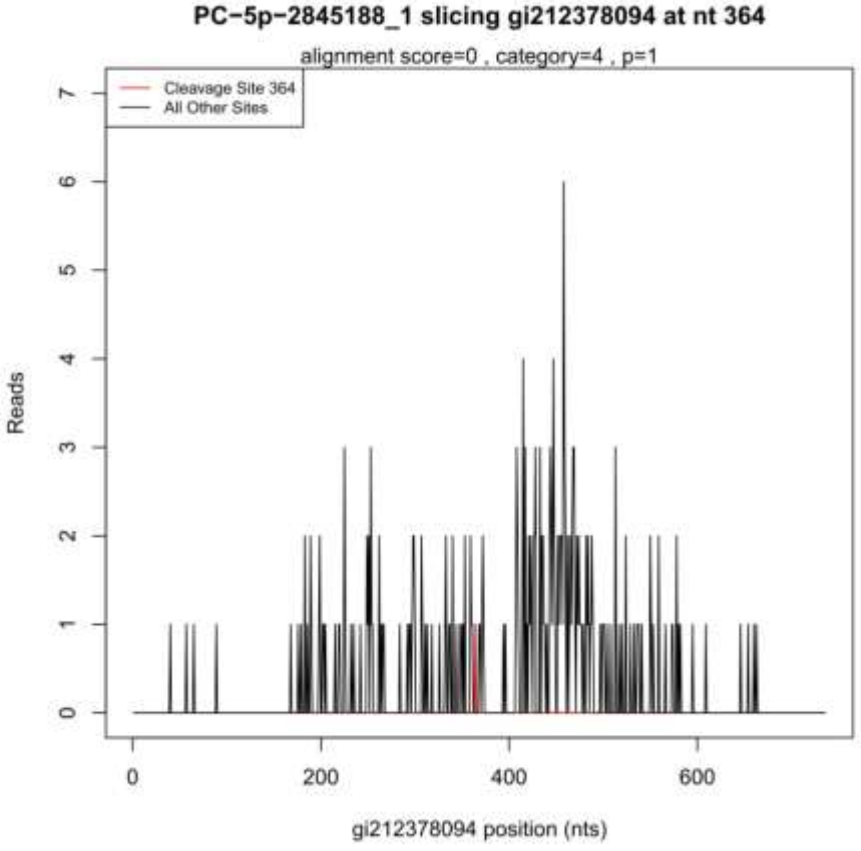
PC−5p−2845188_1 slicing gi212378094 at nt 364
alignment score=0 , category=4 , p=1
Cleavage Site 364
All Other Sites
Reads
0
1
2
3
4
5
6
7
0
200
400
600
gi212378094 position (nts)

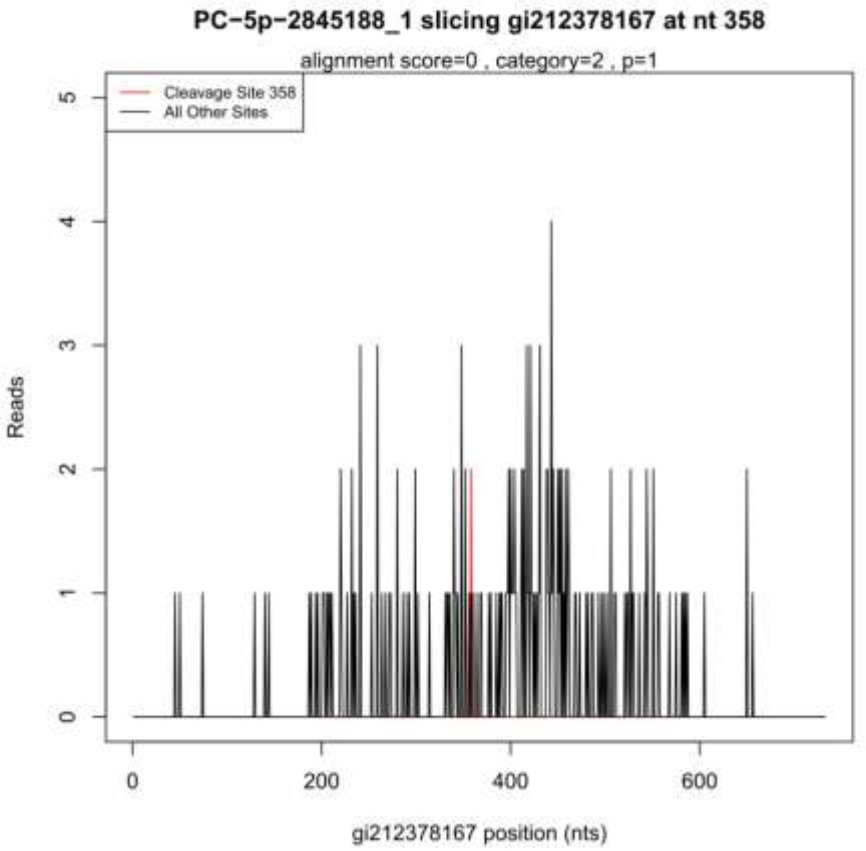
PC−5p−2845188_1 slicing gi212378167 at nt 358
alignment score=0 , category=2 , p=1
Cleavage Site 358
All Other Sites
Reads
0
1
2
3
4
5
0
200
400
600
gi212378167 position (nts)

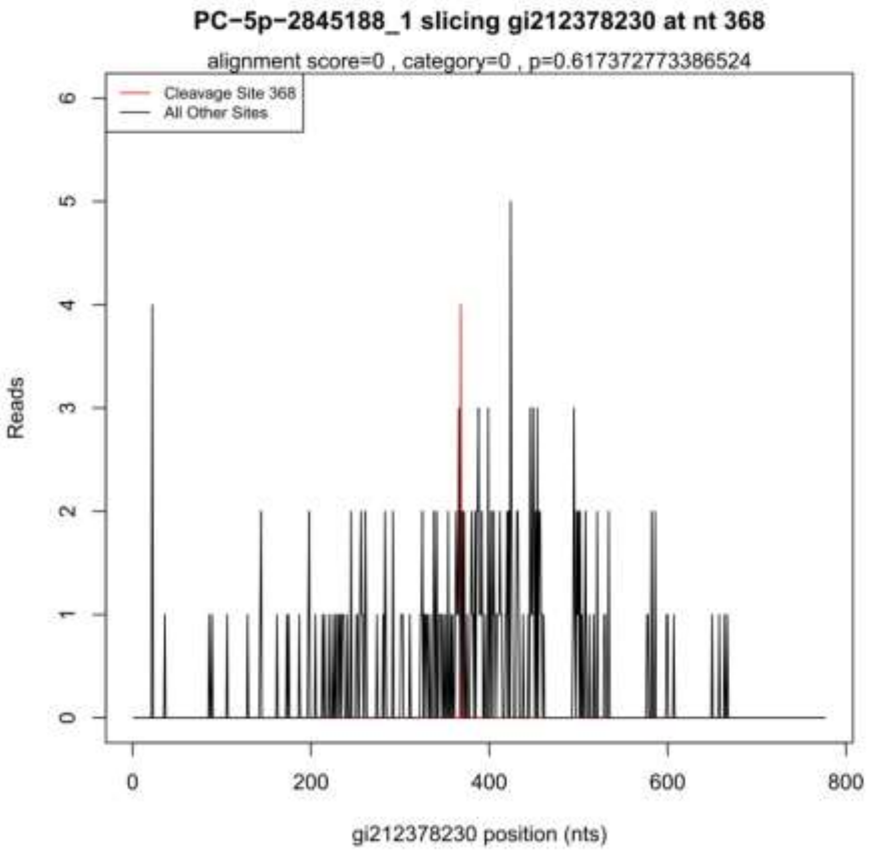
PC−5p−2845188_1 slicing gi212378230 at nt 368
alignment score=0 , category=0 , p=0.617372773386524
Cleavage Site 368
All Other Sites
Reads
0
1
2
3
4
5
6
0
200
400
600
800
gi212378230 position (nts)

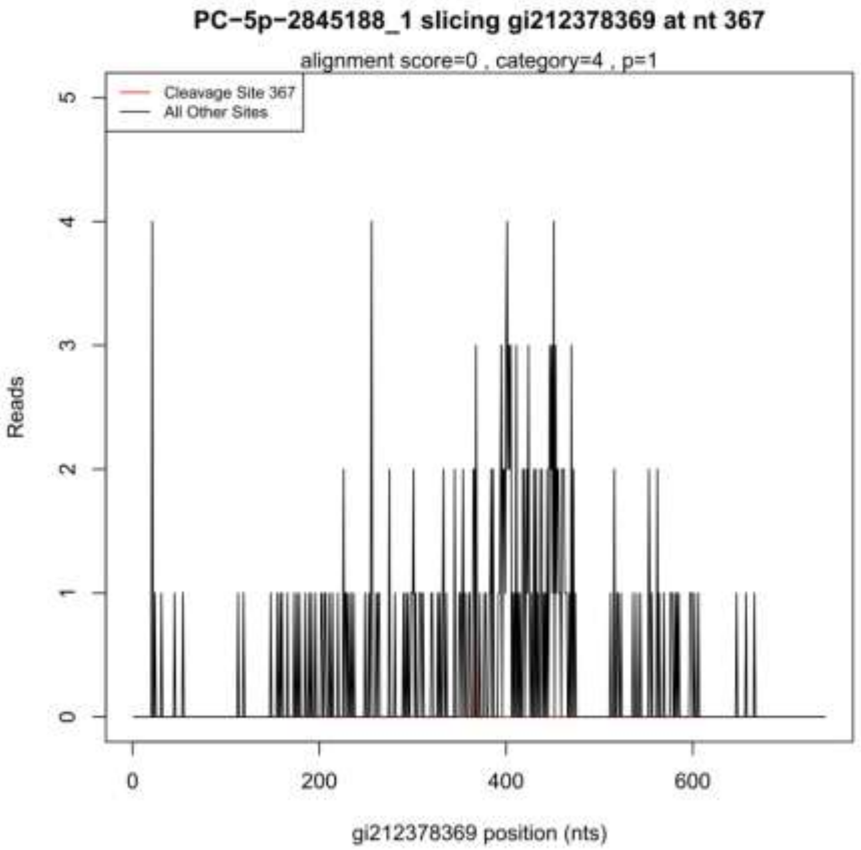
PC−5p−2845188_1 slicing gi212378369 at nt 367
alignment score=0 , category=4 , p=1
Cleavage Site 367
All Other Sites
Reads
0
1
2
3
4
5
0
200
400
600
gi212378369 position (nts)

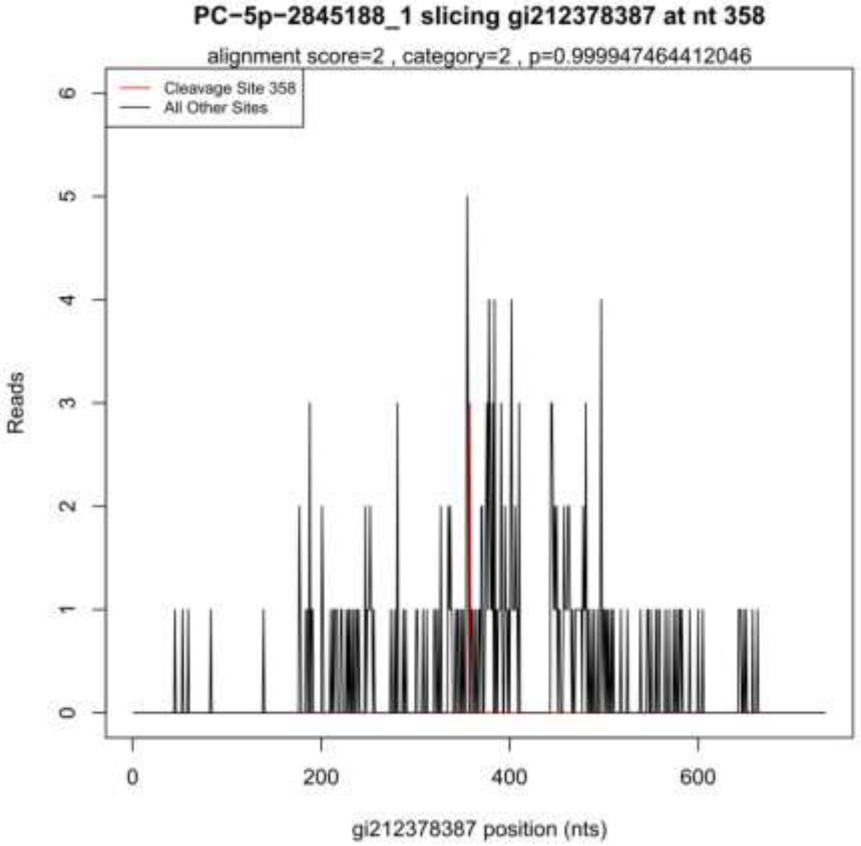
PC−5p−2845188_1 slicing gi212378387 at nt 358
alignment score=2 , category=2 , p=0.999947464412046
Cleavage Site 358
All Other Sites
Reads
0
1
2
3
4
5
6
0
200
400
600
gi212378387 position (nts)

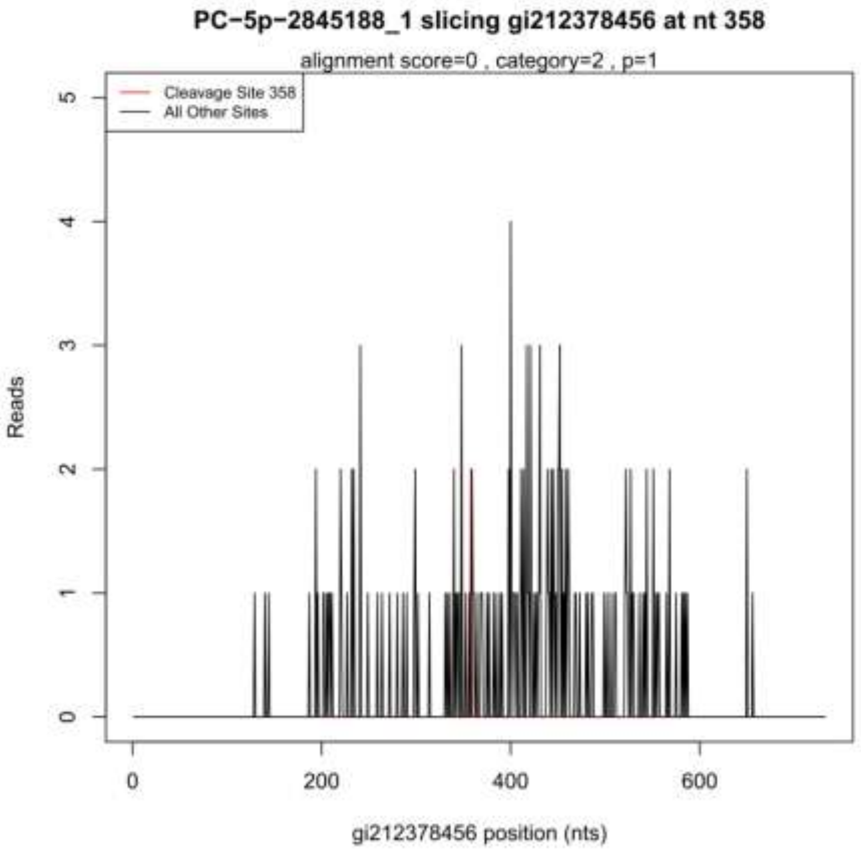
PC−5p−2845188_1 slicing gi212378456 at nt 358
alignment score=0 , category=2 , p=1
Cleavage Site 358
All Other Sites
Reads
0
1
2
3
4
5
0
200
400
600
gi212378456 position (nts)

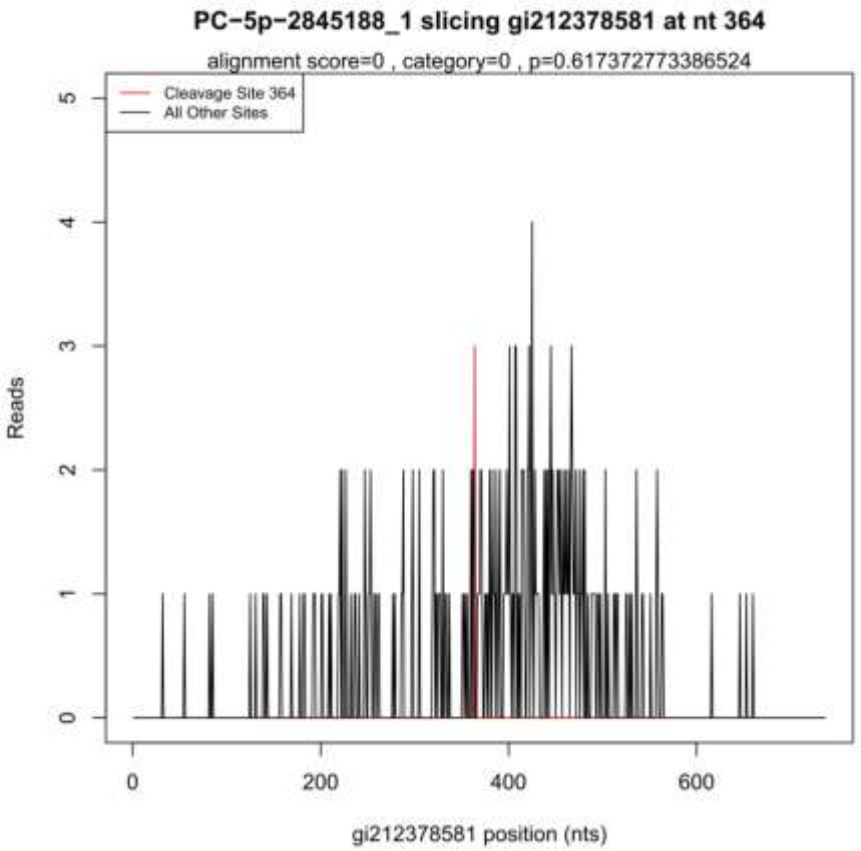
PC−5p−2845188_1 slicing gi212378581 at nt 364
alignment score=0 , category=0 , p=0.617372773386524
Cleavage Site 364
All Other Sites
Reads
0
1
2
3
4
5
0
200
400
600
gi212378581 position (nts)

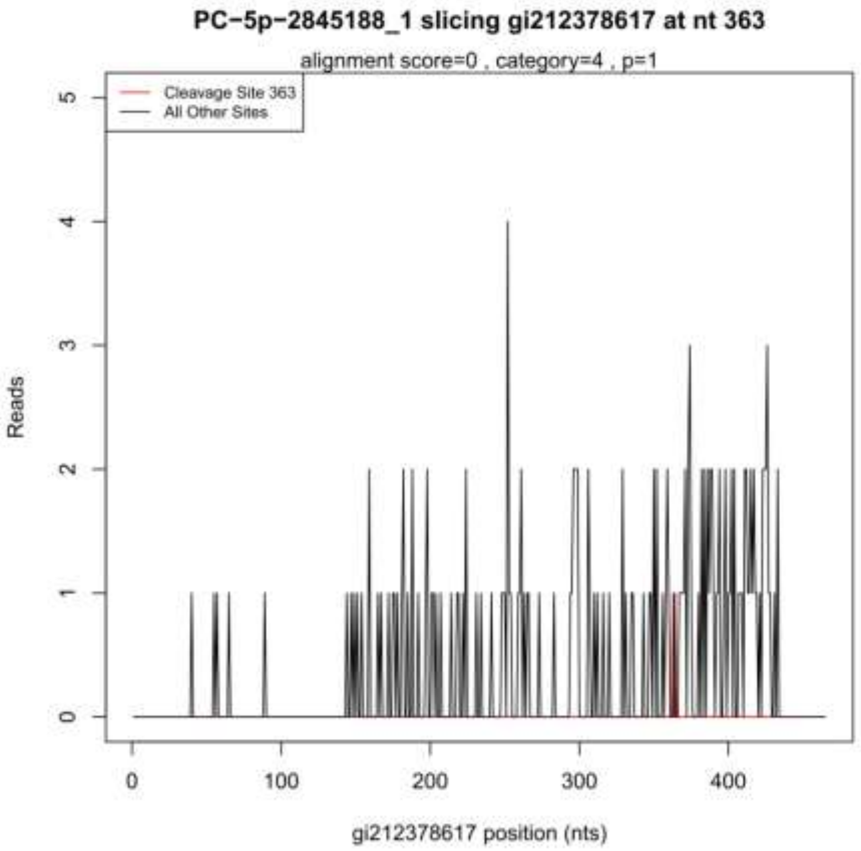
PC−5p−2845188_1 slicing gi212378617 at nt 363
alignment score=0 , category=4 , p=1
Cleavage Site 363
All Other Sites
Reads
gi212378617 position (nts)

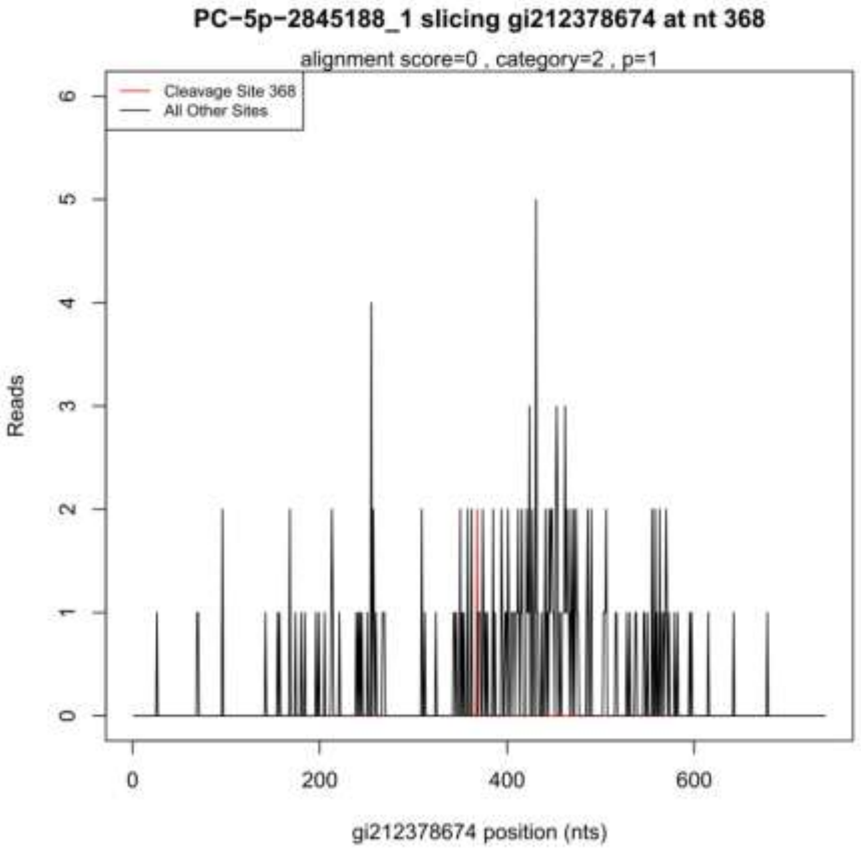
PC−5p−2845188_1 slicing gi212378674 at nt 368
alignment score=0 , category=2 , p=1
Cleavage Site 368
All Other Sites
Reads
gi212378674 position (nts)

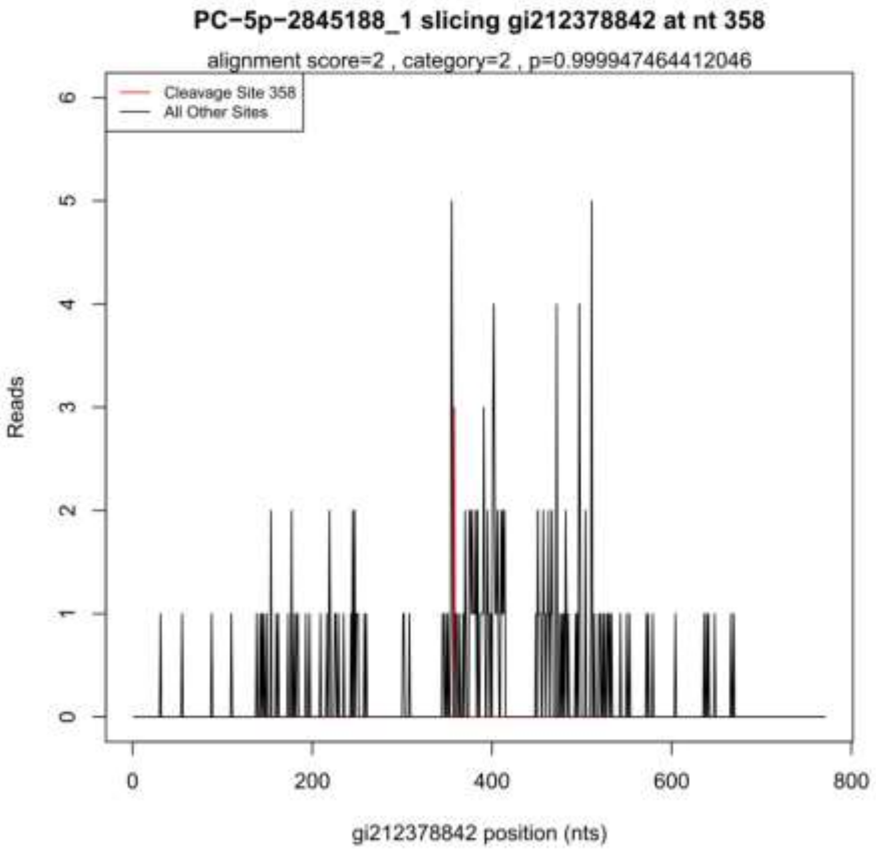

PC−5p−2845188_1 slicing gi212378842 at nt 358
alignment score=2 , category=2 , p=0.999947464412046
Cleavage Site 358
All Other Sites
Reads
0
1
2
3
4
5
6
0
200
400
600
800
gi212378842 position (nts)

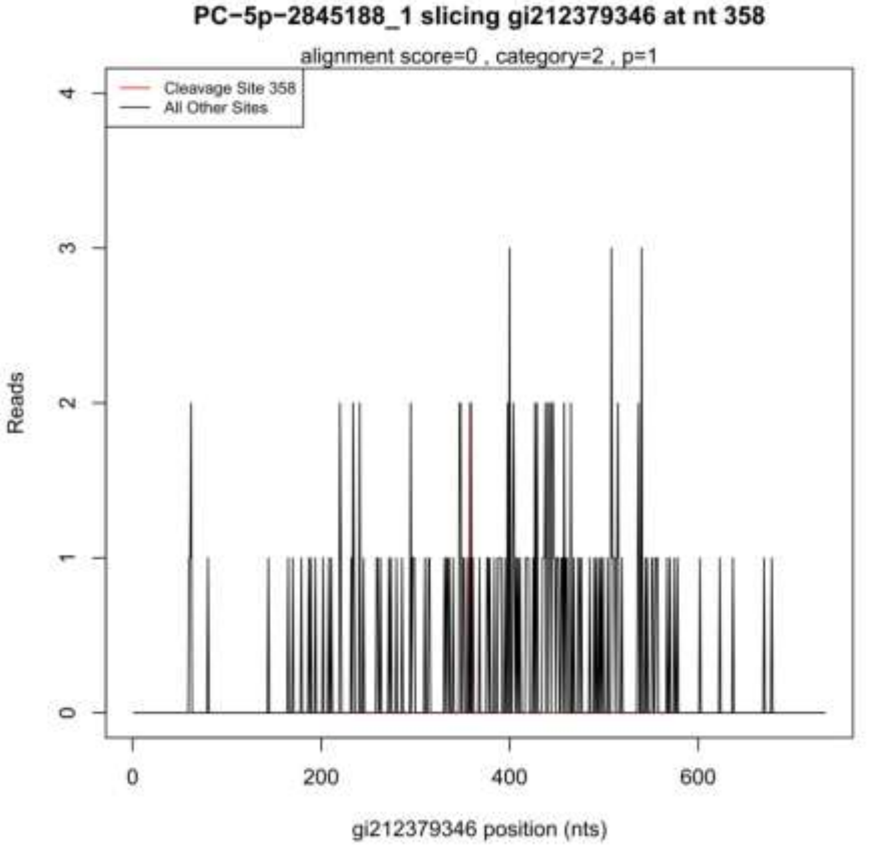

PC−5p−2845188_1 slicing gi212379346 at nt 358
alignment score=0 , category=2 , p=1
Cleavage Site 358
All Other Sites
Reads
0
1
2
3
4
0
200
400
600
gi212379346 position (nts)

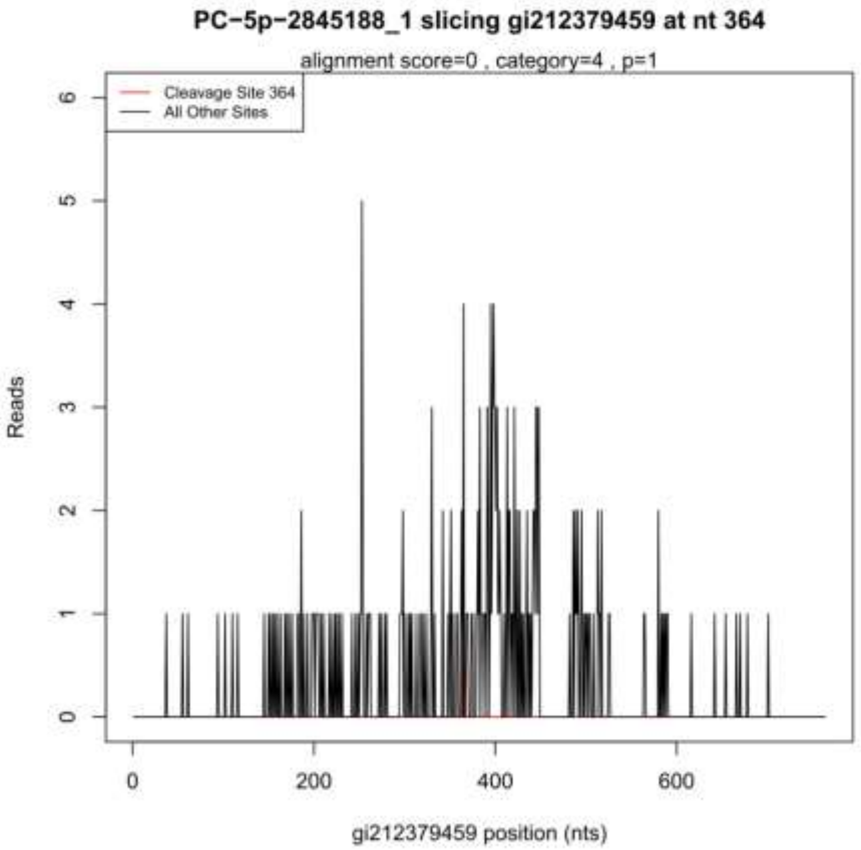
PC-5p-2845188_1 slicing gi212379459 at nt 364
alignment score=0 , category=4 , p=1
Cleavage Site 364
All Other Sites
Reads
0
1
2
3
4
5
6
0
200
400
600
gi212379459 position (nts)

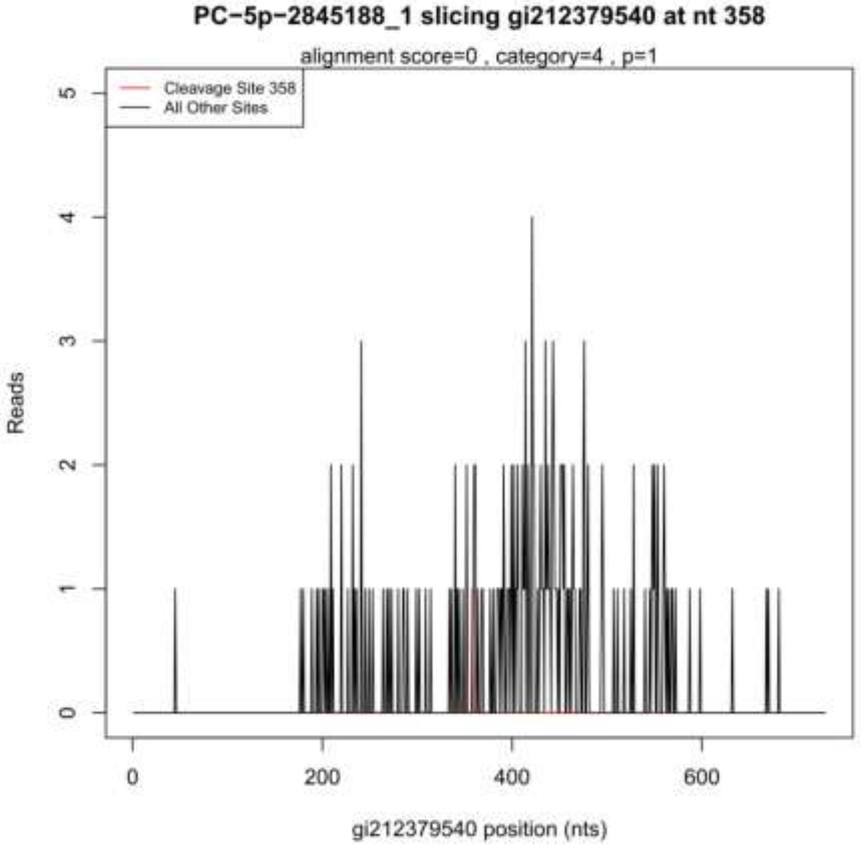
PC-5p-2845188_1 slicing gi212379540 at nt 358
alignment score=0 , category=4 , p=1
Cleavage Site 358
All Other Sites
Reads
0
1
2
3
4
5
0
200
400
600
gi212379540 position (nts)

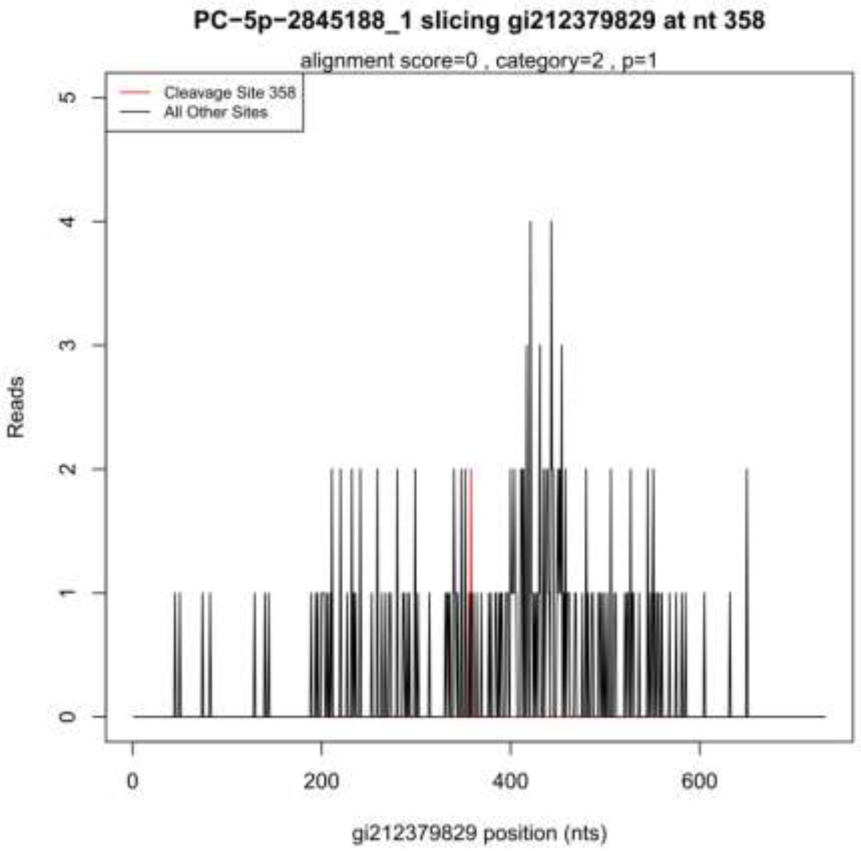
PC−5p−2845188_1 slicing gi212379829 at nt 358
alignment score=0 , category=2 , p=1
Cleavage Site 358
All Other Sites
Reads
gi212379829 position (nts)

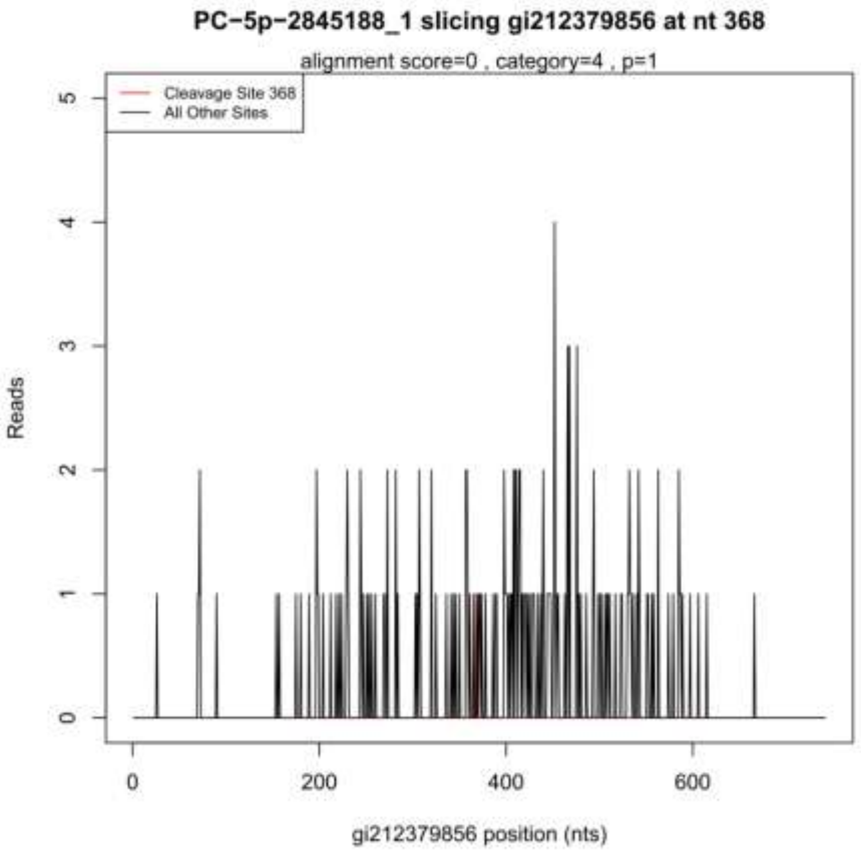
PC−5p−2845188_1 slicing gi212379856 at nt 368
alignment score=0 , category=4 , p=1
Cleavage Site 368
All Other Sites
Reads
gi212379856 position (nts)

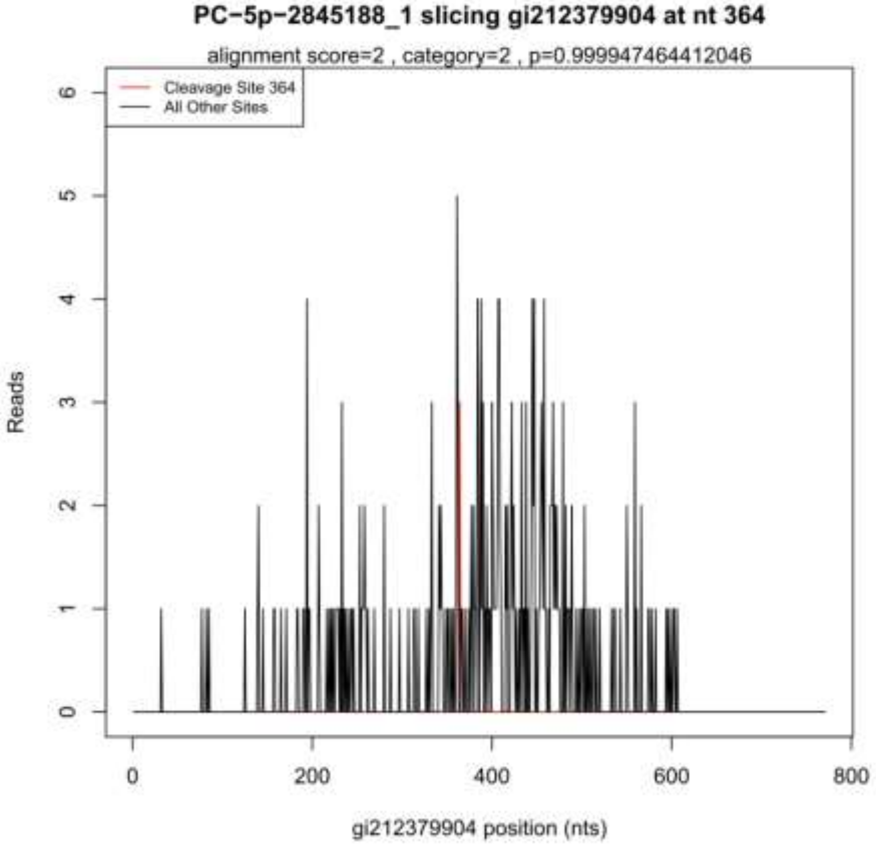

PC−5p−2845188_1 slicing gi212379904 at nt 364
alignment score=2 , category=2 , p=0.999947464412046
Cleavage Site 364
All Other Sites
Reads
0
1
2
3
4
5
6
0
200
400
600
800
gi212379904 position (nts)

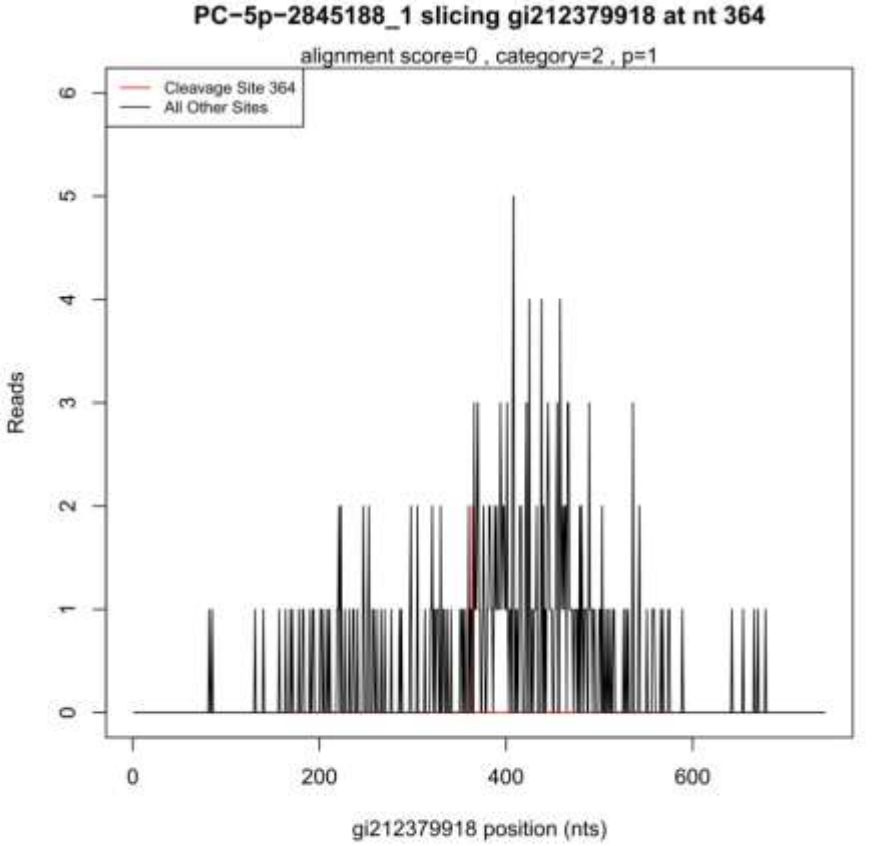

PC−5p−2845188_1 slicing gi212379918 at nt 364
alignment score=0 , category=2 , p=1
Cleavage Site 364
All Other Sites
Reads
0
1
2
3
4
5
6
0
200
400
600
gi212379918 position (nts)

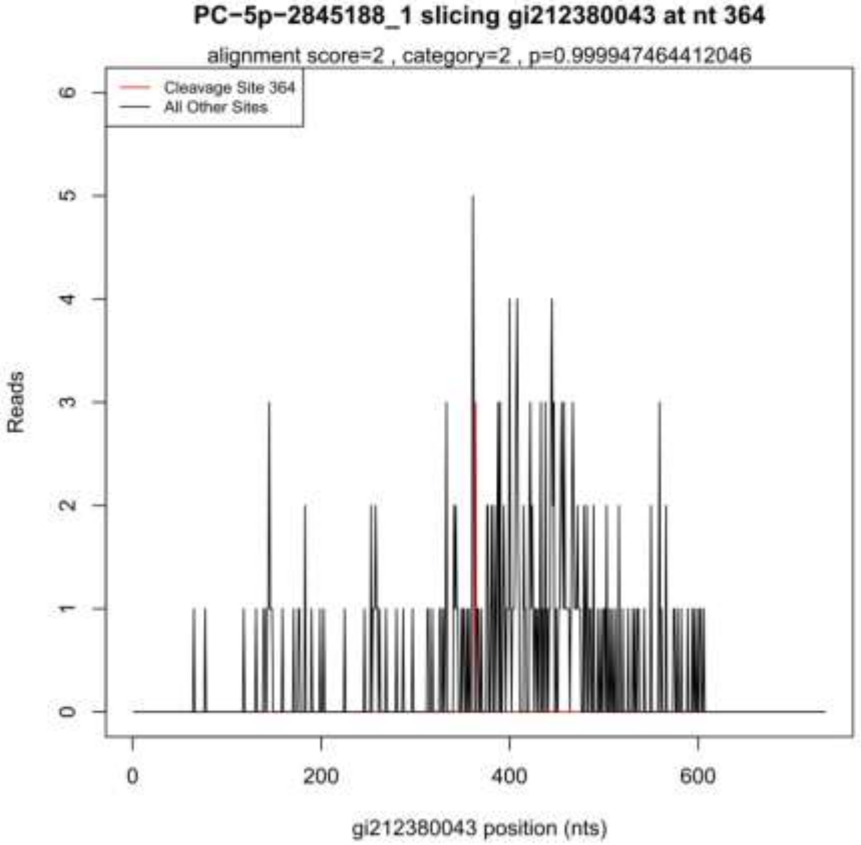
PC−5p−2845188_1 slicing gi212380043 at nt 364
alignment score=2 , category=2 , p=0.999947464412046
Cleavage Site 364
All Other Sites
Reads
gi212380043 position (nts)

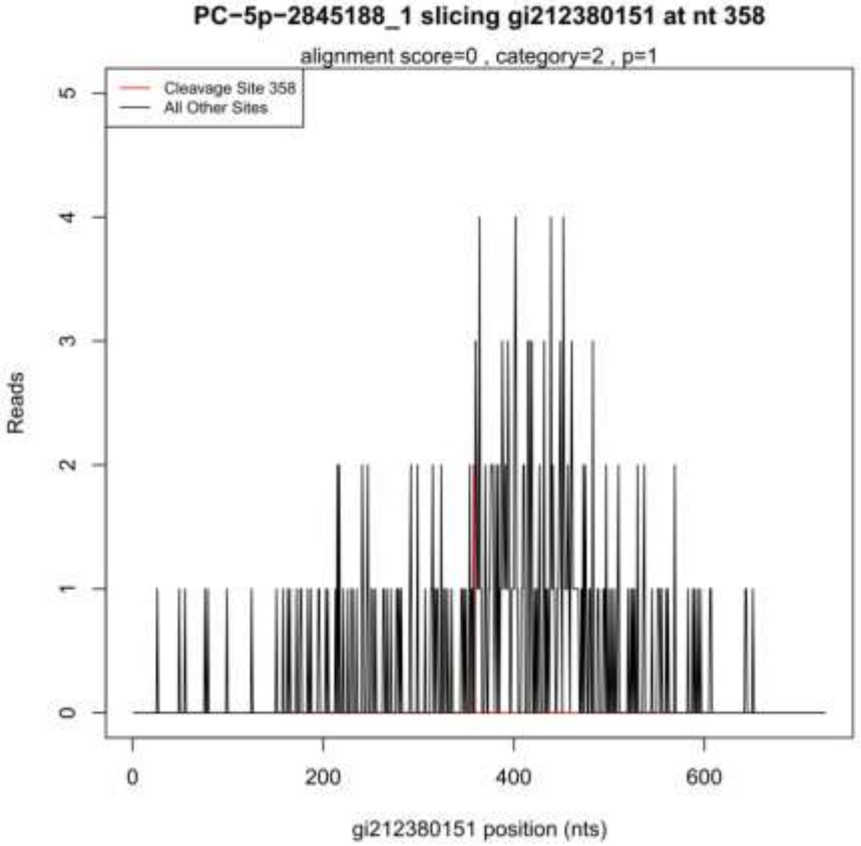
PC−5p−2845188_1 slicing gi212380151 at nt 358
alignment score=0 , category=2 , p=1
Cleavage Site 358
All Other Sites
Reads
gi212380151 position (nts)

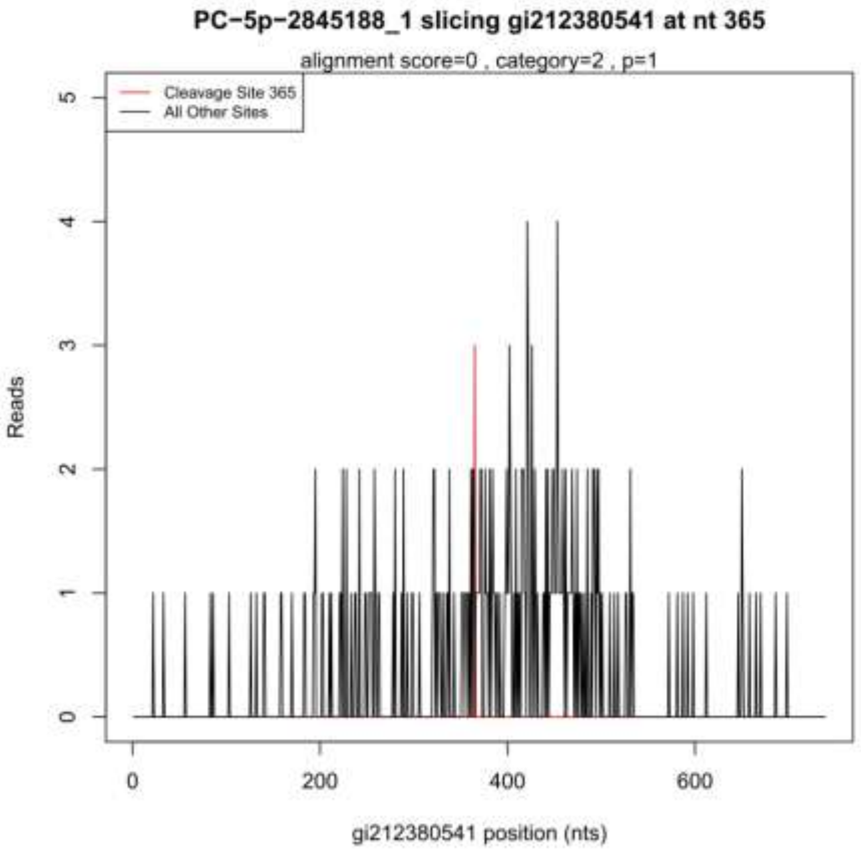
PC-5p-2845188_1 slicing gi212380541 at nt 365
alignment score=0 , category=2 , p=1
Cleavage Site 365
All Other Sites
Reads
gi212380541 position (nts)

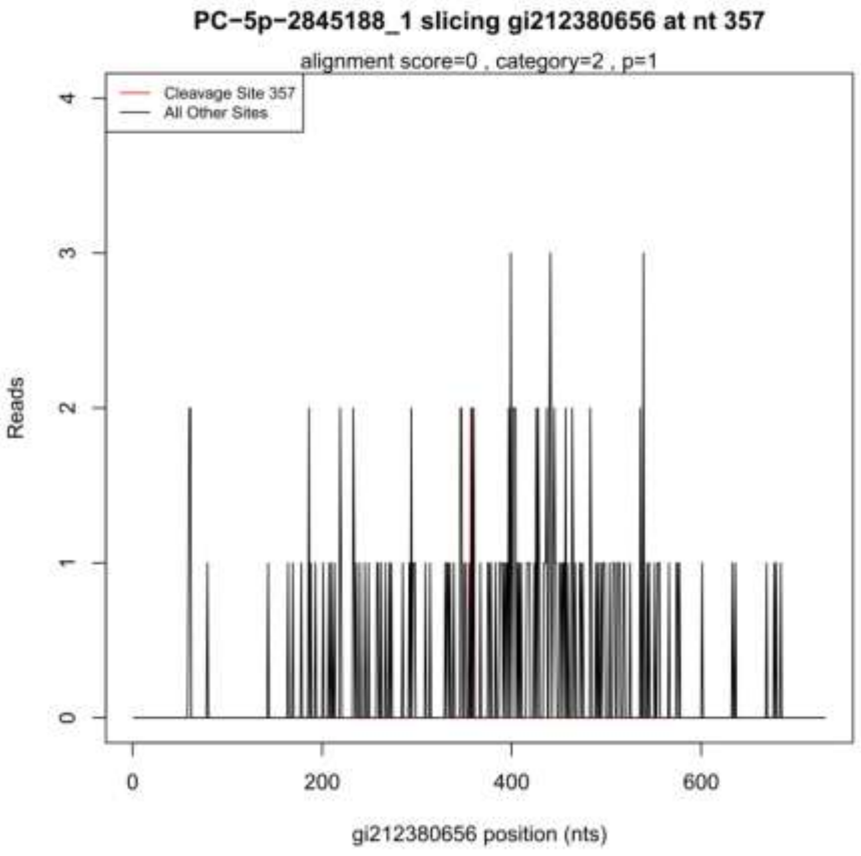
PC-5p-2845188_1 slicing gi212380656 at nt 357
alignment score=0 , category=2 , p=1
Cleavage Site 357
All Other Sites
Reads
gi212380656 position (nts)

PC−5p−2845188_1 slicing gi212380720 at nt 364

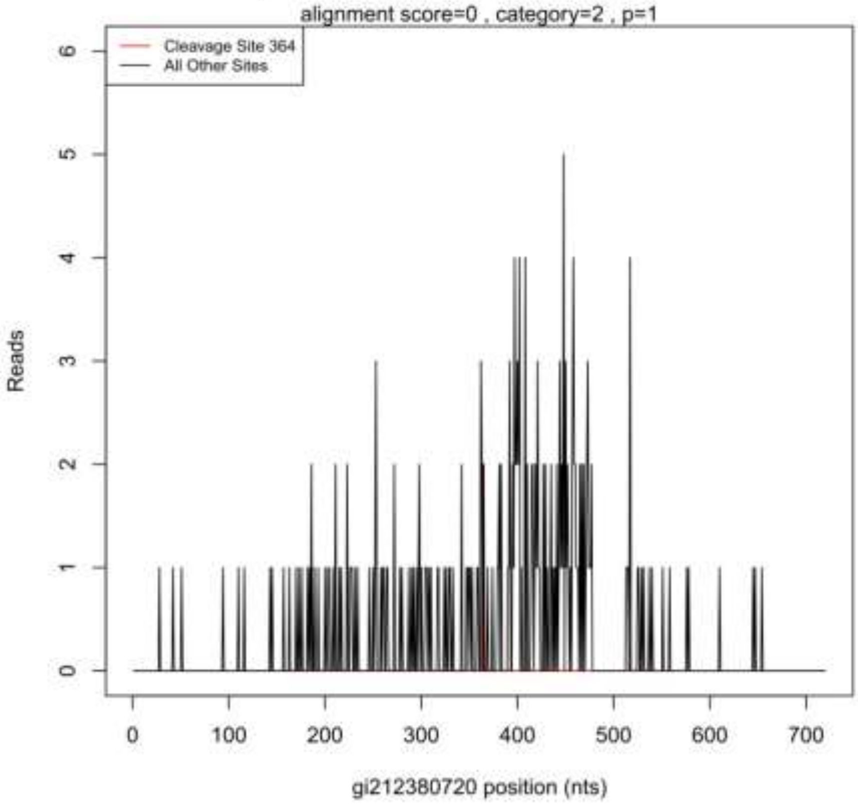

PC−5p−2845188_1 slicing gi366888519 at nt 347

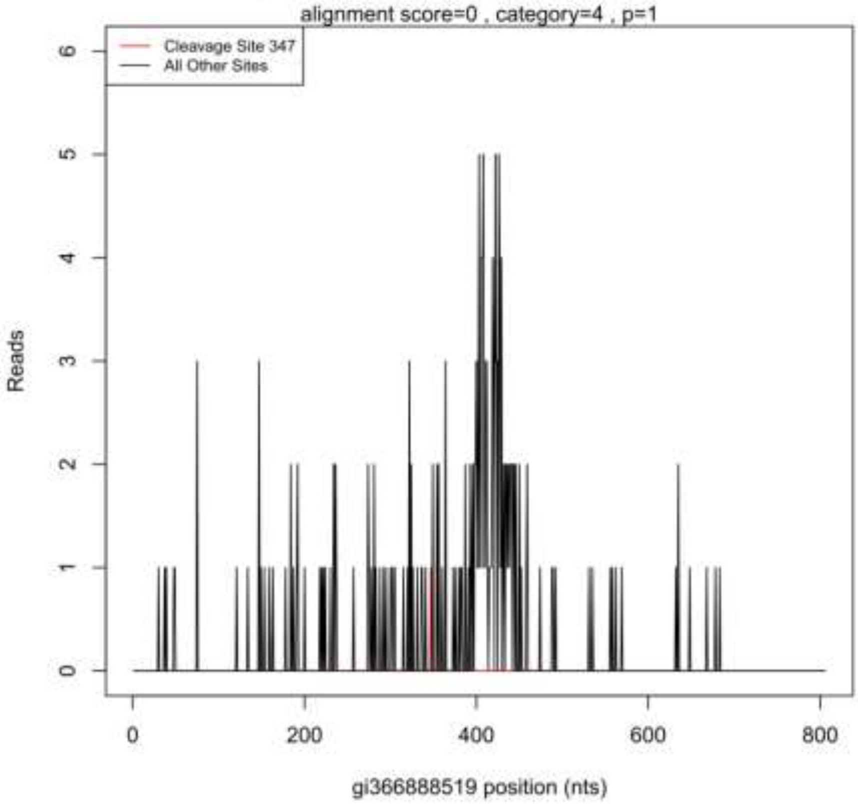

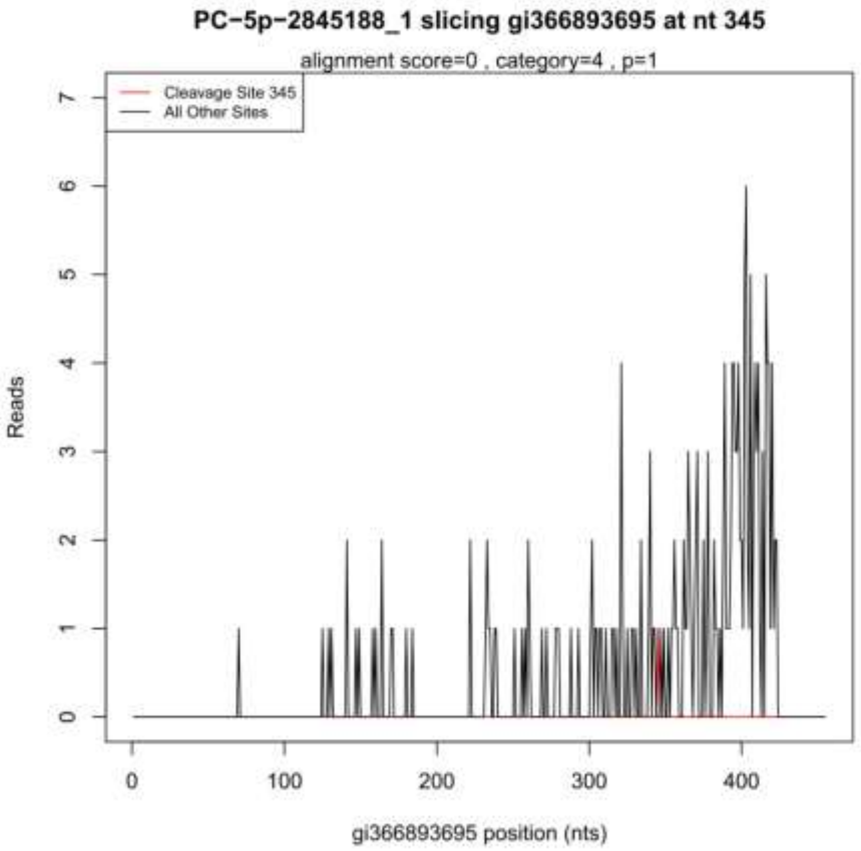
PC−5p−2845188_1 slicing gi366893695 at nt 345
alignment score=0 , category=4 , p=1
Cleavage Site 345
All Other Sites
Reads
gi366893695 position (nts)

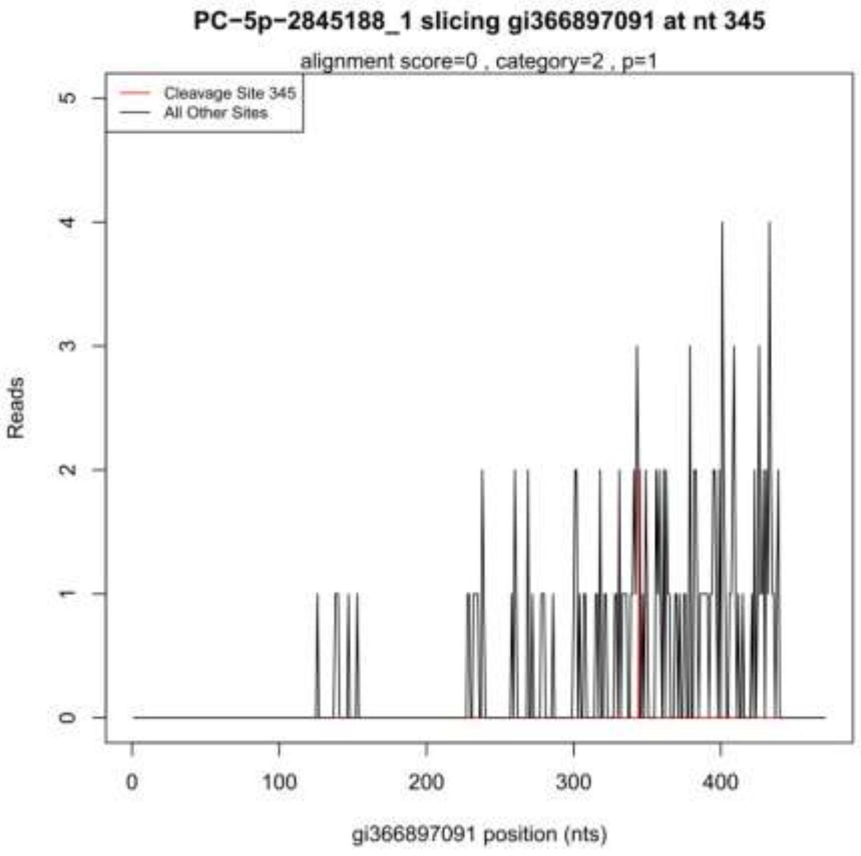
PC−5p−2845188_1 slicing gi366897091 at nt 345
alignment score=0 , category=2 , p=1
Cleavage Site 345
All Other Sites
Reads
gi366897091 position (nts)

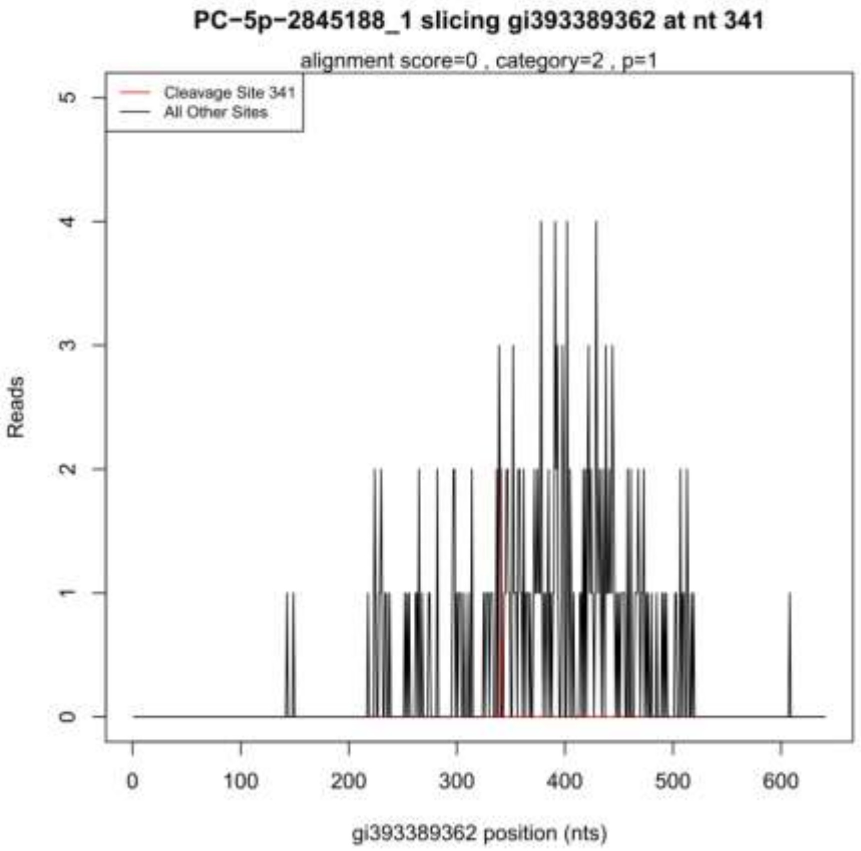
PC−5p−2845188_1 slicing gi393389362 at nt 341
alignment score=0 , category=2 , p=1
Cleavage Site 341
All Other Sites
Reads
0
1
2
3
4
5
0
100
200
300
400
500
600
gi393389362 position (nts)

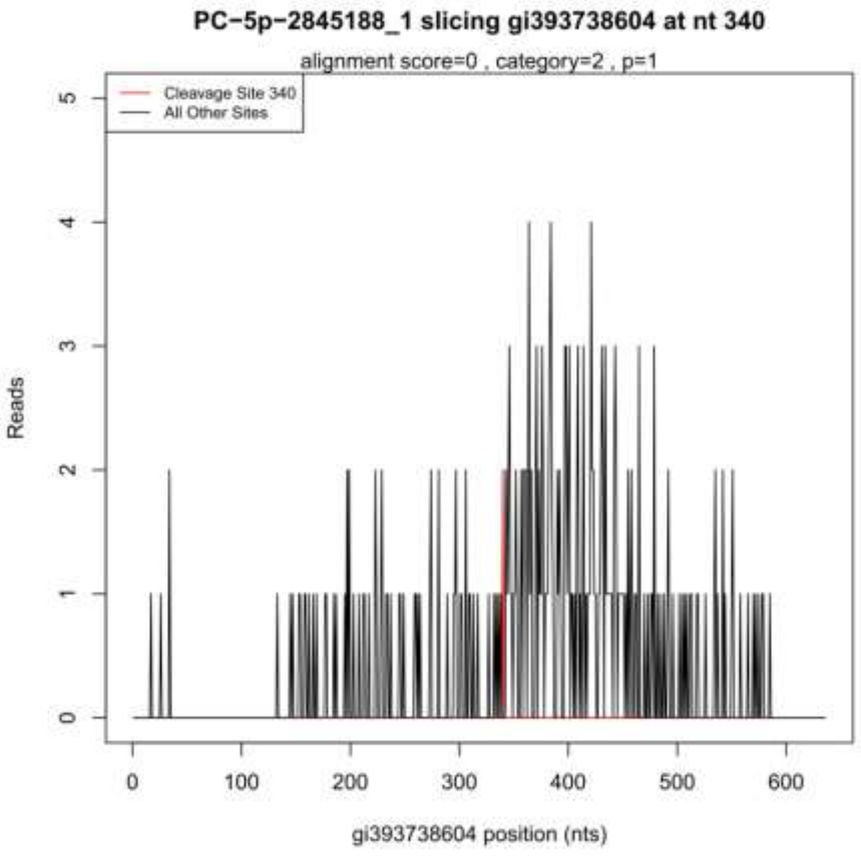
PC−5p−2845188_1 slicing gi393738604 at nt 340
alignment score=0 , category=2 , p=1
Cleavage Site 340
All Other Sites
Reads
0
1
2
3
4
5
0
100
200
300
400
500
600
gi393738604 position (nts)

PC−5p−2845188_1 slicing gi393739039 at nt 303

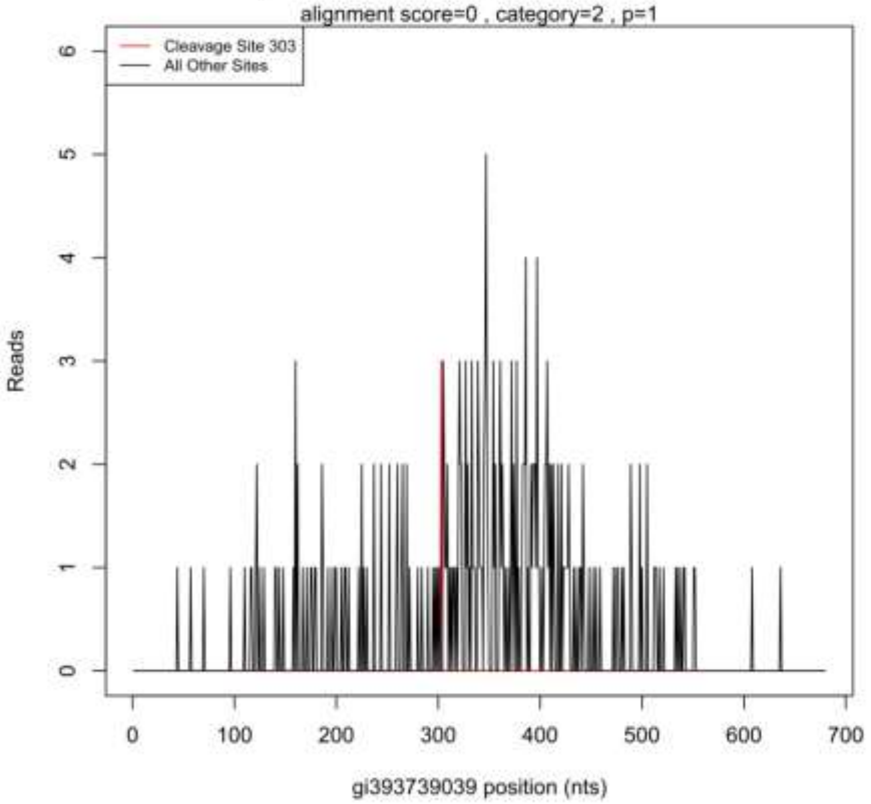

PC−5p−2845188_1 slicing gi393739499 at nt 323

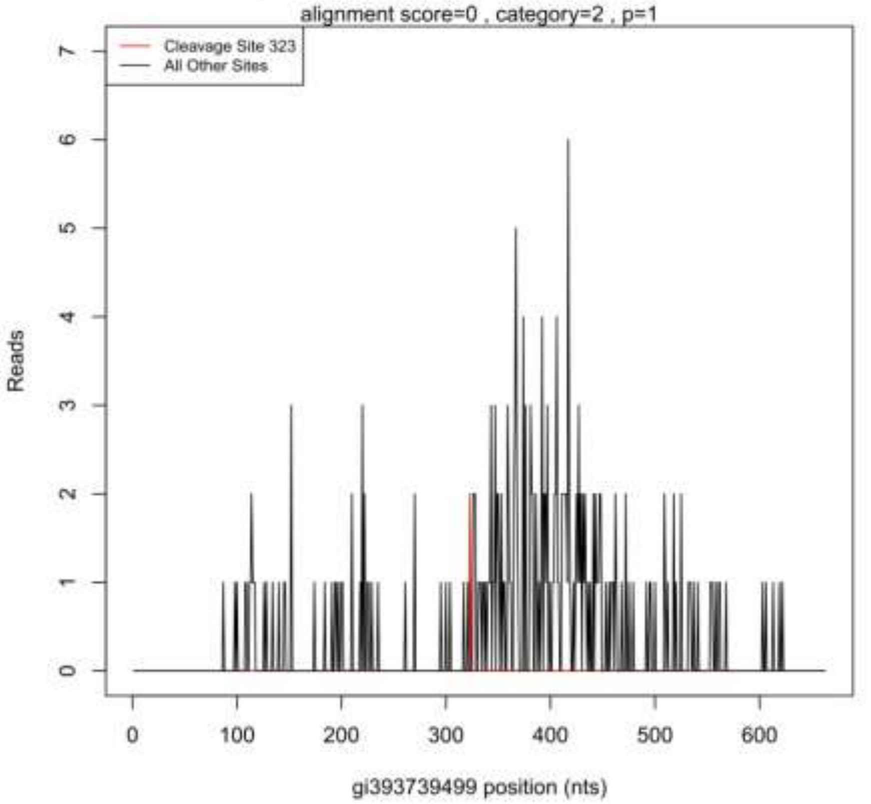

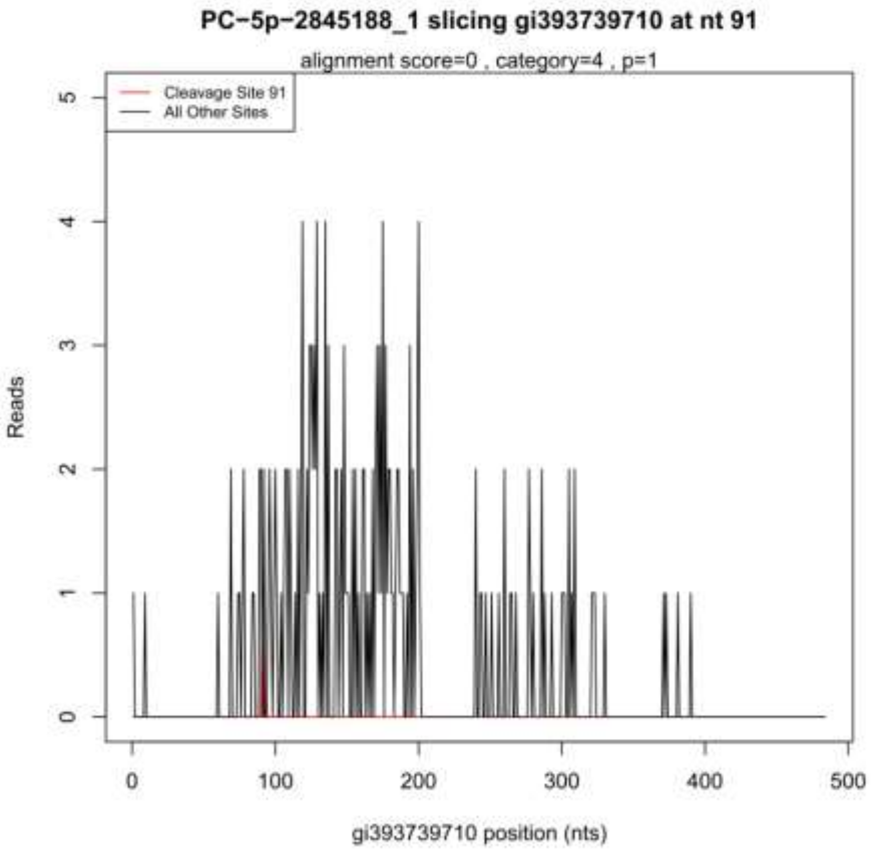
PC−5p−2845188_1 slicing gi393739710 at nt 91
alignment score=0 , category=4 , p=1
Cleavage Site 91
All Other Sites
Reads
0
1
2
3
4
5
0
100
200
300
400
500
gi393739710 position (nts)

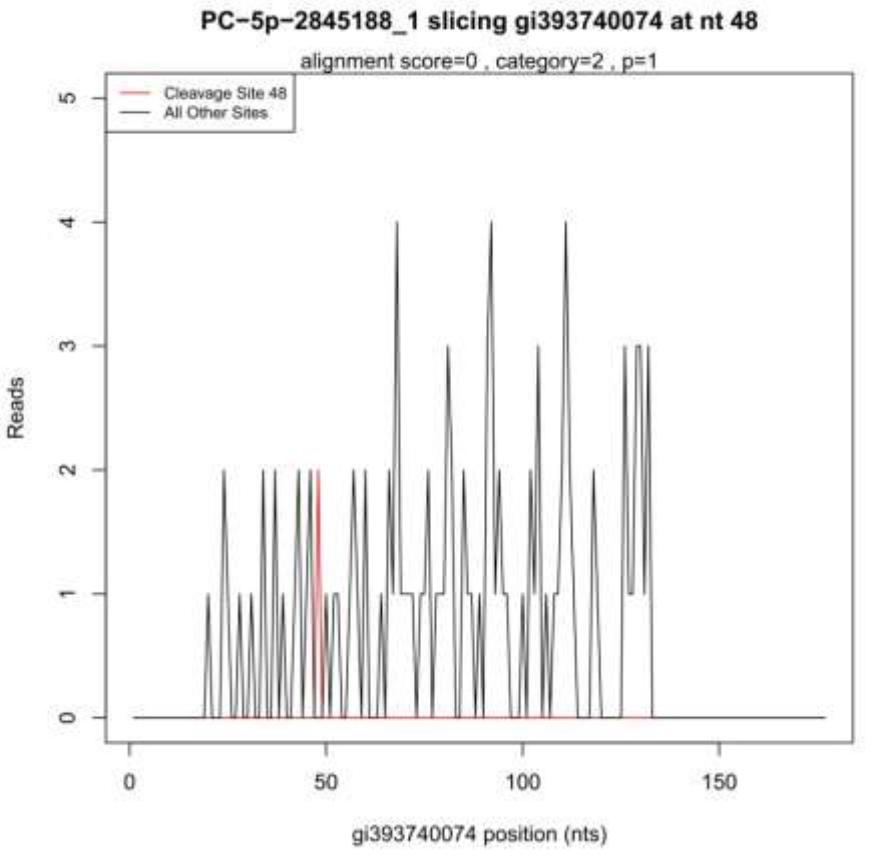
PC−5p−2845188_1 slicing gi393740074 at nt 48
alignment score=0 , category=2 , p=1
Cleavage Site 48
All Other Sites
Reads
0
1
2
3
4
5
0
50
100
150
gi393740074 position (nts)

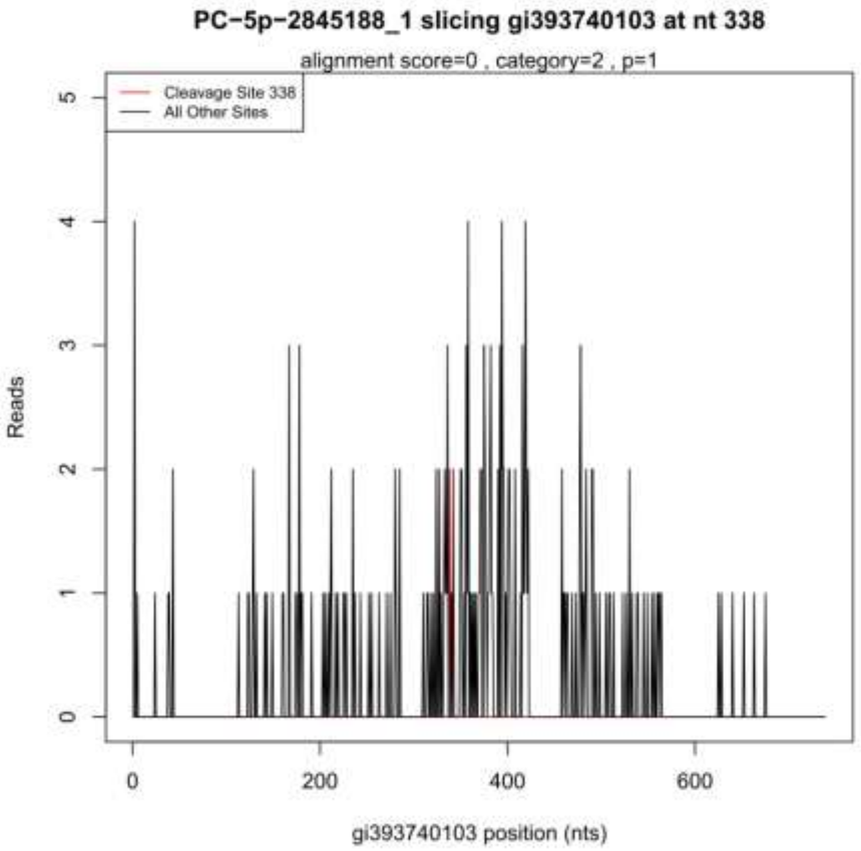
PC−5p−2845188_1 slicing gi393740103 at nt 338
alignment score=0 , category=2 , p=1
Cleavage Site 338
All Other Sites
Reads
0
1
2
3
4
5
0
200
400
600
gi393740103 position (nts)

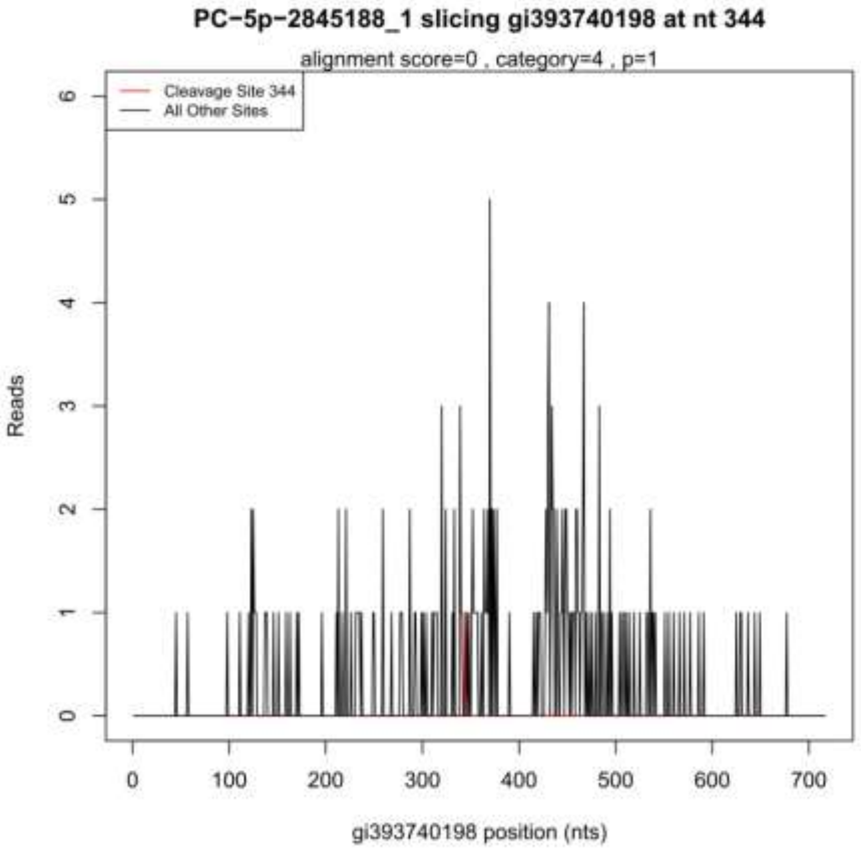
PC−5p−2845188_1 slicing gi393740198 at nt 344
alignment score=0 , category=4 , p=1
Cleavage Site 344
All Other Sites
Reads
0
1
2
3
4
5
6
0
100
200
300
400
500
600
700
gi393740198 position (nts)

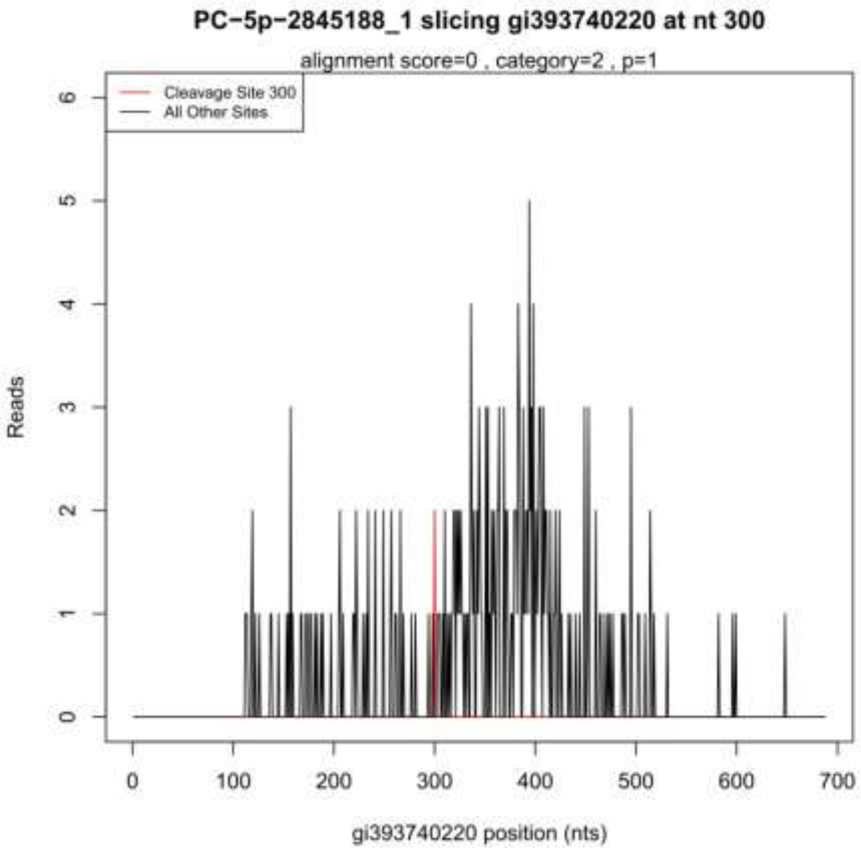
PC−5p−2845188_1 slicing gi393740220 at nt 300
alignment score=0 , category=2 , p=1
Cleavage Site 300
All Other Sites
Reads
gi393740220 position (nts)

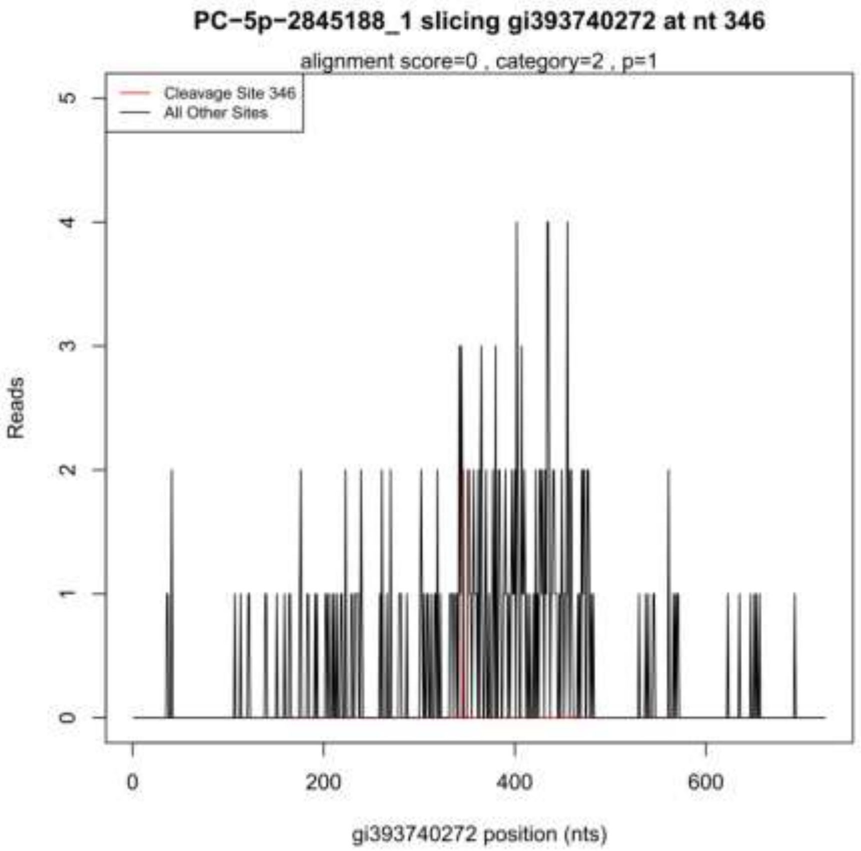
PC−5p−2845188_1 slicing gi393740272 at nt 346
alignment score=0 , category=2 , p=1
Cleavage Site 346
All Other Sites
Reads
gi393740272 position (nts)

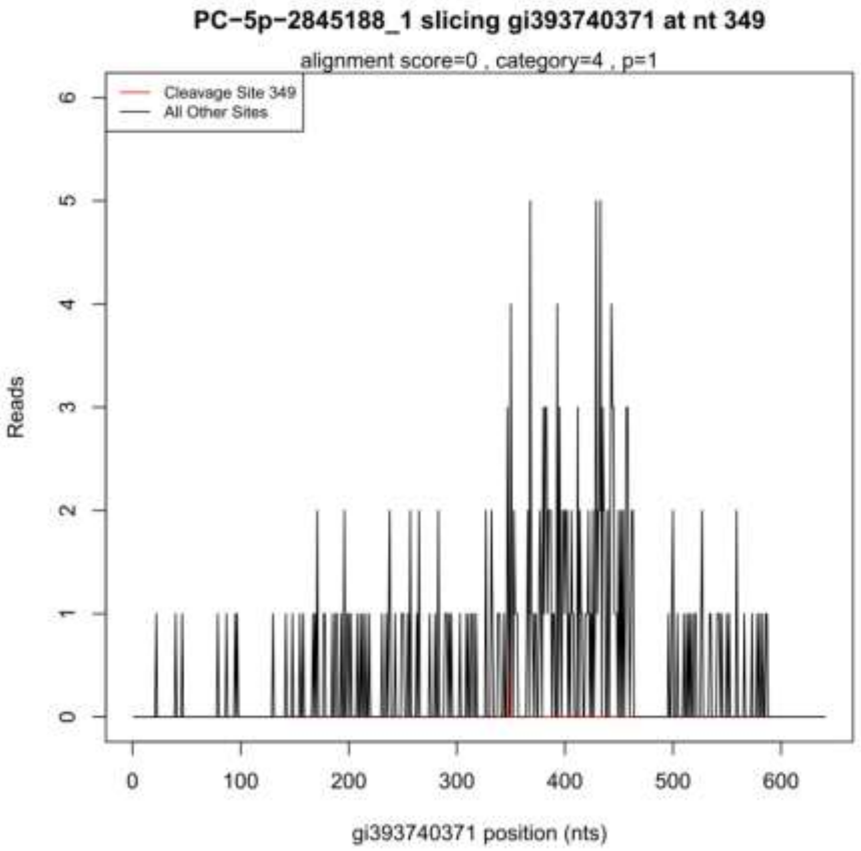
PC−5p−2845188_1 slicing gi393740371 at nt 349
alignment score=0 , category=4 , p=1
Cleavage Site 349
All Other Sites
Reads
gi393740371 position (nts)

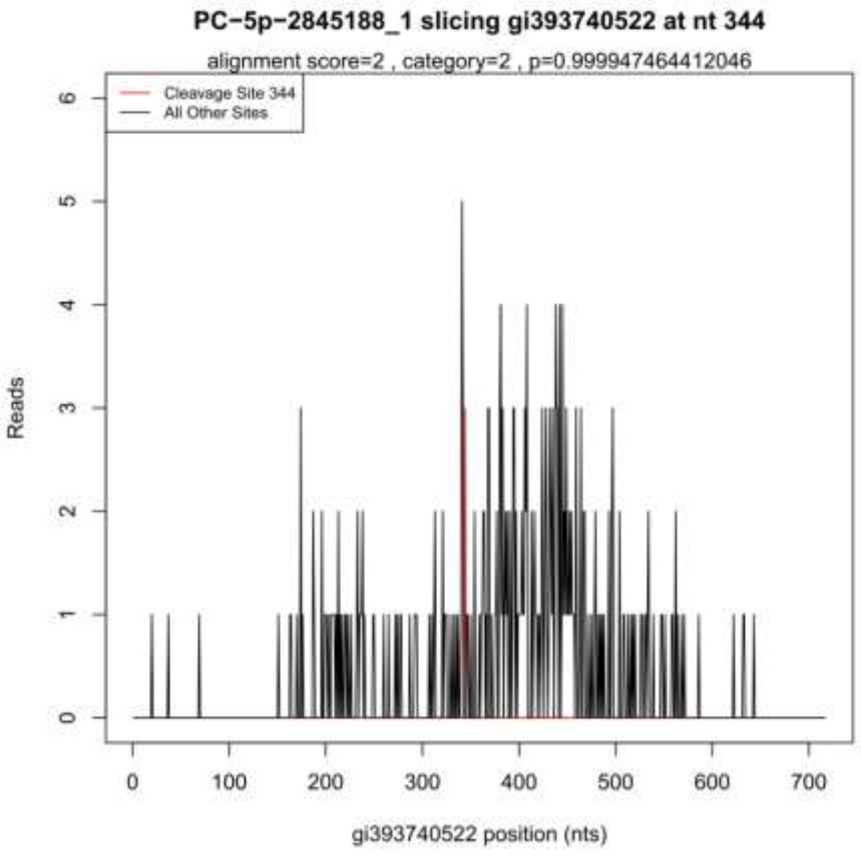
PC−5p−2845188_1 slicing gi393740522 at nt 344
alignment score=2 , category=2 , p=0.999947464412046
Cleavage Site 344
All Other Sites
Reads
gi393740522 position (nts)

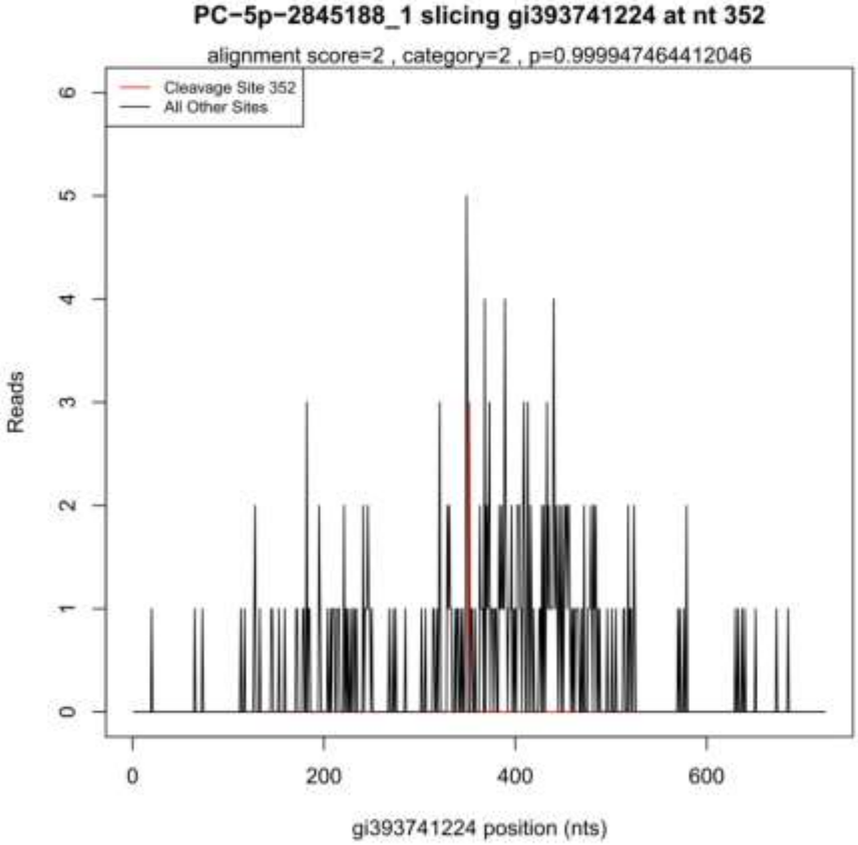
PC−5p−2845188_1 slicing gi393741224 at nt 352
alignment score=2 , category=2 , p=0.999947464412046
Cleavage Site 352
All Other Sites
Reads
0
1
2
3
4
5
6
0
200
400
600
gi393741224 position (nts)

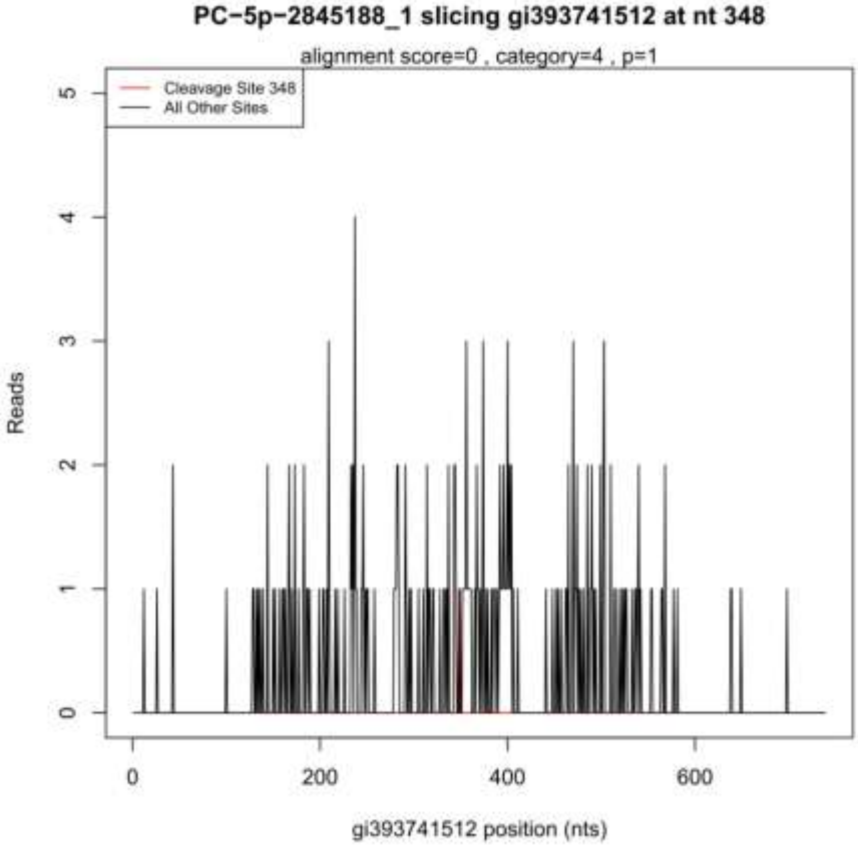
PC−5p−2845188_1 slicing gi393741512 at nt 348
alignment score=0 , category=4 , p=1
Cleavage Site 348
All Other Sites
Reads
0
1
2
3
4
5
0
200
400
600
gi393741512 position (nts)

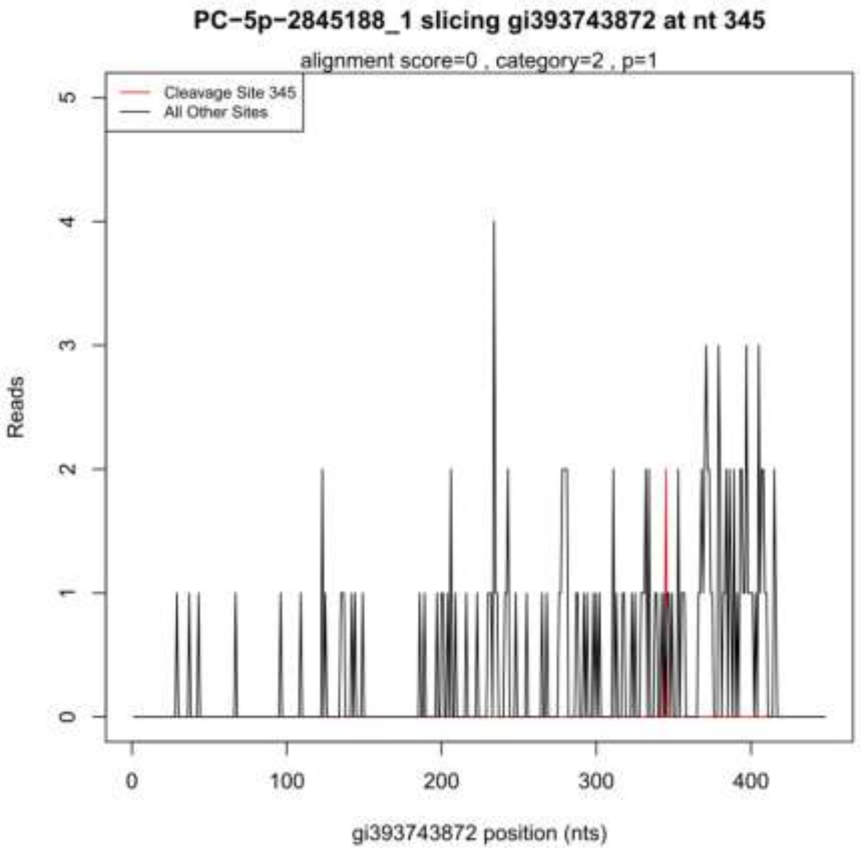
PC−5p−2845188_1 slicing gi393743872 at nt 345
alignment score=0 , category=2 , p=1
Cleavage Site 345
All Other Sites
Reads
gi393743872 position (nts)

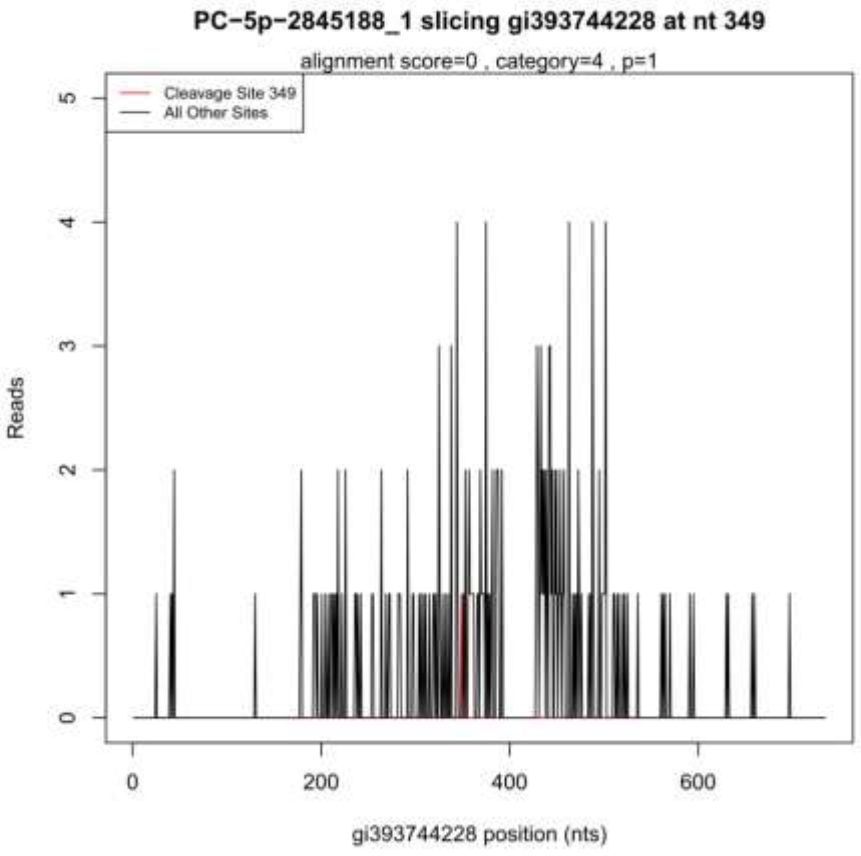
PC−5p−2845188_1 slicing gi393744228 at nt 349
alignment score=0 , category=4 , p=1
Cleavage Site 349
All Other Sites
Reads
gi393744228 position (nts)

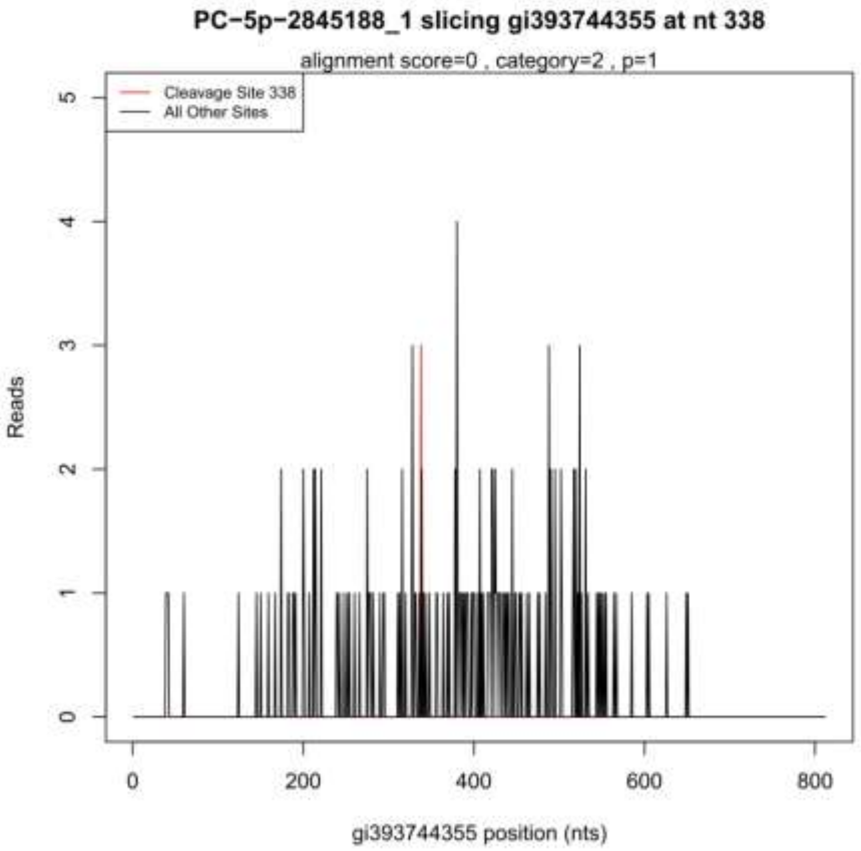
PC−5p−2845188_1 slicing gi393744355 at nt 338
alignment score=0 , category=2 , p=1
Cleavage Site 338
All Other Sites
Reads
gi393744355 position (nts)

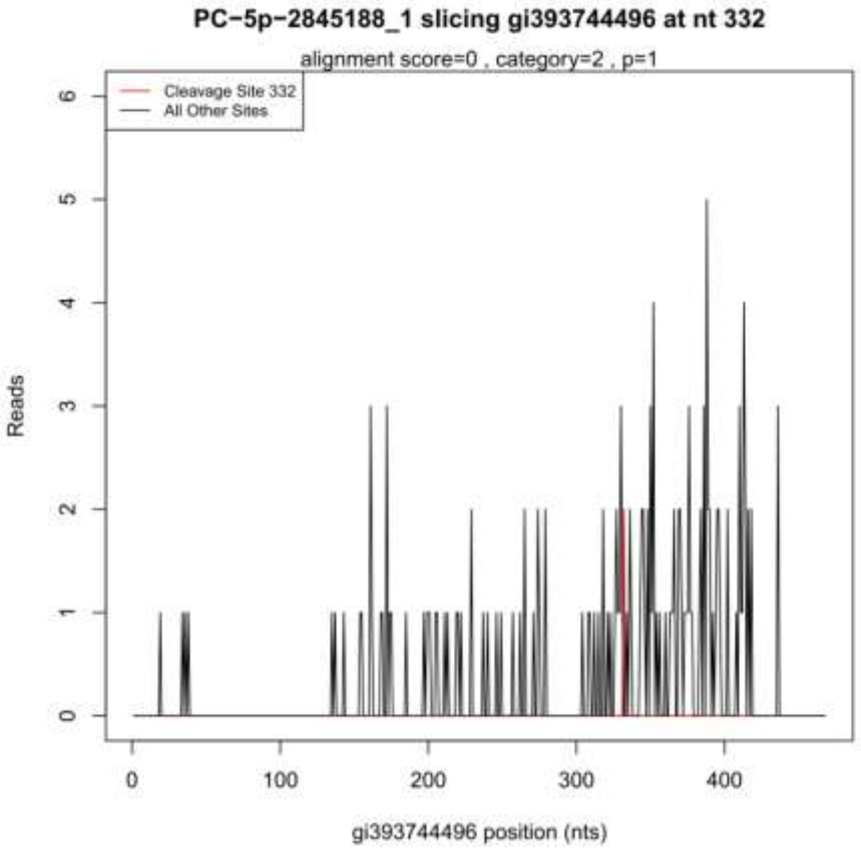
PC−5p−2845188_1 slicing gi393744496 at nt 332
alignment score=0 , category=2 , p=1
Cleavage Site 332
All Other Sites
Reads
gi393744496 position (nts)

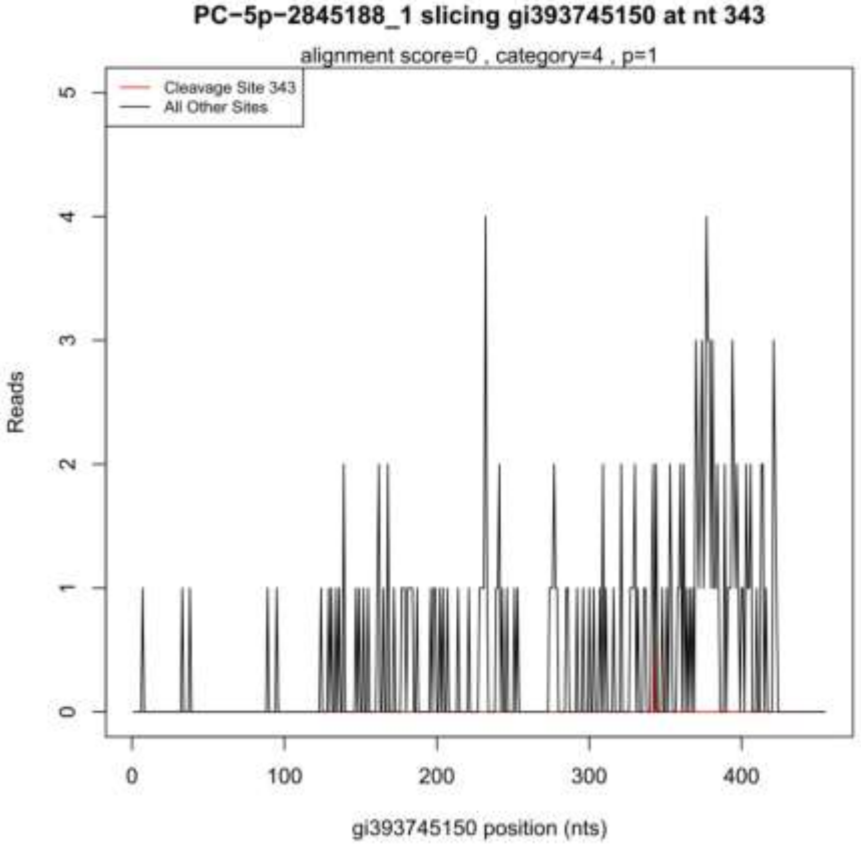
PC−5p−2845188_1 slicing gi393745150 at nt 343
alignment score=0 , category=4 , p=1
Cleavage Site 343
All Other Sites
Reads
gi393745150 position (nts)

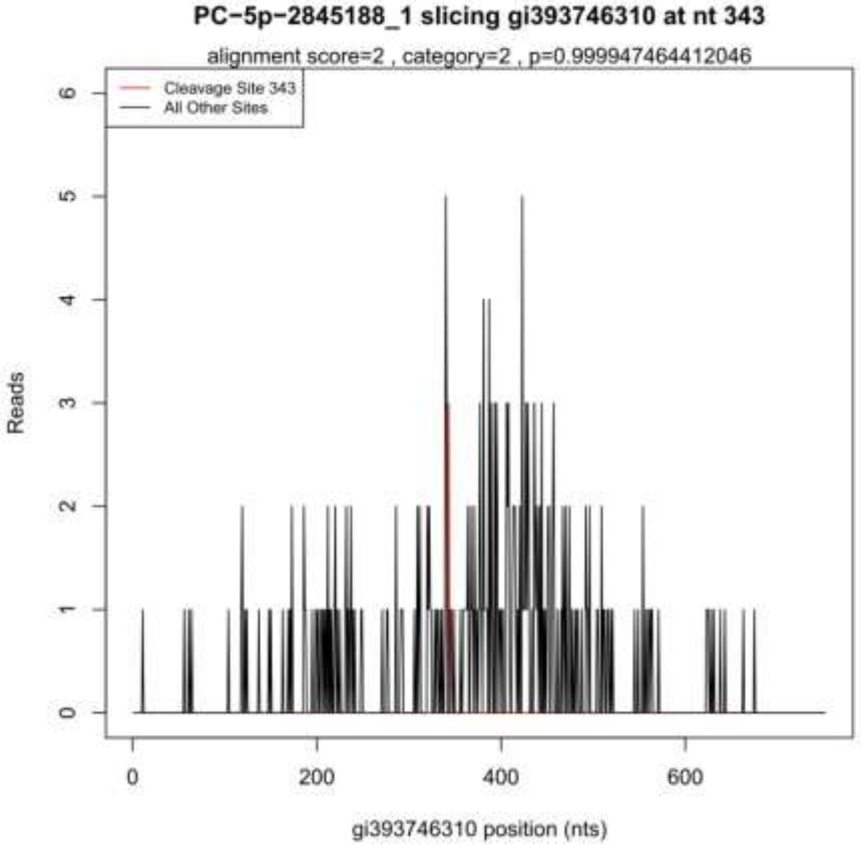
PC−5p−2845188_1 slicing gi393746310 at nt 343
alignment score=2 , category=2 , p=0.999947464412046
Cleavage Site 343
All Other Sites
Reads
gi393746310 position (nts)

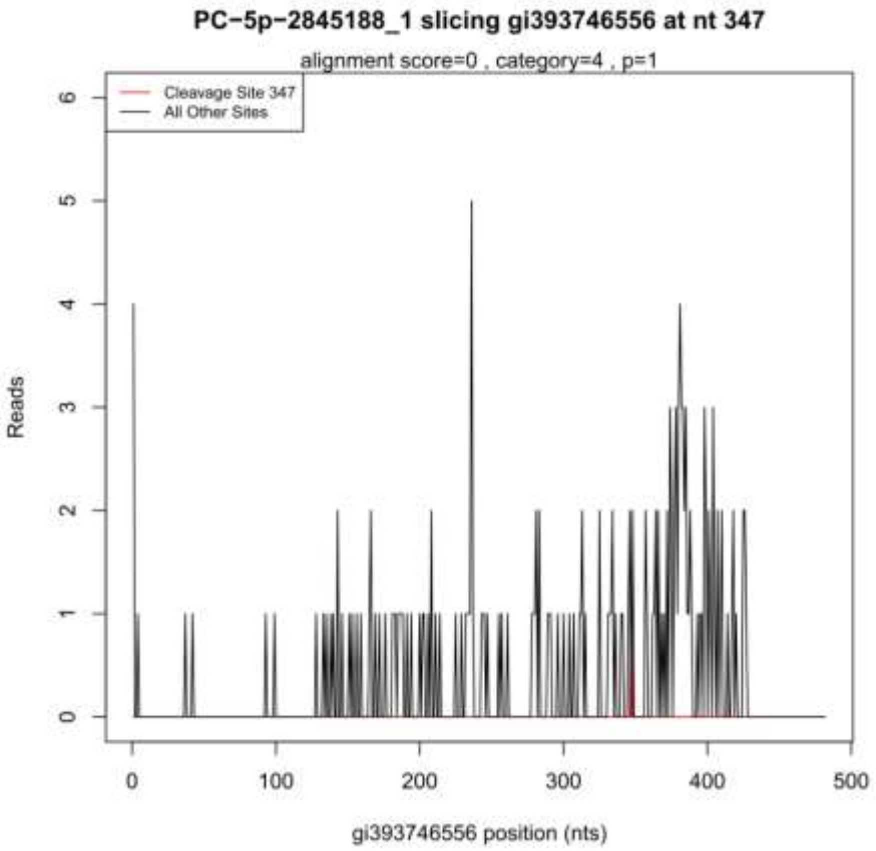
PC−5p−2845188_1 slicing gi393746556 at nt 347
alignment score=0 , category=4 , p=1
Cleavage Site 347
All Other Sites
Reads
gi393746556 position (nts)

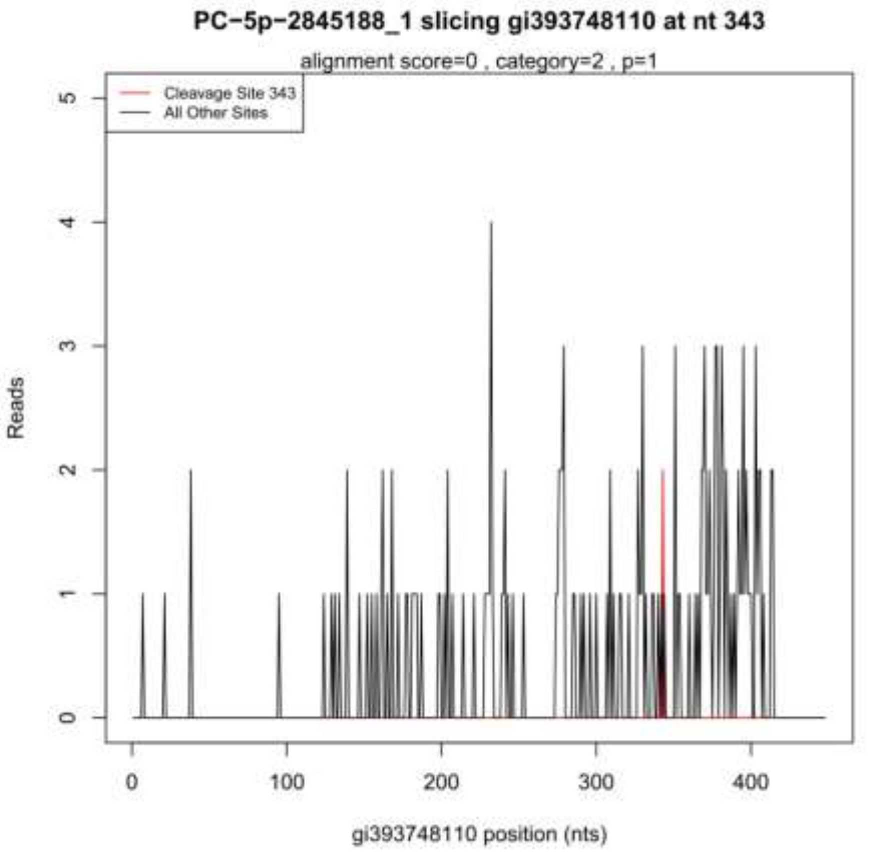
PC−5p−2845188_1 slicing gi393748110 at nt 343
alignment score=0 , category=2 , p=1
Cleavage Site 343
All Other Sites
Reads
gi393748110 position (nts)

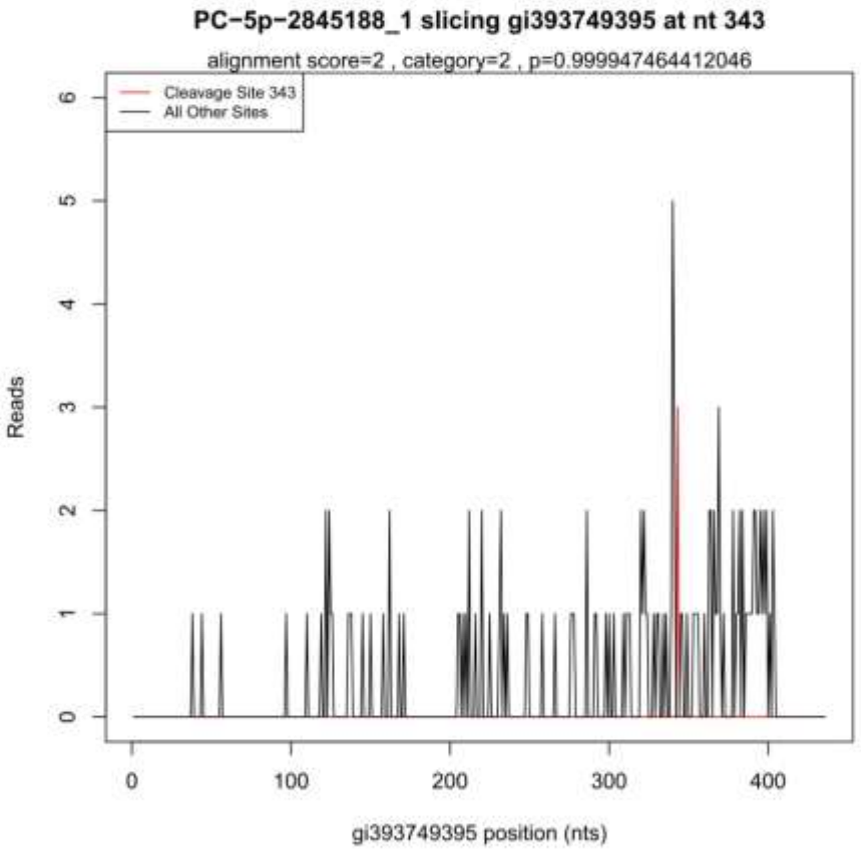
PC−5p−2845188_1 slicing gi393749395 at nt 343
alignment score=2 , category=2 , p=0.999947464412046
Cleavage Site 343
All Other Sites
Reads
gi393749395 position (nts)

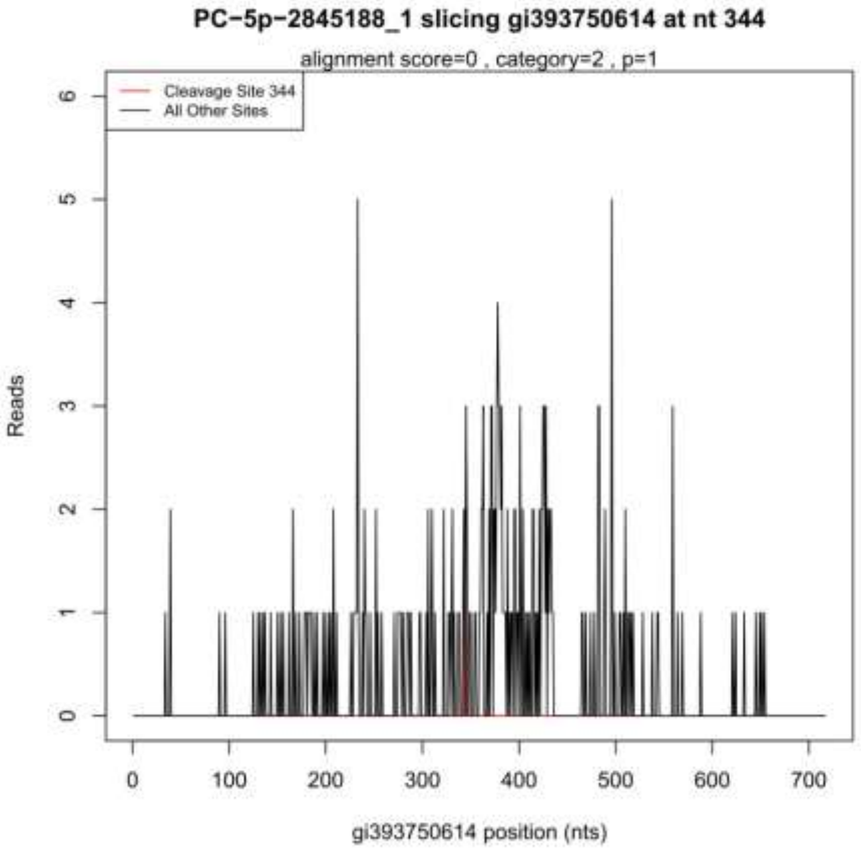
PC−5p−2845188_1 slicing gi393750614 at nt 344
alignment score=0 , category=2 , p=1
Cleavage Site 344
All Other Sites
Reads
gi393750614 position (nts)

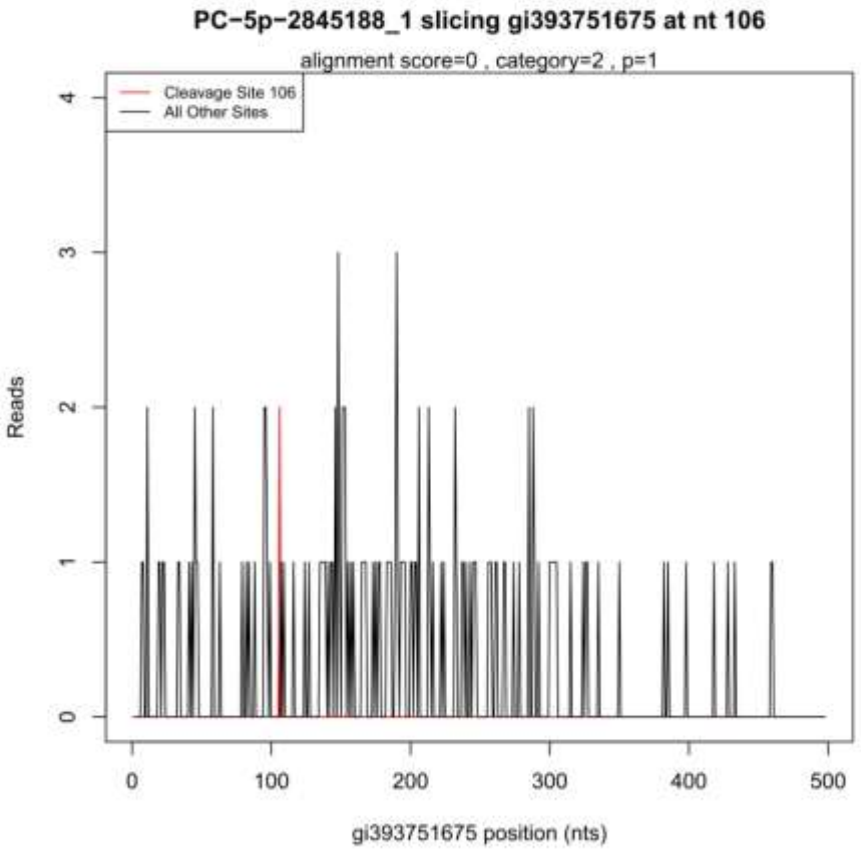
PC−5p−2845188_1 slicing gi393751675 at nt 106
alignment score=0 , category=2 , p=1
Cleavage Site 106
All Other Sites
Reads
0
1
2
3
4
0
100
200
300
400
500
gi393751675 position (nts)

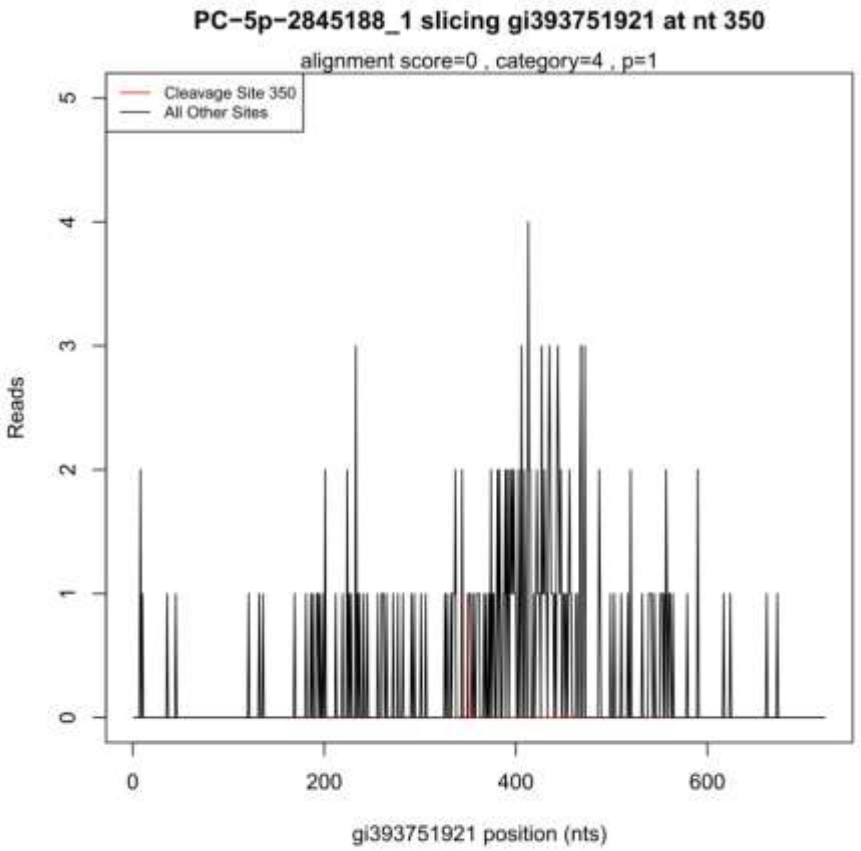
PC−5p−2845188_1 slicing gi393751921 at nt 350
alignment score=0 , category=4 , p=1
Cleavage Site 350
All Other Sites
Reads
0
1
2
3
4
5
0
200
400
600
gi393751921 position (nts)

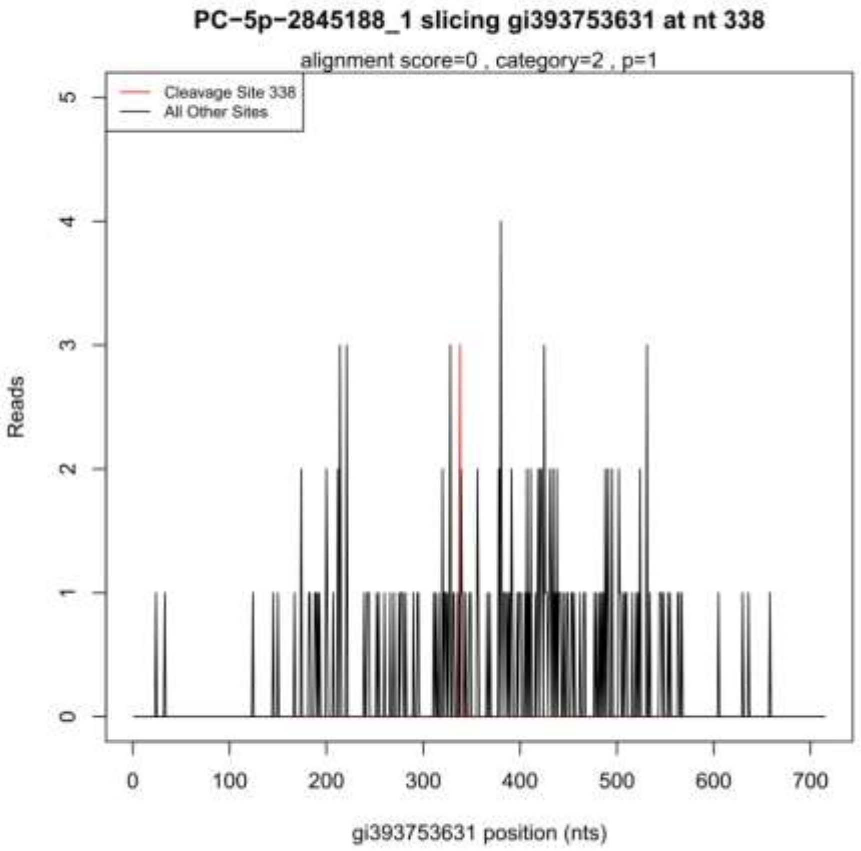
PC−5p−2845188_1 slicing gi393753631 at nt 338
alignment score=0 , category=2 , p=1
Cleavage Site 338
All Other Sites
Reads
gi393753631 position (nts)

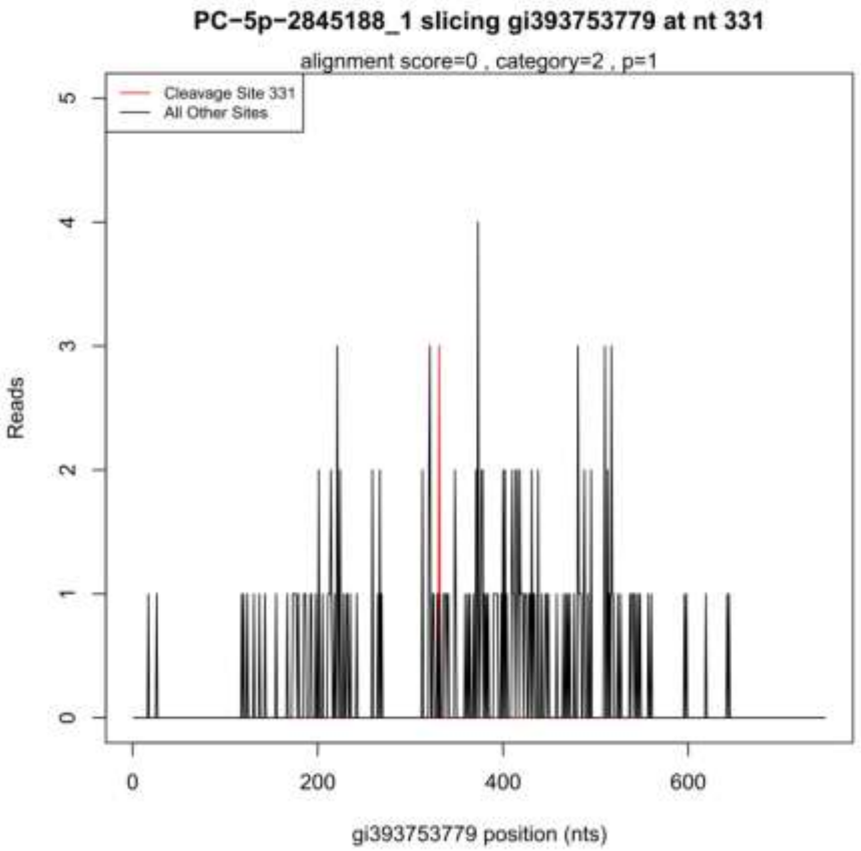
PC−5p−2845188_1 slicing gi393753779 at nt 331
alignment score=0 , category=2 , p=1
Cleavage Site 331
All Other Sites
Reads
gi393753779 position (nts)

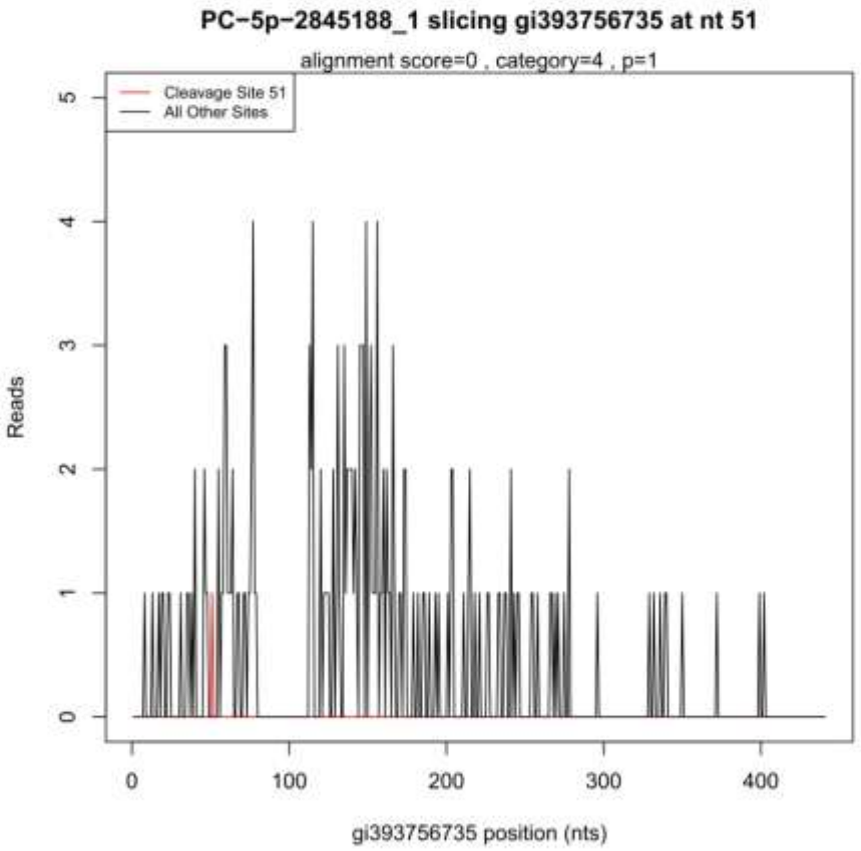
PC−5p−2845188_1 slicing gi393756735 at nt 51
alignment score=0 , category=4 , p=1
Cleavage Site 51
All Other Sites
Reads
gi393756735 position (nts)

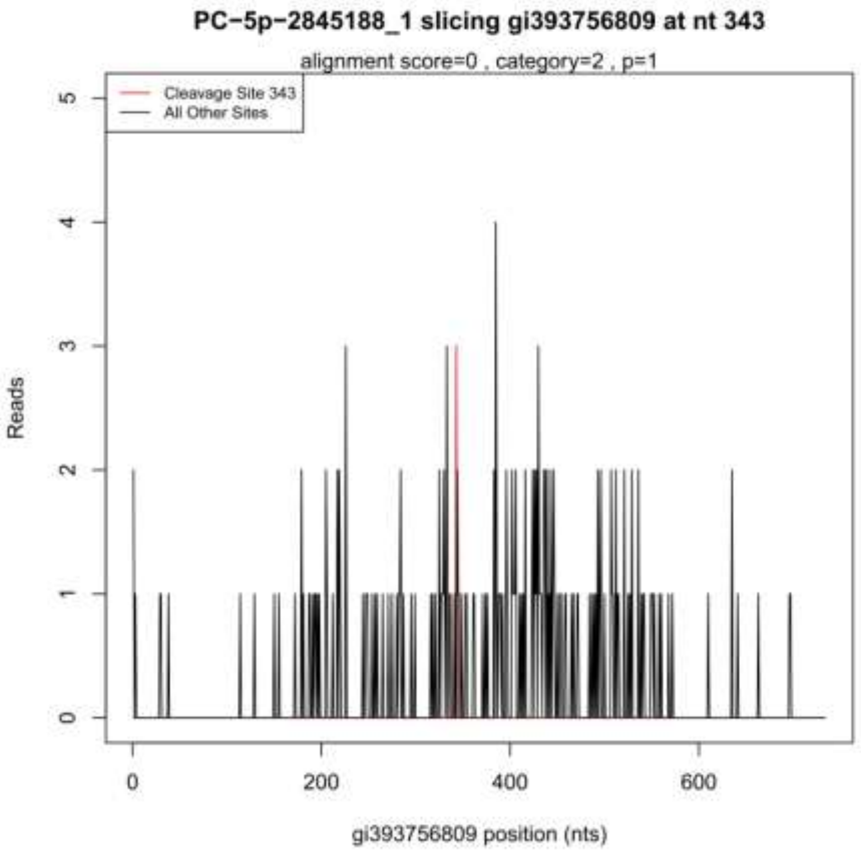
PC−5p−2845188_1 slicing gi393756809 at nt 343
alignment score=0 , category=2 , p=1
Cleavage Site 343
All Other Sites
Reads
gi393756809 position (nts)

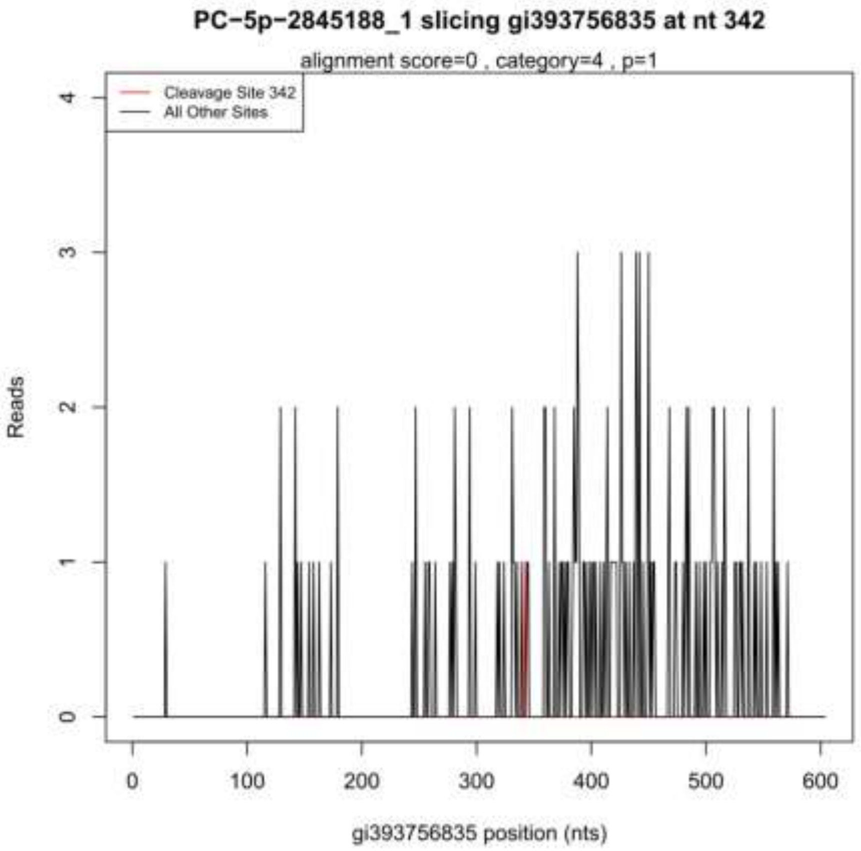
PC−5p−2845188_1 slicing gi393756835 at nt 342
alignment score=0 , category=4 , p=1
Cleavage Site 342
All Other Sites
Reads
0
1
2
3
4
0
100
200
300
400
500
600
gi393756835 position (nts)

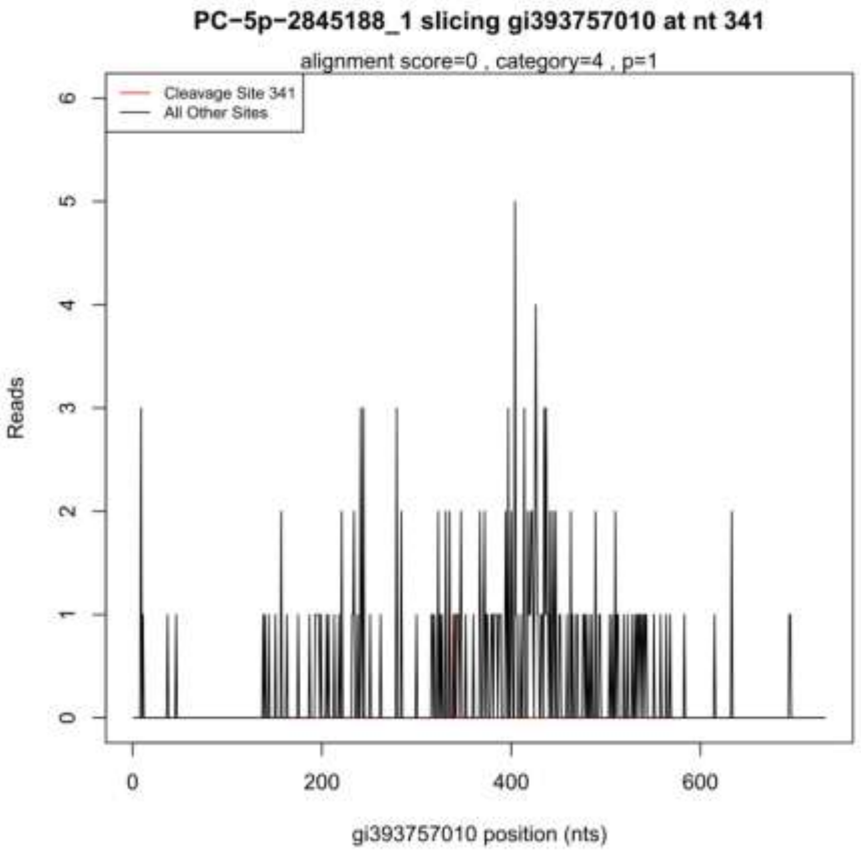
PC−5p−2845188_1 slicing gi393757010 at nt 341
alignment score=0 , category=4 , p=1
Cleavage Site 341
All Other Sites
Reads
0
1
2
3
4
5
6
0
200
400
600
gi393757010 position (nts)

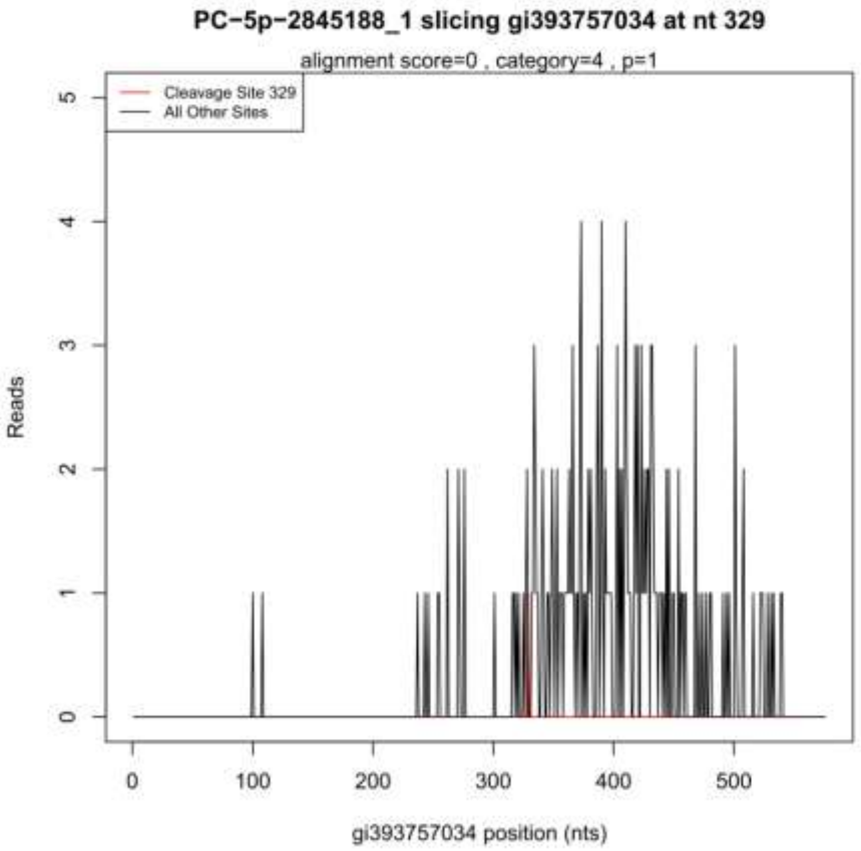
PC-5p-2845188_1 slicing gi393757034 at nt 329
alignment score=0 , category=4 , p=1
Cleavage Site 329
All Other Sites
Reads
0
1
2
3
4
5
0
100
200
300
400
500
gi393757034 position (nts)

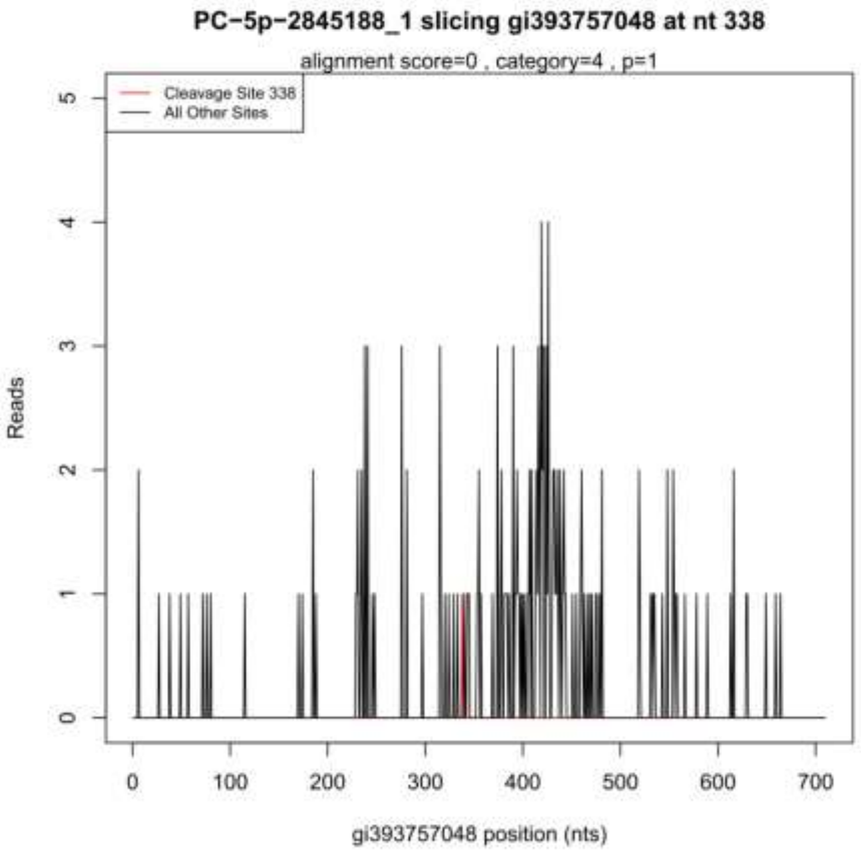
PC-5p-2845188_1 slicing gi393757048 at nt 338
alignment score=0 , category=4 , p=1
Cleavage Site 338
All Other Sites
Reads
0
1
2
3
4
5
0
100
200
300
400
500
600
700
gi393757048 position (nts)

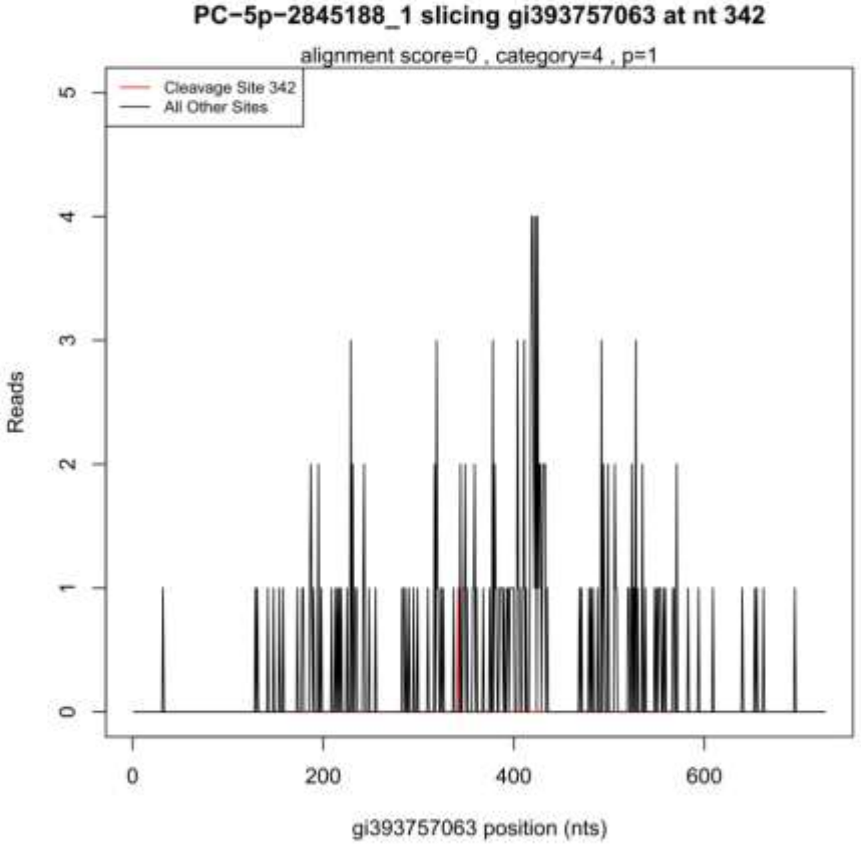
PC-5p-2845188_1 slicing gi393757063 at nt 342
alignment score=0 , category=4 , p=1
Cleavage Site 342
All Other Sites
Reads
0
1
2
3
4
5
0
200
400
600
gi393757063 position (nts)

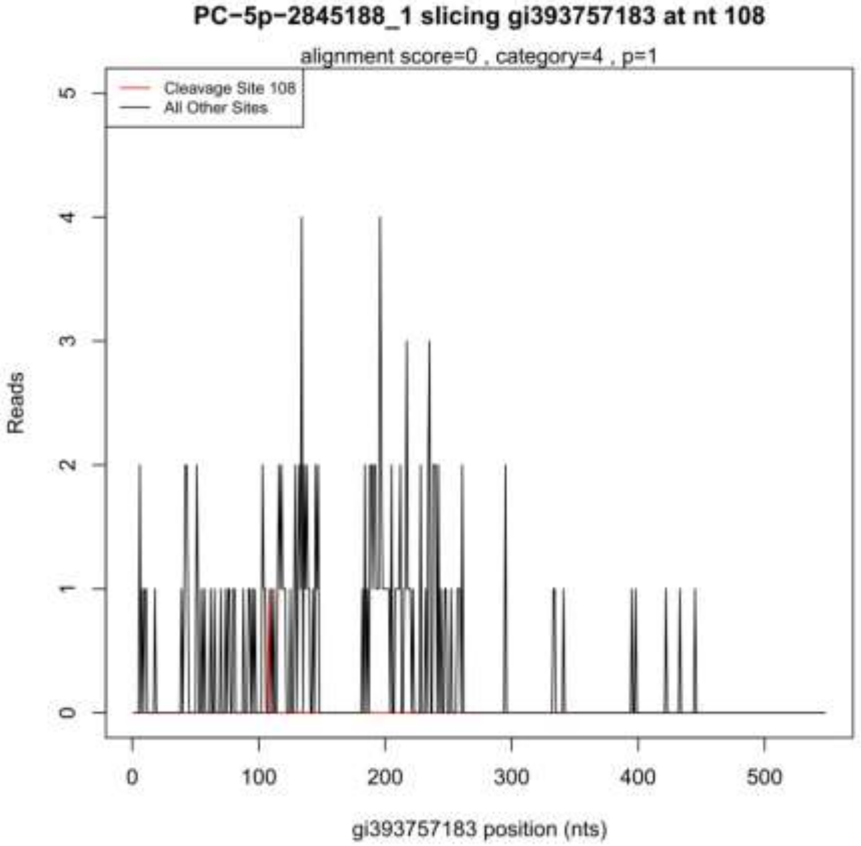
PC-5p-2845188_1 slicing gi393757183 at nt 108
alignment score=0 , category=4 , p=1
Cleavage Site 108
All Other Sites
Reads
0
1
2
3
4
5
0
100
200
300
400
500
gi393757183 position (nts)

PC−5p−3011344_1 slicing gi366894572 at nt 143

alignment score=3 , category=4 , p=0.490323532405333

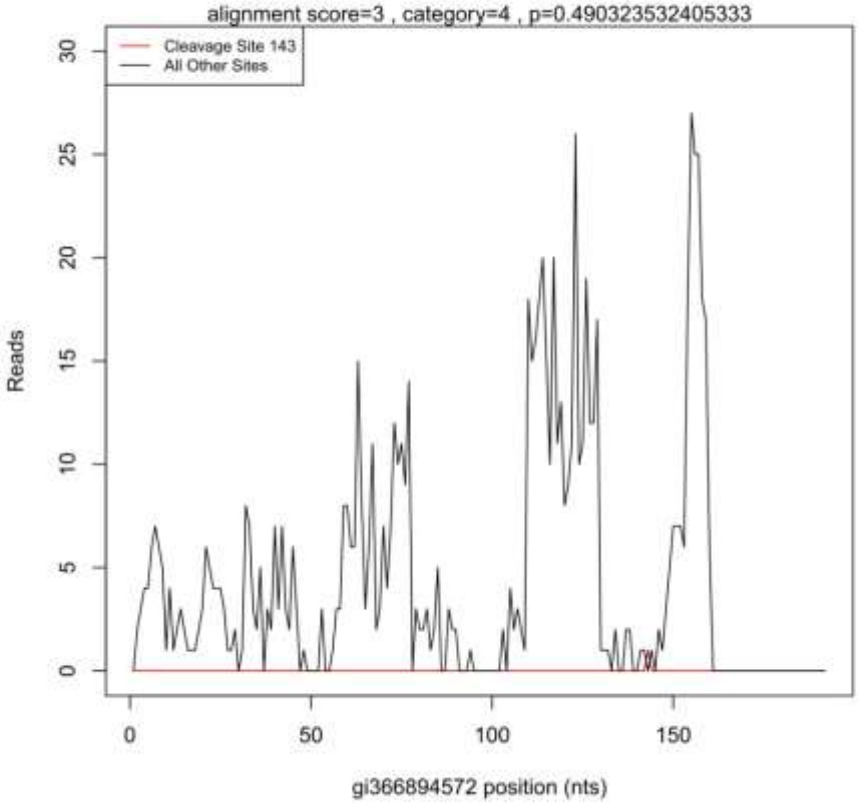

PC−5p−314691_3 slicing gi393744756 at nt 55

alignment score=4 , category=4 , p=0.901489219000185

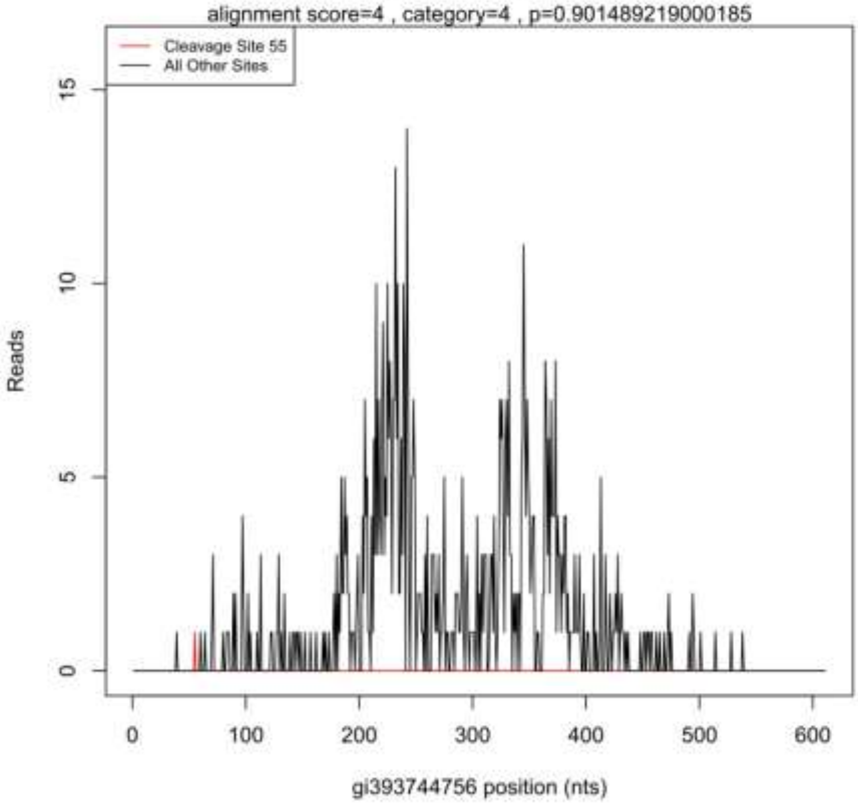

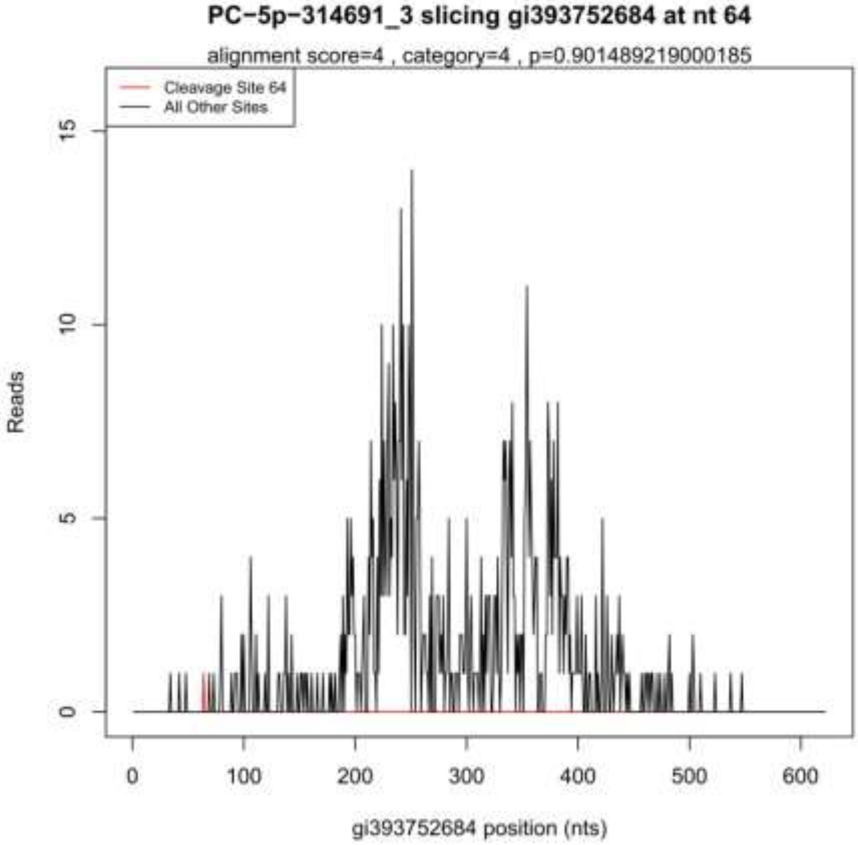

PC−5p−314691_3 slicing gi393752684 at nt 64
alignment score=4 , category=4 , p=0.901489219000185
Cleavage Site 64
All Other Sites
Reads
0
5
10
15
0
100
200
300
400
500
600
gi393752684 position (nts)

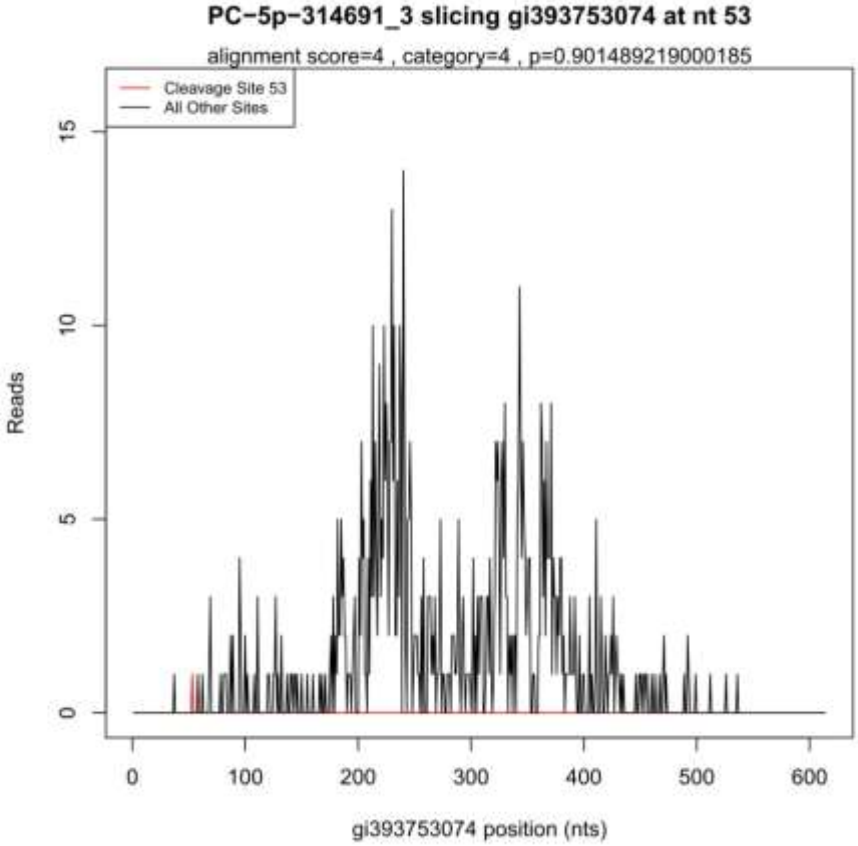

PC−5p−314691_3 slicing gi393753074 at nt 53
alignment score=4 , category=4 , p=0.901489219000185
Cleavage Site 53
All Other Sites
Reads
0
5
10
15
0
100
200
300
400
500
600
gi393753074 position (nts)

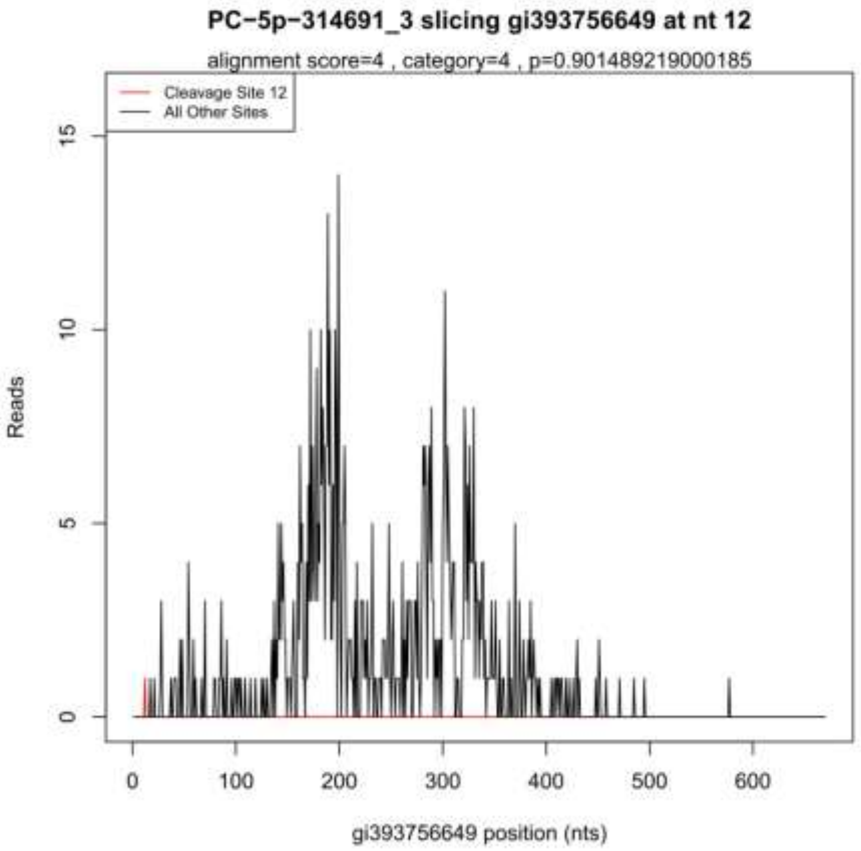
PC−5p−314691_3 slicing gi393756649 at nt 12
alignment score=4 , category=4 , p=0.901489219000185
Cleavage Site 12
All Other Sites
Reads
0
5
10
15
0
100
200
300
400
500
600
gi393756649 position (nts)

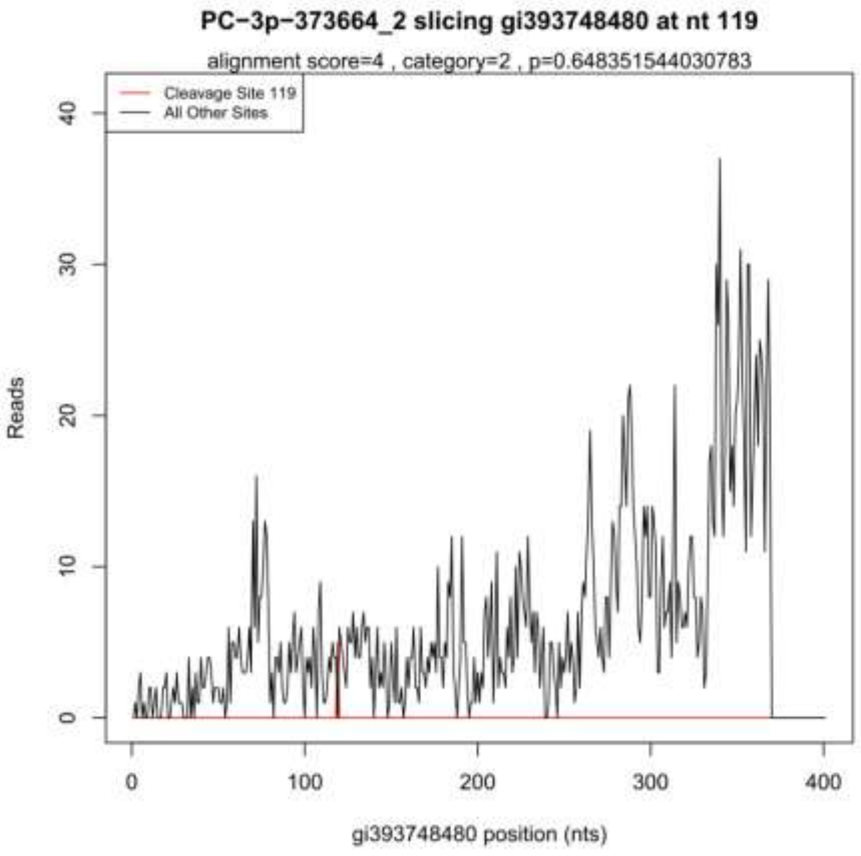
PC−3p−373664_2 slicing gi393748480 at nt 119
alignment score=4 , category=2 , p=0.648351544030783
Cleavage Site 119
All Other Sites
Reads
0
10
20
30
40
0
100
200
300
400
gi393748480 position (nts)

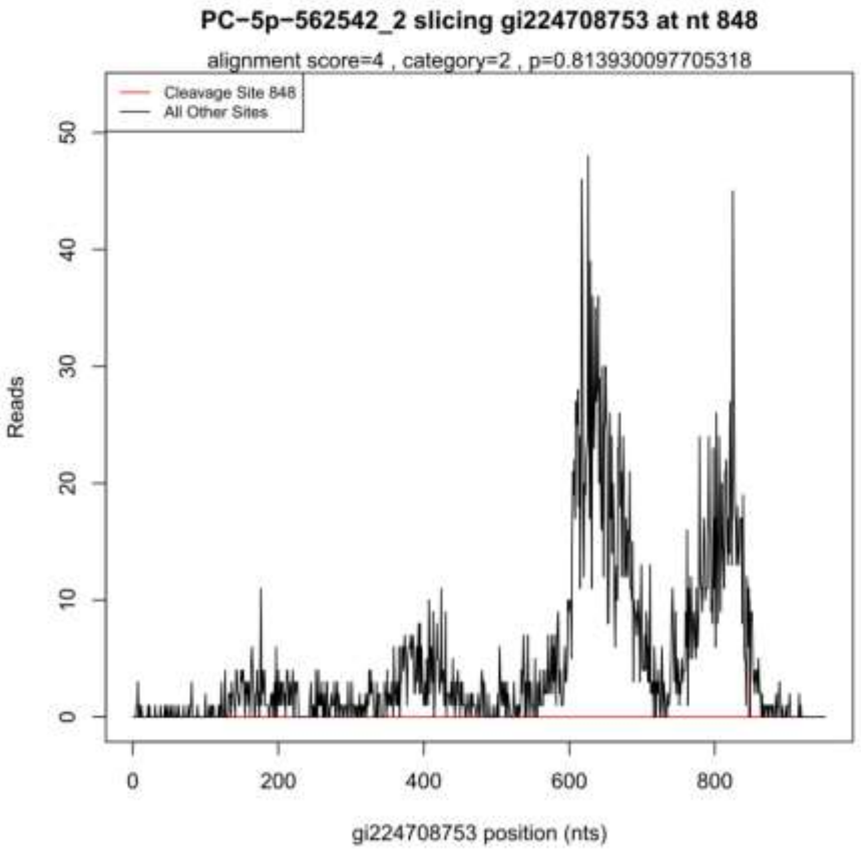
PC−5p−562542_2 slicing gi224708753 at nt 848
alignment score=4 , category=2 , p=0.813930097705318
Cleavage Site 848
All Other Sites
Reads
0
10
20
30
40
50
0
200
400
600
800
gi224708753 position (nts)

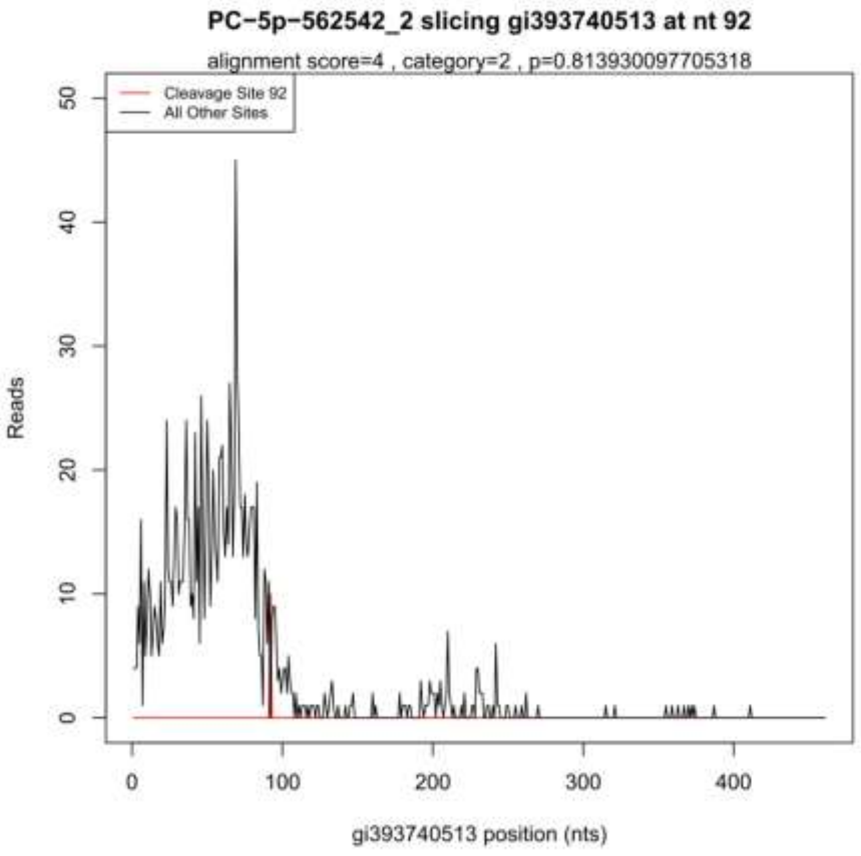
PC−5p−562542_2 slicing gi393740513 at nt 92
alignment score=4 , category=2 , p=0.813930097705318
Cleavage Site 92
All Other Sites
Reads
0
10
20
30
40
50
0
100
200
300
400
gi393740513 position (nts)

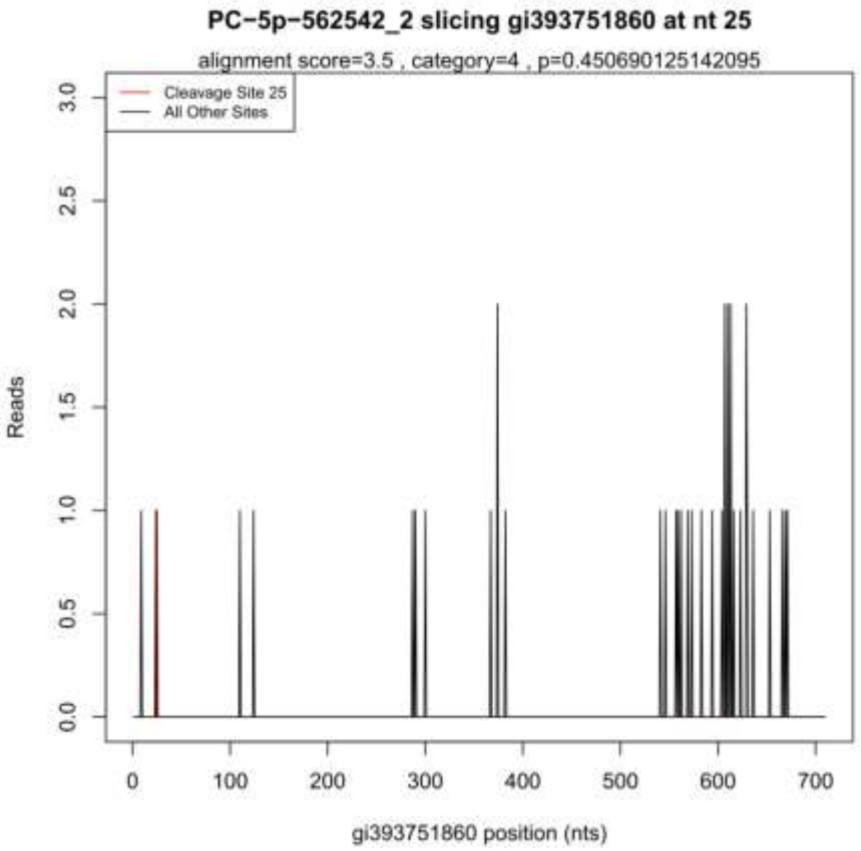
PC−5p−562542_2 slicing gi393751860 at nt 25
alignment score=3.5 , category=4 , p=0.450690125142095
Cleavage Site 25
All Other Sites
Reads
gi393751860 position (nts)

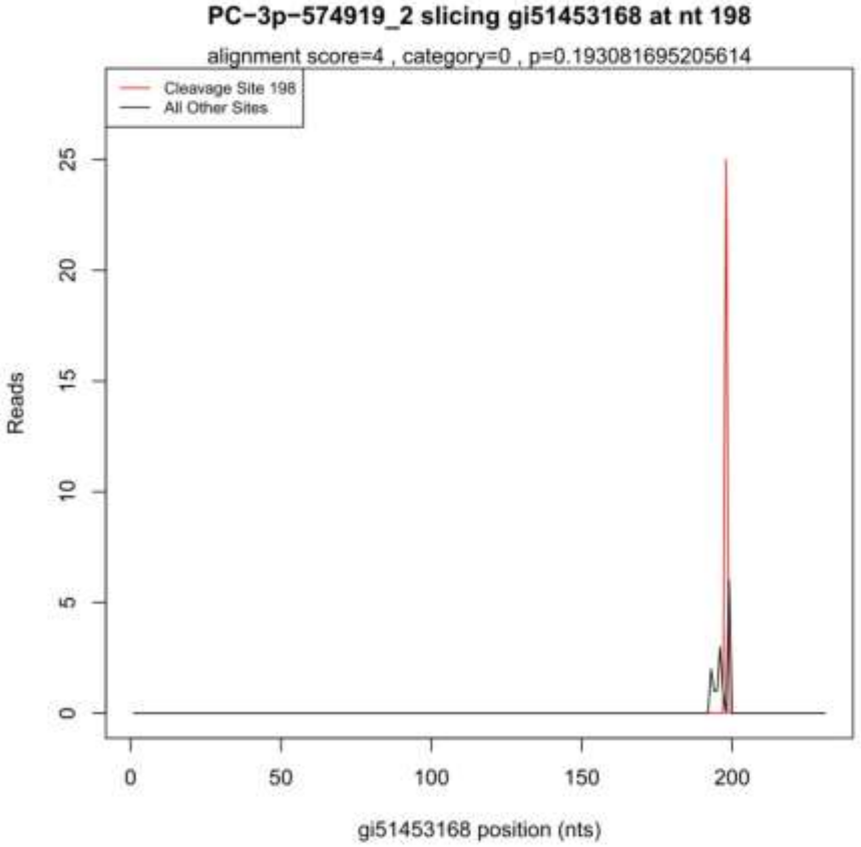
PC−3p−574919_2 slicing gi51453168 at nt 198
alignment score=4 , category=0 , p=0.193081695205614
Cleavage Site 198
All Other Sites
Reads
gi51453168 position (nts)

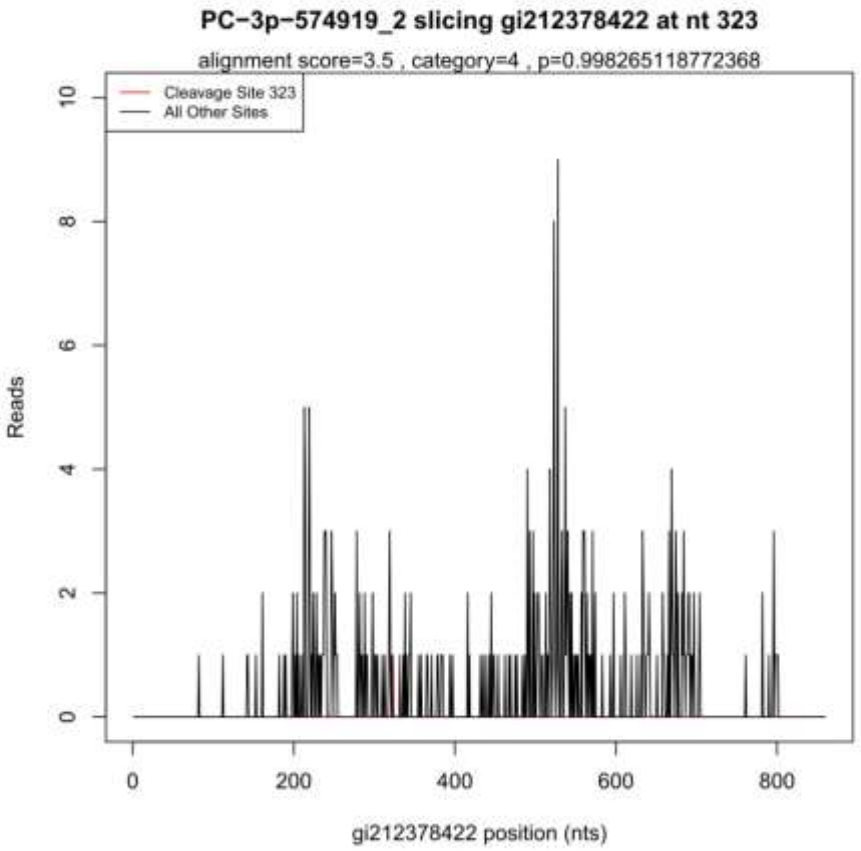
PC−3p−574919_2 slicing gi212378422 at nt 323
alignment score=3.5 , category=4 , p=0.998265118772368
Cleavage Site 323
All Other Sites
Reads
0
2
4
6
8
10
0
200
400
600
800
gi212378422 position (nts)

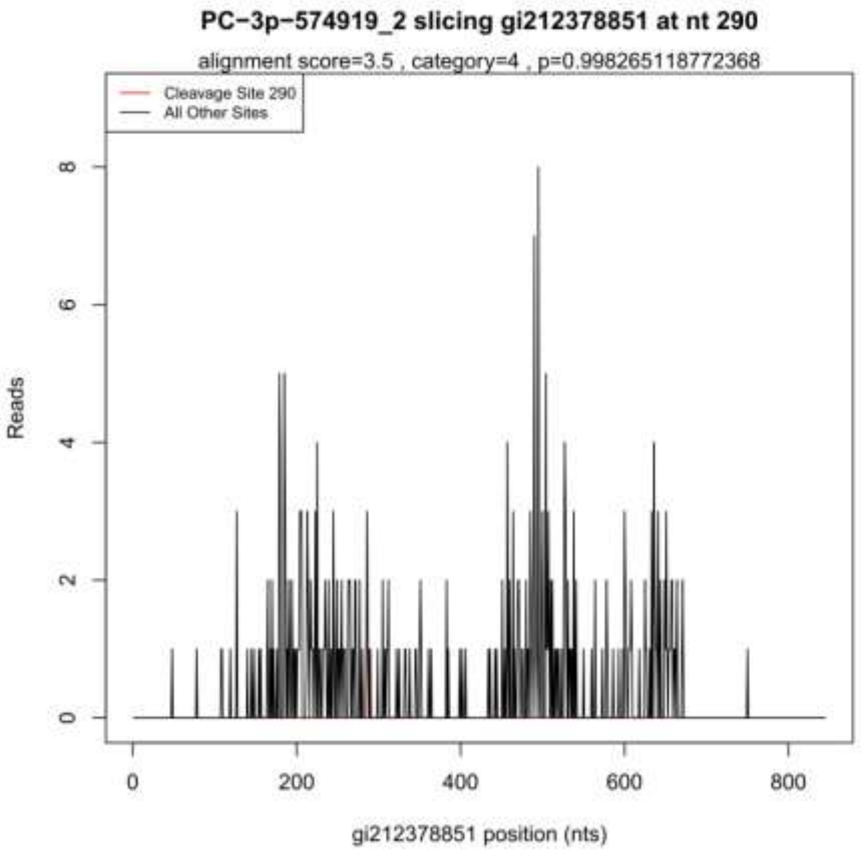
PC−3p−574919_2 slicing gi212378851 at nt 290
alignment score=3.5 , category=4 , p=0.998265118772368
Cleavage Site 290
All Other Sites
Reads
0
2
4
6
8
0
200
400
600
800
gi212378851 position (nts)

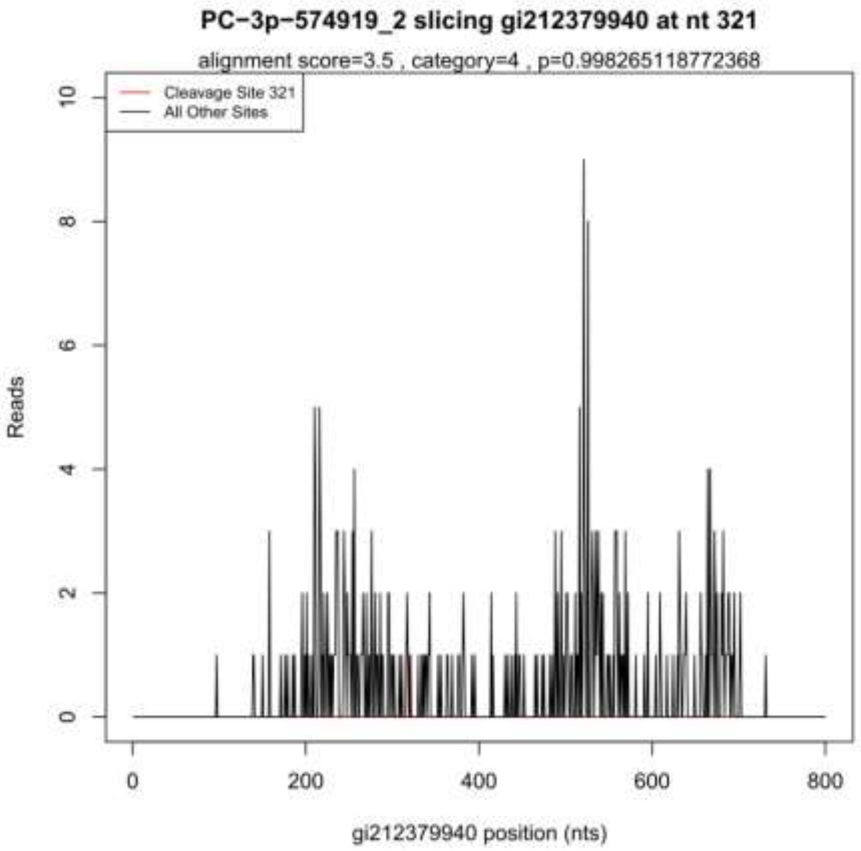
PC−3p−574919_2 slicing gi212379940 at nt 321
alignment score=3.5 , category=4 , p=0.998265118772368
Cleavage Site 321
All Other Sites
Reads
gi212379940 position (nts)

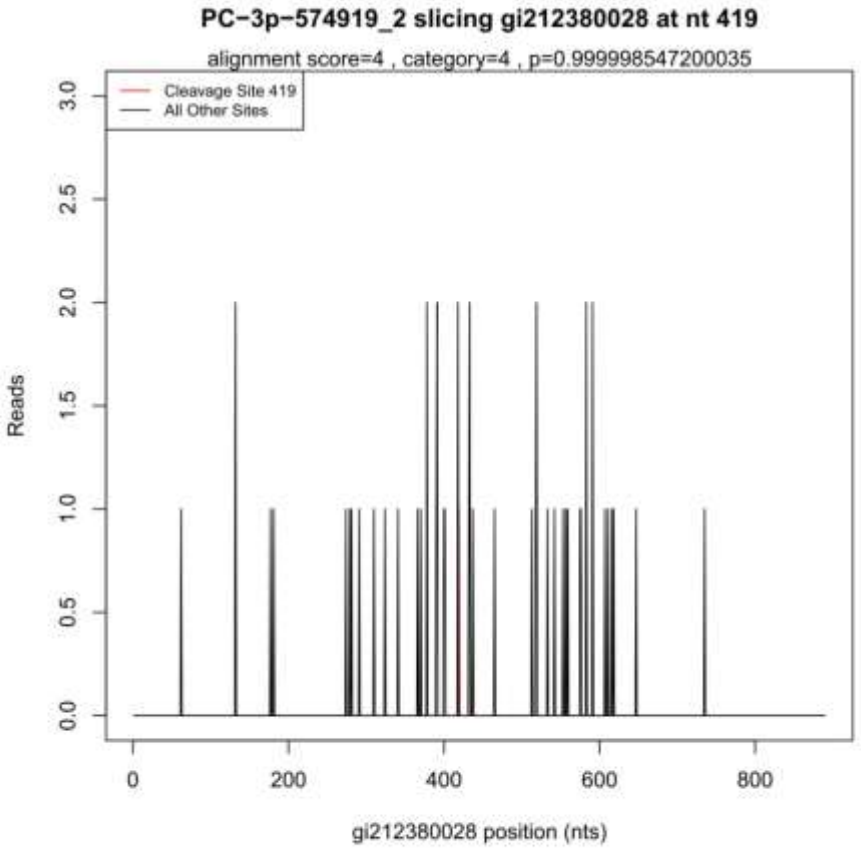
PC−3p−574919_2 slicing gi212380028 at nt 419
alignment score=4 , category=4 , p=0.999998547200035
Cleavage Site 419
All Other Sites
Reads
gi212380028 position (nts)

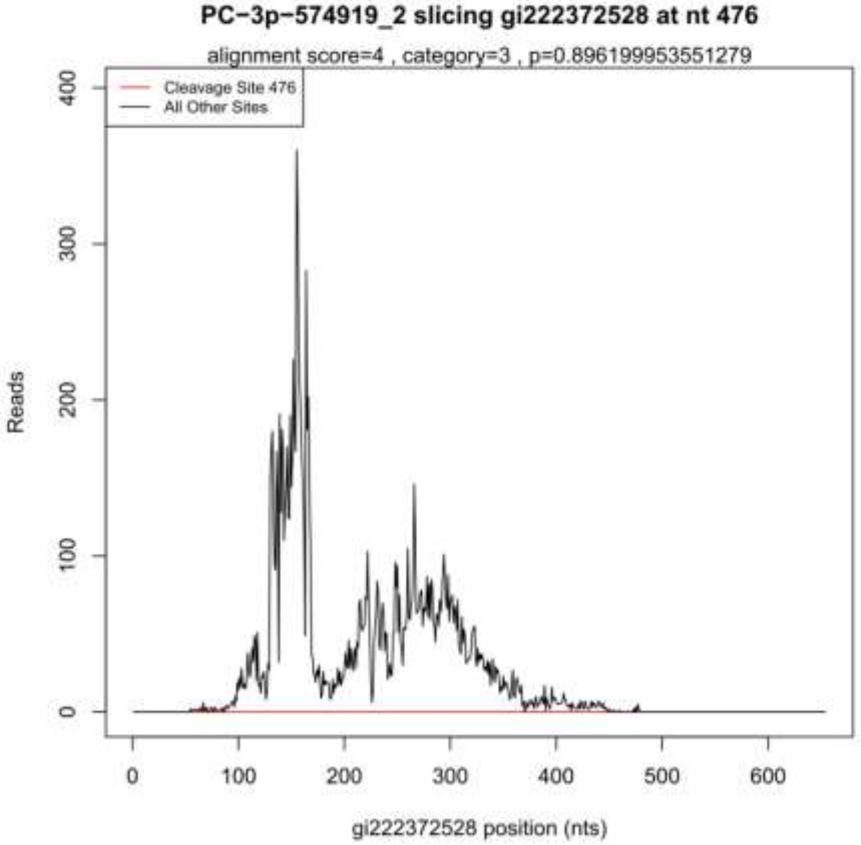
PC−3p−574919_2 slicing gi222372528 at nt 476
alignment score=4 , category=3 , p=0.896199953551279
Cleavage Site 476
All Other Sites
Reads
0
100
200
300
400
500
600
gi222372528 position (nts)

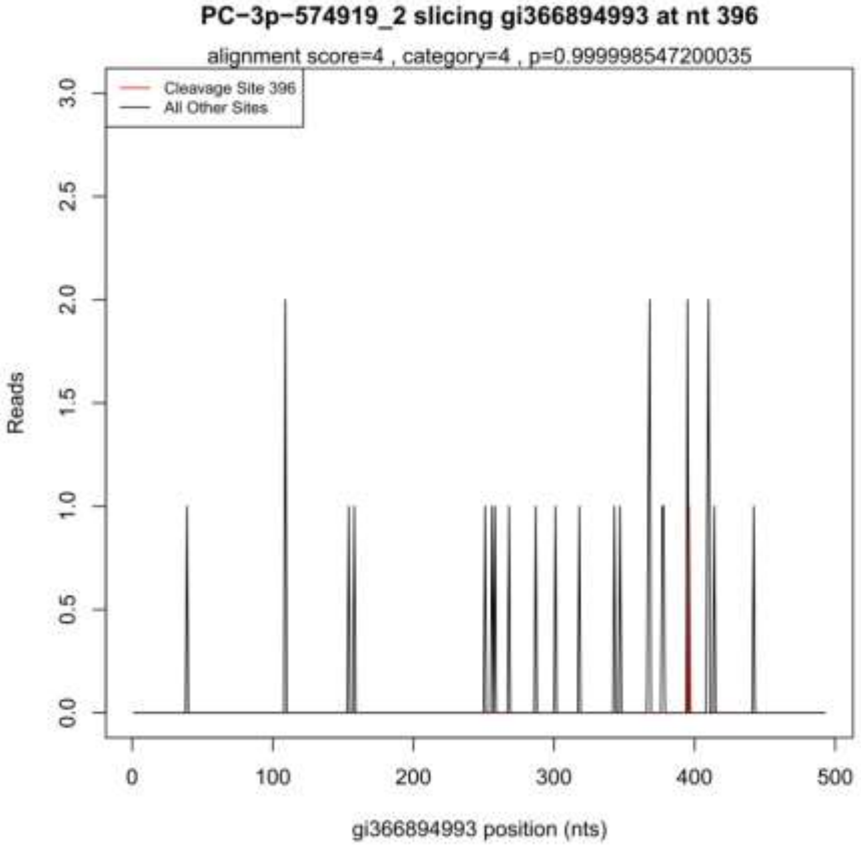
PC−3p−574919_2 slicing gi366894993 at nt 396
alignment score=4 , category=4 , p=0.999998547200035
Cleavage Site 396
All Other Sites
Reads
0.0
0.5
1.0
1.5
2.0
2.5
3.0
0
100
200
300
400
500
gi366894993 position (nts)

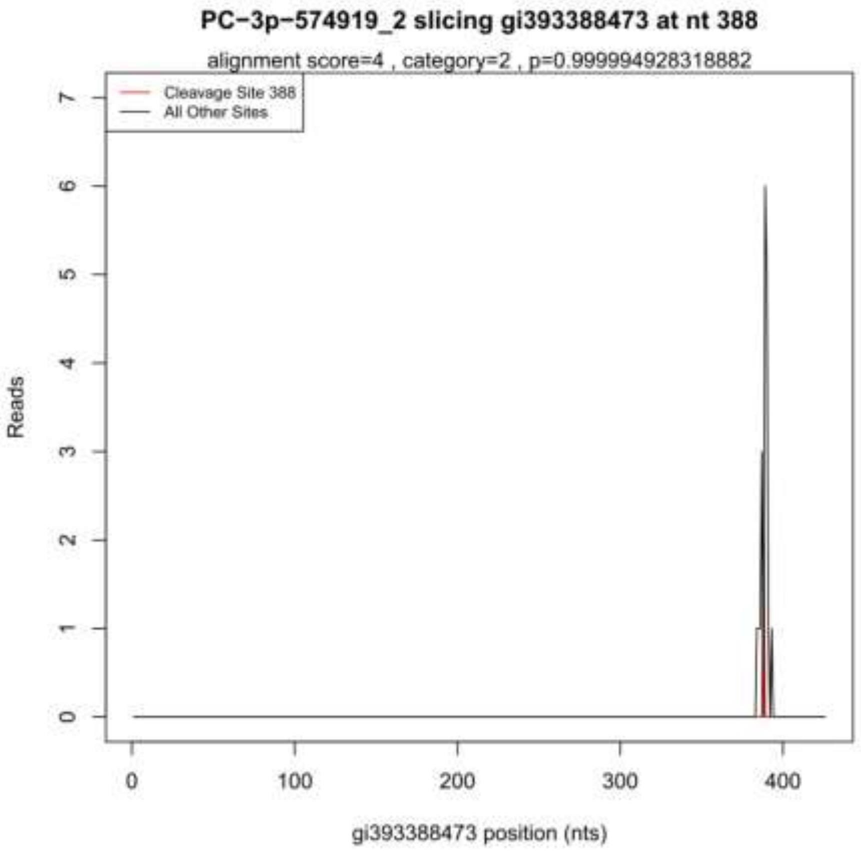
PC−3p−574919_2 slicing gi393388473 at nt 388
alignment score=4 , category=2 , p=0.999994928318882
Cleavage Site 388
All Other Sites
Reads
gi393388473 position (nts)

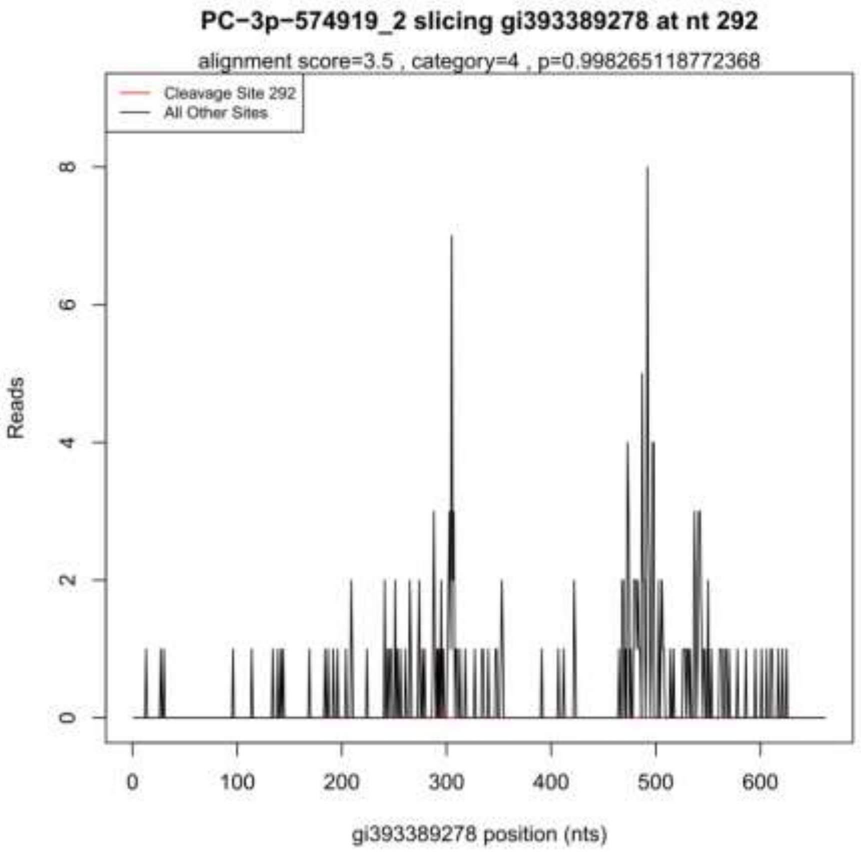
PC−3p−574919_2 slicing gi393389278 at nt 292
alignment score=3.5 , category=4 , p=0.998265118772368
Cleavage Site 292
All Other Sites
Reads
gi393389278 position (nts)

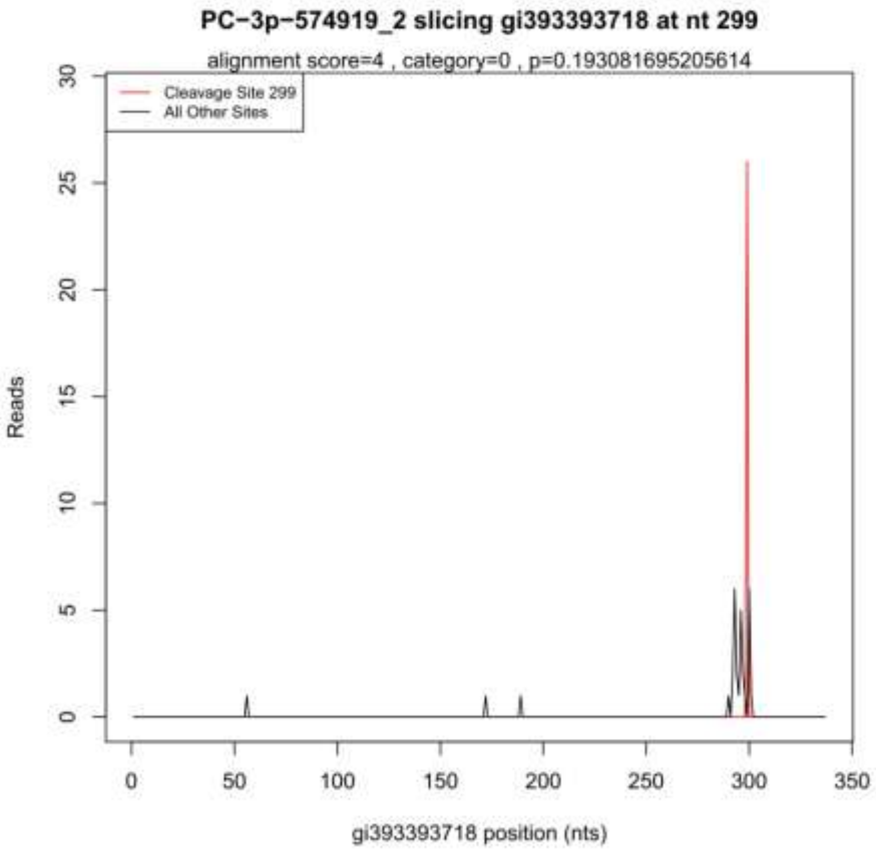
PC−3p−574919_2 slicing gi393393718 at nt 299
alignment score=4 , category=0 , p=0.193081695205614
Cleavage Site 299
All Other Sites
Reads
0
5
10
15
20
25
30
0
50
100
150
200
250
300
350
gi393393718 position (nts)

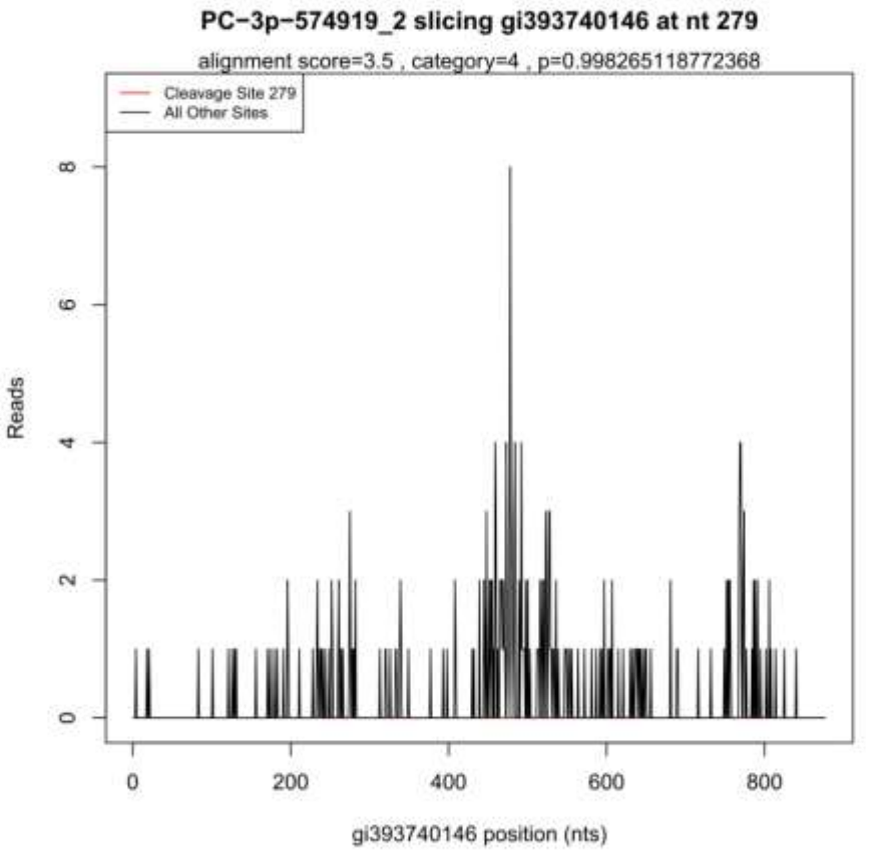
PC−3p−574919_2 slicing gi393740146 at nt 279
alignment score=3.5 , category=4 , p=0.998265118772368
Cleavage Site 279
All Other Sites
Reads
0
2
4
6
8
0
200
400
600
800
gi393740146 position (nts)

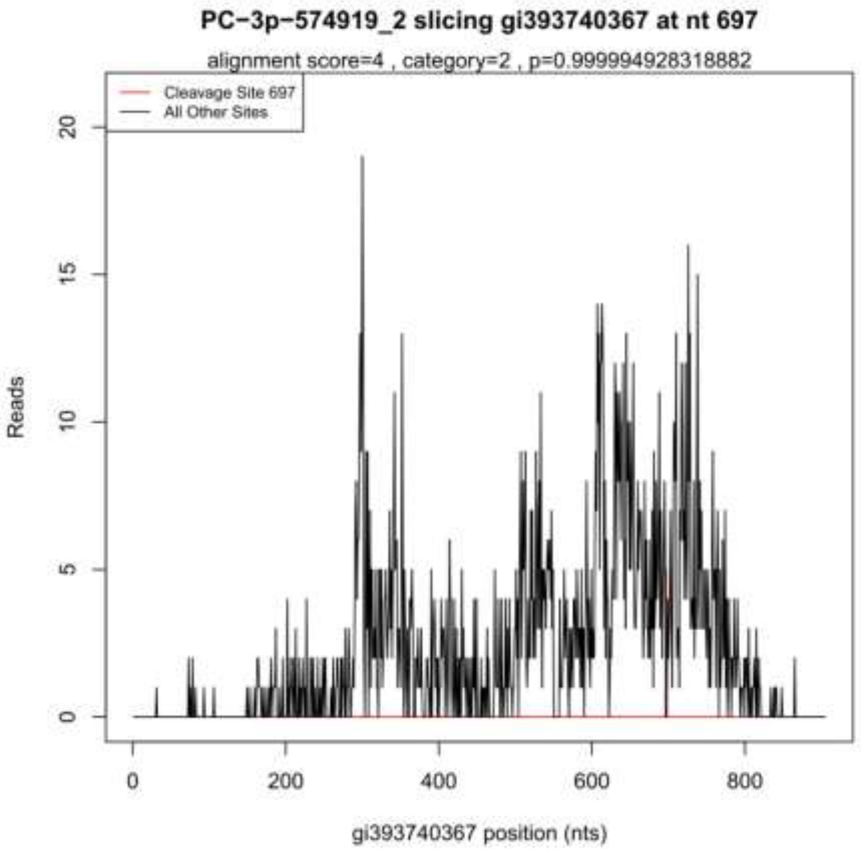
PC−3p−574919_2 slicing gi393740367 at nt 697
alignment score=4 , category=2 , p=0.999994928318882
Cleavage Site 697
All Other Sites
Reads
0
5
10
15
20
0
200
400
600
800
gi393740367 position (nts)

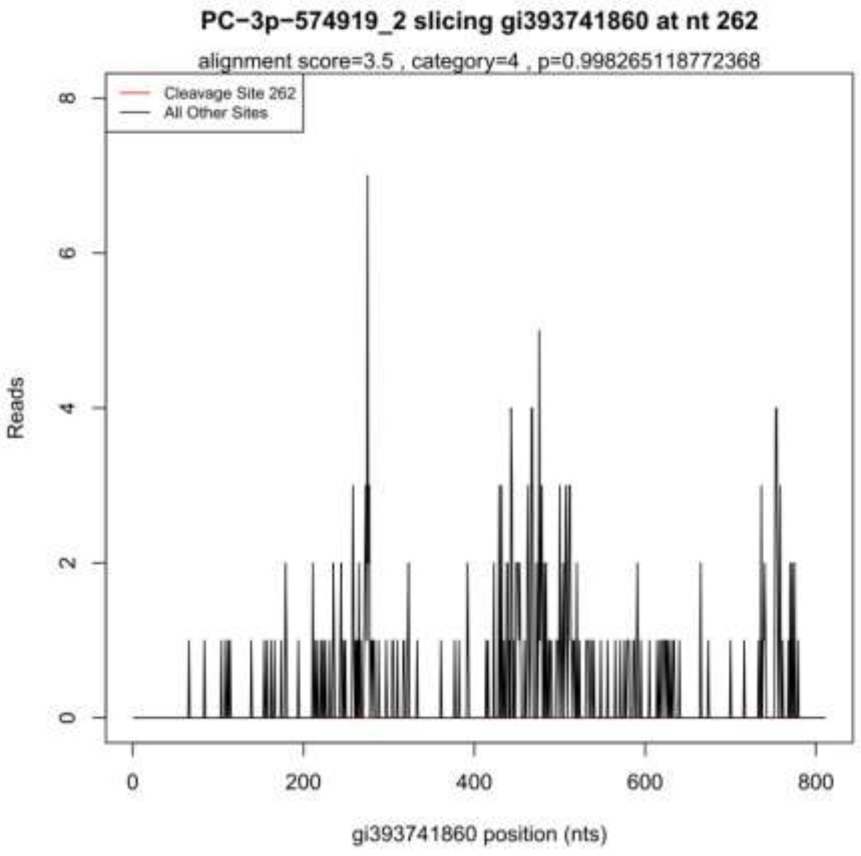
PC−3p−574919_2 slicing gi393741860 at nt 262
alignment score=3.5 , category=4 , p=0.998265118772368
Cleavage Site 262
All Other Sites
Reads
0
2
4
6
8
0
200
400
600
800
gi393741860 position (nts)

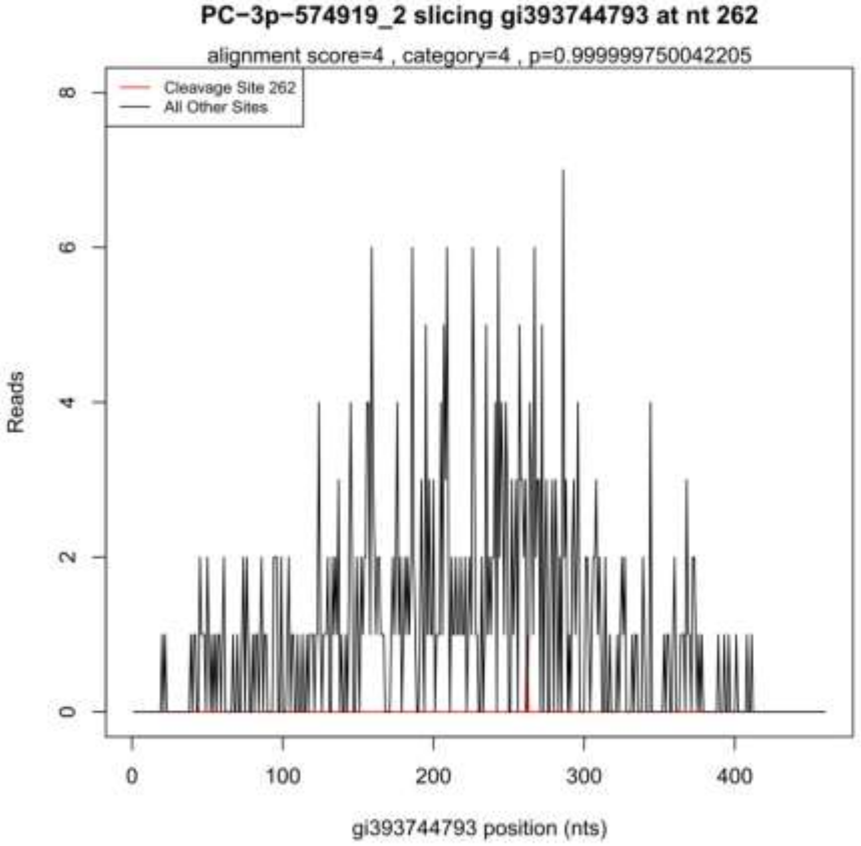

PC−3p−574919_2 slicing gi393744793 at nt 262
alignment score=4 , category=4 , p=0.999999750042205
Cleavage Site 262
All Other Sites
Reads
gi393744793 position (nts)

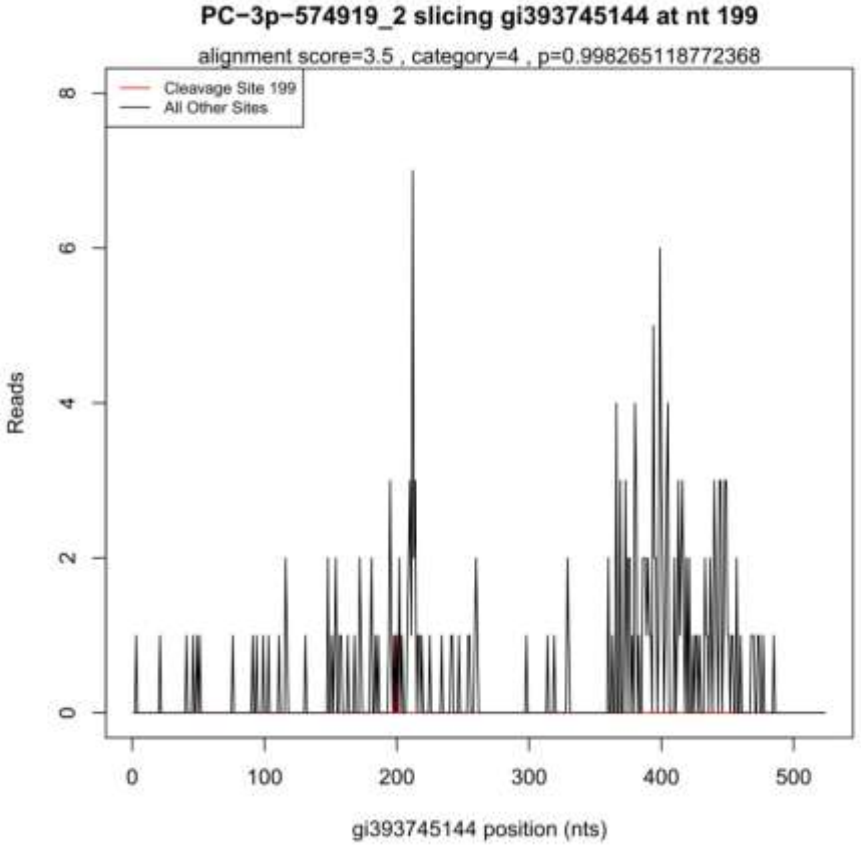

PC−3p−574919_2 slicing gi393745144 at nt 199
alignment score=3.5 , category=4 , p=0.998265118772368
Cleavage Site 199
All Other Sites
Reads
gi393745144 position (nts)

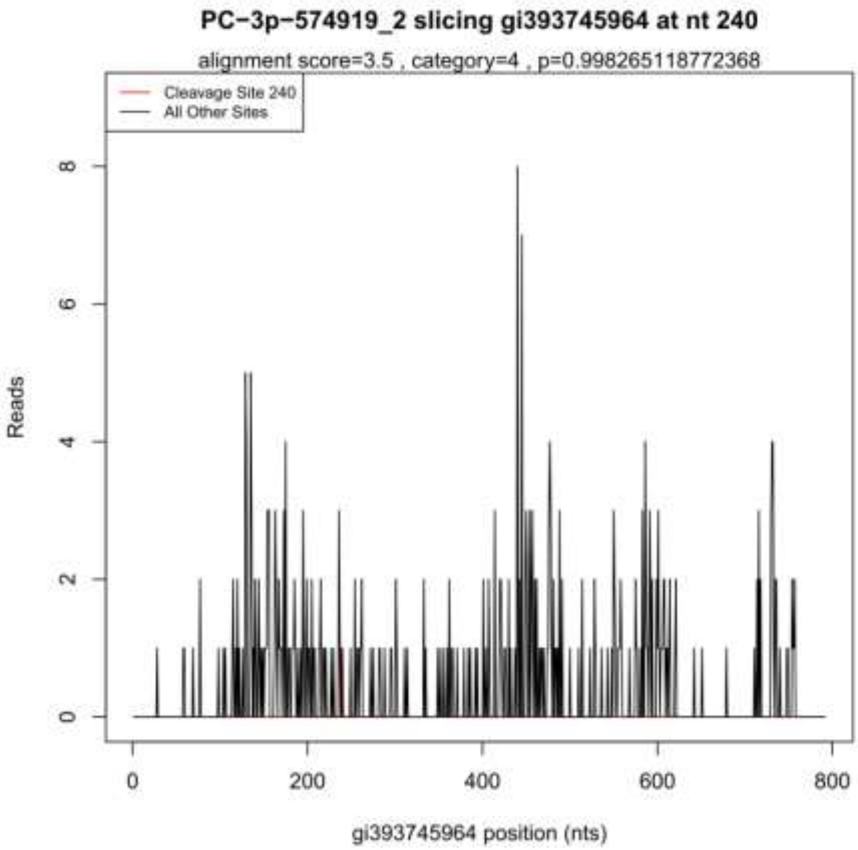
PC−3p−574919_2 slicing gi393745964 at nt 240
alignment score=3.5 , category=4 , p=0.998265118772368
Cleavage Site 240
All Other Sites
Reads
0
2
4
6
8
0
200
400
600
800
gi393745964 position (nts)

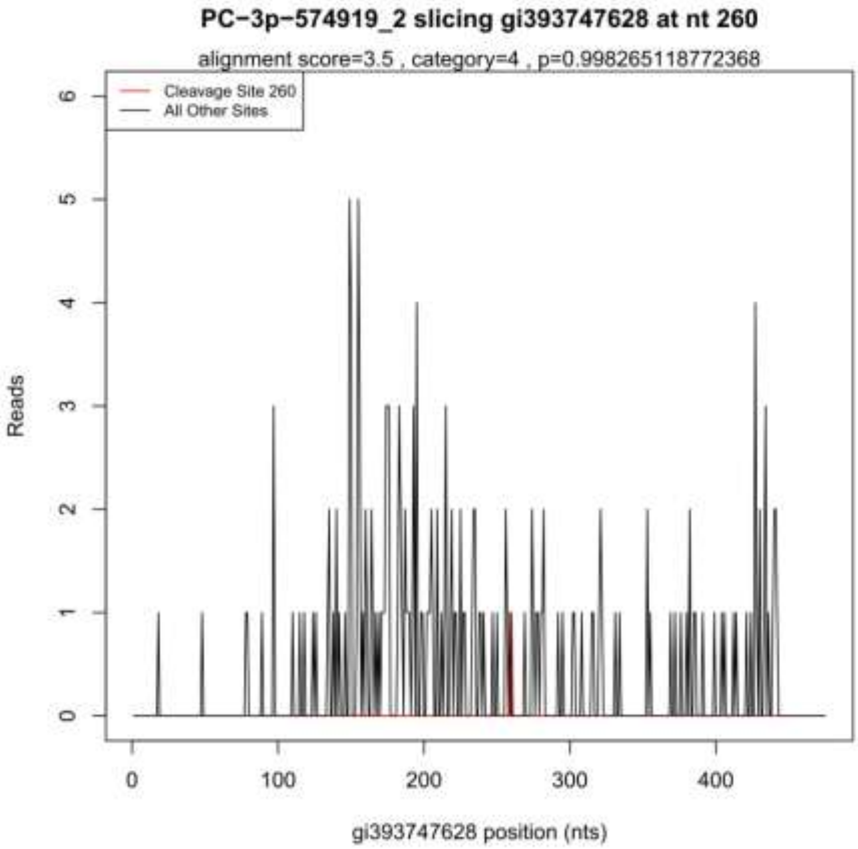
PC−3p−574919_2 slicing gi393747628 at nt 260
alignment score=3.5 , category=4 , p=0.998265118772368
Cleavage Site 260
All Other Sites
Reads
0
1
2
3
4
5
6
0
100
200
300
400
gi393747628 position (nts)

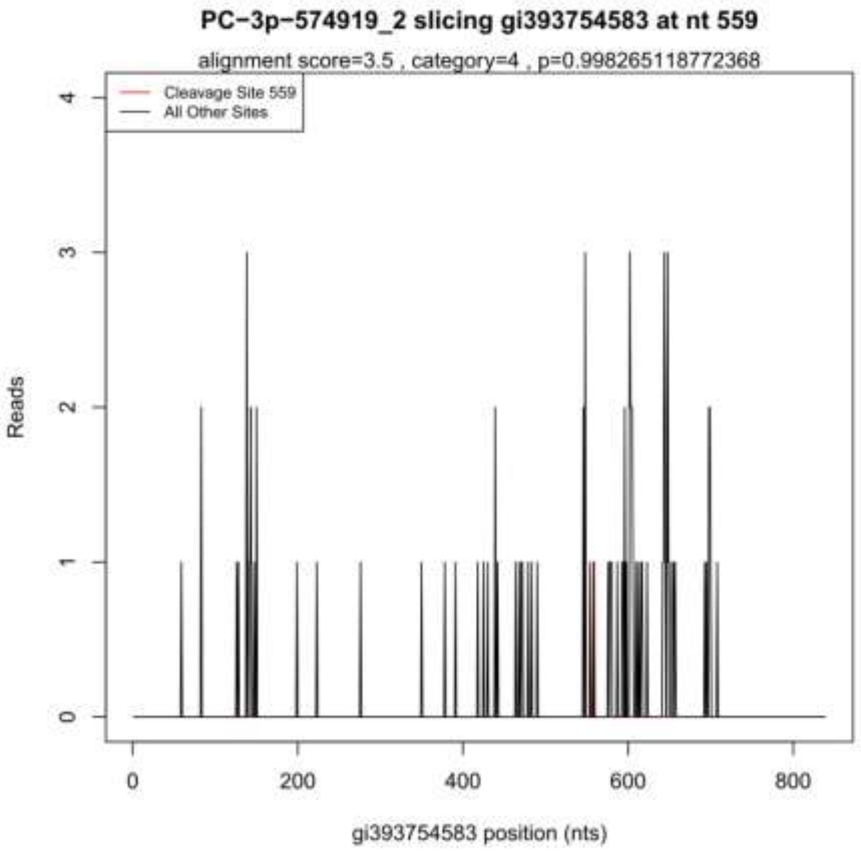
PC−3p−574919_2 slicing gi393754583 at nt 559
alignment score=3.5 , category=4 , p=0.998265118772368
Cleavage Site 559
All Other Sites
Reads
gi393754583 position (nts)

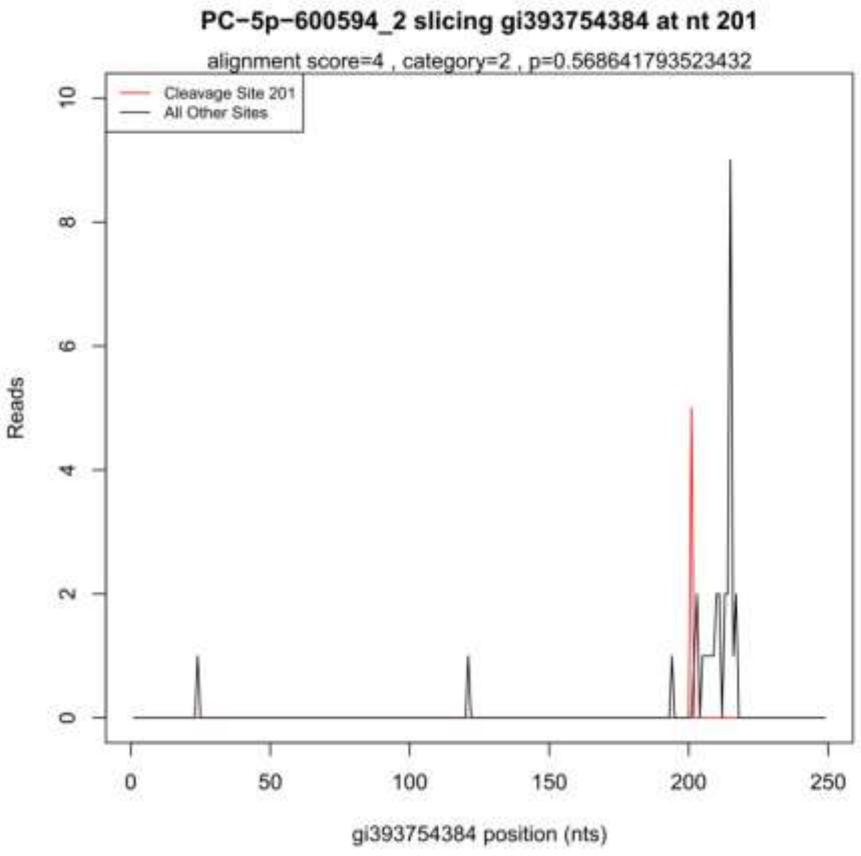
PC−5p−600594_2 slicing gi393754384 at nt 201
alignment score=4 , category=2 , p=0.568641793523432
Cleavage Site 201
All Other Sites
Reads
gi393754384 position (nts)

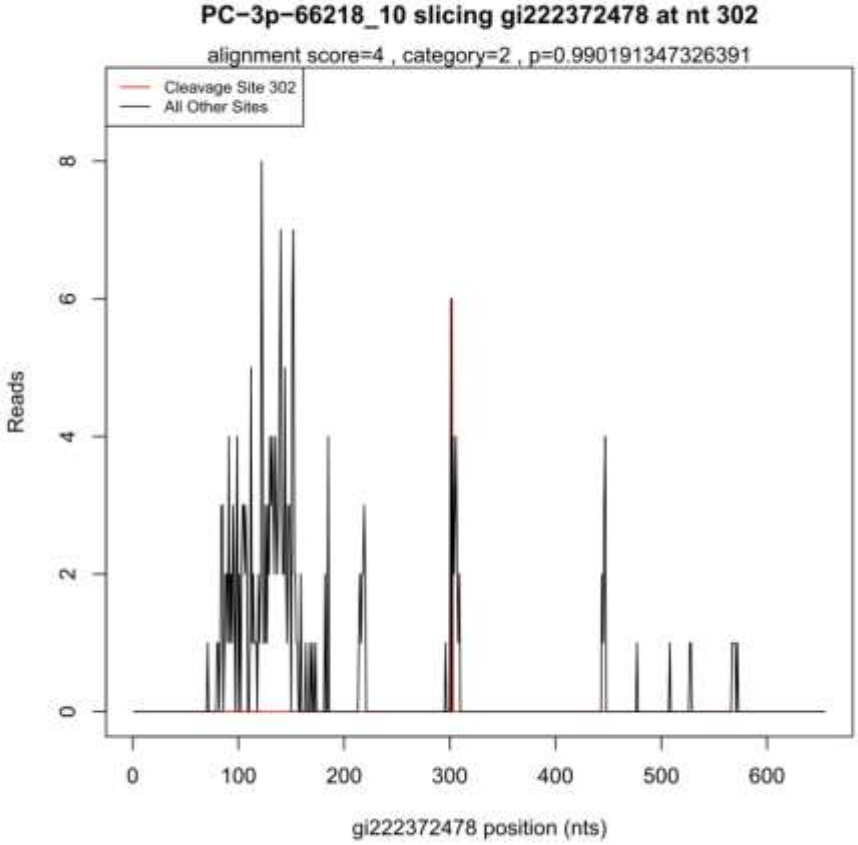

PC−3p−66218_10 slicing gi222372478 at nt 302
alignment score=4 , category=2 , p=0.990191347326391
Cleavage Site 302
All Other Sites
Reads
0
2
4
6
8
0
100
200
300
400
500
600
gi222372478 position (nts)

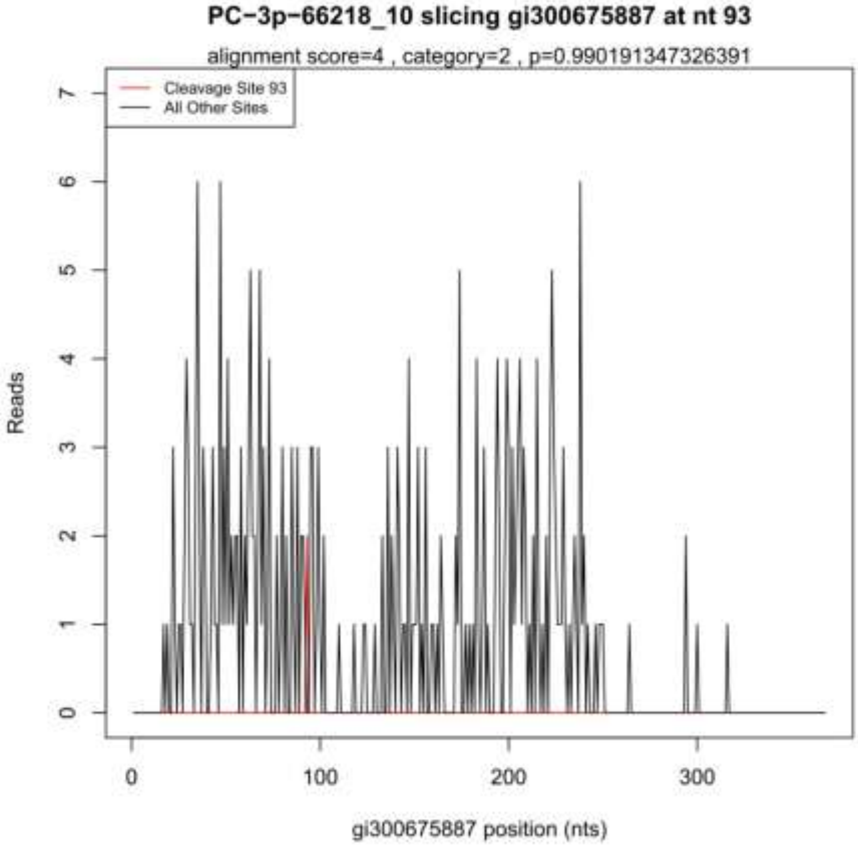

PC−3p−66218_10 slicing gi300675887 at nt 93
alignment score=4 , category=2 , p=0.990191347326391
Cleavage Site 93
All Other Sites
Reads
0
1
2
3
4
5
6
7
0
100
200
300
gi300675887 position (nts)

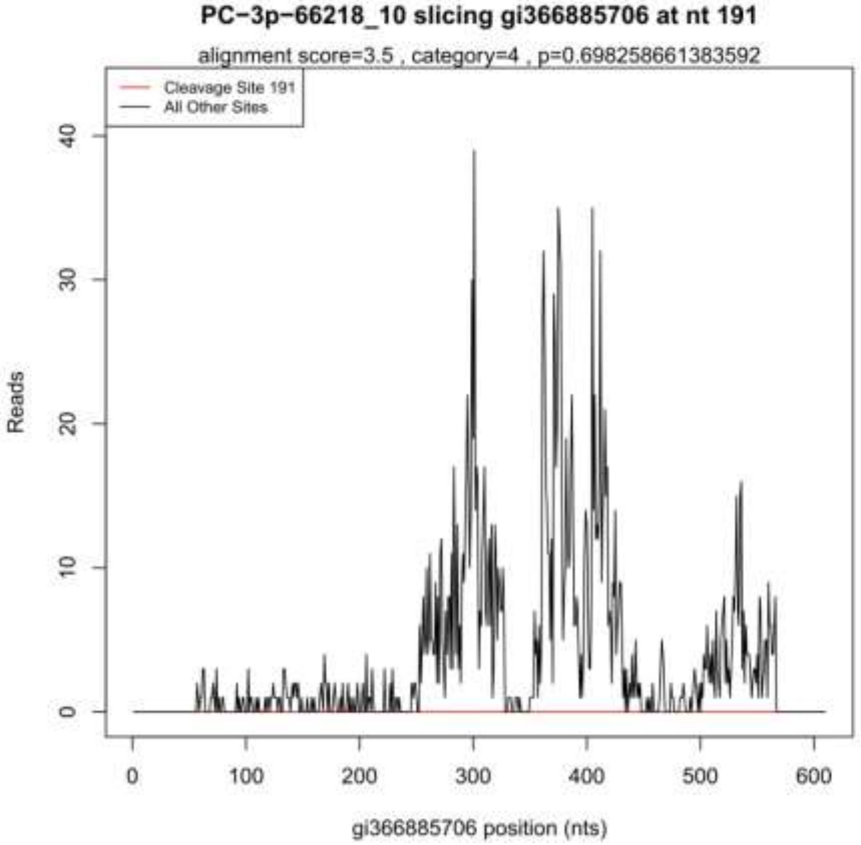
PC−3p−66218_10 slicing gi366885706 at nt 191
alignment score=3.5 , category=4 , p=0.698258661383592
Cleavage Site 191
All Other Sites
Reads
0
10
20
30
40
0
100
200
300
400
500
600
gi366885706 position (nts)

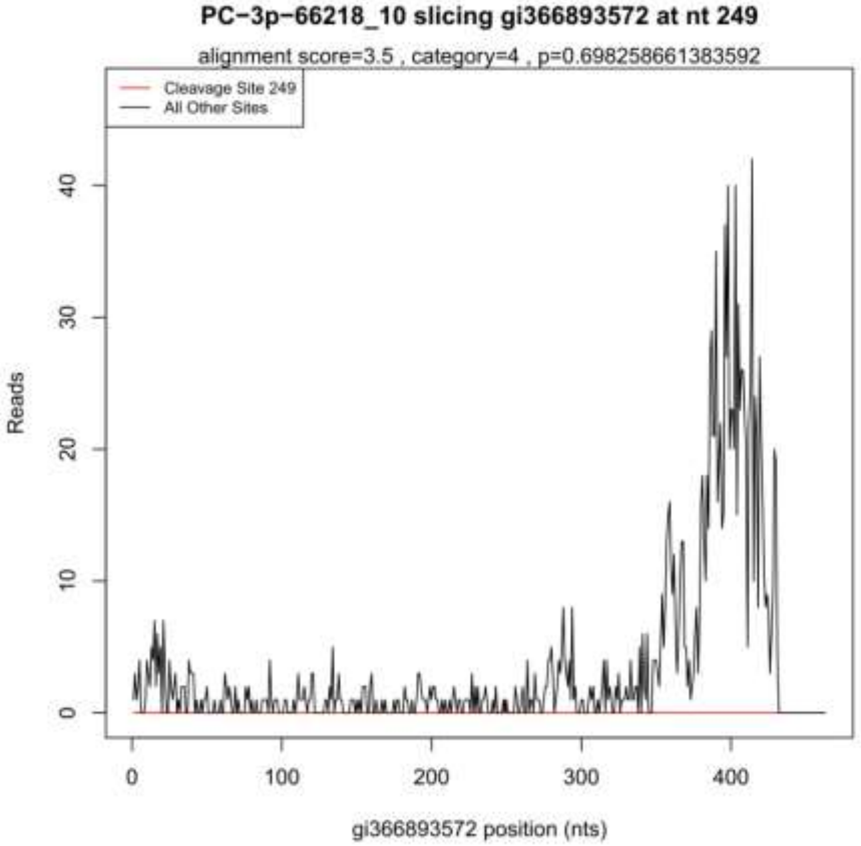
PC−3p−66218_10 slicing gi366893572 at nt 249
alignment score=3.5 , category=4 , p=0.698258661383592
Cleavage Site 249
All Other Sites
Reads
0
10
20
30
40
0
100
200
300
400
gi366893572 position (nts)

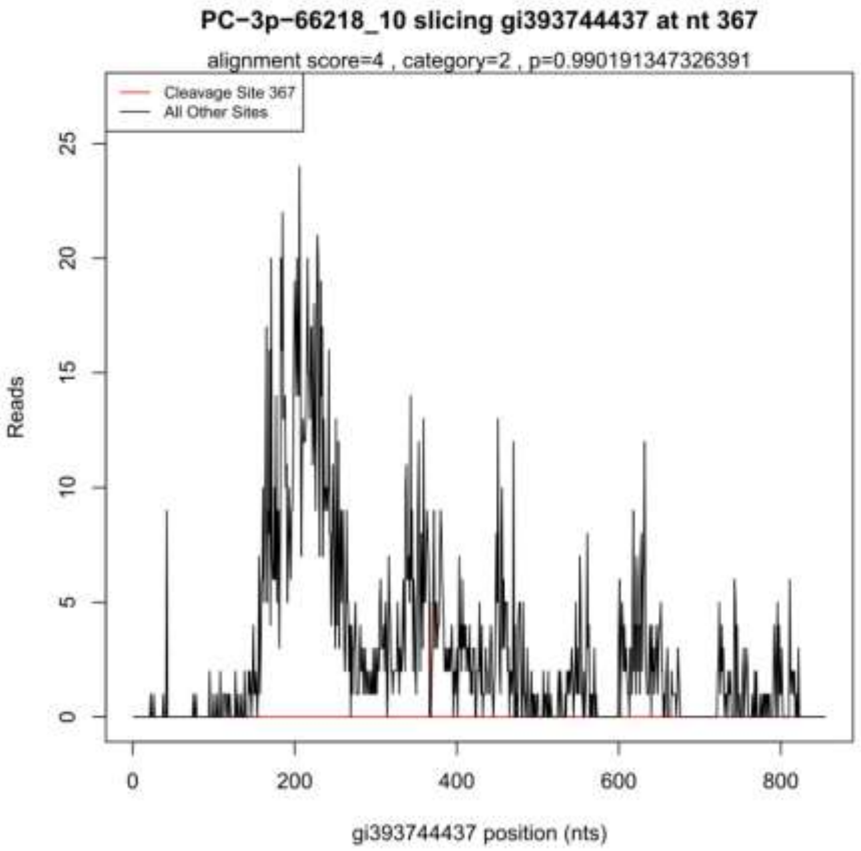
PC−3p−66218_10 slicing gi393744437 at nt 367
alignment score=4 , category=2 , p=0.990191347326391
Cleavage Site 367
All Other Sites
Reads
0
5
10
15
20
25
0
200
400
600
800
gi393744437 position (nts)

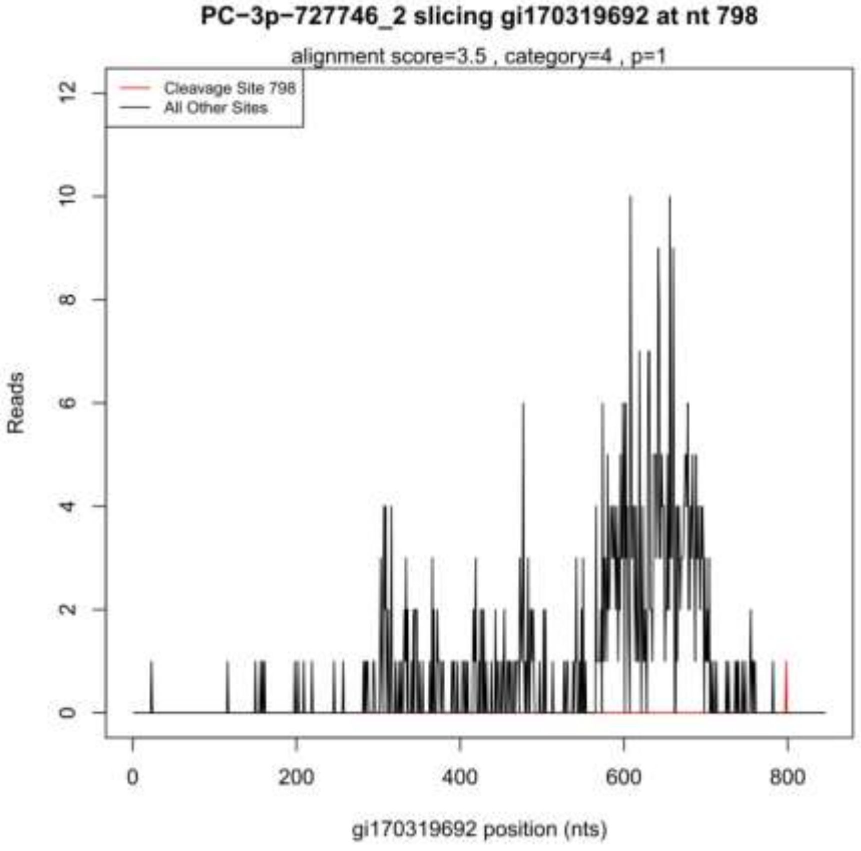
PC−3p−727746_2 slicing gi170319692 at nt 798
alignment score=3.5 , category=4 , p=1
Cleavage Site 798
All Other Sites
Reads
0
2
4
6
8
10
12
0
200
400
600
800
gi170319692 position (nts)

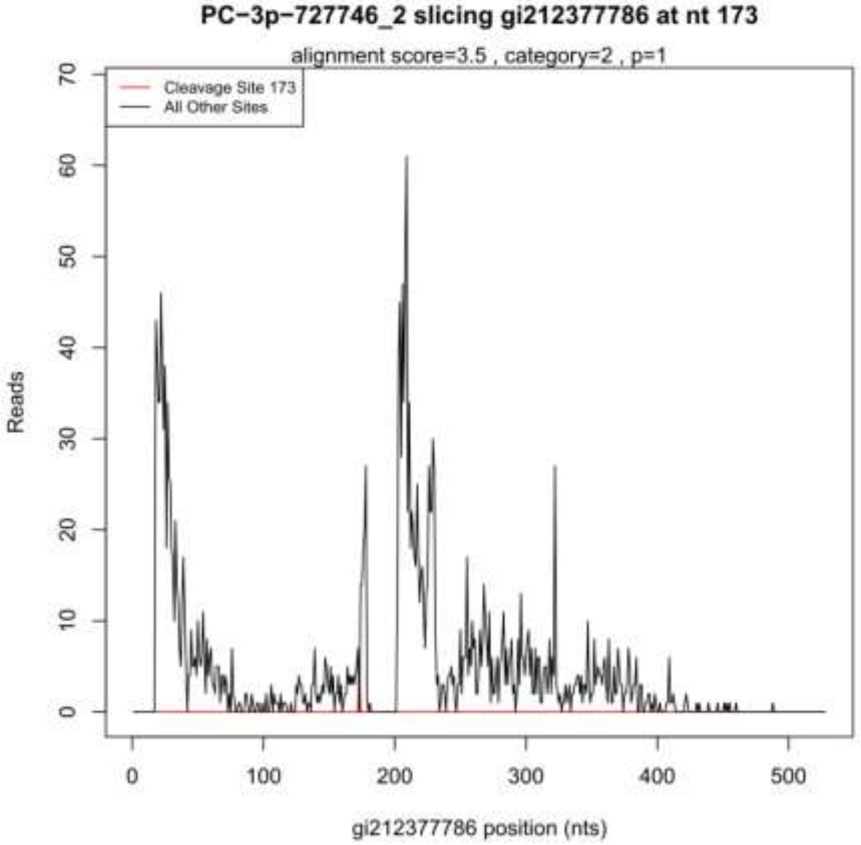
PC−3p−727746_2 slicing gi212377786 at nt 173
alignment score=3.5 , category=2 , p=1
Cleavage Site 173
All Other Sites
Reads
0
10
20
30
40
50
60
70
0
100
200
300
400
500
gi212377786 position (nts)

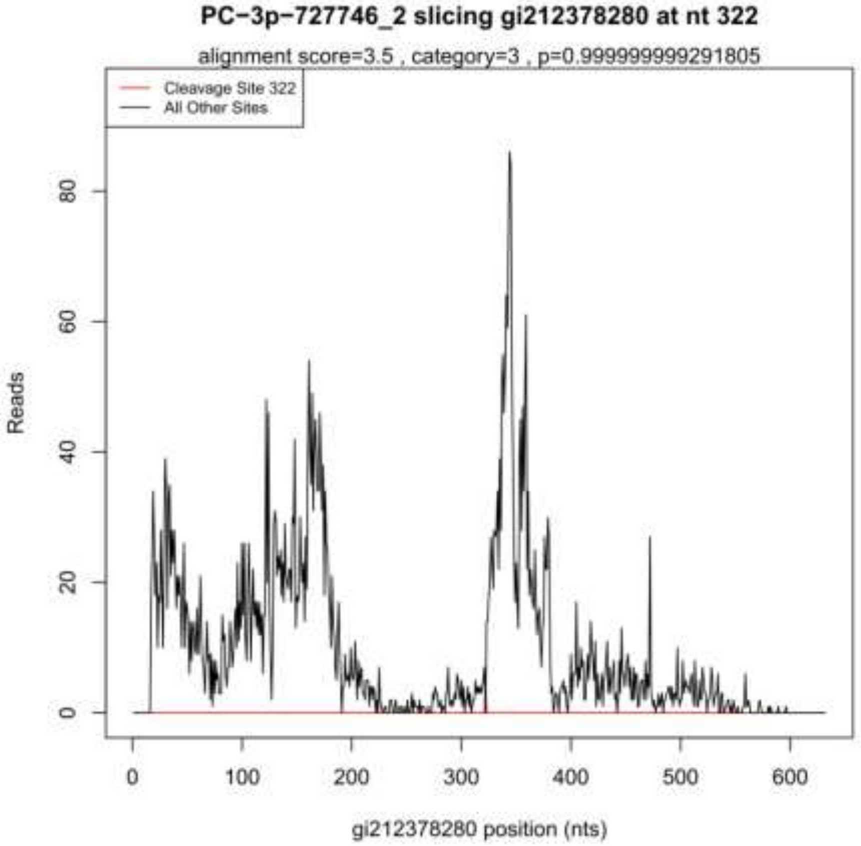
PC−3p−727746_2 slicing gi212378280 at nt 322
alignment score=3.5 , category=3 , p=0.999999999291805
Cleavage Site 322
All Other Sites
Reads
0
20
40
60
80
0
100
200
300
400
500
600
gi212378280 position (nts)

PC−3p−727746_2 slicing gi393393386 at nt 66

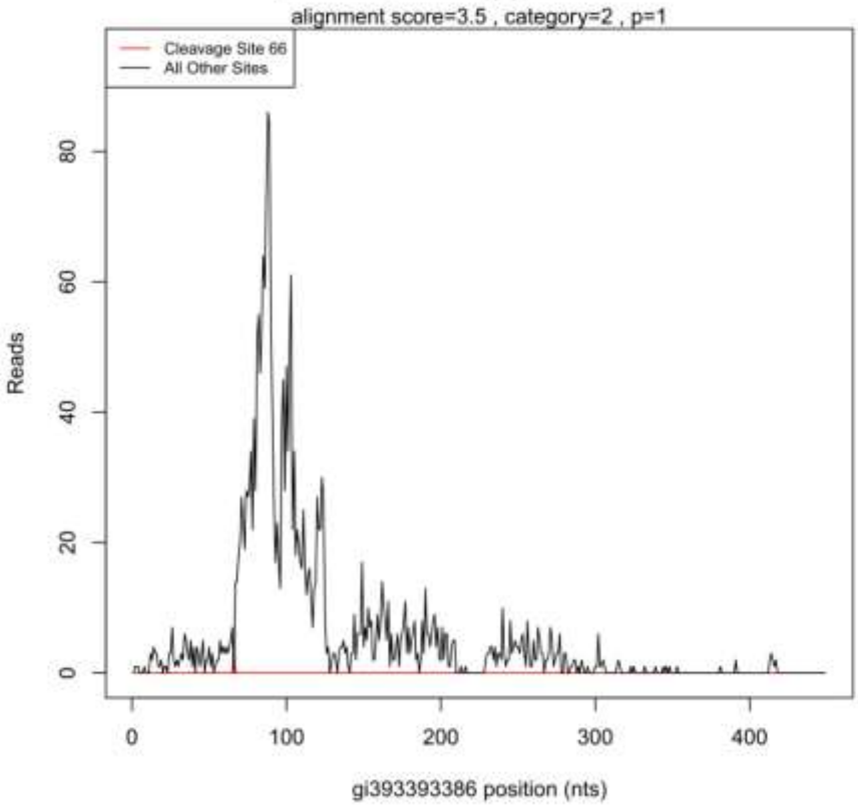

PC−3p−727746_2 slicing gi393740969 at nt 427

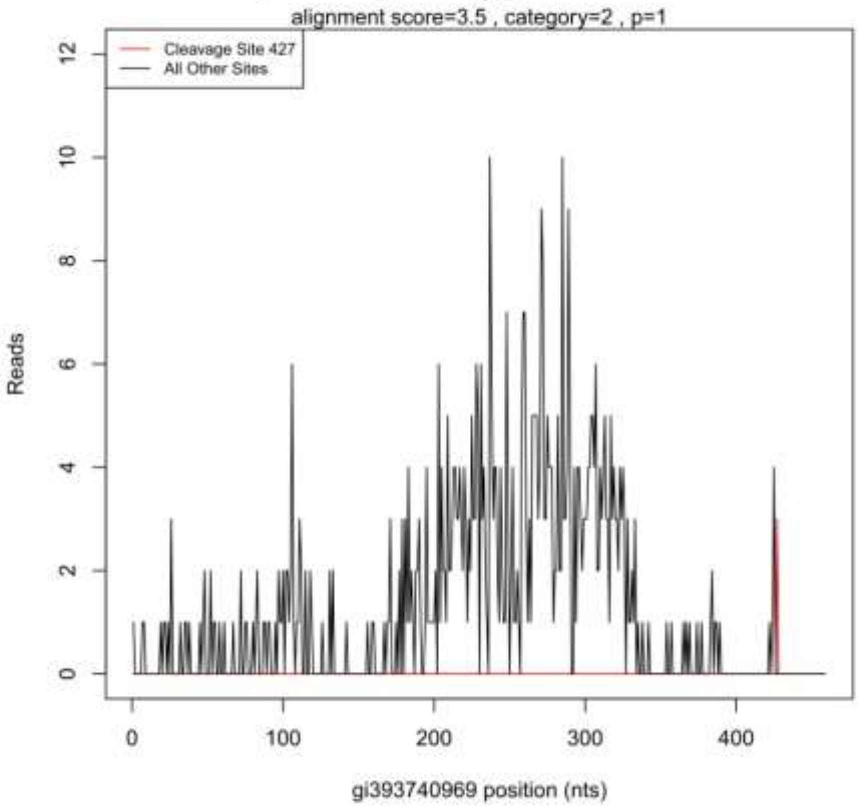

PC−3p−727746_2 slicing gi393756208 at nt 652

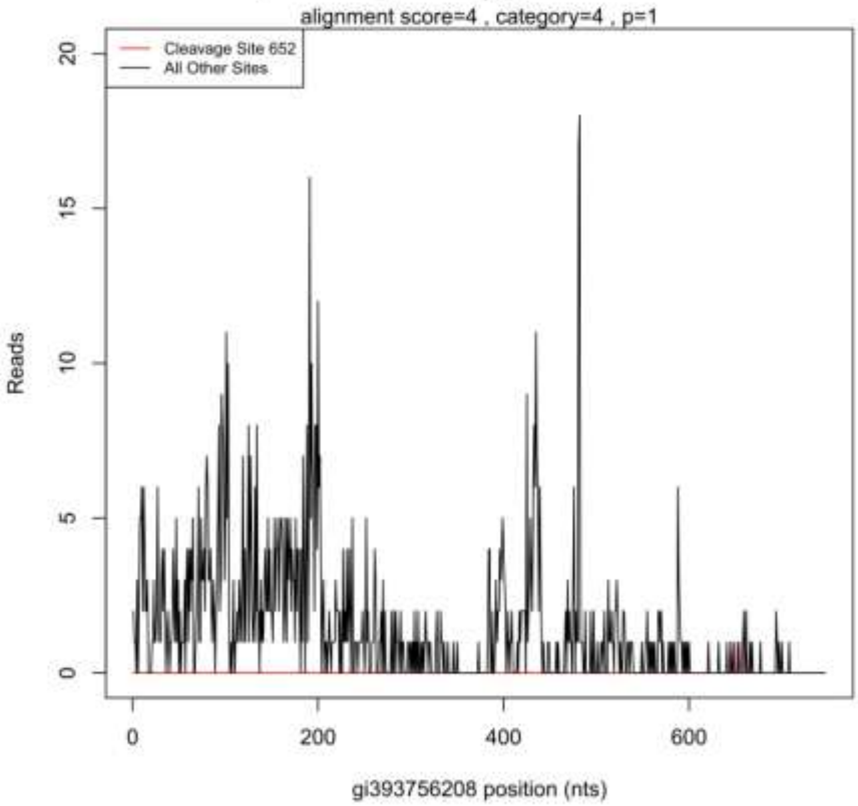

PC−3p−727746_2 slicing gi393756234 at nt 287

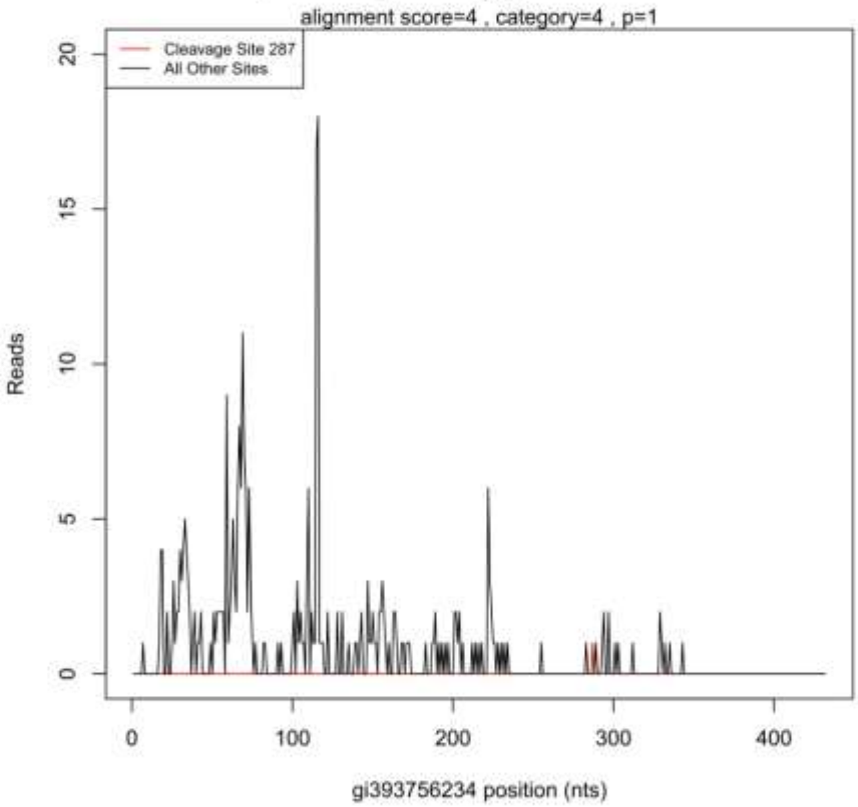

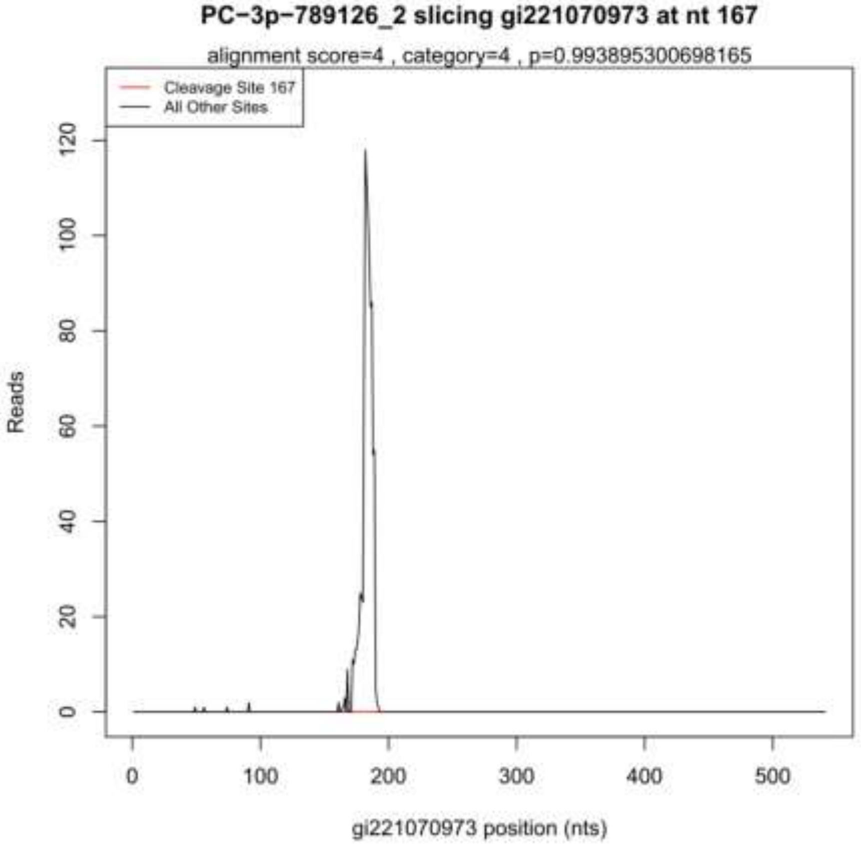
PC−3p−789126_2 slicing gi221070973 at nt 167
alignment score=4 , category=4 , p=0.993895300698165
Cleavage Site 167
All Other Sites
Reads
gi221070973 position (nts)

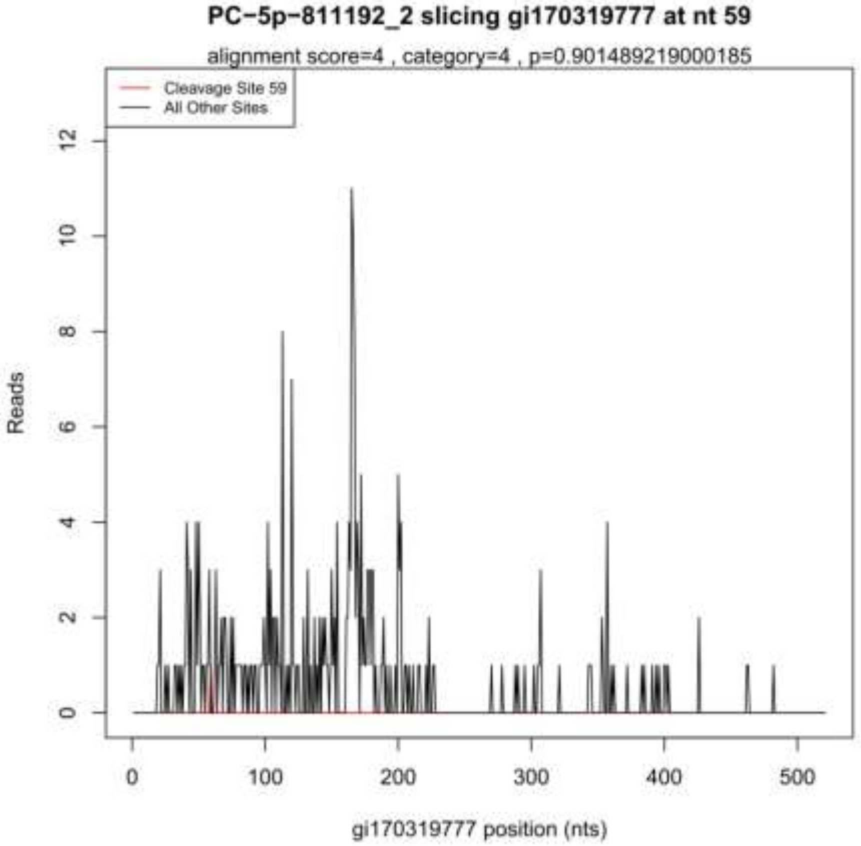
PC−5p−811192_2 slicing gi170319777 at nt 59
alignment score=4 , category=4 , p=0.901489219000185
Cleavage Site 59
All Other Sites
Reads
gi170319777 position (nts)

PC−5p−811192_2 slicing gi393389151 at nt 435

alignment score=4 , category=4 , p=0.901489219000185

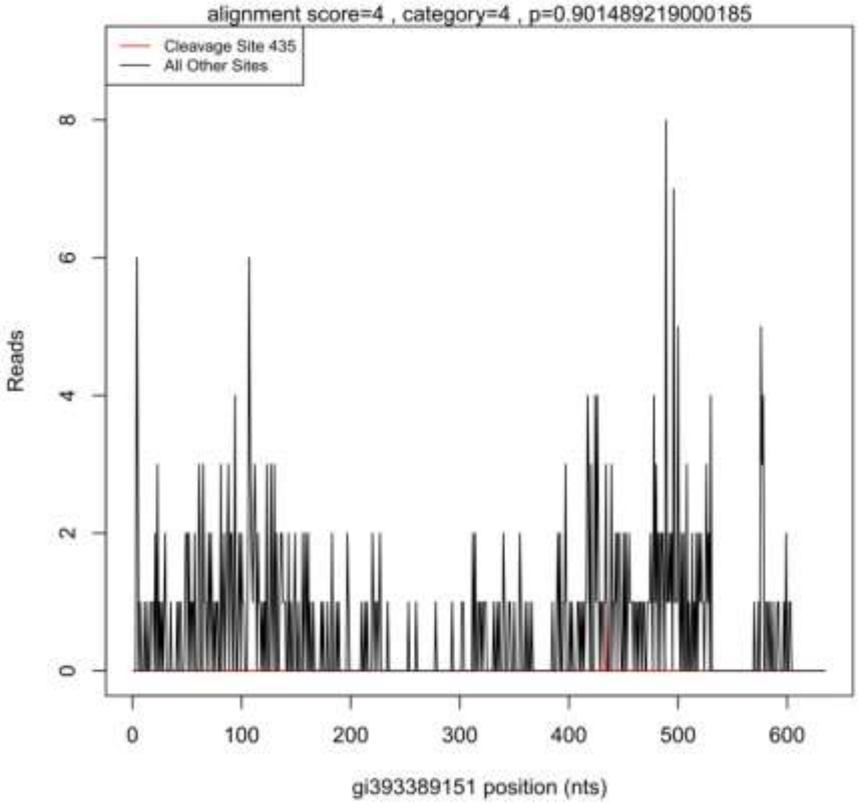

PC−3p−834235_2 slicing gi393749009 at nt 678

alignment score=3 , category=4 , p=0.448962013083513

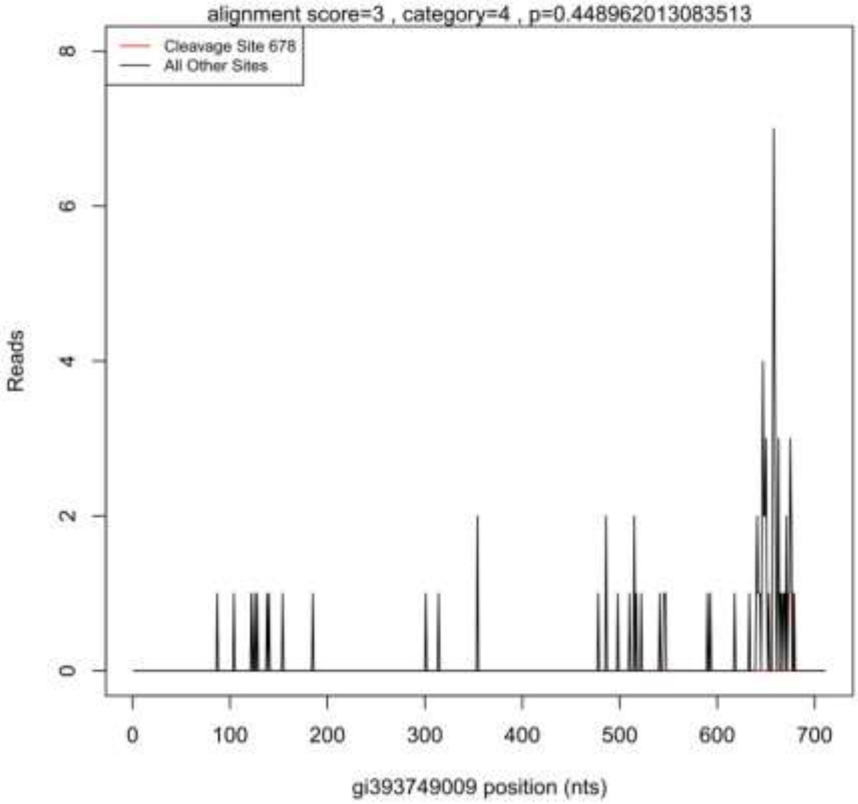

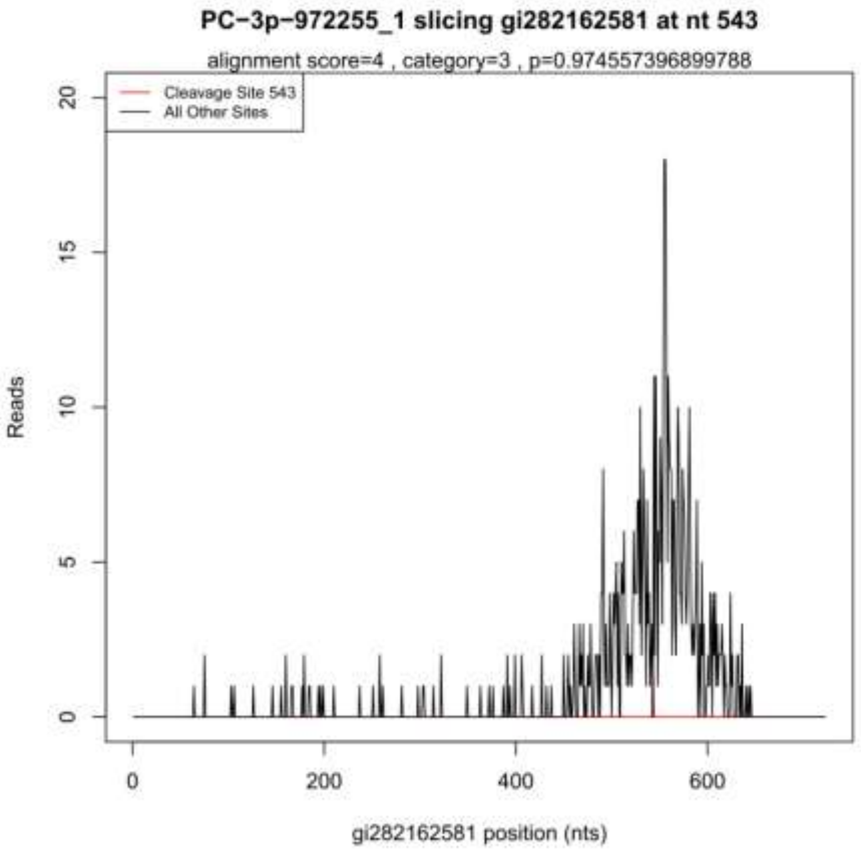
PC−3p−972255_1 slicing gi282162581 at nt 543
alignment score=4 , category=3 , p=0.974557396899788
Cleavage Site 543
All Other Sites
Reads
0
5
10
15
20
0
200
400
600
gi282162581 position (nts)

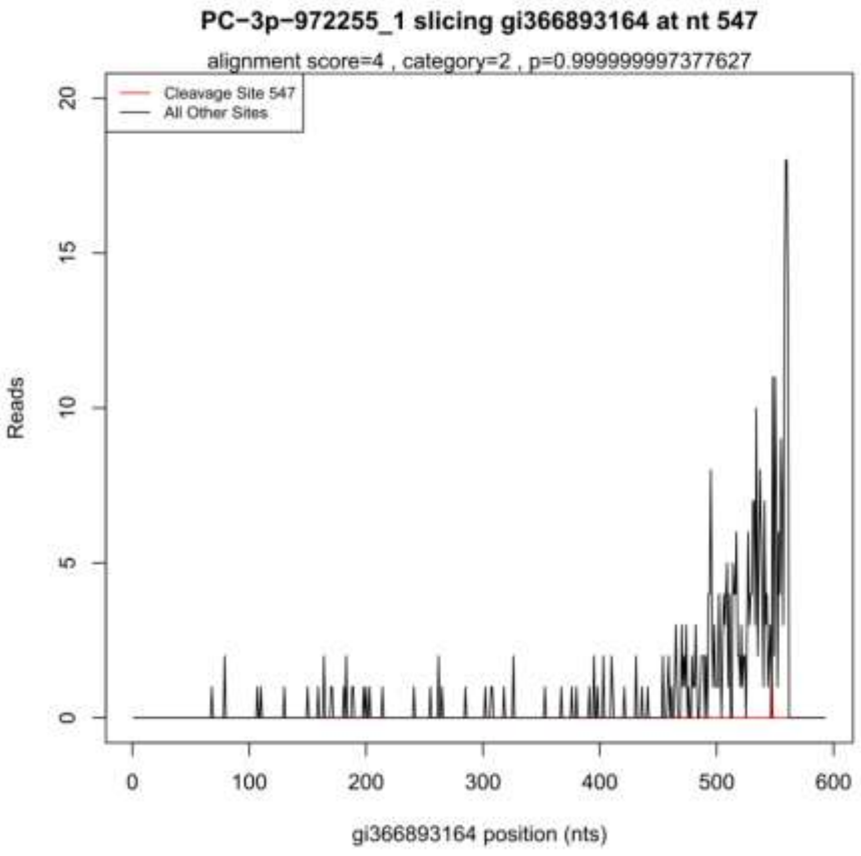
PC−3p−972255_1 slicing gi366893164 at nt 547
alignment score=4 , category=2 , p=0.999999997377627
Cleavage Site 547
All Other Sites
Reads
0
5
10
15
20
0
100
200
300
400
500
600
gi366893164 position (nts)

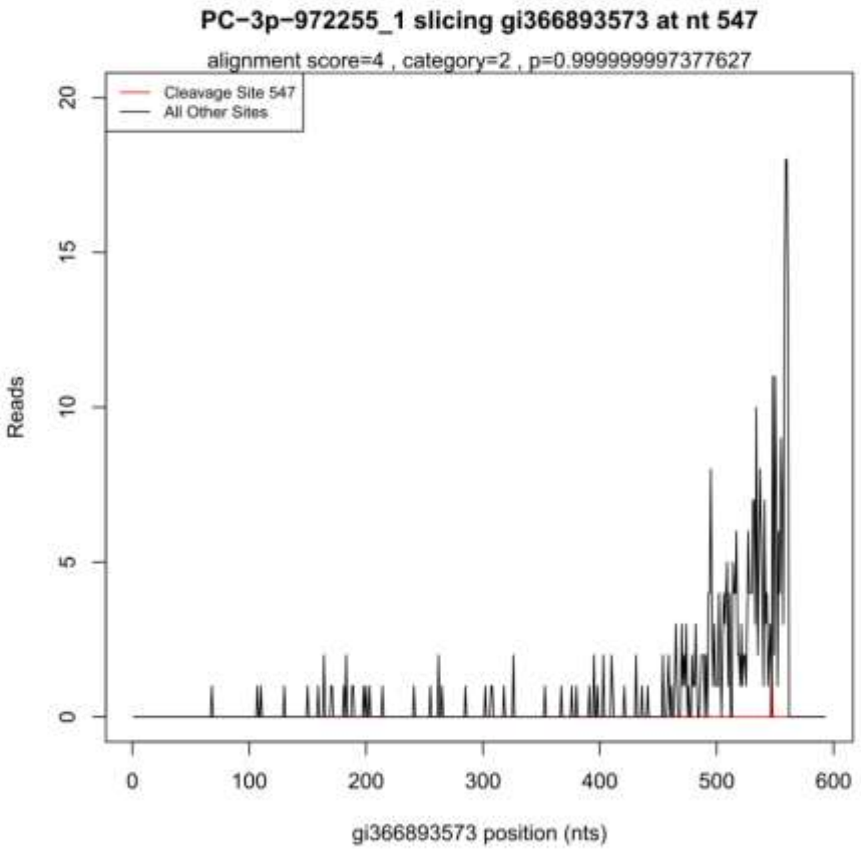
PC−3p−972255_1 slicing gi366893573 at nt 547
alignment score=4 , category=2 , p=0.999999997377627
Cleavage Site 547
All Other Sites
Reads
0
5
10
15
20
0
100
200
300
400
500
600
gi366893573 position (nts)

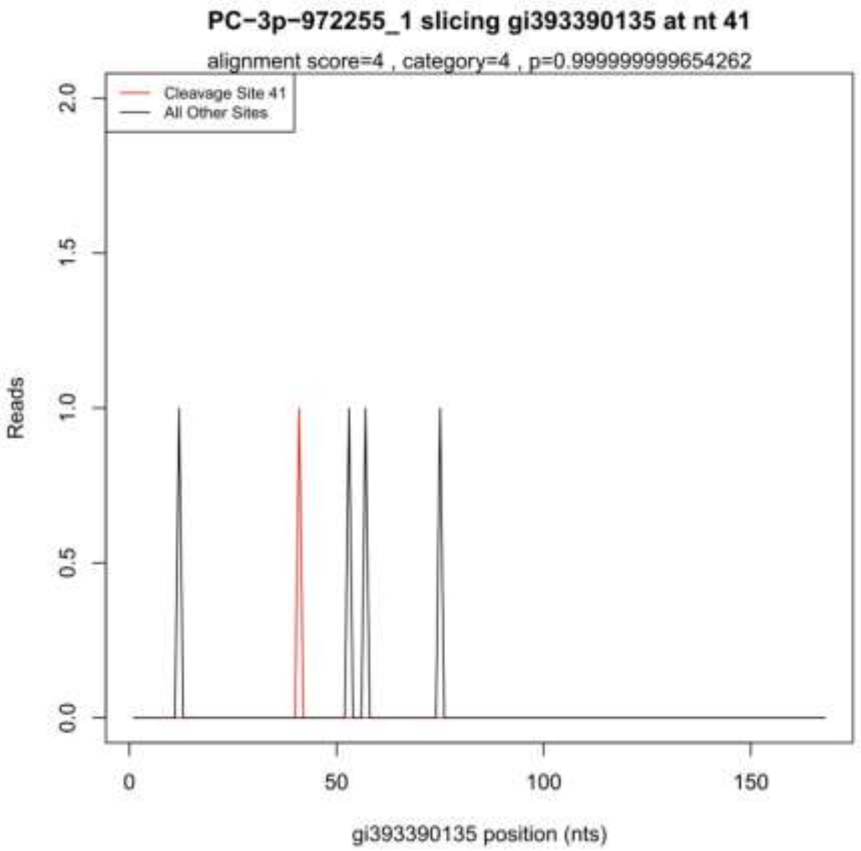
PC−3p−972255_1 slicing gi393390135 at nt 41
alignment score=4 , category=4 , p=0.999999999654262
Cleavage Site 41
All Other Sites
Reads
0.0
0.5
1.0
1.5
2.0
0
50
100
150
gi393390135 position (nts)

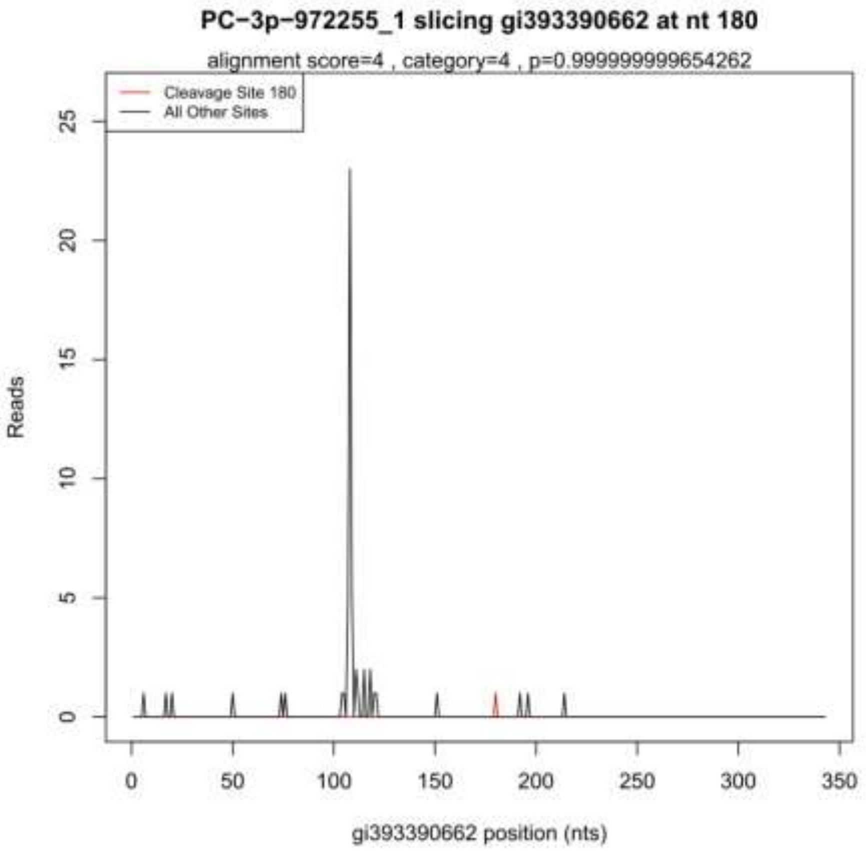
PC−3p−972255_1 slicing gi393390662 at nt 180
alignment score=4 , category=4 , p=0.999999999654262
Cleavage Site 180
All Other Sites
Reads
0
5
10
15
20
25
0
50
100
150
200
250
300
350
gi393390662 position (nts)

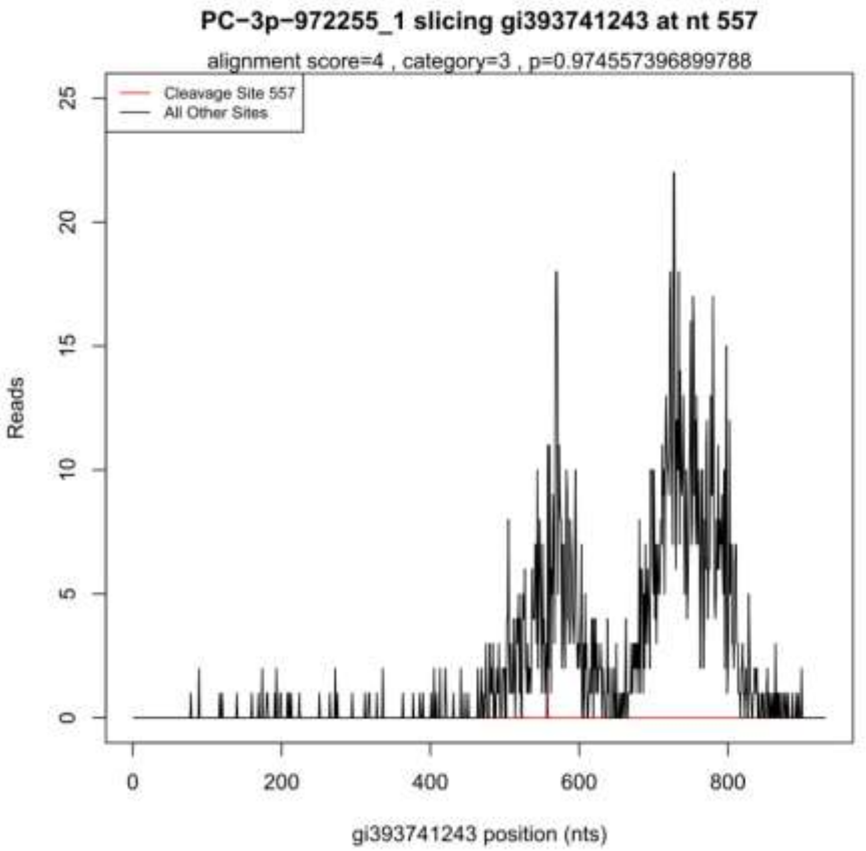
PC−3p−972255_1 slicing gi393741243 at nt 557
alignment score=4 , category=3 , p=0.974557396899788
Cleavage Site 557
All Other Sites
Reads
0
5
10
15
20
25
0
200
400
600
800
gi393741243 position (nts)

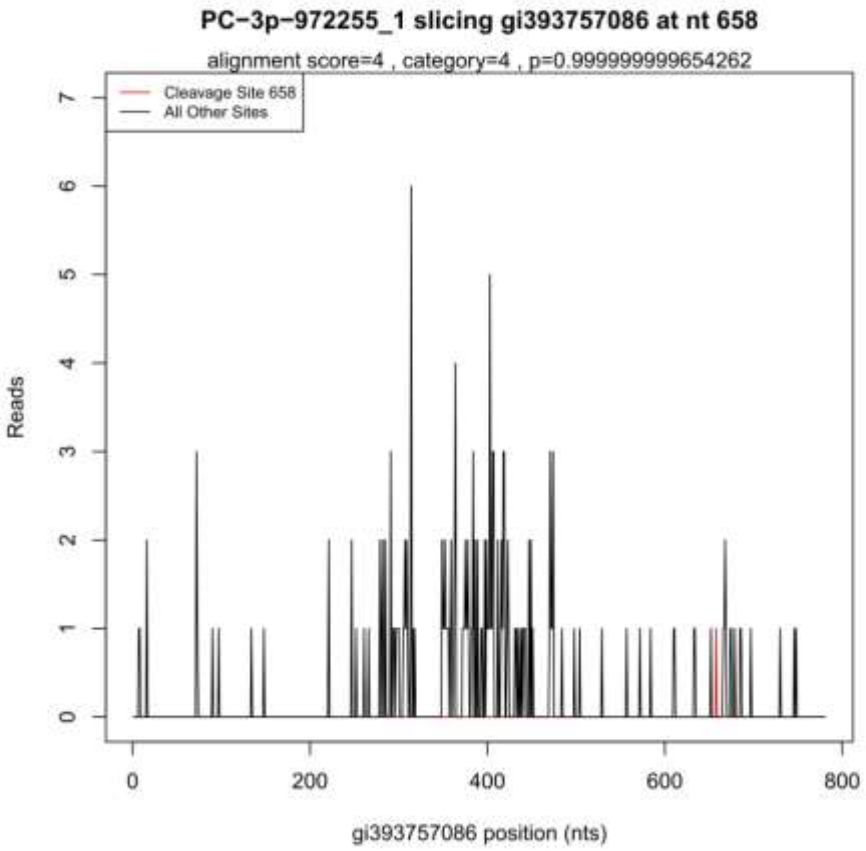

Figure S5 t-plot for targets of the new miRNA candidates found in +C and –C libraries of *camellia sinensis*. Signature abundance throughout the length of the transcript is show. Arrows indicate signature consistent with miRNA-directed cleavage.
