# Supplementary material for: Identification and characterization of cold-responsive microRNAs in tea plant (Camellia sinensis) and their targets using high-throughput sequencing and degradome analysis
Source: BMC Plant Biol. 2014 Oct 21;14:271. doi: 10.1186/s12870-014-0271-x (PMC4209041; doi:10.1186/s12870-014-0271-x)
Supplement: Additional file 10: Table S5. — Targets for conserved miRNAs from C. sinensis treated with cold (+C) and without cold (−C). [file 12870_2014_271_MOESM10_ESM.pdf]

## Table S5 Targets for conserved miRNAs from *C. sinensis* treated with cold (+C) and without cold (-C)

| miRNA family | Targe ID | Alignment Score | Alignment Range | Cleavage Site | -C Library | | +C Library | | Annotation |
|---|---|---|---|---|---|---|---|---|---|
| | | | | | Category | Reads | Category | Reads | |
| csn-miR156 | gi212380427 | 4 | 557-573 | 564 | 2 | 3 | | | Acyl-CoA N-acyltransferases (NAT) superfamily protein |
| | gi212380702 | 4 | 187-202 | 194 | 2 | 4 | | | unknown protein |
| | gi222372708 | 4 | 384-401 | 392 | 4 | 1 | | | unknown protein |
| | gi393393029 | 4 | 609-624 | 616 | | 4 | 1 | | unknown protein |
| | gi393741040 | 2 | 803-819 | 810 | 2 | 2 | | | unknown protein |
| | gi393741737 | 4 | 333-349 | 340 | 2 | 11 | 4 | 1 | unknown protein |
| | gi393744893 | 4 | 332-348 | 339 | 2 | 11 | 4 | 1 | unknown protein |
| | gi393747022 | 3 | 370-387 | 378 | | 4 | 1 | | Protein kinase superfamily protein |
| | gi393748378 | 4 | 507-524 | 515 | 4 | 1 | | | unknown protein |
| | gi393749243 | 4 | 532-548 | 539 | 2 | 3 | | | Acyl-CoA N-acyltransferases (NAT) superfamily protein |
| | gi393750016 | 4 | 844-859 | 851 | | 4 | 1 | | unknown protein |
| | gi393738656 | 4 | 62-81 | 72 | 2 | 3 | | | Cytochrome b-c1 complex, subunit 8 protein |
| | gi393740979 | 4 | 45-64 | 55 | 2 | 3 | | | Cytochrome b-c1 complex, subunit 8 protein |
| | gi393742300 | 4 | 23-42 | 33 | 2 | 3 | | | Cytochrome b-c1 complex, subunit 8 protein |
| | gi393745341 | 4 | 13-32 | 23 | 2 | 3 | | | Cytochrome b-c1 complex, subunit 8 protein |
| csn-miR160 | gi170319674 | 4 | 322-337 | 328 | | 4 | 1 | | Ribosomal protein L18e/L15 superfamily protein |
| | gi170319675 | 4 | 322-337 | 328 | | 4 | 1 | | Ribosomal protein L18e/L15 superfamily protein |
| | gi170319676 | 4 | 293-308 | 299 | | 4 | 1 | | Ribosomal protein L18e/L15 superfamily protein |
| | gi171355249 | 4 | 246-261 | 252 | 4 | 1 | | | unknown protein |
| | gi212379345 | 4 | 172-186 | 177 | 4 | 1 | | | RNA-binding (RRM/RBD/RNP motifs) family protein |
| | gi212379364 | 4 | 172-186 | 177 | 4 | 1 | | | RNA-binding (RRM/RBD/RNP motifs) family protein |
| | gi222372683 | 3.5 | 59-74 | 65 | 3 | 5 | 3 | 6 | Protein kinase superfamily protein |

| | | | | | | | | |
|---|---|---|---|---|---|---|---|---|
| gi259016492 | 4 | 89-104 | 95 | 4 | 1 | 2 | 2 | unknown protein |
| gi283580549 | 3.5 | 302-317 | 308 | 4 | 1 | 4 | 1 | Copper amine oxidase family protein |
| gi284026223 | 4 | 413-428 | 419 | | 4 | 1 | | unknown protein |
| gi327360761 | 3 | 163-178 | 169 | 2 | 8 | 3 | 2 | transposable element gene |
| gi347360201 | 4 | 194-208 | 199 | 4 | 1 | 4 | 1 | unknown protein |
| gi347360269 | 4 | 658-672 | 663 | 4 | 1 | 4 | 1 | Integrase-type DNA-binding superfamily protein |
| gi347360325 | 4 | 658-672 | 663 | 4 | 1 | 4 | 1 | Integrase-type DNA-binding superfamily protein |
| gi366883012 | 4 | 135-149 | 140 | 4 | 1 | | | RNA-binding (RRM/RBD/RNP motifs) family protein |
| gi366888436 | 4 | 157-171 | 162 | 4 | 1 | | | RNA-binding (RRM/RBD/RNP motifs) family protein |
| gi366889807 | 3.5 | 190-205 | 196 | 4 | 1 | 4 | 1 | Copper amine oxidase family protein |
| gi366889868 | 3.5 | 190-205 | 196 | 4 | 1 | 4 | 1 | Copper amine oxidase family protein |
| gi366889919 | 4 | 136-150 | 141 | 4 | 1 | | | VQ motif-containing protein |
| gi366896530 | 3.5 | 441-456 | 447 | 4 | 1 | 4 | 1 | Copper amine oxidase family protein |
| gi366896551 | 3.5 | 190-205 | 196 | 4 | 1 | | | Copper amine oxidase family protein |
| gi366896712 | 3.5 | 441-456 | 447 | | 4 | 1 | | Copper amine oxidase family protein |
| gi366896749 | 3.5 | 88-103 | 94 | | 4 | 1 | | Copper amine oxidase family protein |
| gi366896785 | 3.5 | 441-456 | 447 | | 4 | 1 | | Copper amine oxidase family protein |
| gi366896968 | 3.5 | 441-456 | 447 | 4 | 1 | 4 | 1 | Copper amine oxidase family protein |
| gi366897084 | 3.5 | 190-205 | 196 | 4 | 1 | | | Copper amine oxidase family protein |
| gi366897466 | 3.5 | 230-245 | 236 | 4 | 1 | 4 | 1 | Copper amine oxidase family protein |
| gi383479182 | 4 | 100-115 | 106 | | 4 | 1 | | unknown protein |
| gi393388375 | 4 | 79-94 | 85 | 4 | 1 | 2 | 2 | unknown protein |
| gi393388906 | 4 | 87-102 | 93 | 2 | 2 | | | unknown protein |
| gi393389011 | 3 | 197-212 | 203 | 4 | 1 | | | unknown protein |
| gi393390346 | 4 | 191-206 | 197 | 4 | 1 | 4 | 1 | unknown protein |
| gi393392131 | 4 | 132-146 | 137 | 4 | 1 | | | other RNA |

| | | | | | | | | |
|---|---|---|---|---|---|---|---|---|
| gi393392896 | 4 | 393-408 | 399 | | 2 | 2 | | Cysteine proteinases superfamily protein |
| gi393393263 | 4 | 360-375 | 366 | | 2 | 2 | | Cysteine proteinases superfamily protein |
| gi393740767 | 4 | 411-426 | 417 | | 4 | 1 | | unknown protein |
| gi393741649 | 4 | 67-81 | 72 | 4 | 1 | | | unknown protein |
| gi393742154 | 4 | 166-181 | 172 | 4 | 1 | | | unknown protein |
| gi393742160 | 4 | 352-367 | 358 | 2 | 12 | 2 | 3 | unknown protein |
| gi393742988 | 4 | 175-189 | 180 | 2 | 2 | | | alpha/beta-Hydrolases superfamily protein |
| gi393743382 | 4 | 406-421 | 412 | 4 | 1 | | | unknown protein |
| gi393743433 | 4 | 391-406 | 397 | 2 | 12 | 2 | 3 | unknown protein |
| gi393743777 | 4 | 152-168 | 159 | 4 | 1 | | | secE/sec61-gamma protein transport protein |
| gi393743810 | 4 | 169-183 | 174 | 4 | 1 | | | alpha/beta-Hydrolases superfamily protein |
| gi393743974 | 4 | 188-203 | 194 | 4 | 1 | 4 | 1 | unknown protein |
| gi393744249 | 4 | 117-132 | 123 | | 4 | 1 | | unknown protein |
| gi393744356 | 4 | 123-137 | 128 | 2 | 2 | | | unknown protein |
| gi393744764 | 4 | 292-307 | 298 | | 4 | 1 | | Ribosomal protein L18e/L15 superfamily protein |
| gi393745575 | 4 | 167-181 | 172 | 4 | 1 | | | unknown protein; |
| gi393746155 | 4 | 128-143 | 134 | | 4 | 1 | | unknown protein |
| gi393746234 | 3 | 643-659 | 650 | 4 | 1 | | | unknown protein |
| gi393746235 | 4 | 408-423 | 414 | | 4 | 1 | | unknown protein |
| gi393746386 | 4 | 398-413 | 404 | | 4 | 1 | | unknown protein |
| gi393746610 | 4 | 150-164 | 155 | 4 | 1 | | | RNA-binding (RRM/RBD/RNP motifs) family protein |
| gi393746961 | 4 | 157-171 | 162 | 4 | 1 | | | RNA-binding (RRM/RBD/RNP motifs) family protein |
| gi393747711 | 4 | 288-304 | 294 | 2 | 5 | 3 | 2 | transposable element gene |
| gi393748413 | 4 | 344-359 | 350 | | 3 | 2 | | Cysteine proteinases superfamily protein |
| gi393748570 | 3.5 | 128-143 | 134 | 4 | 1 | | | Copper amine oxidase family protein |
| gi393748864 | 4 | 94-109 | 100 | 4 | 1 | | | unknown protein |

| | | | | | | | | |
|---|---|---|---|---|---|---|---|---|
| gi393748894 | 4 | 397-412 | 403 | | 4 | 1 | | unknown protein |
| gi393749487 | 4 | 344-359 | 350 | | 3 | 2 | | Cysteine proteinases superfamily protein |
| gi393749631 | 4 | 383-398 | 389 | 2 | 12 | 2 | 3 | Microsomal signal peptidase 12 kDa subunit (SPC12) |
| gi393750300 | 4 | 394-409 | 400 | | 4 | 1 | | unknown protein |
| gi393750316 | 3.5 | 645-660 | 651 | 4 | 1 | 4 | 1 | Copper amine oxidase family protein |
| gi393750458 | 4 | 296-311 | 302 | | 4 | 1 | | Ribosomal protein L18e/L15 superfamily protein |
| gi393750477 | 4 | 295-310 | 301 | | 4 | 1 | | Ribosomal protein L18e/L15 superfamily protein |
| gi393750760 | 4 | 583-598 | 589 | | 4 | 1 | | unknown protein |
| gi393750813 | 4 | 150-165 | 156 | 4 | 1 | | | unknown protein |
| gi393751340 | 4 | 222-237 | 228 | | 4 | 1 | | unknown protein |
| gi393751801 | 4 | 401-416 | 407 | | 4 | 1 | | unknown protein |
| gi393751826 | 4 | 158-172 | 163 | 4 | 1 | | | alpha/beta-Hydrolases superfamily protein |
| gi393751982 | 4 | 151-165 | 156 | 2 | 2 | | | RNA-binding (RRM/RBD/RNP motifs) family protein |
| gi393752010 | 4 | 97-112 | 103 | 4 | 1 | 4 | 1 | unknown protein |
| gi393752416 | 4 | 398-413 | 404 | | 4 | 1 | | unknown protein |
| gi393752459 | 3 | 644-660 | 651 | 4 | 1 | | | unknown protein |
| gi393752659 | 4 | 43-58 | 49 | 4 | 1 | 4 | 1 | unknown protein |
| gi393752677 | 4 | 179-194 | 185 | | 2 | 3 | | unknown protein |
| gi393752992 | 4 | 391-406 | 397 | | 4 | 1 | | unknown protein |
| gi393754403 | 4 | 200-214 | 205 | 4 | 1 | 4 | 1 | unknown protein |
| gi393754523 | 4 | 397-412 | 403 | | 4 | 1 | | unknown protein |
| gi393754558 | 4 | 410-425 | 416 | | 4 | 1 | | unknown protein |
| gi51452941 | 4 | 147-161 | 152 | 3 | 2 | | | RNA-binding (RRM/RBD/RNP motifs) family protein |
| gi51453910 | 4 | 9-24 | 15 | | 4 | 1 | | unknown protein |
| gi51454085 | 4 | 70-85 | 76 | 4 | 1 | | | unknown protein |
| gi55281910 | 4 | 414-429 | 420 | | 4 | 1 | | unknown protein |

| | | | | | | | | | |
|---|---|---|---|---|---|---|---|---|---|
| csn-miR164 | gi393747400 | 4 | 206-226 | 217 | 4 | 1 | 4 | 1 | Late embryogenesis abundant protein (LEA) family protein |
| | gi51452831 | 4 | 151-171 | 162 | 4 | 1 | 4 | 1 | Late embryogenesis abundant protein (LEA) family protein |
| csn-miR167 | gi212379810 | 3 | 577-596 | 587 | 4 | 1 | | | unknown protein |
| | gi393741034 | 3 | 561-580 | 571 | 4 | 1 | | | unknown protein |
| | gi393741579 | 3 | 564-583 | 574 | 4 | 1 | | | unknown protein |
| | gi393748929 | 4 | 24-45 | 35 | 0 | 26 | 0 | 17 | unknown protein |
| csn-miR171 | gi170319630 | 3 | 601-615 | 606 | | 2 | 2 | | unknown protein |
| | gi171355277 | 4 | 51-65 | 56 | 4 | 1 | 4 | 1 | unknown protein |
| | gi212378117 | 4 | 180-195 | 185 | 2 | 5 | 2 | 7 | unknown protein |
| | gi212378239 | 4 | 305-320 | 310 | 4 | 1 | 4 | 1 | unknown protein |
| | gi212379608 | 3.5 | 386-399 | 391 | 4 | 1 | | | unknown protein |
| | gi212380260 | 4 | 148-163 | 153 | 2 | 8 | 2 | 11 | unknown protein |
| | gi366892790 | 4 | 158-172 | 163 | 4 | 1 | 4 | 1 | unknown protein |
| | gi393388960 | 4 | 265-280 | 270 | 4 | 1 | | | unknown protein |
| | gi393389180 | 4 | 444-459 | 449 | 2 | 8 | 2 | 11 | unknown protein |
| | gi393738945 | 3 | 642-656 | 647 | | 2 | 2 | | unknown protein |
| | gi393739105 | 4 | 769-784 | 775 | 4 | 1 | 2 | 2 | unknown protein |
| | gi393739739 | 4 | 601-616 | 606 | 2 | 8 | 2 | 11 | unknown protein |
| | gi393740498 | 4 | 47-61 | 52 | | 4 | 1 | | unknown protein |
| | gi393740523 | 4 | 100-114 | 105 | 4 | 1 | | | unknown protein |
| | gi393740541 | 4 | 630-645 | 635 | 2 | 3 | 2 | 4 | unknown protein |
| | gi393740835 | 4 | 103-117 | 108 | 4 | 1 | | | unknown protein |
| | gi393740881 | 4 | 105-119 | 110 | 4 | 1 | | | unknown protein |
| | gi393741019 | 4 | 106-120 | 111 | 4 | 1 | | | unknown protein |
| | gi393741112 | 4 | 102-116 | 107 | 4 | 1 | | | unknown protein |
| | gi393741349 | 4 | 640-655 | 645 | 2 | 3 | 2 | 4 | unknown protein |

| | | | | | | | | |
|---|---|---|---|---|---|---|---|---|
| gi393741488 | 4 | 83-97 | 88 | 4 | 1 | | | unknown protein |
| gi393741523 | 4 | 618-633 | 623 | | 4 | 1 | | unknown protein |
| gi393741639 | 4 | 49-63 | 54 | | 4 | 1 | | unknown protein |
| gi393741933 | 4 | 478-493 | 483 | 2 | 2 | 4 | 1 | Translation initiation factor IF2/IF5 |
| gi393744267 | 4 | 100-114 | 105 | | 4 | 1 | | unknown protein |
| gi393744696 | 4 | 631-646 | 636 | 2 | 3 | | | unknown protein |
| gi393744708 | 4 | 630-645 | 635 | | 2 | 2 | | unknown protein |
| gi393744796 | 4 | 41-55 | 46 | 4 | 1 | | | unknown protein |
| gi393744830 | 4 | 364-379 | 369 | 2 | 3 | 2 | 4 | unknown protein |
| gi393744926 | 4 | 102-116 | 107 | 4 | 1 | | | unknown protein |
| gi393745964 | 4 | 427-442 | 432 | | 4 | 1 | | Ras-related small GTP-binding family protein |
| gi393746120 | 4 | 102-116 | 107 | 4 | 1 | | | unknown protein |
| gi393746146 | 4 | 611-626 | 616 | 2 | 3 | 2 | 4 | unknown protein |
| gi393746189 | 4 | 103-117 | 108 | | 4 | 1 | | unknown protein |
| gi393746263 | 4 | 71-85 | 76 | 4 | 1 | | | unknown protein |
| gi393747498 | 4 | 485-500 | 490 | 4 | 1 | | | Peroxidase superfamily protein |
| gi393748616 | 4 | 49-63 | 54 | | 4 | 1 | | unknown protein |
| gi393748743 | 4 | 148-162 | 153 | 2 | 2 | 4 | 1 | unknown protein |
| gi393749138 | 3 | 578-592 | 583 | | 2 | 2 | | unknown protein |
| gi393750071 | 3 | 686-700 | 691 | | 4 | 1 | | unknown protein |
| gi393751104 | 4 | 306-320 | 311 | 4 | 1 | | | unknown protein |
| gi393751380 | 4 | 87-101 | 92 | 4 | 1 | | | unknown protein |
| gi393751528 | 4 | 93-107 | 98 | 4 | 1 | | | unknown protein |
| gi393751691 | 4 | 102-116 | 107 | 4 | 1 | | | unknown protein |
| gi393751780 | 4 | 307-321 | 312 | 4 | 1 | | | unknown protein |
| gi393751860 | 2.5 | 618-632 | 623 | 4 | 1 | | | SEC7-like guanine nucleotide exchange family protein |

| | | | | | | | | |
|---|---|---|---|---|---|---|---|---|
| gi393751934 | 3 | 582-596 | 587 | 4 | 1 | 2 | 3 | unknown protein |
| gi393752065 | 4 | 100-114 | 105 | | 4 | 1 | | unknown protein |
| gi393752214 | 4 | 165-179 | 170 | | 4 | 1 | | unknown protein |
| gi393752441 | 4 | 103-117 | 108 | | 4 | 1 | | unknown protein |
| gi393752884 | 4 | 636-651 | 641 | 2 | 3 | 2 | 4 | unknown protein |
| gi393753024 | 4 | 763-778 | 769 | 4 | 1 | 2 | 2 | unknown protein |
| gi393753517 | 4 | 87-101 | 92 | 4 | 1 | | | unknown protein |
| gi393753853 | 4 | 160-174 | 165 | 4 | 1 | 4 | 1 | unknown protein |
| gi393754087 | 4 | 70-84 | 75 | 4 | 1 | | | unknown protein |
| gi393754199 | 4 | 307-321 | 312 | 4 | 1 | | | unknown protein |
| gi393754278 | 4 | 101-115 | 106 | | 4 | 1 | | unknown protein |
| gi393754376 | 4 | 102-116 | 107 | 4 | 1 | | | unknown protein |
| gi393754527 | 4 | 106-120 | 111 | | 4 | 1 | | unknown protein |
| gi393756490 | 4 | 168-183 | 173 | | 4 | 1 | | unknown protein |
| gi393757018 | 4 | 57-71 | 62 | 4 | 1 | | | unknown protein |
| gi51453335 | 4 | 178-193 | 183 | 2 | 8 | 2 | 11 | unknown protein |
| gi51530180 | 4 | 407-421 | 412 | 3 | 3 | | | Chaperone DnaJ-domain superfamily protein |
| gi220966071 | 4 | 119-136 | 127 | 4 | 1 | 4 | 1 | Cyclophilin-like peptidyl-prolyl cis-trans isomerase family protein |
| gi220966090 | 4 | 119-136 | 127 | 4 | 1 | 4 | 1 | Cyclophilin-like peptidyl-prolyl cis-trans isomerase family protein |
| gi222457654 | 4 | 119-136 | 127 | 4 | 1 | 4 | 1 | Cyclophilin-like peptidyl-prolyl cis-trans isomerase family protein |
| gi282162067 | 4 | 61-78 | 69 | 2 | 4 | | | RNA-binding (RRM/RBD/RNP motifs) family protein |
| gi283049871 | 4 | 61-78 | 69 | 2 | 4 | | | RNA-binding (RRM/RBD/RNP motifs) family protein |
| gi366882798 | 3 | 449-467 | 458 | 4 | 1 | | | unknown protein |
| gi366883134 | 3 | 449-467 | 458 | 4 | 1 | | | unknown protein |
| gi366883854 | 3 | 449-467 | 458 | 4 | 1 | | | unknown protein |
| gi366889326 | 3 | 197-215 | 206 | 4 | 1 | | | unknown protein |

| | | | | | | | | | |
|---|---|---|---|---|---|---|---|---|---|
| | gi366894328 | 4 | 97-114 | 105 | 4 | 1 | 4 | 1 | Cyclophilin-like peptidyl-prolyl cis-trans isomerase family protein |
| | gi366895757 | 4 | 97-114 | 105 | 4 | 1 | 4 | 1 | Cyclophilin-like peptidyl-prolyl cis-trans isomerase family protein |
| | gi366895773 | 4 | 97-114 | 105 | 4 | 1 | 4 | 1 | Cyclophilin-like peptidyl-prolyl cis-trans isomerase family protein |
| | gi393739664 | 3 | 302-320 | 311 | 4 | 1 | | | unknown protein |
| | gi393740039 | 3 | 524-542 | 533 | 4 | 1 | | | unknown protein |
| csn-miR172 | gi167046990 | 4 | 24-46 | 37 | 4 | 1 | 2 | 2 | Ubiquitin supergroup;Ribosomal protein L40e |
| | gi170319957 | 4 | 376-399 | 390 | | 2 | 2 | | RING/U-box superfamily protein |
| | gi183186665 | 3.5 | 247-269 | 260 | 3 | 3 | 4 | 1 | Dormancy/auxin associated family protein |
| | gi212377709 | 3.5 | 30-52 | 43 | 4 | 1 | | | Cytochrome bd ubiquinol oxidase, 14kDa subunit |
| | gi212377797 | 3.5 | 445-467 | 458 | 3 | 2 | 4 | 1 | Dormancy/auxin associated family protein |
| | gi212378080 | 2.5 | 57-79 | 70 | 4 | 1 | | | Cytochrome bd ubiquinol oxidase, 14kDa subunit |
| | gi212378138 | 3.5 | 433-455 | 446 | 4 | 1 | | | Dormancy/auxin associated family protein |
| | gi212378218 | 3.5 | 440-462 | 453 | 3 | 2 | 4 | 1 | Dormancy/auxin associated family protein |
| | gi212378786 | 4 | 50-72 | 63 | | 4 | 1 | | transposable element gene |
| | gi212378841 | 3.5 | 269-291 | 282 | 3 | 3 | 4 | 1 | Dormancy/auxin associated family protein |
| | gi212379916 | 4 | 312-334 | 325 | 2 | 28 | 2 | 29 | alpha/beta-Hydrolases superfamily protein |
| | gi212380147 | 3.5 | 430-452 | 443 | 4 | 1 | | | Dormancy/auxin associated family protein |
| | gi212380629 | 3.5 | 446-468 | 459 | 3 | 2 | 4 | 1 | Dormancy/auxin associated family protein |
| | gi212380931 | 3.5 | 440-462 | 453 | 4 | 1 | | | Dormancy/auxin associated family protein |
| | gi215399041 | 4 | 545-568 | 558 | 4 | 1 | 4 | 1 | RING/U-box superfamily protein |
| | gi221071112 | 3 | 178-200 | 191 | | 4 | 1 | | proline-rich family protein |
| | gi343702829 | 3.5 | 100-122 | 113 | 4 | 1 | | | AT hook motif DNA-binding family protein |
| | gi366887458 | 4 | 65-87 | 78 | 3 | 2 | | | Ribosomal protein L31e family protein |
| | gi366889014 | 2.5 | 154-176 | 167 | 4 | 1 | | | unknown protein |
| | gi366889102 | 4 | 22-44 | 35 | 4 | 1 | 2 | 2 | Ubiquitin supergroup;Ribosomal protein L40e |
| | gi366889444 | 4 | 35-57 | 48 | | 4 | 1 | | Sec14p-like phosphatidylinositol transfer family protein |

| | | | | | | | | |
|---|---|---|---|---|---|---|---|---|
| gi366889587 | 3 | 368-390 | 381 | | 4 | 1 | | proline-rich family protein |
| gi366889967 | 4 | 88-111 | 101 | 2 | 2 | | | transposable element gene |
| gi366892849 | 3.5 | 416-438 | 429 | 4 | 1 | | | Dormancy/auxin associated family protein |
| gi366895173 | 3.5 | 114-136 | 127 | 4 | 1 | | | unknown protein |
| gi393390124 | 2.5 | 51-73 | 64 | 4 | 1 | | | Cytochrome bd ubiquinol oxidase, 14kDa subunit |
| gi393390358 | 2.5 | 34-56 | 47 | 4 | 1 | | | other RNA |
| gi393391865 | 3 | 32-54 | 45 | 2 | 2 | 4 | 1 | alpha/beta-Hydrolases superfamily protein |
| gi393392131 | 3 | 16-38 | 29 | 2 | 2 | 4 | 1 | other RNA |
| gi393392283 | 4 | 58-81 | 71 | 2 | 2 | 4 | 1 | RING/U-box superfamily protein |
| gi393738626 | 3.5 | 469-491 | 482 | 4 | 1 | | | Dormancy/auxin associated family protein |
| gi393738780 | 3.5 | 407-429 | 420 | 3 | 2 | 4 | 1 | Dormancy/auxin associated family protein |
| gi393742988 | 3 | 59-81 | 72 | 2 | 2 | 4 | 1 | alpha/beta-Hydrolases superfamily protein |
| gi393742991 | 3 | 27-49 | 40 | 4 | 1 | | | unknown protein |
| gi393743810 | 3 | 53-75 | 66 | 2 | 2 | 4 | 1 | alpha/beta-Hydrolases superfamily protein |
| gi393744727 | 2 | 44-66 | 57 | 2 | 2 | 2 | 2 | Uncharacterised protein family SERF |
| gi393745575 | 3 | 51-73 | 64 | 2 | 2 | 4 | 1 | unknown protein |
| gi393745863 | 4 | 313-336 | 326 | 4 | 1 | 4 | 1 | Leucine-rich repeat (LRR) family protein |
| gi393745960 | 4 | 565-588 | 578 | 3 | 3 | | | Leucine-rich repeat (LRR) family protein |
| gi393746082 | 4 | 558-581 | 571 | 3 | 3 | | | Leucine-rich repeat (LRR) family protein |
| gi393746140 | 4 | 502-525 | 515 | 4 | 1 | | | Leucine-rich repeat (LRR) family protein |
| gi393746198 | 4 | 321-344 | 334 | 4 | 1 | 4 | 1 | Leucine-rich repeat (LRR) family protein |
| gi393746372 | 4 | 558-581 | 571 | 3 | 2 | 4 | 1 | Leucine-rich repeat (LRR) family protein |
| gi393747849 | 2.5 | 15-37 | 28 | 4 | 1 | | | Cytochrome bd ubiquinol oxidase, 14kDa subunit |
| gi393748228 | 4 | 451-474 | 464 | 3 | 3 | | | Leucine-rich repeat (LRR) family protein |
| gi393748300 | 4 | 560-583 | 573 | 3 | 2 | | | Leucine-rich repeat (LRR) family protein |
| gi393748386 | 2.5 | 7-29 | 20 | 4 | 1 | | | Cytochrome bd ubiquinol oxidase, 14kDa subunit |

| | | | | | | | | |
|---|---|---|---|---|---|---|---|---|
| gi393748463 | 2.5 | 560-582 | 573 | 4 | 1 | | | unknown protein |
| gi393748504 | 4 | 530-553 | 543 | 4 | 1 | | | Leucine-rich repeat (LRR) family protein |
| gi393748593 | 4 | 561-584 | 574 | 4 | 1 | 4 | 1 | Leucine-rich repeat (LRR) family protein |
| gi393748639 | 4 | 559-582 | 572 | 4 | 1 | | | Leucine-rich repeat (LRR) family protein |
| gi393749113 | 4 | 117-140 | 130 | 2 | 3 | 4 | 1 | Leucine-rich repeat (LRR) family protein |
| gi393749154 | 4 | 555-578 | 568 | 3 | 2 | 4 | 1 | Leucine-rich repeat (LRR) family protein |
| gi393749197 | 4 | 208-231 | 221 | 4 | 1 | 4 | 1 | Leucine-rich repeat (LRR) family protein |
| gi393749344 | 4 | 555-578 | 568 | 4 | 1 | 4 | 1 | Leucine-rich repeat (LRR) family protein |
| gi393749383 | 4 | 557-580 | 570 | 3 | 4 | 4 | 1 | Leucine-rich repeat (LRR) family protein |
| gi393749523 | 4 | 558-581 | 571 | 3 | 3 | 4 | 1 | Leucine-rich repeat (LRR) family protein |
| gi393749747 | 4 | 557-580 | 570 | 4 | 1 | | | Leucine-rich repeat (LRR) family protein |
| gi393749751 | 4 | 305-328 | 318 | 4 | 1 | | | Leucine-rich repeat (LRR) family protein |
| gi393750006 | 4 | 105-128 | 118 | 3 | 2 | 4 | 1 | Leucine-rich repeat (LRR) family protein |
| gi393750252 | 4 | 562-585 | 575 | 4 | 1 | 4 | 1 | Leucine-rich repeat (LRR) family protein |
| gi393750797 | 2 | 42-64 | 55 | 2 | 2 | 2 | 2 | Uncharacterised protein family SERF |
| gi393750860 | 4 | 137-160 | 150 | 3 | 2 | 4 | 1 | Leucine-rich repeat (LRR) family protein |
| gi393750913 | 4 | 558-581 | 571 | 4 | 1 | 4 | 1 | Leucine-rich repeat (LRR) family protein |
| gi393751179 | 4 | 560-583 | 573 | 3 | 2 | 4 | 1 | Leucine-rich repeat (LRR) family protein |
| gi393751826 | 3 | 42-64 | 55 | 2 | 2 | 4 | 1 | alpha/beta-Hydrolases superfamily protein |
| gi393752228 | 4 | 557-580 | 570 | 3 | 3 | | | Leucine-rich repeat (LRR) family protein |
| gi393752332 | 4 | 336-359 | 349 | 3 | 2 | 4 | 1 | Leucine-rich repeat (LRR) family protein |
| gi393752718 | 4 | 570-593 | 583 | 3 | 2 | 4 | 1 | Leucine-rich repeat (LRR) family protein |
| gi393753194 | 4 | 558-581 | 571 | 4 | 1 | | | Leucine-rich repeat (LRR) family protein |
| gi393753261 | 4 | 562-585 | 575 | 3 | 3 | 4 | 1 | Leucine-rich repeat (LRR) family protein |
| gi393753606 | 4 | 500-522 | 513 | | 2 | 2 | | Tetratricopeptide repeat (TPR)-like superfamily protein |
| gi393754011 | 4 | 555-578 | 568 | 3 | 3 | 4 | 1 | Leucine-rich repeat (LRR) family protein |

| | | | | | | | | | |
|---|---|---|---|---|---|---|---|---|---|
| | gi393754187 | 4 | 452-475 | 465 | 3 | 3 | 4 | 1 | Leucine-rich repeat (LRR) family protein |
| | gi393754631 | 4 | 13-35 | 26 | 4 | 1 | 2 | 2 | Ubiquitin supergroup;Ribosomal protein L40e |
| | gi393757162 | 3.5 | 394-416 | 407 | 3 | 2 | 4 | 1 | Dormancy/auxin associated family protein |
| | gi51453048 | 4 | 103-126 | 116 | 3 | 3 | 4 | 1 | RING/U-box superfamily protein |
| | gi51454080 | 4 | 23-45 | 36 | 4 | 1 | 2 | 2 | Ubiquitin supergroup;Ribosomal protein L40e |
| | gi221758807 | 4 | 93-113 | 104 | | 2 | 2 | | unknown protein |
| csn-miR319 | gi170319482 | 4 | 119-133 | 124 | 4 | 1 | | | unknown protein |
| | gi170319483 | 4 | 119-133 | 124 | 4 | 1 | | | unknown protein |
| | gi170319517 | 4 | 190-203 | 195 | 4 | 1 | | | Histone superfamily protein |
| | gi170319622 | 4 | 167-181 | 172 | 4 | 1 | 2 | 3 | unknown protein |
| | gi170319718 | 4 | 176-190 | 181 | 2 | 2 | | | unknown protein |
| | gi170319719 | 4 | 176-190 | 181 | 2 | 2 | | | unknown protein |
| | gi170319720 | 4 | 32-46 | 37 | 2 | 2 | | | unknown protein |
| | gi170319865 | 4 | 158-172 | 163 | 4 | 1 | 2 | 2 | unknown protein |
| | gi170319866 | 4 | 157-171 | 162 | 4 | 1 | 2 | 2 | unknown protein |
| | gi170319867 | 4 | 154-168 | 159 | 4 | 1 | 2 | 3 | unknown protein |
| | gi170319868 | 4 | 154-168 | 159 | 4 | 1 | 4 | 1 | unknown protein |
| | gi170319869 | 4 | 154-168 | 159 | | 2 | 2 | | unknown protein |
| | gi170319870 | 4 | 154-168 | 159 | 4 | 1 | 4 | 1 | unknown protein |
| | gi170319872 | 4 | 28-42 | 33 | | 2 | 3 | | unknown protein |
| | gi171355231 | 4 | 140-154 | 145 | 4 | 1 | | | unknown protein |
| | gi206583671 | 4 | 120-134 | 125 | 4 | 1 | 4 | 1 | unknown protein |
| | gi212377956 | 4 | 190-203 | 195 | 4 | 1 | | | Histone superfamily protein |
| | gi212378294 | 4 | 176-190 | 181 | 2 | 2 | | | unknown protein |
| | gi212378772 | 4 | 70-84 | 75 | | 4 | 1 | | unknown protein |
| | gi212379345 | 4 | 539-553 | 544 | 4 | 1 | 4 | 1 | RNA-binding (RRM/RBD/RNP motifs) family protein |

| | | | | | | | | |
|---|---|---|---|---|---|---|---|---|
| gi212379364 | 4 | 539-553 | 544 | 4 | 1 | 4 | 1 | RNA-binding (RRM/RBD/RNP motifs) family protein |
| gi212379569 | 3.5 | 223-237 | 228 | 3 | 2 | 4 | 1 | unknown protein |
| gi212380194 | 4 | 119-133 | 124 | 4 | 1 | | | unknown protein |
| gi212380235 | 4 | 154-168 | 159 | 4 | 1 | 4 | 1 | unknown protein |
| gi212380670 | 4 | 176-190 | 181 | 2 | 2 | | | unknown protein |
| gi220966150 | 3 | 247-261 | 252 | 4 | 1 | | | O-fucosyltransferase family protein |
| gi220966158 | 3 | 247-261 | 252 | 4 | 1 | | | O-fucosyltransferase family protein |
| gi220966325 | 3 | 247-261 | 252 | 4 | 1 | | | unknown protein |
| gi221070896 | 4 | 249-263 | 254 | 2 | 2 | | | Protein kinase superfamily protein |
| gi221071597 | 4 | 265-279 | 270 | | 2 | 3 | | unknown protein |
| gi221758596 | 3.5 | 63-77 | 68 | 4 | 1 | | | unknown protein |
| gi221758896 | 4 | 97-111 | 102 | | 2 | 2 | | unknown protein |
| gi222457788 | 3 | 333-347 | 338 | 4 | 1 | | | O-fucosyltransferase family protein |
| gi222457803 | 3 | 331-345 | 336 | 4 | 1 | | | Protein kinase superfamily protein |
| gi224708655 | 4 | 134-148 | 139 | | 4 | 1 | | unknown protein |
| gi283049723 | 4 | 328-342 | 333 | 3 | 4 | 3 | 4 | transposable element gene |
| gi295345419 | 4 | 299-313 | 304 | | 3 | 2 | | transposable element gene |
| gi295345438 | 2.5 | 105-119 | 110 | | 4 | 1 | | Protein prenylyltransferase superfamily protein |
| gi313605332 | 4 | 200-214 | 205 | | 2 | 3 | | unknown protein |
| gi327361093 | 4 | 335-349 | 340 | 2 | 4 | 2 | 3 | transposable element gene |
| gi340628976 | 4 | 139-153 | 144 | 2 | 2 | | | unknown protein |
| gi366883103 | 4 | 139-153 | 144 | | 0 | 2 | | unknown protein |
| gi366887357 | 4 | 202-216 | 207 | | 2 | 3 | | unknown protein |
| gi366888131 | 4 | 45-58 | 50 | 4 | 1 | | | NmrA-like negative transcriptional regulator family protein |
| gi366889455 | 4 | 202-216 | 207 | | 4 | 1 | | unknown protein |
| gi366892566 | 4 | 202-216 | 207 | | 4 | 1 | | unknown protein |

| | | | | | | | | |
|---|---|---|---|---|---|---|---|---|
| gi366893559 | 4 | 202-216 | 207 | | 2 | 2 | | unknown protein |
| gi366893651 | 4 | 274-288 | 279 | 4 | 1 | 4 | 1 | transposable element gene |
| gi366894358 | 3 | 216-230 | 221 | 4 | 1 | | | unknown protein |
| gi366895310 | 4 | 303-317 | 308 | 2 | 2 | | | unknown protein |
| gi366895384 | 3 | 225-239 | 230 | 4 | 1 | | | unknown protein |
| gi366895413 | 3 | 225-239 | 230 | 4 | 1 | | | unknown protein |
| gi366896149 | 3 | 225-239 | 230 | 4 | 1 | | | unknown protein |
| gi366896160 | 3 | 225-239 | 230 | 4 | 1 | | | unknown protein |
| gi390125031 | 4 | 119-134 | 125 | | 4 | 1 | | unknown protein |
| gi390125056 | 4 | 171-186 | 177 | | 4 | 1 | | unknown protein |
| gi393389193 | 4 | 137-151 | 142 | 4 | 1 | 2 | 2 | unknown protein |
| gi393389392 | 4 | 262-277 | 268 | | 4 | 1 | | unknown protein |
| gi393390074 | 4 | 185-199 | 190 | 4 | 1 | 4 | 1 | unknown protein |
| gi393390706 | 4 | 146-160 | 151 | | 4 | 1 | | Saposin-like aspartyl protease family protein |
| gi393391973 | 4 | 137-151 | 142 | 4 | 1 | 4 | 1 | unknown protein |
| gi393391984 | 4 | 137-151 | 142 | | 0 | 3 | | unknown protein |
| gi393392125 | 4 | 262-277 | 268 | | 4 | 1 | | unknown protein |
| gi393739066 | 4 | 190-204 | 195 | | 2 | 3 | | unknown protein |
| gi393740312 | 4 | 281-295 | 286 | 4 | 1 | 4 | 1 | unknown protein |
| gi393740443 | 4 | 169-184 | 175 | | 4 | 1 | | unknown protein |
| gi393740640 | 4 | 260-275 | 266 | | 4 | 1 | | unknown protein |
| gi393740799 | 4 | 265-280 | 271 | | 4 | 1 | | unknown protein |
| gi393740904 | 4 | 263-278 | 269 | | 4 | 1 | | unknown protein |
| gi393740956 | 4 | 64-78 | 69 | | 2 | 2 | | Cytochrome B6, pseudogene |
| gi393740957 | 4 | 151-165 | 156 | 4 | 1 | | | unknown protein |
| gi393741154 | 4 | 182-196 | 187 | 2 | 2 | 4 | 1 | unknown protein |

| | | | | | | | | |
|---|---|---|---|---|---|---|---|---|
| gi393741371 | 4 | 265-280 | 271 | | 4 | 1 | | unknown protein |
| gi393741384 | 4 | 58-72 | 63 | | 2 | 2 | | Cytochrome B6, pseudogene |
| gi393741565 | 4 | 63-77 | 68 | | 2 | 2 | | Cytochrome B6, pseudogene |
| gi393741649 | 4 | 434-448 | 439 | | 2 | 3 | | unknown protein |
| gi393742138 | 4 | 413-427 | 418 | 4 | 1 | | | Ribosomal protein L18e/L15 superfamily protein |
| gi393742745 | 4 | 175-188 | 180 | 4 | 1 | | | Histone superfamily protein |
| gi393742878 | 4 | 446-460 | 451 | 2 | 9 | 2 | 2 | unknown protein |
| gi393743089 | 4 | 122-136 | 127 | 4 | 1 | 2 | 3 | unknown protein |
| gi393744286 | 4 | 266-281 | 272 | | 4 | 1 | | unknown protein |
| gi393744306 | 4 | 263-278 | 269 | | 4 | 1 | | metalloendopeptidases;zinc ion binding |
| gi393744356 | 4 | 490-504 | 495 | 4 | 1 | | | unknown protein |
| gi393744474 | 4 | 250-265 | 256 | | 4 | 1 | | unknown protein |
| gi393744794 | 4 | 120-135 | 126 | | 4 | 1 | | unknown protein |
| gi393744908 | 4 | 264-279 | 270 | | 4 | 1 | | unknown protein |
| gi393744934 | 4 | 263-278 | 269 | | 4 | 1 | | unknown protein |
| gi393745188 | 4 | 446-460 | 451 | 2 | 12 | 2 | 2 | unknown protein |
| gi393745218 | 4 | 446-460 | 451 | 2 | 12 | 2 | 2 | unknown protein |
| gi393746222 | 4 | 327-341 | 332 | | 4 | 1 | | unknown protein |
| gi393746610 | 4 | 517-531 | 522 | 4 | 1 | 4 | 1 | RNA-binding (RRM/RBD/RNP motifs) family protein |
| gi393746893 | 4 | 171-185 | 176 | 4 | 1 | | | unknown protein |
| gi393746961 | 4 | 524-538 | 529 | 4 | 1 | 4 | 1 | RNA-binding (RRM/RBD/RNP motifs) family protein |
| gi393747175 | 4 | 155-168 | 160 | 4 | 1 | | | Histone superfamily protein |
| gi393747955 | 4 | 160-174 | 165 | 2 | 4 | 4 | 1 | unknown protein |
| gi393748463 | 3.5 | 244-258 | 249 | 2 | 4 | | | unknown protein |
| gi393748633 | 4 | 66-80 | 71 | | 2 | 2 | | Cytochrome B6, pseudogene, similar to Cytochrome B6 |
| gi393748881 | 3.5 | 470-484 | 475 | 2 | 3 | | | zinc finger (C2H2 type) family protein |

| | | | | | | | | |
|---|---|---|---|---|---|---|---|---|
| gi393749528 | 4 | 233-248 | 239 | | 4 | 1 | | unknown protein |
| gi393749925 | 3 | 341-355 | 346 | | 4 | 1 | | Transmembrane amino acid transporter family protein |
| gi393750125 | 4 | 151-165 | 156 | 2 | 2 | | | unknown protein |
| gi393750187 | 4 | 128-142 | 133 | 4 | 1 | 4 | 1 | unknown protein |
| gi393750762 | 4 | 257-272 | 263 | | 4 | 1 | | unknown protein |
| gi393751060 | 4 | 97-111 | 102 | 4 | 1 | | | unknown protein |
| gi393751217 | 4 | 171-184 | 176 | 4 | 1 | | | Histone superfamily protein |
| gi393751500 | 4 | 154-169 | 160 | | 4 | 1 | | unknown protein |
| gi393751826 | 4 | 525-539 | 530 | | 2 | 3 | | alpha/beta-Hydrolases superfamily protein |
| gi393751982 | 4 | 518-532 | 523 | 4 | 1 | 4 | 1 | RNA-binding (RRM/RBD/RNP motifs) family protein |
| gi393752334 | 4 | 145-159 | 150 | 4 | 1 | 2 | 3 | transposable element gene |
| gi393752687 | 4 | 146-160 | 151 | 4 | 1 | 2 | 3 | unknown protein |
| gi393753182 | 3.5 | 388-402 | 393 | 2 | 3 | | | unknown protein |
| gi393754106 | 4 | 53-66 | 58 | 4 | 1 | | | NmrA-like negative transcriptional regulator family protein |
| gi393755901 | 4 | 63-76 | 68 | 4 | 1 | | | NmrA-like negative transcriptional regulator family protein |
| gi393756182 | 4 | 86-101 | 92 | | 4 | 1 | | unknown protein |
| gi393756205 | 4 | 243-258 | 249 | | 4 | 1 | | unknown protein |
| gi393756254 | 4 | 439-453 | 444 | 2 | 9 | 2 | 2 | unknown protein |
| gi393757025 | 3.5 | 809-823 | 814 | 2 | 4 | 2 | 3 | unknown protein |
| gi51452848 | 4 | 254-268 | 259 | 4 | 1 | | | unknown protein |
| gi51452849 | 4 | 135-149 | 140 | | 0 | 3 | | unknown protein |
| gi51452941 | 4 | 514-528 | 519 | 4 | 1 | 4 | 1 | RNA-binding (RRM/RBD/RNP motifs) family protein |
| gi51453491 | 4 | 139-153 | 144 | | 2 | 2 | | unknown protein |
| gi51453761 | 4 | 159-173 | 164 | 2 | 2 | | | unknown protein |
| gi51453870 | 4 | 569-583 | 574 | 3 | 3 | 3 | 4 | transposable element gene |
| gi51530166 | 4 | 538-551 | 542 | 4 | 1 | | | Protein prenylyltransferase superfamily protein |

| | | | | | | | | | |
|---|---|---|---|---|---|---|---|---|---|
| | gi51454104 | 3.5 | 20-39 | 30 | 0 | 8 | 0 | 7 | unknown protein |
| | gi51454104 | 2.5 | 21-39 | 30 | 0 | 8 | 0 | 7 | unknown protein |
| csn-miR390 | gi366887611 | 4 | 26-45 | 36 | 1 | 2 | | | unknown protein |
| csn-miR393 | gi366889364 | 2 | 382-402 | 393 | 0 | 139 | 0 | 53 | unknown protein |
| | gi366889365 | 2 | 382-402 | 393 | 0 | 139 | 0 | 53 | unknown protein |
| | gi366889396 | 2 | 382-402 | 393 | 0 | 139 | 0 | 53 | unknown protein |
| csn-miR395 | gi343702799 | 3 | 100-114 | 105 | 2 | 2 | | | unknown protein |
| | gi366893760 | 4 | 2-15 | 7 | | 4 | 1 | | transposable element gene |
| | gi393392094 | 3.5 | 71-84 | 76 | 4 | 1 | | | unknown protein |
| | gi393740799 | 4 | 52-66 | 57 | 4 | 1 | 2 | 2 | unknown protein |
| | gi393741730 | 4 | 54-68 | 59 | 4 | 1 | 2 | 2 | unknown protein |
| | gi393741883 | 4 | 319-332 | 324 | 2 | 2 | | | rRNA |
| | gi393744700 | 3 | 75-89 | 80 | 2 | 2 | | | unknown protein |
| | gi393745753 | 4 | 86-100 | 91 | 4 | 1 | | | unknown protein |
| | gi393746028 | 3.5 | 81-95 | 86 | | 4 | 1 | | unknown protein |
| | gi212378452 | 3.5 | 655-674 | 665 | | 2 | 2 | | ATPase, F0/V0 complex, subunit C protein |
| | gi393739681 | 3.5 | 496-515 | 506 | | 2 | 2 | | ATPase, F0/V0 complex, subunit C protein |
| csn-miR396 | gi259016488 | 4 | 92-111 | 103 | 3 | 2 | 4 | 1 | unknown protein |
| | gi366892248 | 4 | 21-40 | 32 | 3 | 2 | 4 | 1 | unknown protein |
| | gi393741998 | 4 | 236-255 | 247 | 3 | 2 | 4 | 1 | Plant calmodulin-binding protein-related |
| | gi393748313 | 4 | 229-248 | 240 | 3 | 2 | 4 | 1 | unknown protein |
| | gi393752237 | 4 | 231-250 | 242 | 3 | 2 | 4 | 1 | unknown protein |
| | gi393753170 | 4 | 220-239 | 231 | 3 | 2 | 4 | 1 | unknown protein |
| | gi393756422 | 4 | 220-239 | 231 | 3 | 2 | 4 | 1 | transposable element gene |
| | gi51453520 | 4 | 234-253 | 245 | 3 | 2 | 4 | 1 | unknown protein |
| | gi212381014 | 4 | 485-505 | 496 | 2 | 5 | 2 | 4 | unknown protein |

| | | | | | | | | |
|---|---|---|---|---|---|---|---|---|
| gi393390016 | 4 | 238-258 | 249 | 2 | 5 | 2 | 4 | unknown protein |
| gi170319934 | 4 | 282-296 | 287 | 2 | 2 | | | Ribosomal protein L32e |
| gi171355257 | 4 | 115-128 | 120 | 4 | 1 | | | unknown protein |
| gi212377668 | 4 | 582-596 | 587 | | 4 | 1 | | unknown protein |
| gi212377722 | 4 | 581-595 | 586 | | 4 | 1 | | unknown protein |
| gi212378214 | 4 | 582-596 | 587 | | 4 | 1 | | unknown protein |
| gi212378592 | 4 | 547-561 | 552 | 4 | 1 | | | pseudogene, hypothetical protein |
| gi212379353 | 4 | 582-596 | 587 | | 4 | 1 | | unknown protein |
| gi212379476 | 4 | 547-561 | 552 | 4 | 1 | | | pseudogene, hypothetical protein |
| gi212380839 | 4 | 583-597 | 588 | | 4 | 1 | | unknown protein |
| gi212380938 | 4 | 582-596 | 587 | | 4 | 1 | | unknown protein |
| gi212381047 | 4 | 582-596 | 587 | | 4 | 1 | | unknown protein |
| gi220966035 | 3 | 33-47 | 38 | 2 | 2 | 2 | 5 | unknown protein |
| gi220966336 | 3 | 33-47 | 38 | 2 | 2 | 2 | 5 | unknown protein |
| gi220966392 | 3 | 33-47 | 38 | 2 | 2 | 2 | 5 | unknown protein |
| gi221071321 | 4 | 112-126 | 117 | 4 | 1 | | | Protein kinase superfamily protein |
| gi222457813 | 3 | 116-130 | 121 | 2 | 2 | 2 | 5 | unknown protein |
| gi283049686 | 3 | 9-23 | 14 | 2 | 2 | 2 | 5 | unknown protein |
| gi284026217 | 3.5 | 163-177 | 168 | | 4 | 1 | | Unknown gene |
| gi295345443 | 3.5 | 193-207 | 198 | | 4 | 1 | | Protein prenylyltransferase superfamily protein |
| gi313605023 | 4 | 56-70 | 61 | 4 | 1 | | | Protein kinase superfamily protein |
| gi343702789 | 4 | 125-139 | 130 | 4 | 1 | | | unknown protein |
| gi343702828 | 2 | 55-69 | 60 | | 4 | 1 | | unknown protein |
| gi366886192 | 4 | 231-245 | 236 | | 4 | 1 | | unknown protein |
| gi366887690 | 4 | 231-245 | 236 | | 4 | 1 | | unknown protein |
| gi366887701 | 4 | 231-245 | 236 | | 4 | 1 | | unknown protein |

| | | | | | | | | |
|---|---|---|---|---|---|---|---|---|
| gi366888481 | 3.5 | 141-155 | 146 | | 4 | 1 | | unknown protein |
| gi366888652 | 3.5 | 272-286 | 277 | | 4 | 1 | | unknown protein |
| gi366888654 | 3.5 | 140-154 | 145 | | 4 | 1 | | unknown protein |
| gi366888841 | 3.5 | 271-285 | 276 | | 4 | 1 | | unknown protein |
| gi366895395 | 3 | 11-25 | 16 | 2 | 2 | 2 | 5 | unknown protein |
| gi366895529 | 3 | 11-25 | 16 | 2 | 2 | 2 | 5 | unknown protein |
| gi366895727 | 3 | 11-25 | 16 | 2 | 2 | 2 | 5 | unknown protein |
| gi366896170 | 3 | 11-25 | 16 | 2 | 2 | 2 | 5 | unknown protein |
| gi393389198 | 3 | 256-270 | 261 | 4 | 1 | | | unknown protein |
| gi393390983 | 3.5 | 158-172 | 163 | 2 | 2 | 2 | 4 | unknown protein |
| gi393392488 | 4 | 208-222 | 213 | 4 | 1 | | | unknown protein |
| gi393393739 | 4 | 202-217 | 207 | 4 | 1 | | | unknown protein |
| gi393738856 | 4 | 396-410 | 401 | | 4 | 1 | | AAA-type ATPase family protein |
| gi393740518 | 3 | 276-290 | 281 | 2 | 2 | 2 | 5 | unknown protein |
| gi393741151 | 4 | 243-257 | 248 | | 4 | 1 | | unknown protein |
| gi393741904 | 3.5 | 804-818 | 809 | 2 | 5 | | | O-methyltransferase family protein |
| gi393744303 | 4 | 539-553 | 544 | | 4 | 1 | | unknown protein |
| gi393747355 | 4 | 105-118 | 110 | 4 | 1 | | | transposable element gene |
| gi393748545 | 3.5 | 659-673 | 664 | | 4 | 1 | | unknown protein |
| gi393749183 | 3.5 | 824-838 | 829 | | 4 | 1 | | unknown protein |
| gi393749433 | 3.5 | 542-556 | 547 | | 4 | 1 | | unknown protein |
| gi393749564 | 4 | 514-528 | 519 | | 4 | 1 | | unknown protein |
| gi393749877 | 4 | 114-129 | 119 | 4 | 1 | | | unknown protein |
| gi393750864 | 4 | 557-571 | 562 | | 4 | 1 | | unknown protein |
| gi393751111 | 4 | 192-205 | 197 | 4 | 1 | 4 | 1 | Ras-related small GTP-binding family protein |
| gi393751629 | 2 | 21-35 | 26 | | 4 | 1 | | transposable element gene |

| | | | | | | | | | |
|---|---|---|---|---|---|---|---|---|---|
| | gi212381014 | 4 | 486-505 | 496 | 2 | 5 | 2 | 4 | unknown protein |
| | gi393390016 | 4 | 239-258 | 249 | 2 | 5 | 2 | 4 | unknown protein |
| | gi212381014 | 3 | 486-505 | 496 | 2 | 5 | 2 | 4 | unknown protein |
| | gi393390016 | 3 | 239-258 | 249 | 2 | 5 | 2 | 4 | unknown protein |
| | gi393746173 | 4 | 182-203 | 193 | 2 | 7 | 4 | 1 | unknown protein |
| | gi393746583 | 4 | 418-437 | 428 | 2 | 5 | 0 | 4 | unknown protein |
| | gi212381014 | 4 | 484-504 | 495 | | 4 | 1 | | unknown protein |
| | gi393390016 | 4 | 237-257 | 248 | | 4 | 1 | | unknown protein |
| csn-miR398 | gi393389492 | 4 | 88-110 | 101 | 2 | 3 | | | Rubredoxin-like superfamily protein |
| | gi393748835 | 4 | 87-109 | 100 | 2 | 3 | | | Rubredoxin-like superfamily protein |
| | gi393389492 | 4 | 89-110 | 101 | 2 | 3 | | | Rubredoxin-like superfamily protein |
| | gi393748835 | 4 | 88-109 | 100 | 2 | 3 | | | Rubredoxin-like superfamily protein |
| csn-miR408 | gi206583693 | 2.5 | 115-136 | 127 | 0 | 84 | 2 | 4 | transposable element gene |
| | gi212377727 | 3.5 | 540-561 | 552 | 4 | 1 | | | unknown protein |
| | gi212378124 | 3.5 | 638-659 | 650 | 4 | 1 | | | unknown protein |
| | gi224708643 | 2.5 | 115-136 | 127 | 0 | 91 | 2 | 4 | unknown protein |
| | gi224708735 | 3 | 115-136 | 127 | 0 | 87 | 2 | 4 | unknown protein |
| | gi393740307 | 3.5 | 401-422 | 413 | 4 | 1 | | | unknown protein |
| | gi393744371 | 3.5 | 631-652 | 643 | 4 | 1 | | | unknown protein |
| | gi393749899 | 2.5 | 111-132 | 123 | 0 | 87 | 2 | 5 | VQ motif-containing protein |
| | gi393750043 | 2.5 | 119-140 | 131 | 0 | 82 | 2 | 3 | AGC kinase family protein |
| | gi393751157 | 2 | 113-134 | 125 | 0 | 83 | 0 | 4 | unknown protein |
| | gi393751598 | 2.5 | 112-133 | 124 | 0 | 95 | 2 | 4 | VQ motif-containing protein |
| | gi393751677 | 2.5 | 109-130 | 121 | 0 | 82 | 2 | 2 | AGC kinase family protein |
| | gi393751996 | 2.5 | 111-132 | 123 | 0 | 108 | 0 | 5 | unknown protein |
| | gi393752580 | 2.5 | 113-134 | 125 | 0 | 90 | 2 | 4 | AGC kinase family protein |

| | | | | | | | | |
|---|---|---|---|---|---|---|---|---|
| gi393752762 | 2.5 | 111-132 | 123 | 0 | 89 | 2 | 4 | AGC kinase family protein |
| gi393752798 | 2.5 | 120-141 | 132 | 0 | 82 | 2 | 3 | VQ motif-containing protein |
| gi393752927 | 2.5 | 110-131 | 122 | 0 | 90 | 2 | 4 | AGC kinase family protein |
| gi393753536 | 3.5 | 270-291 | 282 | 4 | 1 | | | unknown protein |
| gi393757166 | 3.5 | 133-154 | 145 | 4 | 1 | | | unknown protein |
| gi224708755 | 3.5 | 208-225 | 216 | 3 | 2 | 2 | 6 | unknown protein |
| gi366891697 | 4 | 106-122 | 113 | 1 | 2 | | | unknown protein |
| gi366893443 | 3 | 338-354 | 345 | | 4 | 1 | | unknown protein |
| gi366896582 | 3.5 | 199-216 | 207 | 3 | 2 | 2 | 6 | unknown protein |
| gi393389199 | 4 | 313-329 | 320 | 2 | 3 | | | Ribosomal L29 family protein |
| gi393740035 | 4 | 211-227 | 218 | 4 | 1 | 4 | 1 | Ribosomal protein L6 family protein |
| gi393742479 | 4 | 16-33 | 24 | 3 | 2 | | | unknown protein |
| gi393743422 | 4 | 136-152 | 143 | | 4 | 1 | | WW-domain-binding protein |
| gi393743692 | 4 | 163-179 | 170 | | 4 | 1 | | WW-domain-binding protein |
| gi393743990 | 4 | 291-307 | 298 | 4 | 1 | 4 | 1 | Ribosomal protein L6 family protein |
| gi393745957 | 3.5 | 182-199 | 190 | 3 | 2 | 2 | 6 | unknown protein |
| gi393747102 | 4 | 230-246 | 237 | | 4 | 1 | | unknown protein |
| gi393747473 | 4 | 44-61 | 52 | 3 | 2 | | | unknown protein |
| gi393749725 | 4 | 272-288 | 279 | 4 | 1 | 4 | 1 | Ribosomal protein L6 family protein |
| gi393753685 | 4 | 279-295 | 286 | 4 | 1 | 4 | 1 | Ribosomal protein L6 family protein |
| gi393754335 | 3.5 | 201-218 | 209 | 3 | 2 | 2 | 6 | unknown protein |
| gi51453359 | 3.5 | 214-231 | 222 | 3 | 2 | 2 | 6 | unknown protein |
| gi51453947 | 3.5 | 237-254 | 245 | 3 | 2 | 2 | 6 | unknown protein |
| gi55281989 | 4 | 166-182 | 173 | 4 | 1 | 4 | 1 | Ribosomal protein L6 family protein |
