# Supplementary material for: Identification and characterization of cold-responsive microRNAs in tea plant (Camellia sinensis) and their targets using high-throughput sequencing and degradome analysis
Source: BMC Plant Biol. 2014 Oct 21;14:271. doi: 10.1186/s12870-014-0271-x (PMC4209041; doi:10.1186/s12870-014-0271-x)
Supplement: Additional file 11: Table S6. — Targets for new miRNA candidates from C. sinensis treated with cold (+C) and without cold (−C). [file 12870_2014_271_MOESM11_ESM.pdf]

Table S6 Targets for novel miRNAs from *C. sinensis* treated with cold (+C) and without cold (-C)

miRNA family	Target	Alignment Score	Alignment Range	Cleavage Site	-C Library		+C Library		Annotation
					Category	Reads	Category	Reads	
csn-smR35	gi393753144	4	765-785	776		4	1		Leucine-rich repeat (LRR) family protein
csn-smR49	gi343702601	4	604-626	617	4	1			unknown protein
	gi393739968	3	545-567	558	4	1			unknown protein
	gi393746178	3	91-113	104	4	1			unknown protein
	gi343702601	4	606-626	617	4	1			unknown protein
	gi393739968	3	547-567	558	4	1			unknown protein
	gi393746178	3	93-113	104	4	1			unknown protein
	gi393746364	2	759-782	773	4	1			unknown protein
	gi393751507	2	759-782	773	4	1			unknown protein
	gi393751706	2	247-270	261	4	1			unknown protein
	gi393751968	2	752-775	766	4	1			unknown protein
csn-smR77	gi393739015	3.5	53-74	65	4	1			unknown protein
	gi393739072	3.5	41-62	53	4	1			unknown protein
	gi393739159	3.5	19-40	31	4	1			Protein kinase super family protein
	gi393746084	4	434-454	445	3	2	4	1	unknown protein
	gi393748634	3	413-433	424	3	2	4	1	unknown protein
	gi393749372	3	415-435	426	2	2	4	1	unknown protein
	gi393750447	4	437-457	448	3	2	4	1	unknown protein
	gi393750456	4	72-92	83	2	2	4	1	unknown protein
	gi393751118	3	454-474	465	2	2	4	1	unknown protein
	gi393752050	3.5	40-61	52	4	1			unknown protein
	gi393756517	3	411-431	422	2	2	4	1	unknown protein

	gi393756583	3.5	45-66	57	4	1			unknown protein
	gi51453113	4	436-456	447	3	2	4	1	unknown protein
csn-smR2383	gi393738770	4	466-482	473	4	1	2	2	Ribosomal protein S4 (RPS4A) family protein
	gi393745887	4	354-370	361	4	1	2	2	Ribosomal protein S4 (RPS4A) family protein
	gi393747216	4	30-48	38	4	1			unknown protein
	gi393747308	4	477-493	484	4	1	2	2	Ribosomal protein S4 (RPS4A) family protein
	gi393750638	4	291-308	299	4	1			DNA glycosylase superfamily protein
	gi88605435	4	175-192	183	4	1			DNA glycosylase superfamily protein
csn-smR7	gi393388419	0	263-286	277	4	1			unknown protein
	gi393750821	0	654-677	668	4	1			unknown protein
	gi393388419	0	355-379	370	2	2			unknown protein
	gi393750821	0	746-770	761	3	2			unknown protein
csn-smR31	gi313605263	3	152-175	166	4	1			unknown protein
csn-smR15	gi212377615	4	105-124	115	4	1			unknown protein
	gi328684027	4	181-202	192	4	1	4	1	unknown protein
	gi366886971	4	119-140	130	4	1	4	1	unknown protein
	gi366887575	4	19-38	29	4	1			AMP-dependent synthetase and ligase family protein
	gi393744773	3.5	578-598	589	4	1			unknown protein
	gi393748225	4	217-238	228	4	1	4	1	unknown protein
	gi313605213	4	59-82	73	3	3	4	1	unknown protein
	gi328684027	4	254-277	268	3	2	4	1	unknown protein
	gi366893859	4	121-144	135	3	3	4	1	unknown protein
	gi393753168	4	217-240	231	4	1	4	1	pseudogene
csn-smR3736	gi393748480	4	107-128	119	2	5			unknown protein
csn-smR3999	gi170319664	4	459-476	467	4	1	2	4	Ribosomal protein S13/S15
	gi170319665	4	455-472	463	4	1	2	4	Ribosomal protein S13/S15

	gi170319666	4	455-472	463	4	1	2	4	Ribosomal protein S13/S15
	gi170319667	4	455-472	463	4	1	2	4	Ribosomal protein S13/S15
	gi212378862	4	365-382	373	4	1	2	4	Ribosomal protein S13/S15
	gi212380625	4	457-474	465	4	1	2	3	Ribosomal protein S13/S15
	gi393738749	4	444-461	452	4	1	2	3	Ribosomal protein S13/S15
	gi393739471	4	442-459	450	4	1	2	3	Ribosomal protein S13/S15
	gi393741164	4	430-447	438	4	1	2	3	Ribosomal protein S13/S15
	gi393754228	4	433-450	441	4	1	2	4	Ribosomal protein S13/S15
	gi51530191	4	146-163	154		2	4		Ribosomal protein S13/S15
csn-smR28	gi393751216	1	52-75	66	4	1			unknown protein
csn-smR5749	gi212378422	3.5	318-332	323	2	2	4	1	Ras-related small GTP-binding family protein
	gi212378851	3.5	285-299	290	2	2	4	1	Ras-related small GTP-binding family protein
	gi212379940	3.5	316-330	321	2	2	4	1	Ras-related small GTP-binding family protein
	gi212380028	4	413-428	419		4	1		unknown protein
	gi222372528	4	471-485	476		3	2		Protein prenylyltransferase superfamily protein
	gi366894993	4	390-405	396		4	1		unknown protein
	gi393388473	4	383-397	388		2	2		Histone superfamily protein
	gi393389278	3.5	287-301	292	2	2	4	1	Ras-related small GTP-binding family protein
	gi393393718	4	293-307	299	2	7	0	26	transposable element gene
	gi393740146	3.5	274-288	279	2	2	4	1	Ras-related small GTP-binding family protein
	gi393740367	4	691-706	697		2	5		unknown protein
	gi393741860	3.5	257-271	262	2	2	4	1	Ras-related small GTP-binding family protein
	gi393744793	4	255-271	262	4	1			unknown protein
	gi393745144	3.5	194-208	199	2	2	4	1	Ras-related small GTP-binding family protein
	gi393745964	3.5	235-249	240	2	2	4	1	Ras-related small GTP-binding family protein
	gi393747628	3.5	255-269	260	2	2	4	1	Ras-related small GTP-binding family protein

	gi393754583	3.5	553-568	559		4	1		RNA-binding KH domain-containing protein
	gi51453168	4	192-206	198	2	7	0	25	unknown protein
csn-smR65	gi327360503	2	170-190	181	1	4	0	6	unknown protein
csn-smR6621	gi222372478	4	295-311	302	2	8	2	6	unknown protein
	gi300675887	4	86-101	93	2	3	2	2	Cysteine proteinases superfamily protein
	gi366885706	3.5	184-200	191	4	1			unknown protein
	gi366893572	3.5	242-258	249	4	1			unknown protein
	gi393744437	4	360-376	367	2	8	2	5	unknown protein
csn-smR7277	gi170319692	3.5	784-807	798		4	1		pseudogene, putative ADP-ribosylation factor
	gi212377786	3.5	159-182	173	2	39	2	5	unknown protein
	gi212378280	3.5	308-331	322	2	39	3	5	unknown protein
	gi393393386	3.5	52-75	66	2	39	2	5	transposable element gene
	gi393740969	3.5	413-436	427	4	1	2	3	pseudogene, putative ADP-ribosylation factor
	gi393756208	4	638-661	652	4	1			unknown protein
	gi393756234	4	272-296	287	4	1			unknown protein
csn-smR7891	gi221070973	4	158-175	167	4	1	4	1	transposable element gene
csn-smR8342	gi393749009	3	670-687	678	2	2	4	1	unknown protein
csn-smR13	gi393739968	4	543-563	554		3	2		unknown protein
	gi393746178	4	89-109	100		2	2		unknown protein
	gi393749772	2.5	219-238	229		4	1		unknown protein
	gi393750695	2.5	283-302	293		4	1		unknown protein
csn-smR9722	gi282162581	4	535-552	543	3	7	3	2	unknown protein
	gi366893164	4	539-556	547	2	7	2	2	alpha/beta-Hydrolases superfamily protein
	gi366893573	4	539-556	547	2	7	2	2	unknown protein
	gi393390135	4	32-50	41		4	1		unknown protein
	gi393390662	4	171-189	180		4	1		Drought-responsive family protein

	gi393741243	4	549-566	557	3	7	3	2	alpha/beta-Hydrolases superfamily protein
	gi393757086	4	650-668	658		4	1		transposable element gene
csn-smR69	gi393388419	4	262-285	276	2	2			unknown protein
	gi393750821	4	653-676	667	3	2			unknown protein
	gi393388419	4	345-364	355	2	3			unknown protein
	gi393750821	4	736-755	746	3	3			unknown protein
	gi393750994	4	591-610	601	2	15	2	6	unknown protein
csn-smR29	gi393746173	3.5	286-306	297	4	1			unknown protein
csn-smR1014	gi393388419	0	356-379	370	2	2			unknown protein
	gi393750821	0	747-770	761	3	2			unknown protein
csn-smR39	gi393389955	0	538-559	550	4	1			unknown protein
	gi393739719	0	512-533	524	2	2			unknown protein
	gi393744366	2	514-535	526	4	1			unknown protein
	gi393748367	0	509-530	521	3	2			unknown protein
	gi393749396	0	428-449	440	2	2			unknown protein
	gi393750053	0	514-535	526	2	2			unknown protein
	gi393750276	0	519-540	531	3	2			unknown protein
	gi393750728	0	559-580	571	2	2			unknown protein
	gi393750907	0	284-305	296	2	2			unknown protein
	gi393752241	0	512-533	524	3	2			unknown protein
csn-smR85	gi55282192	4	383-403	394	3	3	3	2	unknown protein
csn-smR2408	gi212379903	2	485-502	493	2	2			plastid developmental protein DAG, putative
	gi212381042	2	498-515	506	3	2			plastid developmental protein DAG, putative
	gi284026220	4	308-326	317	4	1	2	2	unknown protein
	gi300675853	2	281-298	289	2	2			plastid developmental protein DAG, putative
	gi366885780	4	104-122	113	3	2	2	21	Protein kinase superfamily protein

	gi393741699	4	244-262	253	1	3			Small nuclear ribonucleoprotein family protein
	gi393754218	4	246-264	255	1	3			Small nuclear ribonucleoprotein family protein
	gi51453506	4	268-286	277	3	2			winged-helix DNA-binding transcription factor family protein
csn-smR72	gi393753794	4	137-157	148	4	1			transposable element gene
csn-smR2592	gi224708755	4	195-216	207	2	13	2	5	unknown protein
	gi366896582	4	186-207	198	2	13	2	5	unknown protein
	gi393393815	4	30-50	41	2	2			unknown protein
	gi393738632	4	361-381	372	3	2			unknown protein
	gi393738980	4	464-484	475	3	2			zinc finger (C3HC4-type RING finger) family protein
	gi393745957	4	169-190	181	2	13	2	5	unknown protein
	gi393748336	4	182-202	193	2	2			unknown protein
	gi393749100	4	154-174	165	2	2			unknown protein
	gi393753435	4	567-587	578	3	2			Proline-rich extensin-like family protein
	gi393756685	4	7-27	18	2	2			unknown protein
	gi51453947	4	224-245	236	2	13	2	5	unknown protein
csn-smR2845	gi212377971	1	351-367	358	4	1	4	1	unknown protein
	gi212378044	2	361-377	368	2	3			unknown protein
	gi212378086	0	361-377	368	2	2	4	1	unknown protein
	gi212378094	0	357-373	364	4	1			unknown protein
	gi212378167	0	351-367	358	2	2	2	2	unknown protein
	gi212378230	0	361-377	368	0	4			unknown protein
	gi212378369	0	360-376	367	4	1			unknown protein
	gi212378387	2	351-367	358	2	3			unknown protein
	gi212378456	0	351-367	358	4	1	2	2	unknown protein
	gi212378581	0	357-373	364	0	3			unknown protein
	gi212378617	0	356-372	363	4	1			unknown protein

gi212378674	0	361-377	368	2	2	2	2	unknown protein
gi212378842	2	351-367	358	2	3			unknown protein
gi212379346	0	351-367	358	4	1	2	2	unknown protein
gi212379459	0	357-373	364	4	1			unknown protein
gi212379540	0	351-367	358	2	2	4	1	unknown protein
gi212379829	0	351-367	358	2	2	2	2	unknown protein
gi212379856	0	361-377	368	2	2	4	1	unknown protein
gi212379904	2	357-373	364	2	3			unknown protein
gi212379918	0	357-373	364	2	2			unknown protein
gi212380043	2	357-373	364	2	3			unknown protein
gi212380151	0	351-367	358	2	2			unknown protein
gi212380541	0	358-374	365	2	3			unknown protein
gi212380656	0	350-366	357		2	2		unknown protein
gi212380720	0	357-373	364	2	2			unknown protein
gi366888519	0	340-356	347	4	1	4	1	unknown protein
gi366893695	0	338-354	345	4	1			unknown protein
gi366897091	0	338-354	345	2	2			Dormancy/auxin associated family protein
gi393389362	0	334-350	341	2	2			AGC kinase family protein
gi393738604	0	333-349	340	2	2			unknown protein
gi393739039	0	296-312	303	2	3			unknown protein
gi393739499	0	316-332	323	2	2			unknown protein
gi393739710	0	84-100	91	4	1			unknown protein
gi393740074	0	41-57	48	2	2			unknown protein
gi393740103	0	331-347	338	2	2			unknown protein
gi393740198	0	337-353	344	4	1			unknown protein
gi393740220	0	293-309	300	2	2			unknown protein

gi393740272	0	339-355	346	2	2			unknown protein
gi393740371	0	342-358	349	4	1			unknown protein
gi393740522	2	337-353	344	2	3			unknown protein
gi393741224	2	345-361	352	2	3			unknown protein
gi393741512	0	341-357	348	4	1			unknown protein
gi393743872	0	338-354	345	2	2			unknown protein
gi393744228	0	342-358	349	4	1			unknown protein
gi393744355	0	331-347	338	4	1	2	3	unknown protein
gi393744496	0	325-341	332	2	2			unknown protein
gi393745150	0	336-352	343	4	1			unknown protein
gi393746310	2	336-352	343	2	3			unknown protein
gi393746556	0	340-356	347	4	1			unknown protein
gi393748110	0	336-352	343	2	2			unknown protein
gi393749395	2	336-352	343	2	3			unknown protein
gi393750614	0	337-353	344	2	2			unknown protein
gi393751675	0	99-115	106		2	2		unknown protein
gi393751921	0	343-359	350	2	2	4	1	unknown protein
gi393753631	0	331-347	338	2	2	2	3	unknown protein
gi393753779	0	324-340	331		2	3		unknown protein
gi393756735	0	44-60	51	4	1			unknown protein
gi393756809	0	336-352	343	4	1	2	3	unknown protein
gi393756835	0	335-351	342		4	1		unknown protein
gi393757010	0	334-350	341	2	2	4	1	unknown protein
gi393757034	0	322-338	329	4	1			RWD domain-containing protein
gi393757048	0	331-347	338	2	2	4	1	unknown protein
gi393757063	0	335-351	342	2	2	4	1	unknown protein

	gi393757183	0	101-117	108	4	1			unknown protein
	gi51452794	2	340-356	347	2	3			unknown protein
	gi51530274	0	207-223	214	2	2			unknown protein
csn-smR34	gi366893895	4	199-218	210	4	1			unknown protein
csn-smR3011	gi366894572	3	131-152	143	4	1			unknown protein
csn-smR3146	gi393744756	4	46-64	55		4	1		Translation protein SH3-like family protein
	gi393752684	4	55-73	64		4	1		Translation protein SH3-like family protein
	gi393753074	4	44-62	53		4	1		Translation protein SH3-like family protein
	gi393756649	4	3-21	12		4	1		Translation protein SH3-like family protein
csn-smR37	gi313605263	4	207-230	221	1	2	2	2	unknown protein
csn-smR9	gi313605213	3.5	59-82	73	3	3	4	1	unknown protein
	gi328684027	3.5	254-277	268	3	2	4	1	unknown protein
	gi366893859	3.5	121-144	135	3	3	4	1	unknown protein
	gi393753168	2	217-240	231	4	1	4	1	pseudogene, similar to receptor-like kinase
csn-smR431	gi340629166	4	200-216	207	2	3	2	3	unknown protein
csn-smR5625	gi224708753	4	837-857	848	2	10	2	10	pseudogene, hypothetical protein
	gi393740513	4	81-101	92	2	10	2	10	unknown protein
	gi393751860	3.5	15-33	25	4	1			SEC7-like guanine nucleotide exchange family protein
csn-smR3933	gi393389955	1	536-559	550	4	1			unknown protein
	gi393739719	1	510-533	524	2	2			unknown protein
	gi393744366	3	512-535	526	4	1			unknown protein
	gi393748367	1	507-530	521	3	2			unknown protein
	gi393749396	1	426-449	440	2	2			unknown protein
	gi393750053	1	512-535	526	2	2			unknown protein
	gi393750276	1	517-540	531	3	2			unknown protein
	gi393750728	1	557-580	571	2	2			unknown protein

	gi393750907	1	282-305	296	2	2			unknown protein
	gi393752241	1	510-533	524	3	2			unknown protein
csn-smR6005	gi393754384	4	191-210	201		2	5		unknown protein
csn-smR32	gi313605213	2.5	62-82	73	3	3	4	1	unknown protein
	gi328684027	2.5	257-277	268	3	2	4	1	unknown protein
	gi366893859	2.5	124-144	135	3	3	4	1	unknown protein
	gi393388517	2.5	223-243	234	3	2	4	1	unknown protein
	gi393748225	3	294-314	305	3	2	4	1	unknown protein
	gi393753168	2	220-240	231	4	1	4	1	pseudogene, similar to receptor-like kinase
csn-smR8111	gi170319777	4	51-68	59		4	1		unknown protein
	gi393389151	4	427-444	435		4	1		VQ motif-containing protein
csn-smR68	gi171355124	3	212-232	223		4	1		unknown protein
	gi171355259	3	212-232	223		4	1		unknown protein
	gi313605213	3.5	74-94	85	4	1	4	1	unknown protein
	gi366893859	3.5	136-156	147	4	1	4	1	unknown protein
	gi393745804	4	89-109	100	2	2			unknown protein
csn-smR5	gi393748405	4	105-125	116	3	2			Ribosomal protein L19e family protein
	gi393749713	4	105-125	116	3	2			Ribosomal protein L19e family protein
	gi393750761	4	97-117	108	3	2			Ribosomal protein L19e family protein
