# Supplementary material for: Identification and characterization of cold-responsive microRNAs in tea plant (Camellia sinensis) and their targets using high-throughput sequencing and degradome analysis
Source: BMC Plant Biol. 2014 Oct 21;14:271. doi: 10.1186/s12870-014-0271-x (PMC4209041; doi:10.1186/s12870-014-0271-x)
Supplement: Additional file 12: Table S7. — Sequences of primers used for the reverse transcription and Quantitative real-time PCR experiments. [file 12870_2014_271_MOESM12_ESM.pdf]

Table S7. Primers sequences of the reverse transcription and Quantitative real-time PCR experiments

Assay name	RT primer (5' to 3')	Quantitative real-time PCR primer	
		**Forward Primer (5' to 3')**	**Reversed Primer (5' to 3')**
csn-smR65	GTCGTATCCAGTGCAGGGTCCGAGGTATTCGCACTGGATACGACT TCTTC	GGCGGCATACGGGTTCAT	CAGTGCAGGGTCCGAGGT
csn-miR156b	GTCGTATCCAGTGCAGGGTCCGAGGTATTCGCACTGGATACGACG TGCTC	GGCGGCTGACAGAAGAGAGT	CAGTGCAGGGTCCGAGGTAT
csn-miR164a	GTCGTATCCAGTGCAGGGTCCGAGGTATTCGCACTGGATACGACT GCACG	GCGGTGGAGAAGCAGGGTA	CGCAGGGTCCGAGGTATT
csn-miR167a	CTCAGCGGCTGTCGTGGACTGCGCGCTGCCGCTGAGGGTGAAGC	CGGCGGAGATCATATGGC	GGCTGTCGTGGACTGCG
csn-miR408	GCGTGGTCCACACCACCTGAGCCGCCACGACCACGCGAGCCAGG	GCCGCCTGCACTGCCT	TCCACACCACCTGAGCCG
csn-miR5072	GTCGTATCCAGTGCAGGGTCCGAGGTATTCGCACTGGATACGACT GGCGA	CCAGCCATCCCCAGCG	CAGTGCAGGGTCCGAGGTAT
